# Supplementary material for: Convergence of monosynaptic and polysynaptic sensory paths onto common motor outputs in a Drosophila feeding connectome
Source: eLife. 2018 Dec 11;7:e40247. doi: 10.7554/eLife.40247 (PMC6289573; doi:10.7554/eLife.40247)
Supplement: Figure 1—source data 1. — Different names exist from analysis of structures analyzed at different stages of development (embryo, larva) and imaging methodology by different authors (Hertweck, 1931; Bodenstein, 1950; Campos-Ortega and Hartenstein, 1985; Schoofs et al., 2010; Hartenstein et al., 2018; Kendroud et al., 2018). The table provides a cross-reference for the readers but should not be taken as a definitive canonical version. At the larval stage, the different nerves enter the CNS from the periphery as single entities. These then split apart into different branches, each with a new set of nerve names, within the CNS. Our usage of the term PaN derives from an analogous nerve described in Calliphora (Ludwig, 1949) and Drosophila (Schoofs et al., 2010). [file elife-40247-fig1-data1.docx]

| Nerve name  (in larva) | Axon bundles | Terminology  (in embryo and larva) | Source |
| --- | --- | --- | --- |
| Antennal nerve (AN) | **1,2,3** | Antennennerv (an)  Antenna nerve (en)  Antennal nerve (an)  Compound pharyngeal nerve (phn)  Pharyngeal nerve (phn) | Hertweck, 1931  Bodenstein, 1950  Schoofs, 2010  Hartenstein, 2017  Kendroud, 2017 |
|  | **1** | Medial root of pharyngeal nerve (pn_m_) | Hartenstein, 2017 |
|  | **2** | Labral nerve (ln)  Hypopharyngeal nerve (hpn)  Anterior root of pharyngeal nerve (pn_a_)  Posterior root of pharyngeal nerve (pn_p_) | Campos-Ortega, 1985  Campos-Ortega, 1985  Kendroud, 2017  Kendroud, 2017 |
|  | **3** | Antennal nerve (an)  Posterior antennal nerve (an_p_) | Kendroud, 2017  Kendroud, 2017 |
| Maxillary nerve (MxN) | **1,2,3** | MN Nerv (mn)  Maxillary nerve (mn)  Maxillary nerve (mn)  Combined maxillary-labial nerve (lbn)  Labial nerve (lbn)  Combined labial nerve (lbn)  Compound labial nerve (lbn) | Hertweck, 1931  Bodenstein, 1950  Schoofs, 2010  Hartenstein, 2017  Kendroud, 2017  Kendroud, 2017  Kendroud, 2017 |
|  | **1** | Labial nerve (lbn)  Labial segmental nerve (ln)  Anterior root of labial nerve (ln_a_) | Campos-Ortega, 1985  Hartenstein, 2017  Kendroud, 2017 |
|  | **2** | Maxillary nerve (mn)  Maxillary segmental nerve (mn)  Maxillary root of labial nerve (mn)  Anterior bundle of the mn (mn_a_)  Posterior bundle of the mn (mn_p_) | Campos-Ortega, 1985  Hartenstein, 2017  Kendroud, 2017  Kendroud, 2017  Kendroud, 2017 |
|  | **3** | Labial nerve (lbn)  Labial segmental nerve (ln)  Posterior root of labial nerve (ln_p_) | Campos-Ortega, 1985  Hartenstein, 2017  Kendroud, 2017 |
| Prothoracic accessory nerve (PaN) | **1** | Prothoracic accessory nerve (PaN)  Prothoracic accessory nerve (PaN) | Ludwig, 1949  Schoofs, 2010 |
