# Supplementary material for: Convergence of monosynaptic and polysynaptic sensory paths onto common motor outputs in a Drosophila feeding connectome
Source: eLife. 2018 Dec 11;7:e40247. doi: 10.7554/eLife.40247 (PMC6289573; doi:10.7554/eLife.40247)
Supplement: Supplementary file 1. — Reconstructions of antennal nerve (AN) sensory neurons, maxillary nerve (MxN) sensory neurons, prothoracic accessory nerve (PaN) sensory neurons, serotonergic modulatory output neurons (Se0), pharyngeal motor neurons (PMN/e .g. AN-L-motor-05), maxillary nerve motor neurons (MxN motor) and prothoracic accessory nerve motor neurons (PaN motor). A dorsal view of each neuron is shown on the left, and a lateral view on the right. Neuron IDs (e.g. ‘123456’) and names (e.g. AN-L-Sens-B1-Aca-01) are provided. Digital 3D model of the neuropil is shown in grey. 3D models of synaptic input and output compartments are colored based on Figure 2. Outline of the nervous system is not shown. Table shows number of synapses of a given row neuron to a column neuron group. Column groups represent sensory neurons (ACa, AVa, AVp, ACal, ACp, ACpl, VM), neuroendocrine output neurons (Dilps, DMS, DH44), serotonergic modulatory output neurons (Se0ens, Se0ph), pharyngeal motor neurons (PMN), MxN motor neurons, PaN motor neurons and projection neurons to Kenyon cells (olfactory PNs, gustatory PNs, multiglomerular PNs, unknown PNs, thermo PNs, visual PNs). [file elife-40247-supp1.pdf]

ID: 2284021
name: AN-L-Sens-B1-ACa-01

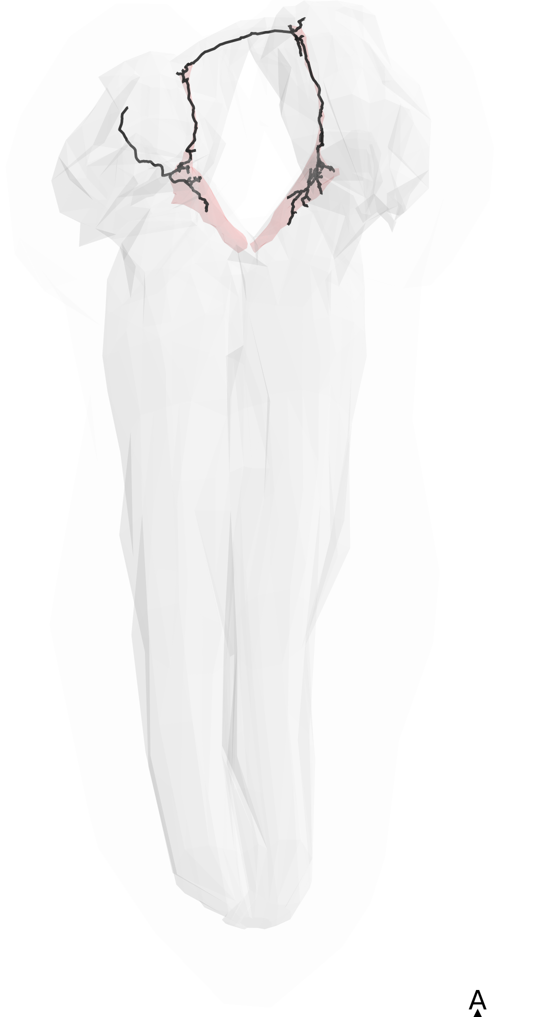

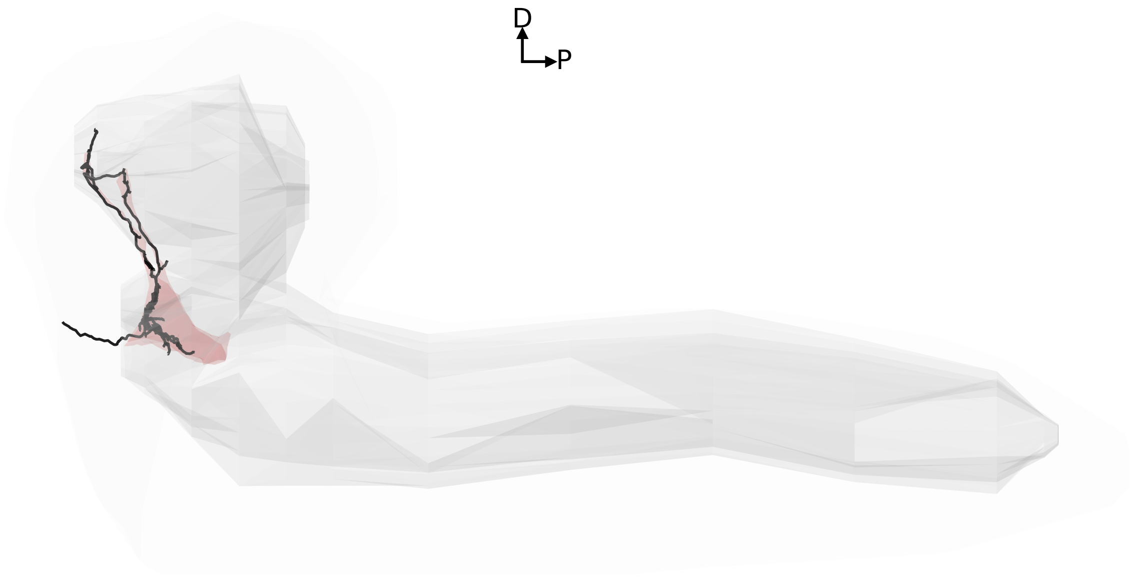

ID	*name*	SCACa	SCAVa	SCAVp	SCACal	SCACp	SCACpl	SCVM	IPCs	DMS	DH44	Se0ens	Se0ph	PMN LR	MN motor neurons	PaN motor neurons	olfactory PNs	gustatory PNs	multiglomerular PNs	unknown PNs	thermo PNs	visual PNs
2284021	AN-L-Sens-B1-ACa-01	3	0	0	0	1	0	0	39	14	1	2	0	0	0	0	0	0	0	0	0	0

ID: 13744425
name: AN-L-Sens-B1-ACa-02

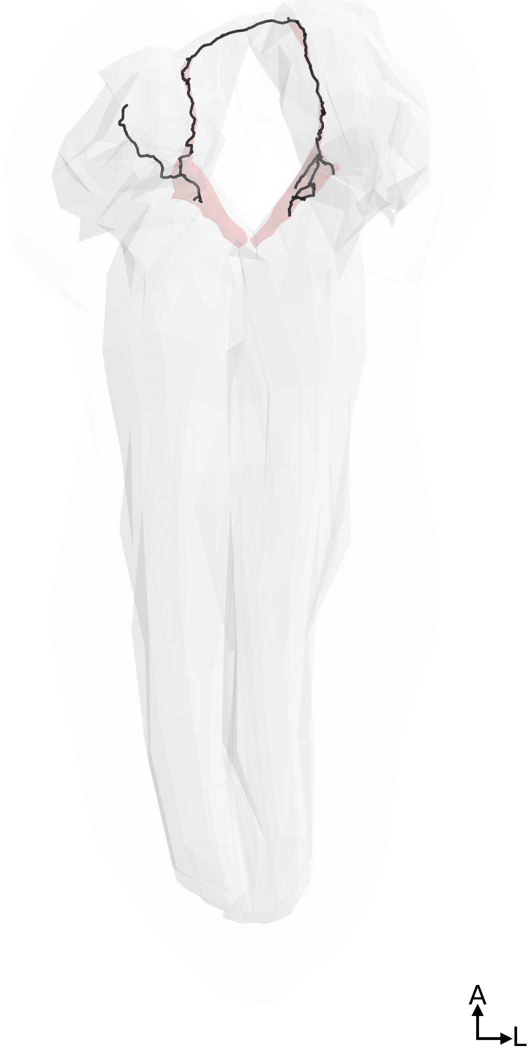

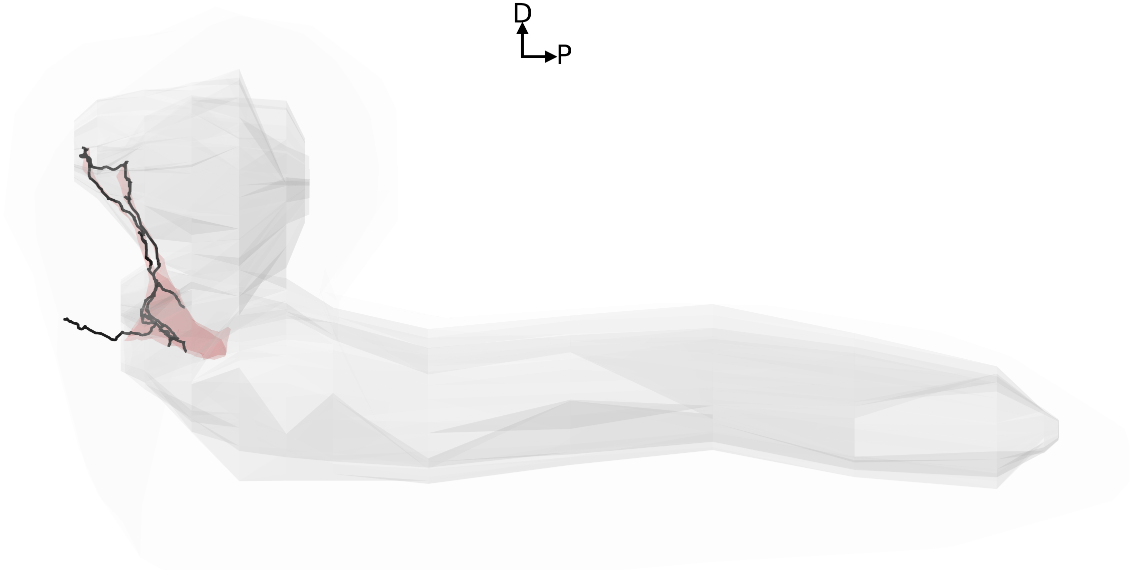

ID	*name*	SCACa	SCAVa	SCAVp	SCACal	SCACp	SCACpl	SCVM	IPCs	DMS	DH44	Se0ens	Se0ph	PMN LR	MN motor neurons	PaN motor neurons	olfactory PNs	gustatory PNs	multiglomerular PNs	unknown PNs	thermo PNs	visual PNs
13744425	AN-L-Sens-B1-ACa-02	8	0	0	0	0	0	0	32	18	6	1	0	0	0	0	0	0	0	0	0	0

ID: 15770032
name: AN-L-Sens-B1-ACa-03

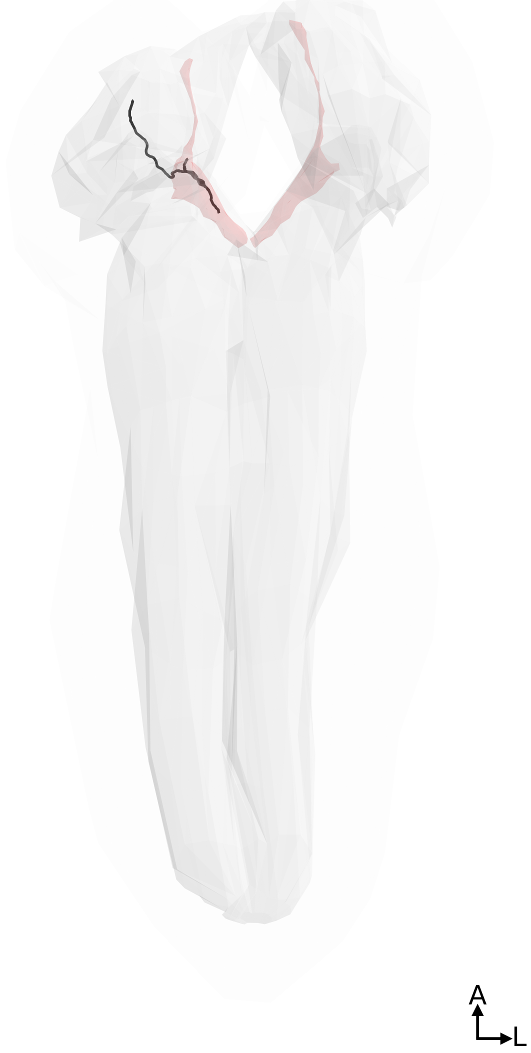

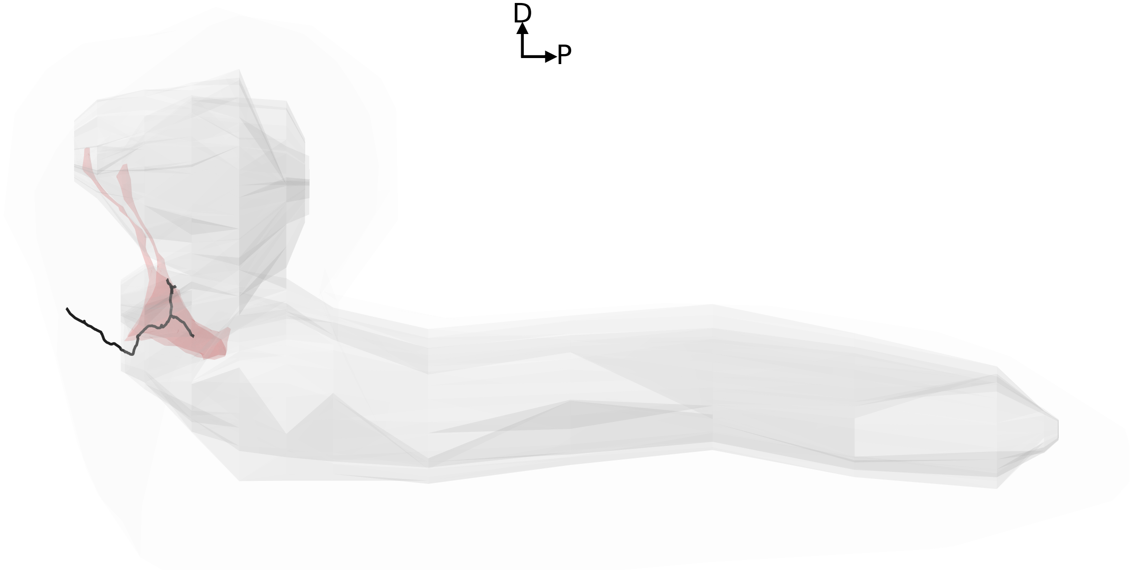

ID	*name*	SCACa	SCAVa	SCAVp	SCACal	SCACp	SCACpl	SCVM	IPCs	DMS	DH44	Se0ens	Se0ph	PMN LR	MN motor neurons	PaN motor neurons	olfactory PNs	gustatory PNs	multiglomerular PNs	unknown PNs	thermo PNs	visual PNs
15770032	AN-L-Sens-B1-ACa-03	2	0	0	0	0	0	0	0	0	22	2	0	0	0	0	0	0	0	0	0	0

ID: 1367171
name: AN-L-Sens-B1-ACa-04

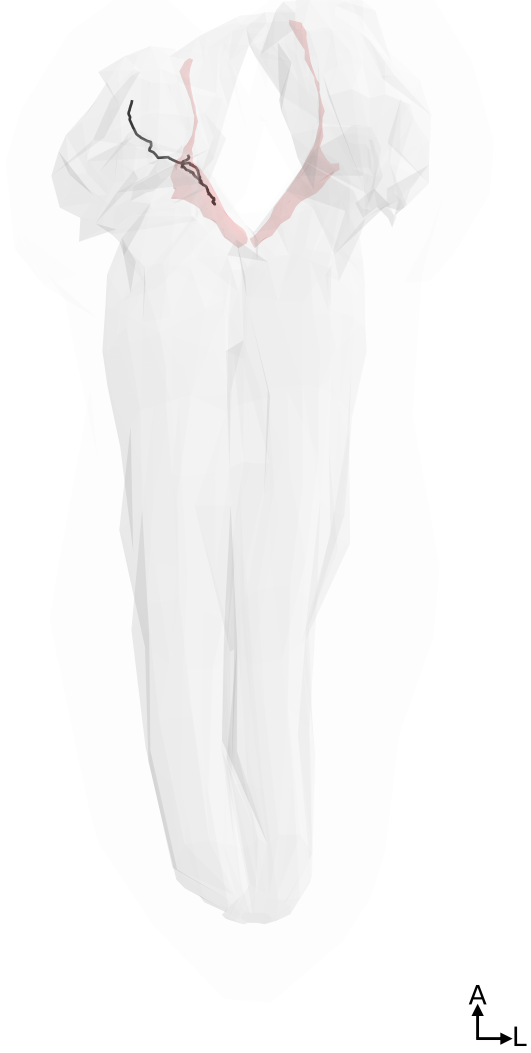

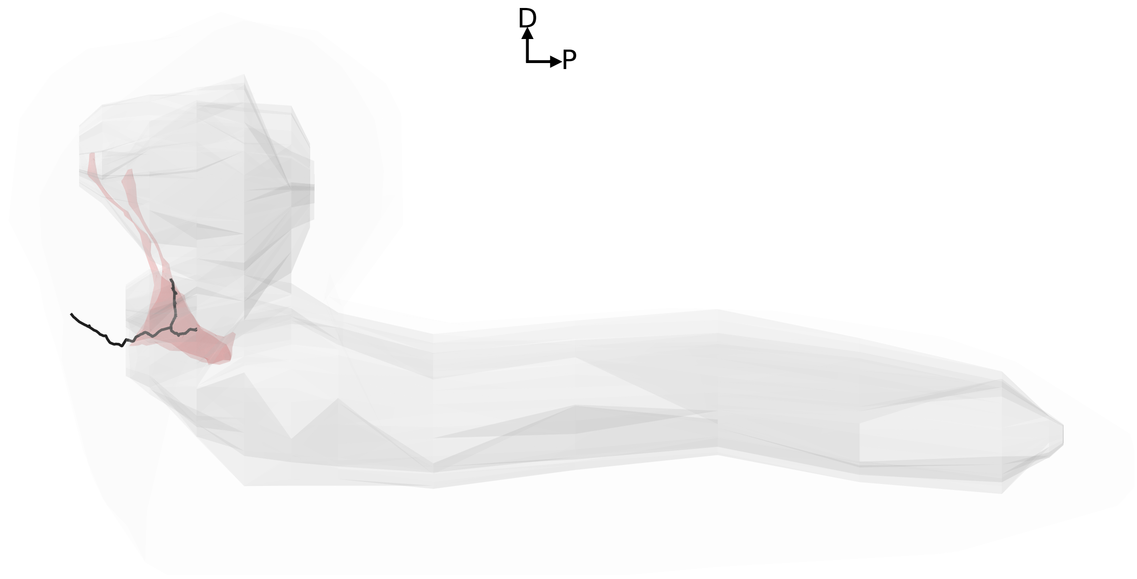

ID	name	SCACa	SCAVa	SCAVp	SCACal	SCACp	SCACpl	SCVM	IPCs	DMS	DH44	Se0ens	Se0ph	PMN LR	MN motor neurons	PaN motor neurons	olfactory PNs	gustatory PNs	multiglomerular PNs	unknown PNs	thermo PNs	visual PNs
1367171	AN-L-Sens-B1-ACa-04	2	0	0	0	0	0	0	0	1	17	3	0	0	0	0	0	0	0	0	0	0

ID: 1398253
name: AN-L-Sens-B1-ACa-05

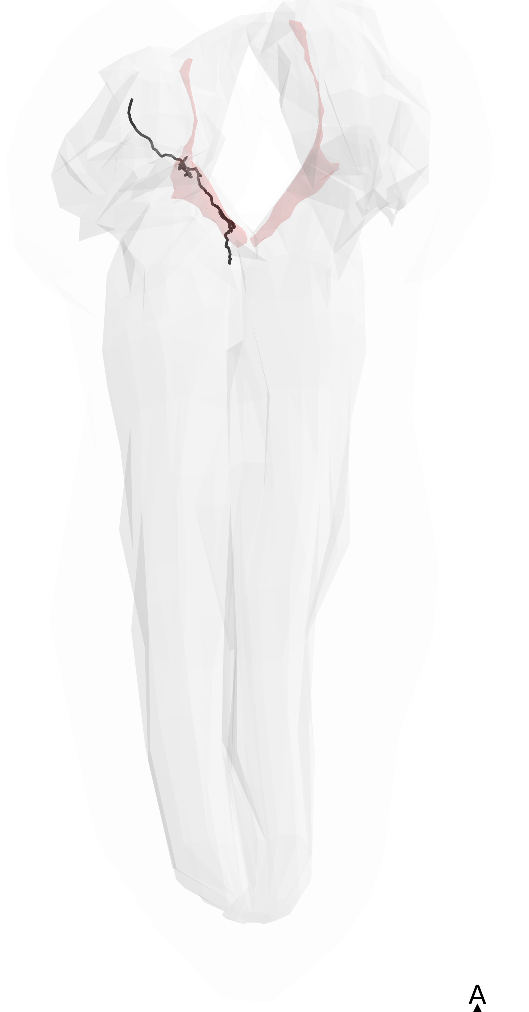

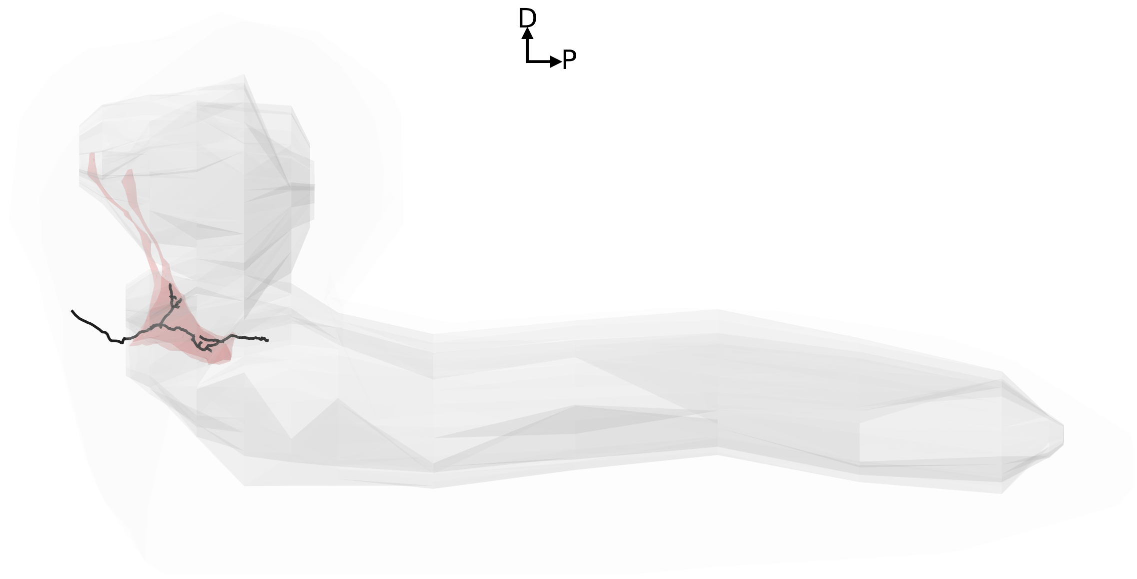

ID	name	SCACa	SCAVa	SCAVp	SCACal	SCACp	SCACpl	SCVM	IPCs	DMS	DH44	Se0ens	Se0ph	PMN LR	MN motor neurons	PaN motor neurons	olfactory PNs	gustatory PNs	multiglomerular PNs	unknown PNs	thermo PNs	visual PNs
1398253	AN-L-Sens-B1-ACa-05	0	0	0	0	0	0	0	1	0	1	0	0	0	0	0	0	0	0	0	0	0

ID: 1392771
name: AN-L-Sens-B1-ACa-06

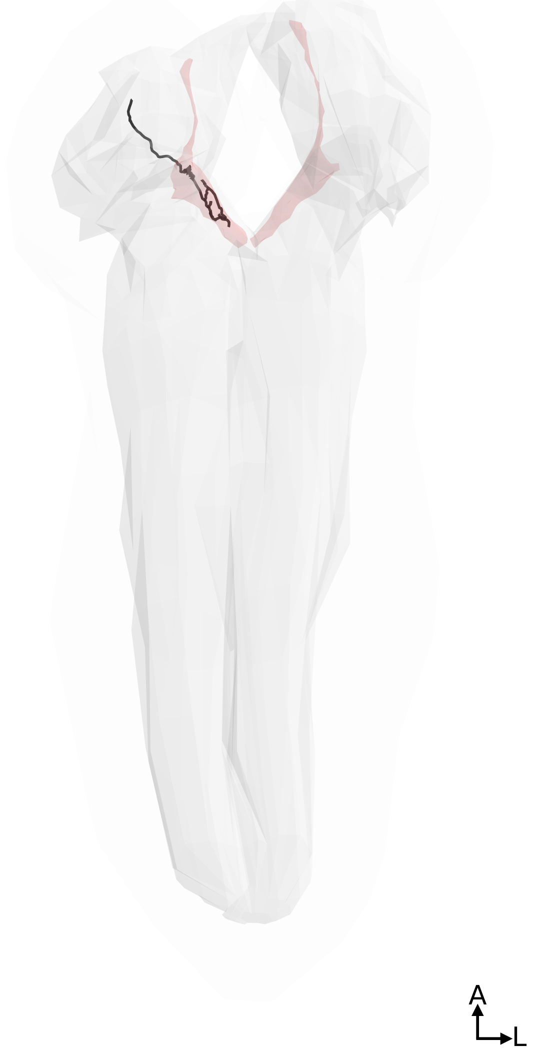

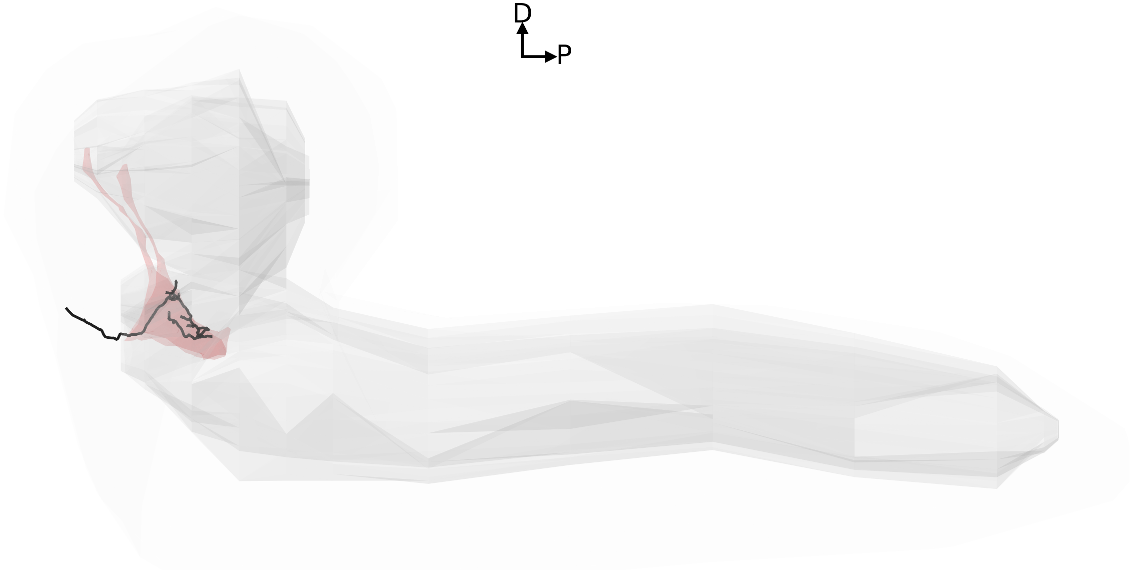

ID	*name*	SCACa	SCAVa	SCAVp	SCACal	SCACp	SCACpl	SCVM	IPCs	DMS	DH44	Se0ens	Se0ph	PMN LR	MN motor neurons	PaN motor neurons	olfactory PNs	gustatory PNs	multiglomerular PNs	unknown PNs	thermo PNs	visual PNs
1392771	AN-L-Sens-B1-ACa-06	0	0	0	0	0	0	0	0	0	0	0	0	0	0	0	0	0	0	0	0	0

ID: 1377586
name: AN-L-Sens-B1-ACa-07

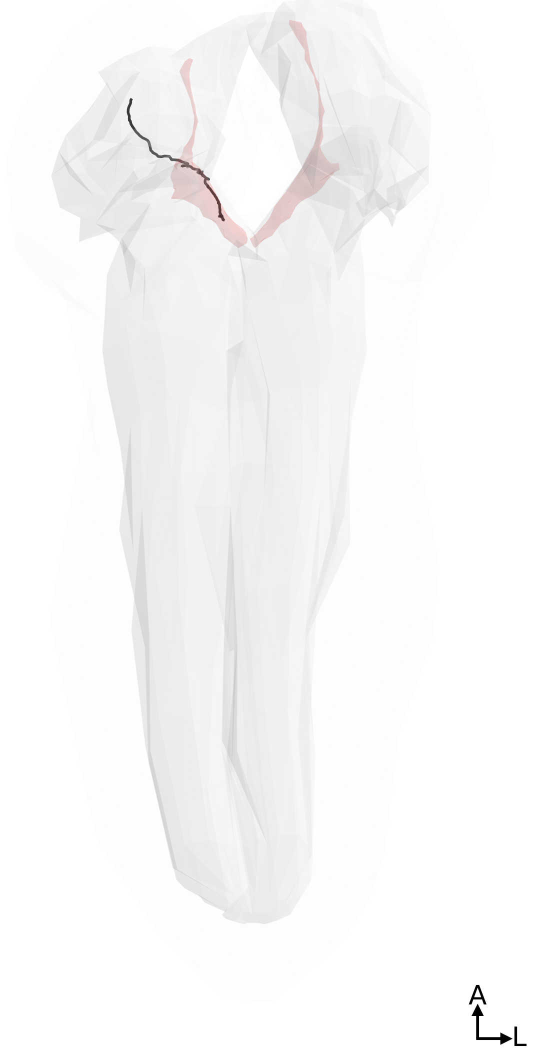

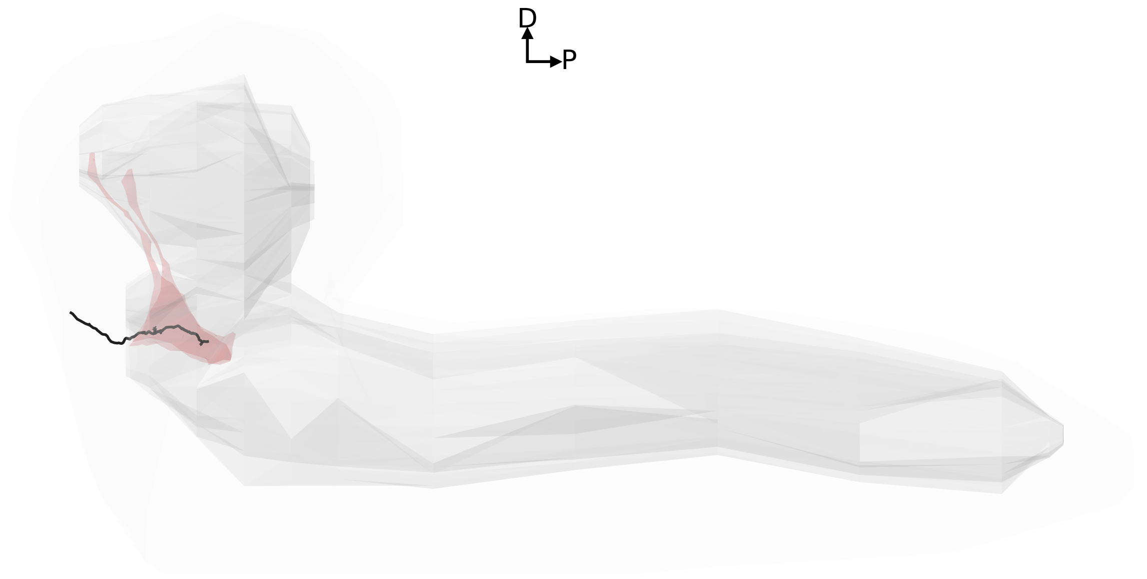

ID	*name*	SCACa	SCAVa	SCAVp	SCACal	SCACp	SCACpl	SCVM	IPCs	DMS	DH44	Se0ens	Se0ph	PMN LR	MN motor neurons	PaN motor neurons	olfactory PNs	gustatory PNs	multiglomerular PNs	unknown PNs	thermo PNs	visual PNs
1377586	AN-L-Sens-B1-ACa-07	3	0	0	0	0	0	0	2	1	0	2	0	0	0	0	0	0	0	0	0	0

ID: 15769419
name: AN-L-Sens-B1-ACa-08

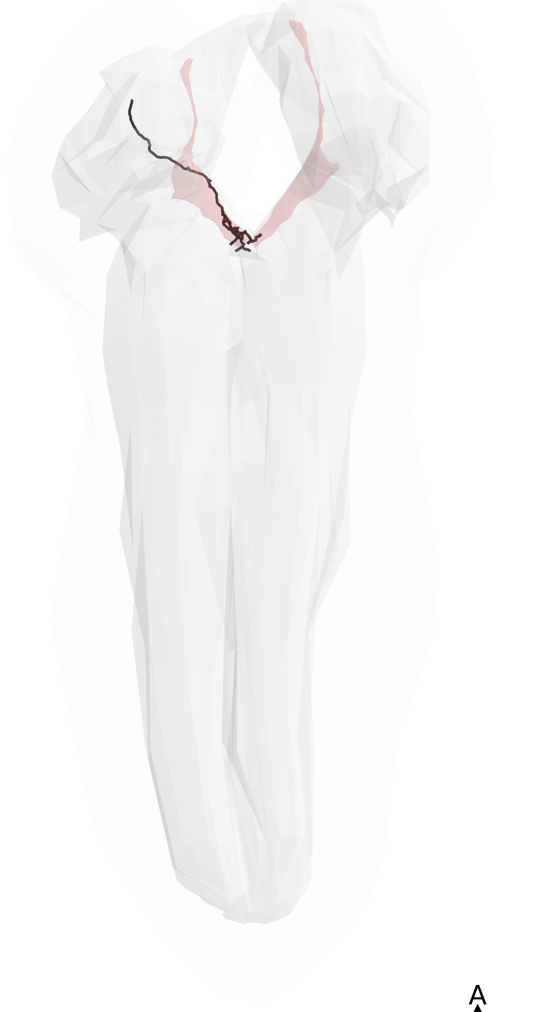

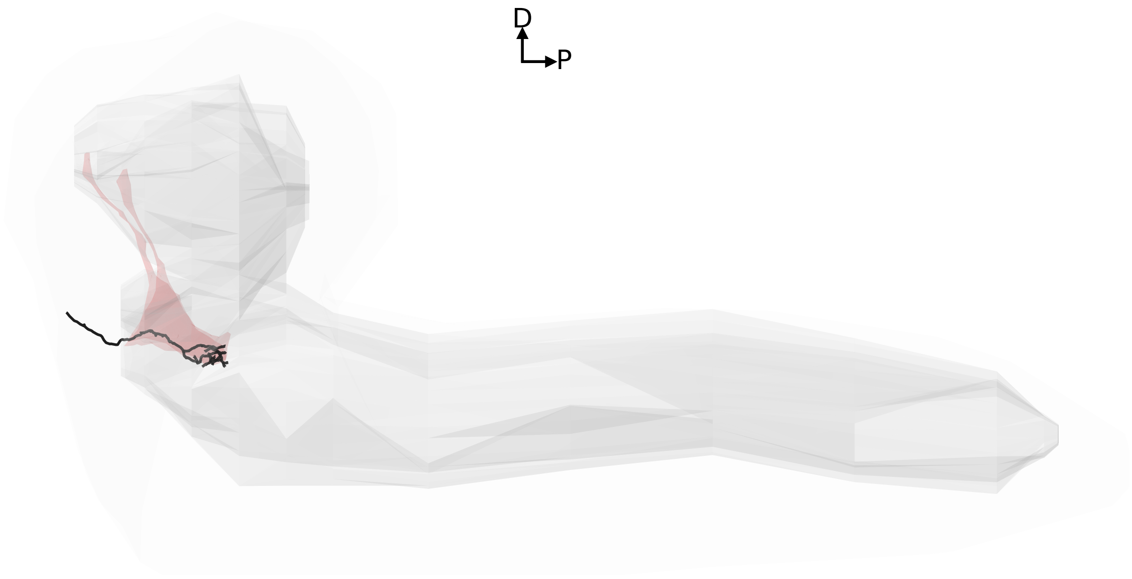

ID	*name*	SCACa	SCAVa	SCAVp	SCACal	SCACp	SCACpl	SCVM	IPCs	DMS	DH44	Se0ens	Se0ph	PMN LR	MN motor neurons	PaN motor neurons	olfactory PNs	gustatory PNs	multiglomerular PNs	unknown PNs	thermo PNs	visual PNs
15769419	AN-L-Sens-B1-ACa-08	5	0	0	0	0	0	0	0	0	0	5	0	0	0	0	0	0	0	0	0	0

ID: 1405493
name: AN-L-Sens-B1-ACa-09

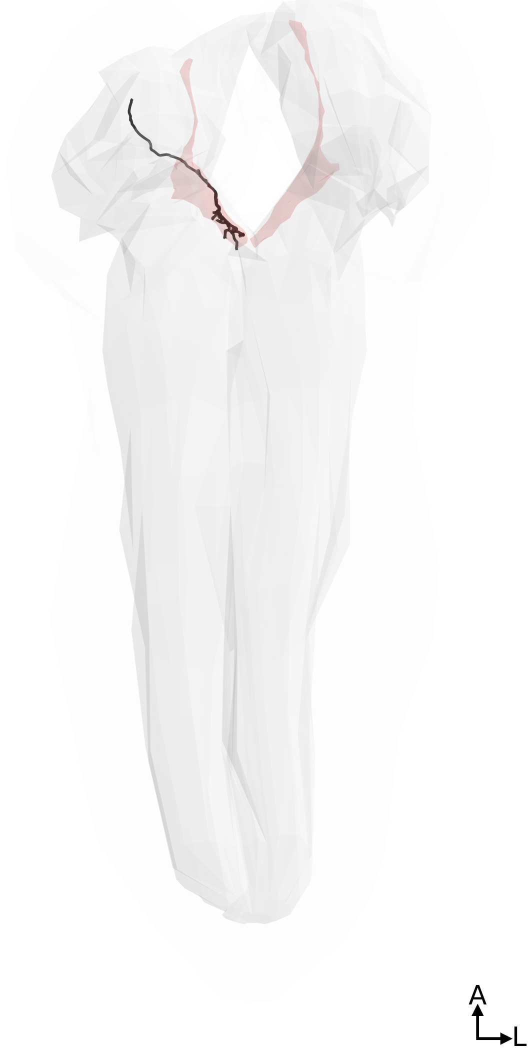

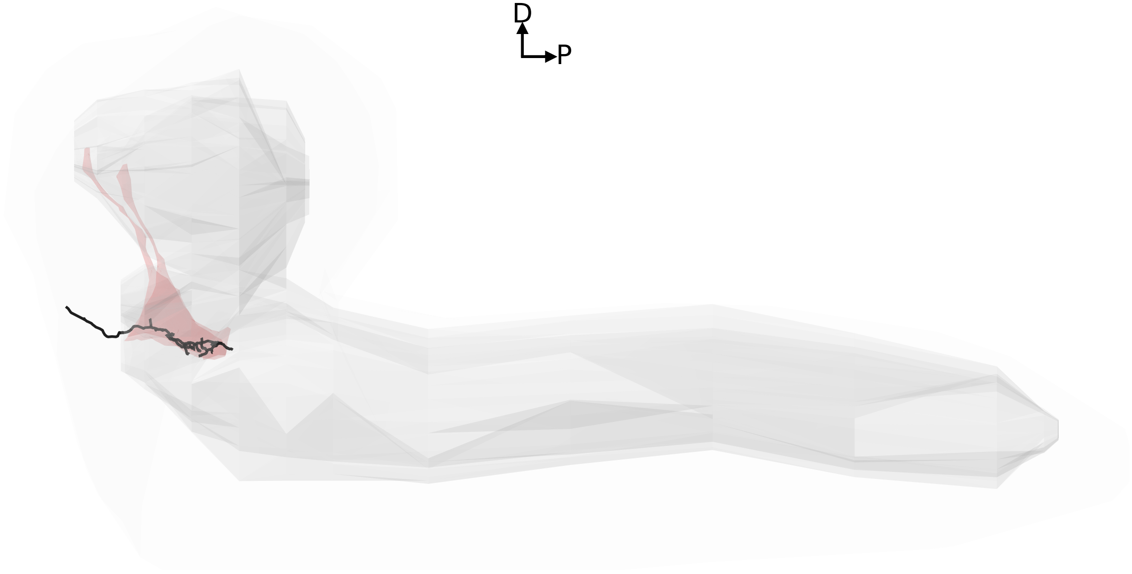

ID	*name*	SCACa	SCAVa	SCAVp	SCACal	SCACp	SCACpl	SCVM	IPCs	DMS	DH44	Se0ens	Se0ph	PMN LR	MN motor neurons	PaN motor neurons	olfactory PNs	gustatory PNs	multiglomerular PNs	unknown PNs	thermo PNs	visual PNs
1405493	AN-L-Sens-B1-ACa-09	5	1	0	0	0	0	0	0	1	1	3	0	0	0	0	0	0	0	0	0	0

ID: 40152
name: AN-L-Sens-B1-ACa-10

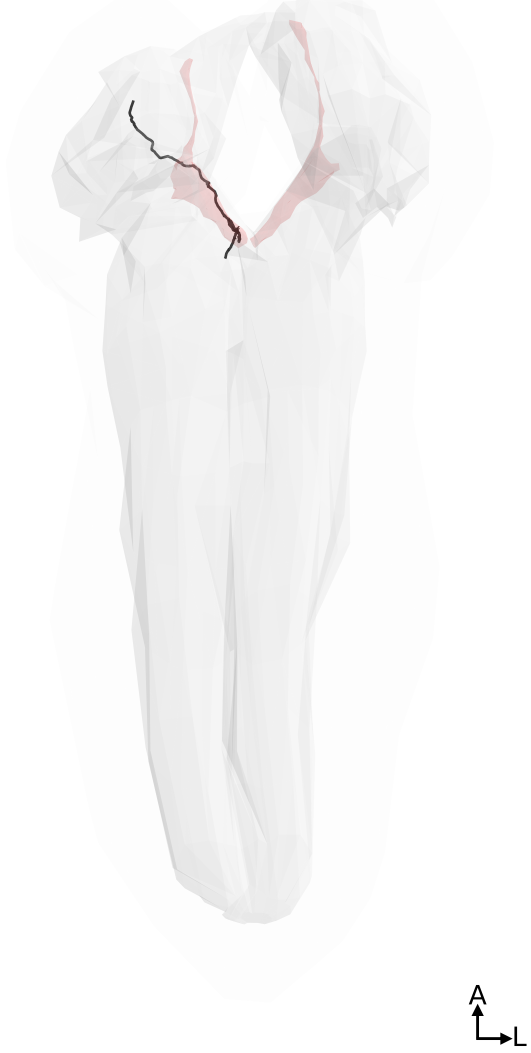

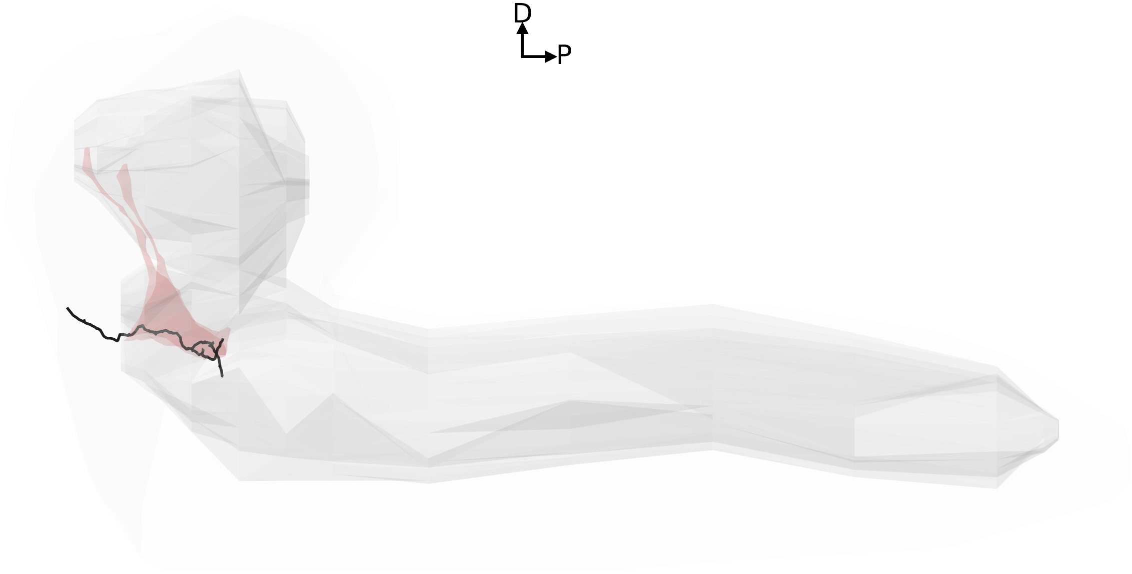

ID	*name*	SCACa	SCAVa	SCAVp	SCACal	SCACp	SCACpl	SCVM	IPCs	DMS	DH44	Se0ens	Se0ph	PMN LR	MN motor neurons	PaN motor neurons	olfactory PNs	gustatory PNs	multiglomerular PNs	unknown PNs	thermo PNs	visual PNs
40152	AN-L-Sens-B1-ACa-10	3	1	0	0	0	0	0	2	7	1	1	0	0	0	0	0	0	0	0	0	0

ID: 1395971
name: AN-L-Sens-B1-ACa-11

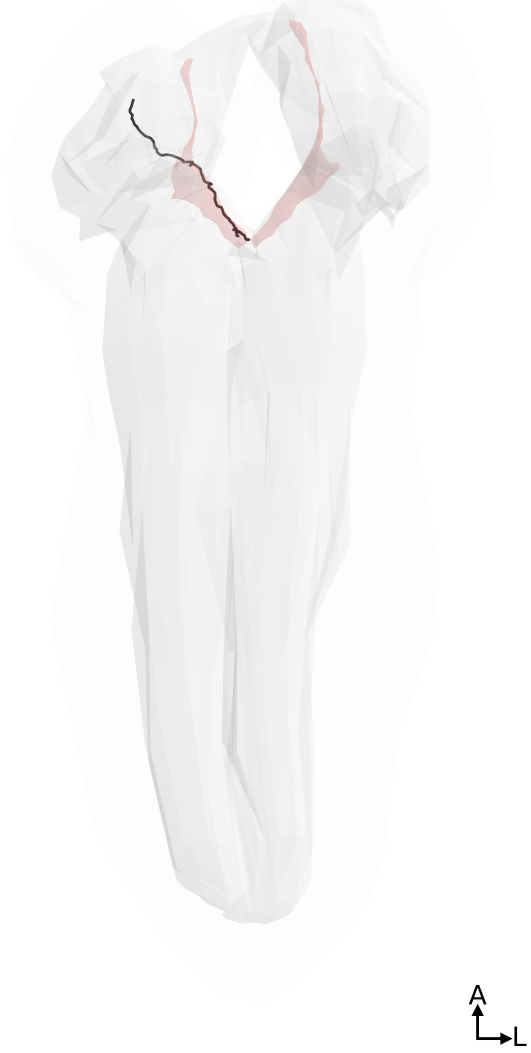

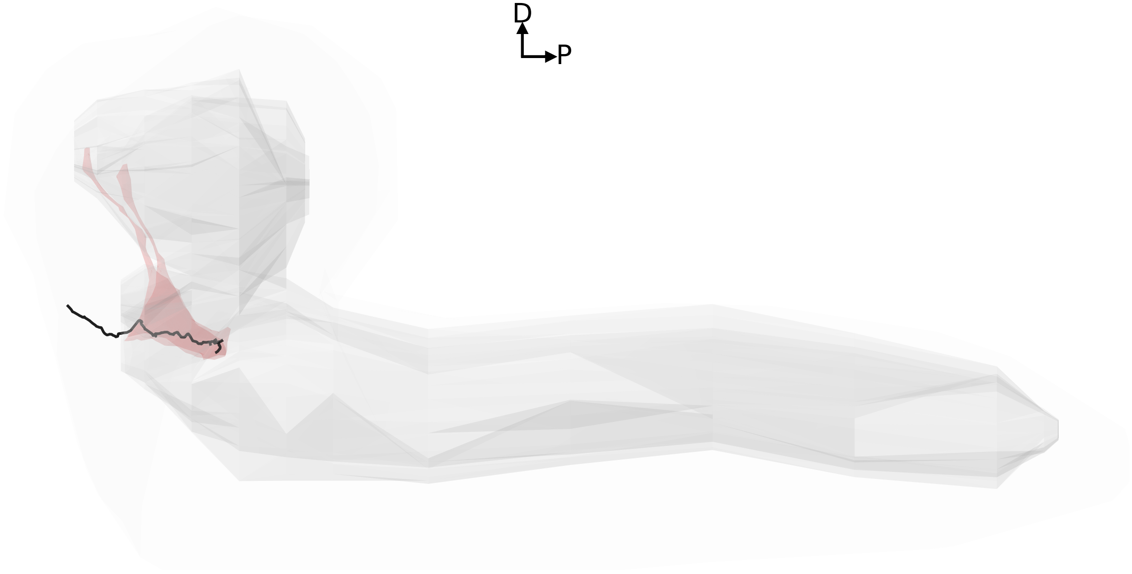

ID	*name*	SCACa	SCAVa	SCAVp	SCACal	SCACp	SCACpl	SCVM	IPCs	DMS	DH44	Se0ens	Se0ph	PMN LR	MN motor neurons	PaN motor neurons	olfactory PNs	gustatory PNs	multiglomerular PNs	unknown PNs	thermo PNs	visual PNs
1395971	AN-L-Sens-B1-ACa-11	4	0	0	0	0	0	0	1	4	0	2	0	0	0	0	0	0	0	0	0	0

ID: 1400725
name: AN-L-Sens-B1-ACa-12

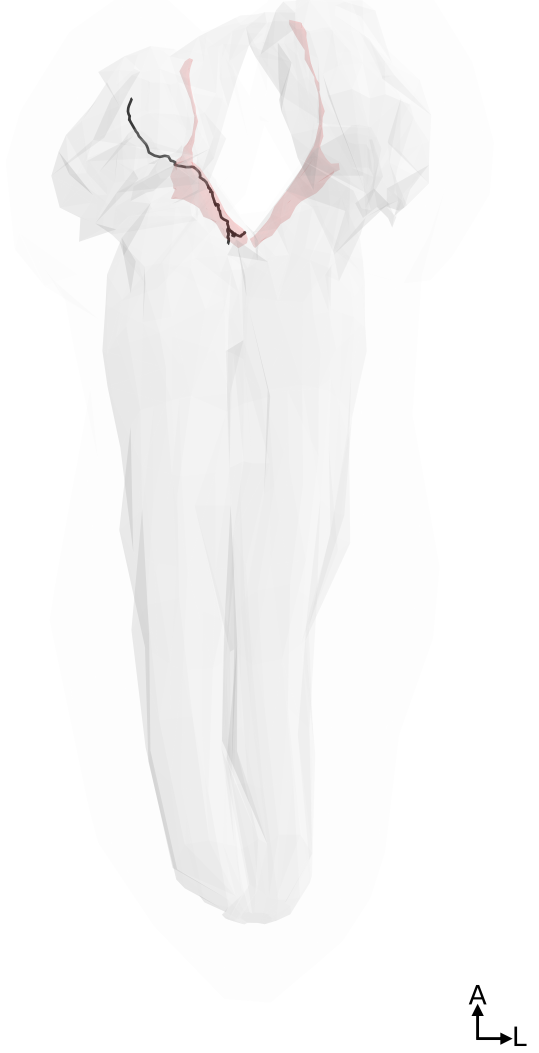

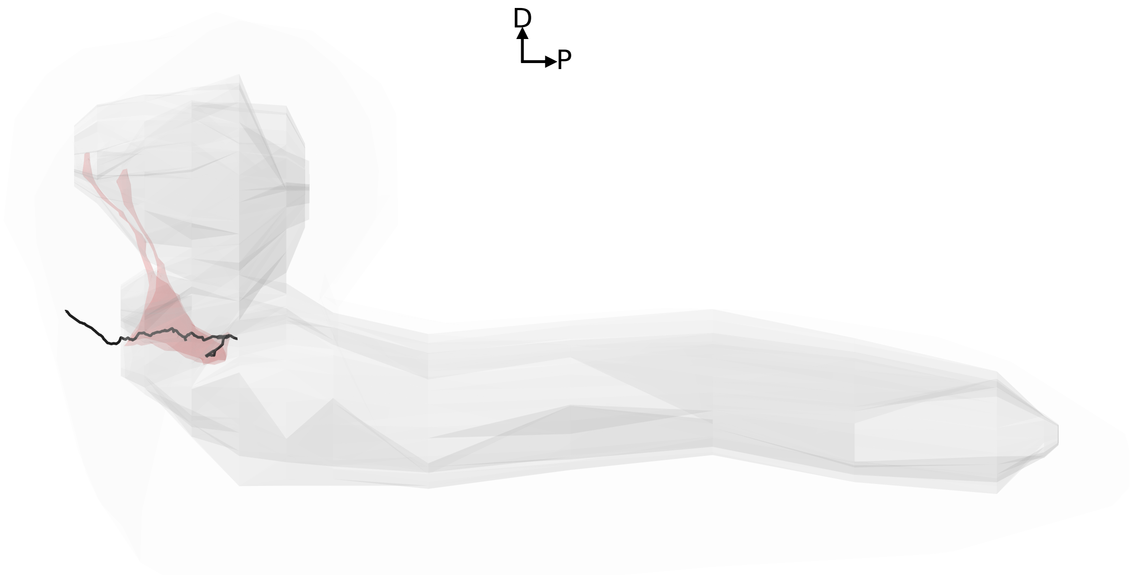

ID	*name*	SCACa	SCAVa	SCAVp	SCACal	SCACp	SCACpl	SCVM	IPCs	DMS	DH44	Se0ens	Se0ph	PMN LR	MN motor neurons	PaN motor neurons	olfactory PNs	gustatory PNs	multiglomerular PNs	unknown PNs	thermo PNs	visual PNs
1400725	AN-L-Sens-B1-ACa-12	1	0	0	0	0	0	0	0	0	3	0	0	0	0	0	0	0	0	0	0	0

ID: 9838294
name: AN-L-Sens-B1-ACa-13

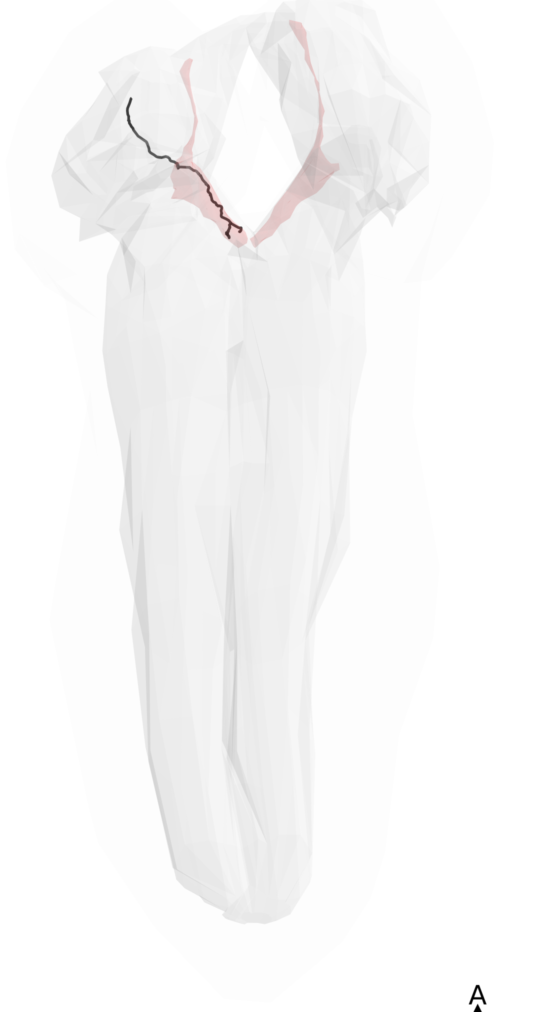

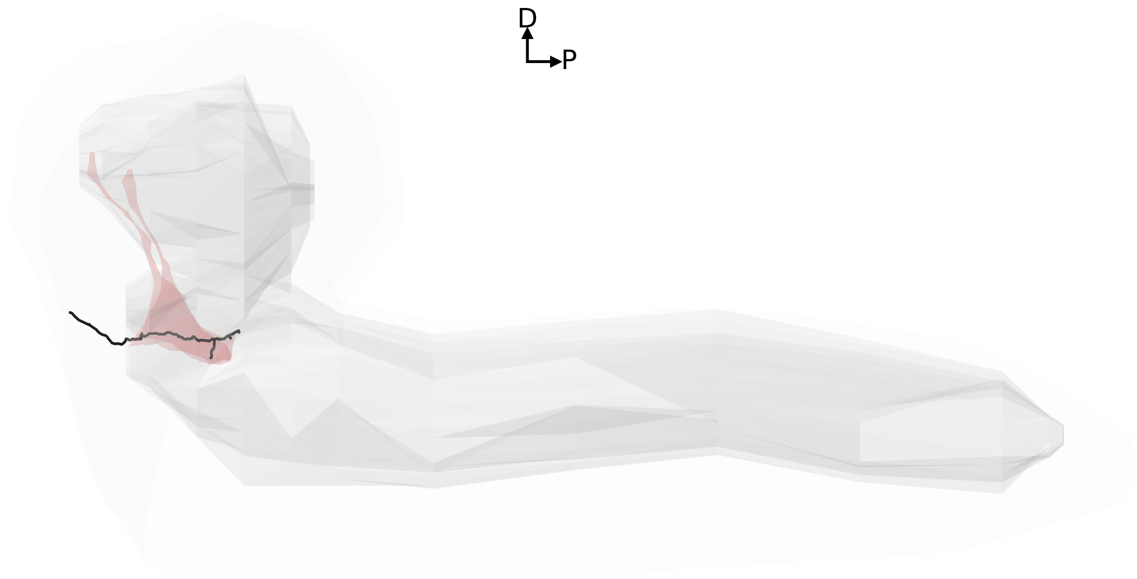

ID	name	SCACa	SCAVa	SCAVp	SCACal	SCACp	SCACpl	SCVM	IPCs	DMS	DH44	Se0ens	Se0ph	PMN LR	MN motor neurons	PaN motor neurons	olfactory PNs	gustatory PNs	multiglomerular PNs	unknown PNs	thermo PNs	visual PNs
9838294	AN-L-Sens-B1-ACa-13	0	0	0	0	0	0	0	0	0	1	0	0	0	0	0	0	0	0	0	0	0

ID: 1406463
name: AN-L-Sens-B1-ACa-14

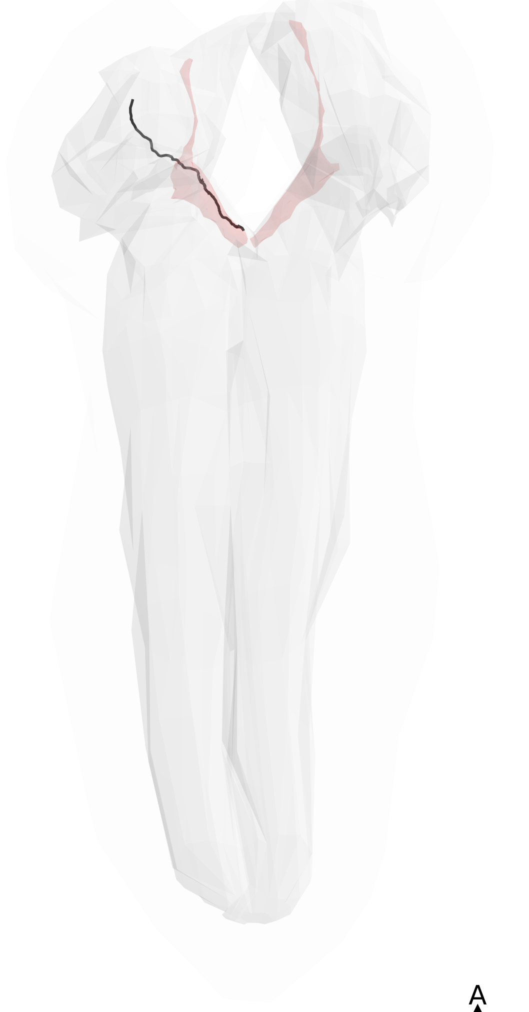

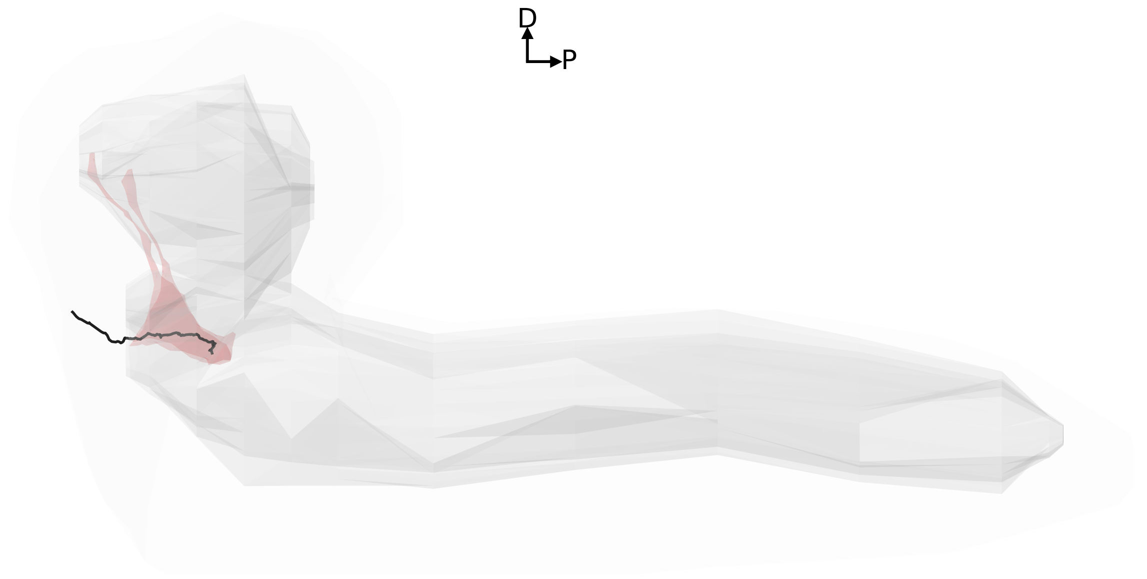

ID	*name*	SCACa	SCAVa	SCAVp	SCACal	SCACp	SCACpl	SCVM	IPCs	DMS	DH44	Se0ens	Se0ph	PMN LR	MN motor neurons	PaN motor neurons	olfactory PNs	gustatory PNs	multiglomerular PNs	unknown PNs	thermo PNs	visual PNs
1406463	AN-L-Sens-B1-ACa-14	1	0	0	0	0	0	0	0	0	1	0	0	0	0	0	0	0	0	0	0	0

ID: 1391179
name: AN-L-Sens-B1-ACa-15

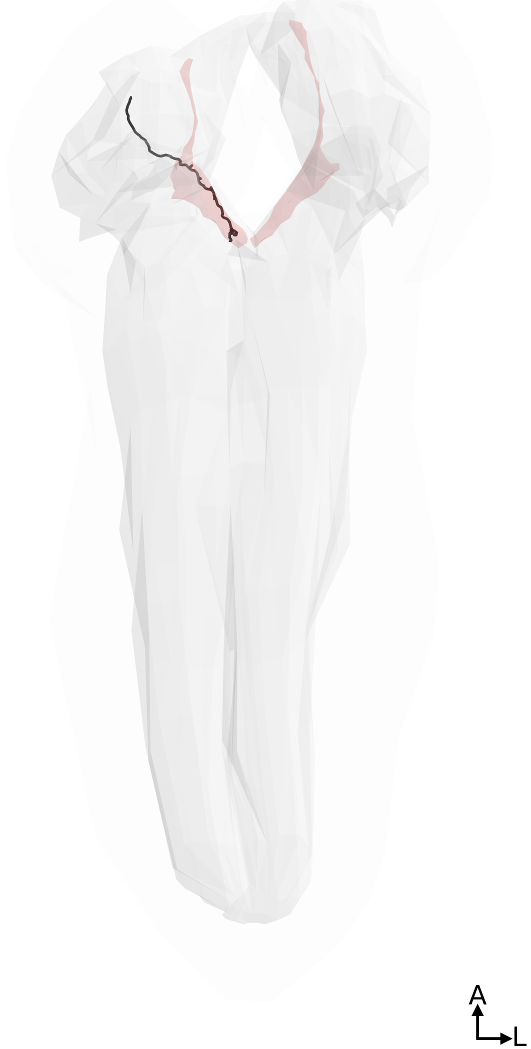

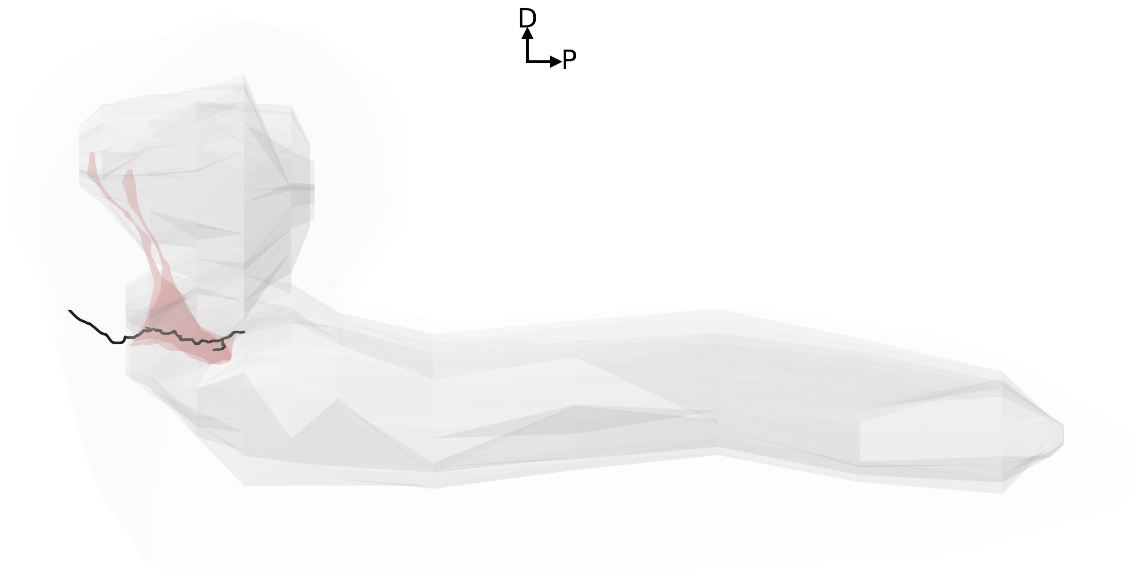

ID	name	SCACa	SCAVa	SCAVp	SCACal	SCACp	SCACpl	SCVM	IPCs	DMS	DH44	Se0ens	Se0ph	PMN LR	MN motor neurons	PaN motor neurons	olfactory PNs	gustatory PNs	multiglomerular PNs	unknown PNs	thermo PNs	visual PNs
1391179	AN-L-Sens-B1-ACa-15	3	0	0	0	0	0	0	0	0	6	0	0	0	0	0	0	0	0	0	0	0

ID: 2456826
name: AN-R-Sens-B1-ACa-01

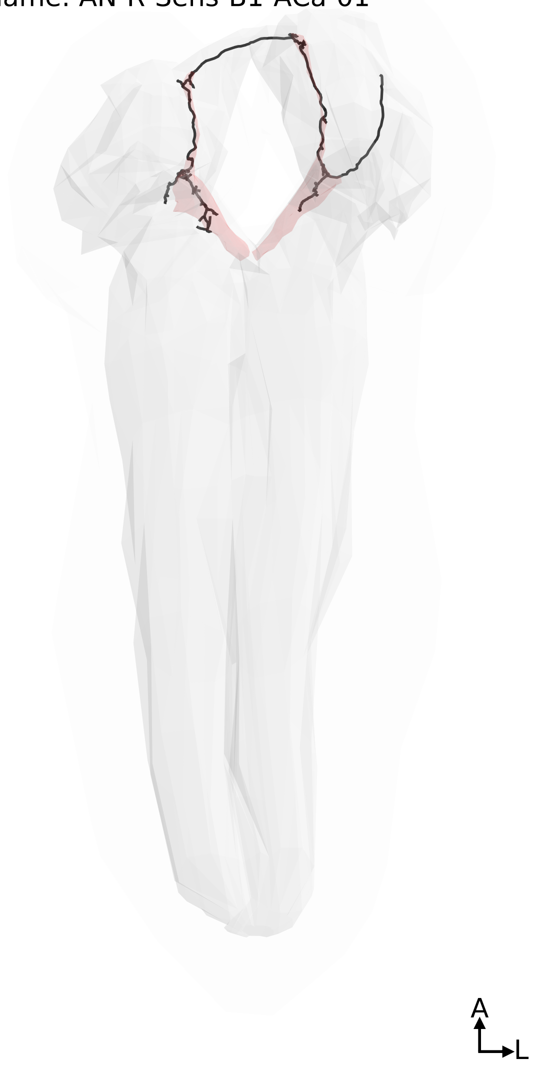

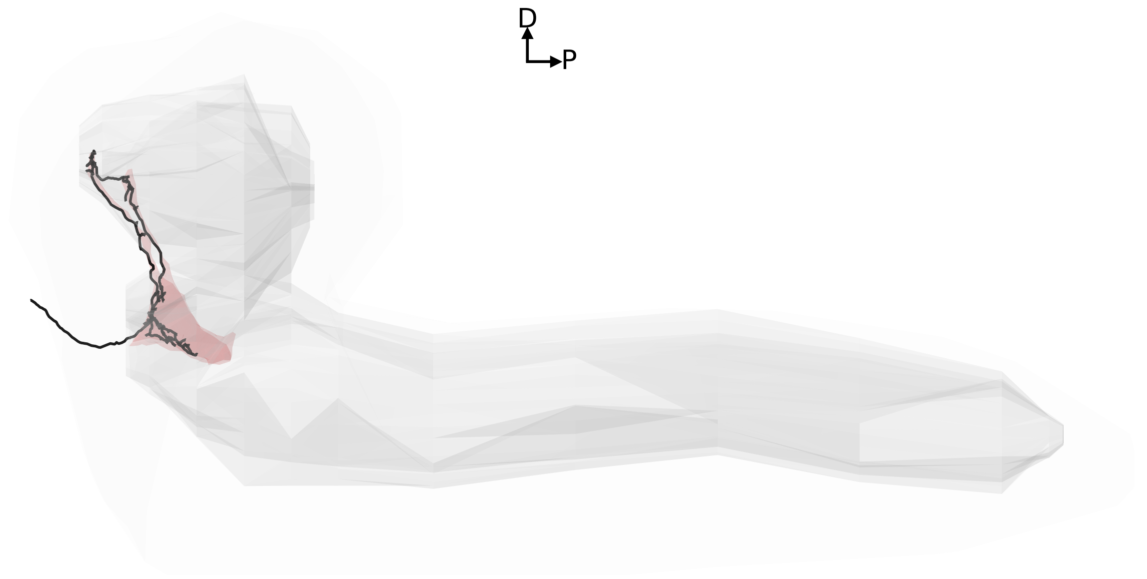

ID	*name*	SCACa	SCAVa	SCAVp	SCACal	SCACp	SCACpl	SCVM	IPCs	DMS	DH44	Se0ens	Se0ph	PMN LR	MN motor neurons	PaN motor neurons	olfactory PNs	gustatory PNs	multiglomerular PNs	unknown PNs	thermo PNs	visual PNs
2456826	AN-R-Sens-B1-ACa-01	4	0	0	0	0	0	0	17	13	0	0	0	0	0	0	0	0	0	0	0	0

ID: 2340336
name: AN-R-Sens-B1-ACa-02

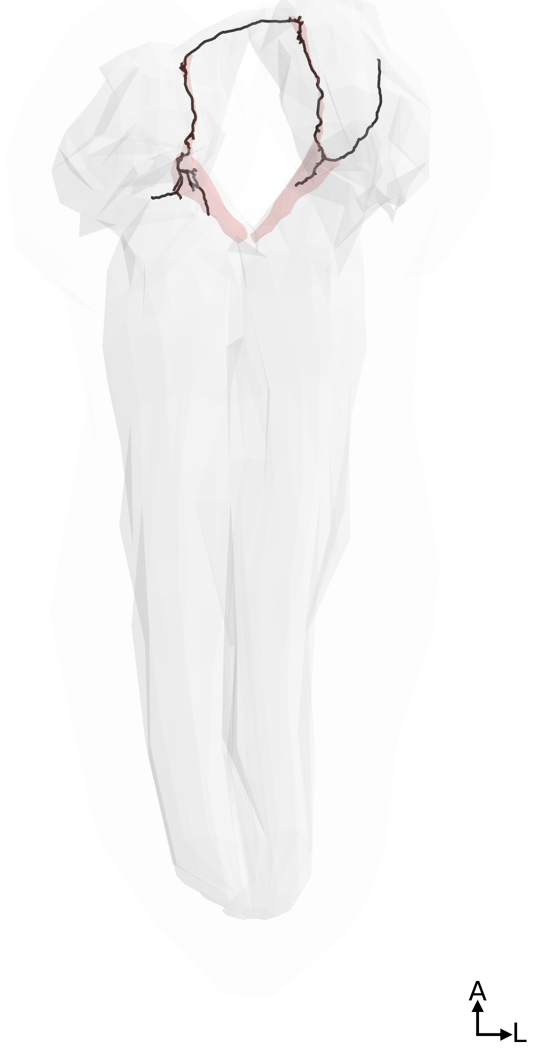

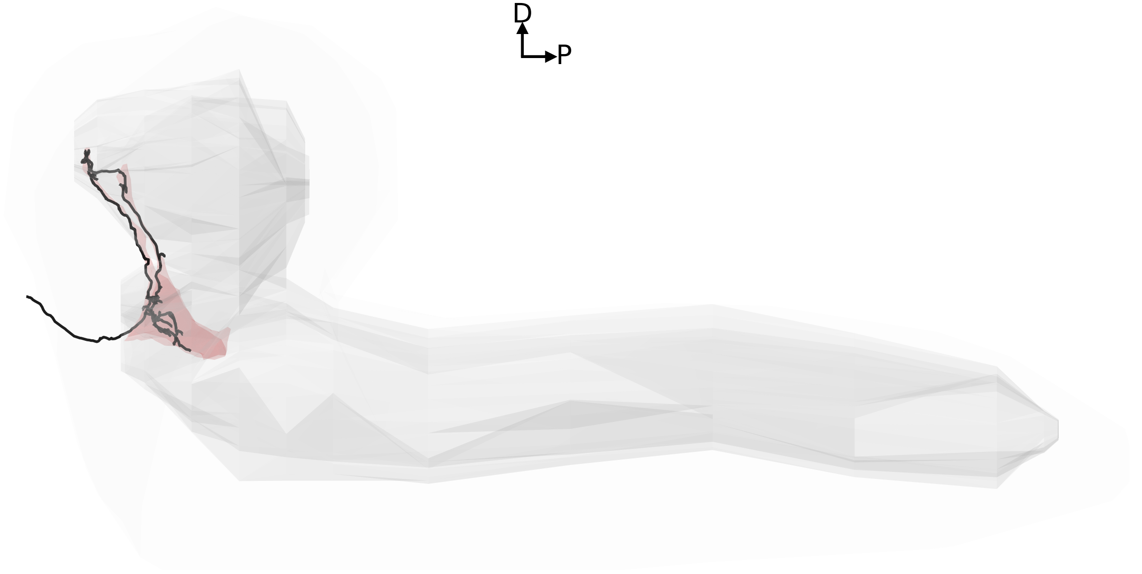

ID	*name*	SCACa	SCAVa	SCAVp	SCACal	SCACp	SCACpl	SCVM	IPCs	DMS	DH44	Se0ens	Se0ph	PMN LR	MN motor neurons	PaN motor neurons	olfactory PNs	gustatory PNs	multiglomerular PNs	unknown PNs	thermo PNs	visual PNs
2340336	AN-R-Sens-B1-ACa-02	5	0	0	0	0	0	0	33	9	0	0	0	0	0	0	0	0	0	0	0	0

ID: 3556191
name: AN-R-Sens-B1-ACa-03

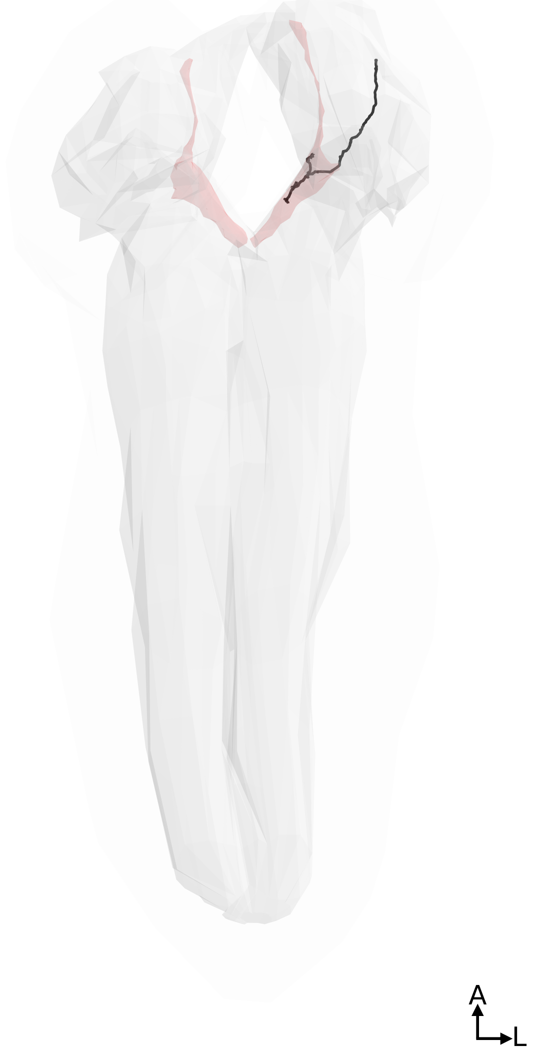

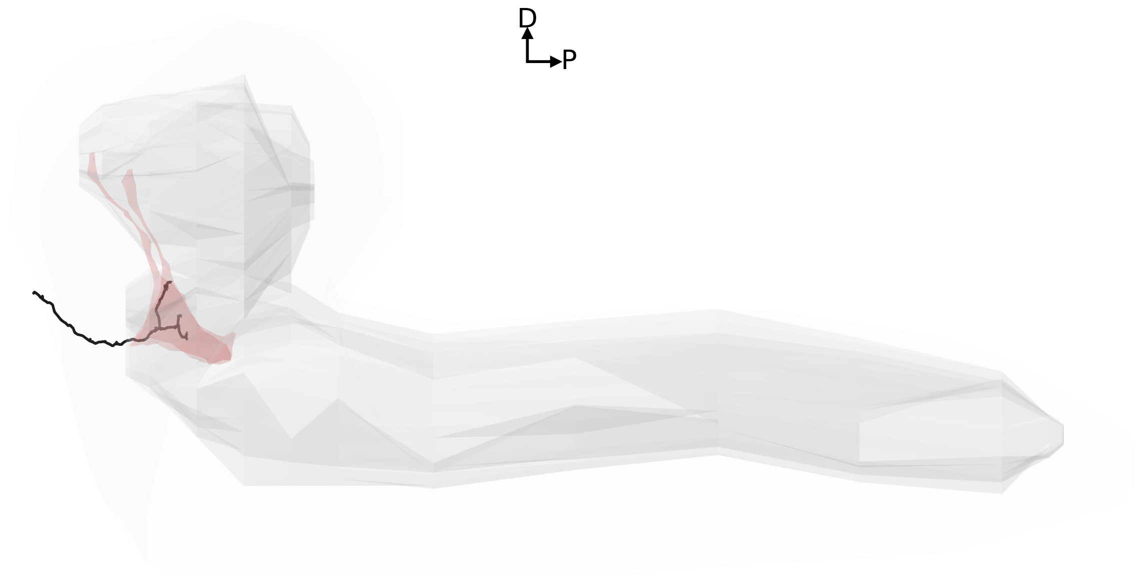

ID	*name*	SCACa	SCAVa	SCAVp	SCACal	SCACp	SCACpl	SCVM	IPCs	DMS	DH44	Se0ens	Se0ph	PMN LR	MN motor neurons	PaN motor neurons	olfactory PNs	gustatory PNs	multiglomerular PNs	unknown PNs	thermo PNs	visual PNs
3556191	AN-R-Sens-B1-ACa-03	2	0	0	0	0	0	0	0	0	14	1	0	0	0	0	0	0	0	0	0	0

ID: 15559195
name: AN-R-Sens-B1-ACa-04

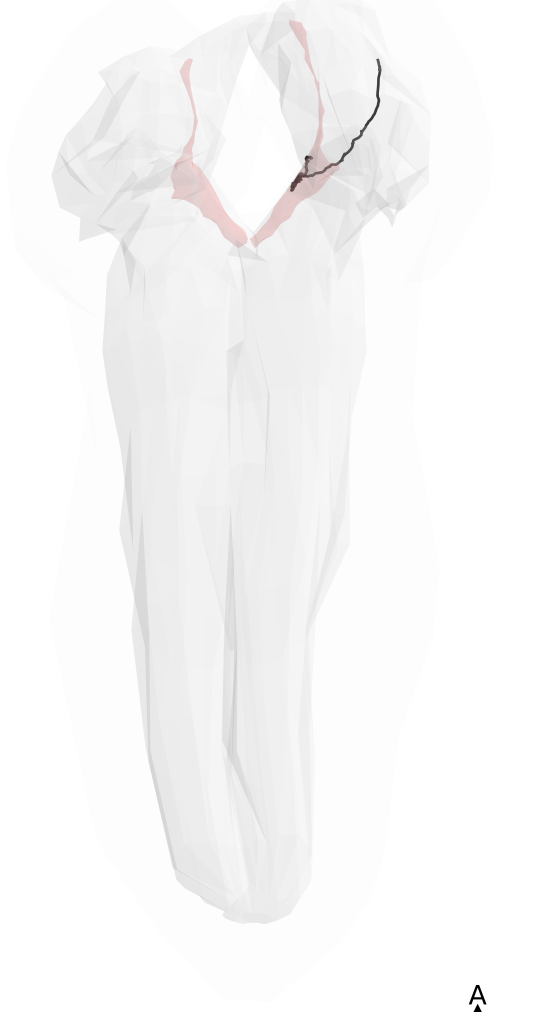

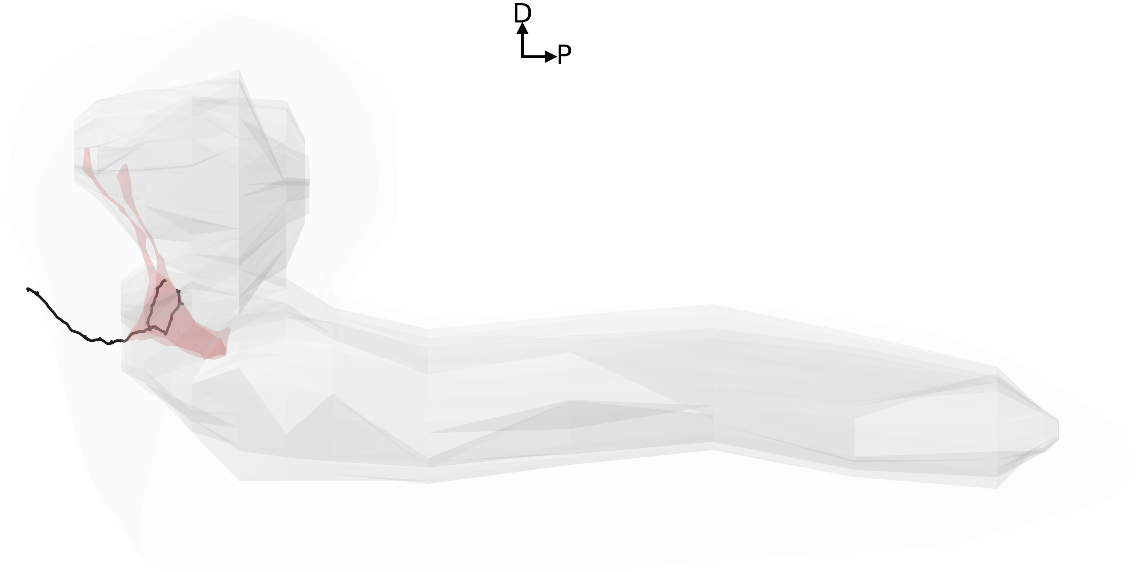

ID	*name*	SCACa	SCAVa	SCAVp	SCACal	SCACp	SCACpl	SCVM	IPCs	DMS	DH44	Se0ens	Se0ph	PMN LR	MN motor neurons	PaN motor neurons	olfactory PNs	gustatory PNs	multiglomerular PNs	unknown PNs	thermo PNs	visual PNs
15559195	AN-R-Sens-B1-ACa-04	0	0	0	0	0	0	0	0	1	11	1	0	0	0	0	0	0	0	0	0	0

ID: 15563553
name: AN-R-Sens-B1-ACa-05

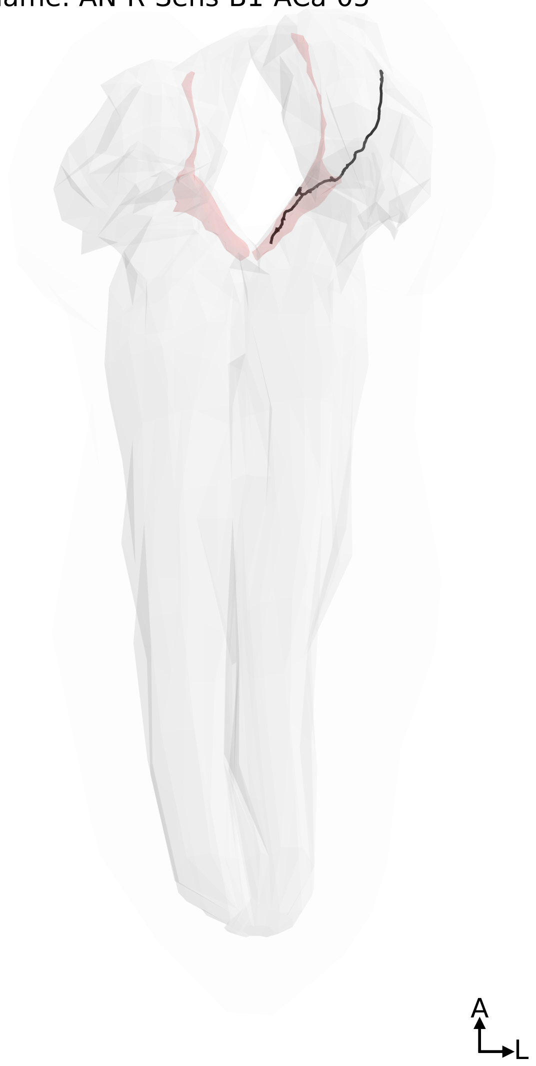

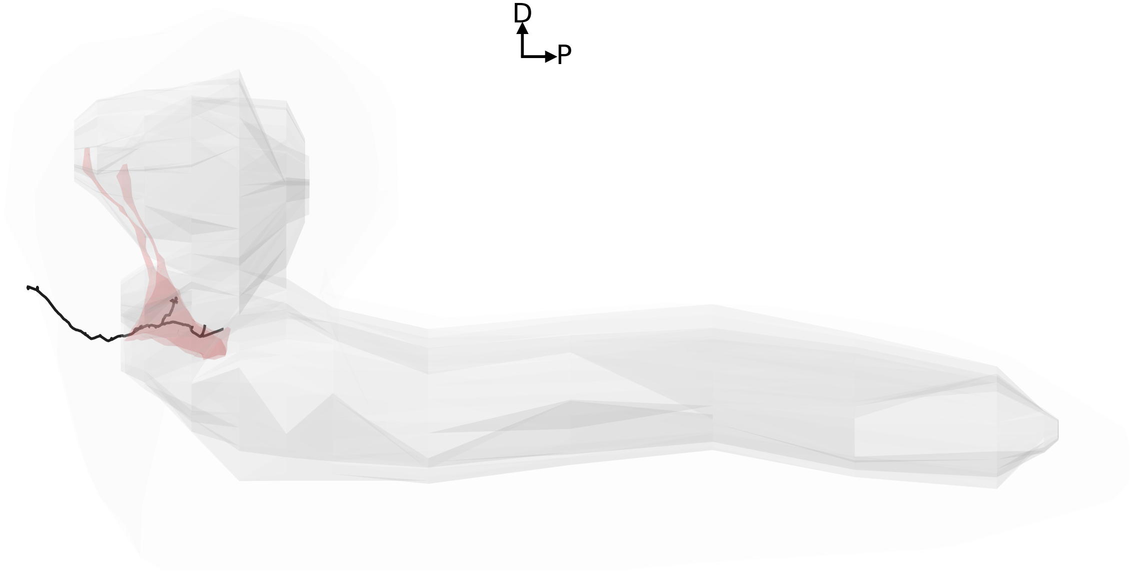

ID	*name*	SCACa	SCAVa	SCAVp	SCACal	SCACp	SCACpl	SCVM	IPCs	DMS	DH44	Se0ens	Se0ph	PMN LR	MN motor neurons	PaN motor neurons	olfactory PNs	gustatory PNs	multiglomerular PNs	unknown PNs	thermo PNs	visual PNs
15563553	AN-R-Sens-B1-ACa-05	0	0	0	0	0	0	0	1	0	0	2	0	0	0	0	0	0	0	0	0	0

ID: 15574199
name: AN-R-Sens-B1-ACa-06

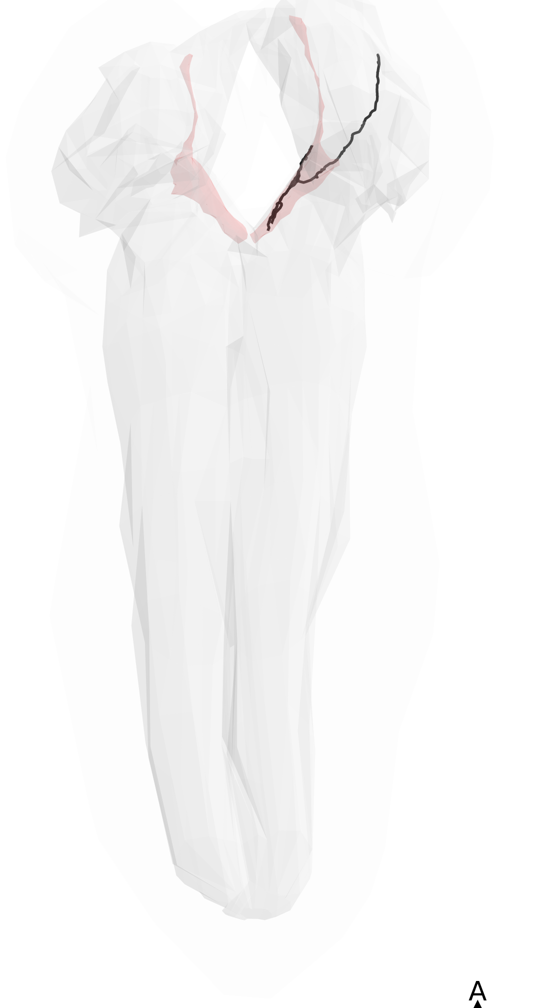

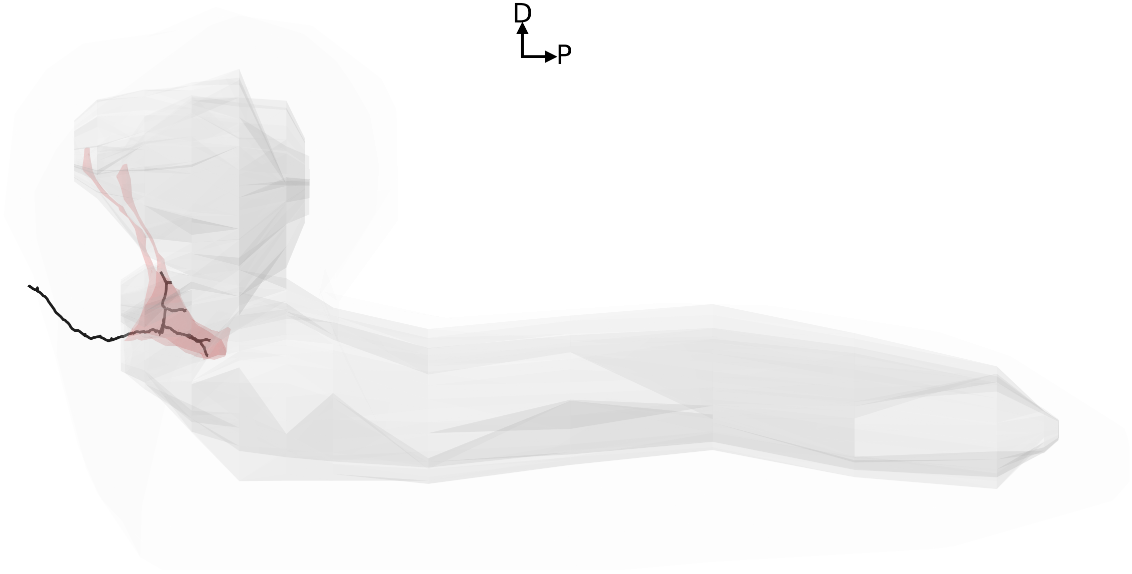

ID	*name*	SCACa	SCAVa	SCAVp	SCACal	SCACp	SCACpl	SCVM	IPCs	DMS	DH44	Se0ens	Se0ph	PMN LR	MN motor neurons	PaN motor neurons	olfactory PNs	gustatory PNs	multiglomerular PNs	unknown PNs	thermo PNs	visual PNs
15574199	AN-R-Sens-B1-ACa-06	1	0	0	0	0	0	0	0	0	1	0	0	0	0	0	0	0	0	0	0	0

ID: 15562137
name: AN-R-Sens-B1-ACa-07

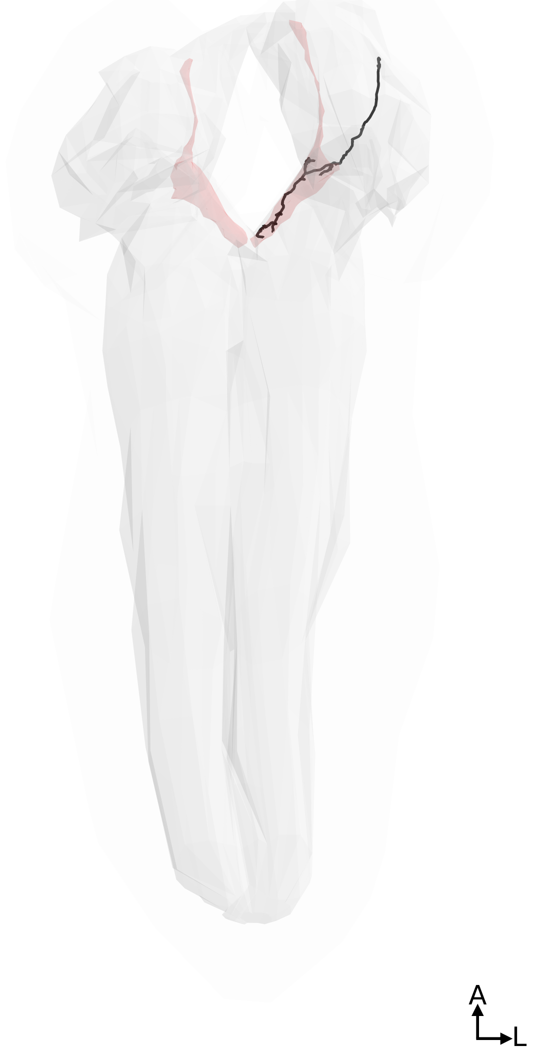

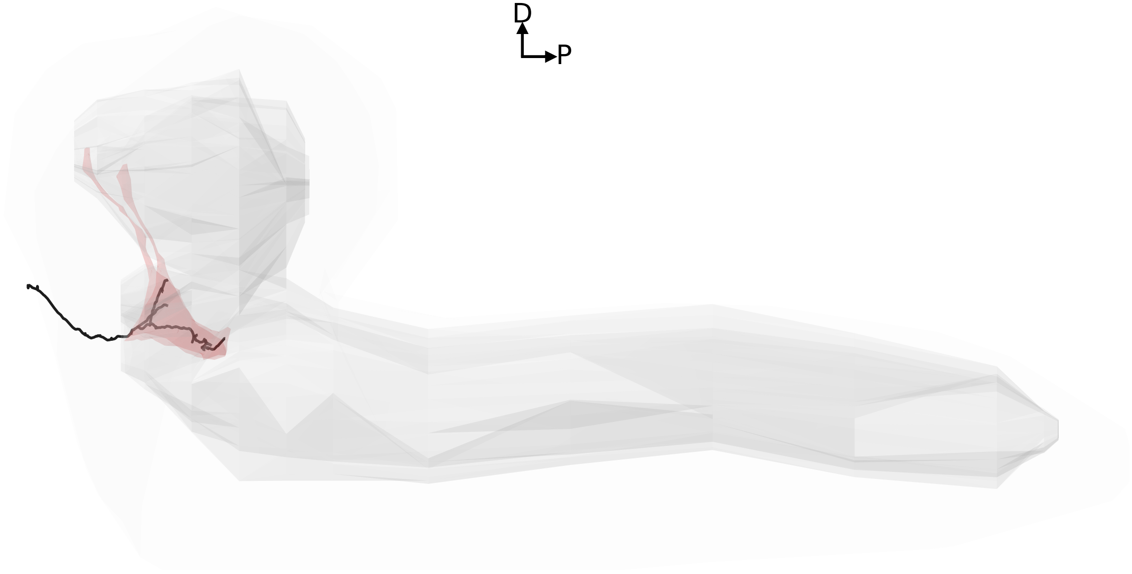

ID	*name*	SCACa	SCAVa	SCAVp	SCACal	SCACp	SCACpl	SCVM	IPCs	DMS	DH44	Se0ens	Se0ph	PMN LR	MN motor neurons	PaN motor neurons	olfactory PNs	gustatory PNs	multiglomerular PNs	unknown PNs	thermo PNs	visual PNs
15562137	AN-R-Sens-B1-ACa-07	3	2	0	0	0	0	0	0	0	3	1	0	0	0	0	0	0	0	0	0	0

ID: 15574231
name: AN-R-Sens-B1-ACa-08

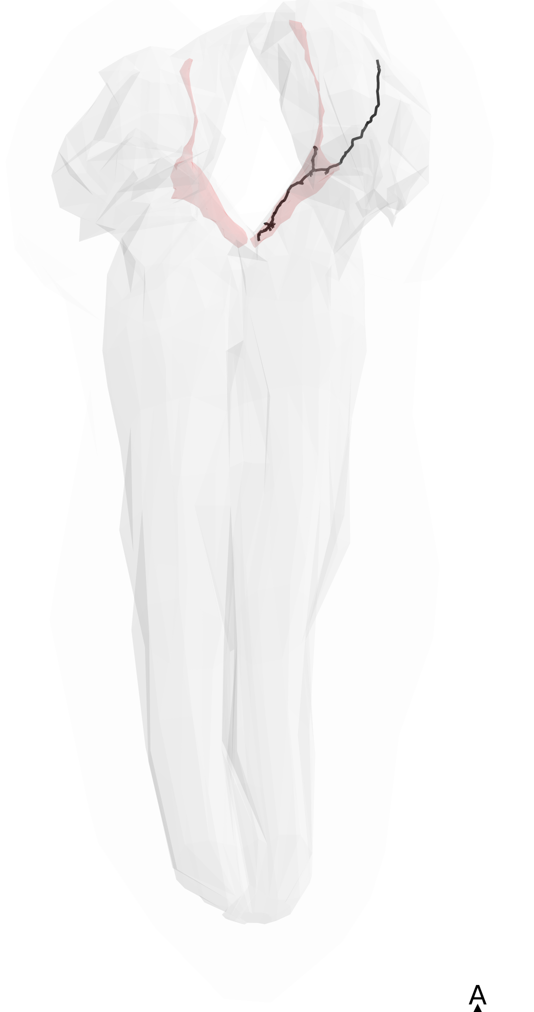

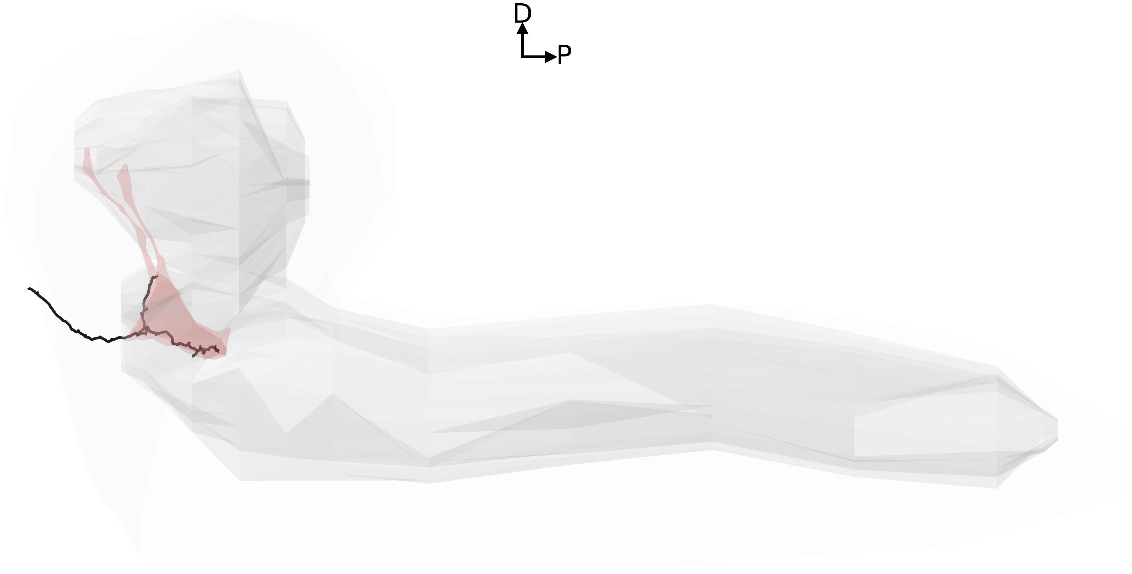

ID	*name*	SCACa	SCAVa	SCAVp	SCACal	SCACp	SCACpl	SCVM	IPCs	DMS	DH44	Se0ens	Se0ph	PMN LR	MN motor neurons	PaN motor neurons	olfactory PNs	gustatory PNs	multiglomerular PNs	unknown PNs	thermo PNs	visual PNs
15574231	AN-R-Sens-B1-ACa-08	11	0	0	0	0	0	0	1	3	0	2	0	0	0	0	0	0	0	0	0	0

ID: 15573396
name: AN-R-Sens-B1-ACa-09

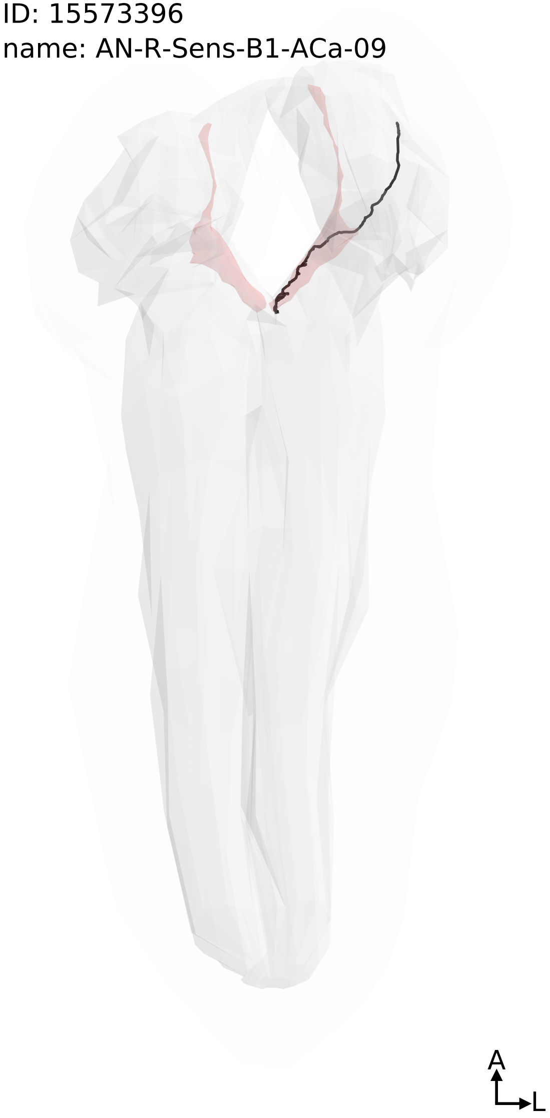

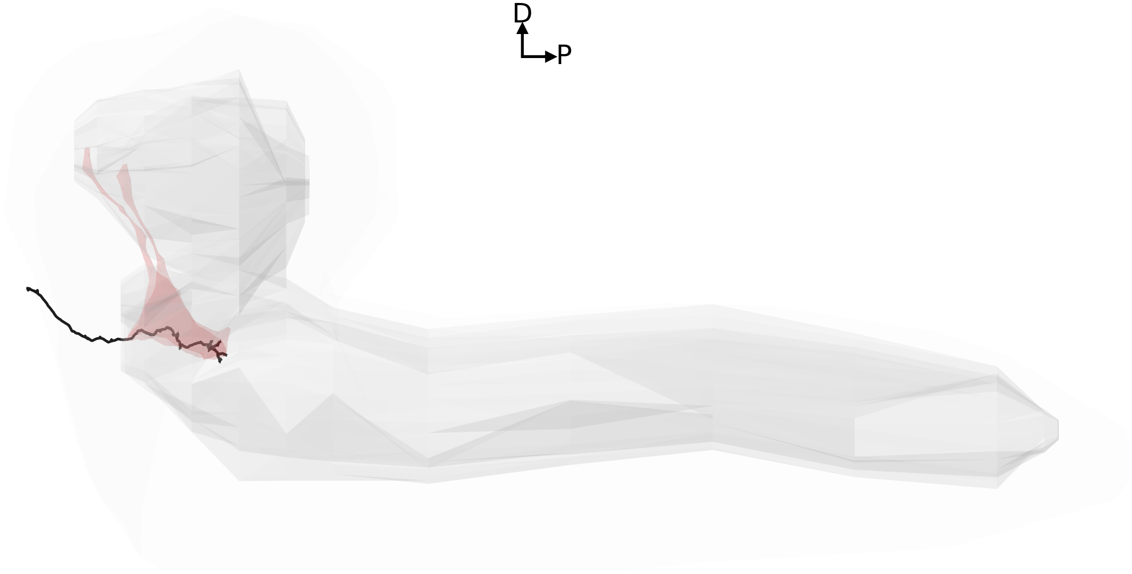

ID	name	SCACa	SCAVa	SCAVp	SCACal	SCACp	SCACpl	SCVM	IPCs	DMS	DH44	Se0ens	Se0ph	PMN LR	MN motor neurons	PaN motor neurons	olfactory PNs	gustatory PNs	multiglomerular PNs	unknown PNs	thermo PNs	visual PNs
15573396	AN-R-Sens-B1-ACa-09	6	0	0	0	0	0	0	0	5	0	0	0	0	0	0	0	0	0	0	0	0

ID: 15548694
name: AN-R-Sens-B1-ACa-10

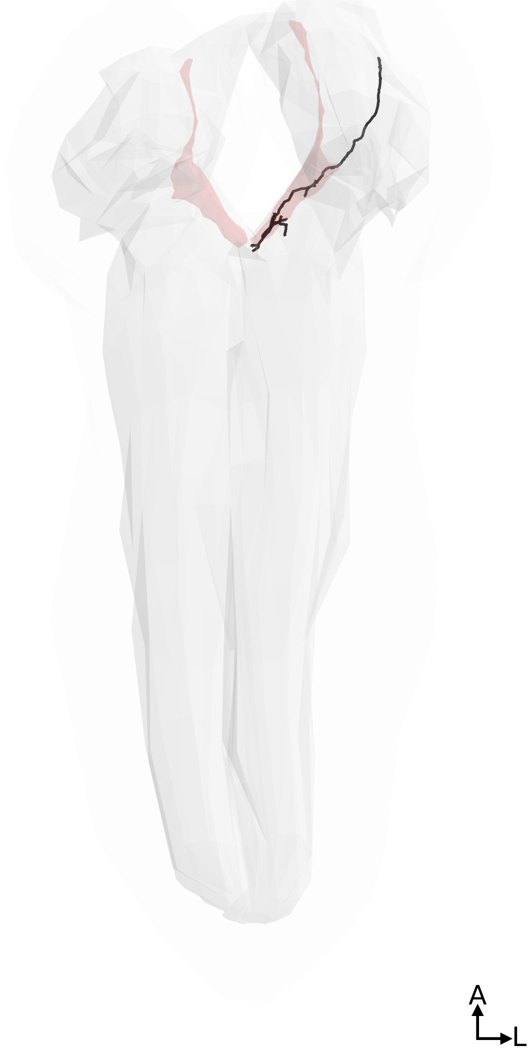

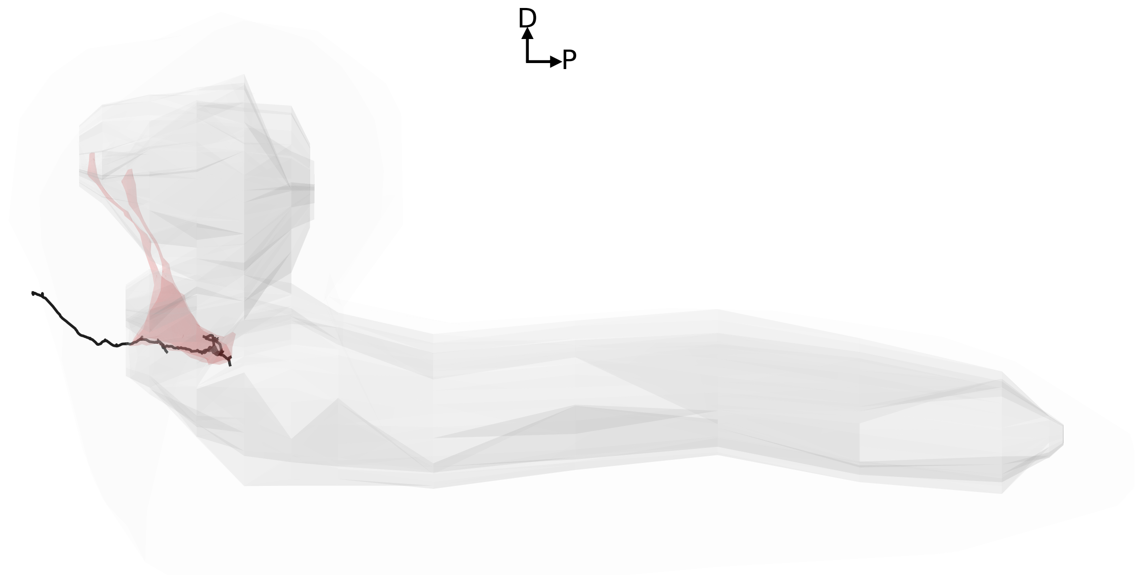

ID	*name*	SCACa	SCAVa	SCAVp	SCACal	SCACp	SCACpl	SCVM	IPCs	DMS	DH44	Se0ens	Se0ph	PMN LR	MN motor neurons	PaN motor neurons	olfactory PNs	gustatory PNs	multiglomerular PNs	unknown PNs	thermo PNs	visual PNs
15548694	AN-R-Sens-B1-ACa-10	2	0	0	0	0	0	0	0	1	1	2	0	0	0	0	0	0	0	0	0	0

ID: 15586536
name: AN-R-Sens-B1-ACa-11

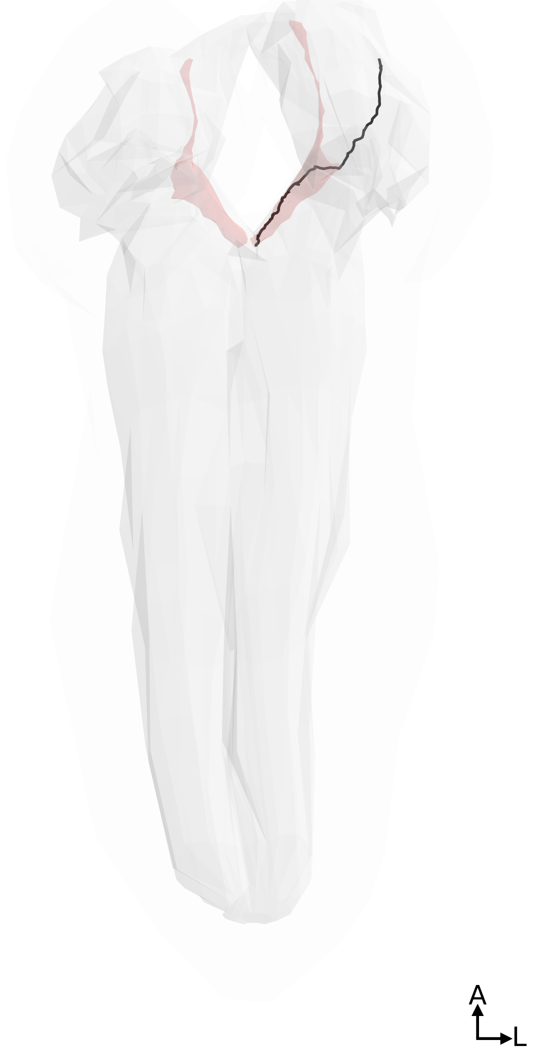

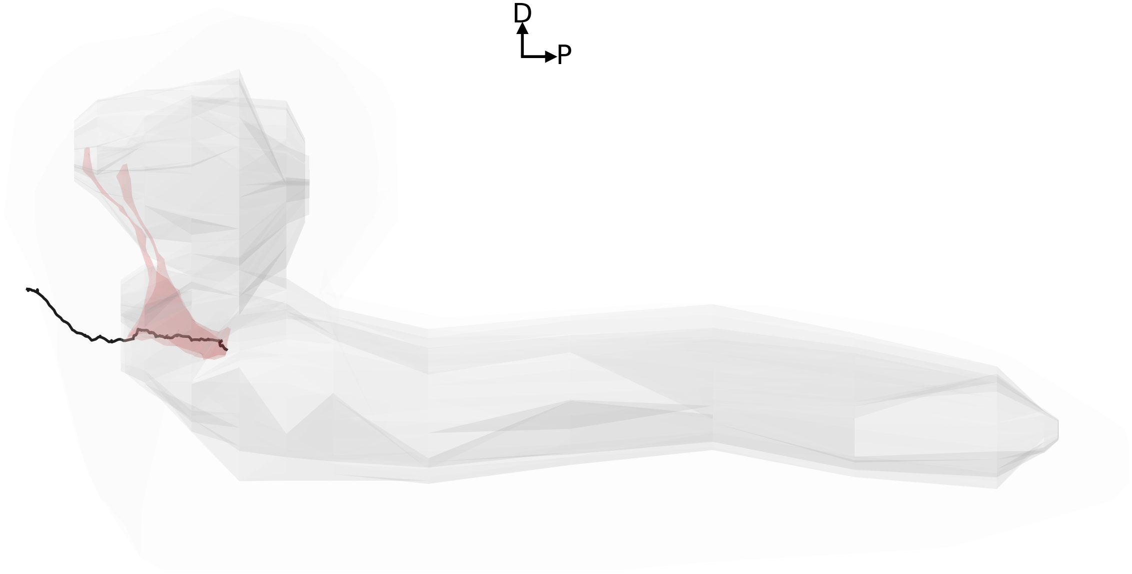

ID	*name*	SCACa	SCAVa	SCAVp	SCACal	SCACp	SCACpl	SCVM	IPCs	DMS	DH44	Se0ens	Se0ph	PMN LR	MN motor neurons	PaN motor neurons	olfactory PNs	gustatory PNs	multiglomerular PNs	unknown PNs	thermo PNs	visual PNs
15586536	AN-R-Sens-B1-ACa-11	2	0	0	0	0	0	0	0	2	1	0	0	0	0	0	0	0	0	0	0	0

ID: 15573381
name: AN-R-Sens-B1-ACa-12

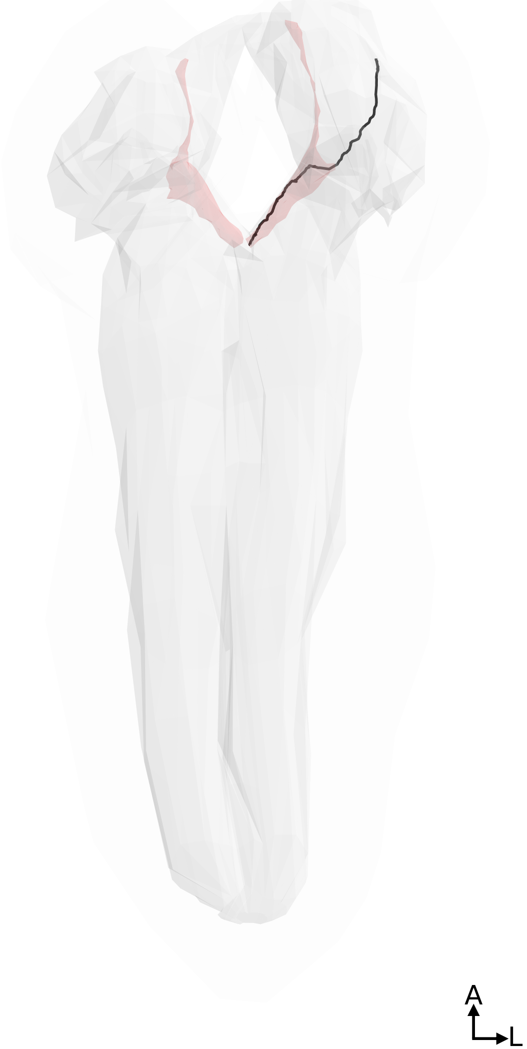

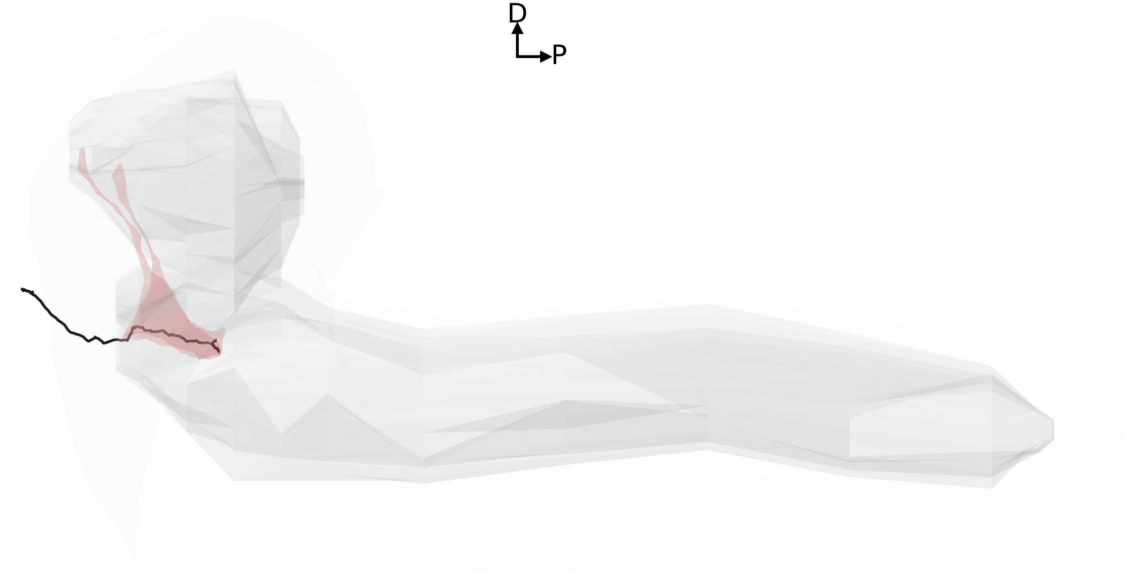

ID	*name*	SCACa	SCAVa	SCAVp	SCACal	SCACp	SCACpl	SCVM	IPCs	DMS	DH44	Se0ens	Se0ph	PMN LR	MN motor neurons	PaN motor neurons	olfactory PNs	gustatory PNs	multiglomerular PNs	unknown PNs	thermo PNs	visual PNs
15573381	AN-R-Sens-B1-ACa-12	4	0	0	0	0	0	0	0	0	0	0	0	0	0	0	0	0	0	0	0	0

ID: 15561413
name: AN-R-Sens-B1-ACa-13

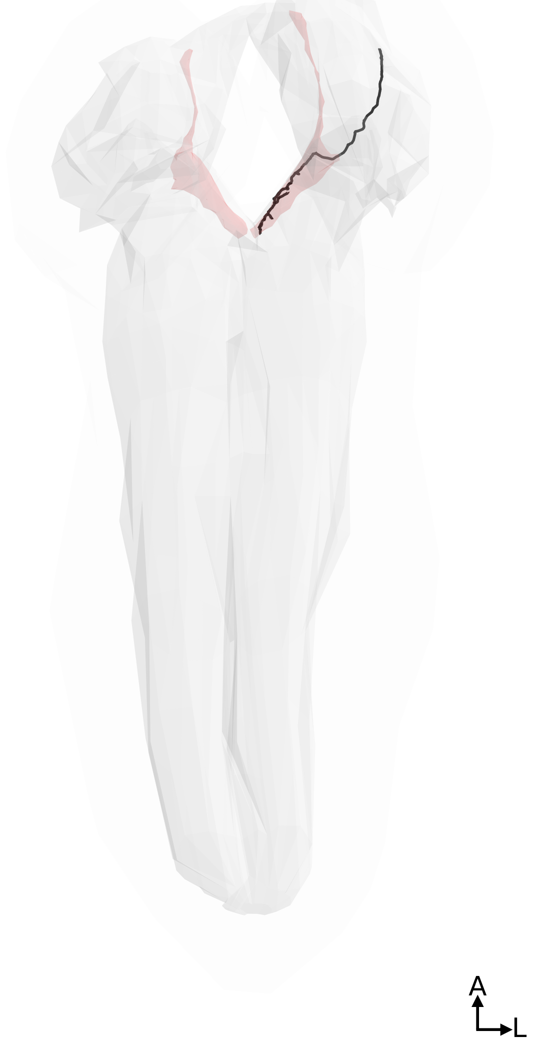

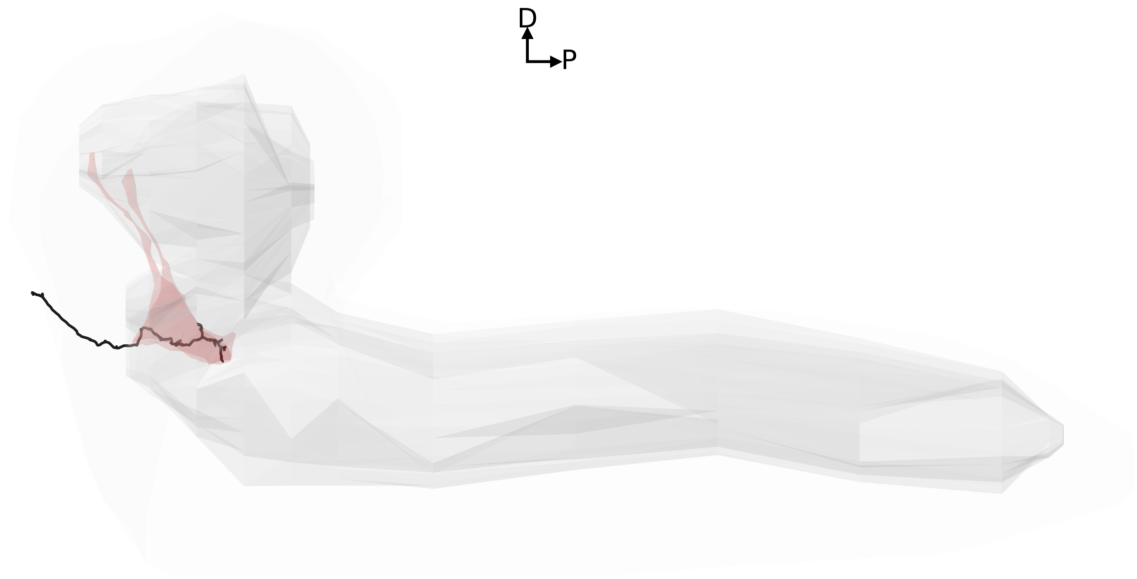

ID	name	SCACa	SCAVa	SCAVp	SCACal	SCACp	SCACpl	SCVM	IPCs	DMS	DH44	Se0ens	Se0ph	PMN LR	MN motor neurons	PaN motor neurons	olfactory PNs	gustatory PNs	multiglomerular PNs	unknown PNs	thermo PNs	visual PNs
15561413	AN-R-Sens-B1-ACa-13	4	0	0	0	0	0	0	0	1	2	0	0	0	0	0	0	0	0	0	0	0

ID: 15573450
name: AN-R-Sens-B1-ACa-14

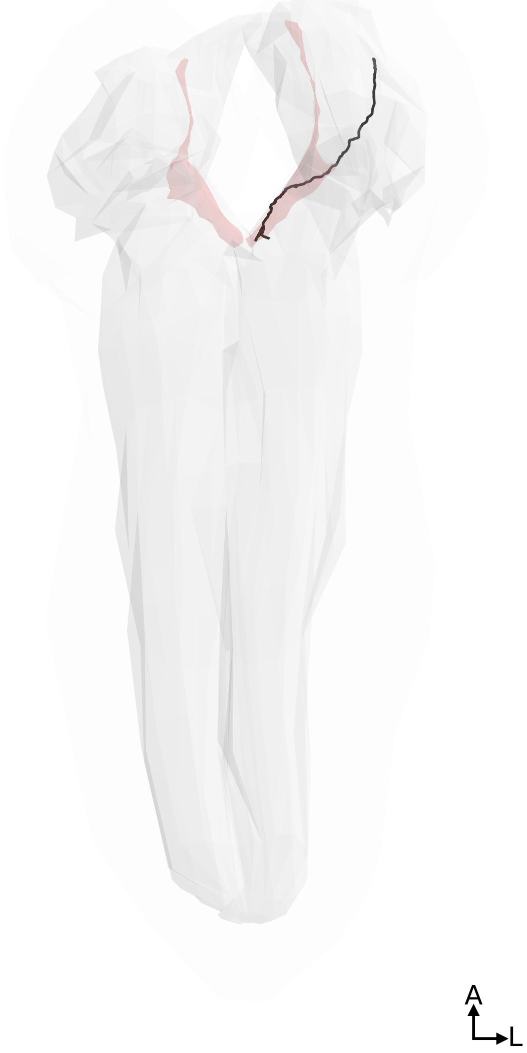

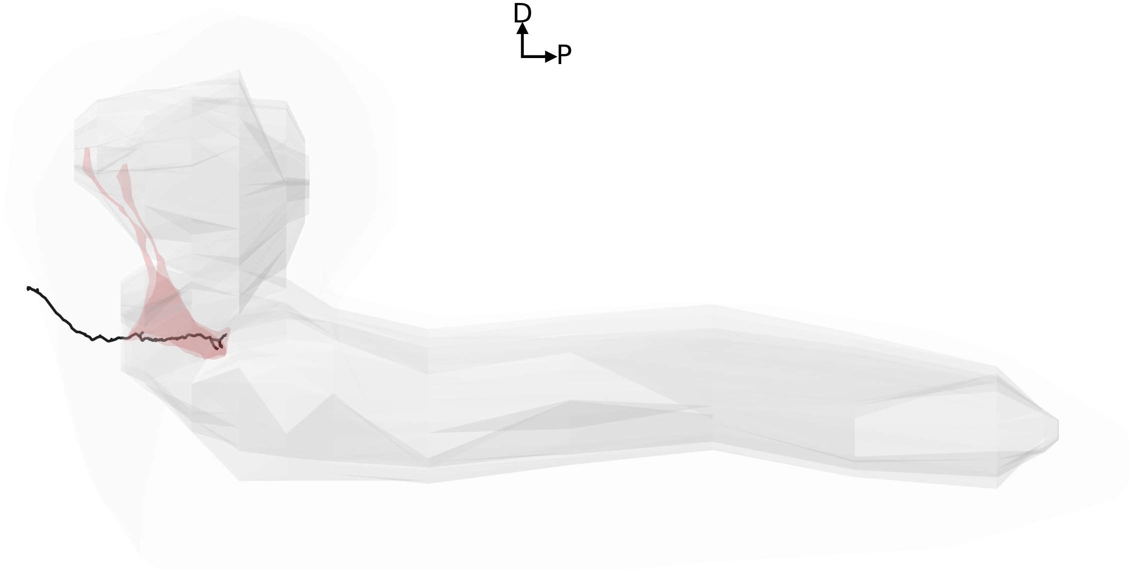

ID	*name*	SCACa	SCAVa	SCAVp	SCACal	SCACp	SCACpl	SCVM	IPCs	DMS	DH44	Se0ens	Se0ph	PMN LR	MN motor neurons	PaN motor neurons	olfactory PNs	gustatory PNs	multiglomerular PNs	unknown PNs	thermo PNs	visual PNs
15573450	AN-R-Sens-B1-ACa-14	2	1	0	0	0	0	0	0	1	2	0	0	0	0	0	0	0	0	0	0	0

ID: 15548107
name: AN-R-Sens-B1-ACa-17

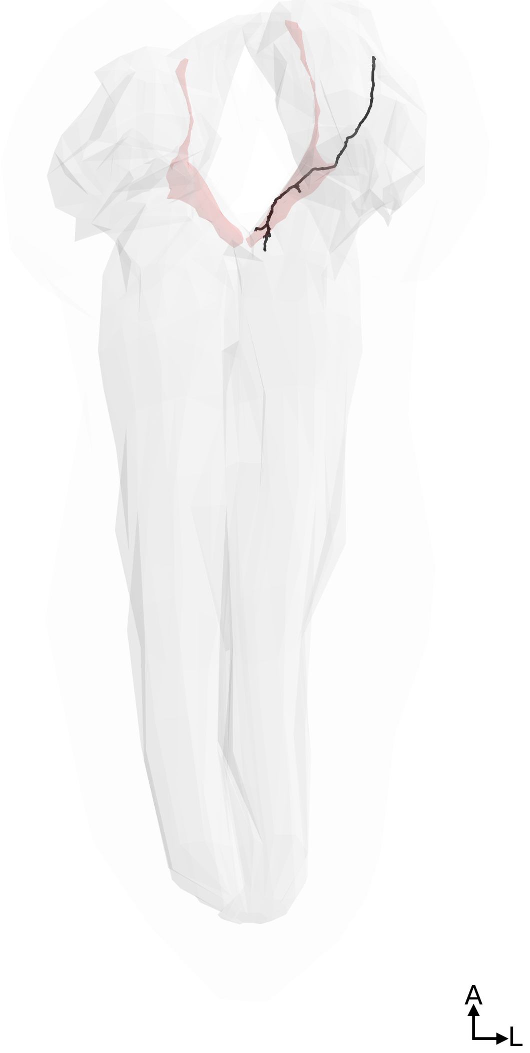

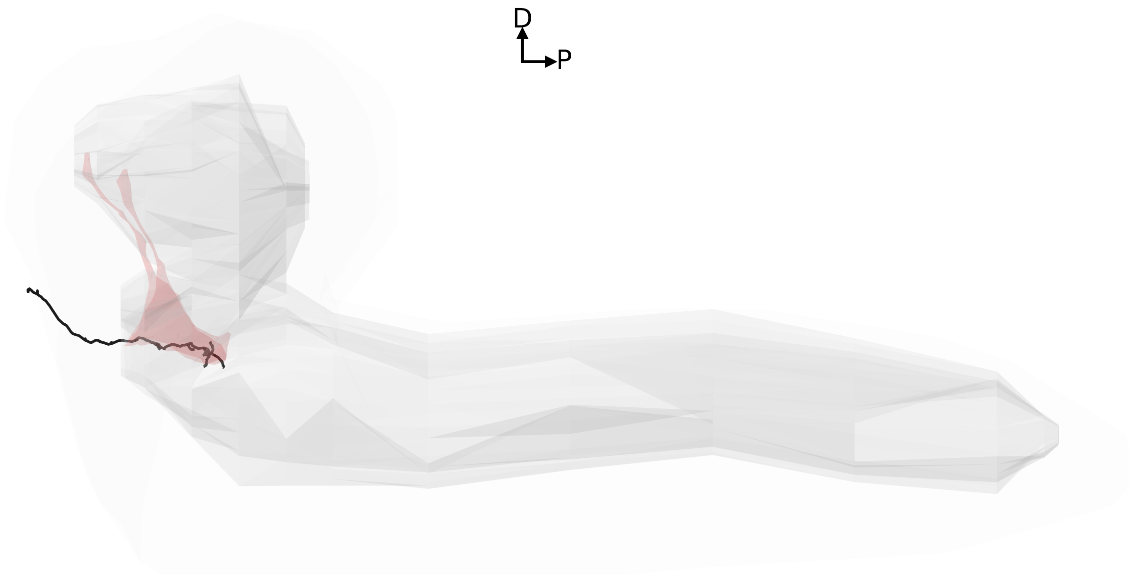

ID	*name*	SCACa	SCAVa	SCAVp	SCACal	SCACp	SCACpl	SCVM	IPCs	DMS	DH44	Se0ens	Se0ph	PMN LR	MN motor neurons	PaN motor neurons	olfactory PNs	gustatory PNs	multiglomerular PNs	unknown PNs	thermo PNs	visual PNs
15548107	AN-R-Sens-B1-ACa-17	2	1	0	0	0	0	0	0	1	0	3	0	0	0	0	0	0	0	0	0	0

ID: 1414206
name: AN-L-Sens-B1-AVa-16

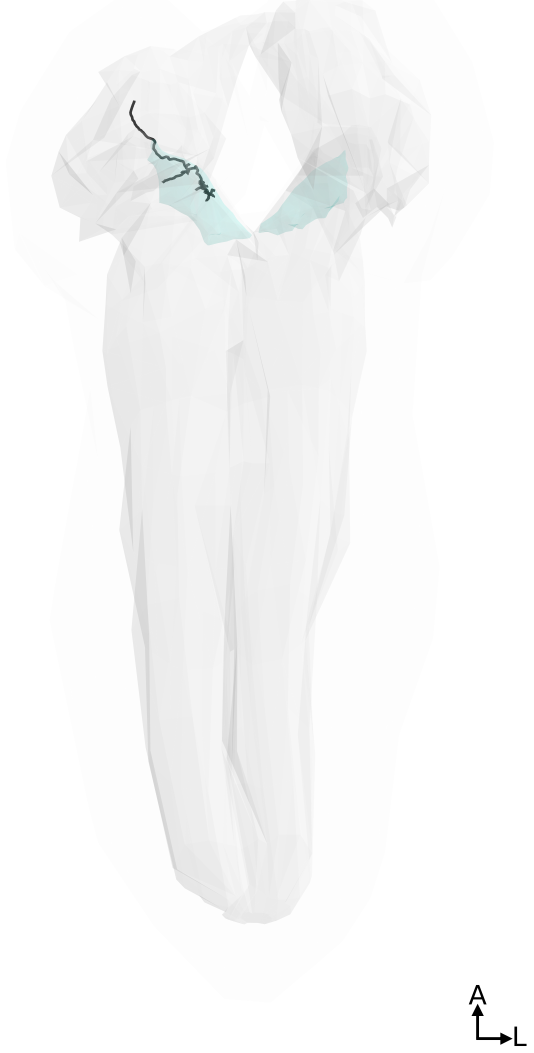

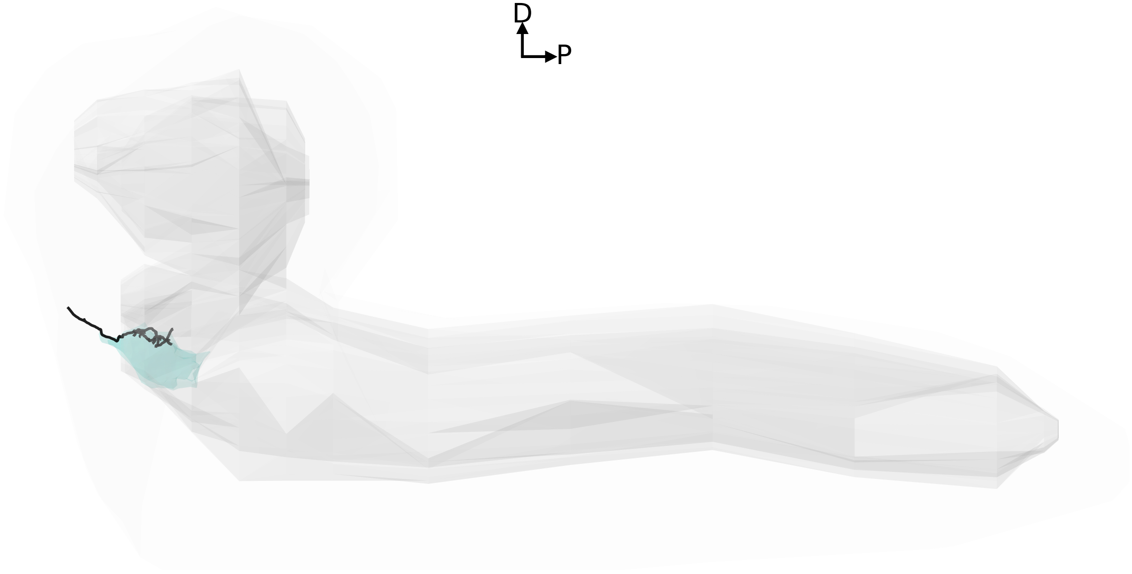

ID	*name*	SCACa	SCAVa	SCAVp	SCACal	SCACp	SCACpl	SCVM	IPCs	DMS	DH44	Se0ens	Se0ph	PMN LR	MN motor neurons	PaN motor neurons	olfactory PNs	gustatory PNs	multiglomerular PNs	unknown PNs	thermo PNs	visual PNs
1414206	AN-L-Sens-B1-AVa-16	2	4	0	0	2	0	0	3	0	0	20	0	0	0	0	0	0	0	0	0	0

ID: 1375101
name: AN-L-Sens-B1-AVa-17

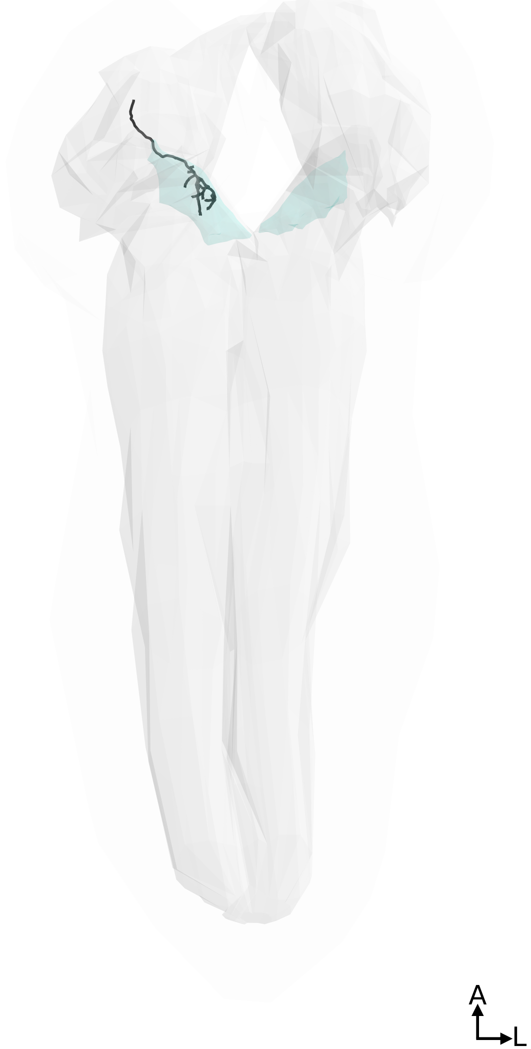

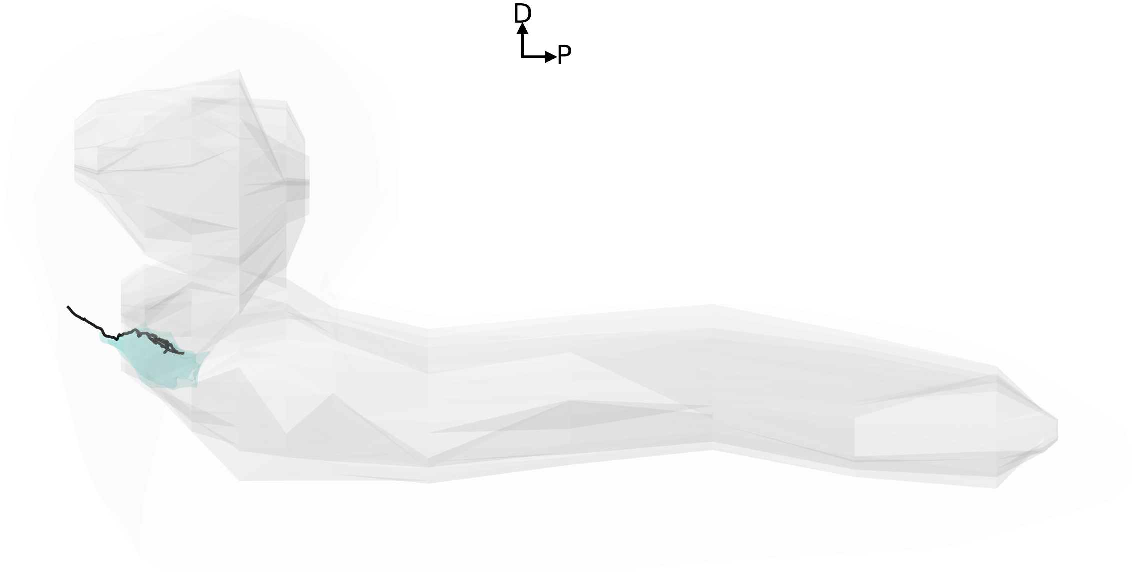

ID	name	SCACa	SCAVa	SCAVp	SCACal	SCACp	SCACpl	SCVM	IPCs	DMS	DH44	Se0ens	Se0ph	PMN LR	MN motor neurons	PaN motor neurons	olfactory PNs	gustatory PNs	multiglomerular PNs	unknown PNs	thermo PNs	visual PNs
1375101	AN-L-Sens-B1-AVa-17	0	5	0	0	0	0	0	1	0	0	26	0	0	0	0	0	0	0	0	0	0

ID: 1432836
name: AN-L-Sens-B1-AVa-18

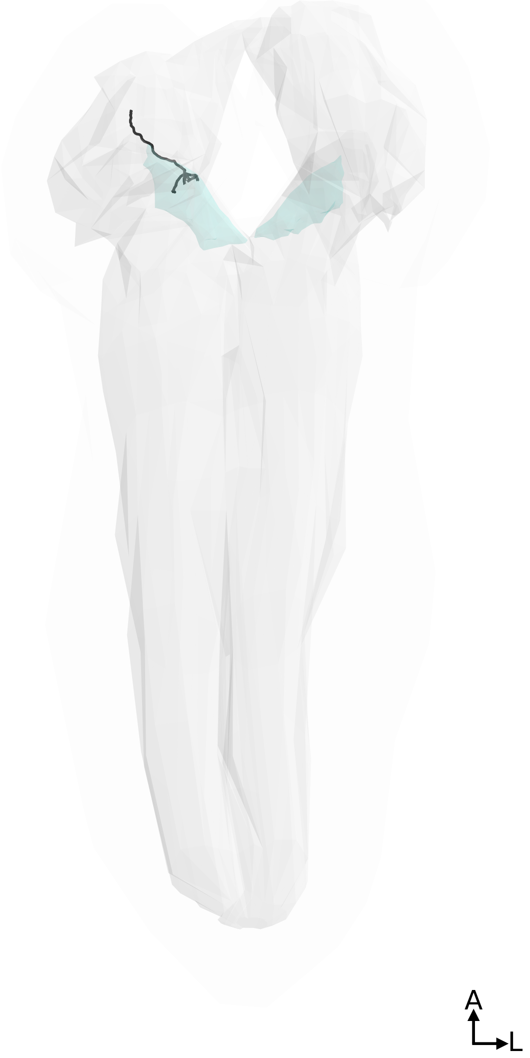

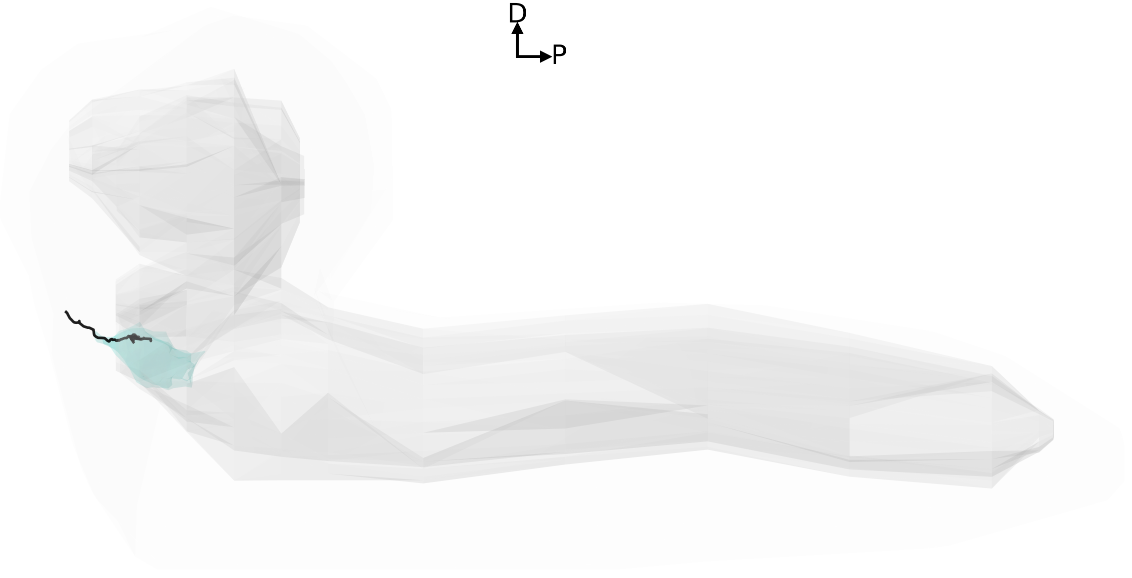

ID	*name*	SCACa	SCAVa	SCAVp	SCACal	SCACp	SCACpl	SCVM	IPCs	DMS	DH44	Se0ens	Se0ph	PMN LR	MN motor neurons	PaN motor neurons	olfactory PNs	gustatory PNs	multiglomerular PNs	unknown PNs	thermo PNs	visual PNs
1432836	AN-L-Sens-B1-AVa-18	0	1	0	0	0	0	0	0	0	0	5	2	0	0	0	0	0	0	0	0	0

ID: 15769680
name: AN-L-Sens-B1-AVa-19

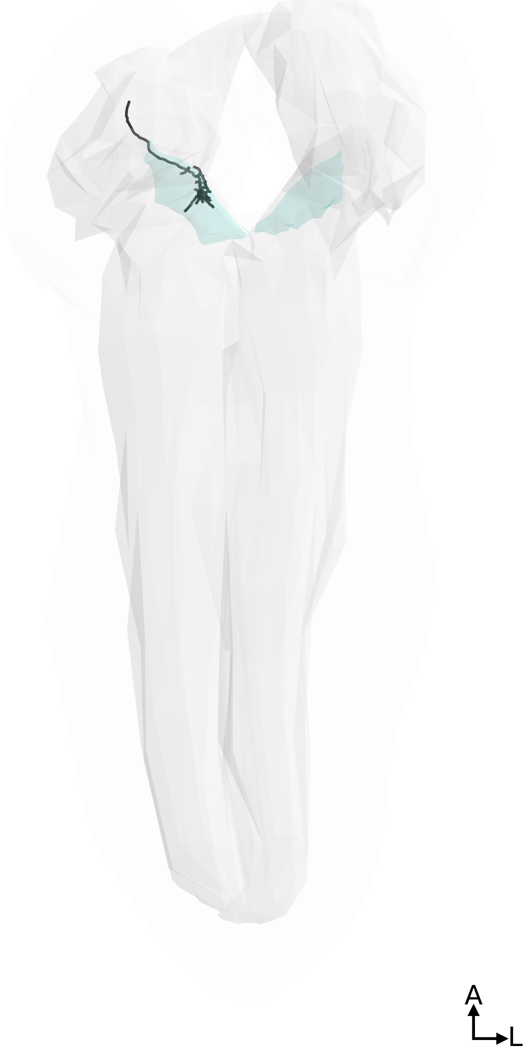

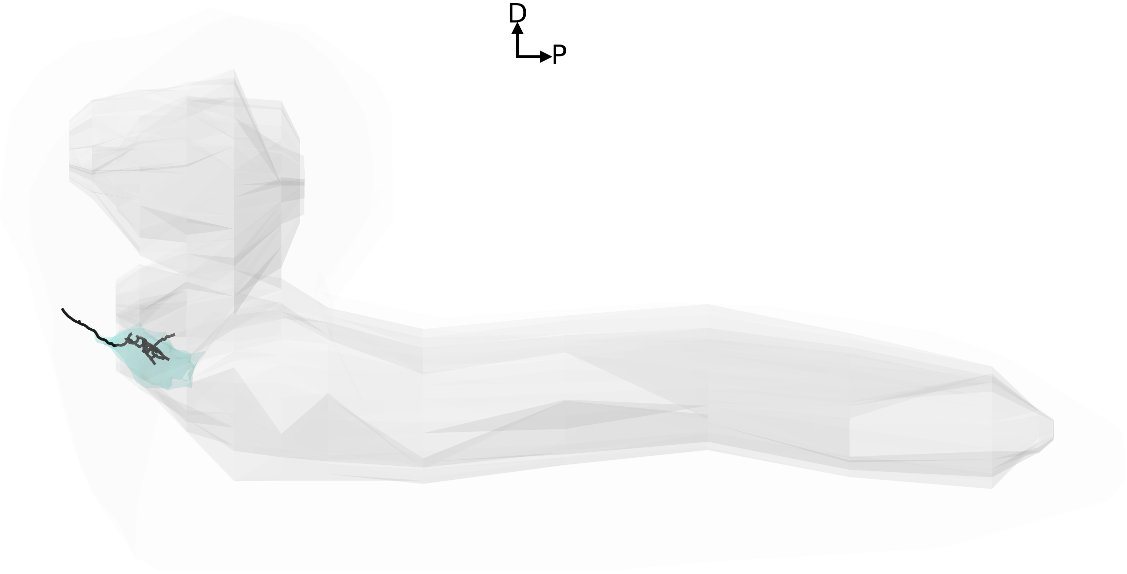

ID	*name*	SCACa	SCAVa	SCAVp	SCACal	SCACp	SCACpl	SCVM	IPCs	DMS	DH44	Se0ens	Se0ph	PMN LR	MN motor neurons	PaN motor neurons	olfactory PNs	gustatory PNs	multiglomerular PNs	unknown PNs	thermo PNs	visual PNs
15769680	AN-L-Sens-B1-AVa-19	0	2	0	0	0	0	0	3	0	0	5	0	0	0	0	0	0	0	0	0	0

ID: 1429969
name: AN-L-Sens-B1-AVa-20

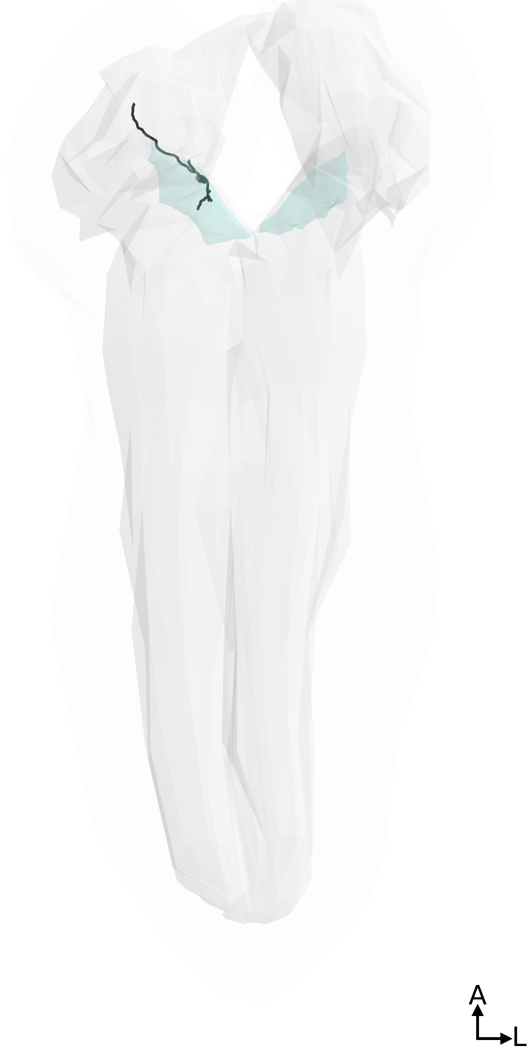

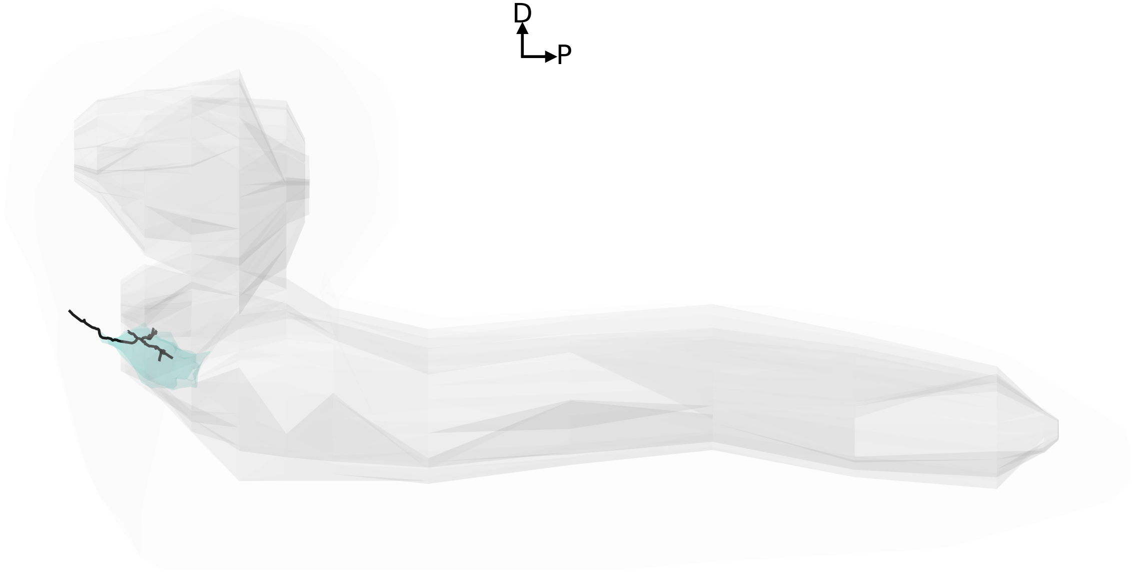

ID	*name*	SCACa	SCAVa	SCAVp	SCACal	SCACp	SCACpl	SCVM	IPCs	DMS	DH44	Se0ens	Se0ph	PMN LR	MN motor neurons	PaN motor neurons	olfactory PNs	gustatory PNs	multiglomerular PNs	unknown PNs	thermo PNs	visual PNs
1429969	AN-L-Sens-B1-AVa-20	0	0	0	0	0	0	0	1	0	0	3	0	0	0	0	0	0	0	0	0	0

ID: 1435466
name: AN-L-Sens-B1-AVa-21

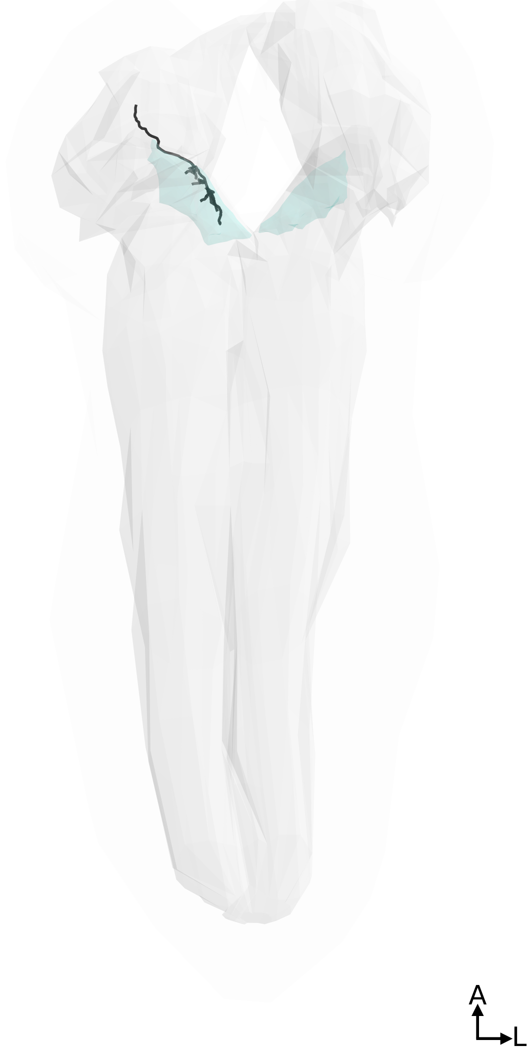

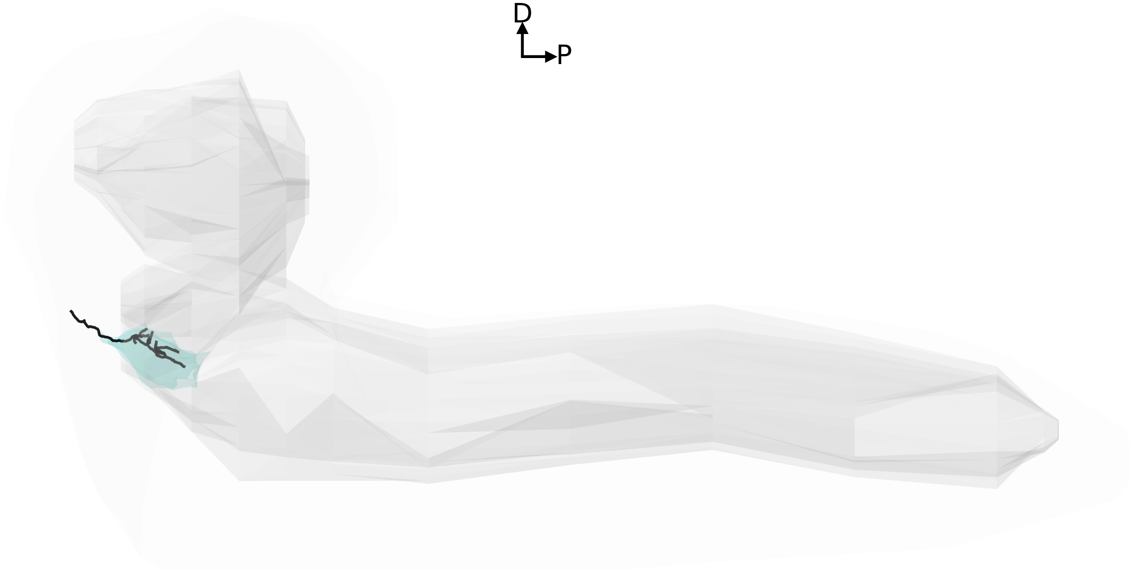

ID	*name*	SCACa	SCAVa	SCAVp	SCACal	SCACp	SCACpl	SCVM	IPCs	DMS	DH44	Se0ens	Se0ph	PMN LR	MN motor neurons	PaN motor neurons	olfactory PNs	gustatory PNs	multiglomerular PNs	unknown PNs	thermo PNs	visual PNs
1435466	AN-L-Sens-B1-AVa-21	0	3	0	0	0	0	0	0	0	0	8	2	0	0	0	0	0	0	0	0	0

ID: 1428271
name: AN-L-Sens-B1-AVa-22

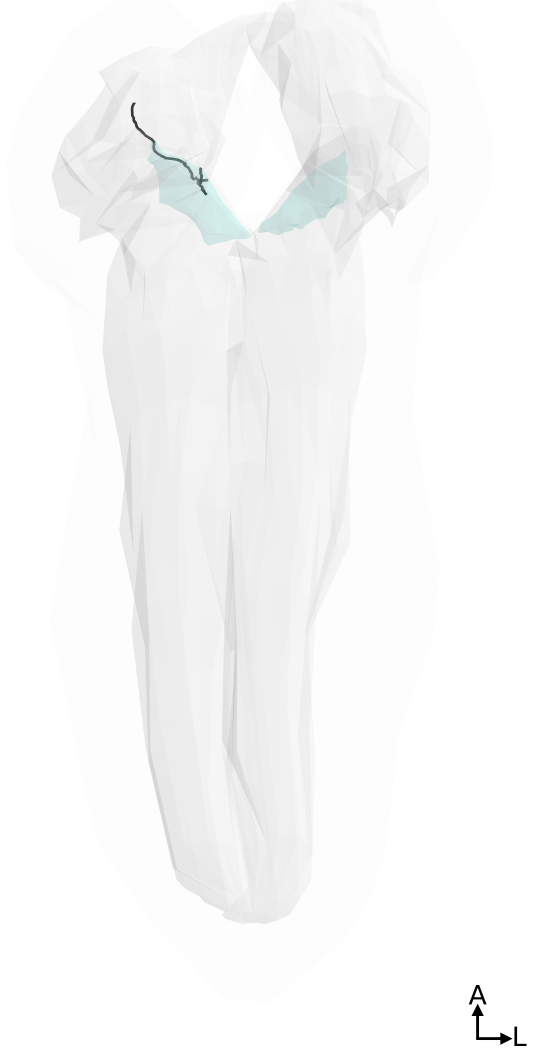

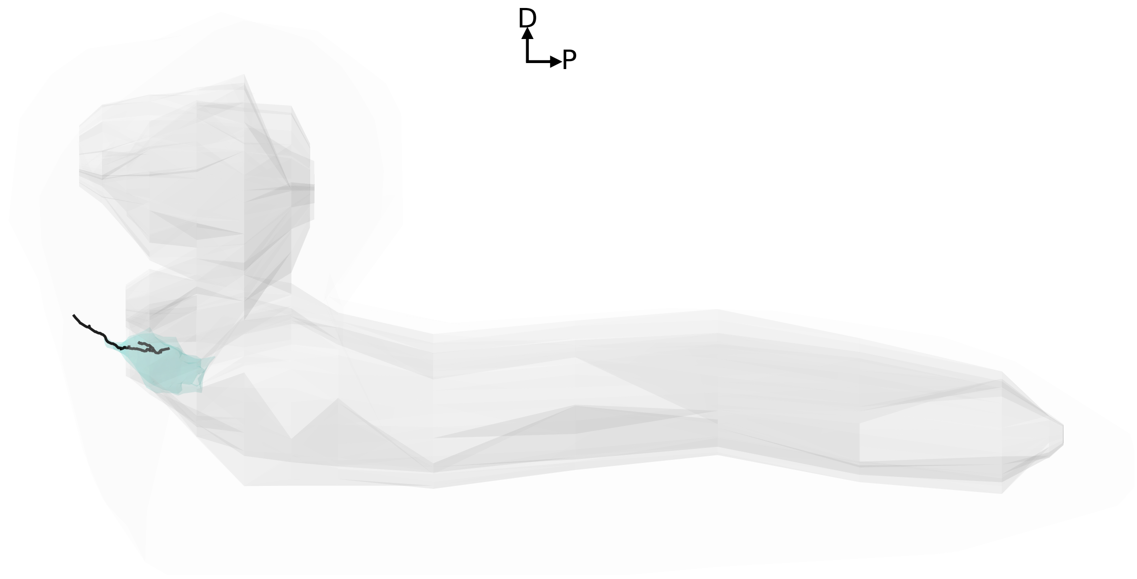

ID	*name*	SCACa	SCAVa	SCAVp	SCACal	SCACp	SCACpl	SCVM	IPCs	DMS	DH44	Se0ens	Se0ph	PMN LR	MN motor neurons	PaN motor neurons	olfactory PNs	gustatory PNs	multiglomerular PNs	unknown PNs	thermo PNs	visual PNs
1428271	AN-L-Sens-B1-AVa-22	0	8	0	0	0	0	0	0	0	0	2	1	5	0	0	0	0	0	0	0	0

ID: 15984797
name: AN-L-Sens-B1-AVa-23

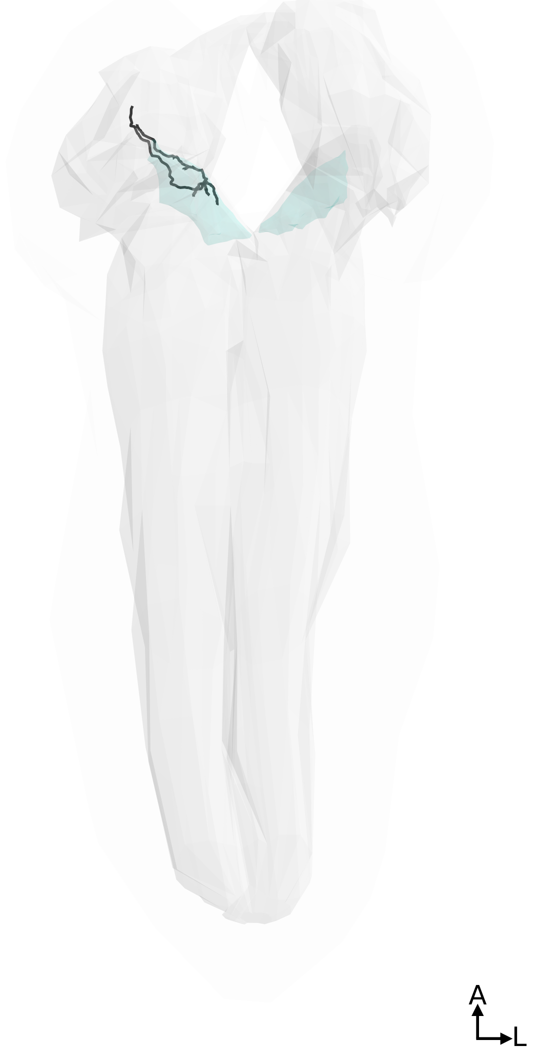

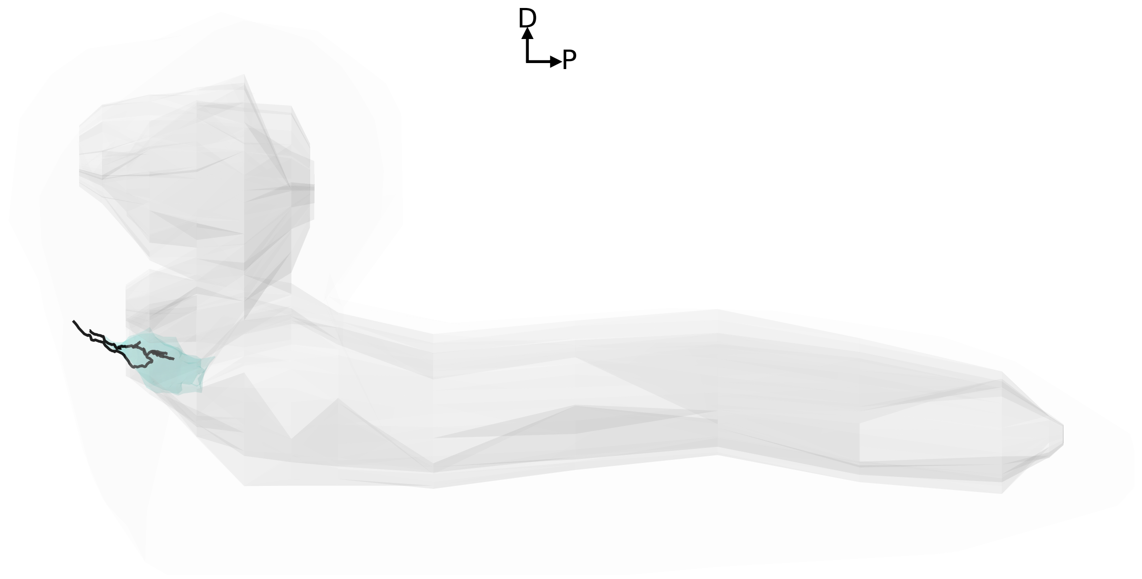

ID	*name*	SCACa	SCAVa	SCAVp	SCACal	SCACp	SCACpl	SCVM	IPCs	DMS	DH44	Se0ens	Se0ph	PMN LR	MN motor neurons	PaN motor neurons	olfactory PNs	gustatory PNs	multiglomerular PNs	unknown PNs	thermo PNs	visual PNs
15984797	AN-L-Sens-B1-AVa-23	0	7	0	0	0	0	0	0	0	0	0	5	3	0	0	0	0	0	0	0	0

ID: 15770904
name: AN-L-Sens-B1-AVa-24

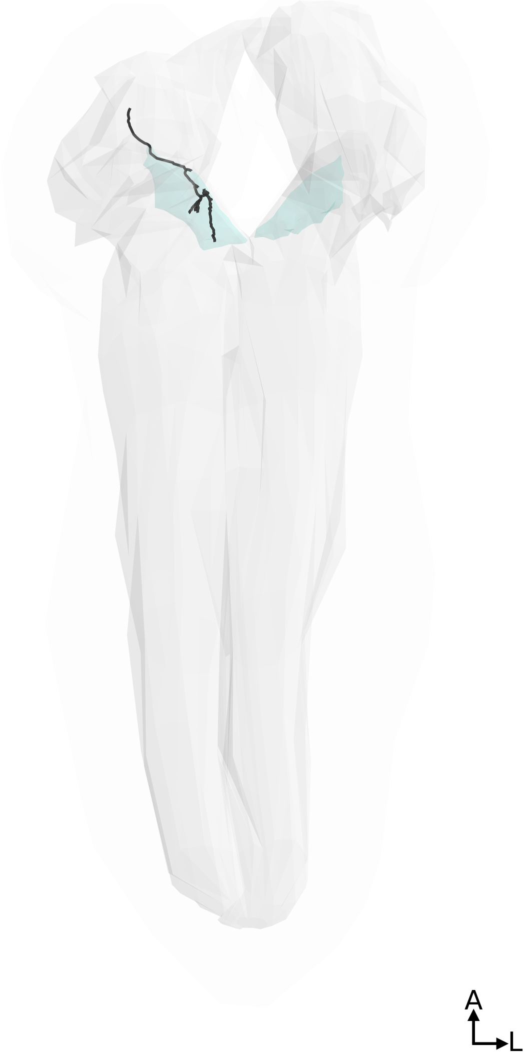

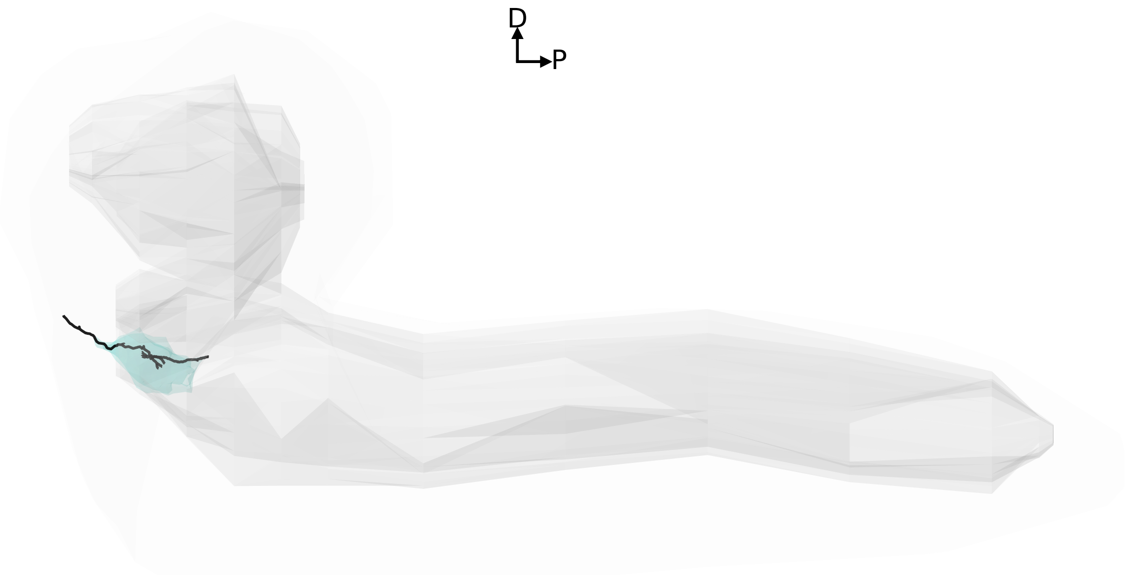

ID	*name*	SCACa	SCAVa	SCAVp	SCACal	SCACp	SCACpl	SCVM	IPCs	DMS	DH44	Se0ens	Se0ph	PMN LR	MN motor neurons	PaN motor neurons	olfactory PNs	gustatory PNs	multiglomerular PNs	unknown PNs	thermo PNs	visual PNs
15770904	AN-L-Sens-B1-AVa-24	0	4	0	0	0	0	0	1	0	2	6	0	2	0	0	0	0	0	0	0	0

ID: 15770945
name: AN-L-Sens-B1-AVa-25

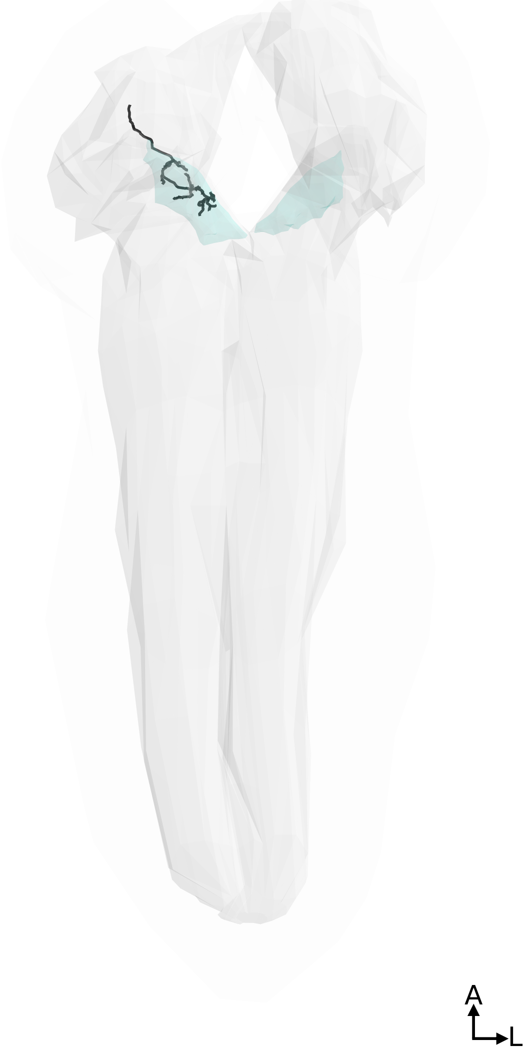

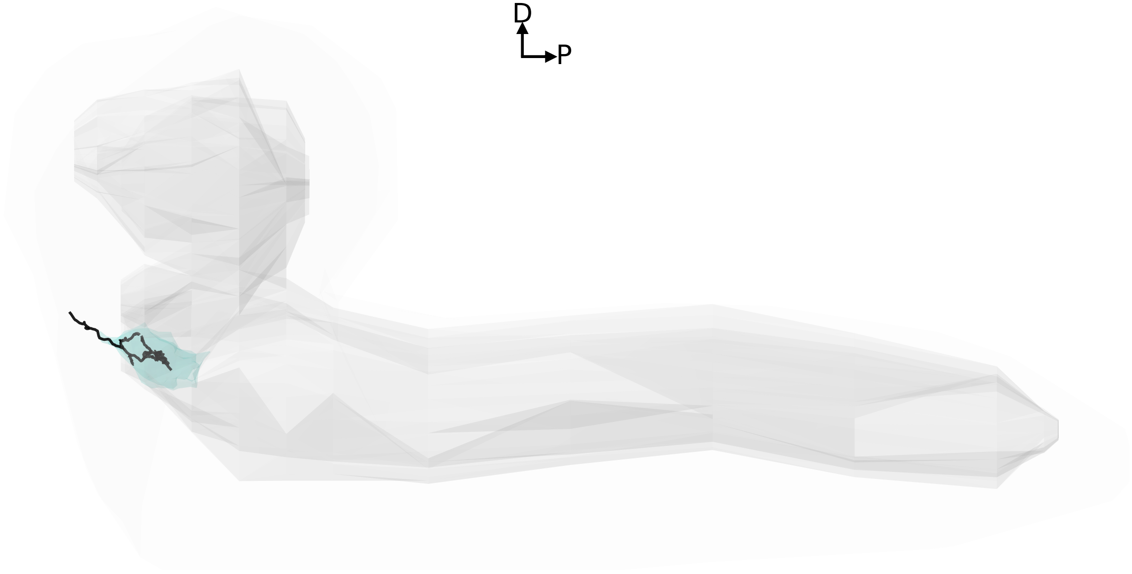

ID	*name*	SCACa	SCAVa	SCAVp	SCACal	SCACp	SCACpl	SCVM	IPCs	DMS	DH44	Se0ens	Se0ph	PMN LR	MN motor neurons	PaN motor neurons	olfactory PNs	gustatory PNs	multiglomerular PNs	unknown PNs	thermo PNs	visual PNs
15770945	AN-L-Sens-B1-AVa-25	0	3	0	0	0	0	0	0	0	0	3	1	0	0	0	0	0	0	0	0	0

ID: 1415168
name: AN-L-Sens-B1-AVa-26

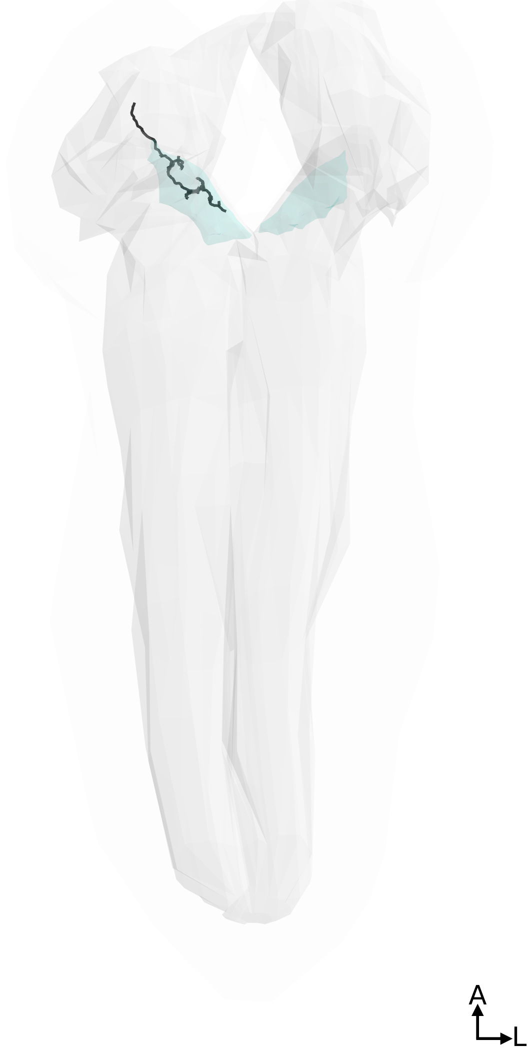

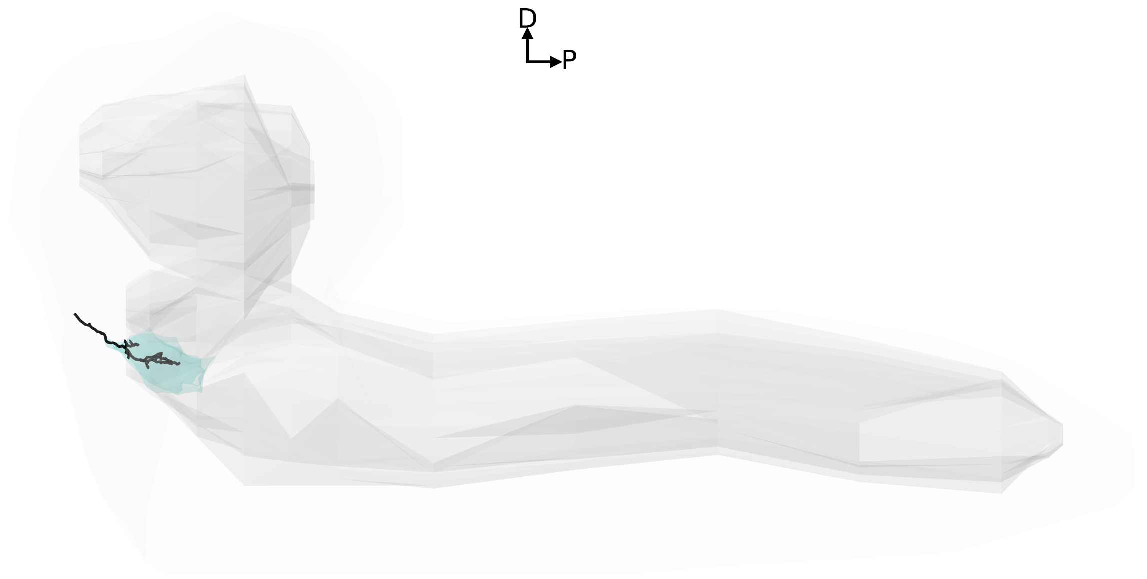

ID	name	SCACa	SCAVa	SCAVp	SCACal	SCACp	SCACpl	SCVM	IPCs	DMS	DH44	Se0ens	Se0ph	PMN LR	MN motor neurons	PaN motor neurons	olfactory PNs	gustatory PNs	multiglomerular PNs	unknown PNs	thermo PNs	visual PNs
1415168	AN-L-Sens-B1-AVa-26	0	17	0	0	0	0	0	0	0	0	2	0	2	0	0	0	0	0	0	0	0

ID: 1431256
name: AN-L-Sens-B1-AVa-27

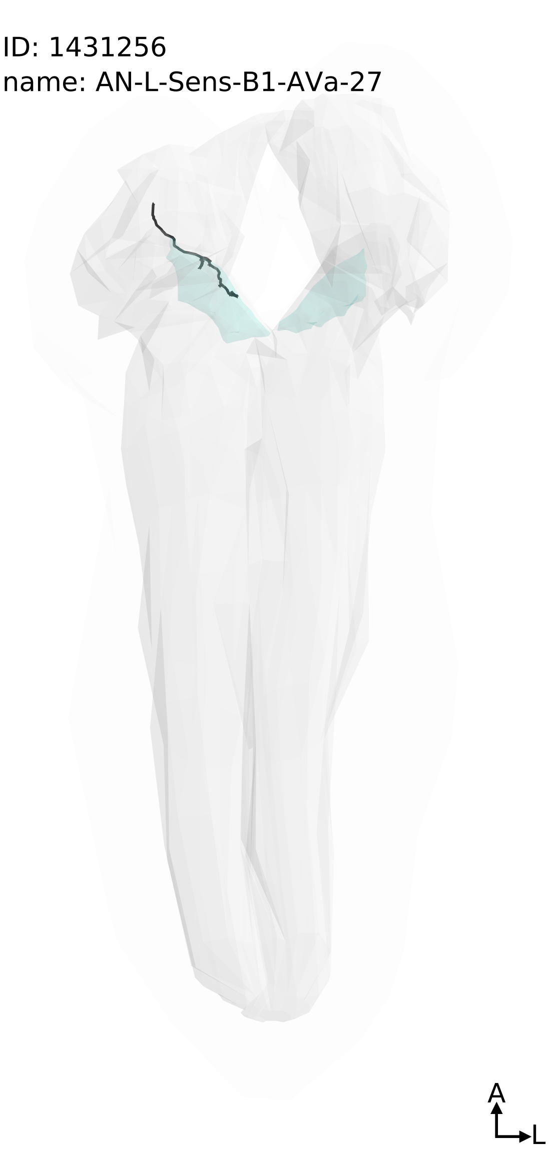

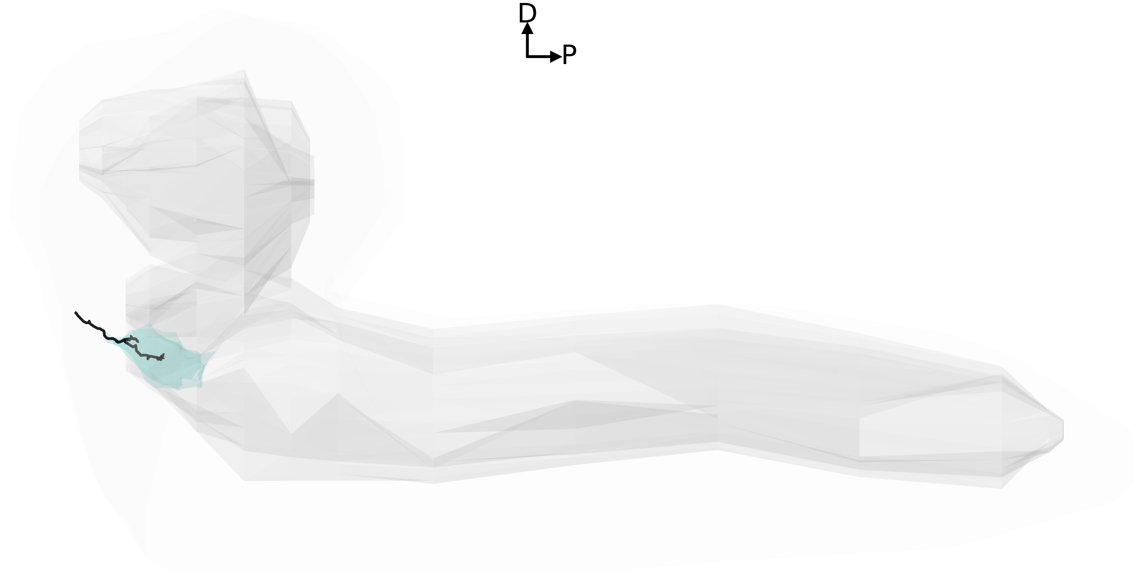

ID	*name*	SCACa	SCAVa	SCAVp	SCACal	SCACp	SCACpl	SCVM	IPCs	DMS	DH44	Se0ens	Se0ph	PMN LR	MN motor neurons	PaN motor neurons	olfactory PNs	gustatory PNs	multiglomerular PNs	unknown PNs	thermo PNs	visual PNs
1431256	AN-L-Sens-B1-AVa-27	0	5	0	0	0	0	0	0	0	0	1	0	0	0	0	0	0	0	0	0	0

ID: 15982306
name: AN-L-Sens-B1-AVa-28

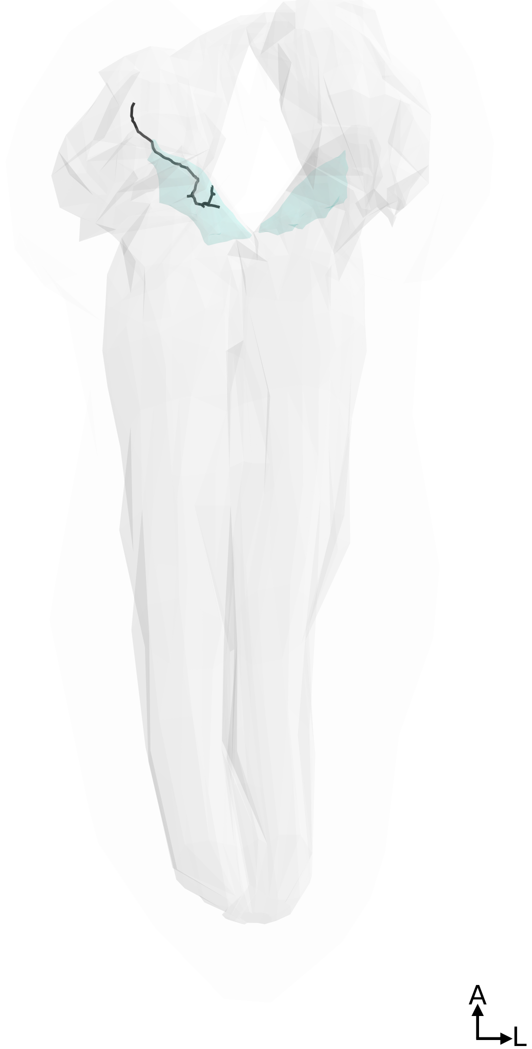

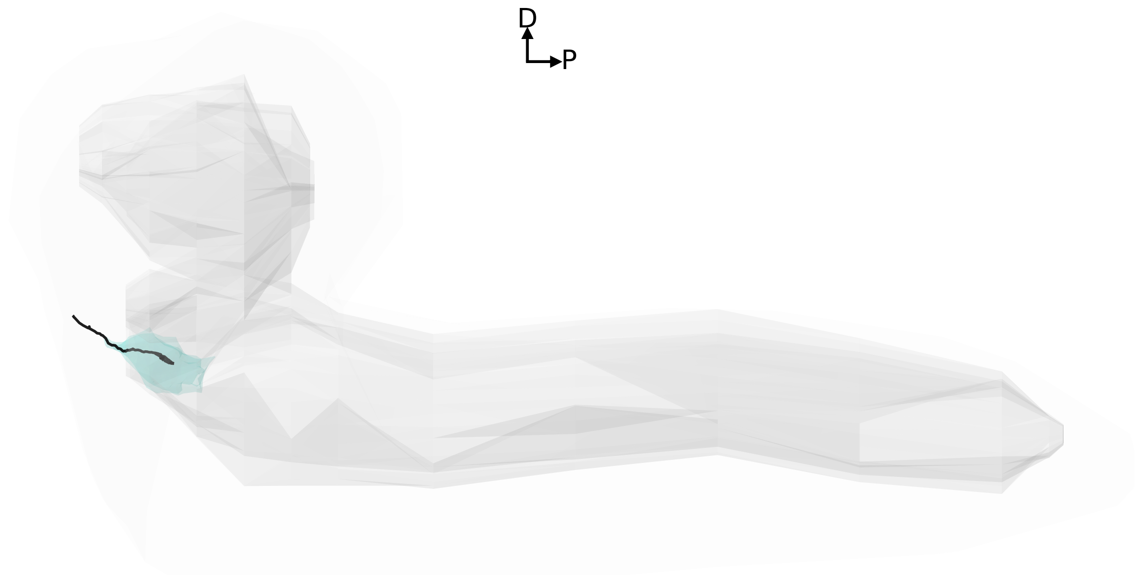

ID	name	SCACa	SCAVa	SCAVp	SCACal	SCACp	SCACpl	SCVM	IPCs	DMS	DH44	Se0ens	Se0ph	PMN LR	MN motor neurons	PaN motor neurons	olfactory PNs	gustatory PNs	multiglomerular PNs	unknown PNs	thermo PNs	visual PNs
15982306	AN-L-Sens-B1-AVa-28	0	1	0	0	0	0	0	0	0	0	3	0	1	0	0	0	0	0	0	0	0

ID: 9820270
name: AN-L-Sens-B1-AVa-29

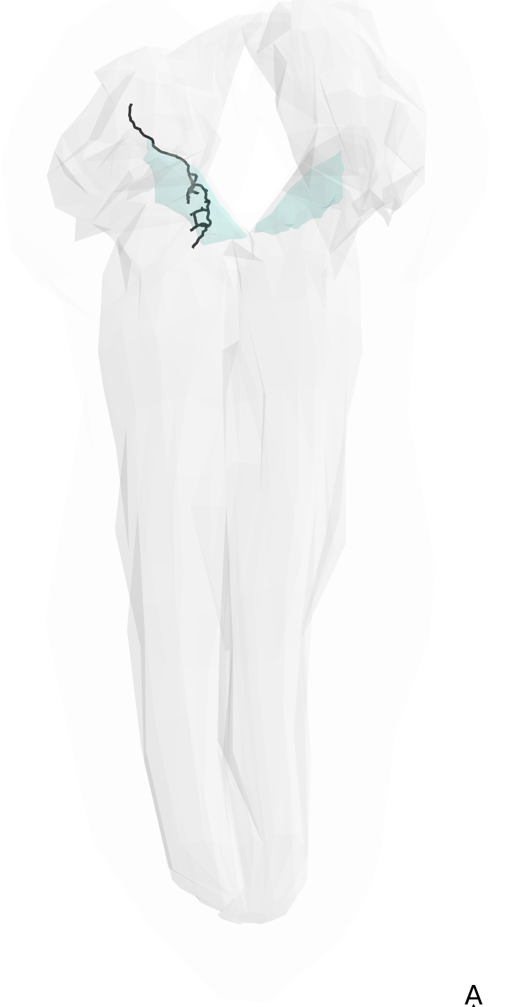

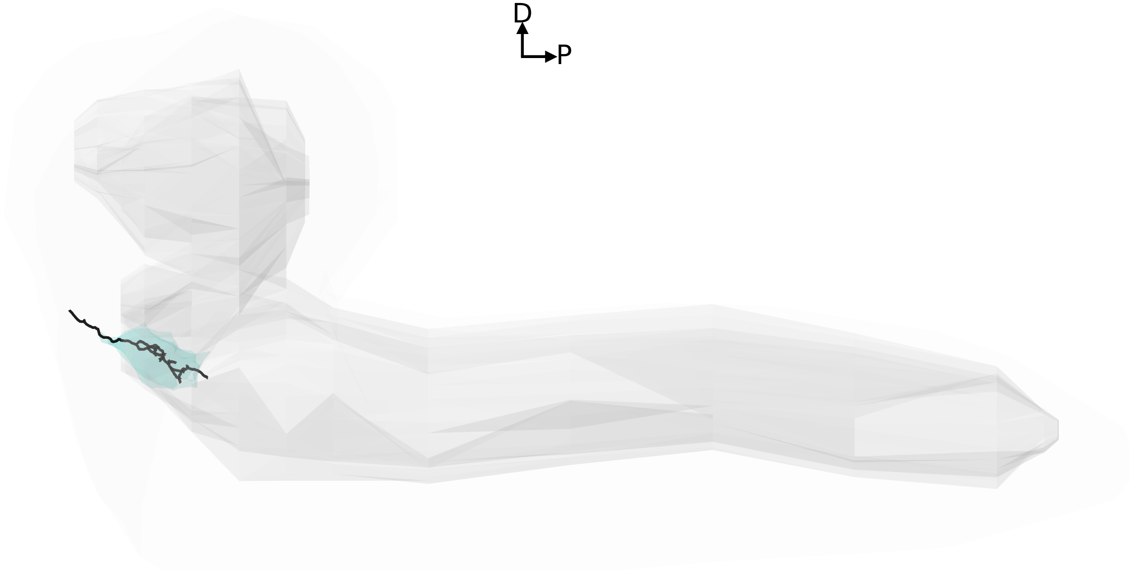

ID	*name*	SCACa	SCAVa	SCAVp	SCACal	SCACp	SCACpl	SCVM	IPCs	DMS	DH44	Se0ens	Se0ph	PMN LR	MN motor neurons	PaN motor neurons	olfactory PNs	gustatory PNs	multiglomerular PNs	unknown PNs	thermo PNs	visual PNs
9820270	AN-L-Sens-B1-AVa-29	0	6	0	0	0	0	0	0	0	0	0	1	1	0	0	0	0	0	0	0	0

ID: 7424178
name: AN-L-Sens-B1-AVa-30

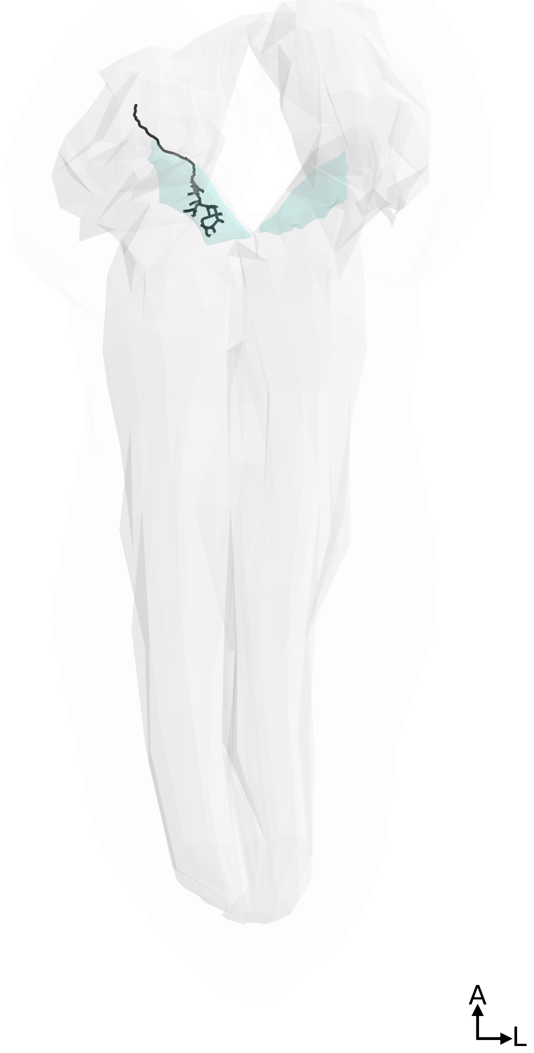

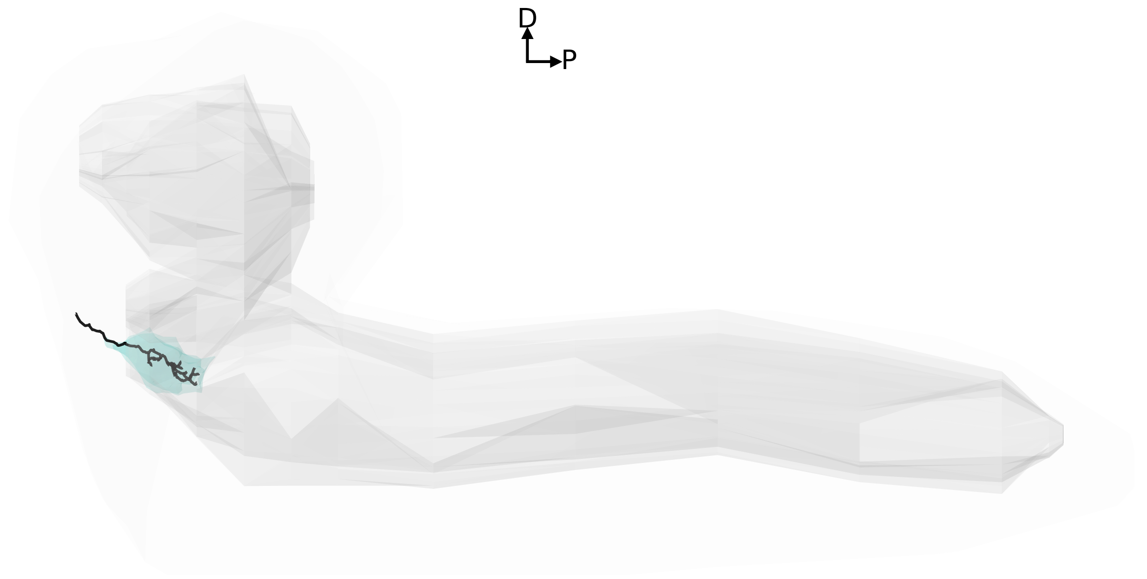

ID	*name*	SCACa	SCAVa	SCAVp	SCACal	SCACp	SCACpl	SCVM	IPCs	DMS	DH44	Se0ens	Se0ph	PMN LR	MN motor neurons	PaN motor neurons	olfactory PNs	gustatory PNs	multiglomerular PNs	unknown PNs	thermo PNs	visual PNs
7424178	AN-L-Sens-B1-AVa-30	0	5	0	0	0	0	0	0	0	0	1	0	2	0	0	0	0	0	0	0	0

ID: 1419315
name: AN-L-Sens-B1-AVa-31

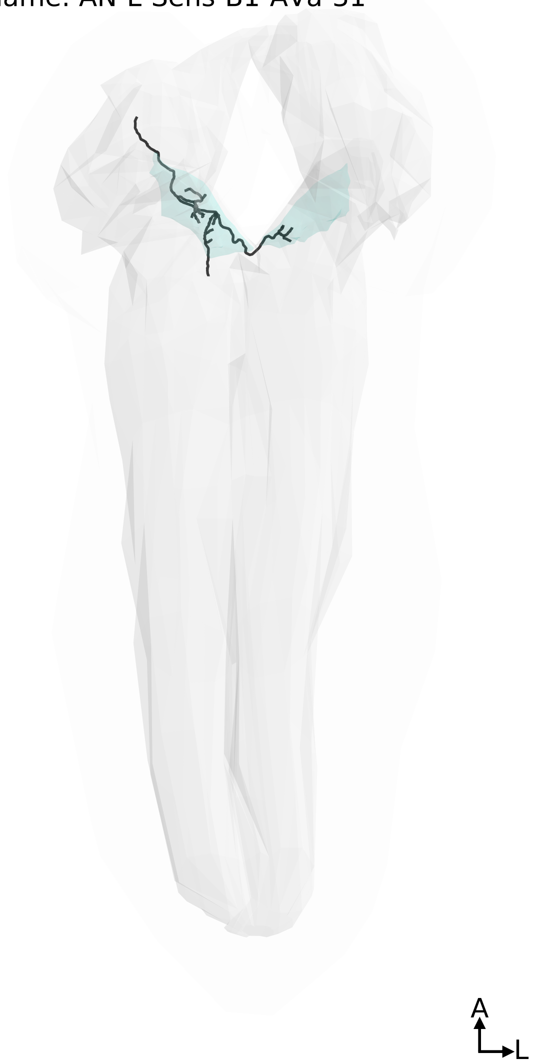

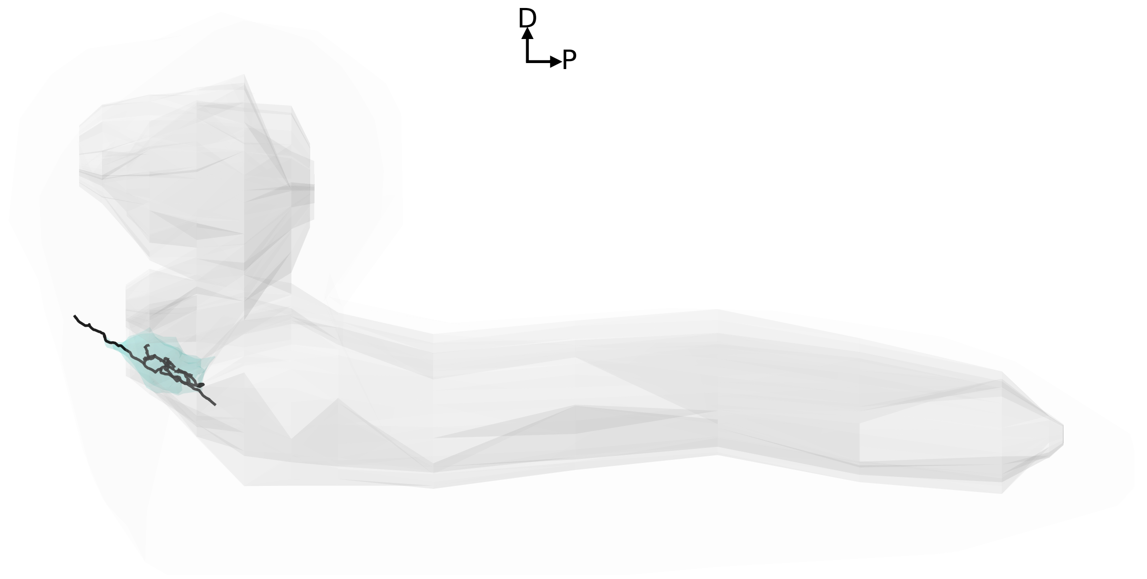

ID	name	SCACa	SCAVa	SCAVp	SCACal	SCACp	SCACpl	SCVM	IPCs	DMS	DH44	Se0ens	Se0ph	PMN LR	MN motor neurons	PaN motor neurons	olfactory PNs	gustatory PNs	multiglomerular PNs	unknown PNs	thermo PNs	visual PNs
1419315	AN-L-Sens-B1-AVa-31	0	10	0	0	0	0	0	0	0	0	0	0	3	0	0	0	0	0	0	0	0

ID: 1427012
name: AN-L-Sens-B1-AVa-32

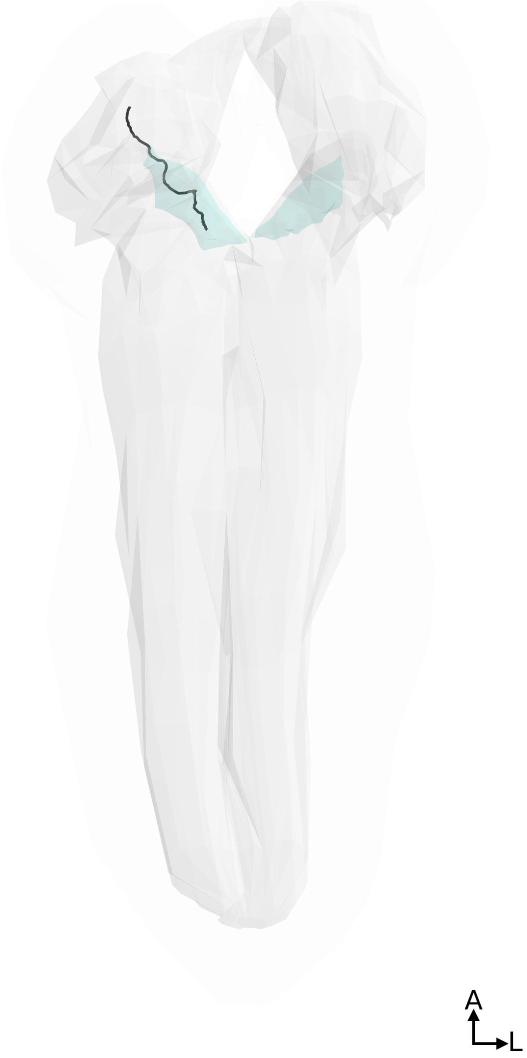

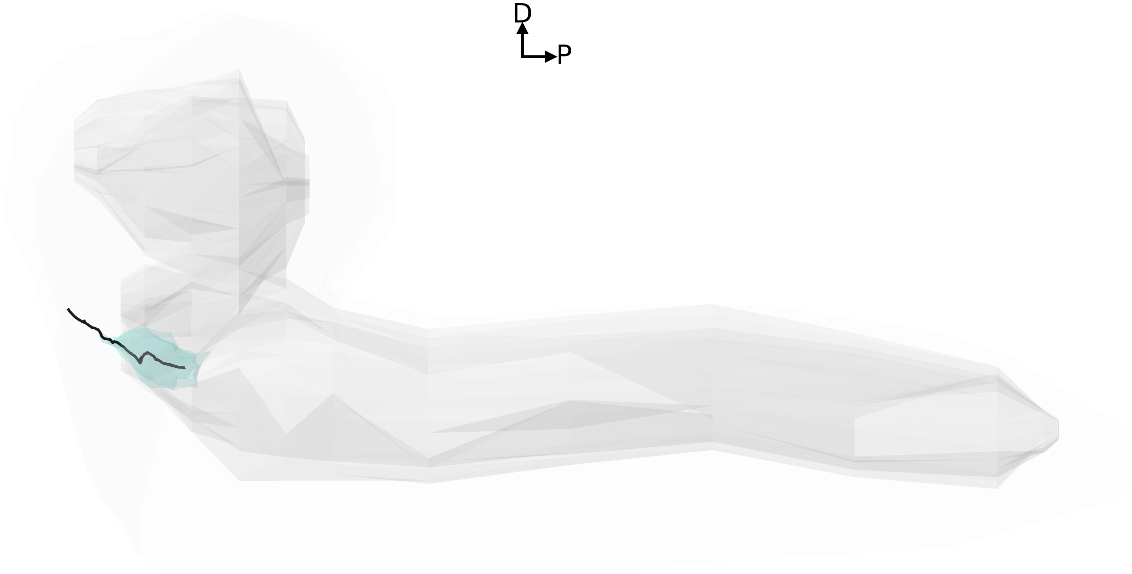

ID	*name*	SCACa	SCAVa	SCAVp	SCACal	SCACp	SCACpl	SCVM	IPCs	DMS	DH44	Se0ens	Se0ph	PMN LR	MN motor neurons	PaN motor neurons	olfactory PNs	gustatory PNs	multiglomerular PNs	unknown PNs	thermo PNs	visual PNs
1427012	AN-L-Sens-B1-AVa-32	0	3	0	0	0	0	0	0	0	0	0	0	1	0	0	0	0	0	0	0	0

ID: 17380936
name: AN-L-Sens-B1-AVa-33

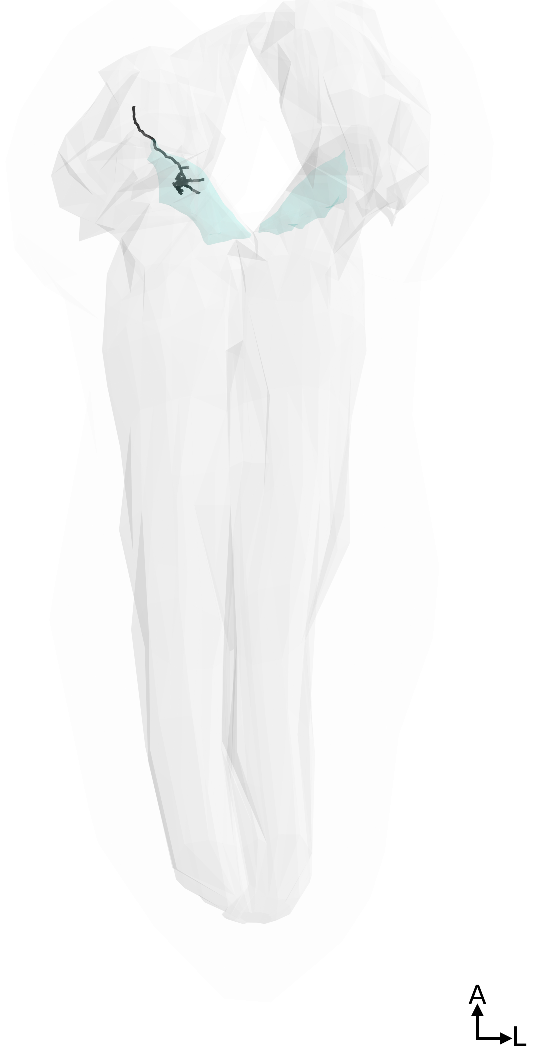

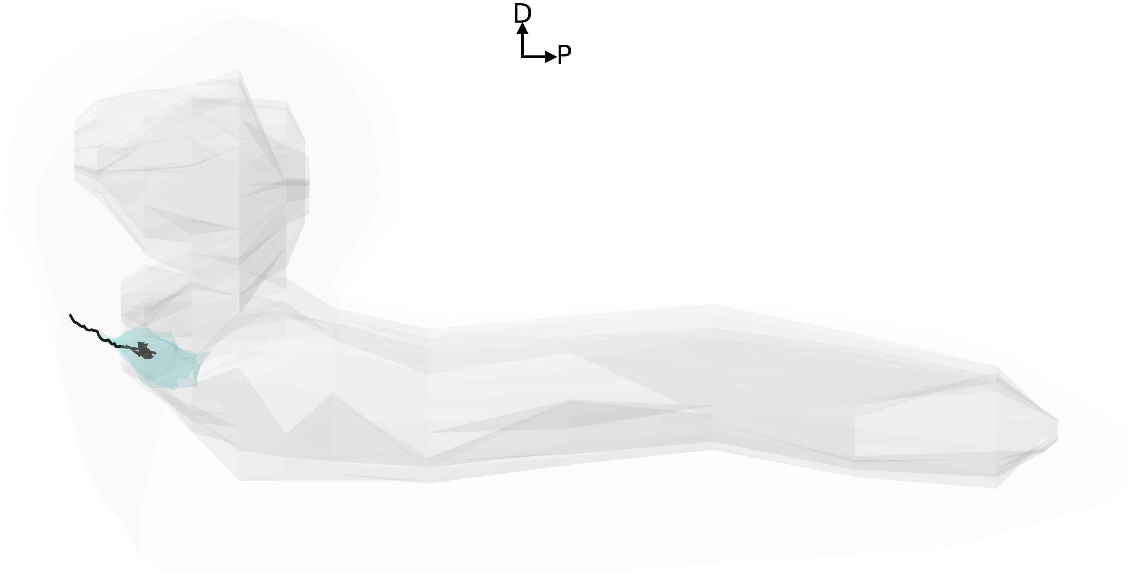

ID	name	SCACa	SCAVa	SCAVp	SCACal	SCACp	SCACpl	SCVM	IPCs	DMS	DH44	Se0ens	Se0ph	PMN LR	MN motor neurons	PaN motor neurons	olfactory PNs	gustatory PNs	multiglomerular PNs	unknown PNs	thermo PNs	visual PNs
17380936	AN-L-Sens-B1-AVa-33	0	6	0	0	0	0	0	0	0	0	5	1	2	0	0	0	0	0	0	0	0

ID: 15983225
name: AN-L-Sens-B1-AVa-34

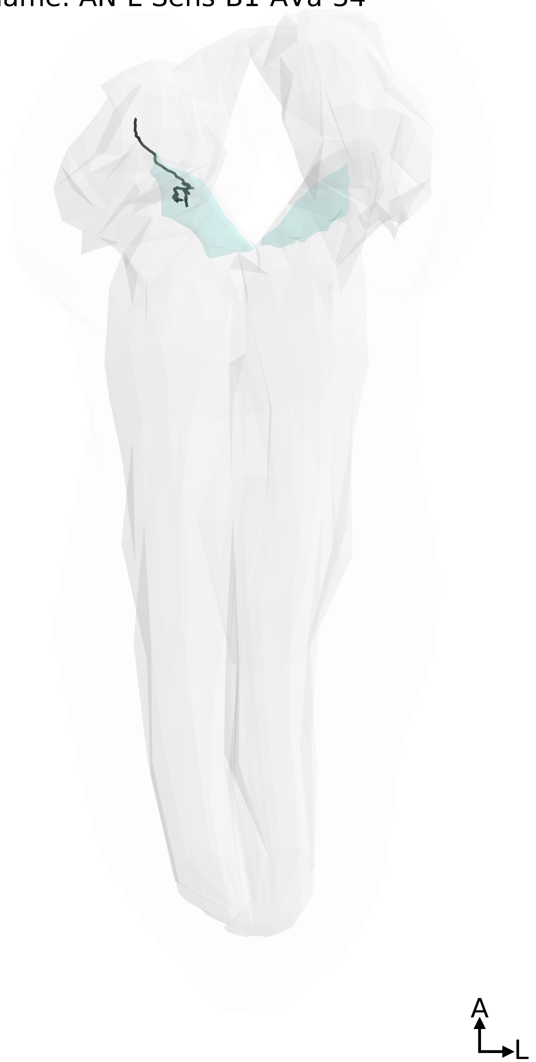

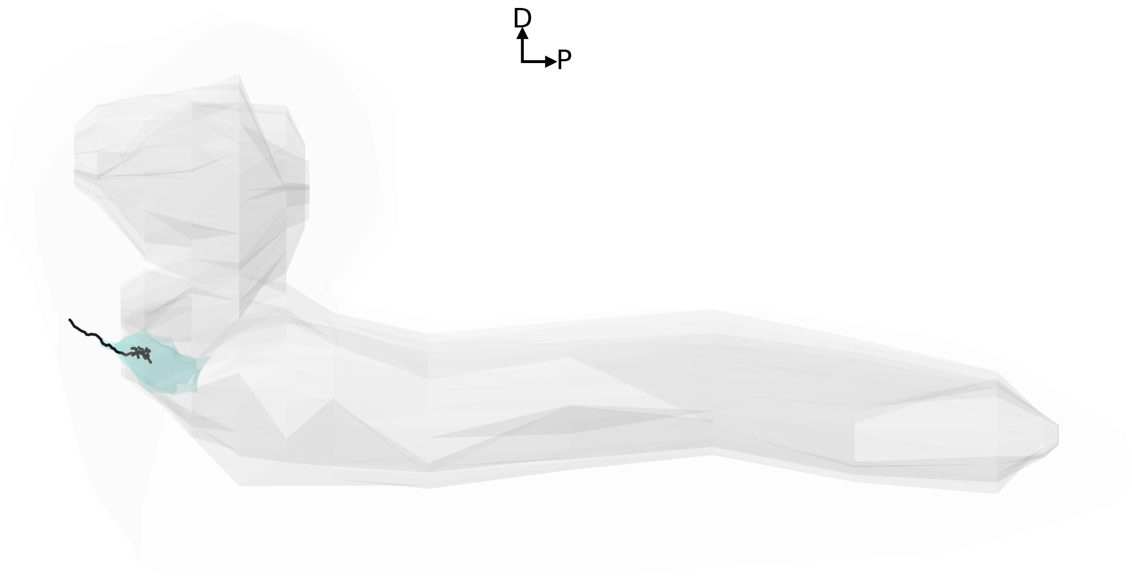

ID	*name*	SCACa	SCAVa	SCAVp	SCACal	SCACp	SCACpl	SCVM	IPCs	DMS	DH44	Se0ens	Se0ph	PMN LR	MN motor neurons	PaN motor neurons	olfactory PNs	gustatory PNs	multiglomerular PNs	unknown PNs	thermo PNs	visual PNs
15983225	AN-L-Sens-B1-AVa-34	0	0	0	0	0	0	0	0	0	0	3	2	5	0	0	0	0	0	0	0	0

ID: 15984231
name: AN-L-Sens-B1-AVa-35

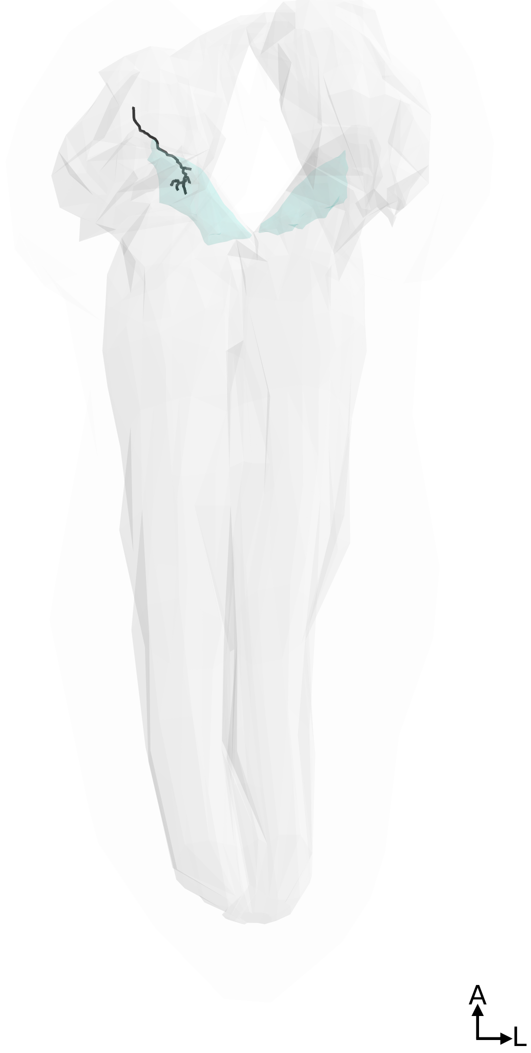

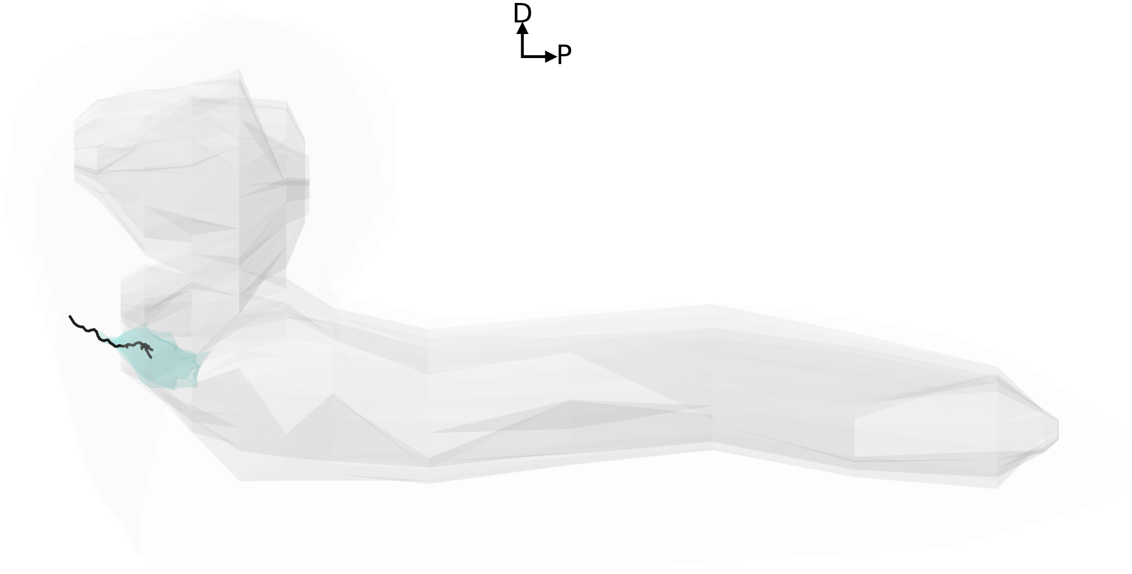

ID	*name*	SCACa	SCAVa	SCAVp	SCACal	SCACp	SCACpl	SCVM	IPCs	DMS	DH44	Se0ens	Se0ph	PMN LR	MN motor neurons	PaN motor neurons	olfactory PNs	gustatory PNs	multiglomerular PNs	unknown PNs	thermo PNs	visual PNs
15984231	AN-L-Sens-B1-AVa-35	0	1	0	0	0	0	0	0	0	0	2	1	0	0	0	0	0	0	0	0	0

ID: 15994926
name: AN-L-Sens-B1-AVa-36

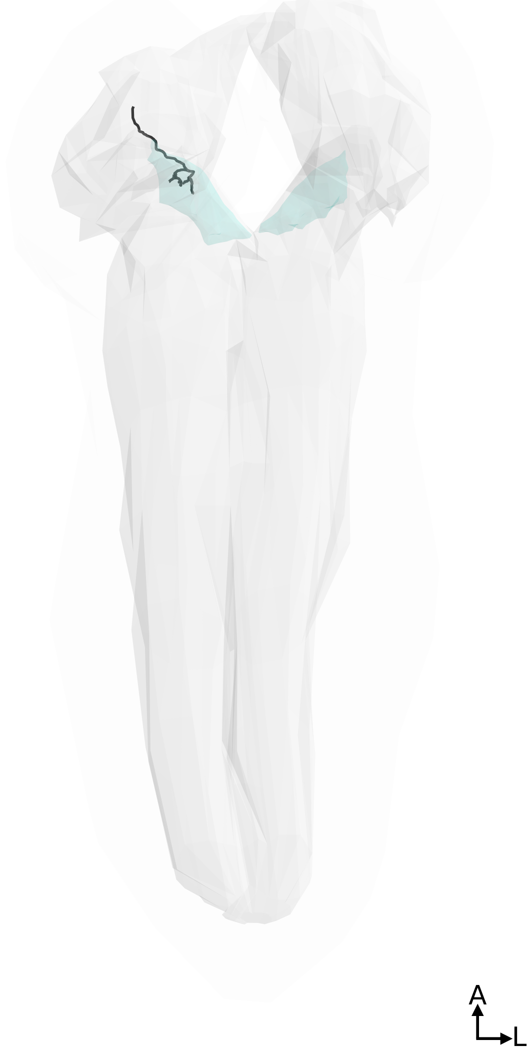

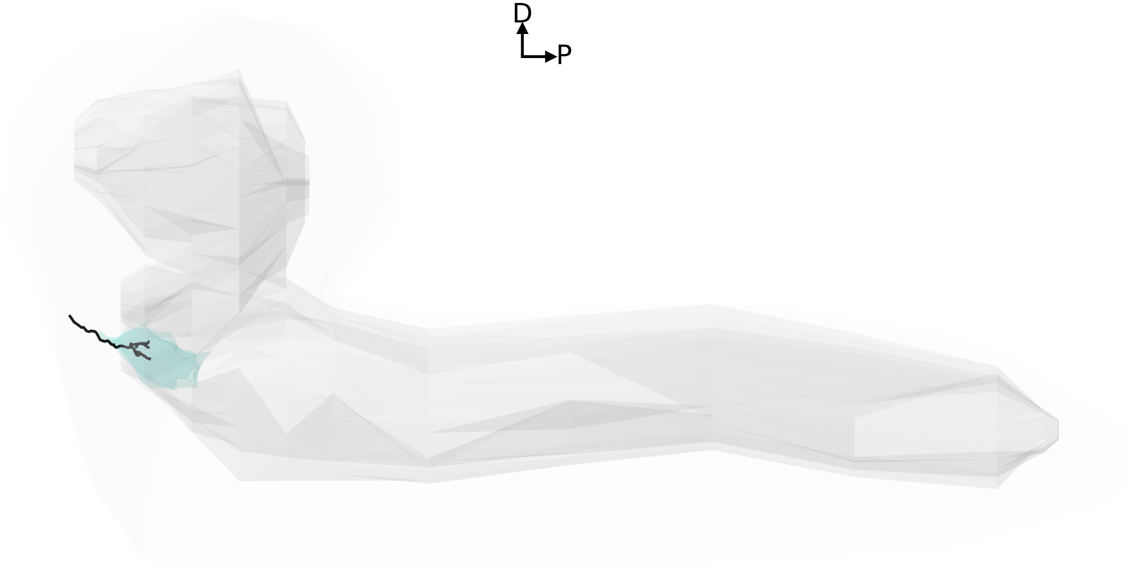

ID	name	SCACa	SCAVa	SCAVp	SCACal	SCACp	SCACpl	SCVM	IPCs	DMS	DH44	Se0ens	Se0ph	PMN LR	MN motor neurons	PaN motor neurons	olfactory PNs	gustatory PNs	multiglomerular PNs	unknown PNs	thermo PNs	visual PNs
15994926	AN-L-Sens-B1-AVa-36	0	1	0	0	0	0	0	0	0	0	2	0	3	0	0	0	0	0	0	0	0

ID: 15995327
name: AN-L-Sens-B1-AVa-37

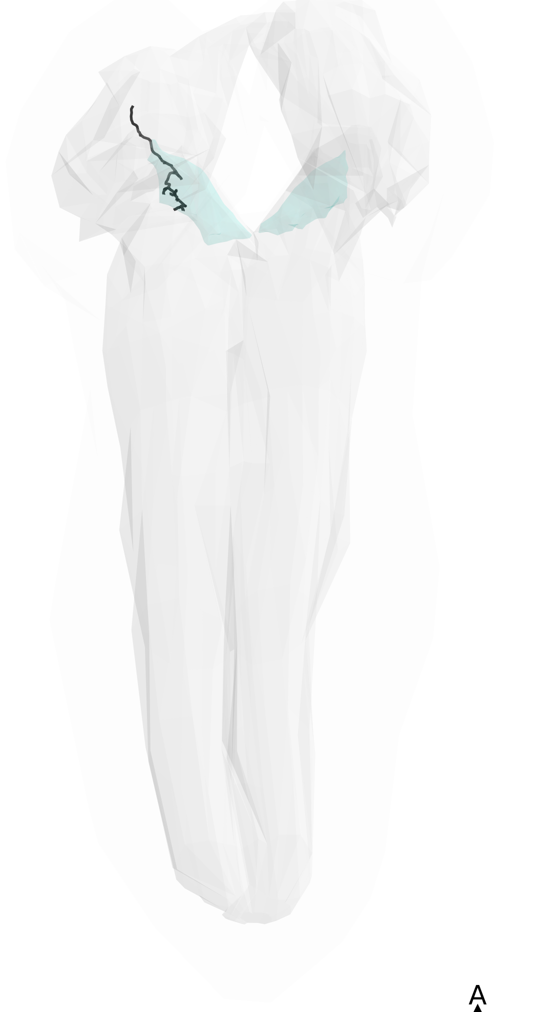

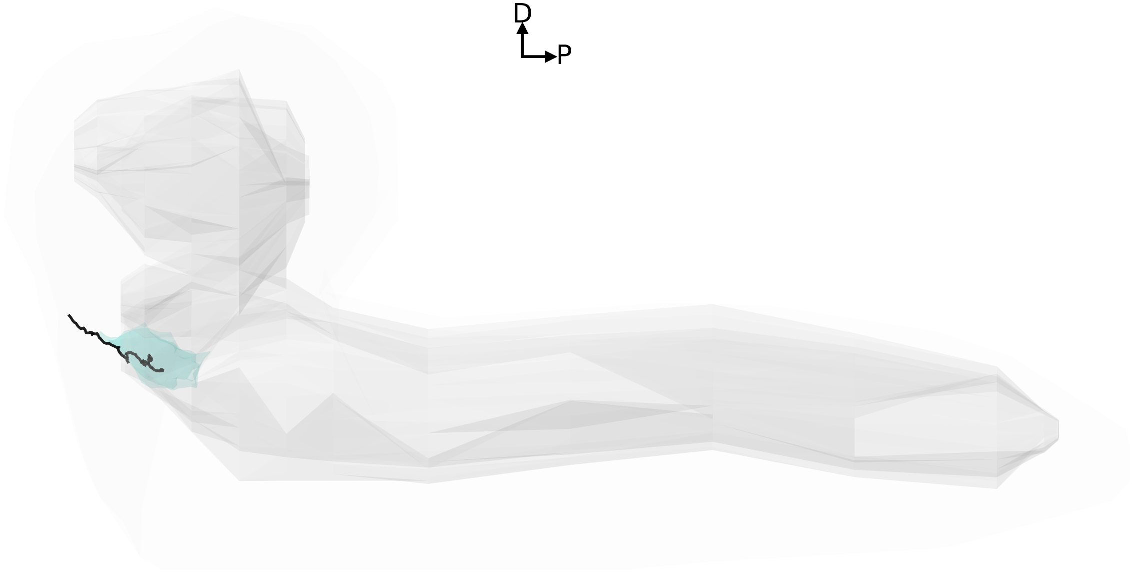

ID	*name*	SCACa	SCAVa	SCAVp	SCACal	SCACp	SCACpl	SCVM	IPCs	DMS	DH44	Se0ens	Se0ph	PMN LR	MN motor neurons	PaN motor neurons	olfactory PNs	gustatory PNs	multiglomerular PNs	unknown PNs	thermo PNs	visual PNs
15995327	AN-L-Sens-B1-AVa-37	0	8	0	0	0	0	0	0	0	0	0	0	37	0	0	0	0	0	0	0	0

ID: 15766999
name: AN-L-Sens-B1-AVa-38

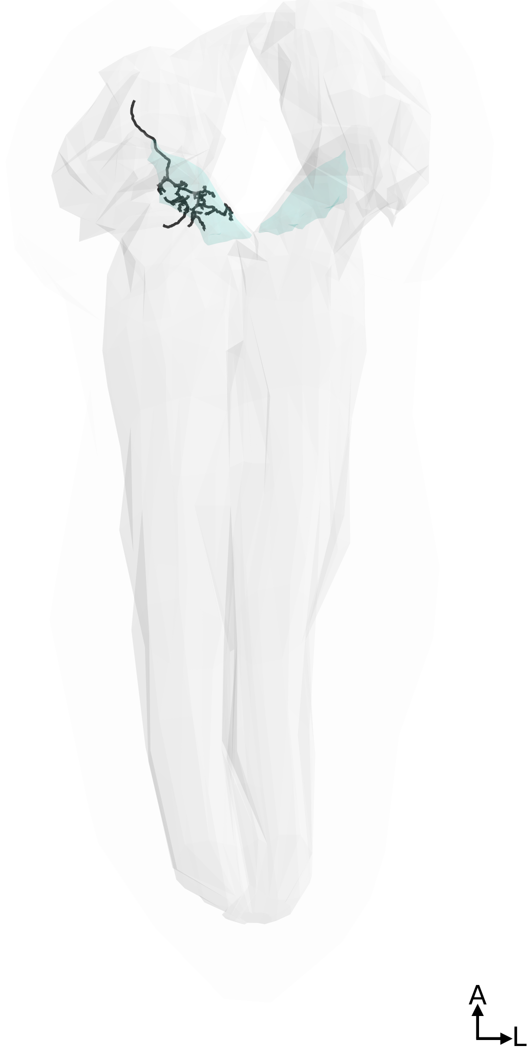

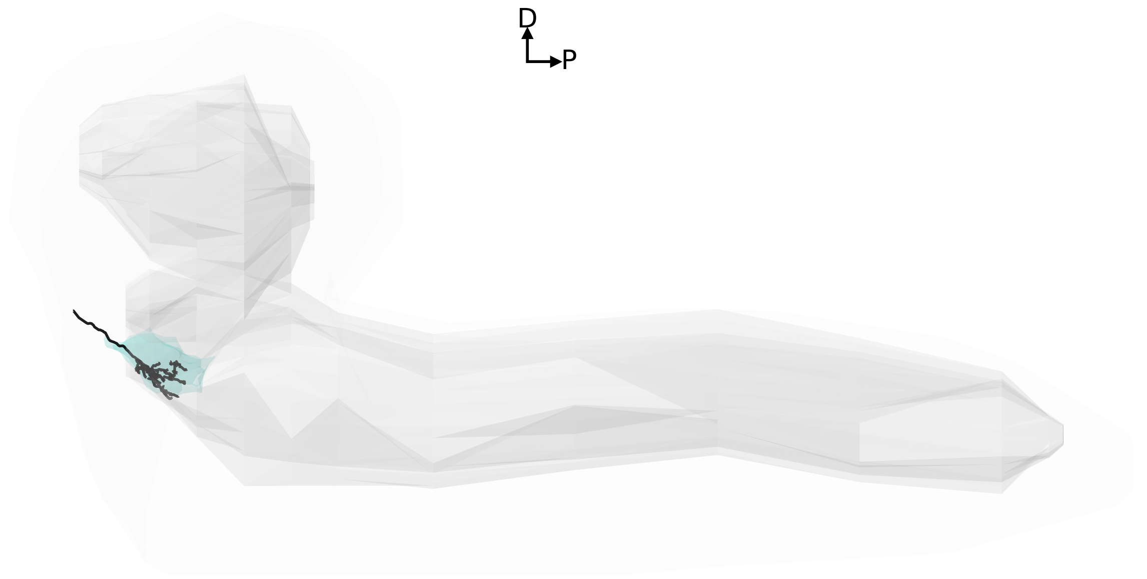

ID	name	SCACa	SCAVa	SCAVp	SCACal	SCACp	SCACpl	SCVM	IPCs	DMS	DH44	Se0ens	Se0ph	PMN LR	MN motor neurons	PaN motor neurons	olfactory PNs	gustatory PNs	multiglomerular PNs	unknown PNs	thermo PNs	visual PNs
15766999	AN-L-Sens-B1-AVa-38	0	1	0	0	0	0	0	0	0	0	0	0	18	0	0	0	0	0	0	0	0

ID: 15983156
name: AN-L-Sens-B1-AVa-39

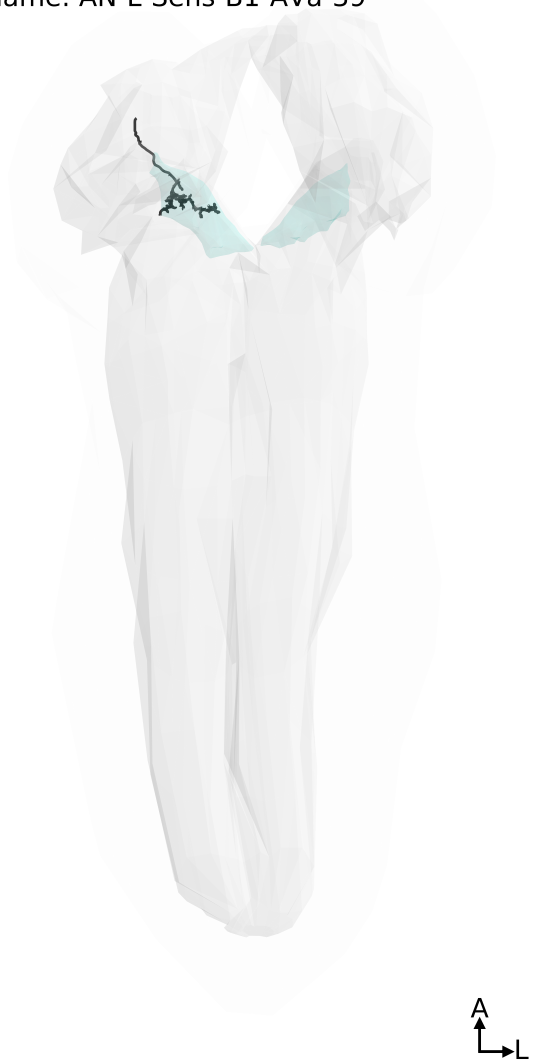

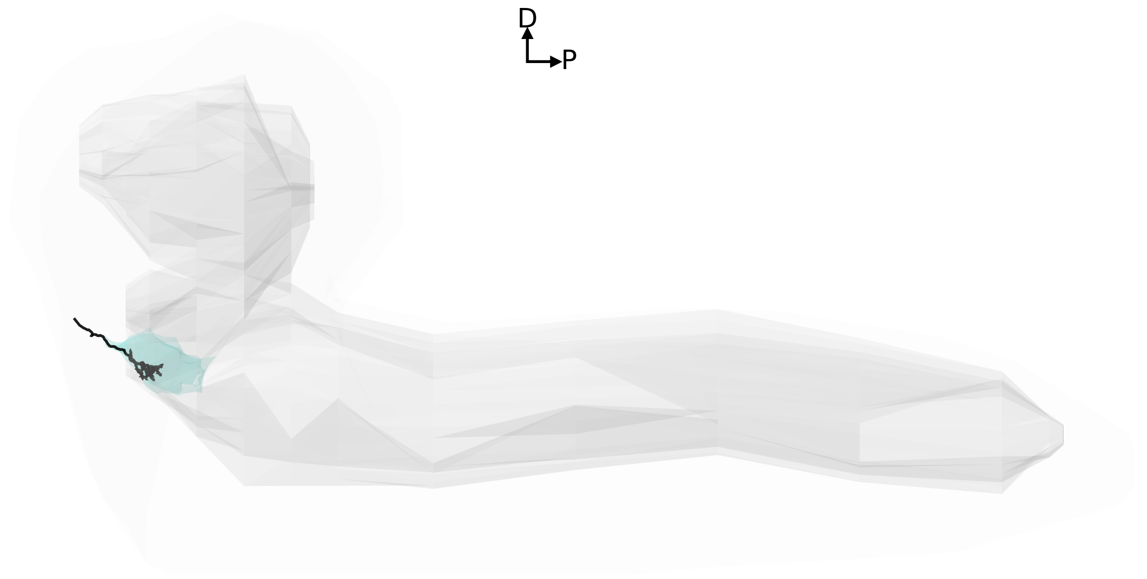

ID	name	SCACa	SCAVa	SCAVp	SCACal	SCACp	SCACpl	SCVM	IPCs	DMS	DH44	Se0ens	Se0ph	PMN LR	MN motor neurons	PaN motor neurons	olfactory PNs	gustatory PNs	multiglomerular PNs	unknown PNs	thermo PNs	visual PNs
15983156	AN-L-Sens-B1-AVa-39	0	8	0	0	0	0	0	0	0	0	0	0	22	0	0	0	0	0	0	0	0

ID: 1351125
name: AN-L-Sens-B1-AVa-40

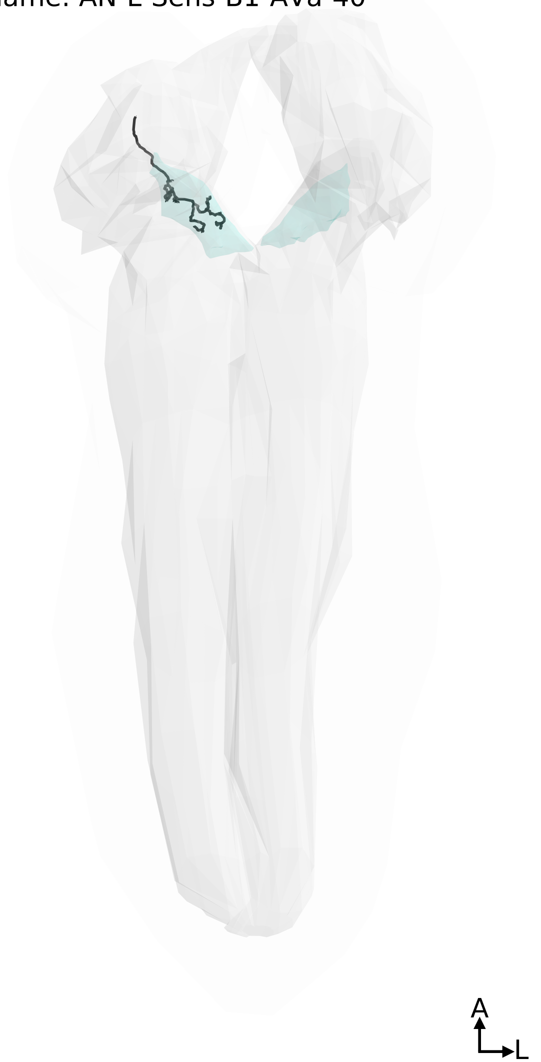

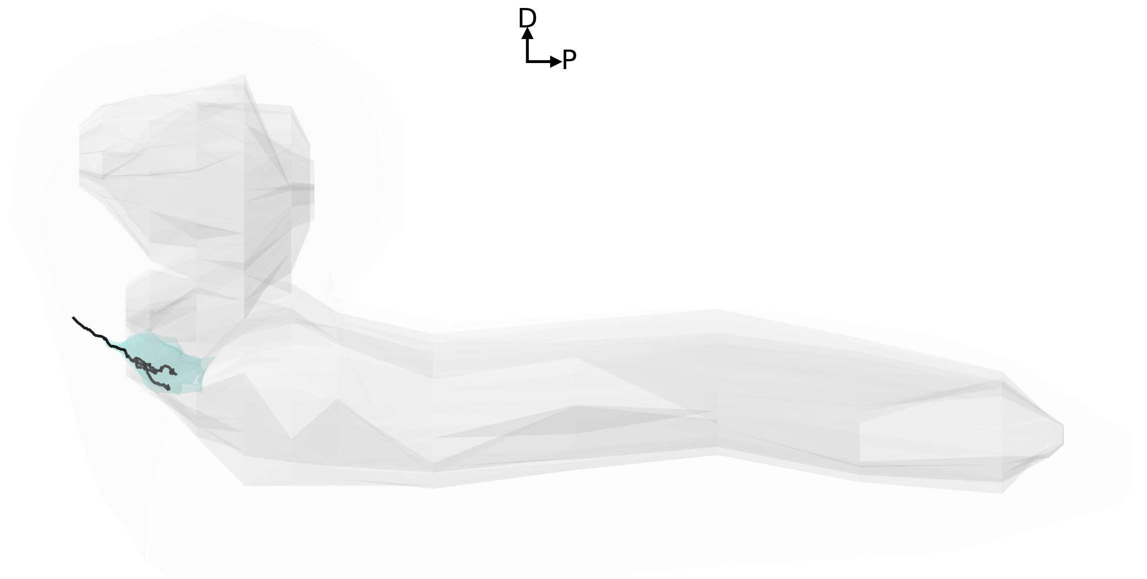

ID	name	SCACa	SCAVa	SCAVp	SCACal	SCACp	SCACpl	SCVM	IPCs	DMS	DH44	Se0ens	Se0ph	PMN LR	MN motor neurons	PaN motor neurons	olfactory PNs	gustatory PNs	multiglomerular PNs	unknown PNs	thermo PNs	visual PNs
1351125	AN-L-Sens-B1-AVa-40	0	13	0	0	0	0	0	0	0	0	0	0	15	0	0	0	0	0	0	0	0

ID: 1709802
name: AN-L-Sens-B2-AVa-24

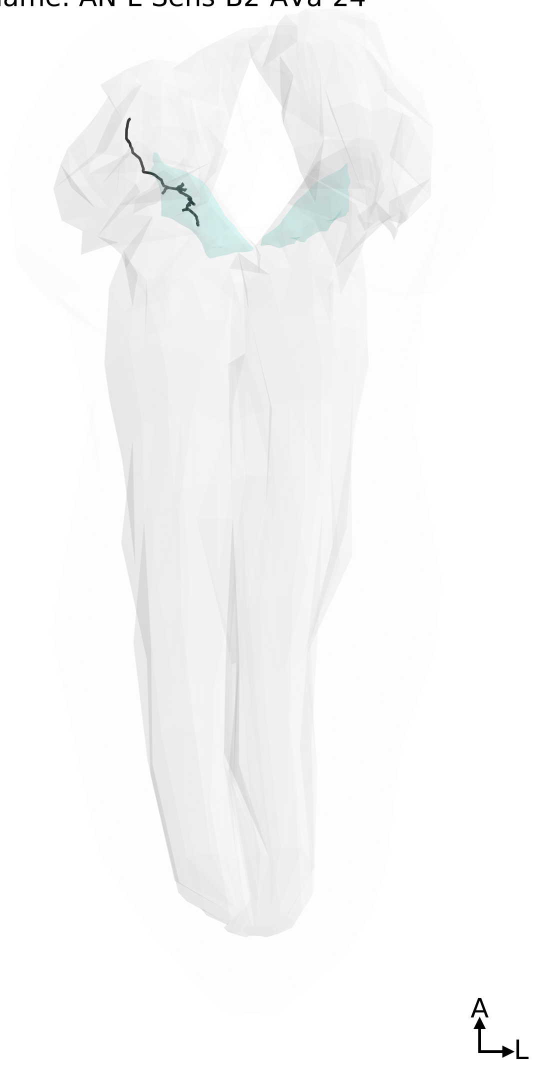

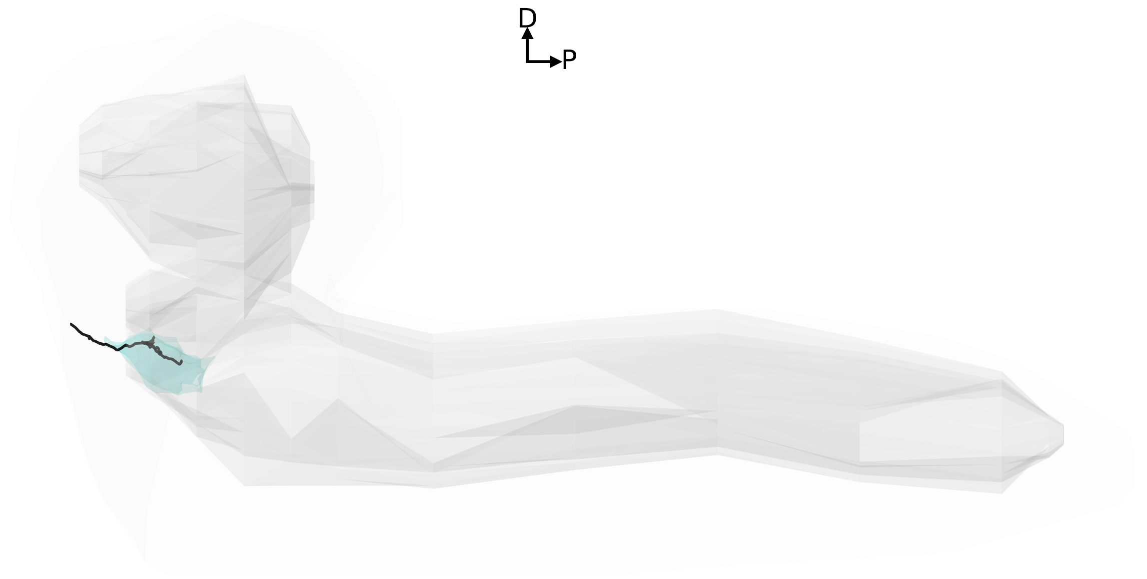

ID	name	SCACa	SCAVa	SCAVp	SCACal	SCACp	SCACpl	SCVM	IPCs	DMS	DH44	Se0ens	Se0ph	PMN LR	MN motor neurons	PaN motor neurons	olfactory PNs	gustatory PNs	multiglomerular PNs	unknown PNs	thermo PNs	visual PNs
1709802	AN-L-Sens-B2-AVa-24	0	3	0	0	0	0	0	0	0	0	0	0	0	0	0	0	0	0	0	0	0

ID: 15998753
name: AN-L-Sens-B2-AVa-25

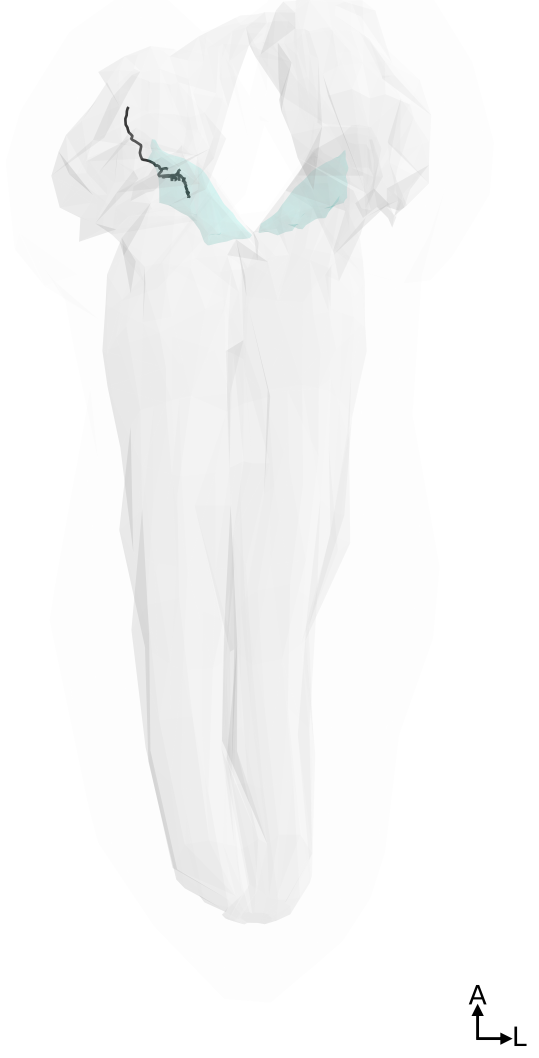

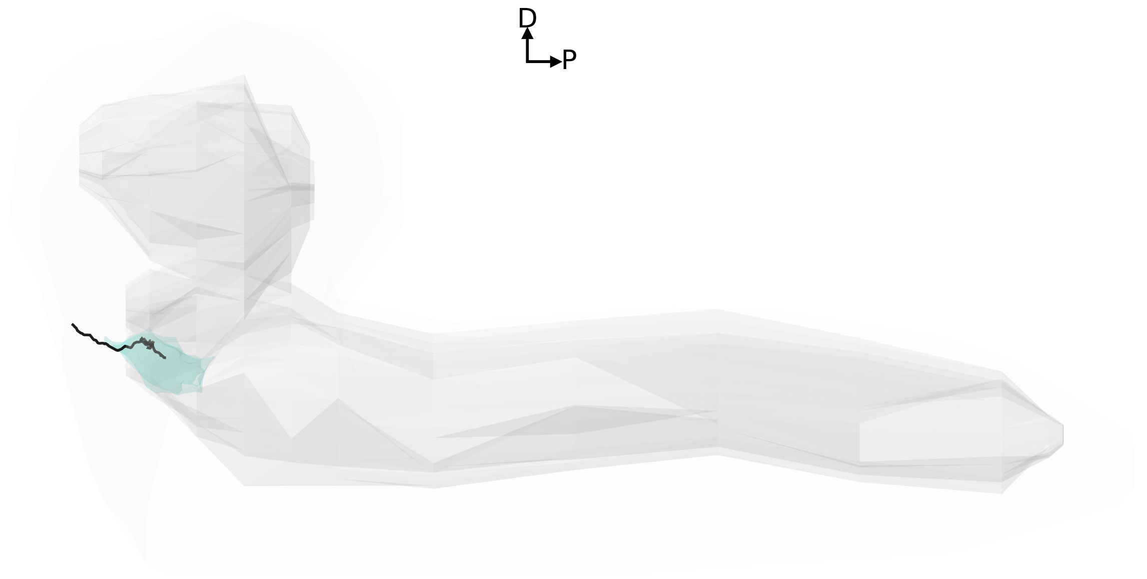

ID	name	SCACa	SCAVa	SCAVp	SCACal	SCACp	SCACpl	SCVM	IPCs	DMS	DH44	Se0ens	Se0ph	PMN LR	MN motor neurons	PaN motor neurons	olfactory PNs	gustatory PNs	multiglomerular PNs	unknown PNs	thermo PNs	visual PNs
15998753	AN-L-Sens-B2-AVa-25	0	2	0	0	0	0	0	0	0	0	0	0	0	0	0	0	0	0	0	0	0

ID: 15574328
name: AN-R-Sens-B1-AVa-15

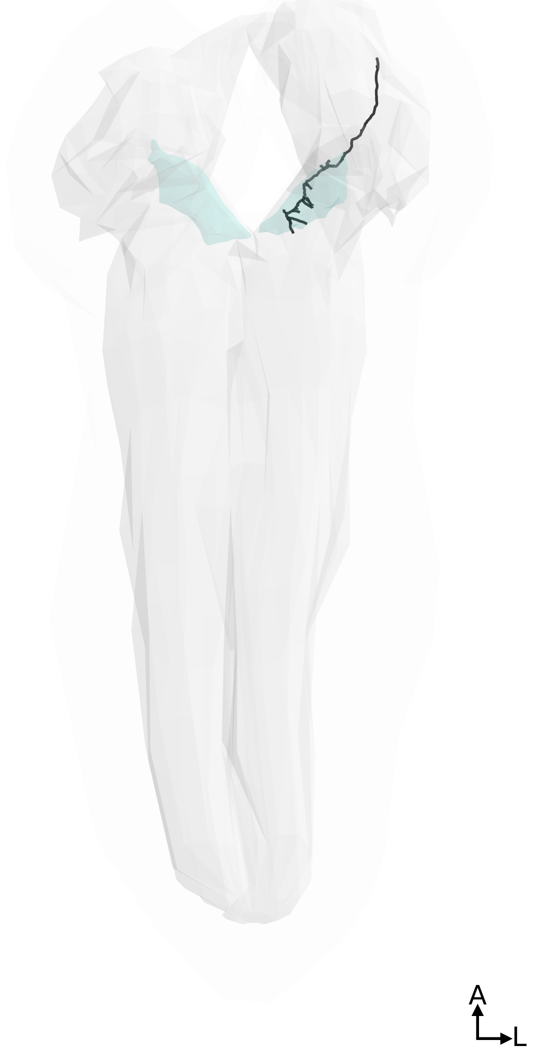

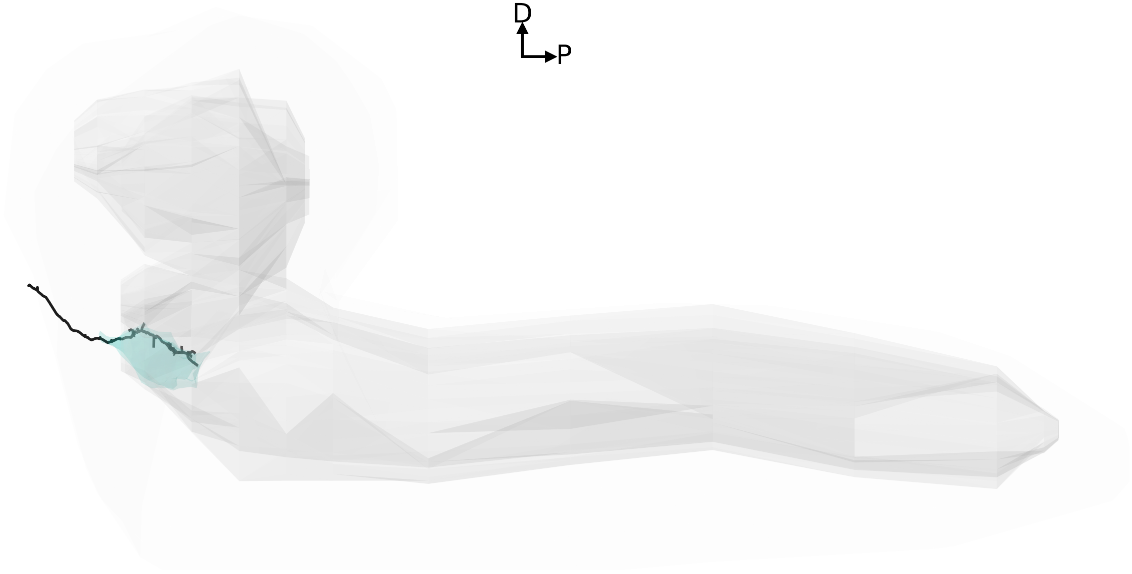

ID	*name*	SCACa	SCAVa	SCAVp	SCACal	SCACp	SCACpl	SCVM	IPCs	DMS	DH44	Se0ens	Se0ph	PMN LR	MN motor neurons	PaN motor neurons	olfactory PNs	gustatory PNs	multiglomerular PNs	unknown PNs	thermo PNs	visual PNs
15574328	AN-R-Sens-B1-AVa-15	4	0	0	0	0	0	0	1	1	1	27	0	0	0	0	0	0	0	0	0	0

ID: 6883831
name: AN-R-Sens-B1-AVa-16

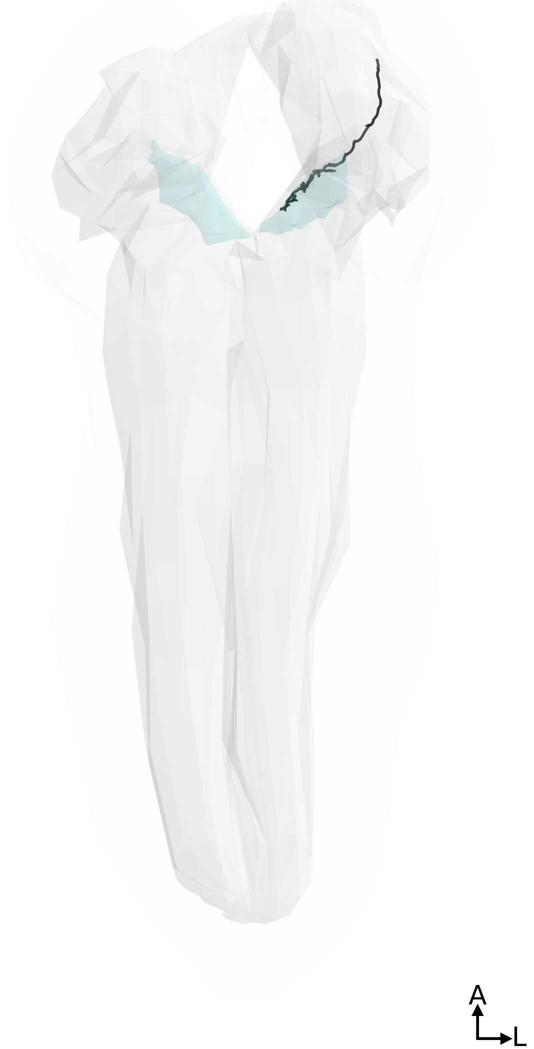

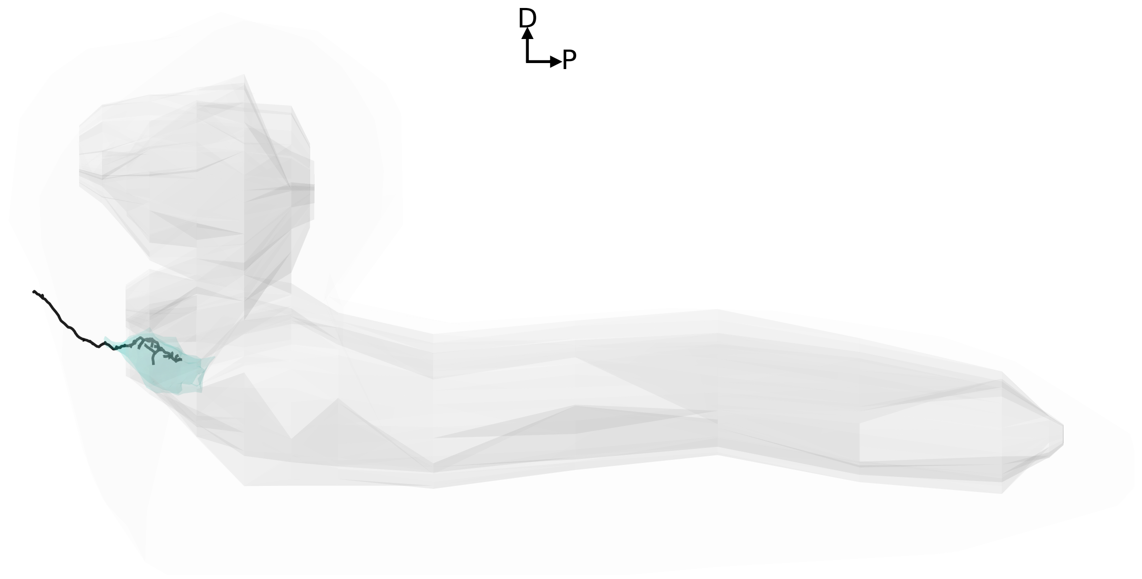

ID	*name*	SCACa	SCAVa	SCAVp	SCACal	SCACp	SCACpl	SCVM	IPCs	DMS	DH44	Se0ens	Se0ph	PMN LR	MN motor neurons	PaN motor neurons	olfactory PNs	gustatory PNs	multiglomerular PNs	unknown PNs	thermo PNs	visual PNs
6883831	AN-R-Sens-B1-AVa-16	1	4	0	0	0	0	0	1	0	0	30	0	0	0	0	0	0	0	0	0	0

ID: 9748269
name: AN-R-Sens-B1-AVa-18

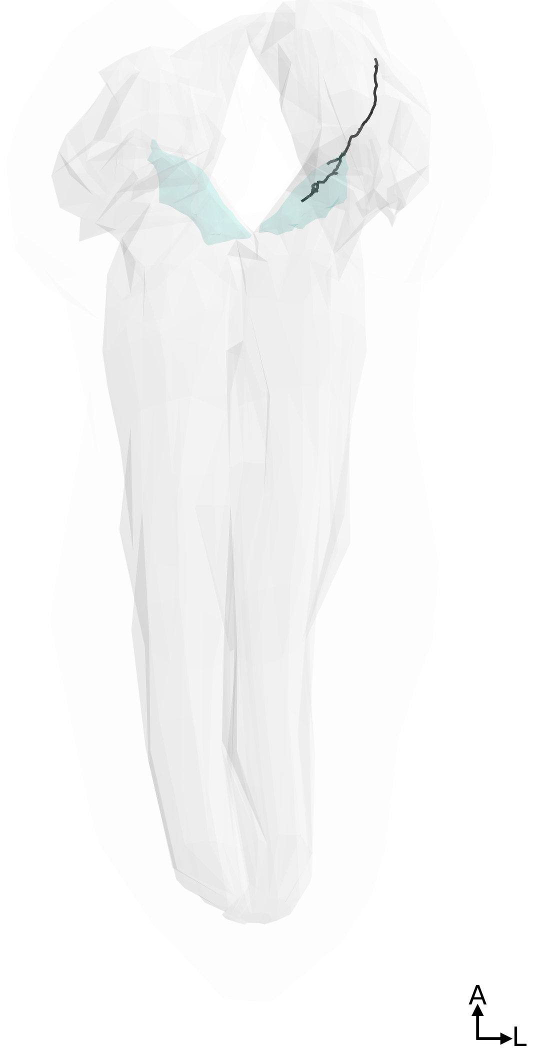

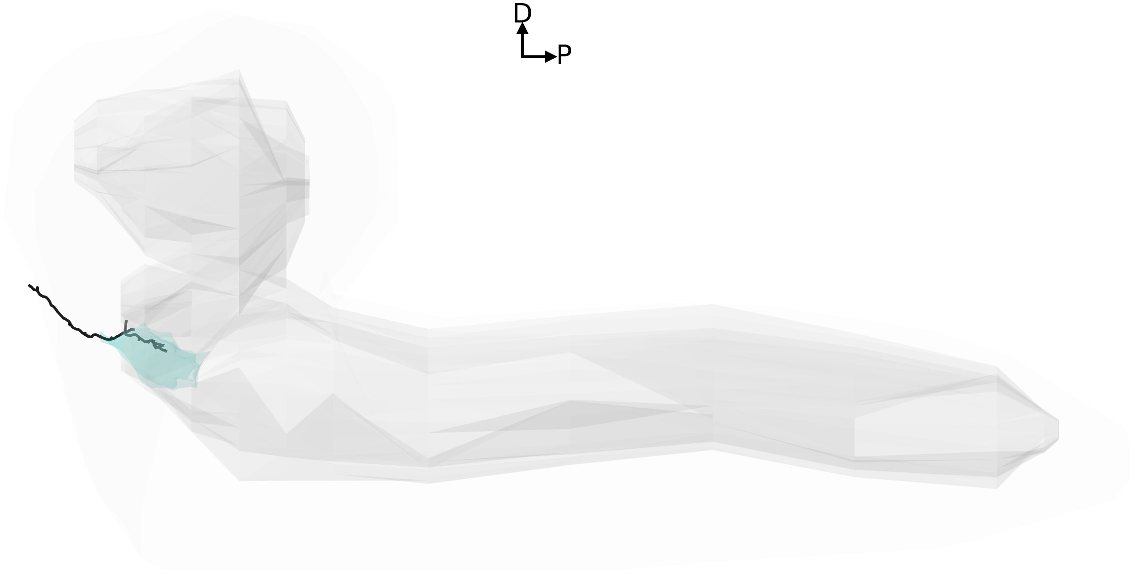

ID	name	SCACa	SCAVa	SCAVp	SCACal	SCACp	SCACpl	SCVM	IPCs	DMS	DH44	Se0ens	Se0ph	PMN LR	MN motor neurons	PaN motor neurons	olfactory PNs	gustatory PNs	multiglomerular PNs	unknown PNs	thermo PNs	visual PNs
9748269	AN-R-Sens-B1-AVa-18	0	2	0	0	0	0	0	0	0	0	2	0	0	0	0	0	0	0	0	0	0

ID: 9716858
name: AN-R-Sens-B1-AVa-19

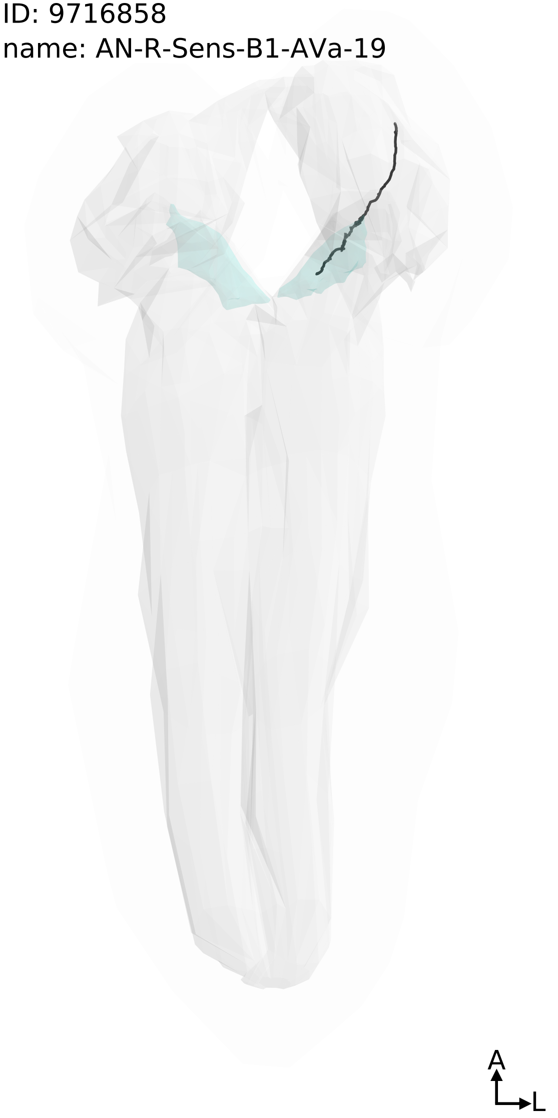

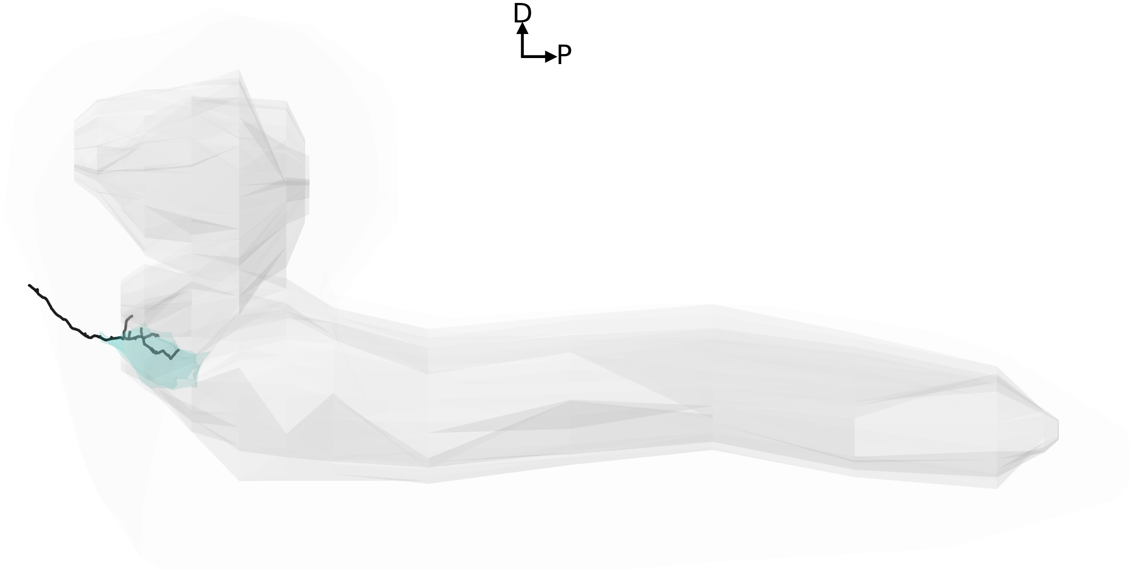

ID	*name*	SCACa	SCAVa	SCAVp	SCACal	SCACp	SCACpl	SCVM	IPCs	DMS	DH44	Se0ens	Se0ph	PMN LR	MN motor neurons	PaN motor neurons	olfactory PNs	gustatory PNs	multiglomerular PNs	unknown PNs	thermo PNs	visual PNs
9716858	AN-R-Sens-B1-AVa-19	0	1	0	0	0	0	0	6	0	0	2	0	0	0	0	0	0	0	0	0	0

ID: 16442344
name: AN-R-Sens-B1-AVa-20

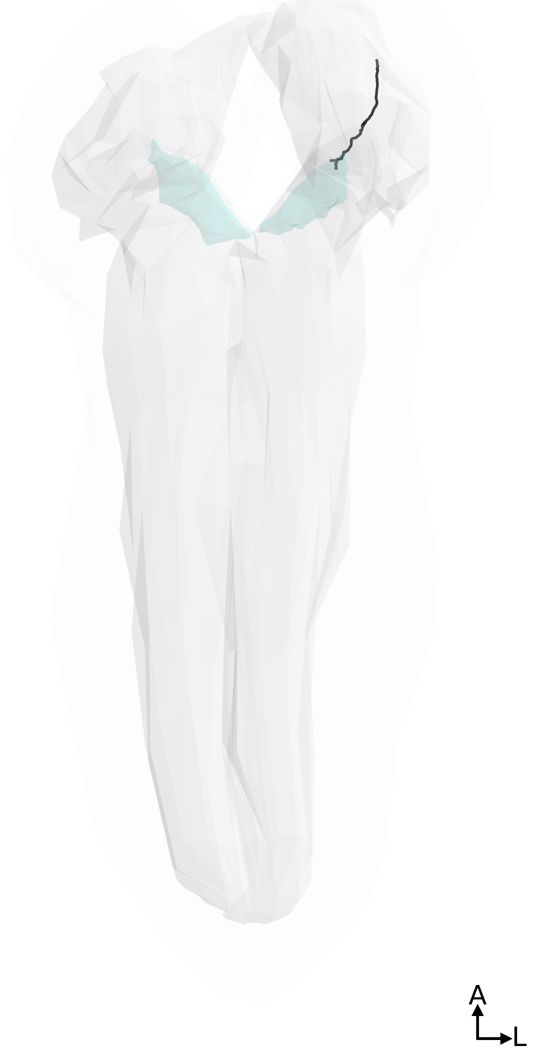

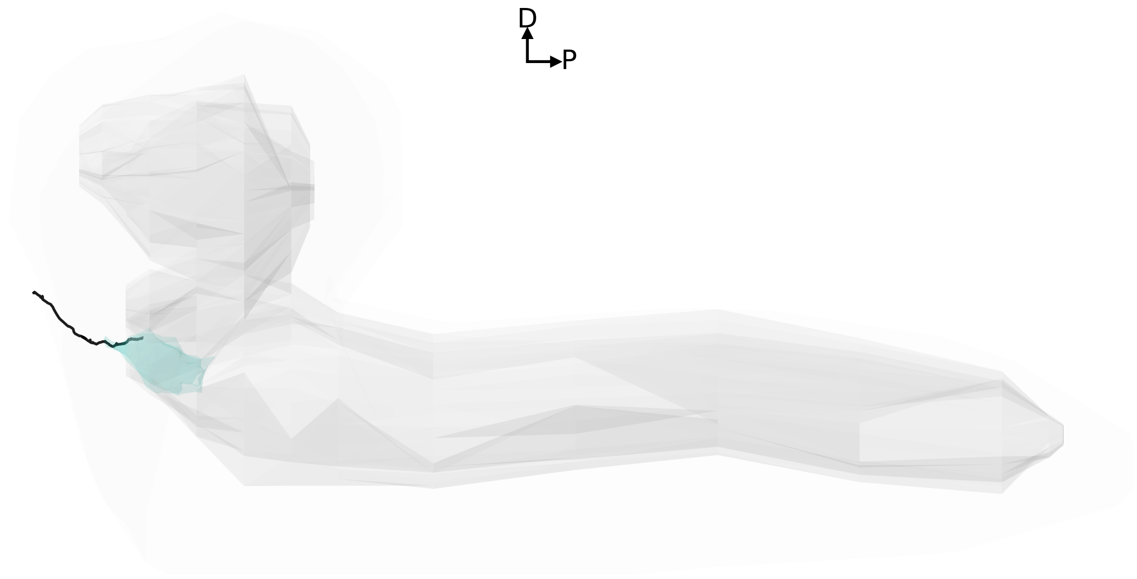

ID	*name*	SCACa	SCAVa	SCAVp	SCACal	SCACp	SCACpl	SCVM	IPCs	DMS	DH44	Se0ens	Se0ph	PMN LR	MN motor neurons	PaN motor neurons	olfactory PNs	gustatory PNs	multiglomerular PNs	unknown PNs	thermo PNs	visual PNs
16442344	AN-R-Sens-B1-AVa-20	0	0	0	0	0	0	0	1	0	0	1	0	0	0	0	0	0	0	0	0	0

ID: 15543068
name: AN-R-Sens-B1-AVa-21

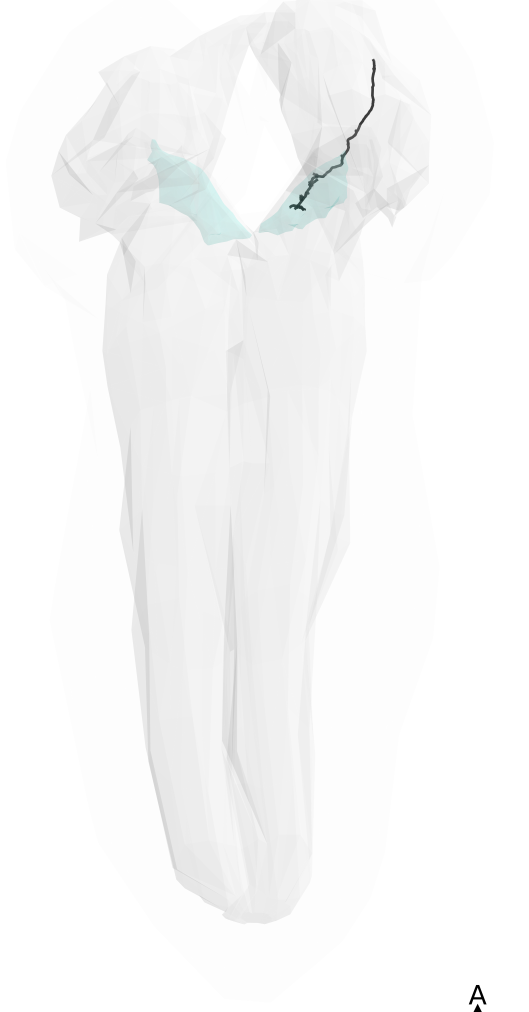

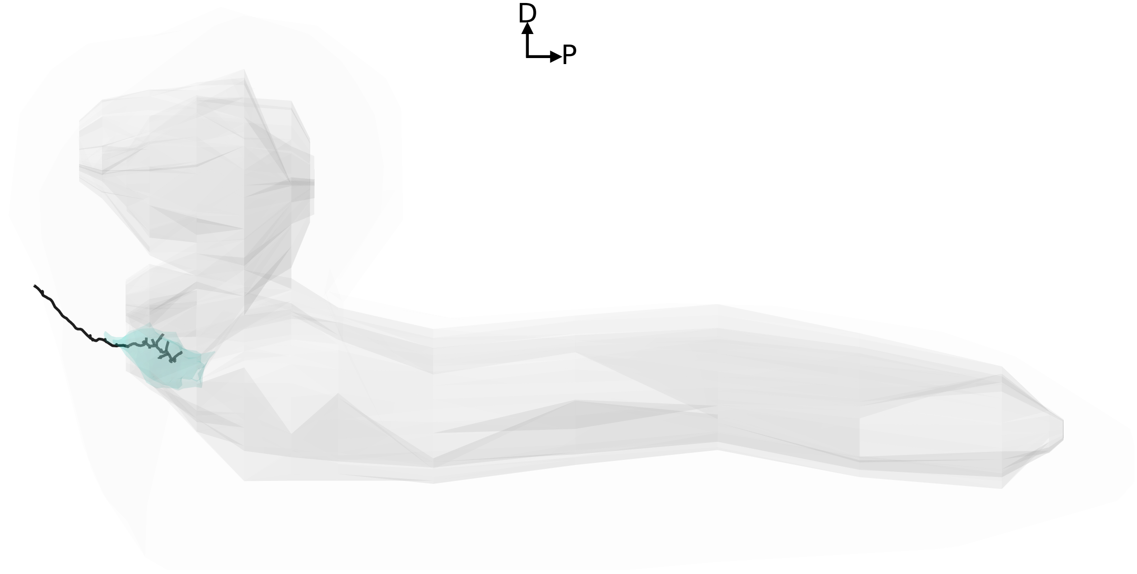

ID	*name*	SCACa	SCAVa	SCAVp	SCACal	SCACp	SCACpl	SCVM	IPCs	DMS	DH44	Se0ens	Se0ph	PMN LR	MN motor neurons	PaN motor neurons	olfactory PNs	gustatory PNs	multiglomerular PNs	unknown PNs	thermo PNs	visual PNs
15543068	AN-R-Sens-B1-AVa-21	0	1	0	0	0	0	0	0	0	1	6	1	0	0	0	0	0	0	0	0	0

ID: 15564807
name: AN-R-Sens-B1-AVa-22

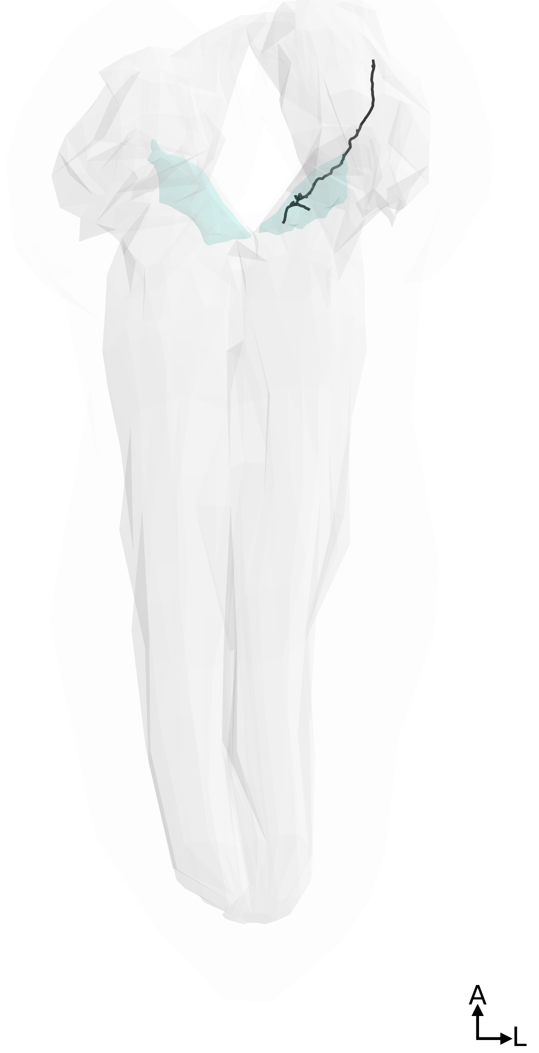

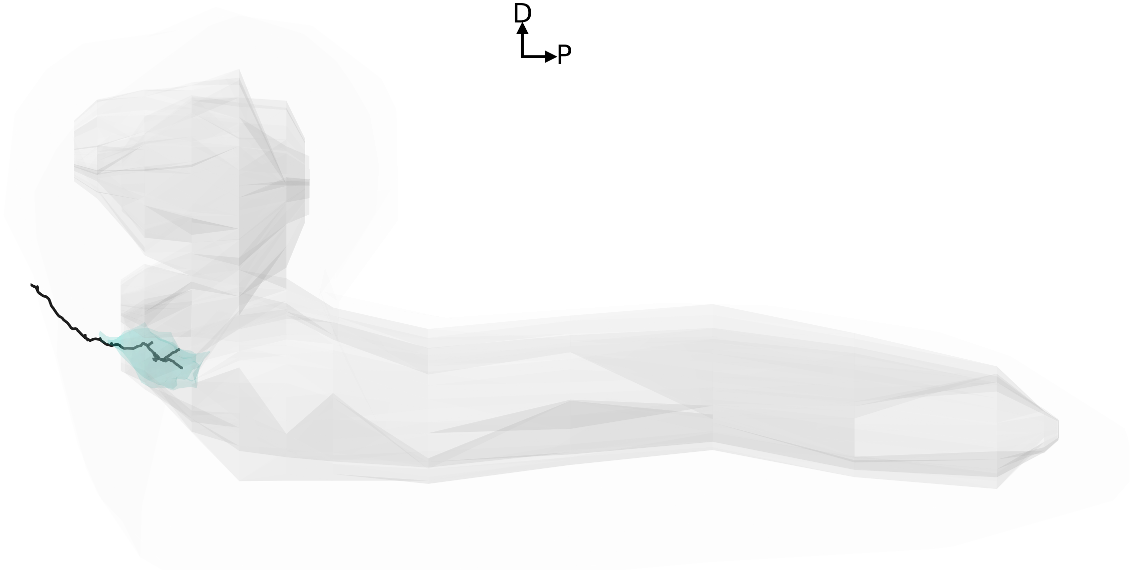

ID	name	SCACa	SCAVa	SCAVp	SCACal	SCACp	SCACpl	SCVM	IPCs	DMS	DH44	Se0ens	Se0ph	PMN LR	MN motor neurons	PaN motor neurons	olfactory PNs	gustatory PNs	multiglomerular PNs	unknown PNs	thermo PNs	visual PNs
15564807	AN-R-Sens-B1-AVa-22	0	4	0	0	0	0	0	0	0	0	3	4	0	0	0	0	0	0	0	0	0

ID: 15564782
name: AN-R-Sens-B1-AVa-23

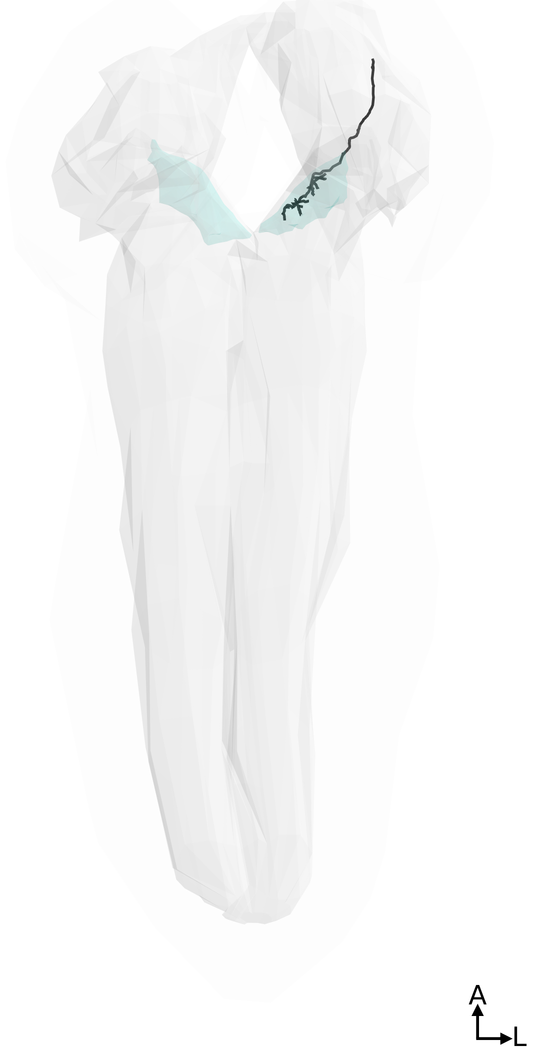

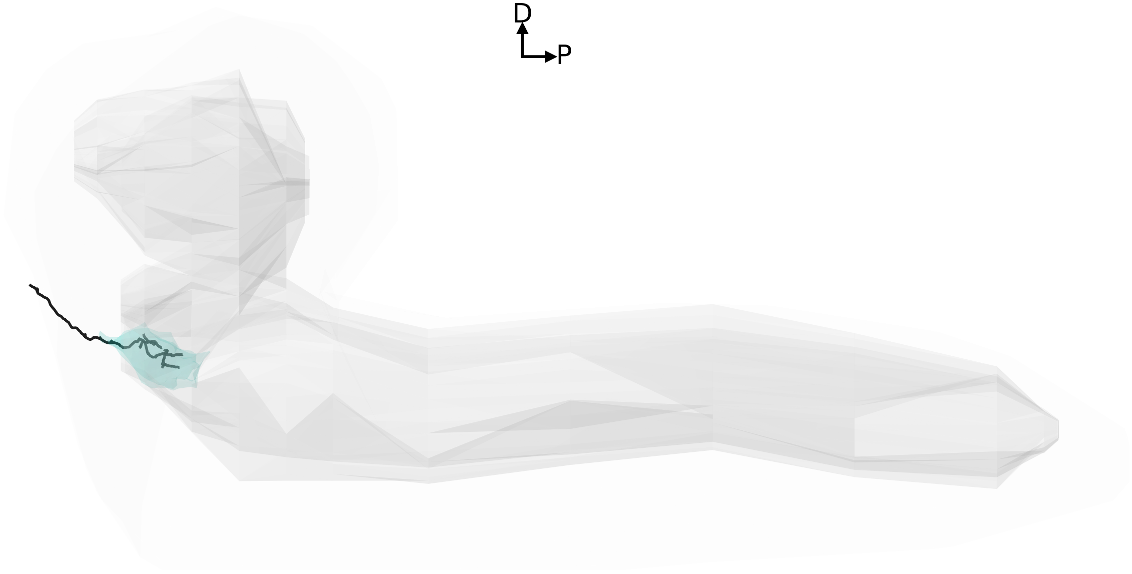

ID	*name*	SCACa	SCAVa	SCAVp	SCACal	SCACp	SCACpl	SCVM	IPCs	DMS	DH44	Se0ens	Se0ph	PMN LR	MN motor neurons	PaN motor neurons	olfactory PNs	gustatory PNs	multiglomerular PNs	unknown PNs	thermo PNs	visual PNs
15564782	AN-R-Sens-B1-AVa-23	0	3	0	0	0	0	0	0	0	0	6	0	0	0	0	0	0	0	0	0	0

ID: 15564706
name: AN-R-Sens-B1-AVa-24

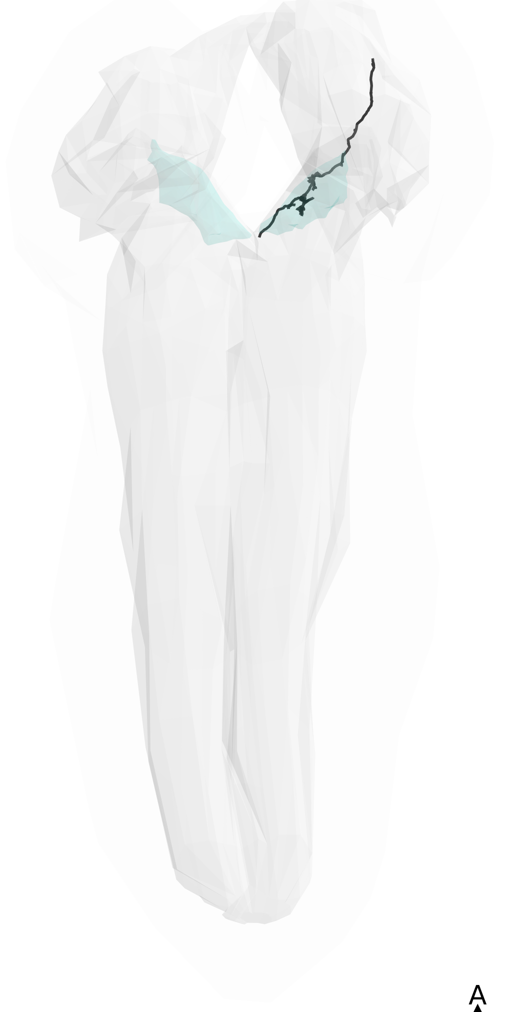

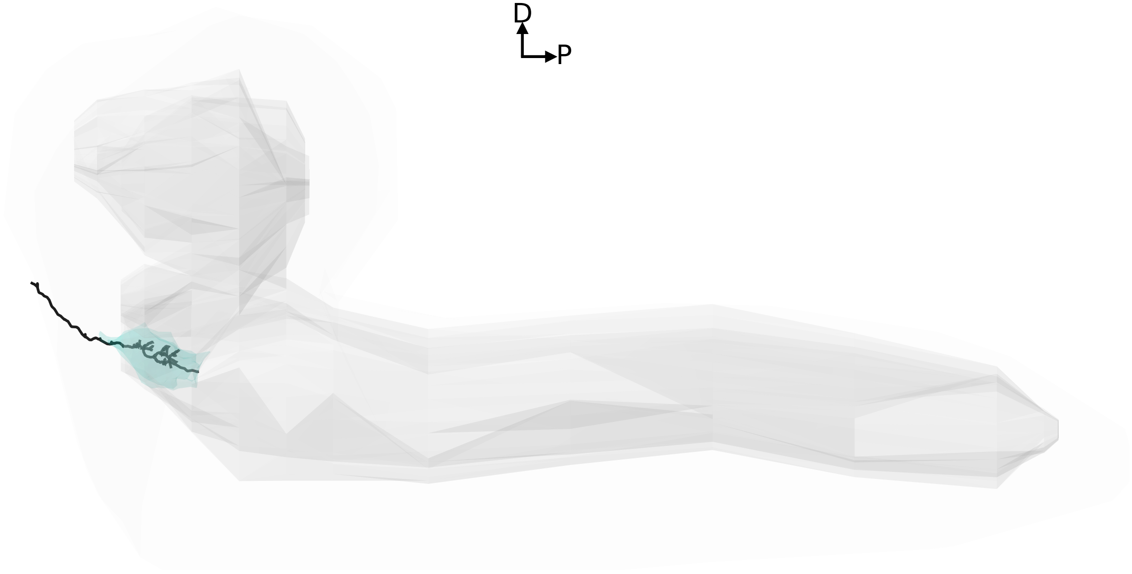

ID	*name*	SCACa	SCAVa	SCAVp	SCACal	SCACp	SCACpl	SCVM	IPCs	DMS	DH44	Se0ens	Se0ph	PMN LR	MN motor neurons	PaN motor neurons	olfactory PNs	gustatory PNs	multiglomerular PNs	unknown PNs	thermo PNs	visual PNs
15564706	AN-R-Sens-B1-AVa-24	0	6	0	0	0	0	0	0	0	1	10	3	0	0	0	0	0	0	0	0	0

ID: 15572414
name: AN-R-Sens-B1-AVa-25

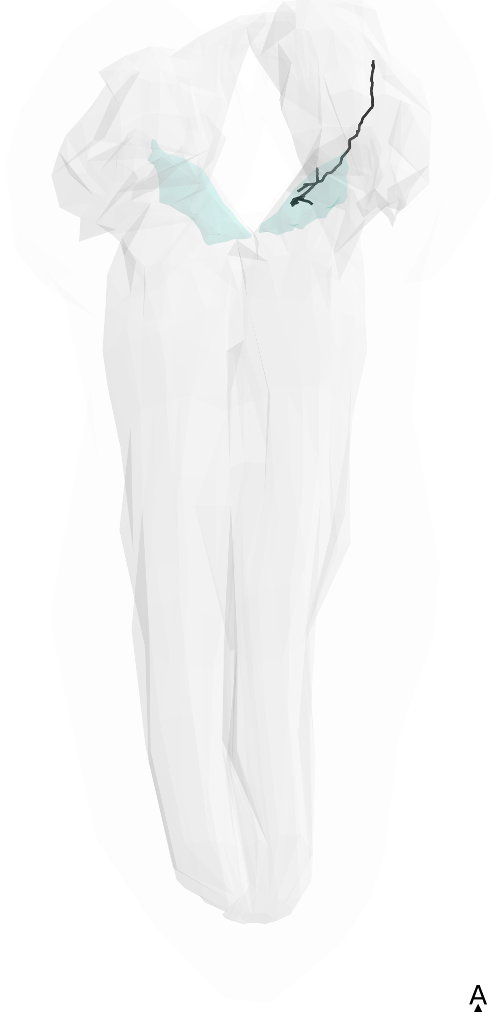

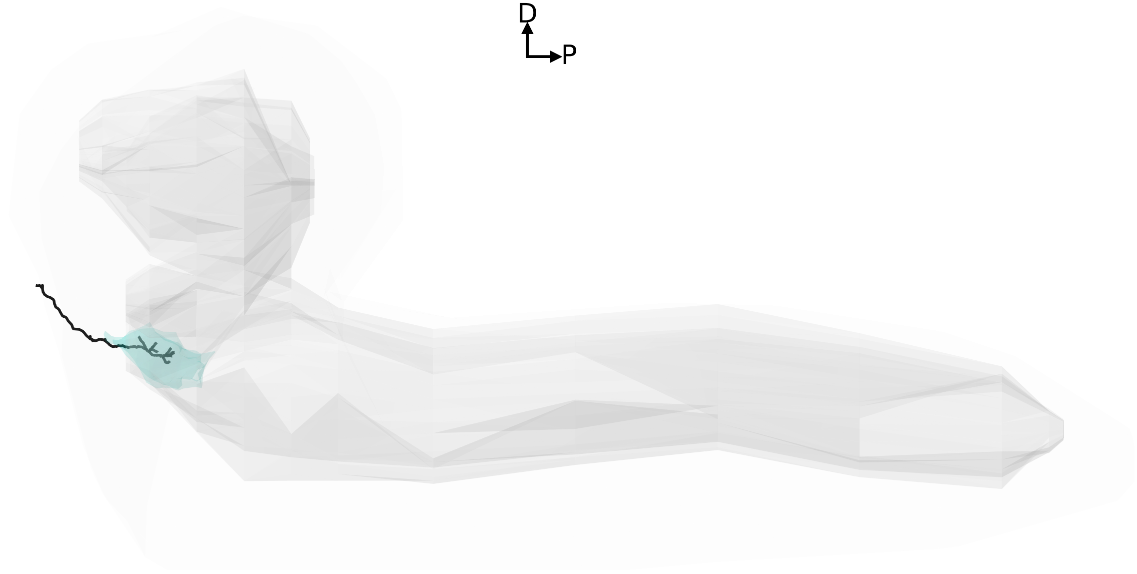

ID	name	SCACa	SCAVa	SCAVp	SCACal	SCACp	SCACpl	SCVM	IPCs	DMS	DH44	Se0ens	Se0ph	PMN LR	MN motor neurons	PaN motor neurons	olfactory PNs	gustatory PNs	multiglomerular PNs	unknown PNs	thermo PNs	visual PNs
15572414	AN-R-Sens-B1-AVa-25	0	5	0	0	0	0	0	0	0	0	4	2	0	0	0	0	0	0	0	0	0

ID: 15575327
name: AN-R-Sens-B1-AVa-26

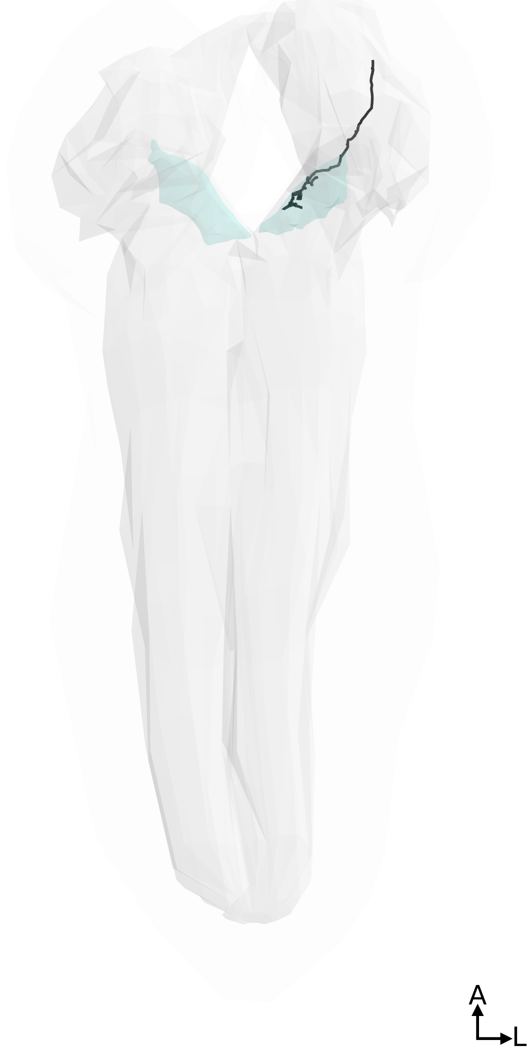

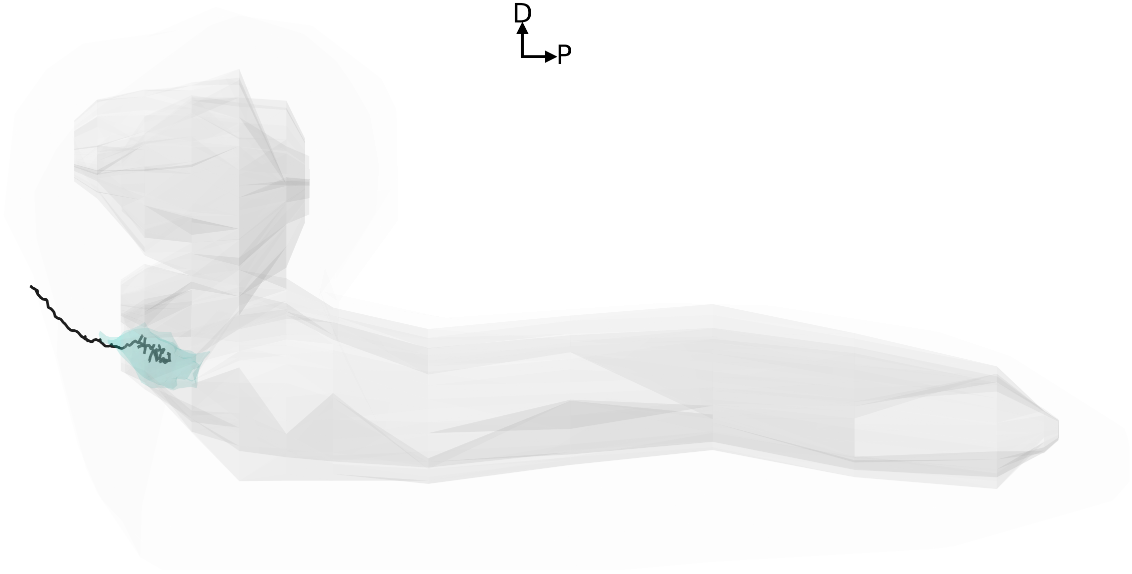

ID	*name*	SCACa	SCAVa	SCAVp	SCACal	SCACp	SCACpl	SCVM	IPCs	DMS	DH44	Se0ens	Se0ph	PMN LR	MN motor neurons	PaN motor neurons	olfactory PNs	gustatory PNs	multiglomerular PNs	unknown PNs	thermo PNs	visual PNs
15575327	AN-R-Sens-B1-AVa-26	0	3	0	0	0	0	0	0	0	3	5	0	0	0	0	0	0	0	0	0	0

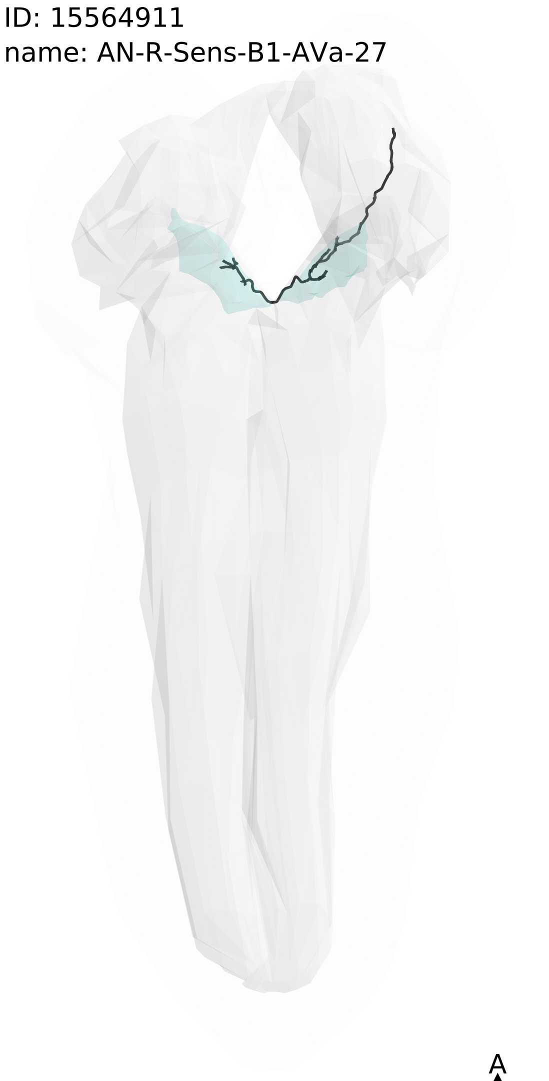

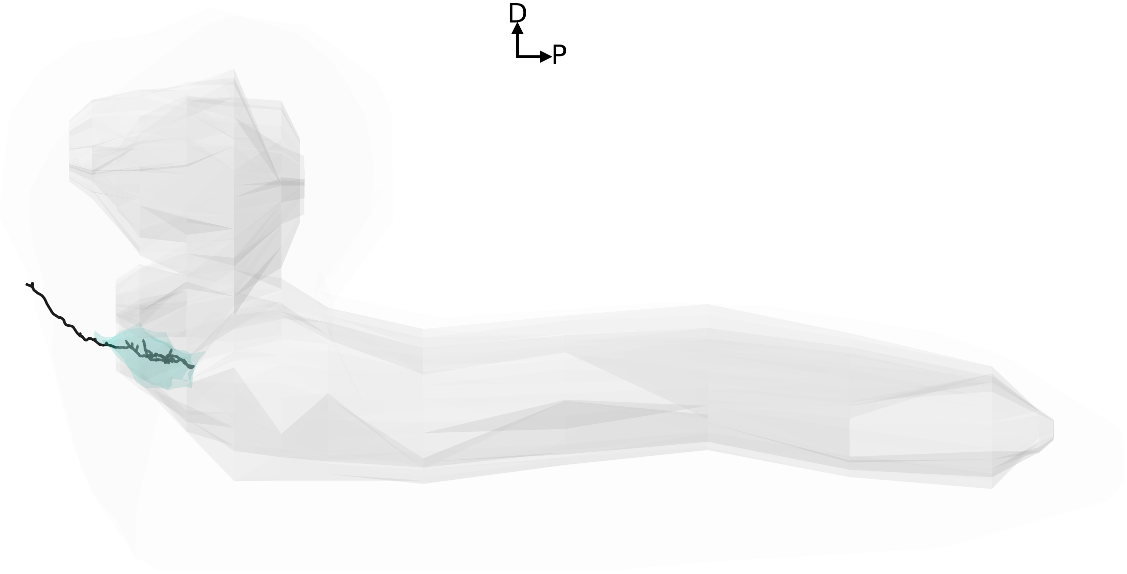

ID	*name*	SCACa	SCAVa	SCAVp	SCACal	SCACp	SCACpl	SCVM	IPCs	DMS	DH44	Se0ens	Se0ph	PMN LR	MN motor neurons	PaN motor neurons	olfactory PNs	gustatory PNs	multiglomerular PNs	unknown PNs	thermo PNs	visual PNs
15564911	AN-R-Sens-B1-AVa-27	0	3	0	0	0	0	0	0	0	0	5	2	0	0	0	0	0	0	0	0	0

ID: 15569561
name: AN-R-Sens-B1-AVa-28

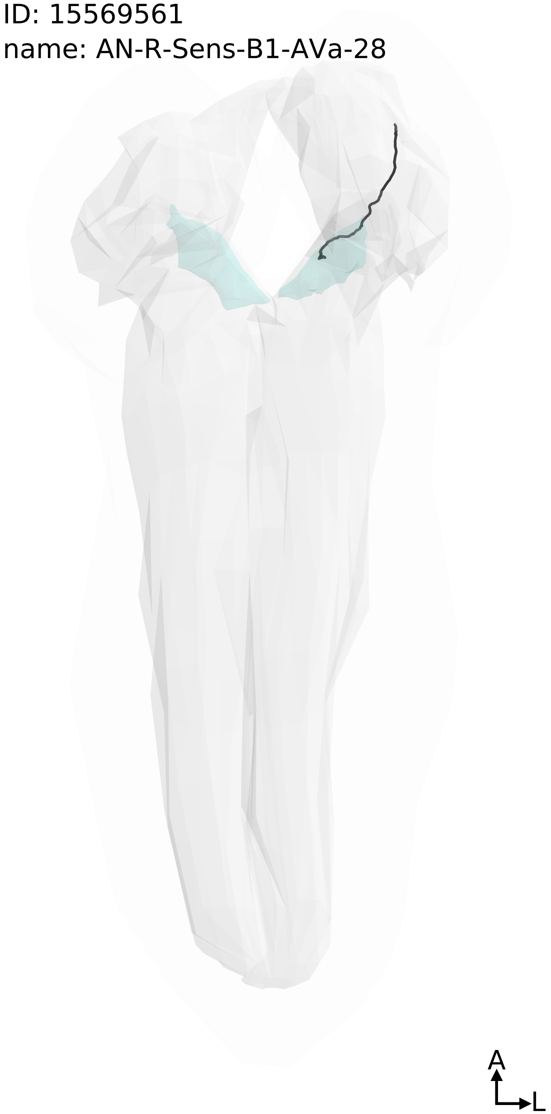

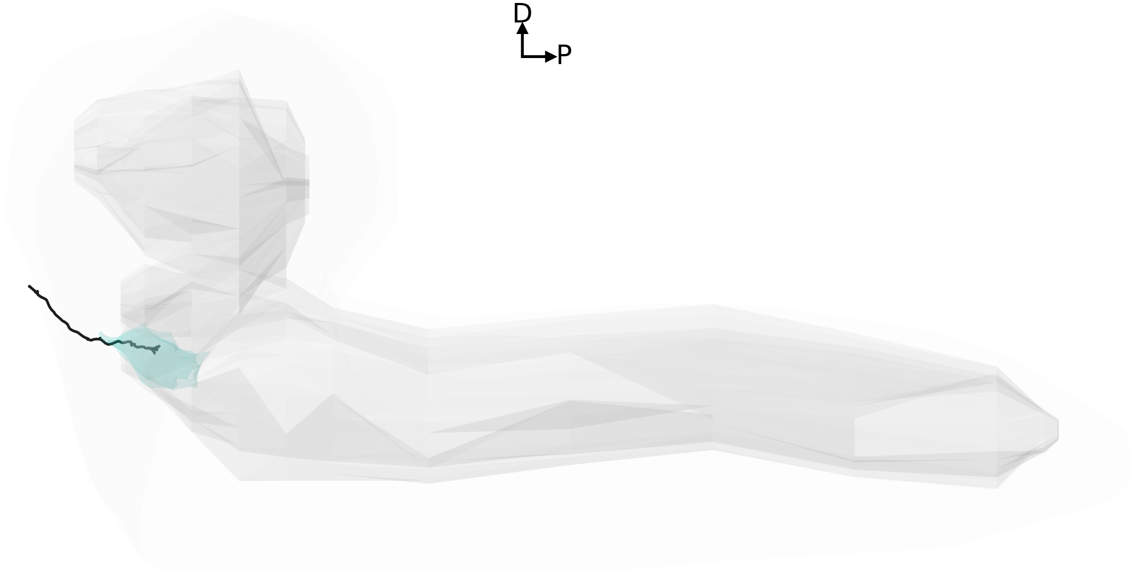

ID	*name*	SCACa	SCAVa	SCAVp	SCACal	SCACp	SCACpl	SCVM	IPCs	DMS	DH44	Se0ens	Se0ph	PMN LR	MN motor neurons	PaN motor neurons	olfactory PNs	gustatory PNs	multiglomerular PNs	unknown PNs	thermo PNs	visual PNs
15569561	AN-R-Sens-B1-AVa-28	1	2	0	0	0	0	0	1	0	0	4	1	0	0	0	0	0	0	0	0	0

ID: 15564661
name: AN-R-Sens-B1-AVa-29

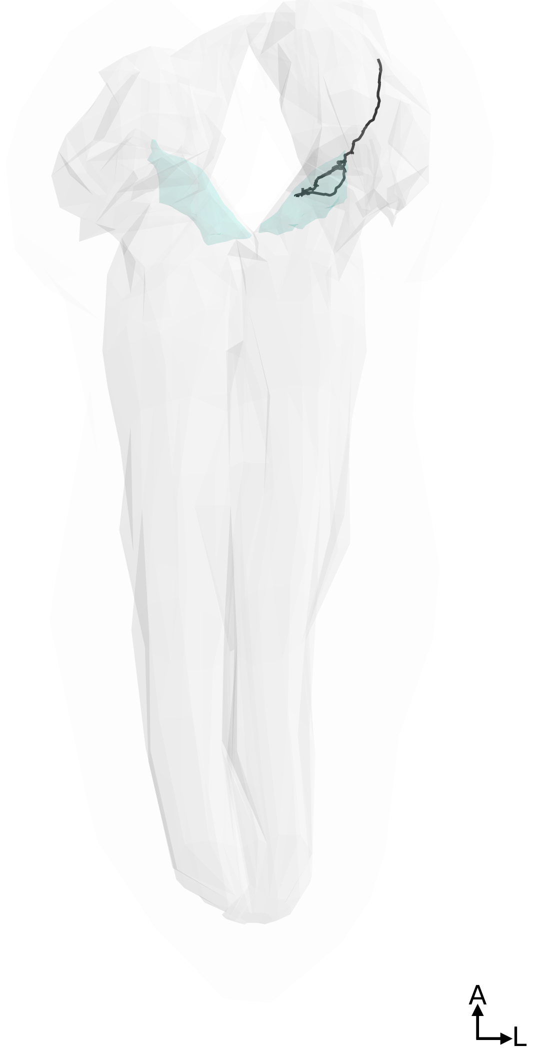

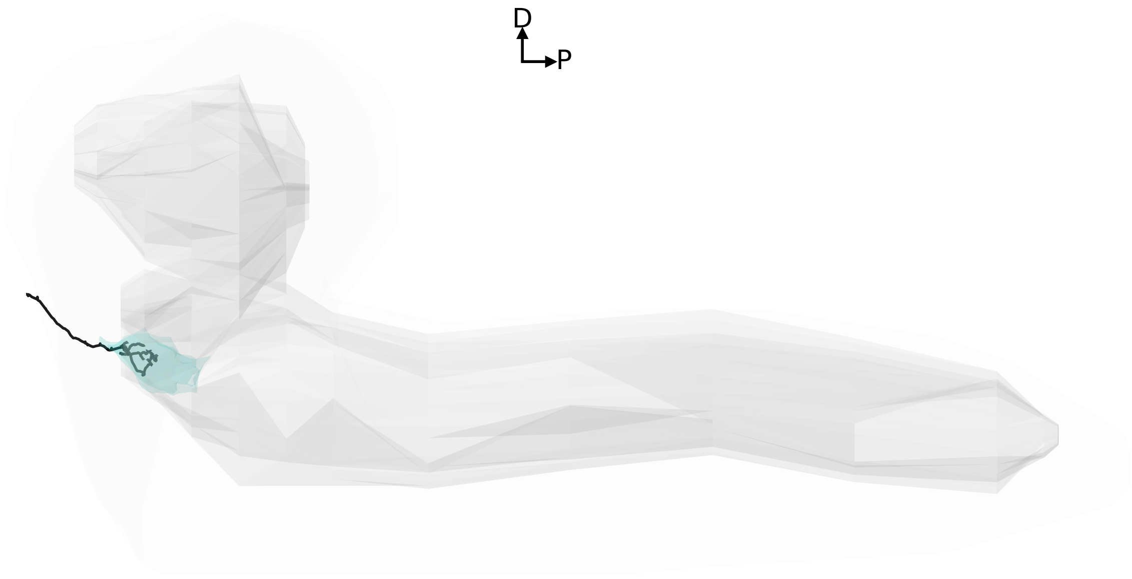

ID	*name*	SCACa	SCAVa	SCAVp	SCACal	SCACp	SCACpl	SCVM	IPCs	DMS	DH44	Se0ens	Se0ph	PMN LR	MN motor neurons	PaN motor neurons	olfactory PNs	gustatory PNs	multiglomerular PNs	unknown PNs	thermo PNs	visual PNs
15564661	AN-R-Sens-B1-AVa-29	0	12	0	0	0	0	0	0	0	0	2	6	0	0	0	0	0	0	0	0	0

ID: 15546924
name: AN-R-Sens-B1-AVa-30

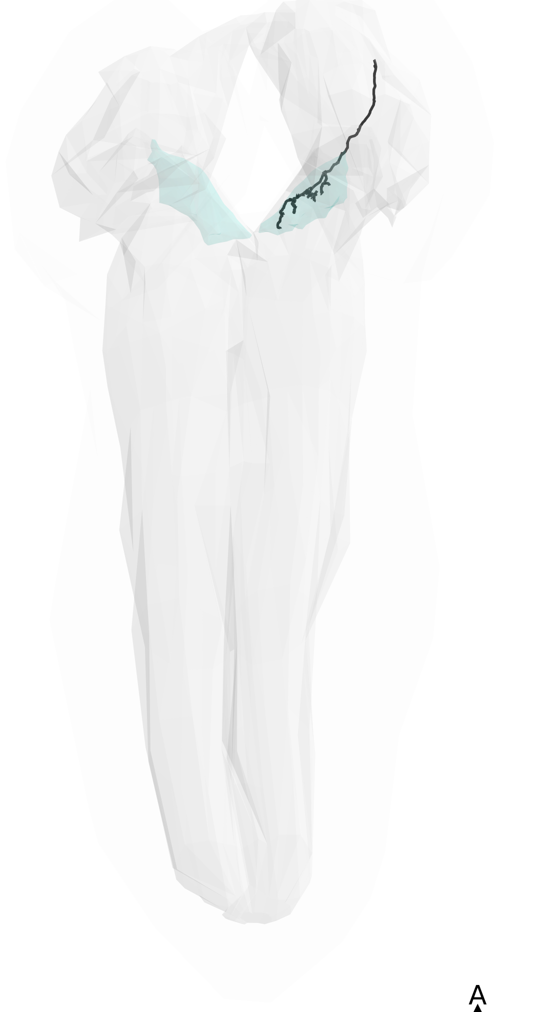

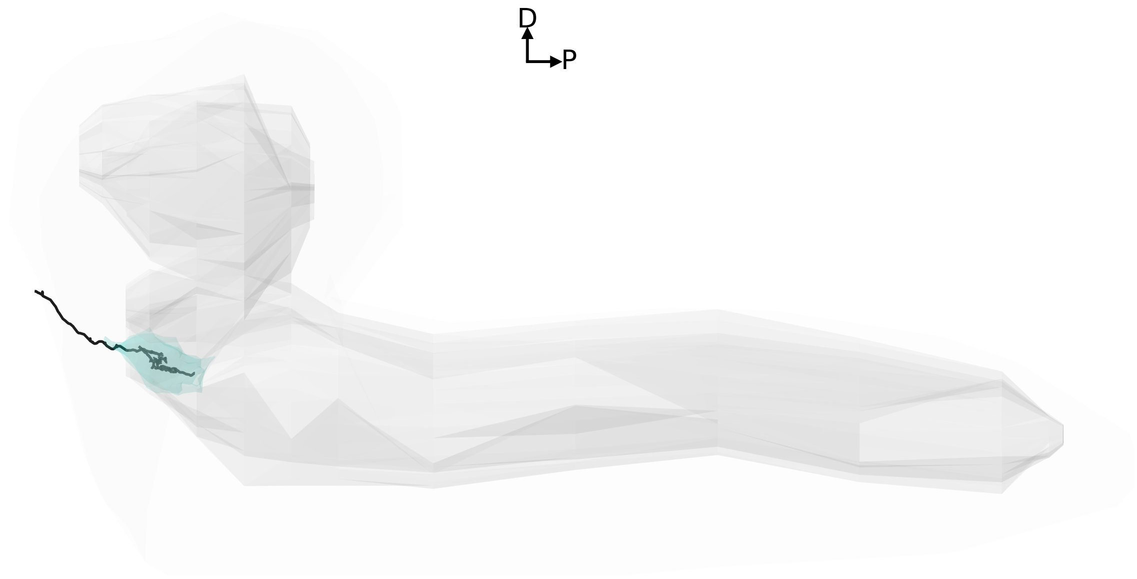

ID	*name*	SCACa	SCAVa	SCAVp	SCACal	SCACp	SCACpl	SCVM	IPCs	DMS	DH44	Se0ens	Se0ph	PMN LR	MN motor neurons	PaN motor neurons	olfactory PNs	gustatory PNs	multiglomerular PNs	unknown PNs	thermo PNs	visual PNs
15546924	AN-R-Sens-B1-AVa-30	0	26	0	0	0	0	0	0	0	0	2	1	0	0	0	0	0	0	0	0	0

ID: 15543394
name: AN-R-Sens-B1-AVa-31

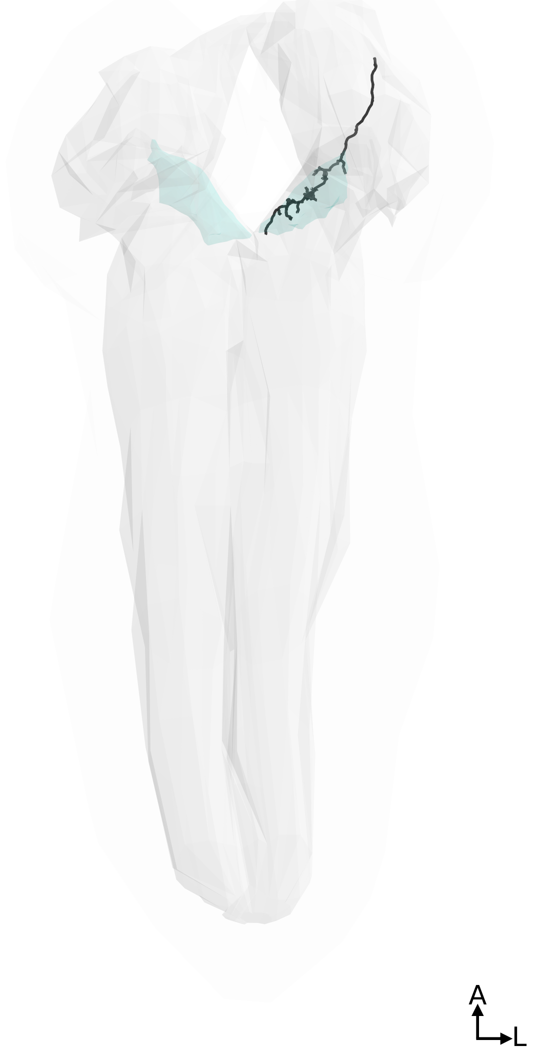

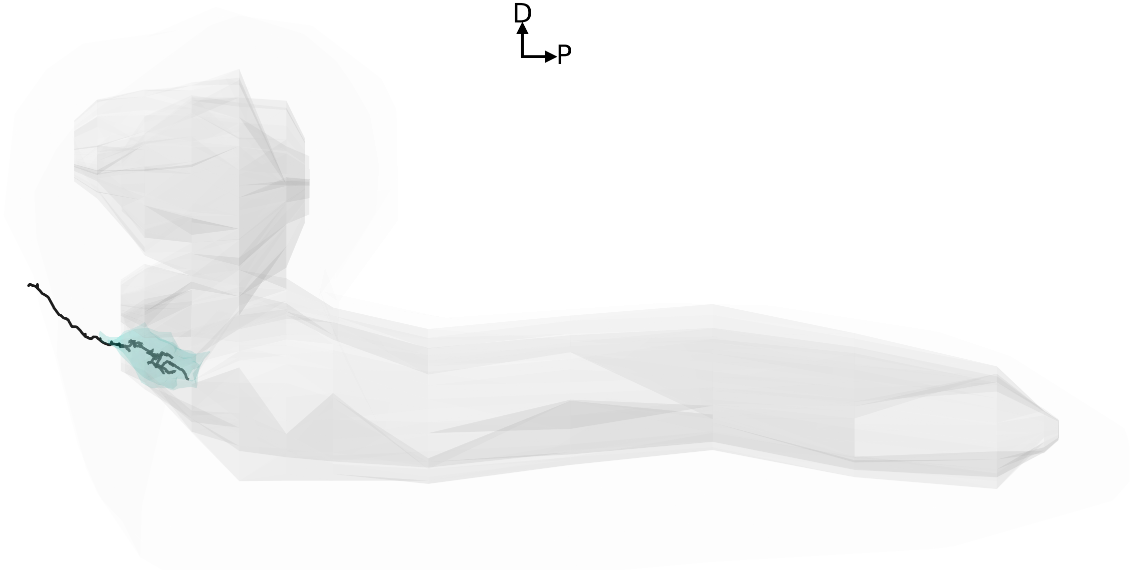

ID	*name*	SCACa	SCAVa	SCAVp	SCACal	SCACp	SCACpl	SCVM	IPCs	DMS	DH44	Se0ens	Se0ph	PMN LR	MN motor neurons	PaN motor neurons	olfactory PNs	gustatory PNs	multiglomerular PNs	unknown PNs	thermo PNs	visual PNs
15543394	AN-R-Sens-B1-AVa-31	0	20	0	0	0	0	0	0	0	0	2	1	2	0	0	0	0	0	0	0	0

ID: 17951153
name: AN-R-Sens-B1-AVa-32

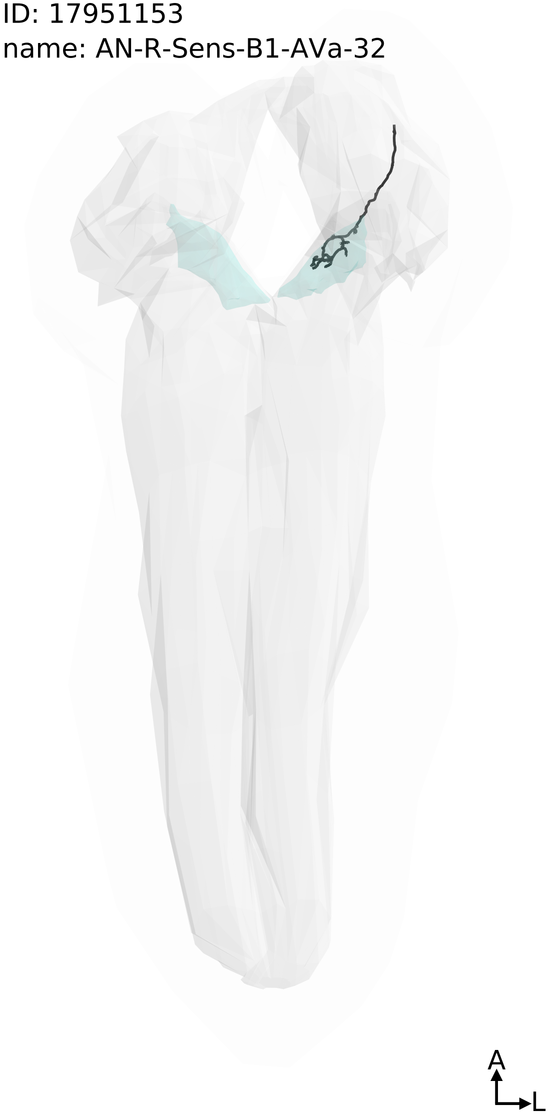

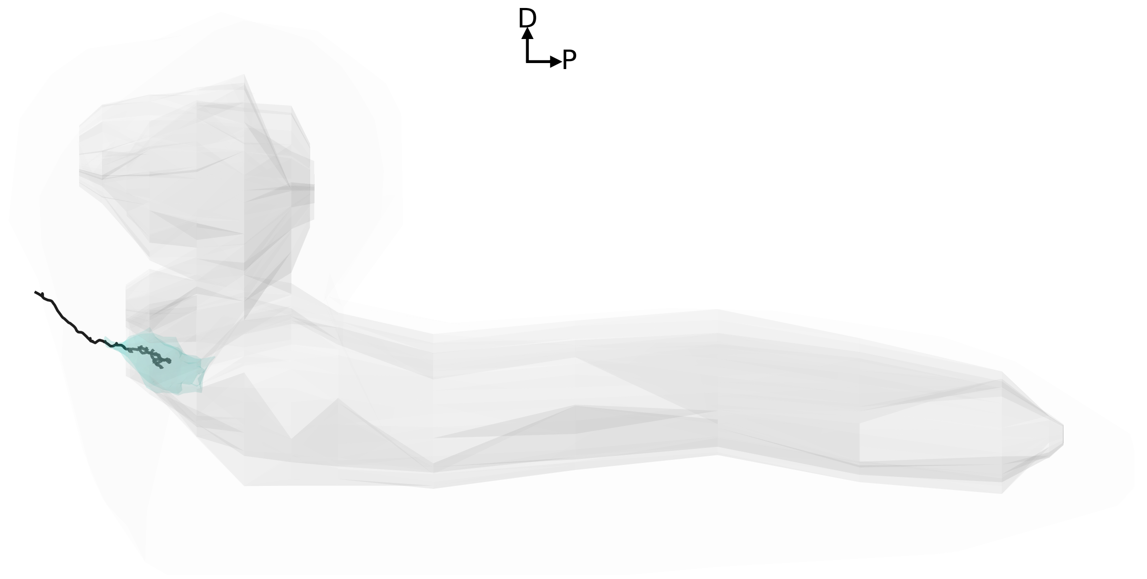

ID	*name*	SCACa	SCAVa	SCAVp	SCACal	SCACp	SCACpl	SCVM	IPCs	DMS	DH44	Se0ens	Se0ph	PMN LR	MN motor neurons	PaN motor neurons	olfactory PNs	gustatory PNs	multiglomerular PNs	unknown PNs	thermo PNs	visual PNs
17951153	AN-R-Sens-B1-AVa-32	1	14	0	0	0	0	0	0	0	0	0	1	0	0	0	0	0	0	0	0	0

ID: 15547732
name: AN-R-Sens-B1-AVa-33

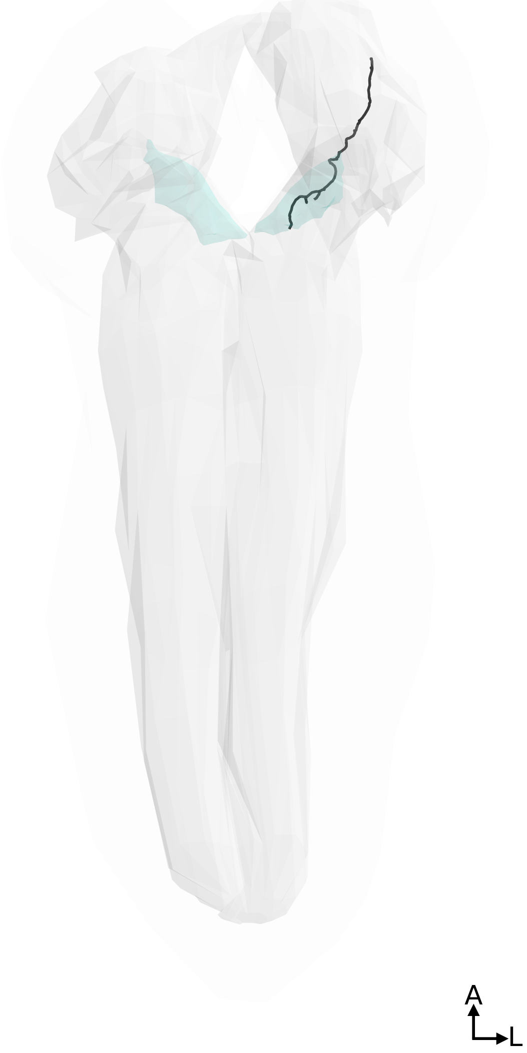

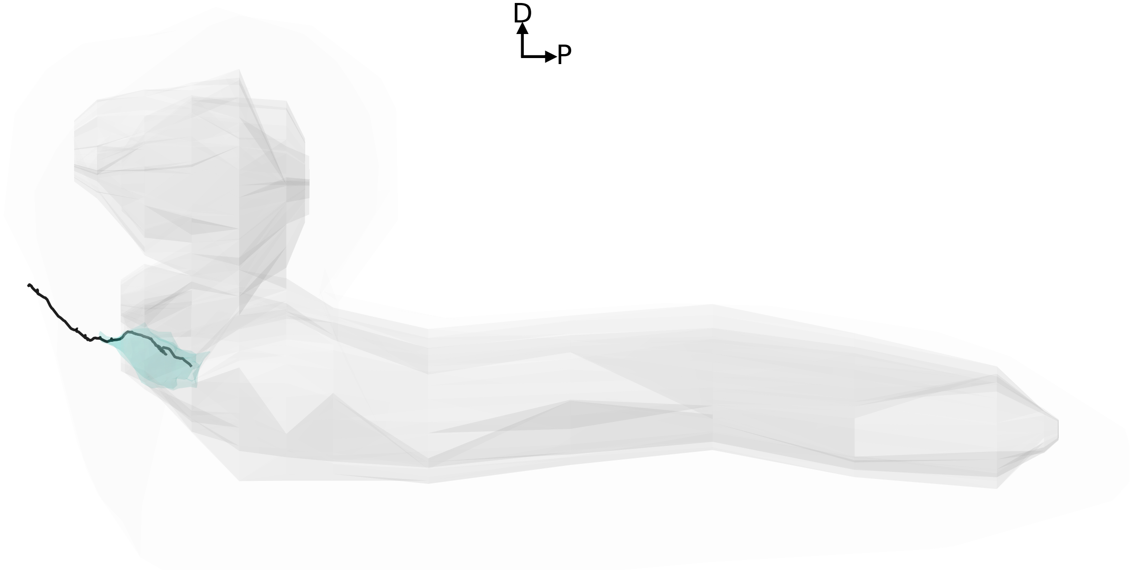

ID	*name*	SCACa	SCAVa	SCAVp	SCACal	SCACp	SCACpl	SCVM	IPCs	DMS	DH44	Se0ens	Se0ph	PMN LR	MN motor neurons	PaN motor neurons	olfactory PNs	gustatory PNs	multiglomerular PNs	unknown PNs	thermo PNs	visual PNs
15547732	AN-R-Sens-B1-AVa-33	0	3	0	0	0	0	0	0	0	0	3	0	0	0	0	0	0	0	0	0	0

ID: 15574941
name: AN-R-Sens-B1-AVa-34

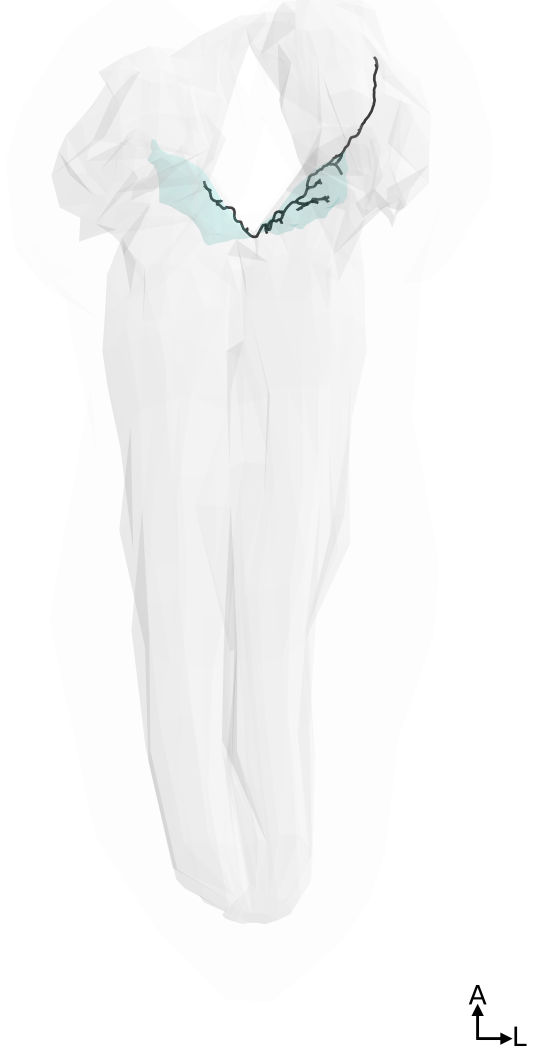

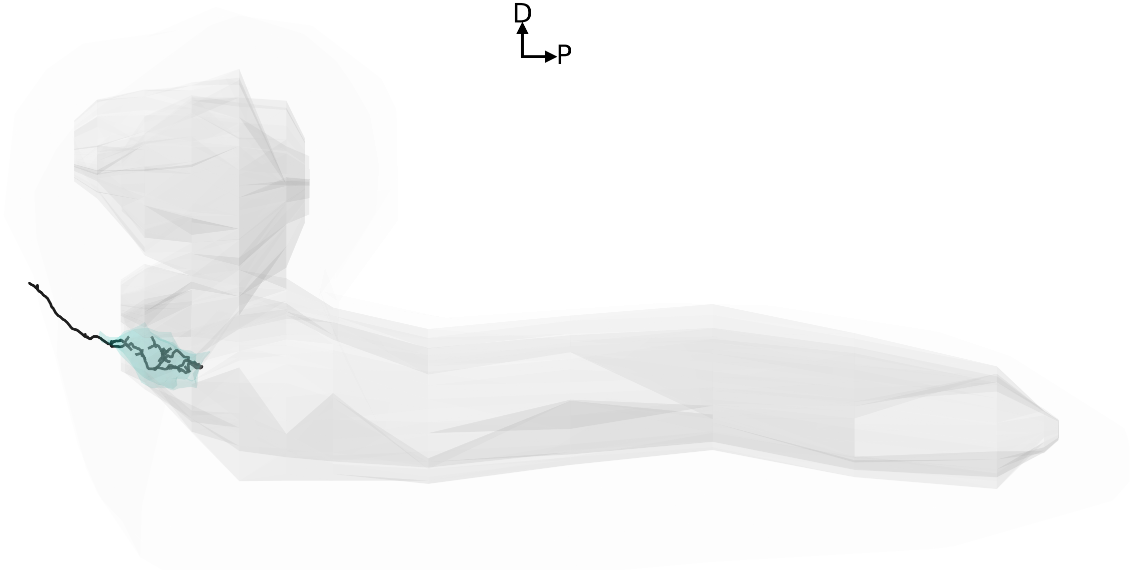

ID	*name*	SCACa	SCAVa	SCAVp	SCACal	SCACp	SCACpl	SCVM	IPCs	DMS	DH44	Se0ens	Se0ph	PMN LR	MN motor neurons	PaN motor neurons	olfactory PNs	gustatory PNs	multiglomerular PNs	unknown PNs	thermo PNs	visual PNs
15574941	AN-R-Sens-B1-AVa-34	1	6	0	0	0	0	0	0	0	0	2	0	1	0	0	0	0	0	0	0	0

ID: 15575232
name: AN-R-Sens-B1-AVa-35

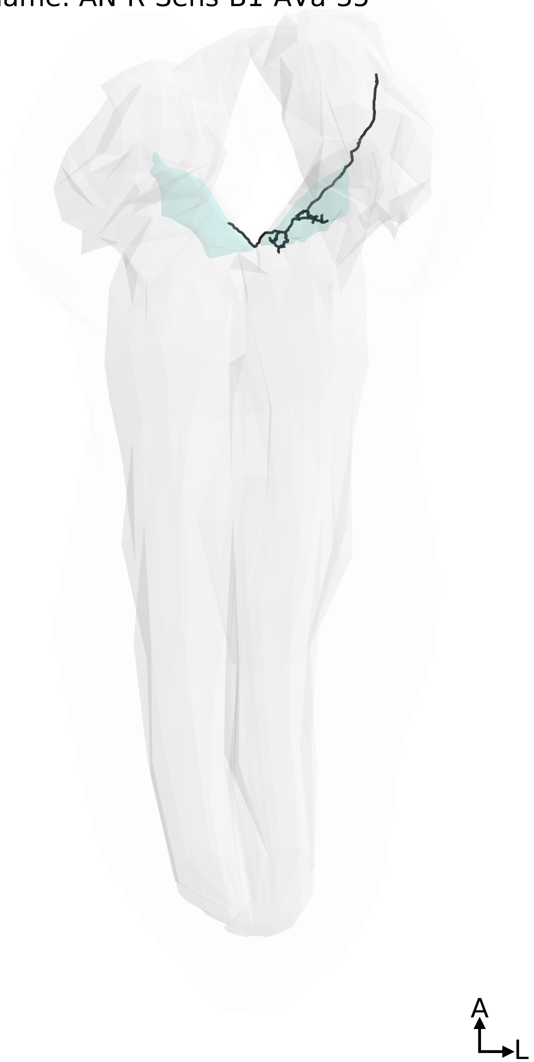

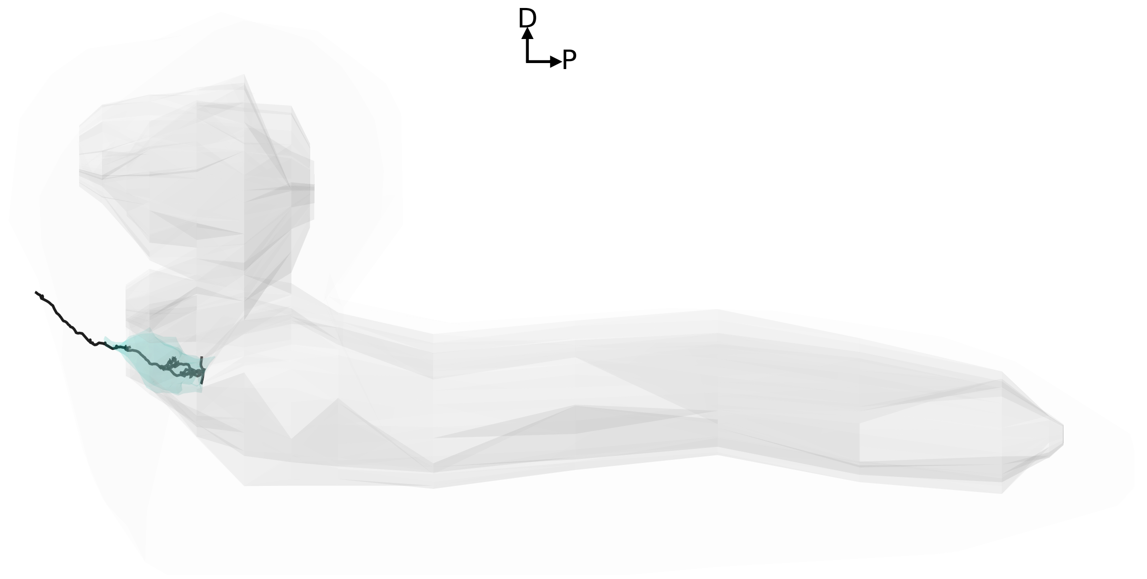

ID	name	SCACa	SCAVa	SCAVp	SCACal	SCACp	SCACpl	SCVM	IPCs	DMS	DH44	Se0ens	Se0ph	PMN LR	MN motor neurons	PaN motor neurons	olfactory PNs	gustatory PNs	multiglomerular PNs	unknown PNs	thermo PNs	visual PNs
15575232	AN-R-Sens-B1-AVa-35	0	7	0	0	0	0	0	0	0	0	0	0	4	0	0	0	0	0	0	0	0

ID: 15575052
name: AN-R-Sens-B1-AVa-36

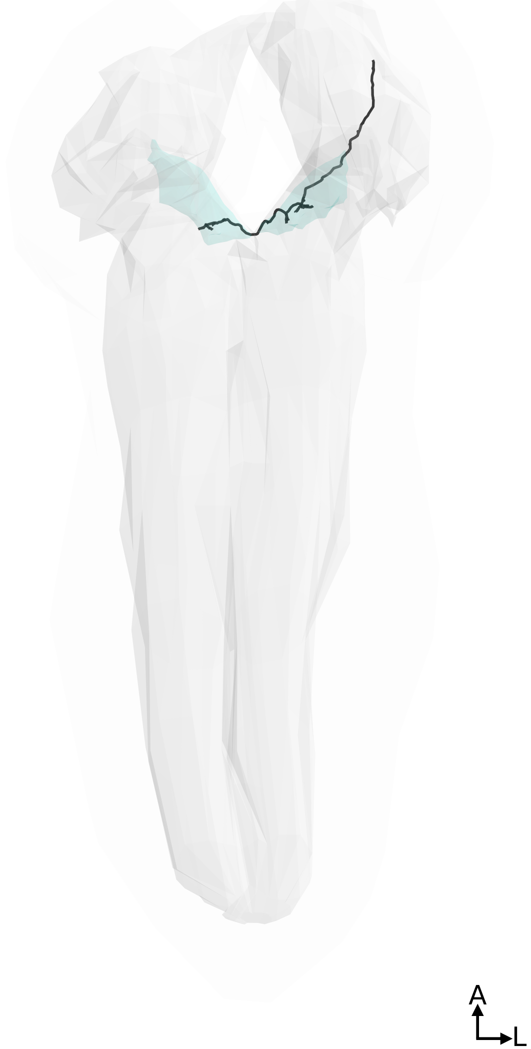

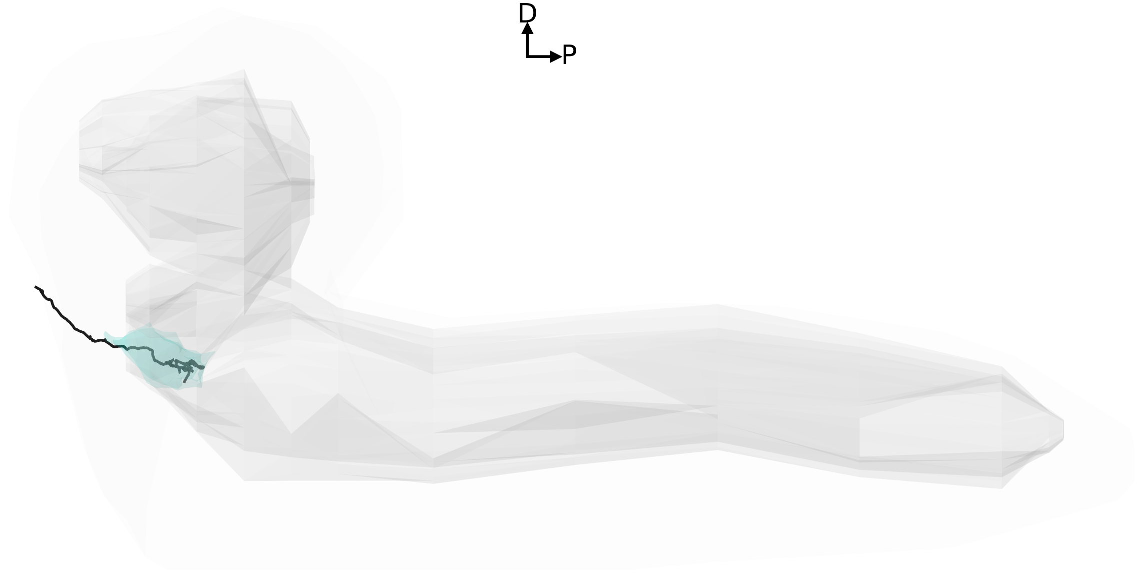

ID	name	SCACa	SCAVa	SCAVp	SCACal	SCACp	SCACpl	SCVM	IPCs	DMS	DH44	Se0ens	Se0ph	PMN LR	MN motor neurons	PaN motor neurons	olfactory PNs	gustatory PNs	multiglomerular PNs	unknown PNs	thermo PNs	visual PNs
15575052	AN-R-Sens-B1-AVa-36	0	4	0	0	0	0	0	0	0	0	0	0	4	0	0	0	0	0	0	0	0

ID: 15564882
name: AN-R-Sens-B1-AVa-37

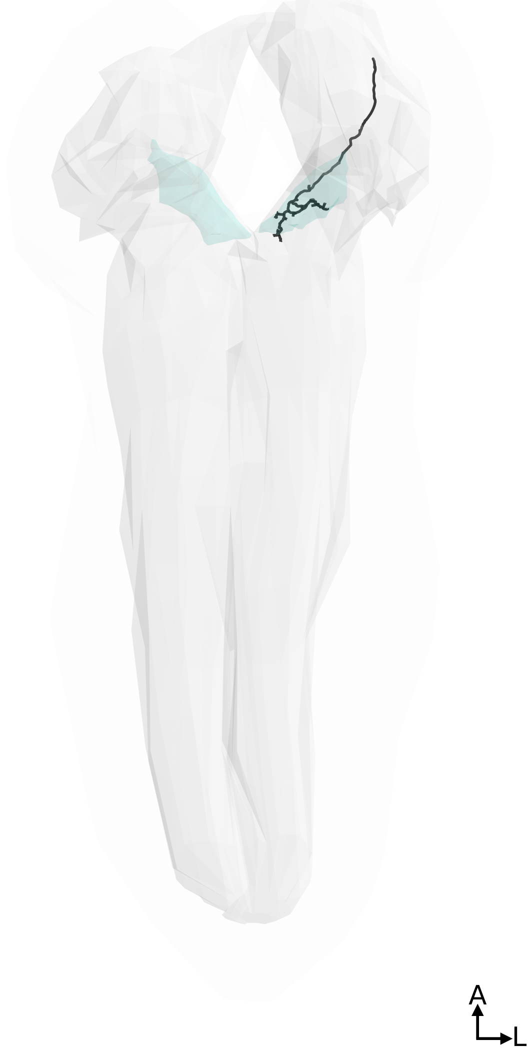

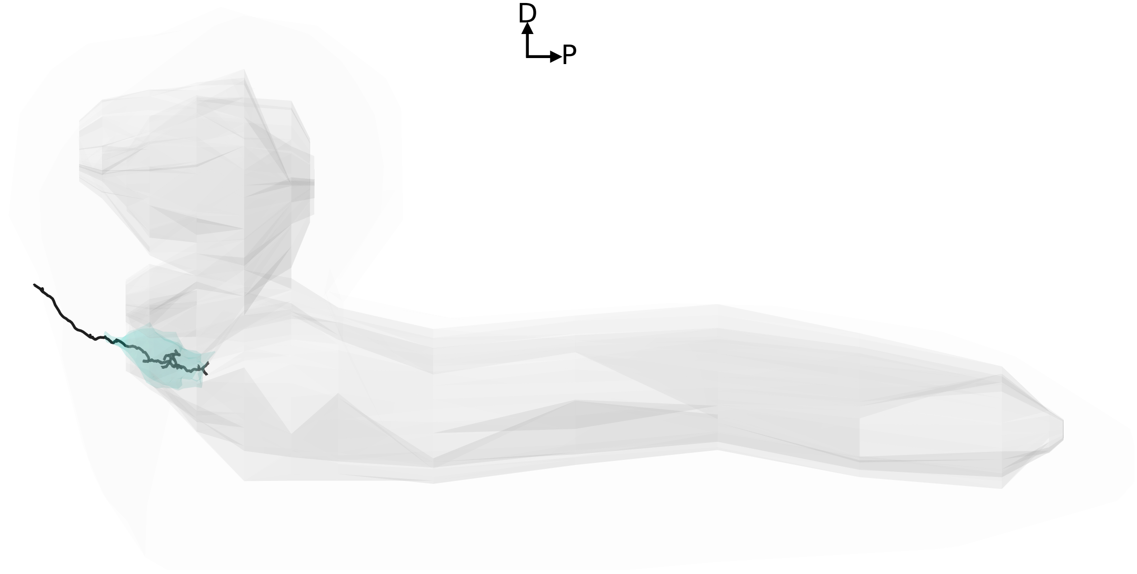

ID	name	SCACa	SCAVa	SCAVp	SCACal	SCACp	SCACpl	SCVM	IPCs	DMS	DH44	Se0ens	Se0ph	PMN LR	MN motor neurons	PaN motor neurons	olfactory PNs	gustatory PNs	multiglomerular PNs	unknown PNs	thermo PNs	visual PNs
15564882	AN-R-Sens-B1-AVa-37	0	3	0	0	0	0	0	0	0	0	1	1	2	0	0	0	0	0	0	0	0

ID: 15574508
name: AN-R-Sens-B1-AVa-38

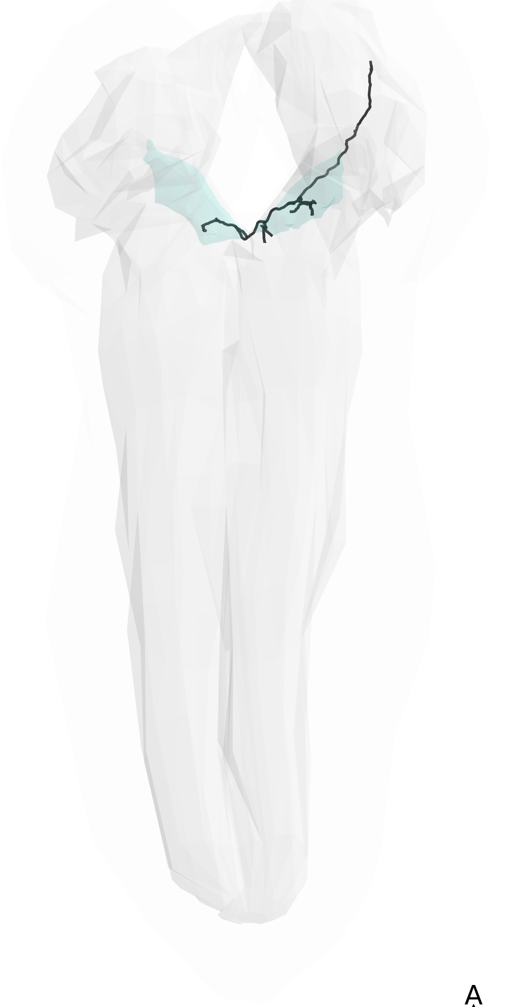

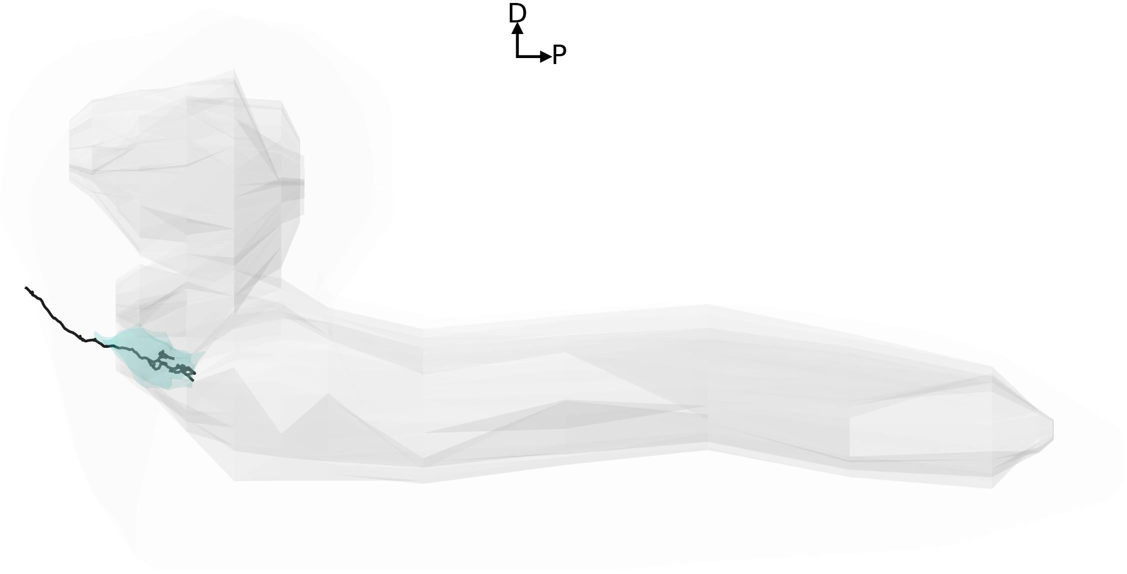

ID	*name*	SCACa	SCAVa	SCAVp	SCACal	SCACp	SCACpl	SCVM	IPCs	DMS	DH44	Se0ens	Se0ph	PMN LR	MN motor neurons	PaN motor neurons	olfactory PNs	gustatory PNs	multiglomerular PNs	unknown PNs	thermo PNs	visual PNs
15574508	AN-R-Sens-B1-AVa-38	0	2	0	0	0	0	0	0	0	0	0	2	2	0	0	0	0	0	0	0	0

ID: 15587166
name: AN-R-Sens-B1-AVa-39

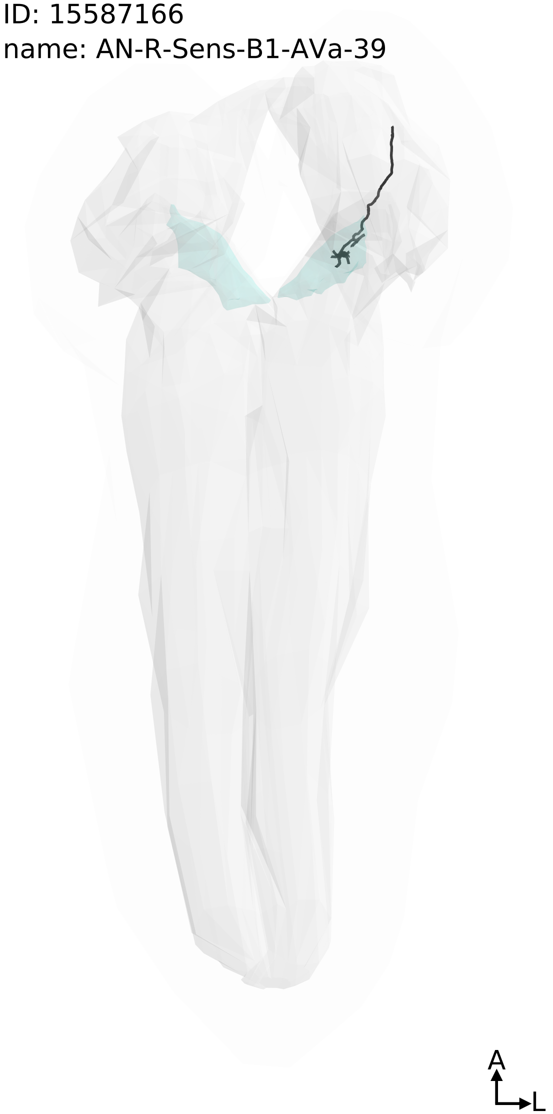

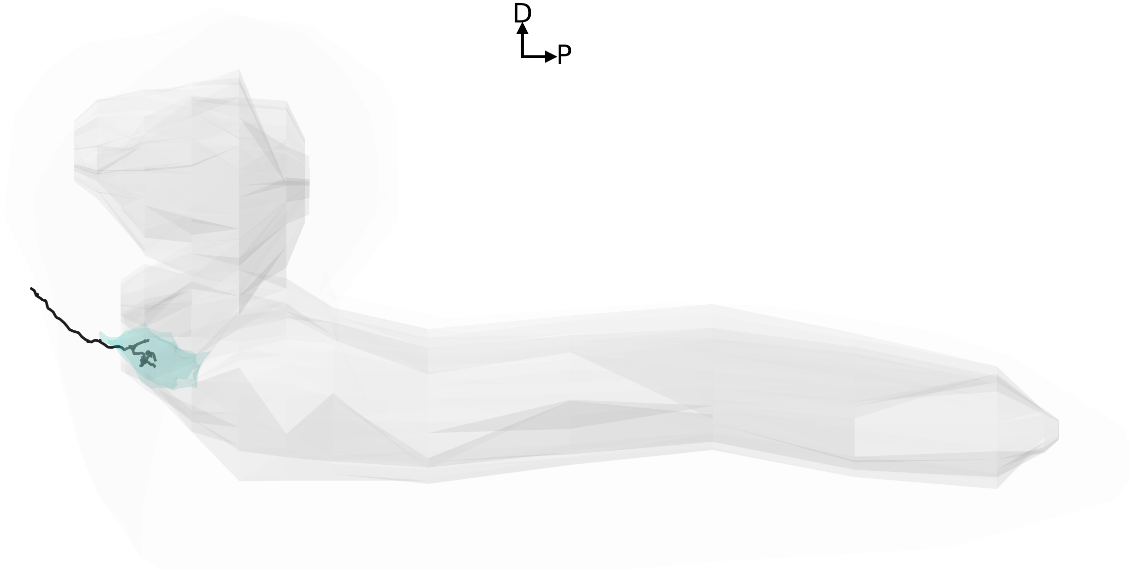

ID	name	SCACa	SCAVa	SCAVp	SCACal	SCACp	SCACpl	SCVM	IPCs	DMS	DH44	Se0ens	Se0ph	PMN LR	MN motor neurons	PaN motor neurons	olfactory PNs	gustatory PNs	multiglomerular PNs	unknown PNs	thermo PNs	visual PNs
15587166	AN-R-Sens-B1-AVa-39	0	3	0	0	0	0	0	0	0	0	0	0	3	0	0	0	0	0	0	0	0

ID: 15576194
name: AN-R-Sens-B1-AVa-40

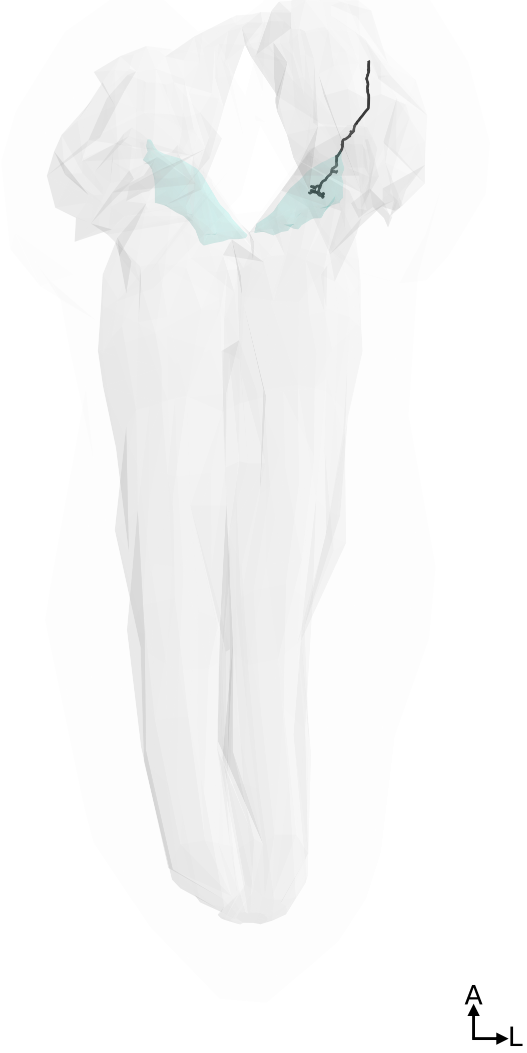

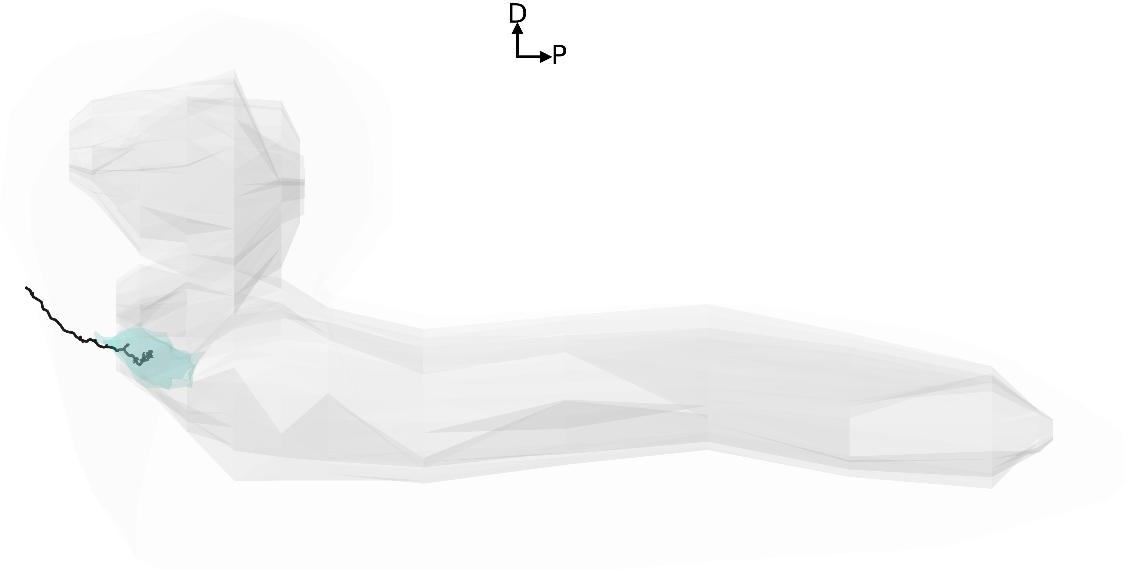

ID	*name*	SCACa	SCAVa	SCAVp	SCACal	SCACp	SCACpl	SCVM	IPCs	DMS	DH44	Se0ens	Se0ph	PMN LR	MN motor neurons	PaN motor neurons	olfactory PNs	gustatory PNs	multiglomerular PNs	unknown PNs	thermo PNs	visual PNs
15576194	AN-R-Sens-B1-AVa-40	0	1	0	0	0	0	0	0	0	0	1	0	1	0	0	0	0	0	0	0	0

ID: 15567466
name: AN-R-Sens-B1-AVa-41

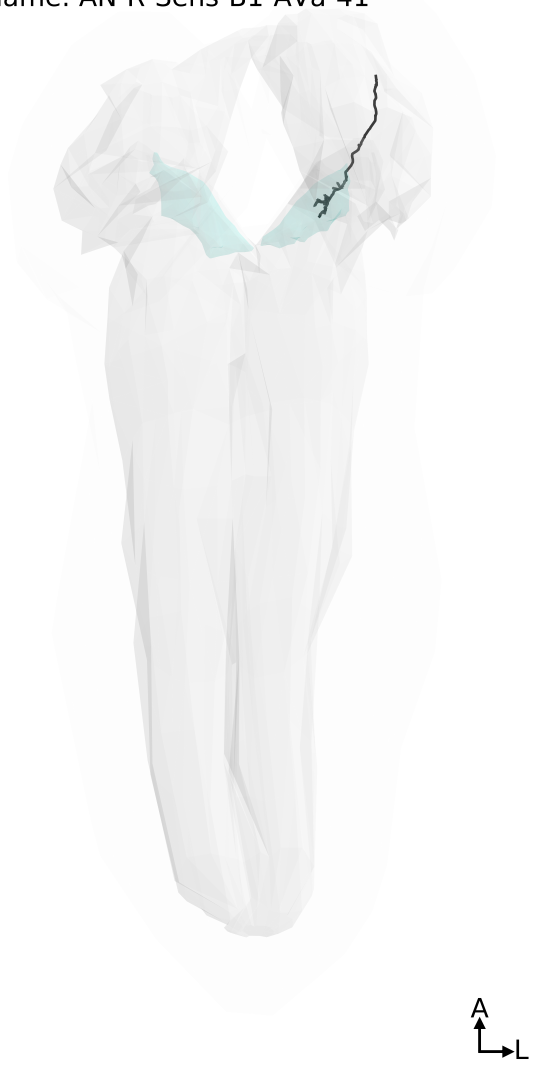

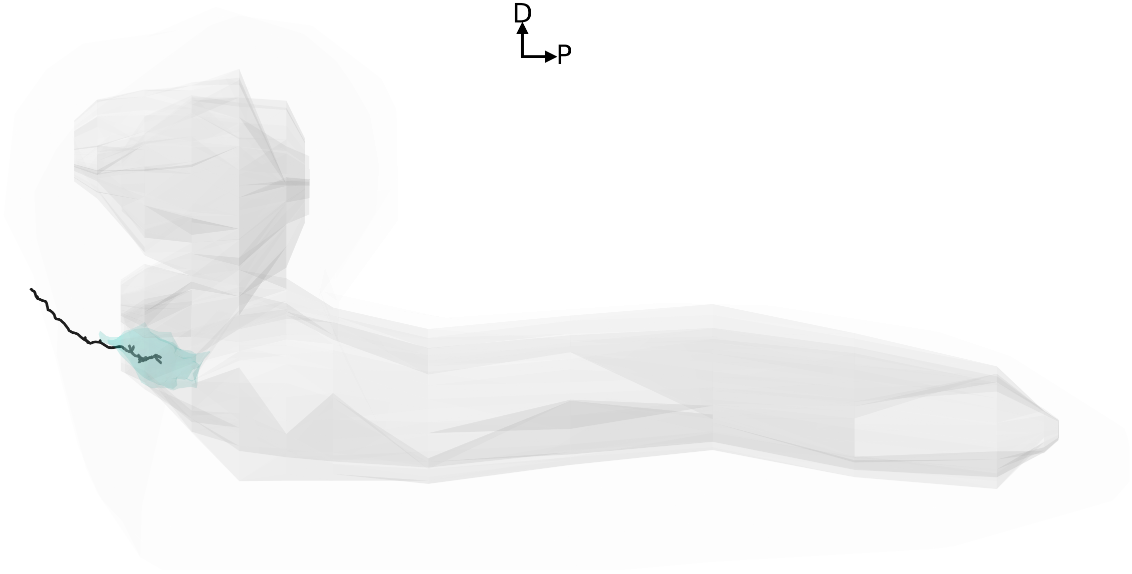

ID	name	SCACa	SCAVa	SCAVp	SCACal	SCACp	SCACpl	SCVM	IPCs	DMS	DH44	Se0ens	Se0ph	PMN LR	MN motor neurons	PaN motor neurons	olfactory PNs	gustatory PNs	multiglomerular PNs	unknown PNs	thermo PNs	visual PNs
15567466	AN-R-Sens-B1-AVa-41	0	0	0	0	0	0	0	0	0	0	0	0	0	0	0	0	0	0	0	0	0

ID: 15542354
name: AN-R-Sens-B1-AVa-42

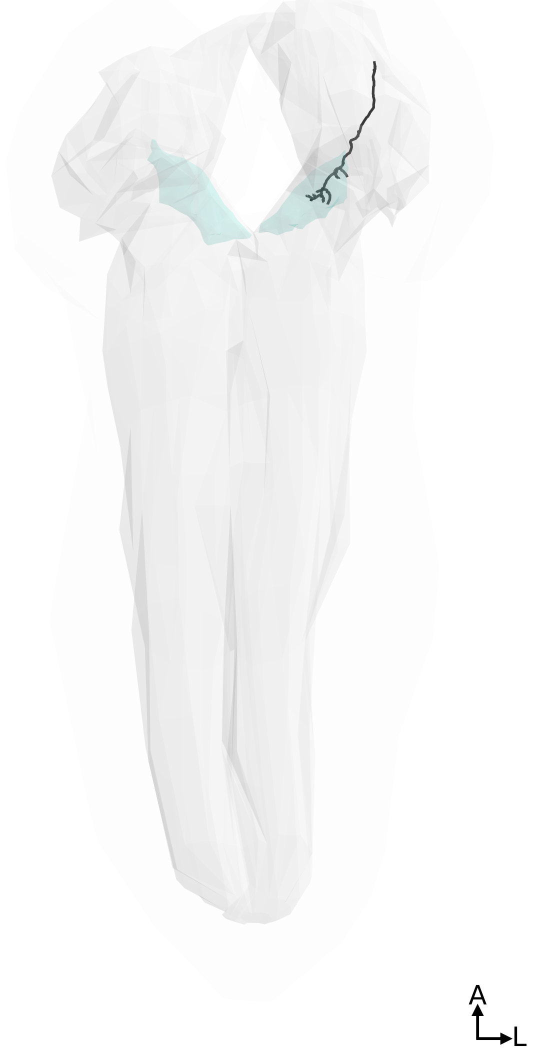

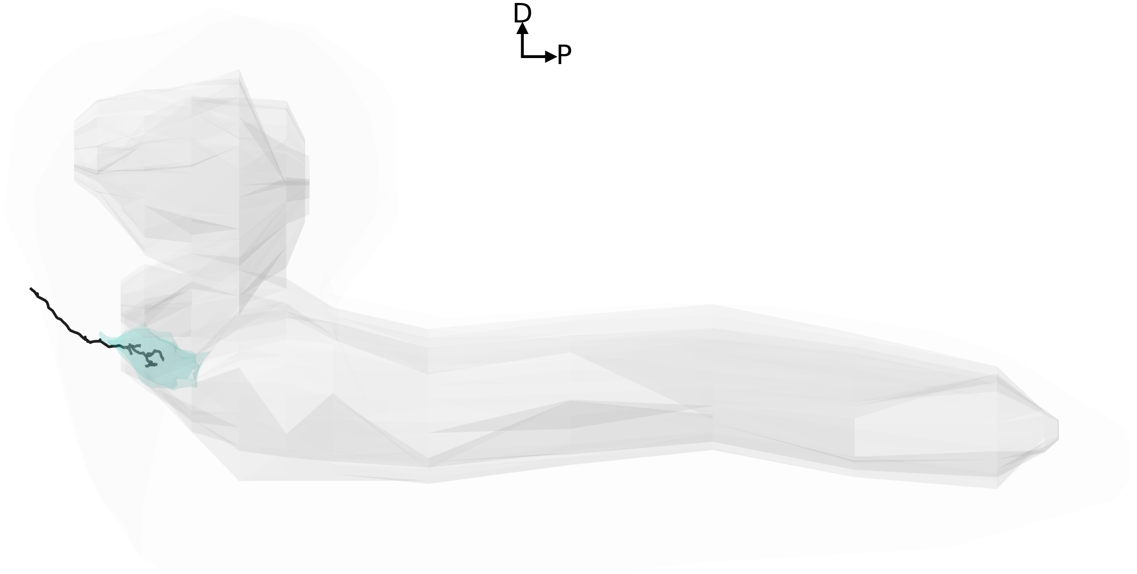

ID	*name*	SCACa	SCAVa	SCAVp	SCACal	SCACp	SCACpl	SCVM	IPCs	DMS	DH44	Se0ens	Se0ph	PMN LR	MN motor neurons	PaN motor neurons	olfactory PNs	gustatory PNs	multiglomerular PNs	unknown PNs	thermo PNs	visual PNs
15542354	AN-R-Sens-B1-AVa-42	0	2	0	0	0	0	0	0	0	0	0	0	2	0	0	0	0	0	0	0	0

ID: 15748726
name: AN-R-Sens-B1-AVa-43

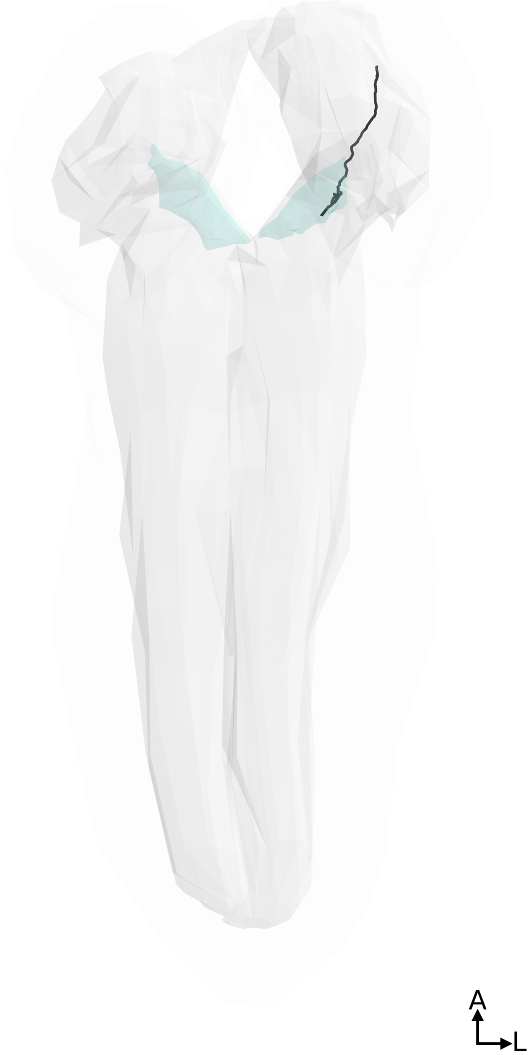

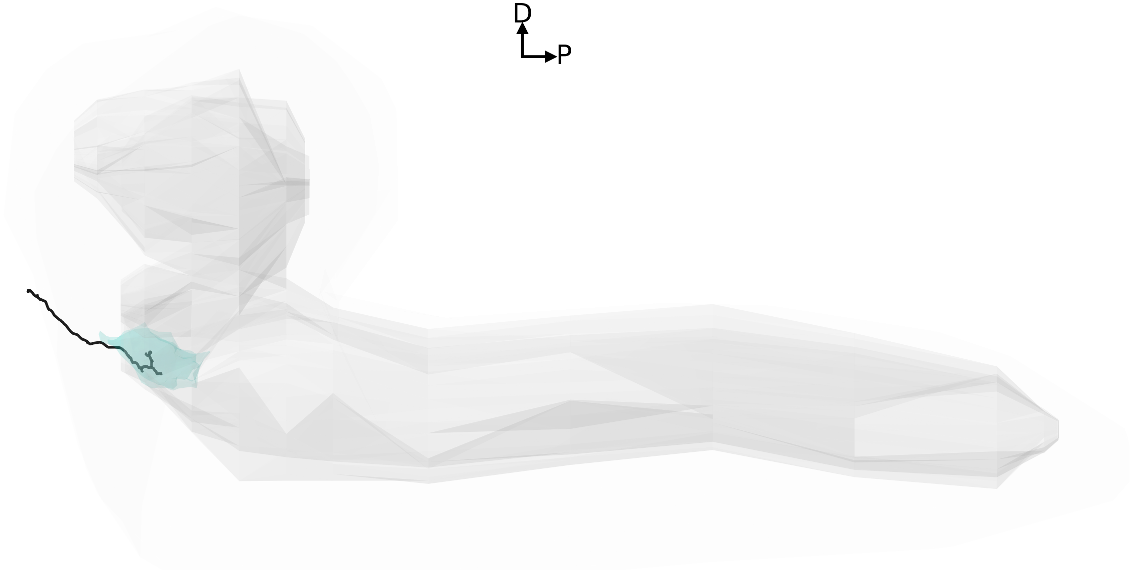

ID	*name*	SCACa	SCAVa	SCAVp	SCACal	SCACp	SCACpl	SCVM	IPCs	DMS	DH44	Se0ens	Se0ph	PMN LR	MN motor neurons	PaN motor neurons	olfactory PNs	gustatory PNs	multiglomerular PNs	unknown PNs	thermo PNs	visual PNs
15748726	AN-R-Sens-B1-AVa-43	0	5	0	0	0	0	0	0	0	0	0	0	32	0	0	0	0	0	0	0	0

ID: 945309
name: AN-R-Sens-B1-AVa-44

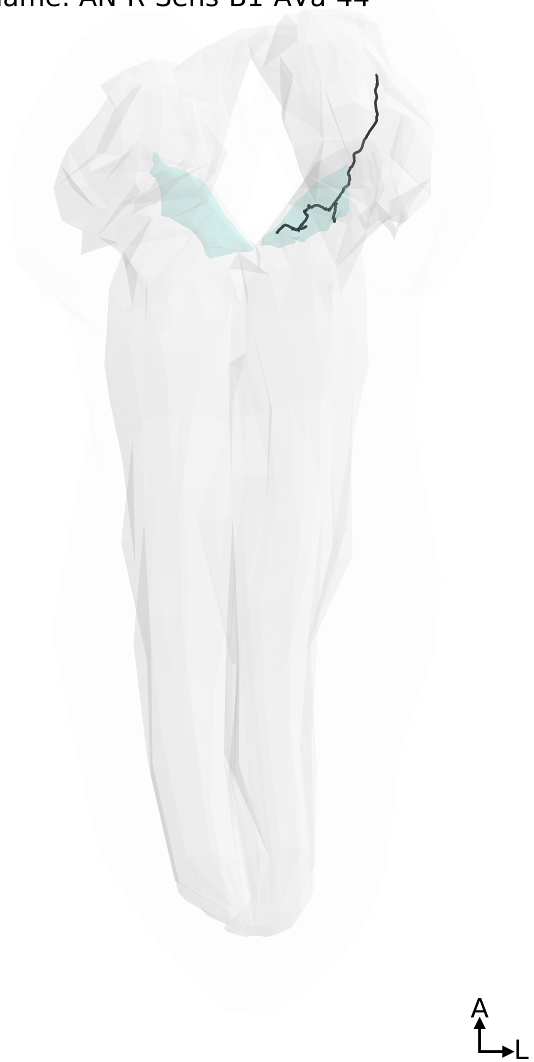

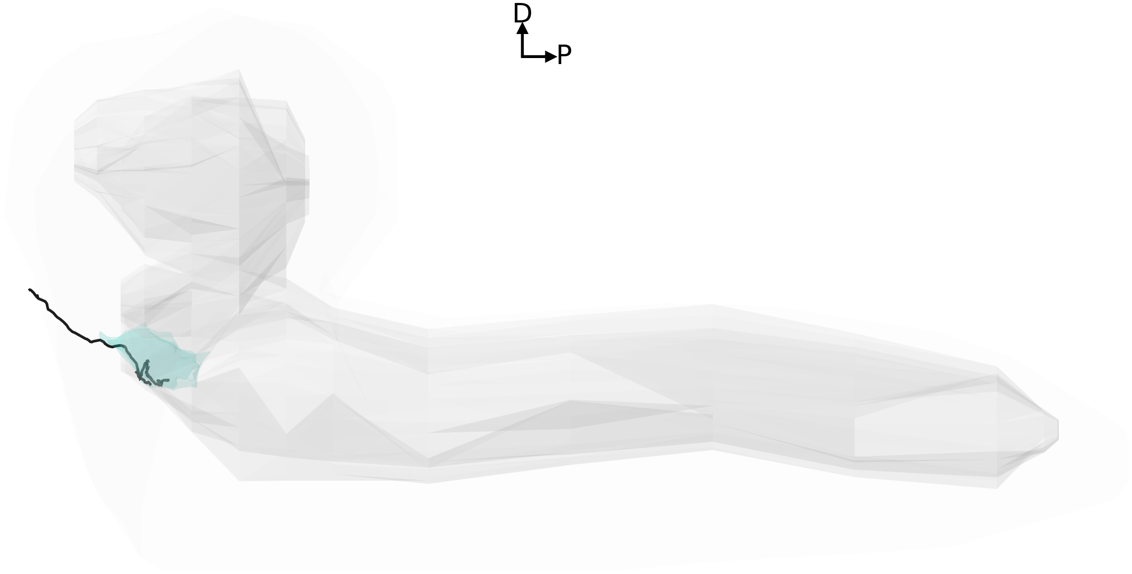

ID	name	SCACa	SCAVa	SCAVp	SCACal	SCACp	SCACpl	SCVM	IPCs	DMS	DH44	Se0ens	Se0ph	PMN LR	MN motor neurons	PaN motor neurons	olfactory PNs	gustatory PNs	multiglomerular PNs	unknown PNs	thermo PNs	visual PNs
945309	AN-R-Sens-B1-AVa-44	0	4	0	0	0	0	0	0	0	0	0	1	12	0	0	0	0	0	0	0	0

ID: 18231999
name: AN-R-Sens-B1-AVa-45

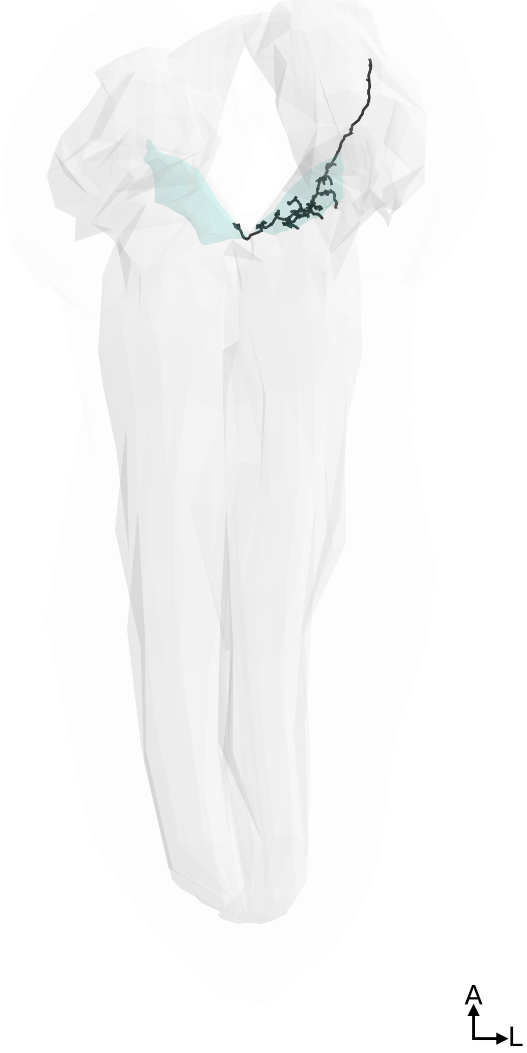

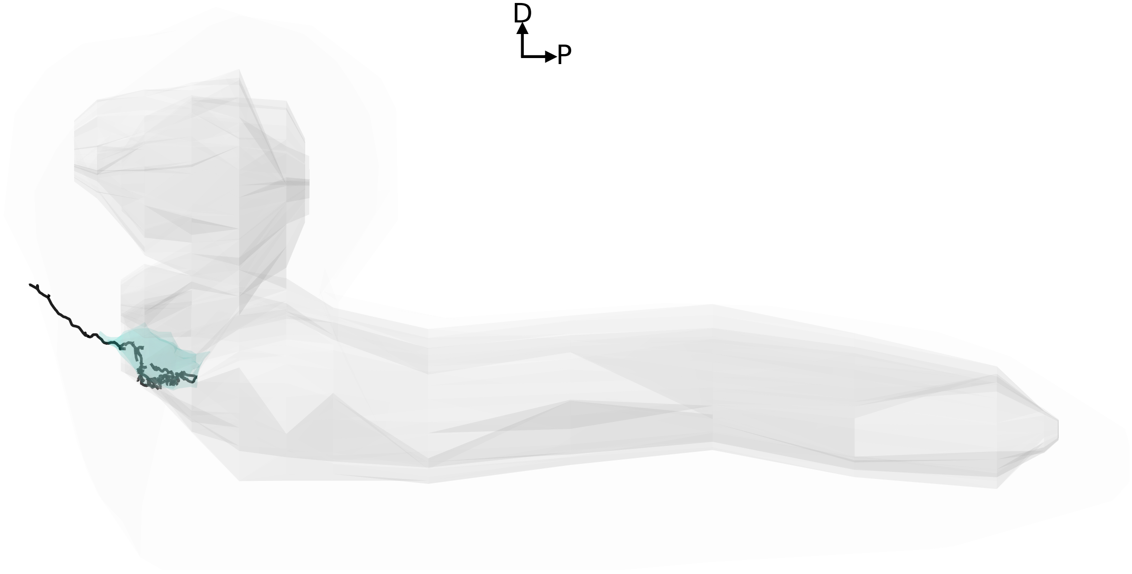

ID	*name*	SCACa	SCAVa	SCAVp	SCACal	SCACp	SCACpl	SCVM	IPCs	DMS	DH44	Se0ens	Se0ph	PMN LR	MN motor neurons	PaN motor neurons	olfactory PNs	gustatory PNs	multiglomerular PNs	unknown PNs	thermo PNs	visual PNs
18231999	AN-R-Sens-B1-AVa-45	0	10	0	0	0	0	0	0	0	0	0	0	38	0	0	0	0	0	0	0	0

ID: 15532105
name: AN-R-Sens-B2-AVa-24

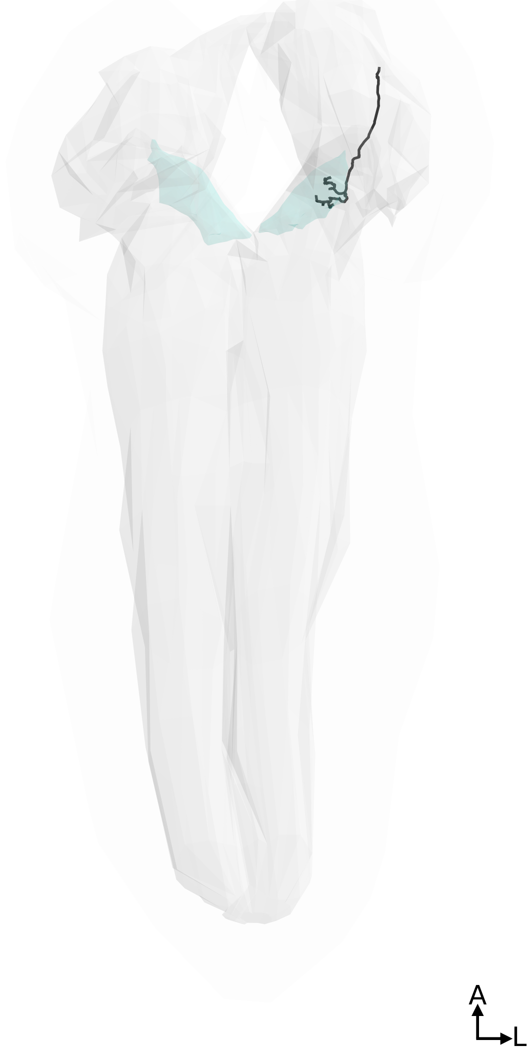

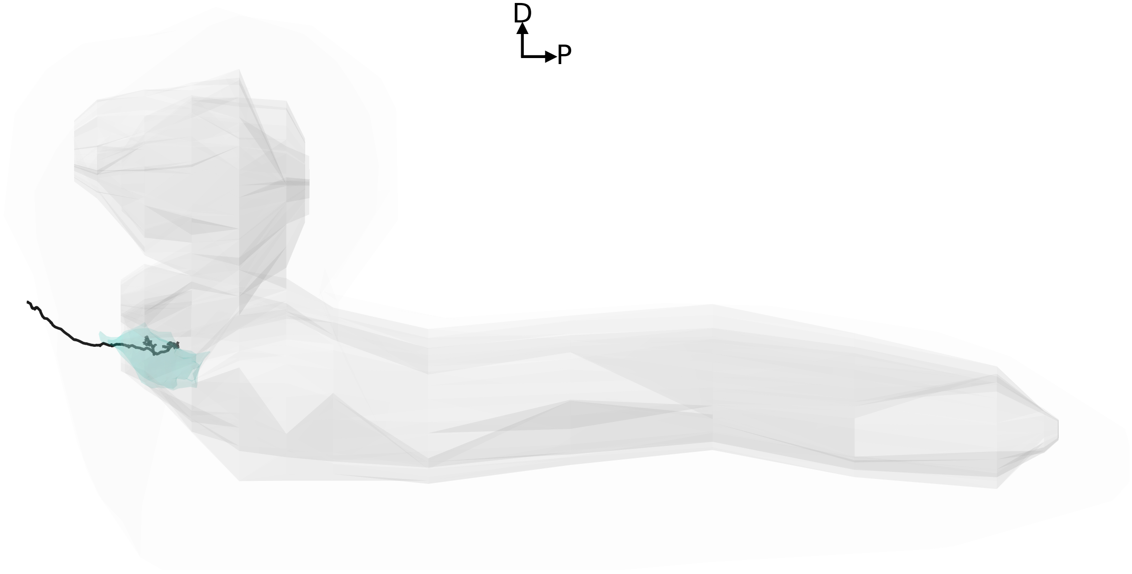

ID	name	SCACa	SCAVa	SCAVp	SCACal	SCACp	SCACpl	SCVM	IPCs	DMS	DH44	Se0ens	Se0ph	PMN LR	MN motor neurons	PaN motor neurons	olfactory PNs	gustatory PNs	multiglomerular PNs	unknown PNs	thermo PNs	visual PNs
15532105	AN-R-Sens-B2-AVa-24	0	1	0	0	1	0	0	0	0	0	0	1	0	0	0	0	0	0	0	0	0

ID: 3192952
name: AN-R-Sens-B2-AVa-25

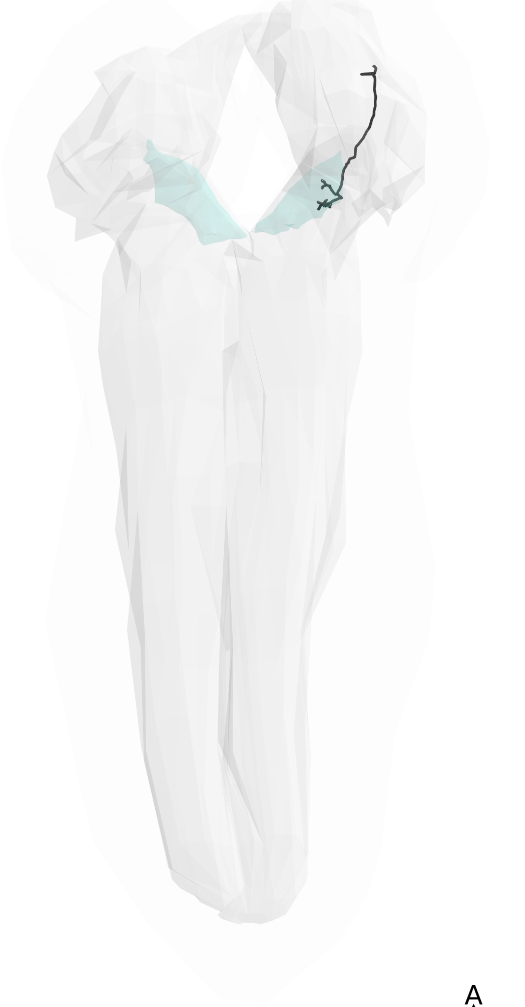

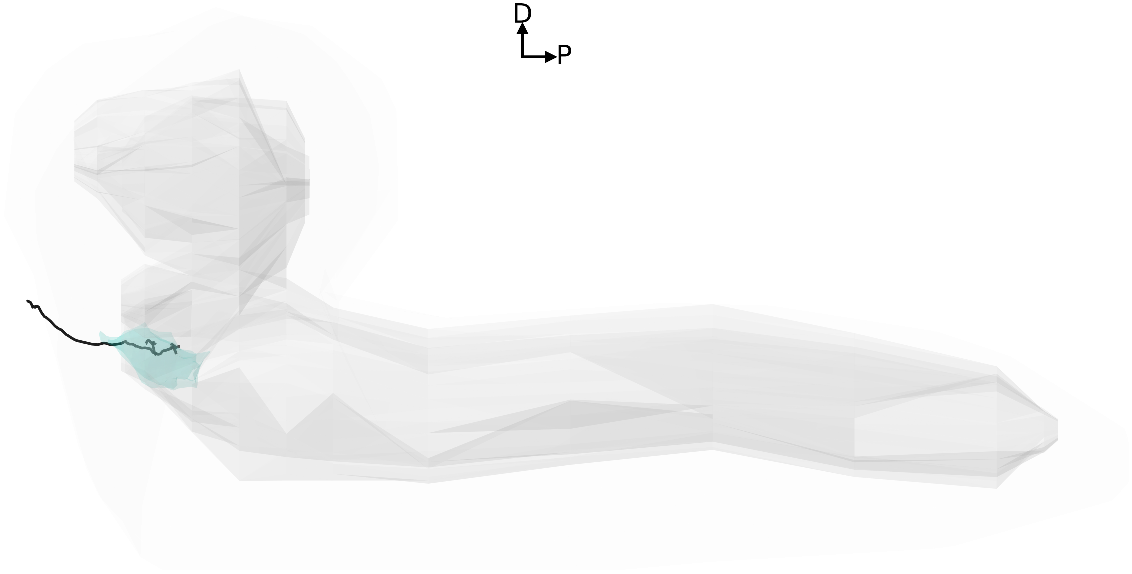

ID	*name*	SCACa	SCAVa	SCAVp	SCACal	SCACp	SCACpl	SCVM	IPCs	DMS	DH44	Se0ens	Se0ph	PMN LR	MN motor neurons	PaN motor neurons	olfactory PNs	gustatory PNs	multiglomerular PNs	unknown PNs	thermo PNs	visual PNs
3192952	AN-R-Sens-B2-AVa-25	0	0	0	0	1	0	0	0	0	0	0	0	0	0	0	0	0	0	0	0	0

ID: 1347886
name: AN-L-Sens-B2-AVp-01

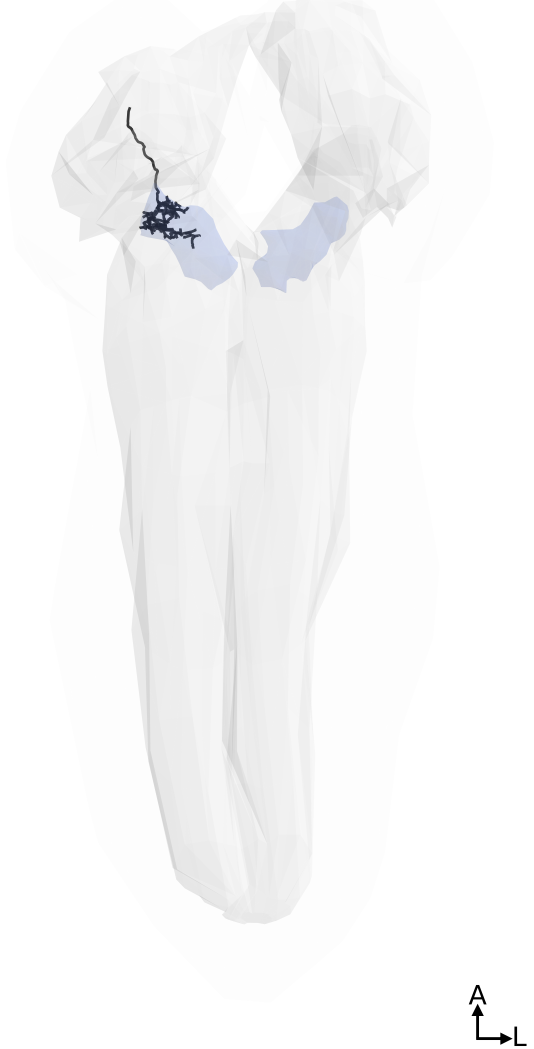

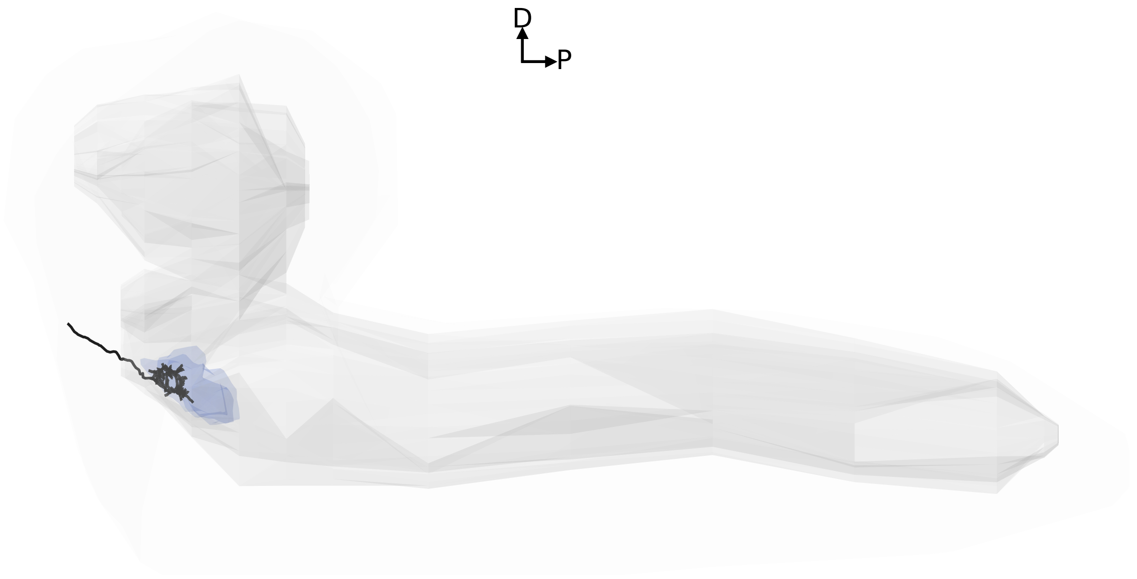

ID	*name*	SCACa	SCAVa	SCAVp	SCACal	SCACp	SCACpl	SCVM	IPCs	DMS	DH44	Se0ens	Se0ph	PMN LR	MN motor neurons	PaN motor neurons	olfactory PNs	gustatory PNs	multiglomerular PNs	unknown PNs	thermo PNs	visual PNs
1347886	AN-L-Sens-B2-AVp-01	0	1	0	0	0	0	0	0	0	0	0	7	6	1	0	0	0	0	0	0	0

ID: 15998899
name: AN-L-Sens-B2-AVp-02

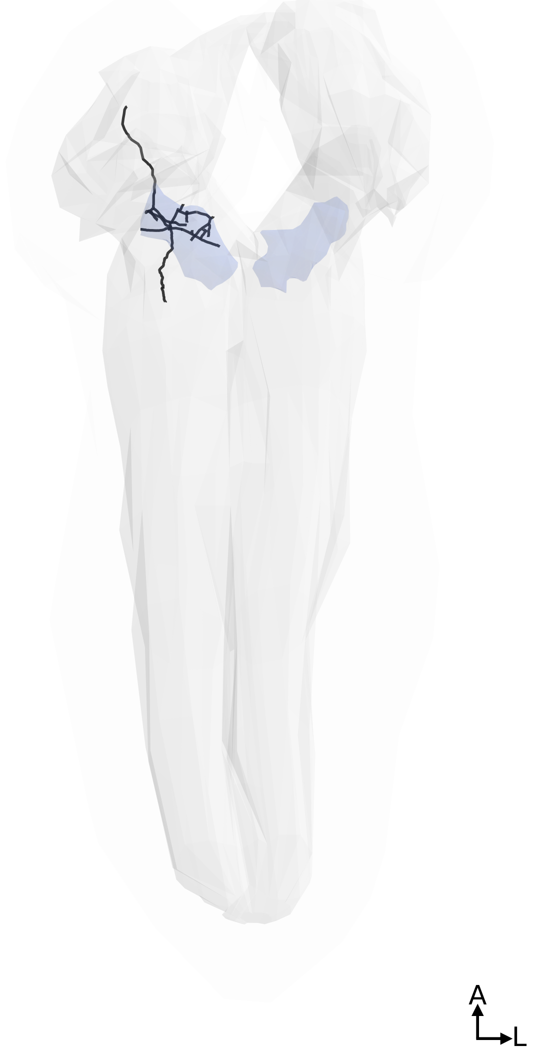

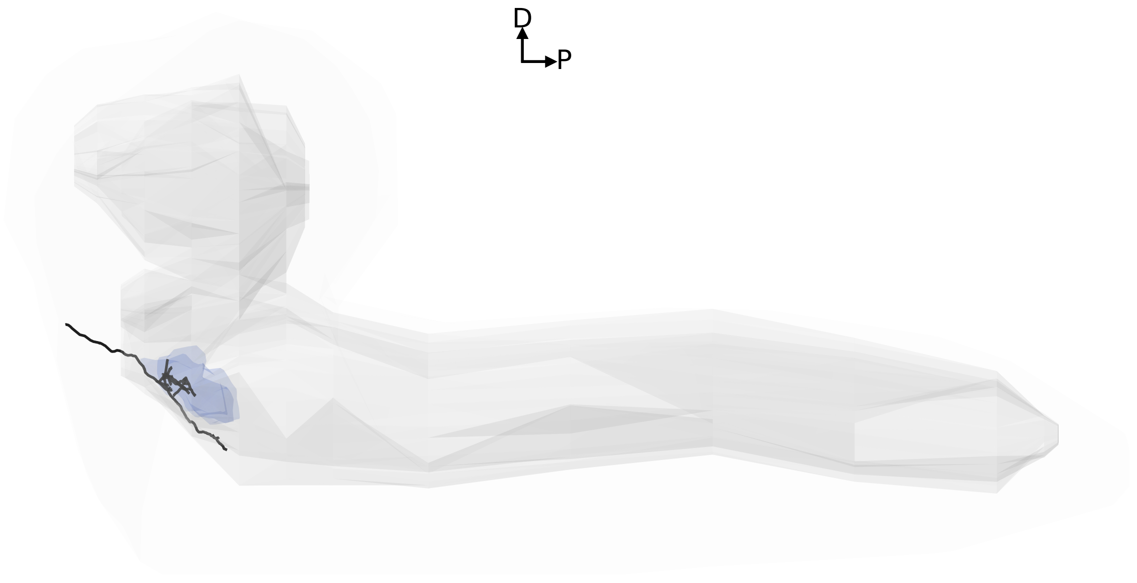

ID	*name*	SCACa	SCAVa	SCAVp	SCACal	SCACp	SCACpl	SCVM	IPCs	DMS	DH44	Se0ens	Se0ph	PMN LR	MN motor neurons	PaN motor neurons	olfactory PNs	gustatory PNs	multiglomerular PNs	unknown PNs	thermo PNs	visual PNs
15998899	AN-L-Sens-B2-AVp-02	0	2	0	0	0	0	1	0	0	0	0	0	4	0	0	0	0	0	0	0	0

ID: 15996160
name: AN-L-Sens-B2-AVp-03

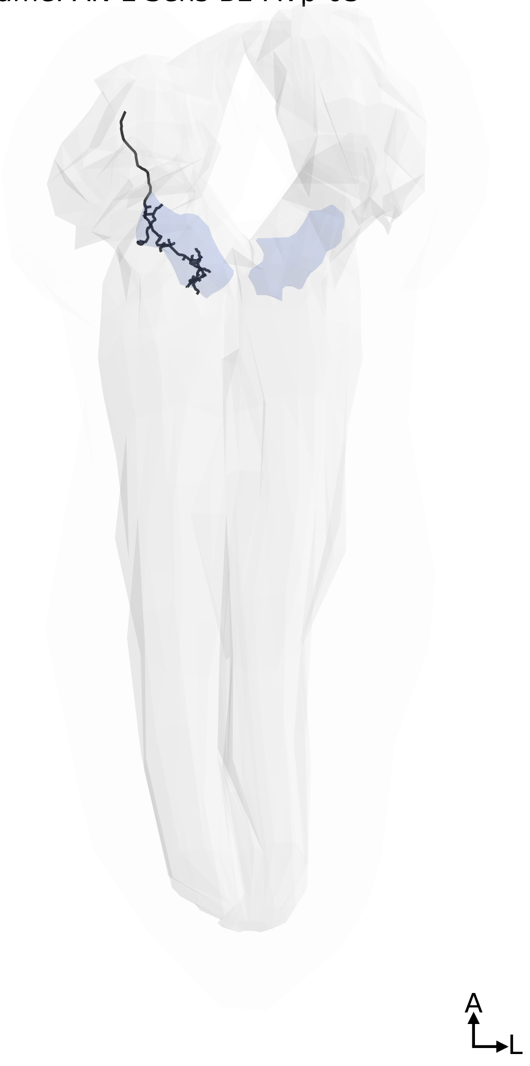

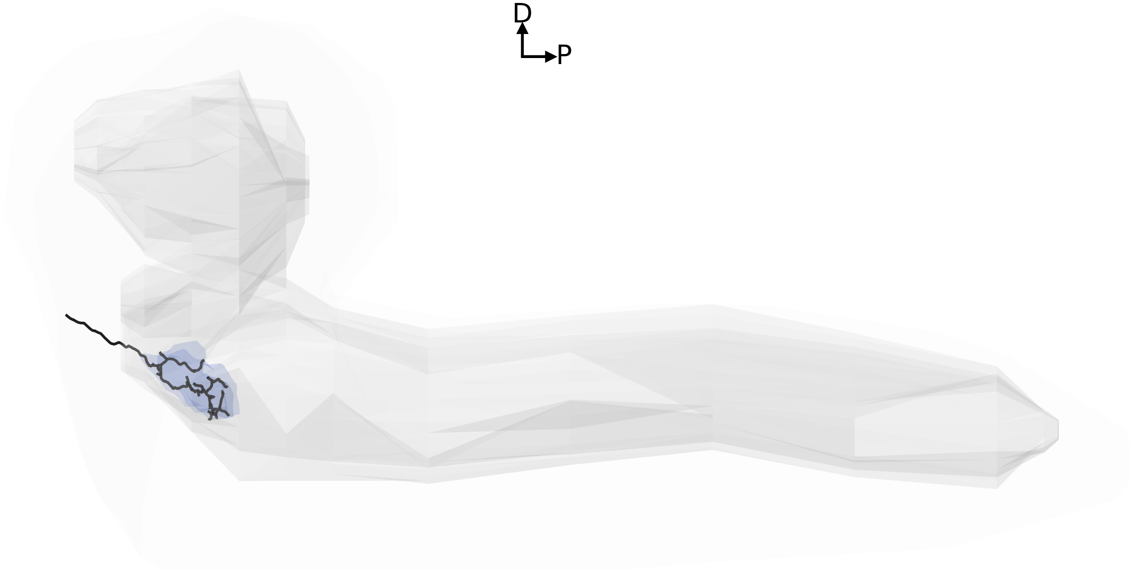

ID	*name*	SCACa	SCAVa	SCAVp	SCACal	SCACp	SCACpl	SCVM	IPCs	DMS	DH44	Se0ens	Se0ph	PMN LR	MN motor neurons	PaN motor neurons	olfactory PNs	gustatory PNs	multiglomerular PNs	unknown PNs	thermo PNs	visual PNs
15996160	AN-L-Sens-B2-AVp-03	0	0	1	0	0	0	0	0	0	0	0	10	2	0	0	0	0	0	0	0	0

ID: 7160160
name: AN-R-Sens-B2-AVp-01

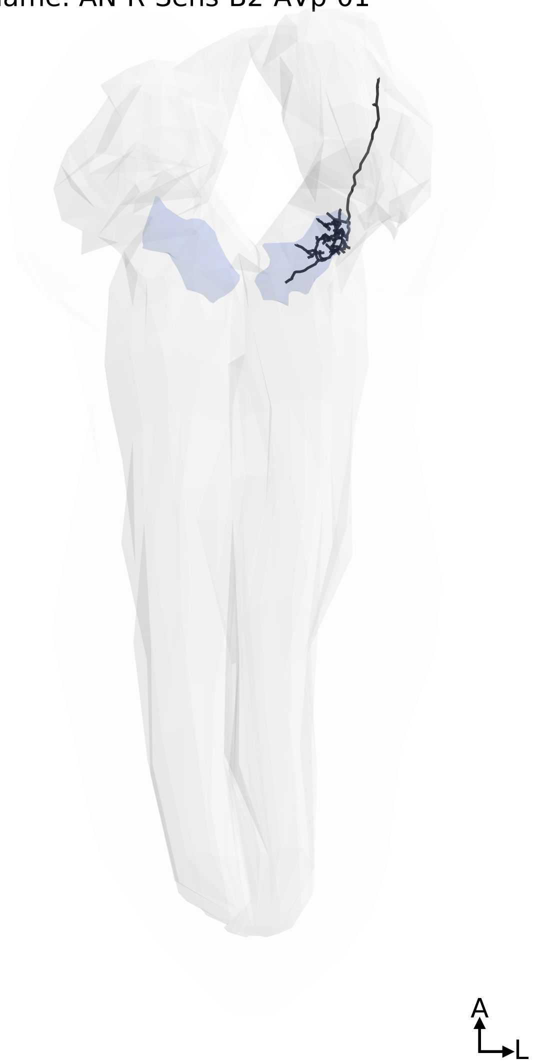

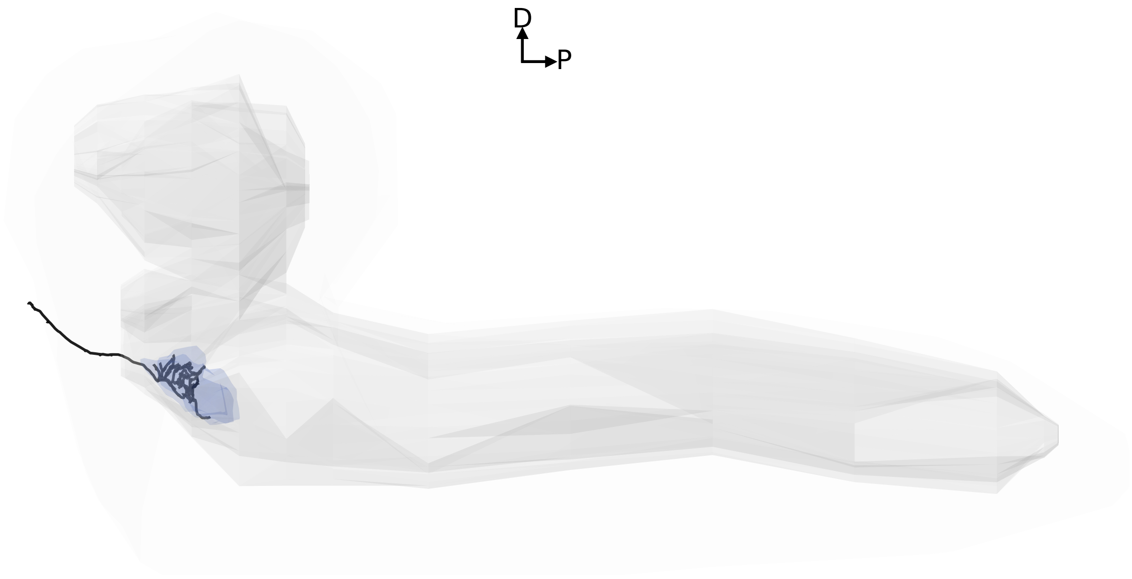

ID	name	SCACa	SCAVa	SCAVp	SCACal	SCACp	SCACpl	SCVM	IPCs	DMS	DH44	Se0ens	Se0ph	PMN LR	MN motor neurons	PaN motor neurons	olfactory PNs	gustatory PNs	multiglomerular PNs	unknown PNs	thermo PNs	visual PNs
7160160	AN-R-Sens-B2-AVp-01	0	0	1	0	0	0	0	0	0	0	0	7	5	0	0	0	0	0	0	0	0

ID: 15533421
name: AN-R-Sens-B2-AVp-02

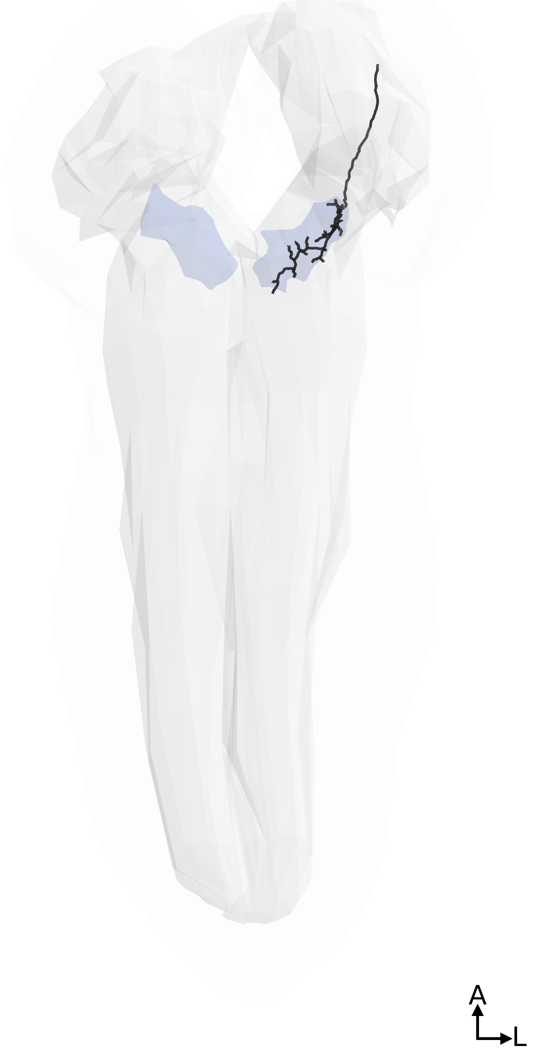

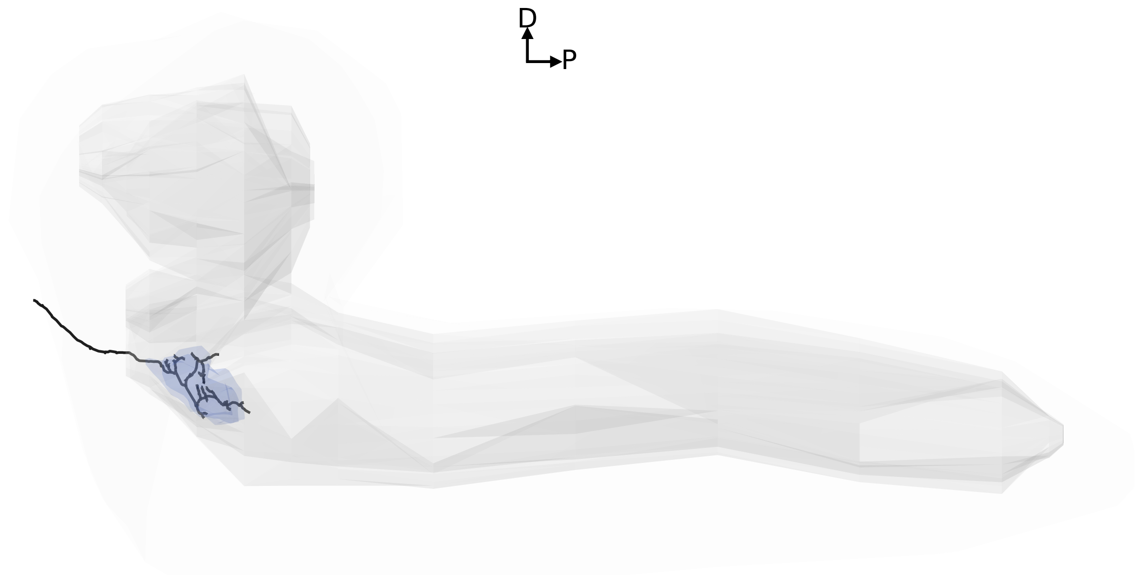

ID	*name*	SCACa	SCAVa	SCAVp	SCACal	SCACp	SCACpl	SCVM	IPCs	DMS	DH44	Se0ens	Se0ph	PMN LR	MN motor neurons	PaN motor neurons	olfactory PNs	gustatory PNs	multiglomerular PNs	unknown PNs	thermo PNs	visual PNs
15533421	AN-R-Sens-B2-AVp-02	0	0	0	0	0	0	0	0	0	0	0	9	2	0	0	0	0	0	0	0	0

ID: 15605657
name: AN-R-Sens-B2-AVp-03

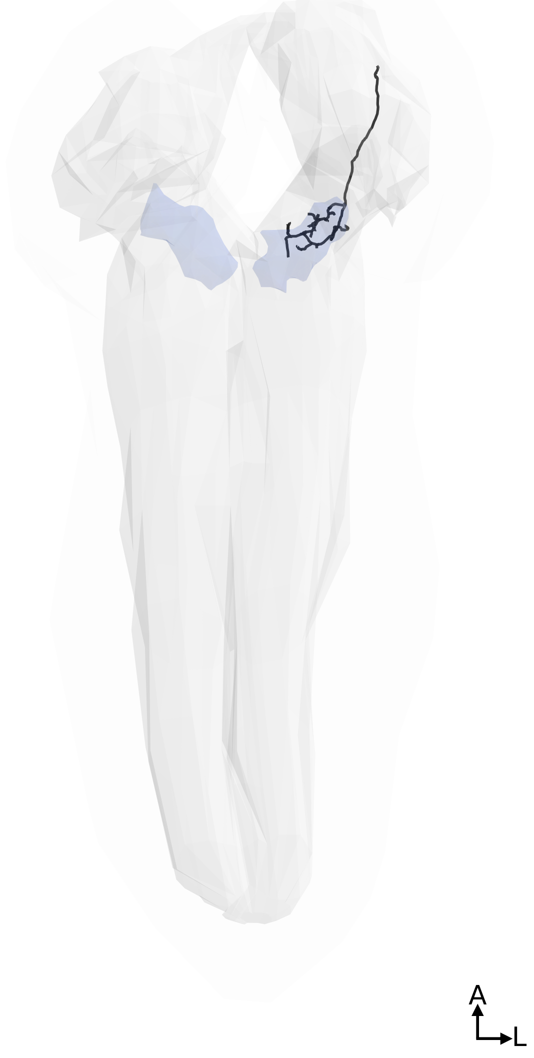

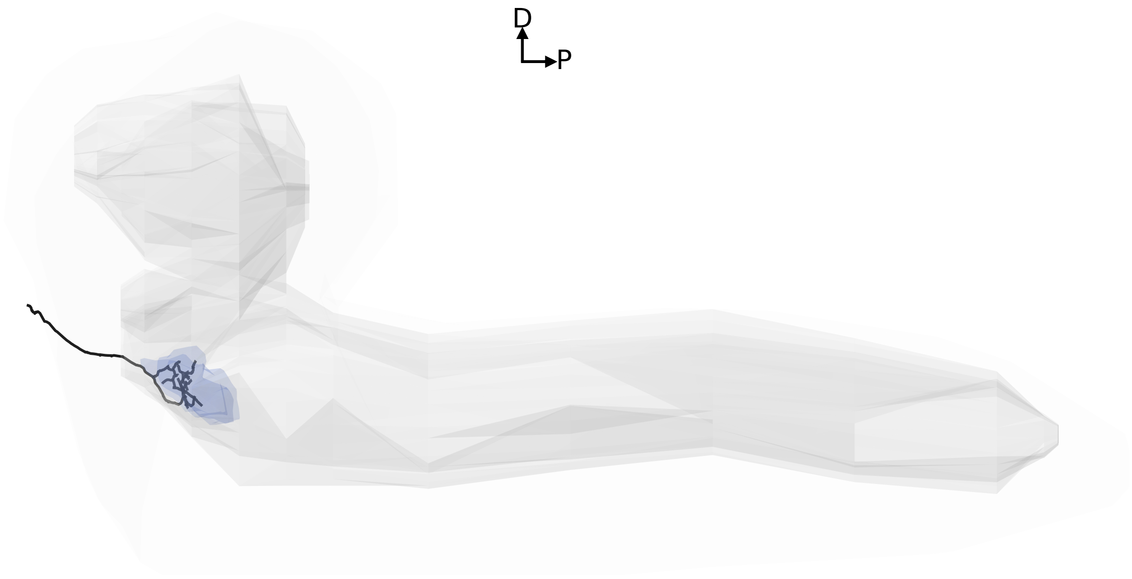

ID	*name*	SCACa	SCAVa	SCAVp	SCACal	SCACp	SCACpl	SCVM	IPCs	DMS	DH44	Se0ens	Se0ph	PMN LR	MN motor neurons	PaN motor neurons	olfactory PNs	gustatory PNs	multiglomerular PNs	unknown PNs	thermo PNs	visual PNs
15605657	AN-R-Sens-B2-AVp-03	0	0	1	0	0	0	0	0	0	0	0	0	1	0	0	0	0	0	0	0	0

ID: 15505837
name: AN-R-Sens-B2-AVp-06

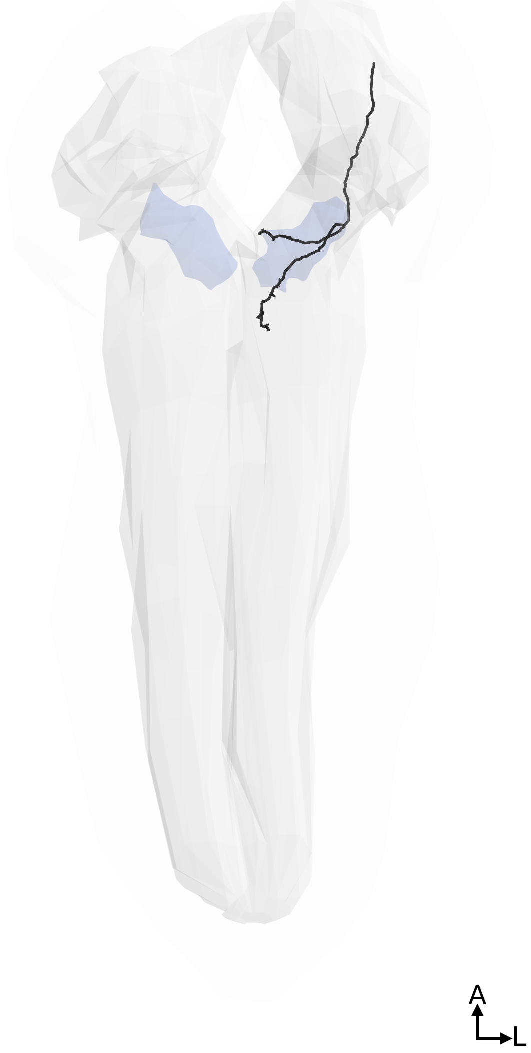

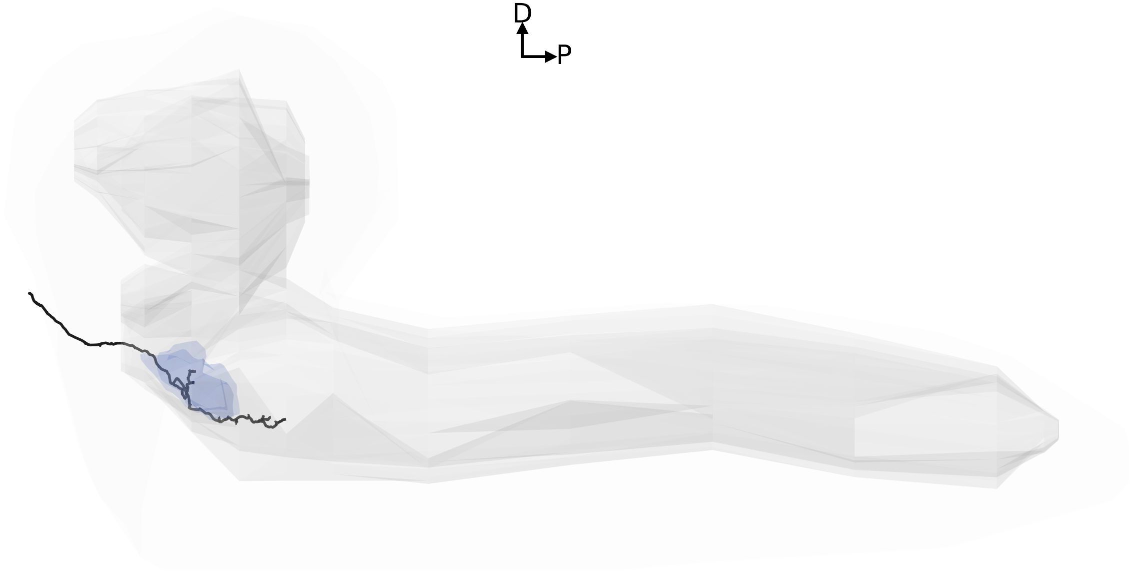

ID	*name*	SCACa	SCAVa	SCAVp	SCACal	SCACp	SCACpl	SCVM	IPCs	DMS	DH44	Se0ens	Se0ph	PMN LR	MN motor neurons	PaN motor neurons	olfactory PNs	gustatory PNs	multiglomerular PNs	unknown PNs	thermo PNs	visual PNs
15505837	AN-R-Sens-B2-AVp-06	0	0	1	0	0	0	2	0	0	0	0	1	4	0	0	0	0	0	0	0	0

ID: 9808022
name: MN-L-Sens-B2-AVp-01

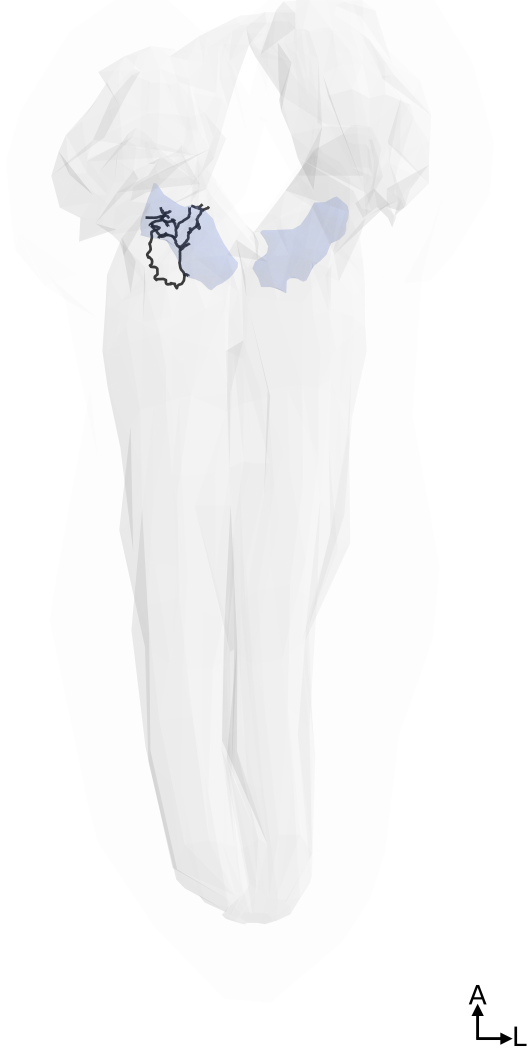

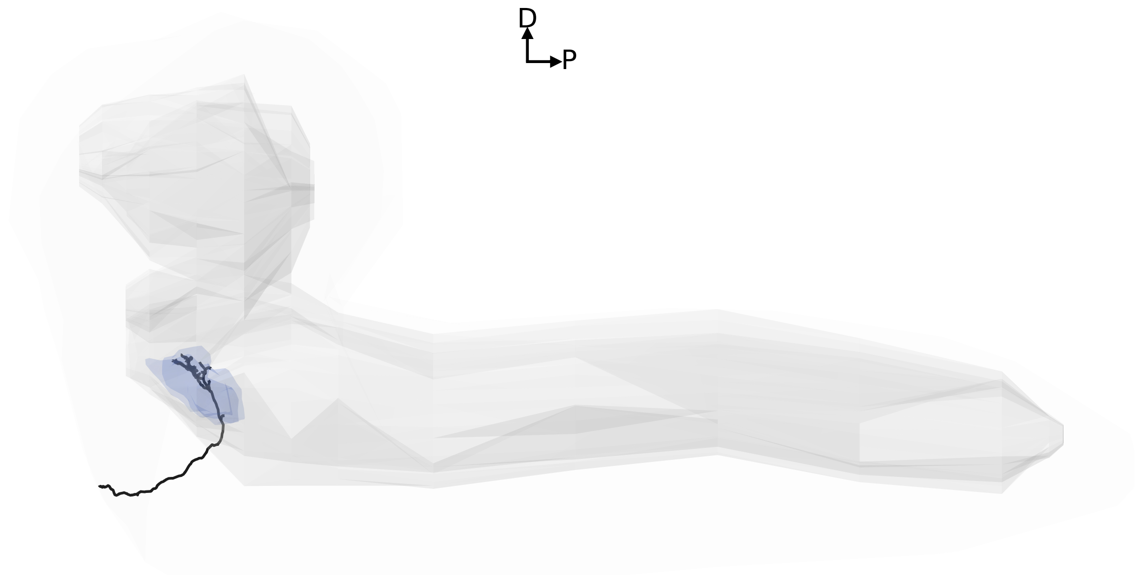

ID	*name*	SCACa	SCAVa	SCAVp	SCACal	SCACp	SCACpl	SCVM	IPCs	DMS	DH44	Se0ens	Se0ph	PMN LR	MN motor neurons	PaN motor neurons	olfactory PNs	gustatory PNs	multiglomerular PNs	unknown PNs	thermo PNs	visual PNs
9808022	MN-L-Sens-B2-AVp-01	0	0	1	0	0	0	0	0	0	0	17	4	0	0	0	0	0	0	0	0	0

ID: 15717426
name: MN-L-Sens-B2-AVp-02

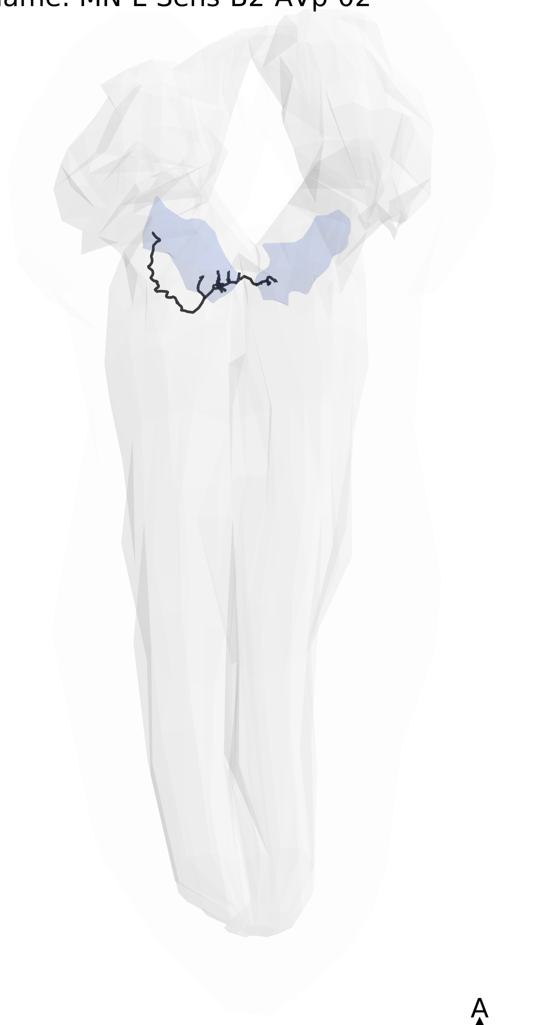

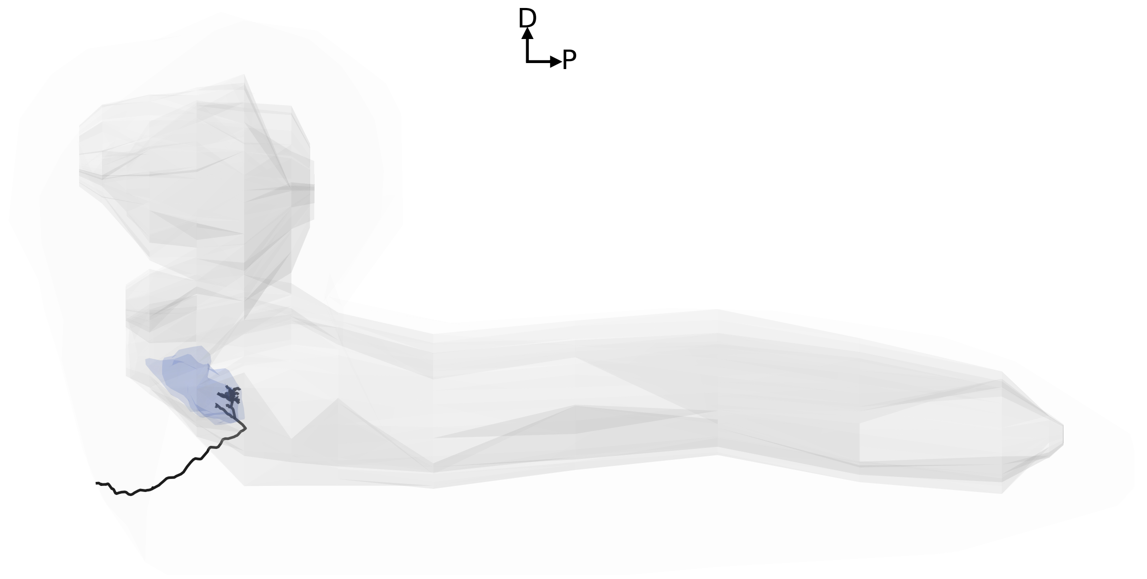

ID	*name*	SCACa	SCAVa	SCAVp	SCACal	SCACp	SCACpl	SCVM	IPCs	DMS	DH44	Se0ens	Se0ph	PMN LR	MN motor neurons	PaN motor neurons	olfactory PNs	gustatory PNs	multiglomerular PNs	unknown PNs	thermo PNs	visual PNs
15717426	MN-L-Sens-B2-AVp-02	0	0	3	0	0	1	0	0	0	0	0	0	0	0	0	0	0	0	0	0	0

ID: 15724385
name: MN-L-Sens-B3-AVp-01

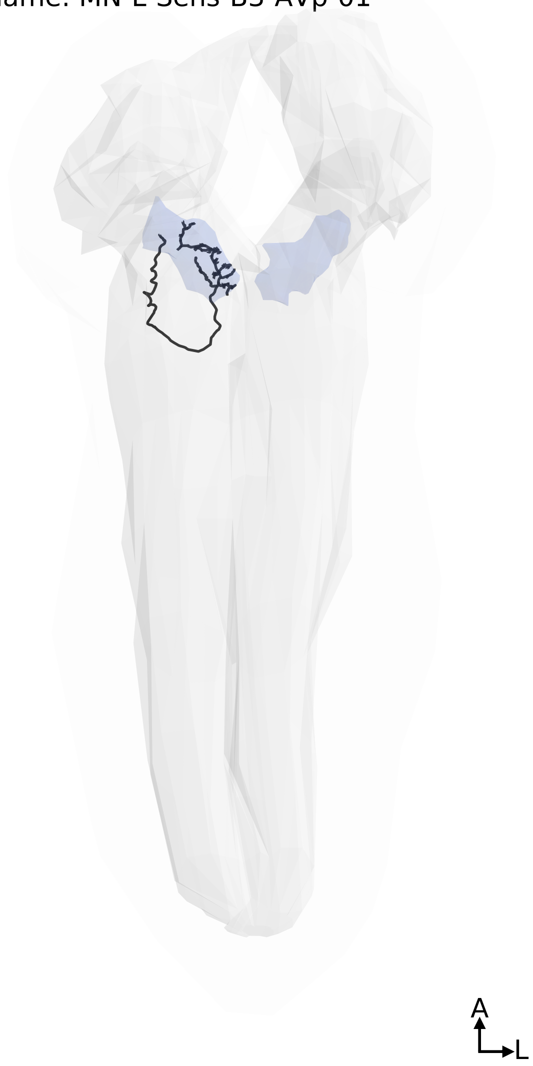

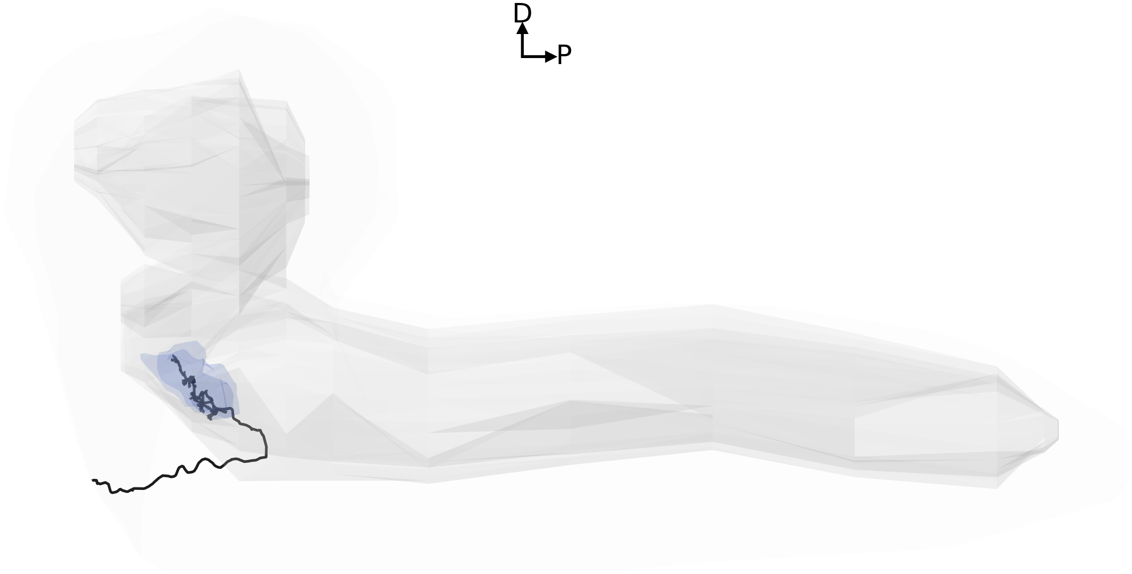

ID	*name*	SCACa	SCAVa	SCAVp	SCACal	SCACp	SCACpl	SCVM	IPCs	DMS	DH44	Se0ens	Se0ph	PMN LR	MN motor neurons	PaN motor neurons	olfactory PNs	gustatory PNs	multiglomerular PNs	unknown PNs	thermo PNs	visual PNs
15724385	MN-L-Sens-B3-AVp-01	0	0	3	0	0	0	0	0	0	0	0	9	0	0	0	0	0	0	0	0	0

ID: 15723479
name: MN-L-Sens-B3-AVp-02

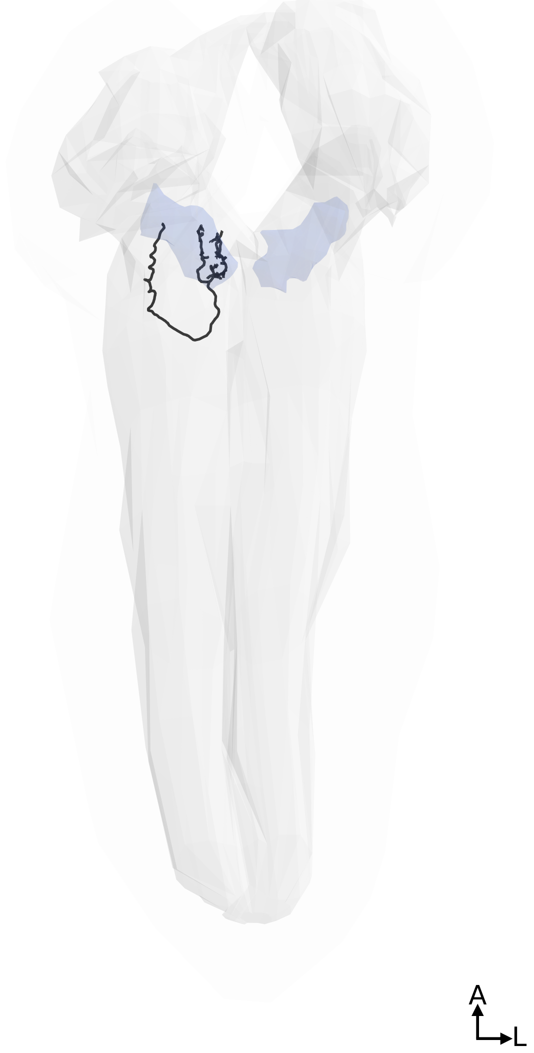

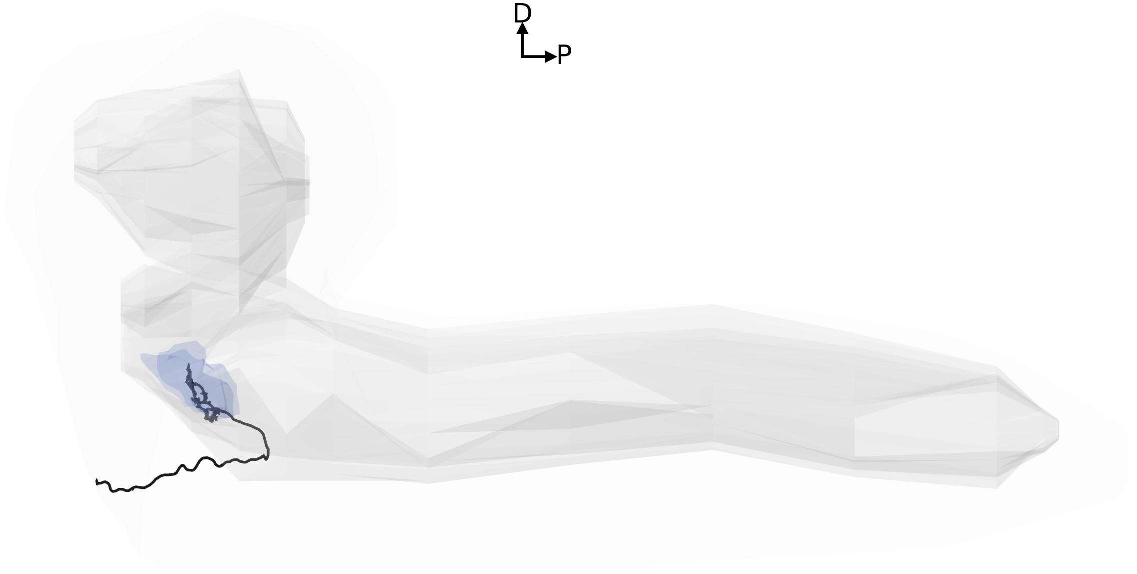

ID	name	SCACa	SCAVa	SCAVp	SCACal	SCACp	SCACpl	SCVM	IPCs	DMS	DH44	Se0ens	Se0ph	PMN LR	MN motor neurons	PaN motor neurons	olfactory PNs	gustatory PNs	multiglomerular PNs	unknown PNs	thermo PNs	visual PNs
15723479	MN-L-Sens-B3-AVp-02	0	0	3	0	0	0	0	0	0	0	0	3	0	0	0	0	0	0	0	0	0

ID: 3032520
name: MN-L-Sens-B3-AVp-03

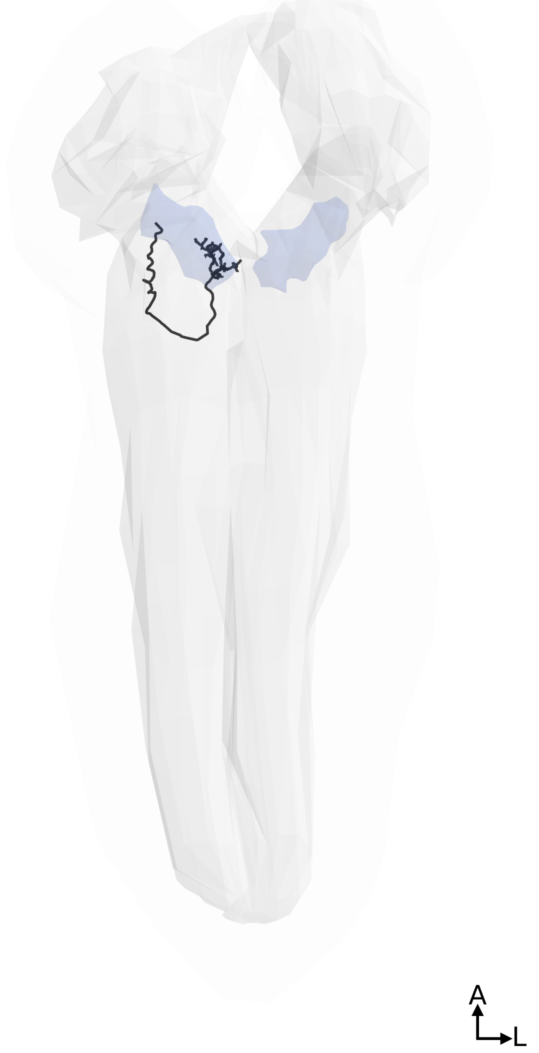

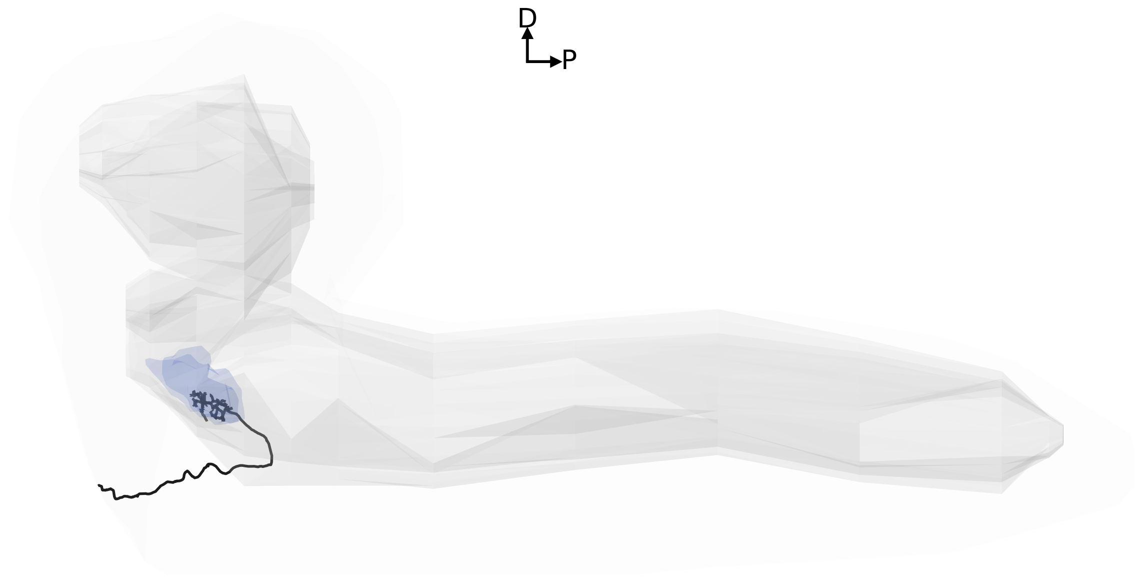

ID	*name*	SCACa	SCAVa	SCAVp	SCACal	SCACp	SCACpl	SCVM	IPCs	DMS	DH44	Se0ens	Se0ph	PMN LR	MN motor neurons	PaN motor neurons	olfactory PNs	gustatory PNs	multiglomerular PNs	unknown PNs	thermo PNs	visual PNs
3032520	MN-L-Sens-B3-AVp-03	0	0	2	0	0	0	0	0	0	0	0	3	1	0	0	0	0	0	0	0	0

ID: 15658013
name: MN-R-Sens-B2-AVp-01

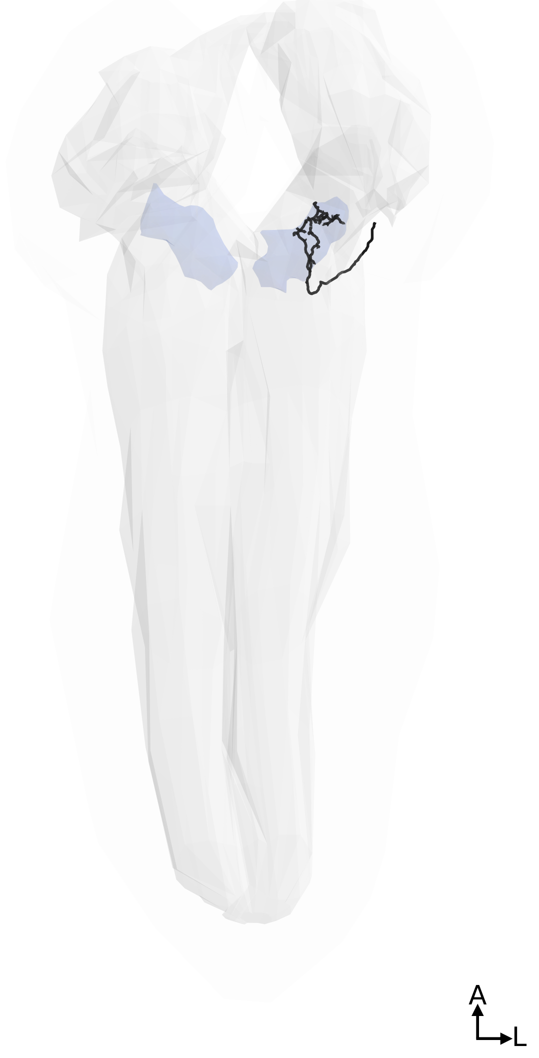

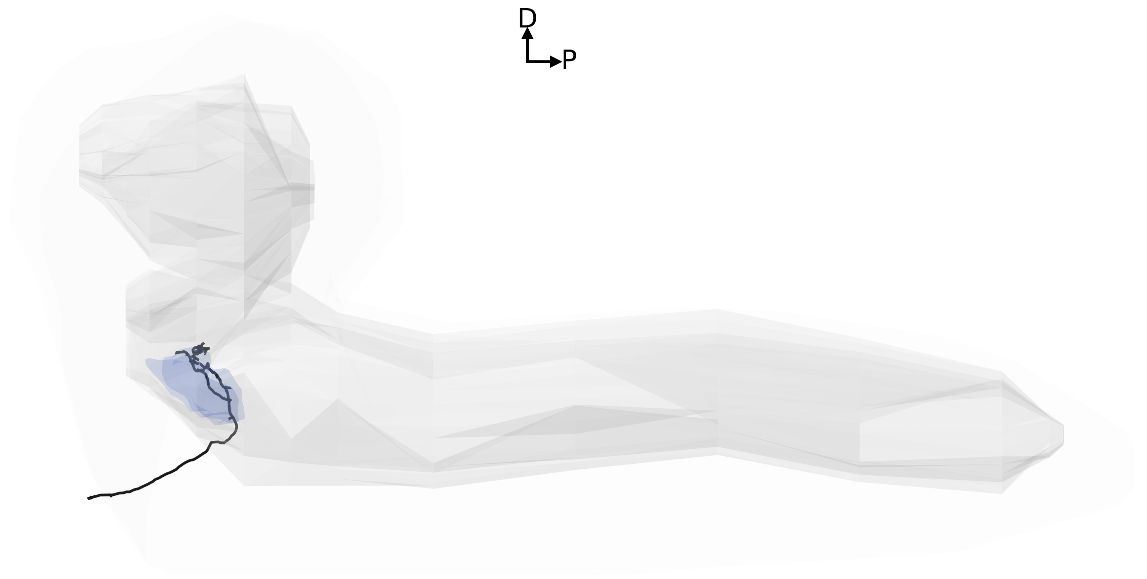

ID	*name*	SCACa	SCAVa	SCAVp	SCACal	SCACp	SCACpl	SCVM	IPCs	DMS	DH44	Se0ens	Se0ph	PMN LR	MN motor neurons	PaN motor neurons	olfactory PNs	gustatory PNs	multiglomerular PNs	unknown PNs	thermo PNs	visual PNs
15658013	MN-R-Sens-B2-AVp-01	0	0	0	0	0	0	0	0	0	0	3	1	0	0	0	0	0	0	0	0	0

ID: 8810739
name: MN-R-Sens-B2-AVp-02

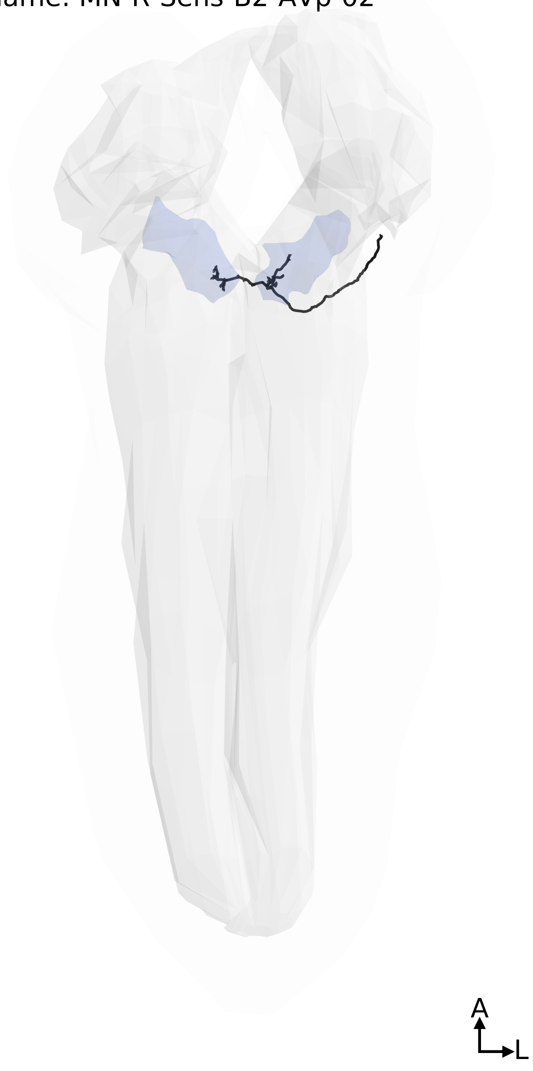

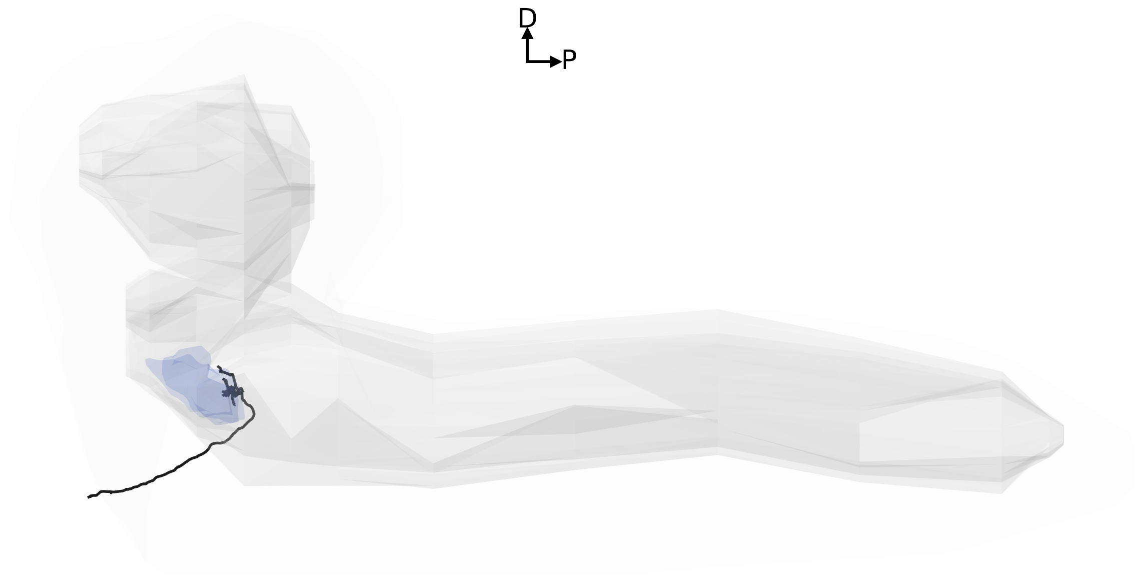

ID	*name*	SCACa	SCAVa	SCAVp	SCACal	SCACp	SCACpl	SCVM	IPCs	DMS	DH44	Se0ens	Se0ph	PMN LR	MN motor neurons	PaN motor neurons	olfactory PNs	gustatory PNs	multiglomerular PNs	unknown PNs	thermo PNs	visual PNs
8810739	MN-R-Sens-B2-AVp-02	0	0	2	0	0	0	0	0	0	0	0	0	0	0	0	0	0	0	0	0	0

ID: 15638968
name: MN-R-Sens-B3-AVp-01

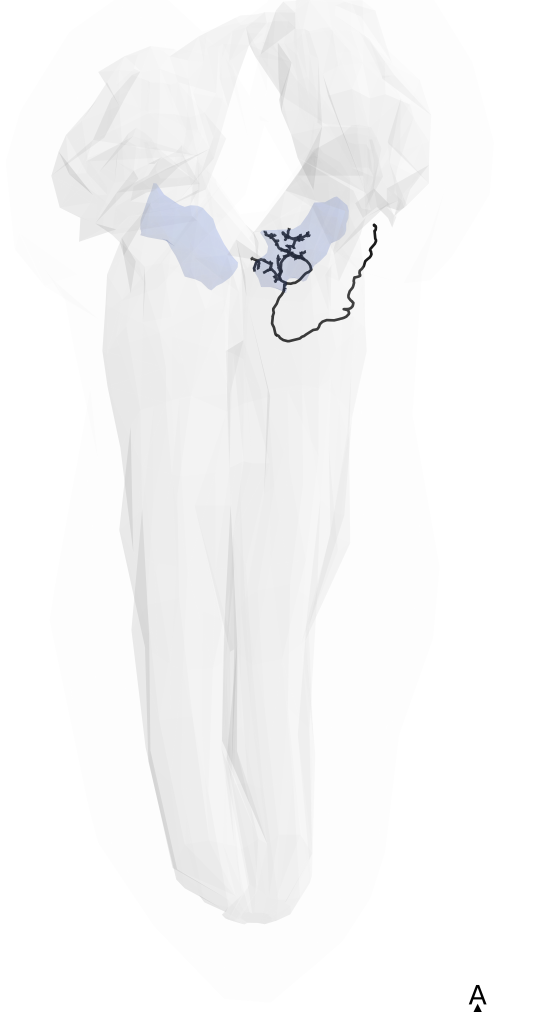

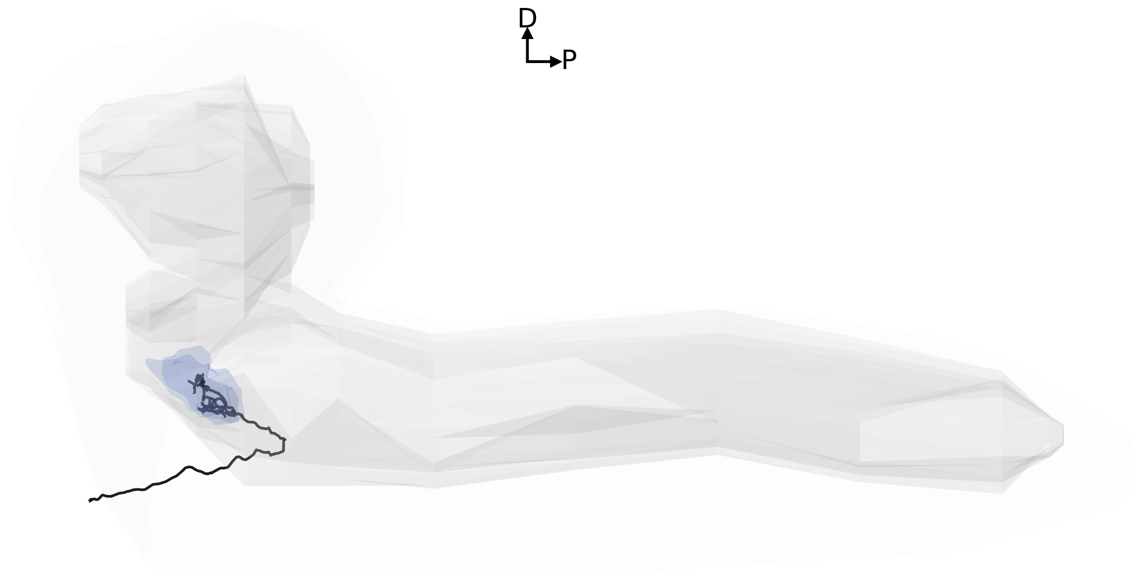

ID	name	SCACa	SCAVa	SCAVp	SCACal	SCACp	SCACpl	SCVM	IPCs	DMS	DH44	Se0ens	Se0ph	PMN LR	MN motor neurons	PaN motor neurons	olfactory PNs	gustatory PNs	multiglomerular PNs	unknown PNs	thermo PNs	visual PNs
15638968	MN-R-Sens-B3-AVp-01	0	0	2	0	0	0	0	0	0	0	0	4	1	0	0	0	0	0	0	0	0

ID: 15640548
name: MN-R-Sens-B3-AVp-02

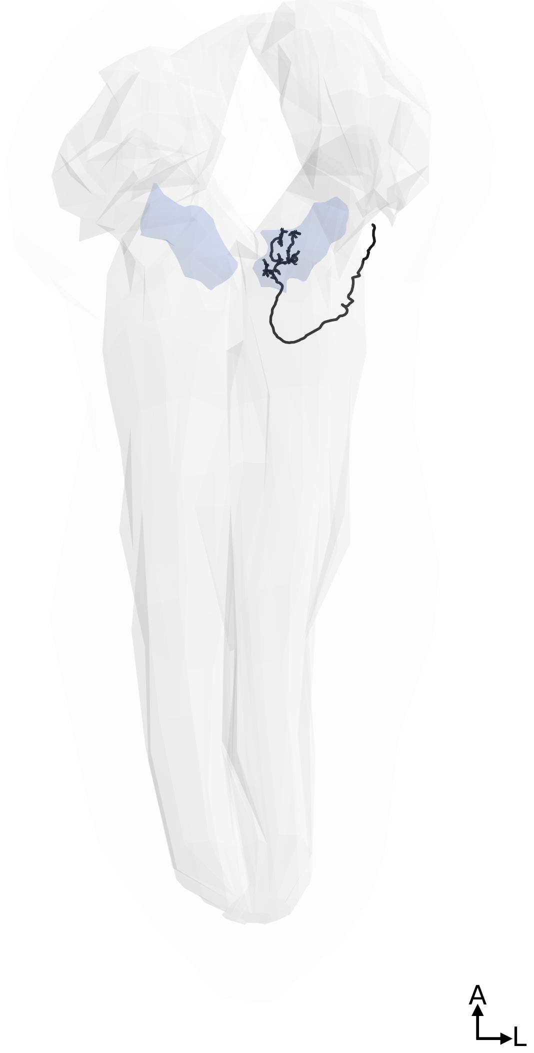

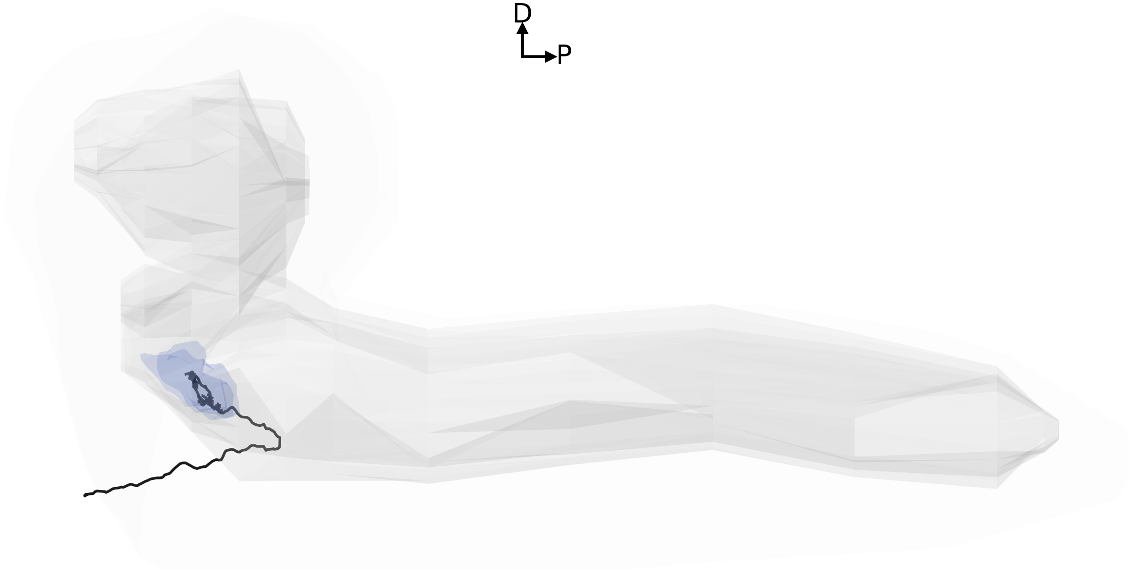

ID	*name*	SCACa	SCAVa	SCAVp	SCACal	SCACp	SCACpl	SCVM	IPCs	DMS	DH44	Se0ens	Se0ph	PMN LR	MN motor neurons	PaN motor neurons	olfactory PNs	gustatory PNs	multiglomerular PNs	unknown PNs	thermo PNs	visual PNs
15640548	MN-R-Sens-B3-AVp-02	0	0	1	0	0	0	0	0	0	0	0	4	0	0	0	0	0	0	0	0	0

ID: 15640425
name: MN-R-Sens-B3-AVp-03

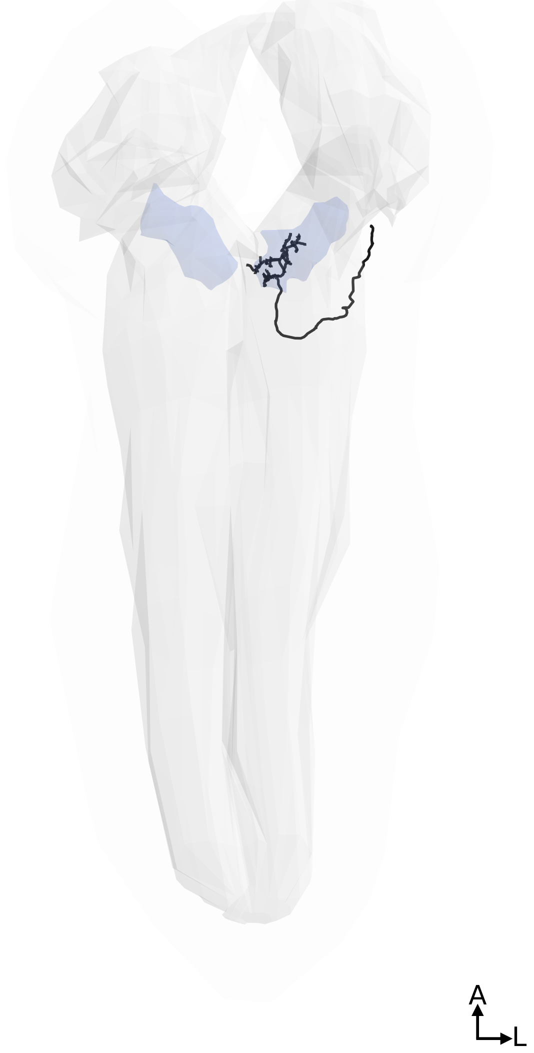

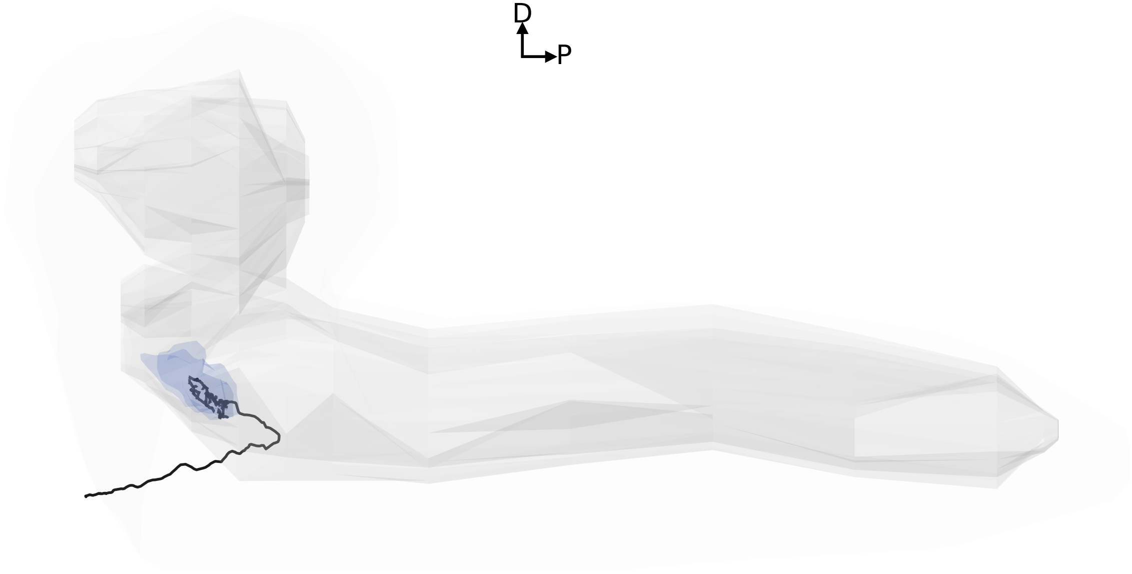

ID	name	SCACa	SCAVa	SCAVp	SCACal	SCACp	SCACpl	SCVM	IPCs	DMS	DH44	Se0ens	Se0ph	PMN LR	MN motor neurons	PaN motor neurons	olfactory PNs	gustatory PNs	multiglomerular PNs	unknown PNs	thermo PNs	visual PNs
15640425	MN-R-Sens-B3-AVp-03	0	0	2	0	0	0	0	0	0	0	0	11	2	0	0	0	0	0	0	0	0

ID: 3608397
name: AN-L-Sens-B3-ACal-01

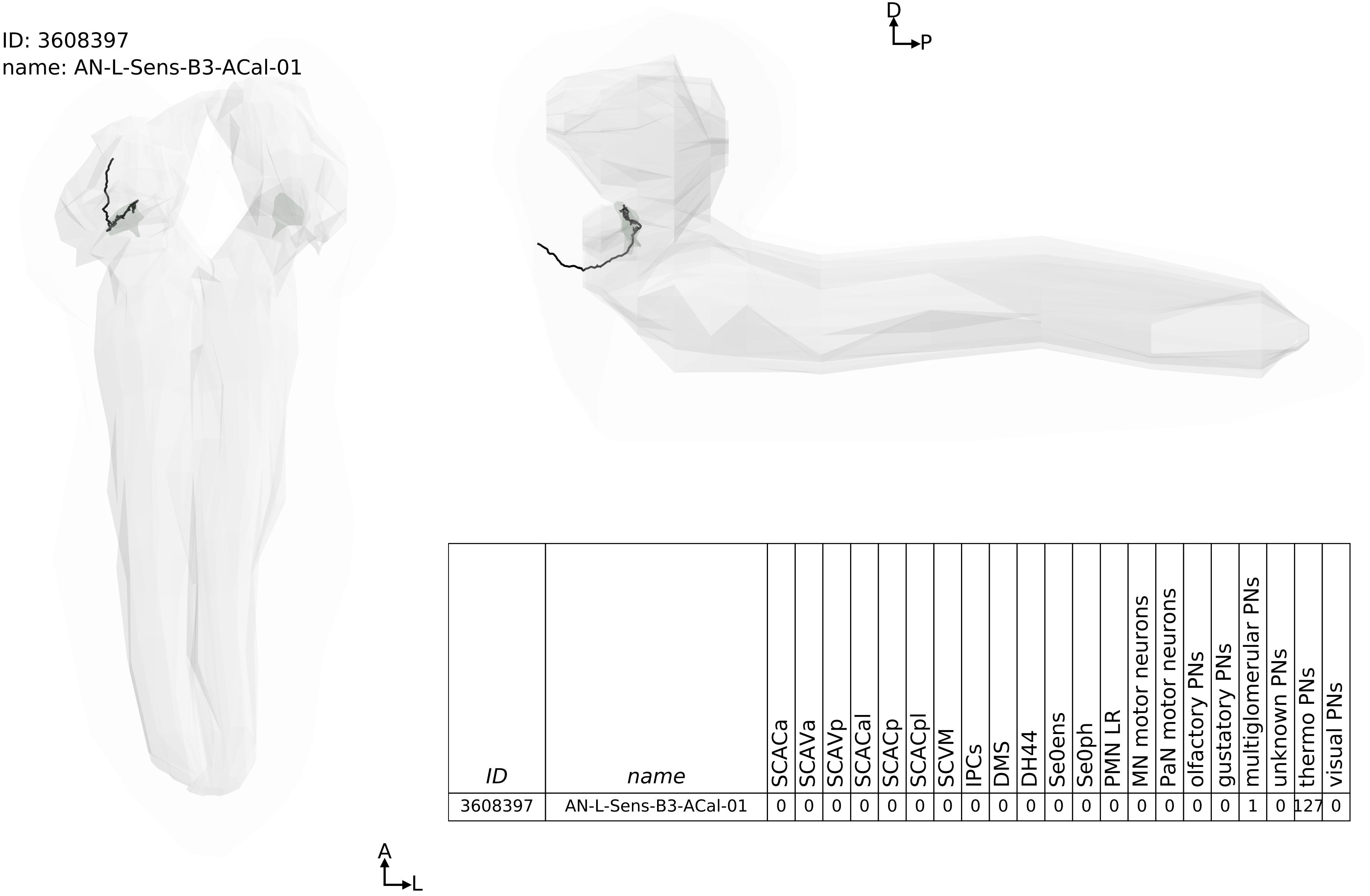

ID	*name*	SCACa	SCAVa	SCAVp	SCACal	SCACp	SCACpl	SCVM	IPCs	DMS	DH44	Se0ens	Se0ph	PMN LR	MN motor neurons	PaN motor neurons	olfactory PNs	gustatory PNs	multiglomerular PNs	unknown PNs	thermo PNs	visual PNs
3608397	AN-L-Sens-B3-ACal-01	0	0	0	0	0	0	0	0	0	0	0	0	0	0	0	0	0	1	0	127	0

ID: 3639968
name: AN-L-Sens-B3-ACal-02

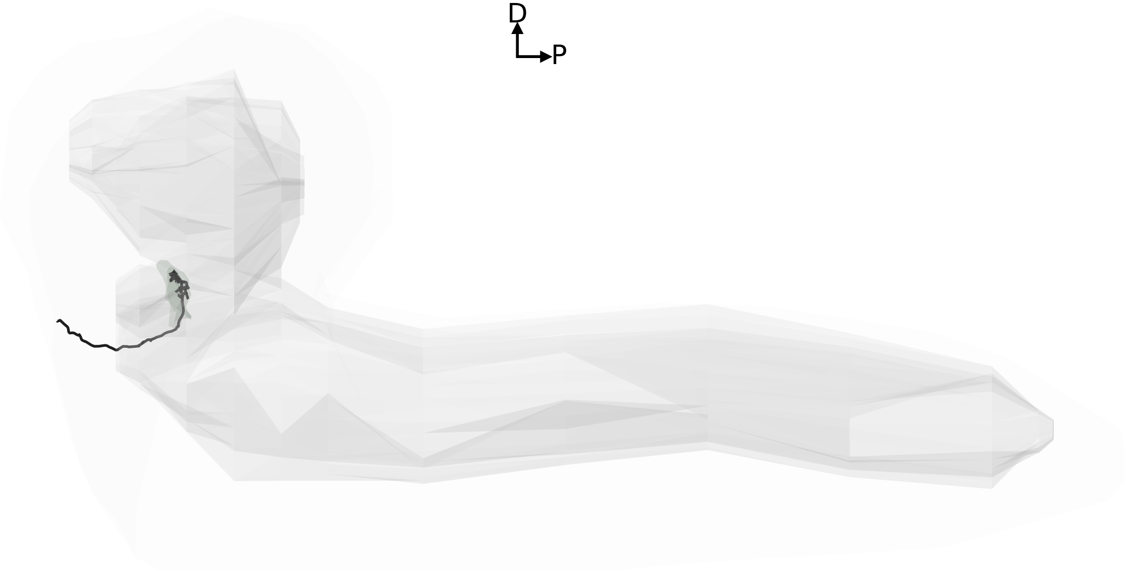

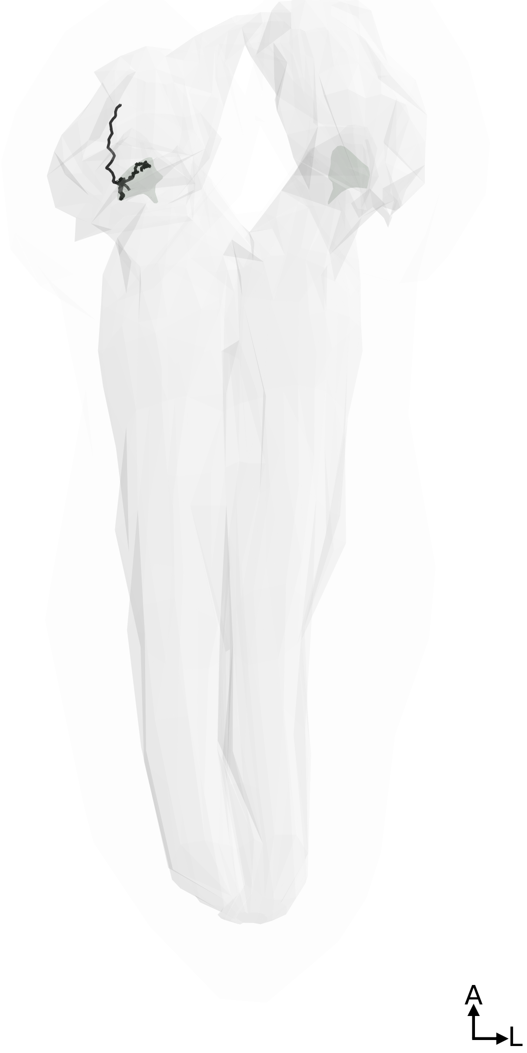

ID	name	SCACa	SCAVa	SCAVp	SCACal	SCACp	SCACpl	SCVM	IPCs	DMS	DH44	Se0ens	Se0ph	PMN LR	MN motor neurons	PaN motor neurons	olfactory PNs	gustatory PNs	multiglomerular PNs	unknown PNs	thermo PNs	visual PNs
3639968	AN-L-Sens-B3-ACal-02	0	0	0	2	0	0	0	0	0	0	0	0	0	0	0	2	0	2	0	131	0

ID: 3609202
name: AN-L-Sens-B3-ACal-03

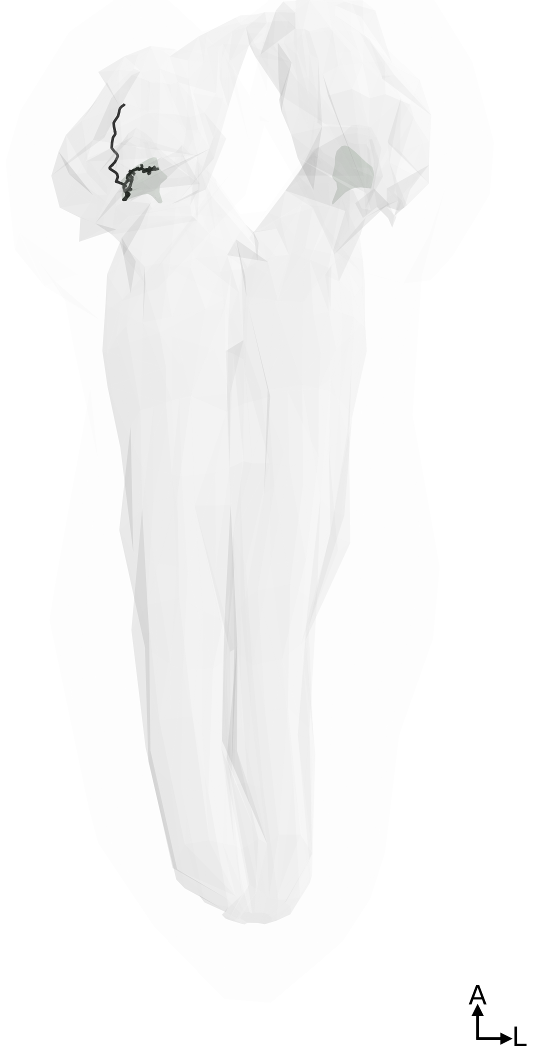

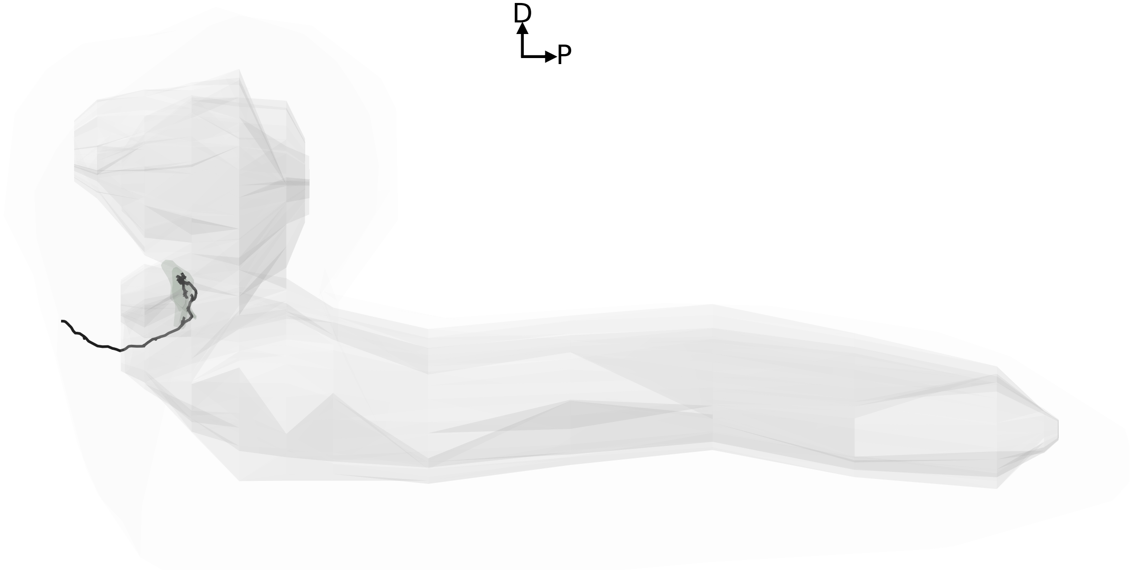

ID	*name*	SCACa	SCAVa	SCAVp	SCACal	SCACp	SCACpl	SCVM	IPCs	DMS	DH44	Se0ens	Se0ph	PMN LR	MN motor neurons	PaN motor neurons	olfactory PNs	gustatory PNs	multiglomerular PNs	unknown PNs	thermo PNs	visual PNs
3609202	AN-L-Sens-B3-ACal-03	0	0	0	0	0	0	0	0	0	0	0	0	0	0	0	1	0	5	0	130	0

ID: 5093516
name: AN-L-Sens-B3-ACal-04

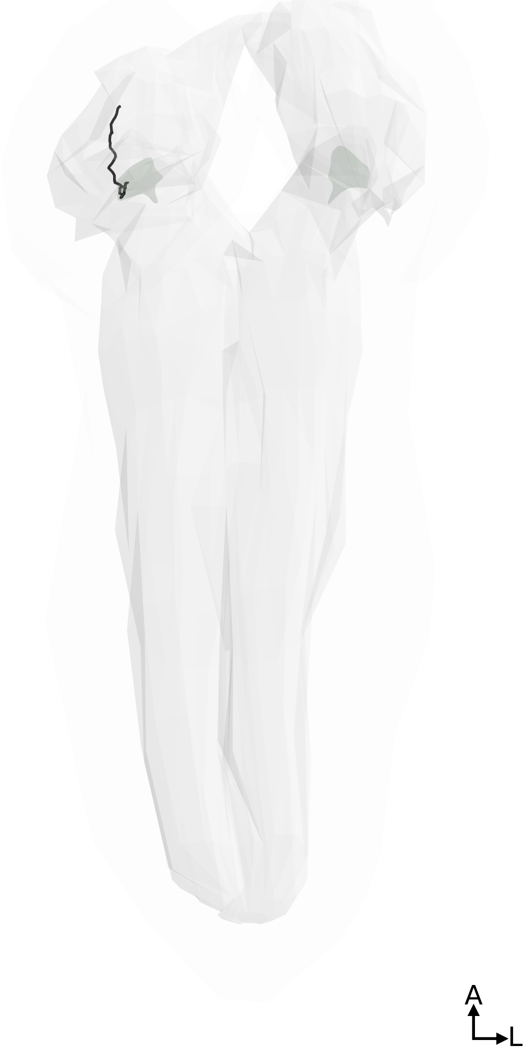

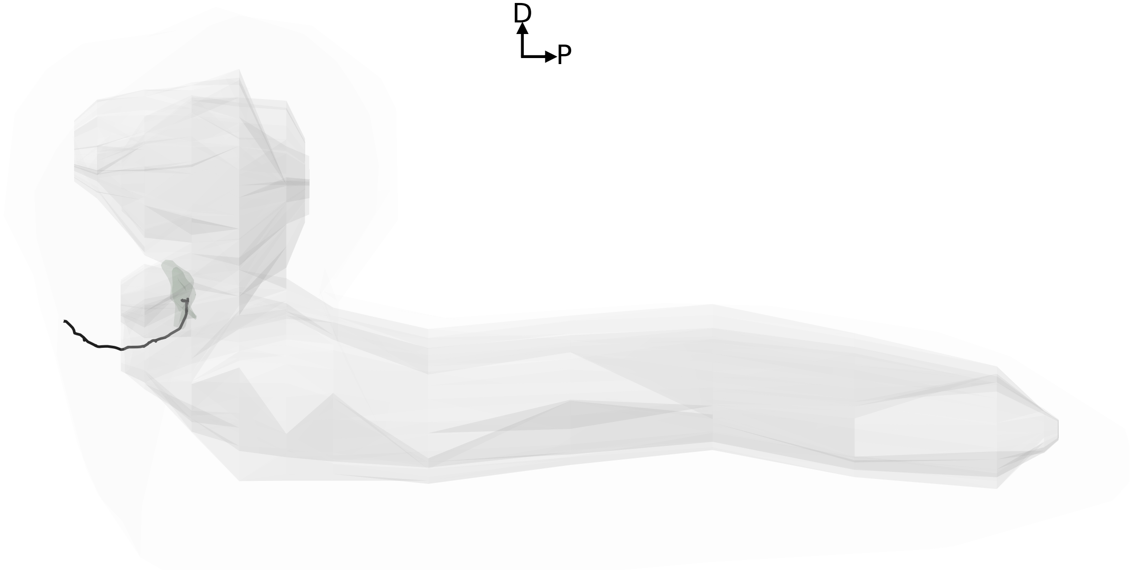

ID	*name*	SCACa	SCAVa	SCAVp	SCACal	SCACp	SCACpl	SCVM	IPCs	DMS	DH44	Se0ens	Se0ph	PMN LR	MN motor neurons	PaN motor neurons	olfactory PNs	gustatory PNs	multiglomerular PNs	unknown PNs	thermo PNs	visual PNs
5093516	AN-L-Sens-B3-ACal-04	0	0	0	0	0	0	0	0	0	0	0	0	0	0	0	0	0	10	0	0	0

ID: 2610744
name: AN-L-Sens-B3-ACal-05

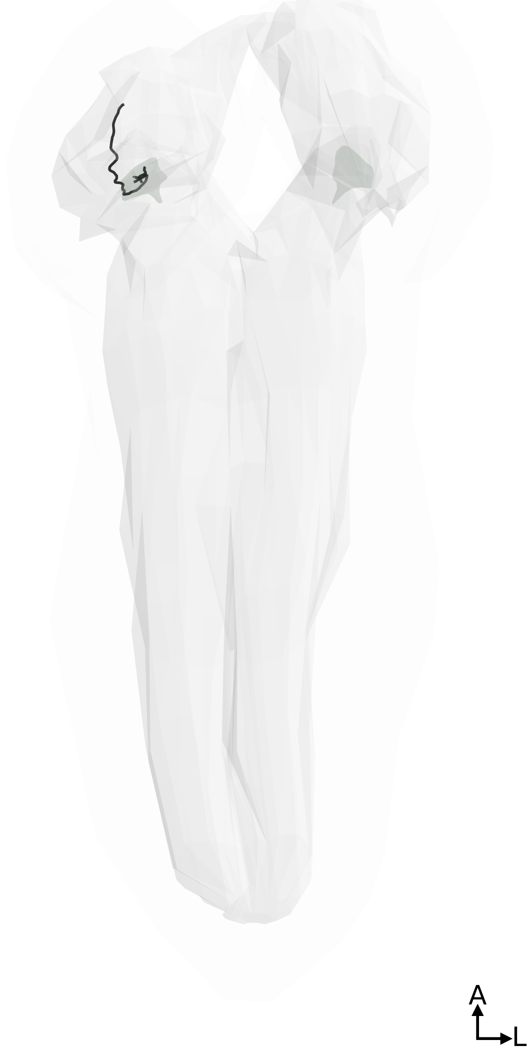

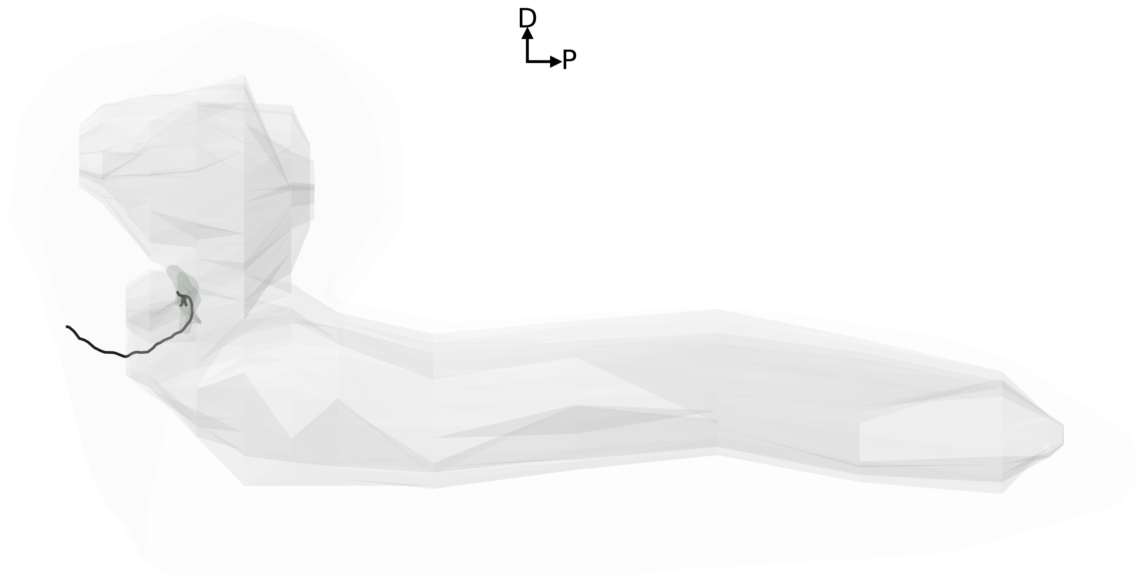

ID	*name*	SCACa	SCAVa	SCAVp	SCACal	SCACp	SCACpl	SCVM	IPCs	DMS	DH44	Se0ens	Se0ph	PMN LR	MN motor neurons	PaN motor neurons	olfactory PNs	gustatory PNs	multiglomerular PNs	unknown PNs	thermo PNs	visual PNs
2610744	AN-L-Sens-B3-ACal-05	0	0	0	0	0	0	0	0	0	0	0	0	0	0	0	0	0	22	0	0	0

ID: 5116147
name: AN-L-Sens-B3-ACal-06

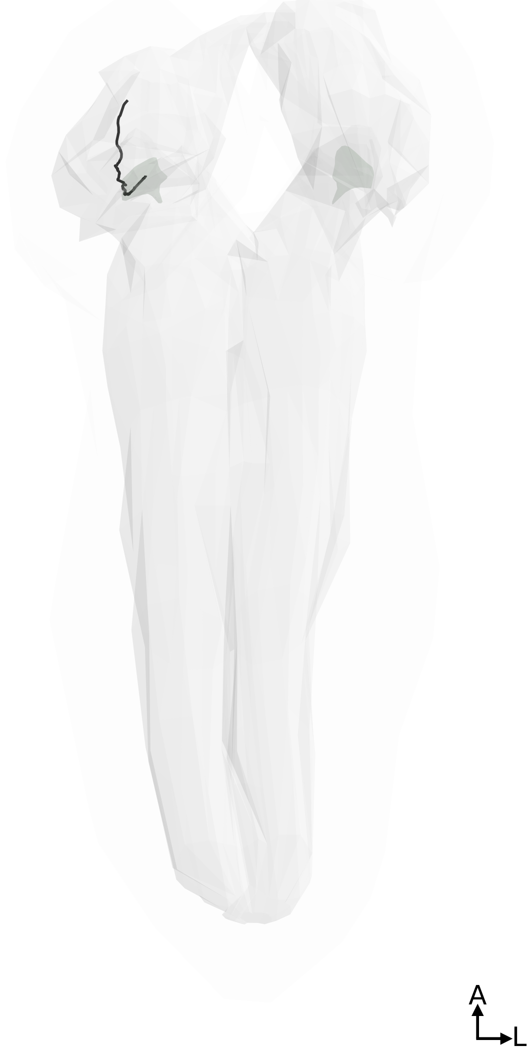

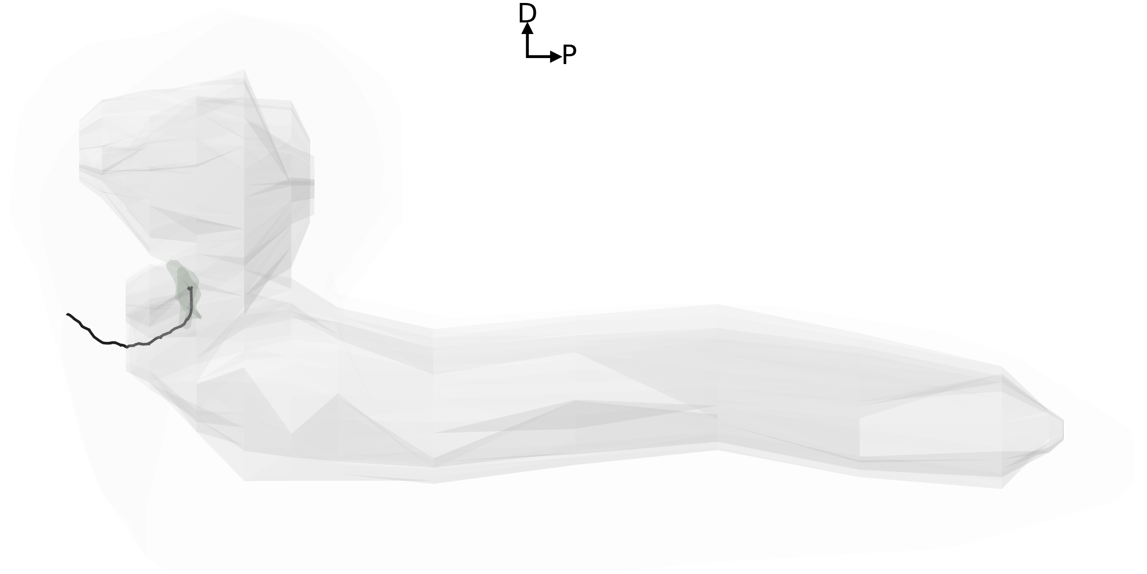

ID	*name*	SCACa	SCAVa	SCAVp	SCACal	SCACp	SCACpl	SCVM	IPCs	DMS	DH44	Se0ens	Se0ph	PMN LR	MN motor neurons	PaN motor neurons	olfactory PNs	gustatory PNs	multiglomerular PNs	unknown PNs	thermo PNs	visual PNs
5116147	AN-L-Sens-B3-ACal-06	0	0	0	2	0	0	0	0	0	0	0	0	0	0	0	0	0	0	0	1	0

ID: 3586984
name: AN-L-Sens-B3-ACal-07

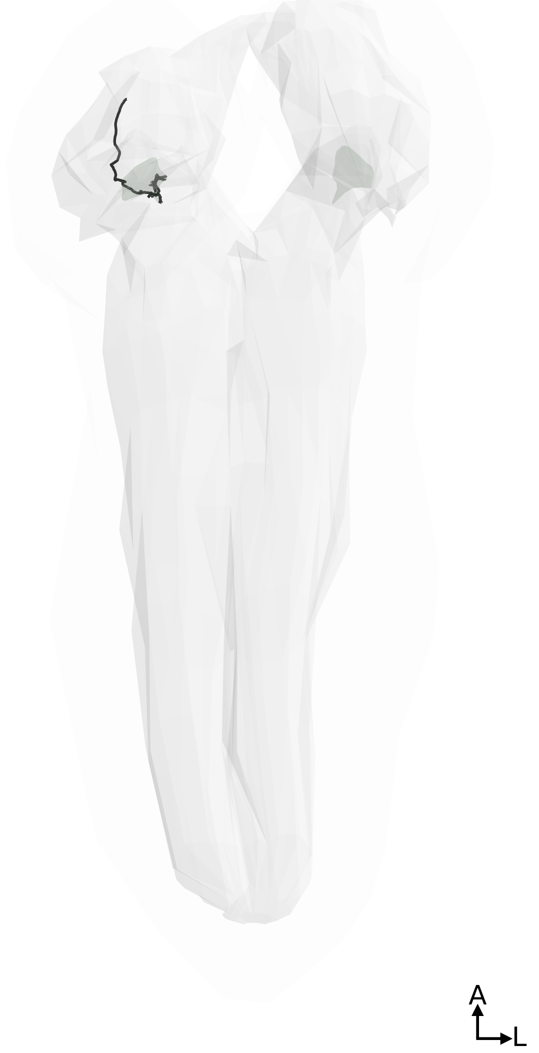

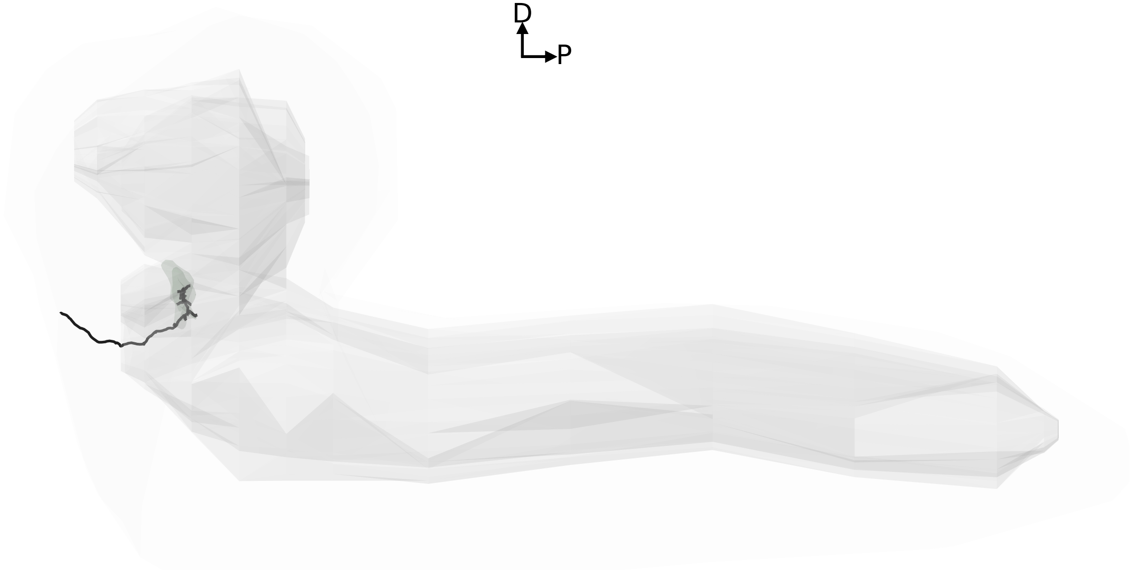

ID	name	SCACa	SCAVa	SCAVp	SCACal	SCACp	SCACpl	SCVM	IPCs	DMS	DH44	Se0ens	Se0ph	PMN LR	MN motor neurons	PaN motor neurons	olfactory PNs	gustatory PNs	multiglomerular PNs	unknown PNs	thermo PNs	visual PNs
3586984	AN-L-Sens-B3-ACal-07	0	0	0	0	1	0	0	0	0	0	0	0	0	0	0	0	4	0	0	0	0

ID: 3650600
name: AN-R-Sens-B3-ACal-01

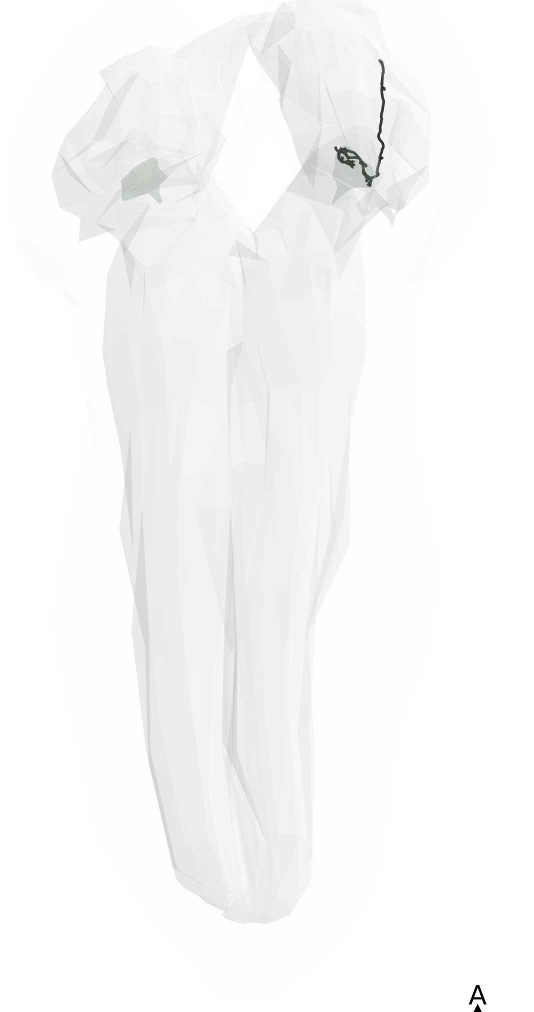

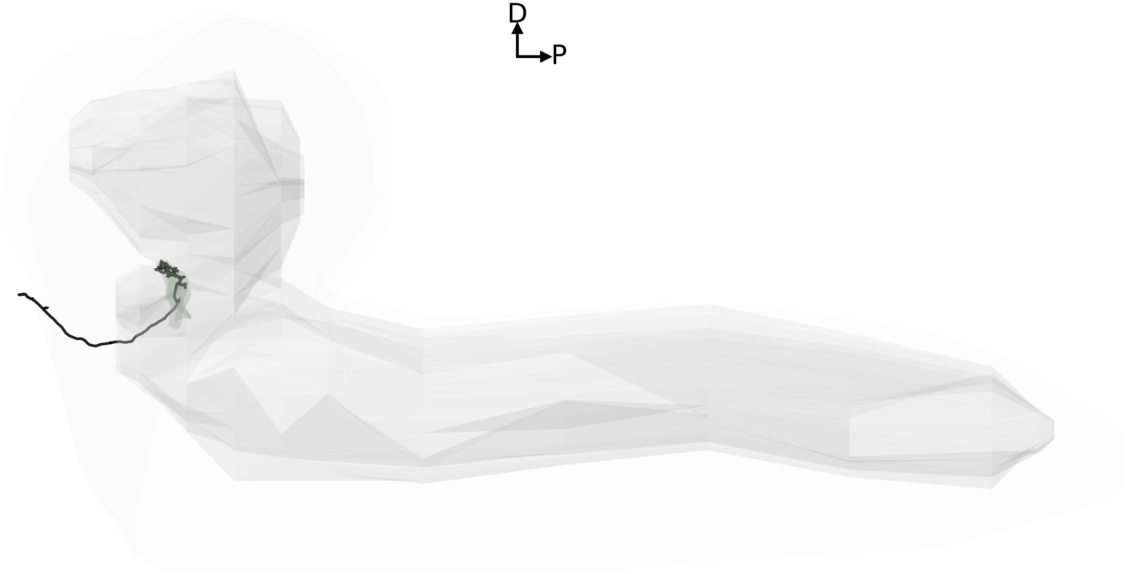

ID	*name*	SCACa	SCAVa	SCAVp	SCACal	SCACp	SCACpl	SCVM	IPCs	DMS	DH44	Se0ens	Se0ph	PMN LR	MN motor neurons	PaN motor neurons	olfactory PNs	gustatory PNs	multiglomerular PNs	unknown PNs	thermo PNs	visual PNs
3650600	AN-R-Sens-B3-ACal-01	0	0	0	4	1	0	0	0	0	0	0	0	0	0	0	0	0	0	0	95	0

ID: 18630153
name: AN-R-Sens-B3-ACal-02

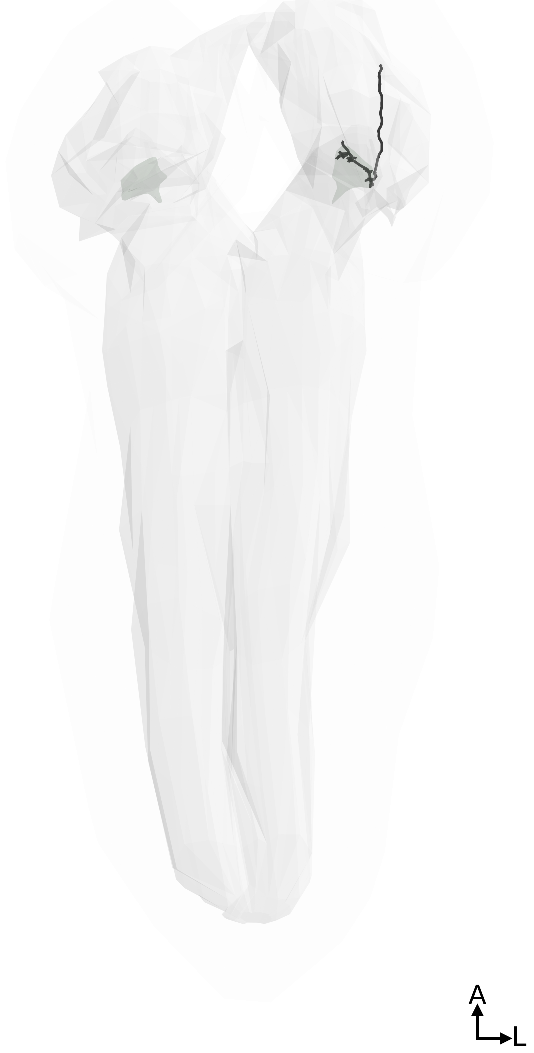

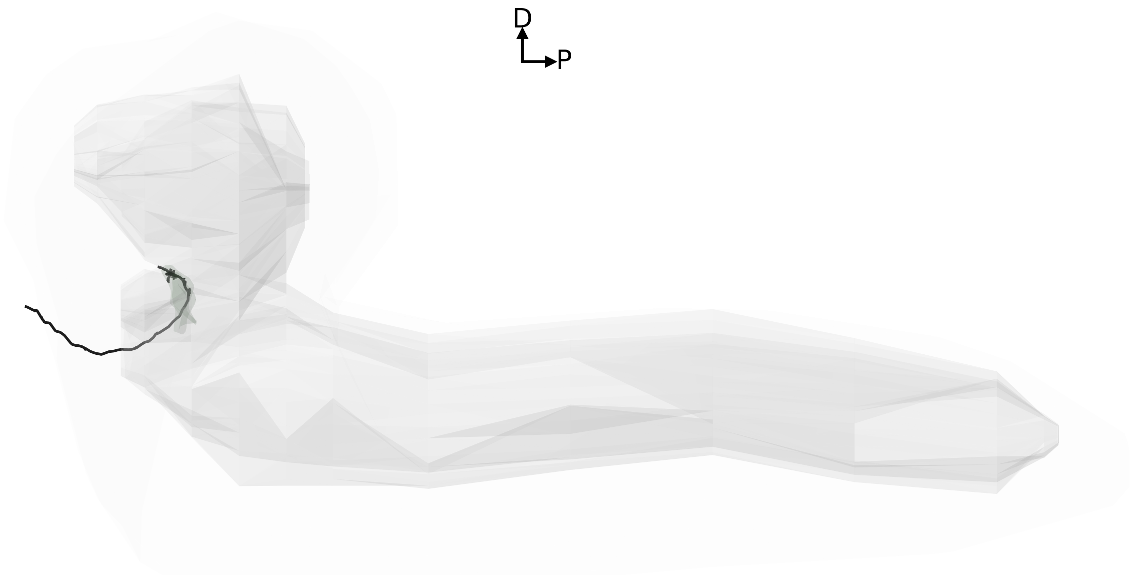

ID	*name*	SCACa	SCAVa	SCAVp	SCACal	SCACp	SCACpl	SCVM	IPCs	DMS	DH44	Se0ens	Se0ph	PMN LR	MN motor neurons	PaN motor neurons	olfactory PNs	gustatory PNs	multiglomerular PNs	unknown PNs	thermo PNs	visual PNs
18630153	AN-R-Sens-B3-ACal-02	0	0	0	0	0	0	0	0	0	0	0	0	0	0	0	0	0	0	0	130	0

ID: 3487306
name: AN-R-Sens-B3-ACal-03

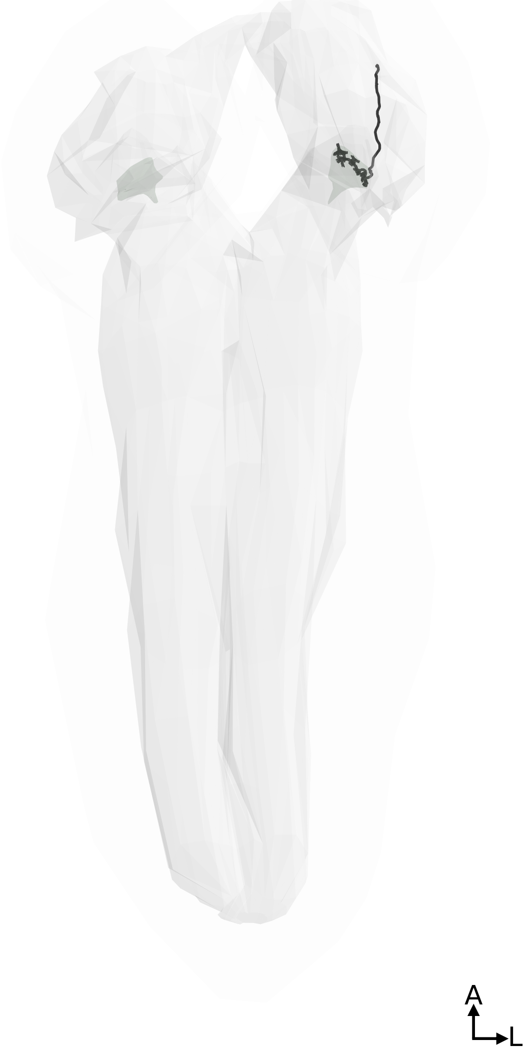

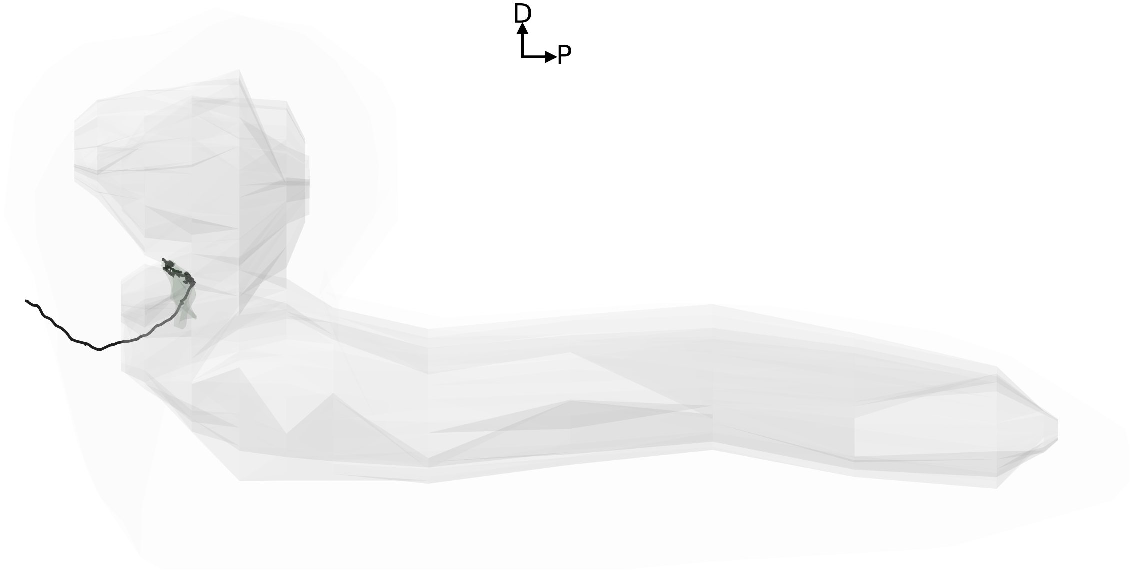

ID	*name*	SCACa	SCAVa	SCAVp	SCACal	SCACp	SCACpl	SCVM	IPCs	DMS	DH44	Se0ens	Se0ph	PMN LR	MN motor neurons	PaN motor neurons	olfactory PNs	gustatory PNs	multiglomerular PNs	unknown PNs	thermo PNs	visual PNs
3487306	AN-R-Sens-B3-ACal-03	0	0	0	4	0	0	0	0	0	0	0	0	0	0	0	0	0	0	0	165	0

ID: 4023881
name: AN-R-Sens-B3-ACal-04

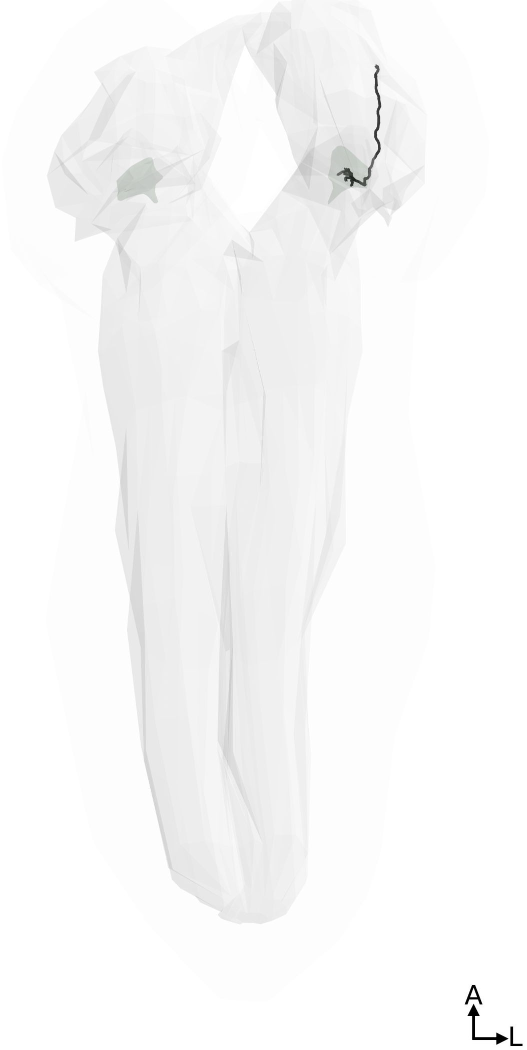

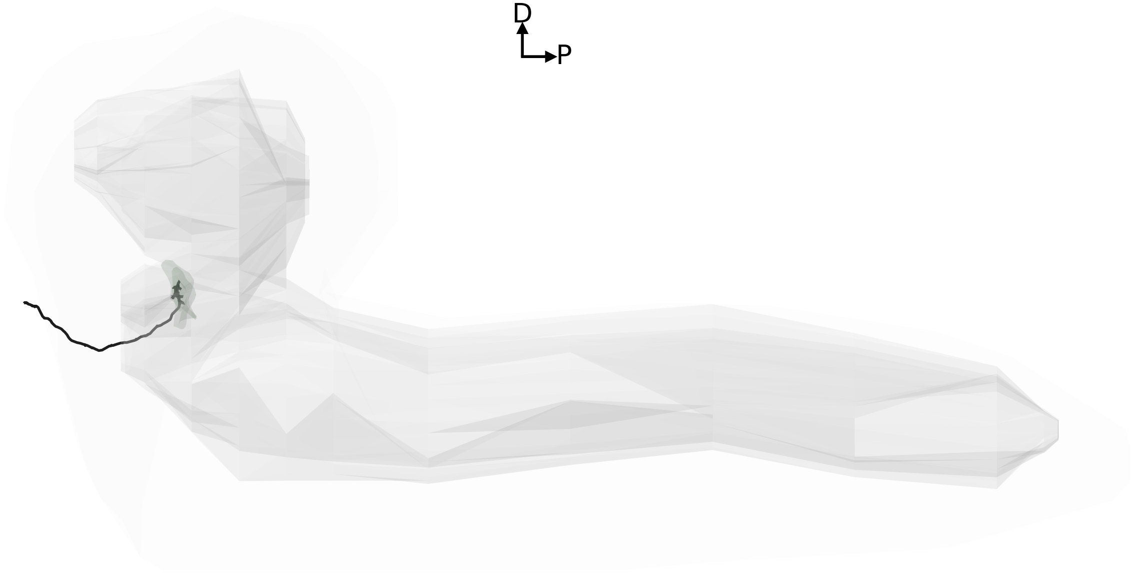

ID	*name*	SCACa	SCAVa	SCAVp	SCACal	SCACp	SCACpl	SCVM	IPCs	DMS	DH44	Se0ens	Se0ph	PMN LR	MN motor neurons	PaN motor neurons	olfactory PNs	gustatory PNs	multiglomerular PNs	unknown PNs	thermo PNs	visual PNs
4023881	AN-R-Sens-B3-ACal-04	0	0	0	0	0	0	0	0	0	0	0	0	0	0	0	0	0	34	0	0	0

ID: 3985829
name: AN-R-Sens-B3-ACal-05

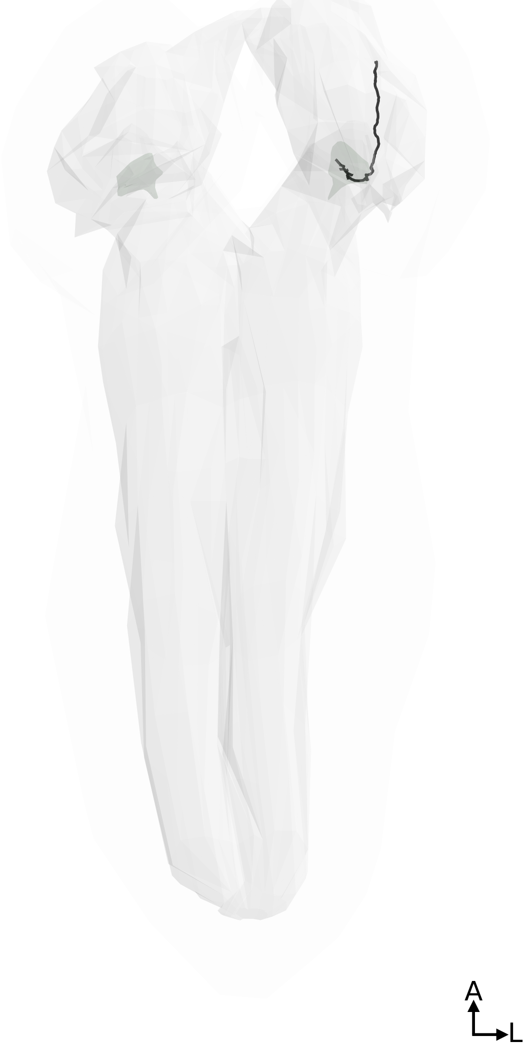

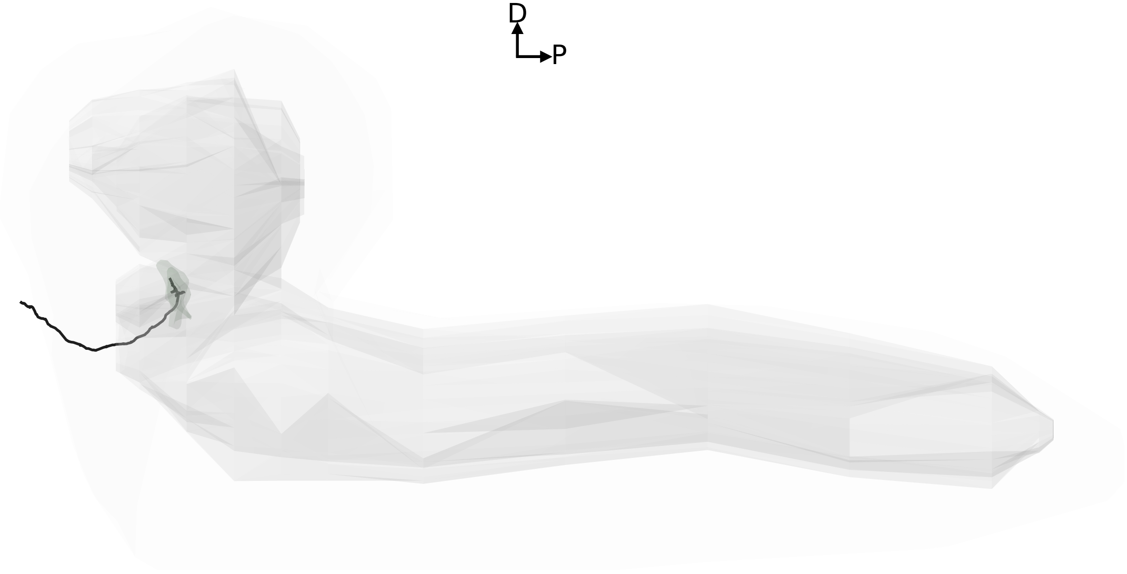

ID	*name*	SCACa	SCAVa	SCAVp	SCACal	SCACp	SCACpl	SCVM	IPCs	DMS	DH44	Se0ens	Se0ph	PMN LR	MN motor neurons	PaN motor neurons	olfactory PNs	gustatory PNs	multiglomerular PNs	unknown PNs	thermo PNs	visual PNs
3985829	AN-R-Sens-B3-ACal-05	0	0	0	0	0	0	0	0	0	0	0	0	0	0	0	0	0	15	0	0	0

ID: 4017089
name: AN-R-Sens-B3-ACal-06

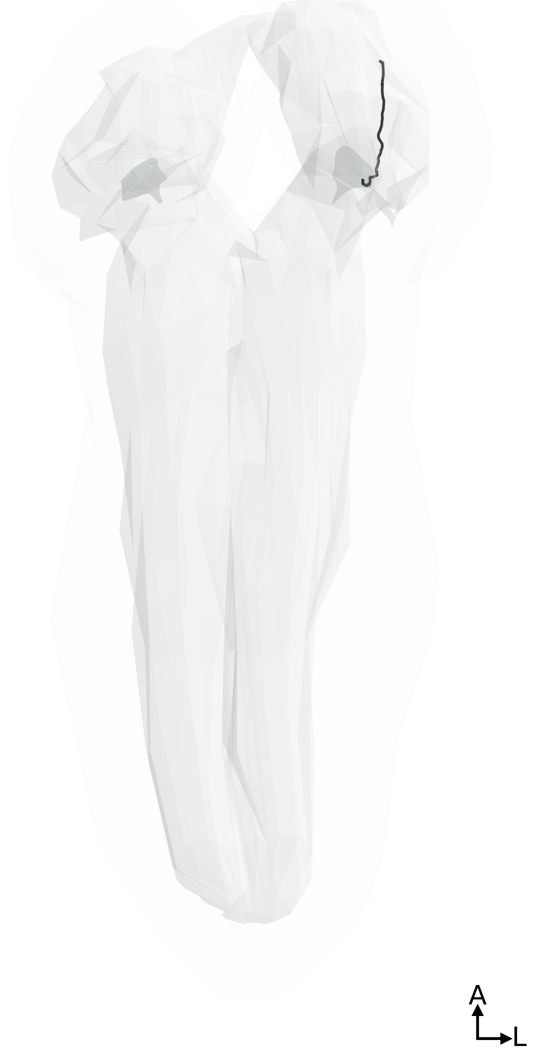

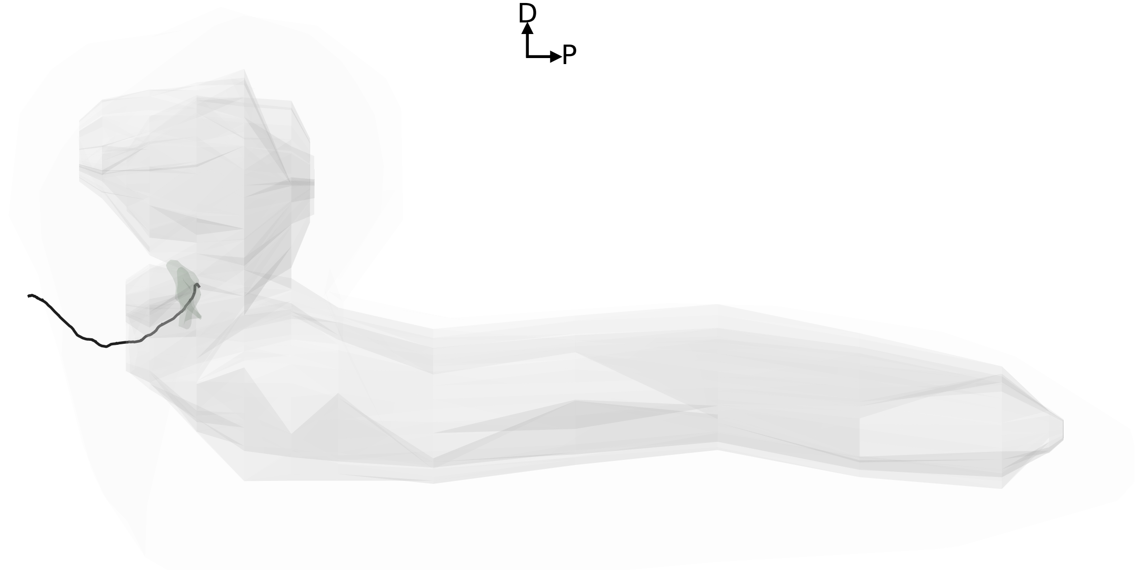

ID	*name*	SCACa	SCAVa	SCAVp	SCACal	SCACp	SCACpl	SCVM	IPCs	DMS	DH44	Se0ens	Se0ph	PMN LR	MN motor neurons	PaN motor neurons	olfactory PNs	gustatory PNs	multiglomerular PNs	unknown PNs	thermo PNs	visual PNs
4017089	AN-R-Sens-B3-ACal-06	0	0	0	0	0	0	0	0	0	0	0	0	0	0	0	0	0	1	0	2	0

ID: 3966683
name: AN-R-Sens-B3-ACal-07

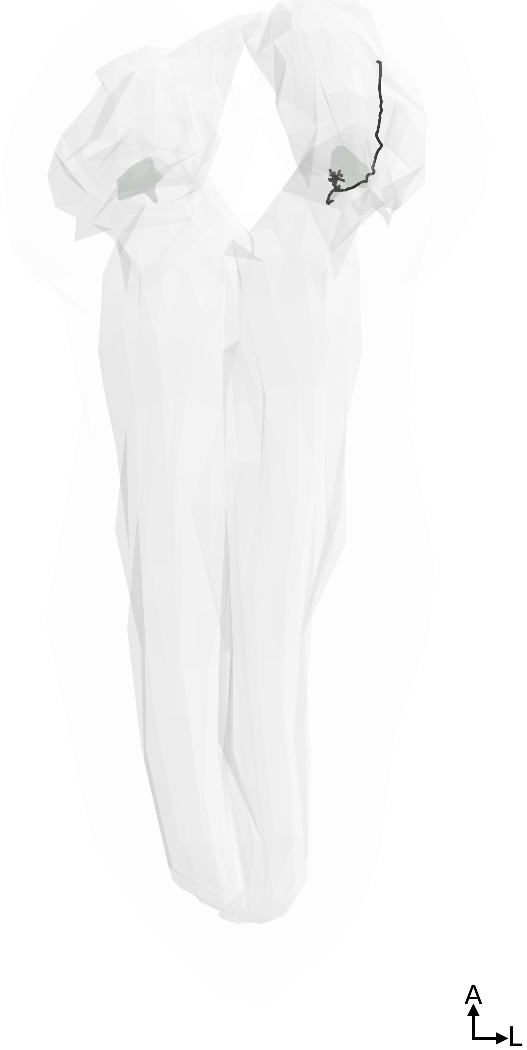

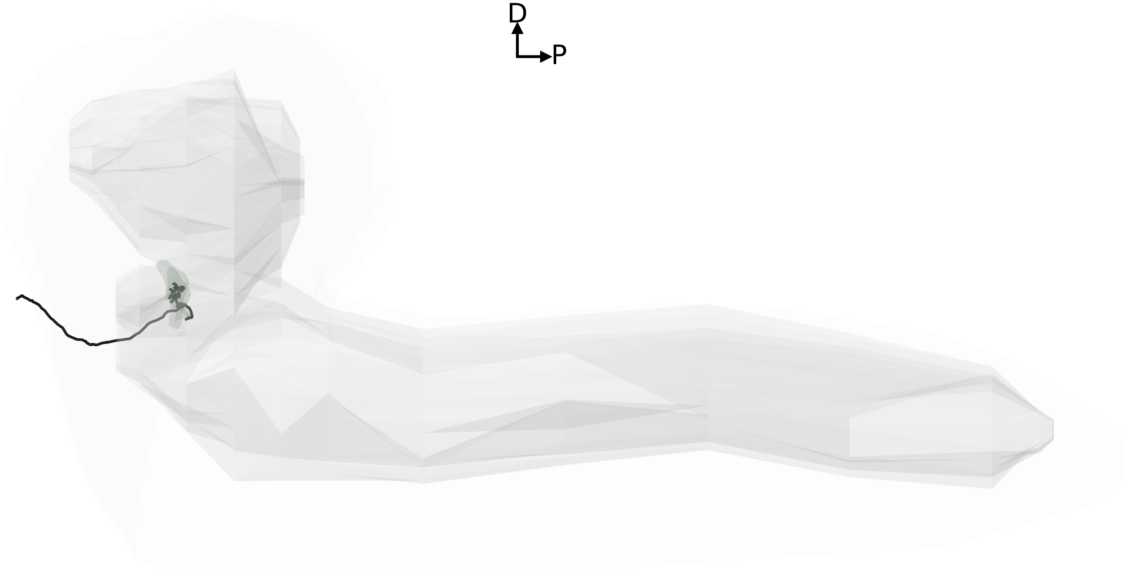

ID	*name*	SCACa	SCAVa	SCAVp	SCACal	SCACp	SCACpl	SCVM	IPCs	DMS	DH44	Se0ens	Se0ph	PMN LR	MN motor neurons	PaN motor neurons	olfactory PNs	gustatory PNs	multiglomerular PNs	unknown PNs	thermo PNs	visual PNs
3966683	AN-R-Sens-B3-ACal-07	0	0	0	0	1	0	0	0	0	0	0	0	0	0	0	0	1	0	0	0	0

ID: 15997666
name: AN-L-Sens-B2-ACp-01

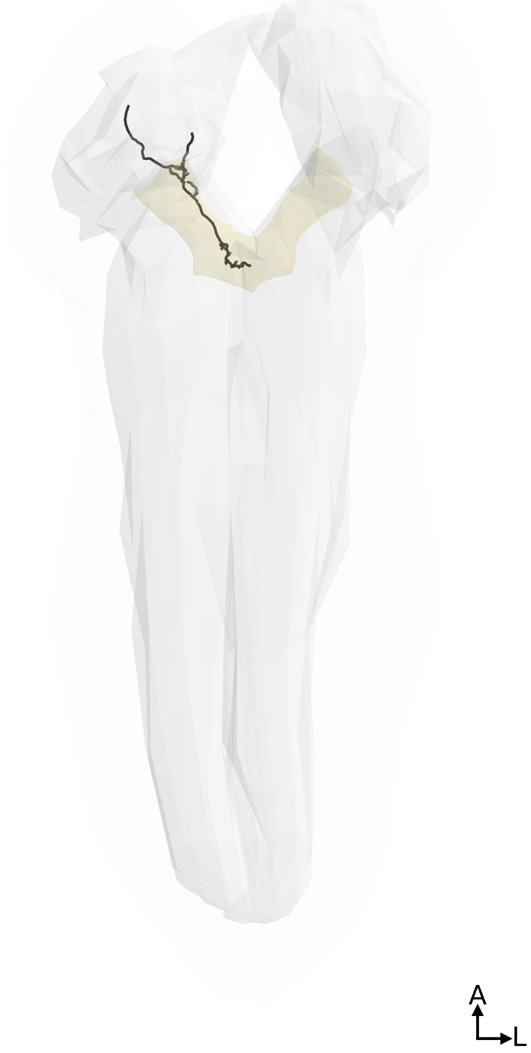

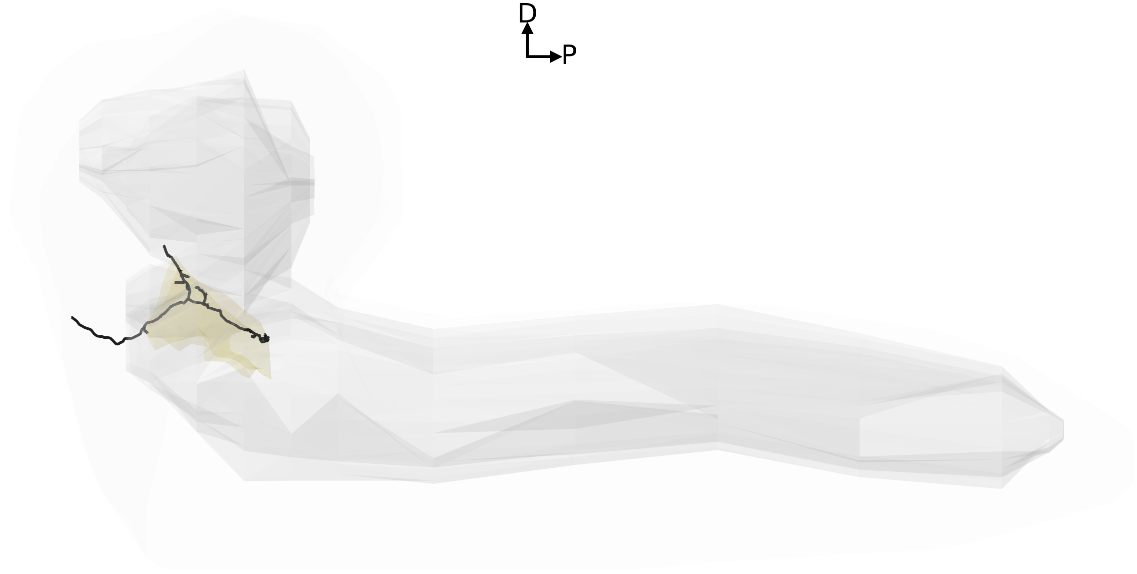

ID	*name*	SCACa	SCAVa	SCAVp	SCACal	SCACp	SCACpl	SCVM	IPCs	DMS	DH44	Se0ens	Se0ph	PMN LR	MN motor neurons	PaN motor neurons	olfactory PNs	gustatory PNs	multiglomerular PNs	unknown PNs	thermo PNs	visual PNs
15997666	AN-L-Sens-B2-ACp-01	0	0	0	0	4	0	0	0	0	0	0	0	0	0	0	0	0	0	0	0	0

ID: 1011675
name: AN-L-Sens-B2-ACp-02

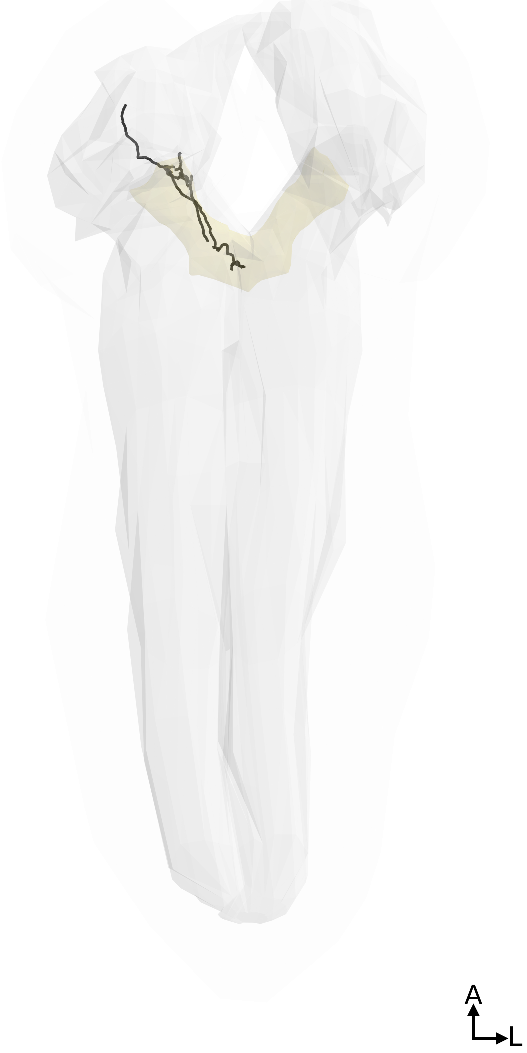

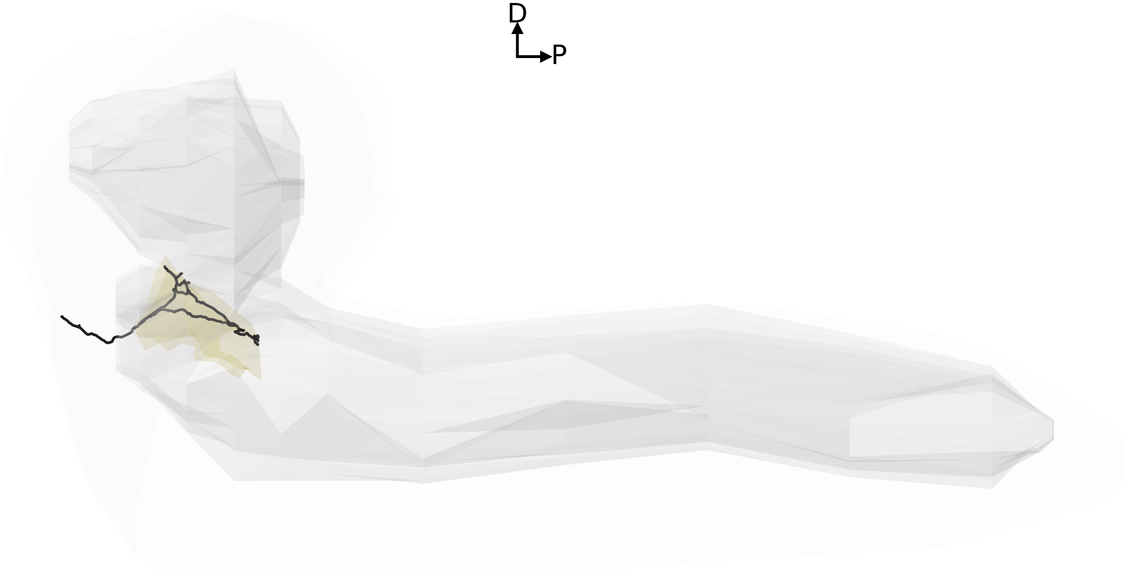

ID	name	SCACa	SCAVa	SCAVp	SCACal	SCACp	SCACpl	SCVM	IPCs	DMS	DH44	Se0ens	Se0ph	PMN LR	MN motor neurons	PaN motor neurons	olfactory PNs	gustatory PNs	multiglomerular PNs	unknown PNs	thermo PNs	visual PNs
1011675	AN-L-Sens-B2-ACp-02	0	0	0	0	3	0	0	0	0	0	0	0	0	0	0	0	0	0	0	0	0

ID: 7439909
name: AN-L-Sens-B2-ACp-03

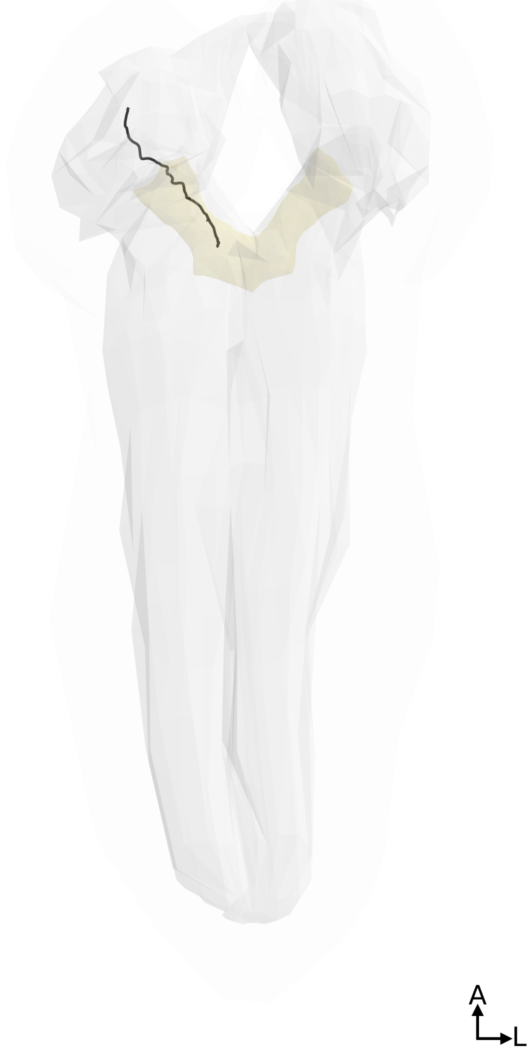

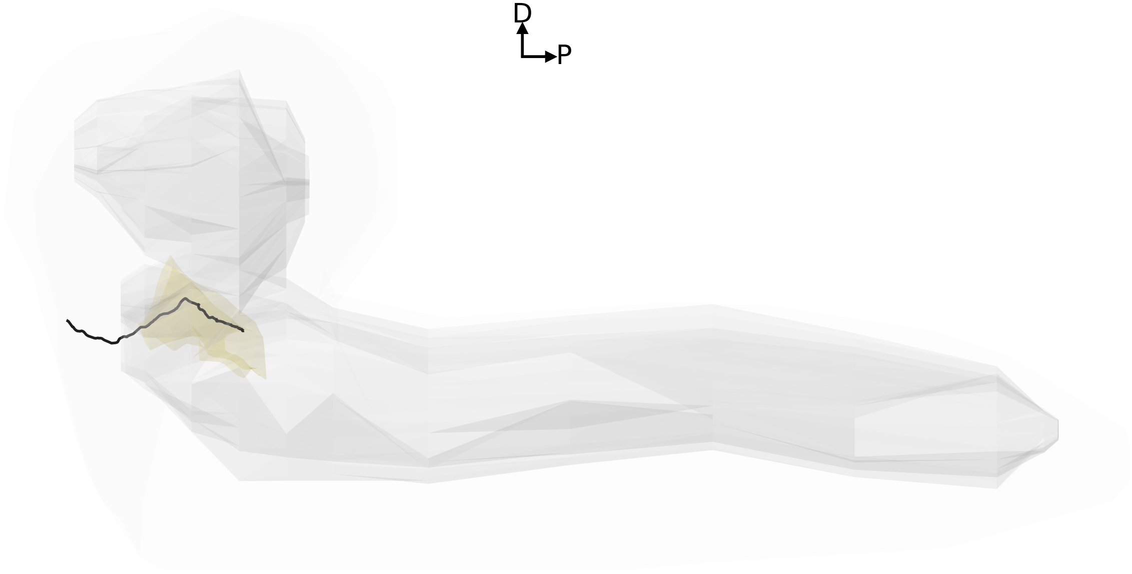

ID	*name*	SCACa	SCAVa	SCAVp	SCACal	SCACp	SCACpl	SCVM	IPCs	DMS	DH44	Se0ens	Se0ph	PMN LR	MN motor neurons	PaN motor neurons	olfactory PNs	gustatory PNs	multiglomerular PNs	unknown PNs	thermo PNs	visual PNs
7439909	AN-L-Sens-B2-ACp-03	0	0	0	0	5	0	0	0	0	0	0	0	0	0	0	0	0	0	0	0	0

ID: 15998782
name: AN-L-Sens-B2-ACp-04

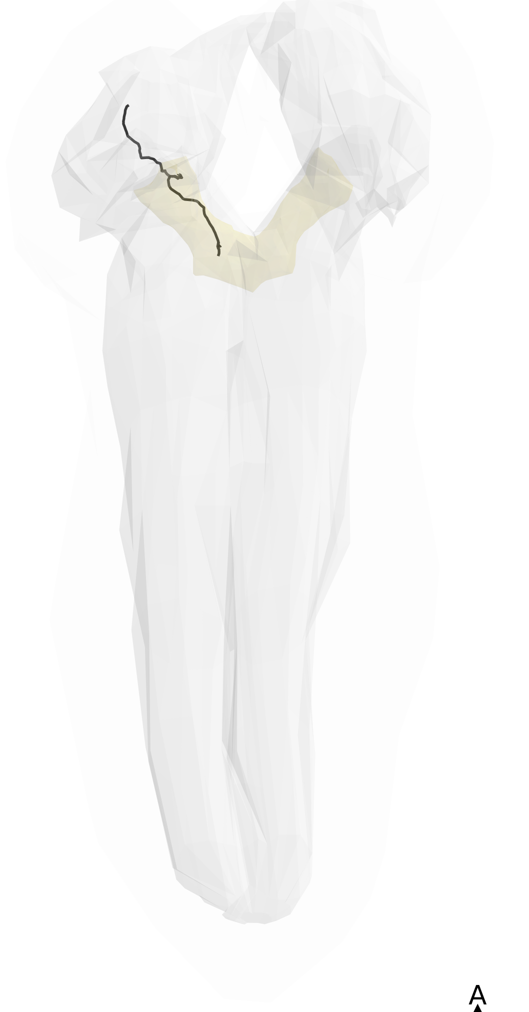

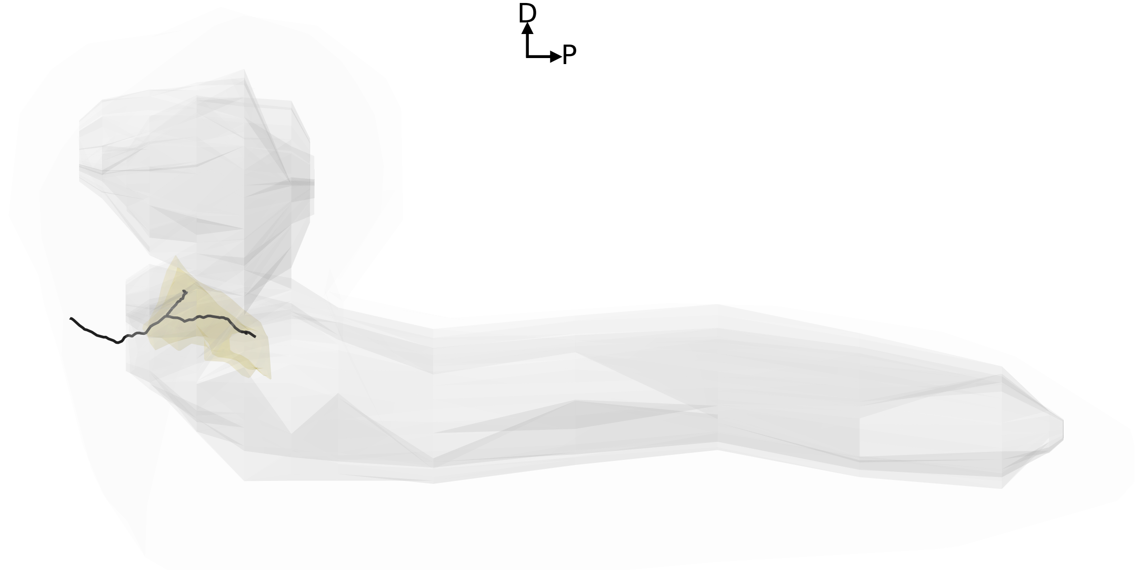

ID	*name*	SCACa	SCAVa	SCAVp	SCACal	SCACp	SCACpl	SCVM	IPCs	DMS	DH44	Se0ens	Se0ph	PMN LR	MN motor neurons	PaN motor neurons	olfactory PNs	gustatory PNs	multiglomerular PNs	unknown PNs	thermo PNs	visual PNs
15998782	AN-L-Sens-B2-ACp-04	0	0	0	0	5	0	0	0	0	0	0	0	0	0	0	0	0	0	0	0	0

ID: 15998469
name: AN-L-Sens-B2-ACp-05

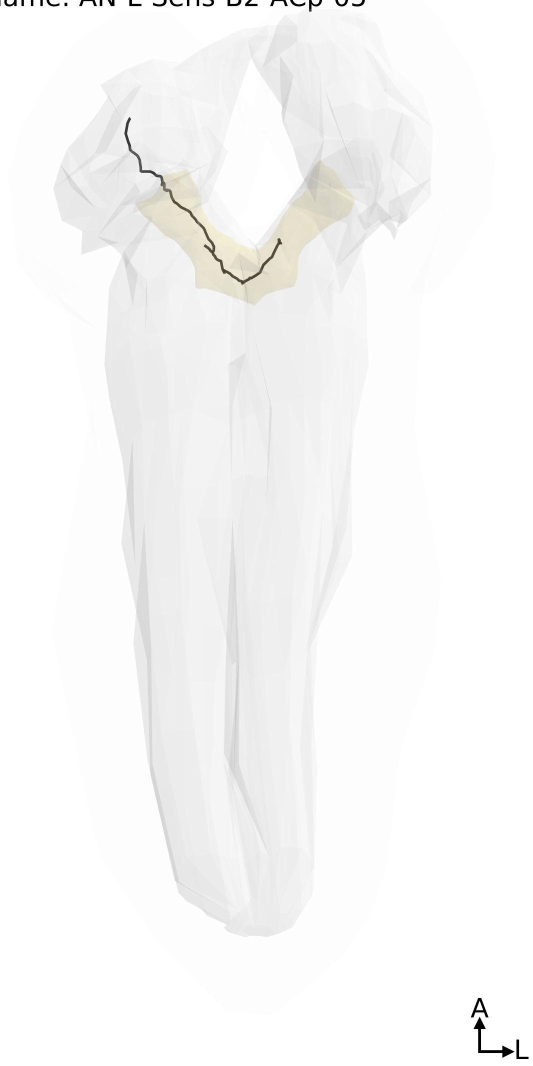

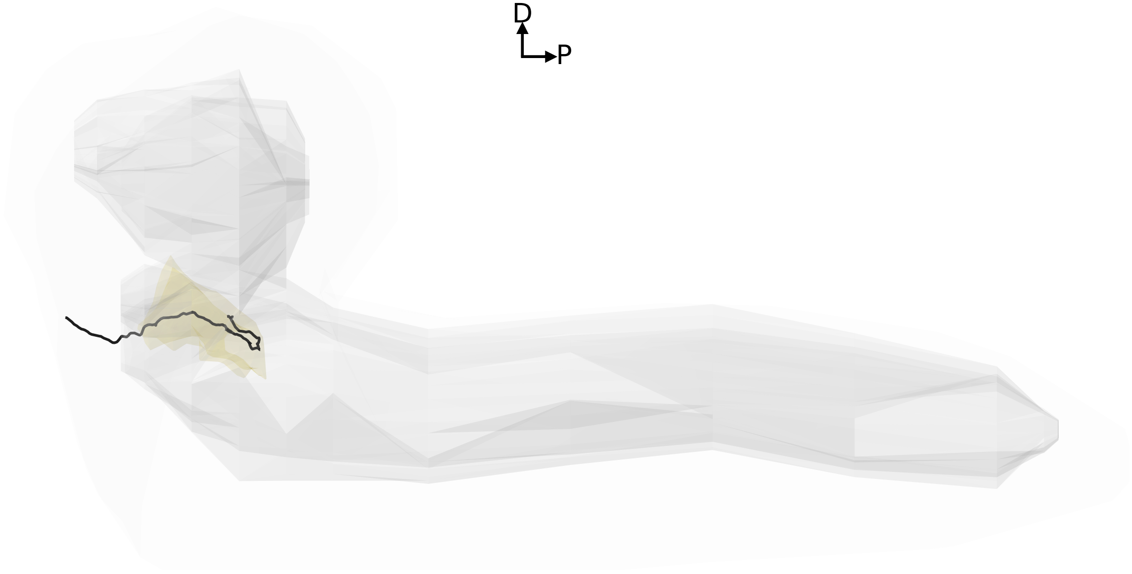

ID	*name*	SCACa	SCAVa	SCAVp	SCACal	SCACp	SCACpl	SCVM	IPCs	DMS	DH44	Se0ens	Se0ph	PMN LR	MN motor neurons	PaN motor neurons	olfactory PNs	gustatory PNs	multiglomerular PNs	unknown PNs	thermo PNs	visual PNs
15998469	AN-L-Sens-B2-ACp-05	0	0	0	0	3	0	0	0	0	0	0	0	0	0	0	0	0	0	0	0	0

ID: 15997983
name: AN-L-Sens-B2-ACp-06

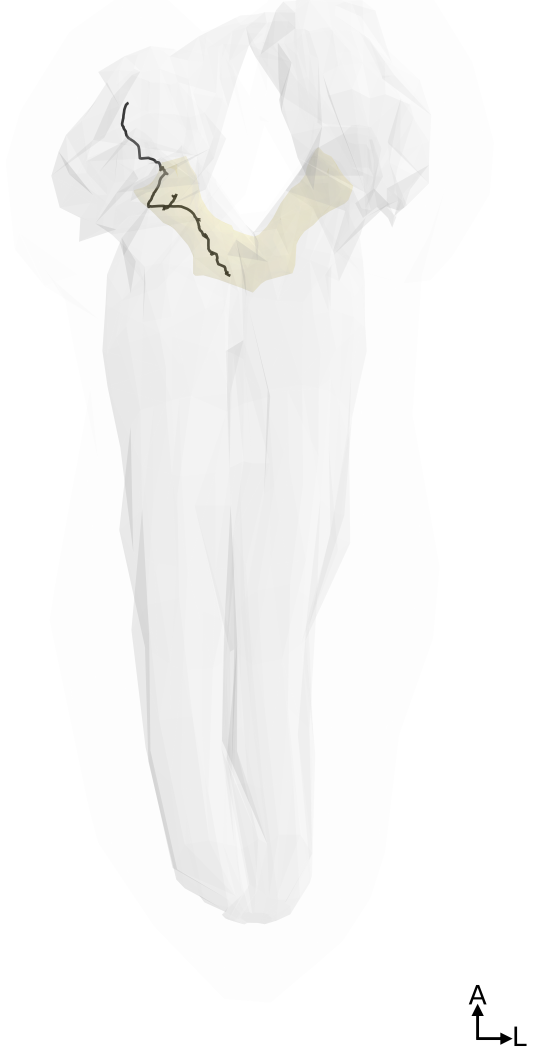

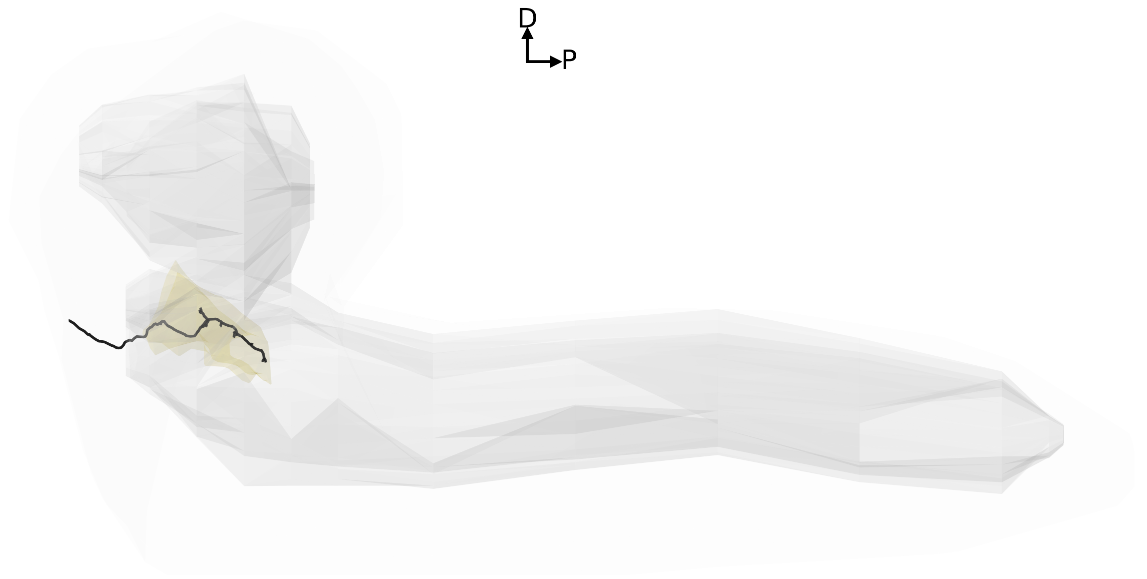

ID	*name*	SCACa	SCAVa	SCAVp	SCACal	SCACp	SCACpl	SCVM	IPCs	DMS	DH44	Se0ens	Se0ph	PMN LR	MN motor neurons	PaN motor neurons	olfactory PNs	gustatory PNs	multiglomerular PNs	unknown PNs	thermo PNs	visual PNs
15997983	AN-L-Sens-B2-ACp-06	0	0	0	0	1	0	0	0	0	0	0	0	0	0	0	0	1	0	0	0	0

ID: 15590761
name: AN-L-Sens-B2-ACp-07

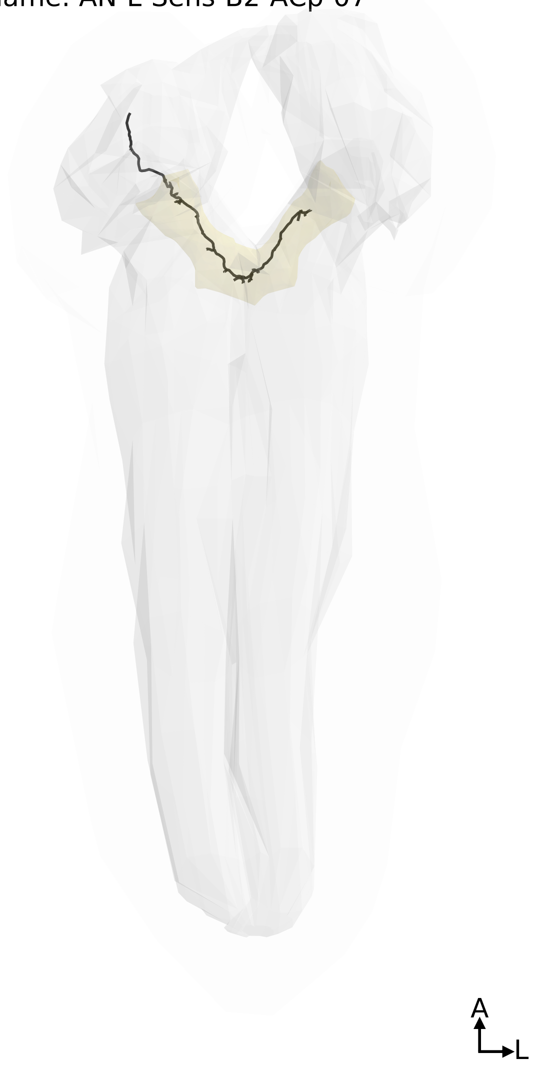

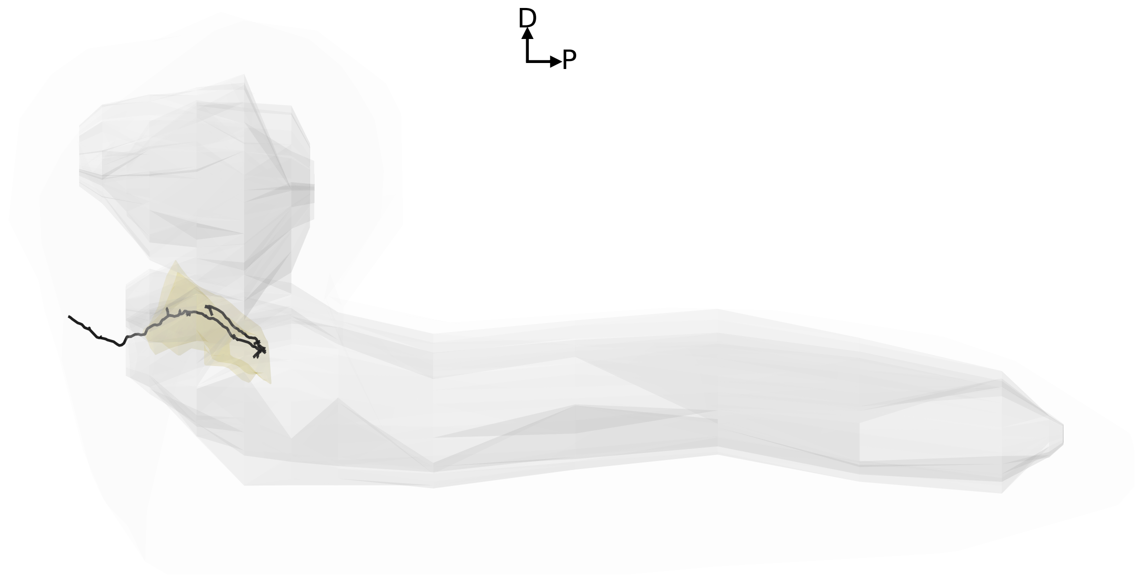

ID	*name*	SCACa	SCAVa	SCAVp	SCACal	SCACp	SCACpl	SCVM	IPCs	DMS	DH44	Se0ens	Se0ph	PMN LR	MN motor neurons	PaN motor neurons	olfactory PNs	gustatory PNs	multiglomerular PNs	unknown PNs	thermo PNs	visual PNs
15590761	AN-L-Sens-B2-ACp-07	0	0	0	0	0	0	0	0	0	0	0	0	0	0	0	0	0	0	0	0	0

ID: 15998218
name: AN-L-Sens-B2-ACp-08

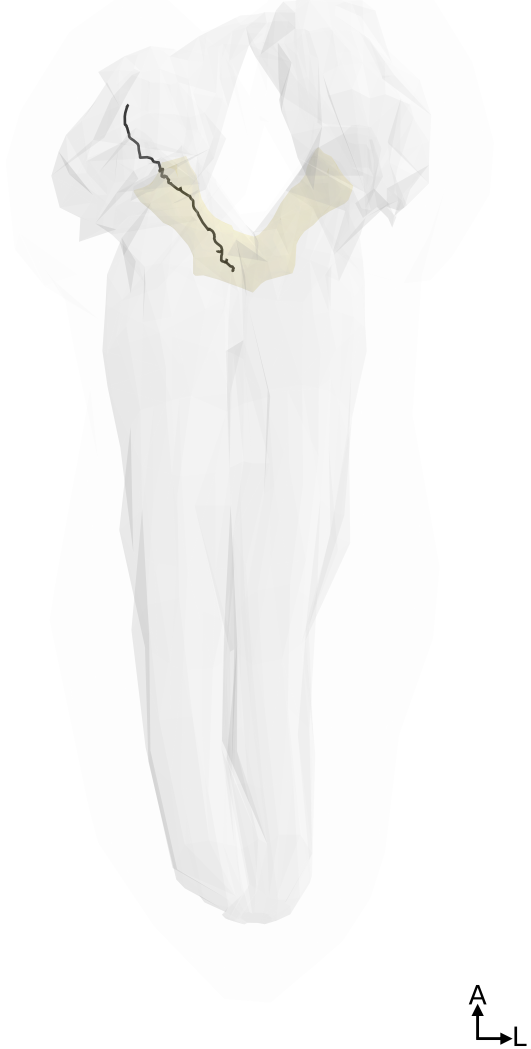

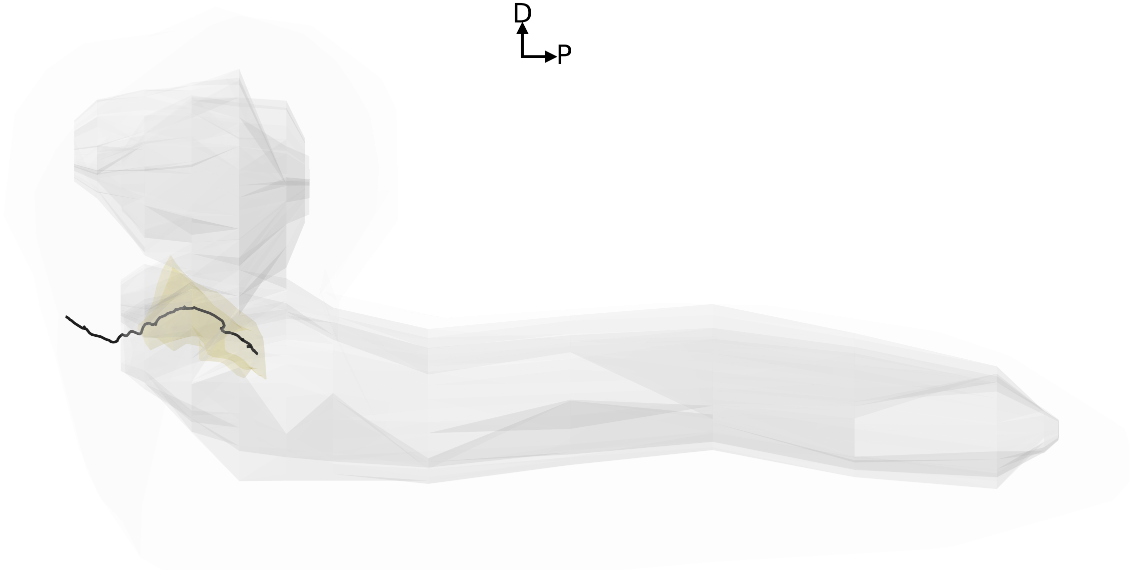

ID	name	SCACa	SCAVa	SCAVp	SCACal	SCACp	SCACpl	SCVM	IPCs	DMS	DH44	Se0ens	Se0ph	PMN LR	MN motor neurons	PaN motor neurons	olfactory PNs	gustatory PNs	multiglomerular PNs	unknown PNs	thermo PNs	visual PNs
15998218	AN-L-Sens-B2-ACp-08	0	0	0	0	1	0	0	0	0	0	0	0	0	0	0	0	0	0	0	0	0

ID: 15998768
name: AN-L-Sens-B2-ACp-09

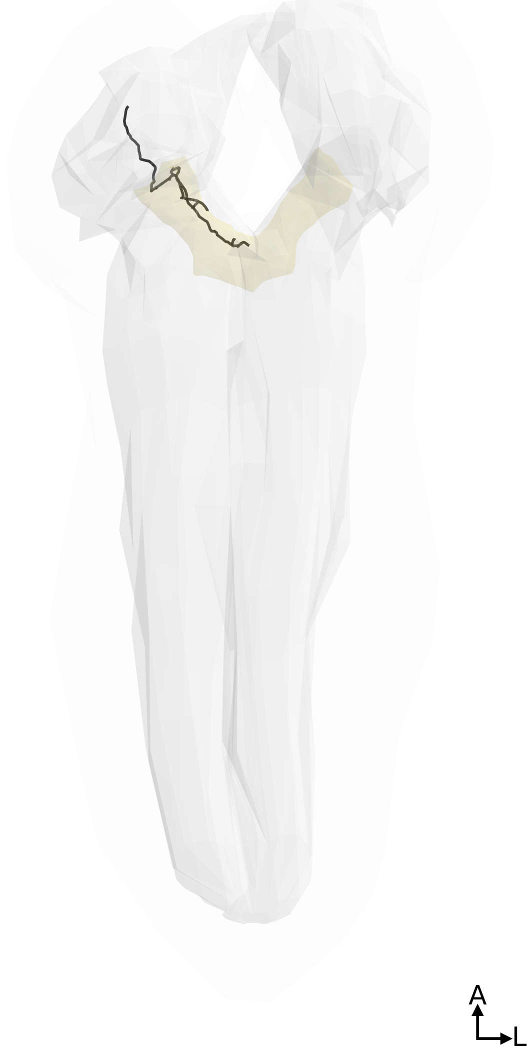

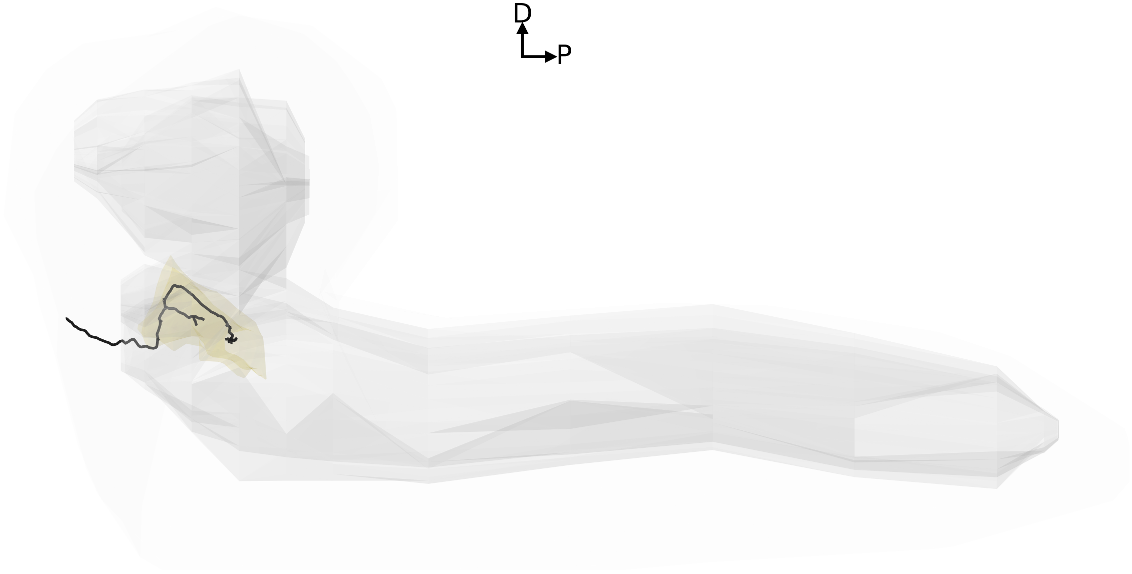

ID	name	SCACa	SCAVa	SCAVp	SCACal	SCACp	SCACpl	SCVM	IPCs	DMS	DH44	Se0ens	Se0ph	PMN LR	MN motor neurons	PaN motor neurons	olfactory PNs	gustatory PNs	multiglomerular PNs	unknown PNs	thermo PNs	visual PNs
15998768	AN-L-Sens-B2-ACp-09	0	0	0	0	2	0	0	0	0	0	0	0	0	0	0	1	1	0	0	0	0

ID: 15998307
name: AN-L-Sens-B2-ACp-10

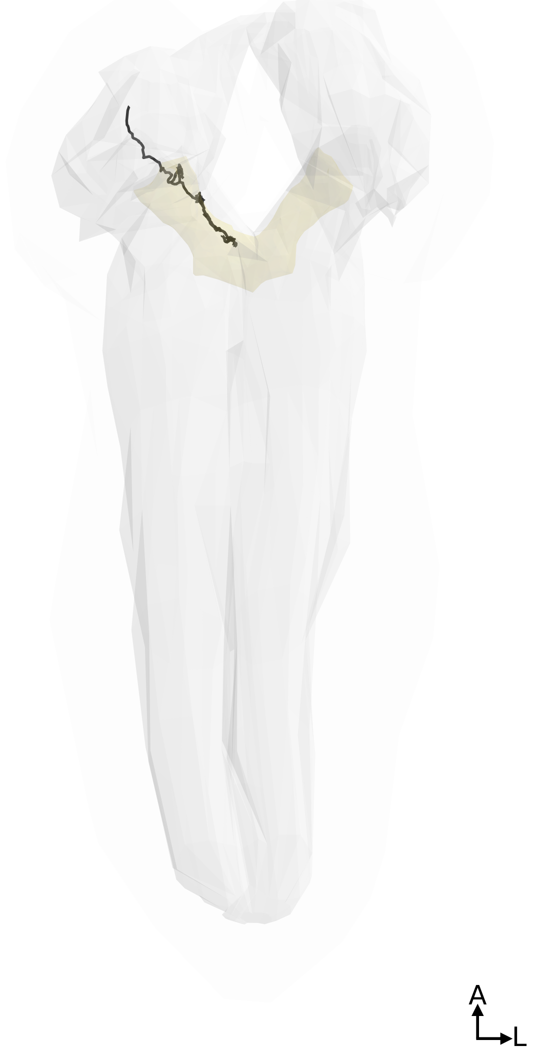

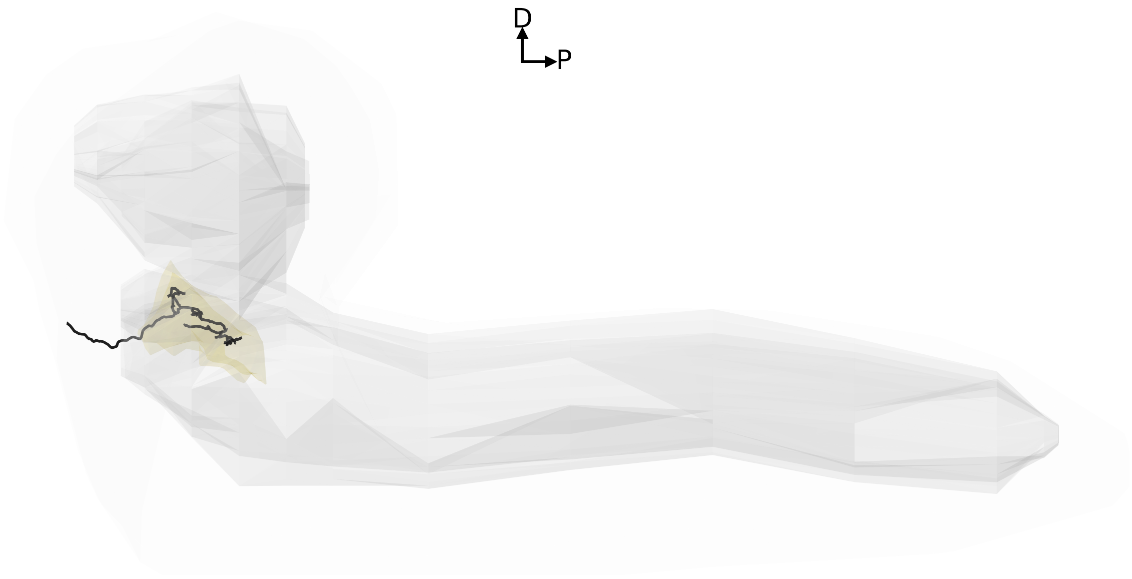

ID	*name*	SCACa	SCAVa	SCAVp	SCACal	SCACp	SCACpl	SCVM	IPCs	DMS	DH44	Se0ens	Se0ph	PMN LR	MN motor neurons	PaN motor neurons	olfactory PNs	gustatory PNs	multiglomerular PNs	unknown PNs	thermo PNs	visual PNs
15998307	AN-L-Sens-B2-ACp-10	0	0	0	0	6	0	0	0	0	0	0	0	0	0	0	1	2	0	0	2	0

ID: 15996518
name: AN-L-Sens-B2-ACp-11

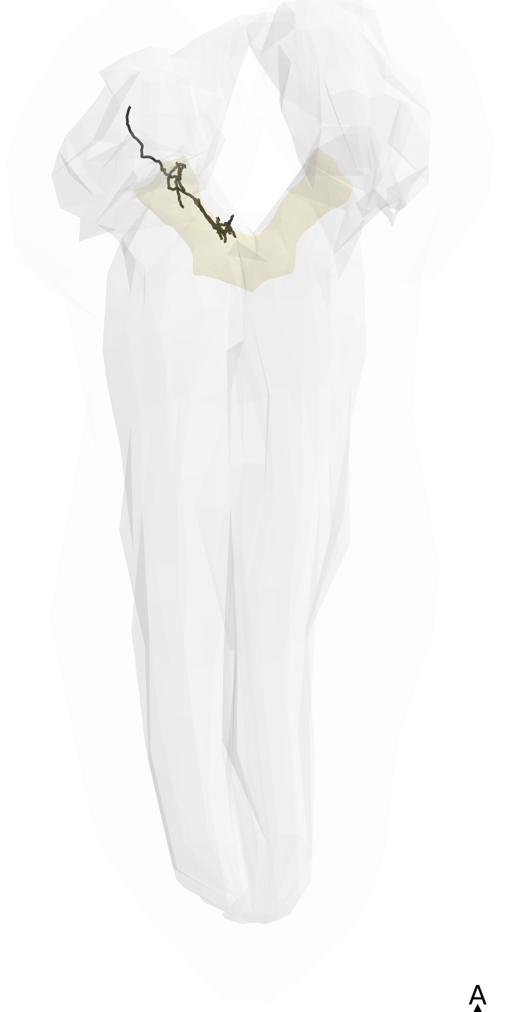

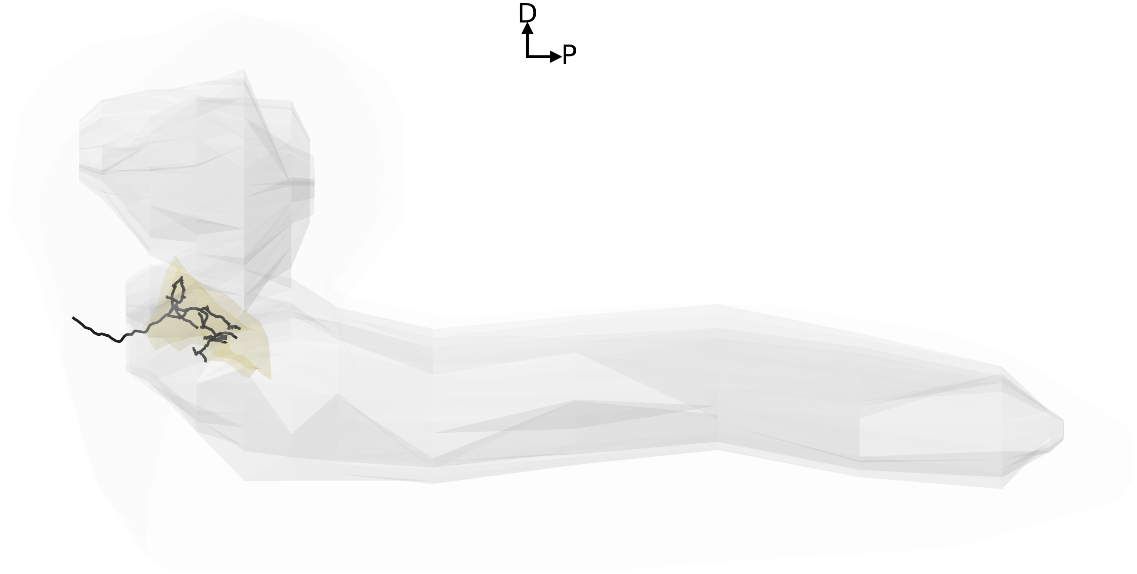

ID	name	SCACa	SCAVa	SCAVp	SCACal	SCACp	SCACpl	SCVM	IPCs	DMS	DH44	Se0ens	Se0ph	PMN LR	MN motor neurons	PaN motor neurons	olfactory PNs	gustatory PNs	multiglomerular PNs	unknown PNs	thermo PNs	visual PNs
15996518	AN-L-Sens-B2-ACp-11	0	0	0	0	7	0	0	0	0	0	0	0	0	0	0	0	1	0	0	0	0

ID: 15998817
name: AN-L-Sens-B2-ACp-12

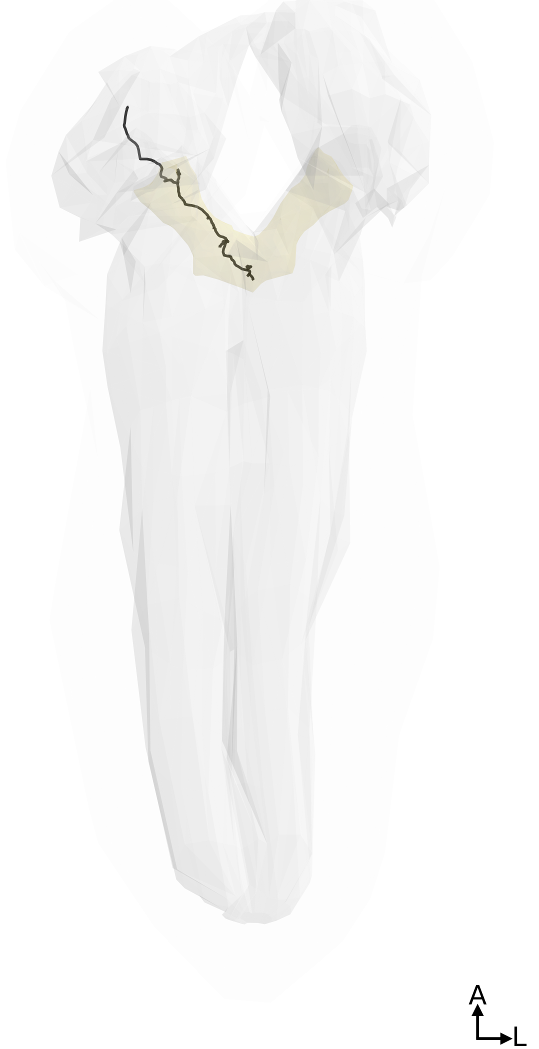

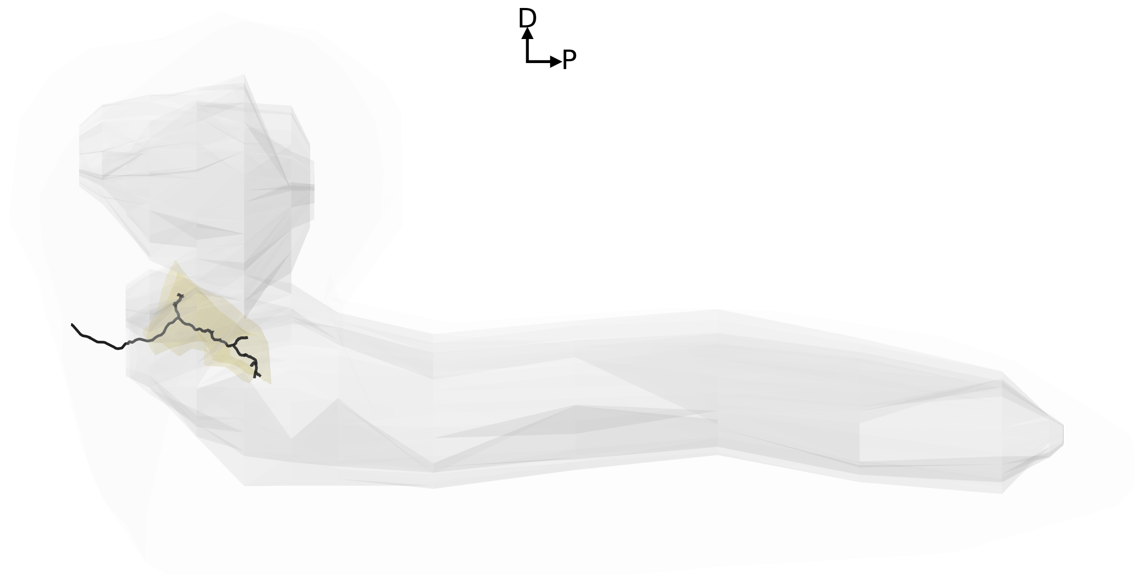

ID	*name*	SCACa	SCAVa	SCAVp	SCACal	SCACp	SCACpl	SCVM	IPCs	DMS	DH44	Se0ens	Se0ph	PMN LR	MN motor neurons	PaN motor neurons	olfactory PNs	gustatory PNs	multiglomerular PNs	unknown PNs	thermo PNs	visual PNs
15998817	AN-L-Sens-B2-ACp-12	0	0	0	0	6	0	0	0	0	0	0	0	0	0	0	0	1	0	0	0	0

ID: 15998100
name: AN-L-Sens-B2-ACp-13

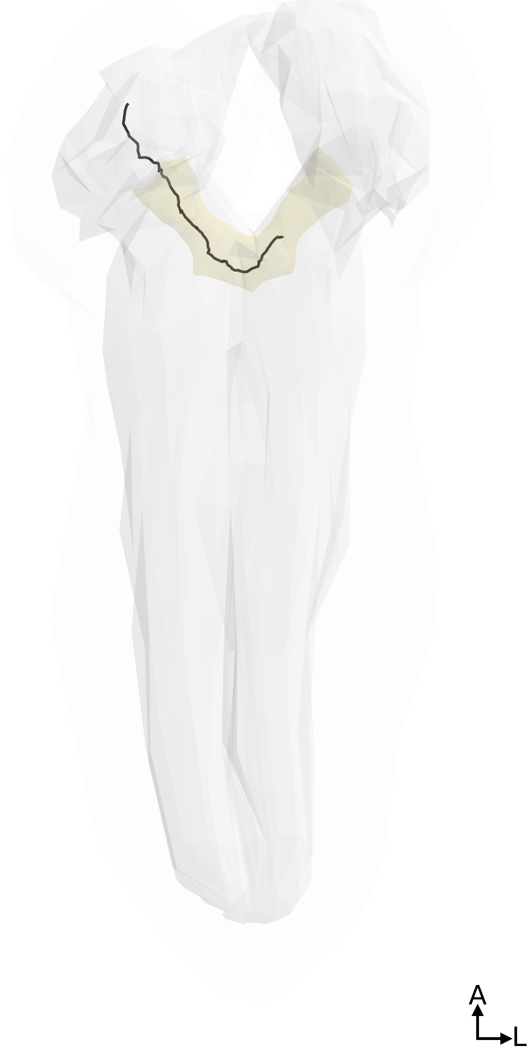

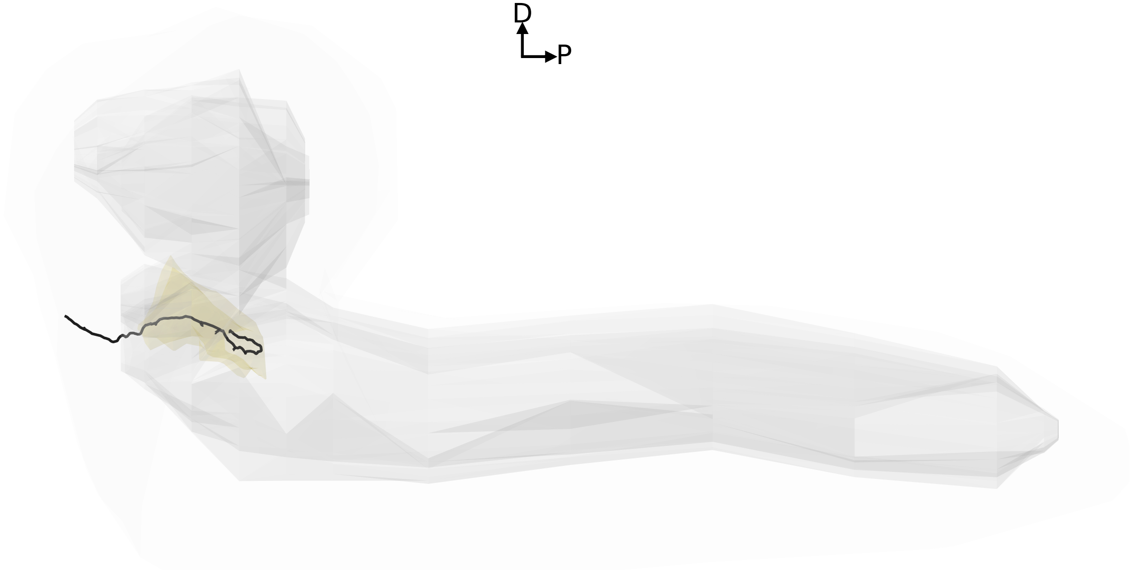

ID	*name*	SCACa	SCAVa	SCAVp	SCACal	SCACp	SCACpl	SCVM	IPCs	DMS	DH44	Se0ens	Se0ph	PMN LR	MN motor neurons	PaN motor neurons	olfactory PNs	gustatory PNs	multiglomerular PNs	unknown PNs	thermo PNs	visual PNs
15998100	AN-L-Sens-B2-ACp-13	0	0	0	0	3	0	0	0	0	0	0	0	0	0	0	0	3	0	0	0	0

ID: 15997645
name: AN-L-Sens-B2-ACp-14

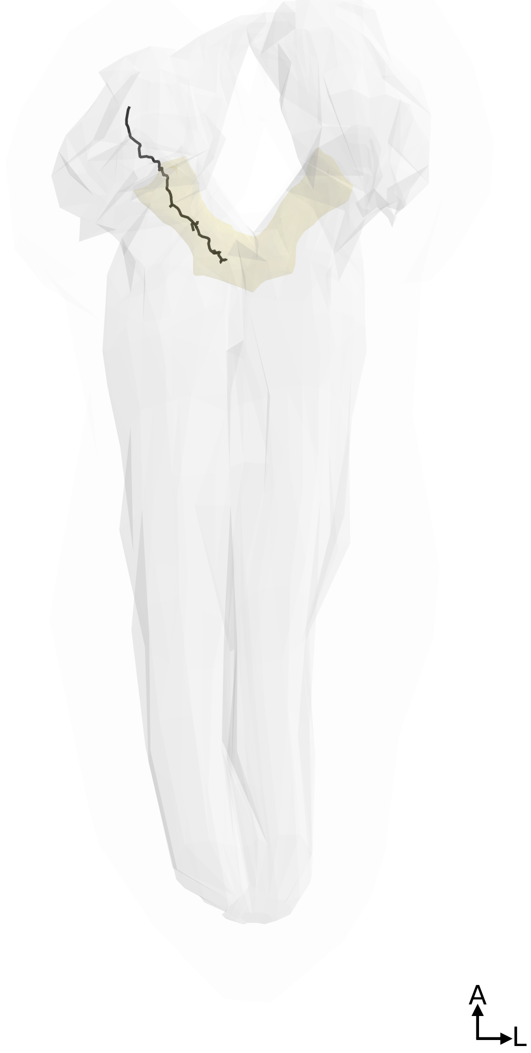

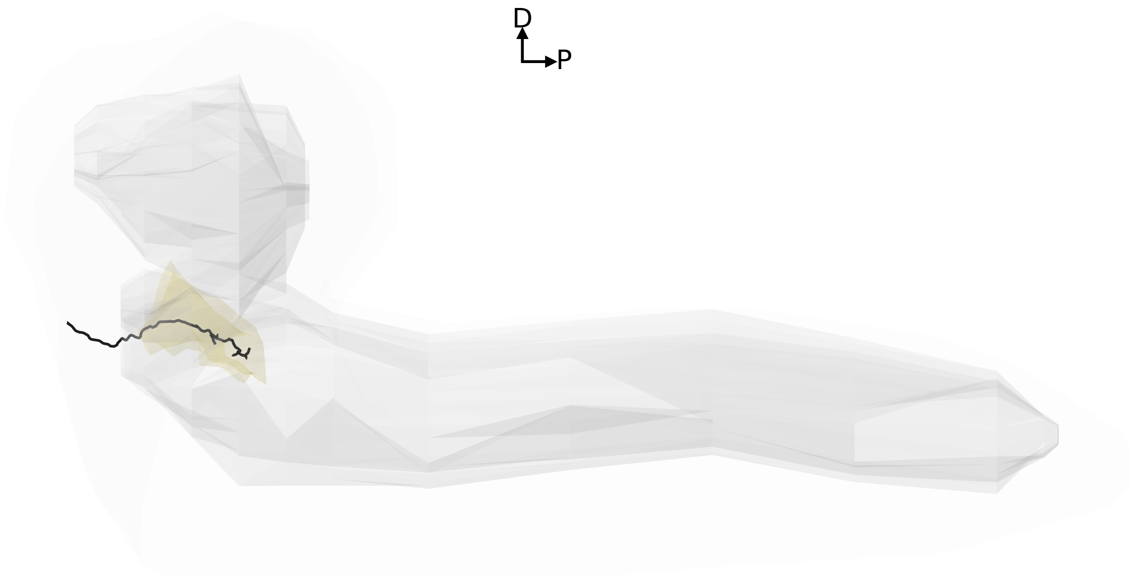

ID	name	SCACa	SCAVa	SCAVp	SCACal	SCACp	SCACpl	SCVM	IPCs	DMS	DH44	Se0ens	Se0ph	PMN LR	MN motor neurons	PaN motor neurons	olfactory PNs	gustatory PNs	multiglomerular PNs	unknown PNs	thermo PNs	visual PNs
15997645	AN-L-Sens-B2-ACp-14	0	0	0	0	6	0	0	0	0	0	0	0	0	0	0	0	7	0	0	0	0

ID: 15998261
name: AN-L-Sens-B2-ACp-15

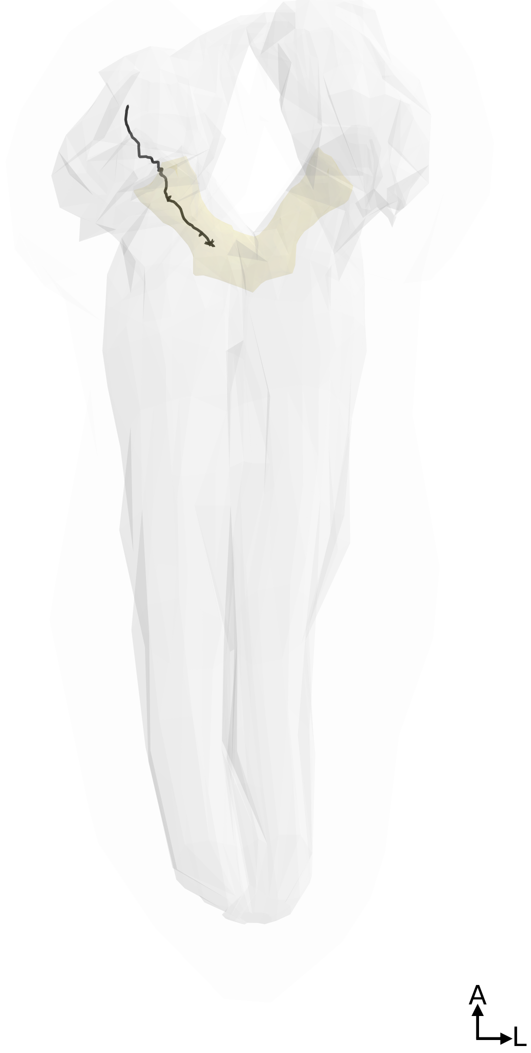

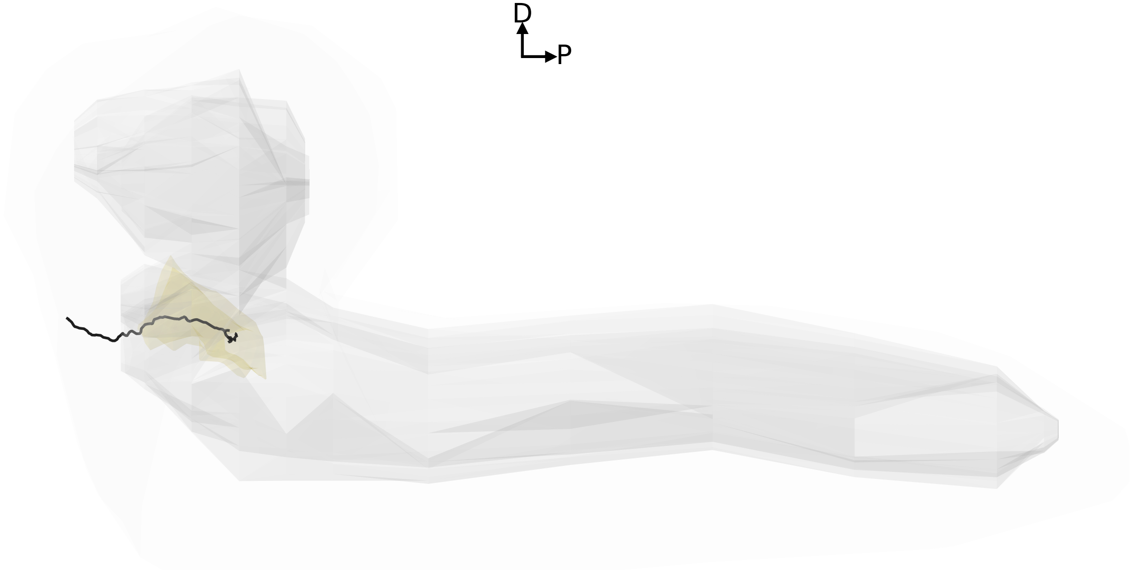

ID	name	SCACa	SCAVa	SCAVp	SCACal	SCACp	SCACpl	SCVM	IPCs	DMS	DH44	Se0ens	Se0ph	PMN LR	MN motor neurons	PaN motor neurons	olfactory PNs	gustatory PNs	multiglomerular PNs	unknown PNs	thermo PNs	visual PNs
15998261	AN-L-Sens-B2-ACp-15	0	0	0	0	0	0	0	0	0	0	0	0	0	0	0	1	0	0	0	0	0

ID: 15997807
name: AN-L-Sens-B2-ACp-16

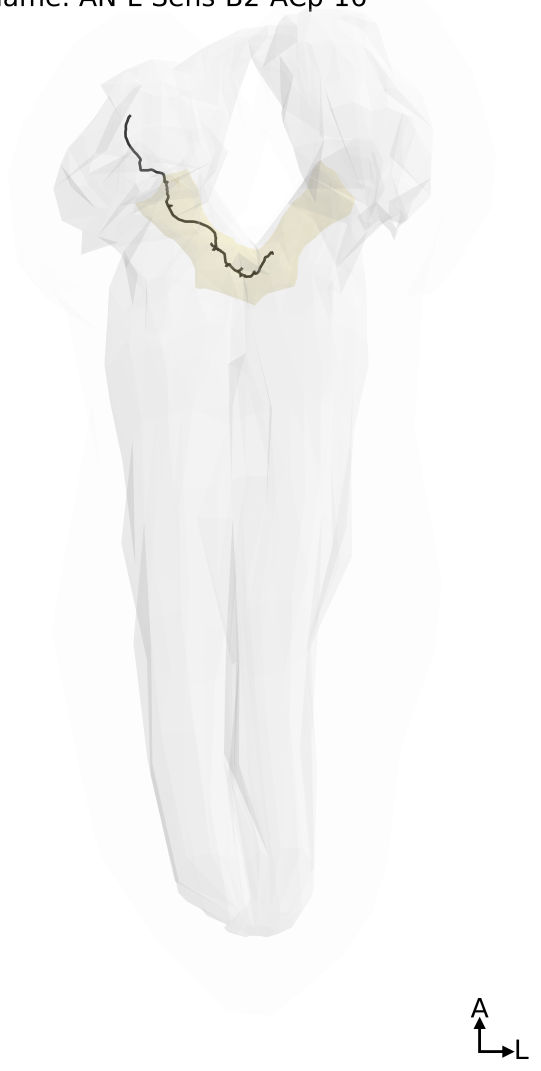

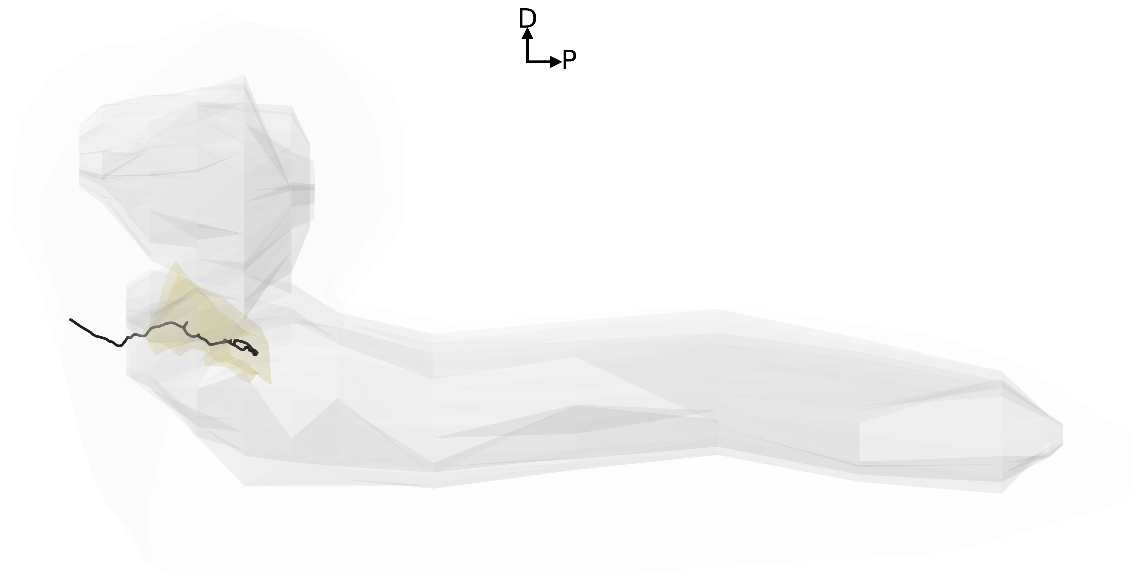

ID	*name*	SCACa	SCAVa	SCAVp	SCACal	SCACp	SCACpl	SCVM	IPCs	DMS	DH44	Se0ens	Se0ph	PMN LR	MN motor neurons	PaN motor neurons	olfactory PNs	gustatory PNs	multiglomerular PNs	unknown PNs	thermo PNs	visual PNs
15997807	AN-L-Sens-B2-ACp-16	0	0	0	0	2	0	0	0	0	0	0	0	0	0	0	0	1	0	0	0	0

ID: 1715990
name: AN-L-Sens-B2-ACp-17

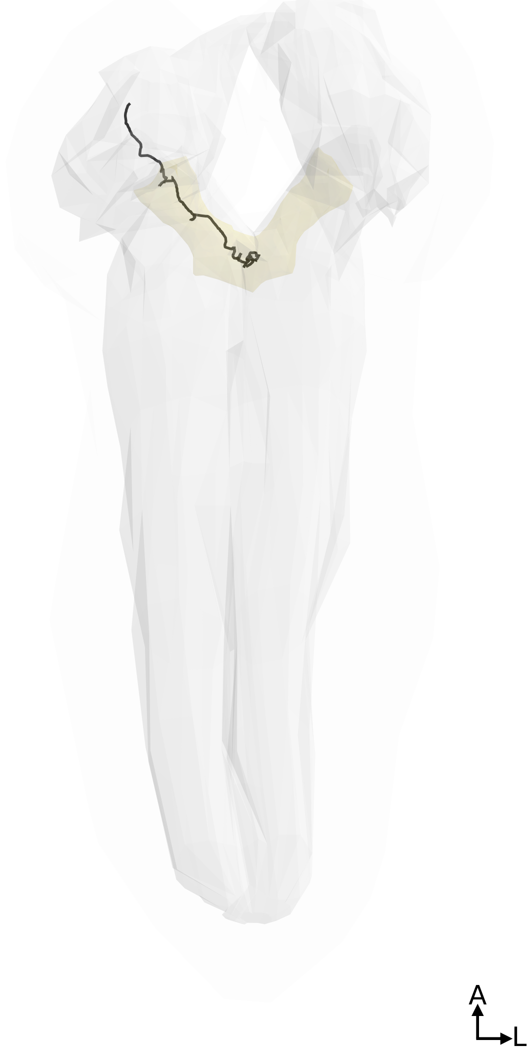

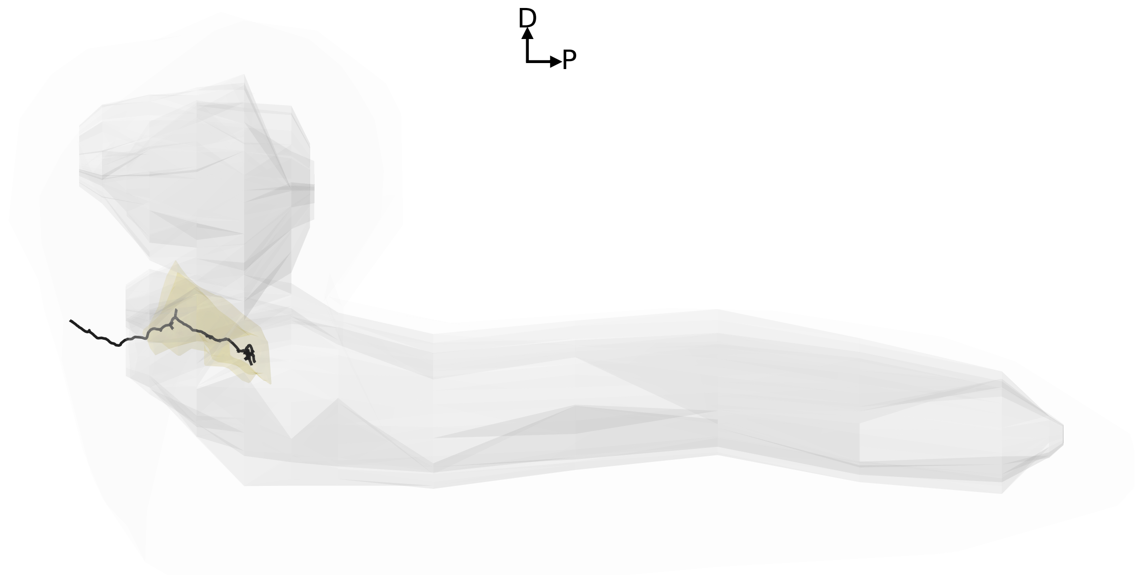

ID	*name*	SCACa	SCAVa	SCAVp	SCACal	SCACp	SCACpl	SCVM	IPCs	DMS	DH44	Se0ens	Se0ph	PMN LR	MN motor neurons	PaN motor neurons	olfactory PNs	gustatory PNs	multiglomerular PNs	unknown PNs	thermo PNs	visual PNs
1715990	AN-L-Sens-B2-ACp-17	0	0	0	0	2	0	0	0	0	0	0	0	0	0	0	1	0	0	0	0	0

ID: 15997692
name: AN-L-Sens-B2-ACp-18

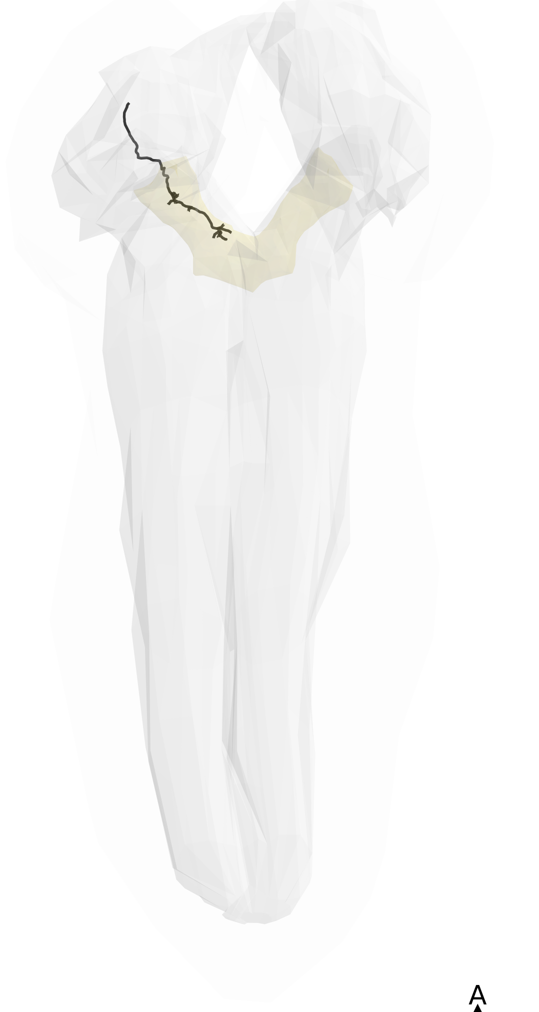

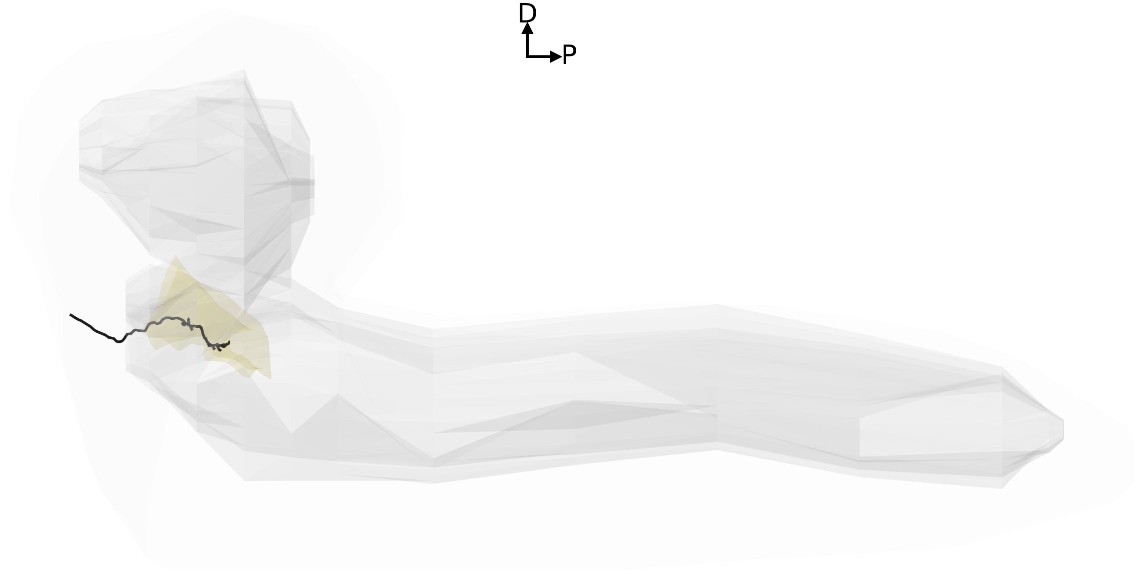

ID	name	SCACa	SCAVa	SCAVp	SCACal	SCACp	SCACpl	SCVM	IPCs	DMS	DH44	Se0ens	Se0ph	PMN LR	MN motor neurons	PaN motor neurons	olfactory PNs	gustatory PNs	multiglomerular PNs	unknown PNs	thermo PNs	visual PNs
15997692	AN-L-Sens-B2-ACp-18	0	0	0	0	4	0	0	0	0	0	0	0	0	0	0	0	0	0	0	0	0

ID: 1706716
name: AN-L-Sens-B2-ACp-19

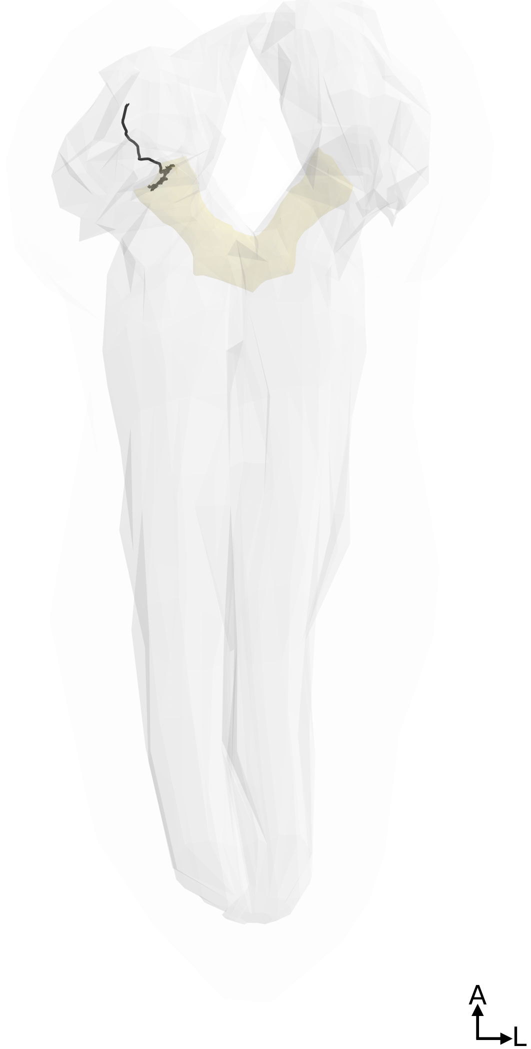

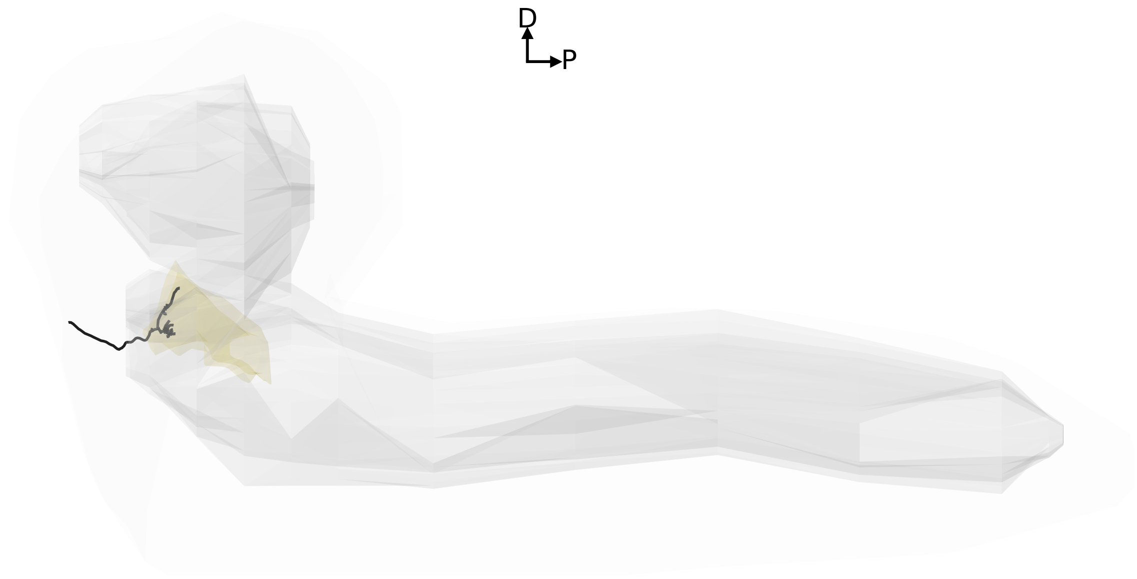

ID	name	SCACa	SCAVa	SCAVp	SCACal	SCACp	SCACpl	SCVM	IPCs	DMS	DH44	Se0ens	Se0ph	PMN LR	MN motor neurons	PaN motor neurons	olfactory PNs	gustatory PNs	multiglomerular PNs	unknown PNs	thermo PNs	visual PNs
1706716	AN-L-Sens-B2-ACp-19	0	0	0	0	1	0	0	0	0	0	0	0	0	0	0	0	0	0	0	0	0

ID: 15995929
name: AN-L-Sens-B2-ACp-20

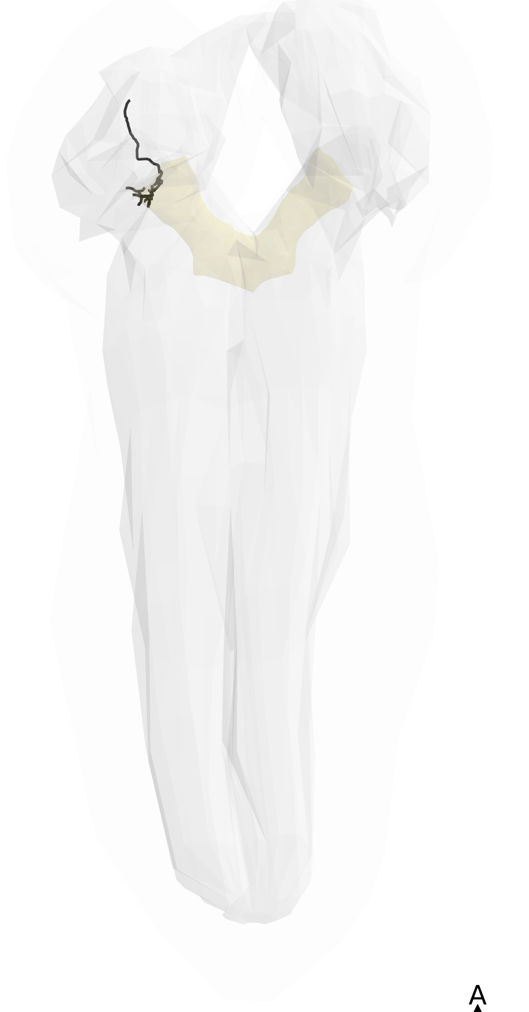

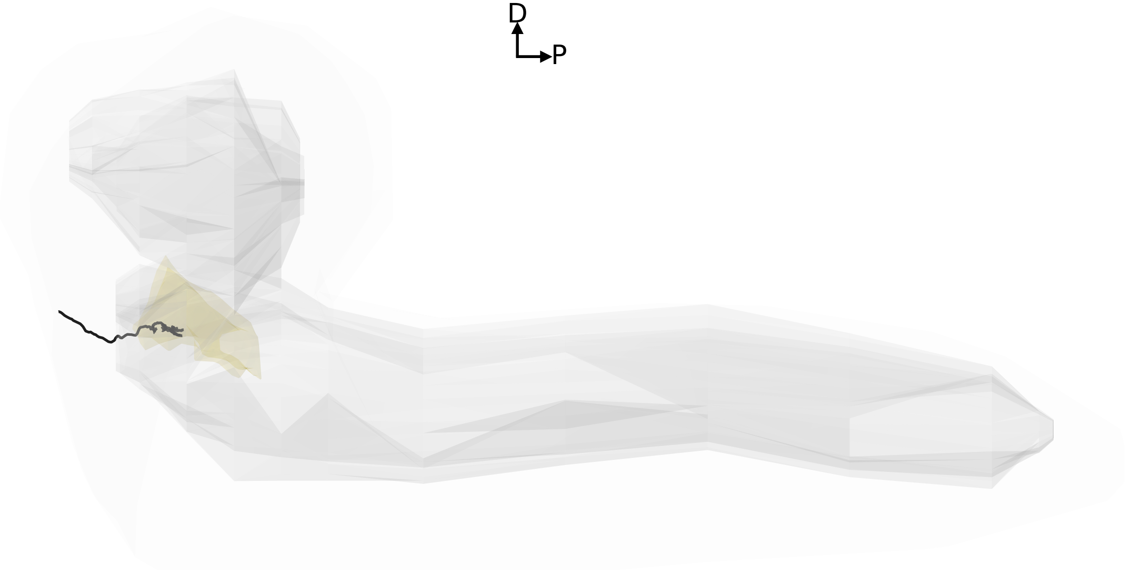

ID	*name*	SCACa	SCAVa	SCAVp	SCACal	SCACp	SCACpl	SCVM	IPCs	DMS	DH44	Se0ens	Se0ph	PMN LR	MN motor neurons	PaN motor neurons	olfactory PNs	gustatory PNs	multiglomerular PNs	unknown PNs	thermo PNs	visual PNs
15995929	AN-L-Sens-B2-ACp-20	0	0	0	0	1	0	0	0	0	0	0	0	0	0	0	0	0	0	0	0	0

ID: 11671564
name: AN-L-Sens-B2-ACp-21

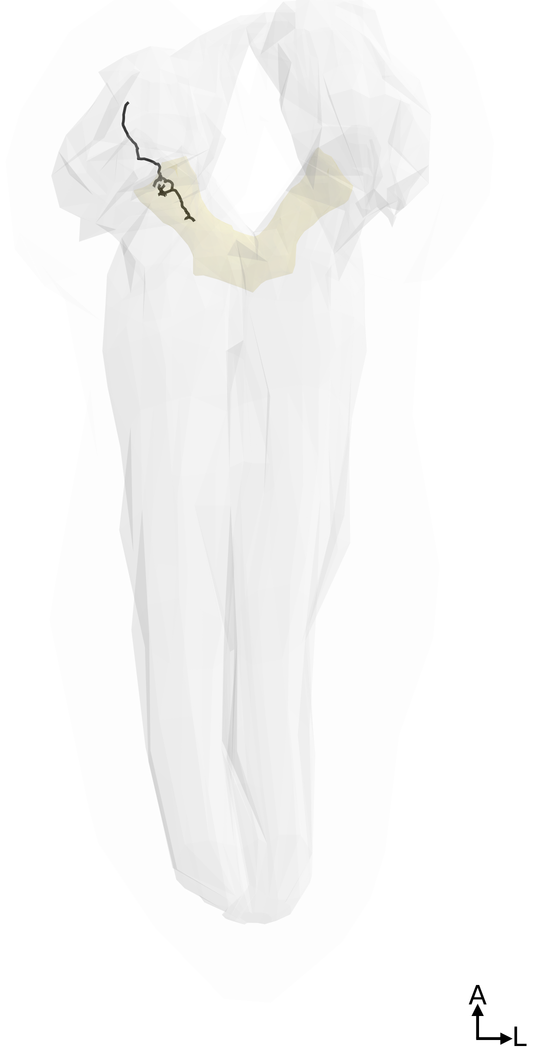

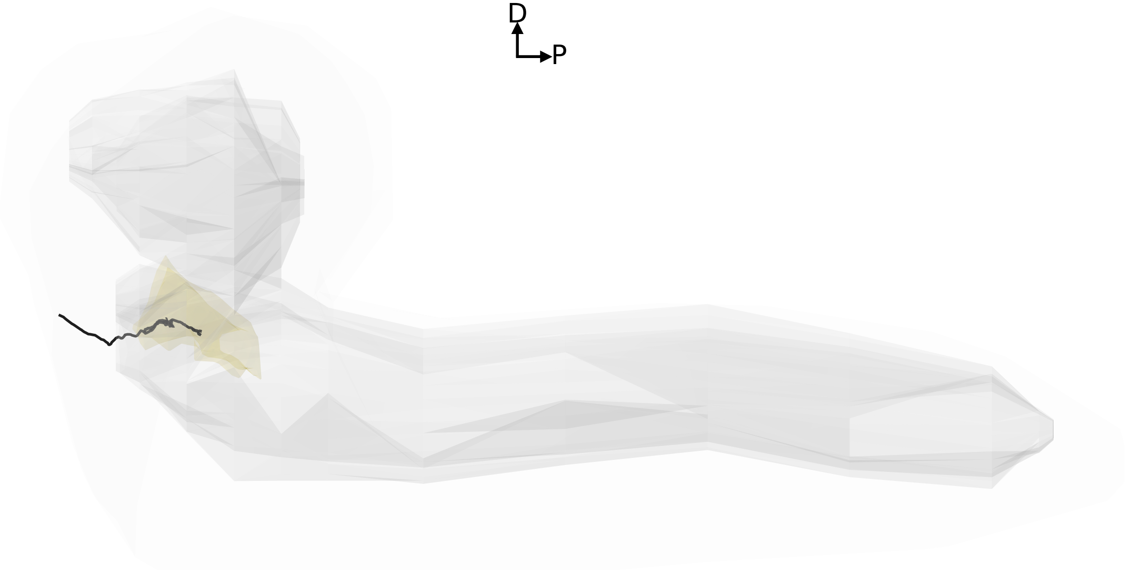

ID	*name*	SCACa	SCAVa	SCAVp	SCACal	SCACp	SCACpl	SCVM	IPCs	DMS	DH44	Se0ens	Se0ph	PMN LR	MN motor neurons	PaN motor neurons	olfactory PNs	gustatory PNs	multiglomerular PNs	unknown PNs	thermo PNs	visual PNs
11671564	AN-L-Sens-B2-ACp-21	0	0	0	0	1	0	0	0	0	0	0	0	0	0	0	11	9	1	0	0	0

ID: 15995404
name: AN-L-Sens-B2-ACp-22

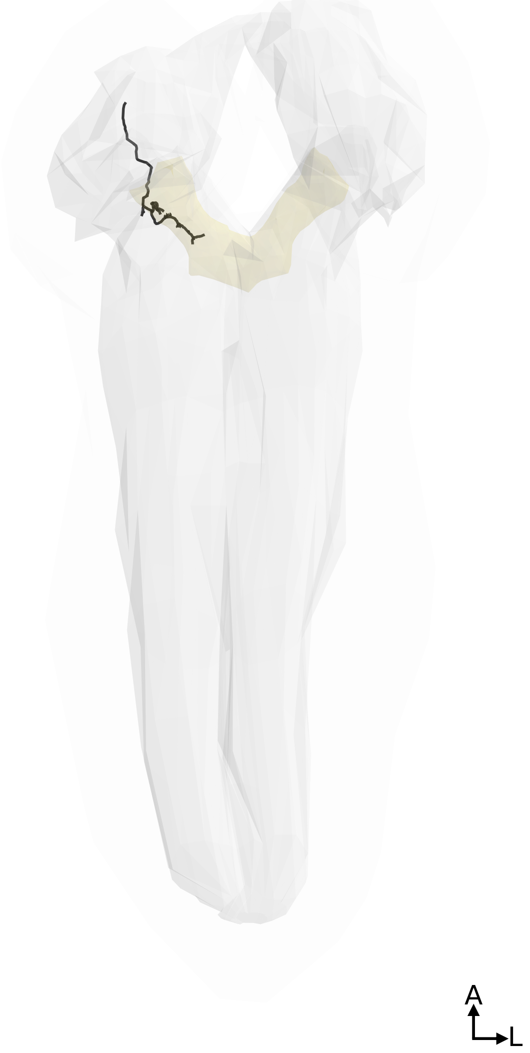

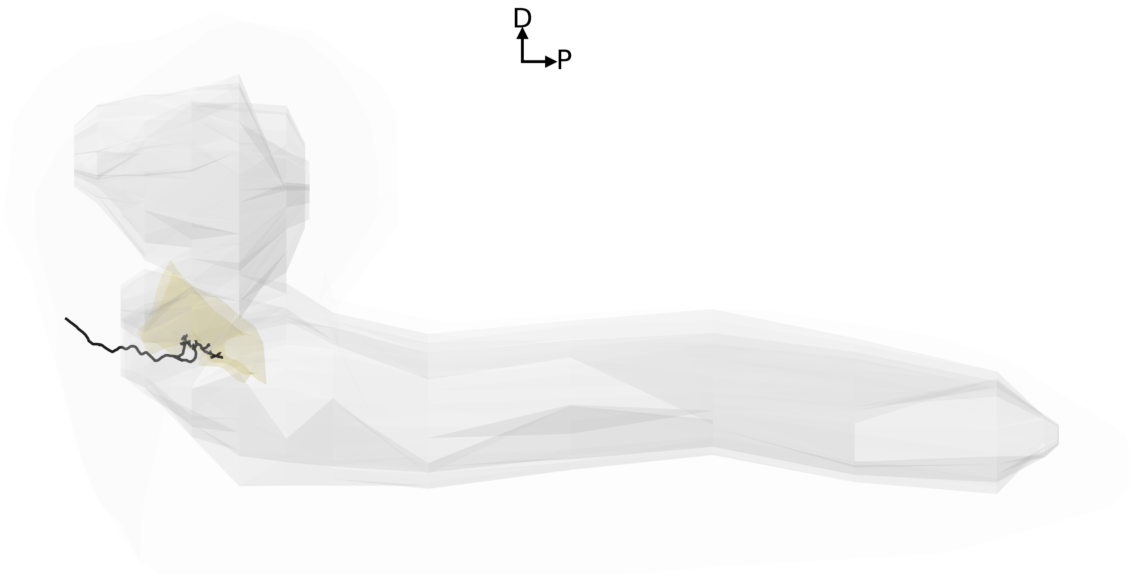

ID	*name*	SCACa	SCAVa	SCAVp	SCACal	SCACp	SCACpl	SCVM	IPCs	DMS	DH44	Se0ens	Se0ph	PMN LR	MN motor neurons	PaN motor neurons	olfactory PNs	gustatory PNs	multiglomerular PNs	unknown PNs	thermo PNs	visual PNs
15995404	AN-L-Sens-B2-ACp-22	0	0	0	0	0	0	0	0	0	0	0	0	0	0	0	0	0	1	0	0	0

ID: 15997929
name: AN-L-Sens-B2-ACp-23

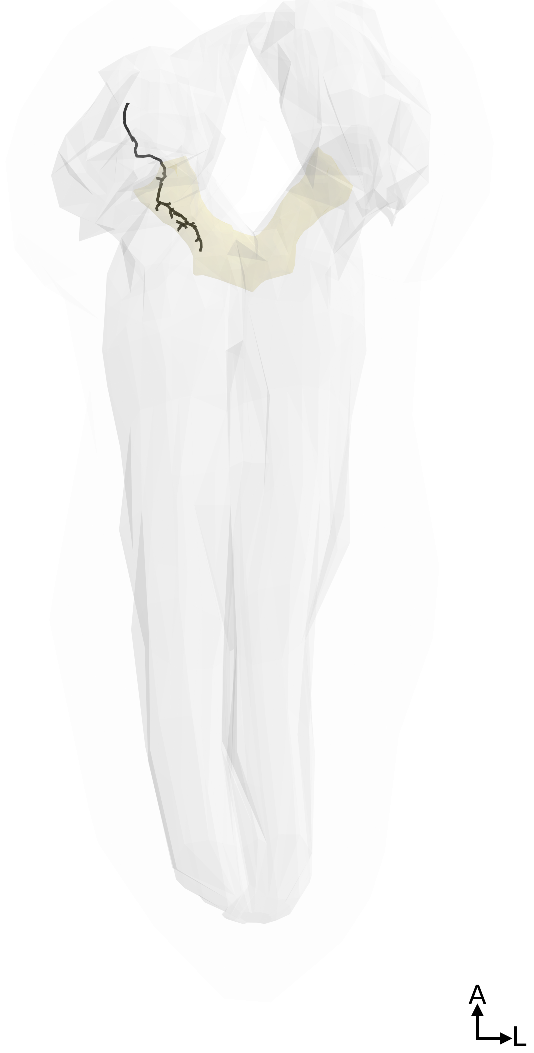

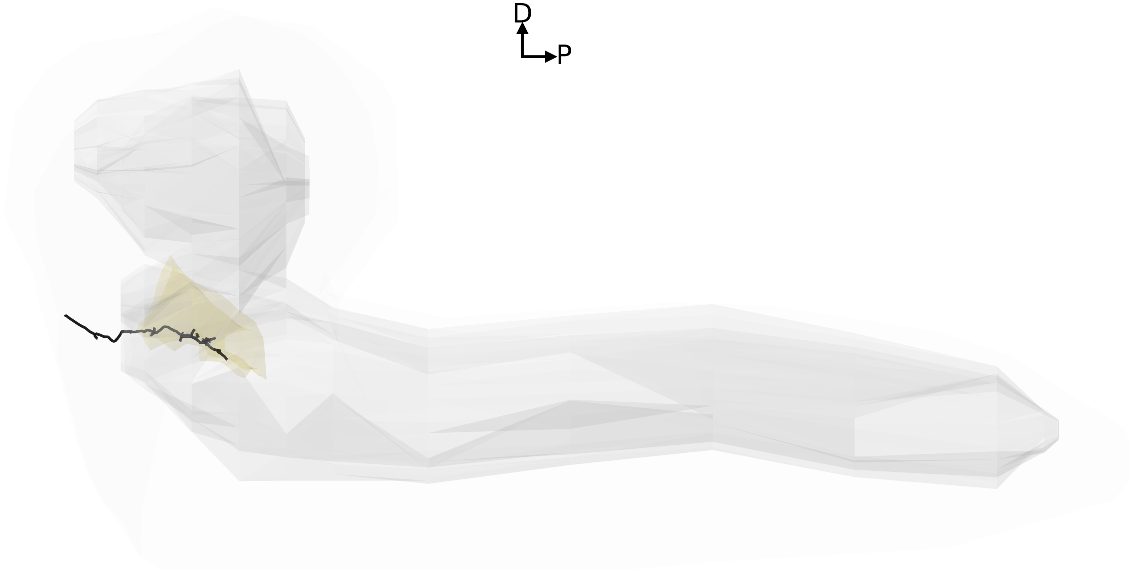

ID	name	SCACa	SCAVa	SCAVp	SCACal	SCACp	SCACpl	SCVM	IPCs	DMS	DH44	Se0ens	Se0ph	PMN LR	MN motor neurons	PaN motor neurons	olfactory PNs	gustatory PNs	multiglomerular PNs	unknown PNs	thermo PNs	visual PNs
15997929	AN-L-Sens-B2-ACp-23	0	0	0	0	0	0	0	0	0	0	0	0	0	0	0	0	0	0	0	0	0

ID: 3652587
name: AN-L-Sens-B3-ACp-08

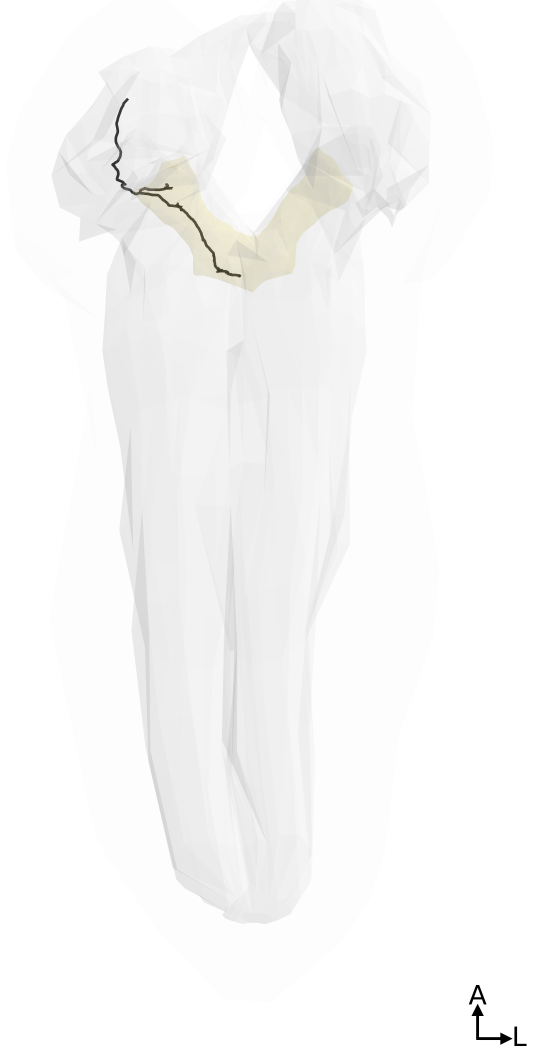

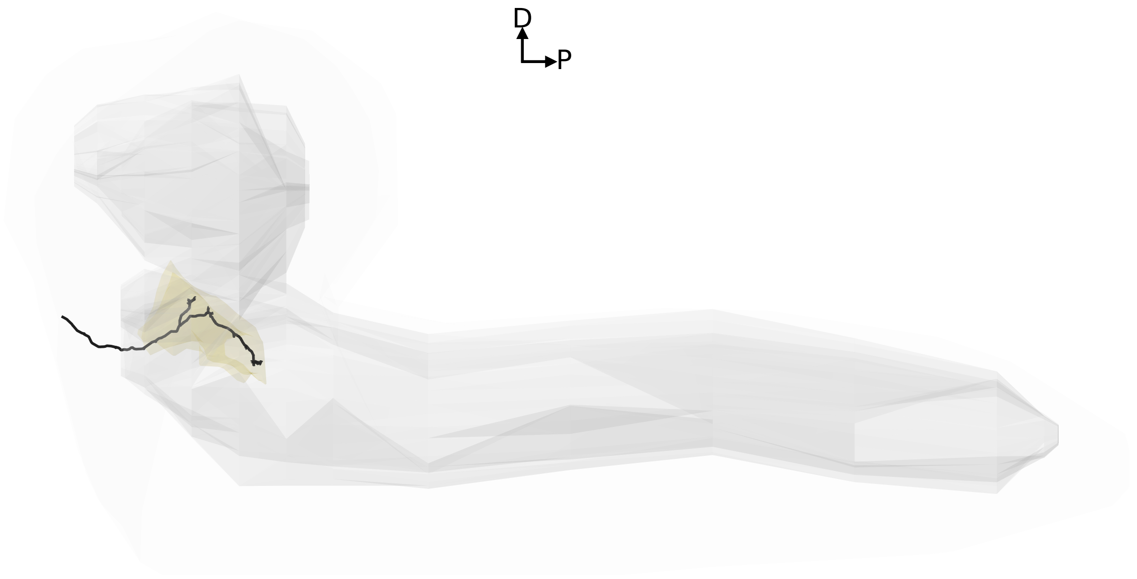

ID	*name*	SCACa	SCAVa	SCAVp	SCACal	SCACp	SCACpl	SCVM	IPCs	DMS	DH44	Se0ens	Se0ph	PMN LR	MN motor neurons	PaN motor neurons	olfactory PNs	gustatory PNs	multiglomerular PNs	unknown PNs	thermo PNs	visual PNs
3652587	AN-L-Sens-B3-ACp-08	0	0	0	0	1	0	0	0	0	0	0	0	0	0	0	0	0	2	0	0	0

ID: 5186461
name: AN-L-Sens-B3-ACp-09

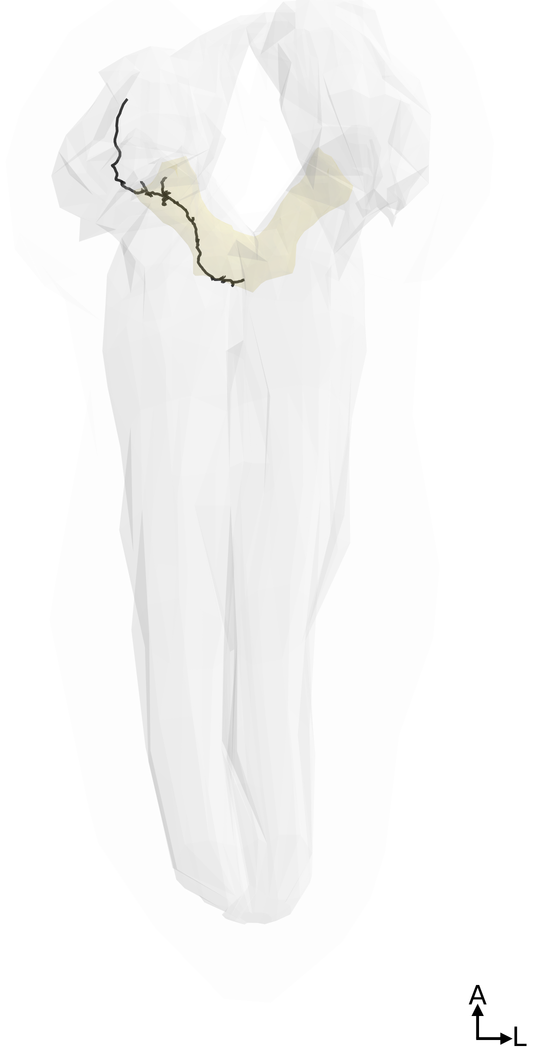

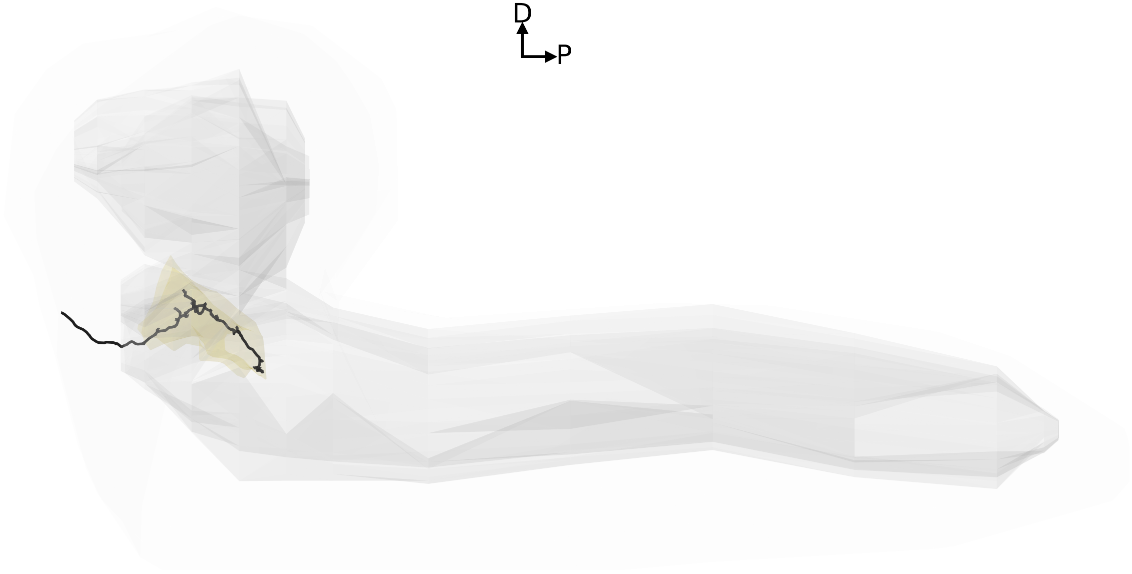

ID	*name*	SCACa	SCAVa	SCAVp	SCACal	SCACp	SCACpl	SCVM	IPCs	DMS	DH44	Se0ens	Se0ph	PMN LR	MN motor neurons	PaN motor neurons	olfactory PNs	gustatory PNs	multiglomerular PNs	unknown PNs	thermo PNs	visual PNs
5186461	AN-L-Sens-B3-ACp-09	0	0	0	1	3	0	0	0	0	0	0	0	0	0	0	12	0	0	0	0	0

ID: 15508735
name: AN-R-Sens-B2-ACp-01

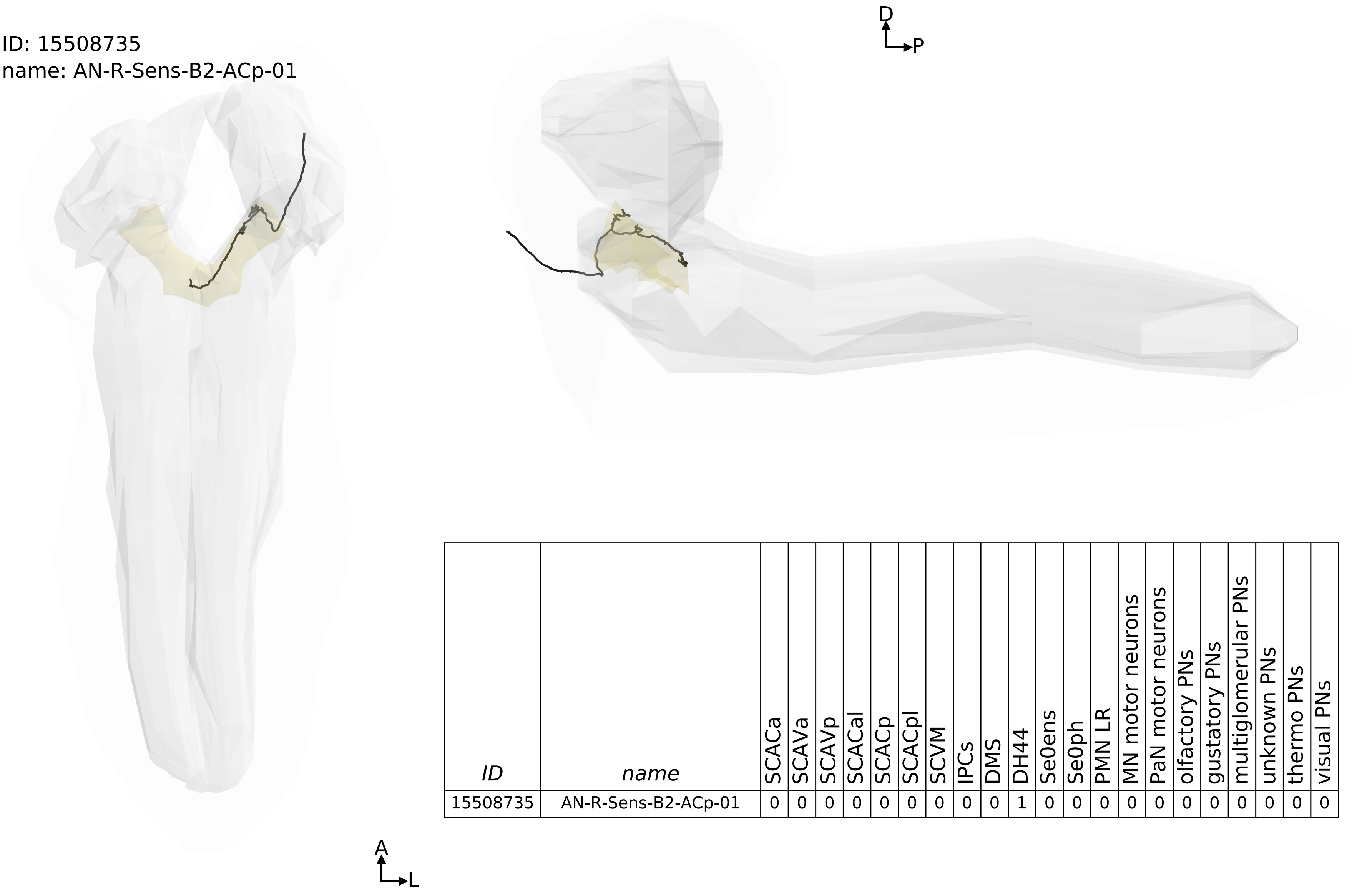

ID	*name*	SCACa	SCAVa	SCAVp	SCACal	SCACp	SCACpl	SCVM	IPCs	DMS	DH44	Se0ens	Se0ph	PMN LR	MN motor neurons	PaN motor neurons	olfactory PNs	gustatory PNs	multiglomerular PNs	unknown PNs	thermo PNs	visual PNs
15508735	AN-R-Sens-B2-ACp-01	0	0	0	0	0	0	0	0	0	1	0	0	0	0	0	0	0	0	0	0	0

ID: 15541944
name: AN-R-Sens-B2-ACp-02

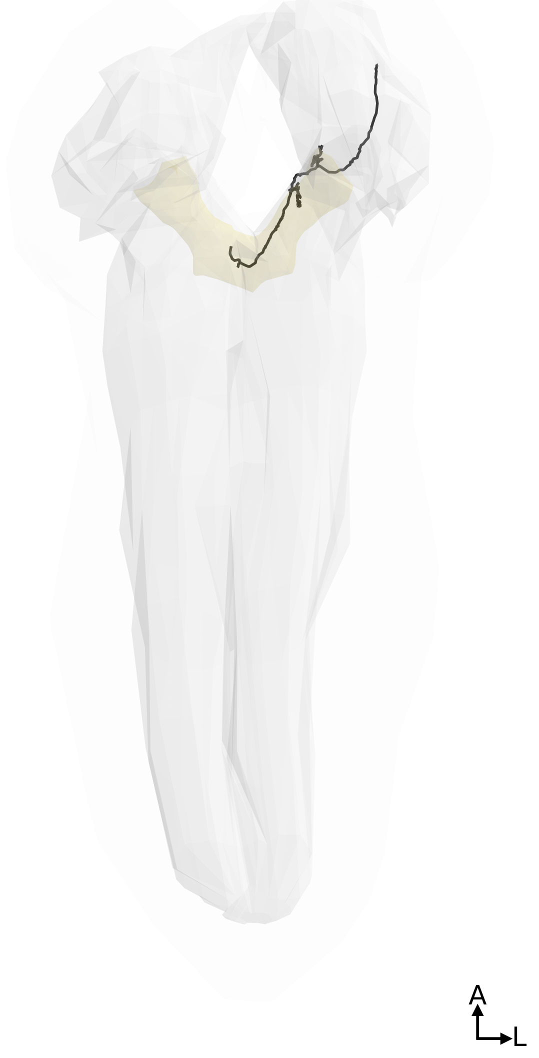

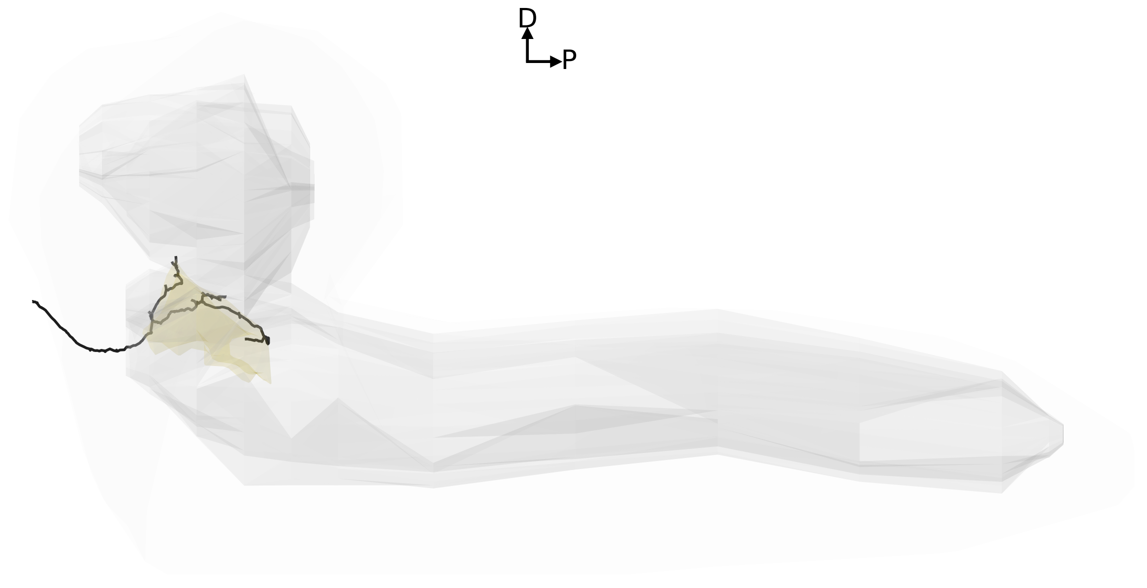

ID	name	SCACa	SCAVa	SCAVp	SCACal	SCACp	SCACpl	SCVM	IPCs	DMS	DH44	Se0ens	Se0ph	PMN LR	MN motor neurons	PaN motor neurons	olfactory PNs	gustatory PNs	multiglomerular PNs	unknown PNs	thermo PNs	visual PNs
15541944	AN-R-Sens-B2-ACp-02	0	0	0	0	1	0	0	0	0	2	0	0	0	0	0	0	0	0	0	0	0

ID: 15531033
name: AN-R-Sens-B2-ACp-03

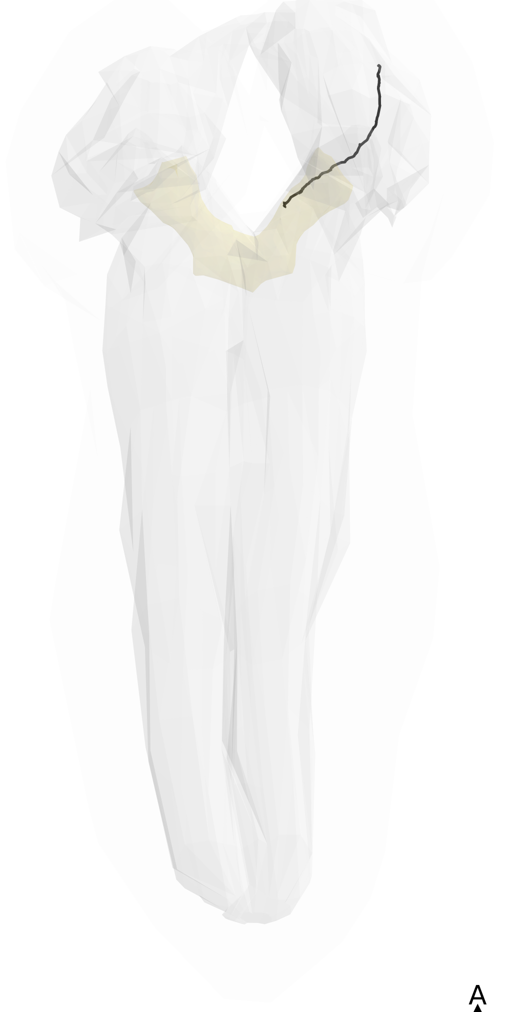

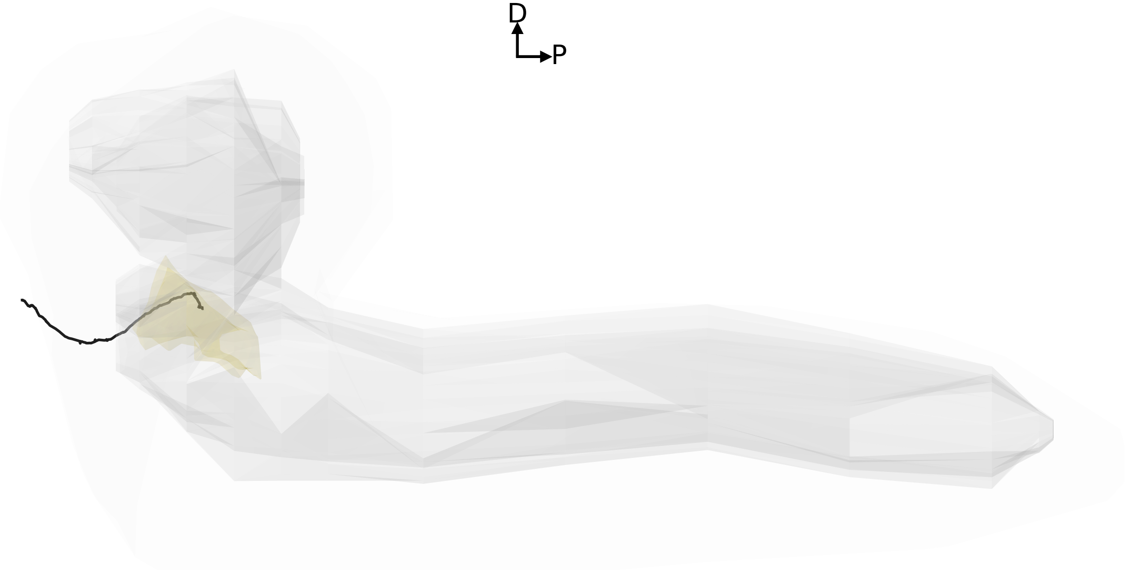

ID	*name*	SCACa	SCAVa	SCAVp	SCACal	SCACp	SCACpl	SCVM	IPCs	DMS	DH44	Se0ens	Se0ph	PMN LR	MN motor neurons	PaN motor neurons	olfactory PNs	gustatory PNs	multiglomerular PNs	unknown PNs	thermo PNs	visual PNs
15531033	AN-R-Sens-B2-ACp-03	0	0	0	0	5	0	0	0	0	0	0	0	0	0	0	0	1	0	0	0	0

ID: 15531916
name: AN-R-Sens-B2-ACp-04

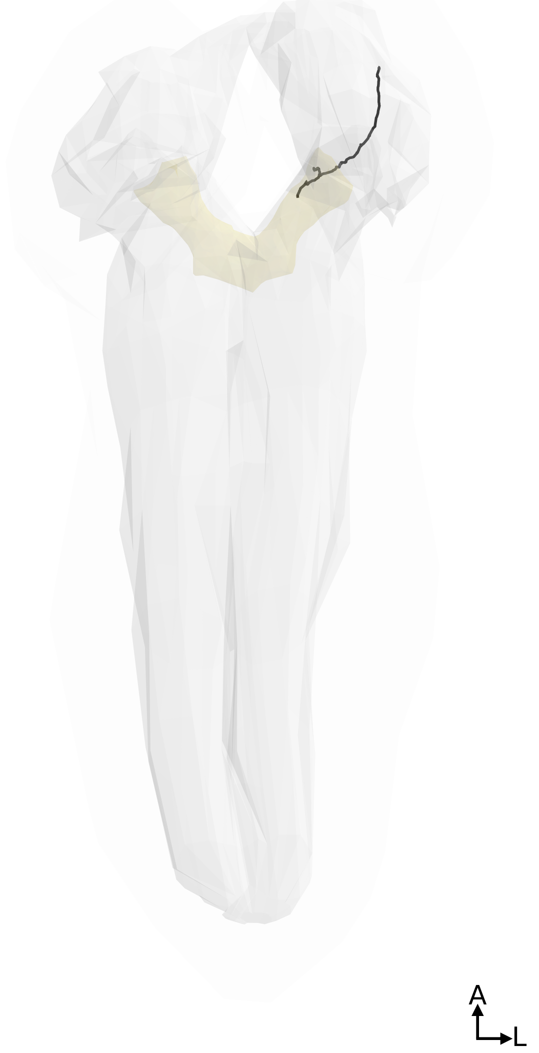

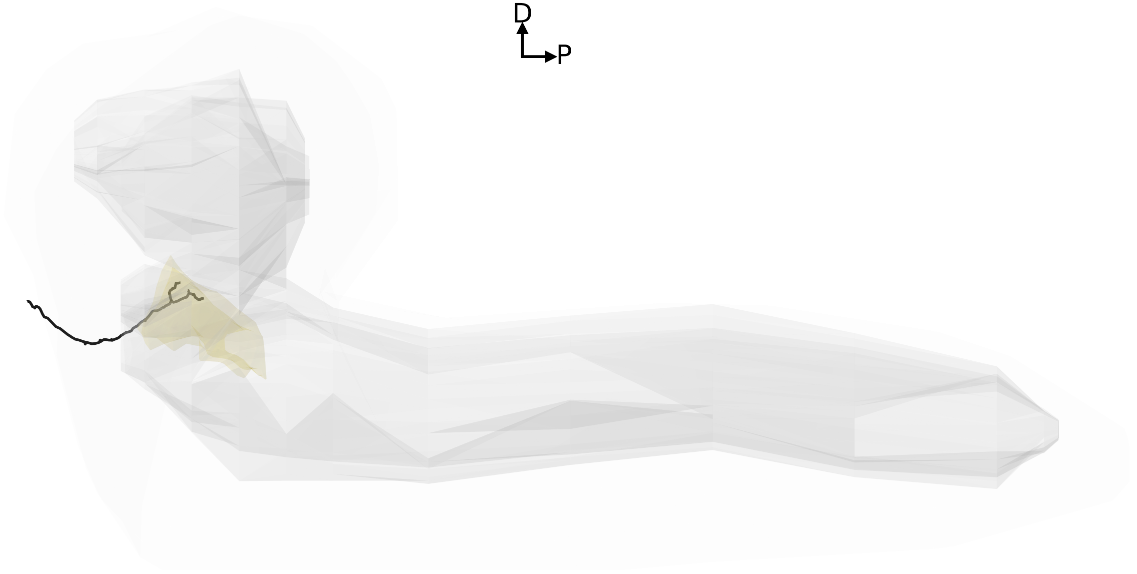

ID	*name*	SCACa	SCAVa	SCAVp	SCACal	SCACp	SCACpl	SCVM	IPCs	DMS	DH44	Se0ens	Se0ph	PMN LR	MN motor neurons	PaN motor neurons	olfactory PNs	gustatory PNs	multiglomerular PNs	unknown PNs	thermo PNs	visual PNs
15531916	AN-R-Sens-B2-ACp-04	0	0	0	0	0	0	0	0	0	0	0	0	0	0	0	0	0	0	0	0	0

ID: 16341507
name: AN-R-Sens-B2-ACp-05

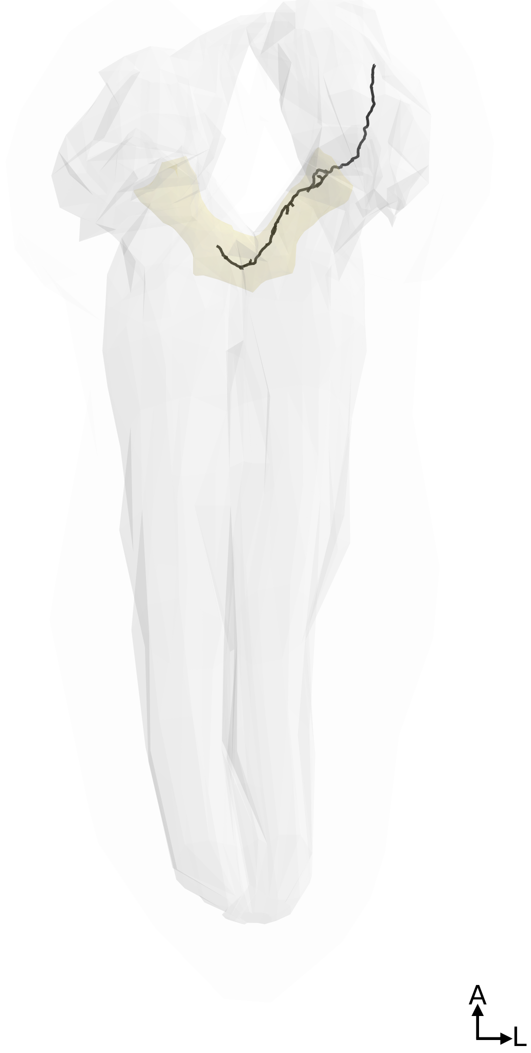

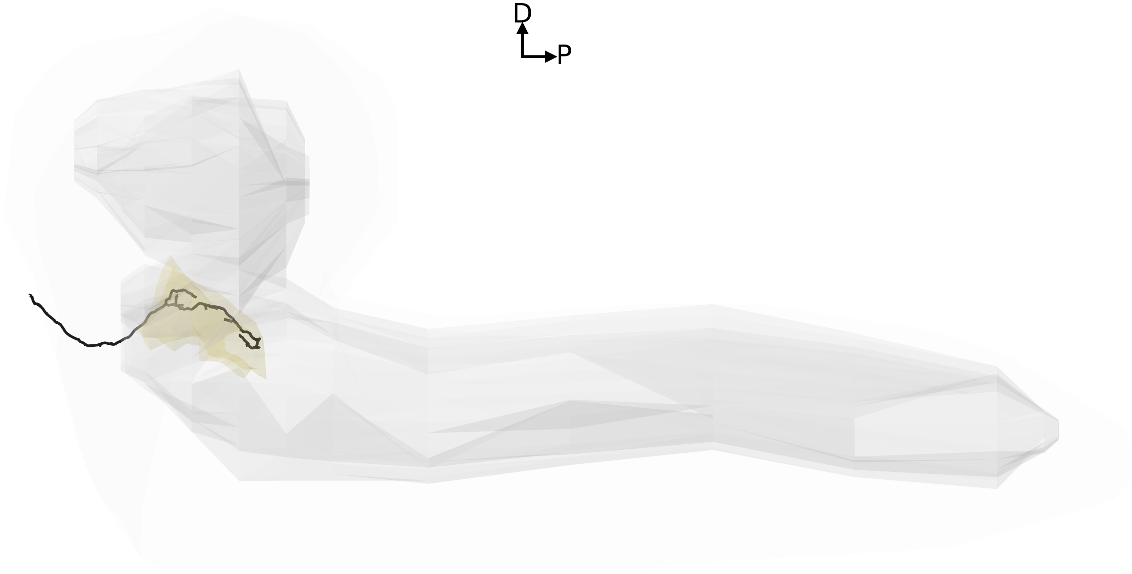

ID	*name*	SCACa	SCAVa	SCAVp	SCACal	SCACp	SCACpl	SCVM	IPCs	DMS	DH44	Se0ens	Se0ph	PMN LR	MN motor neurons	PaN motor neurons	olfactory PNs	gustatory PNs	multiglomerular PNs	unknown PNs	thermo PNs	visual PNs
16341507	AN-R-Sens-B2-ACp-05	0	0	0	0	6	0	0	0	0	0	0	0	0	0	0	0	0	0	0	0	0

ID: 15434200
name: AN-R-Sens-B2-ACp-06

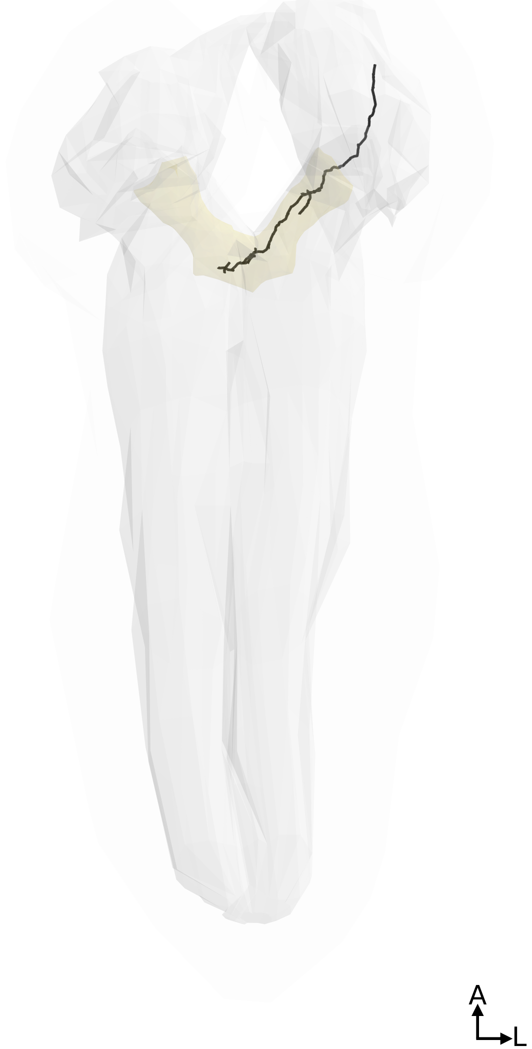

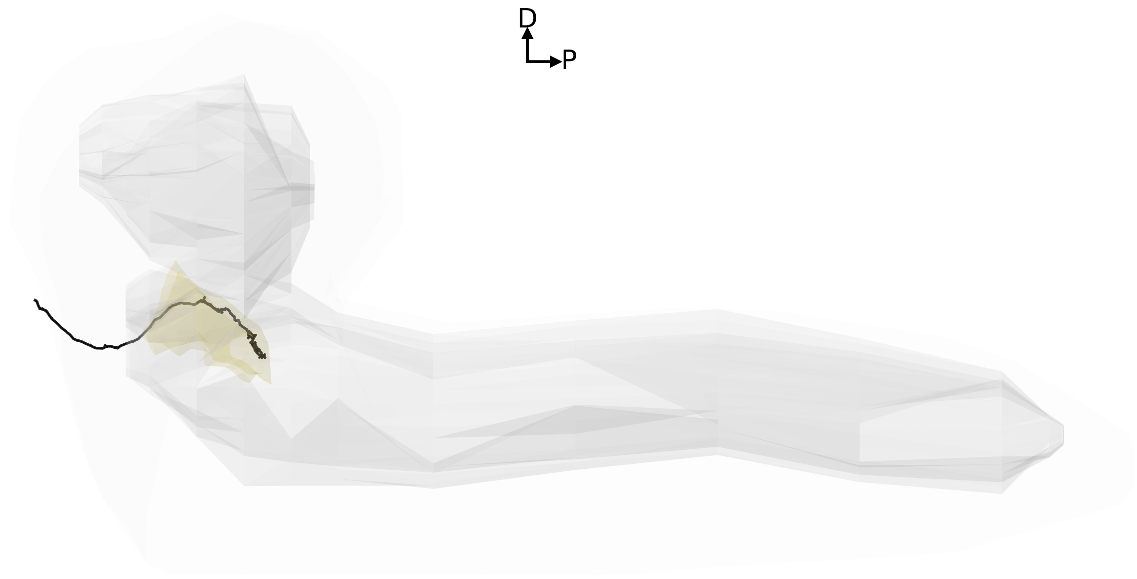

ID	name	SCACa	SCAVa	SCAVp	SCACal	SCACp	SCACpl	SCVM	IPCs	DMS	DH44	Se0ens	Se0ph	PMN LR	MN motor neurons	PaN motor neurons	olfactory PNs	gustatory PNs	multiglomerular PNs	unknown PNs	thermo PNs	visual PNs
15434200	AN-R-Sens-B2-ACp-06	0	0	0	0	4	0	0	0	0	0	0	0	0	0	0	2	1	0	0	1	0

ID: 16341966
name: AN-R-Sens-B2-ACp-07

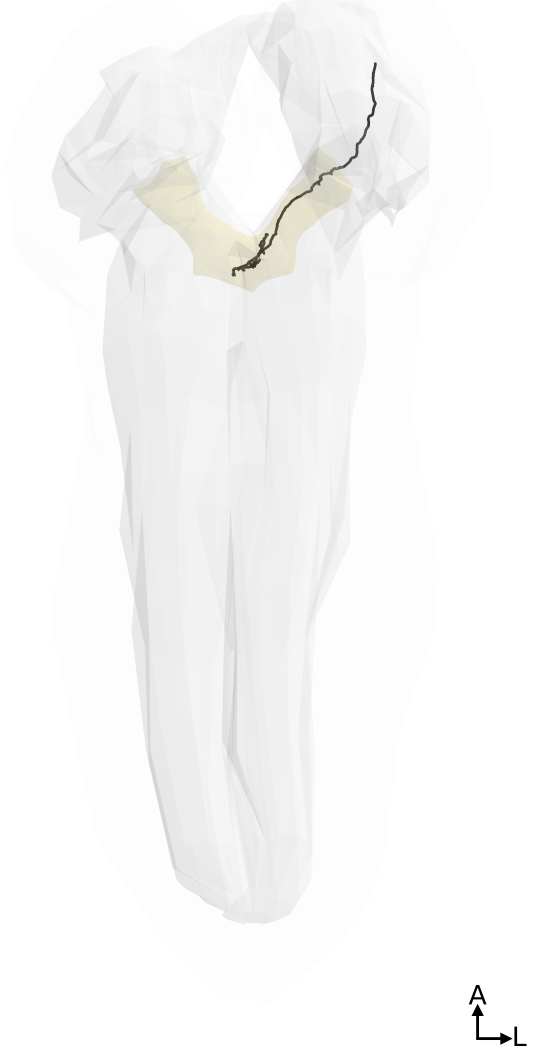

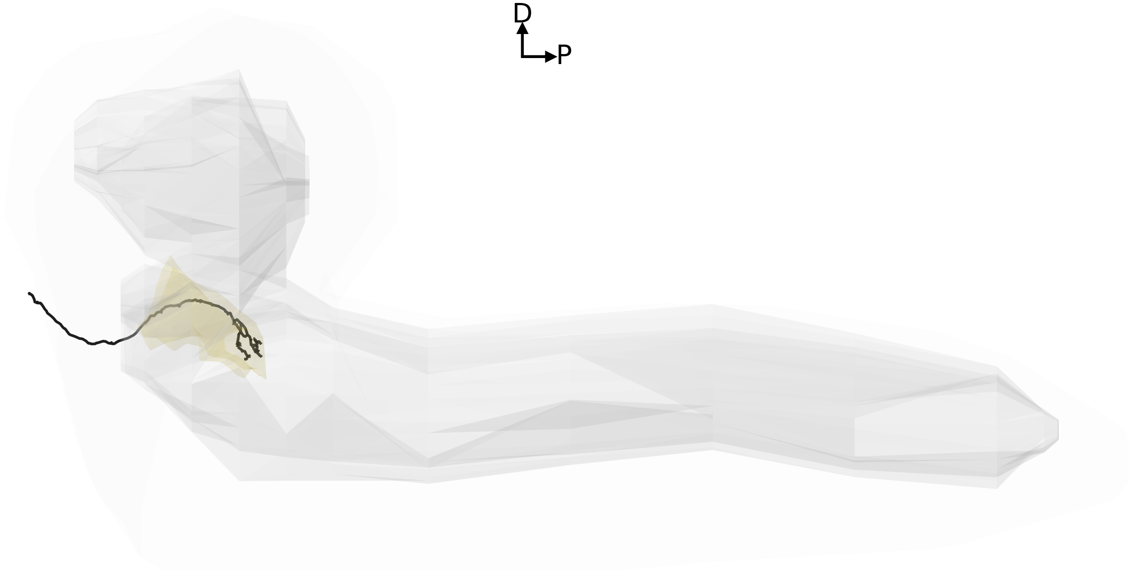

ID	*name*	SCACa	SCAVa	SCAVp	SCACal	SCACp	SCACpl	SCVM	IPCs	DMS	DH44	Se0ens	Se0ph	PMN LR	MN motor neurons	PaN motor neurons	olfactory PNs	gustatory PNs	multiglomerular PNs	unknown PNs	thermo PNs	visual PNs
16341966	AN-R-Sens-B2-ACp-07	0	0	0	0	2	0	0	0	0	0	0	0	0	0	0	0	0	0	0	0	0

ID: 15409328
name: AN-R-Sens-B2-ACp-08

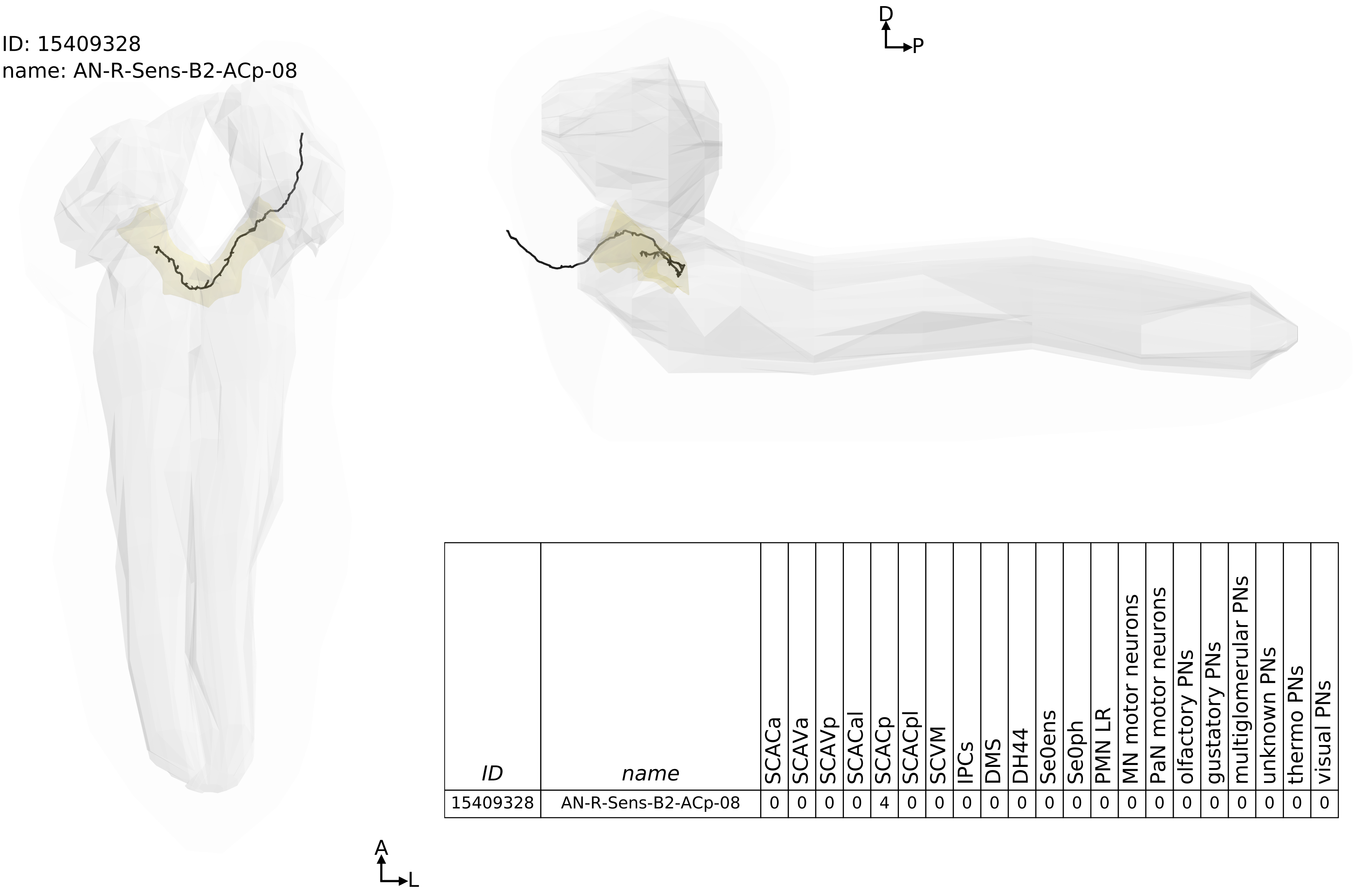

ID	name	SCACa	SCAVa	SCAVp	SCACal	SCACp	SCACpl	SCVM	IPCs	DMS	DH44	Se0ens	Se0ph	PMN LR	MN motor neurons	PaN motor neurons	olfactory PNs	gustatory PNs	multiglomerular PNs	unknown PNs	thermo PNs	visual PNs
15409328	AN-R-Sens-B2-ACp-08	0	0	0	0	4	0	0	0	0	0	0	0	0	0	0	0	0	0	0	0	0

ID: 15451084
name: AN-R-Sens-B2-ACp-09

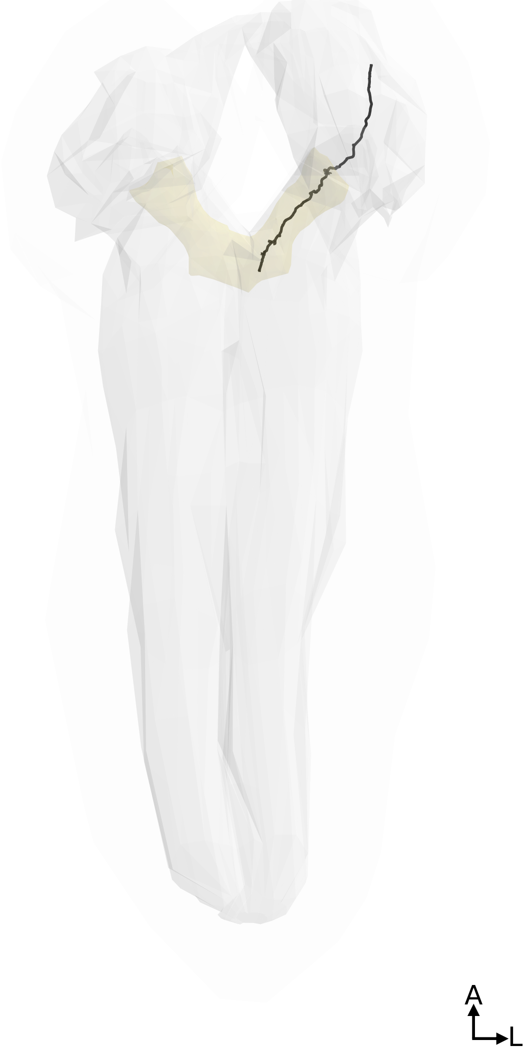

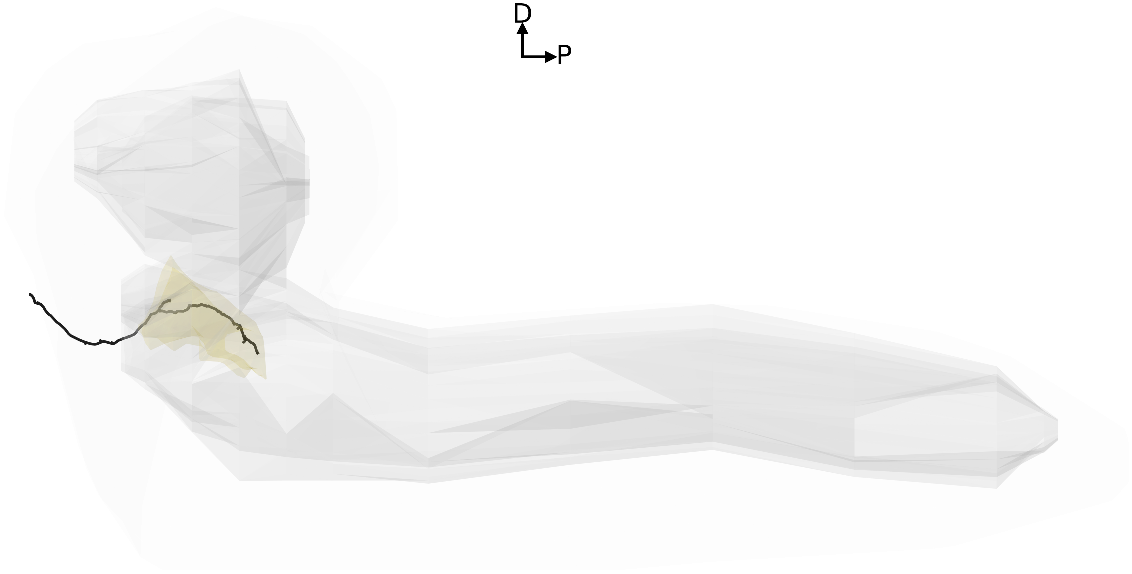

ID	*name*	SCACa	SCAVa	SCAVp	SCACal	SCACp	SCACpl	SCVM	IPCs	DMS	DH44	Se0ens	Se0ph	PMN LR	MN motor neurons	PaN motor neurons	olfactory PNs	gustatory PNs	multiglomerular PNs	unknown PNs	thermo PNs	visual PNs
15451084	AN-R-Sens-B2-ACp-09	0	0	0	0	2	0	0	0	0	0	0	0	0	0	0	0	0	0	0	0	0

ID: 15458316
name: AN-R-Sens-B2-ACp-10

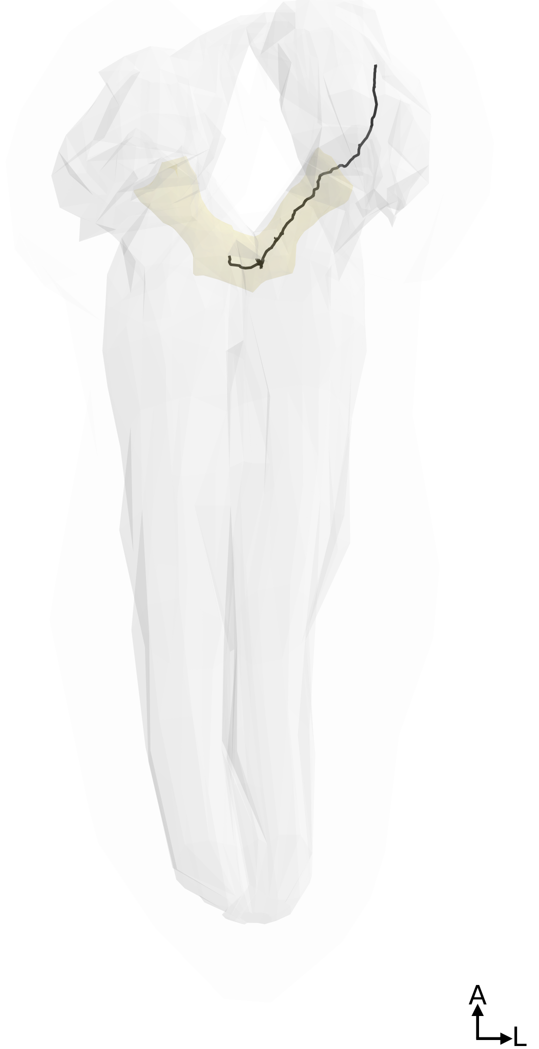

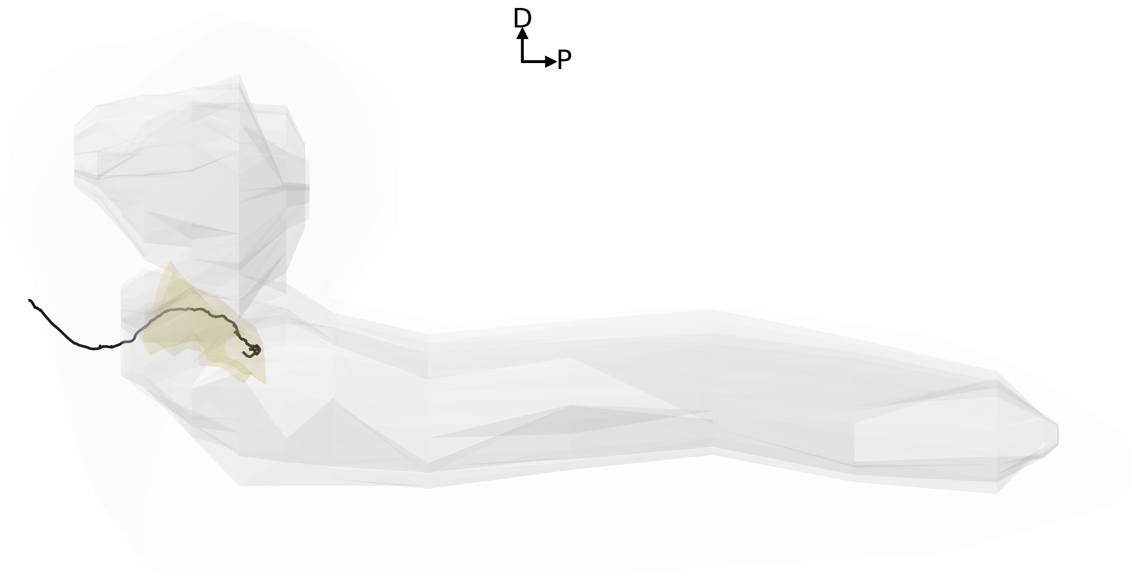

ID	*name*	SCACa	SCAVa	SCAVp	SCACal	SCACp	SCACpl	SCVM	IPCs	DMS	DH44	Se0ens	Se0ph	PMN LR	MN motor neurons	PaN motor neurons	olfactory PNs	gustatory PNs	multiglomerular PNs	unknown PNs	thermo PNs	visual PNs
15458316	AN-R-Sens-B2-ACp-10	0	0	0	0	2	0	0	0	0	0	0	0	0	0	0	0	7	0	0	0	0

ID: 15592696
name: AN-R-Sens-B2-ACp-11

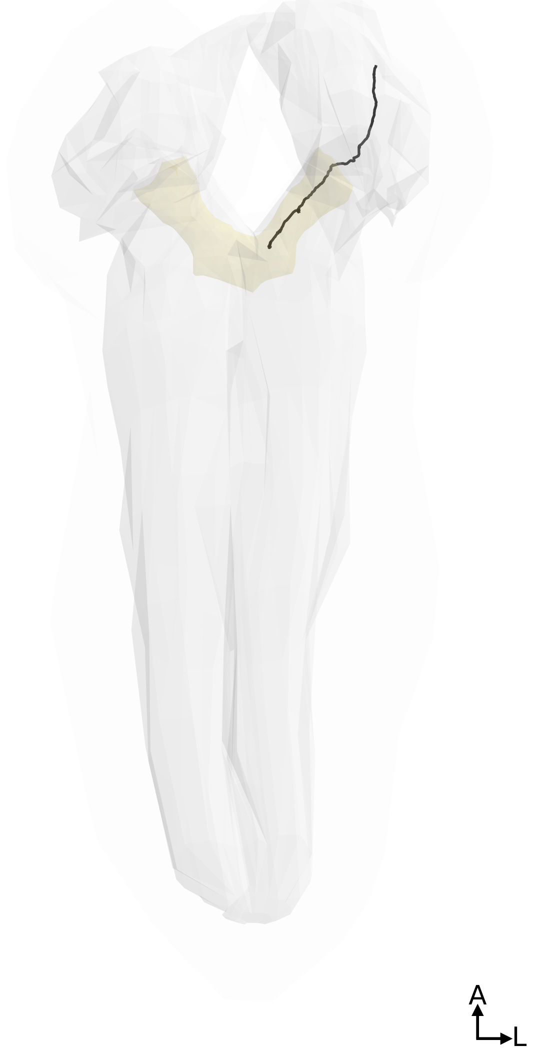

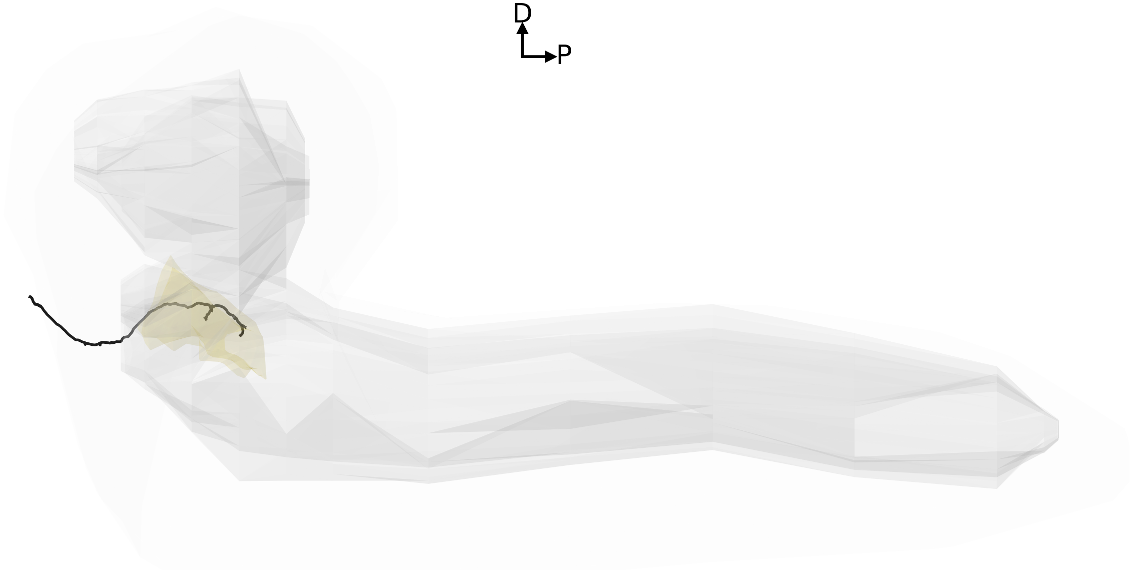

ID	name	SCACa	SCAVa	SCAVp	SCACal	SCACp	SCACpl	SCVM	IPCs	DMS	DH44	Se0ens	Se0ph	PMN LR	MN motor neurons	PaN motor neurons	olfactory PNs	gustatory PNs	multiglomerular PNs	unknown PNs	thermo PNs	visual PNs
15592696	AN-R-Sens-B2-ACp-11	0	0	0	0	1	0	0	0	0	0	0	0	0	0	0	1	7	0	0	0	0

ID: 3795498
name: AN-R-Sens-B2-ACp-12

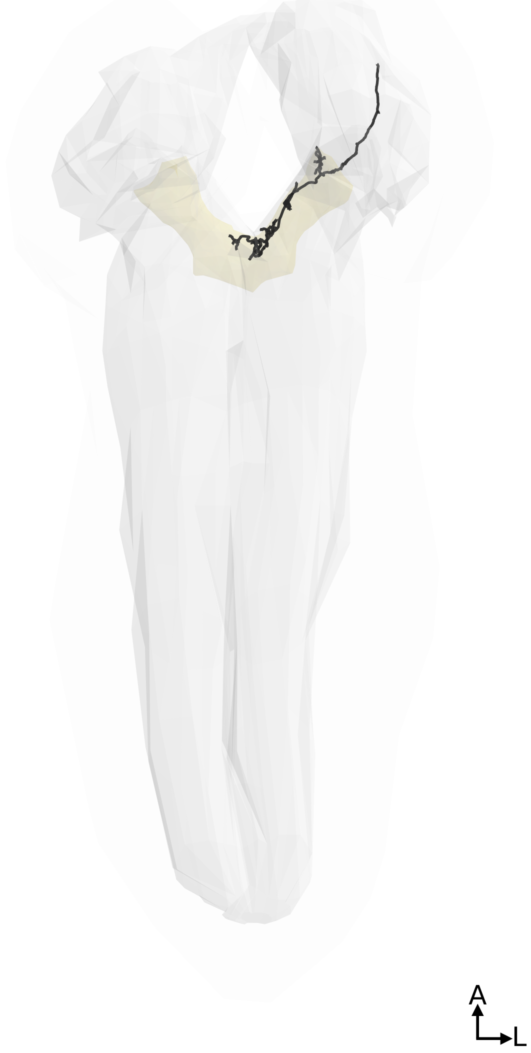

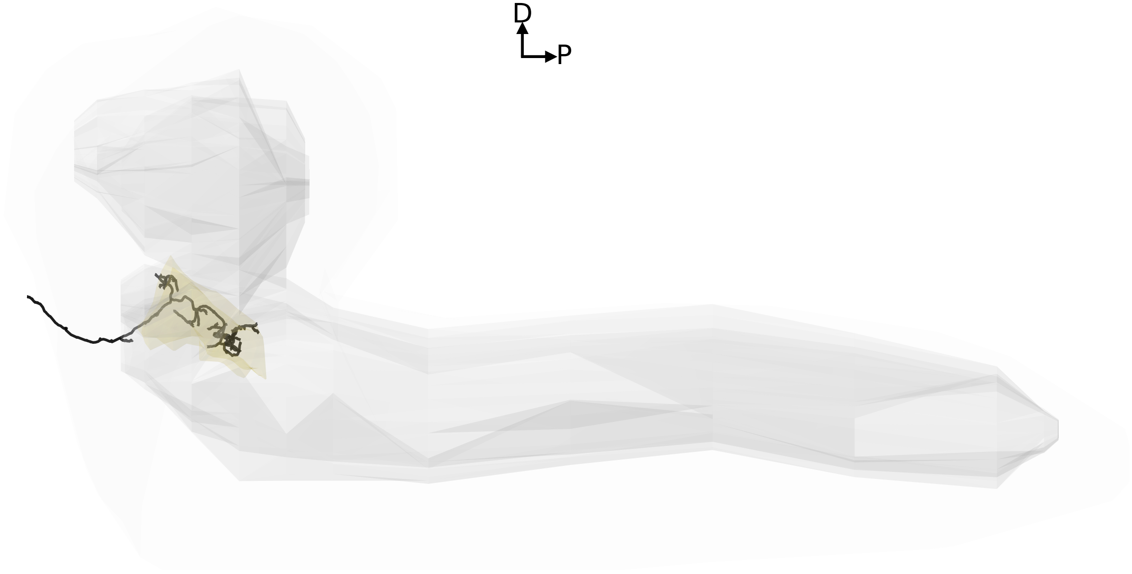

ID	*name*	SCACa	SCAVa	SCAVp	SCACal	SCACp	SCACpl	SCVM	IPCs	DMS	DH44	Se0ens	Se0ph	PMN LR	MN motor neurons	PaN motor neurons	olfactory PNs	gustatory PNs	multiglomerular PNs	unknown PNs	thermo PNs	visual PNs
3795498	AN-R-Sens-B2-ACp-12	0	0	0	0	2	0	0	0	0	0	0	0	0	0	0	0	0	0	0	0	0

ID: 15573283
name: AN-R-Sens-B2-ACp-13

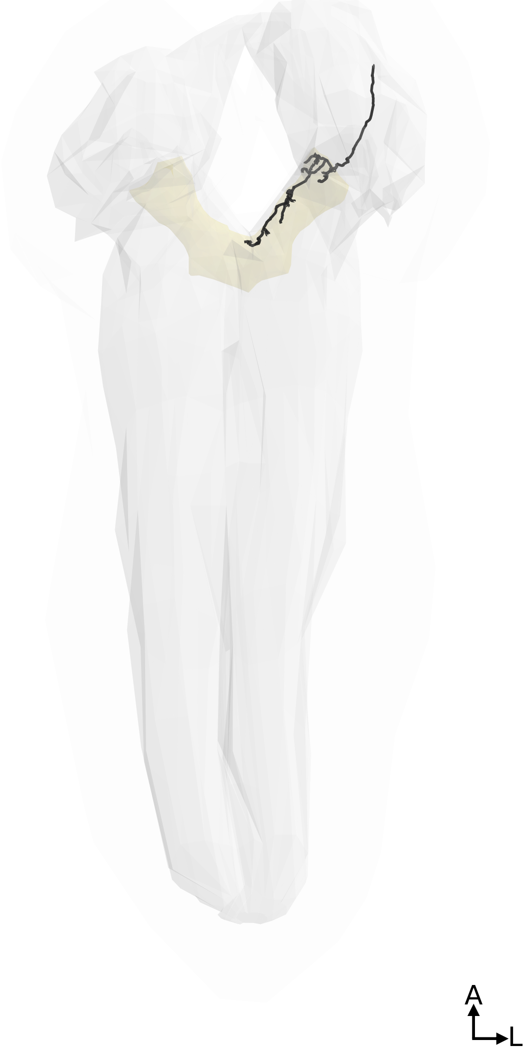

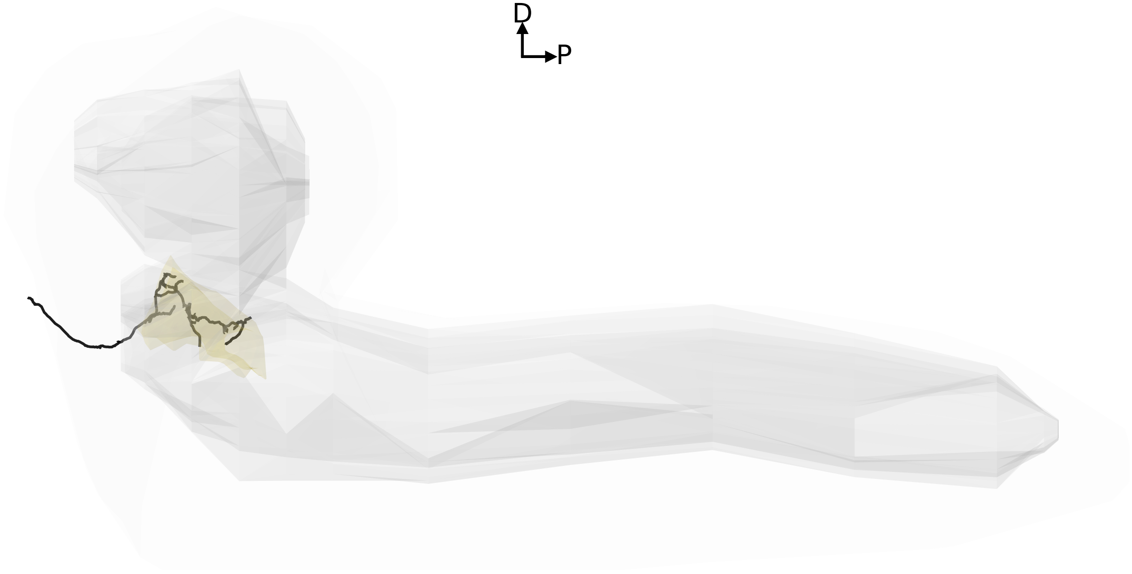

ID	*name*	SCACa	SCAVa	SCAVp	SCACal	SCACp	SCACpl	SCVM	IPCs	DMS	DH44	Se0ens	Se0ph	PMN LR	MN motor neurons	PaN motor neurons	olfactory PNs	gustatory PNs	multiglomerular PNs	unknown PNs	thermo PNs	visual PNs
15573283	AN-R-Sens-B2-ACp-13	1	0	0	0	5	0	0	0	0	1	0	0	0	0	0	0	1	0	0	0	0

ID: 15525858
name: AN-R-Sens-B2-ACp-14

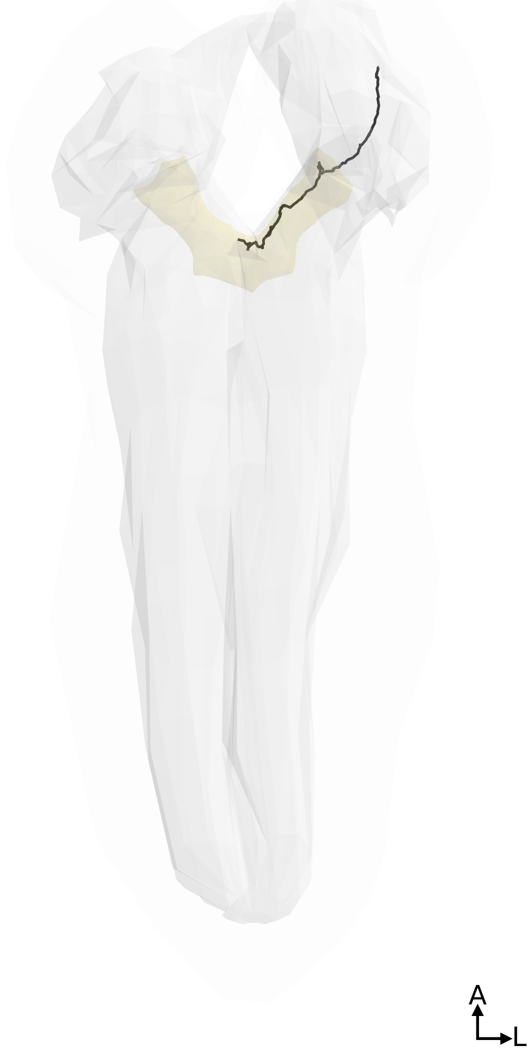

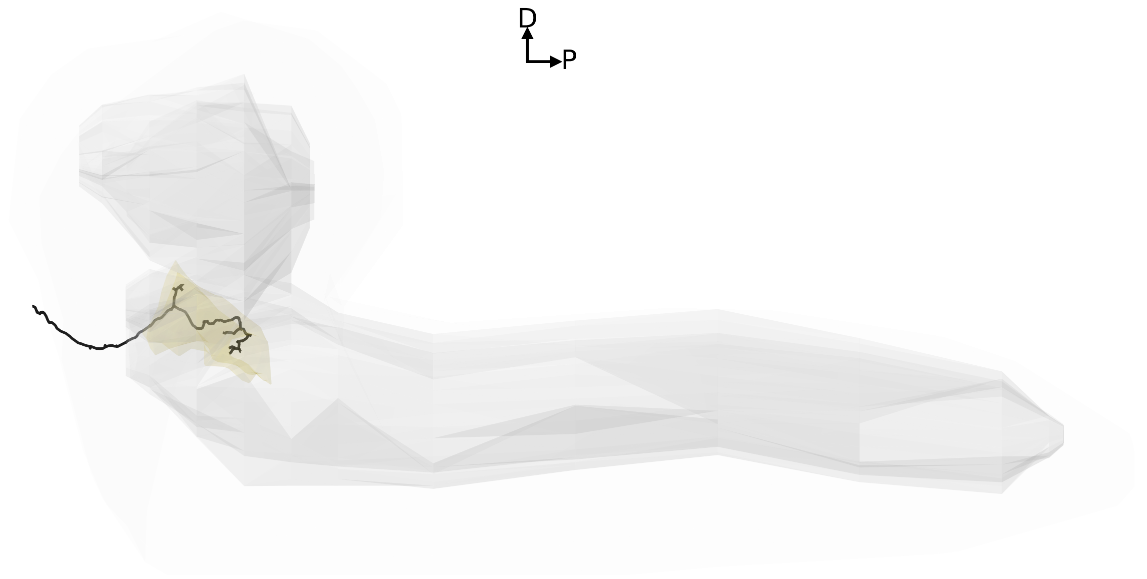

ID	name	SCACa	SCAVa	SCAVp	SCACal	SCACp	SCACpl	SCVM	IPCs	DMS	DH44	Se0ens	Se0ph	PMN LR	MN motor neurons	PaN motor neurons	olfactory PNs	gustatory PNs	multiglomerular PNs	unknown PNs	thermo PNs	visual PNs
15525858	AN-R-Sens-B2-ACp-14	0	0	0	0	1	0	0	0	0	0	0	0	0	0	0	0	0	0	0	0	0

ID: 15530882
name: AN-R-Sens-B2-ACp-15

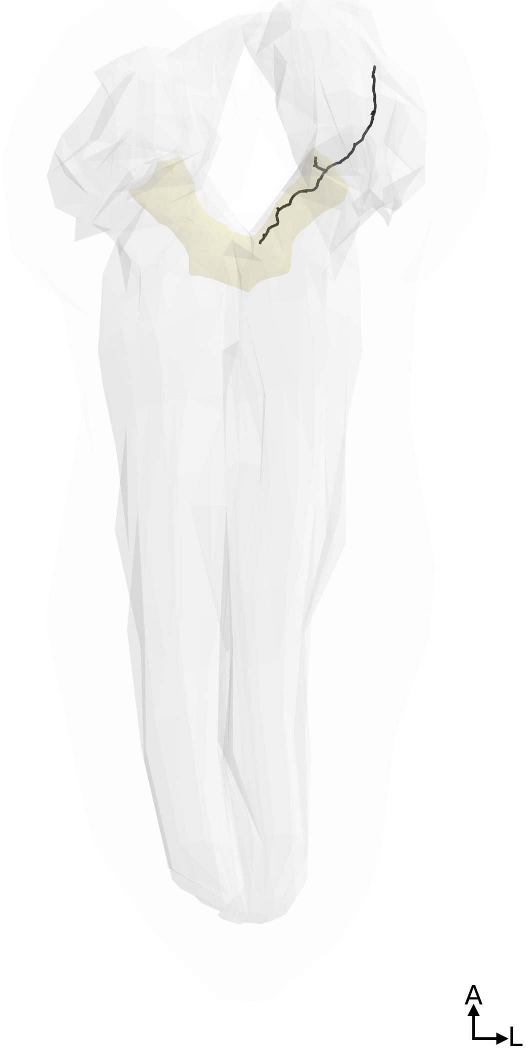

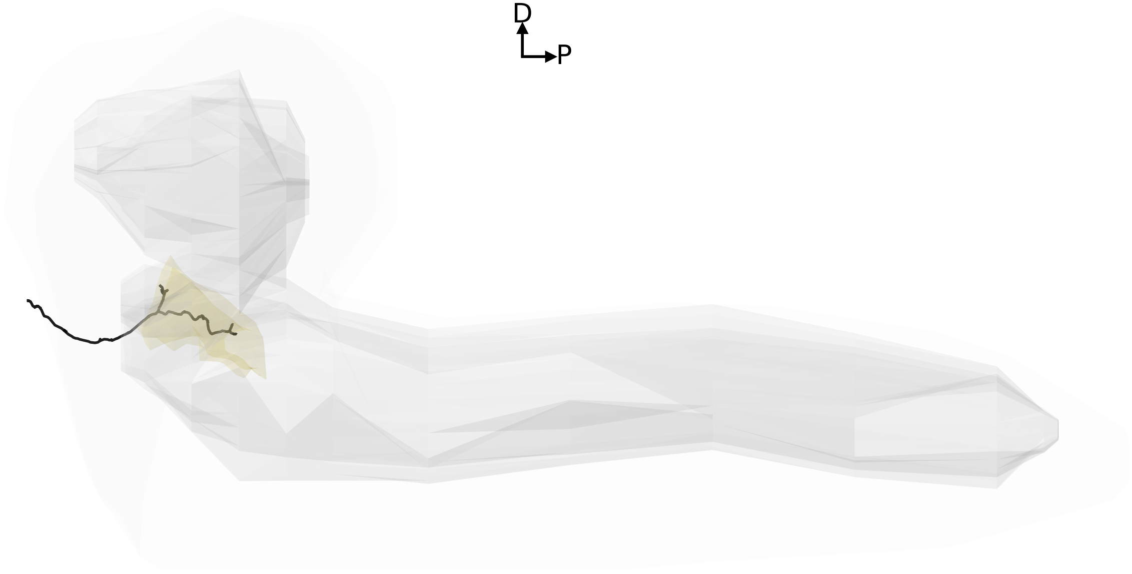

ID	*name*	SCACa	SCAVa	SCAVp	SCACal	SCACp	SCACpl	SCVM	IPCs	DMS	DH44	Se0ens	Se0ph	PMN LR	MN motor neurons	PaN motor neurons	olfactory PNs	gustatory PNs	multiglomerular PNs	unknown PNs	thermo PNs	visual PNs
15530882	AN-R-Sens-B2-ACp-15	0	0	0	0	1	0	0	0	0	0	0	0	0	0	0	0	0	0	0	0	0

ID: 15573331
name: AN-R-Sens-B2-ACp-16

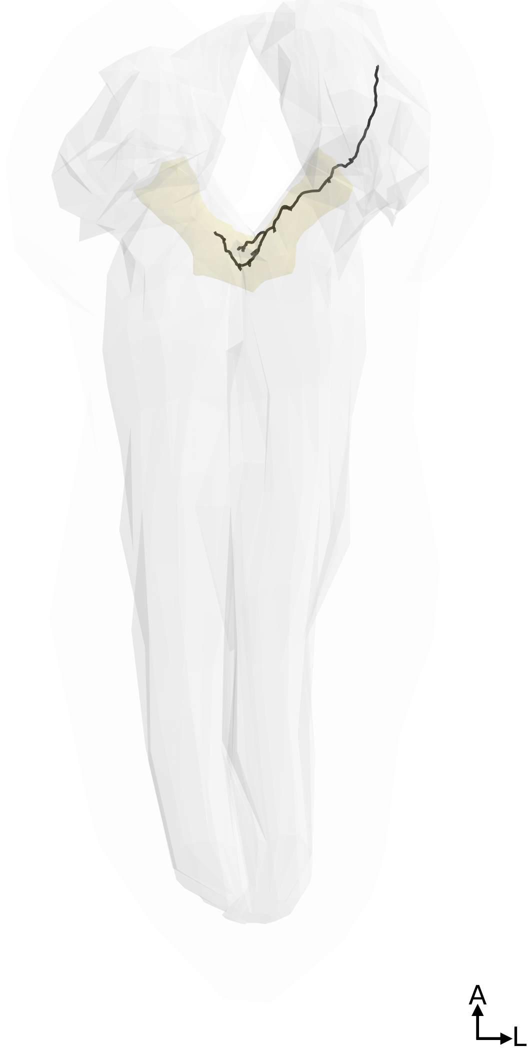

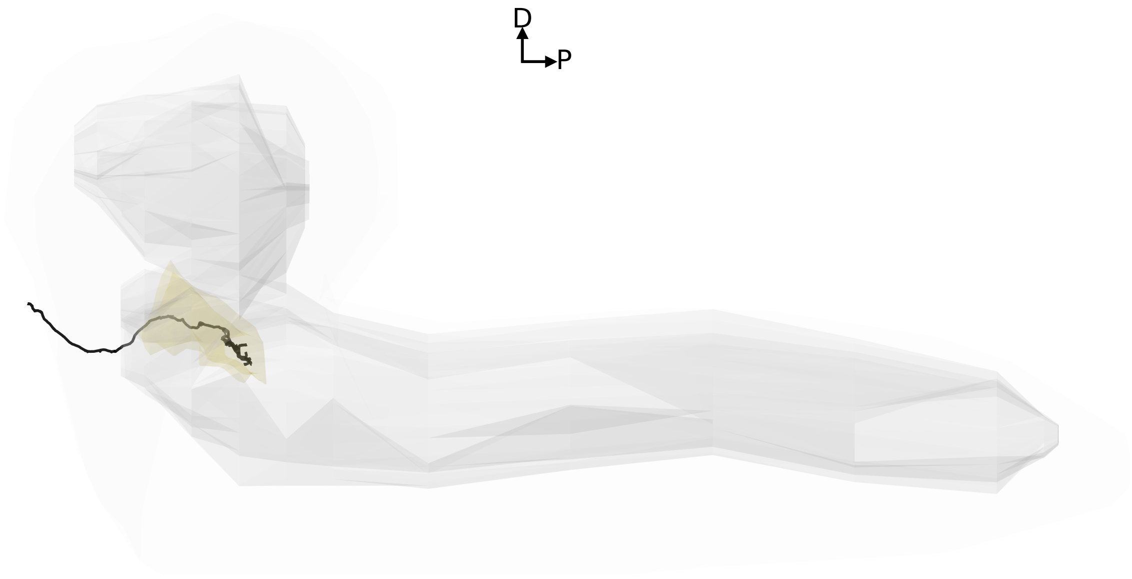

ID	*name*	SCACa	SCAVa	SCAVp	SCACal	SCACp	SCACpl	SCVM	IPCs	DMS	DH44	Se0ens	Se0ph	PMN LR	MN motor neurons	PaN motor neurons	olfactory PNs	gustatory PNs	multiglomerular PNs	unknown PNs	thermo PNs	visual PNs
15573331	AN-R-Sens-B2-ACp-16	0	0	0	0	1	0	0	0	0	1	0	0	0	0	0	2	0	0	0	0	0

ID: 15398807
name: AN-R-Sens-B2-ACp-17

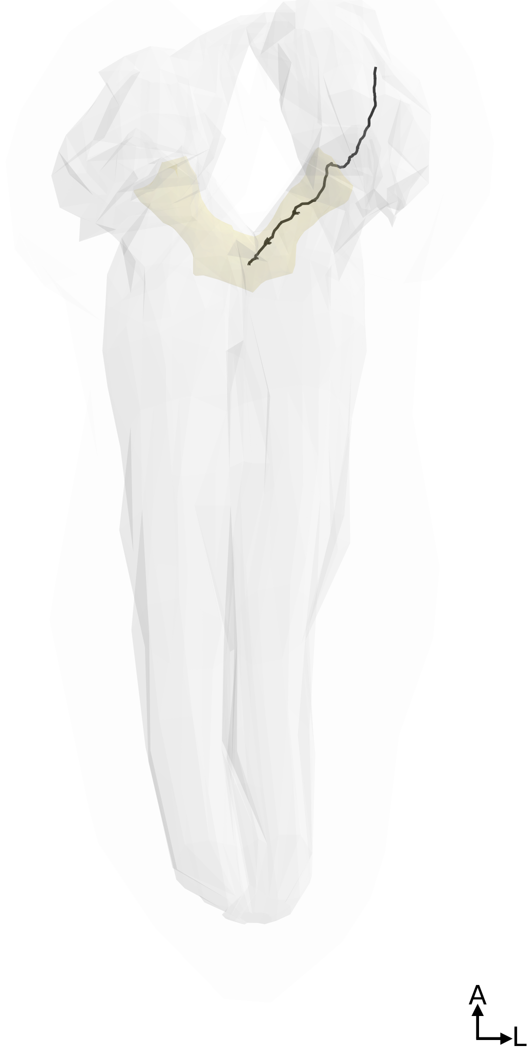

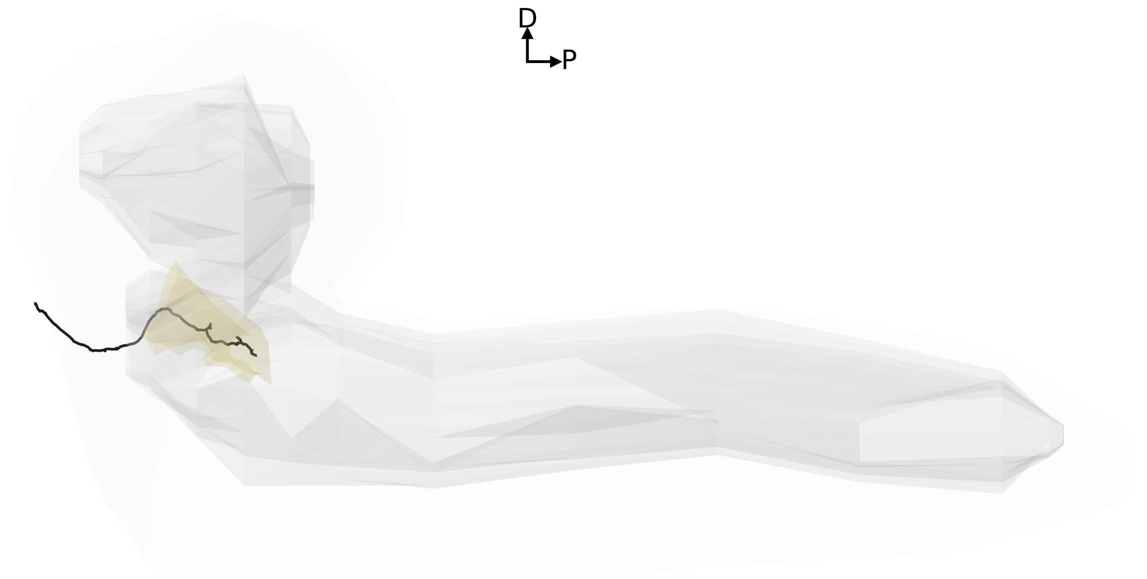

ID	name	SCACa	SCAVa	SCAVp	SCACal	SCACp	SCACpl	SCVM	IPCs	DMS	DH44	Se0ens	Se0ph	PMN LR	MN motor neurons	PaN motor neurons	olfactory PNs	gustatory PNs	multiglomerular PNs	unknown PNs	thermo PNs	visual PNs
15398807	AN-R-Sens-B2-ACp-17	0	0	0	0	2	0	0	0	0	0	0	0	0	0	0	0	5	0	0	0	0

ID: 15506094
name: AN-R-Sens-B2-ACp-18

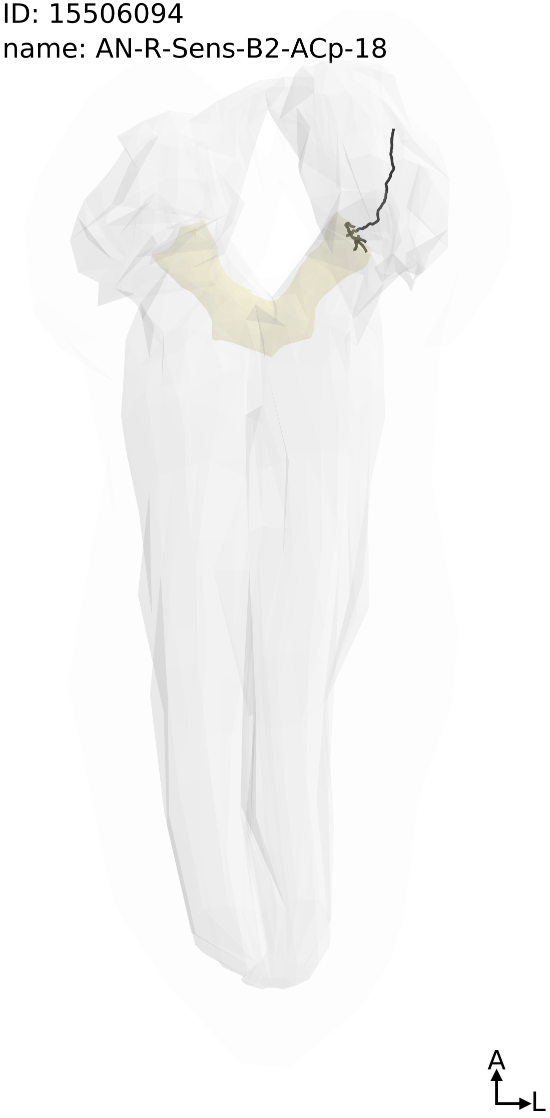

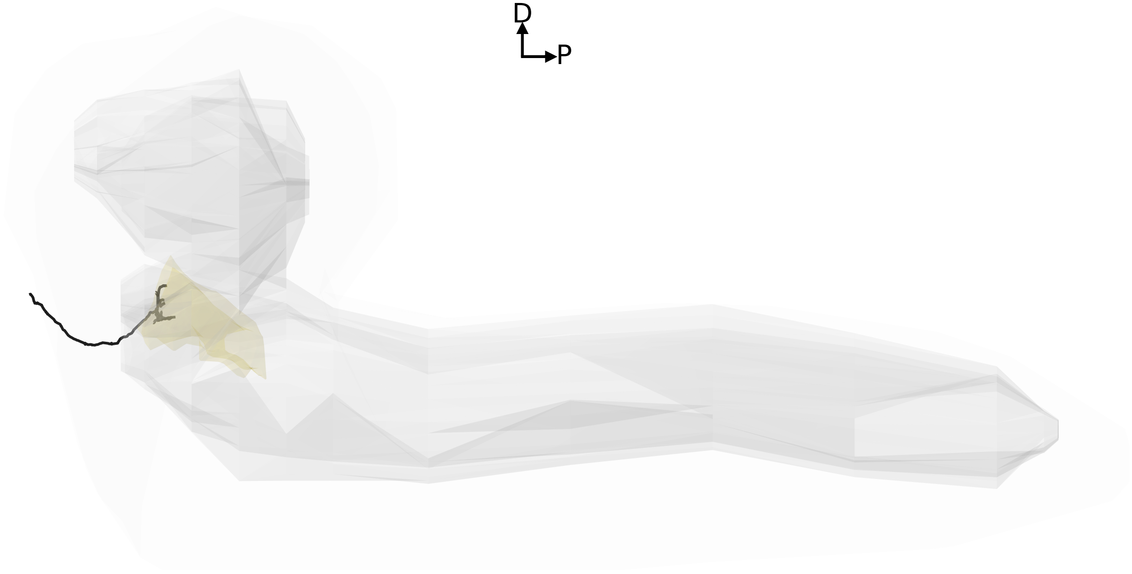

ID	*name*	SCACa	SCAVa	SCAVp	SCACal	SCACp	SCACpl	SCVM	IPCs	DMS	DH44	Se0ens	Se0ph	PMN LR	MN motor neurons	PaN motor neurons	olfactory PNs	gustatory PNs	multiglomerular PNs	unknown PNs	thermo PNs	visual PNs
15506094	AN-R-Sens-B2-ACp-18	0	0	0	0	2	0	0	0	0	0	0	0	0	0	0	0	1	1	0	0	0

ID: 15541976
name: AN-R-Sens-B2-ACp-19

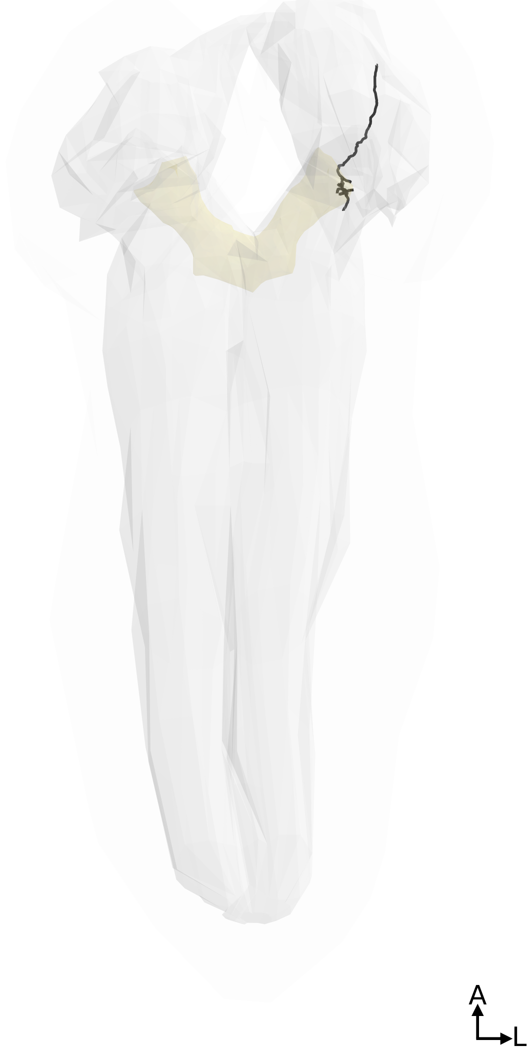

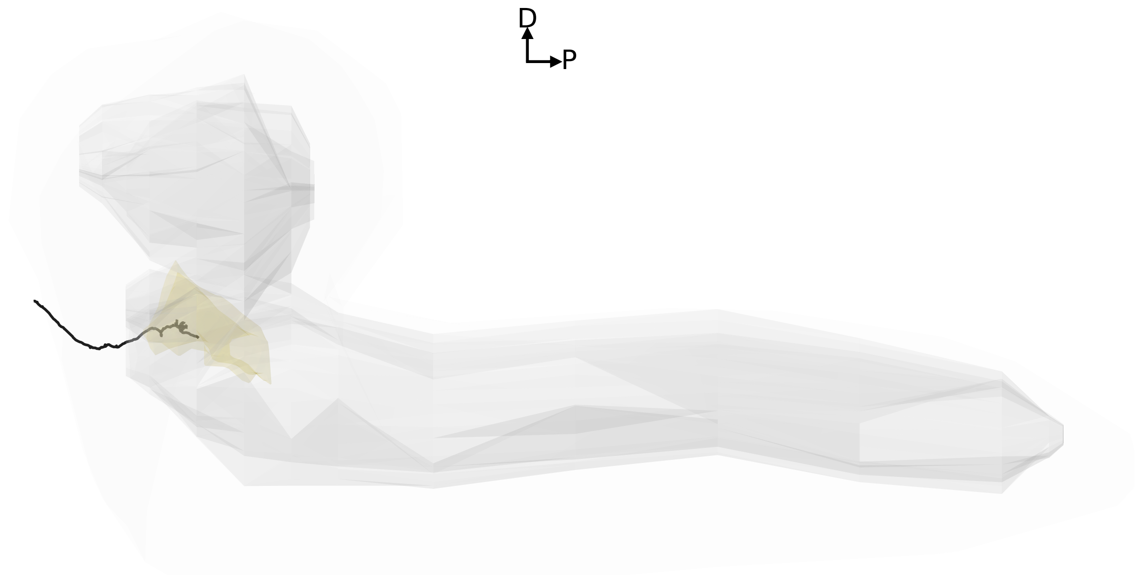

ID	*name*	SCACa	SCAVa	SCAVp	SCACal	SCACp	SCACpl	SCVM	IPCs	DMS	DH44	Se0ens	Se0ph	PMN LR	MN motor neurons	PaN motor neurons	olfactory PNs	gustatory PNs	multiglomerular PNs	unknown PNs	thermo PNs	visual PNs
15541976	AN-R-Sens-B2-ACp-19	0	0	0	0	2	0	0	0	0	0	0	0	0	0	0	0	0	5	0	0	0

ID: 15507888
name: AN-R-Sens-B2-ACp-20

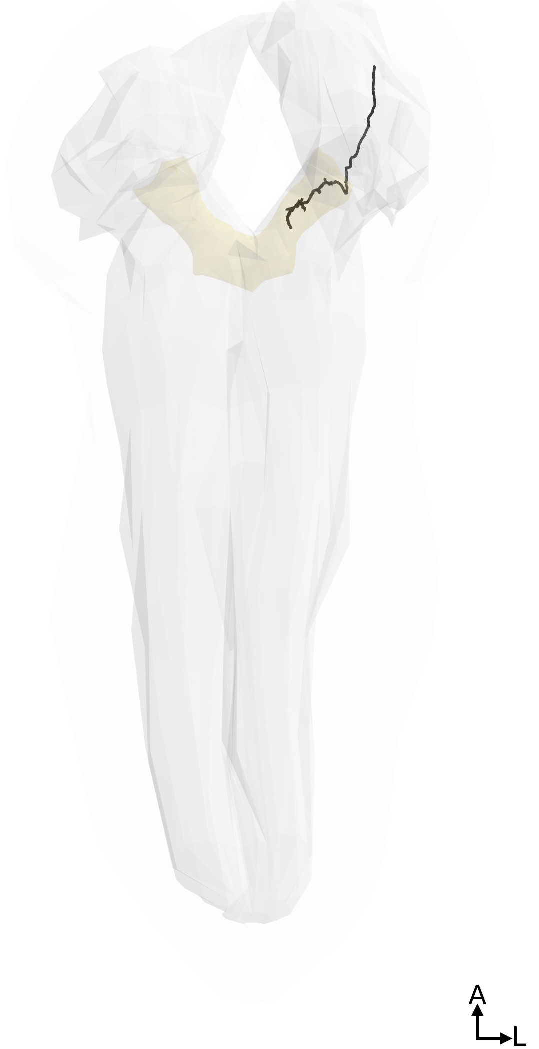

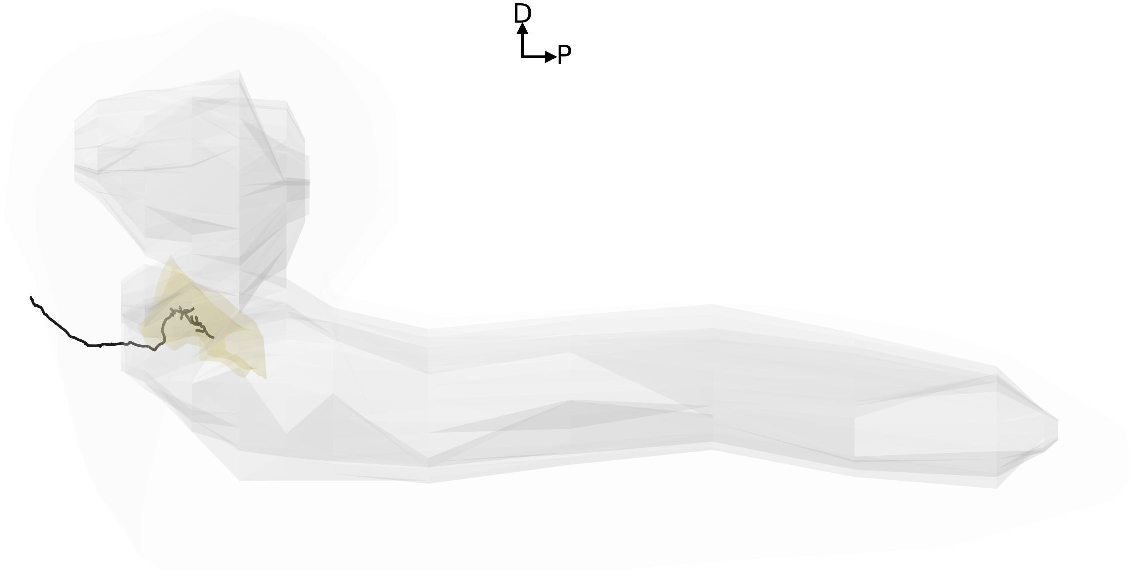

ID	*name*	SCACa	SCAVa	SCAVp	SCACal	SCACp	SCACpl	SCVM	IPCs	DMS	DH44	Se0ens	Se0ph	PMN LR	MN motor neurons	PaN motor neurons	olfactory PNs	gustatory PNs	multiglomerular PNs	unknown PNs	thermo PNs	visual PNs
15507888	AN-R-Sens-B2-ACp-20	0	0	0	0	1	0	0	0	0	0	0	0	0	0	0	1	0	0	0	0	0

ID: 15522777
name: AN-R-Sens-B2-ACp-21

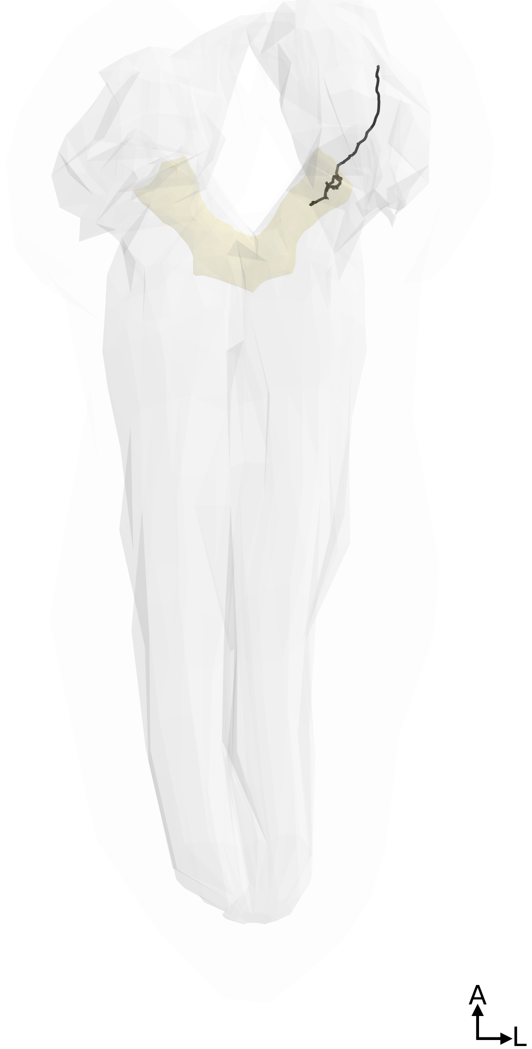

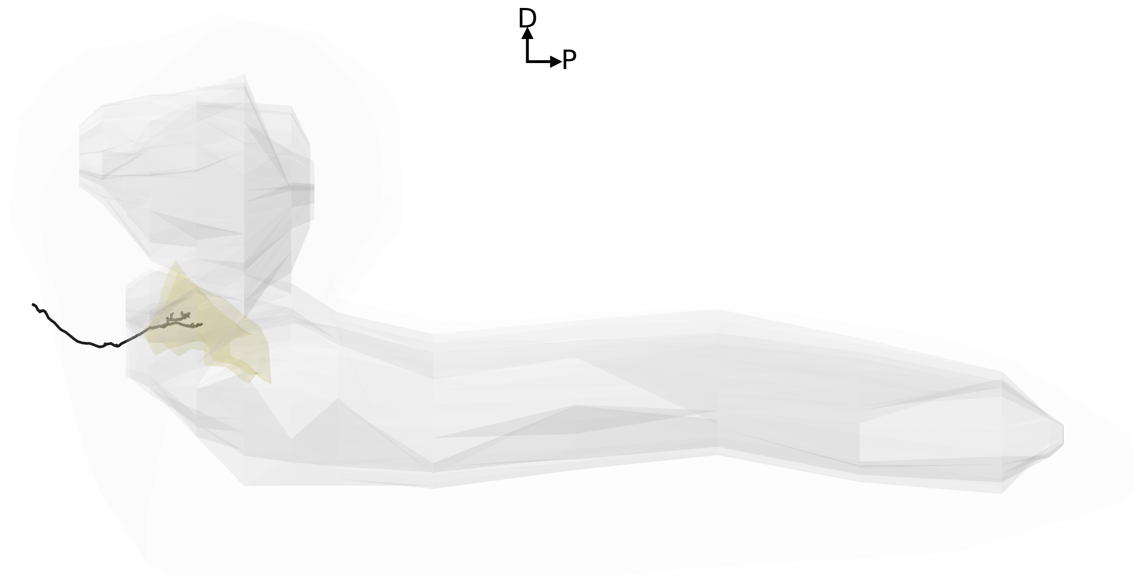

ID	*name*	SCACa	SCAVa	SCAVp	SCACal	SCACp	SCACpl	SCVM	IPCs	DMS	DH44	Se0ens	Se0ph	PMN LR	MN motor neurons	PaN motor neurons	olfactory PNs	gustatory PNs	multiglomerular PNs	unknown PNs	thermo PNs	visual PNs
15522777	AN-R-Sens-B2-ACp-21	0	0	0	0	1	0	0	0	0	0	0	0	0	0	0	9	8	2	0	0	0

ID: 15461769
name: AN-R-Sens-B2-ACp-22

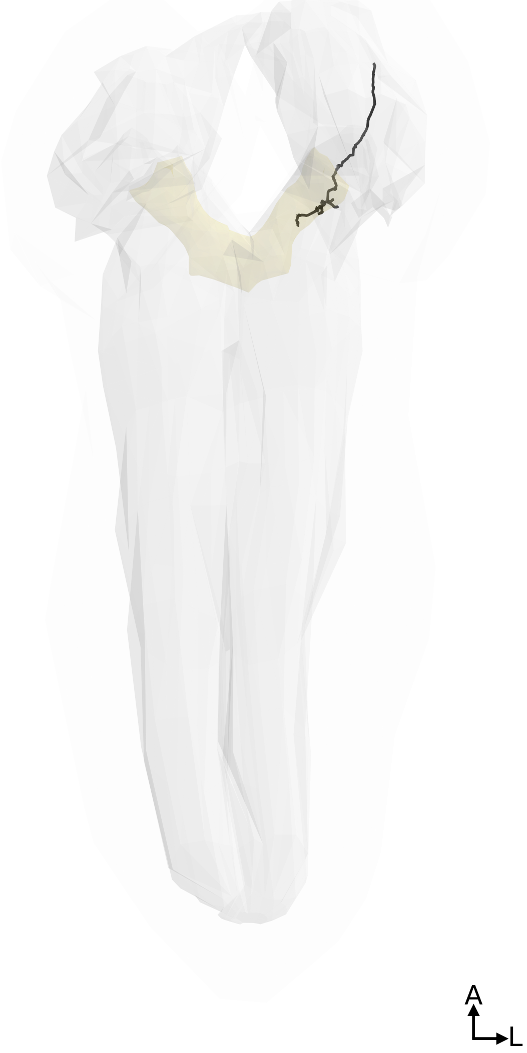

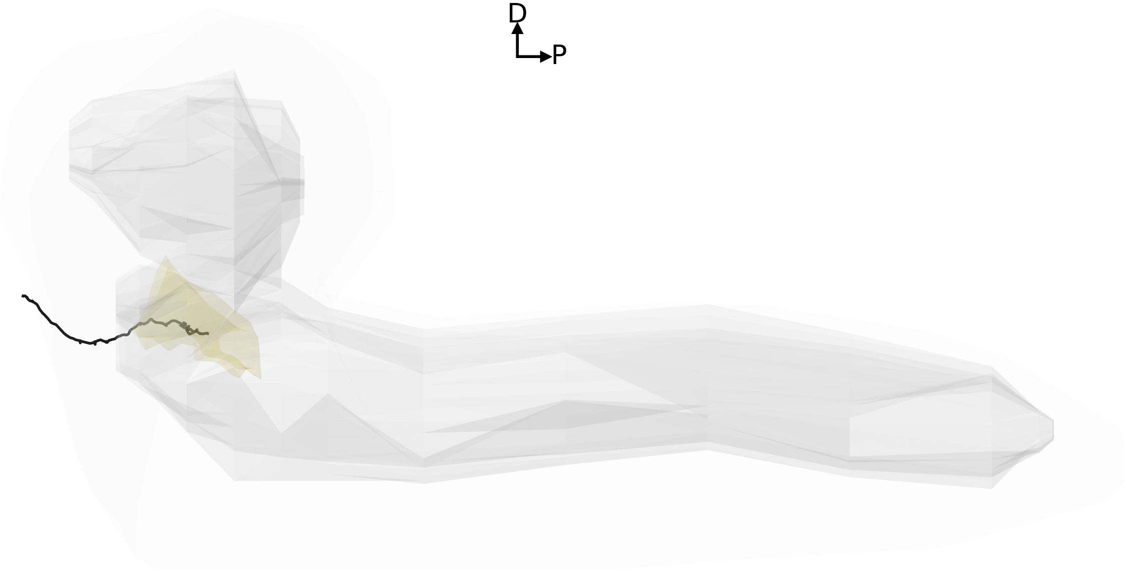

ID	name	SCACa	SCAVa	SCAVp	SCACal	SCACp	SCACpl	SCVM	IPCs	DMS	DH44	Se0ens	Se0ph	PMN LR	MN motor neurons	PaN motor neurons	olfactory PNs	gustatory PNs	multiglomerular PNs	unknown PNs	thermo PNs	visual PNs
15461769	AN-R-Sens-B2-ACp-22	0	0	0	0	2	0	0	0	0	0	0	0	0	0	0	0	0	0	0	0	0

ID: 18620885
name: AN-R-Sens-B2-ACp-23

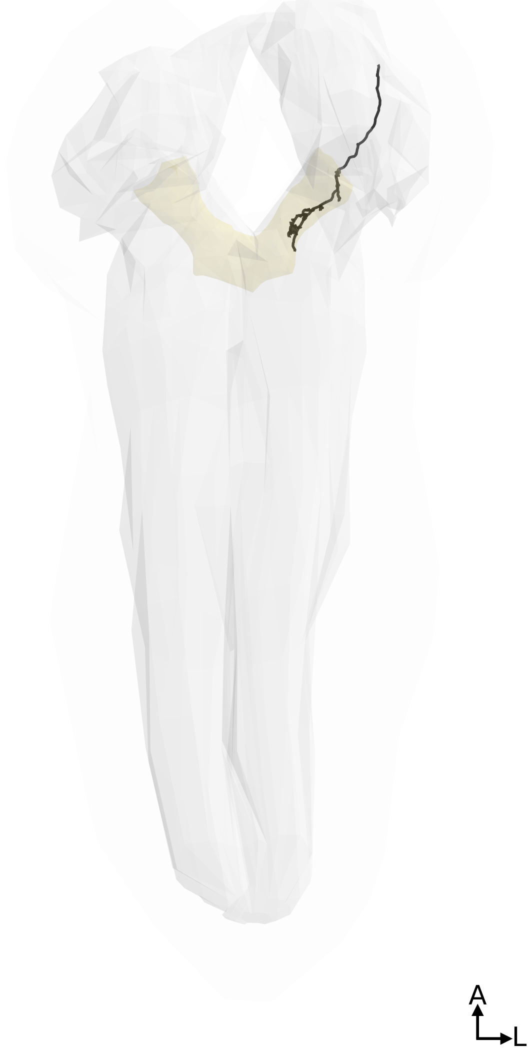

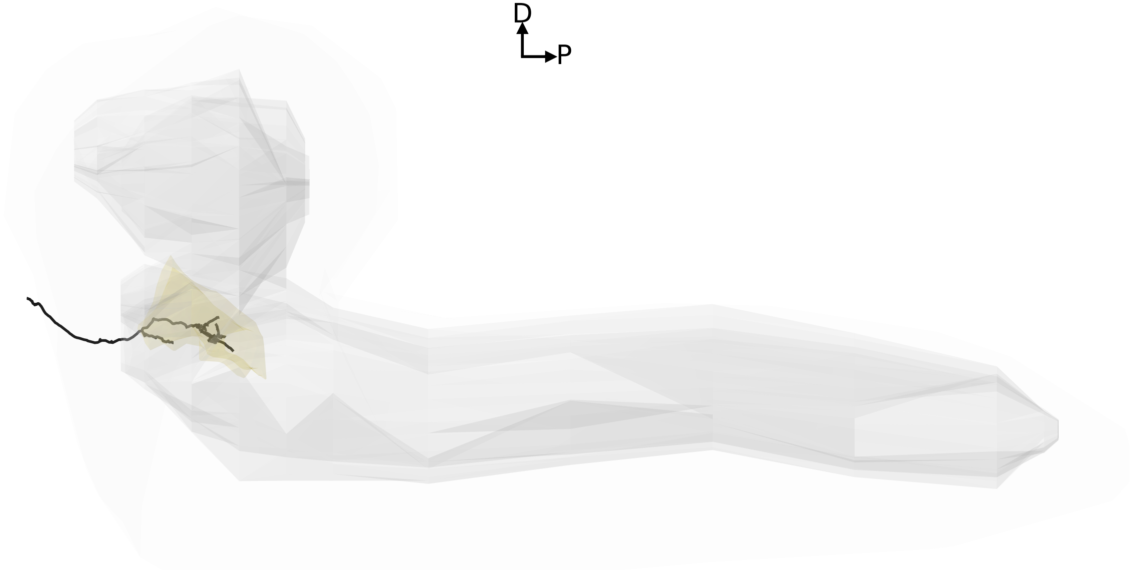

ID	name	SCACa	SCAVa	SCAVp	SCACal	SCACp	SCACpl	SCVM	IPCs	DMS	DH44	Se0ens	Se0ph	PMN LR	MN motor neurons	PaN motor neurons	olfactory PNs	gustatory PNs	multiglomerular PNs	unknown PNs	thermo PNs	visual PNs
18620885	AN-R-Sens-B2-ACp-23	0	0	0	0	4	0	0	0	0	0	0	0	0	0	0	0	0	0	0	0	0

ID: 3822523
name: AN-R-Sens-B3-ACp-08

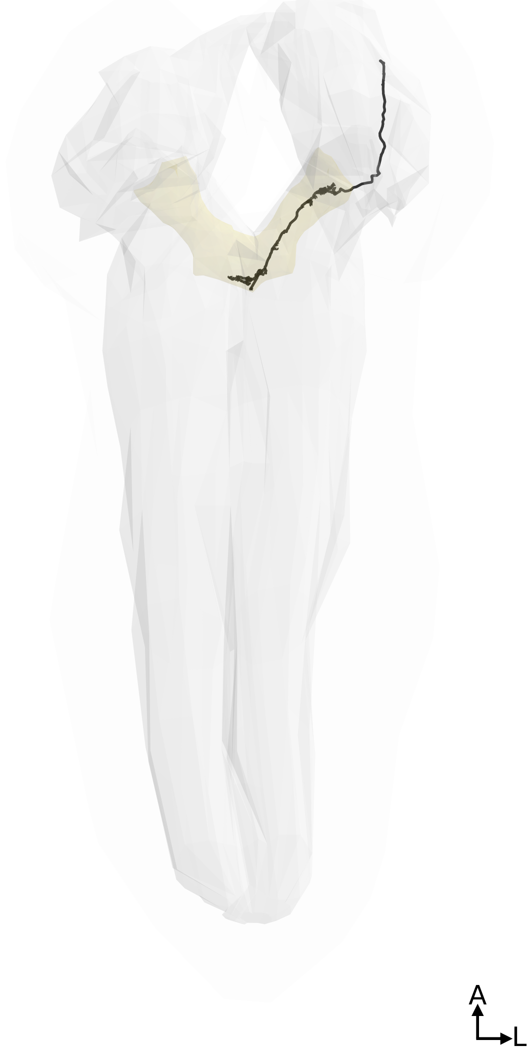

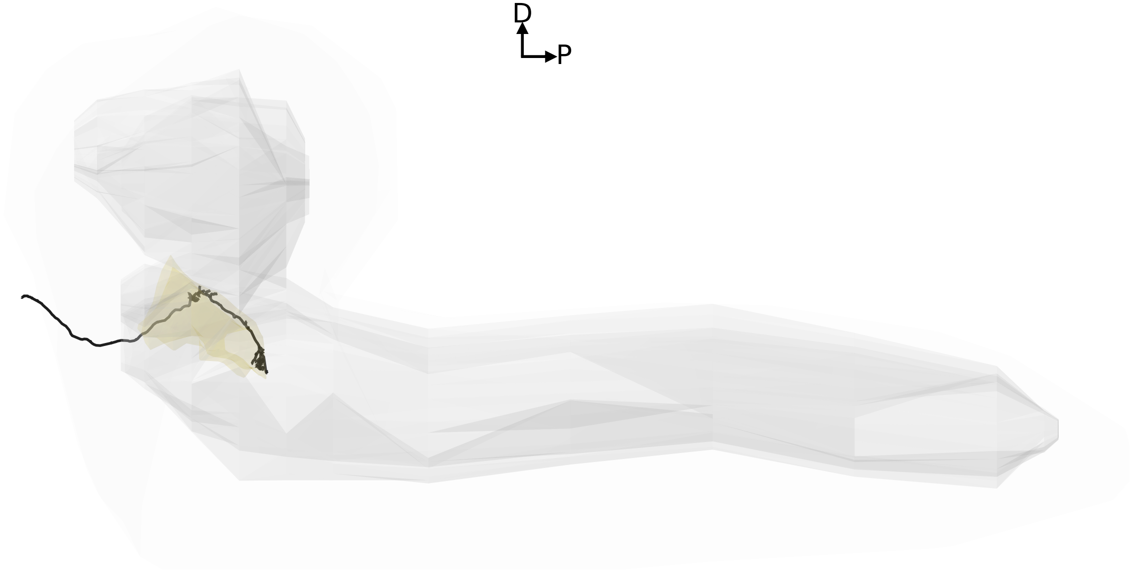

ID	*name*	SCACa	SCAVa	SCAVp	SCACal	SCACp	SCACpl	SCVM	IPCs	DMS	DH44	Se0ens	Se0ph	PMN LR	MN motor neurons	PaN motor neurons	olfactory PNs	gustatory PNs	multiglomerular PNs	unknown PNs	thermo PNs	visual PNs
3822523	AN-R-Sens-B3-ACp-08	0	0	0	0	1	0	0	0	0	0	0	0	0	0	0	0	0	0	0	0	0

ID: 9853425
name: AN-R-Sens-B3-ACp-09

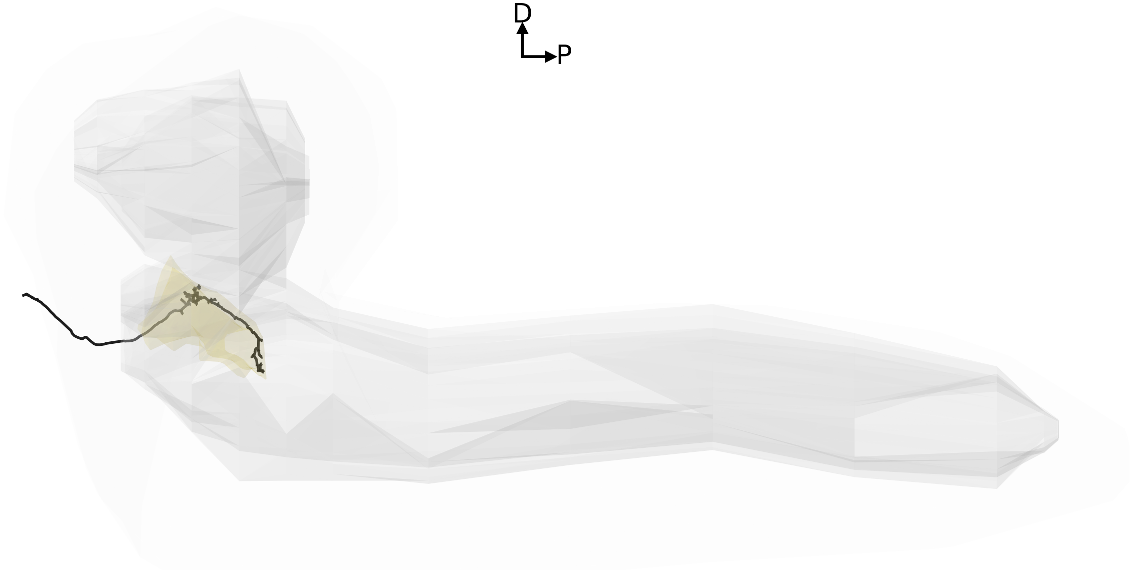

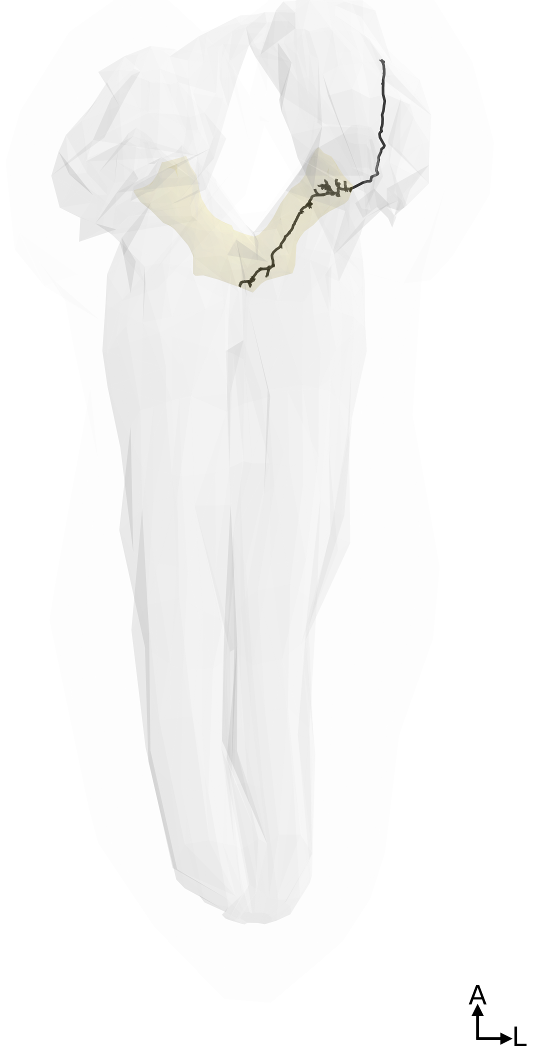

ID	*name*	SCACa	SCAVa	SCAVp	SCACal	SCACp	SCACpl	SCVM	IPCs	DMS	DH44	Se0ens	Se0ph	PMN LR	MN motor neurons	PaN motor neurons	olfactory PNs	gustatory PNs	multiglomerular PNs	unknown PNs	thermo PNs	visual PNs
9853425	AN-R-Sens-B3-ACp-09	0	0	0	1	3	0	0	0	0	0	0	0	0	0	0	5	2	3	0	0	0

ID: 4338596
name: MN-L-Sens-B2-ACp-01

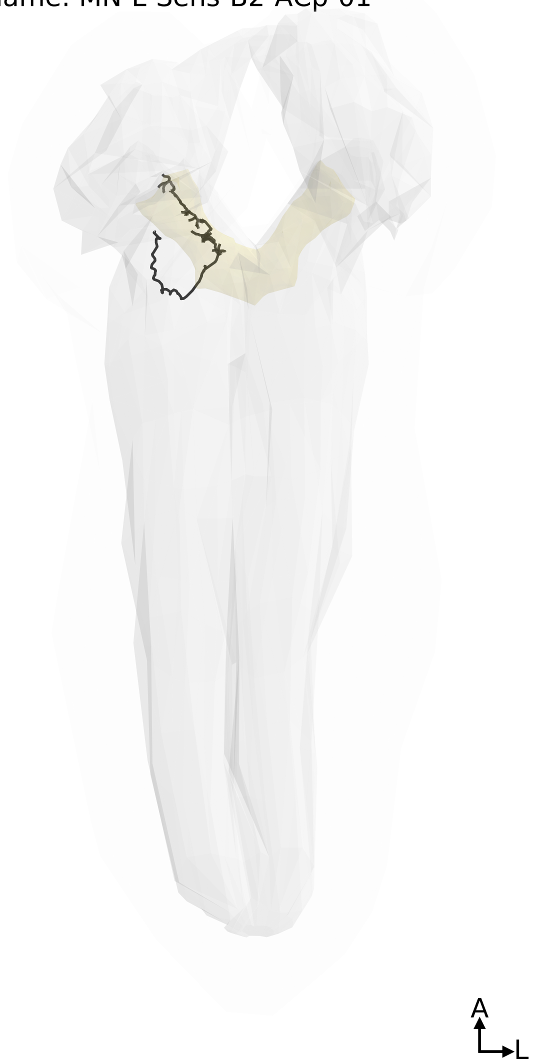

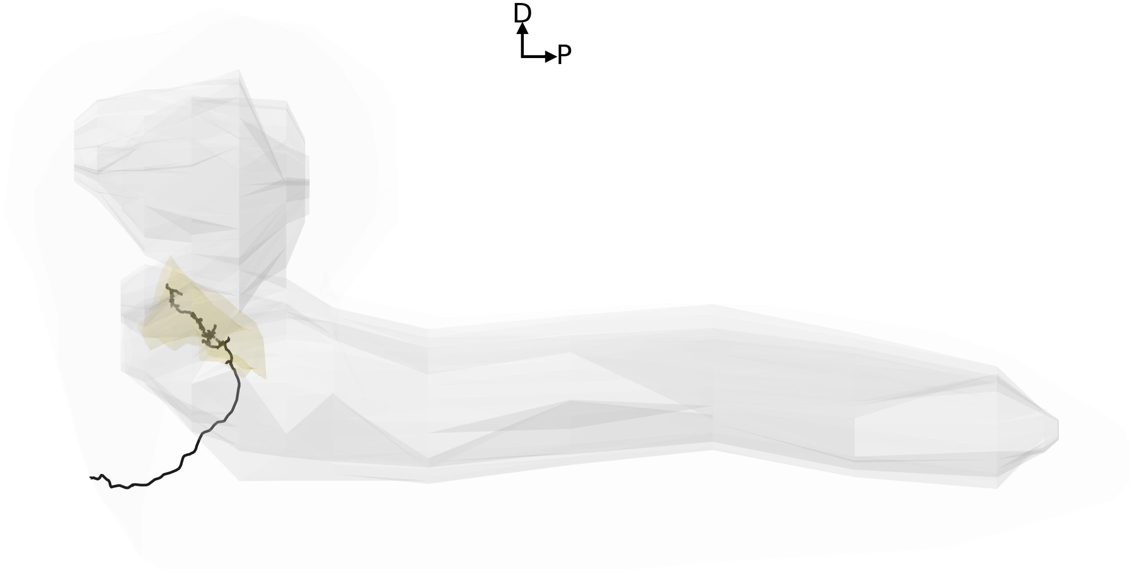

ID	*name*	SCACa	SCAVa	SCAVp	SCACal	SCACp	SCACpl	SCVM	IPCs	DMS	DH44	Se0ens	Se0ph	PMN LR	MN motor neurons	PaN motor neurons	olfactory PNs	gustatory PNs	multiglomerular PNs	unknown PNs	thermo PNs	visual PNs
4338596	MN-L-Sens-B2-ACp-01	0	0	0	0	11	0	0	0	0	0	0	0	0	0	0	1	44	1	0	0	0

ID: 15716701
name: MN-L-Sens-B2-ACp-02

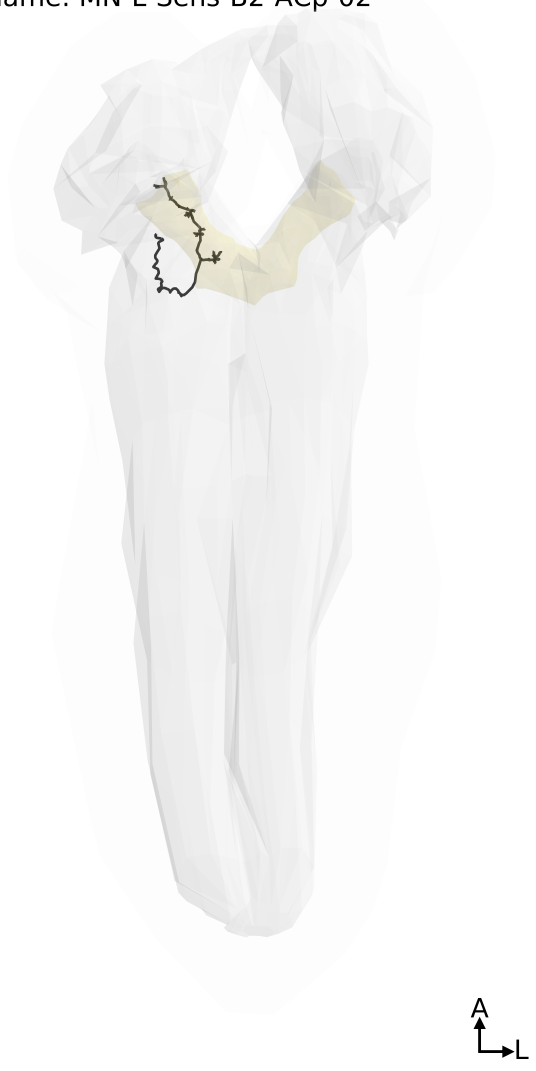

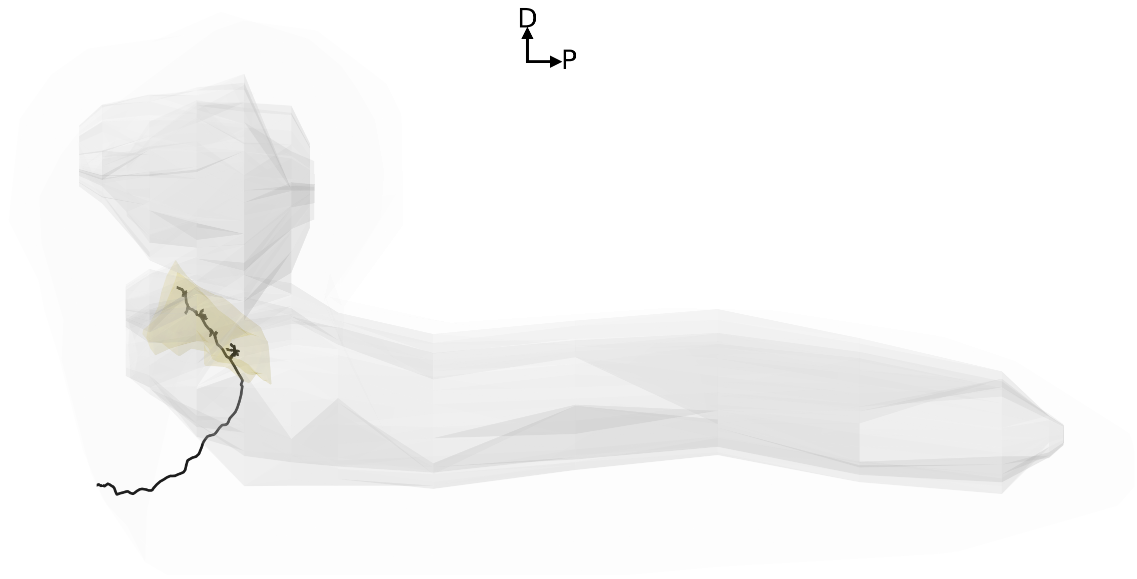

ID	*name*	SCACa	SCAVa	SCAVp	SCACal	SCACp	SCACpl	SCVM	IPCs	DMS	DH44	Se0ens	Se0ph	PMN LR	MN motor neurons	PaN motor neurons	olfactory PNs	gustatory PNs	multiglomerular PNs	unknown PNs	thermo PNs	visual PNs
15716701	MN-L-Sens-B2-ACp-02	0	0	0	0	0	0	0	0	0	0	0	0	0	0	0	0	19	6	0	0	0

ID: 15716309
name: MN-L-Sens-B2-ACp-03

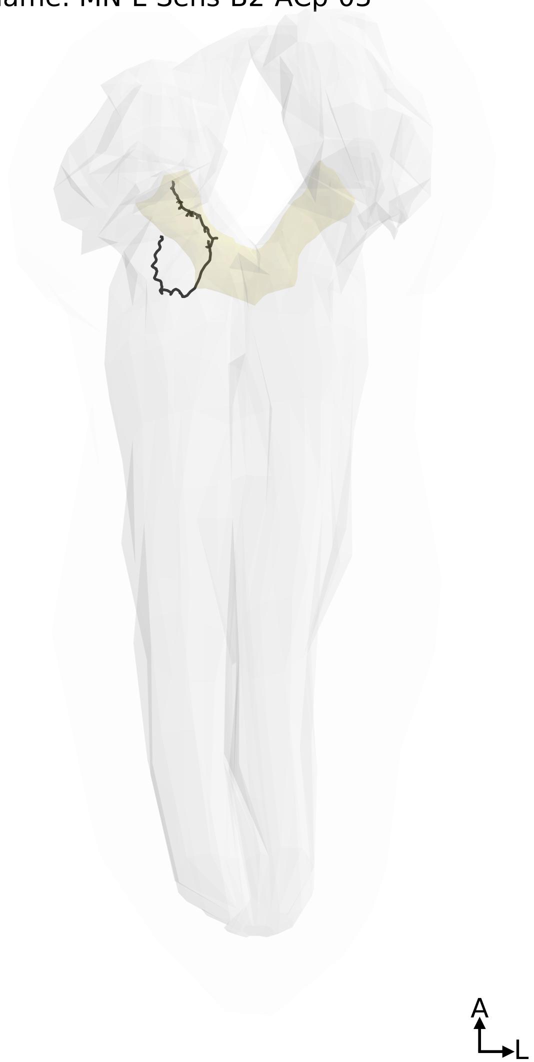

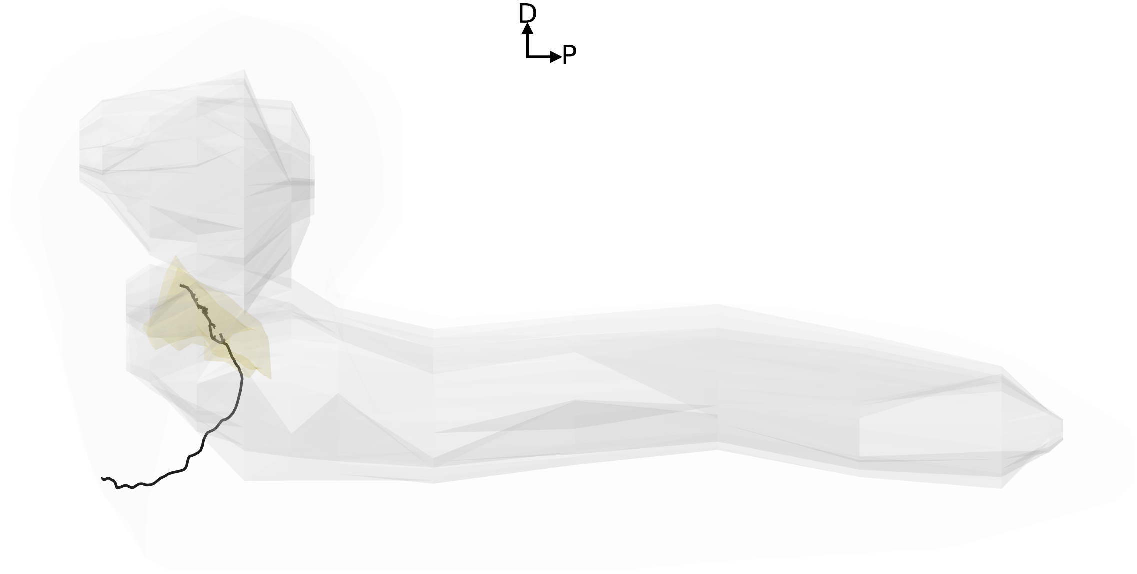

ID	name	SCACa	SCAVa	SCAVp	SCACal	SCACp	SCACpl	SCVM	IPCs	DMS	DH44	Se0ens	Se0ph	PMN LR	MN motor neurons	PaN motor neurons	olfactory PNs	gustatory PNs	multiglomerular PNs	unknown PNs	thermo PNs	visual PNs
15716309	MN-L-Sens-B2-ACp-03	0	0	0	0	4	0	0	0	0	0	0	0	0	0	0	0	10	7	0	0	0

ID: 15715643
name: MN-L-Sens-B2-ACp-04

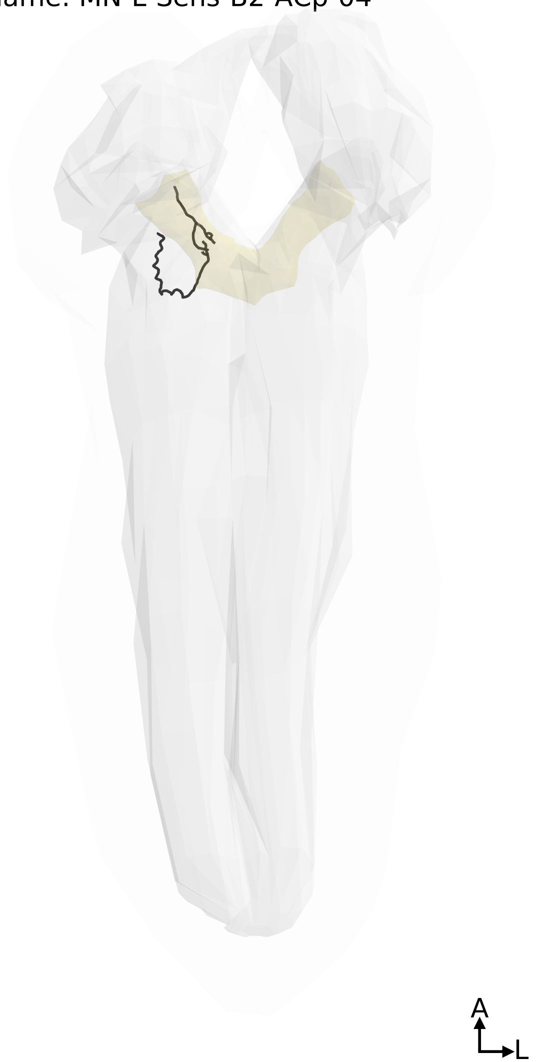

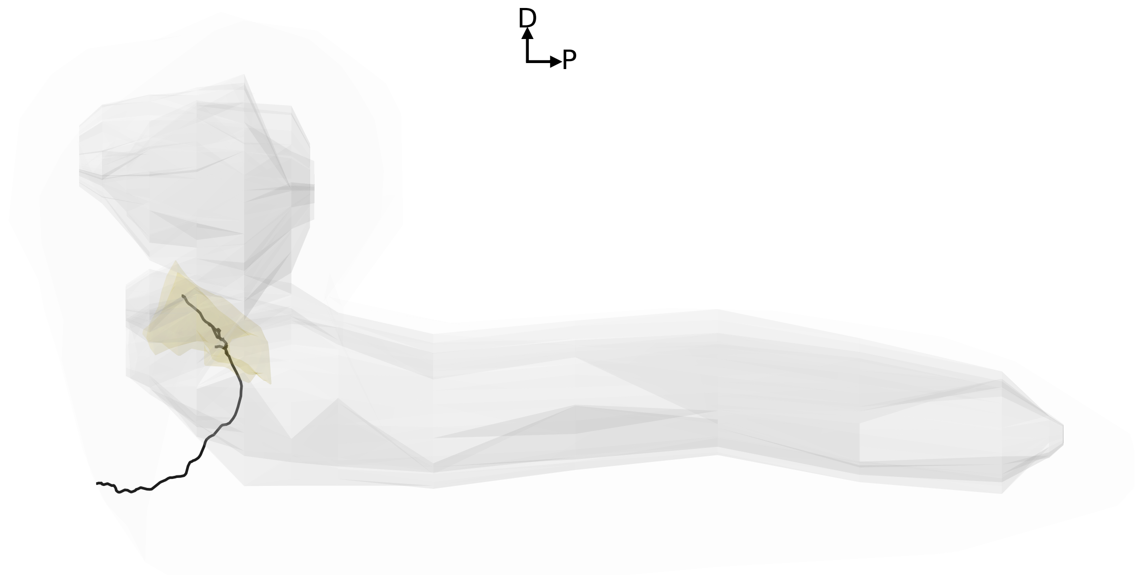

ID	*name*	SCACa	SCAVa	SCAVp	SCACal	SCACp	SCACpl	SCVM	IPCs	DMS	DH44	Se0ens	Se0ph	PMN LR	MN motor neurons	PaN motor neurons	olfactory PNs	gustatory PNs	multiglomerular PNs	unknown PNs	thermo PNs	visual PNs
15715643	MN-L-Sens-B2-ACp-04	0	0	0	0	5	0	0	0	0	0	0	0	0	0	0	0	7	0	0	0	0

ID: 15716094
name: MN-L-Sens-B2-ACp-05

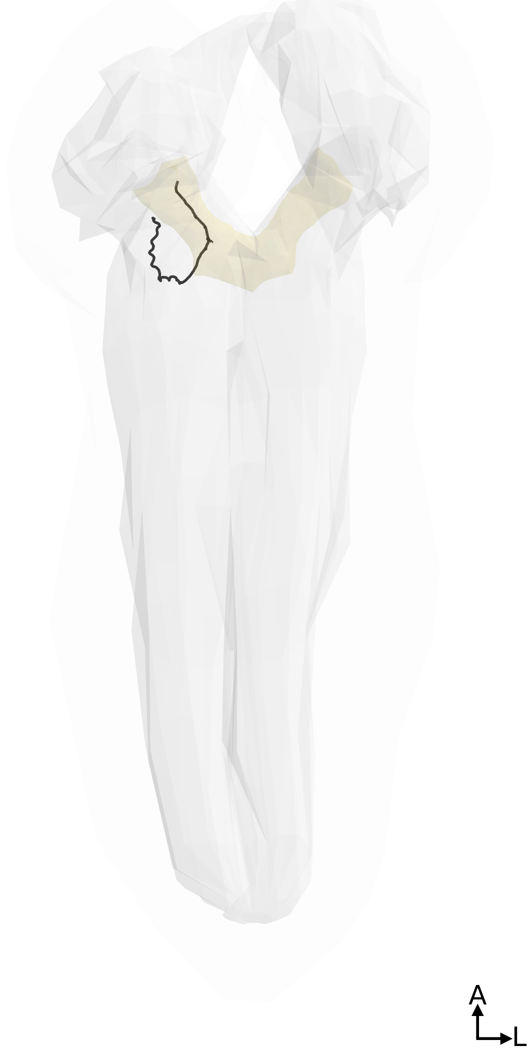

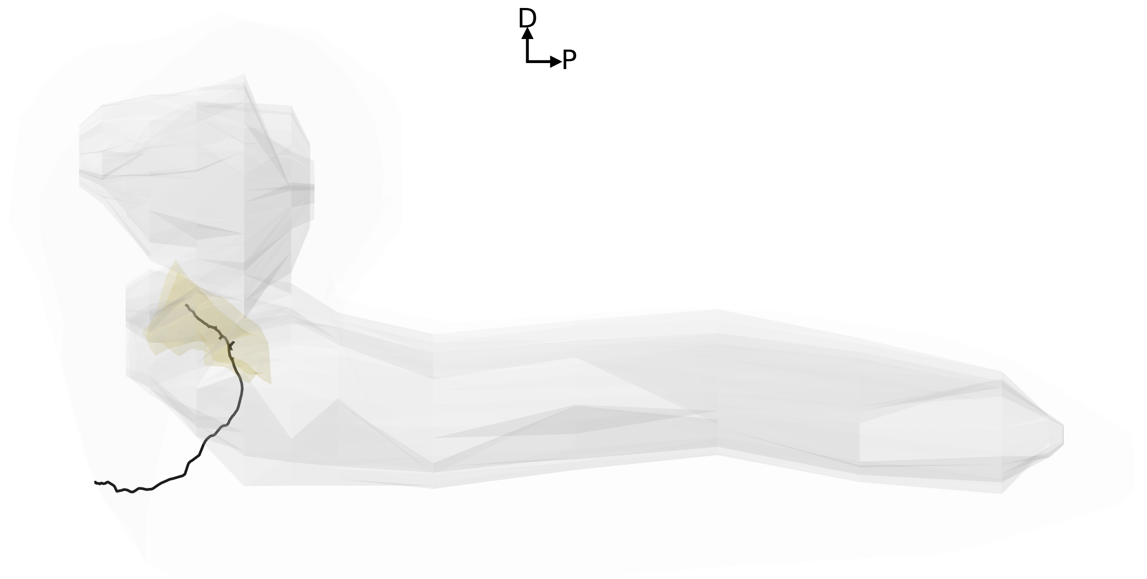

ID	*name*	SCACa	SCAVa	SCAVp	SCACal	SCACp	SCACpl	SCVM	IPCs	DMS	DH44	Se0ens	Se0ph	PMN LR	MN motor neurons	PaN motor neurons	olfactory PNs	gustatory PNs	multiglomerular PNs	unknown PNs	thermo PNs	visual PNs
15716094	MN-L-Sens-B2-ACp-05	0	0	0	0	4	0	0	0	0	0	0	0	0	0	0	0	0	1	0	0	0

ID: 12260696
name: MN-L-Sens-B2-ACp-06

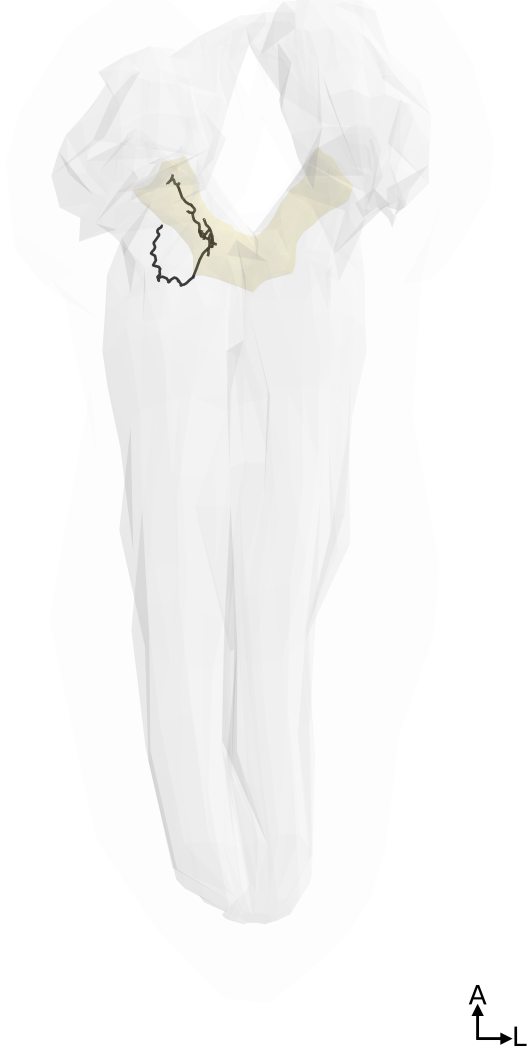

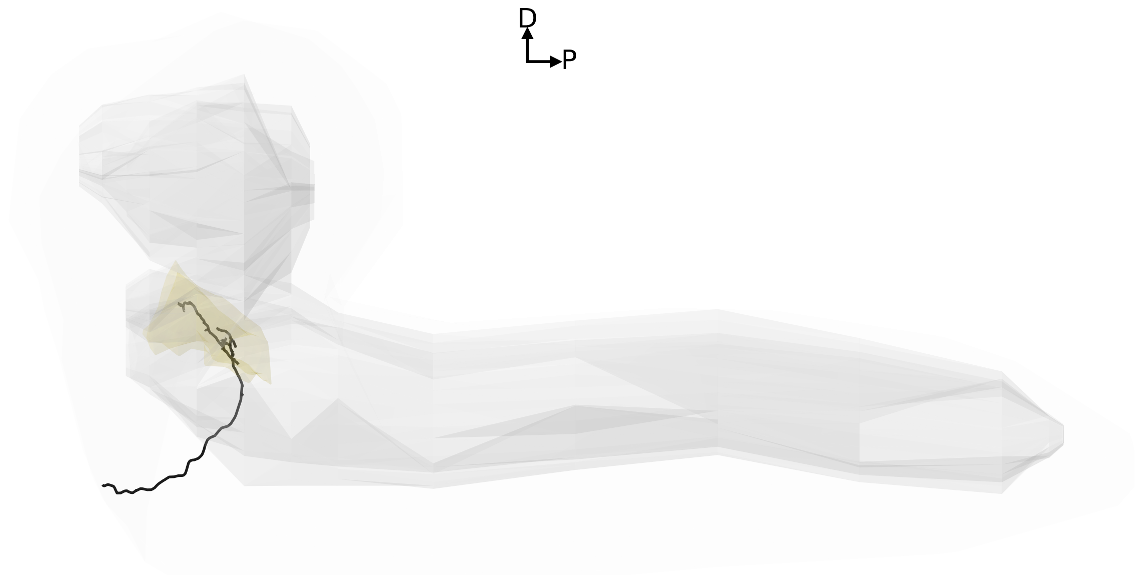

ID	*name*	SCACa	SCAVa	SCAVp	SCACal	SCACp	SCACpl	SCVM	IPCs	DMS	DH44	Se0ens	Se0ph	PMN LR	MN motor neurons	PaN motor neurons	olfactory PNs	gustatory PNs	multiglomerular PNs	unknown PNs	thermo PNs	visual PNs
12260696	MN-L-Sens-B2-ACp-06	0	0	0	0	7	0	0	0	0	0	0	0	0	0	0	0	9	0	0	0	0

ID: 11745381
name: MN-L-Sens-B2-ACp-07

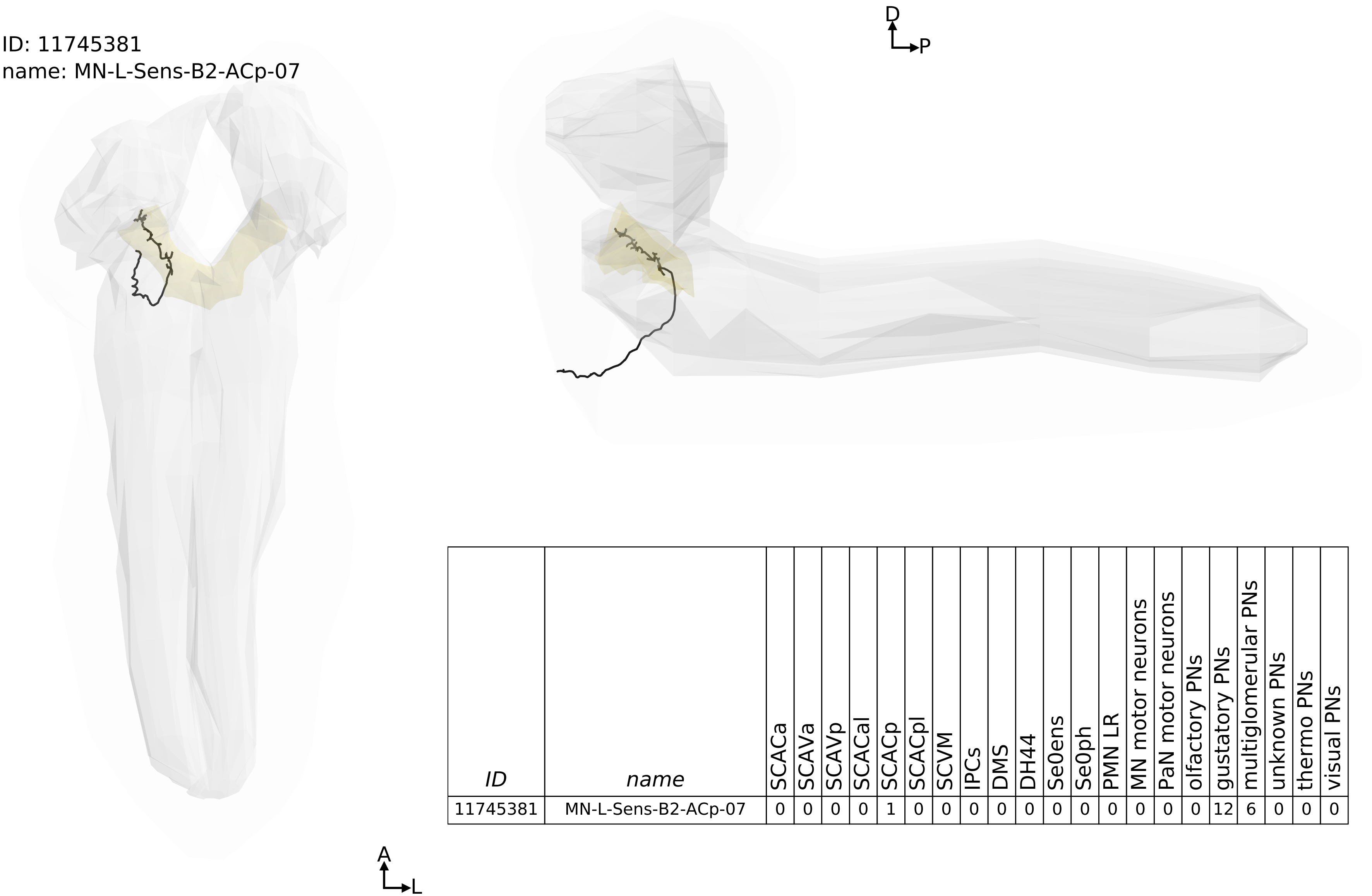

ID	*name*	SCACa	SCAVa	SCAVp	SCACal	SCACp	SCACpl	SCVM	IPCs	DMS	DH44	Se0ens	Se0ph	PMN LR	MN motor neurons	PaN motor neurons	olfactory PNs	gustatory PNs	multiglomerular PNs	unknown PNs	thermo PNs	visual PNs
11745381	MN-L-Sens-B2-ACp-07	0	0	0	0	1	0	0	0	0	0	0	0	0	0	0	0	12	6	0	0	0

ID: 15938337
name: MN-L-Sens-B2-ACp-08

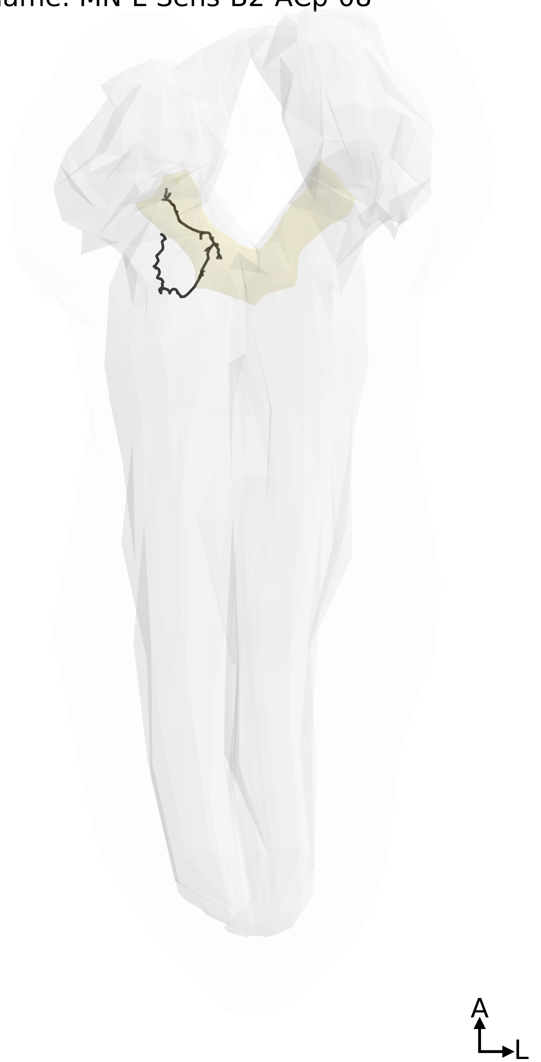

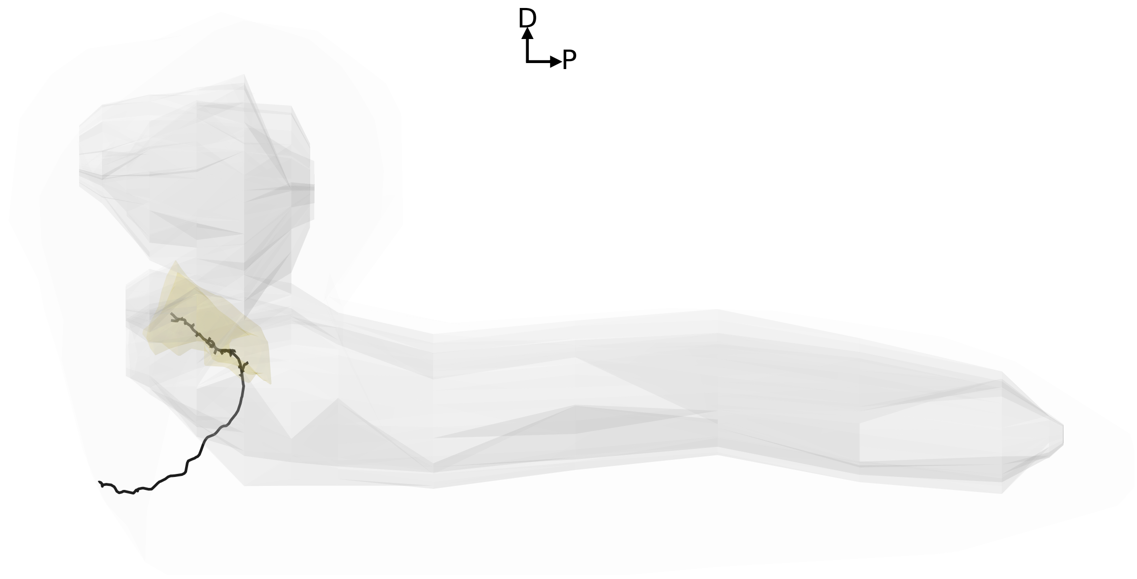

ID	name	SCACa	SCAVa	SCAVp	SCACal	SCACp	SCACpl	SCVM	IPCs	DMS	DH44	Se0ens	Se0ph	PMN LR	MN motor neurons	PaN motor neurons	olfactory PNs	gustatory PNs	multiglomerular PNs	unknown PNs	thermo PNs	visual PNs
15938337	MN-L-Sens-B2-ACp-08	0	0	0	0	5	0	0	0	0	0	0	0	0	0	0	1	1	3	0	0	0

ID: 15690093
name: MN-L-Sens-B2-ACp-09

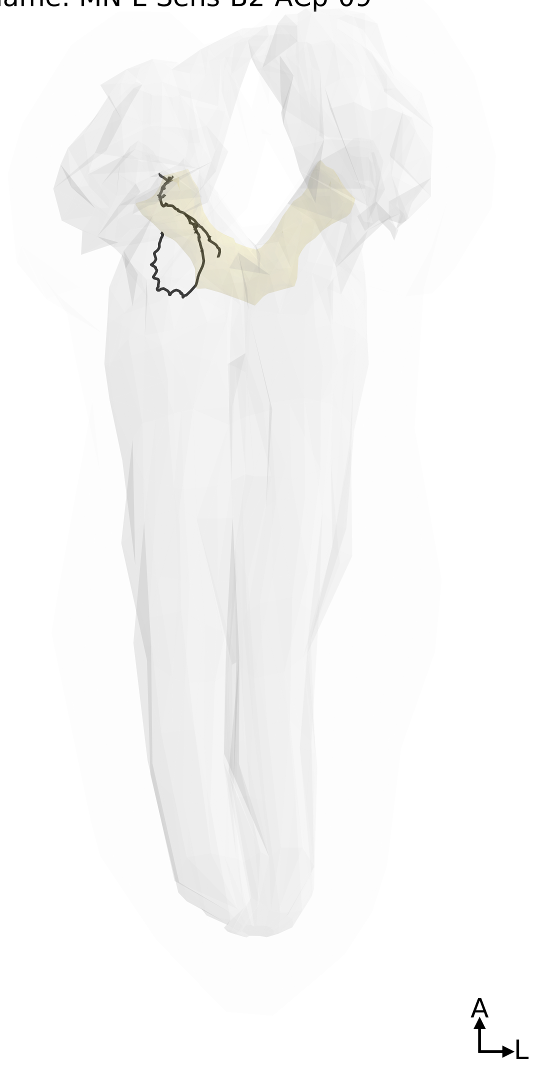

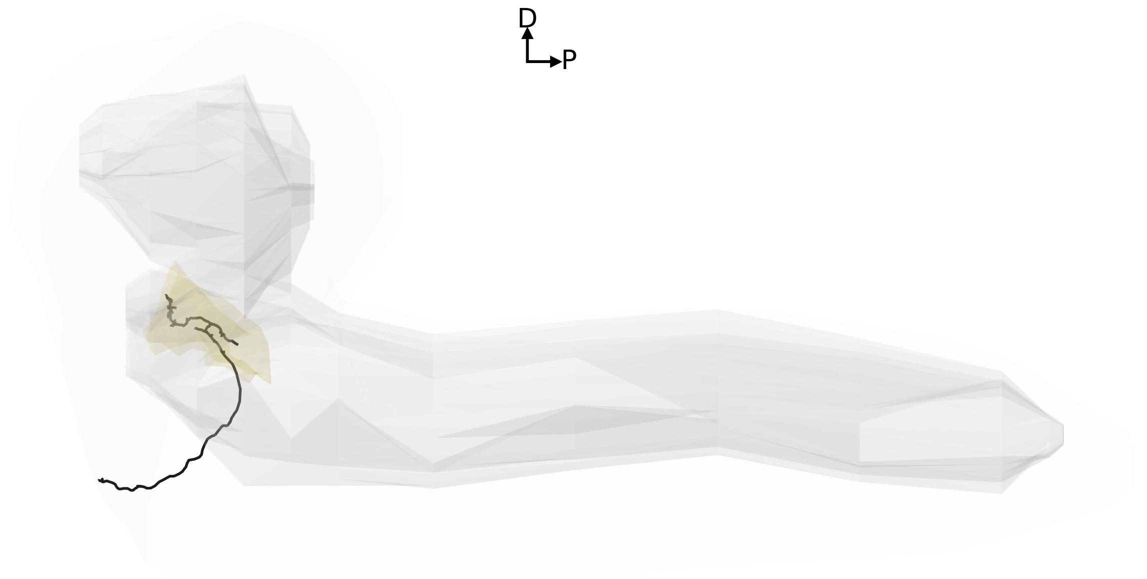

ID	name	SCACa	SCAVa	SCAVp	SCACal	SCACp	SCACpl	SCVM	IPCs	DMS	DH44	Se0ens	Se0ph	PMN LR	MN motor neurons	PaN motor neurons	olfactory PNs	gustatory PNs	multiglomerular PNs	unknown PNs	thermo PNs	visual PNs
15690093	MN-L-Sens-B2-ACp-09	0	0	0	0	7	0	0	0	0	0	0	0	0	0	0	2	33	8	0	0	0

ID: 15679577
name: MN-L-Sens-B2-ACp-10

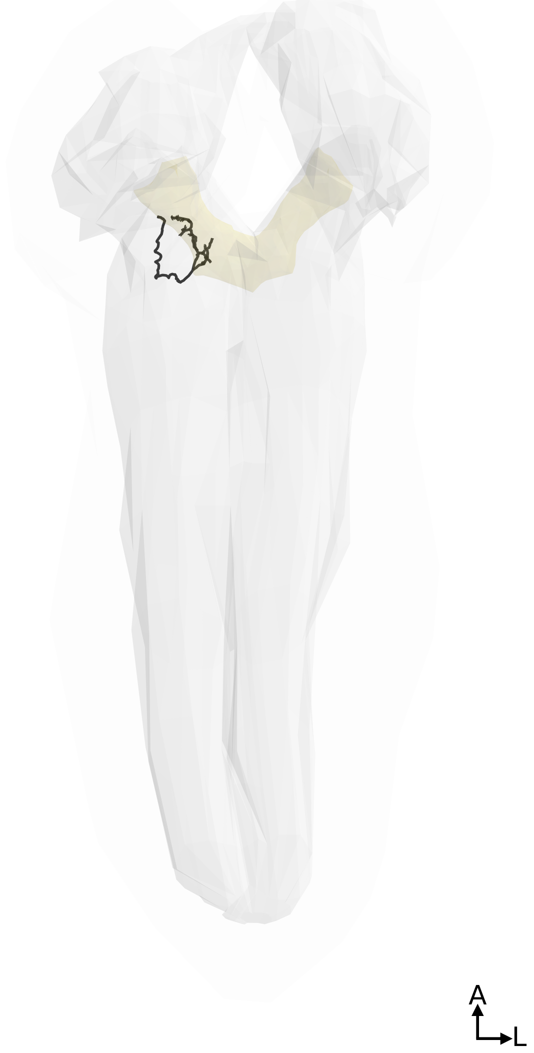

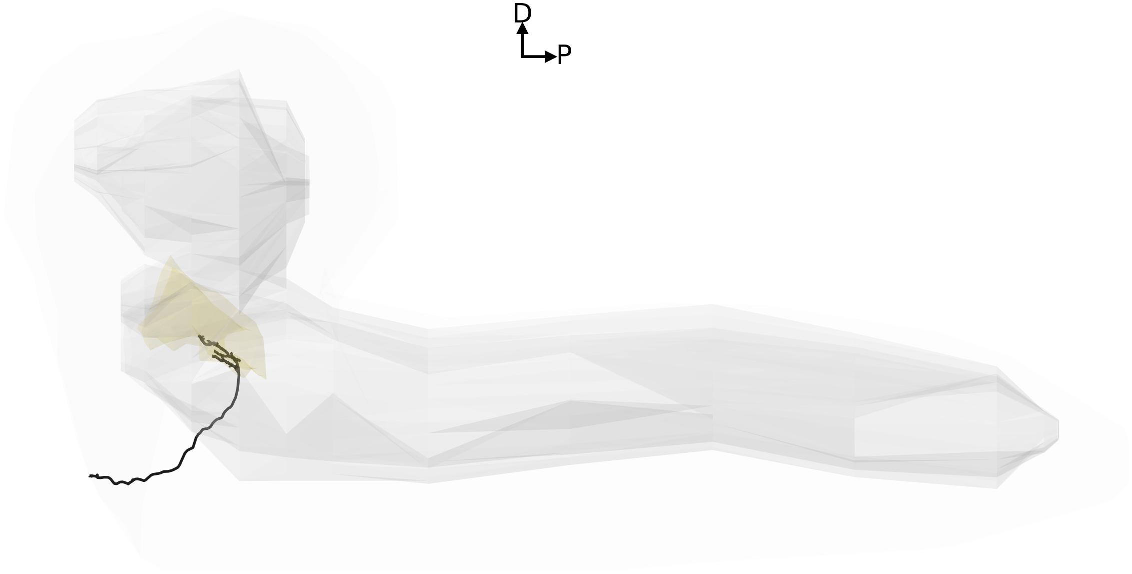

ID	*name*	SCACa	SCAVa	SCAVp	SCACal	SCACp	SCACpl	SCVM	IPCs	DMS	DH44	Se0ens	Se0ph	PMN LR	MN motor neurons	PaN motor neurons	olfactory PNs	gustatory PNs	multiglomerular PNs	unknown PNs	thermo PNs	visual PNs
15679577	MN-L-Sens-B2-ACp-10	0	0	0	0	1	0	0	0	0	0	0	0	0	0	0	0	0	31	0	0	0

ID: 15674262
name: MN-L-Sens-B2-ACp-11

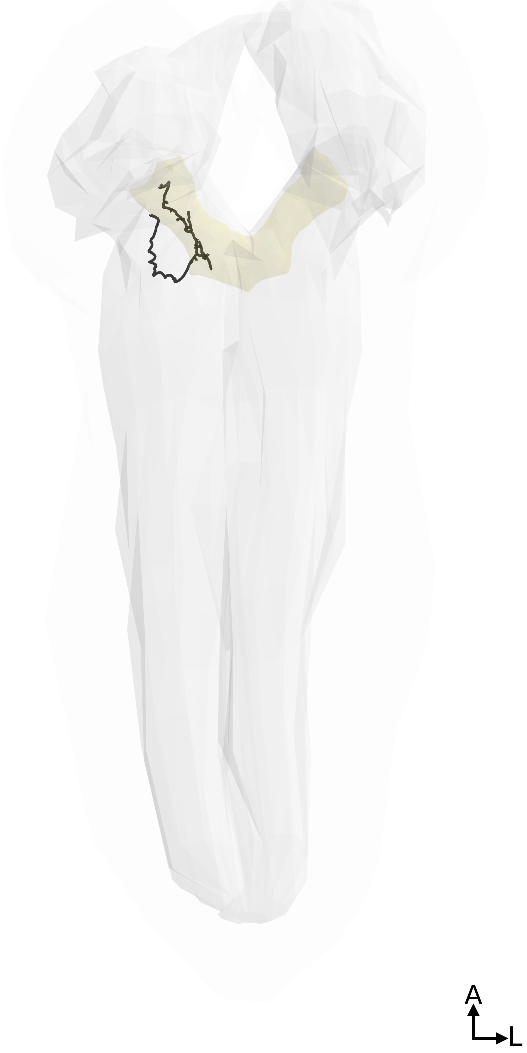

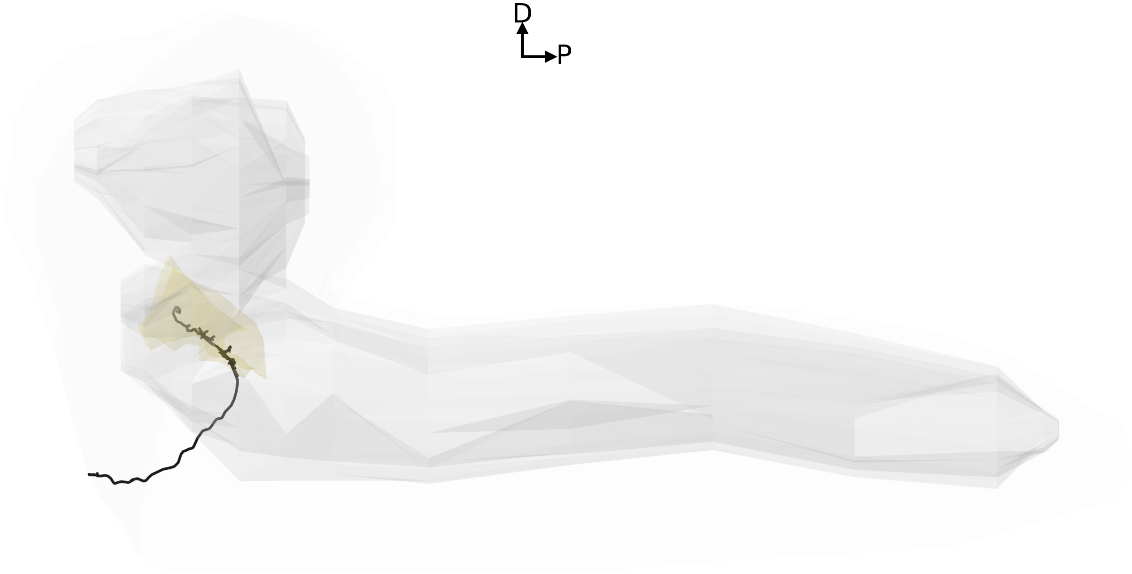

ID	*name*	SCACa	SCAVa	SCAVp	SCACal	SCACp	SCACpl	SCVM	IPCs	DMS	DH44	Se0ens	Se0ph	PMN LR	MN motor neurons	PaN motor neurons	olfactory PNs	gustatory PNs	multiglomerular PNs	unknown PNs	thermo PNs	visual PNs
15674262	MN-L-Sens-B2-ACp-11	0	0	0	0	0	0	0	0	0	0	0	0	0	0	0	0	2	25	0	0	0

ID: 15689373
name: MN-L-Sens-B2-ACp-12

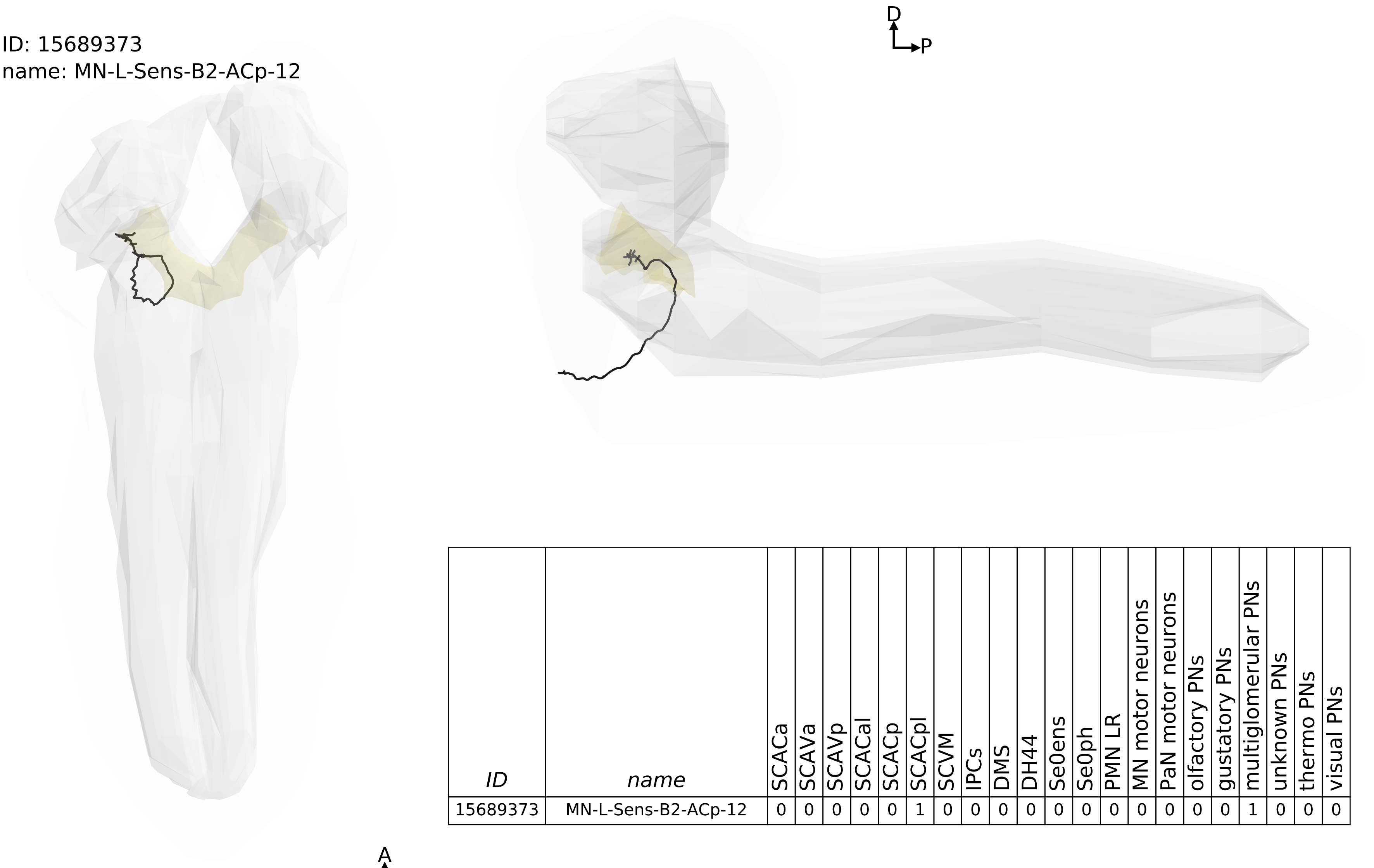

ID	name	SCACa	SCAVa	SCAVp	SCACal	SCACp	SCACpl	SCVM	IPCs	DMS	DH44	Se0ens	Se0ph	PMN LR	MN motor neurons	PaN motor neurons	olfactory PNs	gustatory PNs	multiglomerular PNs	unknown PNs	thermo PNs	visual PNs
15689373	MN-L-Sens-B2-ACp-12	0	0	0	0	0	1	0	0	0	0	0	0	0	0	0	0	0	1	0	0	0

ID: 15716127
name: MN-L-Sens-B2-ACp-13

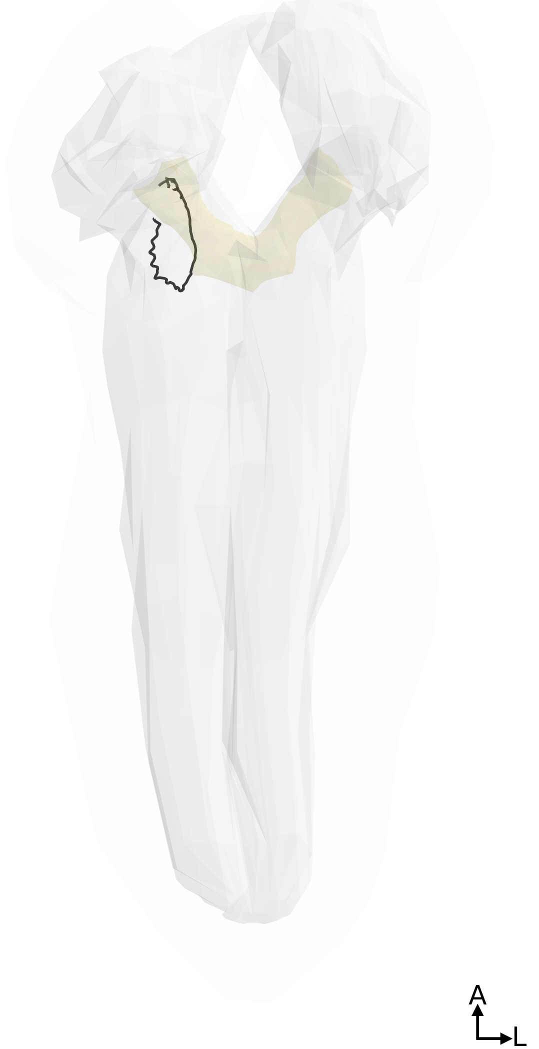

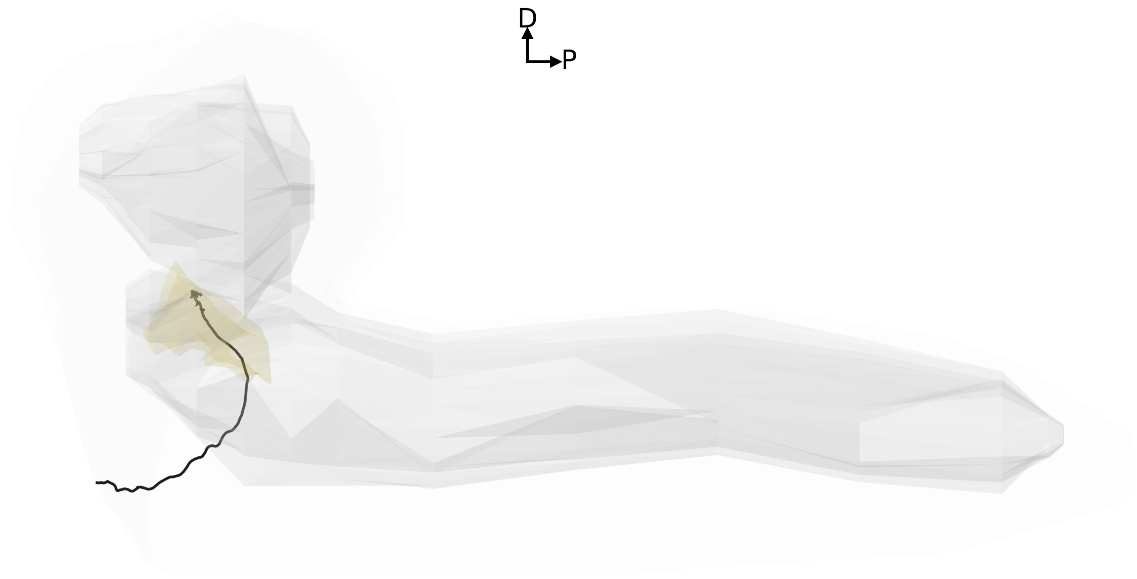

ID	name	SCACa	SCAVa	SCAVp	SCACal	SCACp	SCACpl	SCVM	IPCs	DMS	DH44	Se0ens	Se0ph	PMN LR	MN motor neurons	PaN motor neurons	olfactory PNs	gustatory PNs	multiglomerular PNs	unknown PNs	thermo PNs	visual PNs
15716127	MN-L-Sens-B2-ACp-13	0	0	0	0	0	0	0	0	0	0	0	0	0	0	0	0	0	7	0	0	0

ID: 15686512
name: MN-L-Sens-B2-ACp-14

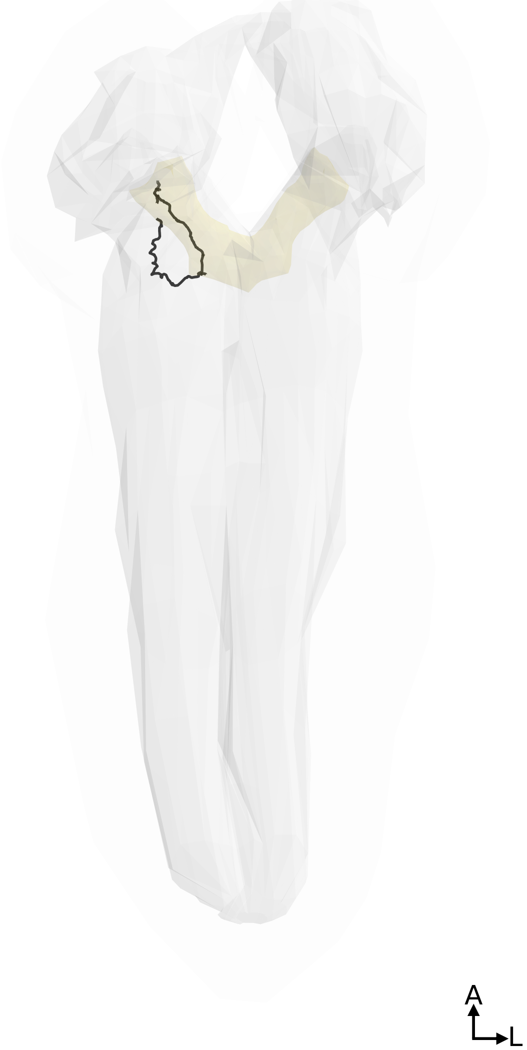

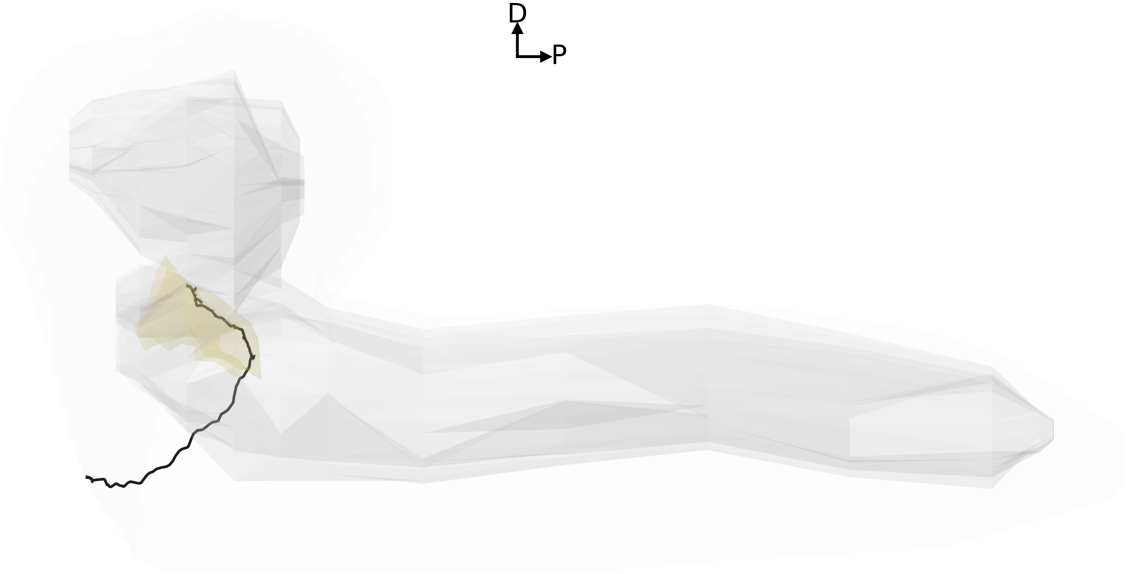

ID	*name*	SCACa	SCAVa	SCAVp	SCACal	SCACp	SCACpl	SCVM	IPCs	DMS	DH44	Se0ens	Se0ph	PMN LR	MN motor neurons	PaN motor neurons	olfactory PNs	gustatory PNs	multiglomerular PNs	unknown PNs	thermo PNs	visual PNs
15686512	MN-L-Sens-B2-ACp-14	0	0	0	0	2	0	0	0	0	0	0	0	0	0	0	0	0	6	0	0	0

ID: 11736972
name: MN-L-Sens-B2-ACp-15

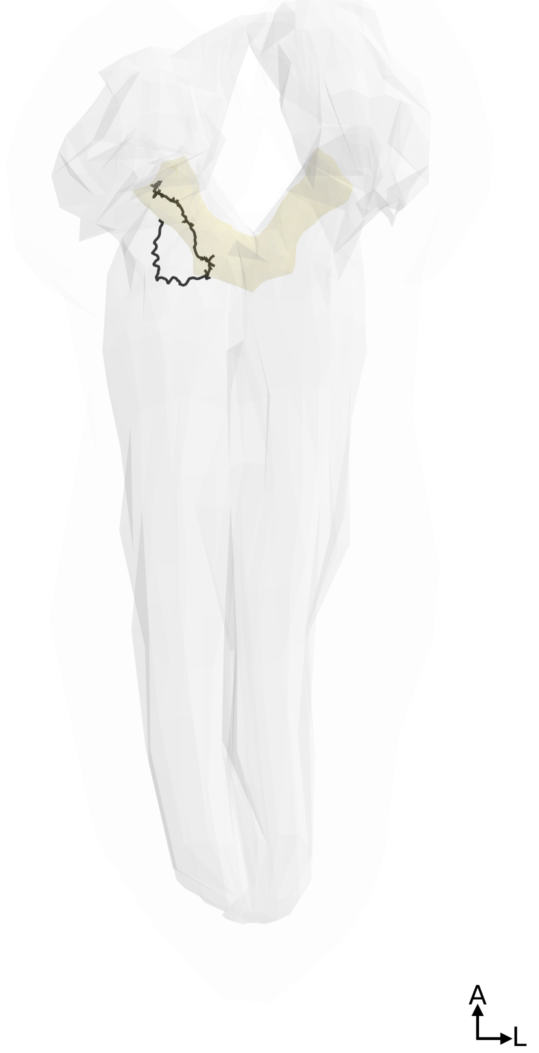

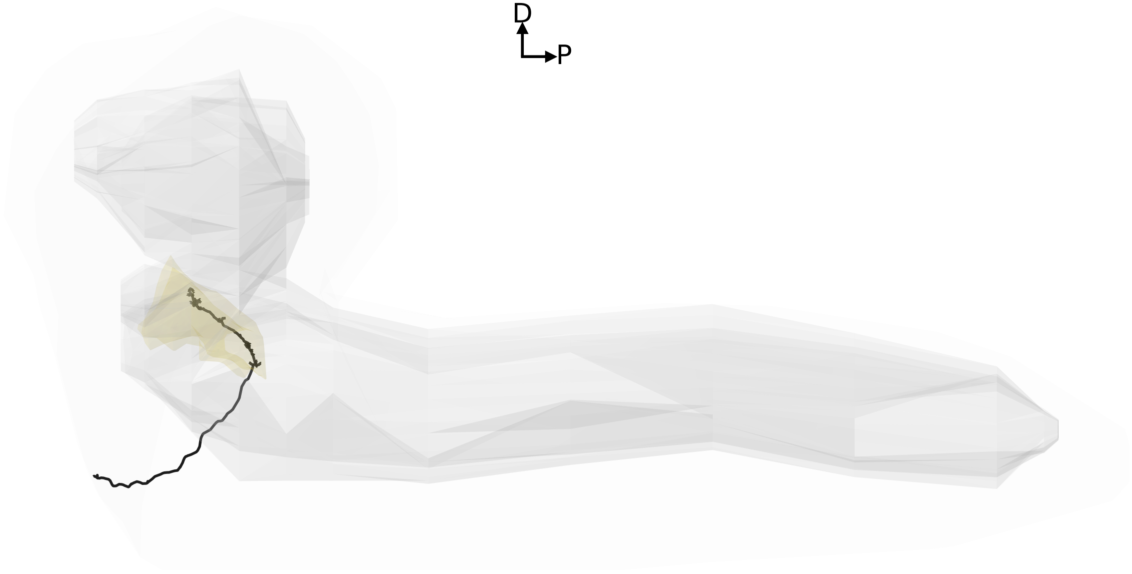

ID	*name*	SCACa	SCAVa	SCAVp	SCACal	SCACp	SCACpl	SCVM	IPCs	DMS	DH44	Se0ens	Se0ph	PMN LR	MN motor neurons	PaN motor neurons	olfactory PNs	gustatory PNs	multiglomerular PNs	unknown PNs	thermo PNs	visual PNs
11736972	MN-L-Sens-B2-ACp-15	0	0	0	0	0	0	0	0	0	0	0	0	0	0	0	1	0	2	0	0	0

ID: 12073881
name: MN-L-Sens-B2-ACp-16

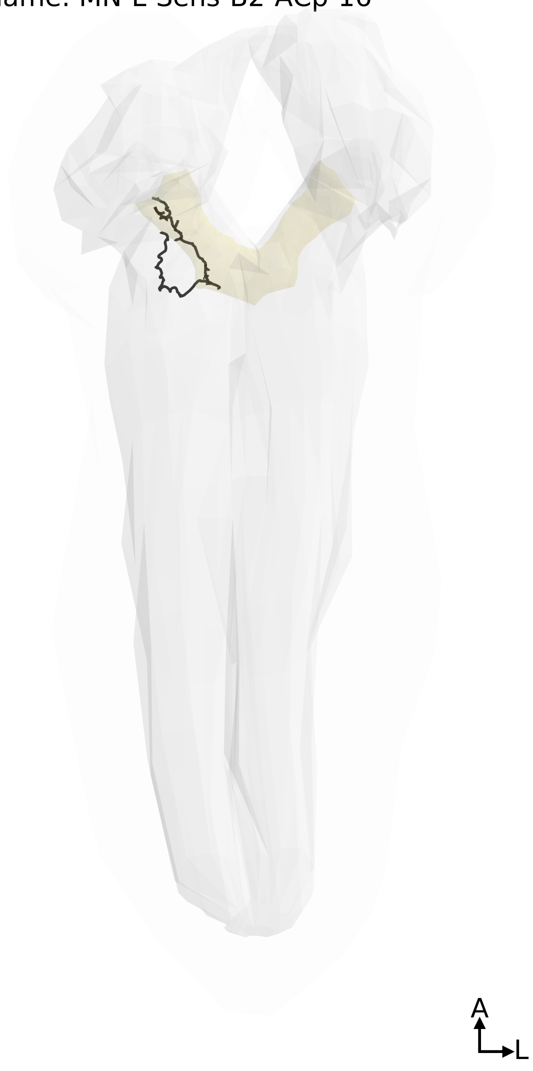

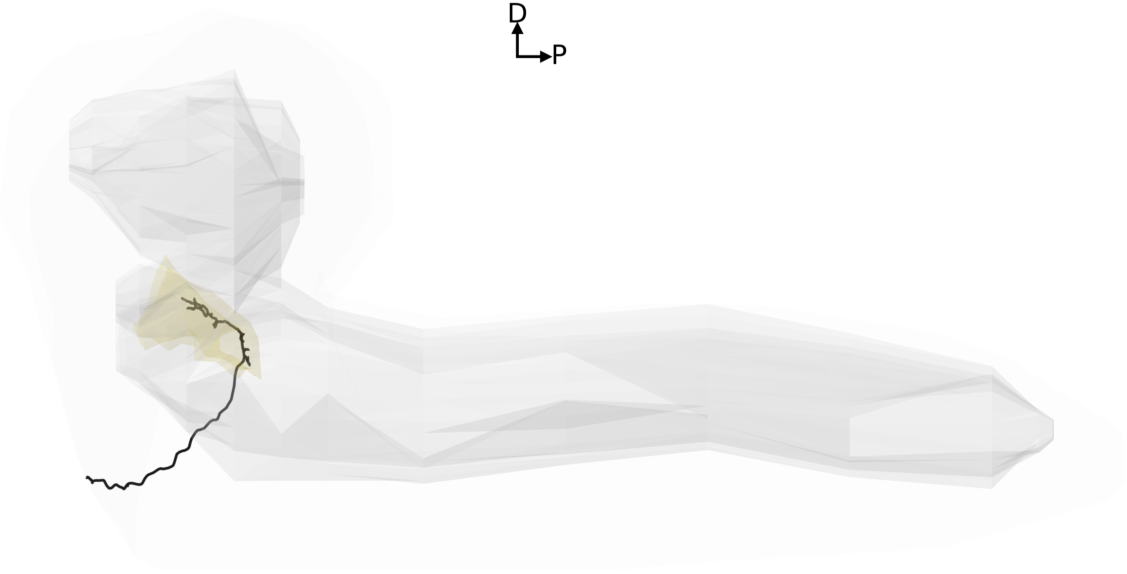

ID	*name*	SCACa	SCAVa	SCAVp	SCACal	SCACp	SCACpl	SCVM	IPCs	DMS	DH44	Se0ens	Se0ph	PMN LR	MN motor neurons	PaN motor neurons	olfactory PNs	gustatory PNs	multiglomerular PNs	unknown PNs	thermo PNs	visual PNs
12073881	MN-L-Sens-B2-ACp-16	0	0	0	0	3	0	0	0	0	0	0	0	0	0	0	4	0	5	0	0	0

ID: 18934019
name: MN-L-Sens-B2-ACp-17

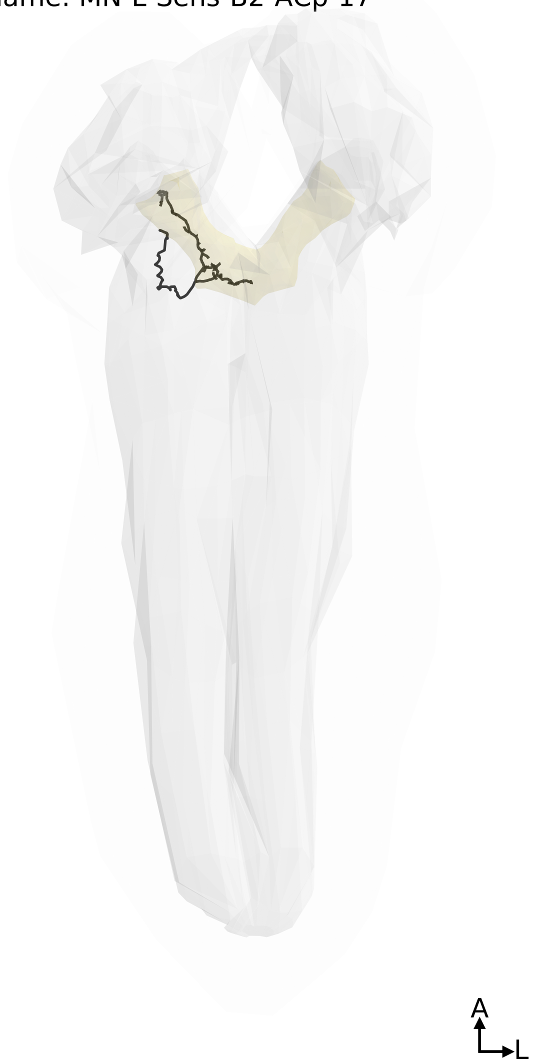

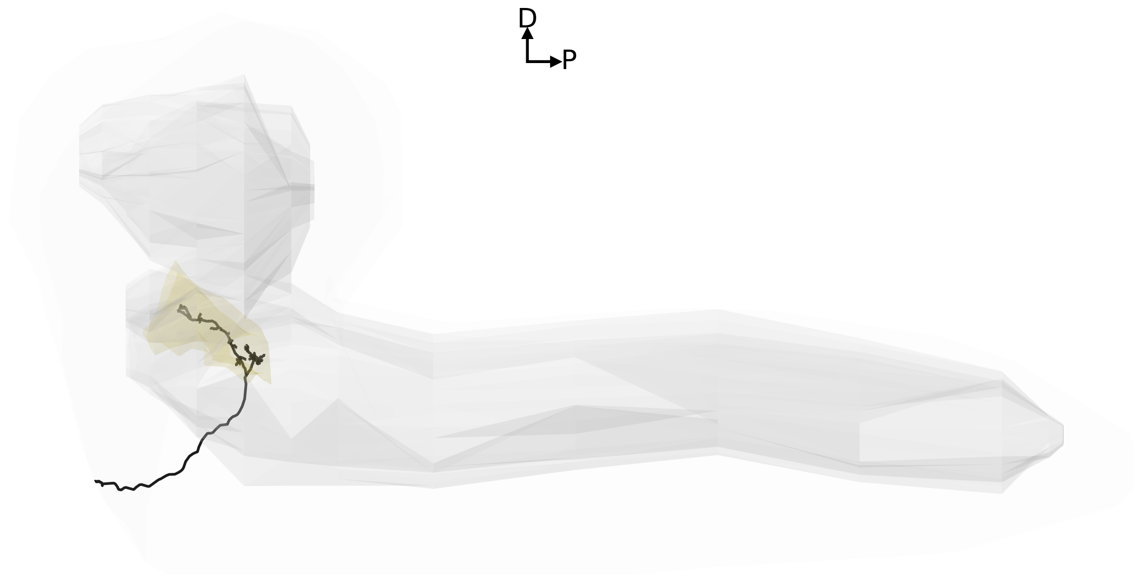

ID	name	SCACa	SCAVa	SCAVp	SCACal	SCACp	SCACpl	SCVM	IPCs	DMS	DH44	Se0ens	Se0ph	PMN LR	MN motor neurons	PaN motor neurons	olfactory PNs	gustatory PNs	multiglomerular PNs	unknown PNs	thermo PNs	visual PNs
18934019	MN-L-Sens-B2-ACp-17	0	0	0	0	2	0	0	0	0	0	0	0	0	0	0	1	5	0	1	0	0

ID: 15677323
name: MN-L-Sens-B2-ACp-18

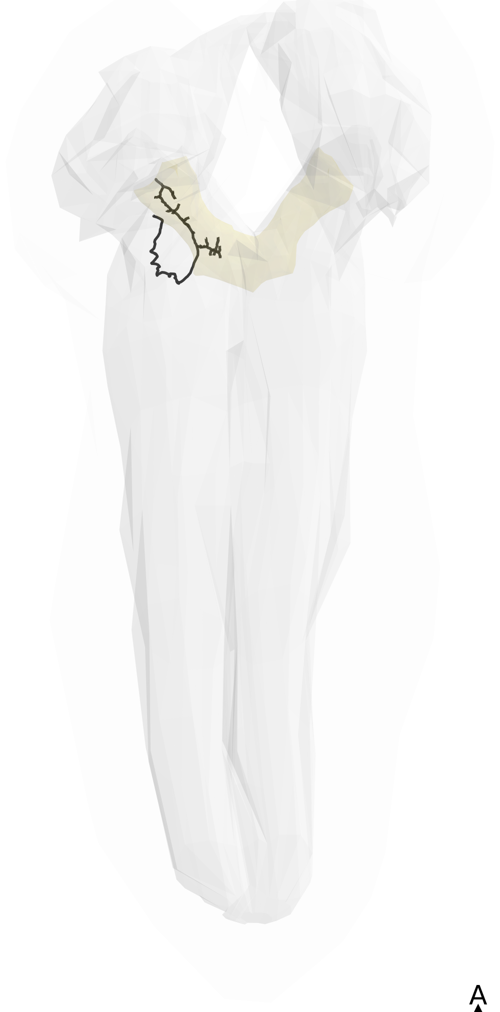

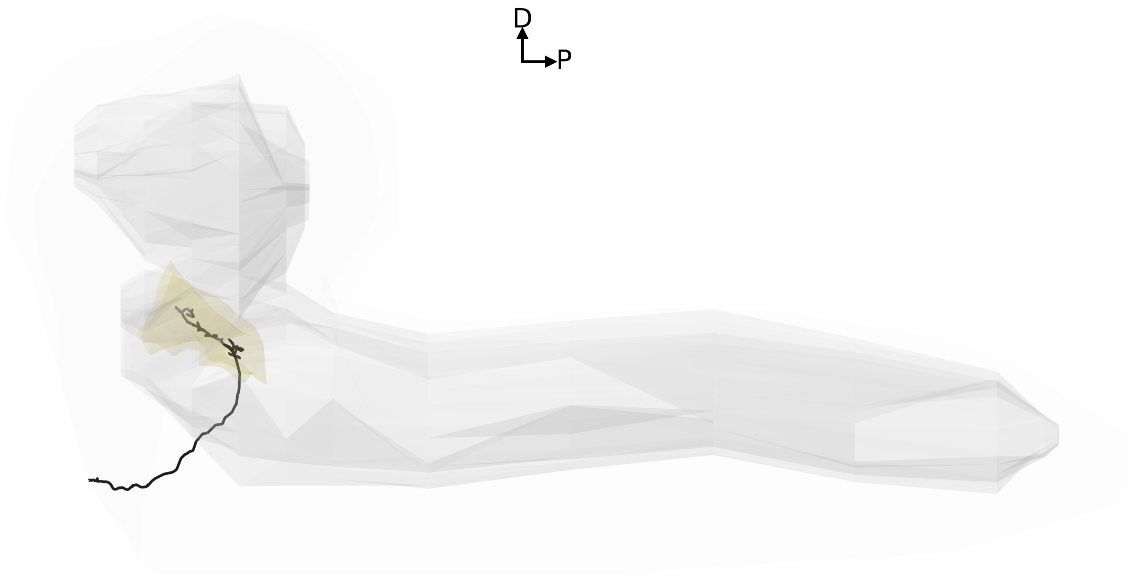

ID	*name*	SCACa	SCAVa	SCAVp	SCACal	SCACp	SCACpl	SCVM	IPCs	DMS	DH44	Se0ens	Se0ph	PMN LR	MN motor neurons	PaN motor neurons	olfactory PNs	gustatory PNs	multiglomerular PNs	unknown PNs	thermo PNs	visual PNs
15677323	MN-L-Sens-B2-ACp-18	0	0	0	0	1	0	0	0	0	0	0	0	0	0	0	0	0	0	1	0	0

ID: 15680093
name: MN-L-Sens-B2-ACp-19

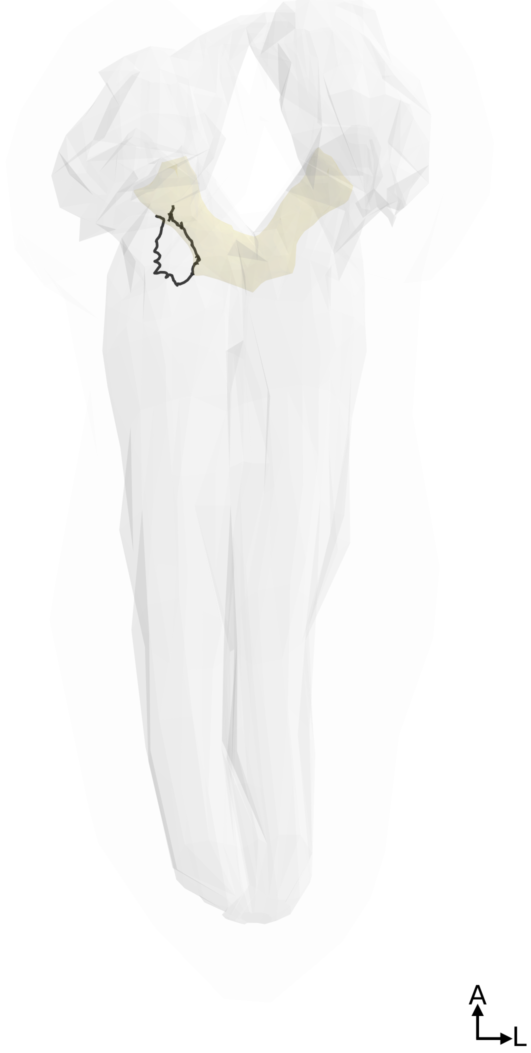

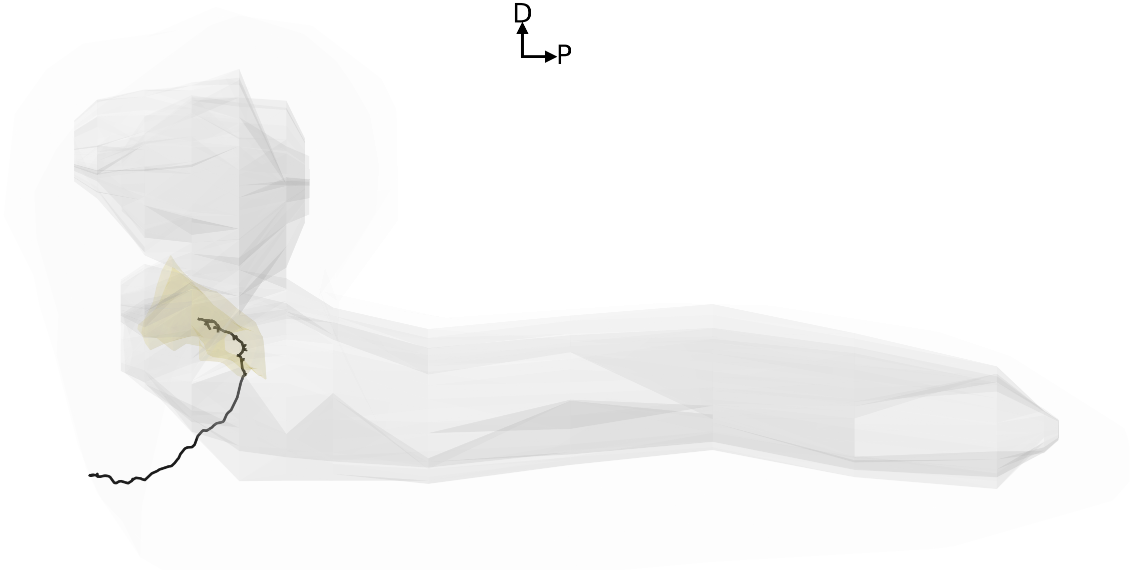

ID	name	SCACa	SCAVa	SCAVp	SCACal	SCACp	SCACpl	SCVM	IPCs	DMS	DH44	Se0ens	Se0ph	PMN LR	MN motor neurons	PaN motor neurons	olfactory PNs	gustatory PNs	multiglomerular PNs	unknown PNs	thermo PNs	visual PNs
15680093	MN-L-Sens-B2-ACp-19	0	0	0	0	2	0	0	0	0	0	0	0	0	0	0	0	0	0	0	0	0

ID: 15688358
name: MN-L-Sens-B2-ACp-20

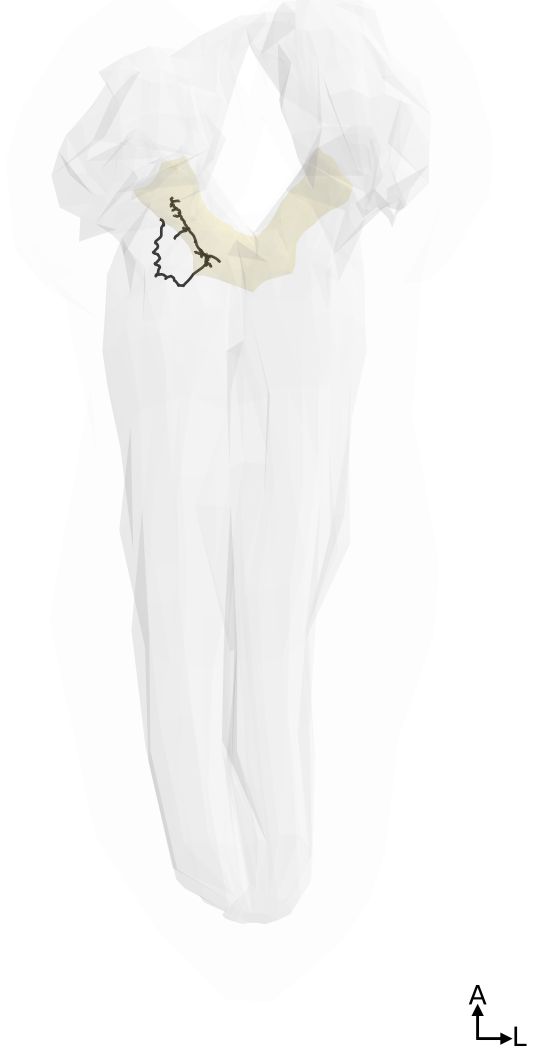

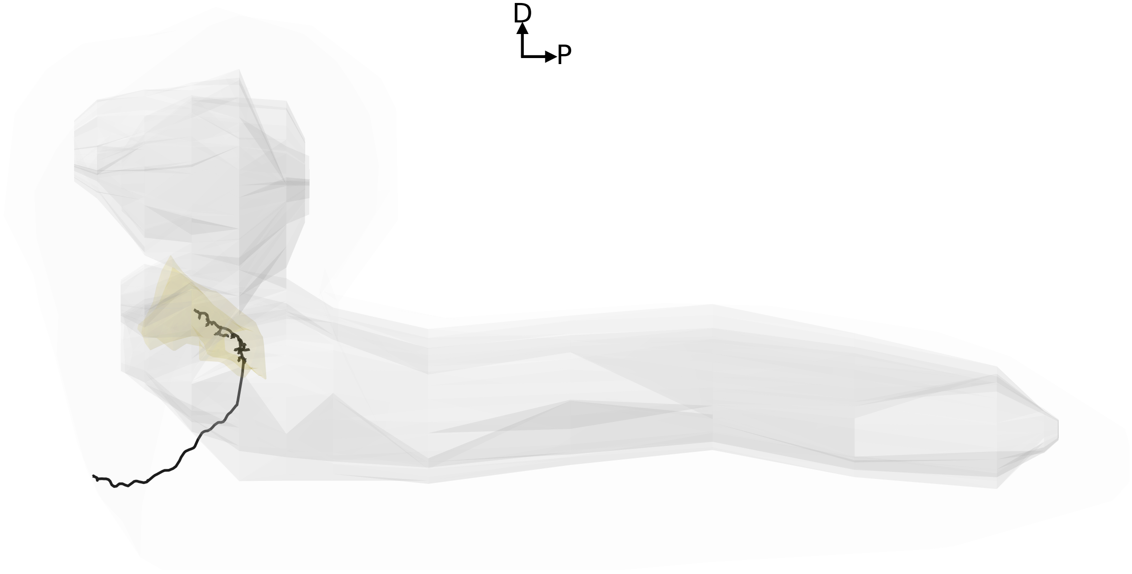

ID	name	SCACa	SCAVa	SCAVp	SCACal	SCACp	SCACpl	SCVM	IPCs	DMS	DH44	Se0ens	Se0ph	PMN LR	MN motor neurons	PaN motor neurons	olfactory PNs	gustatory PNs	multiglomerular PNs	unknown PNs	thermo PNs	visual PNs
15688358	MN-L-Sens-B2-ACp-20	0	0	0	0	3	0	0	0	0	0	0	0	0	0	0	2	0	1	0	0	0

ID: 15681740
name: MN-L-Sens-B2-ACp-21

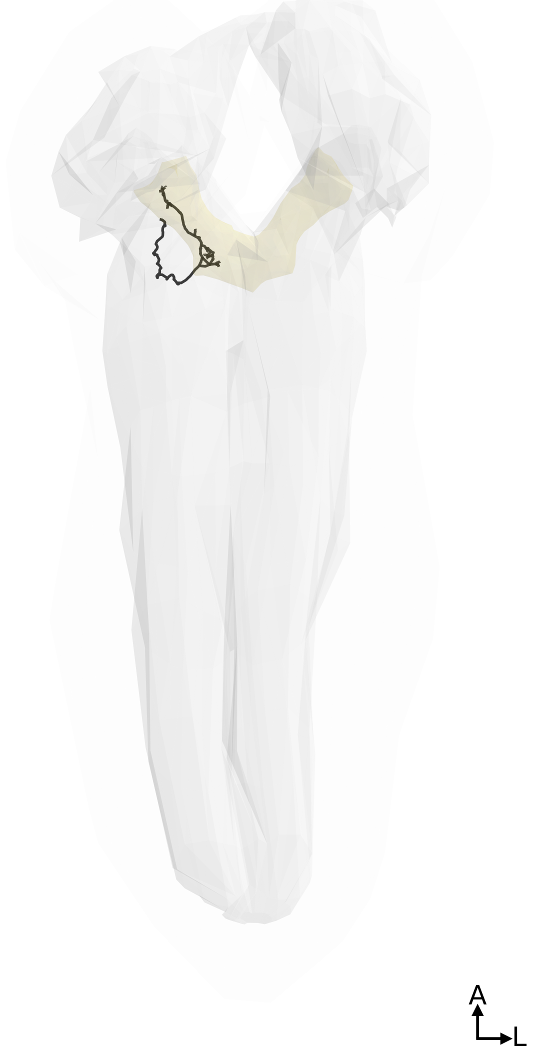

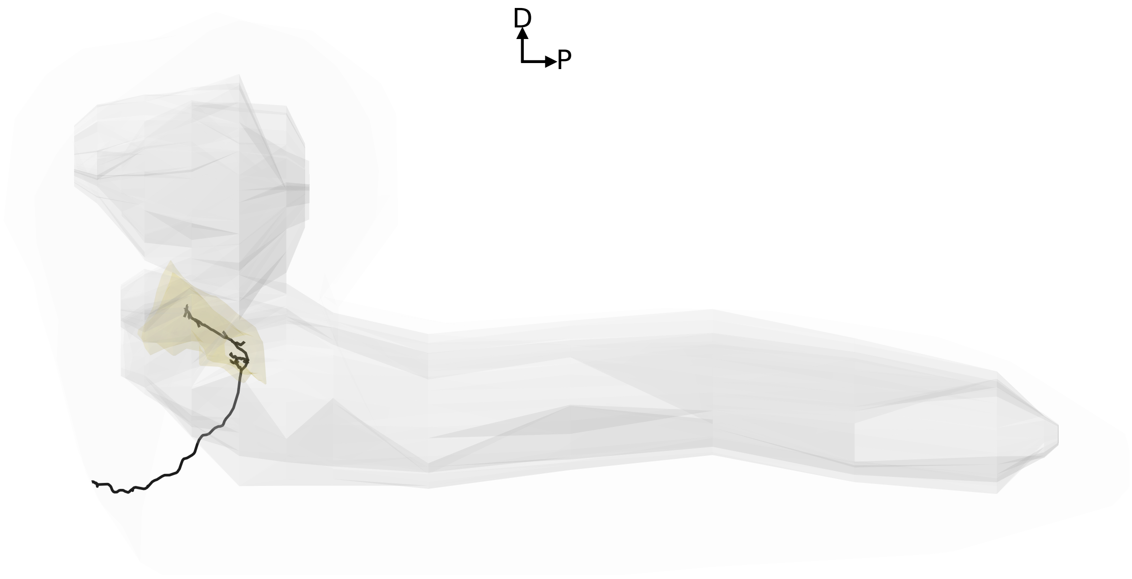

ID	*name*	SCACa	SCAVa	SCAVp	SCACal	SCACp	SCACpl	SCVM	IPCs	DMS	DH44	Se0ens	Se0ph	PMN LR	MN motor neurons	PaN motor neurons	olfactory PNs	gustatory PNs	multiglomerular PNs	unknown PNs	thermo PNs	visual PNs
15681740	MN-L-Sens-B2-ACp-21	0	0	0	0	2	0	0	0	0	0	0	0	0	0	0	2	0	0	0	0	0

ID: 15673867
name: MN-L-Sens-B2-ACp-22

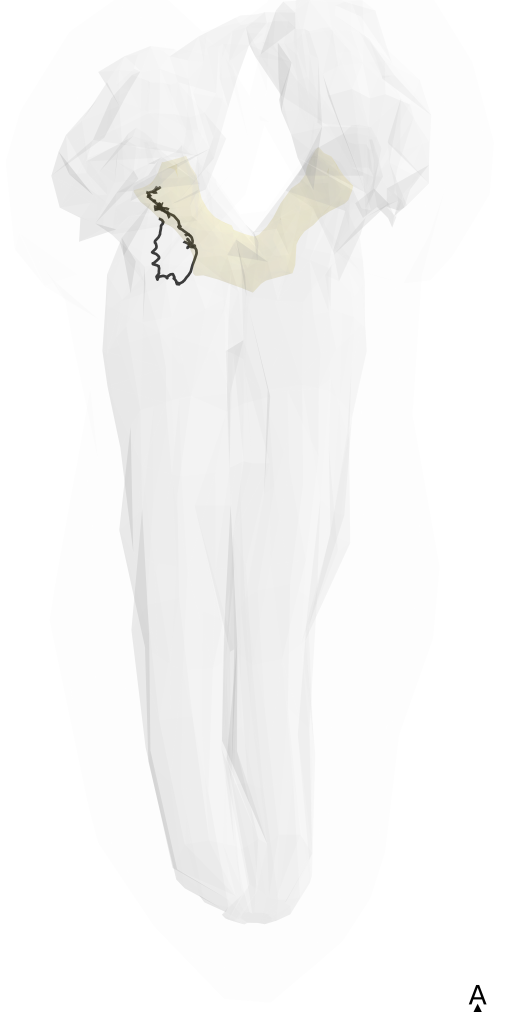

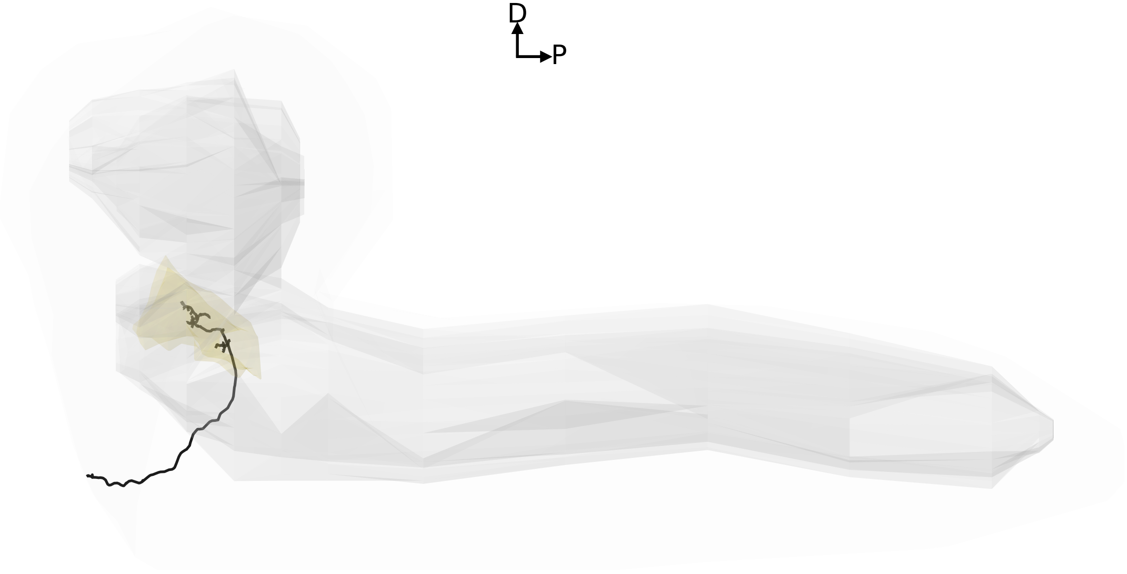

ID	name	SCACa	SCAVa	SCAVp	SCACal	SCACp	SCACpl	SCVM	IPCs	DMS	DH44	Se0ens	Se0ph	PMN LR	MN motor neurons	PaN motor neurons	olfactory PNs	gustatory PNs	multiglomerular PNs	unknown PNs	thermo PNs	visual PNs
15673867	MN-L-Sens-B2-ACp-22	0	0	0	0	0	0	0	0	0	0	0	0	0	0	0	0	0	0	0	0	0

ID: 15723727
name: MN-L-Sens-B3-ACp-01

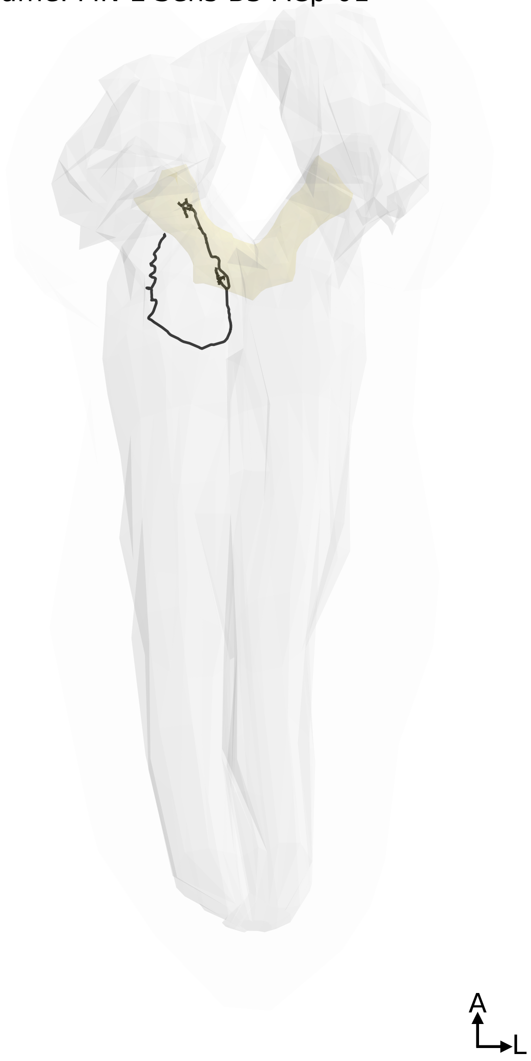

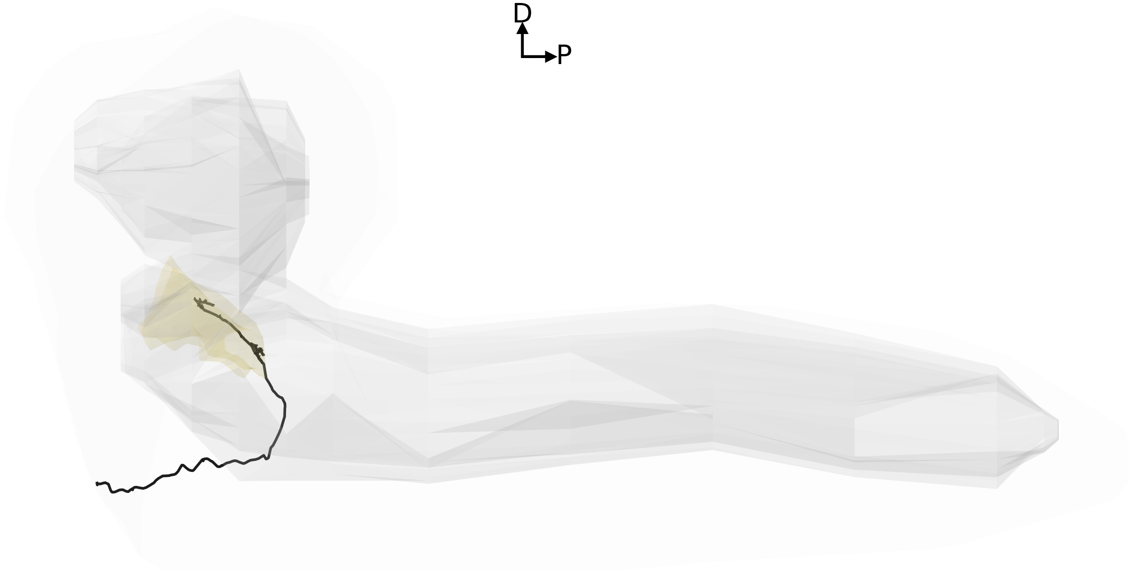

ID	*name*	SCACa	SCAVa	SCAVp	SCACal	SCACp	SCACpl	SCVM	IPCs	DMS	DH44	Se0ens	Se0ph	PMN LR	MN motor neurons	PaN motor neurons	olfactory PNs	gustatory PNs	multiglomerular PNs	unknown PNs	thermo PNs	visual PNs
15723727	MN-L-Sens-B3-ACp-01	0	0	0	0	15	0	0	0	0	0	0	0	0	0	0	2	0	0	0	0	0

ID: 16288872
name: MN-L-Sens-B3-ACp-02

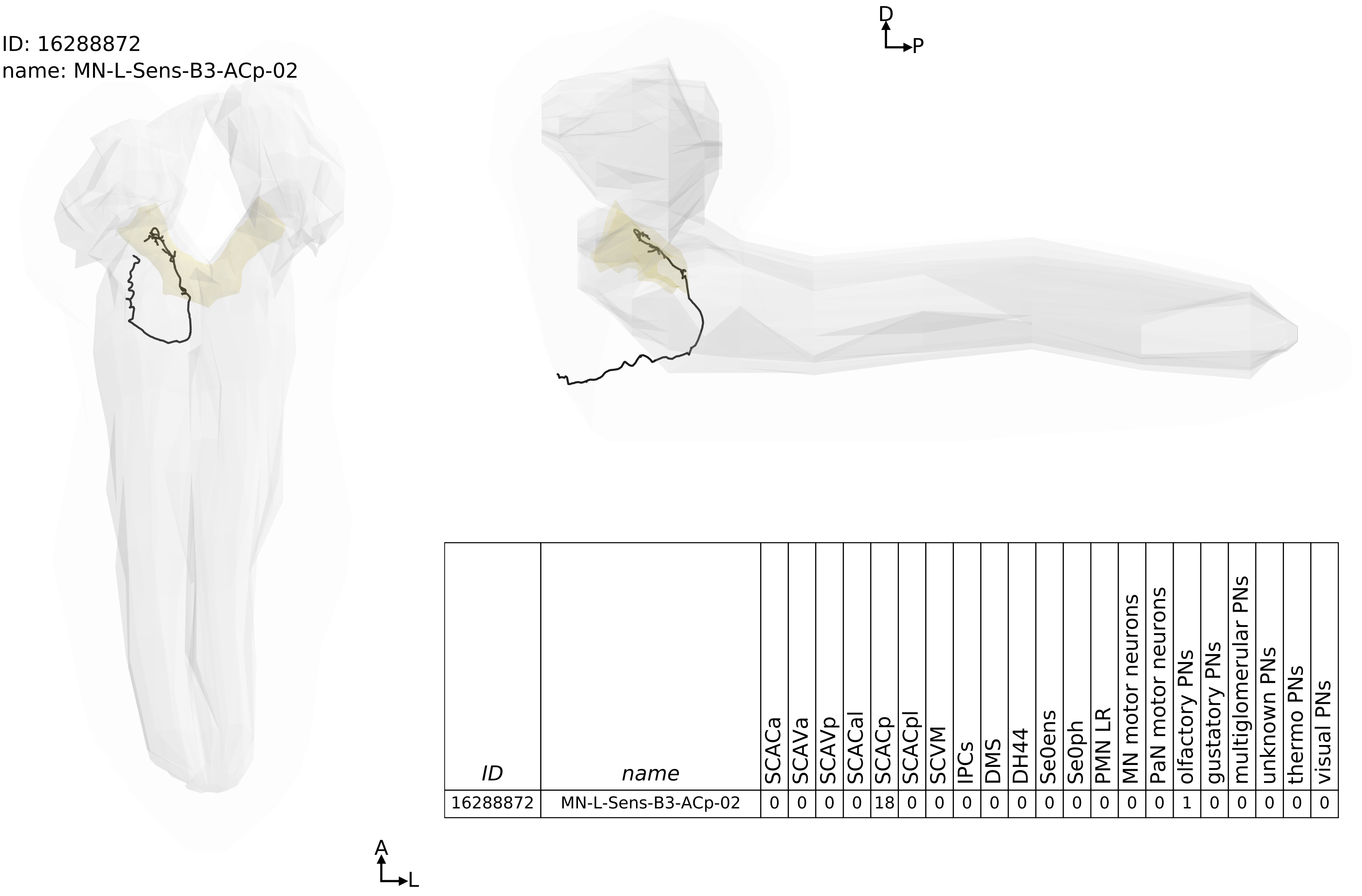

ID	*name*	SCACa	SCAVa	SCAVp	SCACal	SCACp	SCACpl	SCVM	IPCs	DMS	DH44	Se0ens	Se0ph	PMN LR	MN motor neurons	PaN motor neurons	olfactory PNs	gustatory PNs	multiglomerular PNs	unknown PNs	thermo PNs	visual PNs
16288872	MN-L-Sens-B3-ACp-02	0	0	0	0	18	0	0	0	0	0	0	0	0	0	0	1	0	0	0	0	0

ID: 7556564
name: MN-L-Sens-B3-ACp-03

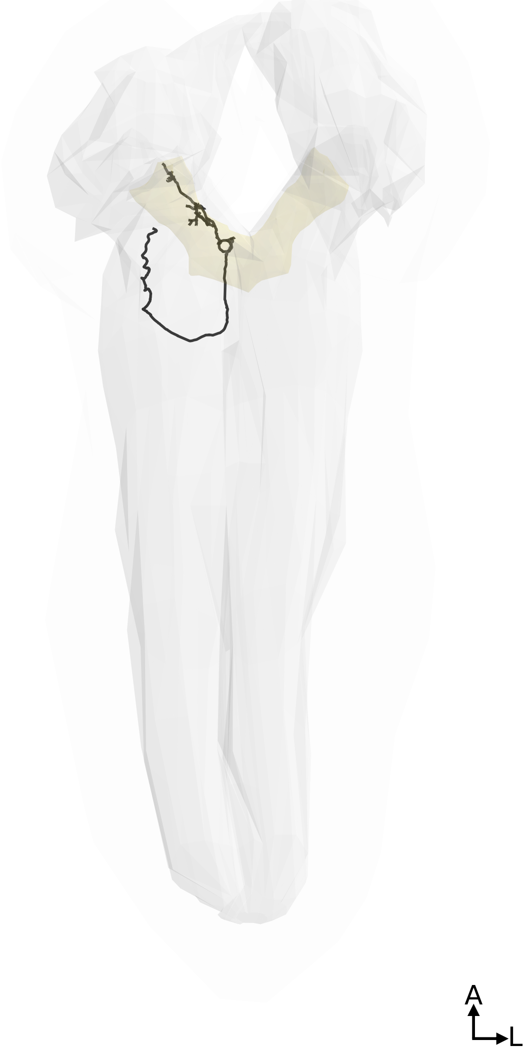

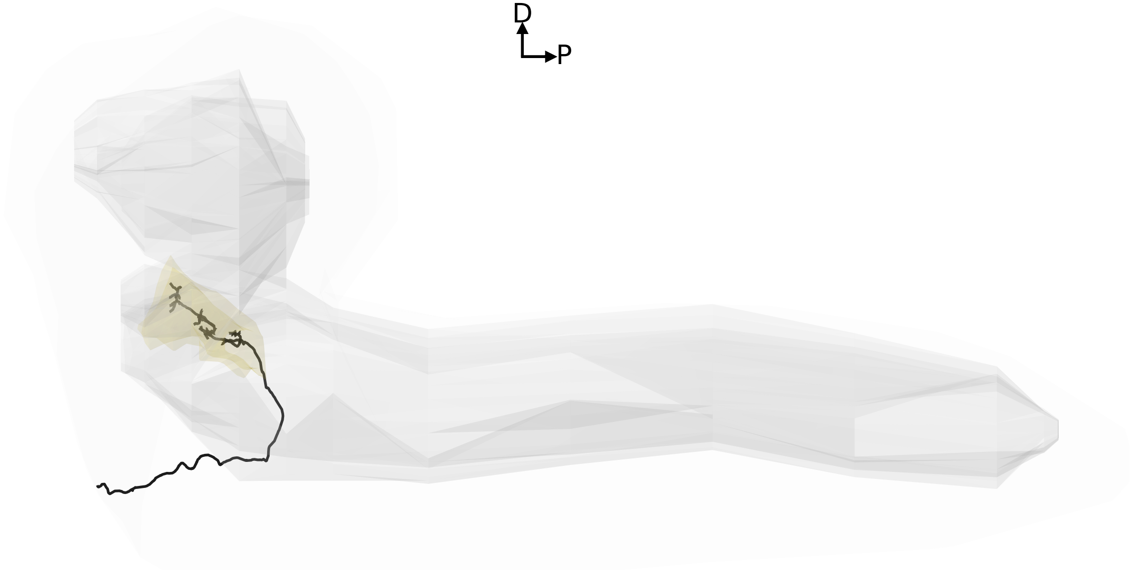

ID	*name*	SCACa	SCAVa	SCAVp	SCACal	SCACp	SCACpl	SCVM	IPCs	DMS	DH44	Se0ens	Se0ph	PMN LR	MN motor neurons	PaN motor neurons	olfactory PNs	gustatory PNs	multiglomerular PNs	unknown PNs	thermo PNs	visual PNs
7556564	MN-L-Sens-B3-ACp-03	0	0	0	0	11	0	0	0	0	0	0	0	0	0	0	0	0	0	0	0	0

ID: 3054101
name: MN-L-Sens-B3-ACp-04

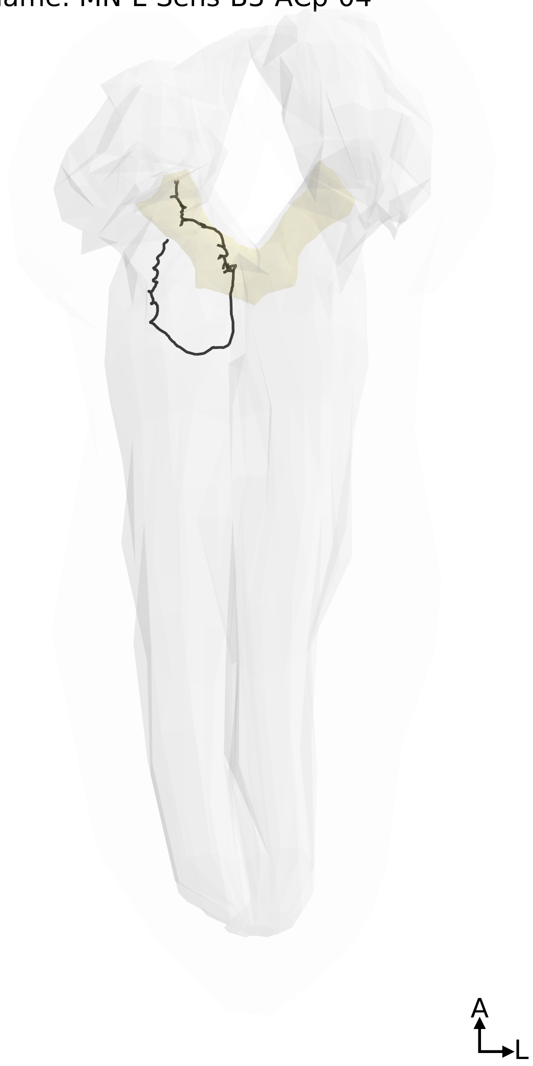

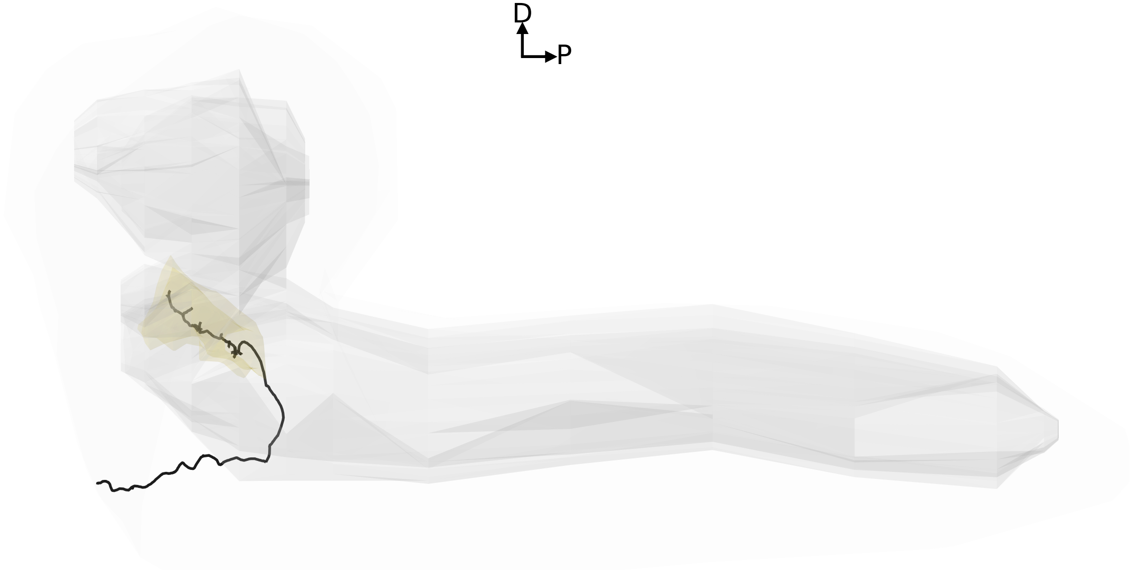

ID	name	SCACa	SCAVa	SCAVp	SCACal	SCACp	SCACpl	SCVM	IPCs	DMS	DH44	Se0ens	Se0ph	PMN LR	MN motor neurons	PaN motor neurons	olfactory PNs	gustatory PNs	multiglomerular PNs	unknown PNs	thermo PNs	visual PNs
3054101	MN-L-Sens-B3-ACp-04	0	0	0	0	5	0	0	0	0	1	0	0	0	0	0	0	0	7	0	0	0

ID: 15717736
name: MN-L-Sens-B3-ACp-05

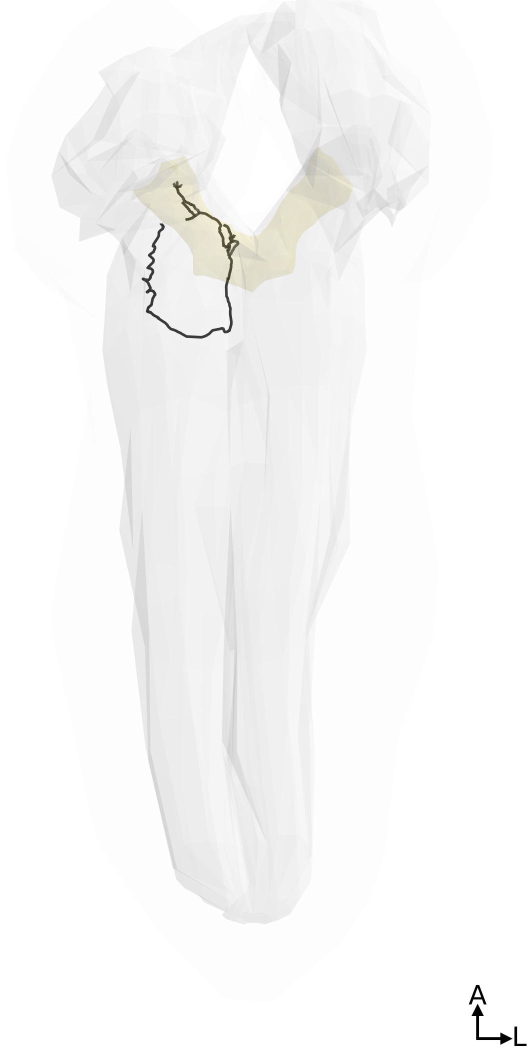

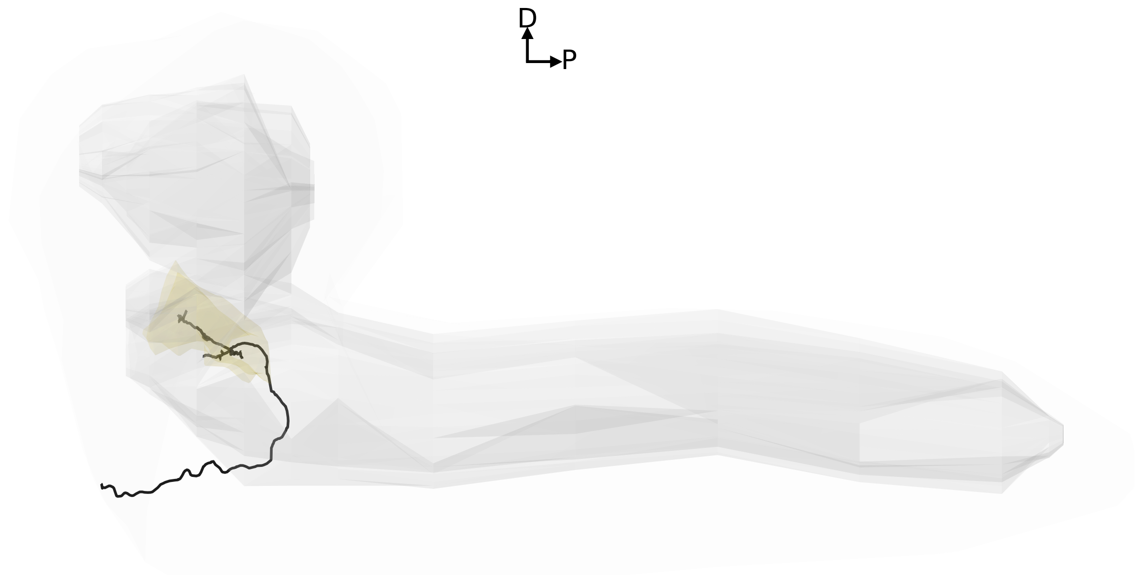

ID	name	SCACa	SCAVa	SCAVp	SCACal	SCACp	SCACpl	SCVM	IPCs	DMS	DH44	Se0ens	Se0ph	PMN LR	MN motor neurons	PaN motor neurons	olfactory PNs	gustatory PNs	multiglomerular PNs	unknown PNs	thermo PNs	visual PNs
15717736	MN-L-Sens-B3-ACp-05	1	0	0	0	9	0	0	0	0	0	0	0	0	0	0	0	0	0	0	0	0

ID: 15718275
name: MN-L-Sens-B3-ACp-06

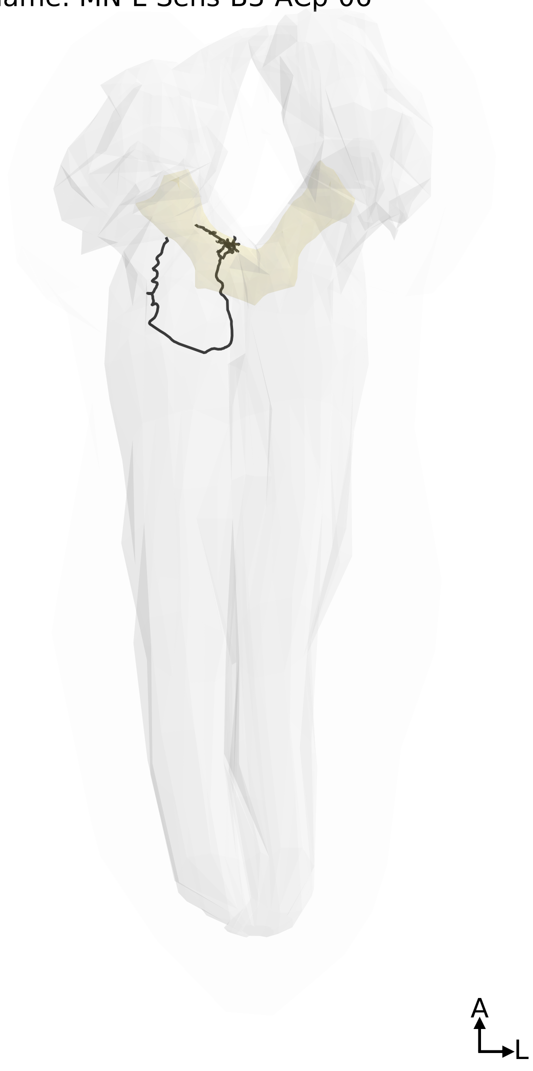

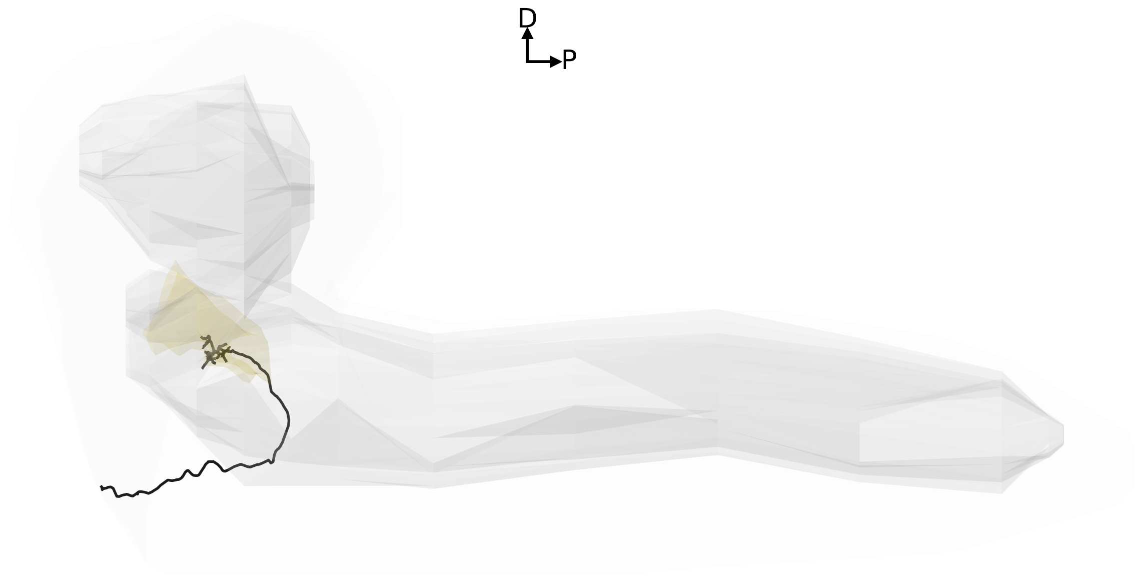

ID	name	SCACa	SCAVa	SCAVp	SCACal	SCACp	SCACpl	SCVM	IPCs	DMS	DH44	Se0ens	Se0ph	PMN LR	MN motor neurons	PaN motor neurons	olfactory PNs	gustatory PNs	multiglomerular PNs	unknown PNs	thermo PNs	visual PNs
15718275	MN-L-Sens-B3-ACp-06	1	0	0	0	1	0	0	0	0	0	0	0	0	0	0	0	0	0	0	0	0

ID: 15652529
name: MN-R-Sens-B2-ACp-01

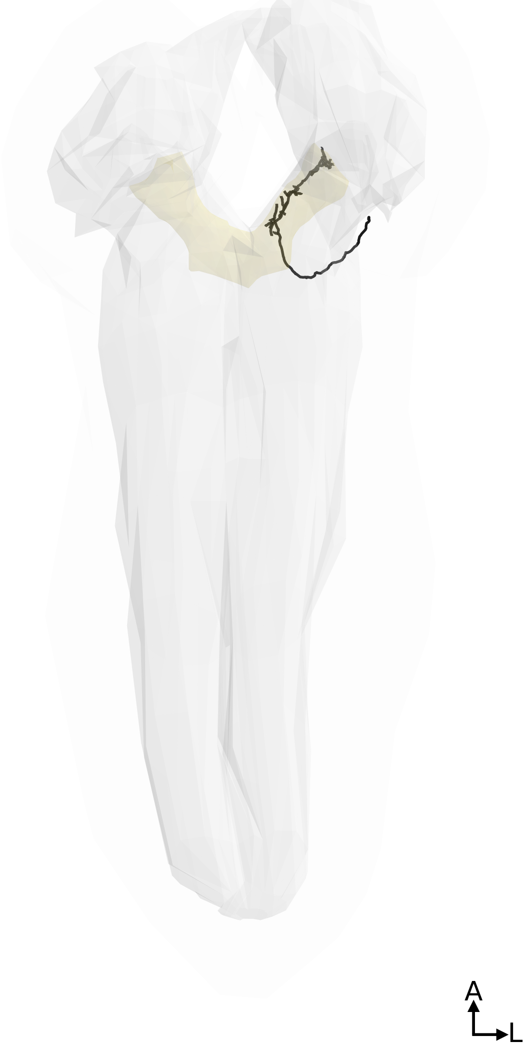

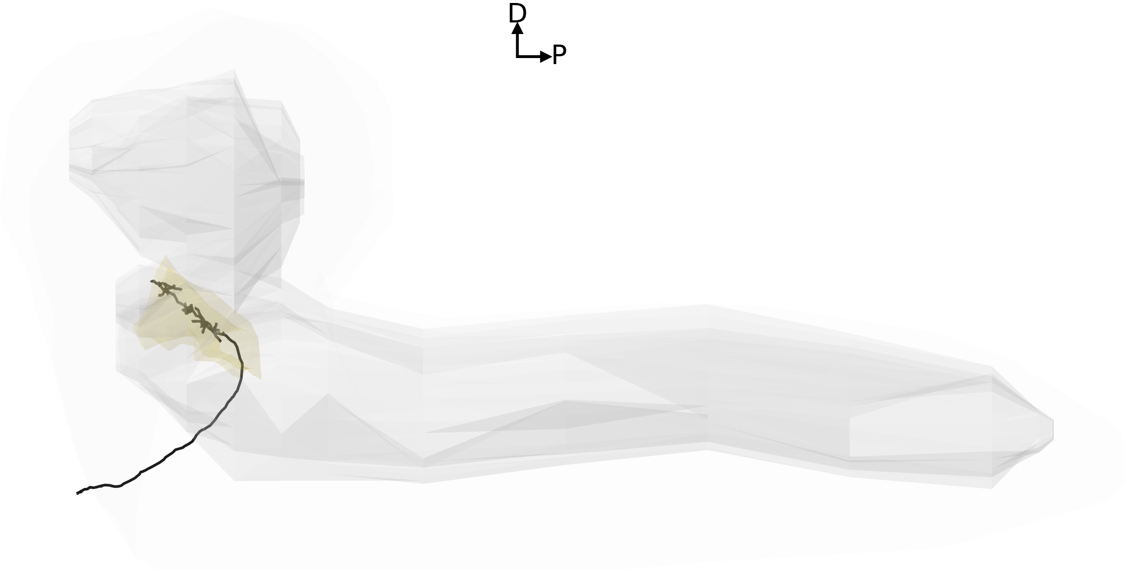

ID	*name*	SCACa	SCAVa	SCAVp	SCACal	SCACp	SCACpl	SCVM	IPCs	DMS	DH44	Se0ens	Se0ph	PMN LR	MN motor neurons	PaN motor neurons	olfactory PNs	gustatory PNs	multiglomerular PNs	unknown PNs	thermo PNs	visual PNs
15652529	MN-R-Sens-B2-ACp-01	0	0	0	0	2	0	0	0	0	0	0	0	0	0	0	1	40	1	0	0	0

ID: 15556868
name: MN-R-Sens-B2-ACp-02

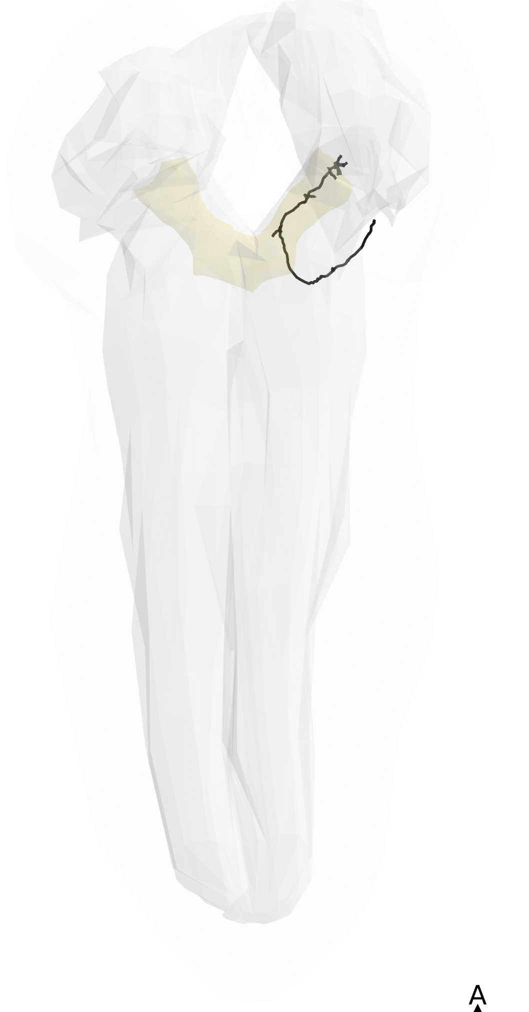

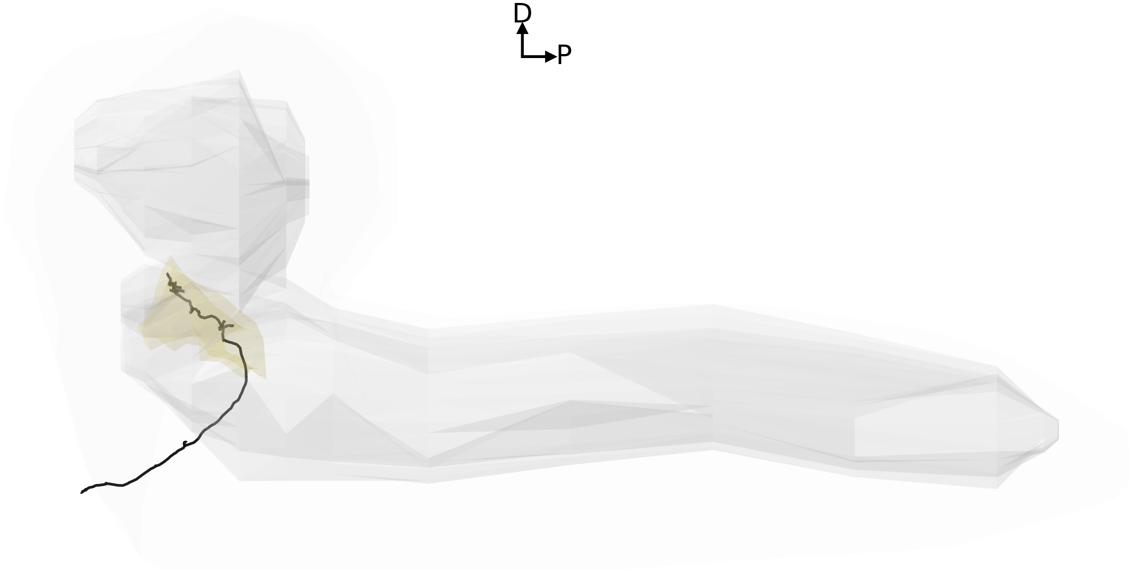

ID	name	SCACa	SCAVa	SCAVp	SCACal	SCACp	SCACpl	SCVM	IPCs	DMS	DH44	Se0ens	Se0ph	PMN LR	MN motor neurons	PaN motor neurons	olfactory PNs	gustatory PNs	multiglomerular PNs	unknown PNs	thermo PNs	visual PNs
15556868	MN-R-Sens-B2-ACp-02	0	0	0	0	4	0	0	0	0	0	0	0	0	0	0	3	1	5	0	0	0

ID: 15736147
name: MN-R-Sens-B2-ACp-03

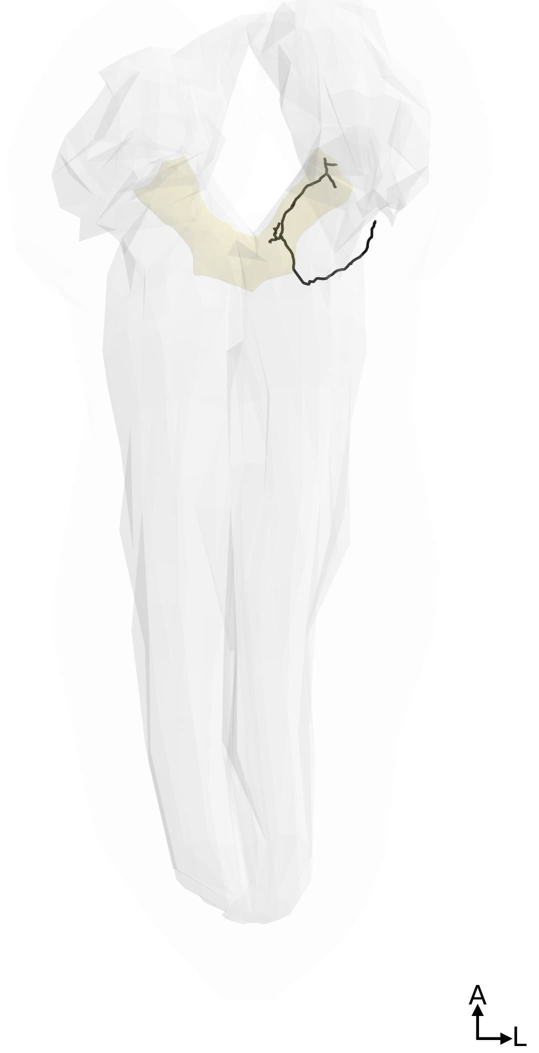

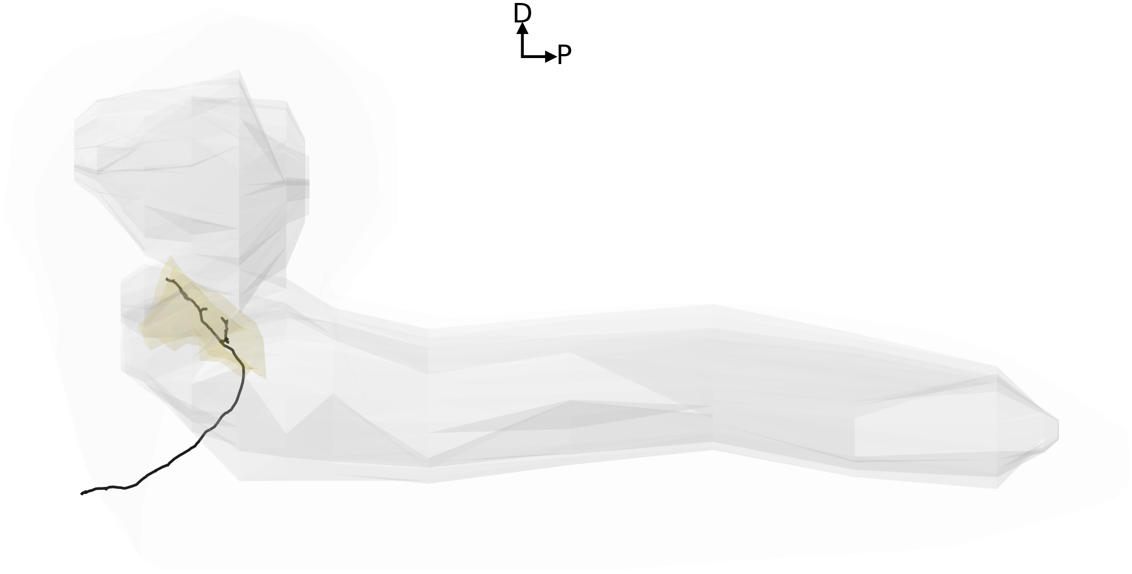

ID	name	SCACa	SCAVa	SCAVp	SCACal	SCACp	SCACpl	SCVM	IPCs	DMS	DH44	Se0ens	Se0ph	PMN LR	MN motor neurons	PaN motor neurons	olfactory PNs	gustatory PNs	multiglomerular PNs	unknown PNs	thermo PNs	visual PNs
15736147	MN-R-Sens-B2-ACp-03	0	0	0	0	4	0	0	0	0	0	0	0	0	0	0	0	8	1	0	0	0

ID: 16341399
name: MN-R-Sens-B2-ACp-04

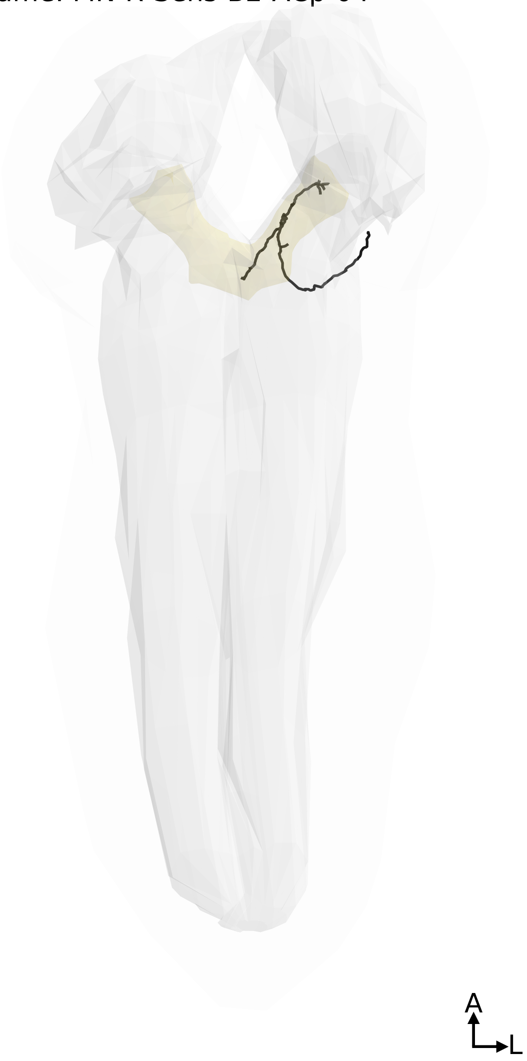

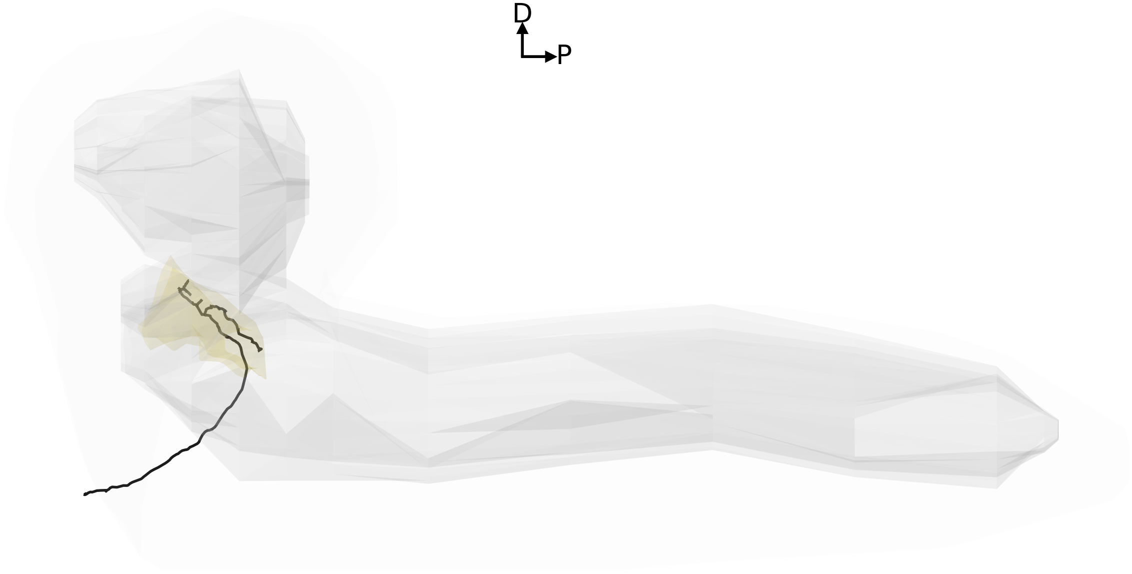

ID	name	SCACa	SCAVa	SCAVp	SCACal	SCACp	SCACpl	SCVM	IPCs	DMS	DH44	Se0ens	Se0ph	PMN LR	MN motor neurons	PaN motor neurons	olfactory PNs	gustatory PNs	multiglomerular PNs	unknown PNs	thermo PNs	visual PNs
16341399	MN-R-Sens-B2-ACp-04	0	0	0	0	7	0	0	0	0	0	0	0	0	0	0	0	16	4	0	1	0

ID: 15615589
name: MN-R-Sens-B2-ACp-05

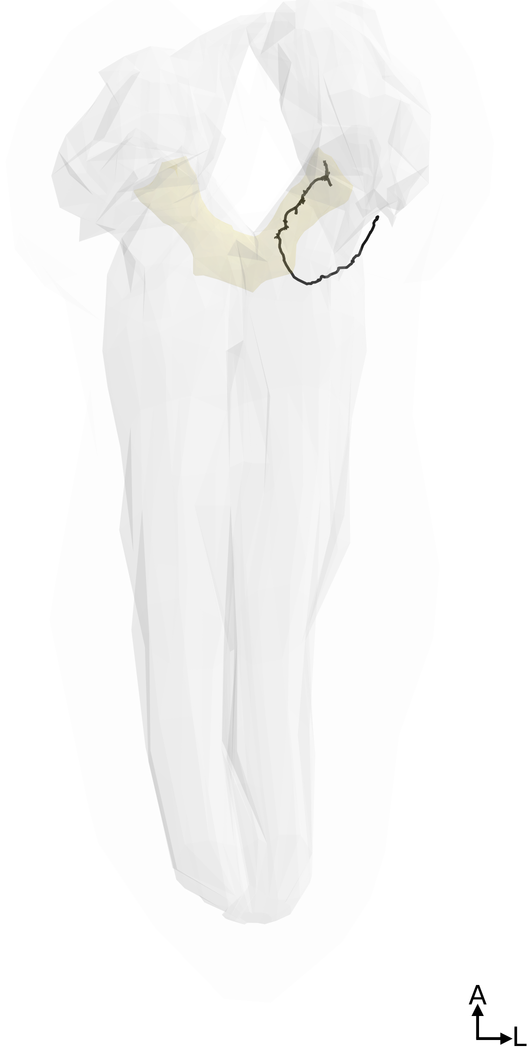

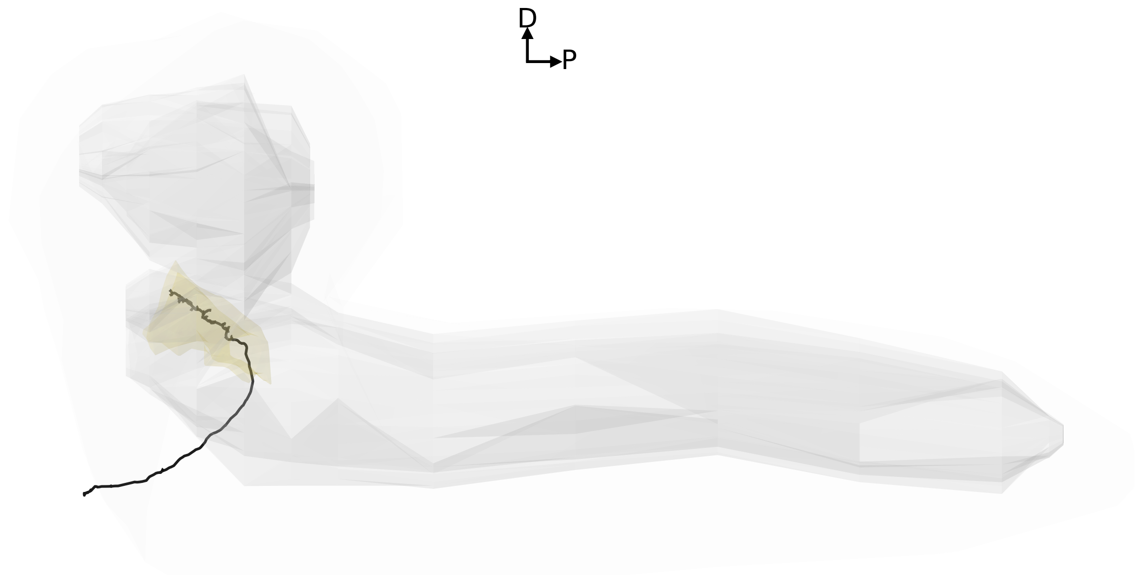

ID	*name*	SCACa	SCAVa	SCAVp	SCACal	SCACp	SCACpl	SCVM	IPCs	DMS	DH44	Se0ens	Se0ph	PMN LR	MN motor neurons	PaN motor neurons	olfactory PNs	gustatory PNs	multiglomerular PNs	unknown PNs	thermo PNs	visual PNs
15615589	MN-R-Sens-B2-ACp-05	0	0	0	0	8	0	0	0	0	0	0	0	0	0	0	0	12	4	0	0	0

ID: 15589362
name: MN-R-Sens-B2-ACp-06

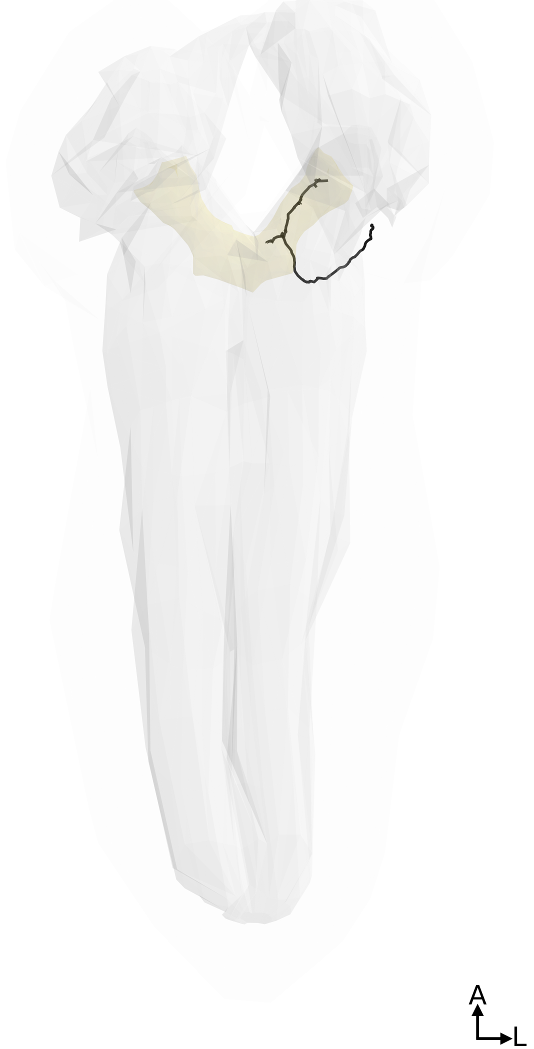

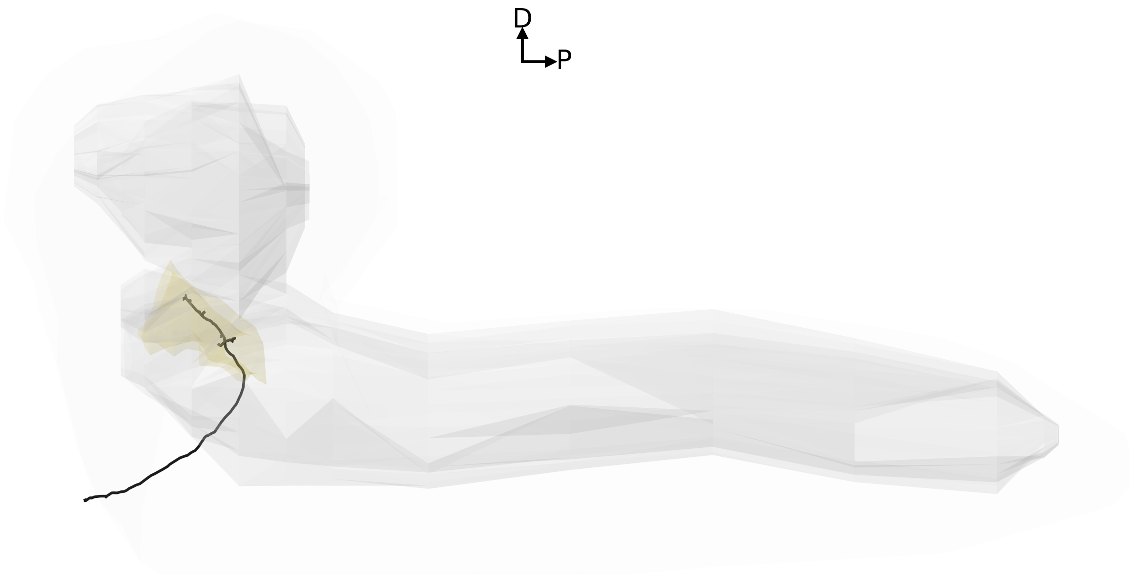

ID	*name*	SCACa	SCAVa	SCAVp	SCACal	SCACp	SCACpl	SCVM	IPCs	DMS	DH44	Se0ens	Se0ph	PMN LR	MN motor neurons	PaN motor neurons	olfactory PNs	gustatory PNs	multiglomerular PNs	unknown PNs	thermo PNs	visual PNs
15589362	MN-R-Sens-B2-ACp-06	0	0	0	0	4	0	0	0	0	0	0	0	0	0	0	0	4	0	0	0	0

ID: 15653432
name: MN-R-Sens-B2-ACp-07

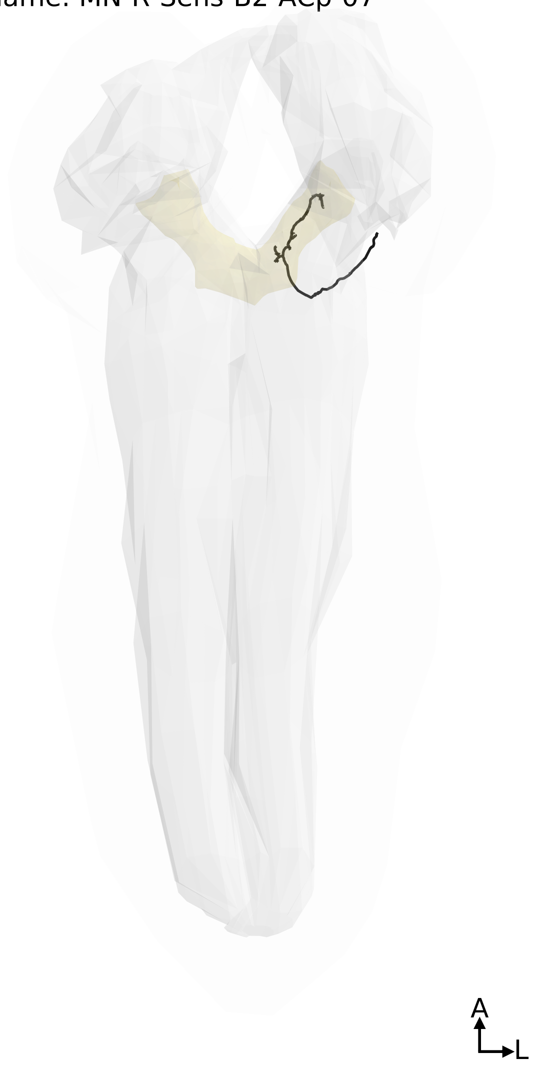

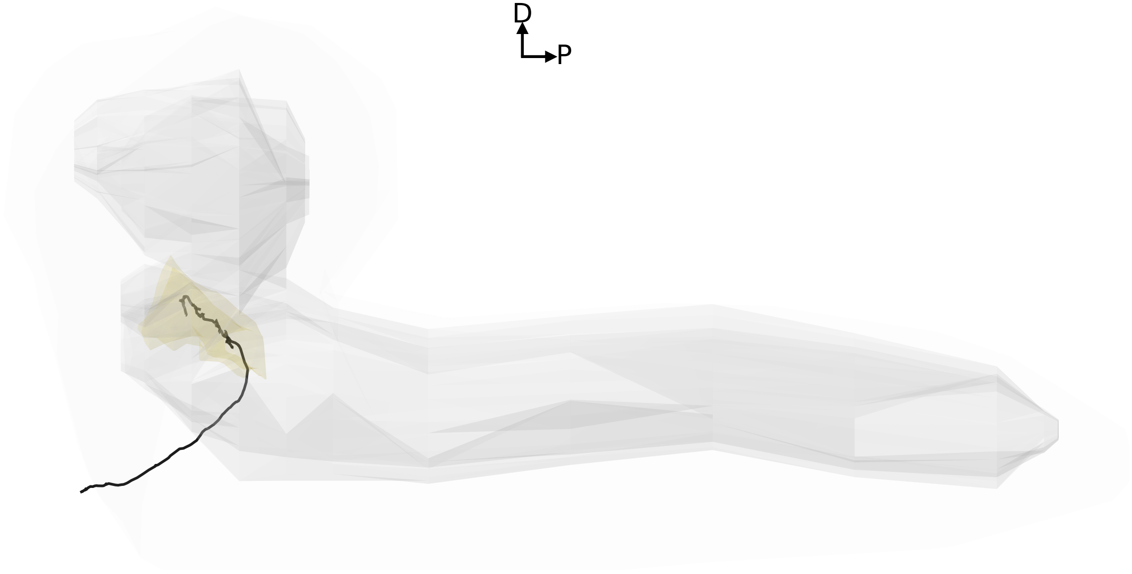

ID	name	SCACa	SCAVa	SCAVp	SCACal	SCACp	SCACpl	SCVM	IPCs	DMS	DH44	Se0ens	Se0ph	PMN LR	MN motor neurons	PaN motor neurons	olfactory PNs	gustatory PNs	multiglomerular PNs	unknown PNs	thermo PNs	visual PNs
15653432	MN-R-Sens-B2-ACp-07	0	0	0	0	4	0	0	0	0	0	0	0	0	0	0	0	14	0	0	0	0

ID: 15595607
name: MN-R-Sens-B2-ACp-08

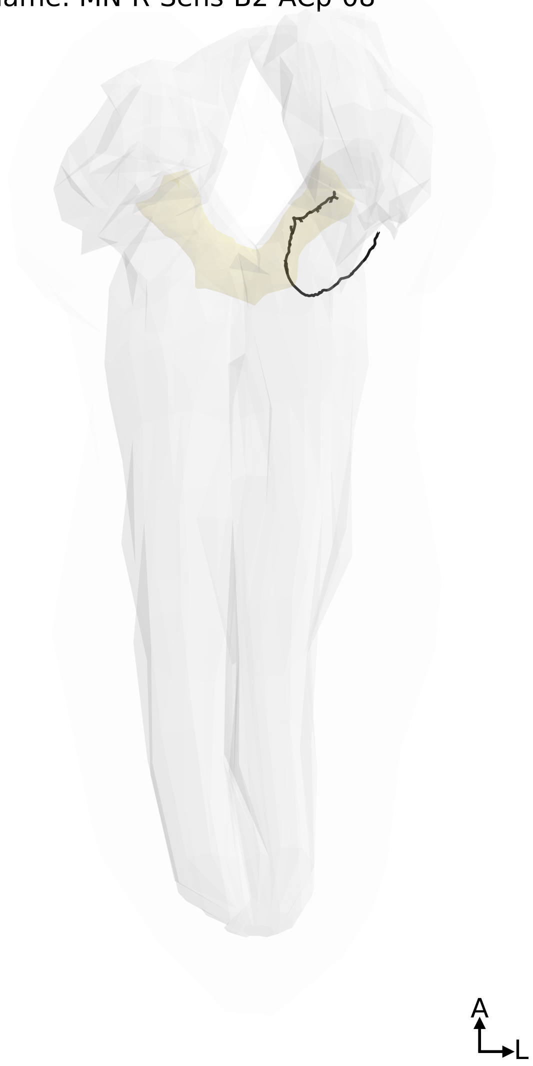

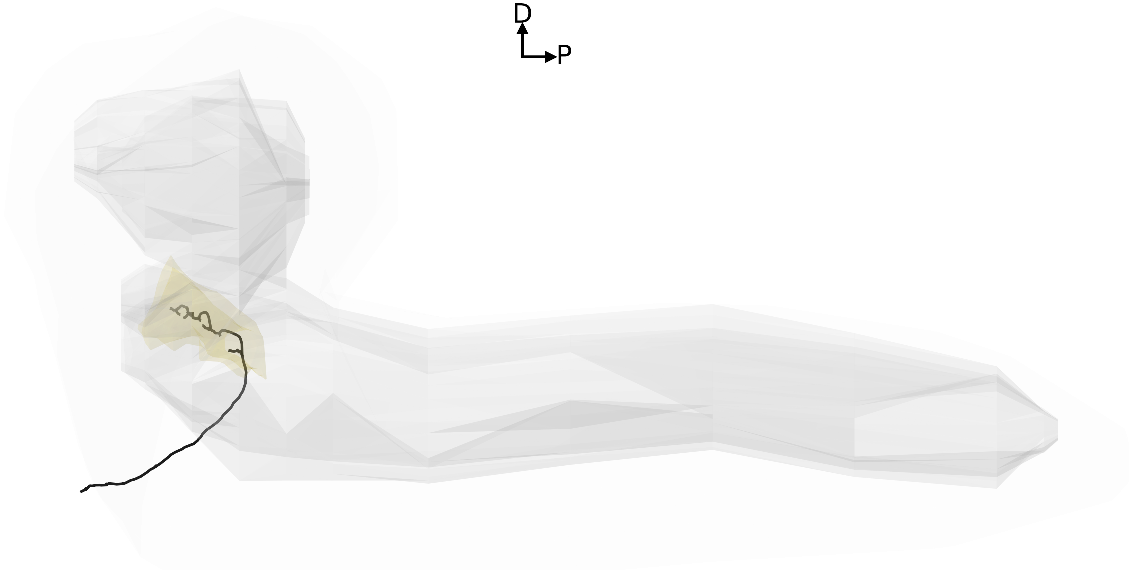

ID	name	SCACa	SCAVa	SCAVp	SCACal	SCACp	SCACpl	SCVM	IPCs	DMS	DH44	Se0ens	Se0ph	PMN LR	MN motor neurons	PaN motor neurons	olfactory PNs	gustatory PNs	multiglomerular PNs	unknown PNs	thermo PNs	visual PNs
15595607	MN-R-Sens-B2-ACp-08	0	0	0	0	1	0	0	0	0	0	0	0	0	0	0	6	0	0	0	0	0

ID: 15615042
name: MN-R-Sens-B2-ACp-09

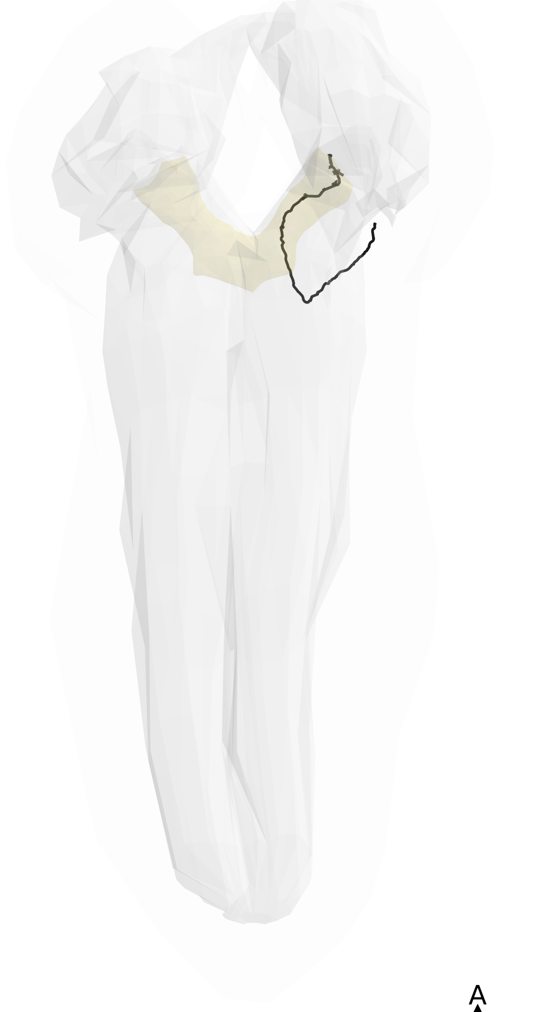

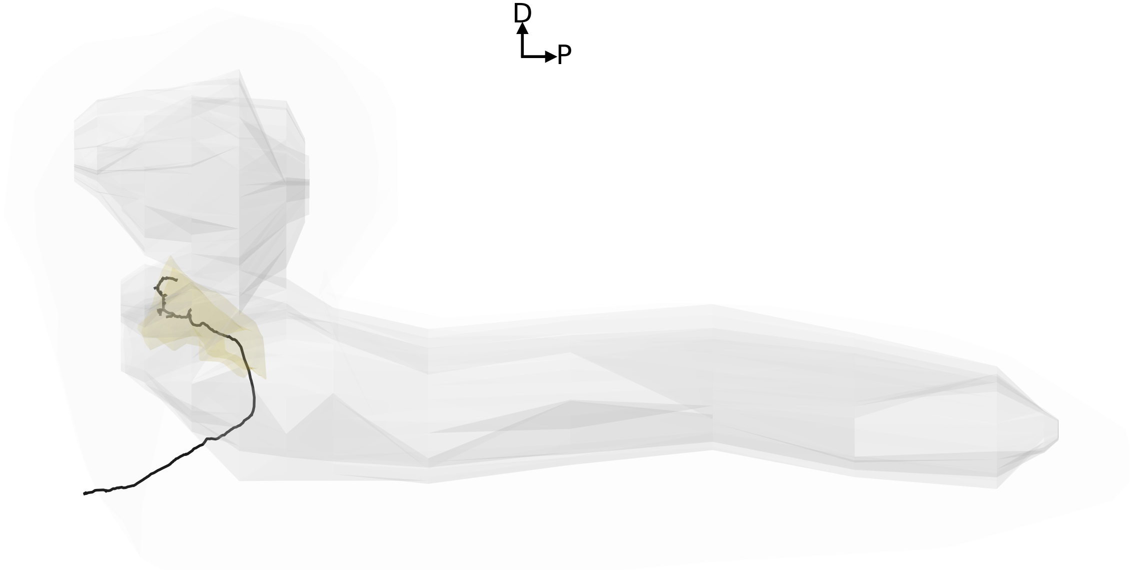

ID	*name*	SCACa	SCAVa	SCAVp	SCACal	SCACp	SCACpl	SCVM	IPCs	DMS	DH44	Se0ens	Se0ph	PMN LR	MN motor neurons	PaN motor neurons	olfactory PNs	gustatory PNs	multiglomerular PNs	unknown PNs	thermo PNs	visual PNs
15615042	MN-R-Sens-B2-ACp-09	0	0	0	0	6	0	0	0	0	0	0	0	0	0	0	1	23	19	0	0	0

ID: 18625446
name: MN-R-Sens-B2-ACp-10

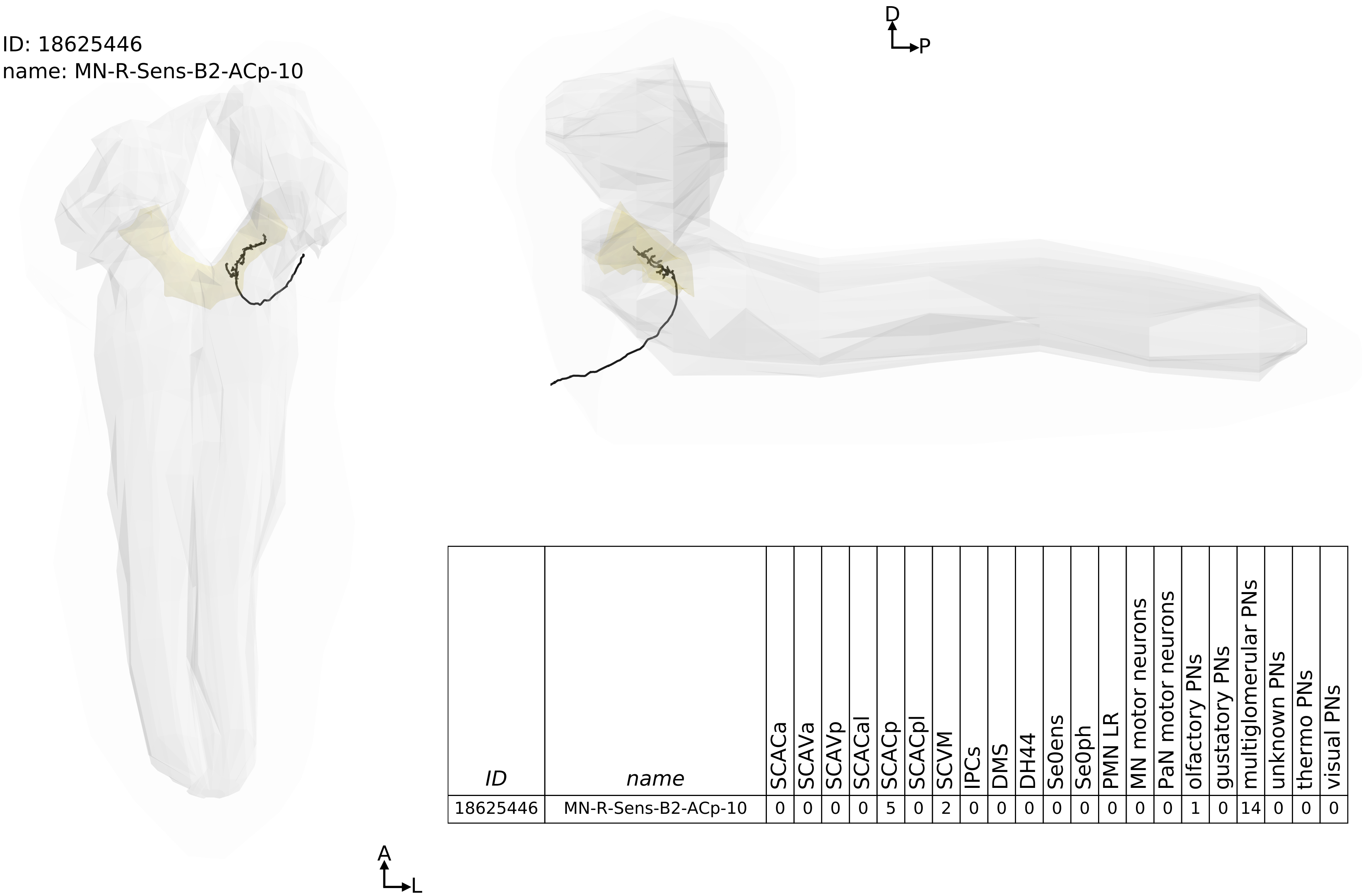

ID	*name*	SCACa	SCAVa	SCAVp	SCACal	SCACp	SCACpl	SCVM	IPCs	DMS	DH44	Se0ens	Se0ph	PMN LR	MN motor neurons	PaN motor neurons	olfactory PNs	gustatory PNs	multiglomerular PNs	unknown PNs	thermo PNs	visual PNs
18625446	MN-R-Sens-B2-ACp-10	0	0	0	0	5	0	2	0	0	0	0	0	0	0	0	1	0	14	0	0	0

ID: 15556081
name: MN-R-Sens-B2-ACp-11

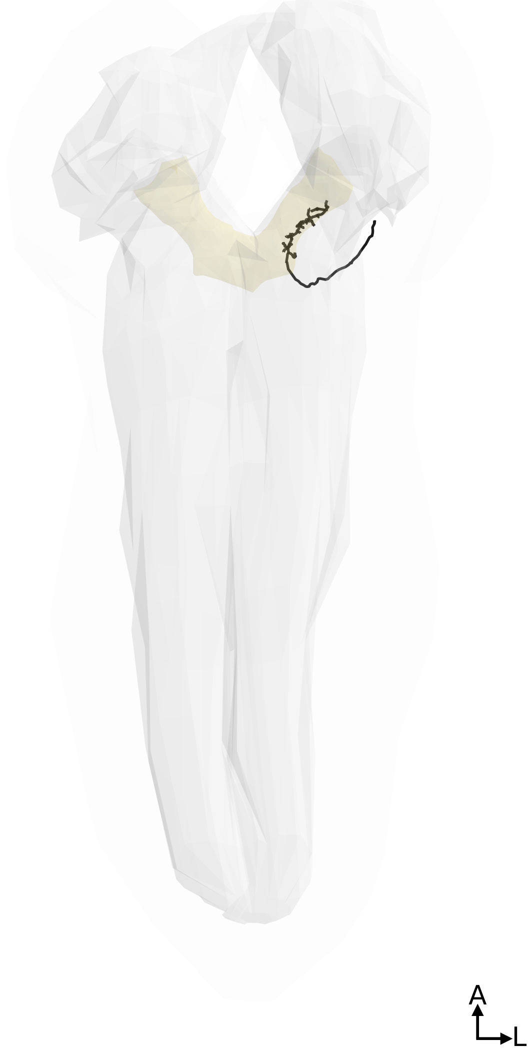

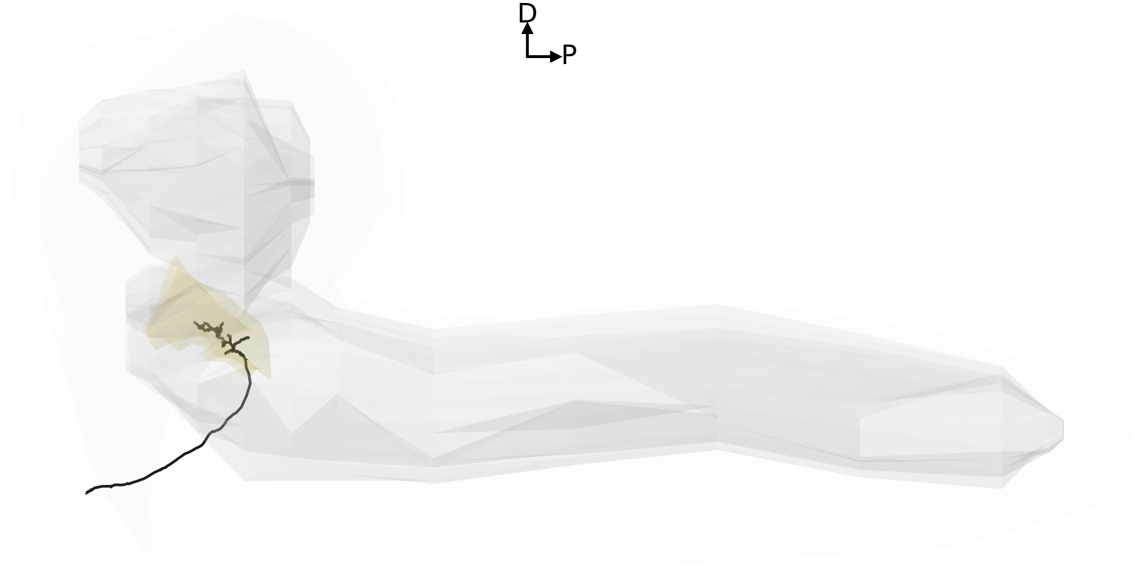

ID	*name*	SCACa	SCAVa	SCAVp	SCACal	SCACp	SCACpl	SCVM	IPCs	DMS	DH44	Se0ens	Se0ph	PMN LR	MN motor neurons	PaN motor neurons	olfactory PNs	gustatory PNs	multiglomerular PNs	unknown PNs	thermo PNs	visual PNs
15556081	MN-R-Sens-B2-ACp-11	0	0	0	0	5	0	0	0	0	0	0	0	0	0	0	0	0	17	0	0	0

ID: 15588691
name: MN-R-Sens-B2-ACp-12

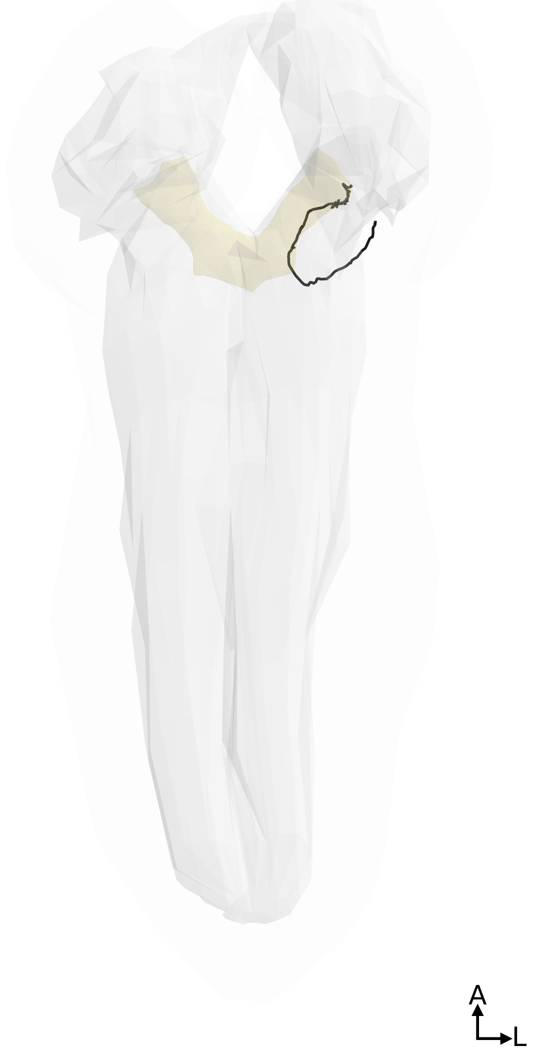

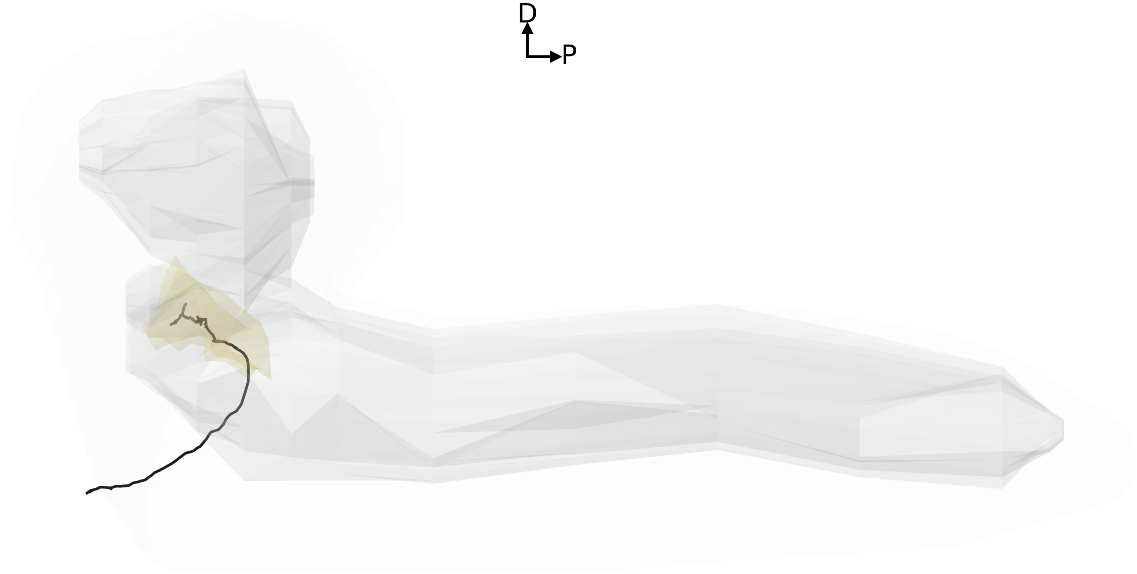

ID	*name*	SCACa	SCAVa	SCAVp	SCACal	SCACp	SCACpl	SCVM	IPCs	DMS	DH44	Se0ens	Se0ph	PMN LR	MN motor neurons	PaN motor neurons	olfactory PNs	gustatory PNs	multiglomerular PNs	unknown PNs	thermo PNs	visual PNs
15588691	MN-R-Sens-B2-ACp-12	0	0	0	0	1	0	0	0	0	0	0	0	0	0	0	1	0	15	0	0	0

ID: 3071301
name: MN-R-Sens-B2-ACp-13

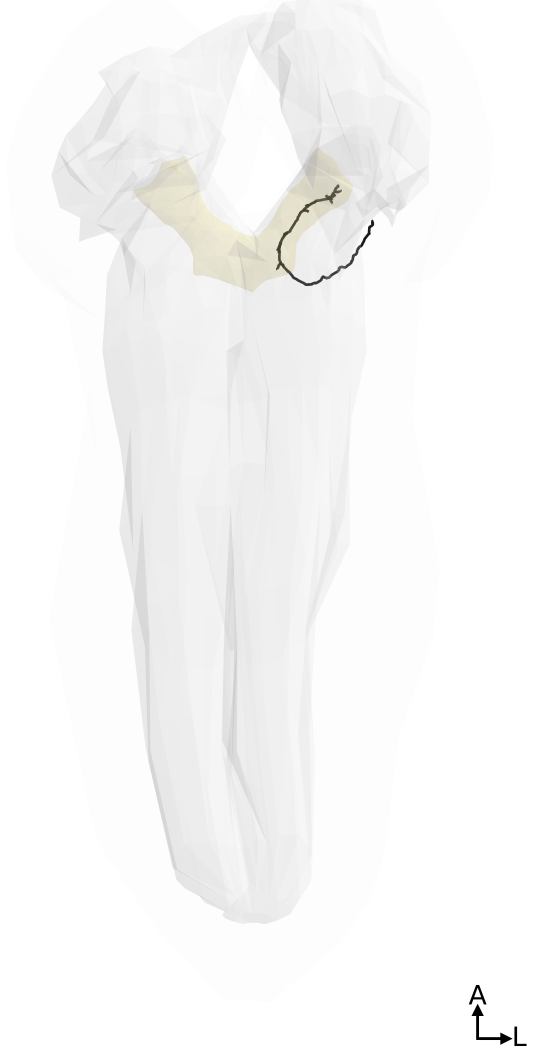

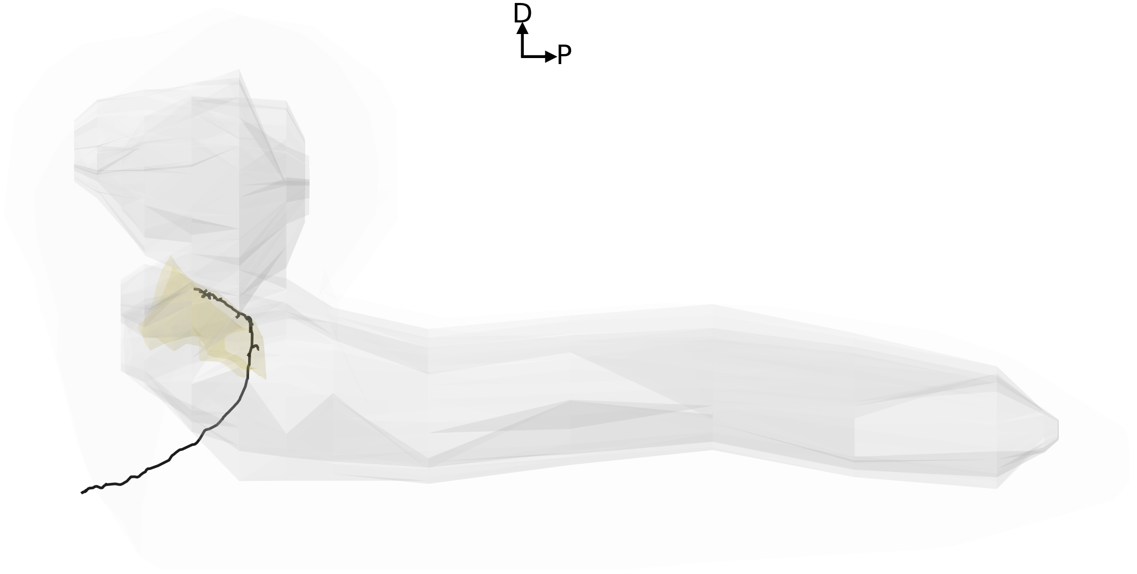

ID	name	SCACa	SCAVa	SCAVp	SCACal	SCACp	SCACpl	SCVM	IPCs	DMS	DH44	Se0ens	Se0ph	PMN LR	MN motor neurons	PaN motor neurons	olfactory PNs	gustatory PNs	multiglomerular PNs	unknown PNs	thermo PNs	visual PNs
3071301	MN-R-Sens-B2-ACp-13	0	0	0	0	0	0	0	0	0	0	0	0	0	0	0	1	0	3	0	0	0

ID: 12253020
name: MN-R-Sens-B2-ACp-14

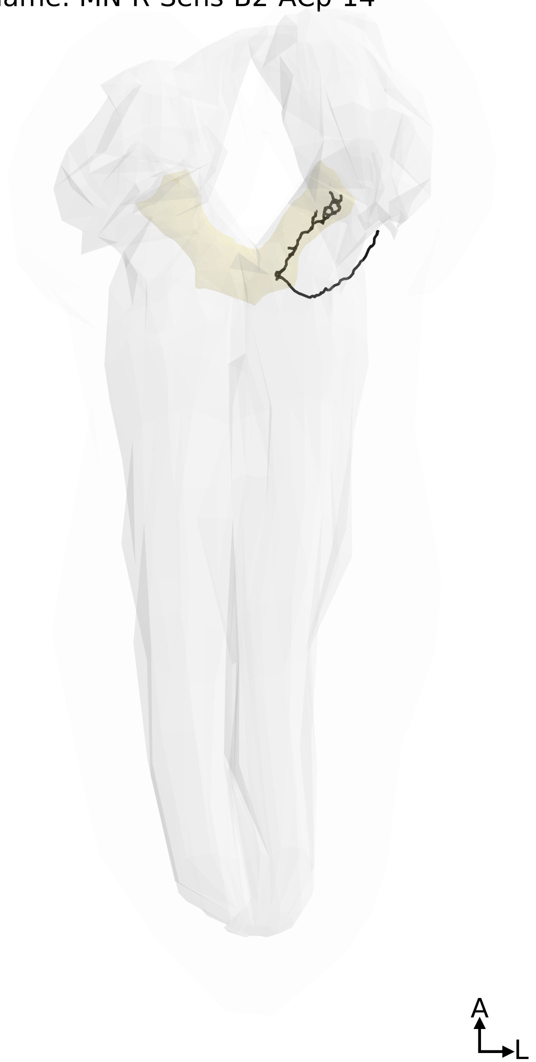

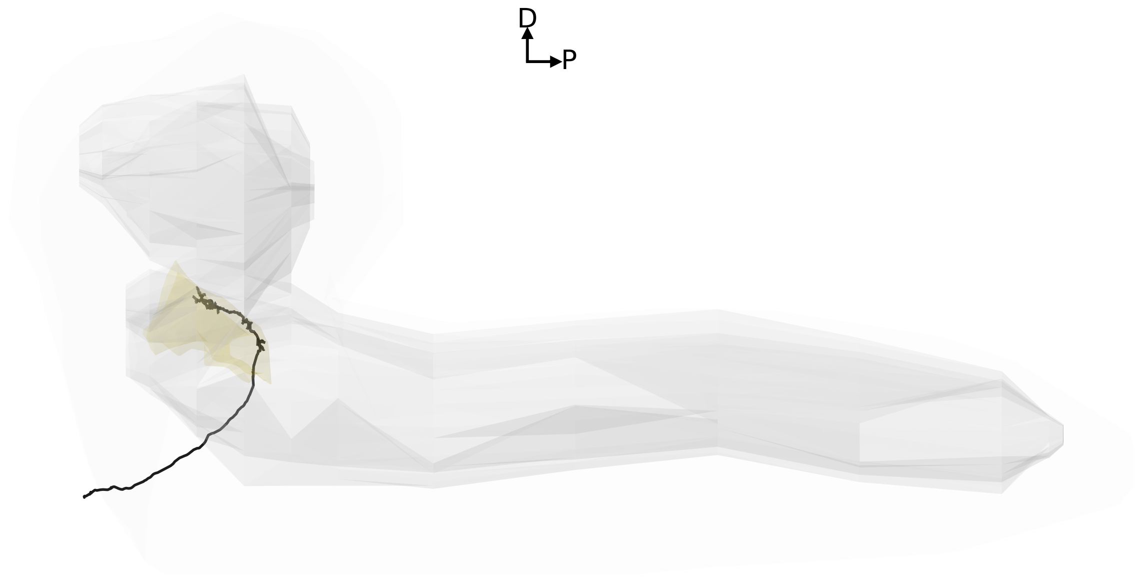

ID	*name*	SCACa	SCAVa	SCAVp	SCACal	SCACp	SCACpl	SCVM	IPCs	DMS	DH44	Se0ens	Se0ph	PMN LR	MN motor neurons	PaN motor neurons	olfactory PNs	gustatory PNs	multiglomerular PNs	unknown PNs	thermo PNs	visual PNs
12253020	MN-R-Sens-B2-ACp-14	0	0	0	0	4	0	0	0	0	0	0	0	0	0	0	3	0	4	0	0	0

ID: 15595823
name: MN-R-Sens-B2-ACp-15

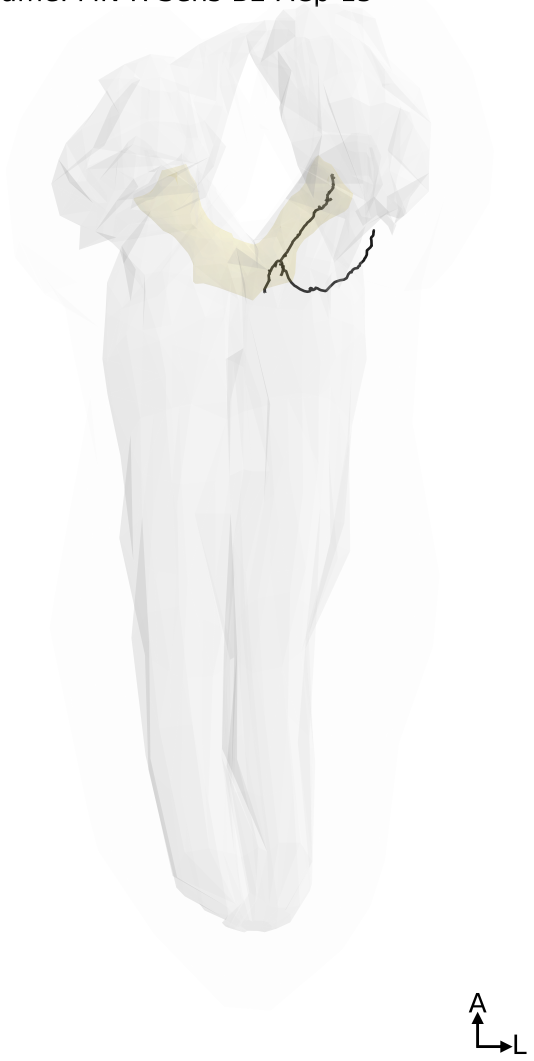

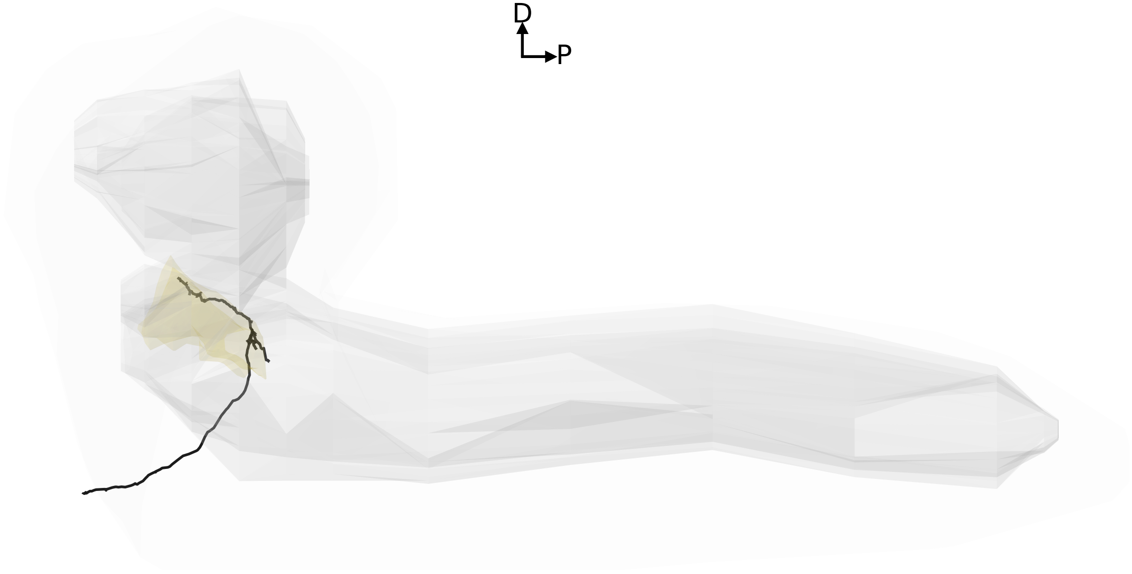

ID	*name*	SCACa	SCAVa	SCAVp	SCACal	SCACp	SCACpl	SCVM	IPCs	DMS	DH44	Se0ens	Se0ph	PMN LR	MN motor neurons	PaN motor neurons	olfactory PNs	gustatory PNs	multiglomerular PNs	unknown PNs	thermo PNs	visual PNs
15595823	MN-R-Sens-B2-ACp-15	0	0	0	0	1	0	0	0	0	0	0	0	0	0	0	0	0	0	0	0	0

ID: 15615081
name: MN-R-Sens-B2-ACp-16

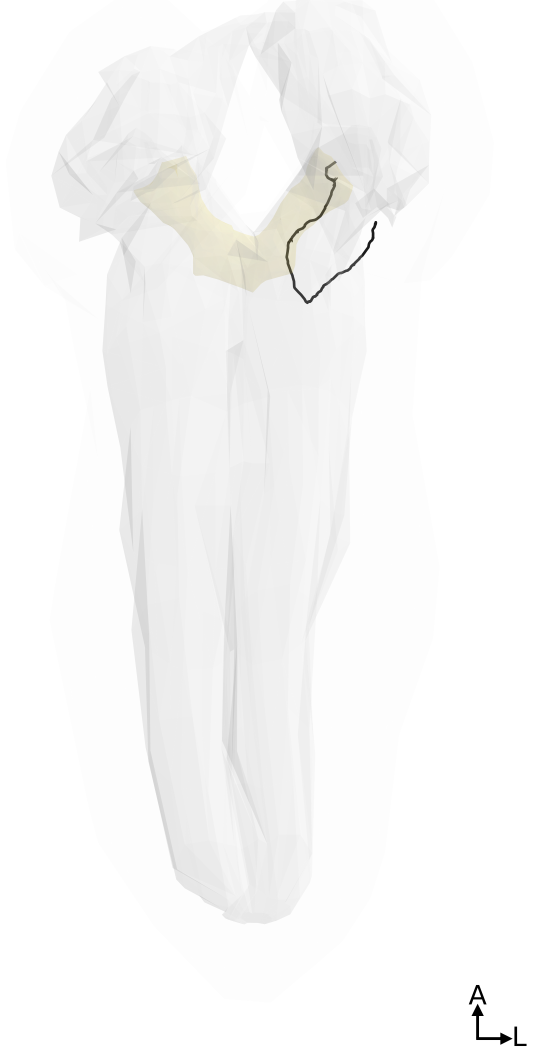

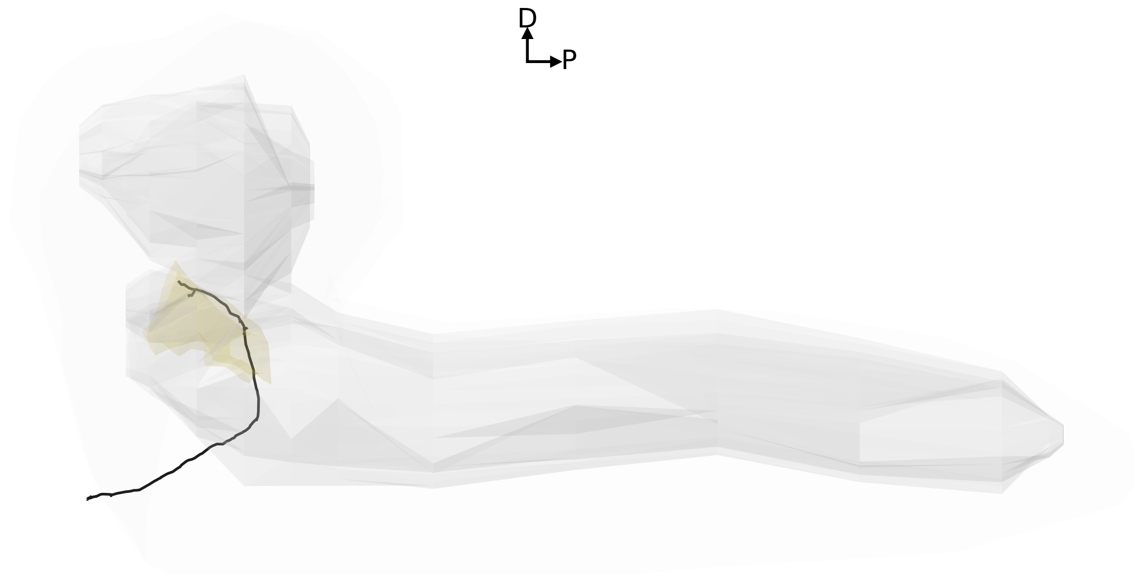

ID	*name*	SCACa	SCAVa	SCAVp	SCACal	SCACp	SCACpl	SCVM	IPCs	DMS	DH44	Se0ens	Se0ph	PMN LR	MN motor neurons	PaN motor neurons	olfactory PNs	gustatory PNs	multiglomerular PNs	unknown PNs	thermo PNs	visual PNs
15615081	MN-R-Sens-B2-ACp-16	0	0	0	0	0	0	0	0	0	0	0	0	0	0	0	0	1	6	0	0	0

ID: 15609885
name: MN-R-Sens-B2-ACp-17

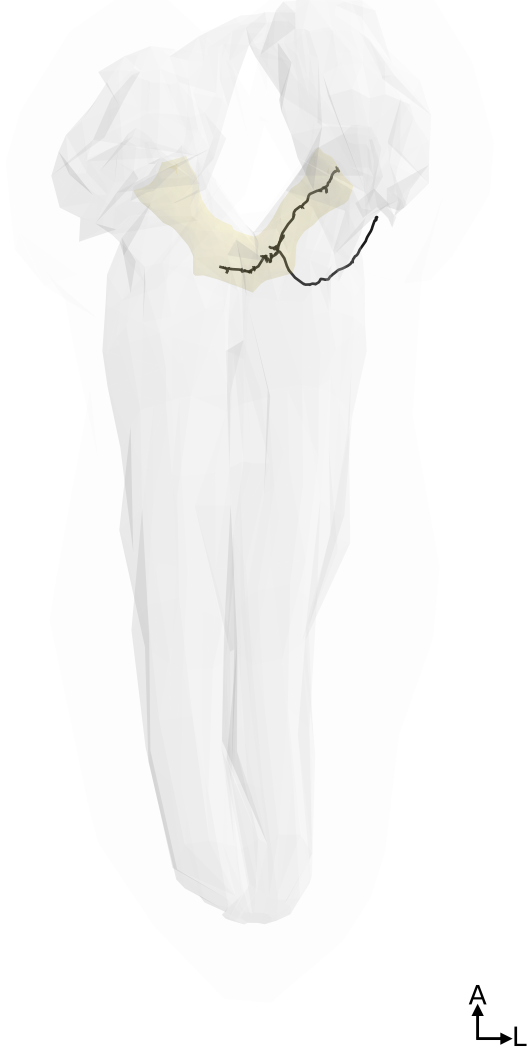

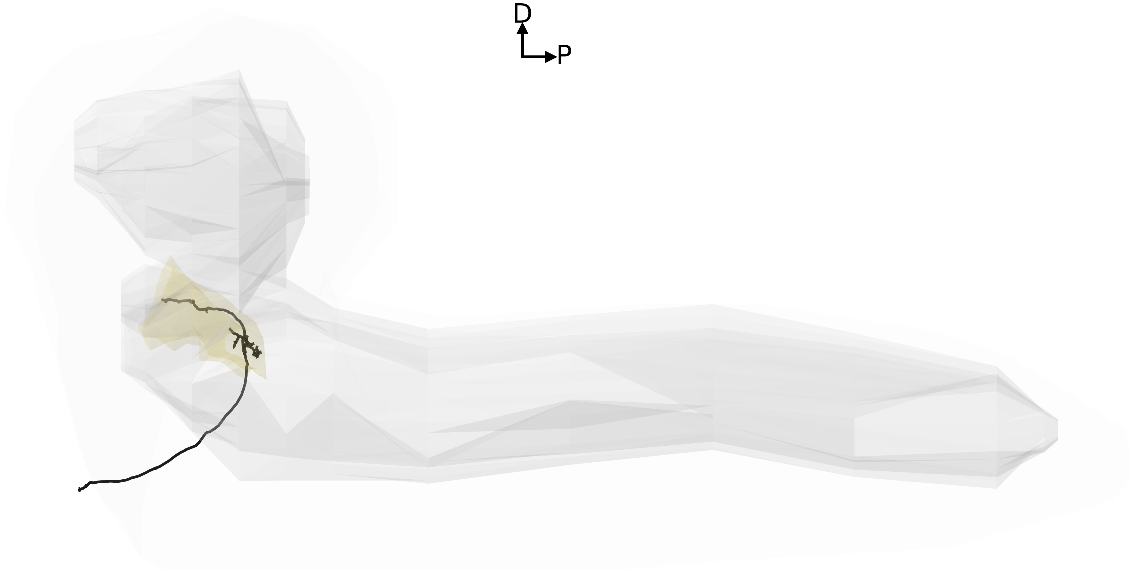

ID	*name*	SCACa	SCAVa	SCAVp	SCACal	SCACp	SCACpl	SCVM	IPCs	DMS	DH44	Se0ens	Se0ph	PMN LR	MN motor neurons	PaN motor neurons	olfactory PNs	gustatory PNs	multiglomerular PNs	unknown PNs	thermo PNs	visual PNs
15609885	MN-R-Sens-B2-ACp-17	0	0	0	0	3	0	0	0	0	0	0	0	0	0	0	2	4	4	0	0	0

ID: 10934438
name: MN-R-Sens-B2-ACp-18

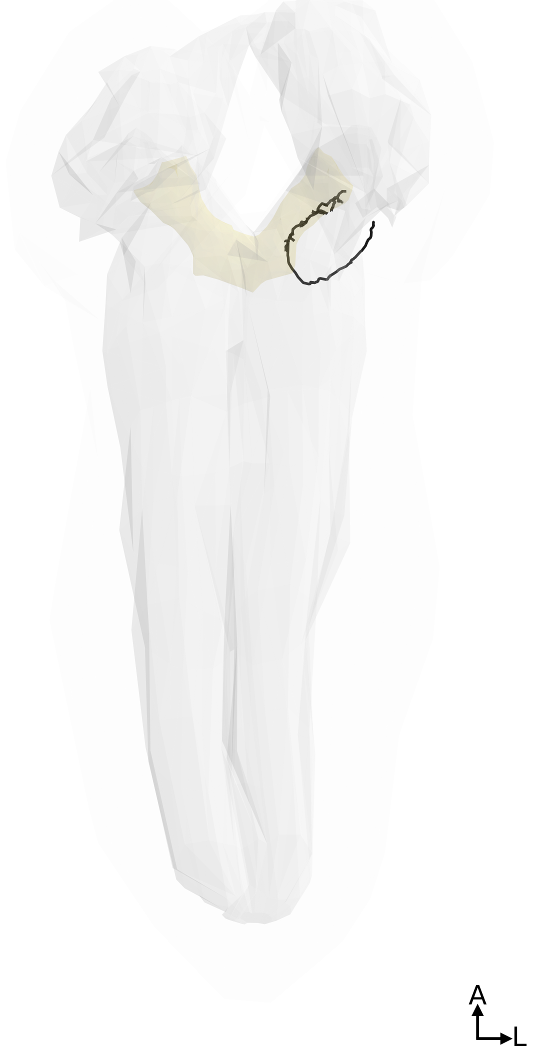

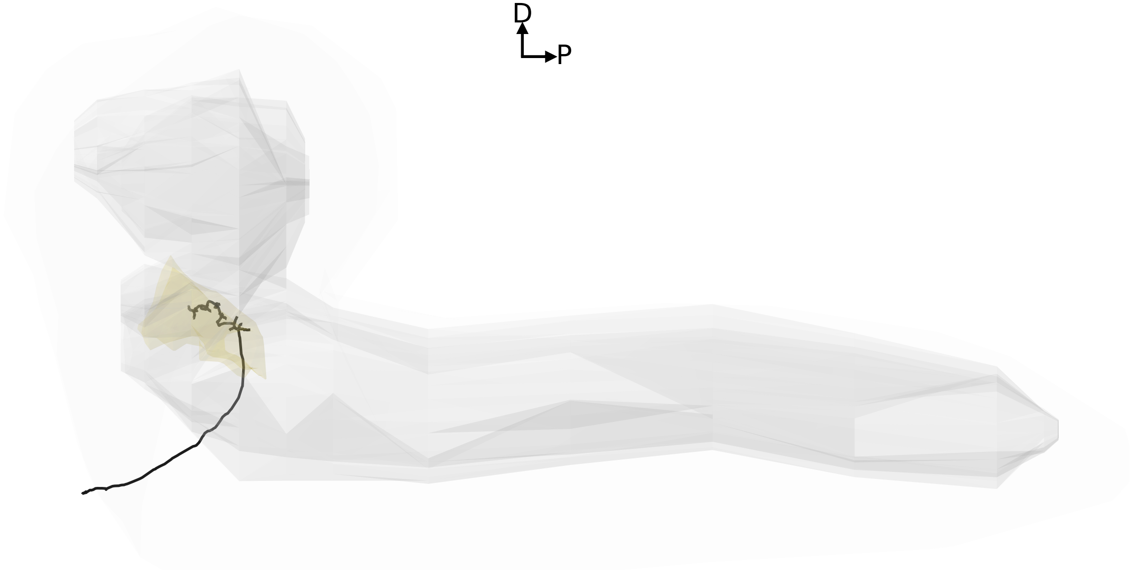

ID	name	SCACa	SCAVa	SCAVp	SCACal	SCACp	SCACpl	SCVM	IPCs	DMS	DH44	Se0ens	Se0ph	PMN LR	MN motor neurons	PaN motor neurons	olfactory PNs	gustatory PNs	multiglomerular PNs	unknown PNs	thermo PNs	visual PNs
10934438	MN-R-Sens-B2-ACp-18	0	0	0	0	1	0	0	0	0	0	0	0	0	0	0	0	0	1	0	0	0

ID: 15653288
name: MN-R-Sens-B2-ACp-19

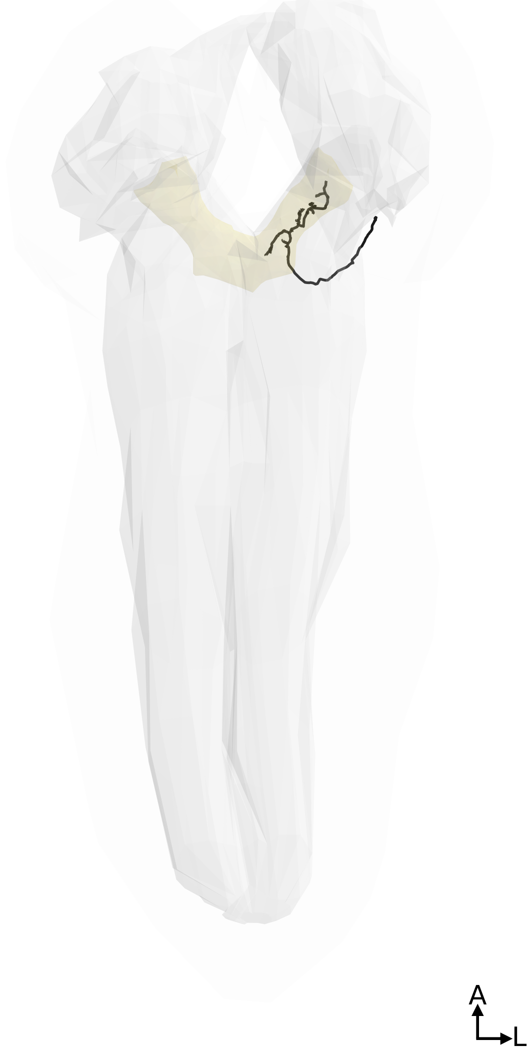

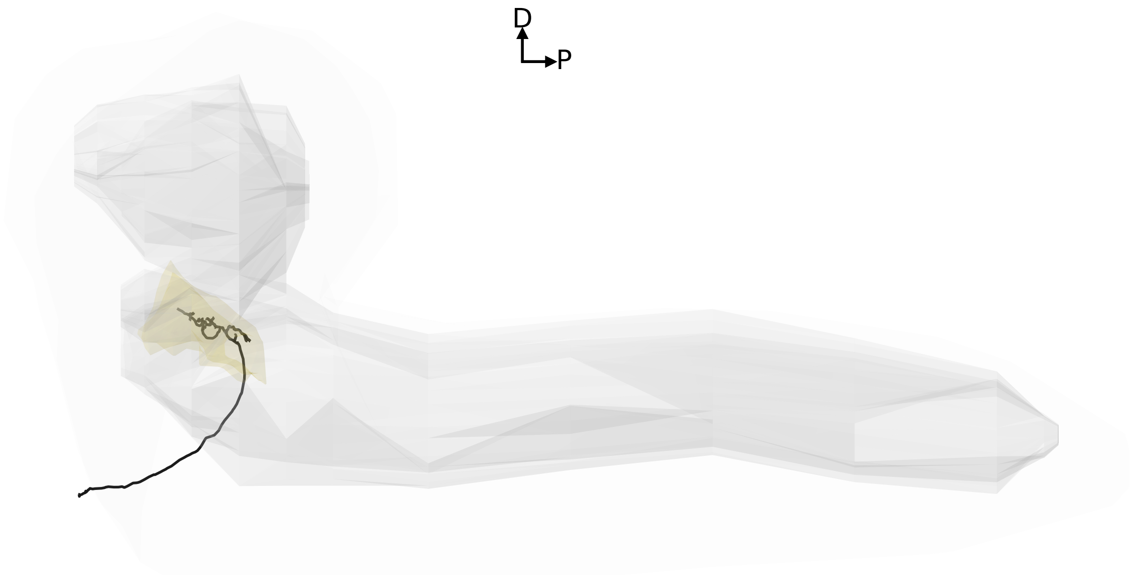

ID	name	SCACa	SCAVa	SCAVp	SCACal	SCACp	SCACpl	SCVM	IPCs	DMS	DH44	Se0ens	Se0ph	PMN LR	MN motor neurons	PaN motor neurons	olfactory PNs	gustatory PNs	multiglomerular PNs	unknown PNs	thermo PNs	visual PNs
15653288	MN-R-Sens-B2-ACp-19	0	0	0	0	3	0	0	0	0	0	0	0	0	0	0	0	0	0	0	0	0

ID: 15588800
name: MN-R-Sens-B2-ACp-20

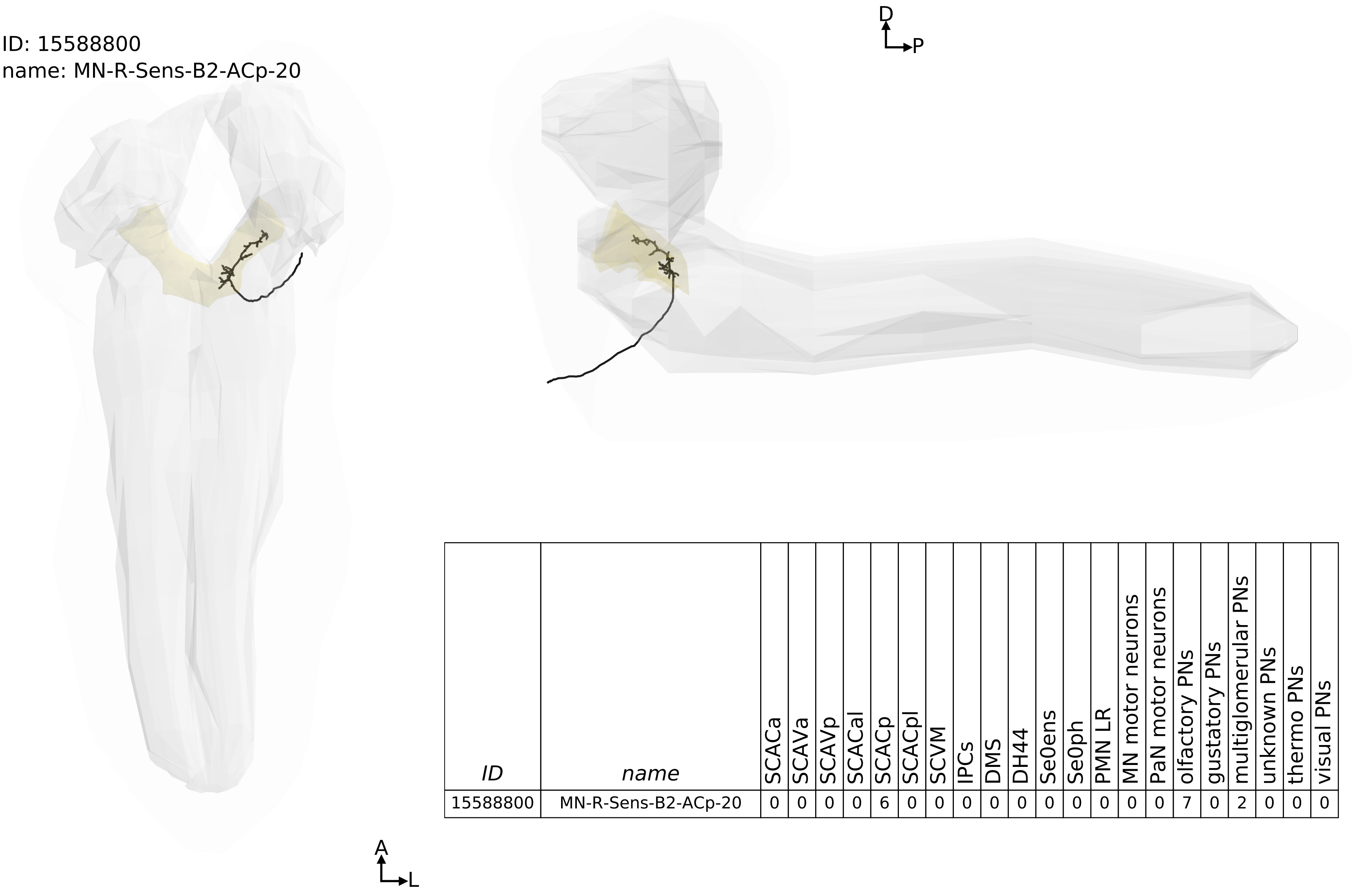

ID	name	SCACa	SCAVa	SCAVp	SCACal	SCACp	SCACpl	SCVM	IPCs	DMS	DH44	Se0ens	Se0ph	PMN LR	MN motor neurons	PaN motor neurons	olfactory PNs	gustatory PNs	multiglomerular PNs	unknown PNs	thermo PNs	visual PNs
15588800	MN-R-Sens-B2-ACp-20	0	0	0	0	6	0	0	0	0	0	0	0	0	0	0	7	0	2	0	0	0

ID: 15589411
name: MN-R-Sens-B2-ACp-21

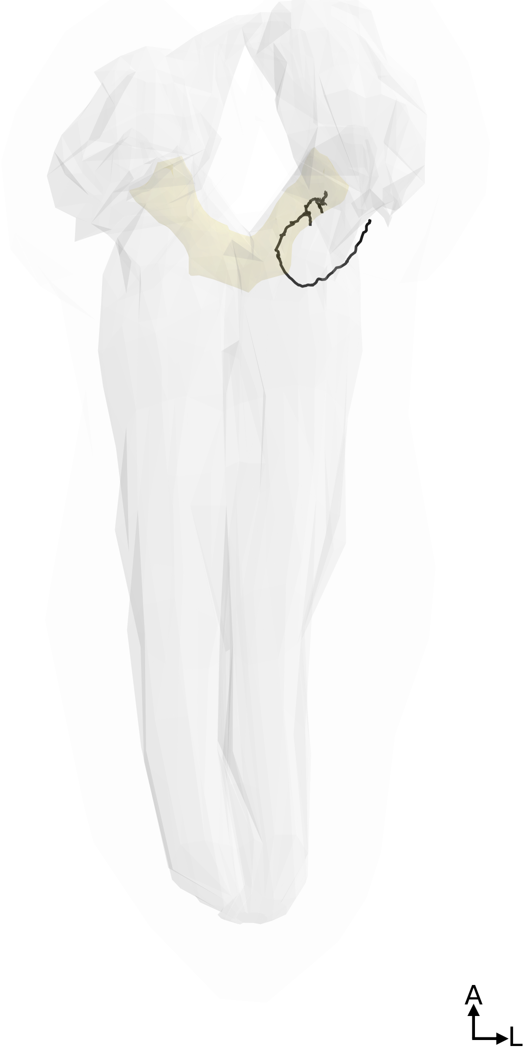

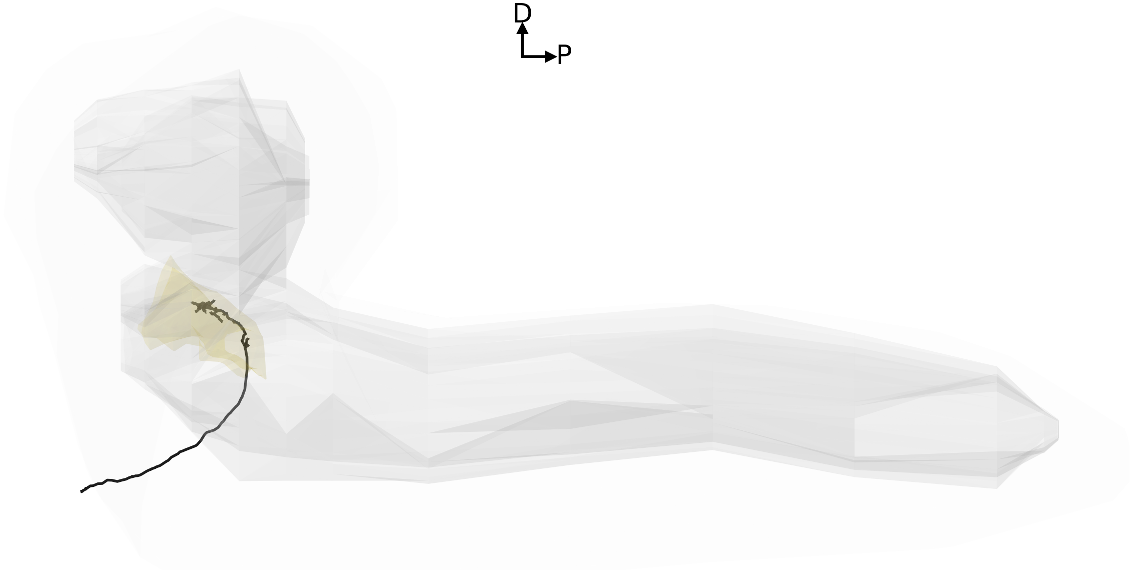

ID	*name*	SCACa	SCAVa	SCAVp	SCACal	SCACp	SCACpl	SCVM	IPCs	DMS	DH44	Se0ens	Se0ph	PMN LR	MN motor neurons	PaN motor neurons	olfactory PNs	gustatory PNs	multiglomerular PNs	unknown PNs	thermo PNs	visual PNs
15589411	MN-R-Sens-B2-ACp-21	0	0	0	0	2	0	0	0	0	0	0	0	0	0	0	3	0	0	0	0	0

ID: 15651438
name: MN-R-Sens-B2-ACp-22

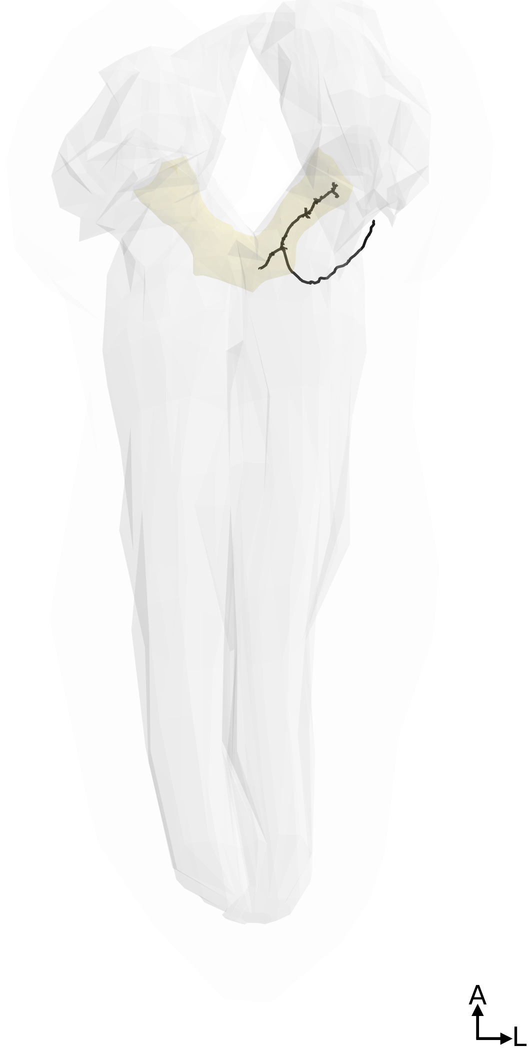

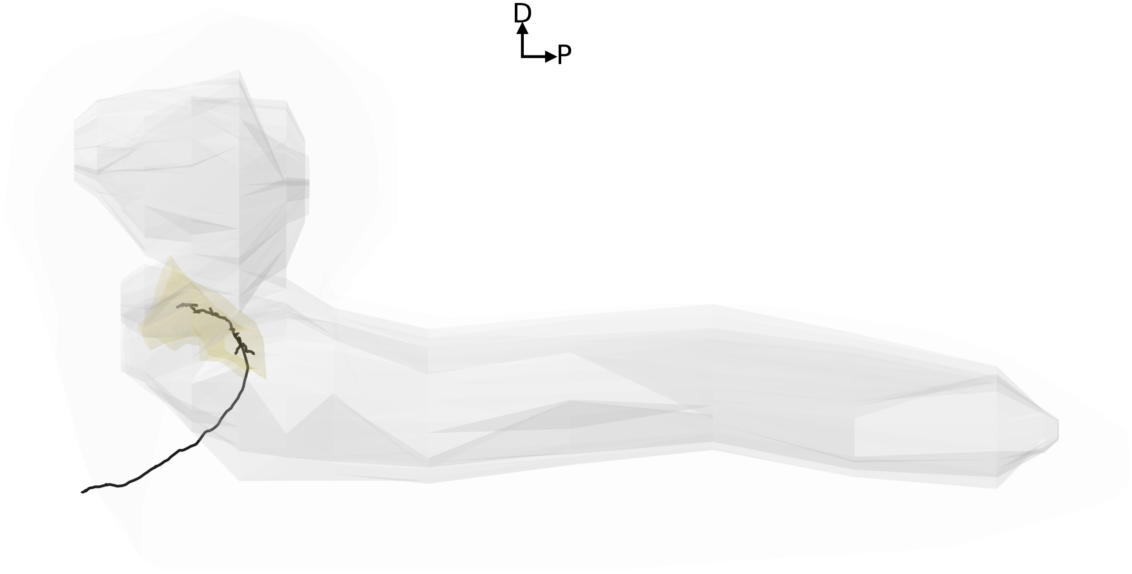

ID	*name*	SCACa	SCAVa	SCAVp	SCACal	SCACp	SCACpl	SCVM	IPCs	DMS	DH44	Se0ens	Se0ph	PMN LR	MN motor neurons	PaN motor neurons	olfactory PNs	gustatory PNs	multiglomerular PNs	unknown PNs	thermo PNs	visual PNs
15651438	MN-R-Sens-B2-ACp-22	0	0	0	0	3	0	0	0	0	0	0	0	0	0	0	0	0	0	0	0	0

ID: 15641926
name: MN-R-Sens-B3-ACp-01

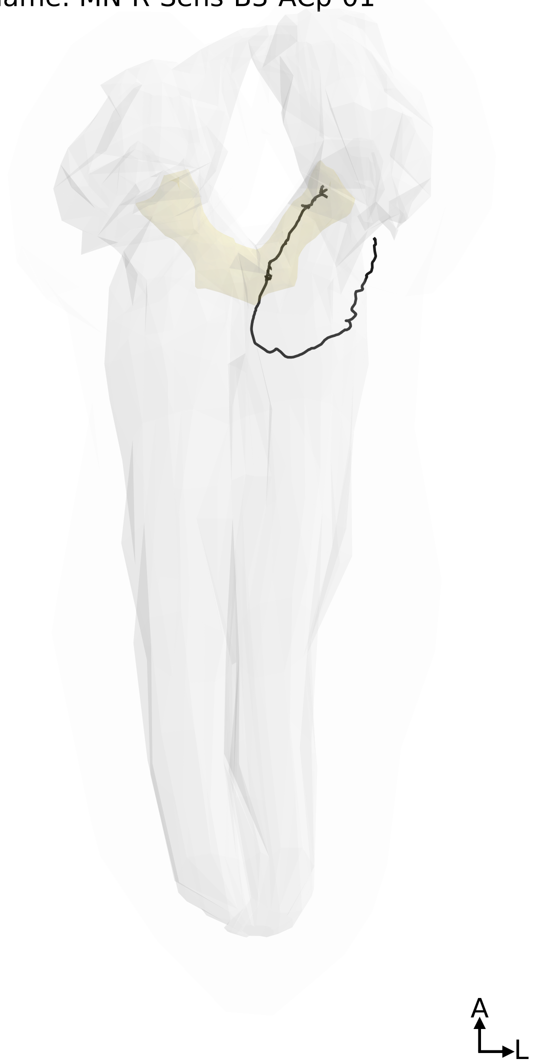

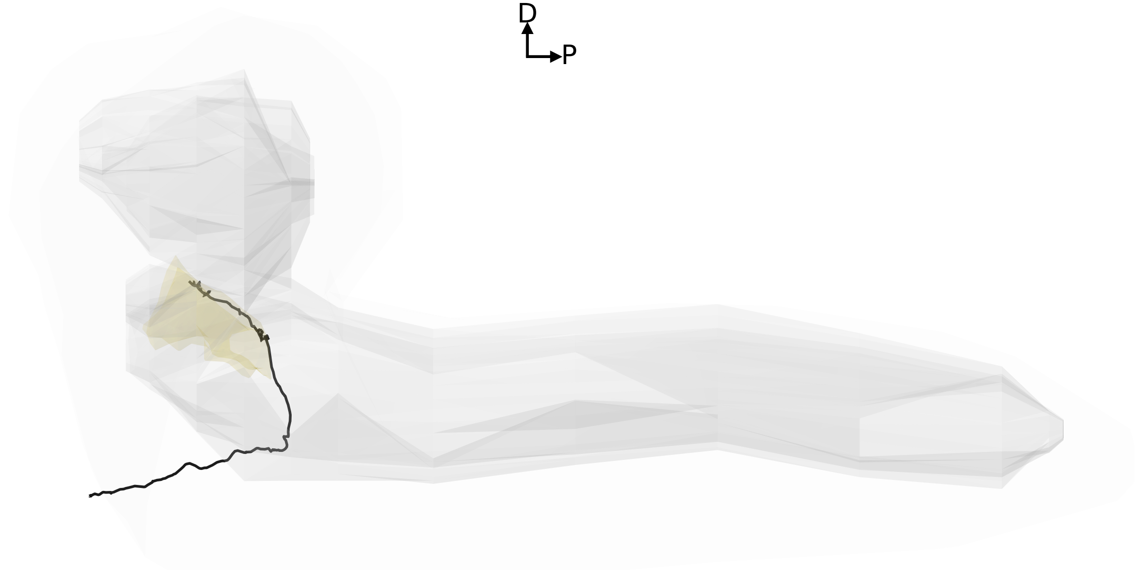

ID	name	SCACa	SCAVa	SCAVp	SCACal	SCACp	SCACpl	SCVM	IPCs	DMS	DH44	Se0ens	Se0ph	PMN LR	MN motor neurons	PaN motor neurons	olfactory PNs	gustatory PNs	multiglomerular PNs	unknown PNs	thermo PNs	visual PNs
15641926	MN-R-Sens-B3-ACp-01	0	0	0	0	17	0	0	0	0	0	0	0	0	0	0	1	0	0	0	1	0

ID: 15639171
name: MN-R-Sens-B3-ACp-02

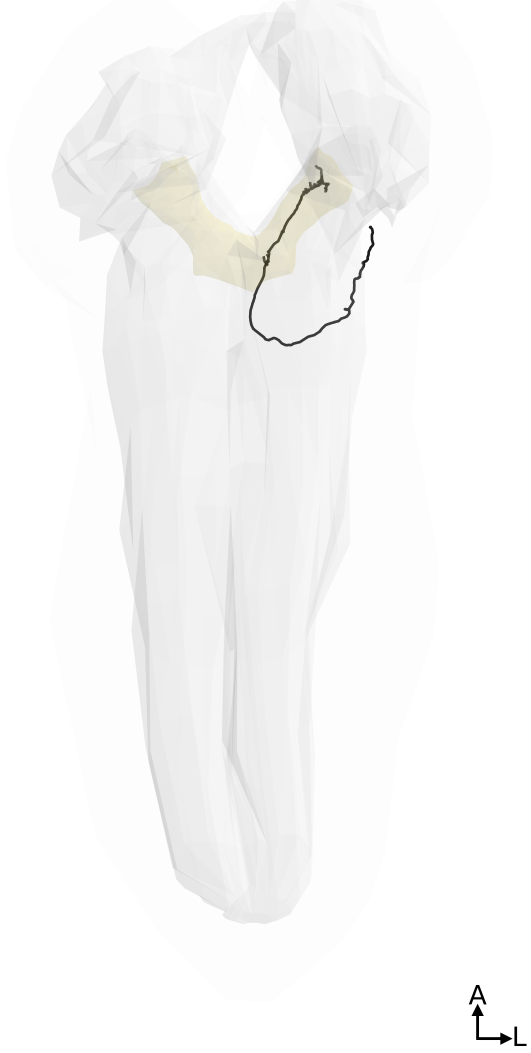

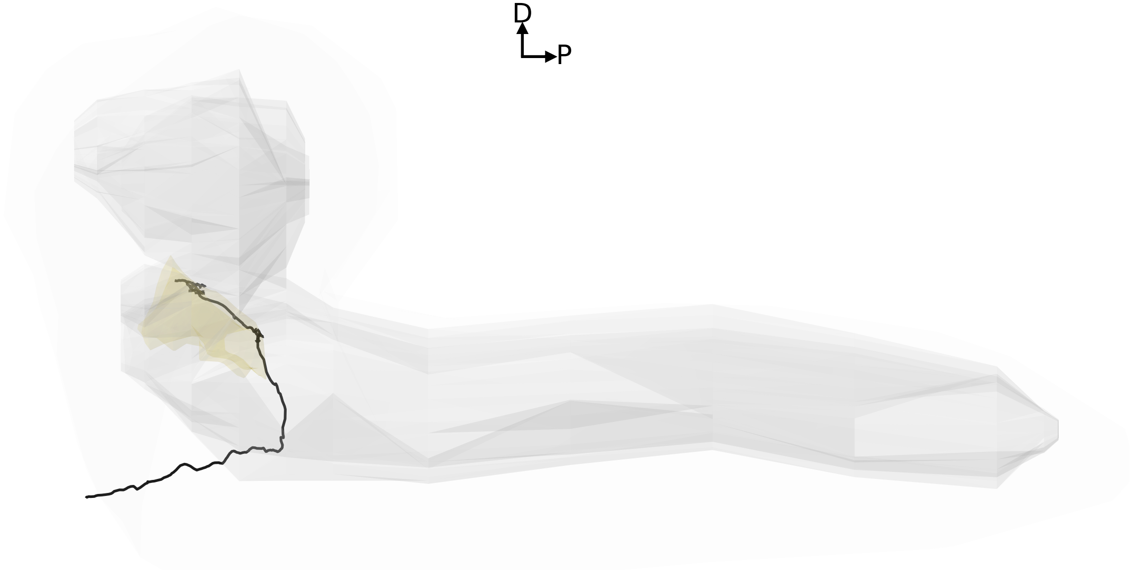

ID	*name*	SCACa	SCAVa	SCAVp	SCACal	SCACp	SCACpl	SCVM	IPCs	DMS	DH44	Se0ens	Se0ph	PMN LR	MN motor neurons	PaN motor neurons	olfactory PNs	gustatory PNs	multiglomerular PNs	unknown PNs	thermo PNs	visual PNs
15639171	MN-R-Sens-B3-ACp-02	0	0	0	0	18	0	0	0	0	0	0	0	0	0	0	2	0	0	0	0	0

ID: 15640893
name: MN-R-Sens-B3-ACp-03

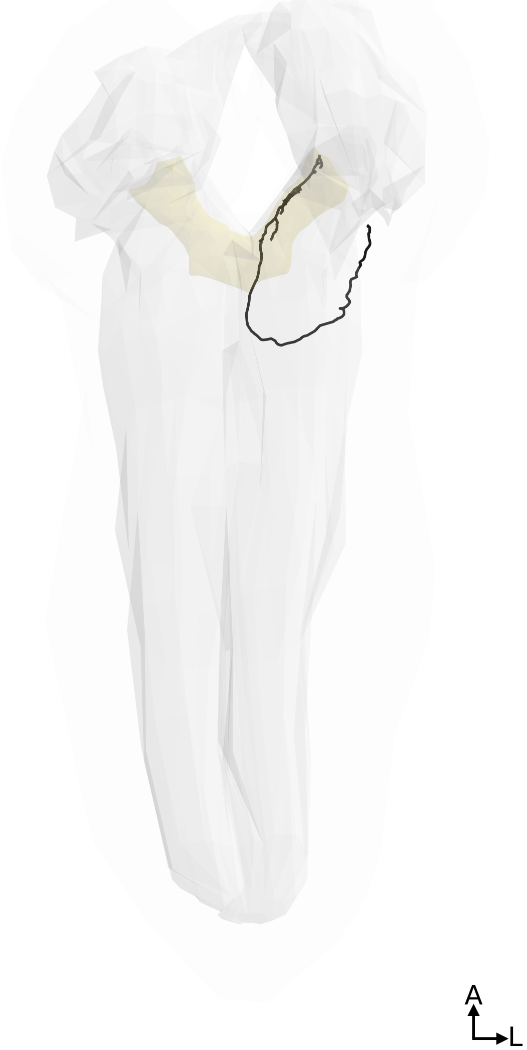

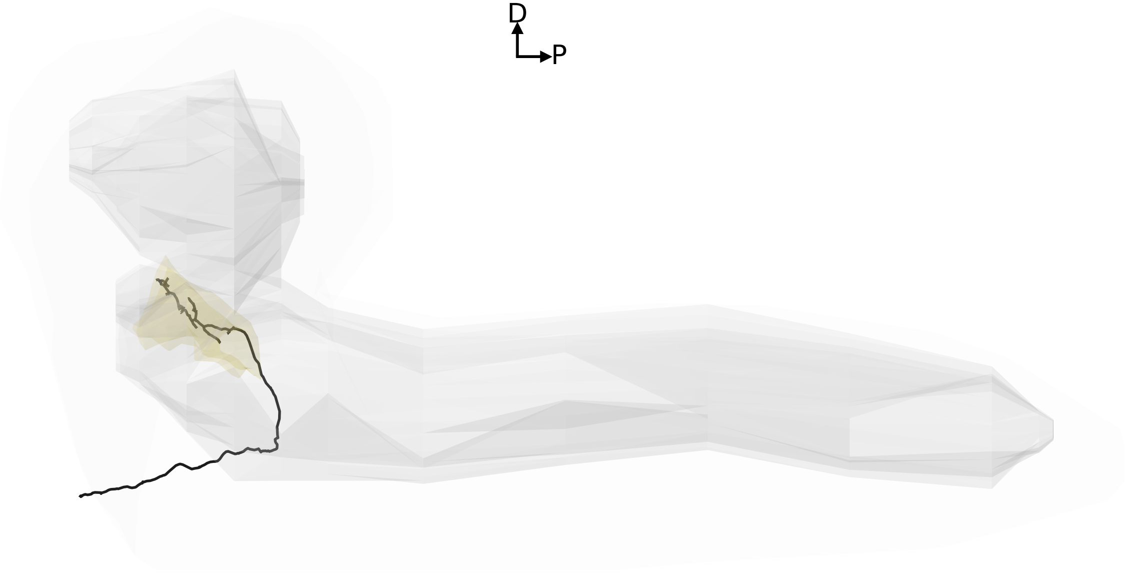

ID	*name*	SCACa	SCAVa	SCAVp	SCACal	SCACp	SCACpl	SCVM	IPCs	DMS	DH44	Se0ens	Se0ph	PMN LR	MN motor neurons	PaN motor neurons	olfactory PNs	gustatory PNs	multiglomerular PNs	unknown PNs	thermo PNs	visual PNs
15640893	MN-R-Sens-B3-ACp-03	1	0	0	0	4	0	0	0	0	1	0	0	0	0	0	0	7	1	0	0	0

ID: 15639020
name: MN-R-Sens-B3-ACp-04

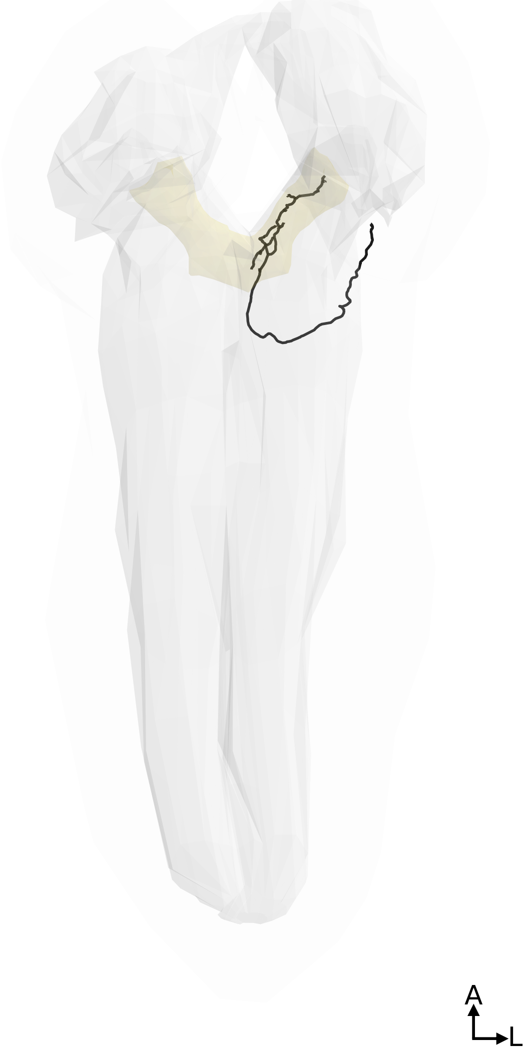

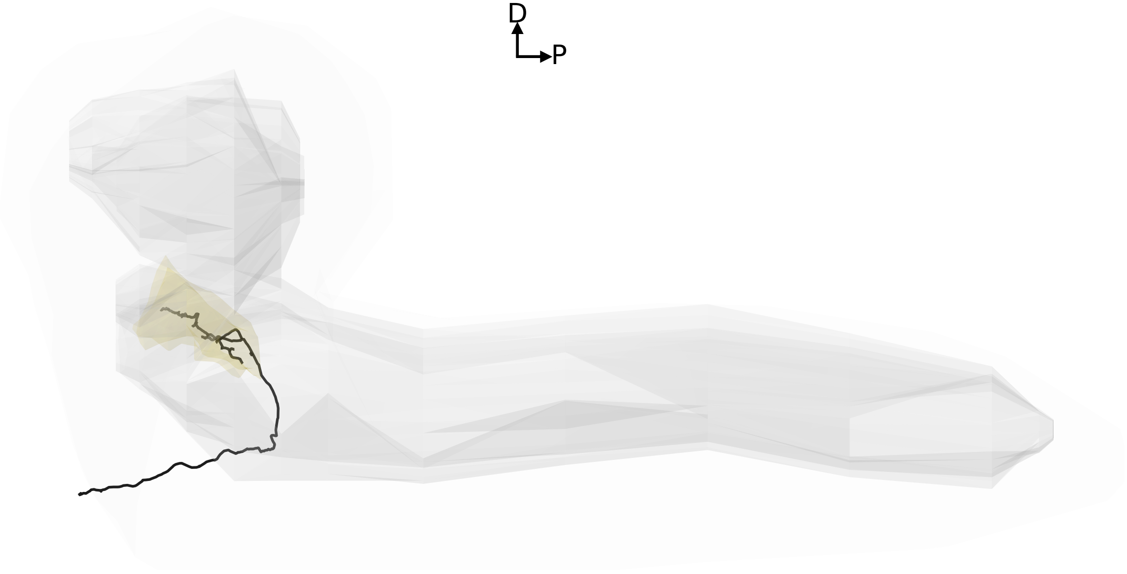

ID	*name*	SCACa	SCAVa	SCAVp	SCACal	SCACp	SCACpl	SCVM	IPCs	DMS	DH44	Se0ens	Se0ph	PMN LR	MN motor neurons	PaN motor neurons	olfactory PNs	gustatory PNs	multiglomerular PNs	unknown PNs	thermo PNs	visual PNs
15639020	MN-R-Sens-B3-ACp-04	0	0	0	0	4	0	0	0	0	0	0	0	0	0	0	1	0	0	0	0	0

ID: 3199456
name: MN-R-Sens-B3-ACp-05

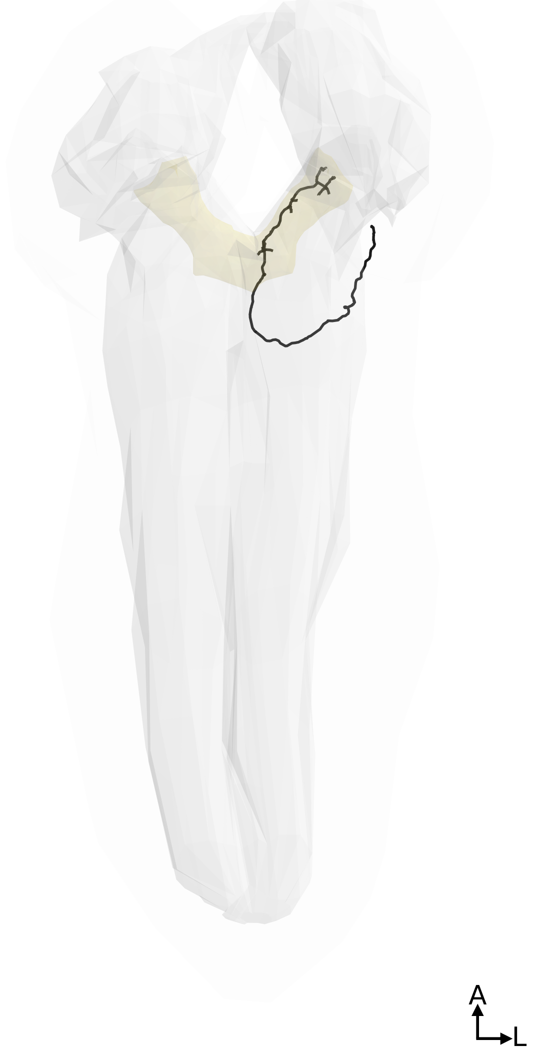

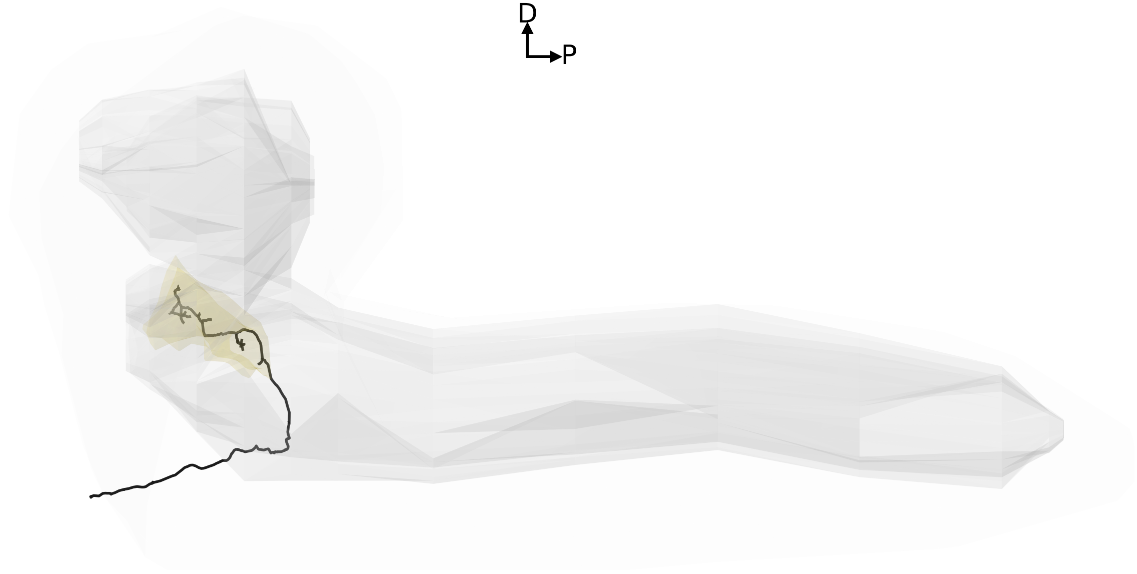

ID	*name*	SCACa	SCAVa	SCAVp	SCACal	SCACp	SCACpl	SCVM	IPCs	DMS	DH44	Se0ens	Se0ph	PMN LR	MN motor neurons	PaN motor neurons	olfactory PNs	gustatory PNs	multiglomerular PNs	unknown PNs	thermo PNs	visual PNs
3199456	MN-R-Sens-B3-ACp-05	0	0	0	0	9	0	0	0	0	0	0	0	0	0	0	0	1	4	0	0	0

ID: 15645825
name: MN-R-Sens-B3-ACp-06

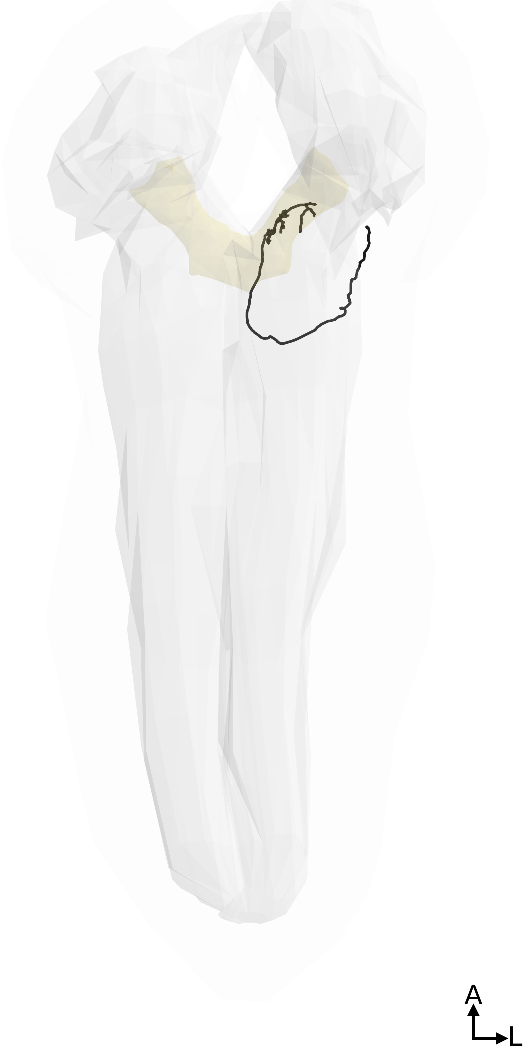

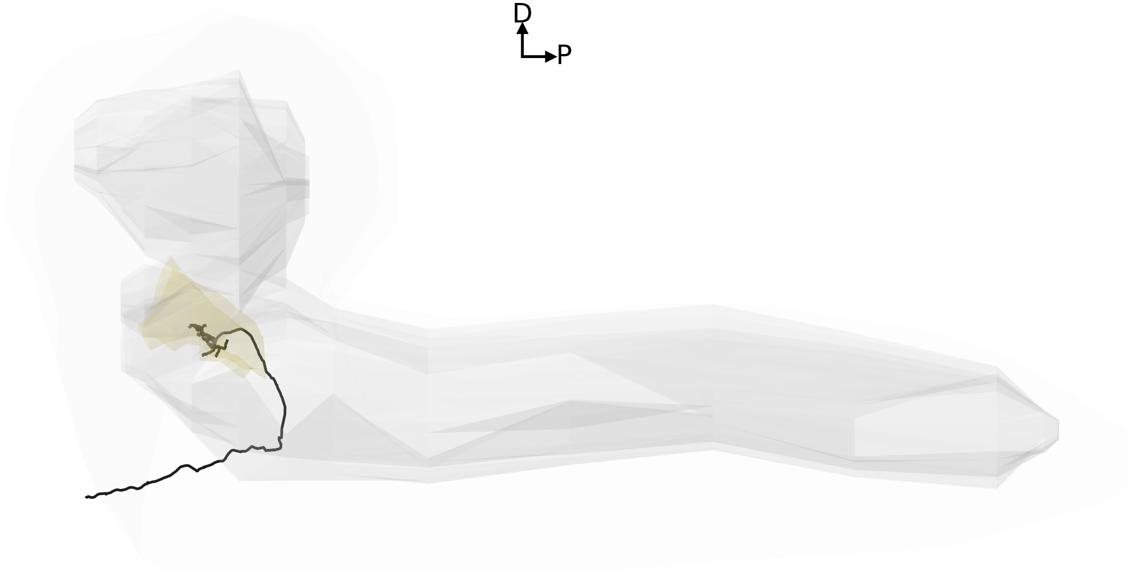

ID	*name*	SCACa	SCAVa	SCAVp	SCACal	SCACp	SCACpl	SCVM	IPCs	DMS	DH44	Se0ens	Se0ph	PMN LR	MN motor neurons	PaN motor neurons	olfactory PNs	gustatory PNs	multiglomerular PNs	unknown PNs	thermo PNs	visual PNs
15645825	MN-R-Sens-B3-ACp-06	0	0	0	0	1	0	0	0	0	0	0	0	0	0	0	0	0	0	0	0	0

ID: 5088293
name: AN-L-Sens-B3-ACpl-10

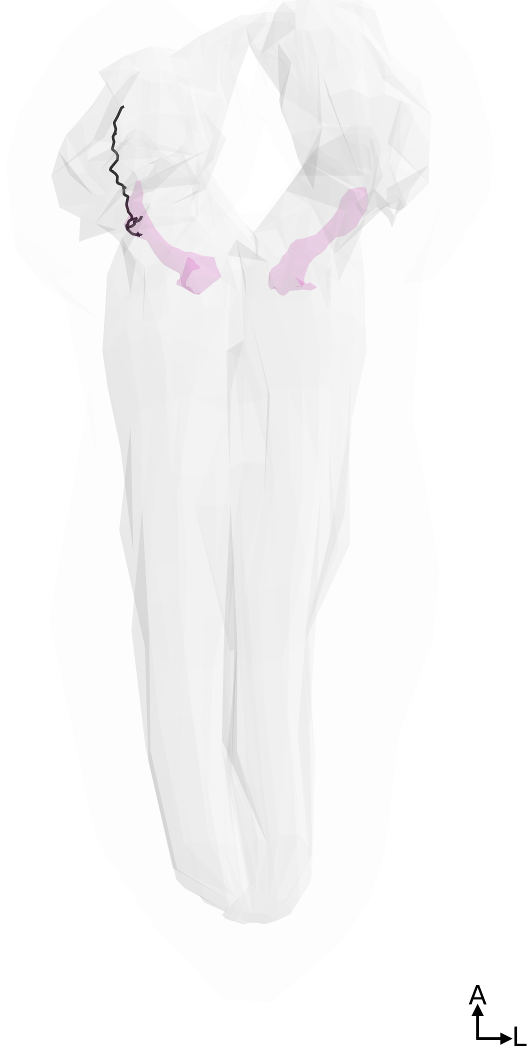

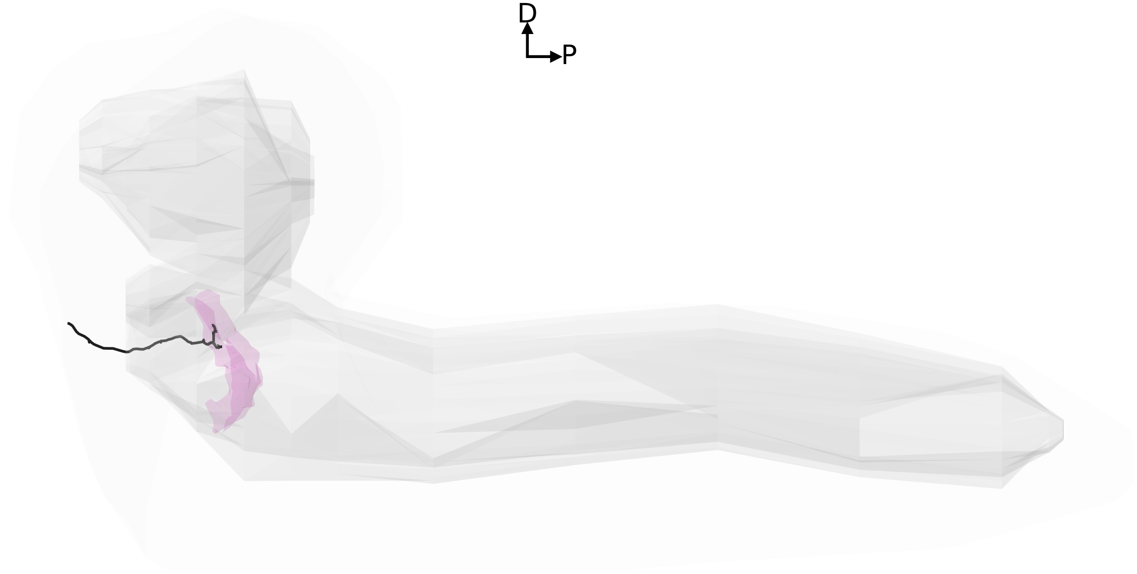

ID	name	SCACa	SCAVa	SCAVp	SCACal	SCACp	SCACpl	SCVM	IPCs	DMS	DH44	Se0ens	Se0ph	PMN LR	MN motor neurons	PaN motor neurons	olfactory PNs	gustatory PNs	multiglomerular PNs	unknown PNs	thermo PNs	visual PNs
5088293	AN-L-Sens-B3-ACpl-10	0	0	0	0	0	0	0	0	0	0	0	0	0	0	0	0	0	0	0	0	0

ID: 4097554
name: AN-R-Sens-B3-ACpl-10

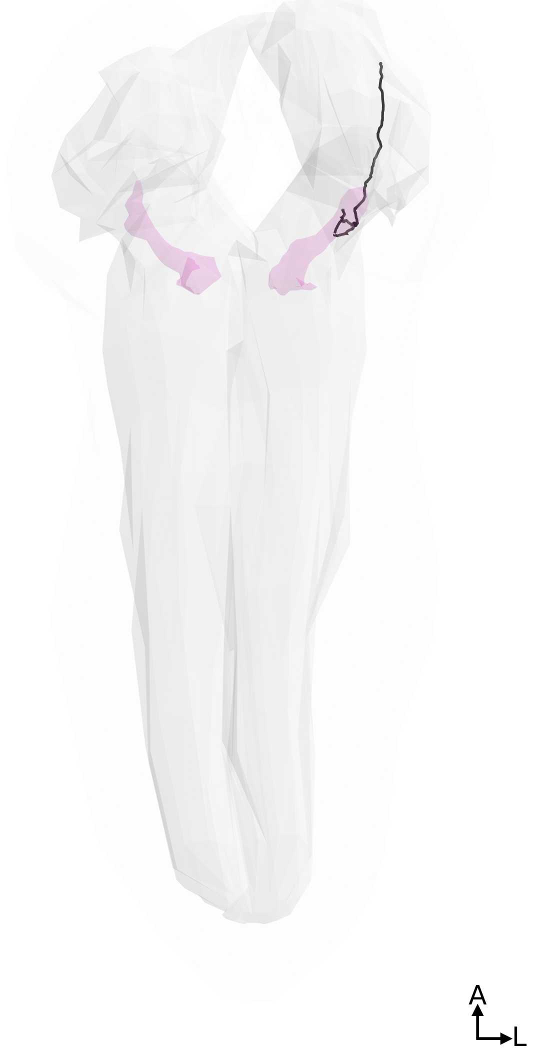

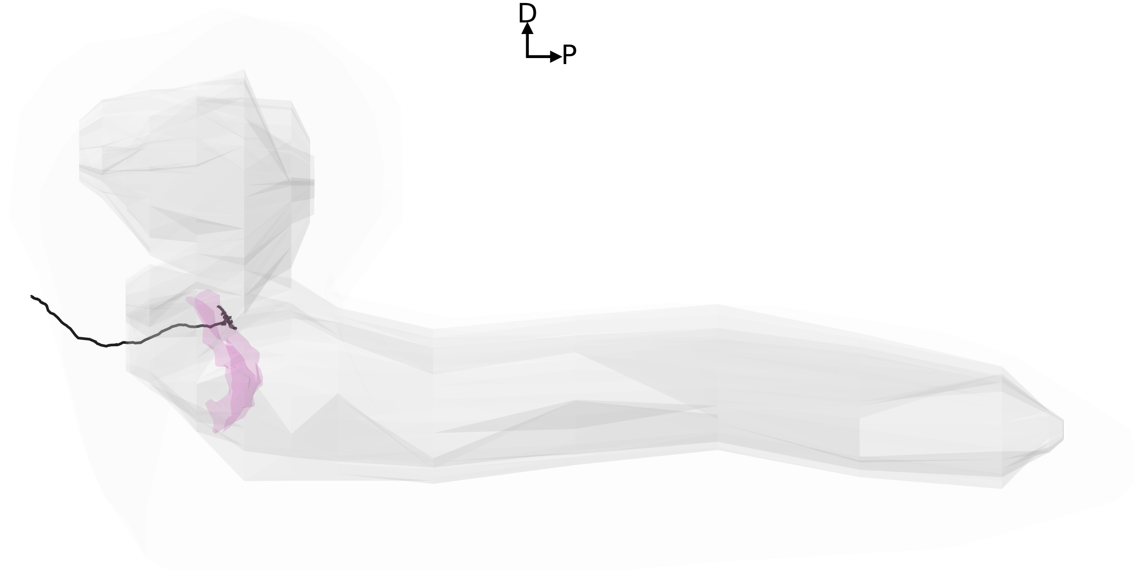

ID	*name*	SCACa	SCAVa	SCAVp	SCACal	SCACp	SCACpl	SCVM	IPCs	DMS	DH44	Se0ens	Se0ph	PMN LR	MN motor neurons	PaN motor neurons	olfactory PNs	gustatory PNs	multiglomerular PNs	unknown PNs	thermo PNs	visual PNs
4097554	AN-R-Sens-B3-ACpl-10	0	0	0	0	0	0	0	0	0	0	0	0	0	0	0	0	0	3	0	0	0

ID: 8055671
name: MN-L-Sens-B1-ACpl-01

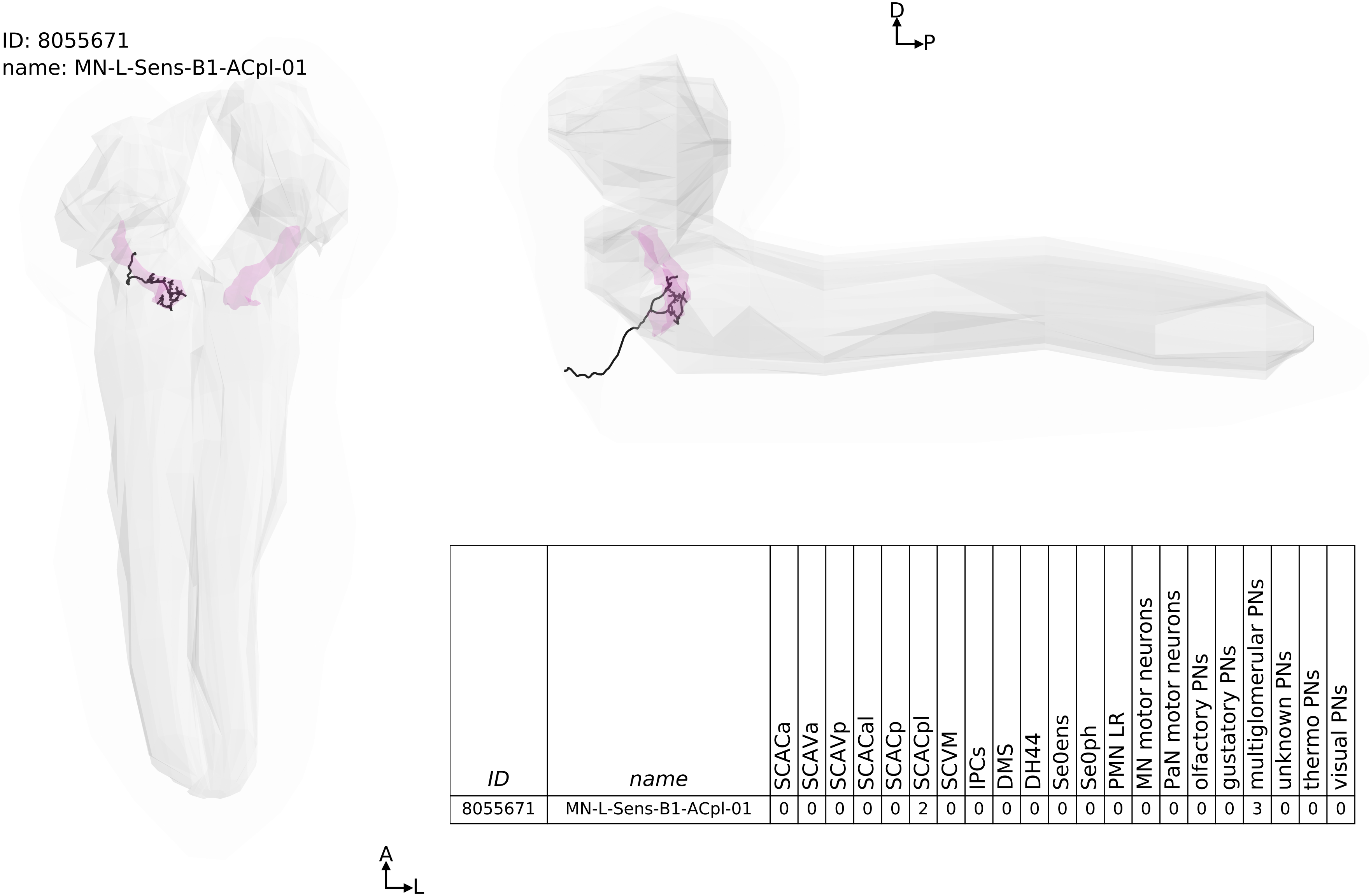

ID	*name*	SCACa	SCAVa	SCAVp	SCACal	SCACp	SCACpl	SCVM	IPCs	DMS	DH44	Se0ens	Se0ph	PMN LR	MN motor neurons	PaN motor neurons	olfactory PNs	gustatory PNs	multiglomerular PNs	unknown PNs	thermo PNs	visual PNs
8055671	MN-L-Sens-B1-ACpl-01	0	0	0	0	0	2	0	0	0	0	0	0	0	0	0	0	0	3	0	0	0

ID: 15714639
name: MN-L-Sens-B2-ACpl-01

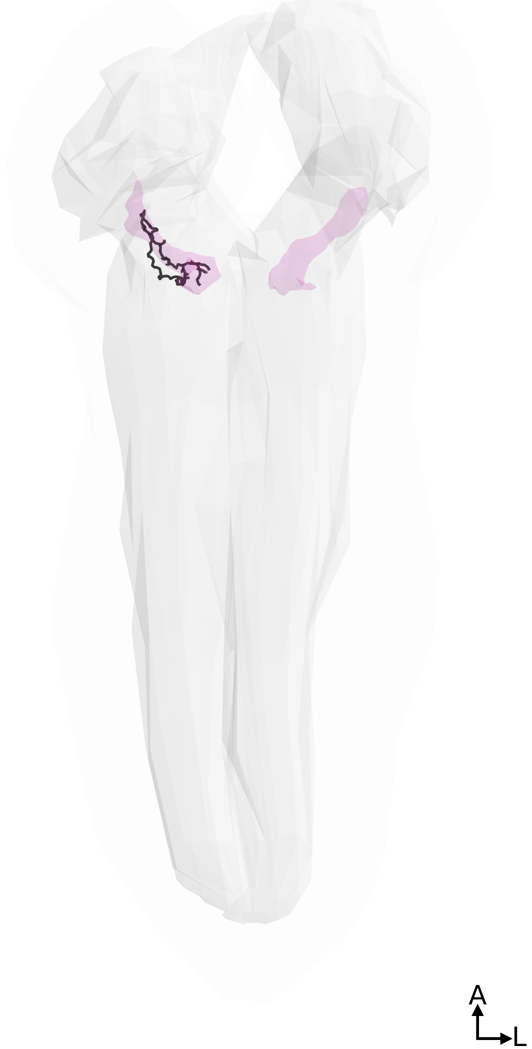

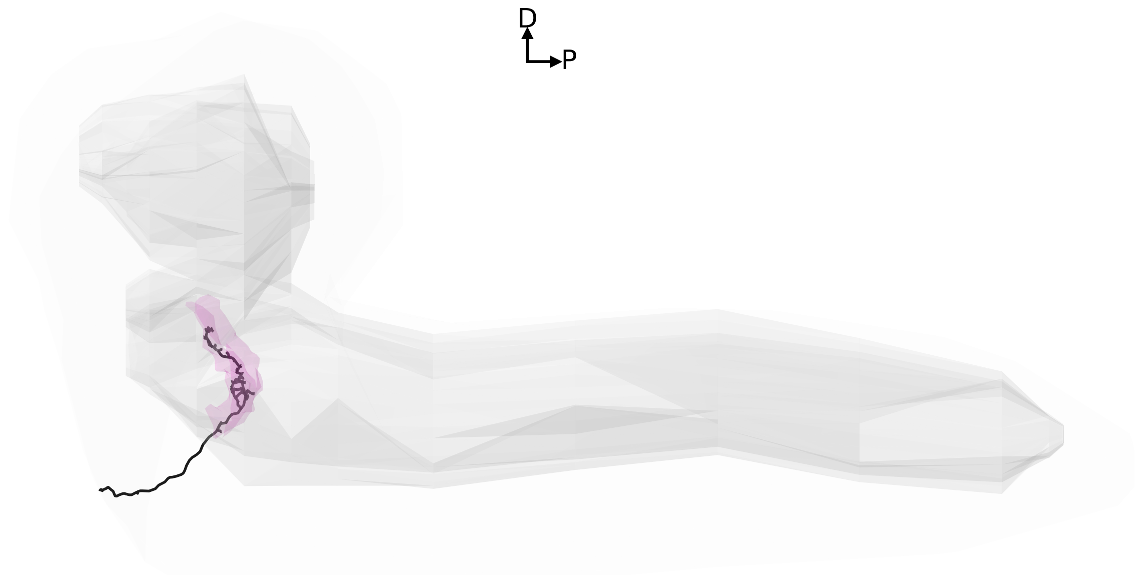

ID	name	SCACa	SCAVa	SCAVp	SCACal	SCACp	SCACpl	SCVM	IPCs	DMS	DH44	Se0ens	Se0ph	PMN LR	MN motor neurons	PaN motor neurons	olfactory PNs	gustatory PNs	multiglomerular PNs	unknown PNs	thermo PNs	visual PNs
15714639	MN-L-Sens-B2-ACpl-01	0	0	0	0	0	1	2	0	0	0	0	0	0	0	0	0	0	23	0	0	0

ID: 15716934
name: MN-L-Sens-B2-ACpl-02

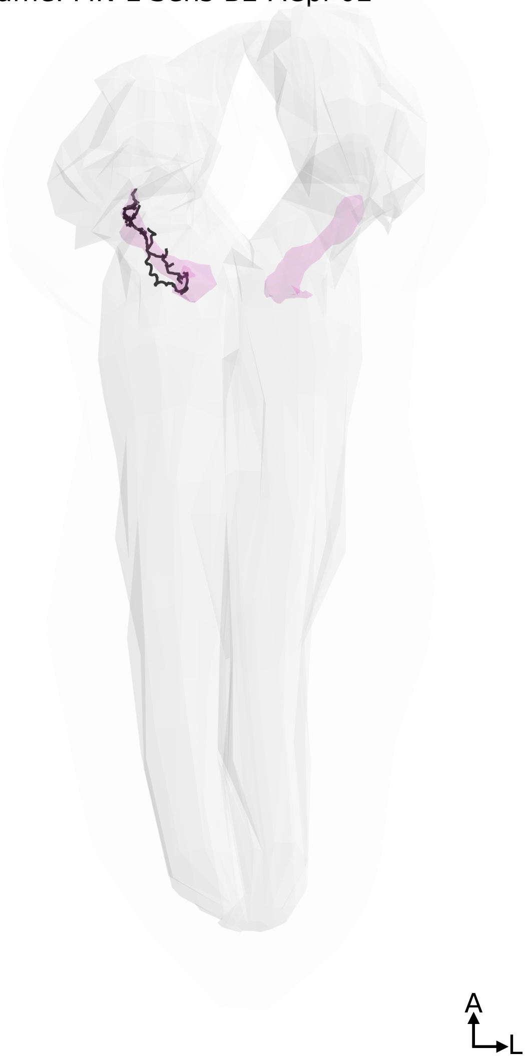

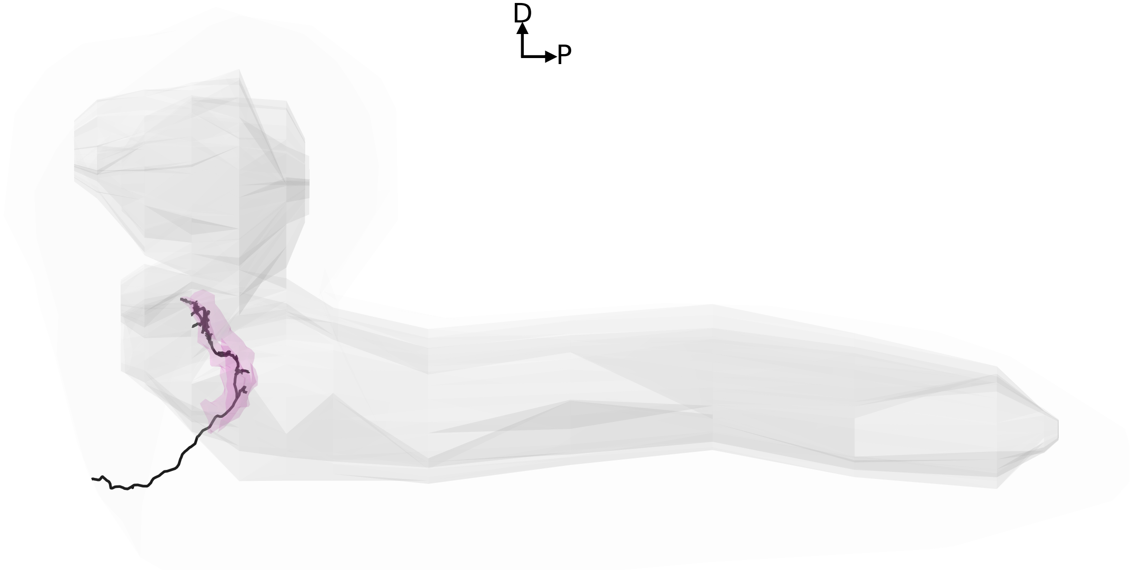

ID	*name*	SCACa	SCAVa	SCAVp	SCACal	SCACp	SCACpl	SCVM	IPCs	DMS	DH44	Se0ens	Se0ph	PMN LR	MN motor neurons	PaN motor neurons	olfactory PNs	gustatory PNs	multiglomerular PNs	unknown PNs	thermo PNs	visual PNs
15716934	MN-L-Sens-B2-ACpl-02	0	0	0	0	0	0	0	0	0	0	0	0	0	0	0	0	0	107	0	0	0

ID: 15673925
name: MN-L-Sens-B2-ACpl-03

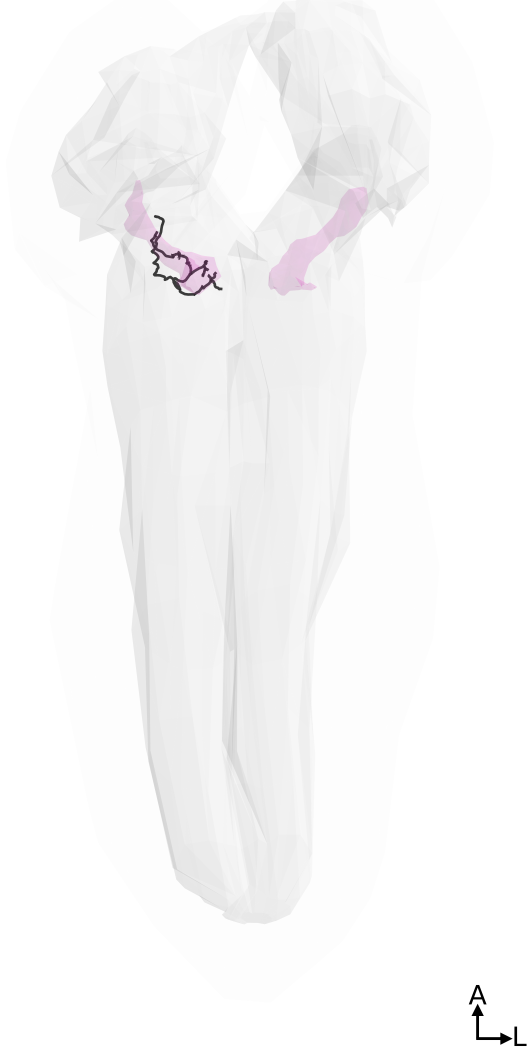

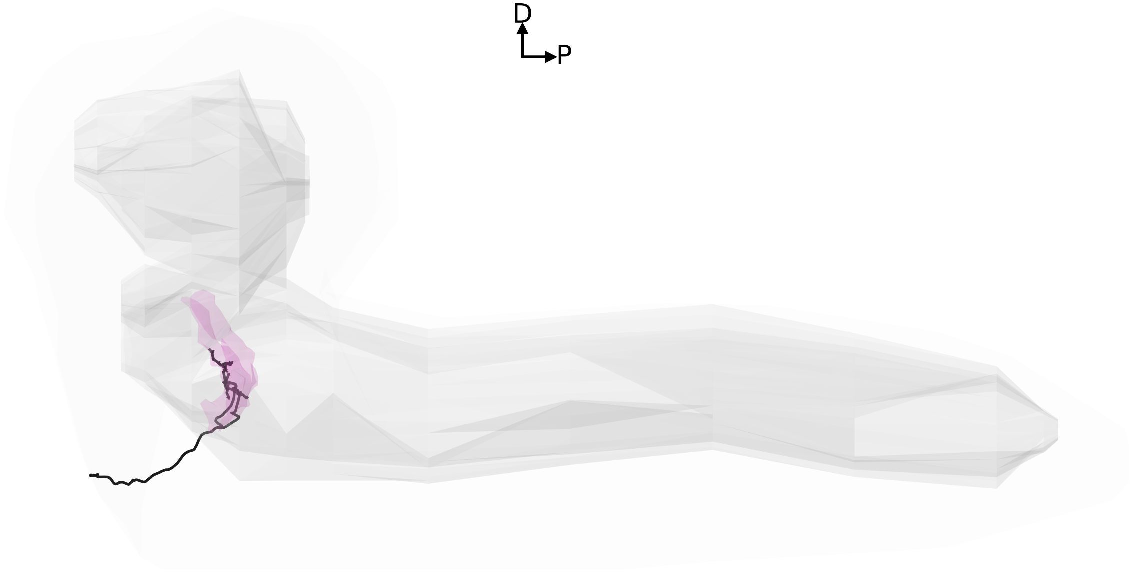

ID	*name*	SCACa	SCAVa	SCAVp	SCACal	SCACp	SCACpl	SCVM	IPCs	DMS	DH44	Se0ens	Se0ph	PMN LR	MN motor neurons	PaN motor neurons	olfactory PNs	gustatory PNs	multiglomerular PNs	unknown PNs	thermo PNs	visual PNs
15673925	MN-L-Sens-B2-ACpl-03	0	0	0	0	0	2	1	0	0	0	0	0	0	0	0	0	0	0	0	0	0

ID: 15717935
name: MN-L-Sens-B3-ACpl-07

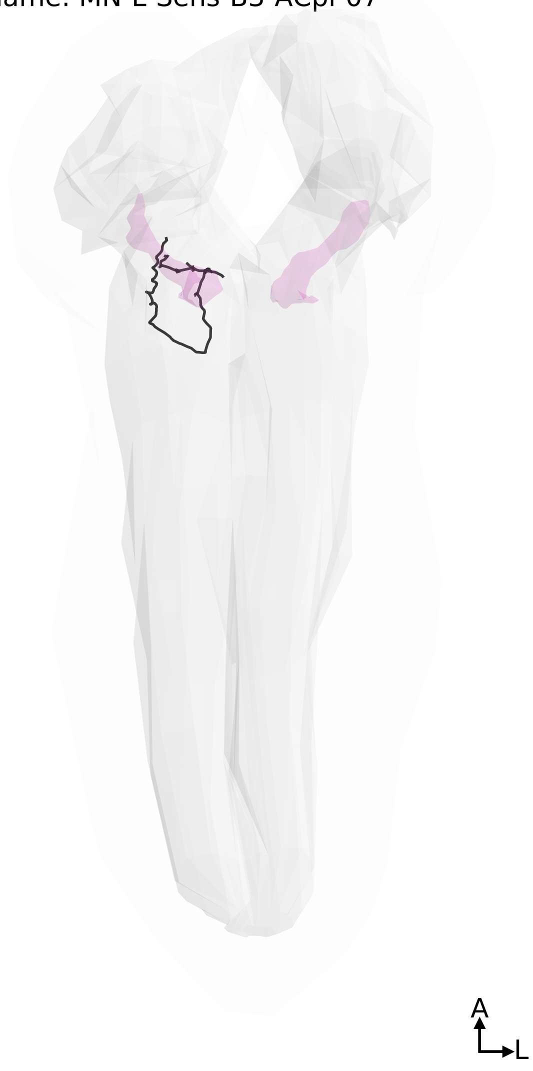

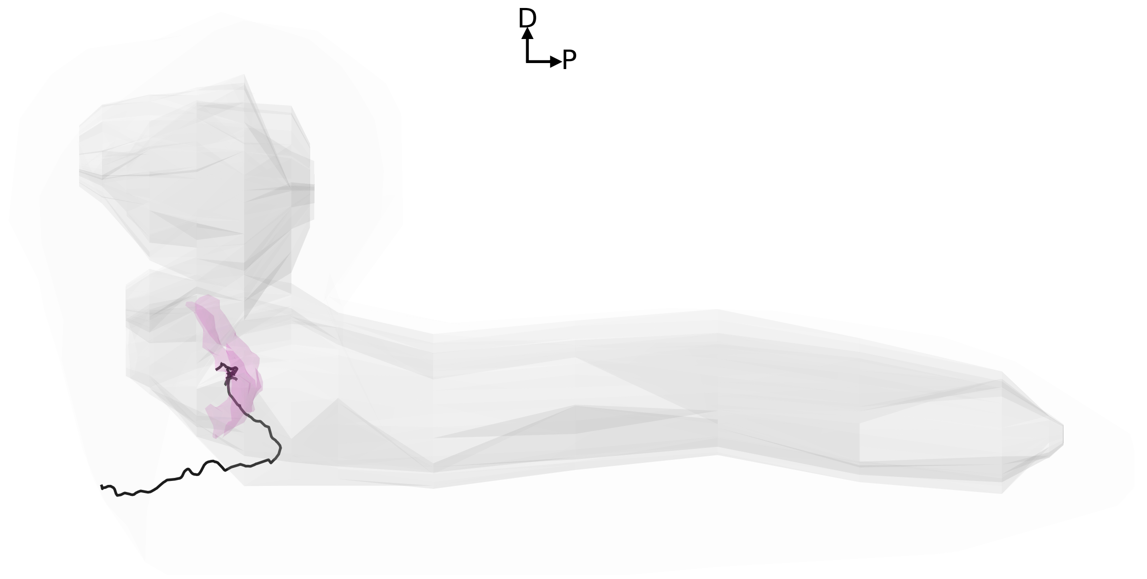

ID	*name*	SCACa	SCAVa	SCAVp	SCACal	SCACp	SCACpl	SCVM	IPCs	DMS	DH44	Se0ens	Se0ph	PMN LR	MN motor neurons	PaN motor neurons	olfactory PNs	gustatory PNs	multiglomerular PNs	unknown PNs	thermo PNs	visual PNs
15717935	MN-L-Sens-B3-ACpl-07	0	0	0	0	0	1	0	0	0	0	0	0	0	0	0	0	0	0	0	0	0

ID: 5448622
name: MN-R-Sens-B1-ACpl-01

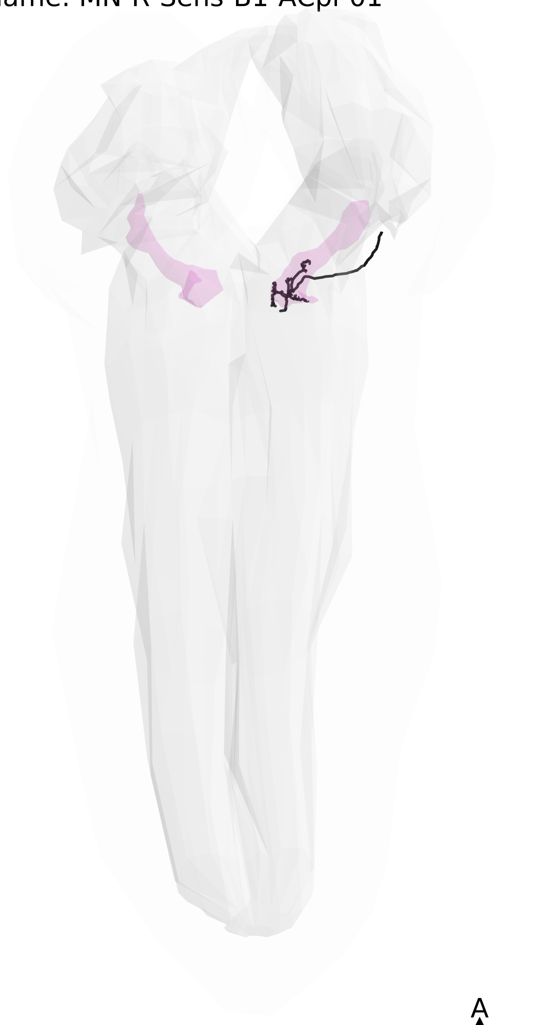

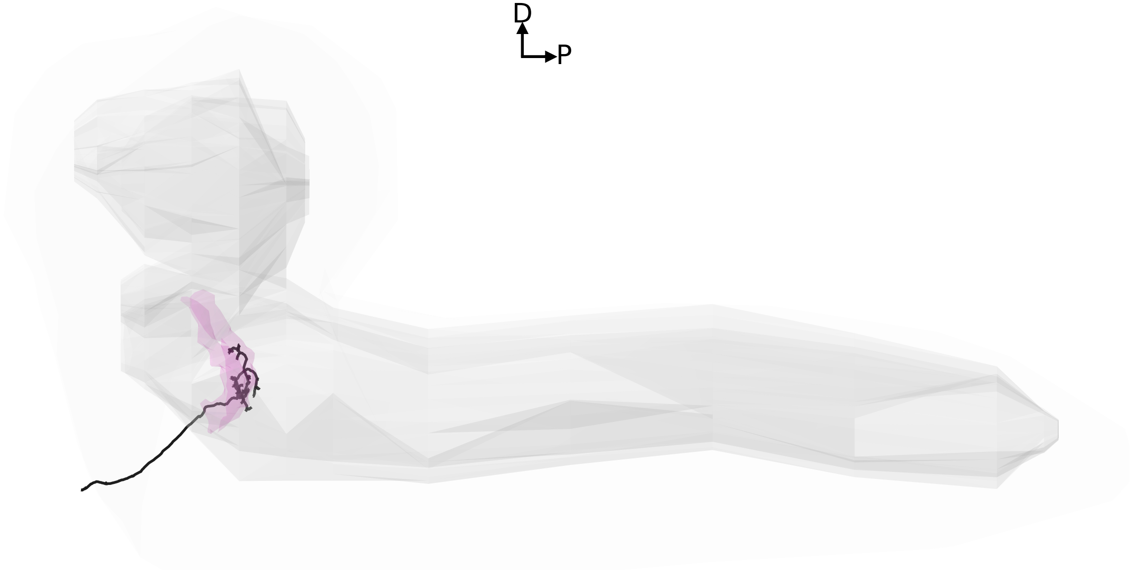

ID	*name*	SCACa	SCAVa	SCAVp	SCACal	SCACp	SCACpl	SCVM	IPCs	DMS	DH44	Se0ens	Se0ph	PMN LR	MN motor neurons	PaN motor neurons	olfactory PNs	gustatory PNs	multiglomerular PNs	unknown PNs	thermo PNs	visual PNs
5448622	MN-R-Sens-B1-ACpl-01	0	0	0	0	0	1	0	0	0	0	0	0	0	0	0	0	0	0	0	0	0

ID: 15595950
name: MN-R-Sens-B2-ACpl-01

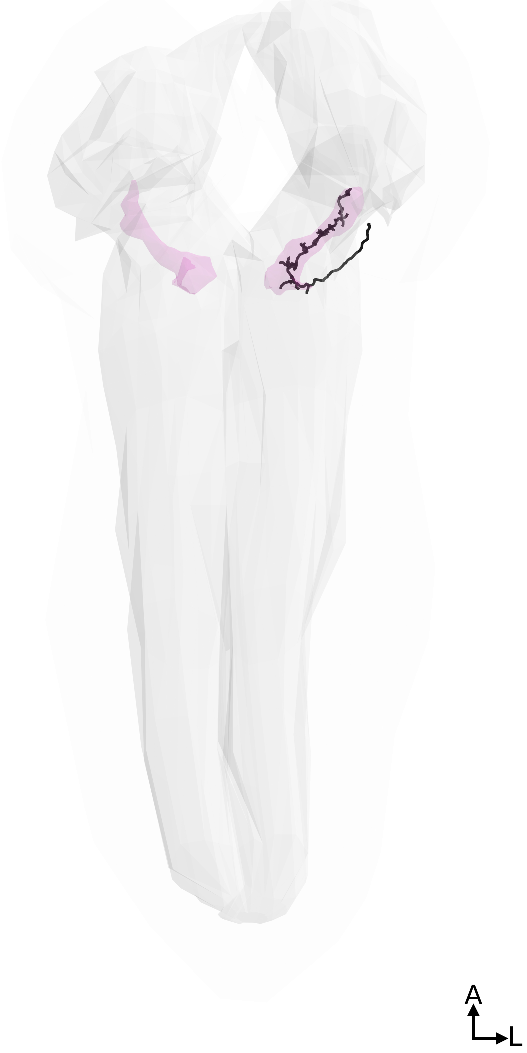

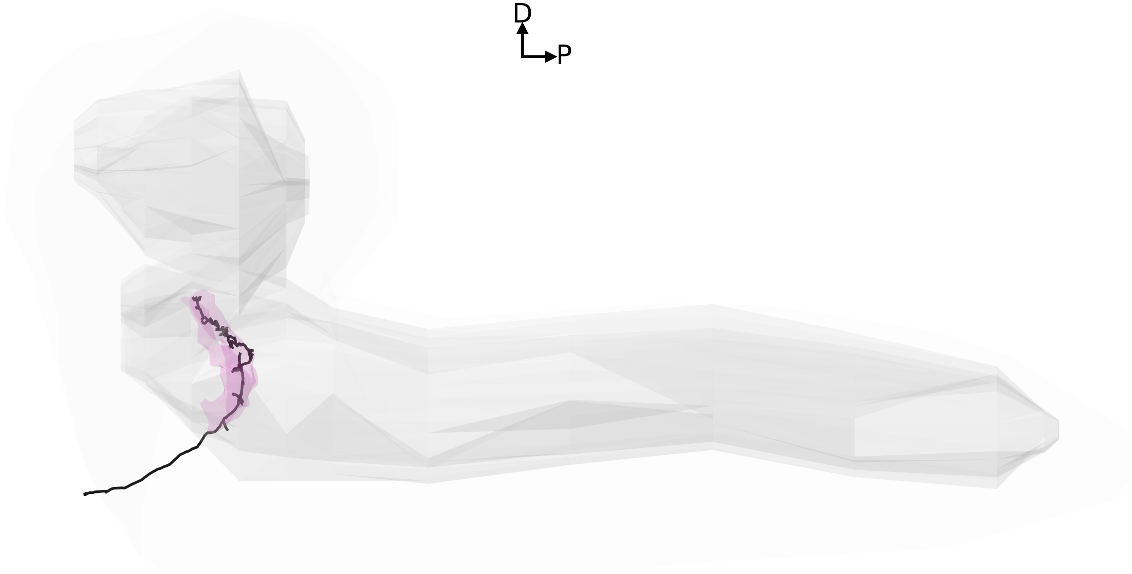

ID	*name*	SCACa	SCAVa	SCAVp	SCACal	SCACp	SCACpl	SCVM	IPCs	DMS	DH44	Se0ens	Se0ph	PMN LR	MN motor neurons	PaN motor neurons	olfactory PNs	gustatory PNs	multiglomerular PNs	unknown PNs	thermo PNs	visual PNs
15595950	MN-R-Sens-B2-ACpl-01	0	0	0	0	0	1	1	0	0	0	0	0	0	0	0	0	0	27	0	0	0

ID: 15746421
name: MN-R-Sens-B2-ACpl-02

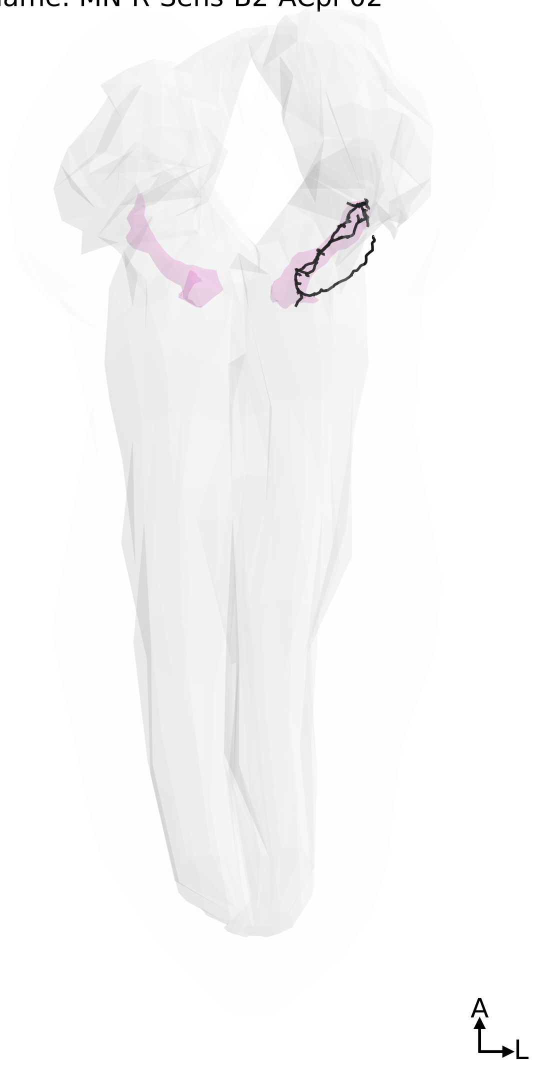

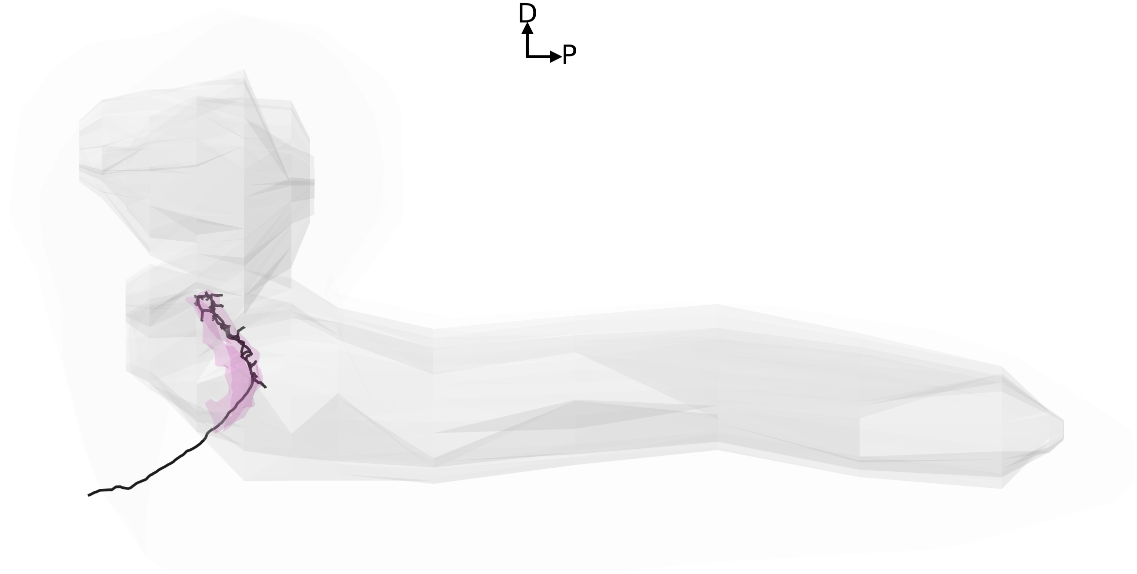

ID	*name*	SCACa	SCAVa	SCAVp	SCACal	SCACp	SCACpl	SCVM	IPCs	DMS	DH44	Se0ens	Se0ph	PMN LR	MN motor neurons	PaN motor neurons	olfactory PNs	gustatory PNs	multiglomerular PNs	unknown PNs	thermo PNs	visual PNs
15746421	MN-R-Sens-B2-ACpl-02	0	0	0	0	1	0	0	0	0	0	0	0	0	0	0	0	0	100	0	0	0

ID: 15615776
name: MN-R-Sens-B2-ACpl-03

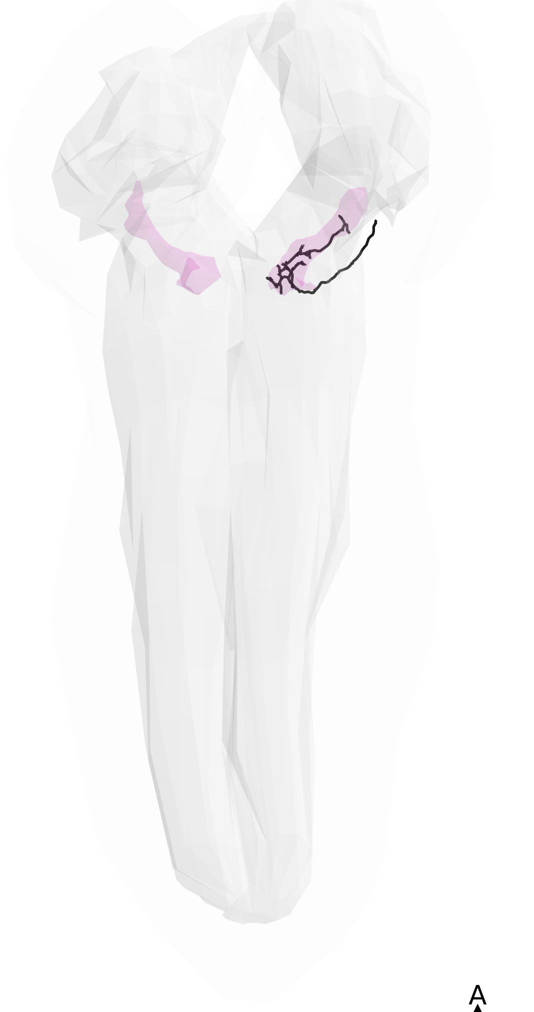

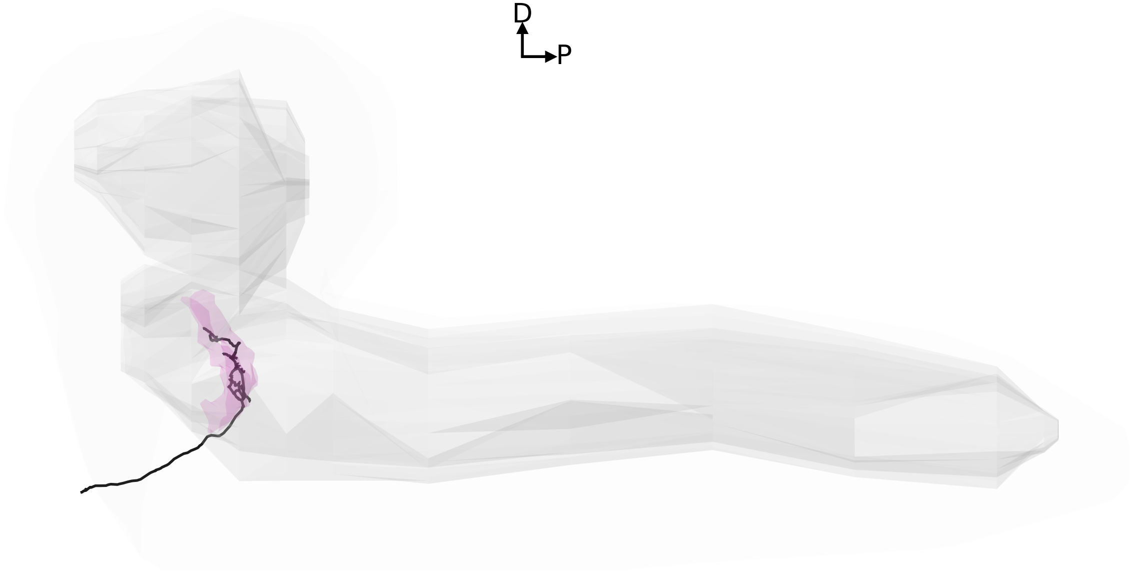

ID	*name*	SCACa	SCAVa	SCAVp	SCACal	SCACp	SCACpl	SCVM	IPCs	DMS	DH44	Se0ens	Se0ph	PMN LR	MN motor neurons	PaN motor neurons	olfactory PNs	gustatory PNs	multiglomerular PNs	unknown PNs	thermo PNs	visual PNs
15615776	MN-R-Sens-B2-ACpl-03	0	0	0	0	0	0	0	0	0	0	0	0	0	0	0	0	0	1	0	0	0

ID: 3123905
name: MN-R-Sens-B3-ACpl-07

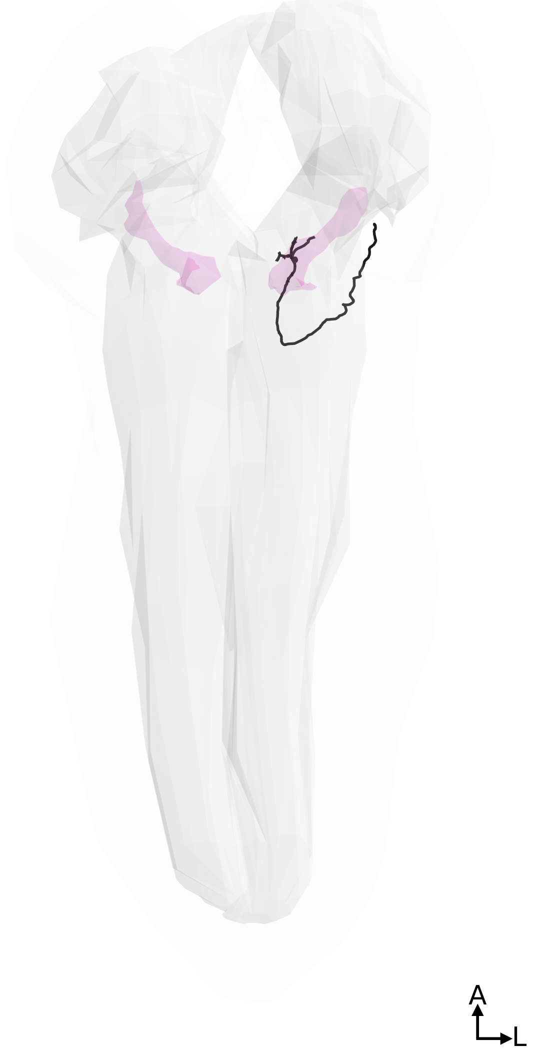

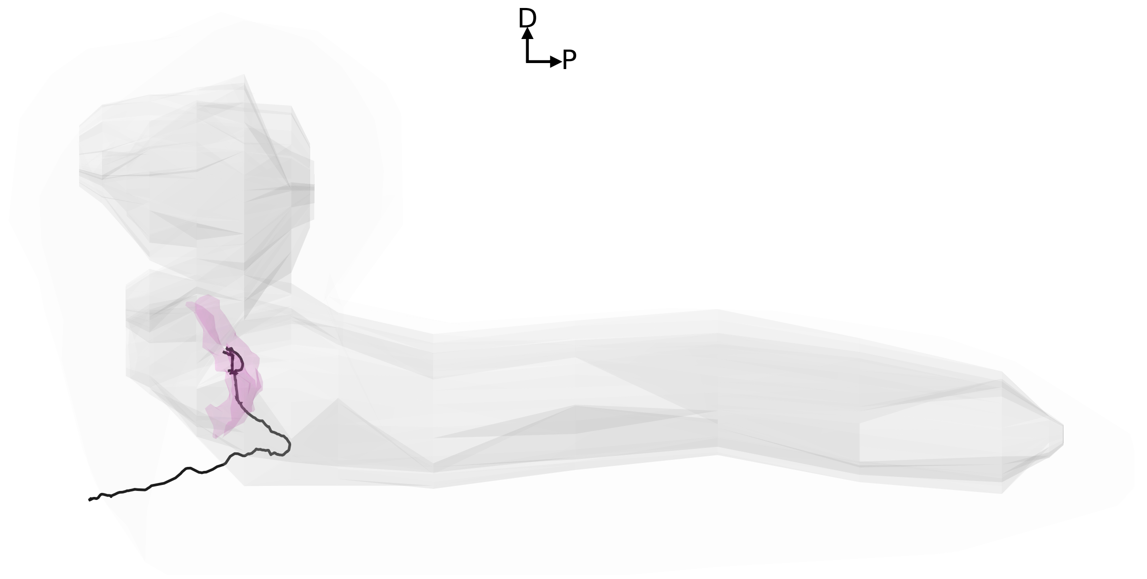

ID	*name*	SCACa	SCAVa	SCAVp	SCACal	SCACp	SCACpl	SCVM	IPCs	DMS	DH44	Se0ens	Se0ph	PMN LR	MN motor neurons	PaN motor neurons	olfactory PNs	gustatory PNs	multiglomerular PNs	unknown PNs	thermo PNs	visual PNs
3123905	MN-R-Sens-B3-ACpl-07	0	0	0	0	0	0	0	0	0	0	0	0	0	0	0	0	0	2	0	0	0

ID: 15998913
name: AN-L-Sens-B2-VM-04

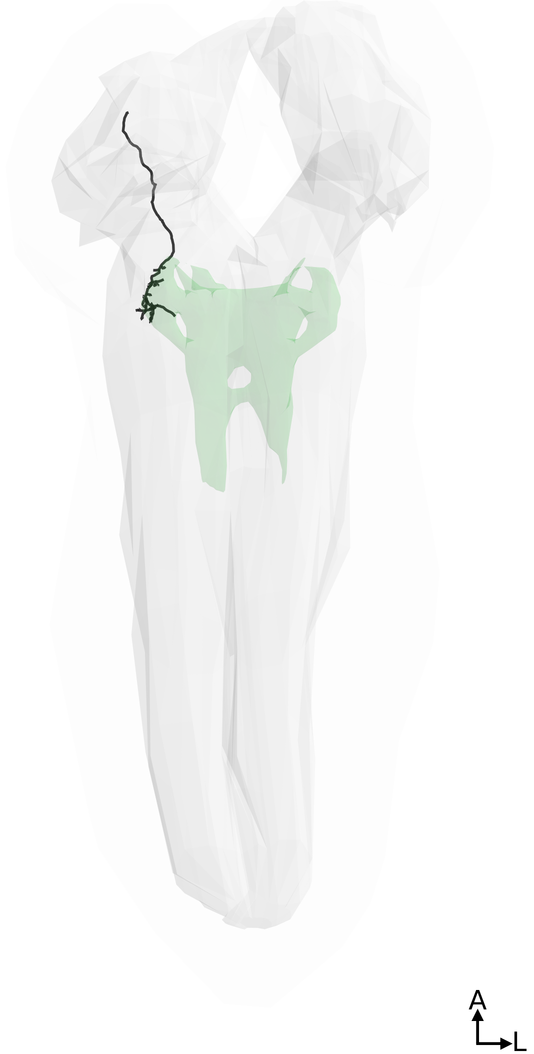

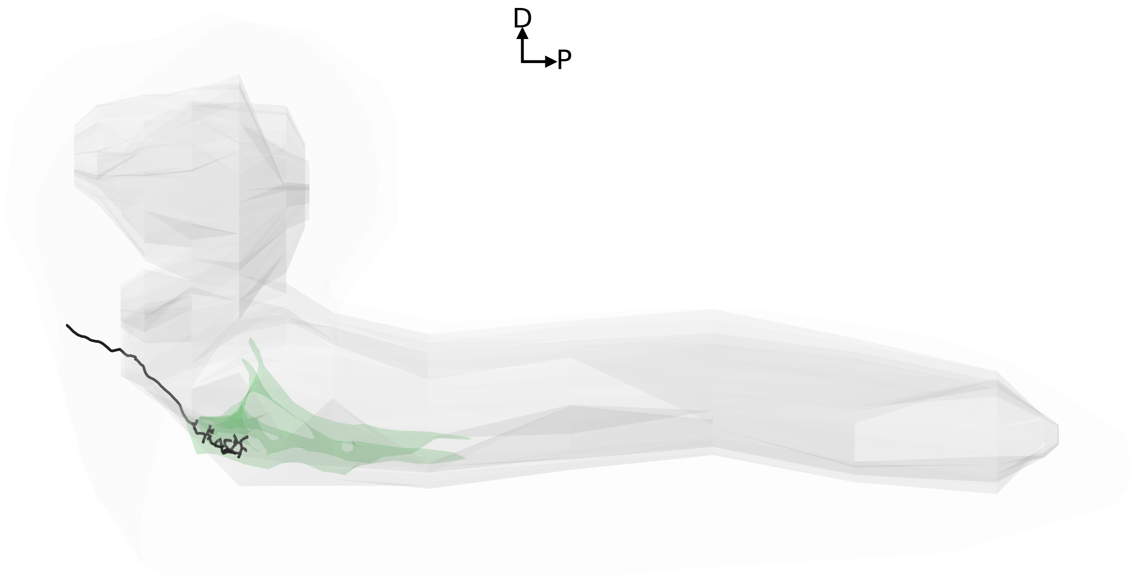

ID	*name*	SCACa	SCAVa	SCAVp	SCACal	SCACp	SCACpl	SCVM	IPCs	DMS	DH44	Se0ens	Se0ph	PMN LR	MN motor neurons	PaN motor neurons	olfactory PNs	gustatory PNs	multiglomerular PNs	unknown PNs	thermo PNs	visual PNs
15998913	AN-L-Sens-B2-VM-04	0	0	0	0	0	0	0	0	0	0	0	0	0	5	2	0	0	0	0	0	0

ID: 15998319
name: AN-L-Sens-B2-VM-05

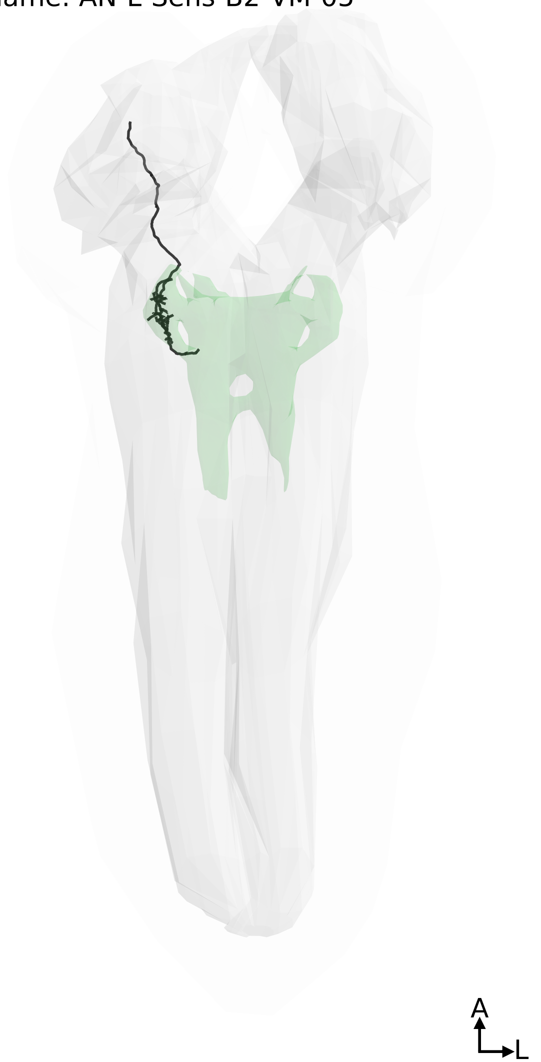

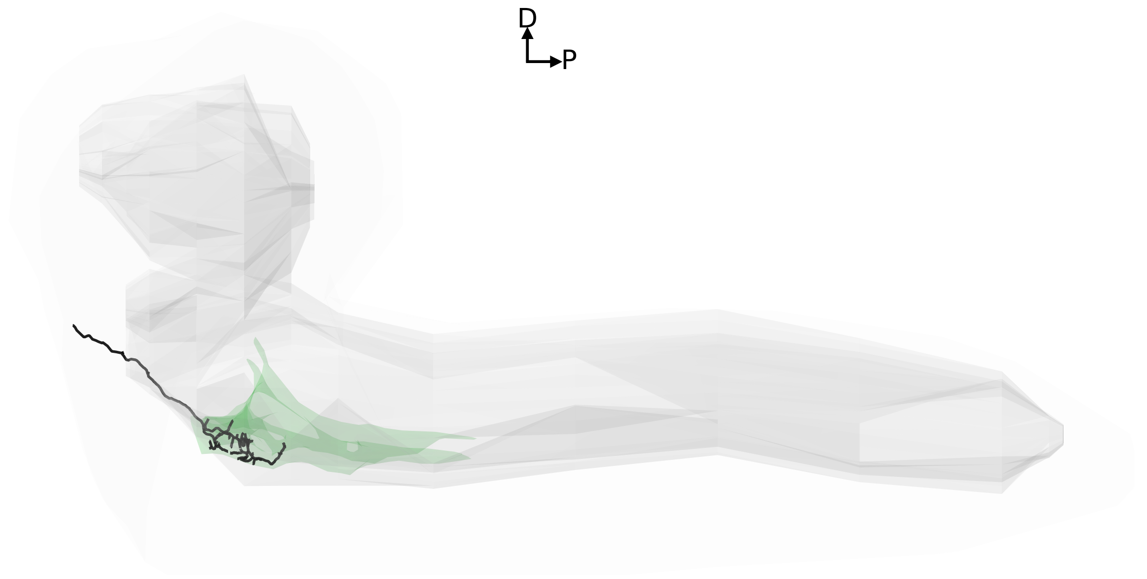

ID	*name*	SCACa	SCAVa	SCAVp	SCACal	SCACp	SCACpl	SCVM	IPCs	DMS	DH44	Se0ens	Se0ph	PMN LR	MN motor neurons	PaN motor neurons	olfactory PNs	gustatory PNs	multiglomerular PNs	unknown PNs	thermo PNs	visual PNs
15998319	AN-L-Sens-B2-VM-05	0	0	0	0	0	0	0	0	0	0	0	0	0	4	0	0	0	0	0	0	0

ID: 15996271
name: AN-L-Sens-B2-VM-06

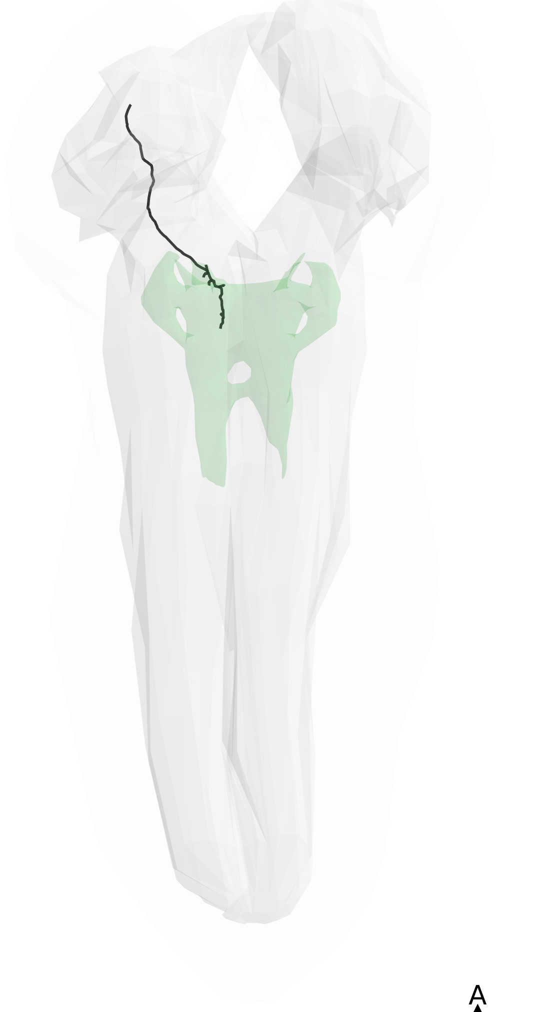

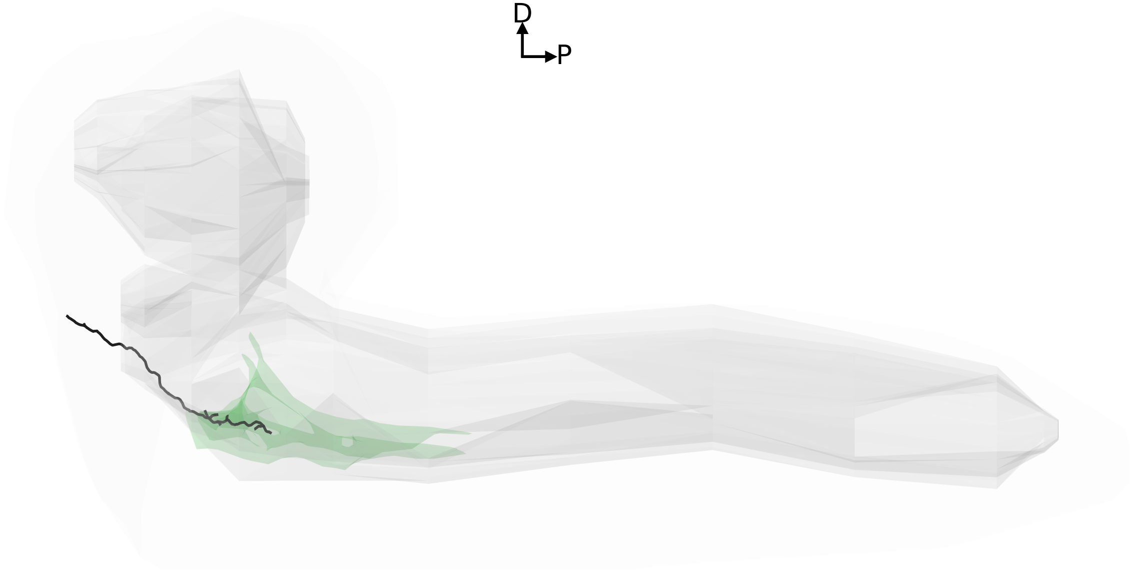

ID	name	SCACa	SCAVa	SCAVp	SCACal	SCACp	SCACpl	SCVM	IPCs	DMS	DH44	Se0ens	Se0ph	PMN LR	MN motor neurons	PaN motor neurons	olfactory PNs	gustatory PNs	multiglomerular PNs	unknown PNs	thermo PNs	visual PNs
15996271	AN-L-Sens-B2-VM-06	0	0	0	0	0	0	4	0	0	0	0	0	0	0	0	0	0	0	0	0	0

ID: 17608754
name: AN-L-Sens-B2-VM-07

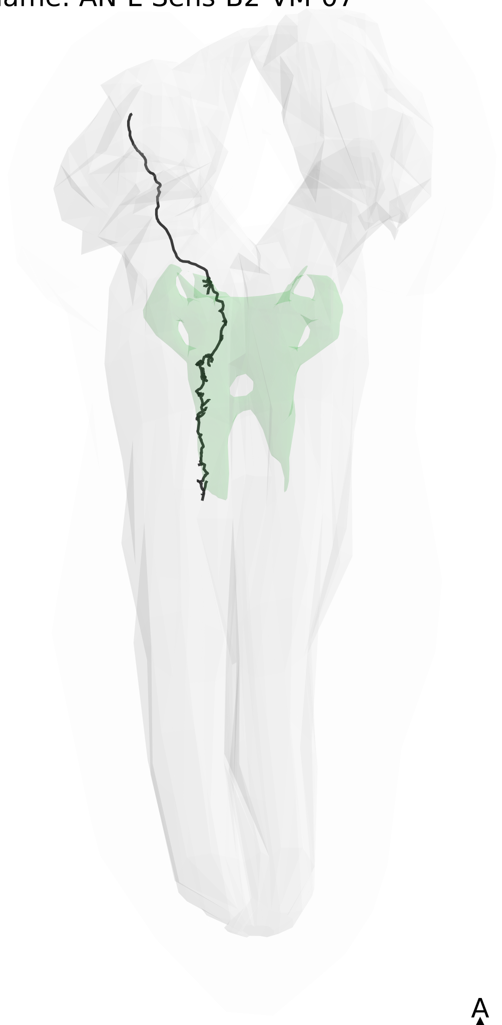

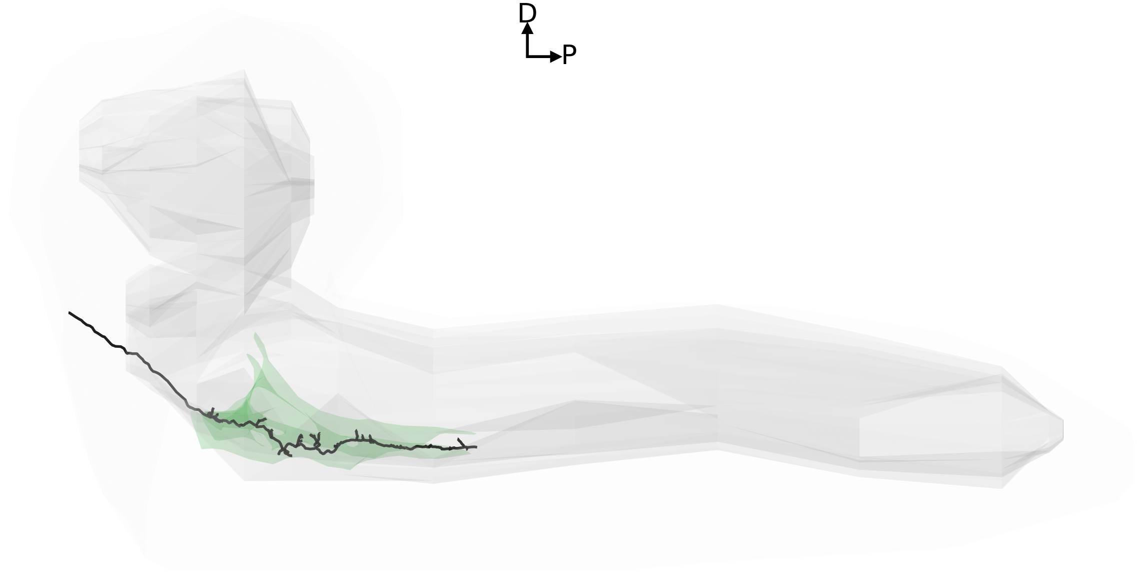

ID	name	SCACa	SCAVa	SCAVp	SCACal	SCACp	SCACpl	SCVM	IPCs	DMS	DH44	Se0ens	Se0ph	PMN LR	MN motor neurons	PaN motor neurons	olfactory PNs	gustatory PNs	multiglomerular PNs	unknown PNs	thermo PNs	visual PNs
17608754	AN-L-Sens-B2-VM-07	0	0	1	0	0	0	4	0	0	0	0	0	0	0	0	0	0	0	0	0	0

ID: 15995861
name: AN-L-Sens-B2-VM-08

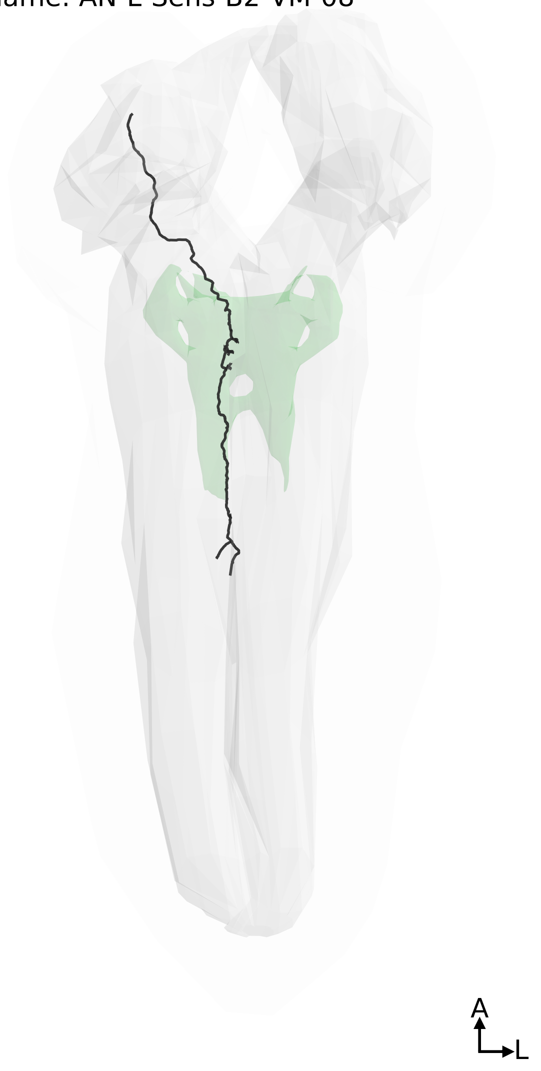

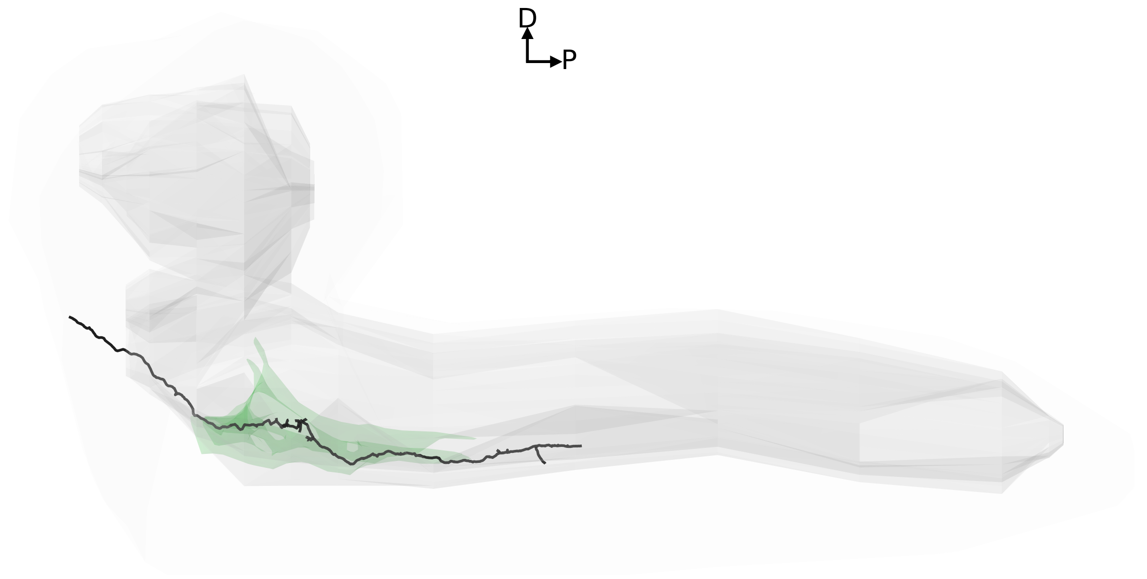

ID	*name*	SCACa	SCAVa	SCAVp	SCACal	SCACp	SCACpl	SCVM	IPCs	DMS	DH44	Se0ens	Se0ph	PMN LR	MN motor neurons	PaN motor neurons	olfactory PNs	gustatory PNs	multiglomerular PNs	unknown PNs	thermo PNs	visual PNs
15995861	AN-L-Sens-B2-VM-08	0	0	0	0	0	0	3	0	0	0	0	0	0	0	0	0	0	0	0	0	0

ID: 5057916
name: AN-L-Sens-B3-VM-11

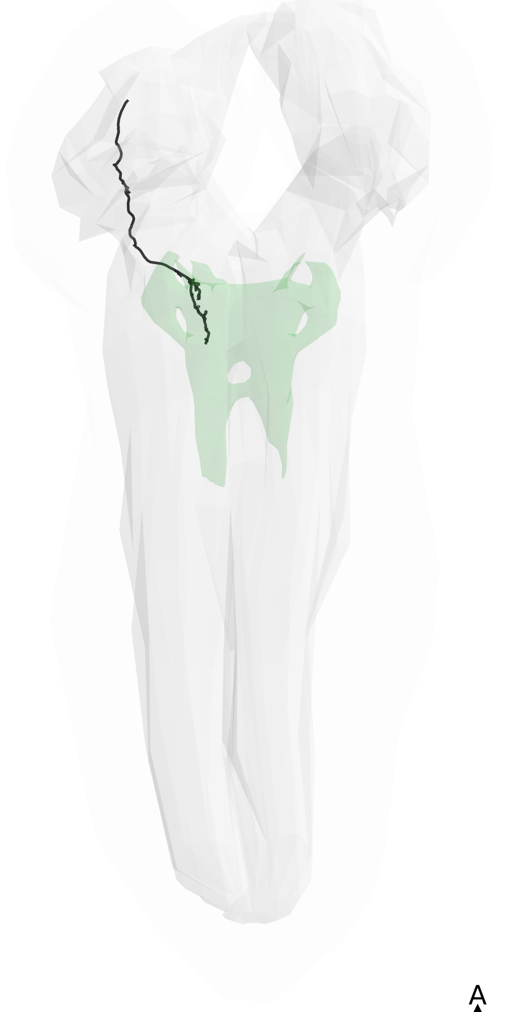

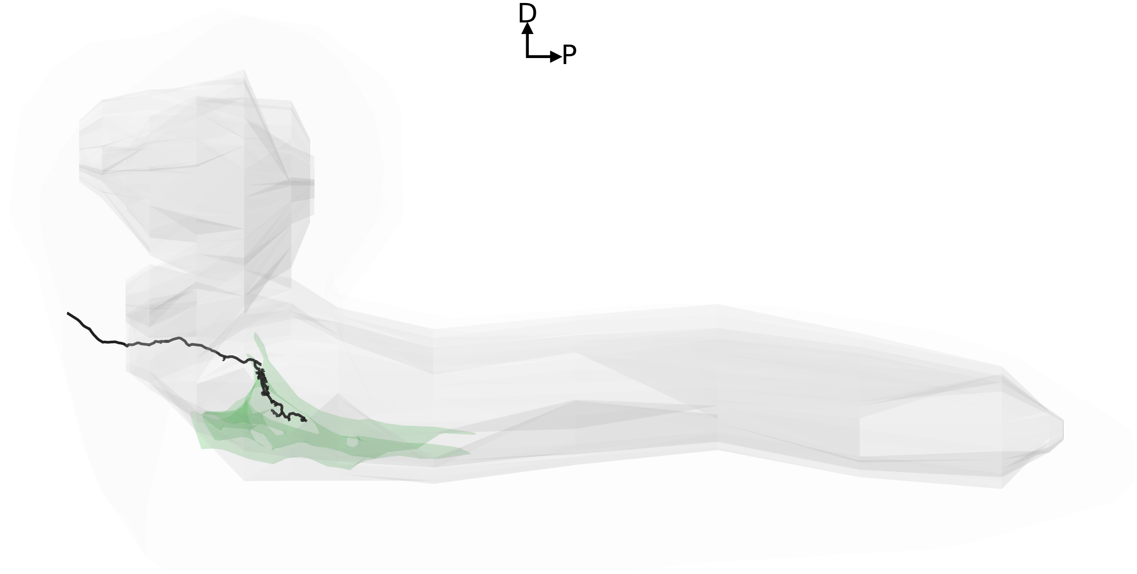

ID	*name*	SCACa	SCAVa	SCAVp	SCACal	SCACp	SCACpl	SCVM	IPCs	DMS	DH44	Se0ens	Se0ph	PMN LR	MN motor neurons	PaN motor neurons	olfactory PNs	gustatory PNs	multiglomerular PNs	unknown PNs	thermo PNs	visual PNs
5057916	AN-L-Sens-B3-VM-11	0	0	0	0	0	0	1	0	0	0	0	0	0	0	0	0	0	0	0	0	0

ID: 5120097
name: AN-L-Sens-B3-VM-12

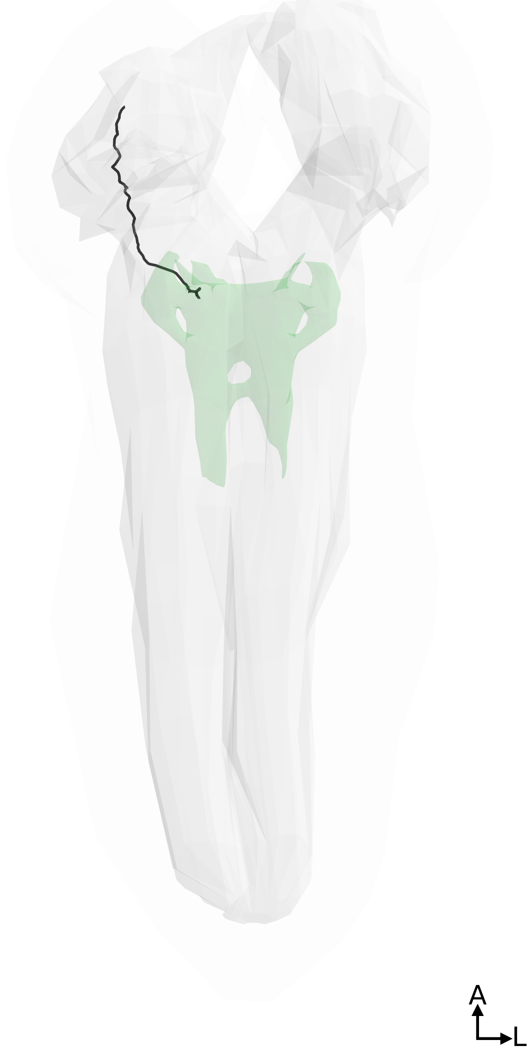

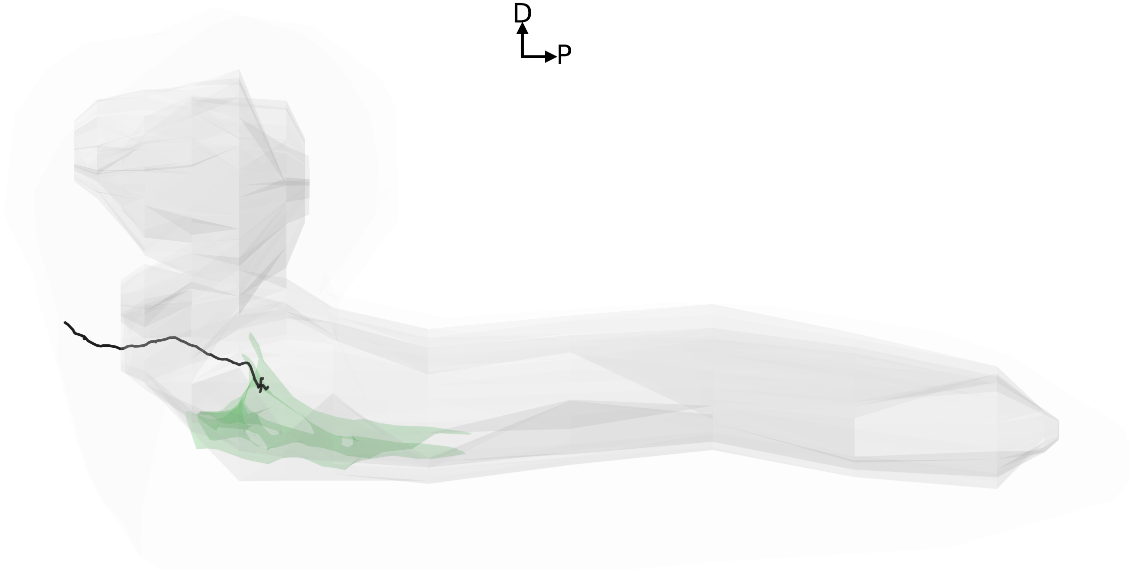

ID	name	SCACa	SCAVa	SCAVp	SCACal	SCACp	SCACpl	SCVM	IPCs	DMS	DH44	Se0ens	Se0ph	PMN LR	MN motor neurons	PaN motor neurons	olfactory PNs	gustatory PNs	multiglomerular PNs	unknown PNs	thermo PNs	visual PNs
5120097	AN-L-Sens-B3-VM-12	0	0	0	0	0	0	1	0	0	0	0	0	0	0	0	0	0	0	0	0	0

ID: 5120990
name: AN-L-Sens-B3-VM-13

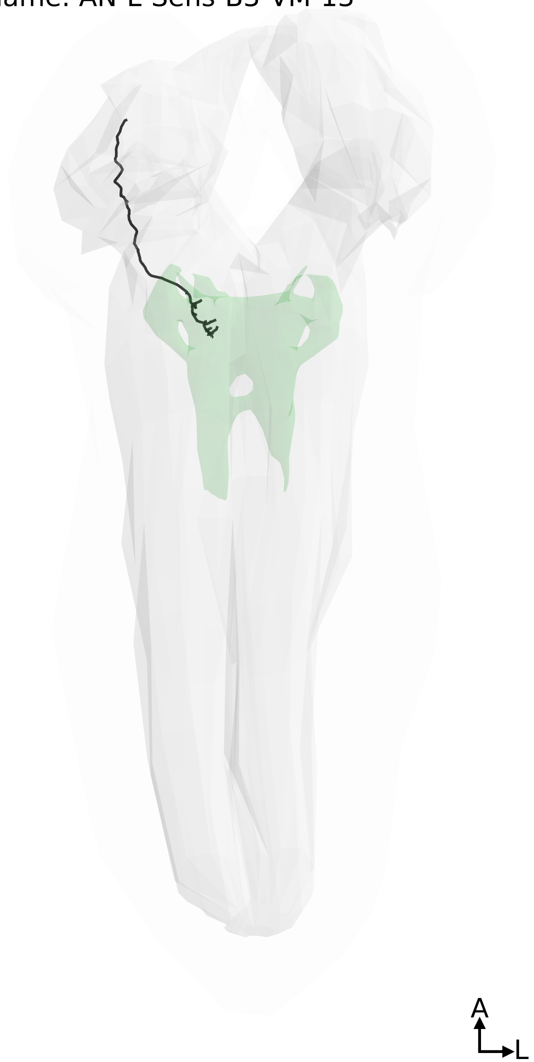

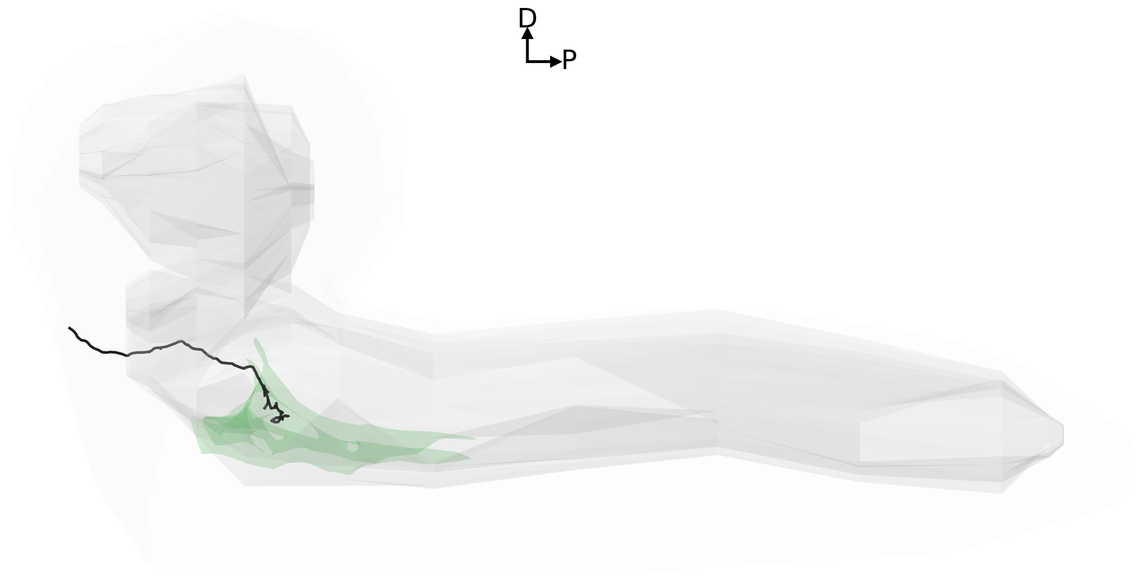

ID	*name*	SCACa	SCAVa	SCAVp	SCACal	SCACp	SCACpl	SCVM	IPCs	DMS	DH44	Se0ens	Se0ph	PMN LR	MN motor neurons	PaN motor neurons	olfactory PNs	gustatory PNs	multiglomerular PNs	unknown PNs	thermo PNs	visual PNs
5120990	AN-L-Sens-B3-VM-13	0	0	0	0	0	0	0	0	0	0	0	0	0	0	0	0	0	0	0	0	0

ID: 5095269
name: AN-L-Sens-B3-VM-14

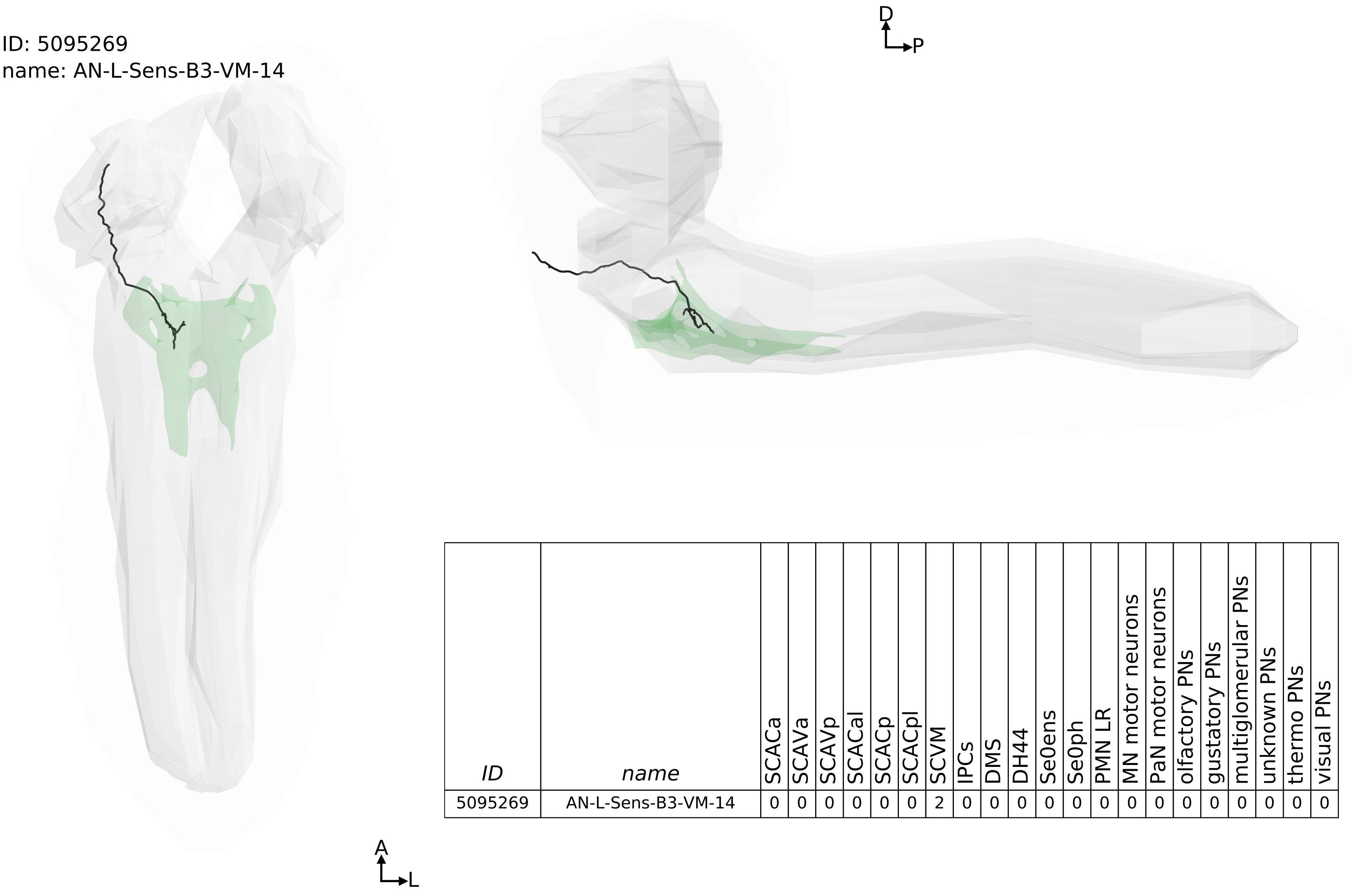

ID	name	SCACa	SCAVa	SCAVp	SCACal	SCACp	SCACpl	SCVM	IPCs	DMS	DH44	Se0ens	Se0ph	PMN LR	MN motor neurons	PaN motor neurons	olfactory PNs	gustatory PNs	multiglomerular PNs	unknown PNs	thermo PNs	visual PNs
5095269	AN-L-Sens-B3-VM-14	0	0	0	0	0	0	2	0	0	0	0	0	0	0	0	0	0	0	0	0	0

ID: 5116861
name: AN-L-Sens-B3-VM-15

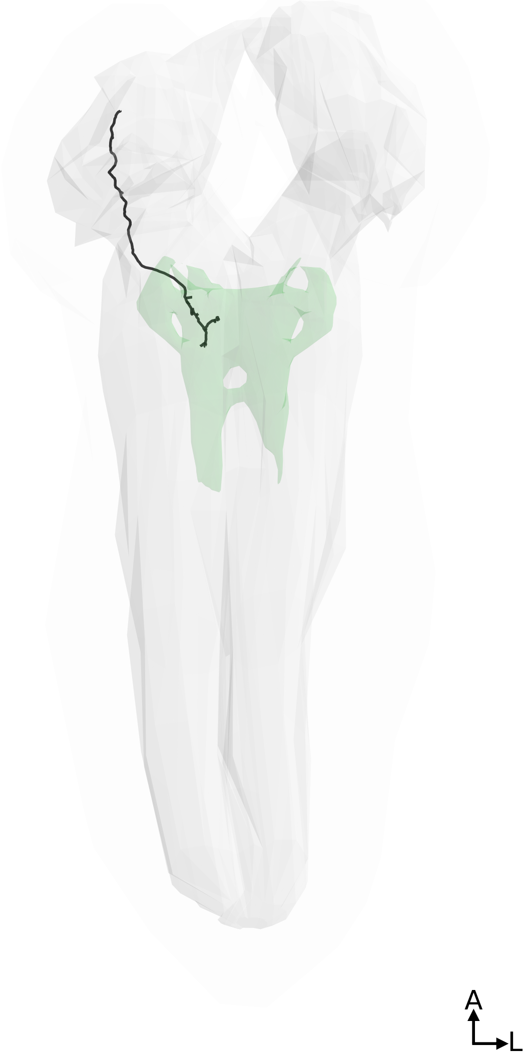

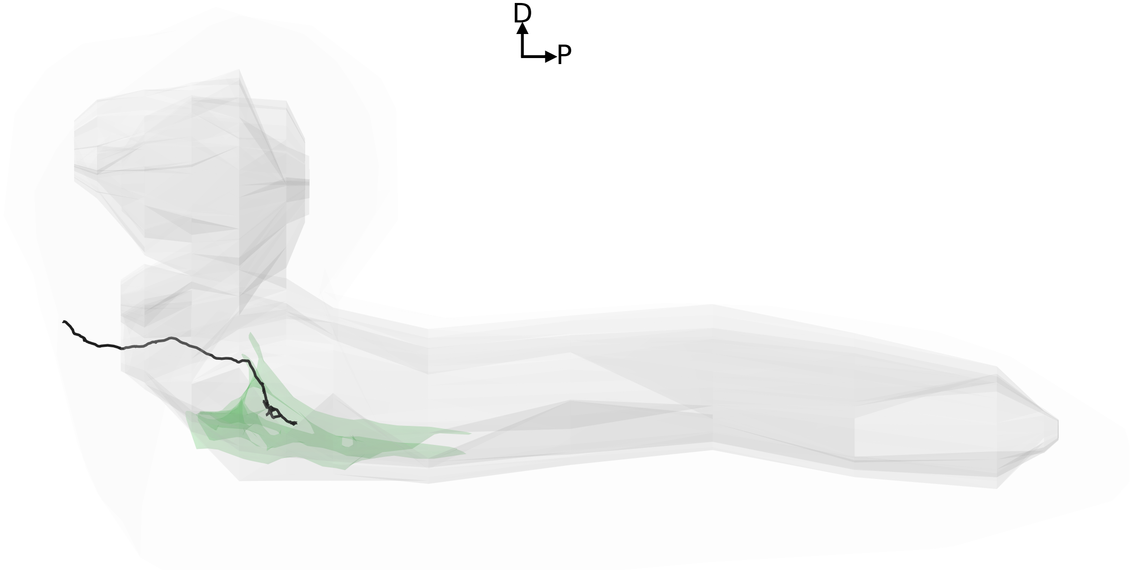

ID	*name*	SCACa	SCAVa	SCAVp	SCACal	SCACp	SCACpl	SCVM	IPCs	DMS	DH44	Se0ens	Se0ph	PMN LR	MN motor neurons	PaN motor neurons	olfactory PNs	gustatory PNs	multiglomerular PNs	unknown PNs	thermo PNs	visual PNs
5116861	AN-L-Sens-B3-VM-15	0	0	0	0	0	0	4	0	0	0	0	0	0	0	0	0	0	0	0	0	0

ID: 15508475
name: AN-R-Sens-B2-VM-04

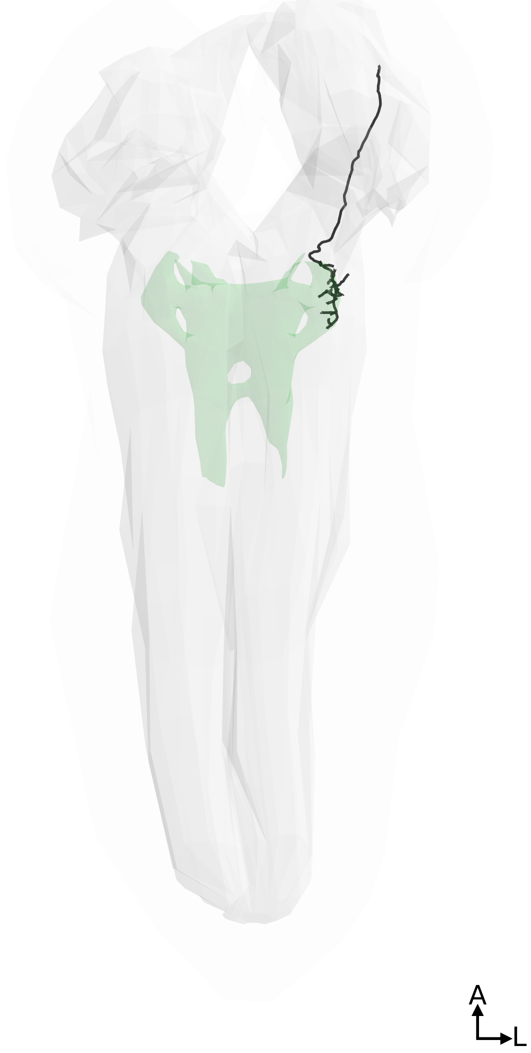

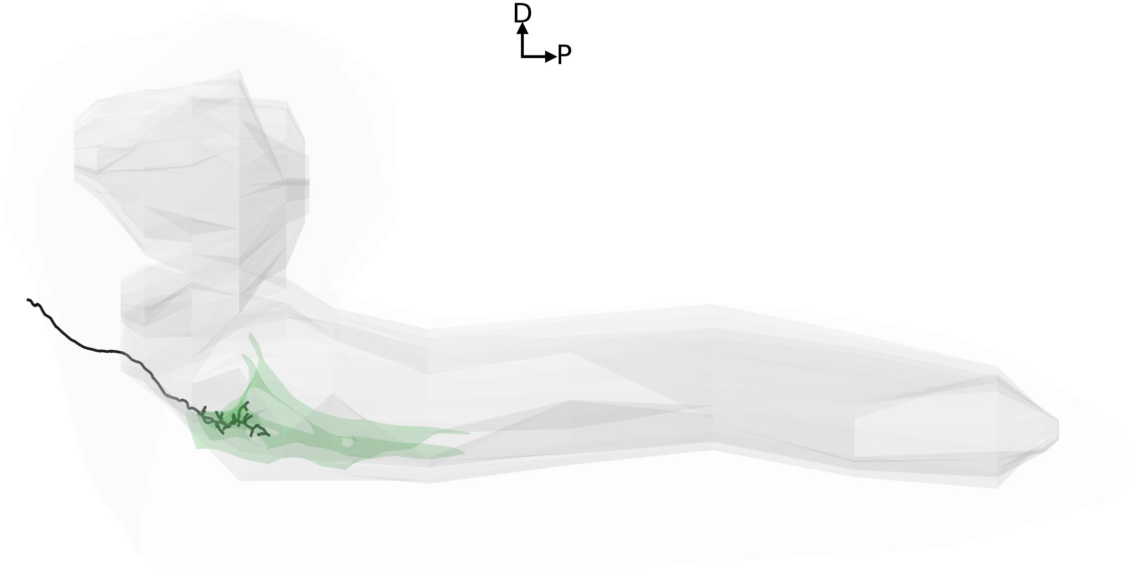

ID	name	SCACa	SCAVa	SCAVp	SCACal	SCACp	SCACpl	SCVM	IPCs	DMS	DH44	Se0ens	Se0ph	PMN LR	MN motor neurons	PaN motor neurons	olfactory PNs	gustatory PNs	multiglomerular PNs	unknown PNs	thermo PNs	visual PNs
15508475	AN-R-Sens-B2-VM-04	0	0	0	0	0	0	1	0	0	0	0	0	0	0	0	0	0	0	0	0	0

ID: 15594038
name: AN-R-Sens-B2-VM-05

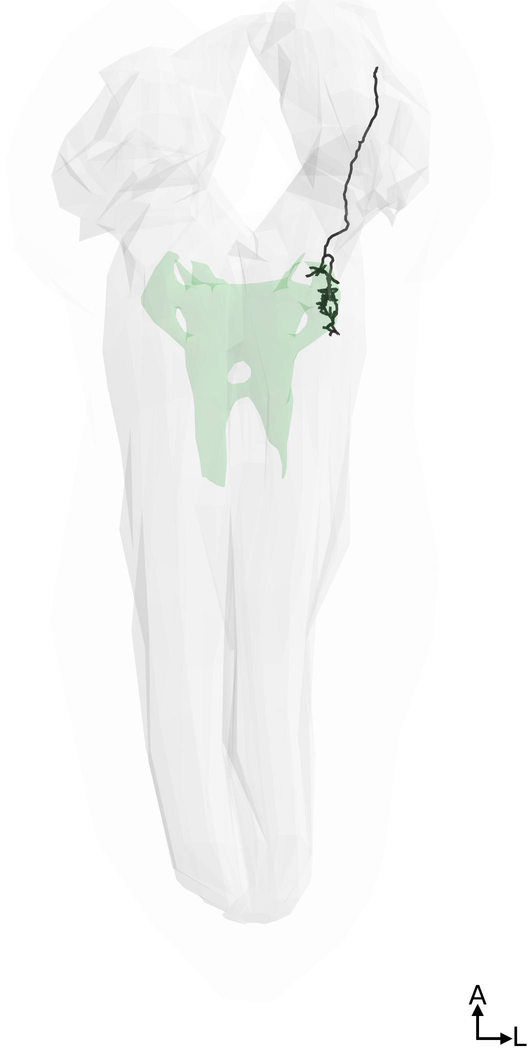

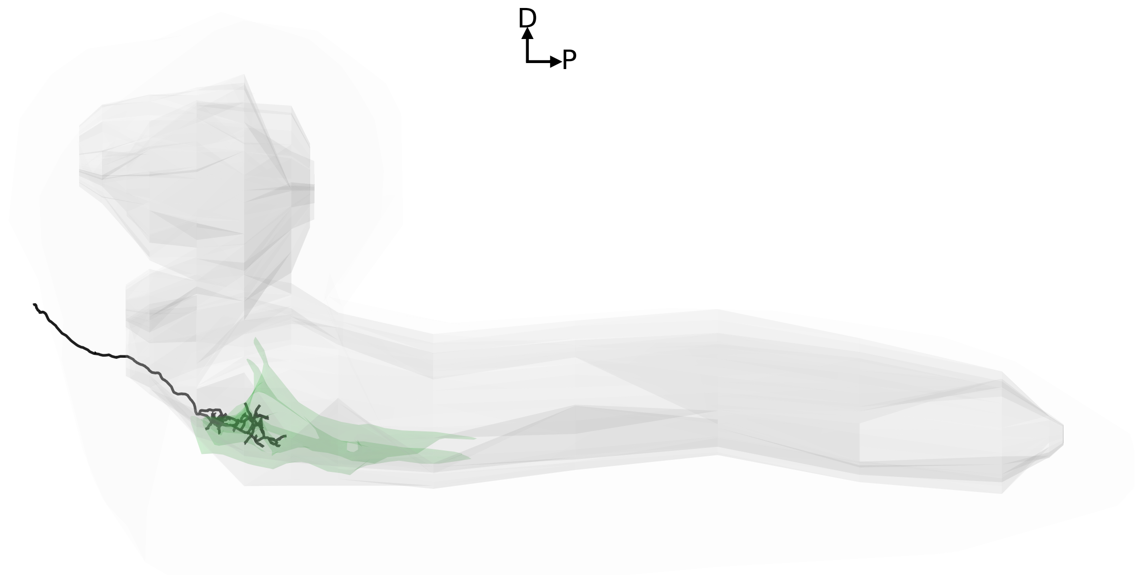

ID	*name*	SCACa	SCAVa	SCAVp	SCACal	SCACp	SCACpl	SCVM	IPCs	DMS	DH44	Se0ens	Se0ph	PMN LR	MN motor neurons	PaN motor neurons	olfactory PNs	gustatory PNs	multiglomerular PNs	unknown PNs	thermo PNs	visual PNs
15594038	AN-R-Sens-B2-VM-05	0	0	0	0	0	0	2	0	0	0	0	0	0	2	0	0	0	0	0	0	0

ID: 16362243
name: AN-R-Sens-B2-VM-07

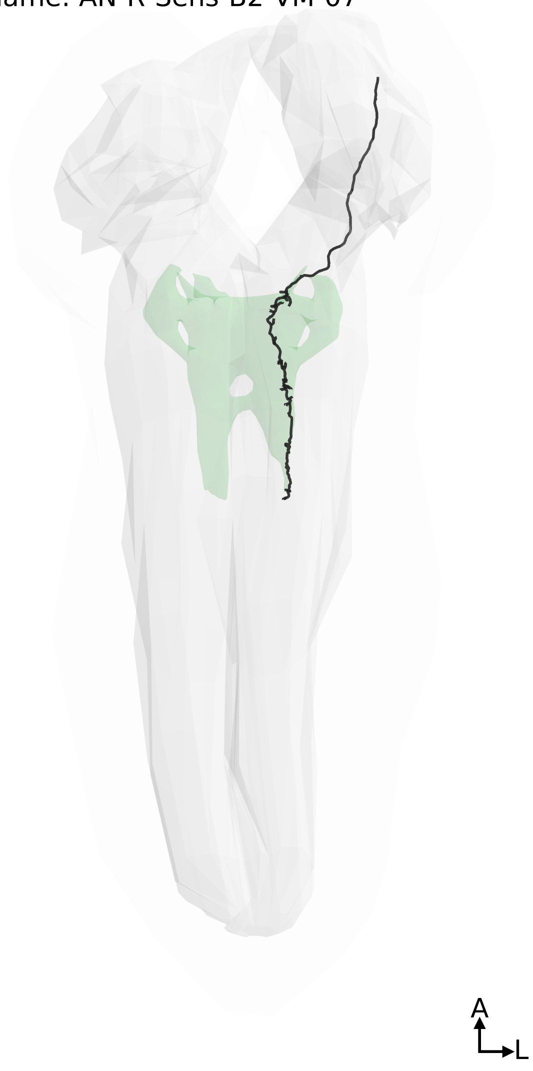

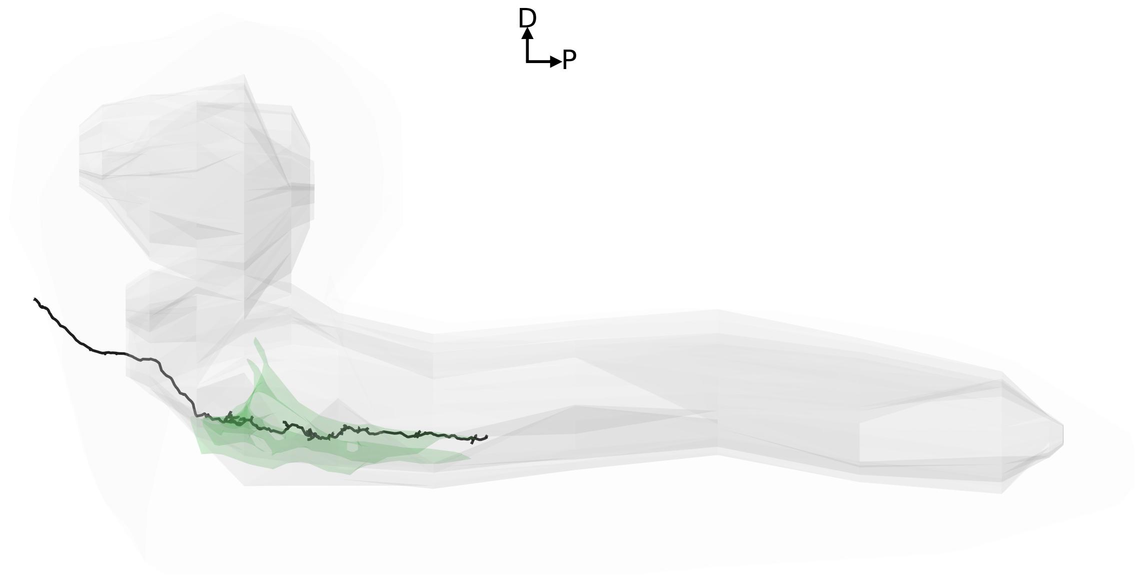

ID	*name*	SCACa	SCAVa	SCAVp	SCACal	SCACp	SCACpl	SCVM	IPCs	DMS	DH44	Se0ens	Se0ph	PMN LR	MN motor neurons	PaN motor neurons	olfactory PNs	gustatory PNs	multiglomerular PNs	unknown PNs	thermo PNs	visual PNs
16362243	AN-R-Sens-B2-VM-07	0	0	0	0	0	0	1	0	0	0	0	0	0	0	0	0	0	0	0	0	0

ID: 15445198
name: AN-R-Sens-B2-VM-08

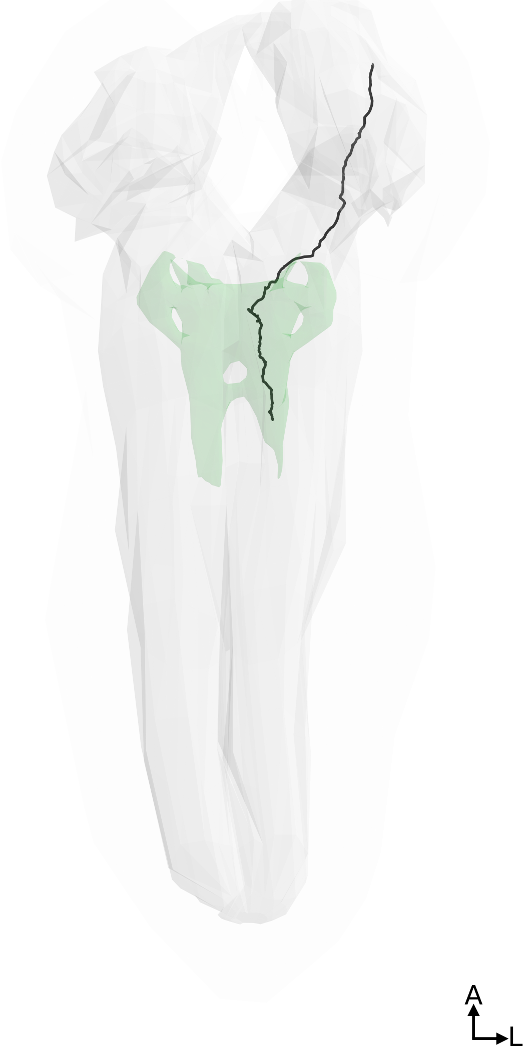

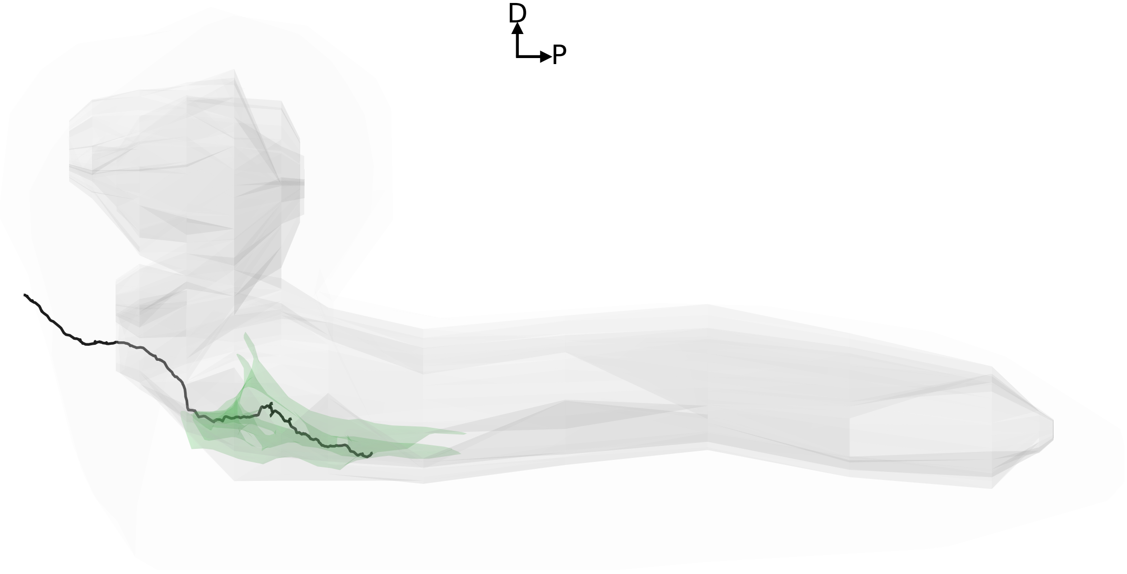

ID	*name*	SCACa	SCAVa	SCAVp	SCACal	SCACp	SCACpl	SCVM	IPCs	DMS	DH44	Se0ens	Se0ph	PMN LR	MN motor neurons	PaN motor neurons	olfactory PNs	gustatory PNs	multiglomerular PNs	unknown PNs	thermo PNs	visual PNs
15445198	AN-R-Sens-B2-VM-08	0	0	0	0	0	0	3	0	0	0	0	0	0	0	0	0	0	0	0	0	0

ID: 16115501
name: AN-R-Sens-B3-VM-11

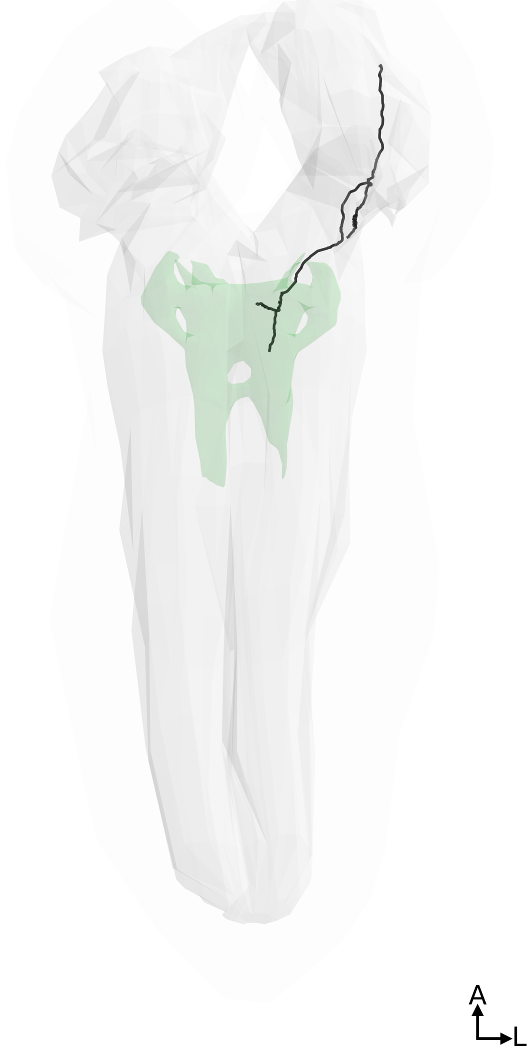

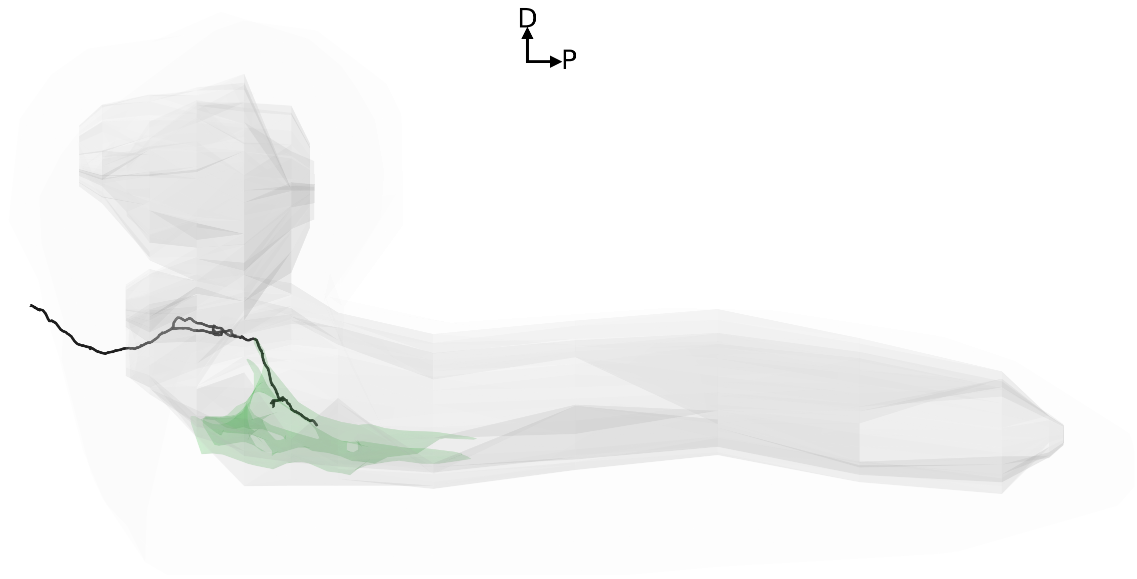

ID	*name*	SCACa	SCAVa	SCAVp	SCACal	SCACp	SCACpl	SCVM	IPCs	DMS	DH44	Se0ens	Se0ph	PMN LR	MN motor neurons	PaN motor neurons	olfactory PNs	gustatory PNs	multiglomerular PNs	unknown PNs	thermo PNs	visual PNs
16115501	AN-R-Sens-B3-VM-11	0	0	0	0	0	0	3	0	0	0	0	0	0	0	0	0	0	1	0	0	0

ID: 4096703
name: AN-R-Sens-B3-VM-12

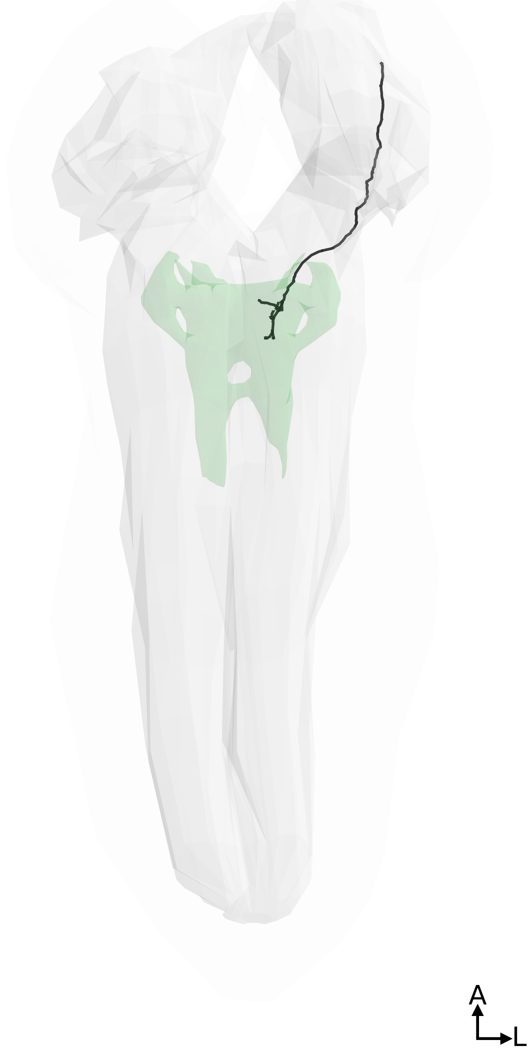

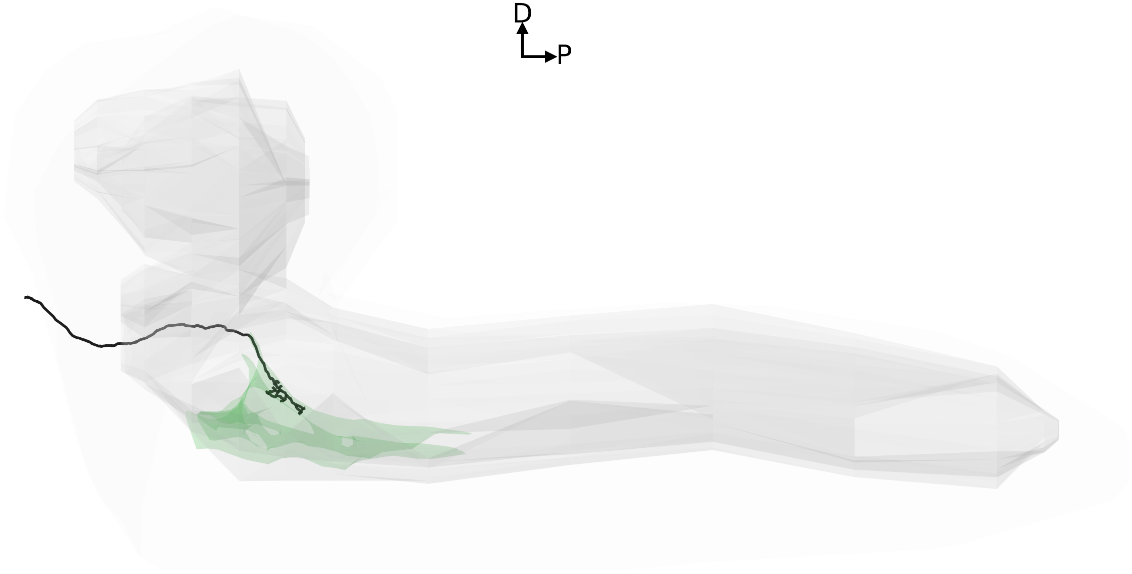

ID	*name*	SCACa	SCAVa	SCAVp	SCACal	SCACp	SCACpl	SCVM	IPCs	DMS	DH44	Se0ens	Se0ph	PMN LR	MN motor neurons	PaN motor neurons	olfactory PNs	gustatory PNs	multiglomerular PNs	unknown PNs	thermo PNs	visual PNs
4096703	AN-R-Sens-B3-VM-12	0	0	0	0	0	0	6	0	0	0	0	0	0	0	0	0	0	0	0	0	0

ID: 4044654
name: AN-R-Sens-B3-VM-13

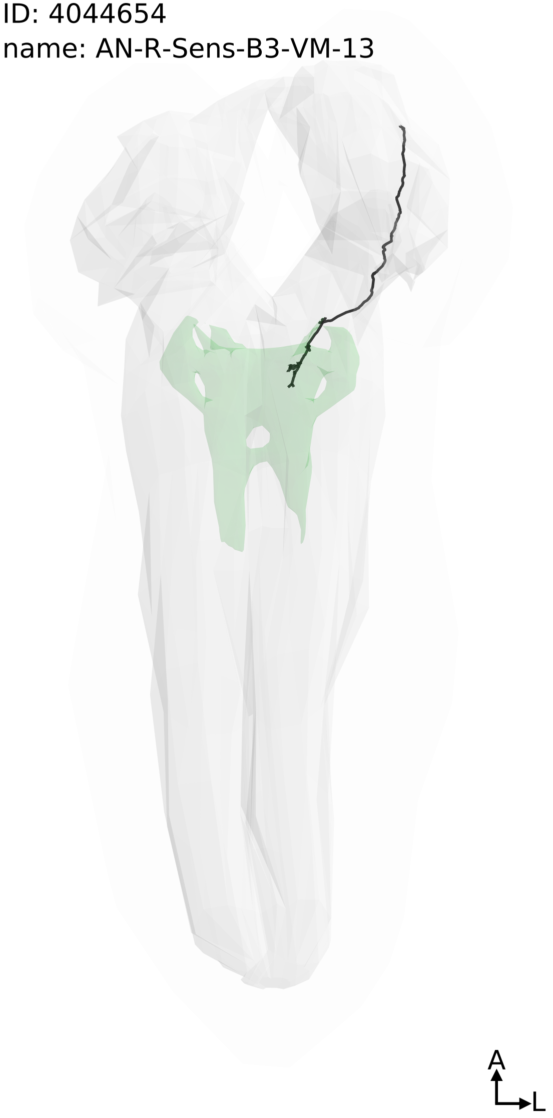

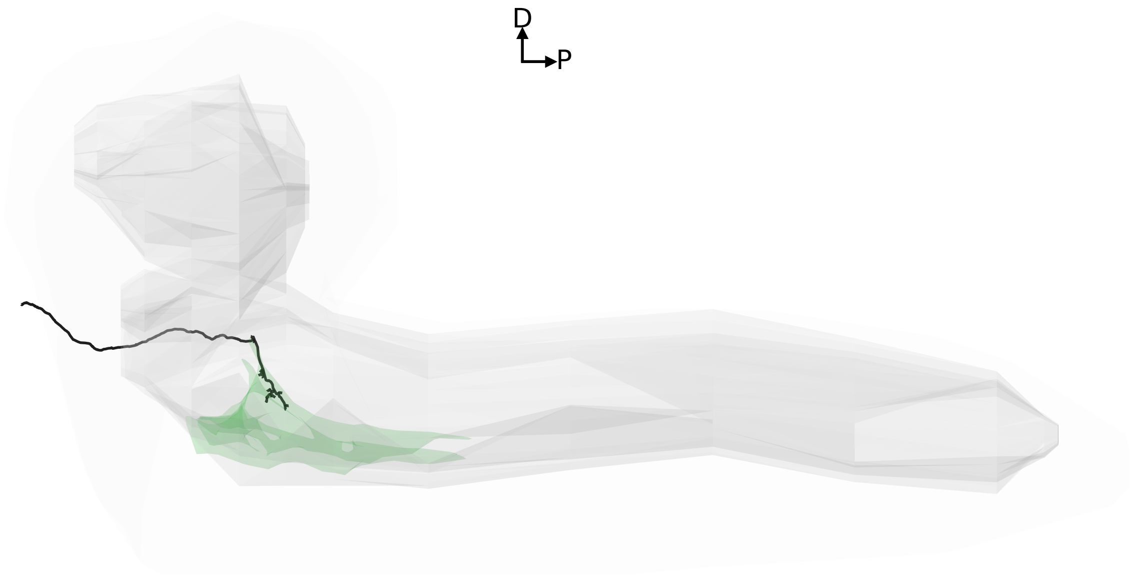

ID	*name*	SCACa	SCAVa	SCAVp	SCACal	SCACp	SCACpl	SCVM	IPCs	DMS	DH44	Se0ens	Se0ph	PMN LR	MN motor neurons	PaN motor neurons	olfactory PNs	gustatory PNs	multiglomerular PNs	unknown PNs	thermo PNs	visual PNs
4044654	AN-R-Sens-B3-VM-13	0	0	0	0	0	0	0	0	0	0	0	0	0	0	0	0	0	0	0	0	0

ID: 4030526
name: AN-R-Sens-B3-VM-14

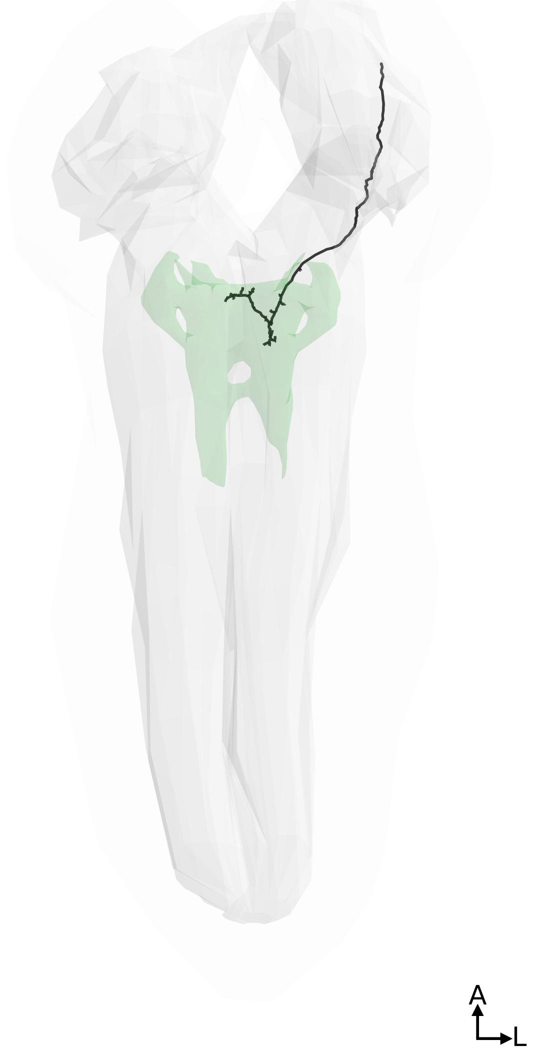

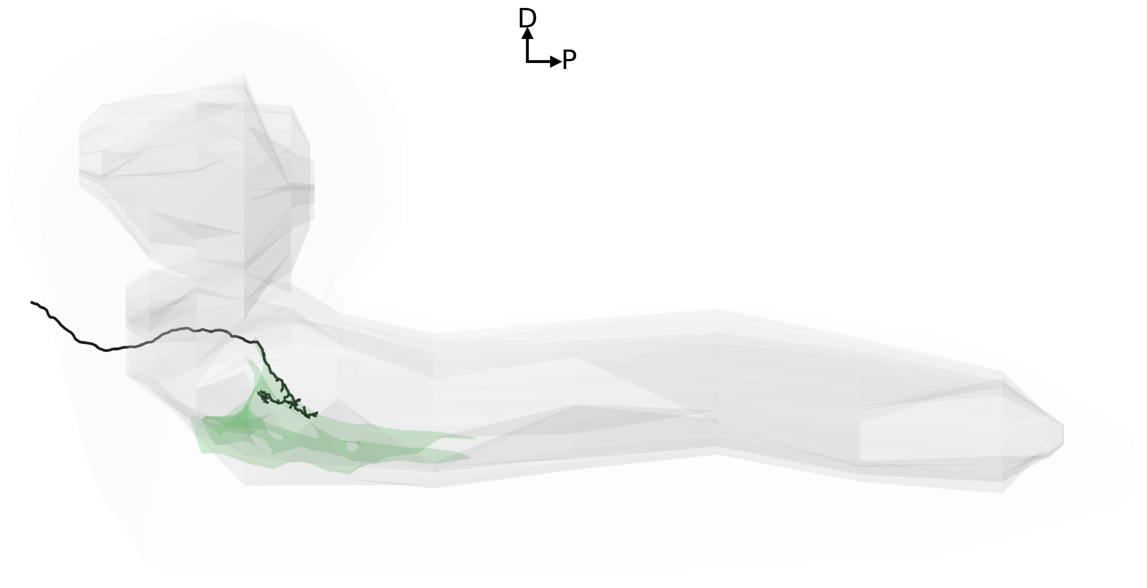

ID	name	SCACa	SCAVa	SCAVp	SCACal	SCACp	SCACpl	SCVM	IPCs	DMS	DH44	Se0ens	Se0ph	PMN LR	MN motor neurons	PaN motor neurons	olfactory PNs	gustatory PNs	multiglomerular PNs	unknown PNs	thermo PNs	visual PNs
4030526	AN-R-Sens-B3-VM-14	0	0	0	0	0	0	6	0	0	0	0	0	0	0	0	0	0	0	0	0	0

ID: 4059758
name: AN-R-Sens-B3-VM-15

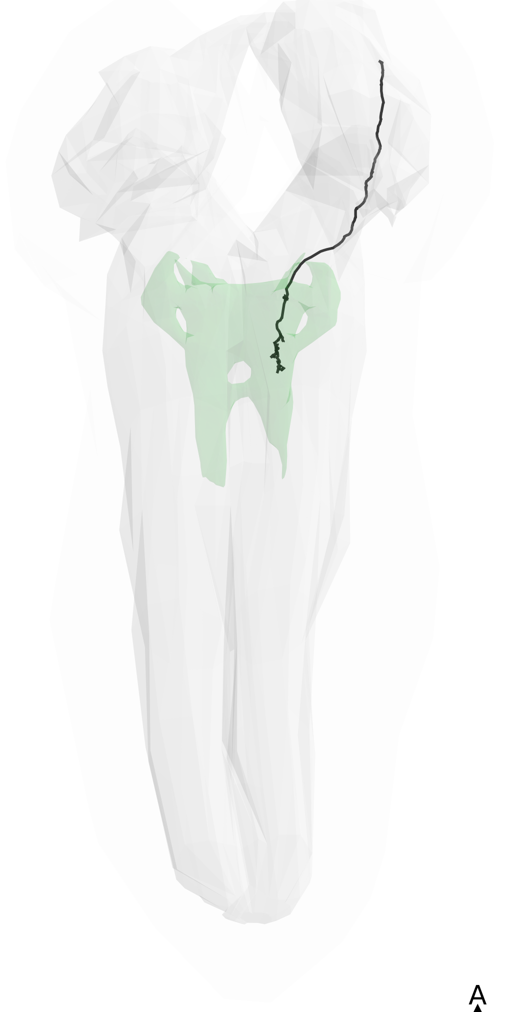

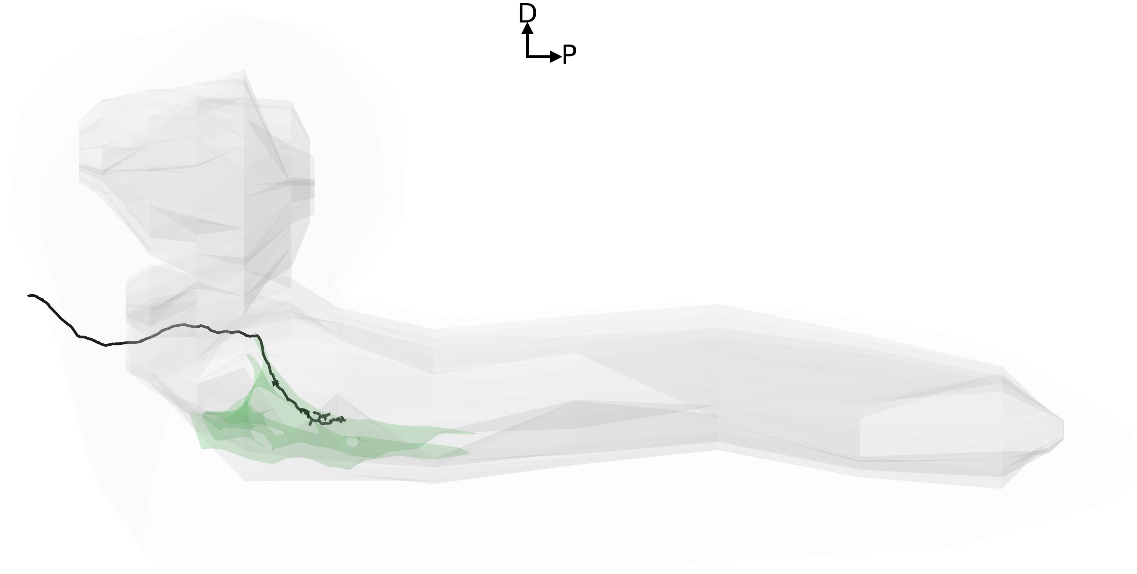

ID	*name*	SCACa	SCAVa	SCAVp	SCACal	SCACp	SCACpl	SCVM	IPCs	DMS	DH44	Se0ens	Se0ph	PMN LR	MN motor neurons	PaN motor neurons	olfactory PNs	gustatory PNs	multiglomerular PNs	unknown PNs	thermo PNs	visual PNs
4059758	AN-R-Sens-B3-VM-15	0	0	0	0	0	0	0	0	0	0	0	0	0	0	0	0	0	0	0	0	0

ID: 15698623
name: MN-L-Sens-B1-VM-02

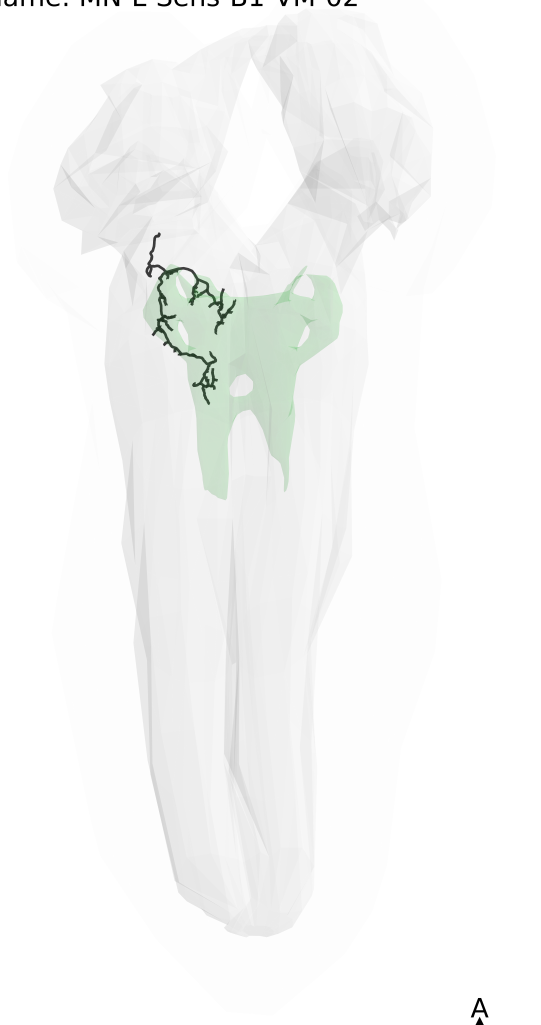

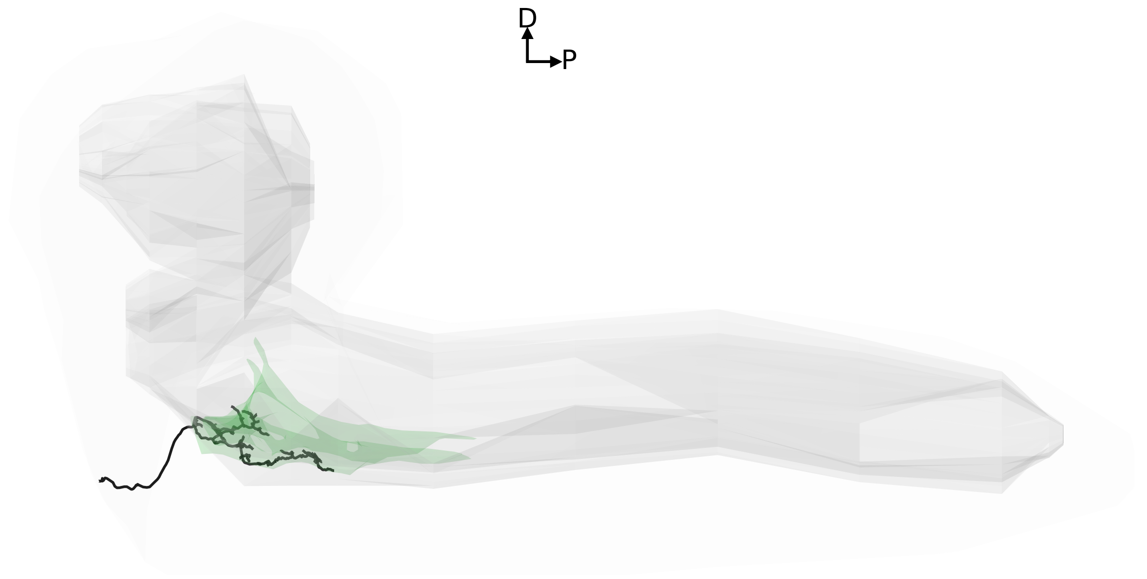

ID	name	SCACa	SCAVa	SCAVp	SCACal	SCACp	SCACpl	SCVM	IPCs	DMS	DH44	Se0ens	Se0ph	PMN LR	MN motor neurons	PaN motor neurons	olfactory PNs	gustatory PNs	multiglomerular PNs	unknown PNs	thermo PNs	visual PNs
15698623	MN-L-Sens-B1-VM-02	0	0	2	0	1	0	8	0	0	0	0	0	0	0	0	0	0	0	0	0	0

ID: 15714330
name: MN-L-Sens-B1-VM-03

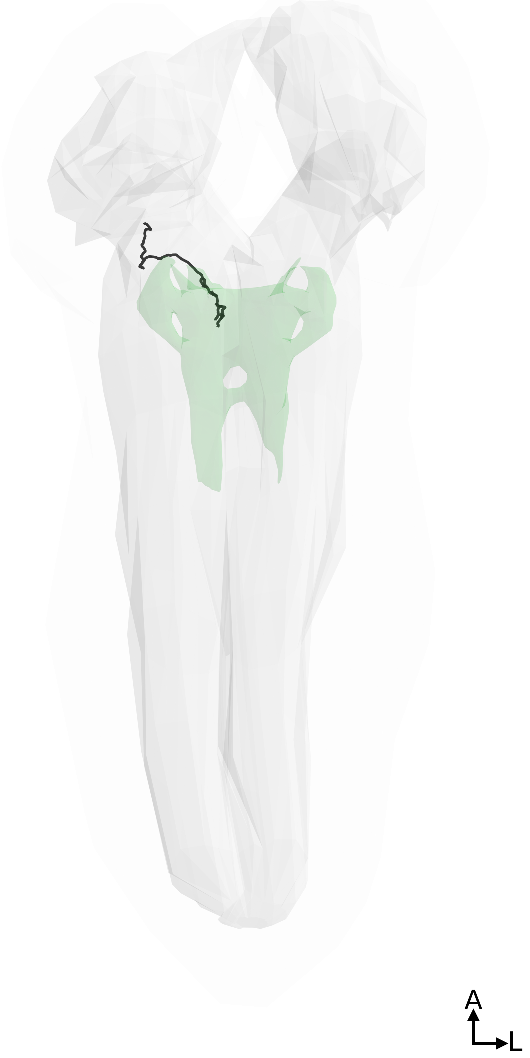

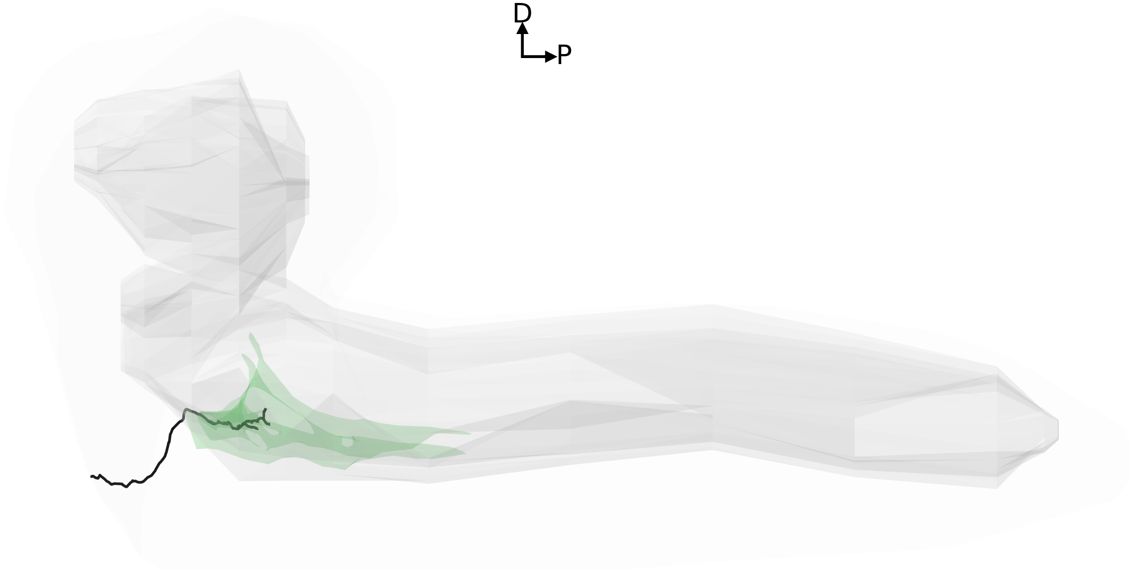

ID	*name*	SCACa	SCAVa	SCAVp	SCACal	SCACp	SCACpl	SCVM	IPCs	DMS	DH44	Se0ens	Se0ph	PMN LR	MN motor neurons	PaN motor neurons	olfactory PNs	gustatory PNs	multiglomerular PNs	unknown PNs	thermo PNs	visual PNs
15714330	MN-L-Sens-B1-VM-03	0	0	0	0	0	0	11	0	0	0	0	0	0	0	0	0	0	0	0	0	0

ID: 15700147
name: MN-L-Sens-B1-VM-04

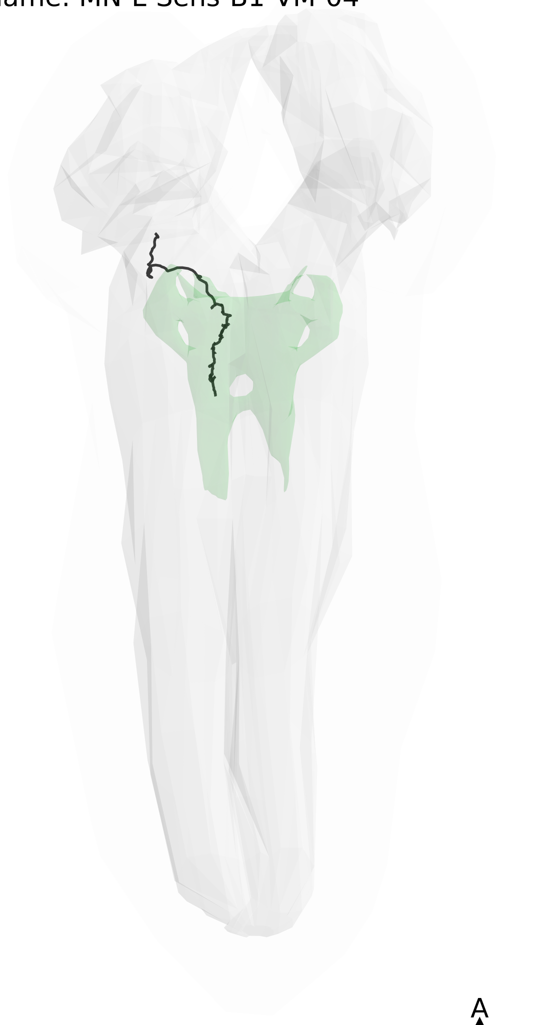

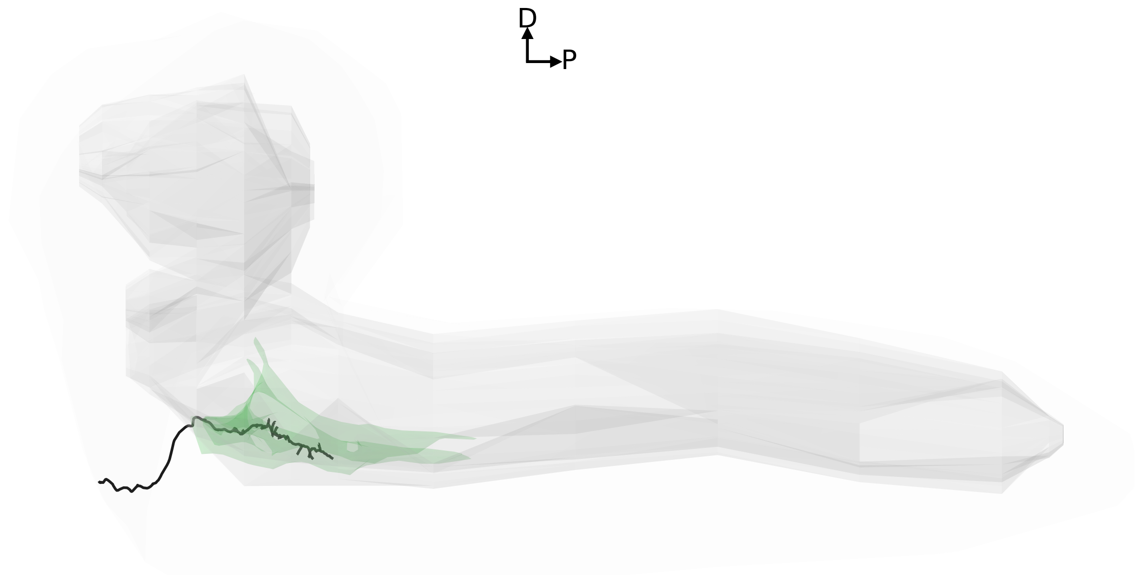

ID	*name*	SCACa	SCAVa	SCAVp	SCACal	SCACp	SCACpl	SCVM	IPCs	DMS	DH44	Se0ens	Se0ph	PMN LR	MN motor neurons	PaN motor neurons	olfactory PNs	gustatory PNs	multiglomerular PNs	unknown PNs	thermo PNs	visual PNs
15700147	MN-L-Sens-B1-VM-04	0	0	0	0	0	0	7	0	0	0	0	0	0	0	0	0	0	0	0	0	0

ID: 15714173
name: MN-L-Sens-B1-VM-05

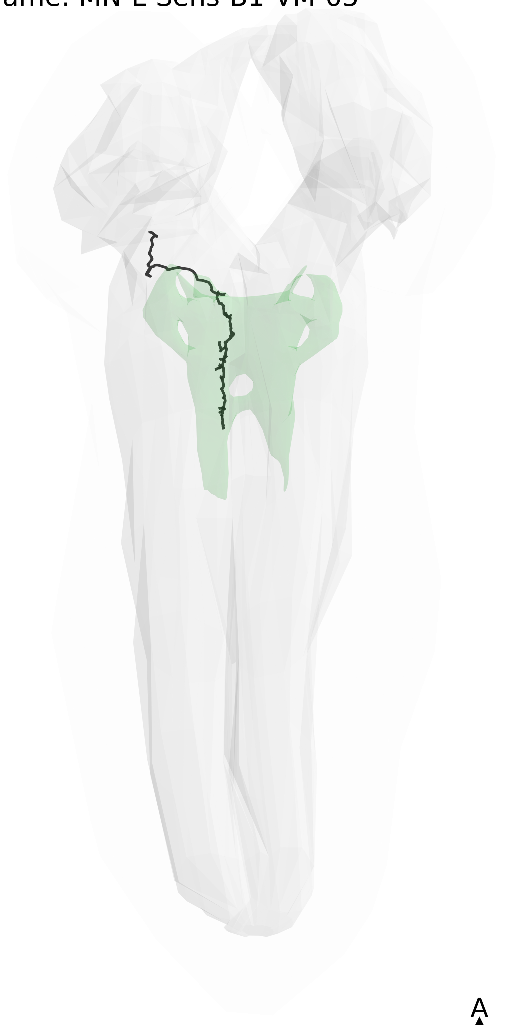

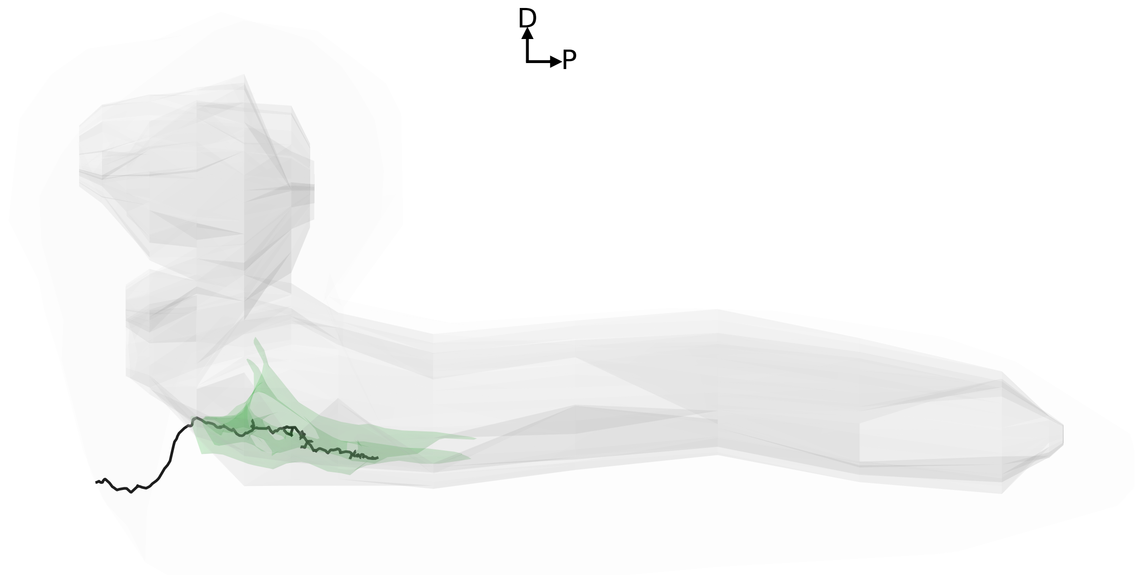

ID	*name*	SCACa	SCAVa	SCAVp	SCACal	SCACp	SCACpl	SCVM	IPCs	DMS	DH44	Se0ens	Se0ph	PMN LR	MN motor neurons	PaN motor neurons	olfactory PNs	gustatory PNs	multiglomerular PNs	unknown PNs	thermo PNs	visual PNs
15714173	MN-L-Sens-B1-VM-05	0	0	0	0	0	1	17	0	0	0	0	0	0	0	0	0	0	0	0	0	0

ID: 15715151
name: MN-L-Sens-B2-VM-01

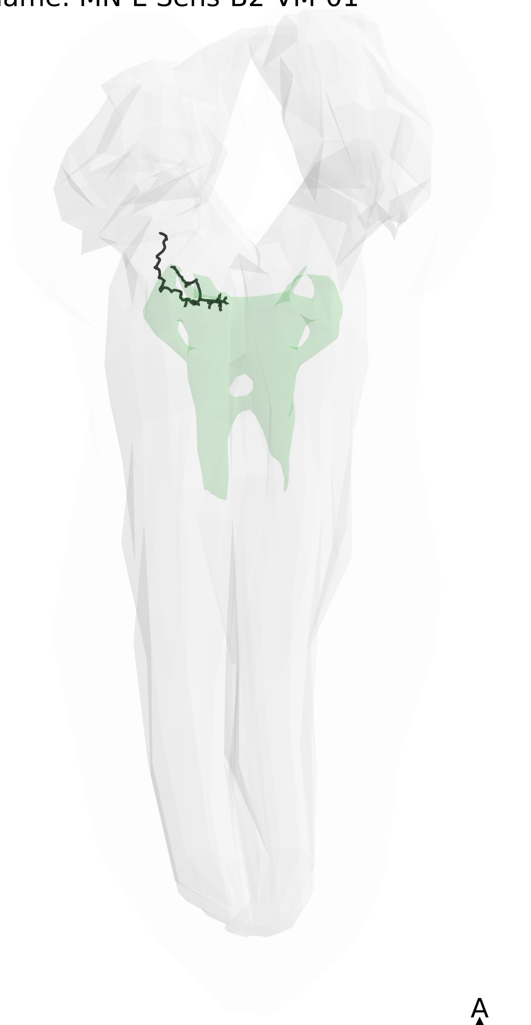

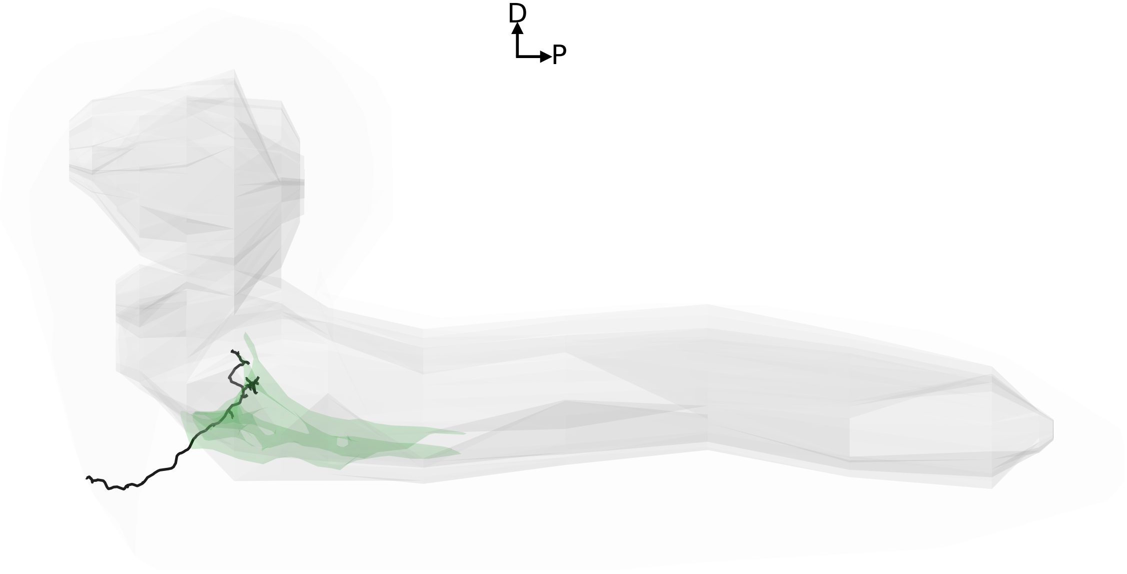

ID	*name*	SCACa	SCAVa	SCAVp	SCACal	SCACp	SCACpl	SCVM	IPCs	DMS	DH44	Se0ens	Se0ph	PMN LR	MN motor neurons	PaN motor neurons	olfactory PNs	gustatory PNs	multiglomerular PNs	unknown PNs	thermo PNs	visual PNs
15715151	MN-L-Sens-B2-VM-01	0	0	0	0	0	0	4	0	0	0	0	0	0	0	0	0	0	0	0	0	0

ID: 15674083
name: MN-L-Sens-B2-VM-02

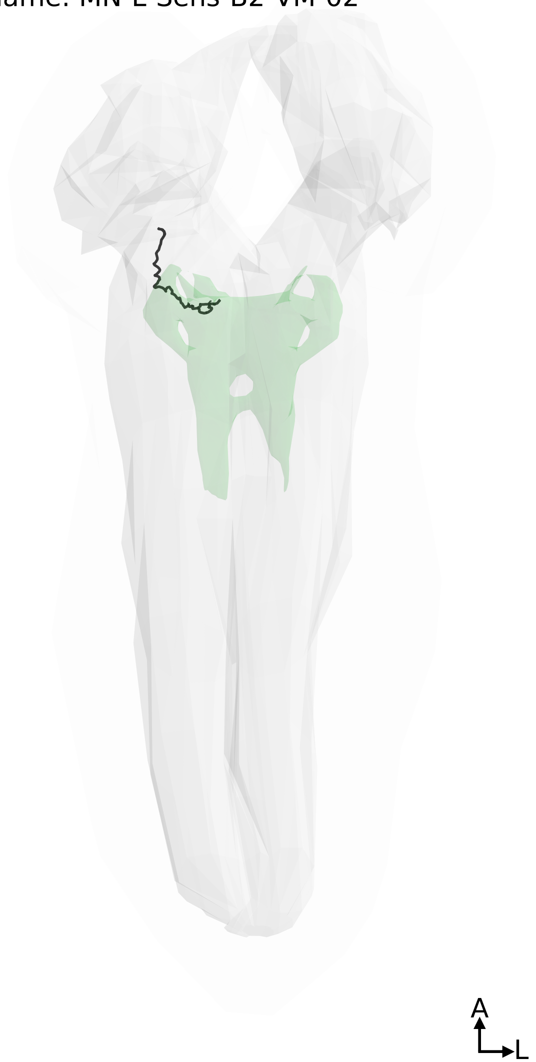

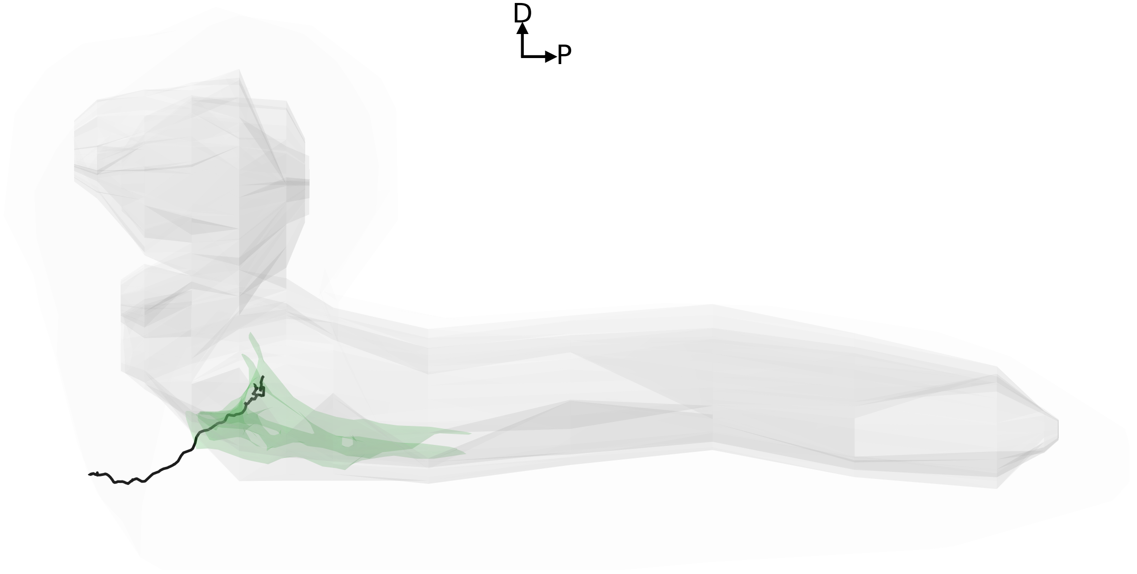

ID	name	SCACa	SCAVa	SCAVp	SCACal	SCACp	SCACpl	SCVM	IPCs	DMS	DH44	Se0ens	Se0ph	PMN LR	MN motor neurons	PaN motor neurons	olfactory PNs	gustatory PNs	multiglomerular PNs	unknown PNs	thermo PNs	visual PNs
15674083	MN-L-Sens-B2-VM-02	0	0	0	0	0	0	4	0	0	0	0	0	0	0	0	0	0	0	0	0	0

ID: 15680710
name: MN-L-Sens-B2-VM-03

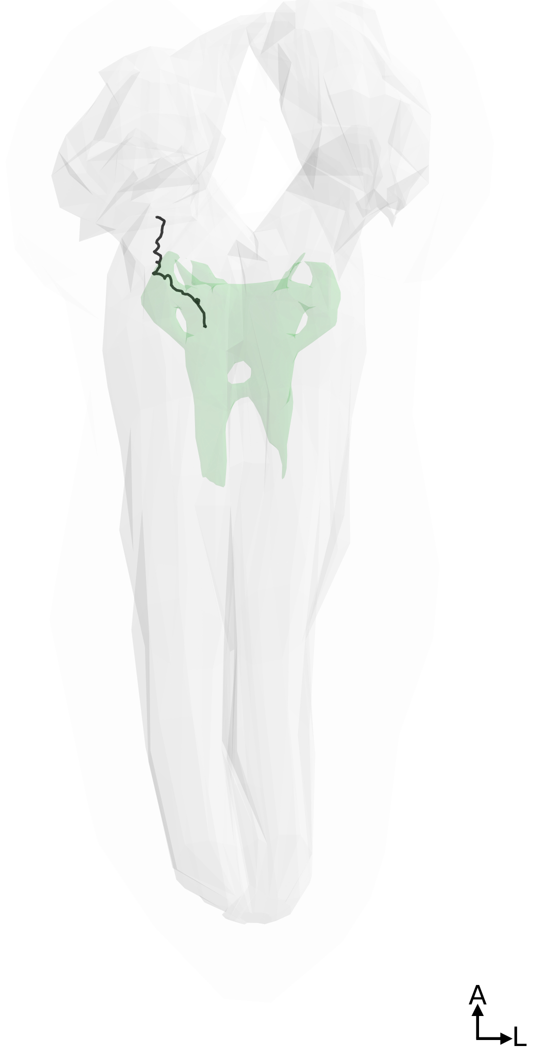

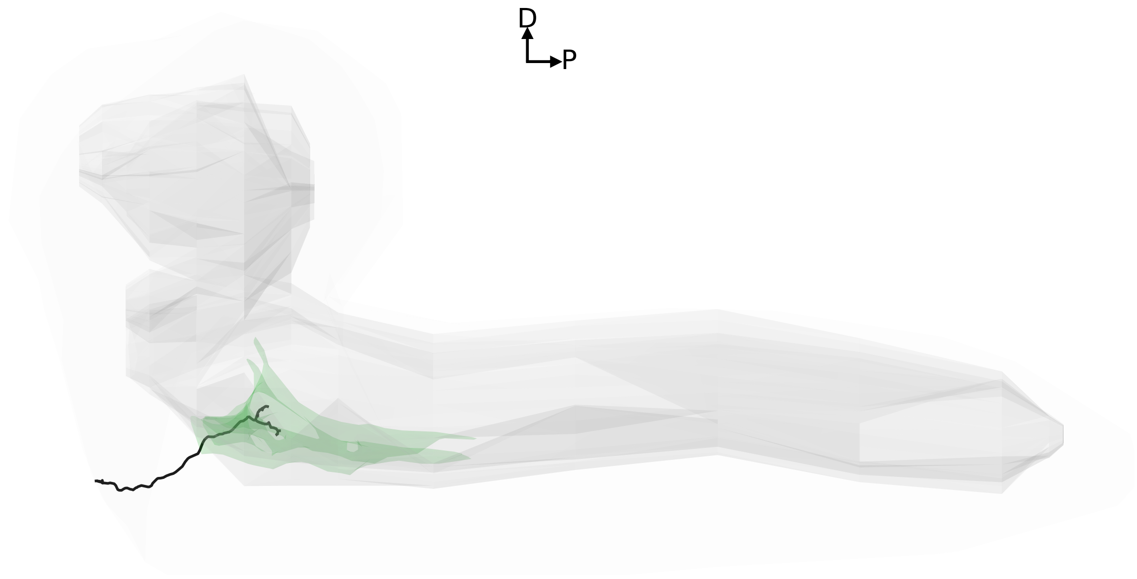

ID	name	SCACa	SCAVa	SCAVp	SCACal	SCACp	SCACpl	SCVM	IPCs	DMS	DH44	Se0ens	Se0ph	PMN LR	MN motor neurons	PaN motor neurons	olfactory PNs	gustatory PNs	multiglomerular PNs	unknown PNs	thermo PNs	visual PNs
15680710	MN-L-Sens-B2-VM-03	0	0	0	0	0	0	4	0	0	0	0	0	0	0	0	0	0	0	0	0	0

ID: 15715796
name: MN-L-Sens-B2-VM-04

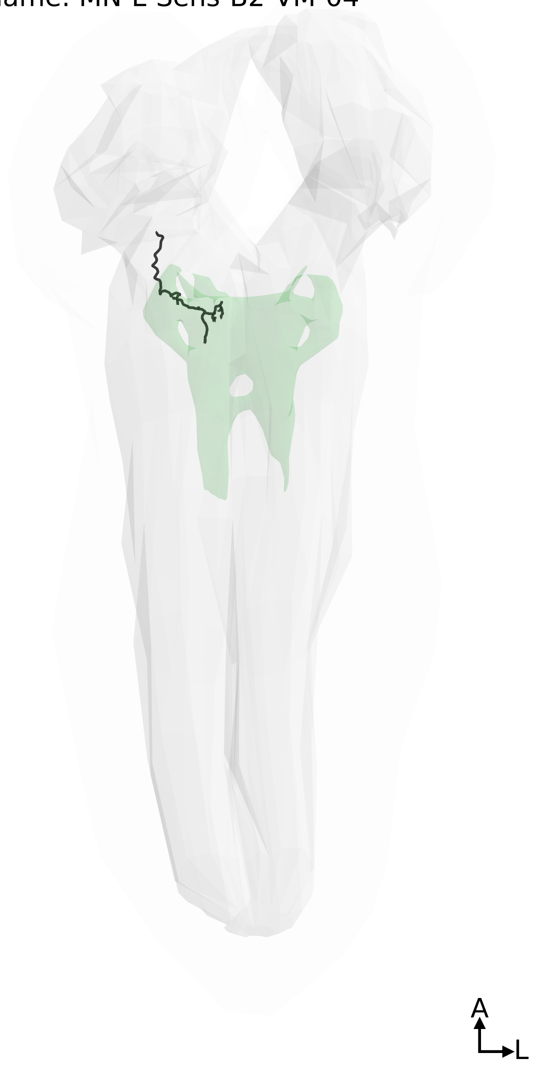

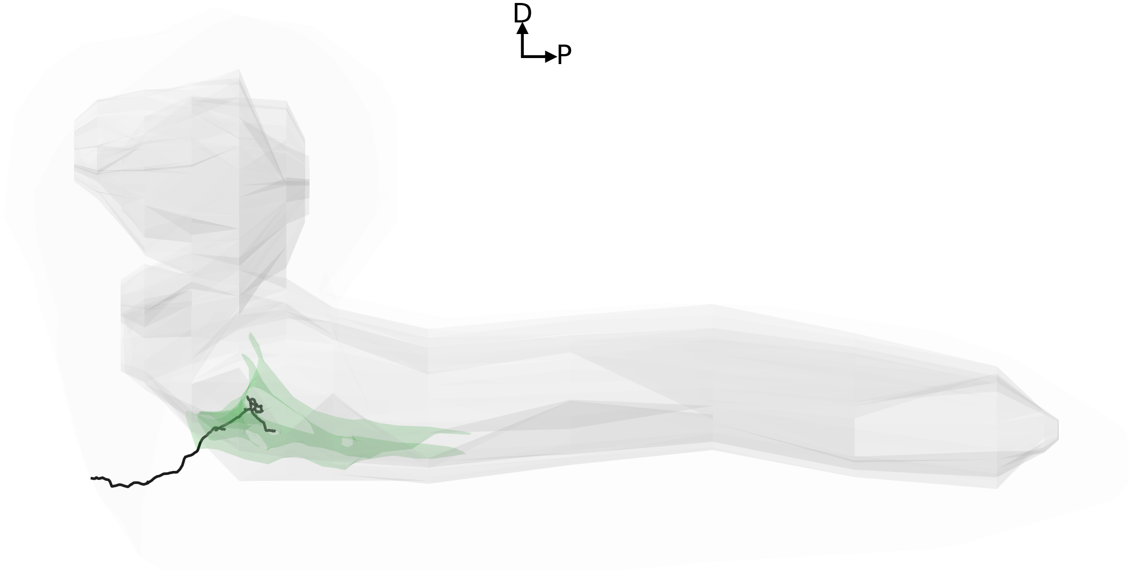

ID	*name*	SCACa	SCAVa	SCAVp	SCACal	SCACp	SCACpl	SCVM	IPCs	DMS	DH44	Se0ens	Se0ph	PMN LR	MN motor neurons	PaN motor neurons	olfactory PNs	gustatory PNs	multiglomerular PNs	unknown PNs	thermo PNs	visual PNs
15715796	MN-L-Sens-B2-VM-04	0	0	0	0	0	0	2	0	0	0	0	0	0	0	0	0	0	0	0	0	0

ID: 15677030
name: MN-L-Sens-B2-VM-05

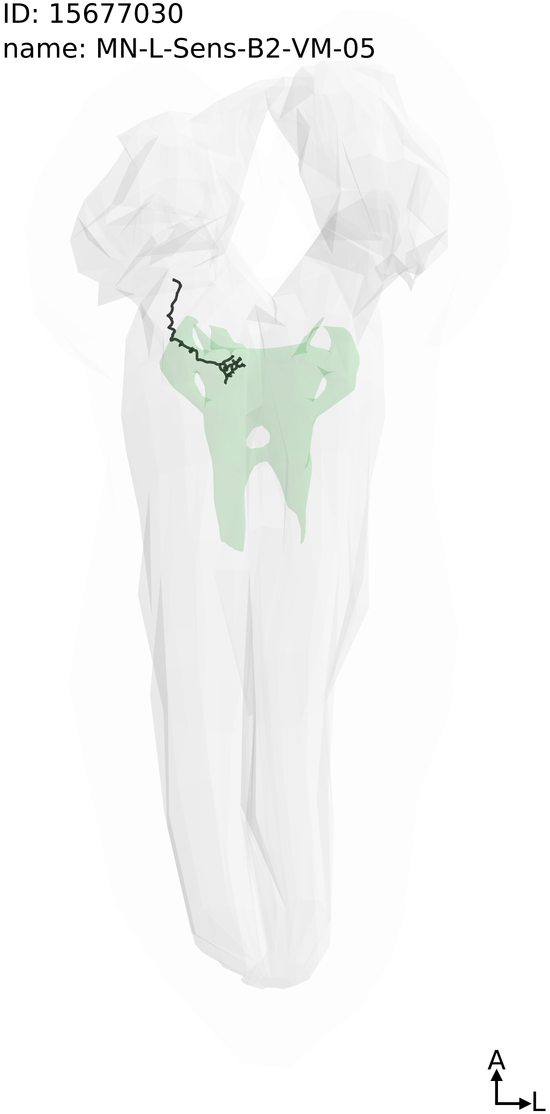

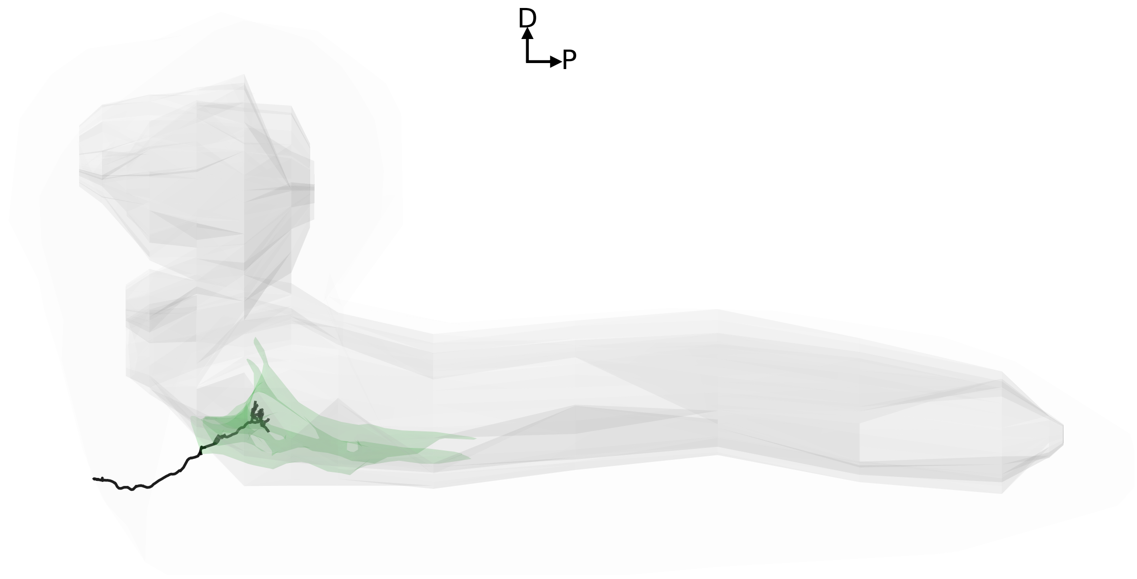

ID	name	SCACa	SCAVa	SCAVp	SCACal	SCACp	SCACpl	SCVM	IPCs	DMS	DH44	Se0ens	Se0ph	PMN LR	MN motor neurons	PaN motor neurons	olfactory PNs	gustatory PNs	multiglomerular PNs	unknown PNs	thermo PNs	visual PNs
15677030	MN-L-Sens-B2-VM-05	0	0	0	0	0	0	4	0	0	0	0	0	0	0	0	0	0	0	0	0	0

ID: 15690955
name: MN-L-Sens-B2-VM-06

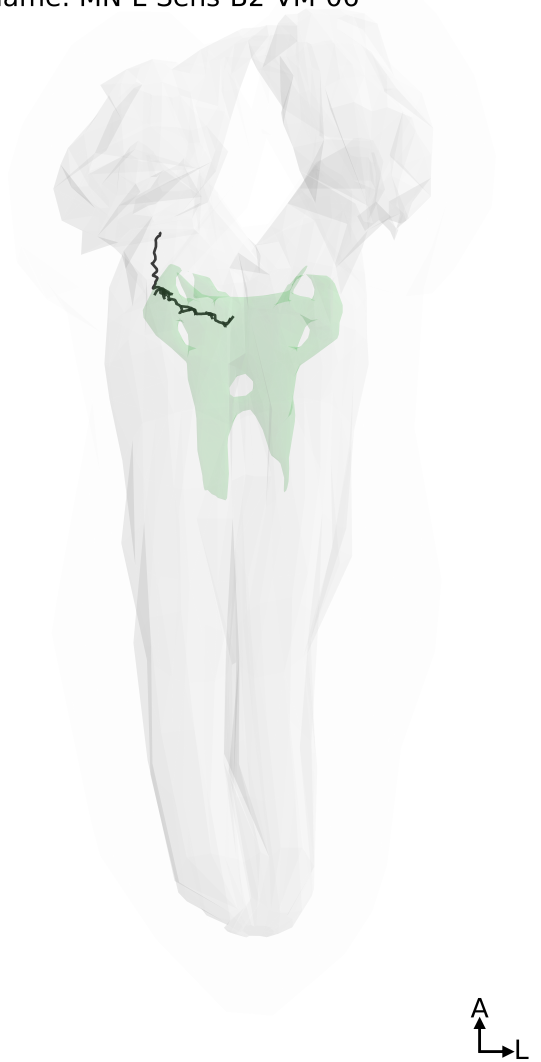

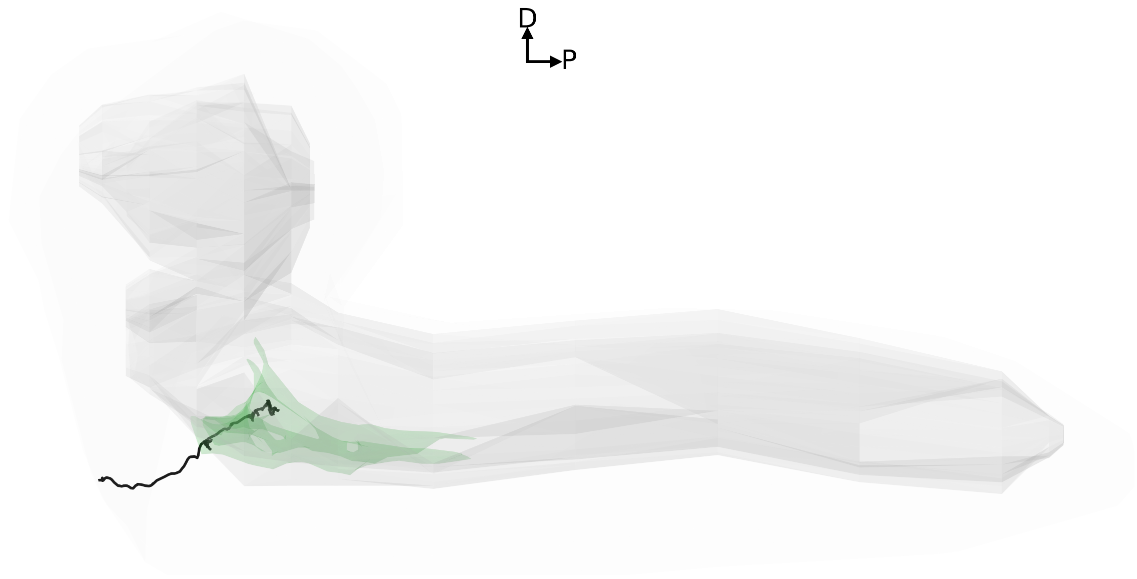

ID	name	SCACa	SCAVa	SCAVp	SCACal	SCACp	SCACpl	SCVM	IPCs	DMS	DH44	Se0ens	Se0ph	PMN LR	MN motor neurons	PaN motor neurons	olfactory PNs	gustatory PNs	multiglomerular PNs	unknown PNs	thermo PNs	visual PNs
15690955	MN-L-Sens-B2-VM-06	0	0	0	0	0	0	0	0	0	0	0	0	0	0	0	0	0	0	0	0	0

ID: 15711222
name: MN-L-Sens-B2-VM-07

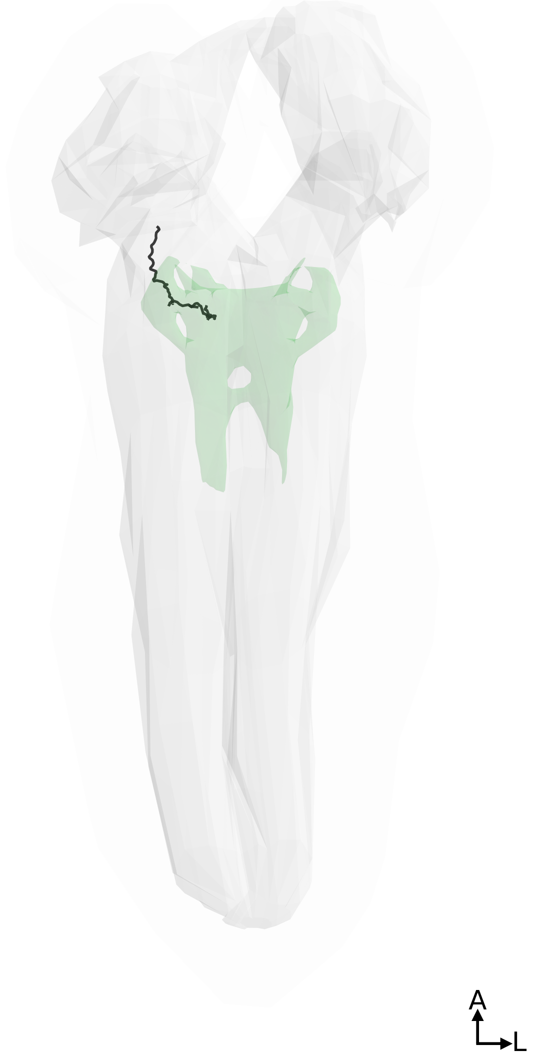

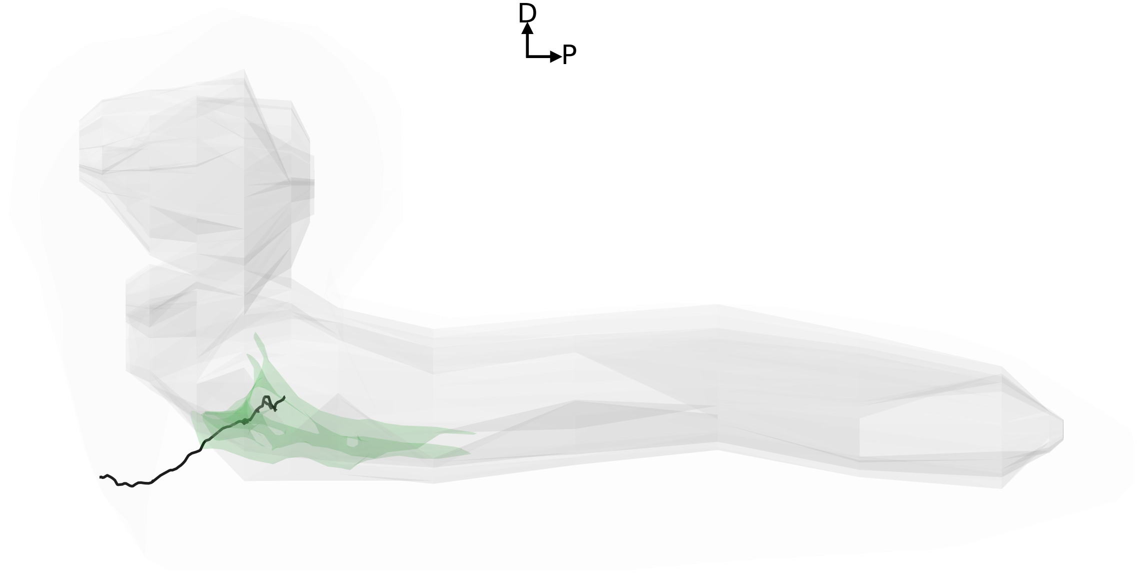

ID	*name*	SCACa	SCAVa	SCAVp	SCACal	SCACp	SCACpl	SCVM	IPCs	DMS	DH44	Se0ens	Se0ph	PMN LR	MN motor neurons	PaN motor neurons	olfactory PNs	gustatory PNs	multiglomerular PNs	unknown PNs	thermo PNs	visual PNs
15711222	MN-L-Sens-B2-VM-07	0	0	0	0	0	0	1	0	0	0	0	0	0	0	0	0	0	0	0	0	0

ID: 15679484
name: MN-L-Sens-B2-VM-08

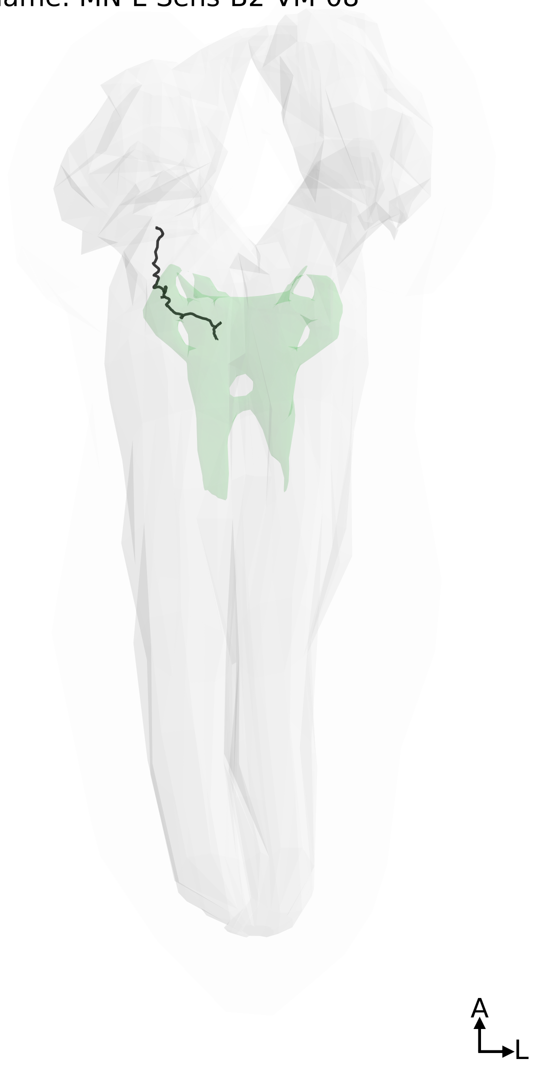

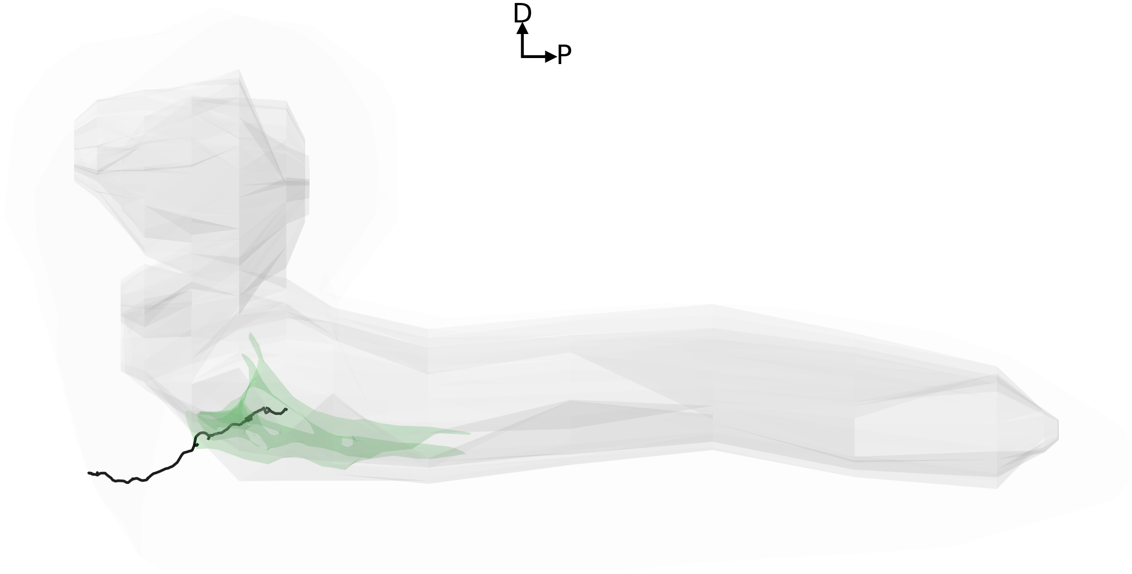

ID	name	SCACa	SCAVa	SCAVp	SCACal	SCACp	SCACpl	SCVM	IPCs	DMS	DH44	Se0ens	Se0ph	PMN LR	MN motor neurons	PaN motor neurons	olfactory PNs	gustatory PNs	multiglomerular PNs	unknown PNs	thermo PNs	visual PNs
15679484	MN-L-Sens-B2-VM-08	0	0	0	0	0	0	1	0	0	0	0	0	0	0	0	0	0	0	0	0	0

ID: 15678441
name: MN-L-Sens-B2-VM-09

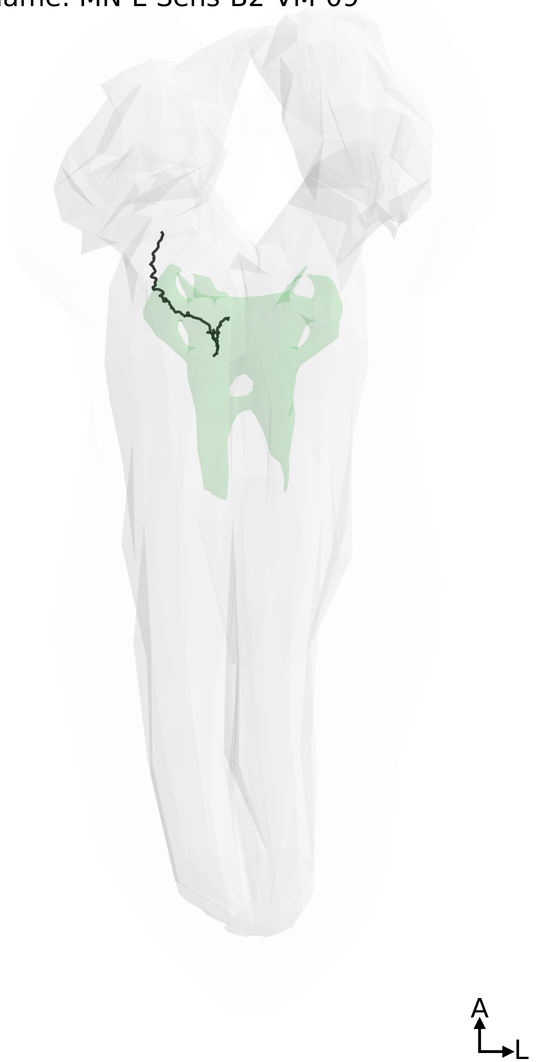

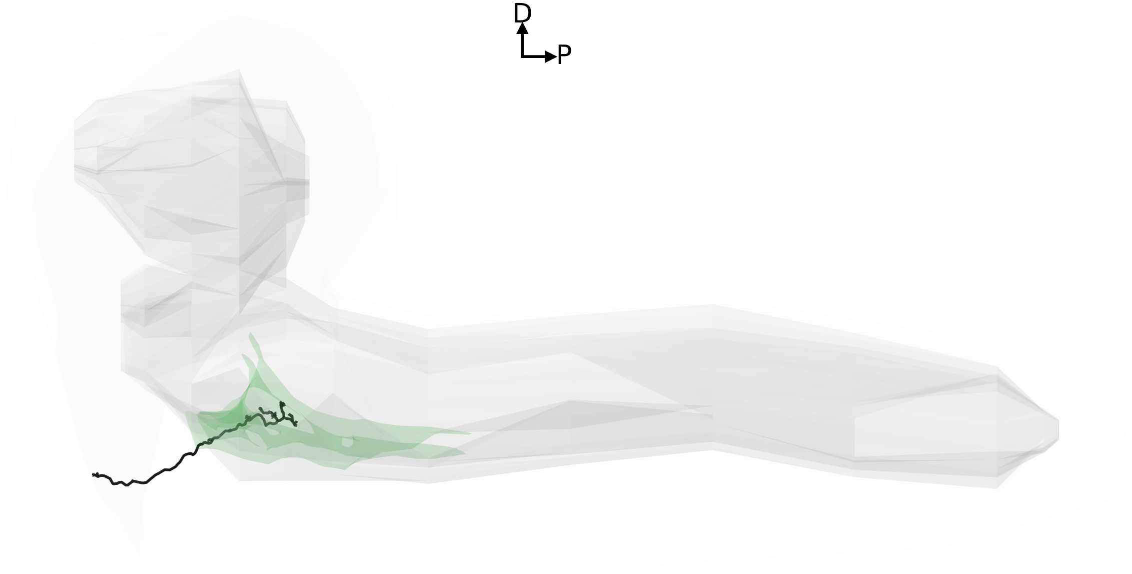

ID	name	SCACa	SCAVa	SCAVp	SCACal	SCACp	SCACpl	SCVM	IPCs	DMS	DH44	Se0ens	Se0ph	PMN LR	MN motor neurons	PaN motor neurons	olfactory PNs	gustatory PNs	multiglomerular PNs	unknown PNs	thermo PNs	visual PNs
15678441	MN-L-Sens-B2-VM-09	0	0	0	0	0	0	2	0	0	0	0	0	0	0	0	0	0	0	0	0	0

ID: 15714845
name: MN-L-Sens-B2-VM-10

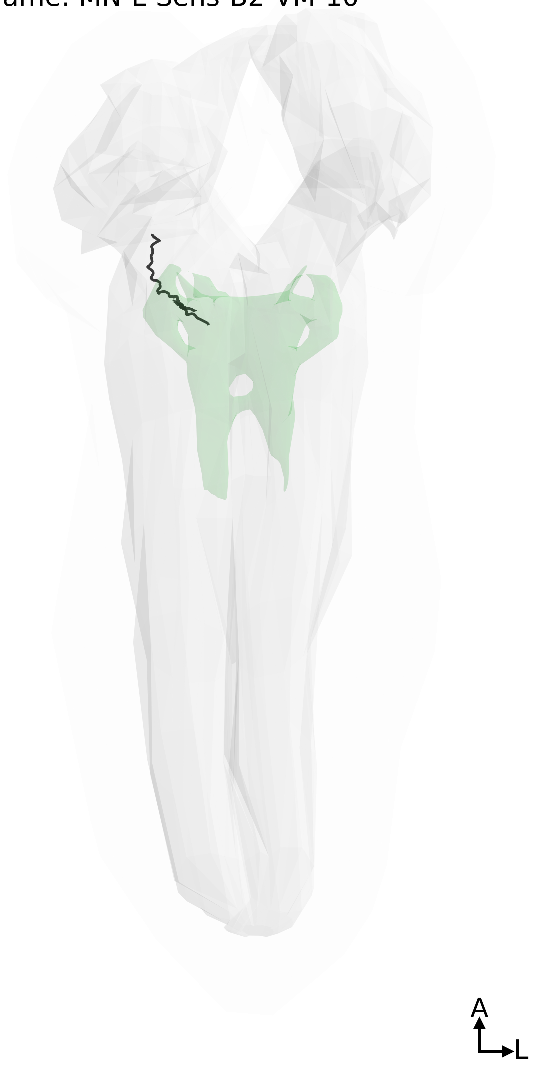

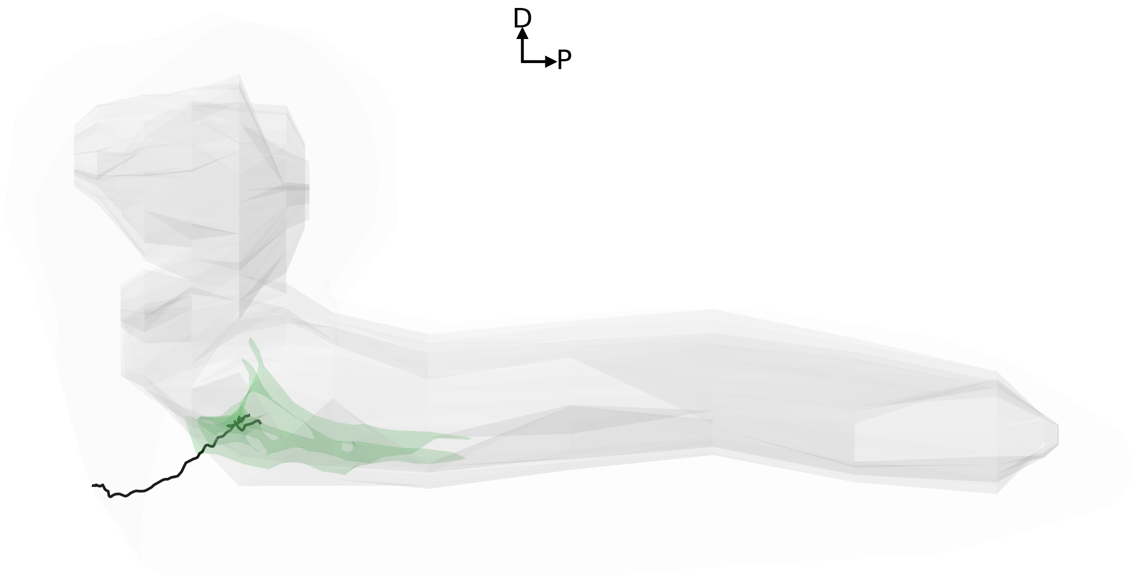

ID	*name*	SCACa	SCAVa	SCAVp	SCACal	SCACp	SCACpl	SCVM	IPCs	DMS	DH44	Se0ens	Se0ph	PMN LR	MN motor neurons	PaN motor neurons	olfactory PNs	gustatory PNs	multiglomerular PNs	unknown PNs	thermo PNs	visual PNs
15714845	MN-L-Sens-B2-VM-10	0	0	0	0	1	0	2	0	0	0	0	0	0	0	0	0	0	0	0	0	0

ID: 15690392
name: MN-L-Sens-B2-VM-11

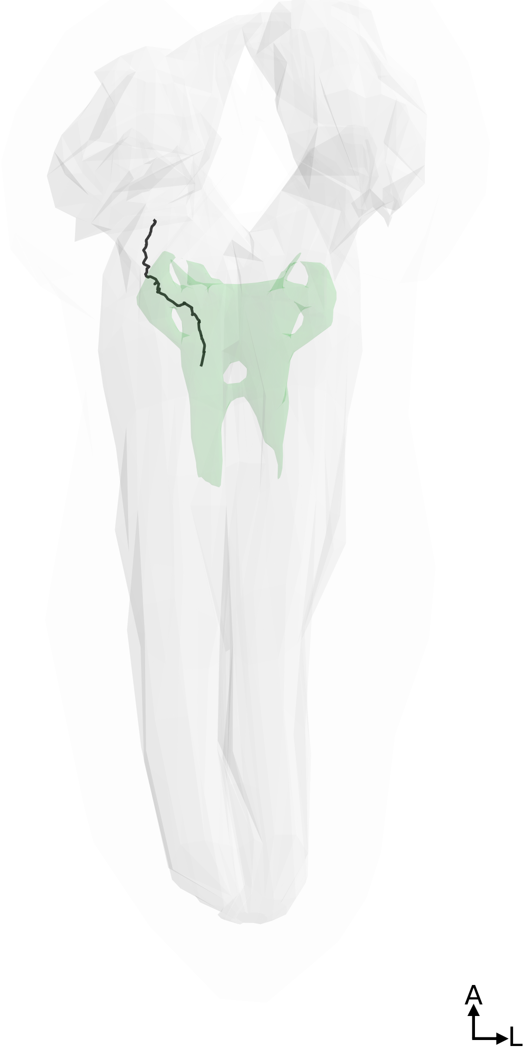

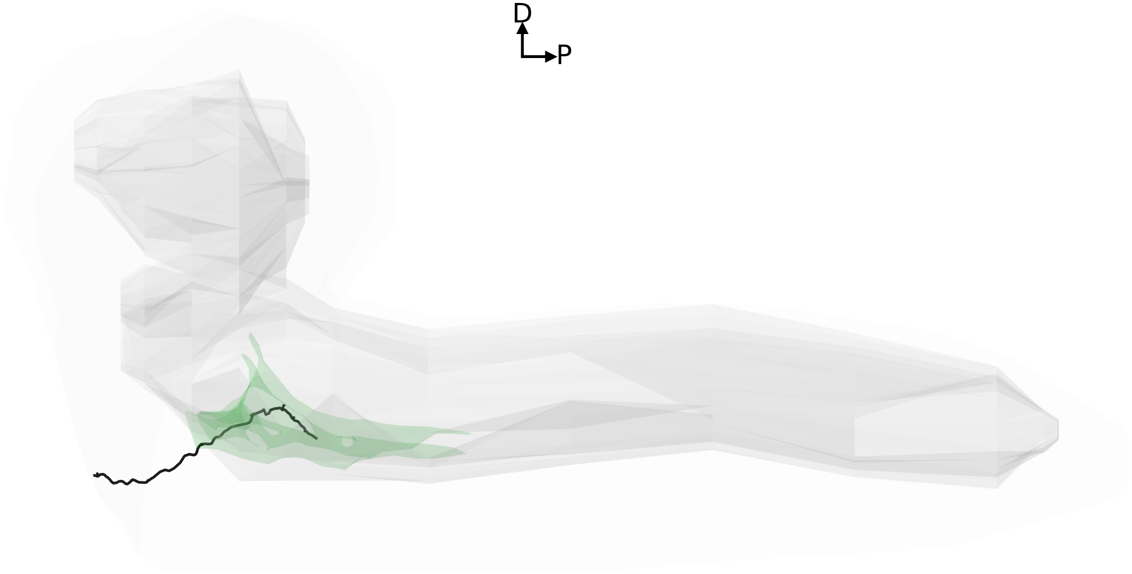

ID	*name*	SCACa	SCAVa	SCAVp	SCACal	SCACp	SCACpl	SCVM	IPCs	DMS	DH44	Se0ens	Se0ph	PMN LR	MN motor neurons	PaN motor neurons	olfactory PNs	gustatory PNs	multiglomerular PNs	unknown PNs	thermo PNs	visual PNs
15690392	MN-L-Sens-B2-VM-11	0	0	0	0	0	0	3	0	0	0	0	0	0	0	0	0	0	0	0	0	0

ID: 15678849
name: MN-L-Sens-B2-VM-12

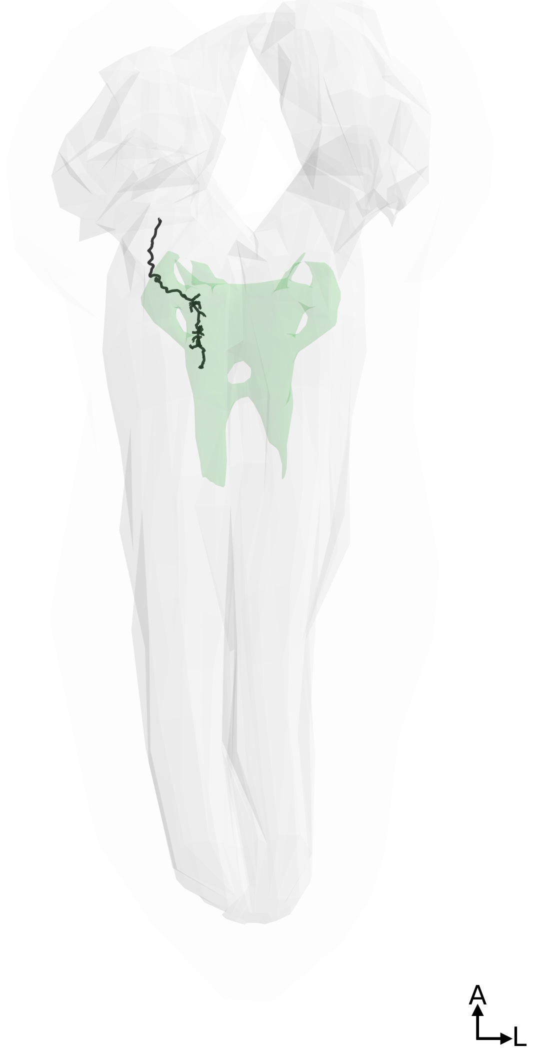

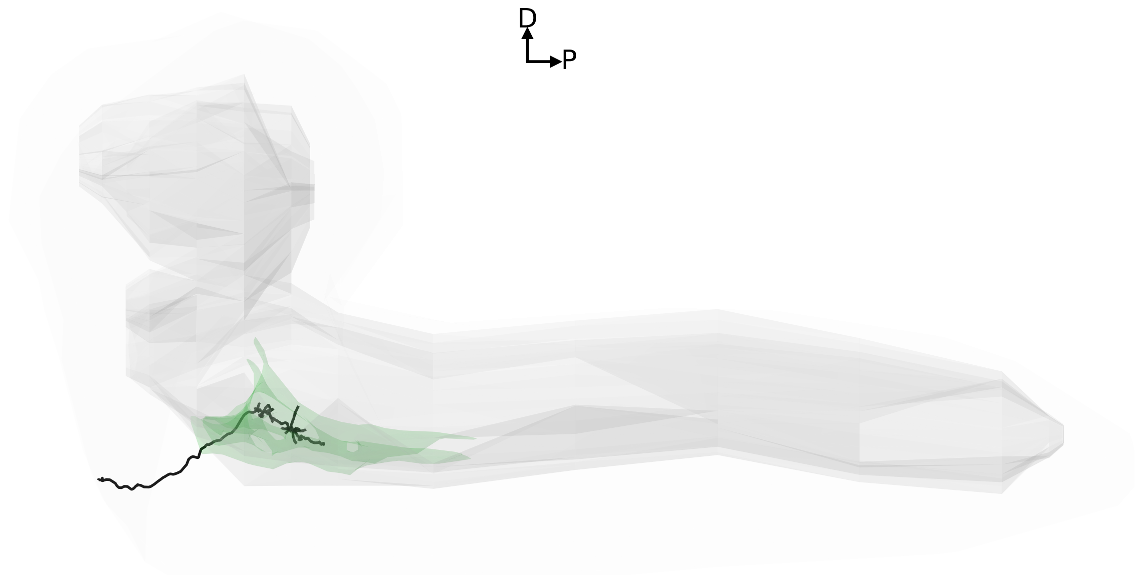

ID	*name*	SCACa	SCAVa	SCAVp	SCACal	SCACp	SCACpl	SCVM	IPCs	DMS	DH44	Se0ens	Se0ph	PMN LR	MN motor neurons	PaN motor neurons	olfactory PNs	gustatory PNs	multiglomerular PNs	unknown PNs	thermo PNs	visual PNs
15678849	MN-L-Sens-B2-VM-12	0	0	0	0	0	0	4	0	0	0	0	0	0	0	0	0	0	0	0	0	0

ID: 15712024
name: MN-L-Sens-B2-VM-13

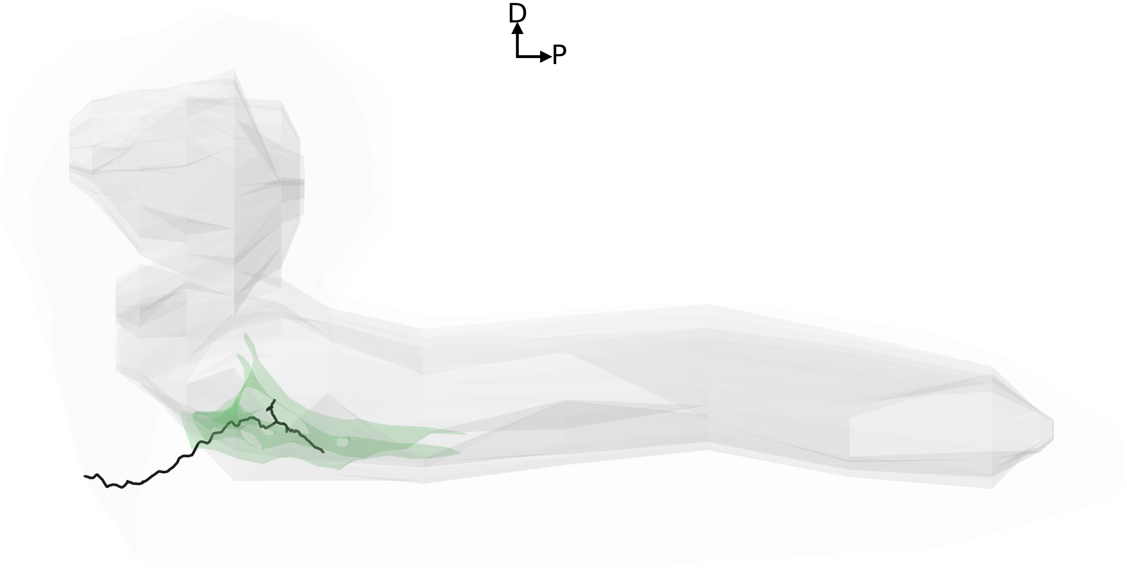

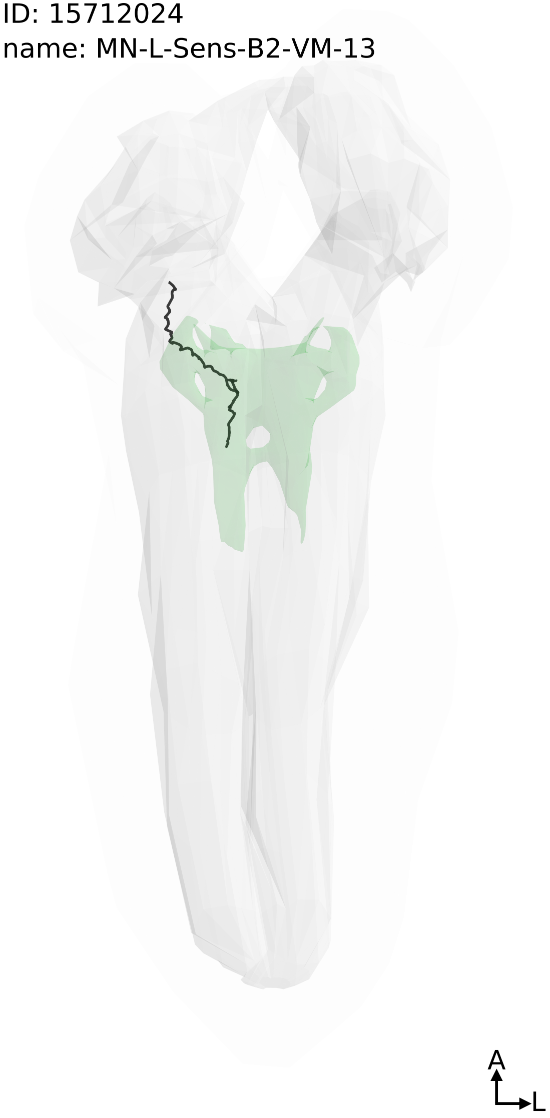

ID	*name*	SCACa	SCAVa	SCAVp	SCACal	SCACp	SCACpl	SCVM	IPCs	DMS	DH44	Se0ens	Se0ph	PMN LR	MN motor neurons	PaN motor neurons	olfactory PNs	gustatory PNs	multiglomerular PNs	unknown PNs	thermo PNs	visual PNs
15712024	MN-L-Sens-B2-VM-13	0	0	0	0	0	0	1	0	0	0	0	0	0	0	0	0	0	0	0	0	0

ID: 15690711
name: MN-L-Sens-B2-VM-14

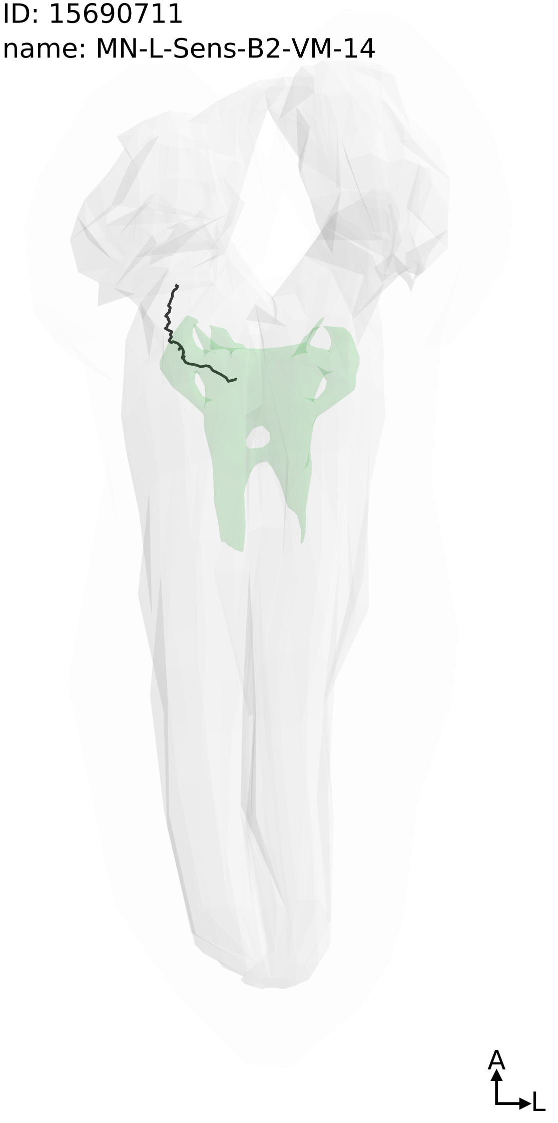

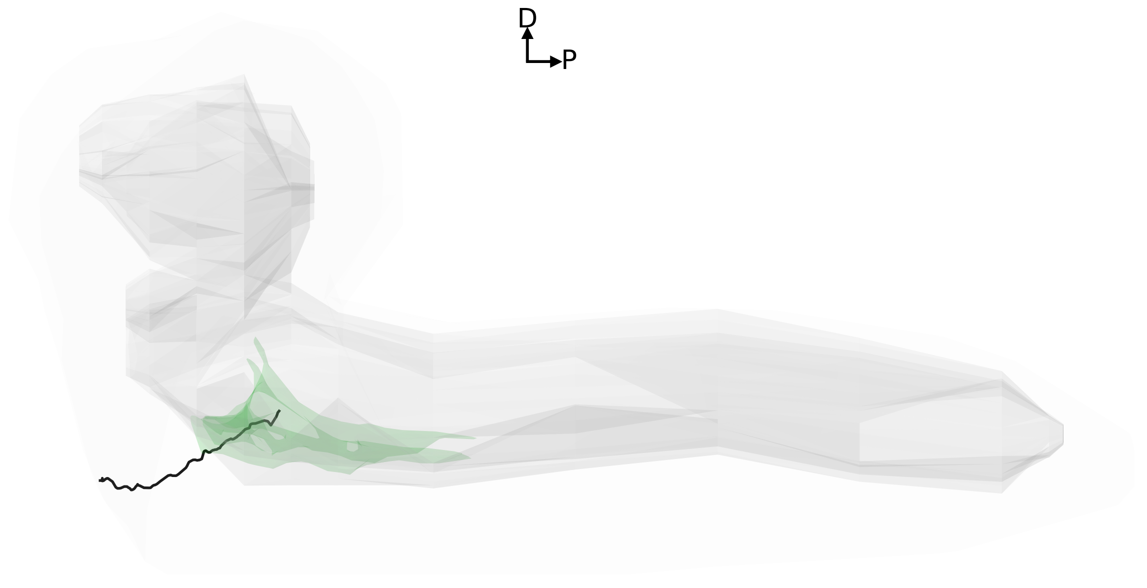

ID	name	SCACa	SCAVa	SCAVp	SCACal	SCACp	SCACpl	SCVM	IPCs	DMS	DH44	Se0ens	Se0ph	PMN LR	MN motor neurons	PaN motor neurons	olfactory PNs	gustatory PNs	multiglomerular PNs	unknown PNs	thermo PNs	visual PNs
15690711	MN-L-Sens-B2-VM-14	0	0	0	0	0	0	0	0	0	0	0	0	0	0	0	0	0	0	0	0	0

ID: 15717229
name: MN-L-Sens-B2-VM-15

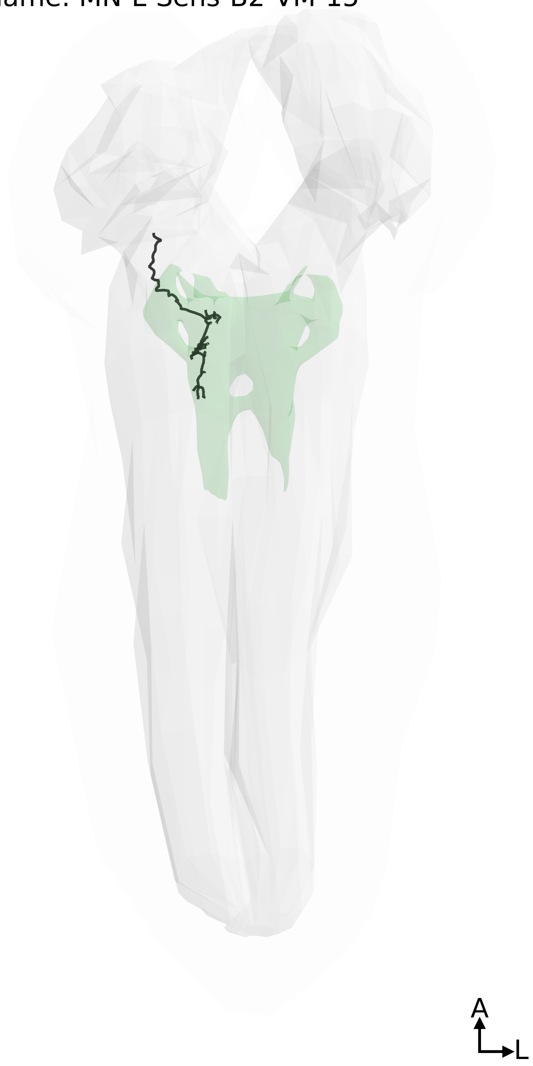

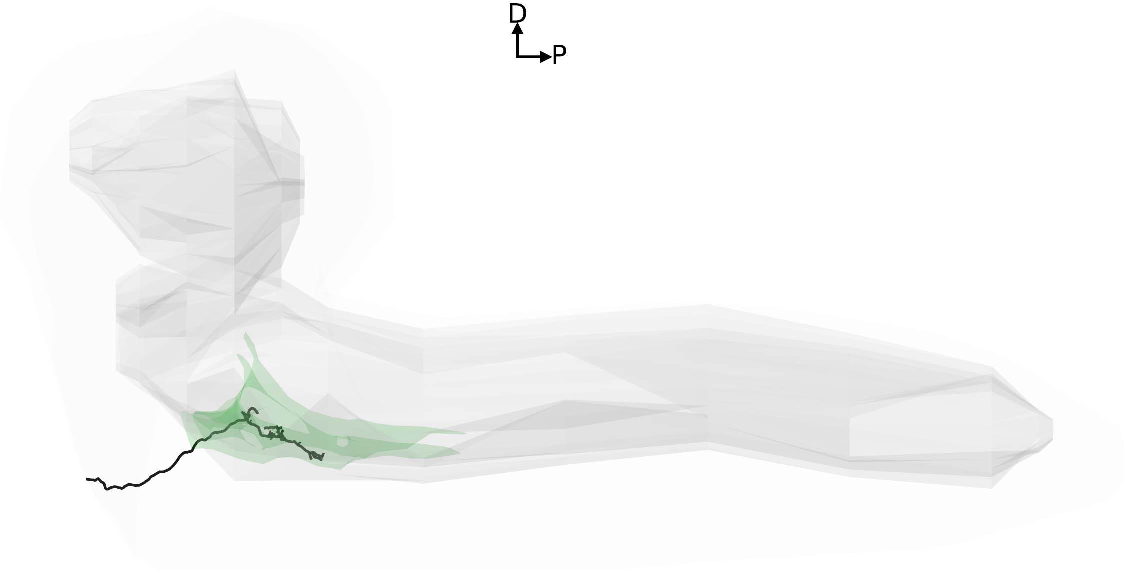

ID	name	SCACa	SCAVa	SCAVp	SCACal	SCACp	SCACpl	SCVM	IPCs	DMS	DH44	Se0ens	Se0ph	PMN LR	MN motor neurons	PaN motor neurons	olfactory PNs	gustatory PNs	multiglomerular PNs	unknown PNs	thermo PNs	visual PNs
15717229	MN-L-Sens-B2-VM-15	0	0	0	0	0	0	4	0	0	0	0	0	0	0	0	0	0	0	0	0	0

ID: 15711289
name: MN-L-Sens-B2-VM-16

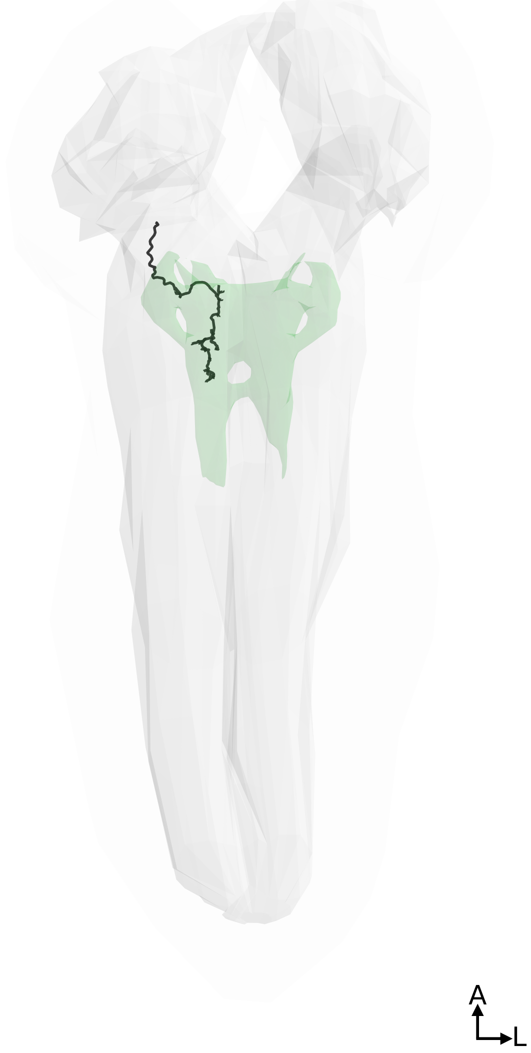

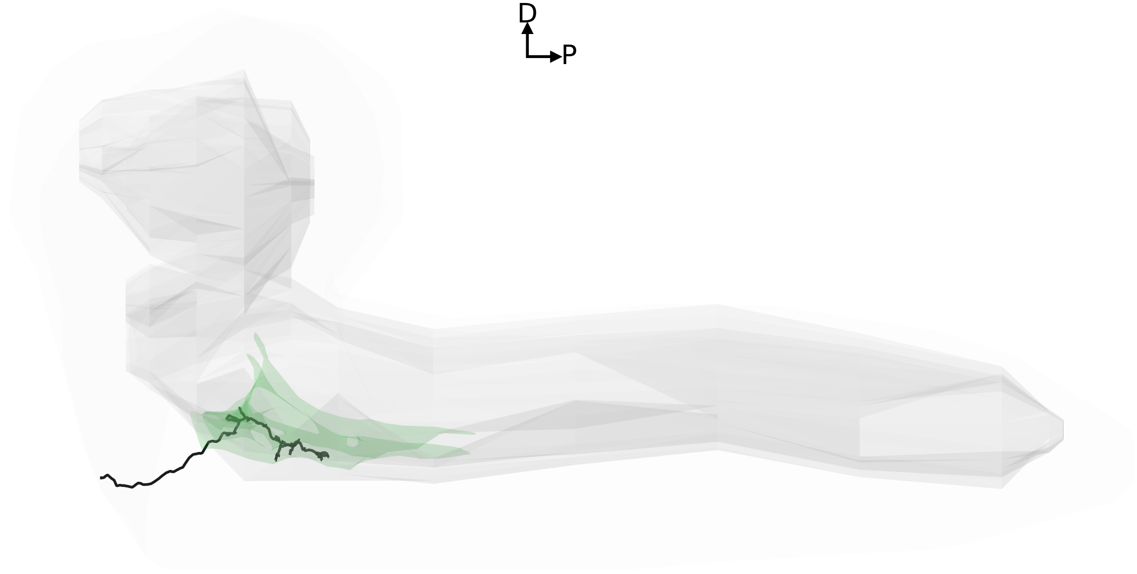

ID	*name*	SCACa	SCAVa	SCAVp	SCACal	SCACp	SCACpl	SCVM	IPCs	DMS	DH44	Se0ens	Se0ph	PMN LR	MN motor neurons	PaN motor neurons	olfactory PNs	gustatory PNs	multiglomerular PNs	unknown PNs	thermo PNs	visual PNs
15711289	MN-L-Sens-B2-VM-16	0	0	1	0	0	0	8	0	0	0	0	0	0	0	0	0	0	0	0	0	0

ID: 15713374
name: MN-L-Sens-B2-VM-17

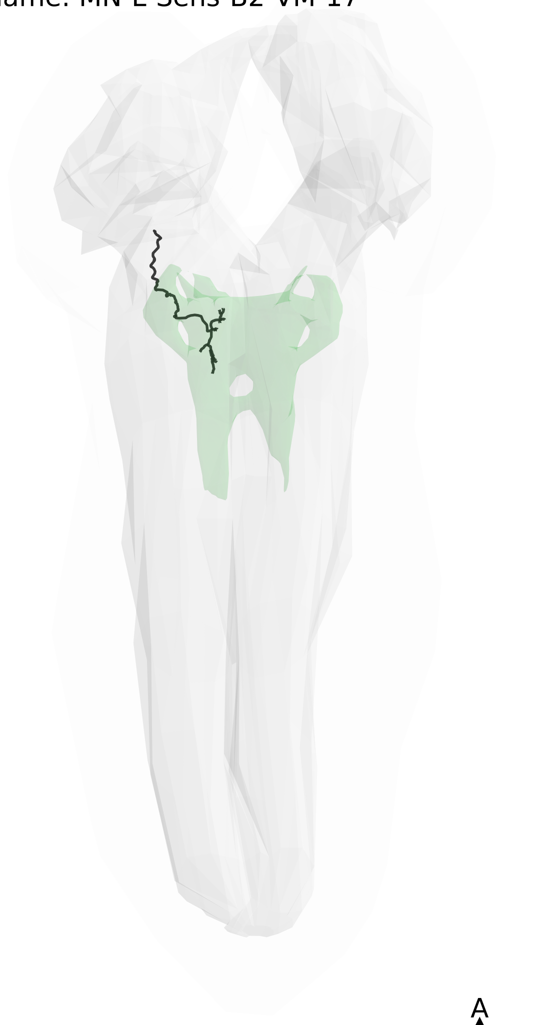

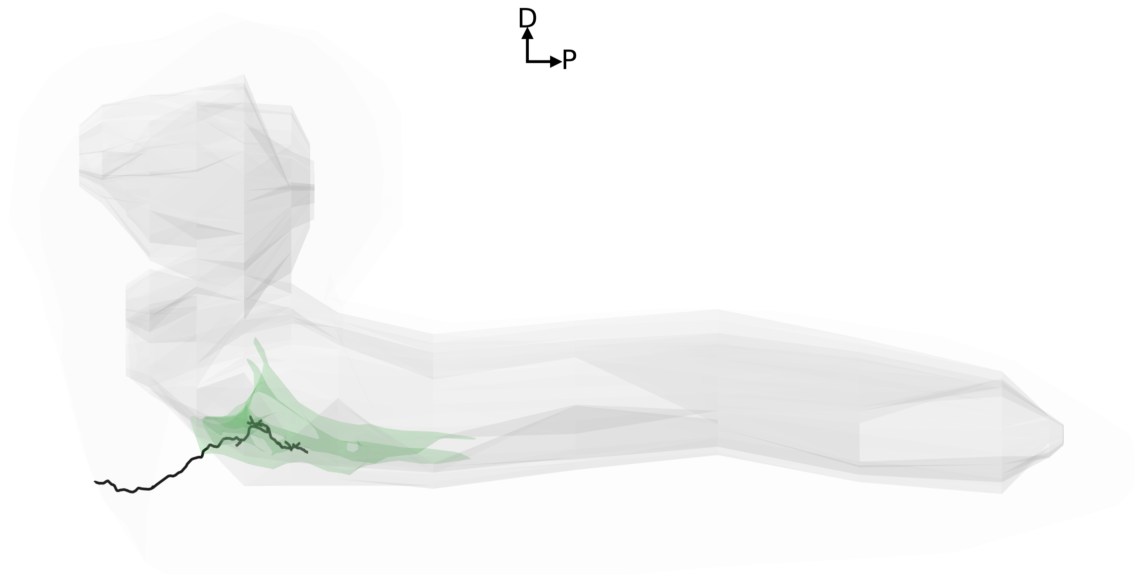

ID	name	SCACa	SCAVa	SCAVp	SCACal	SCACp	SCACpl	SCVM	IPCs	DMS	DH44	Se0ens	Se0ph	PMN LR	MN motor neurons	PaN motor neurons	olfactory PNs	gustatory PNs	multiglomerular PNs	unknown PNs	thermo PNs	visual PNs
15713374	MN-L-Sens-B2-VM-17	0	0	0	0	0	1	2	0	0	0	0	0	0	0	0	0	0	0	0	0	0

ID: 15713206
name: MN-L-Sens-B2-VM-18

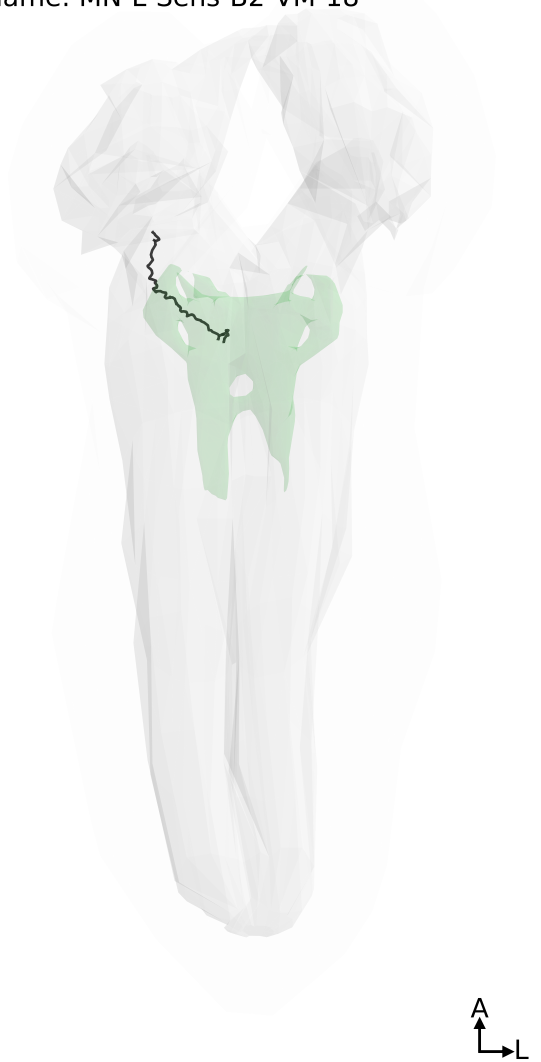

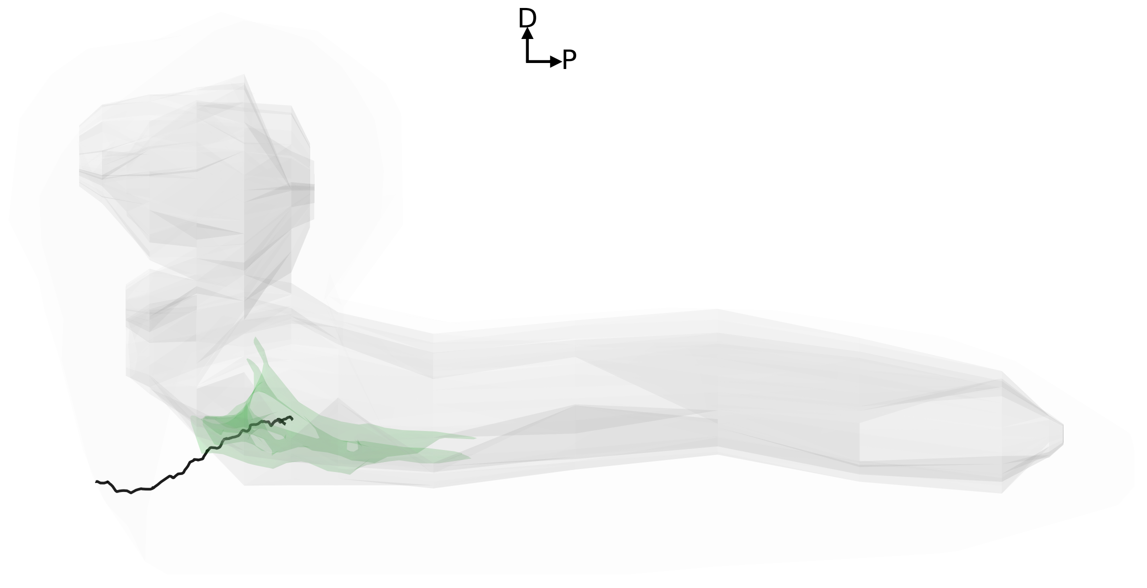

ID	*name*	SCACa	SCAVa	SCAVp	SCACal	SCACp	SCACpl	SCVM	IPCs	DMS	DH44	Se0ens	Se0ph	PMN LR	MN motor neurons	PaN motor neurons	olfactory PNs	gustatory PNs	multiglomerular PNs	unknown PNs	thermo PNs	visual PNs
15713206	MN-L-Sens-B2-VM-18	0	0	0	0	0	0	0	0	0	0	0	0	0	0	0	0	0	0	0	0	0

ID: 3267019
name: MN-L-Sens-B2-VM-19

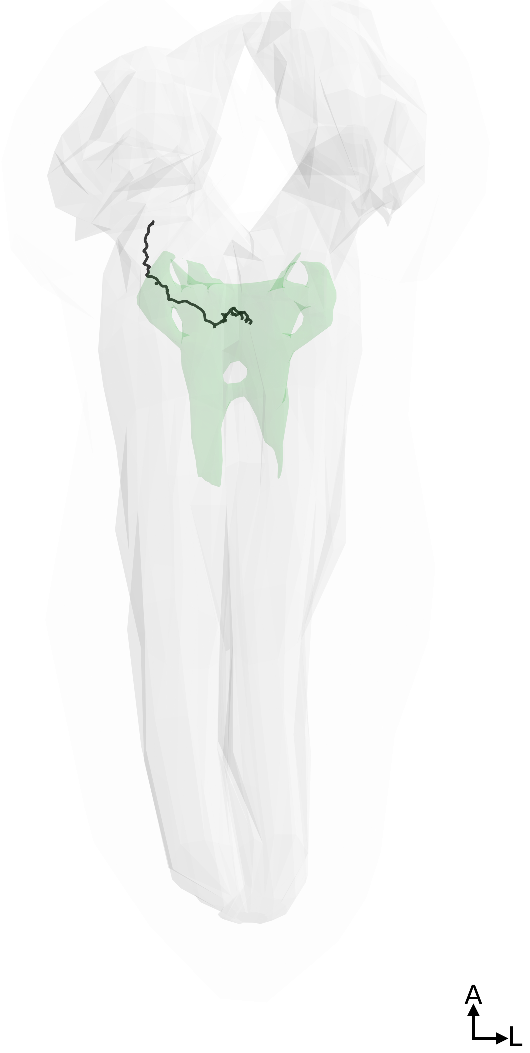

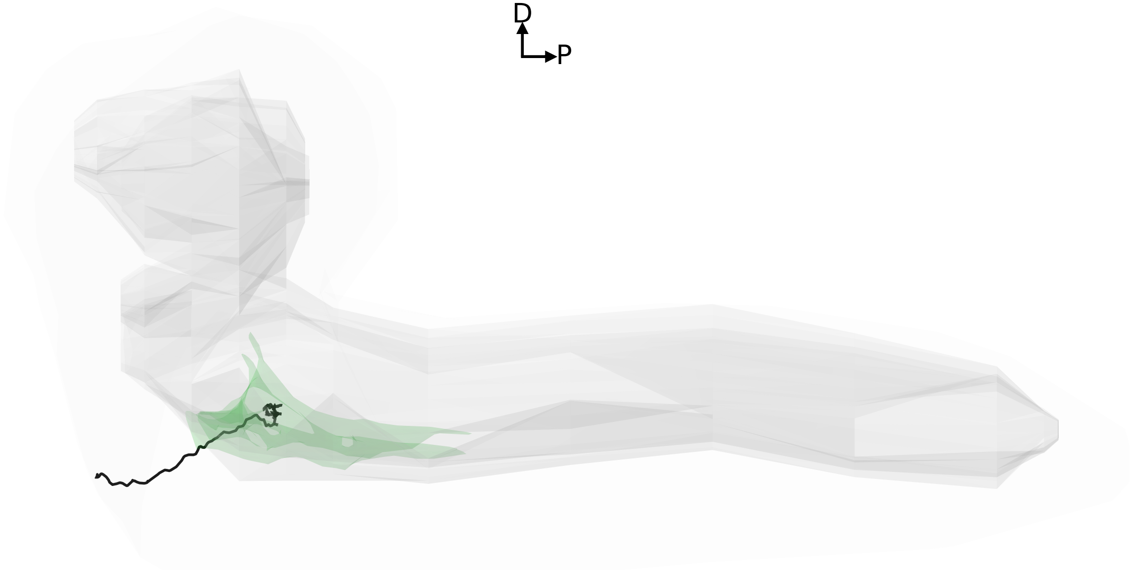

ID	*name*	SCACa	SCAVa	SCAVp	SCACal	SCACp	SCACpl	SCVM	IPCs	DMS	DH44	Se0ens	Se0ph	PMN LR	MN motor neurons	PaN motor neurons	olfactory PNs	gustatory PNs	multiglomerular PNs	unknown PNs	thermo PNs	visual PNs
3267019	MN-L-Sens-B2-VM-19	0	0	0	0	0	0	3	0	0	0	0	0	0	0	0	0	0	0	0	0	0

ID: 15730680
name: MN-L-Sens-B2-VM-20

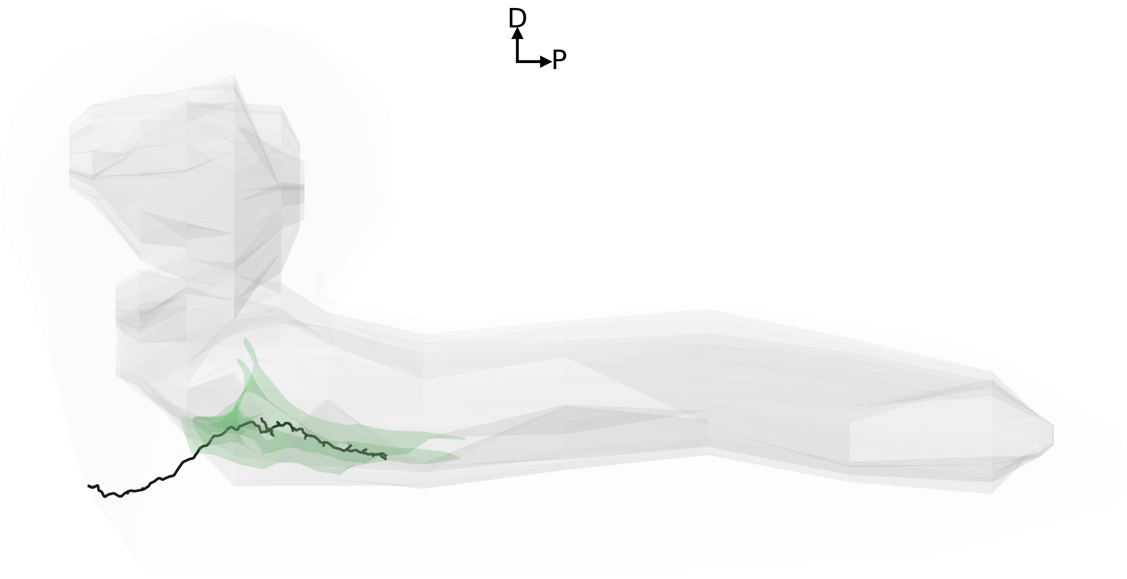

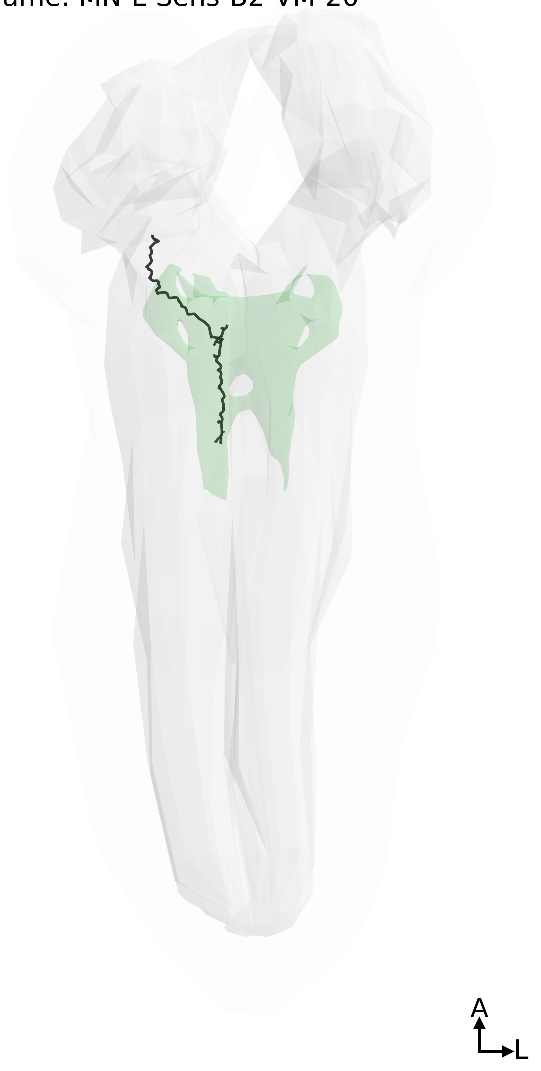

ID	name	SCACa	SCAVa	SCAVp	SCACal	SCACp	SCACpl	SCVM	IPCs	DMS	DH44	Se0ens	Se0ph	PMN LR	MN motor neurons	PaN motor neurons	olfactory PNs	gustatory PNs	multiglomerular PNs	unknown PNs	thermo PNs	visual PNs
15730680	MN-L-Sens-B2-VM-20	0	0	0	0	0	0	1	0	0	0	0	0	0	0	0	0	0	0	0	0	0

ID: 15678833
name: MN-L-Sens-B2-VM-21

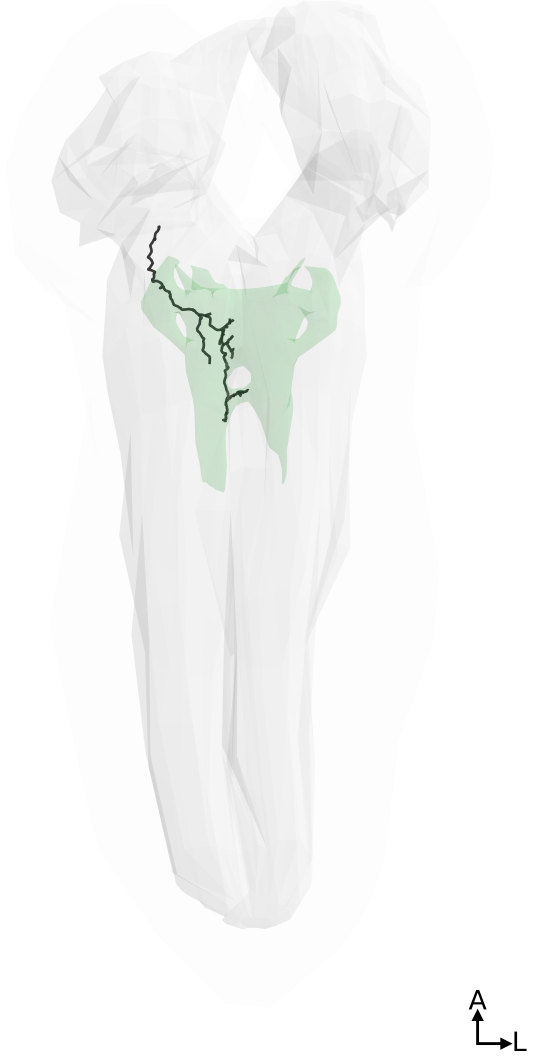

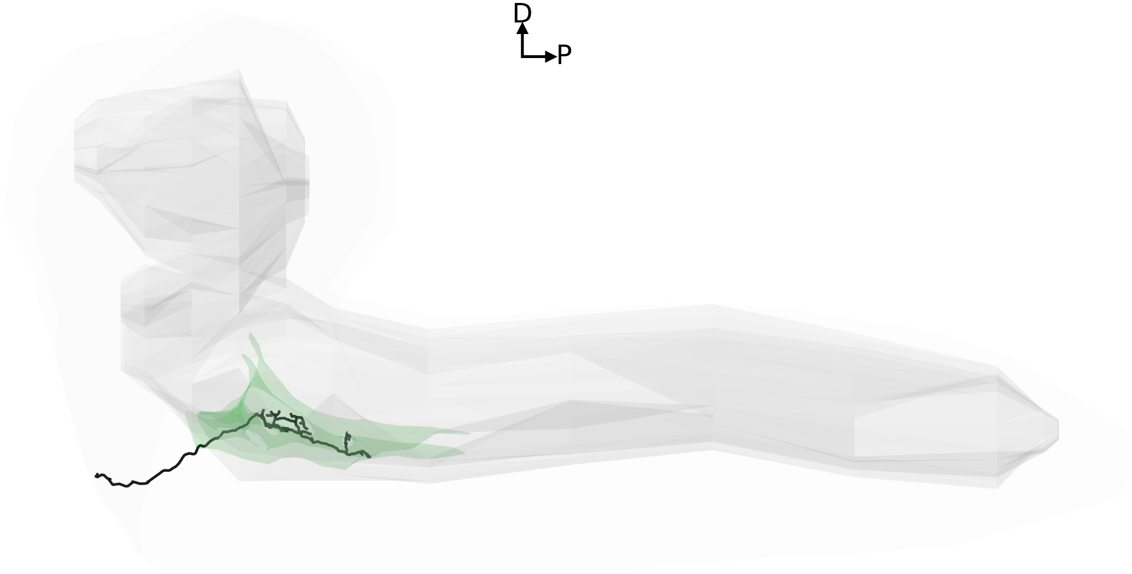

ID	*name*	SCACa	SCAVa	SCAVp	SCACal	SCACp	SCACpl	SCVM	IPCs	DMS	DH44	Se0ens	Se0ph	PMN LR	MN motor neurons	PaN motor neurons	olfactory PNs	gustatory PNs	multiglomerular PNs	unknown PNs	thermo PNs	visual PNs
15678833	MN-L-Sens-B2-VM-21	0	0	0	0	0	0	4	0	0	0	0	0	0	0	0	0	0	0	0	0	0

ID: 15675901
name: MN-L-Sens-B2-VM-22

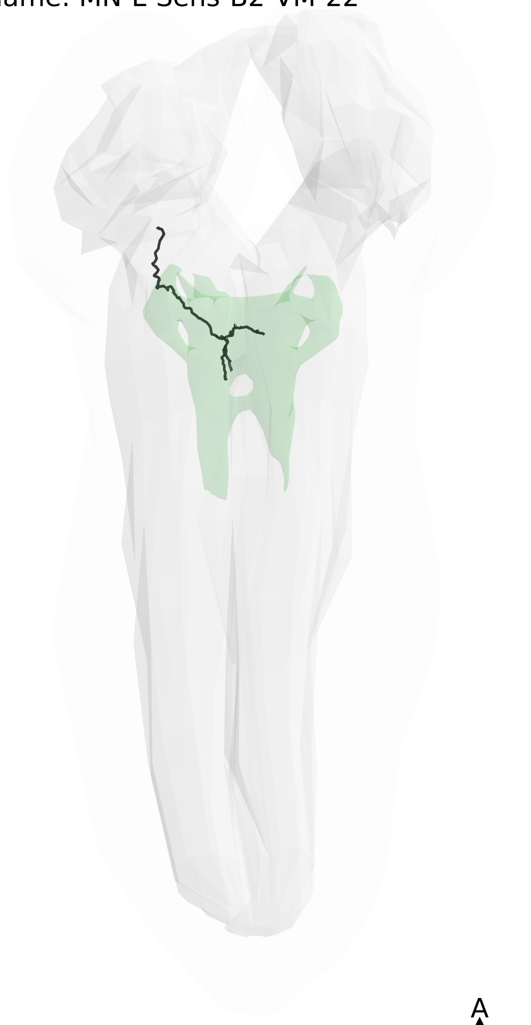

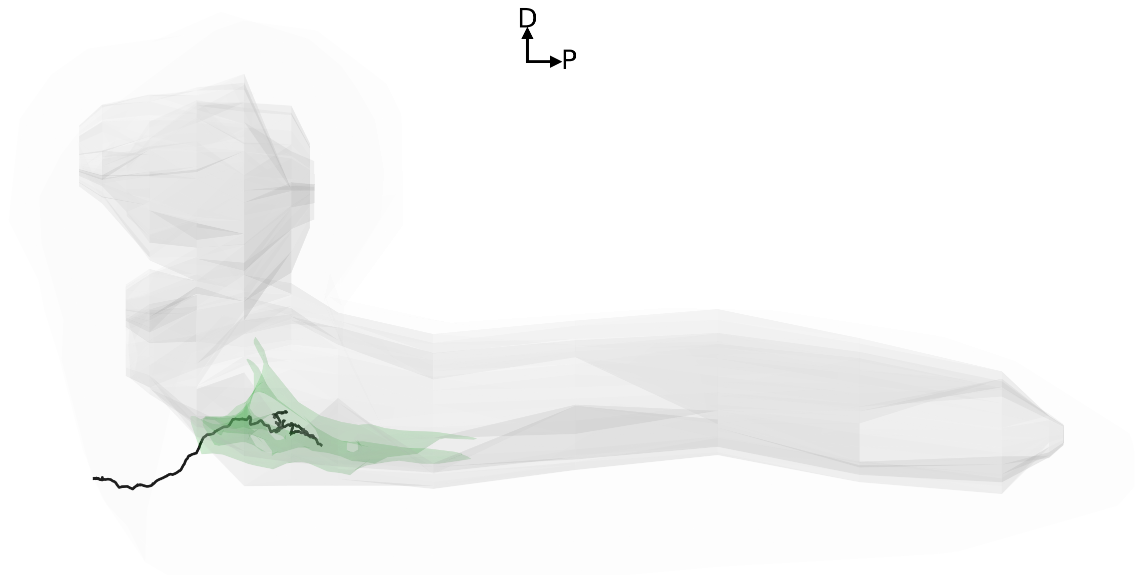

ID	*name*	SCACa	SCAVa	SCAVp	SCACal	SCACp	SCACpl	SCVM	IPCs	DMS	DH44	Se0ens	Se0ph	PMN LR	MN motor neurons	PaN motor neurons	olfactory PNs	gustatory PNs	multiglomerular PNs	unknown PNs	thermo PNs	visual PNs
15675901	MN-L-Sens-B2-VM-22	0	0	0	0	0	0	2	0	0	0	0	0	0	0	0	0	0	0	0	0	0

ID: 15699715
name: MN-L-Sens-B2-VM-23

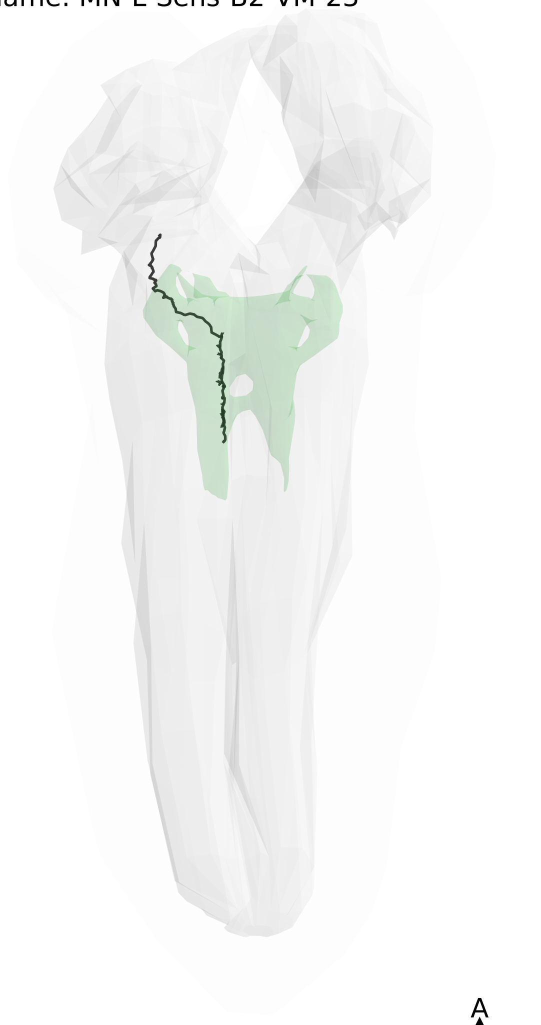

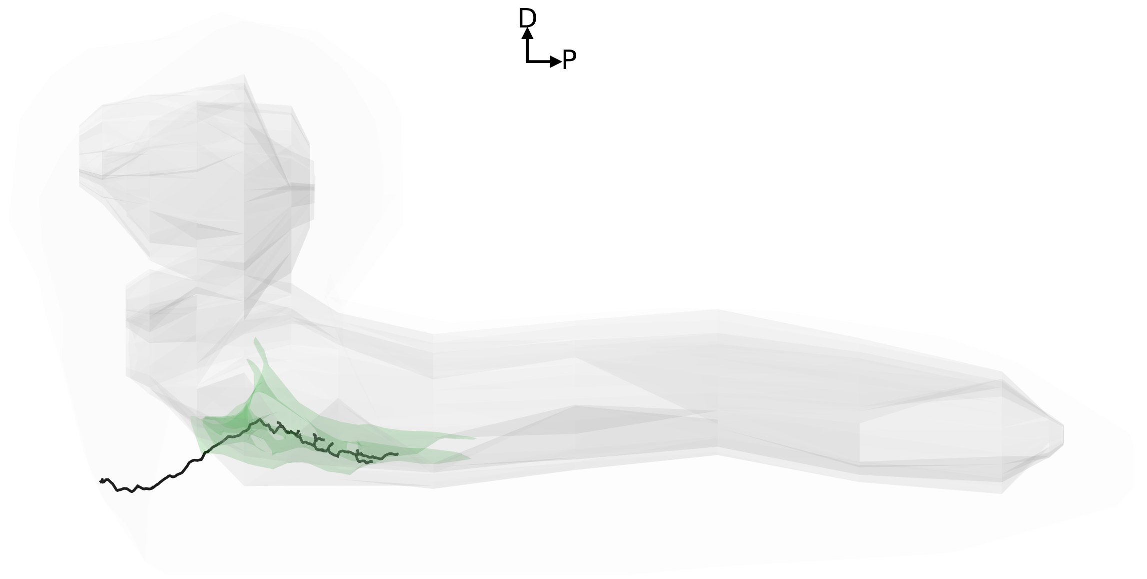

ID	name	SCACa	SCAVa	SCAVp	SCACal	SCACp	SCACpl	SCVM	IPCs	DMS	DH44	Se0ens	Se0ph	PMN LR	MN motor neurons	PaN motor neurons	olfactory PNs	gustatory PNs	multiglomerular PNs	unknown PNs	thermo PNs	visual PNs
15699715	MN-L-Sens-B2-VM-23	0	0	0	0	0	0	7	0	0	0	0	0	0	0	0	0	0	0	0	0	0

ID: 19180425
name: MN-L-Sens-B2-VM-24

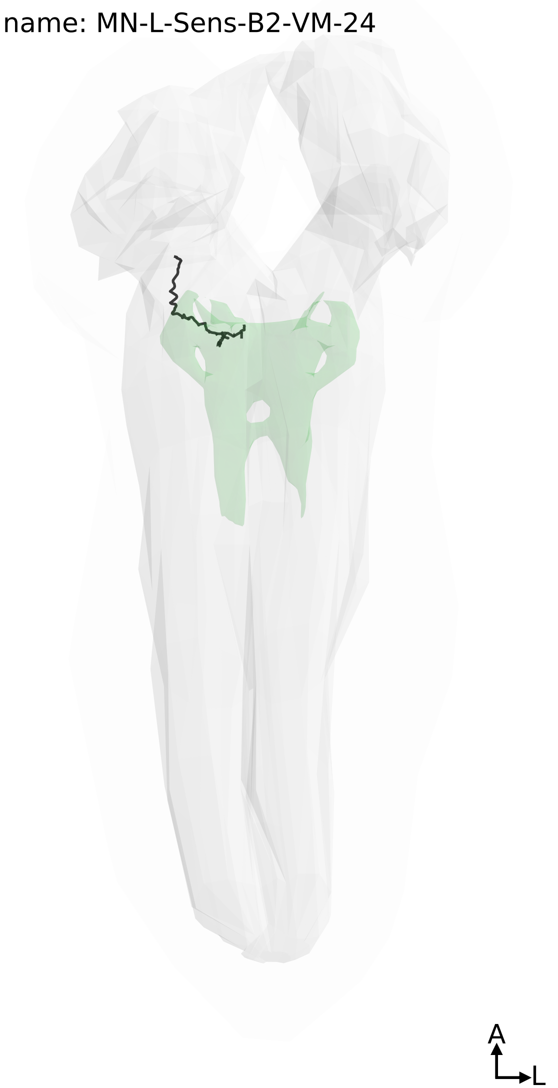

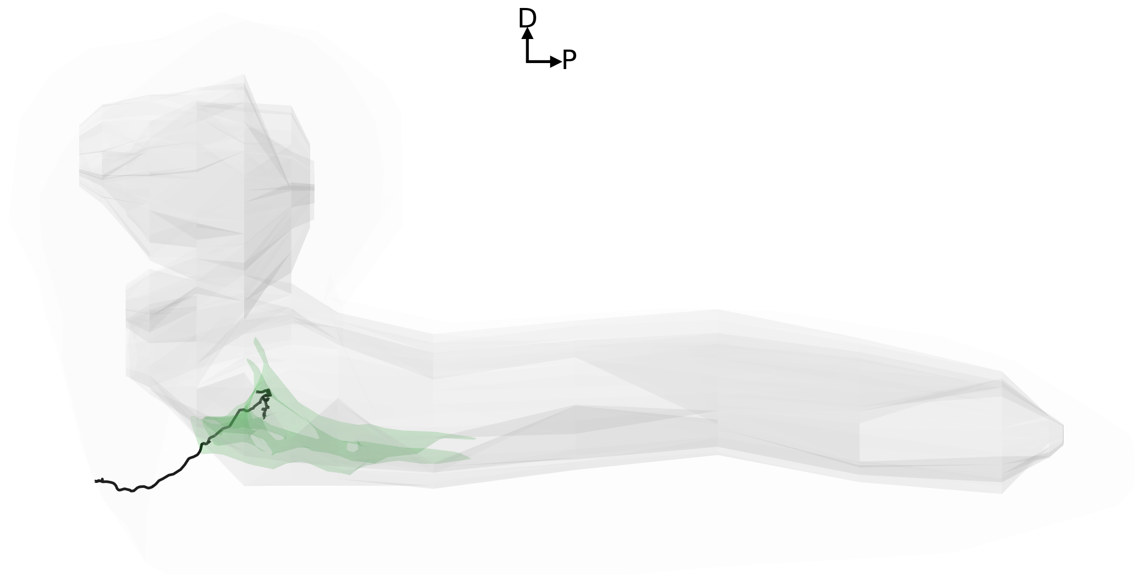

ID	*name*	SCACa	SCAVa	SCAVp	SCACal	SCACp	SCACpl	SCVM	IPCs	DMS	DH44	Se0ens	Se0ph	PMN LR	MN motor neurons	PaN motor neurons	olfactory PNs	gustatory PNs	multiglomerular PNs	unknown PNs	thermo PNs	visual PNs
19180425	MN-L-Sens-B2-VM-24	0	0	0	0	0	0	4	0	0	0	0	0	0	0	0	0	0	0	0	0	0

ID: 15727304
name: MN-L-Sens-B3-VM-04

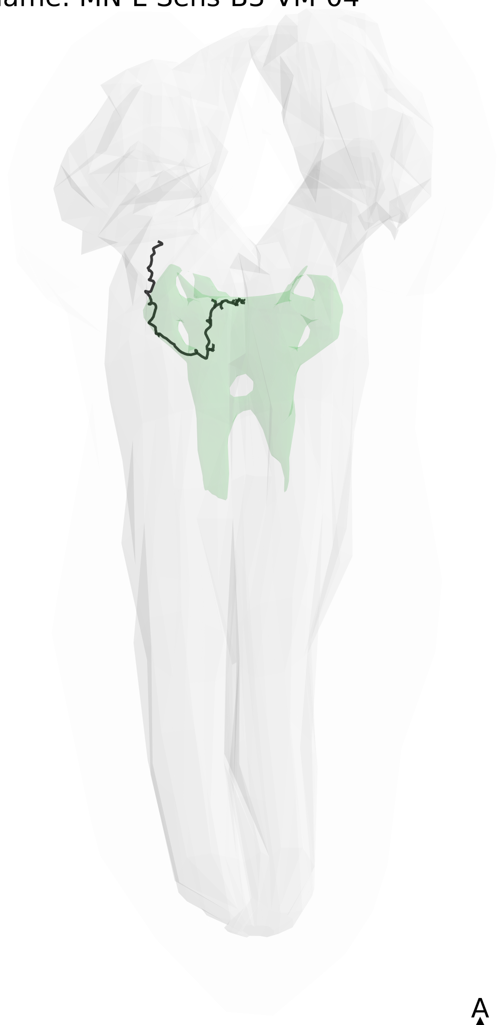

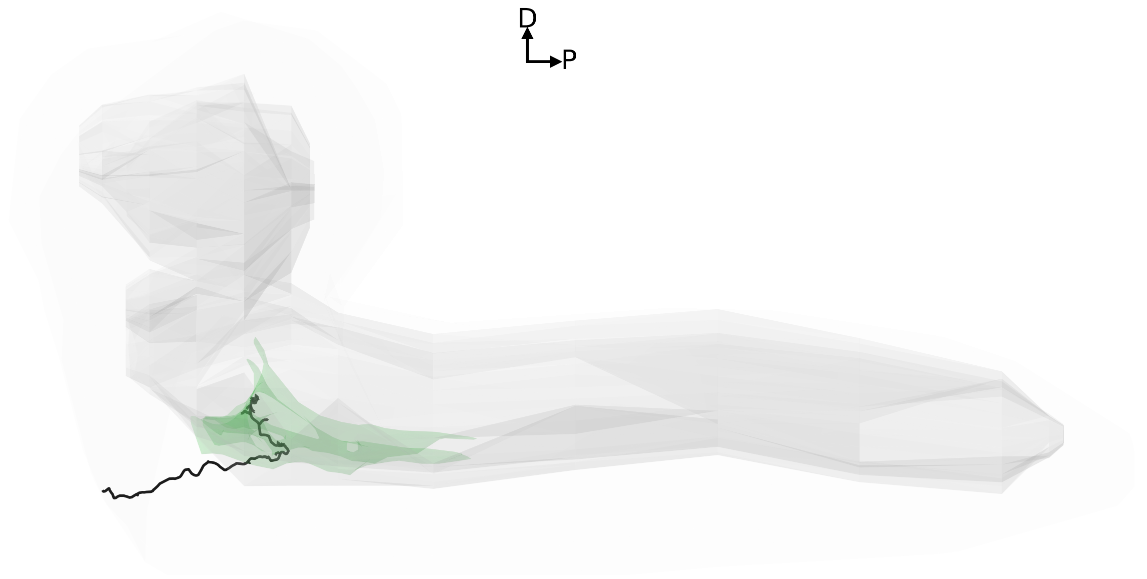

ID	name	SCACa	SCAVa	SCAVp	SCACal	SCACp	SCACpl	SCVM	IPCs	DMS	DH44	Se0ens	Se0ph	PMN LR	MN motor neurons	PaN motor neurons	olfactory PNs	gustatory PNs	multiglomerular PNs	unknown PNs	thermo PNs	visual PNs
15727304	MN-L-Sens-B3-VM-04	0	0	0	0	0	0	0	0	0	0	0	0	0	0	0	0	0	0	0	0	0

ID: 15730645
name: MN-L-Sens-B3-VM-05

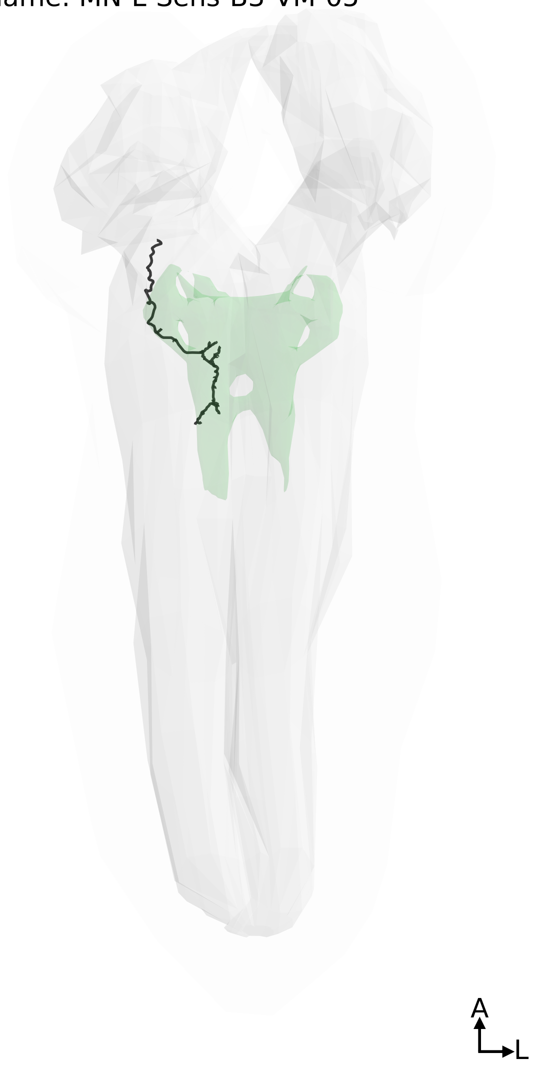

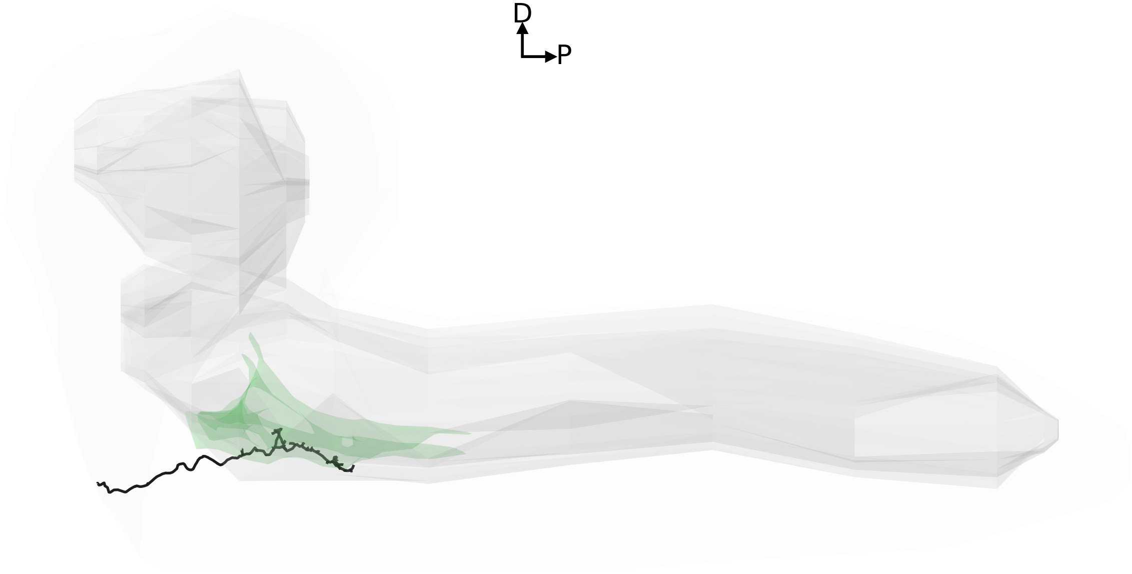

ID	*name*	SCACa	SCAVa	SCAVp	SCACal	SCACp	SCACpl	SCVM	IPCs	DMS	DH44	Se0ens	Se0ph	PMN LR	MN motor neurons	PaN motor neurons	olfactory PNs	gustatory PNs	multiglomerular PNs	unknown PNs	thermo PNs	visual PNs
15730645	MN-L-Sens-B3-VM-05	0	0	0	0	0	0	4	0	0	0	0	0	0	0	0	0	0	0	0	0	0

ID: 15724520
name: MN-L-Sens-B3-VM-06

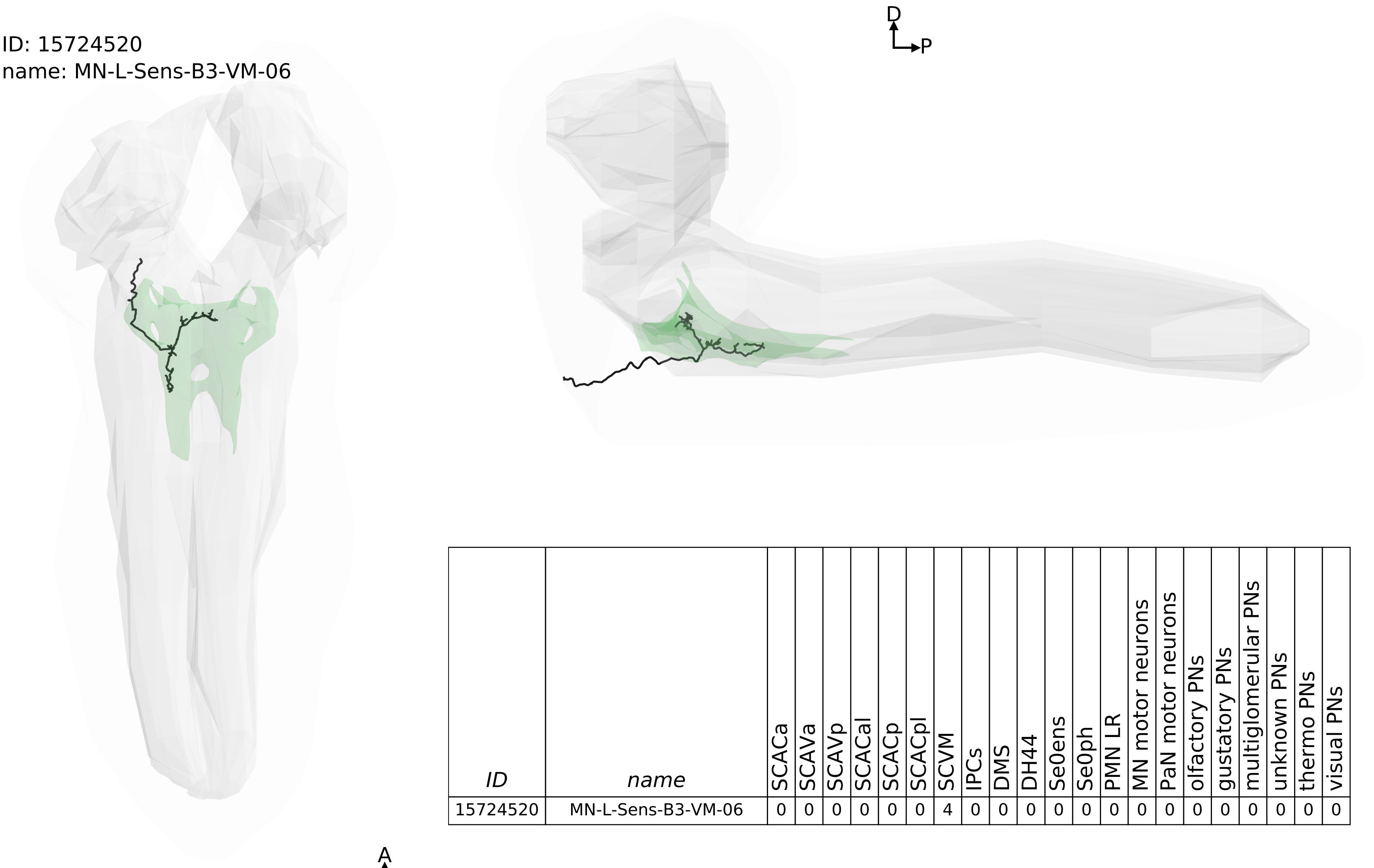

ID	name	SCACa	SCAVa	SCAVp	SCACal	SCACp	SCACpl	SCVM	IPCs	DMS	DH44	Se0ens	Se0ph	PMN LR	MN motor neurons	PaN motor neurons	olfactory PNs	gustatory PNs	multiglomerular PNs	unknown PNs	thermo PNs	visual PNs
15724520	MN-L-Sens-B3-VM-06	0	0	0	0	0	0	4	0	0	0	0	0	0	0	0	0	0	0	0	0	0

ID: 15725673
name: MN-L-Sens-B3-VM-07

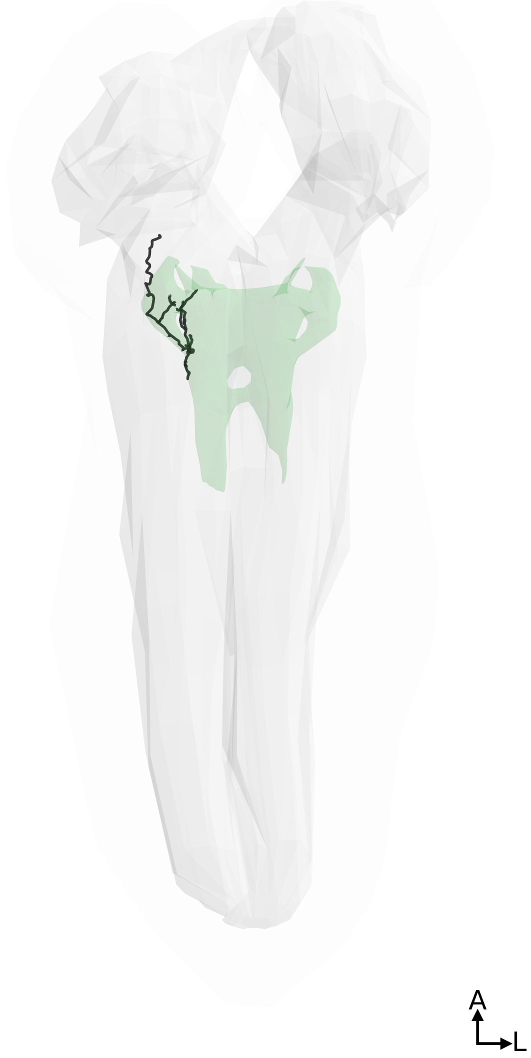

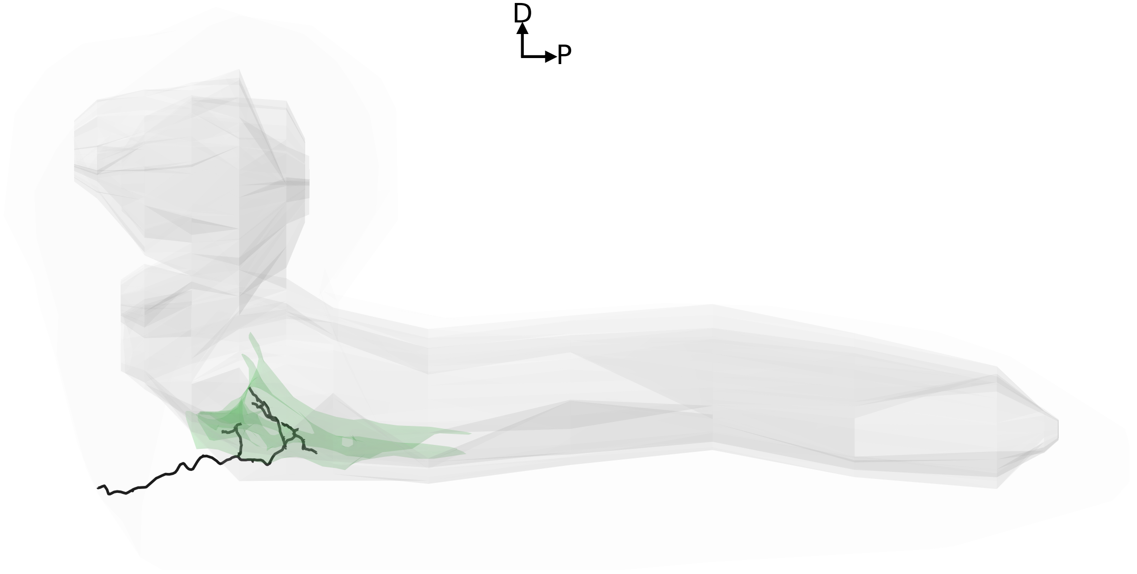

ID	*name*	SCACa	SCAVa	SCAVp	SCACal	SCACp	SCACpl	SCVM	IPCs	DMS	DH44	Se0ens	Se0ph	PMN LR	MN motor neurons	PaN motor neurons	olfactory PNs	gustatory PNs	multiglomerular PNs	unknown PNs	thermo PNs	visual PNs
15725673	MN-L-Sens-B3-VM-07	0	0	0	0	0	0	3	0	0	0	0	0	0	0	0	0	0	0	0	0	0

ID: 15724636
name: MN-L-Sens-B3-VM-08

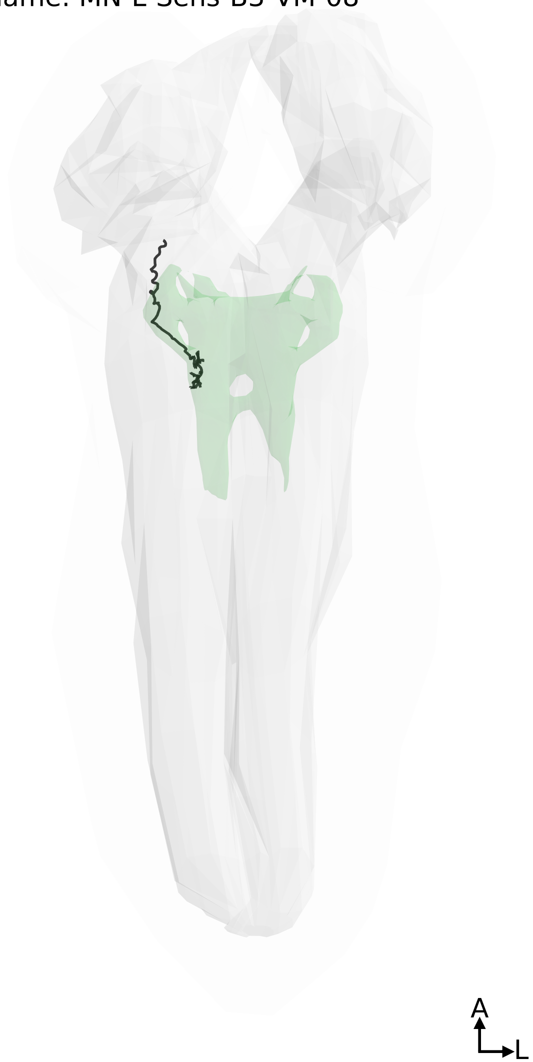

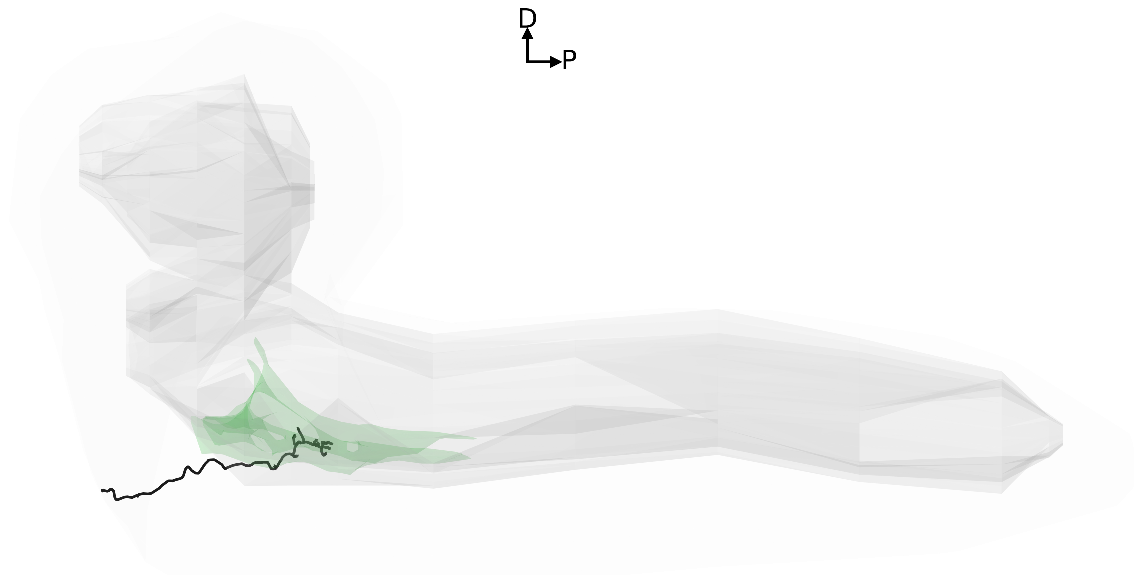

ID	name	SCACa	SCAVa	SCAVp	SCACal	SCACp	SCACpl	SCVM	IPCs	DMS	DH44	Se0ens	Se0ph	PMN LR	MN motor neurons	PaN motor neurons	olfactory PNs	gustatory PNs	multiglomerular PNs	unknown PNs	thermo PNs	visual PNs
15724636	MN-L-Sens-B3-VM-08	0	0	0	0	0	0	9	0	0	0	0	0	0	0	0	0	0	0	0	0	0

ID: 15725545
name: MN-L-Sens-B3-VM-09

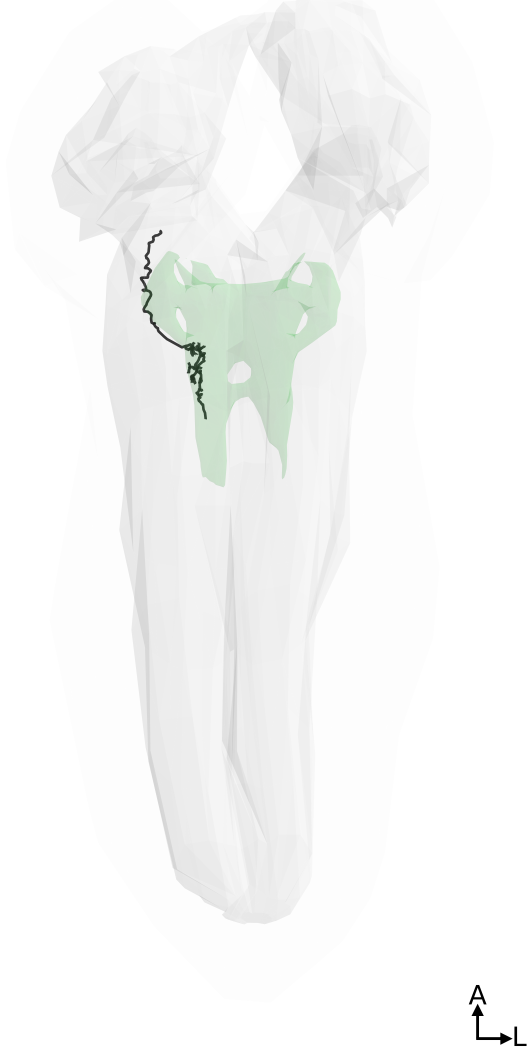

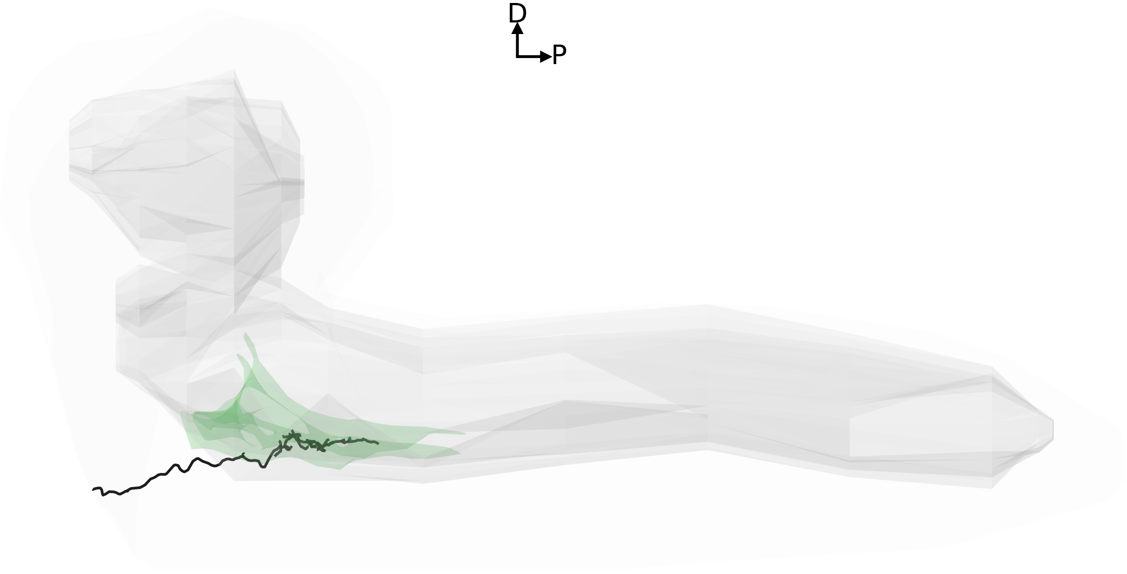

ID	name	SCACa	SCAVa	SCAVp	SCACal	SCACp	SCACpl	SCVM	IPCs	DMS	DH44	Se0ens	Se0ph	PMN LR	MN motor neurons	PaN motor neurons	olfactory PNs	gustatory PNs	multiglomerular PNs	unknown PNs	thermo PNs	visual PNs
15725545	MN-L-Sens-B3-VM-09	0	0	0	0	0	0	10	0	0	0	0	0	0	0	0	0	0	0	0	0	0

ID: 15725127
name: MN-L-Sens-B3-VM-10

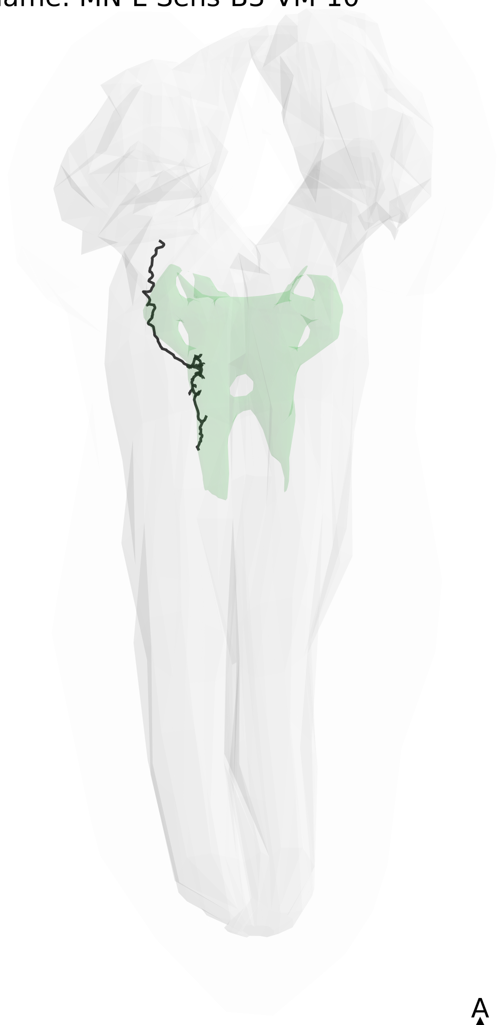

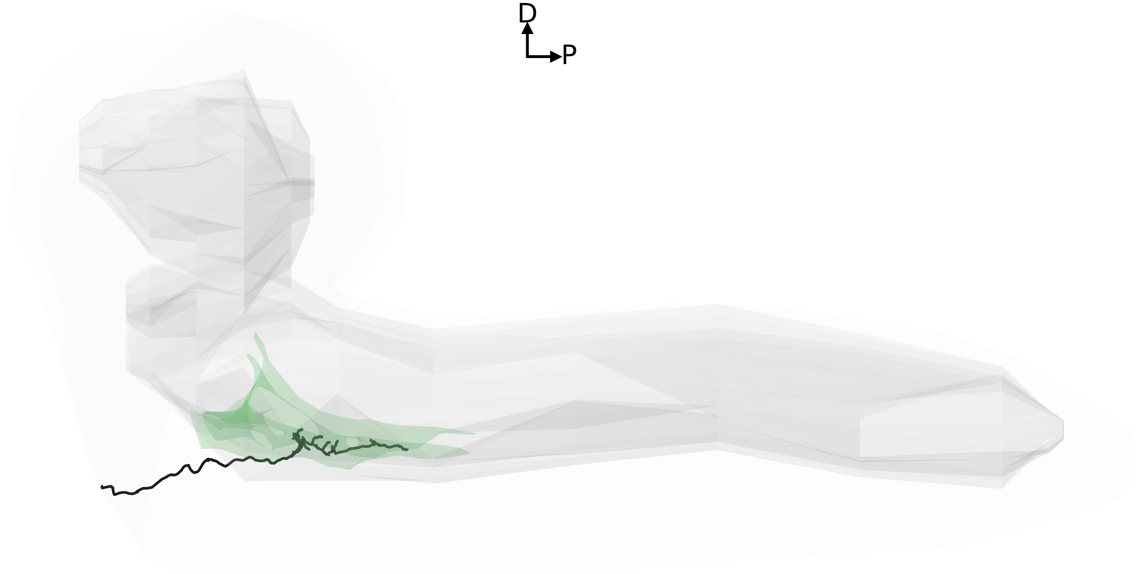

ID	name	SCACa	SCAVa	SCAVp	SCACal	SCACp	SCACpl	SCVM	IPCs	DMS	DH44	Se0ens	Se0ph	PMN LR	MN motor neurons	PaN motor neurons	olfactory PNs	gustatory PNs	multiglomerular PNs	unknown PNs	thermo PNs	visual PNs
15725127	MN-L-Sens-B3-VM-10	0	0	0	0	0	0	6	0	0	0	0	0	0	0	0	0	0	0	0	0	0

A
L

ID: 15724175
name: MN-L-Sens-B3-VM-11

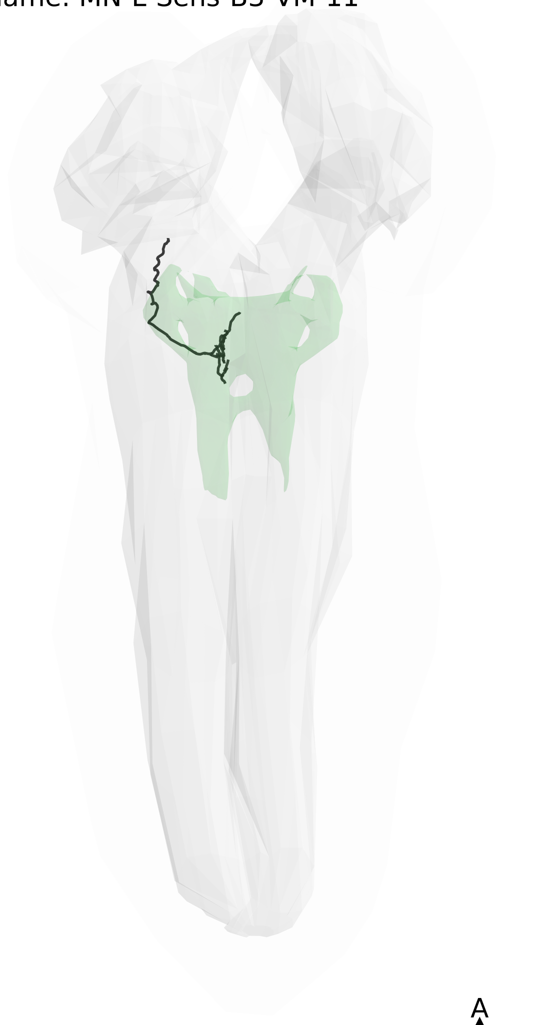

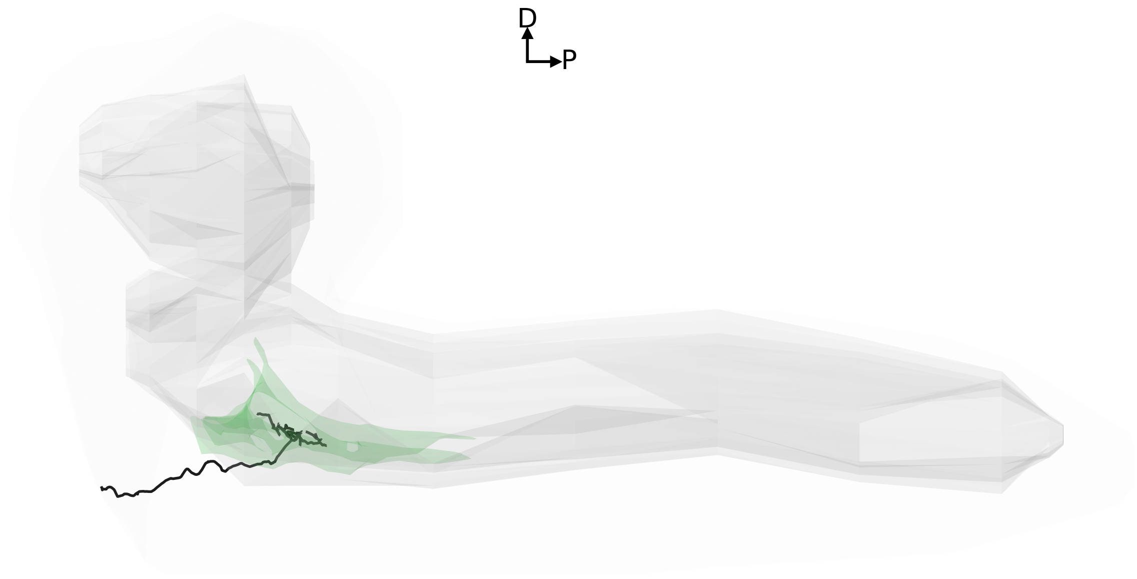

ID	*name*	SCACa	SCAVa	SCAVp	SCACal	SCACp	SCACpl	SCVM	IPCs	DMS	DH44	Se0ens	Se0ph	PMN LR	MN motor neurons	PaN motor neurons	olfactory PNs	gustatory PNs	multiglomerular PNs	unknown PNs	thermo PNs	visual PNs
15724175	MN-L-Sens-B3-VM-11	0	0	0	0	0	1	7	0	0	0	0	0	0	0	0	0	0	0	0	0	0

ID: 3036249
name: MN-L-Sens-B3-VM-12

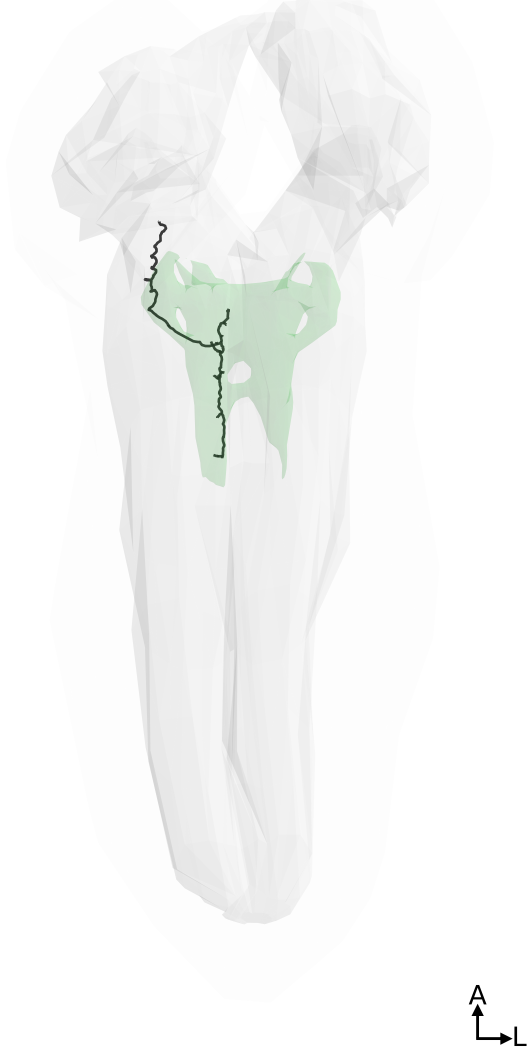

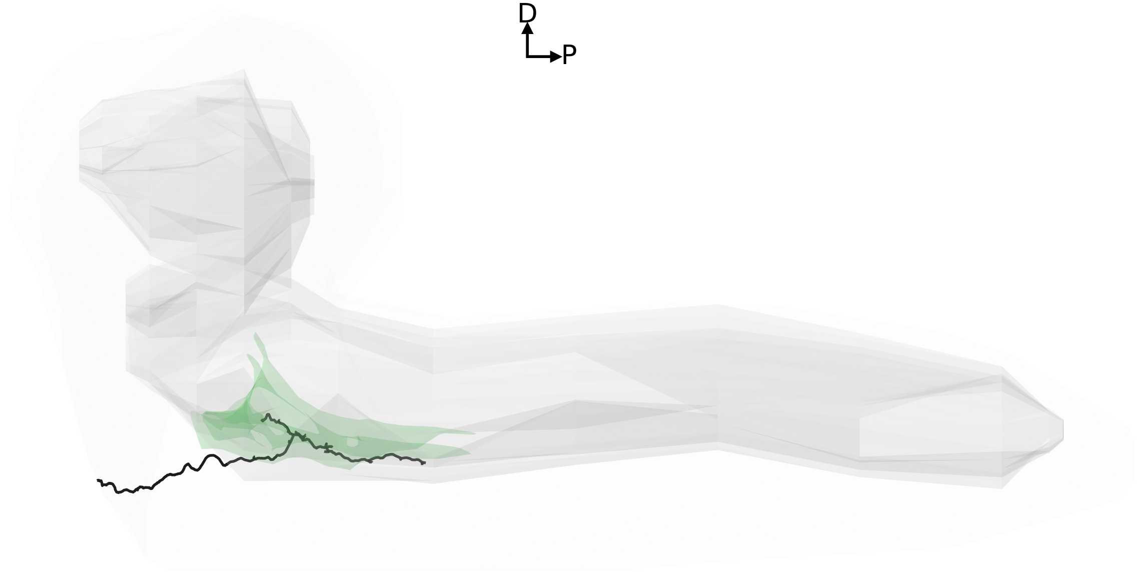

ID	*name*	SCACa	SCAVa	SCAVp	SCACal	SCACp	SCACpl	SCVM	IPCs	DMS	DH44	Se0ens	Se0ph	PMN LR	MN motor neurons	PaN motor neurons	olfactory PNs	gustatory PNs	multiglomerular PNs	unknown PNs	thermo PNs	visual PNs
3036249	MN-L-Sens-B3-VM-12	0	0	0	0	0	0	4	0	0	0	0	0	0	0	0	0	0	0	0	0	0

ID: 15728017
name: MN-L-Sens-B3-VM-13

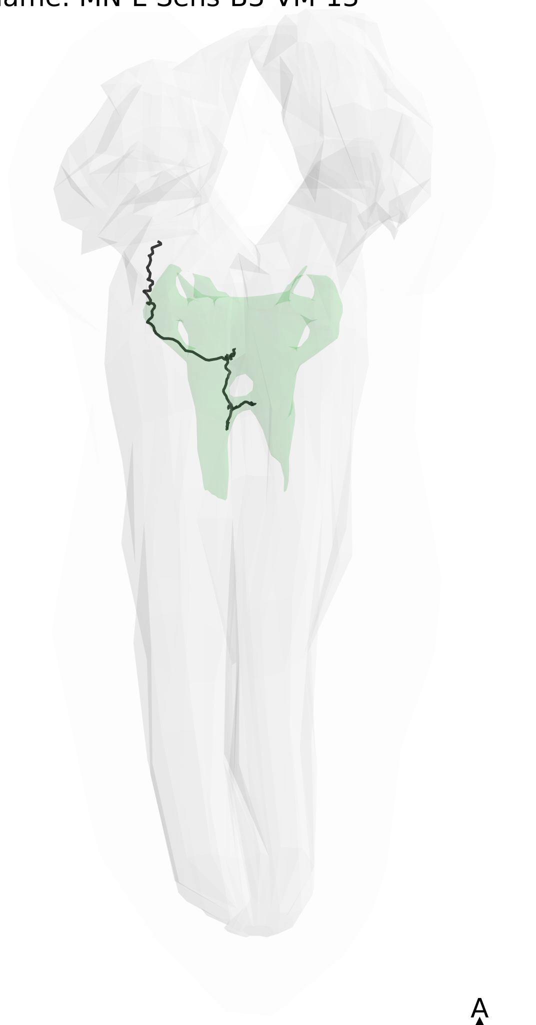

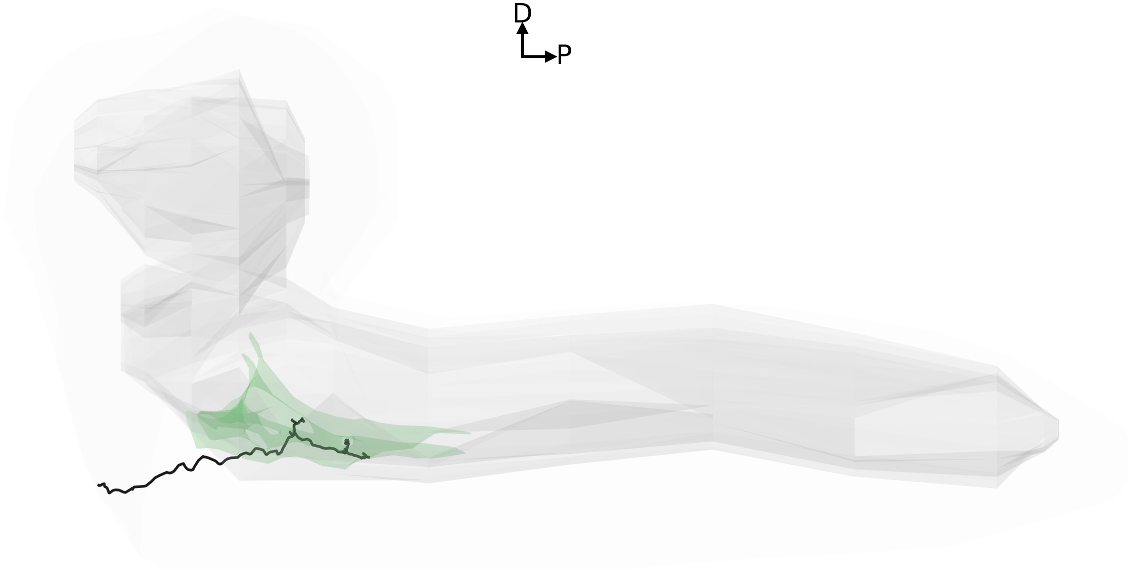

ID	*name*	SCACa	SCAVa	SCAVp	SCACal	SCACp	SCACpl	SCVM	IPCs	DMS	DH44	Se0ens	Se0ph	PMN LR	MN motor neurons	PaN motor neurons	olfactory PNs	gustatory PNs	multiglomerular PNs	unknown PNs	thermo PNs	visual PNs
15728017	MN-L-Sens-B3-VM-13	0	0	0	0	0	0	0	0	0	0	0	0	0	0	0	0	0	0	0	0	0

ID: 3050645
name: MN-L-Sens-B3-VM-14

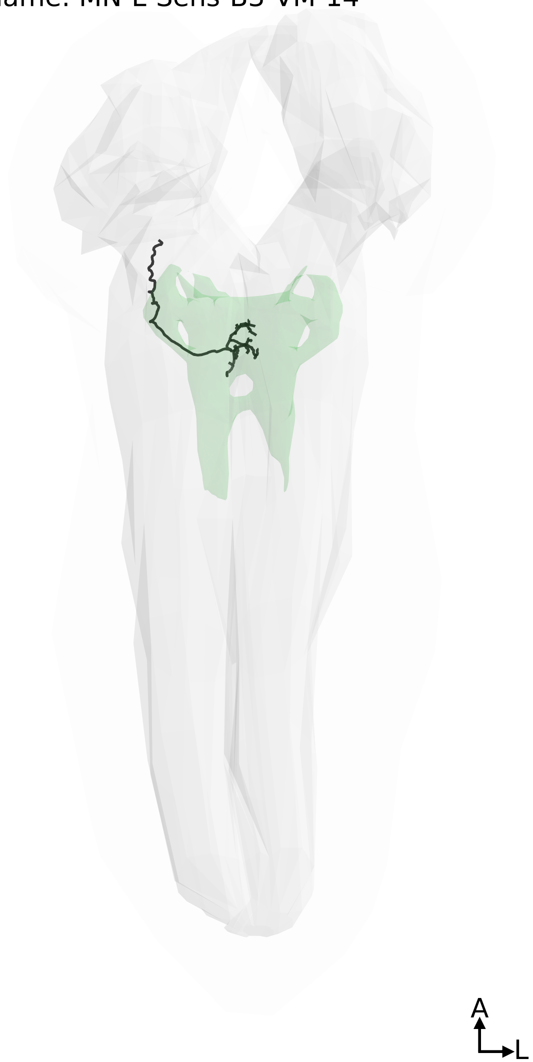

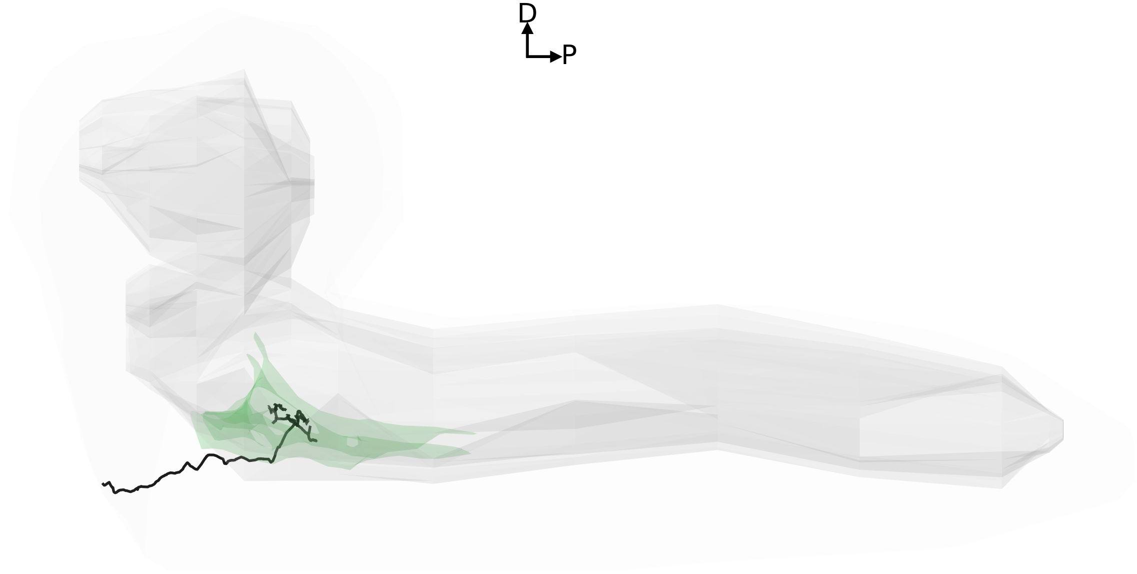

ID	*name*	SCACa	SCAVa	SCAVp	SCACal	SCACp	SCACpl	SCVM	IPCs	DMS	DH44	Se0ens	Se0ph	PMN LR	MN motor neurons	PaN motor neurons	olfactory PNs	gustatory PNs	multiglomerular PNs	unknown PNs	thermo PNs	visual PNs
3050645	MN-L-Sens-B3-VM-14	0	0	0	0	0	0	9	0	0	0	0	0	0	0	0	0	0	0	0	0	0

ID: 15594123
name: MN-R-Sens-B1-VM-02

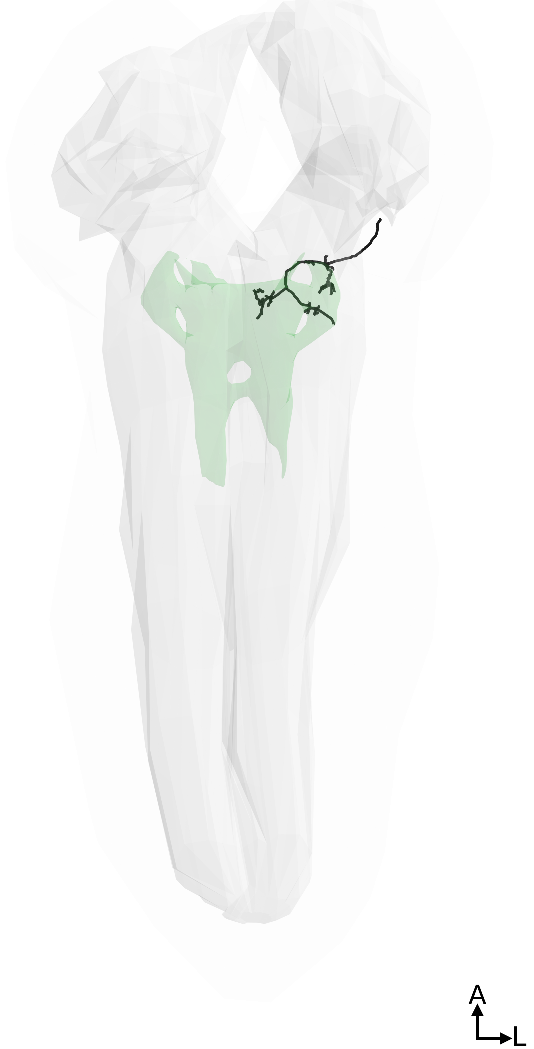

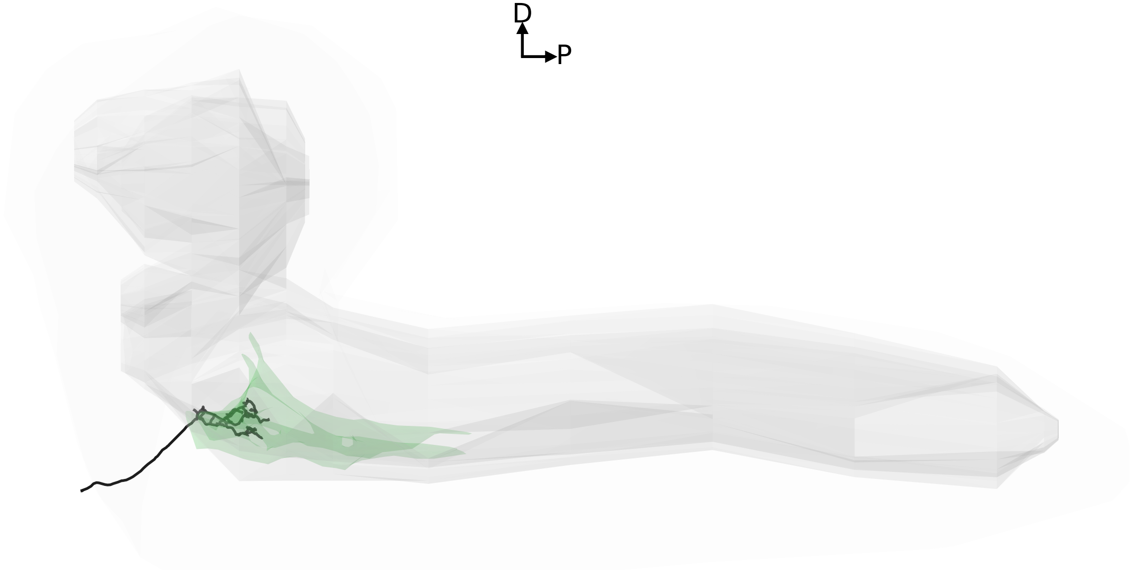

ID	*name*	SCACa	SCAVa	SCAVp	SCACal	SCACp	SCACpl	SCVM	IPCs	DMS	DH44	Se0ens	Se0ph	PMN LR	MN motor neurons	PaN motor neurons	olfactory PNs	gustatory PNs	multiglomerular PNs	unknown PNs	thermo PNs	visual PNs
15594123	MN-R-Sens-B1-VM-02	0	0	0	0	0	0	2	0	0	0	0	0	0	0	0	0	0	0	0	0	0

ID: 15638742
name: MN-R-Sens-B1-VM-03

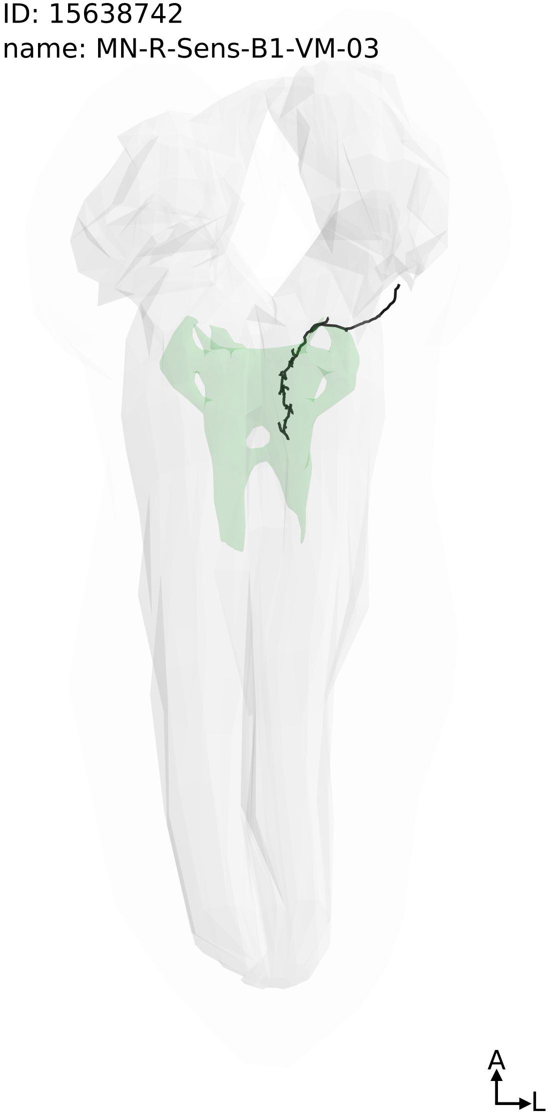

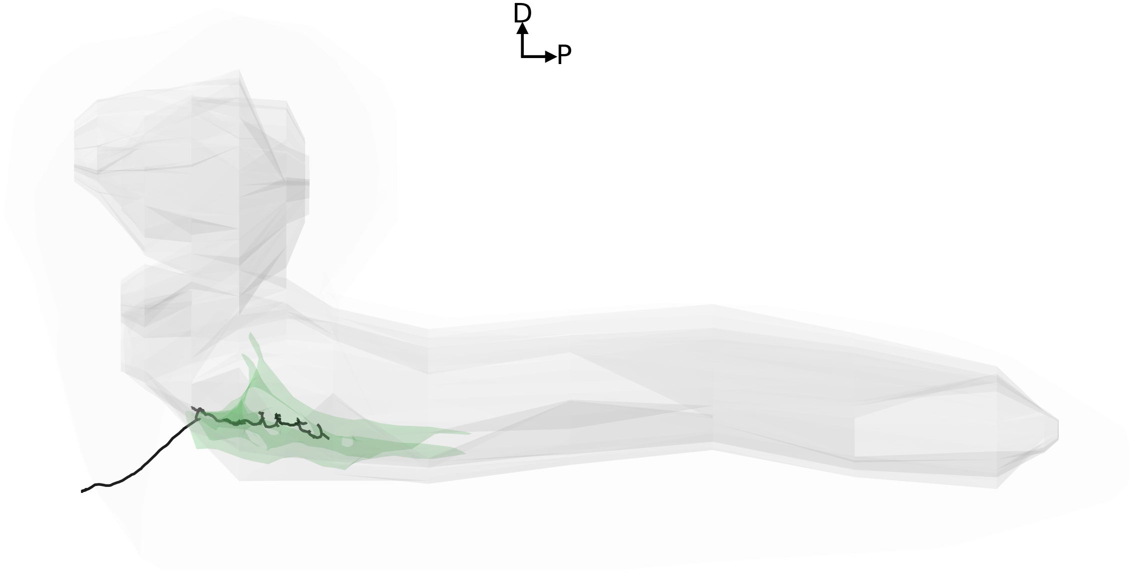

ID	name	SCACa	SCAVa	SCAVp	SCACal	SCACp	SCACpl	SCVM	IPCs	DMS	DH44	Se0ens	Se0ph	PMN LR	MN motor neurons	PaN motor neurons	olfactory PNs	gustatory PNs	multiglomerular PNs	unknown PNs	thermo PNs	visual PNs
15638742	MN-R-Sens-B1-VM-03	0	0	0	0	0	0	7	0	0	0	0	0	0	0	0	0	0	0	0	0	0

ID: 15743905
name: MN-R-Sens-B1-VM-04

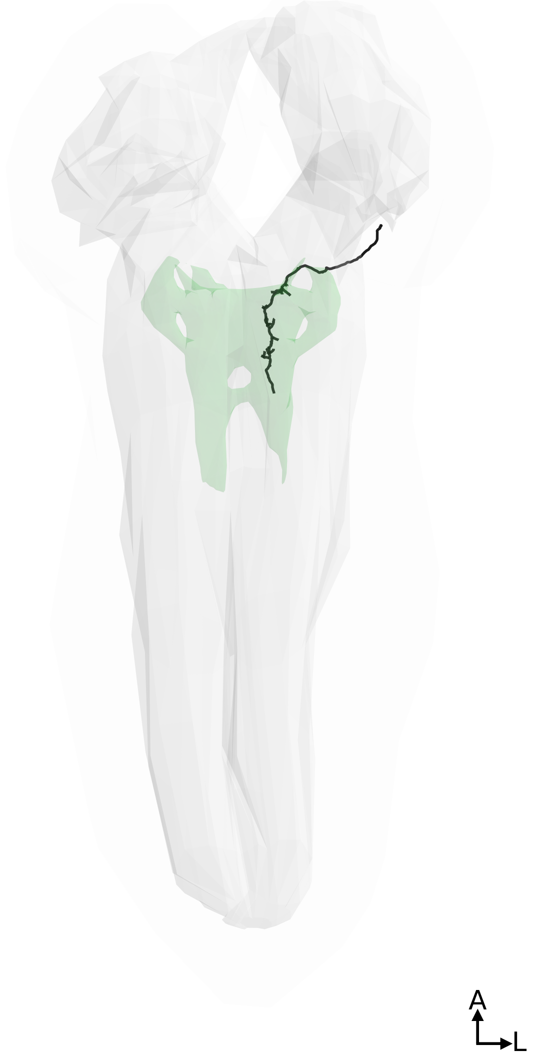

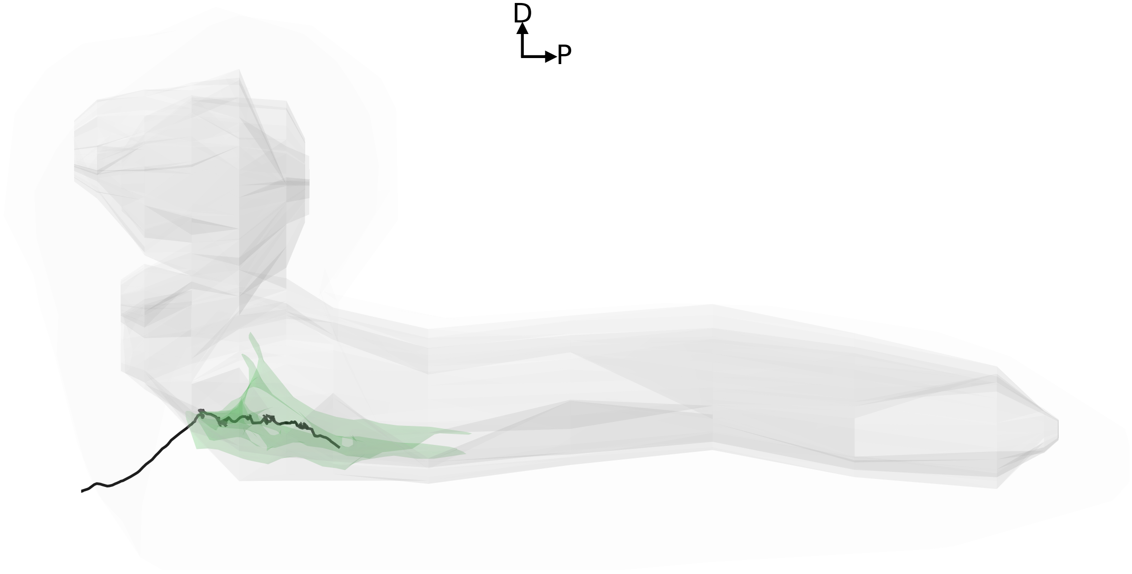

ID	*name*	SCACa	SCAVa	SCAVp	SCACal	SCACp	SCACpl	SCVM	IPCs	DMS	DH44	Se0ens	Se0ph	PMN LR	MN motor neurons	PaN motor neurons	olfactory PNs	gustatory PNs	multiglomerular PNs	unknown PNs	thermo PNs	visual PNs
15743905	MN-R-Sens-B1-VM-04	0	0	0	0	0	0	8	0	0	0	0	0	0	0	0	0	0	0	0	0	0

ID: 15671481
name: MN-R-Sens-B1-VM-05

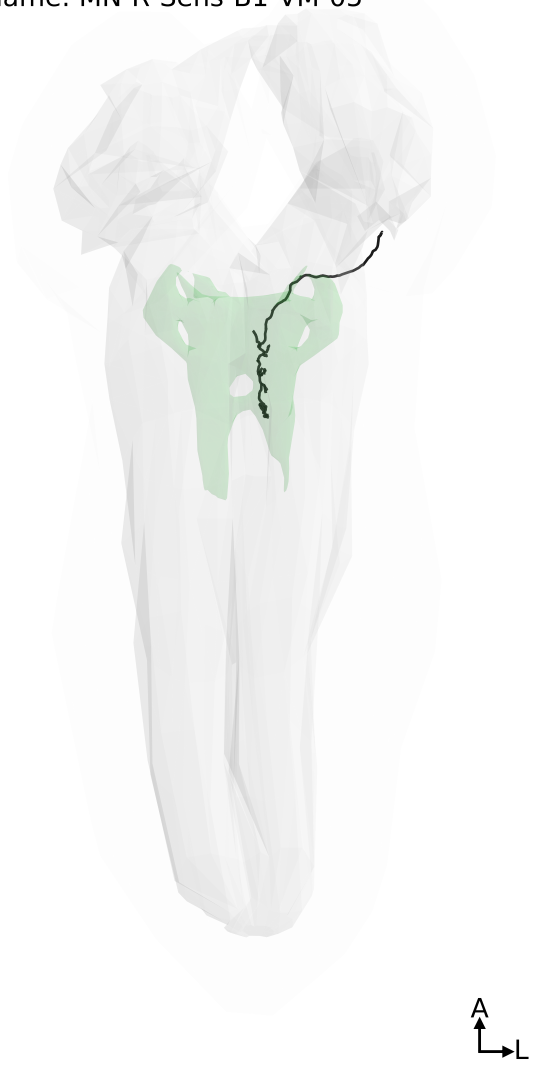

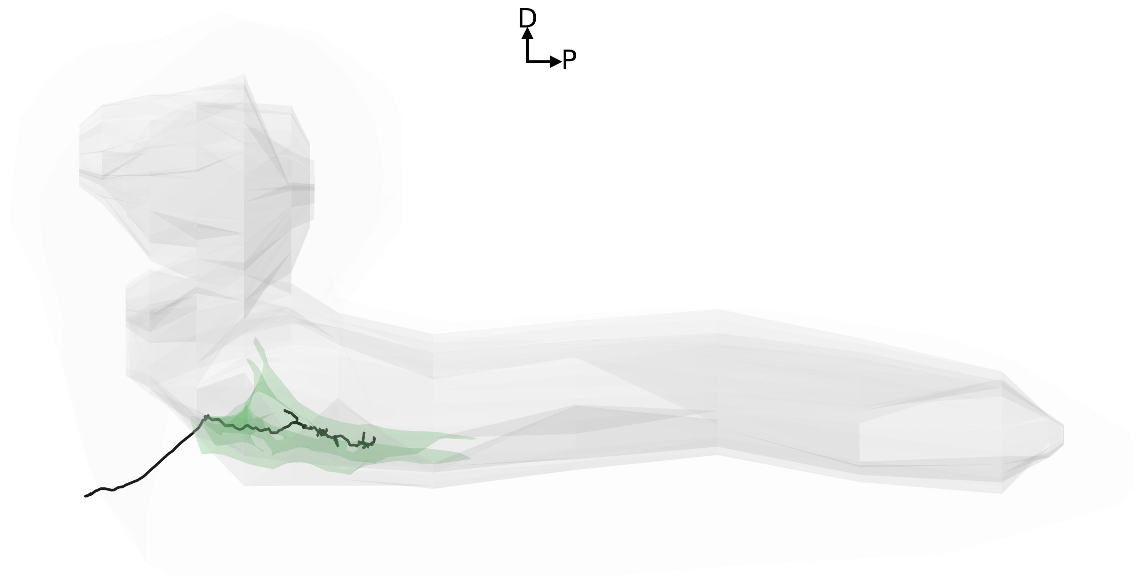

ID	name	SCACa	SCAVa	SCAVp	SCACal	SCACp	SCACpl	SCVM	IPCs	DMS	DH44	Se0ens	Se0ph	PMN LR	MN motor neurons	PaN motor neurons	olfactory PNs	gustatory PNs	multiglomerular PNs	unknown PNs	thermo PNs	visual PNs
15671481	MN-R-Sens-B1-VM-05	0	0	0	0	0	0	11	0	0	0	0	0	0	0	0	0	0	0	0	0	0

ID: 15653927
name: MN-R-Sens-B2-VM-01

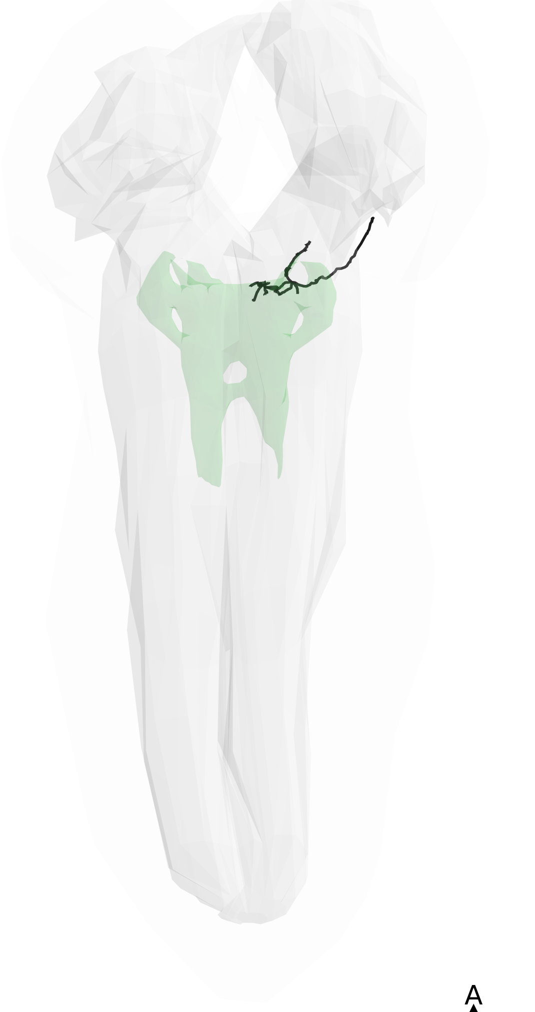

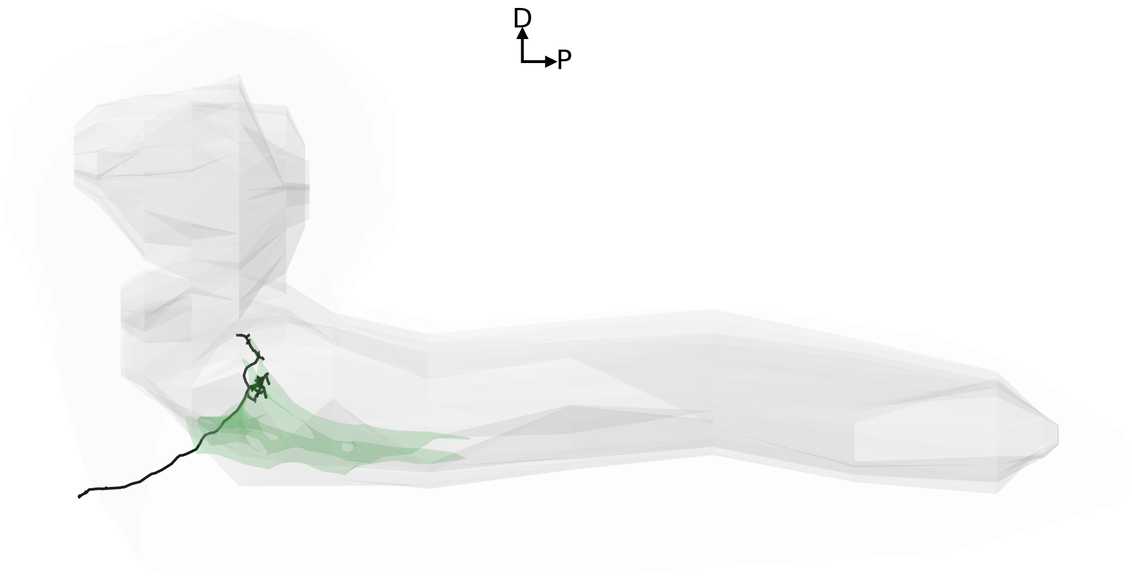

ID	*name*	SCACa	SCAVa	SCAVp	SCACal	SCACp	SCACpl	SCVM	IPCs	DMS	DH44	Se0ens	Se0ph	PMN LR	MN motor neurons	PaN motor neurons	olfactory PNs	gustatory PNs	multiglomerular PNs	unknown PNs	thermo PNs	visual PNs
15653927	MN-R-Sens-B2-VM-01	0	0	0	0	0	0	4	0	0	0	0	0	0	0	0	0	0	0	0	0	0

ID: 15611791
name: MN-R-Sens-B2-VM-02

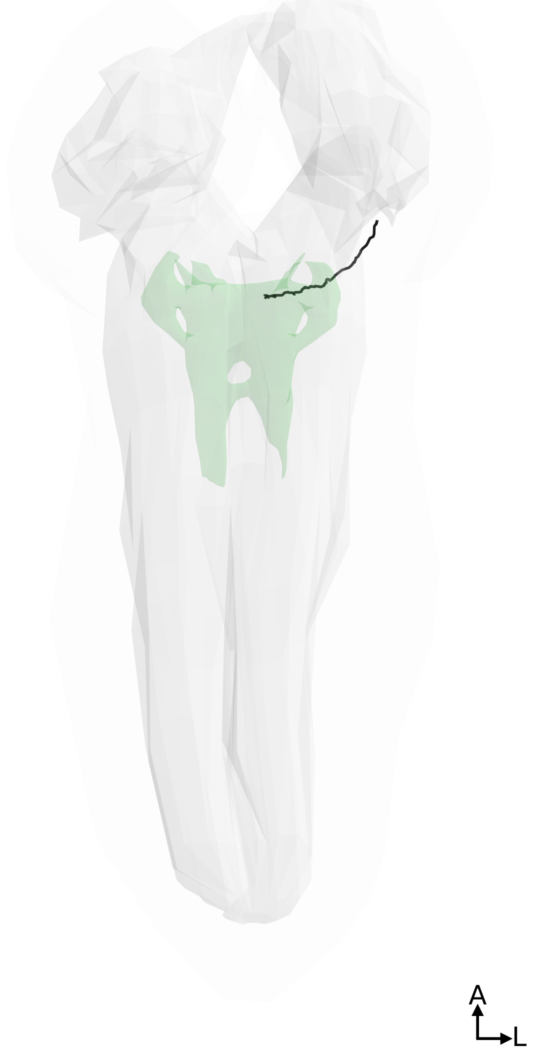

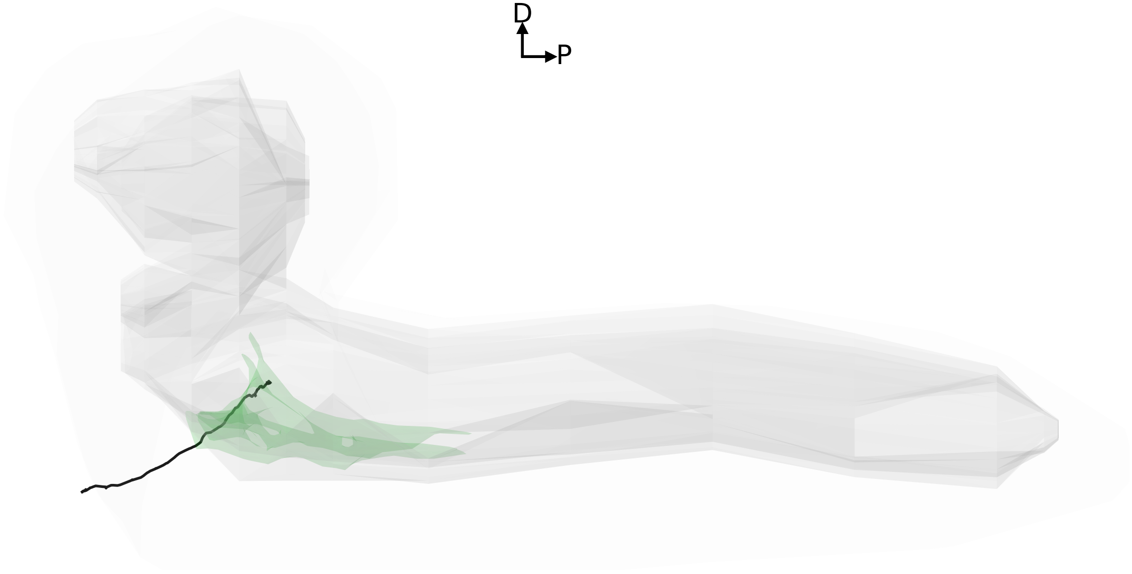

ID	*name*	SCACa	SCAVa	SCAVp	SCACal	SCACp	SCACpl	SCVM	IPCs	DMS	DH44	Se0ens	Se0ph	PMN LR	MN motor neurons	PaN motor neurons	olfactory PNs	gustatory PNs	multiglomerular PNs	unknown PNs	thermo PNs	visual PNs
15611791	MN-R-Sens-B2-VM-02	0	0	0	0	0	0	2	0	0	0	0	0	0	0	0	0	0	0	0	0	0

ID: 15629355
name: MN-R-Sens-B2-VM-03

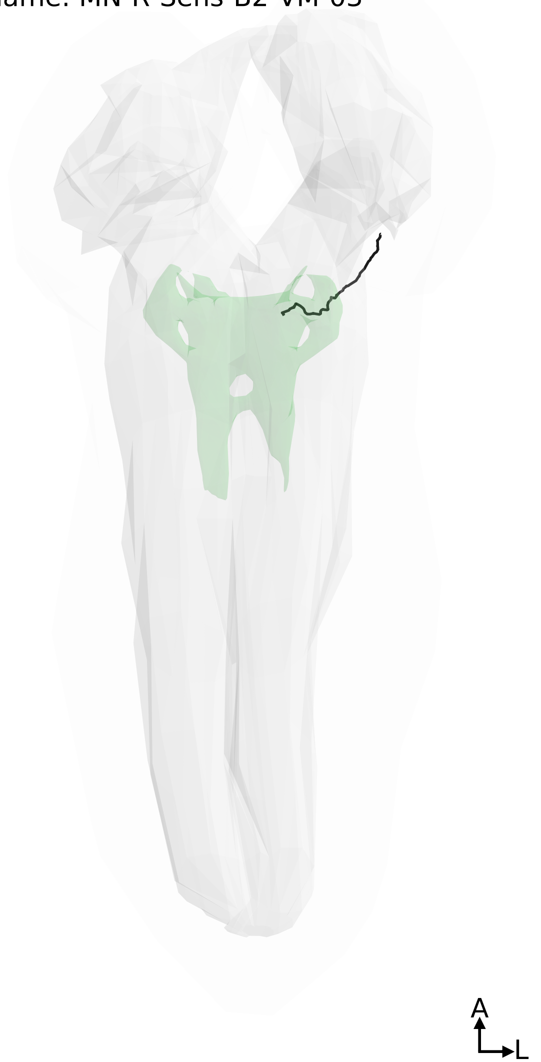

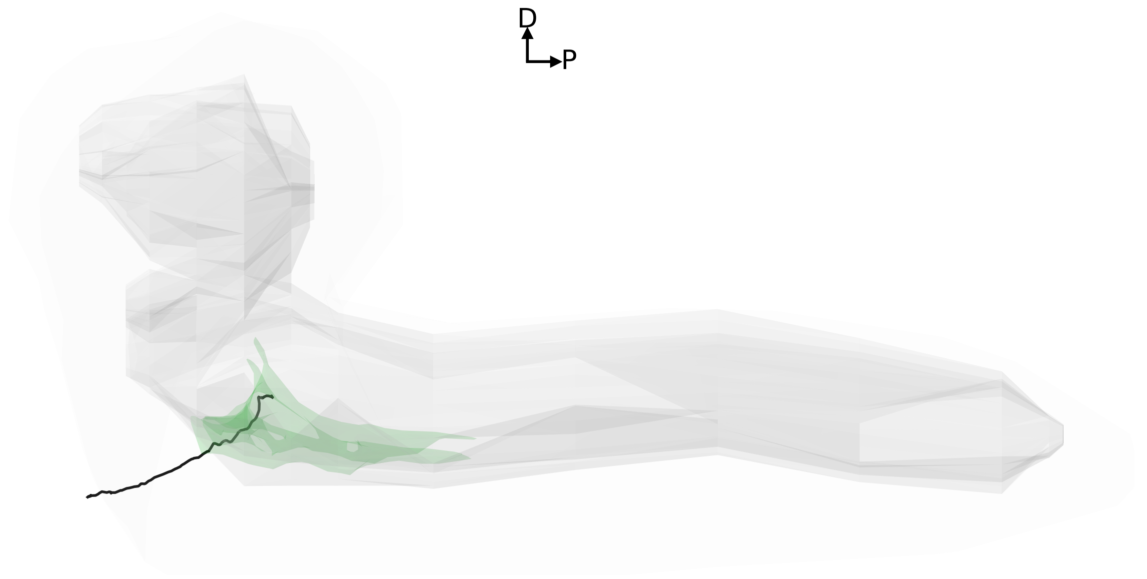

ID	*name*	SCACa	SCAVa	SCAVp	SCACal	SCACp	SCACpl	SCVM	IPCs	DMS	DH44	Se0ens	Se0ph	PMN LR	MN motor neurons	PaN motor neurons	olfactory PNs	gustatory PNs	multiglomerular PNs	unknown PNs	thermo PNs	visual PNs
15629355	MN-R-Sens-B2-VM-03	0	0	0	0	0	0	0	0	0	0	0	0	0	0	0	0	0	0	0	0	0

ID: 15606918
name: MN-R-Sens-B2-VM-04

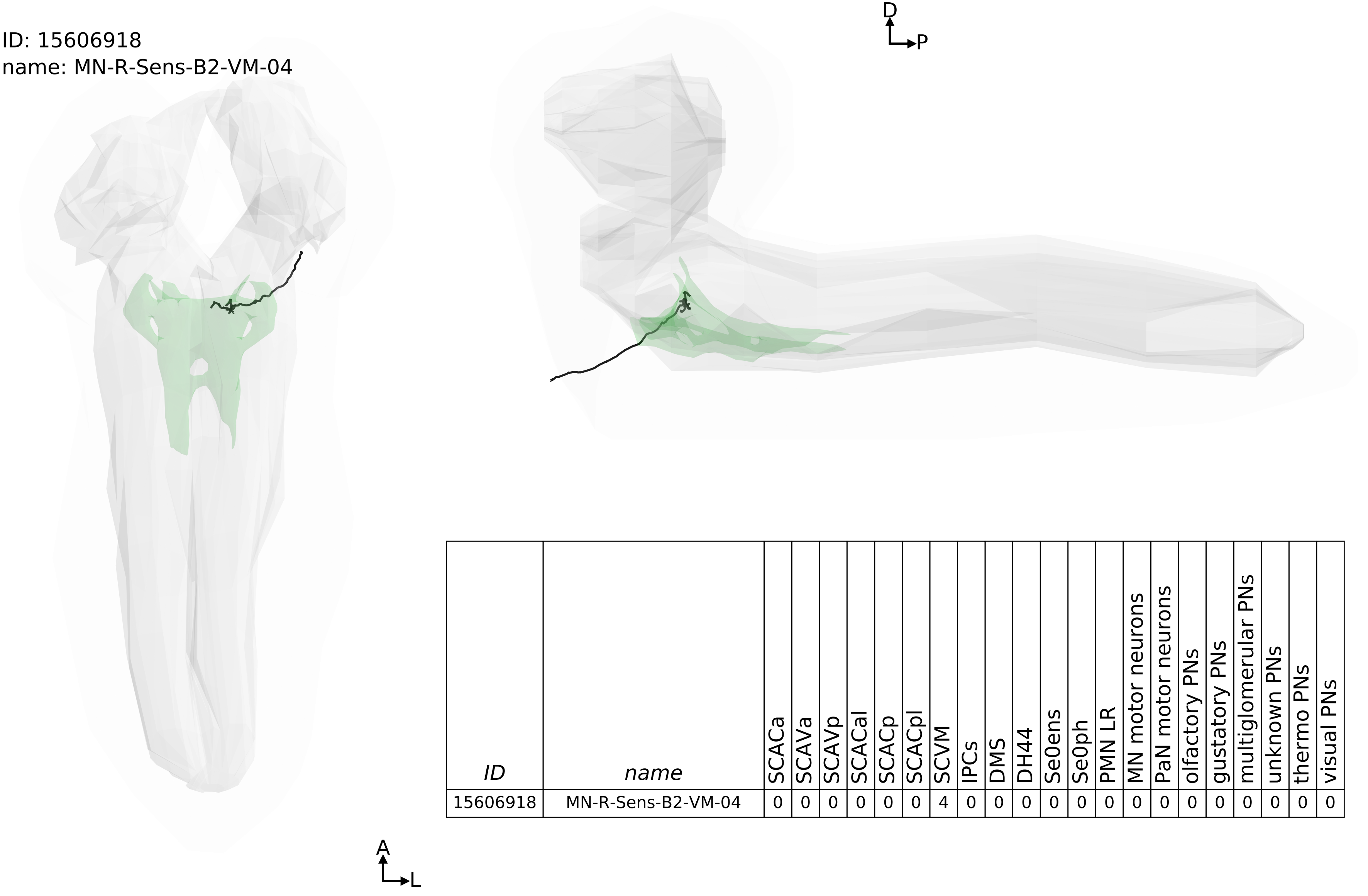

ID	name	SCACa	SCAVa	SCAVp	SCACal	SCACp	SCACpl	SCVM	IPCs	DMS	DH44	Se0ens	Se0ph	PMN LR	MN motor neurons	PaN motor neurons	olfactory PNs	gustatory PNs	multiglomerular PNs	unknown PNs	thermo PNs	visual PNs
15606918	MN-R-Sens-B2-VM-04	0	0	0	0	0	0	4	0	0	0	0	0	0	0	0	0	0	0	0	0	0

ID: 15656435
name: MN-R-Sens-B2-VM-05

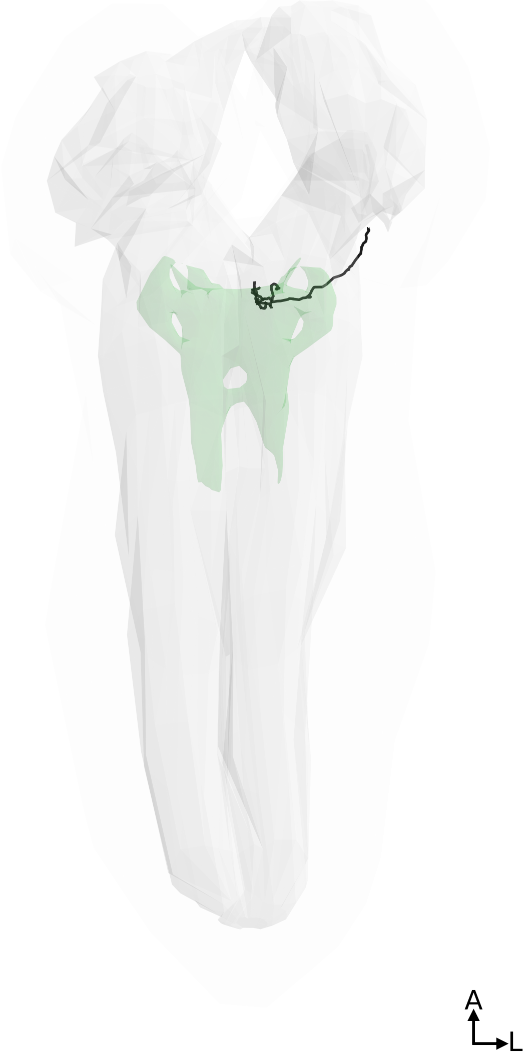

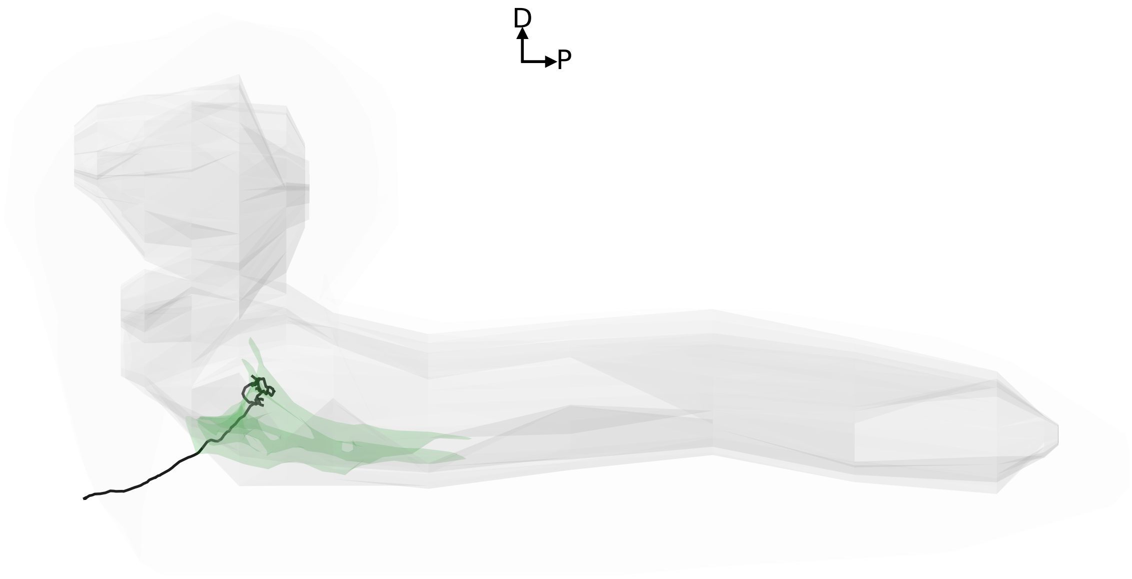

ID	*name*	SCACa	SCAVa	SCAVp	SCACal	SCACp	SCACpl	SCVM	IPCs	DMS	DH44	Se0ens	Se0ph	PMN LR	MN motor neurons	PaN motor neurons	olfactory PNs	gustatory PNs	multiglomerular PNs	unknown PNs	thermo PNs	visual PNs
15656435	MN-R-Sens-B2-VM-05	0	0	0	0	0	1	3	0	0	0	0	0	0	0	0	0	0	0	0	0	0

ID: 15617459
name: MN-R-Sens-B2-VM-06

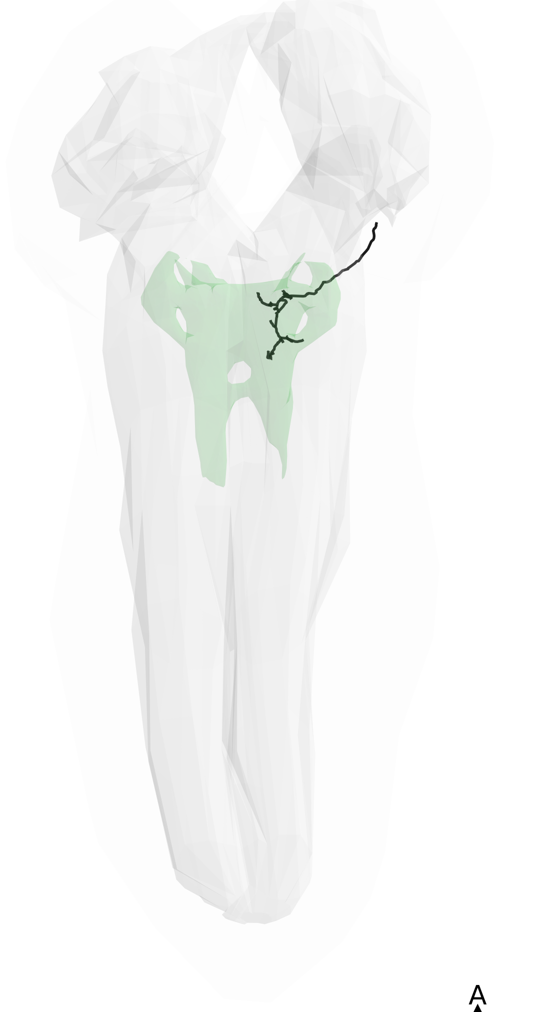

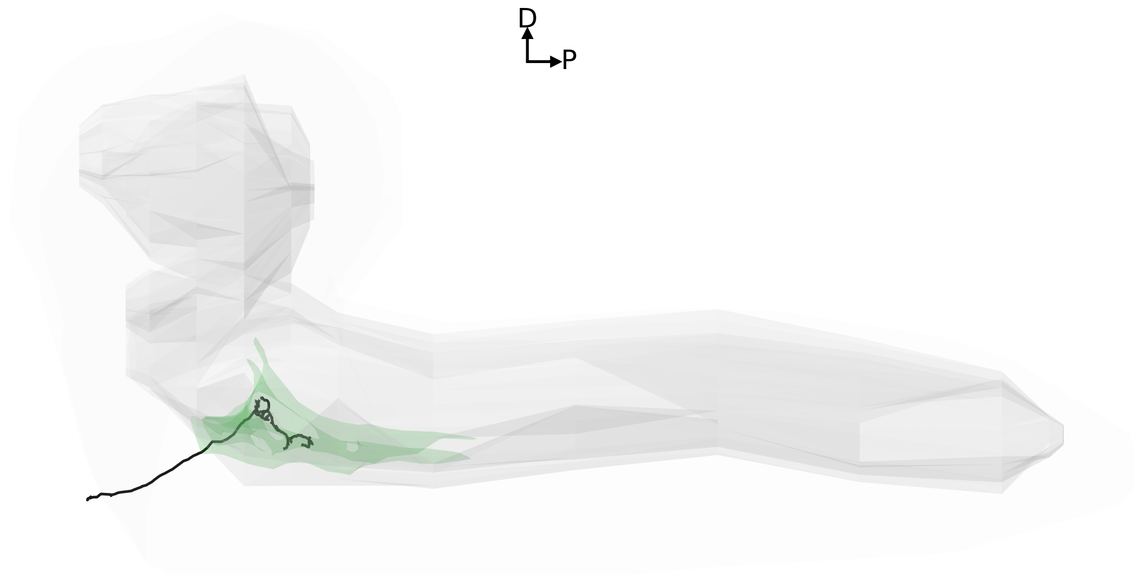

ID	name	SCACa	SCAVa	SCAVp	SCACal	SCACp	SCACpl	SCVM	IPCs	DMS	DH44	Se0ens	Se0ph	PMN LR	MN motor neurons	PaN motor neurons	olfactory PNs	gustatory PNs	multiglomerular PNs	unknown PNs	thermo PNs	visual PNs
15617459	MN-R-Sens-B2-VM-06	0	0	0	0	0	0	5	0	0	0	0	0	0	0	0	0	0	0	0	0	0

ID: 15607180
name: MN-R-Sens-B2-VM-07

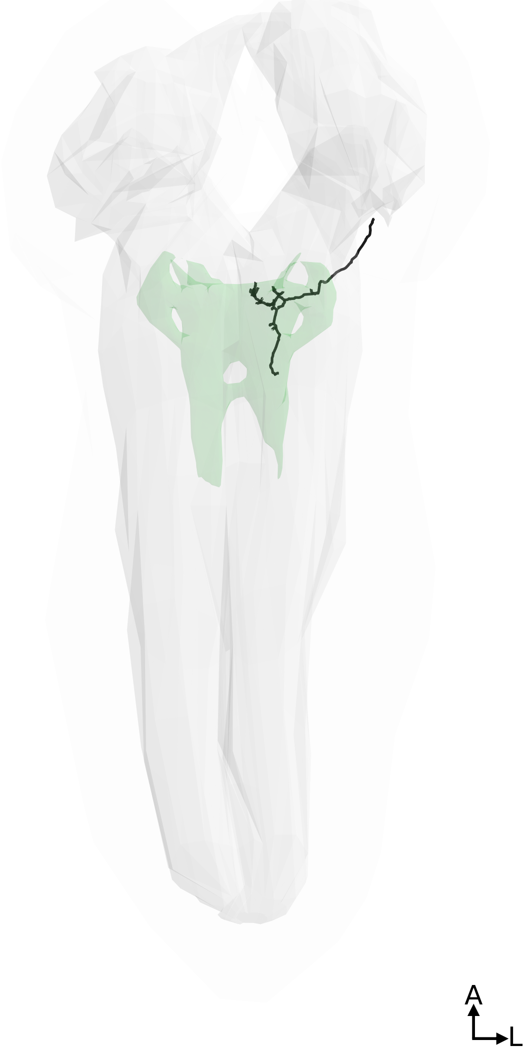

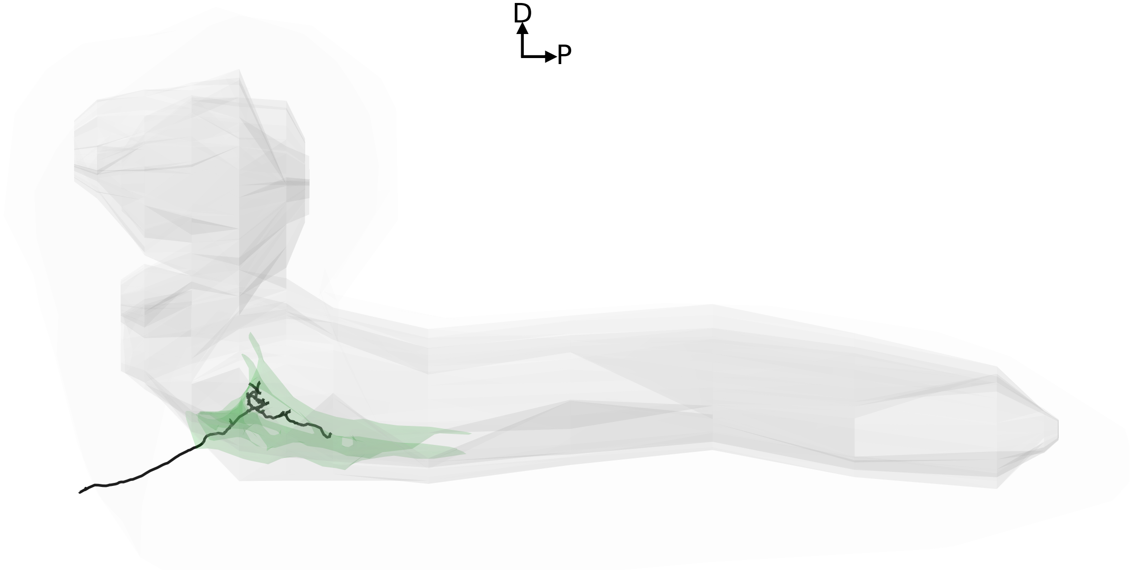

ID	*name*	SCACa	SCAVa	SCAVp	SCACal	SCACp	SCACpl	SCVM	IPCs	DMS	DH44	Se0ens	Se0ph	PMN LR	MN motor neurons	PaN motor neurons	olfactory PNs	gustatory PNs	multiglomerular PNs	unknown PNs	thermo PNs	visual PNs
15607180	MN-R-Sens-B2-VM-07	0	0	0	0	0	1	7	0	0	0	0	0	0	0	0	0	0	0	0	0	0

ID: 15610387
name: MN-R-Sens-B2-VM-08

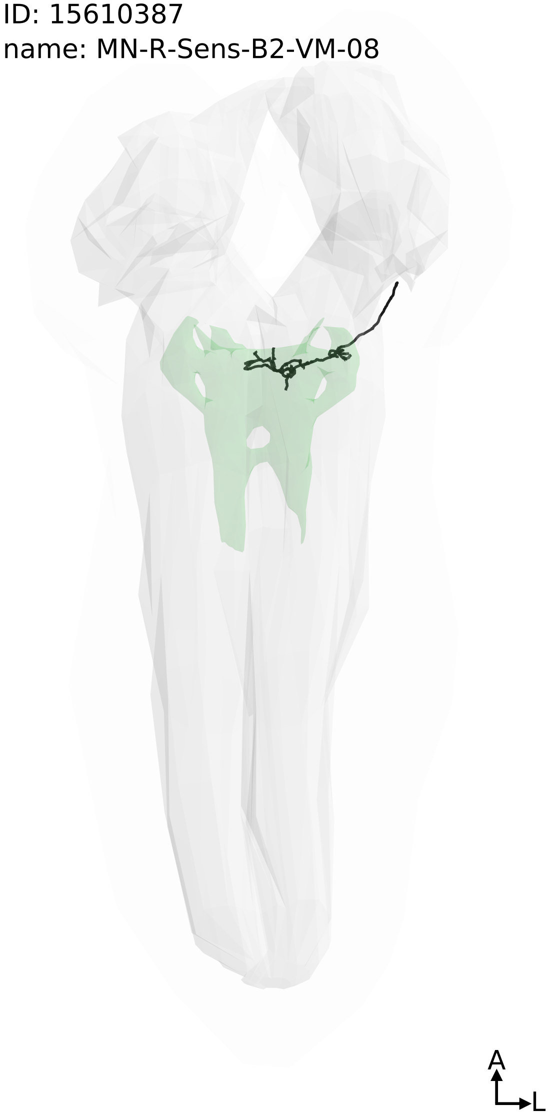

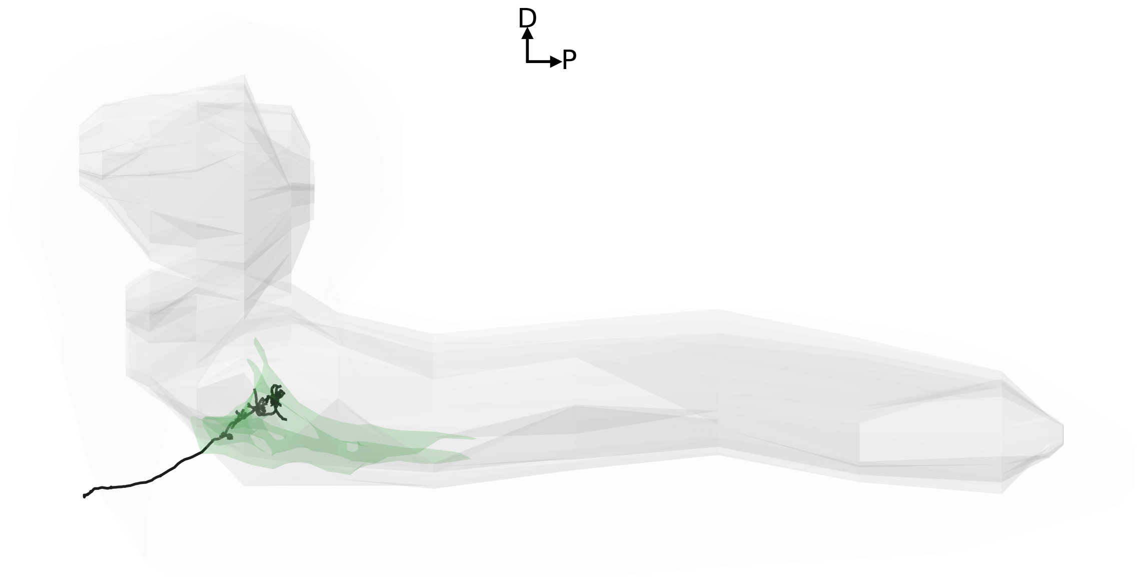

ID	name	SCACa	SCAVa	SCAVp	SCACal	SCACp	SCACpl	SCVM	IPCs	DMS	DH44	Se0ens	Se0ph	PMN LR	MN motor neurons	PaN motor neurons	olfactory PNs	gustatory PNs	multiglomerular PNs	unknown PNs	thermo PNs	visual PNs
15610387	MN-R-Sens-B2-VM-08	0	0	0	0	0	0	2	0	0	0	0	0	0	0	3	0	0	0	0	0	0

ID: 15611642
name: MN-R-Sens-B2-VM-09

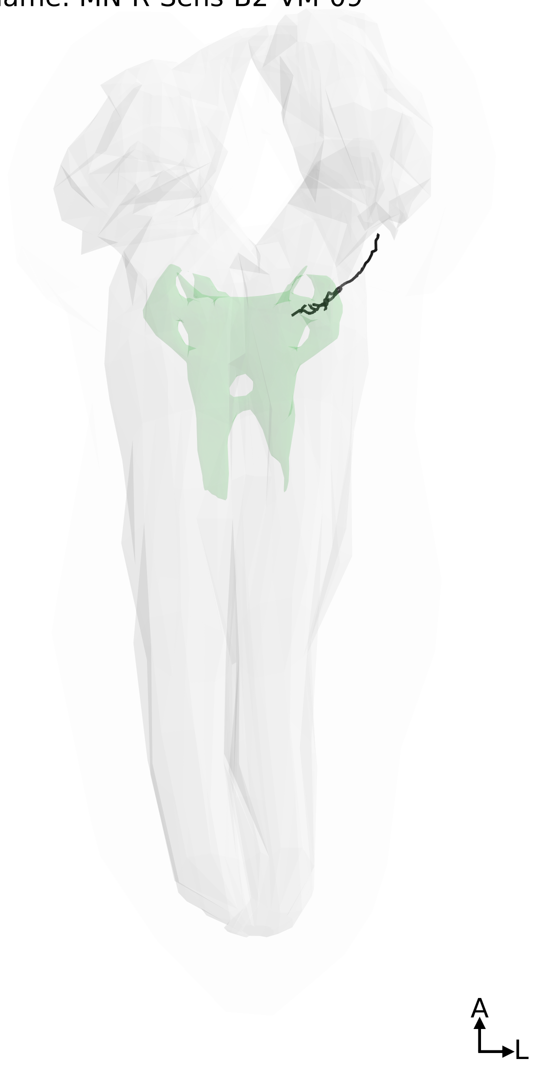

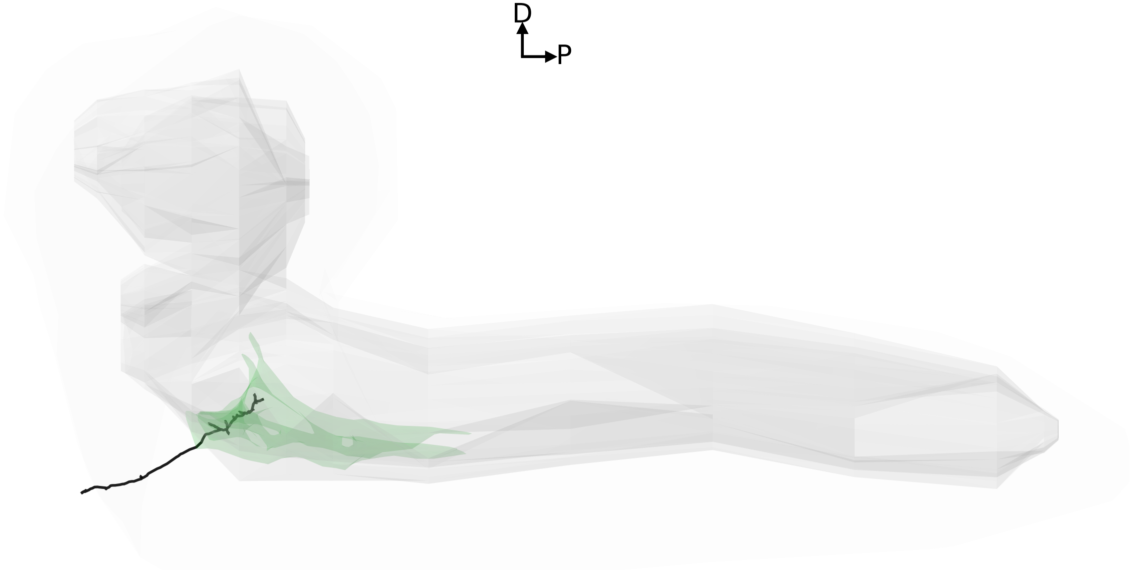

ID	*name*	SCACa	SCAVa	SCAVp	SCACal	SCACp	SCACpl	SCVM	IPCs	DMS	DH44	Se0ens	Se0ph	PMN LR	MN motor neurons	PaN motor neurons	olfactory PNs	gustatory PNs	multiglomerular PNs	unknown PNs	thermo PNs	visual PNs
15611642	MN-R-Sens-B2-VM-09	0	0	0	0	0	0	2	0	0	0	0	0	0	0	0	0	0	0	0	0	0

ID: 15615475
name: MN-R-Sens-B2-VM-10

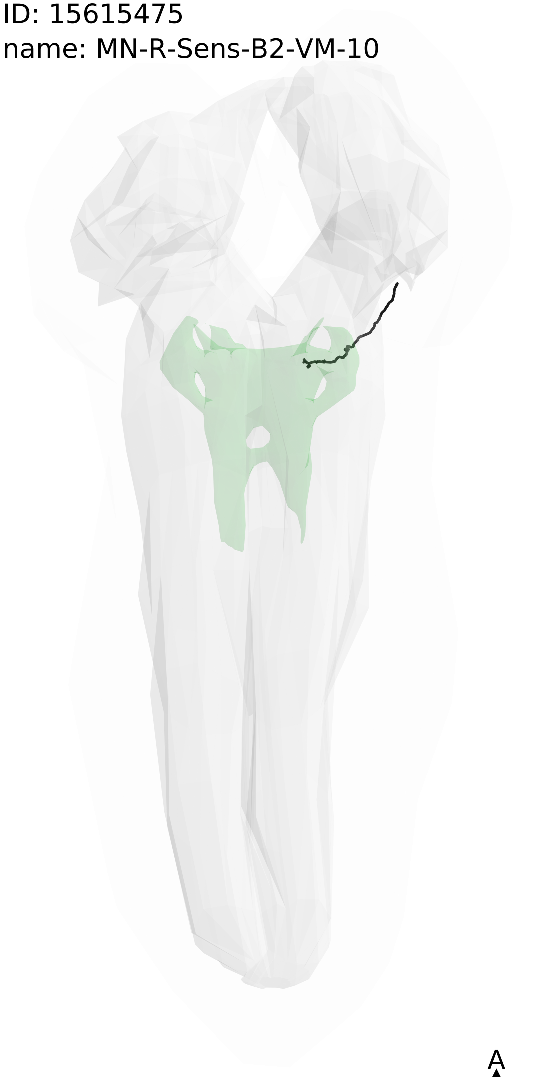

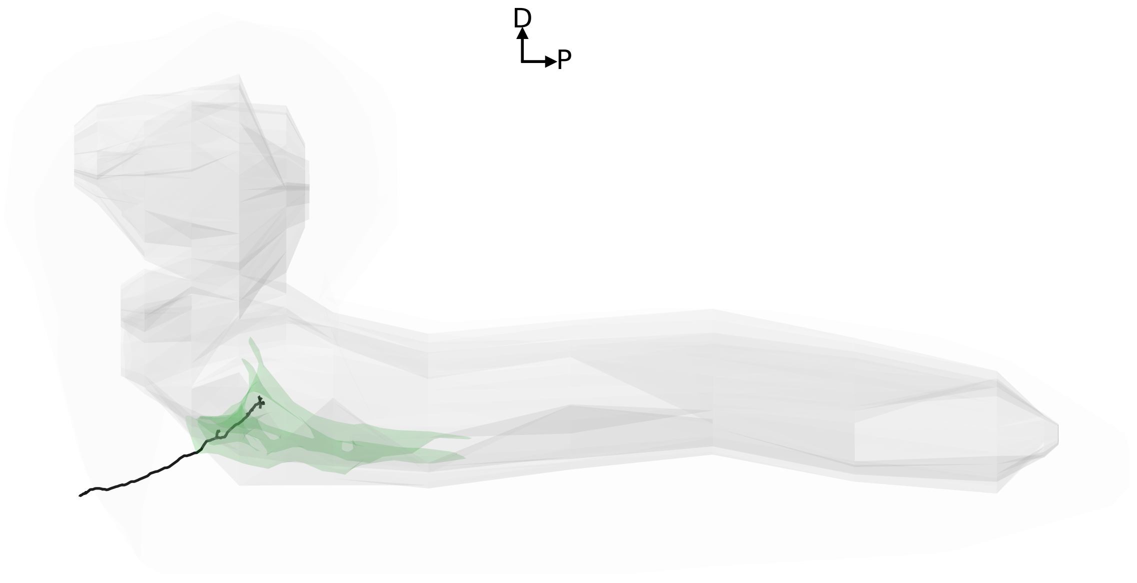

ID	*name*	SCACa	SCAVa	SCAVp	SCACal	SCACp	SCACpl	SCVM	IPCs	DMS	DH44	Se0ens	Se0ph	PMN LR	MN motor neurons	PaN motor neurons	olfactory PNs	gustatory PNs	multiglomerular PNs	unknown PNs	thermo PNs	visual PNs
15615475	MN-R-Sens-B2-VM-10	0	0	0	0	0	0	1	0	0	0	0	0	0	0	0	0	0	0	0	0	0

ID: 15615305
name: MN-R-Sens-B2-VM-11

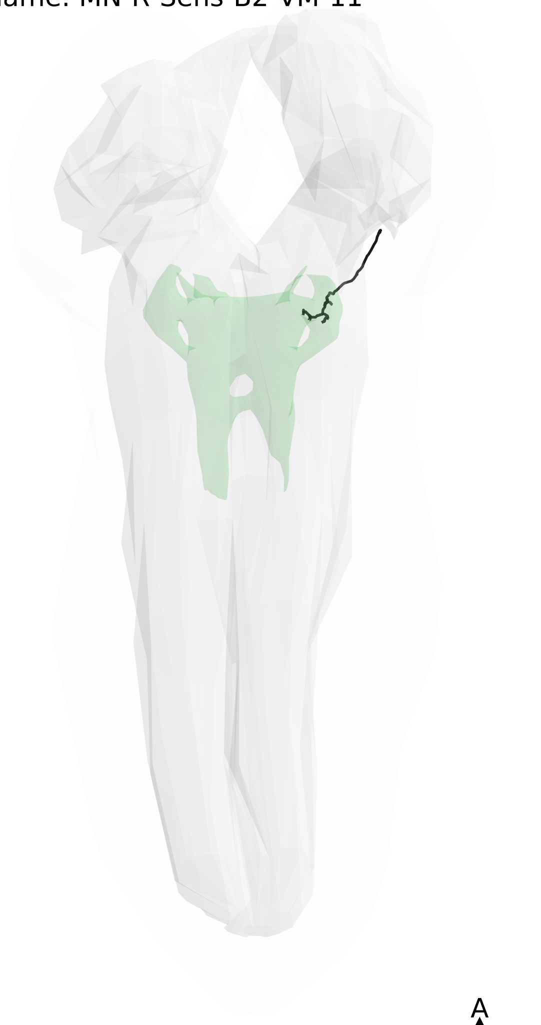

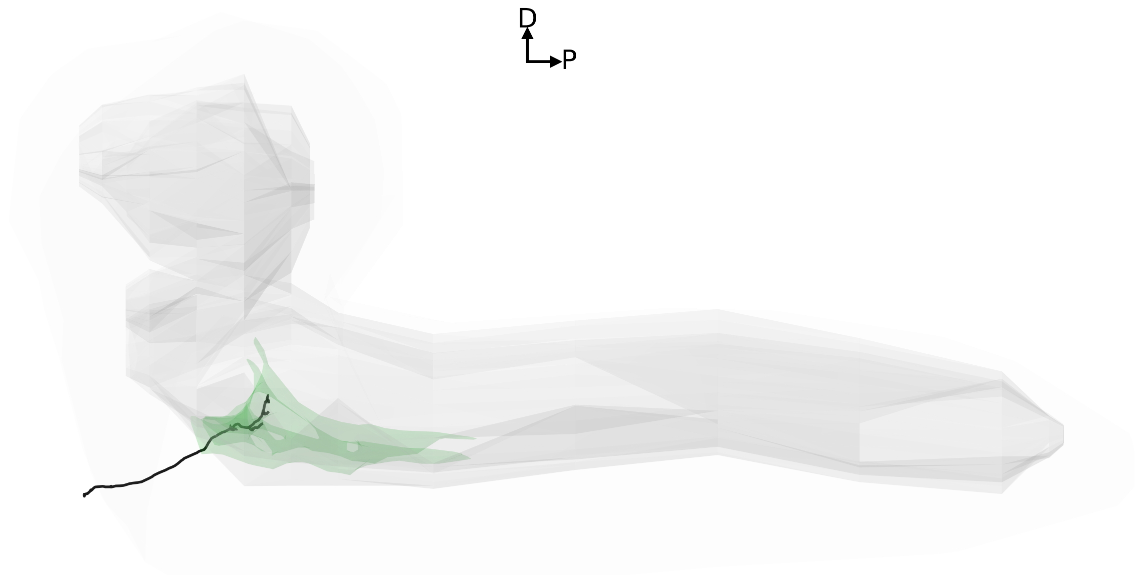

ID	*name*	SCACa	SCAVa	SCAVp	SCACal	SCACp	SCACpl	SCVM	IPCs	DMS	DH44	Se0ens	Se0ph	PMN LR	MN motor neurons	PaN motor neurons	olfactory PNs	gustatory PNs	multiglomerular PNs	unknown PNs	thermo PNs	visual PNs
15615305	MN-R-Sens-B2-VM-11	0	0	0	0	0	0	0	0	0	0	0	0	0	0	0	0	0	0	0	0	0

ID: 15618976
name: MN-R-Sens-B2-VM-12

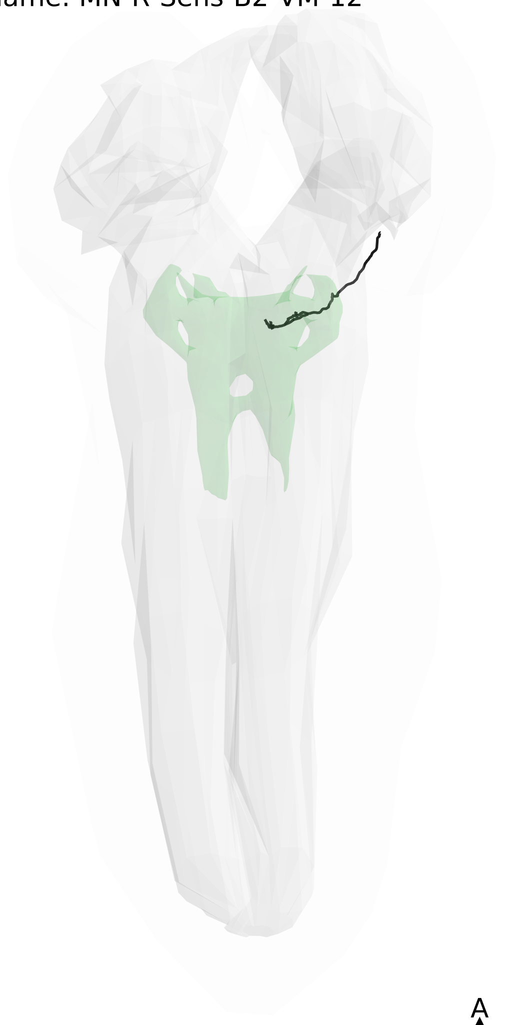

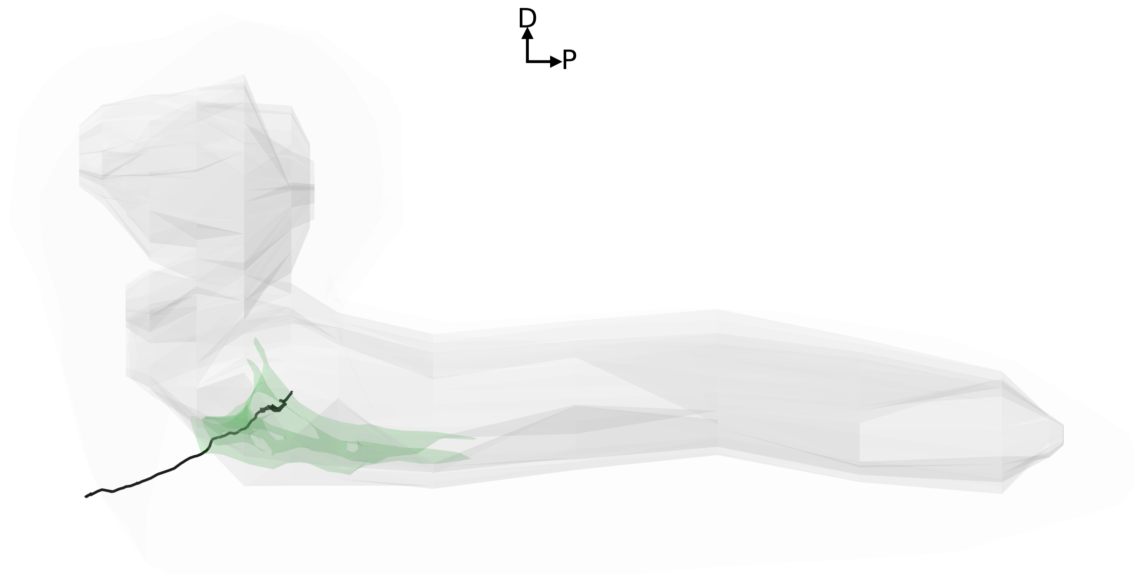

ID	*name*	SCACa	SCAVa	SCAVp	SCACal	SCACp	SCACpl	SCVM	IPCs	DMS	DH44	Se0ens	Se0ph	PMN LR	MN motor neurons	PaN motor neurons	olfactory PNs	gustatory PNs	multiglomerular PNs	unknown PNs	thermo PNs	visual PNs
15618976	MN-R-Sens-B2-VM-12	0	0	0	0	0	0	6	0	0	0	0	0	0	0	0	0	0	0	0	0	0

ID: 15620606
name: MN-R-Sens-B2-VM-13

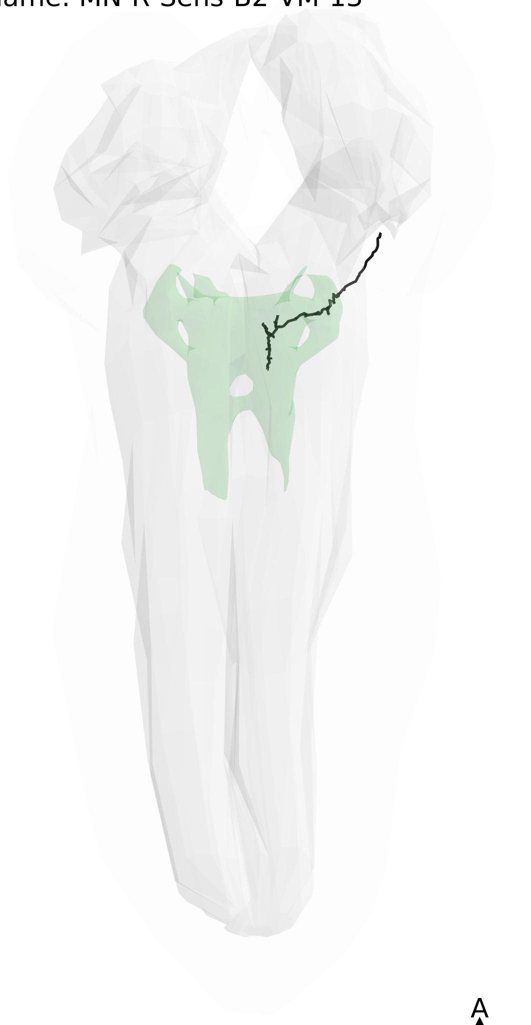

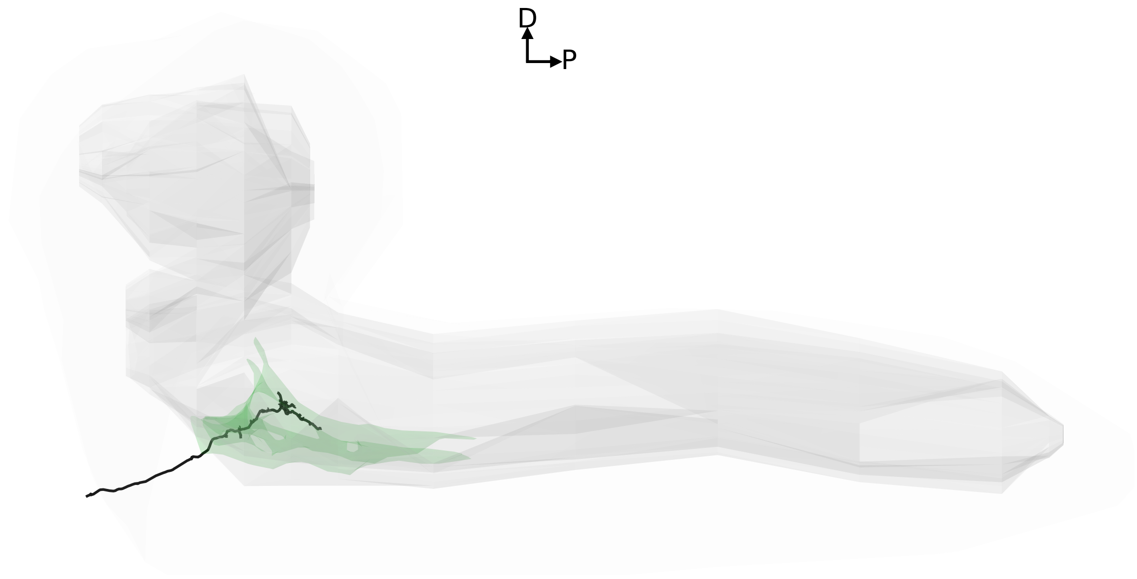

ID	*name*	SCACa	SCAVa	SCAVp	SCACal	SCACp	SCACpl	SCVM	IPCs	DMS	DH44	Se0ens	Se0ph	PMN LR	MN motor neurons	PaN motor neurons	olfactory PNs	gustatory PNs	multiglomerular PNs	unknown PNs	thermo PNs	visual PNs
15620606	MN-R-Sens-B2-VM-13	0	0	0	0	0	0	6	0	0	0	0	0	0	0	0	0	0	0	0	0	0

ID: 15762568
name: MN-R-Sens-B2-VM-14

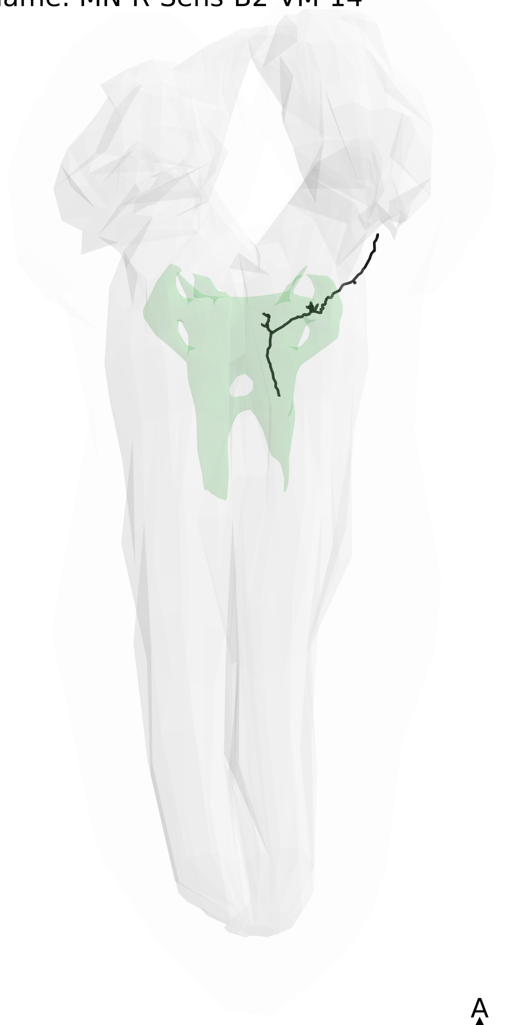

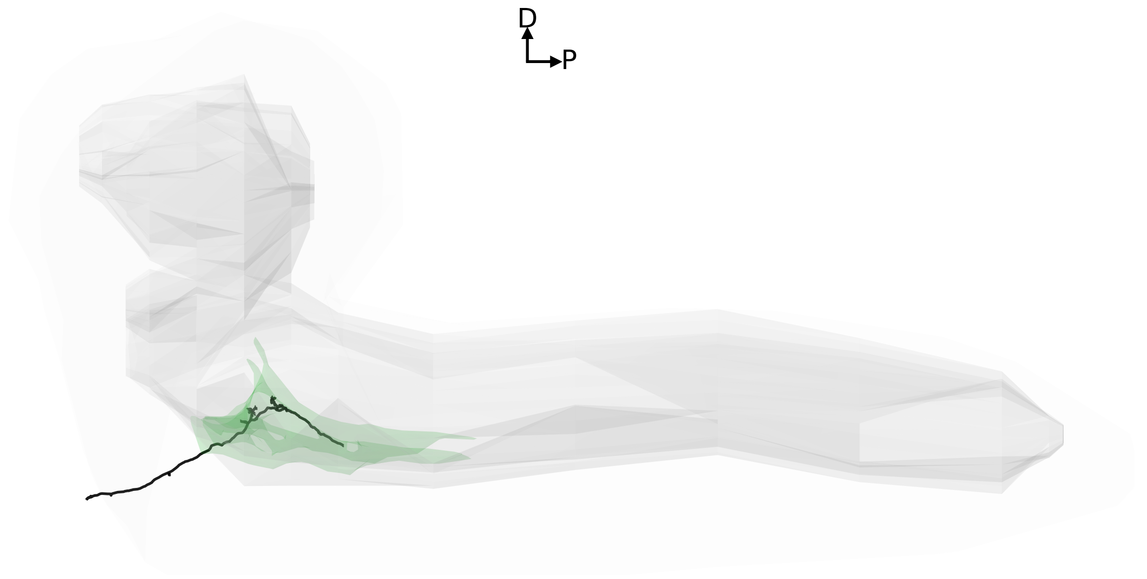

ID	name	SCACa	SCAVa	SCAVp	SCACal	SCACp	SCACpl	SCVM	IPCs	DMS	DH44	Se0ens	Se0ph	PMN LR	MN motor neurons	PaN motor neurons	olfactory PNs	gustatory PNs	multiglomerular PNs	unknown PNs	thermo PNs	visual PNs
15762568	MN-R-Sens-B2-VM-14	0	0	0	0	0	0	6	0	0	0	0	0	0	0	0	0	0	0	0	0	0

ID: 15629241
name: MN-R-Sens-B2-VM-15

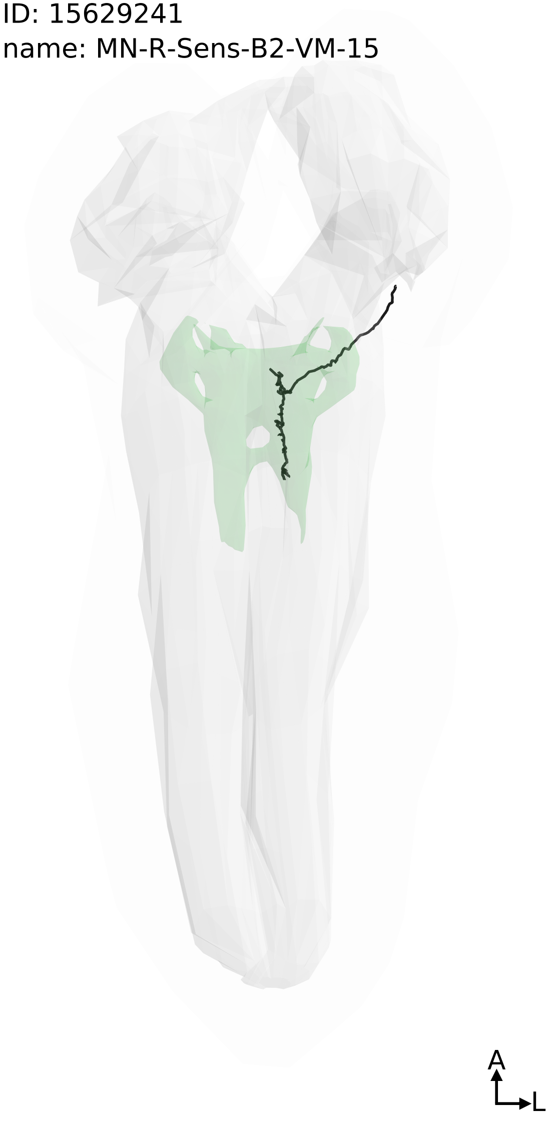

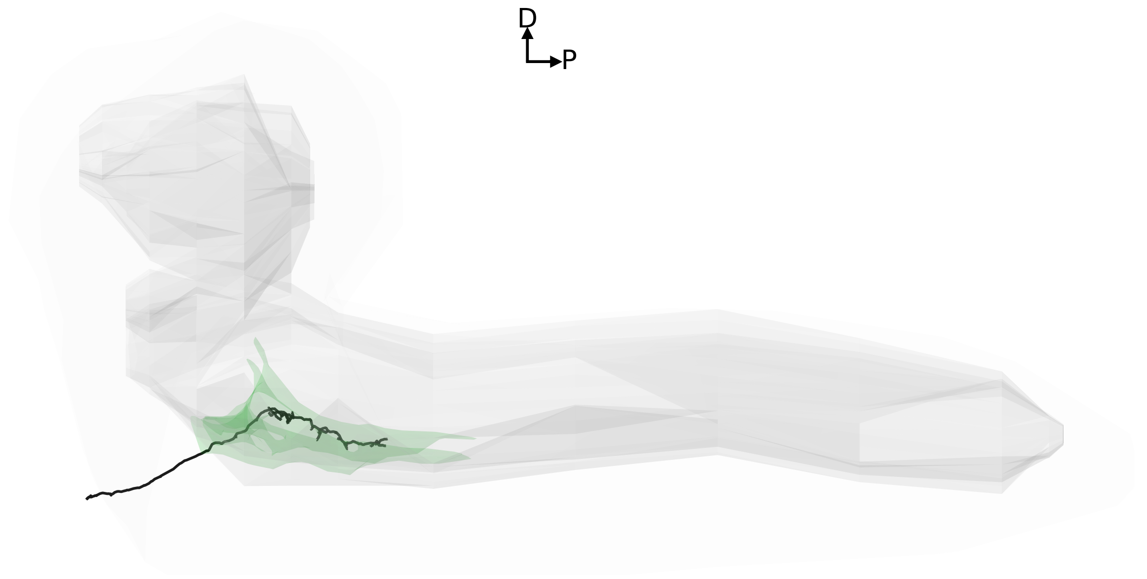

ID	name	SCACa	SCAVa	SCAVp	SCACal	SCACp	SCACpl	SCVM	IPCs	DMS	DH44	Se0ens	Se0ph	PMN LR	MN motor neurons	PaN motor neurons	olfactory PNs	gustatory PNs	multiglomerular PNs	unknown PNs	thermo PNs	visual PNs
15629241	MN-R-Sens-B2-VM-15	0	0	0	0	0	0	7	0	0	0	0	0	0	0	0	0	0	0	0	0	0

ID: 18679889
name: MN-R-Sens-B2-VM-16

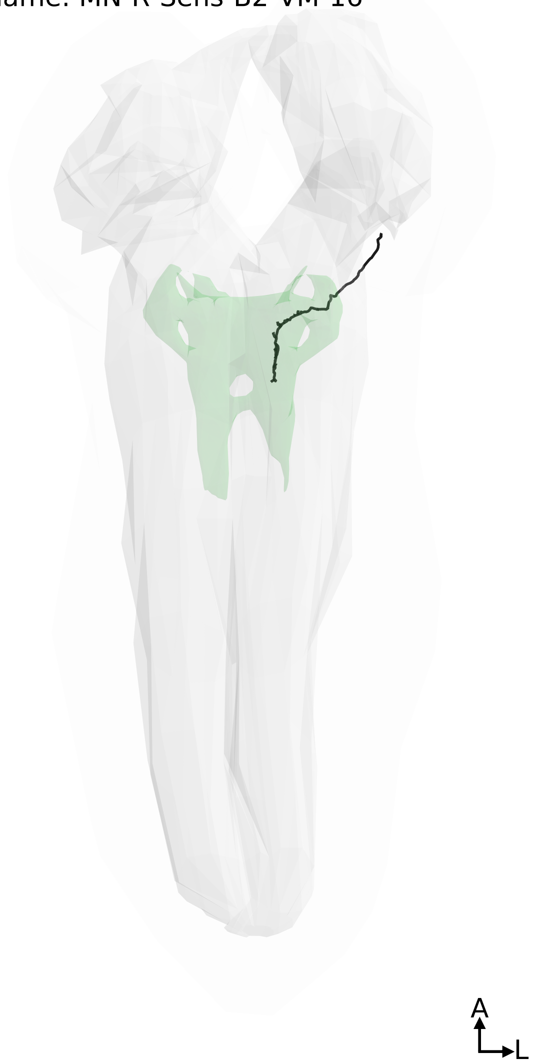

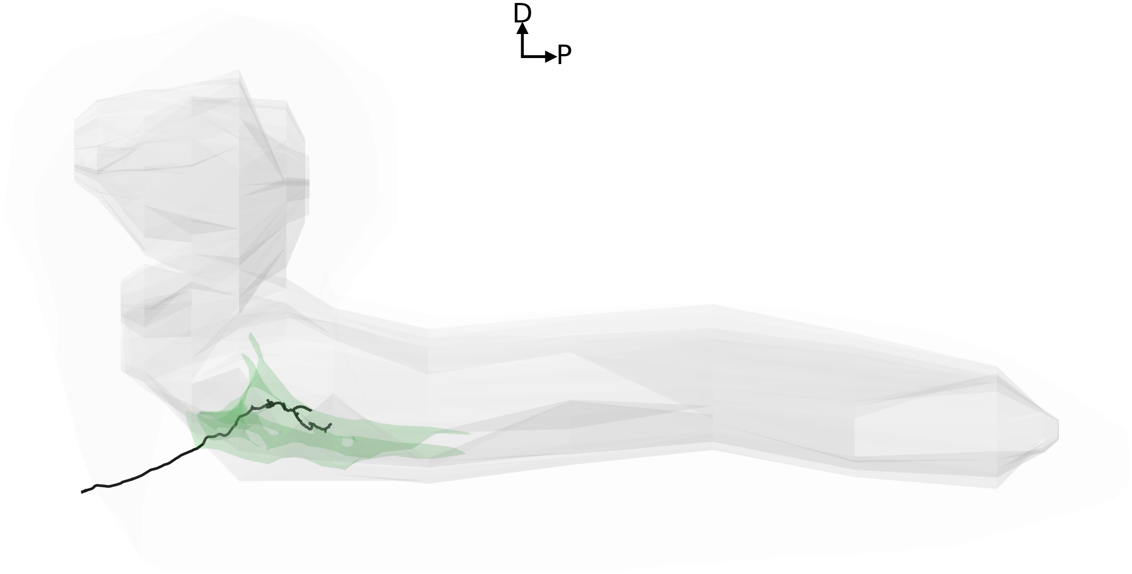

ID	*name*	SCACa	SCAVa	SCAVp	SCACal	SCACp	SCACpl	SCVM	IPCs	DMS	DH44	Se0ens	Se0ph	PMN LR	MN motor neurons	PaN motor neurons	olfactory PNs	gustatory PNs	multiglomerular PNs	unknown PNs	thermo PNs	visual PNs
18679889	MN-R-Sens-B2-VM-16	0	0	0	0	0	0	7	0	0	0	0	0	0	0	0	0	0	0	0	0	0

ID: 15620432
name: MN-R-Sens-B2-VM-17

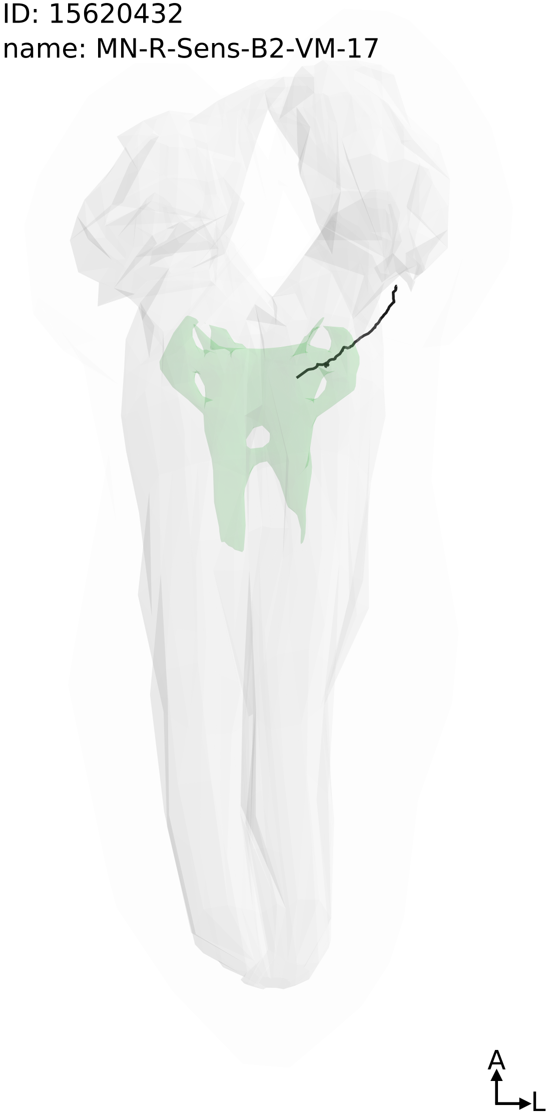

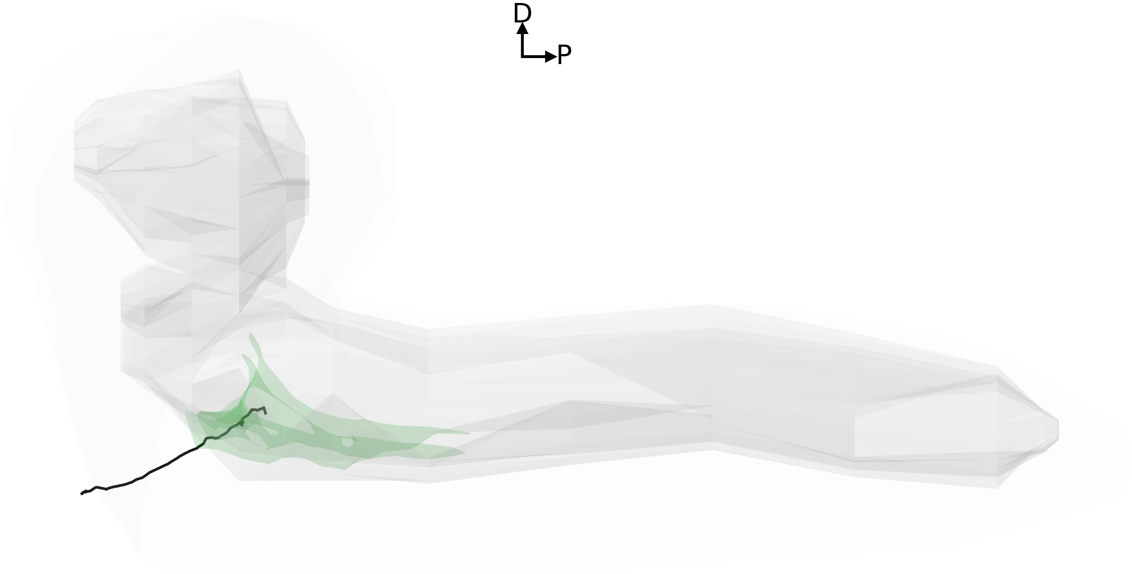

ID	name	SCACa	SCAVa	SCAVp	SCACal	SCACp	SCACpl	SCVM	IPCs	DMS	DH44	Se0ens	Se0ph	PMN LR	MN motor neurons	PaN motor neurons	olfactory PNs	gustatory PNs	multiglomerular PNs	unknown PNs	thermo PNs	visual PNs
15620432	MN-R-Sens-B2-VM-17	0	0	0	0	0	0	2	0	0	0	0	0	0	0	0	0	0	0	0	0	0

ID: 15629888
name: MN-R-Sens-B2-VM-18

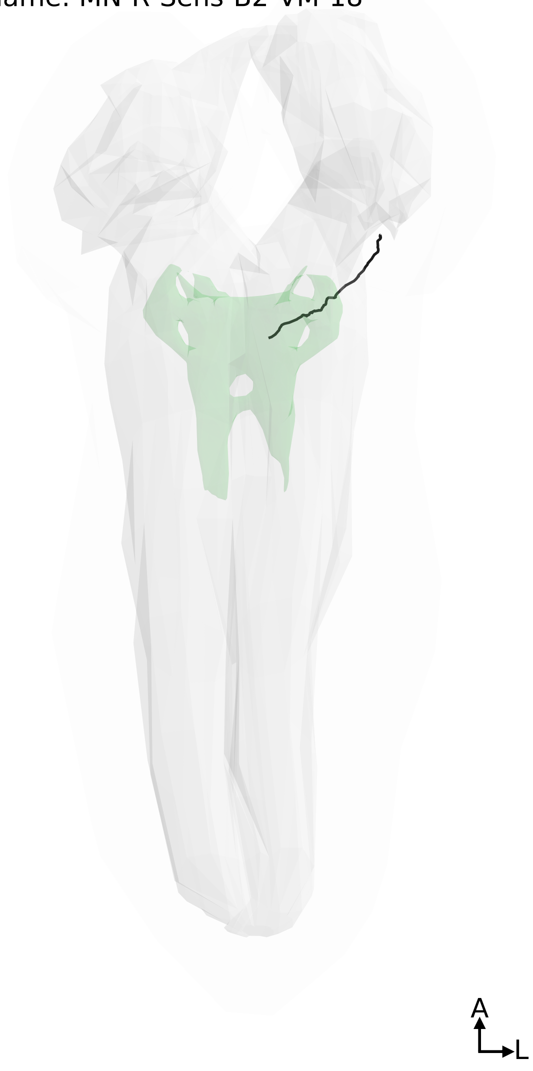

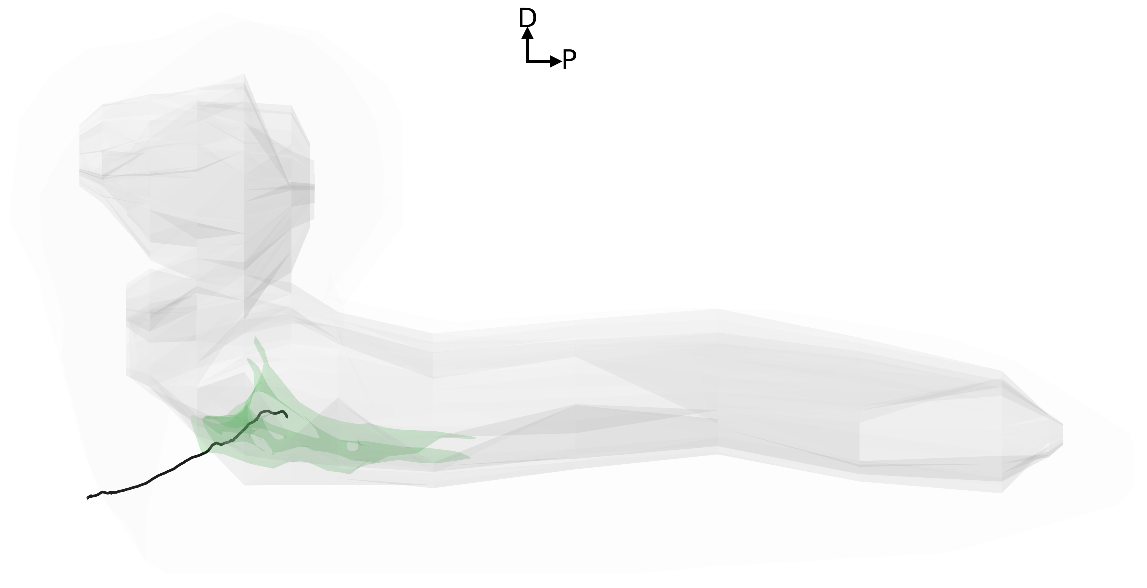

ID	name	SCACa	SCAVa	SCAVp	SCACal	SCACp	SCACpl	SCVM	IPCs	DMS	DH44	Se0ens	Se0ph	PMN LR	MN motor neurons	PaN motor neurons	olfactory PNs	gustatory PNs	multiglomerular PNs	unknown PNs	thermo PNs	visual PNs
15629888	MN-R-Sens-B2-VM-18	0	0	0	0	0	0	3	0	0	0	0	0	0	0	0	0	0	0	0	0	0

ID: 15612241
name: MN-R-Sens-B2-VM-19

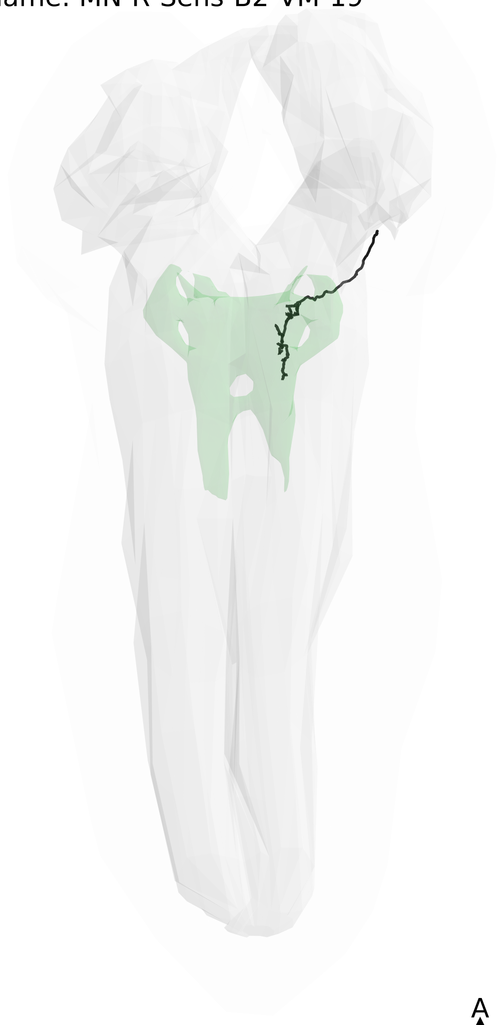

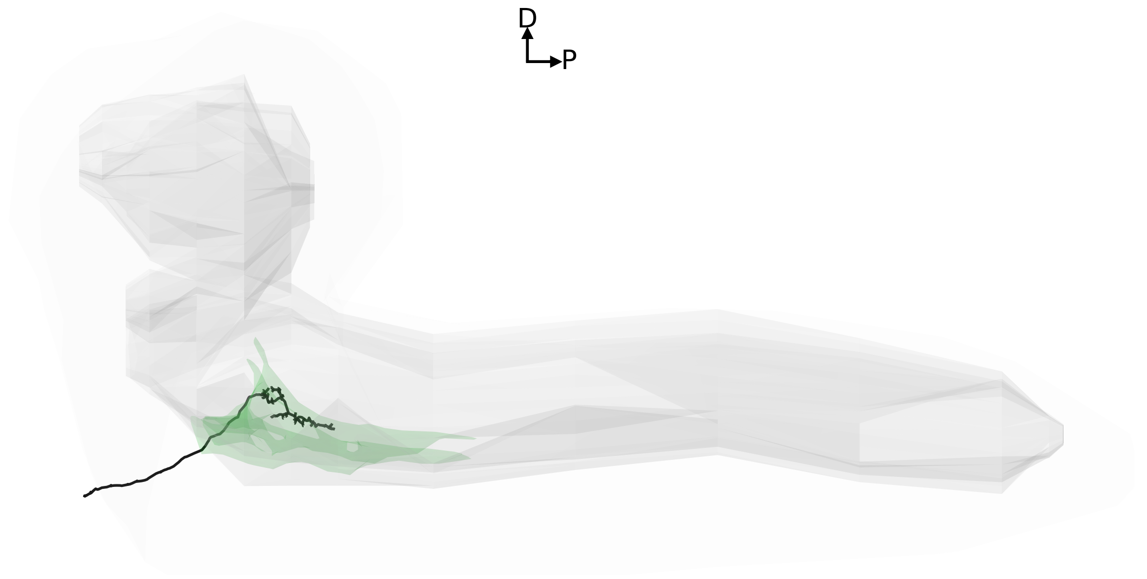

ID	name	SCACa	SCAVa	SCAVp	SCACal	SCACp	SCACpl	SCVM	IPCs	DMS	DH44	Se0ens	Se0ph	PMN LR	MN motor neurons	PaN motor neurons	olfactory PNs	gustatory PNs	multiglomerular PNs	unknown PNs	thermo PNs	visual PNs
15612241	MN-R-Sens-B2-VM-19	0	0	0	0	0	0	9	0	0	0	0	0	0	0	0	0	0	0	0	0	0

A
L

ID: 15618756
name: MN-R-Sens-B2-VM-20

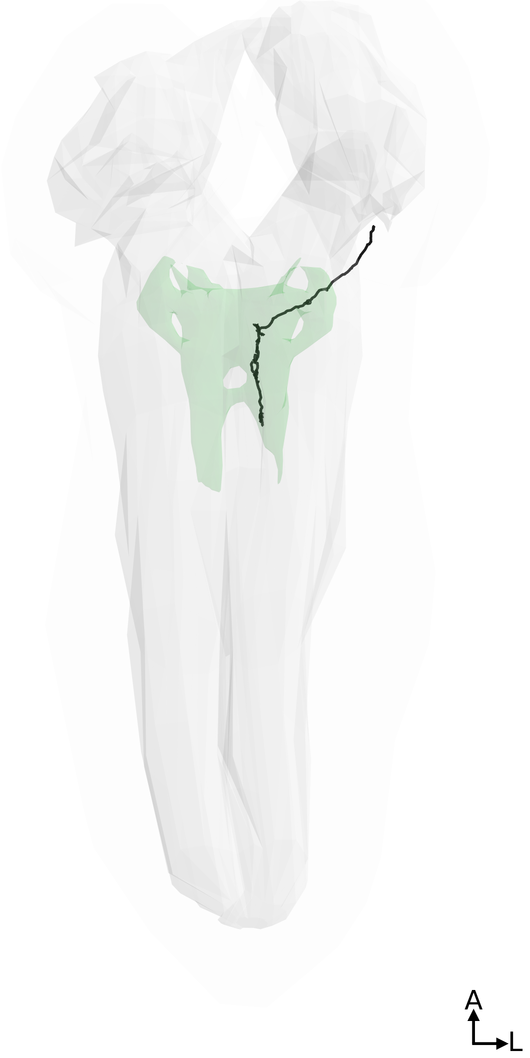

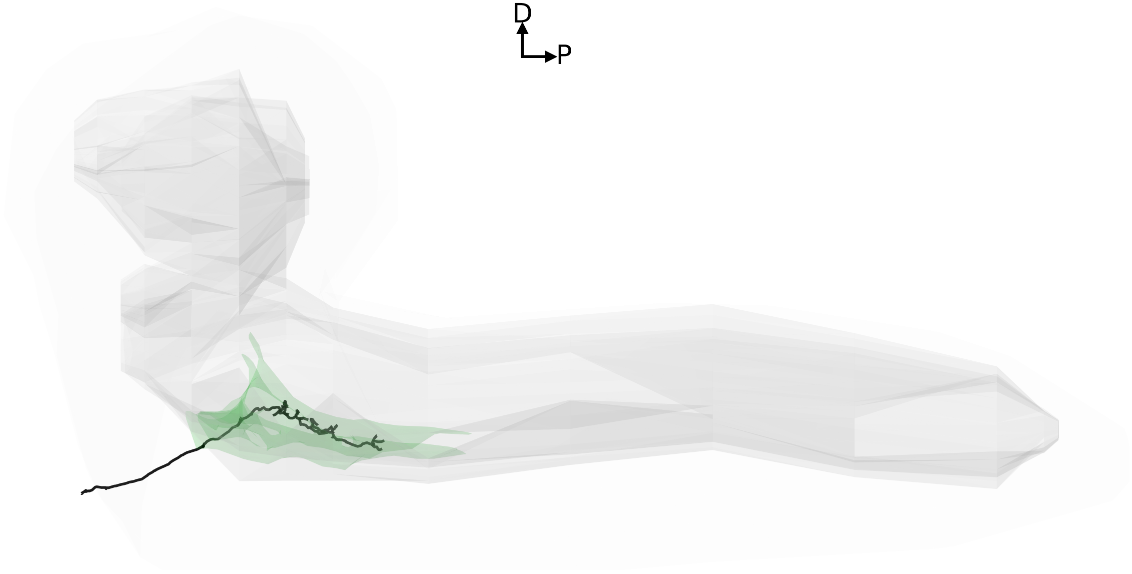

ID	*name*	SCACa	SCAVa	SCAVp	SCACal	SCACp	SCACpl	SCVM	IPCs	DMS	DH44	Se0ens	Se0ph	PMN LR	MN motor neurons	PaN motor neurons	olfactory PNs	gustatory PNs	multiglomerular PNs	unknown PNs	thermo PNs	visual PNs
15618756	MN-R-Sens-B2-VM-20	0	0	0	0	0	0	3	0	0	0	0	0	0	0	0	0	0	0	0	0	0

ID: 3562954
name: MN-R-Sens-B2-VM-21

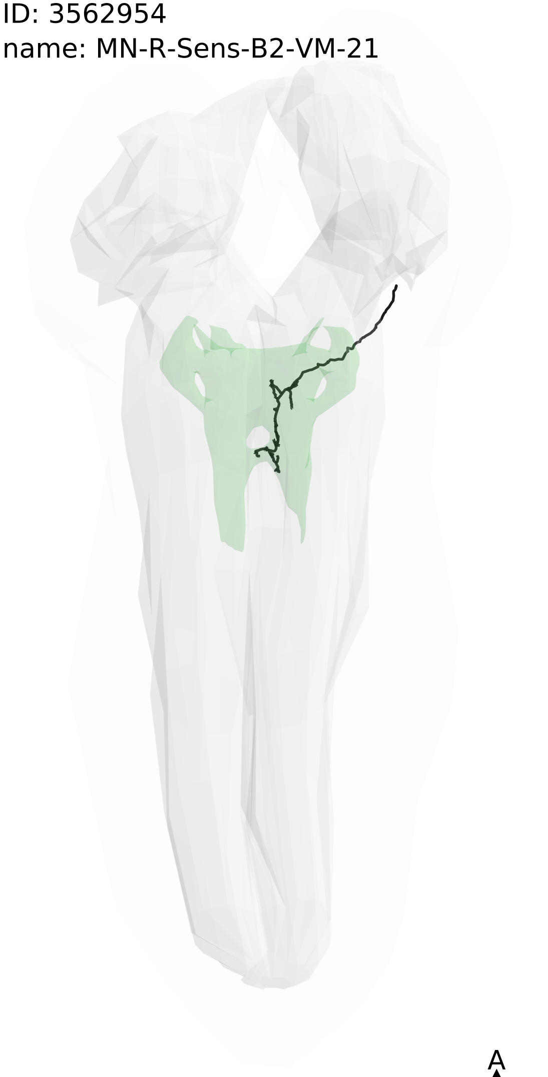

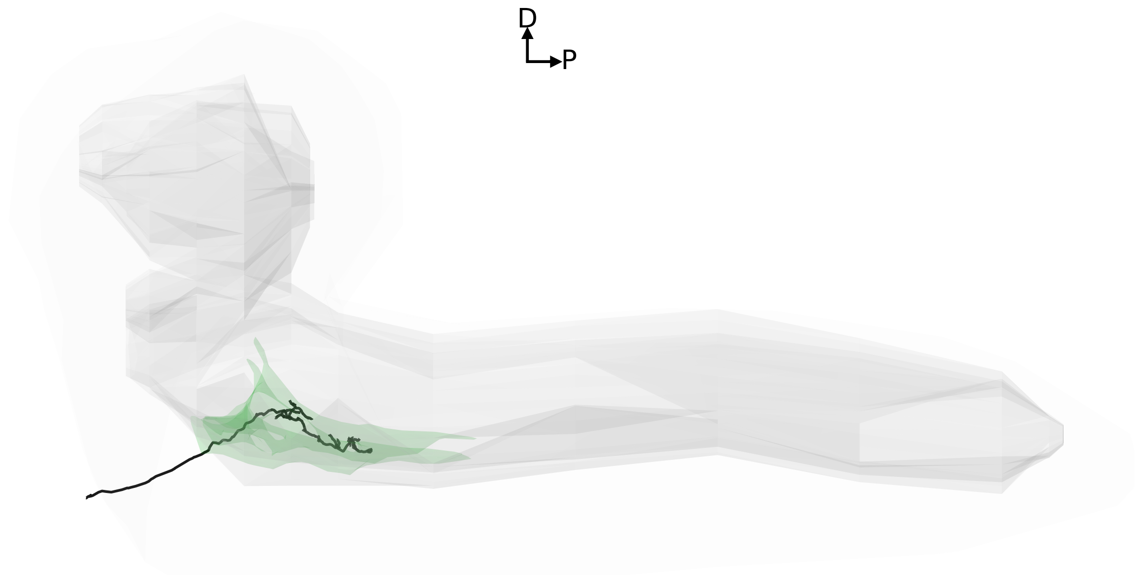

ID	name	SCACa	SCAVa	SCAVp	SCACal	SCACp	SCACpl	SCVM	IPCs	DMS	DH44	Se0ens	Se0ph	PMN LR	MN motor neurons	PaN motor neurons	olfactory PNs	gustatory PNs	multiglomerular PNs	unknown PNs	thermo PNs	visual PNs
3562954	MN-R-Sens-B2-VM-21	0	0	0	0	0	0	9	0	0	0	0	0	0	0	0	0	0	0	0	0	0

ID: 15615877
name: MN-R-Sens-B2-VM-22

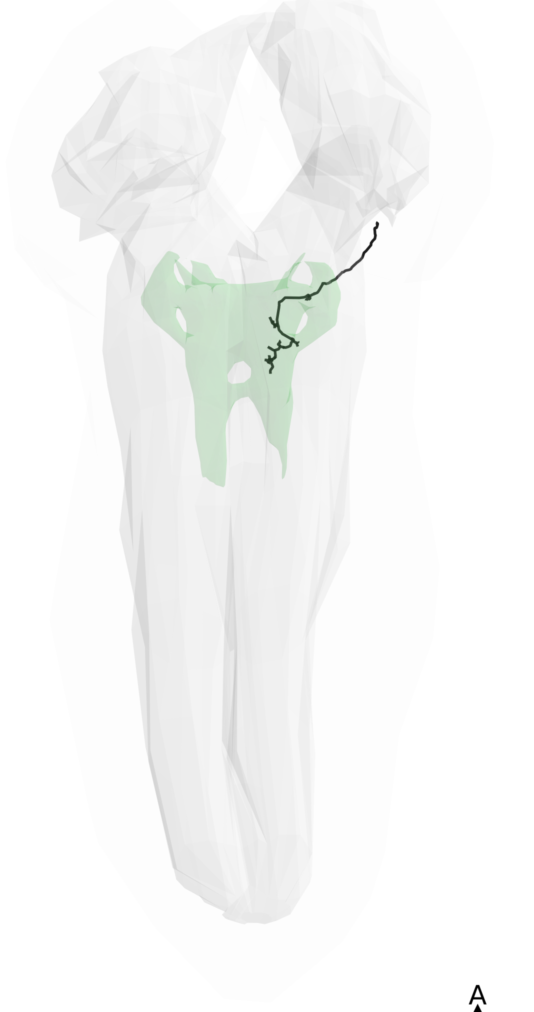

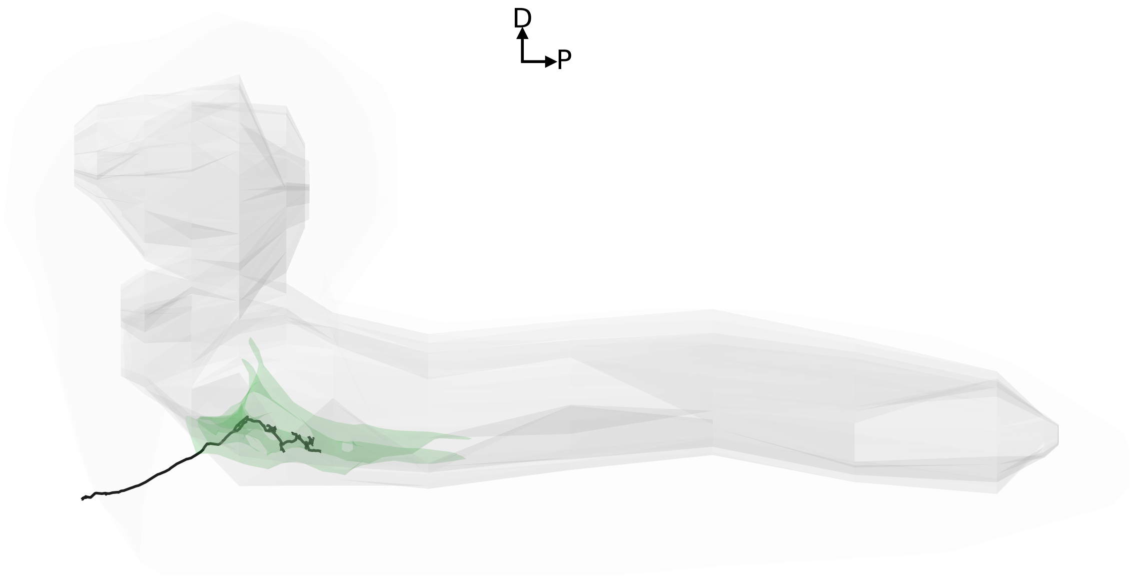

ID	*name*	SCACa	SCAVa	SCAVp	SCACal	SCACp	SCACpl	SCVM	IPCs	DMS	DH44	Se0ens	Se0ph	PMN LR	MN motor neurons	PaN motor neurons	olfactory PNs	gustatory PNs	multiglomerular PNs	unknown PNs	thermo PNs	visual PNs
15615877	MN-R-Sens-B2-VM-22	0	0	1	0	0	0	4	0	0	0	0	0	0	0	0	0	0	0	0	0	0

ID: 18833414
name: MN-R-Sens-B2-VM-23

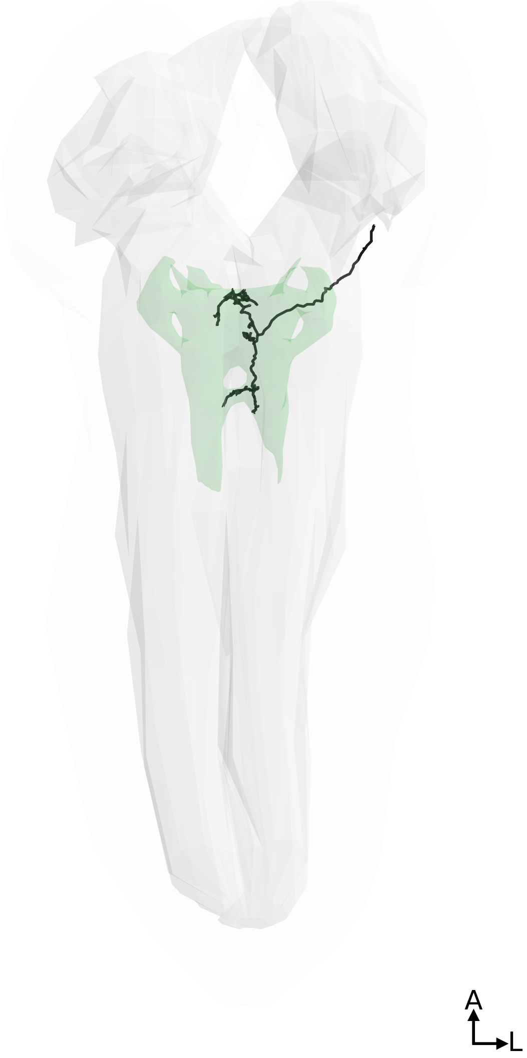

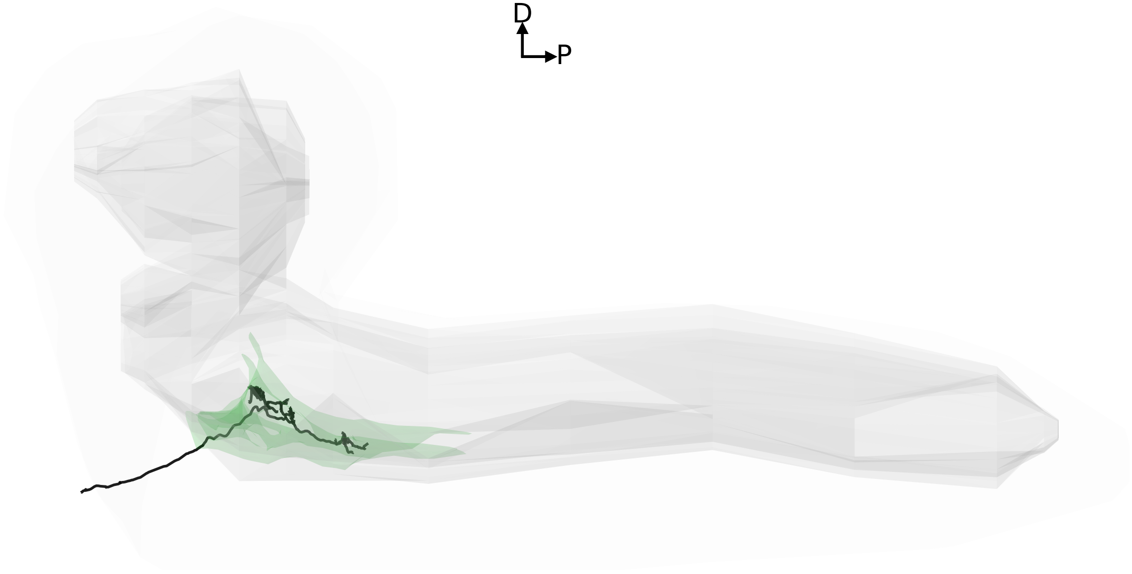

ID	*name*	SCACa	SCAVa	SCAVp	SCACal	SCACp	SCACpl	SCVM	IPCs	DMS	DH44	Se0ens	Se0ph	PMN LR	MN motor neurons	PaN motor neurons	olfactory PNs	gustatory PNs	multiglomerular PNs	unknown PNs	thermo PNs	visual PNs
18833414	MN-R-Sens-B2-VM-23	0	0	0	0	0	0	9	0	0	0	0	0	0	0	0	0	0	0	0	0	0

ID: 15672153
name: MN-R-Sens-B3-VM-04

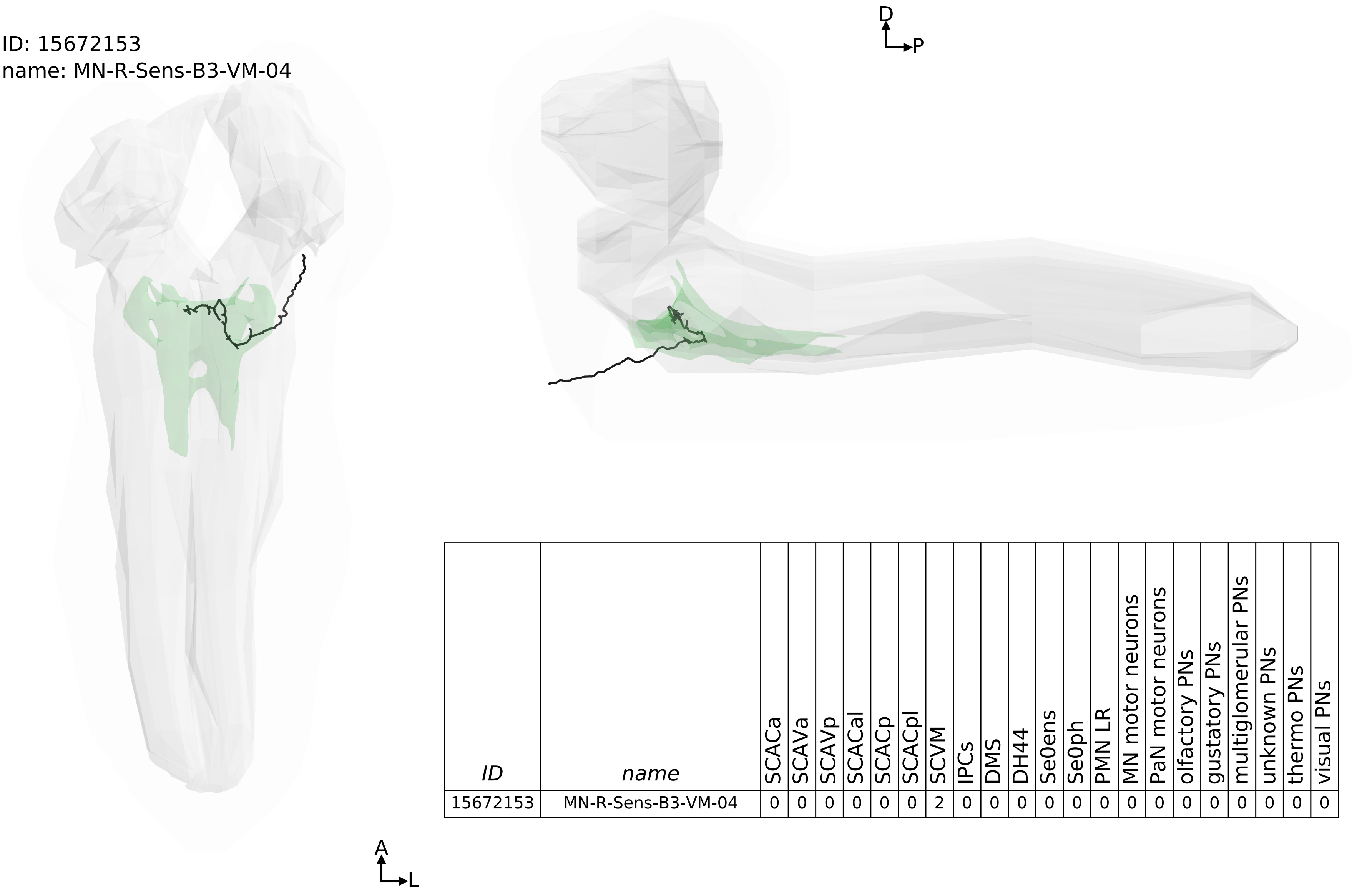

ID	*name*	SCACa	SCAVa	SCAVp	SCACal	SCACp	SCACpl	SCVM	IPCs	DMS	DH44	Se0ens	Se0ph	PMN LR	MN motor neurons	PaN motor neurons	olfactory PNs	gustatory PNs	multiglomerular PNs	unknown PNs	thermo PNs	visual PNs
15672153	MN-R-Sens-B3-VM-04	0	0	0	0	0	0	2	0	0	0	0	0	0	0	0	0	0	0	0	0	0

ID: 15637893
name: MN-R-Sens-B3-VM-05

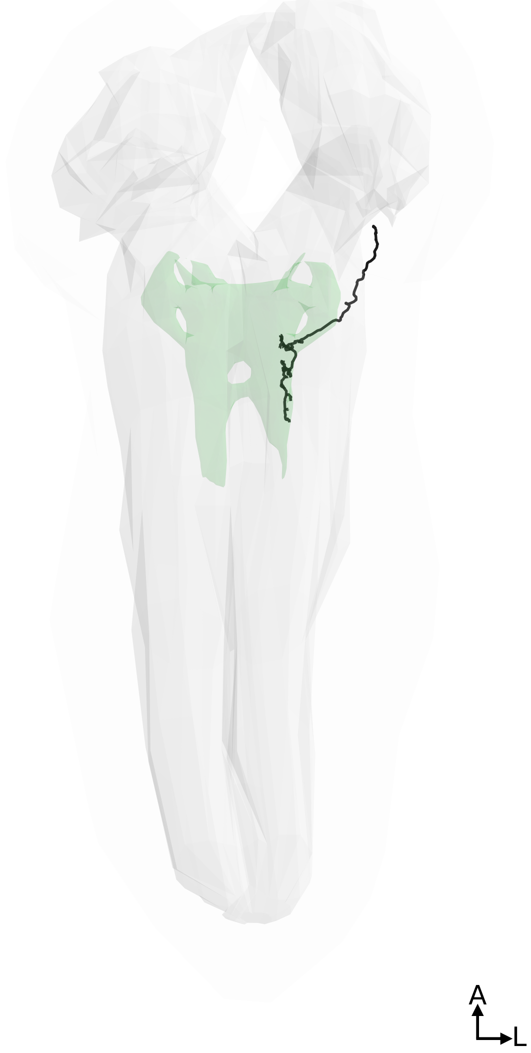

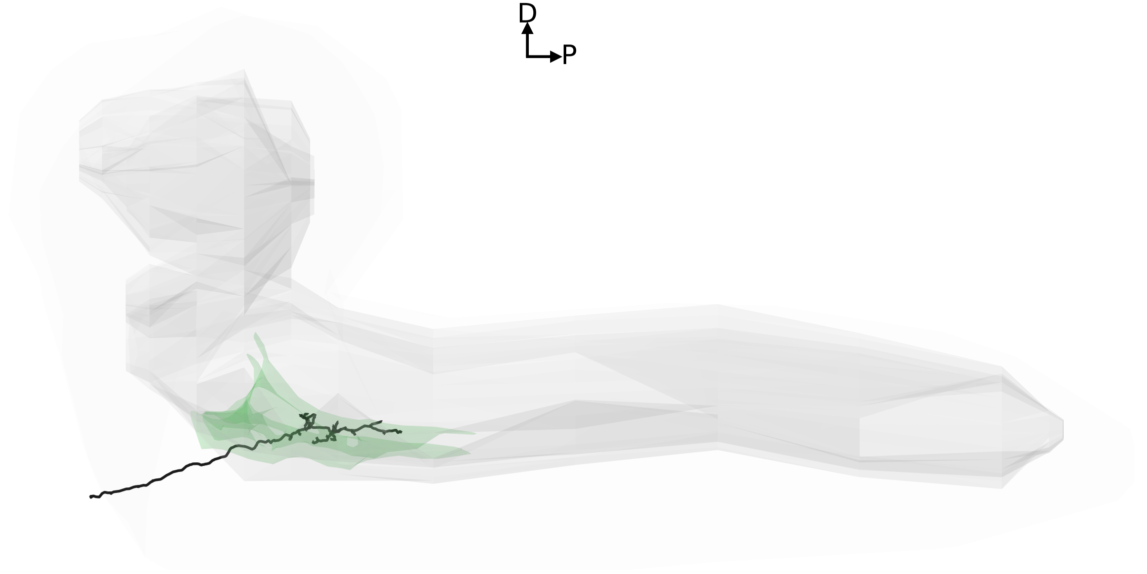

ID	*name*	SCACa	SCAVa	SCAVp	SCACal	SCACp	SCACpl	SCVM	IPCs	DMS	DH44	Se0ens	Se0ph	PMN LR	MN motor neurons	PaN motor neurons	olfactory PNs	gustatory PNs	multiglomerular PNs	unknown PNs	thermo PNs	visual PNs
15637893	MN-R-Sens-B3-VM-05	0	0	0	0	0	0	6	0	0	0	0	0	0	0	0	0	0	0	0	0	0

ID: 15638520
name: MN-R-Sens-B3-VM-06

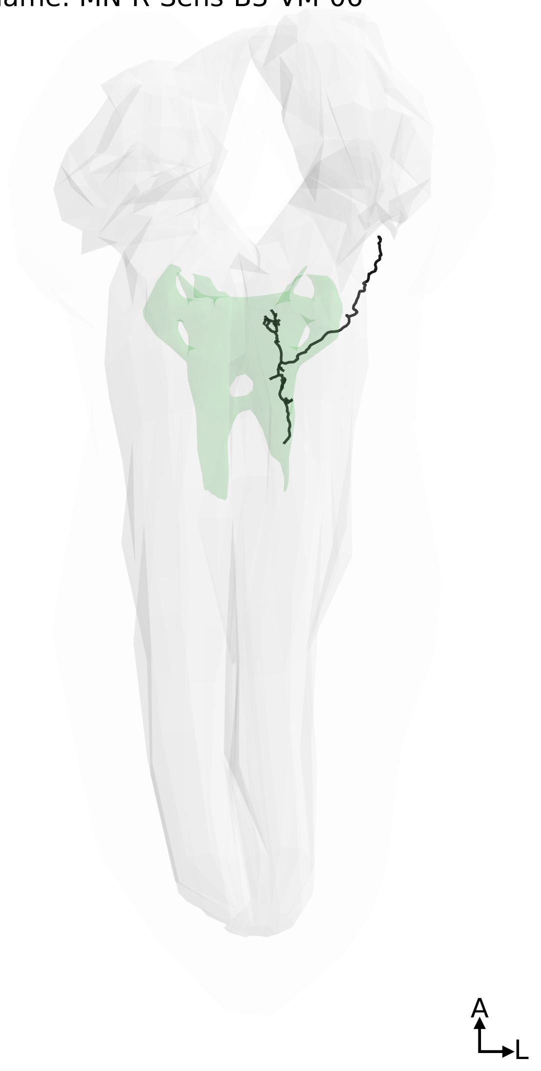

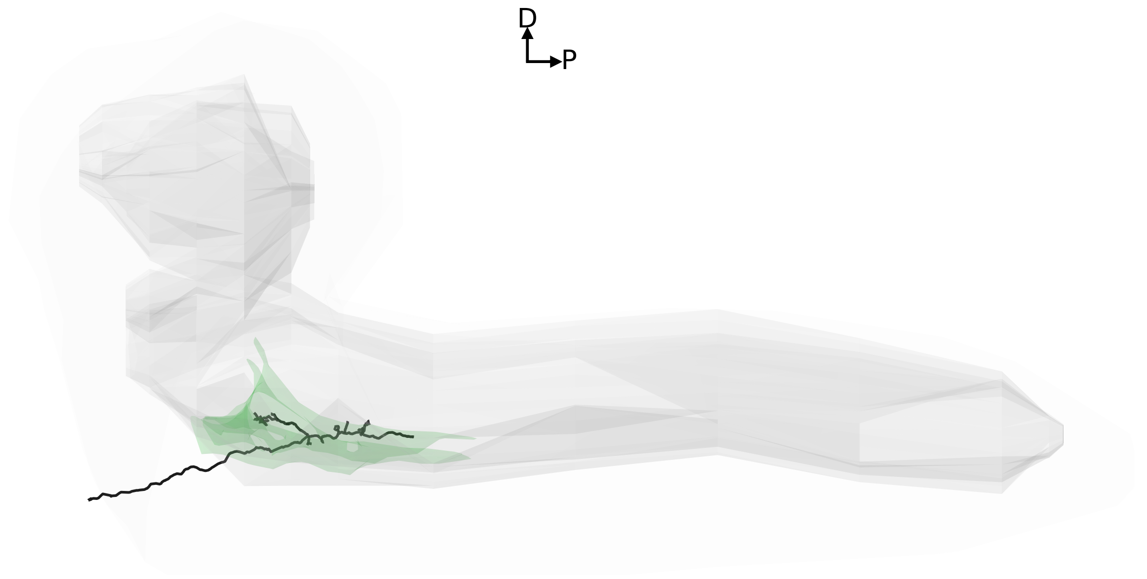

ID	*name*	SCACa	SCAVa	SCAVp	SCACal	SCACp	SCACpl	SCVM	IPCs	DMS	DH44	Se0ens	Se0ph	PMN LR	MN motor neurons	PaN motor neurons	olfactory PNs	gustatory PNs	multiglomerular PNs	unknown PNs	thermo PNs	visual PNs
15638520	MN-R-Sens-B3-VM-06	0	0	0	0	0	0	1	0	0	0	0	0	0	0	0	0	0	0	0	0	0

ID: 18785124
name: MN-R-Sens-B3-VM-07

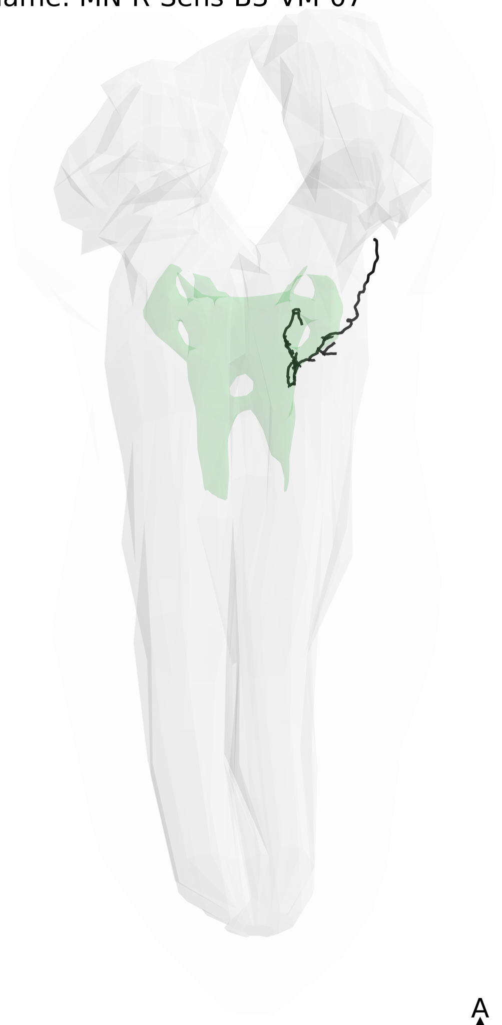

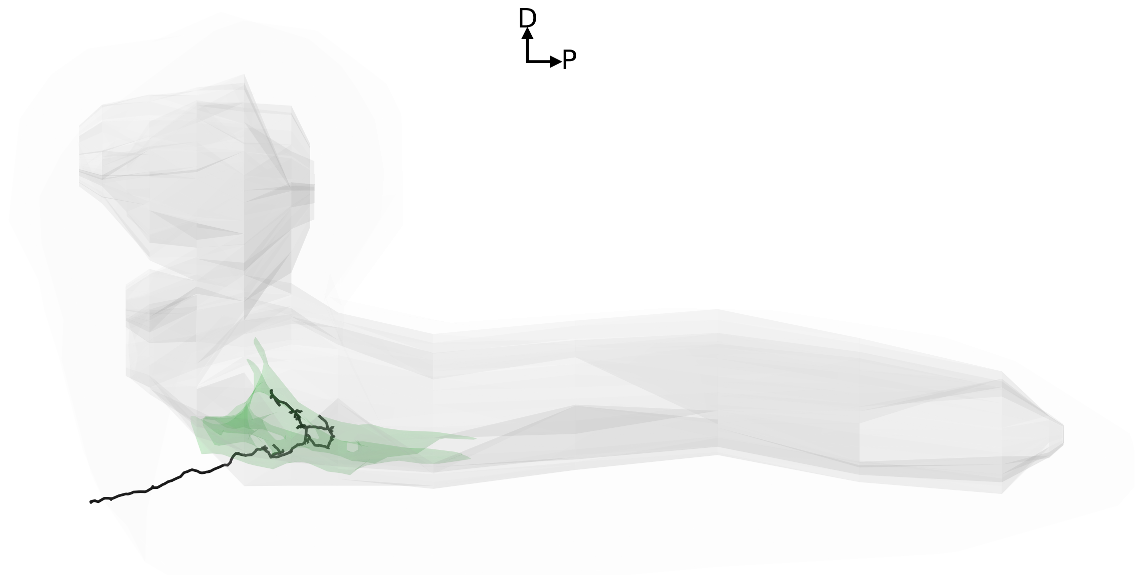

ID	*name*	SCACa	SCAVa	SCAVp	SCACal	SCACp	SCACpl	SCVM	IPCs	DMS	DH44	Se0ens	Se0ph	PMN LR	MN motor neurons	PaN motor neurons	olfactory PNs	gustatory PNs	multiglomerular PNs	unknown PNs	thermo PNs	visual PNs
18785124	MN-R-Sens-B3-VM-07	0	0	0	0	0	0	2	0	0	0	0	0	0	0	0	0	0	0	0	0	0

ID: 15639275
name: MN-R-Sens-B3-VM-08

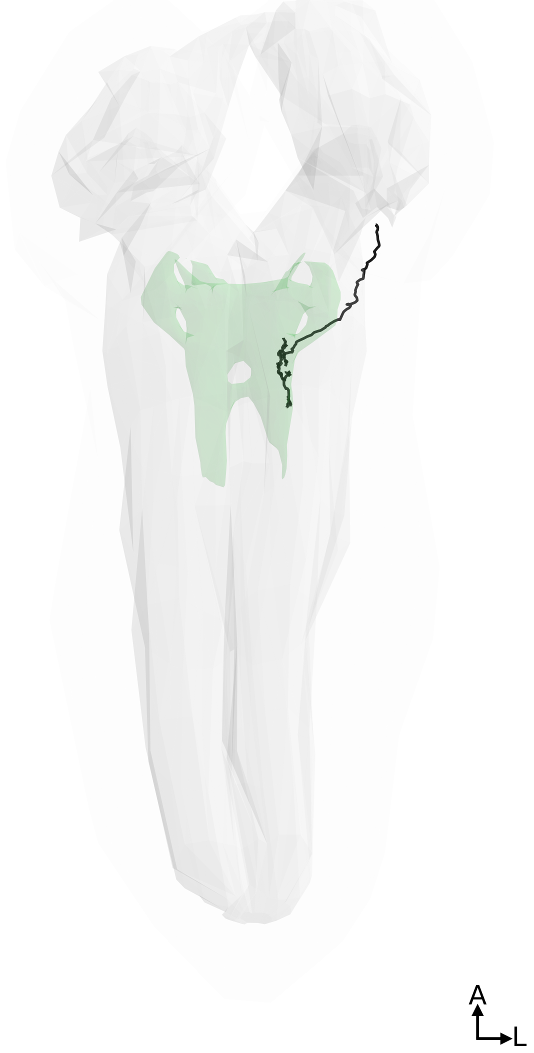

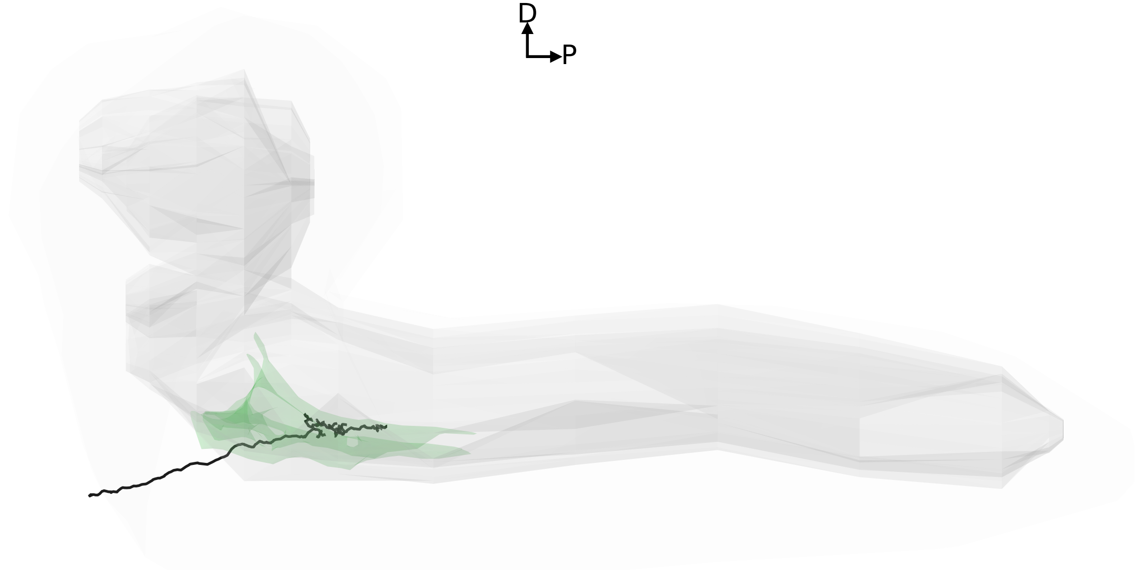

ID	*name*	SCACa	SCAVa	SCAVp	SCACal	SCACp	SCACpl	SCVM	IPCs	DMS	DH44	Se0ens	Se0ph	PMN LR	MN motor neurons	PaN motor neurons	olfactory PNs	gustatory PNs	multiglomerular PNs	unknown PNs	thermo PNs	visual PNs
15639275	MN-R-Sens-B3-VM-08	0	0	0	0	0	0	4	0	0	0	0	0	0	0	0	0	0	0	0	0	0

ID: 15671848
name: MN-R-Sens-B3-VM-09

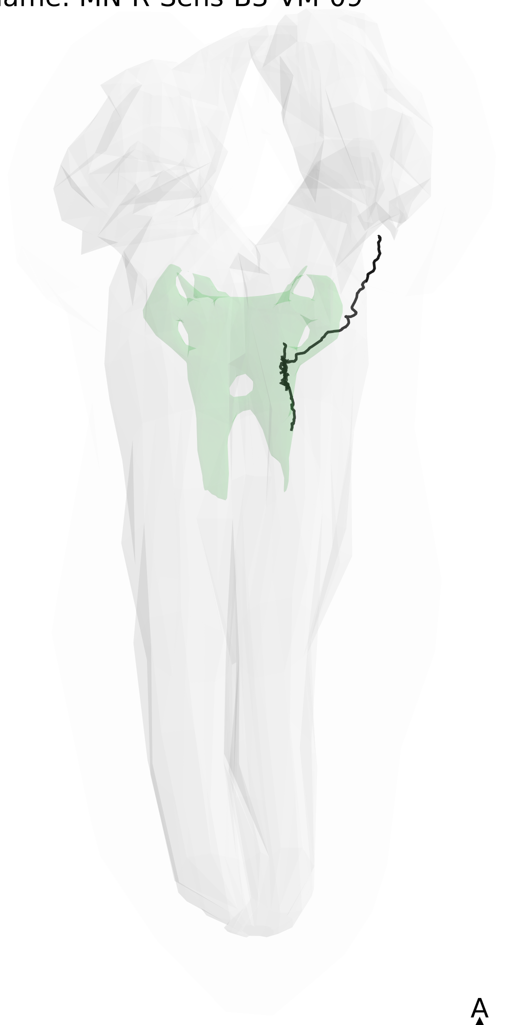

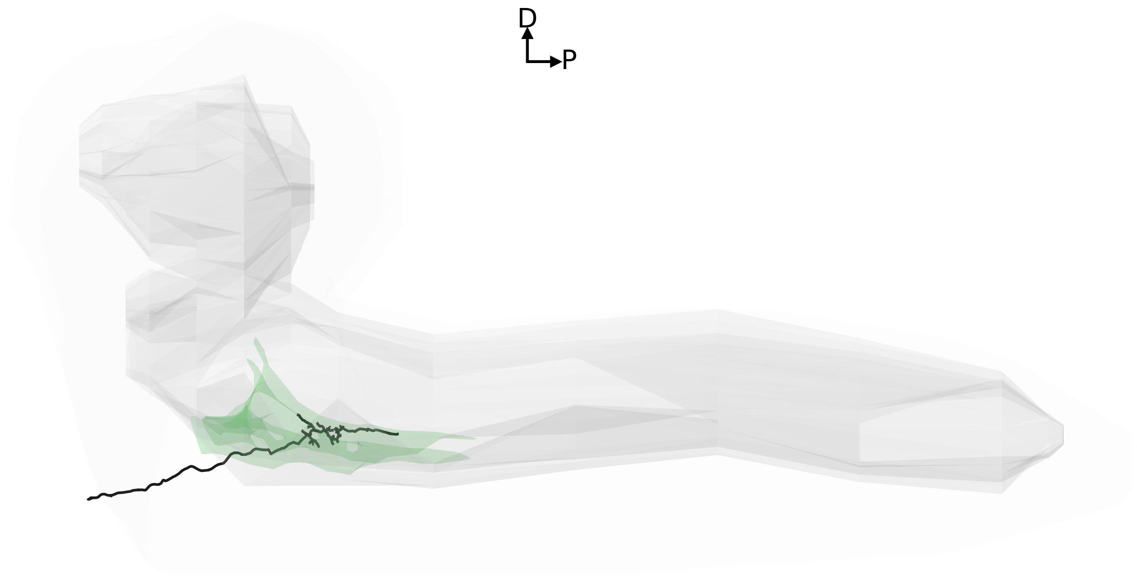

ID	name	SCACa	SCAVa	SCAVp	SCACal	SCACp	SCACpl	SCVM	IPCs	DMS	DH44	Se0ens	Se0ph	PMN LR	MN motor neurons	PaN motor neurons	olfactory PNs	gustatory PNs	multiglomerular PNs	unknown PNs	thermo PNs	visual PNs
15671848	MN-R-Sens-B3-VM-09	0	0	0	0	0	0	4	0	0	0	0	0	0	0	0	0	0	0	0	0	0

ID: 15639548
name: MN-R-Sens-B3-VM-10

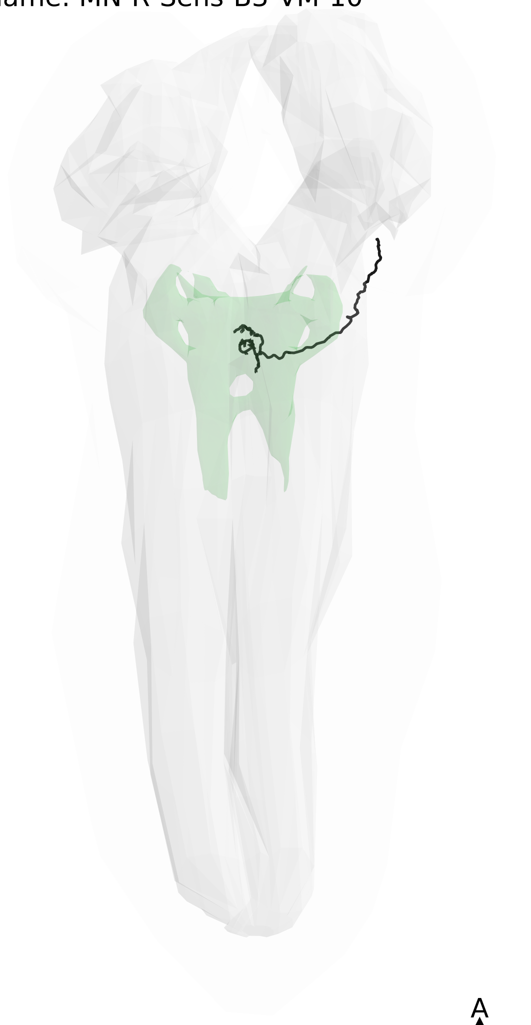

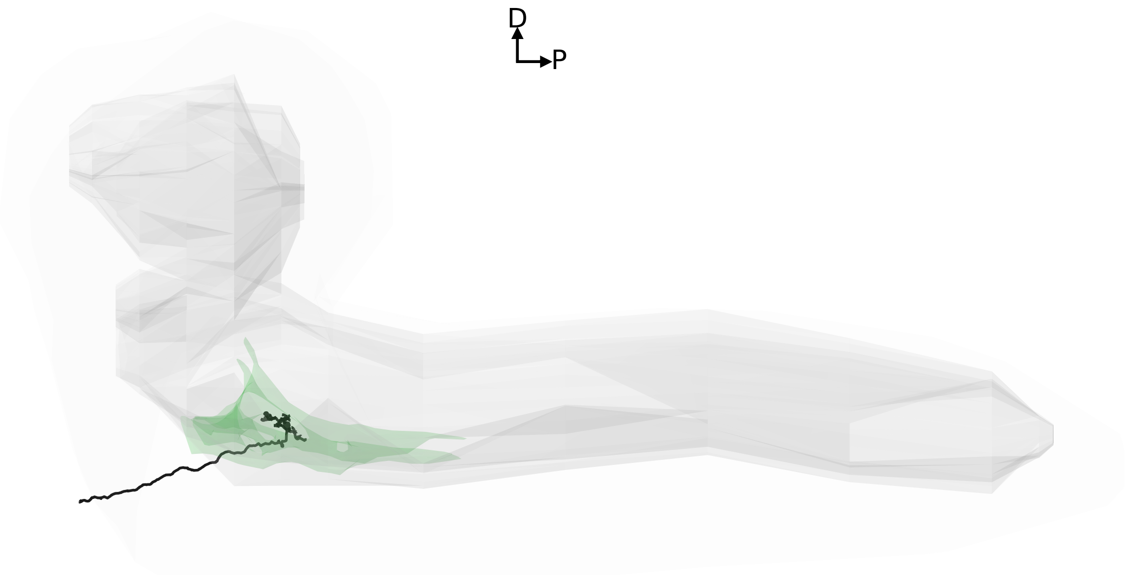

ID	*name*	SCACa	SCAVa	SCAVp	SCACal	SCACp	SCACpl	SCVM	IPCs	DMS	DH44	Se0ens	Se0ph	PMN LR	MN motor neurons	PaN motor neurons	olfactory PNs	gustatory PNs	multiglomerular PNs	unknown PNs	thermo PNs	visual PNs
15639548	MN-R-Sens-B3-VM-10	0	0	0	0	0	0	3	0	0	0	0	0	0	0	0	0	0	0	0	0	0

ID: 15640724
name: MN-R-Sens-B3-VM-11

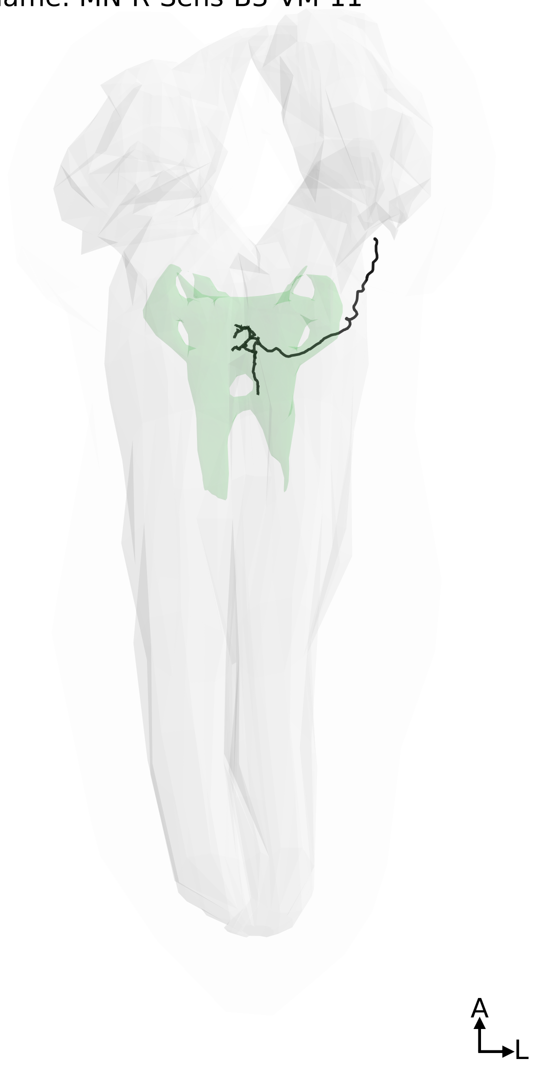

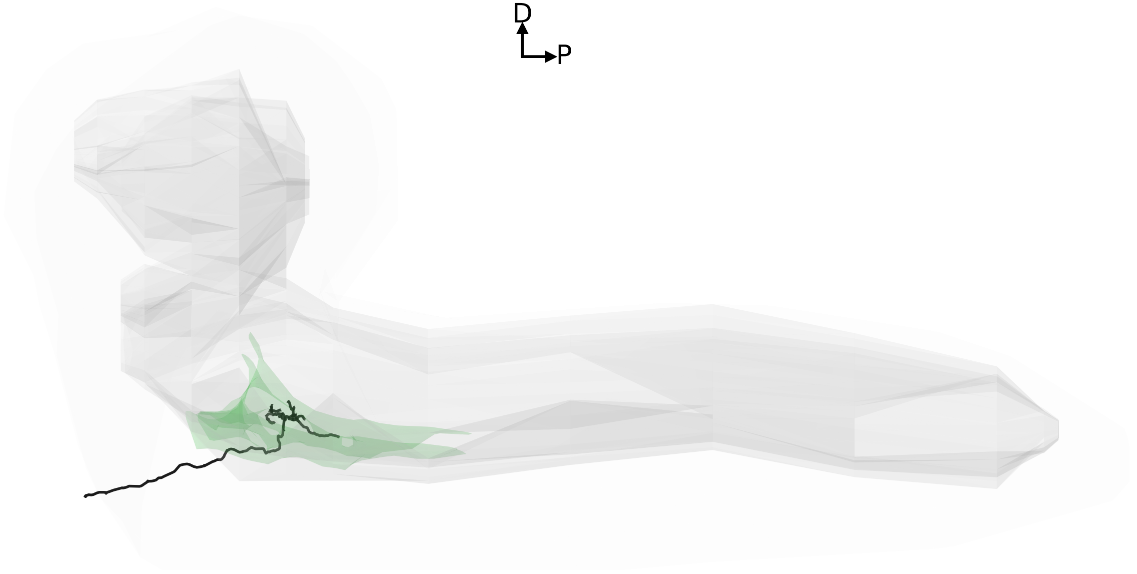

ID	*name*	SCACa	SCAVa	SCAVp	SCACal	SCACp	SCACpl	SCVM	IPCs	DMS	DH44	Se0ens	Se0ph	PMN LR	MN motor neurons	PaN motor neurons	olfactory PNs	gustatory PNs	multiglomerular PNs	unknown PNs	thermo PNs	visual PNs
15640724	MN-R-Sens-B3-VM-11	0	0	0	0	0	0	4	0	0	0	0	0	0	0	1	0	0	0	0	0	0

ID: 15672957
name: MN-R-Sens-B3-VM-12

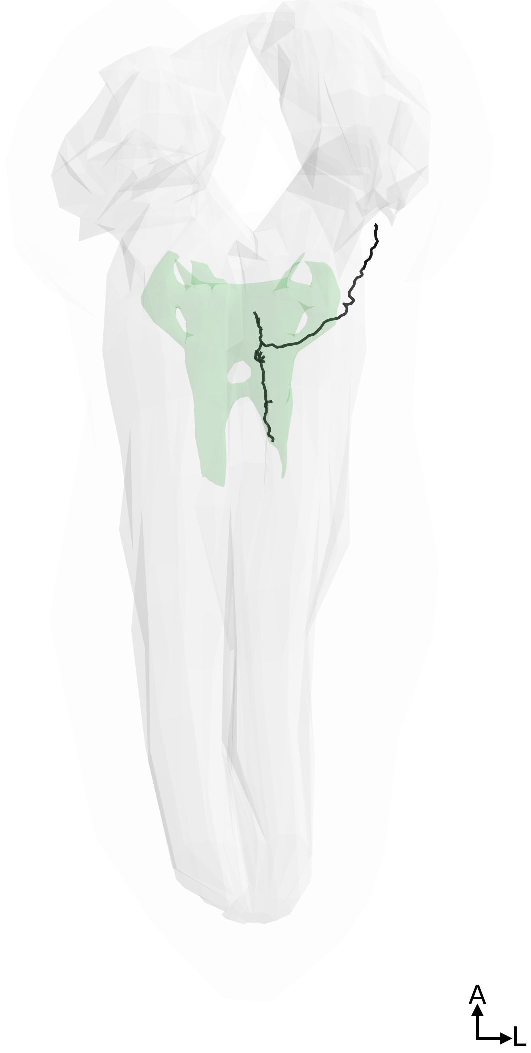

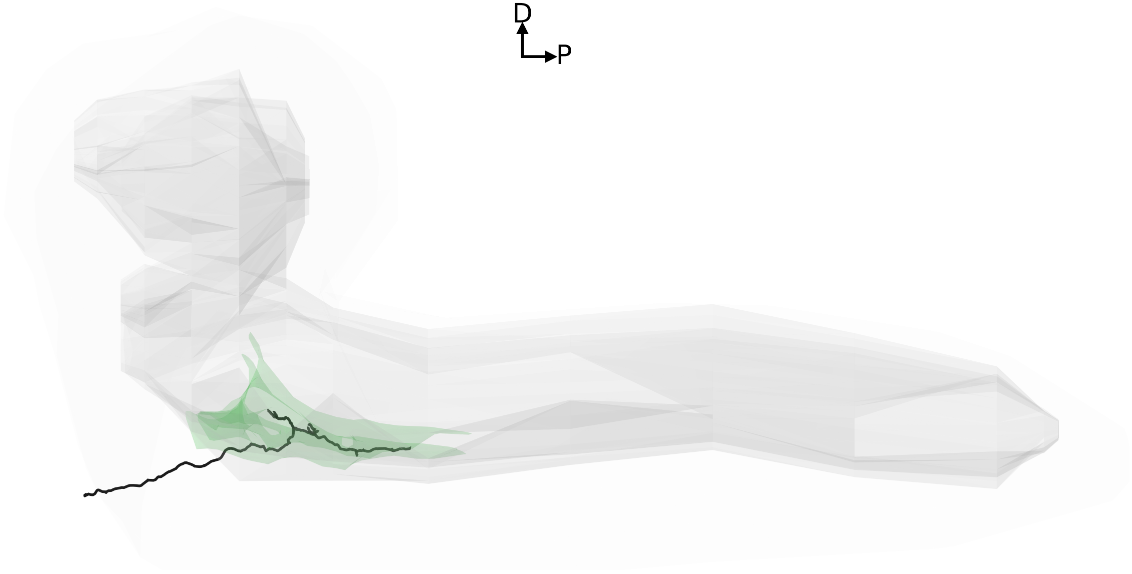

ID	*name*	SCACa	SCAVa	SCAVp	SCACal	SCACp	SCACpl	SCVM	IPCs	DMS	DH44	Se0ens	Se0ph	PMN LR	MN motor neurons	PaN motor neurons	olfactory PNs	gustatory PNs	multiglomerular PNs	unknown PNs	thermo PNs	visual PNs
15672957	MN-R-Sens-B3-VM-12	0	0	0	0	0	0	7	0	0	0	0	0	0	0	0	0	0	0	0	0	0

ID: 15640567
name: MN-R-Sens-B3-VM-13

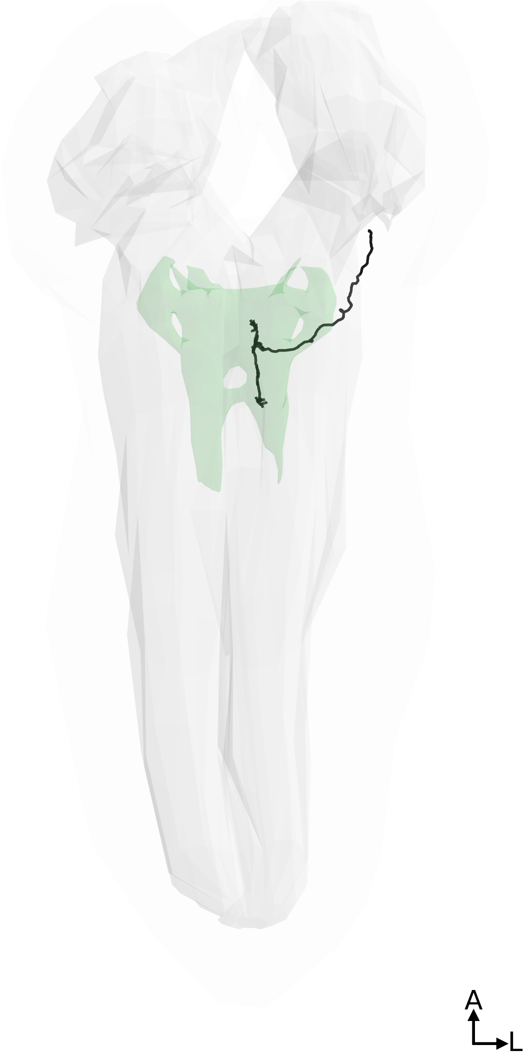

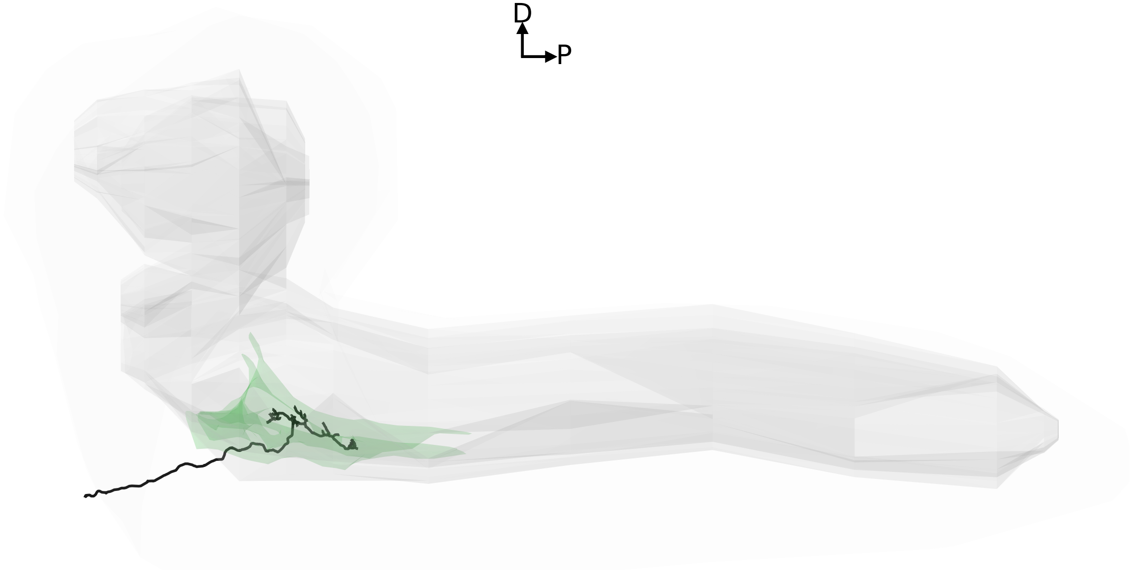

ID	*name*	SCACa	SCAVa	SCAVp	SCACal	SCACp	SCACpl	SCVM	IPCs	DMS	DH44	Se0ens	Se0ph	PMN LR	MN motor neurons	PaN motor neurons	olfactory PNs	gustatory PNs	multiglomerular PNs	unknown PNs	thermo PNs	visual PNs
15640567	MN-R-Sens-B3-VM-13	0	0	0	0	0	0	4	0	0	0	0	0	0	0	0	0	0	0	0	0	0

ID: 15914721
name: MN-R-Sens-B3-VM-14

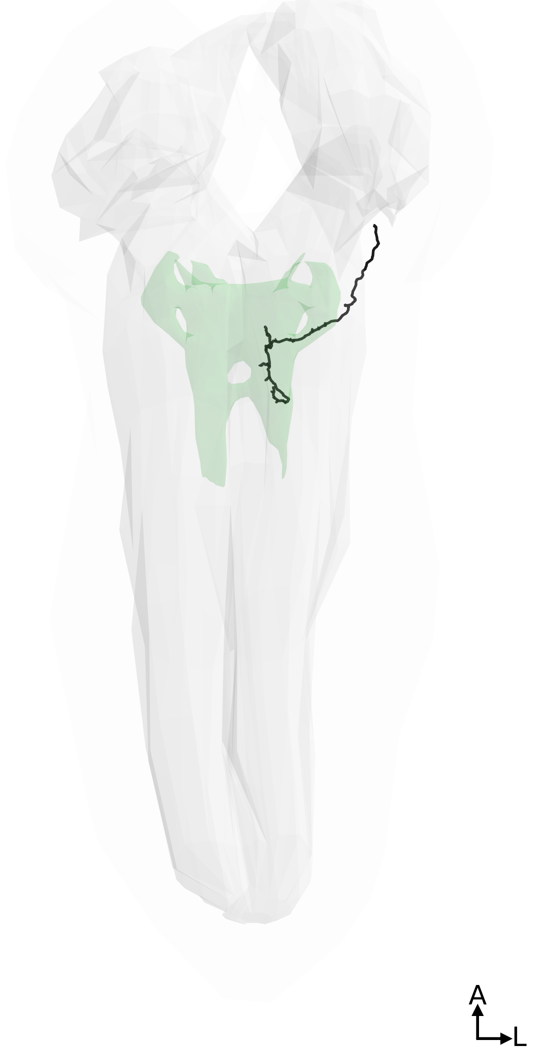

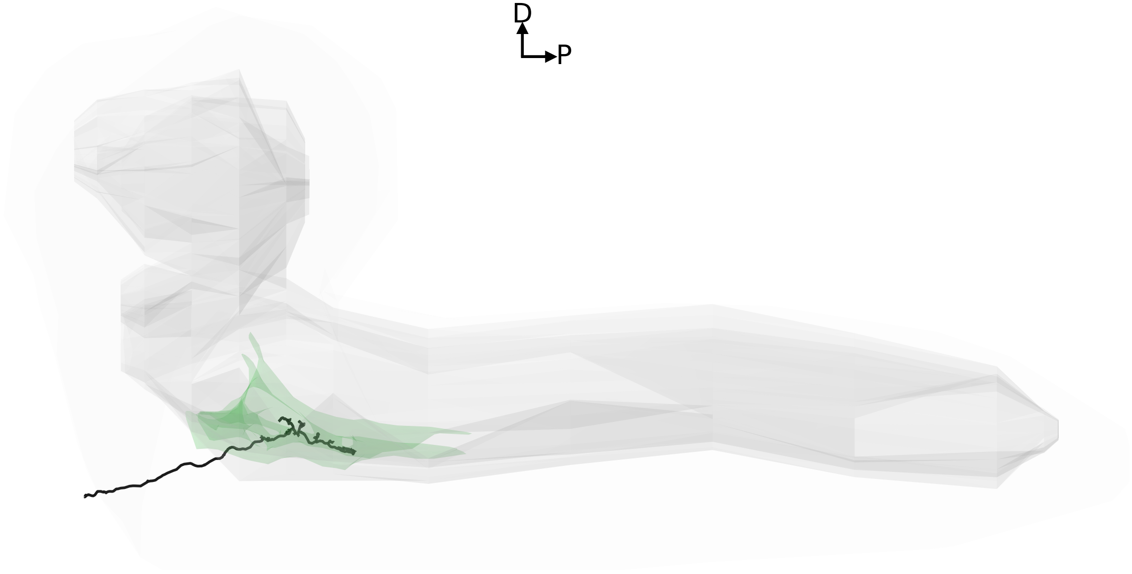

ID	*name*	SCACa	SCAVa	SCAVp	SCACal	SCACp	SCACpl	SCVM	IPCs	DMS	DH44	Se0ens	Se0ph	PMN LR	MN motor neurons	PaN motor neurons	olfactory PNs	gustatory PNs	multiglomerular PNs	unknown PNs	thermo PNs	visual PNs
15914721	MN-R-Sens-B3-VM-14	0	0	0	0	0	0	5	0	0	0	0	0	0	0	2	0	0	0	0	0	0

ID: 2495308
name: PaN-L-Sens-B1-VM-01

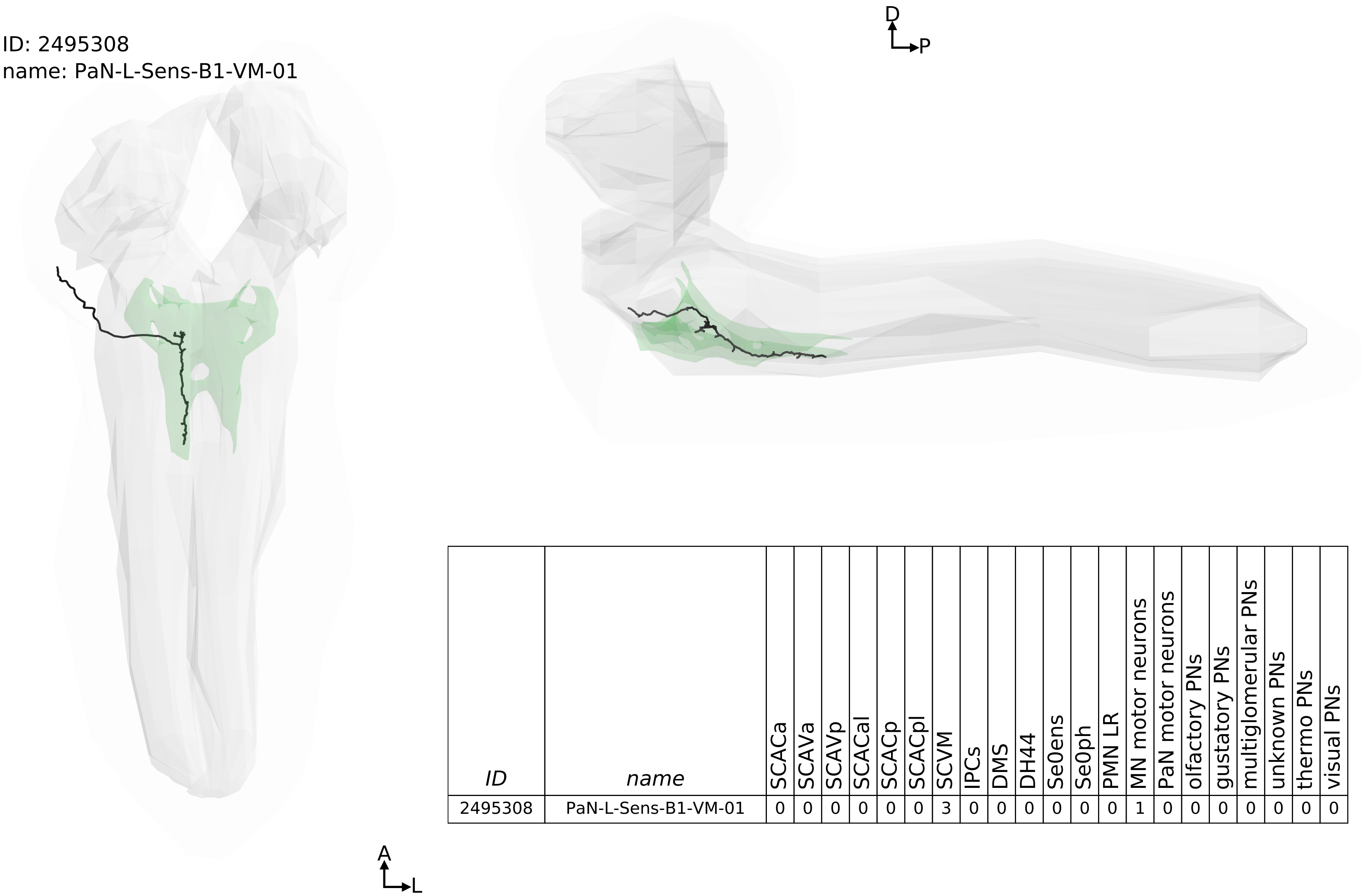

ID	*name*	SCACa	SCAVa	SCAVp	SCACal	SCACp	SCACpl	SCVM	IPCs	DMS	DH44	Se0ens	Se0ph	PMN LR	MN motor neurons	PaN motor neurons	olfactory PNs	gustatory PNs	multiglomerular PNs	unknown PNs	thermo PNs	visual PNs
2495308	PaN-L-Sens-B1-VM-01	0	0	0	0	0	0	3	0	0	0	0	0	0	1	0	0	0	0	0	0	0

ID: 2668719
name: PaN-L-Sens-B1-VM-02

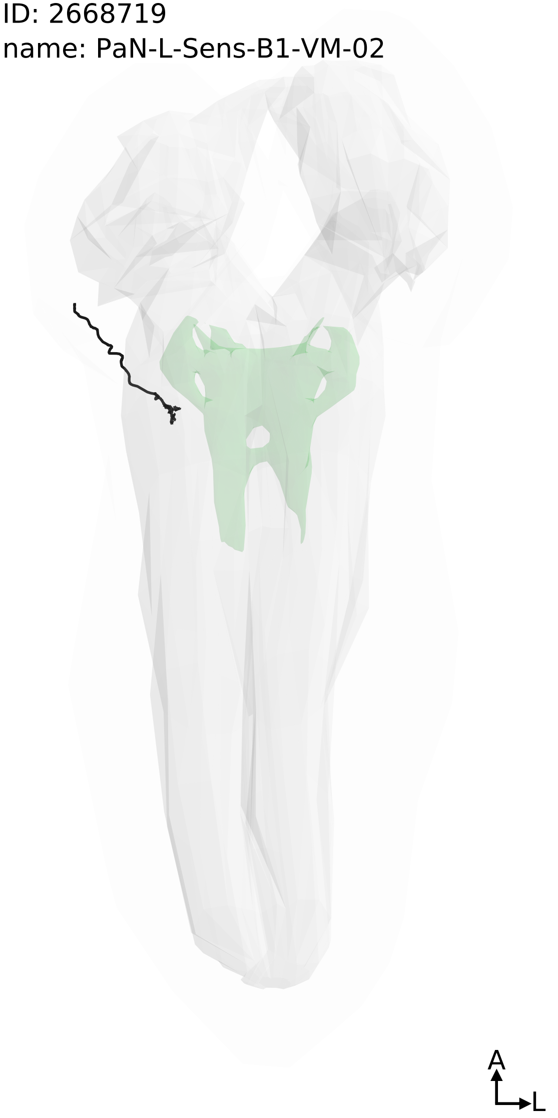

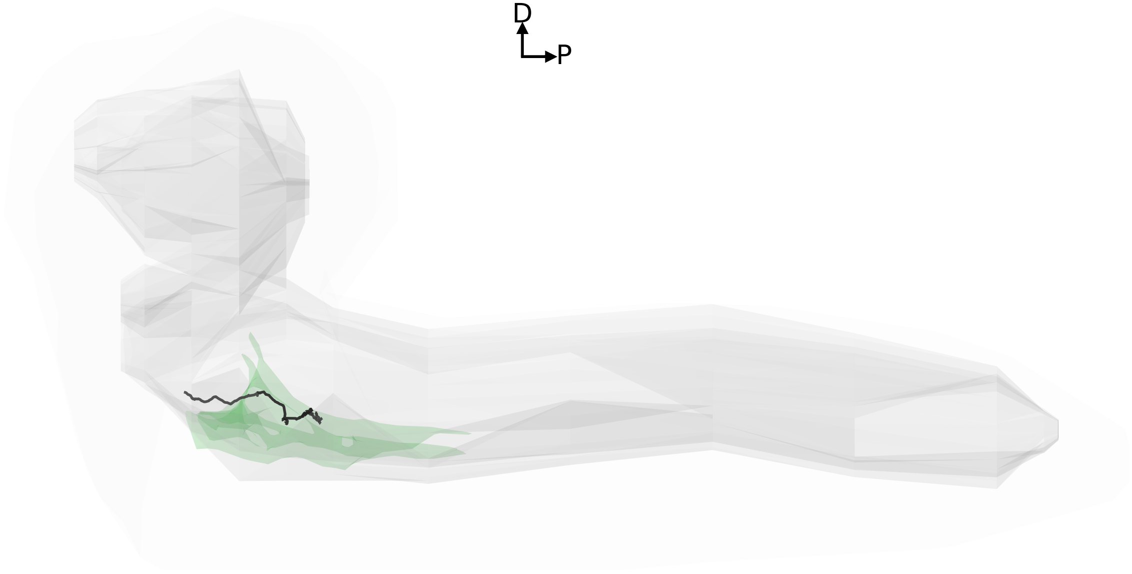

ID	name	SCACa	SCAVa	SCAVp	SCACal	SCACp	SCACpl	SCVM	IPCs	DMS	DH44	Se0ens	Se0ph	PMN LR	MN motor neurons	PaN motor neurons	olfactory PNs	gustatory PNs	multiglomerular PNs	unknown PNs	thermo PNs	visual PNs
2668719	PaN-L-Sens-B1-VM-02	0	0	0	0	0	0	2	0	0	0	0	0	0	0	0	0	0	0	0	0	0

ID: 2669832
name: PaN-L-Sens-B1-VM-03

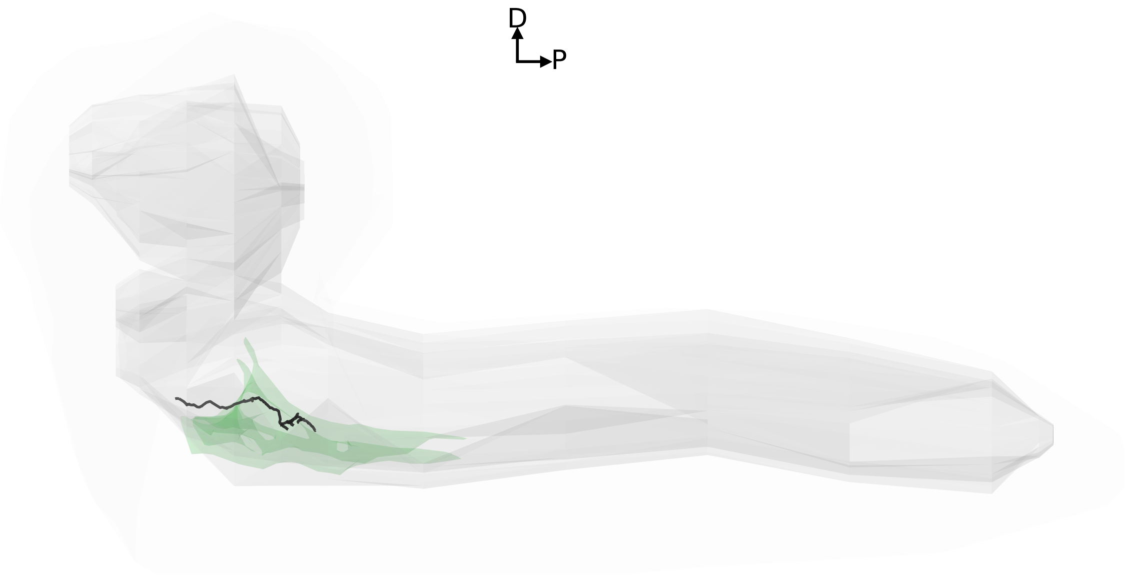

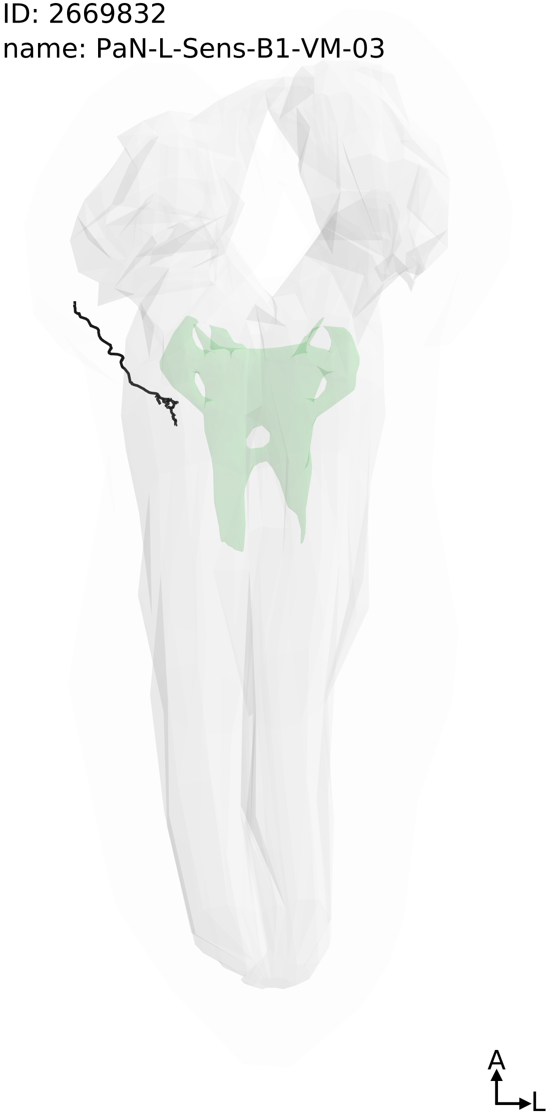

ID	*name*	SCACa	SCAVa	SCAVp	SCACal	SCACp	SCACpl	SCVM	IPCs	DMS	DH44	Se0ens	Se0ph	PMN LR	MN motor neurons	PaN motor neurons	olfactory PNs	gustatory PNs	multiglomerular PNs	unknown PNs	thermo PNs	visual PNs
2669832	PaN-L-Sens-B1-VM-03	0	0	0	0	0	0	2	0	0	0	0	0	0	0	0	0	0	0	0	0	0

ID: 2505675
name: PaN-L-Sens-B1-VM-04

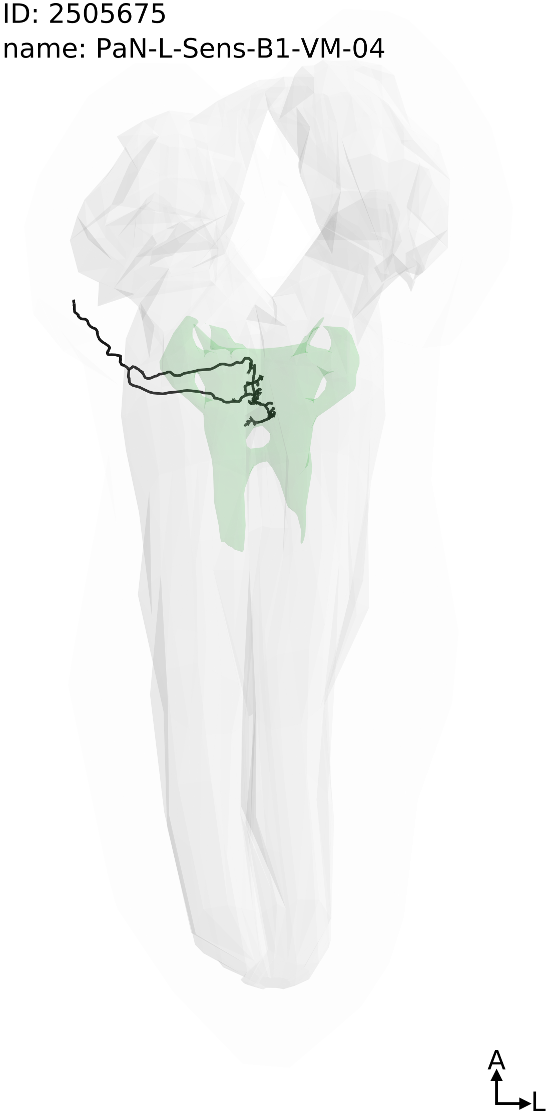

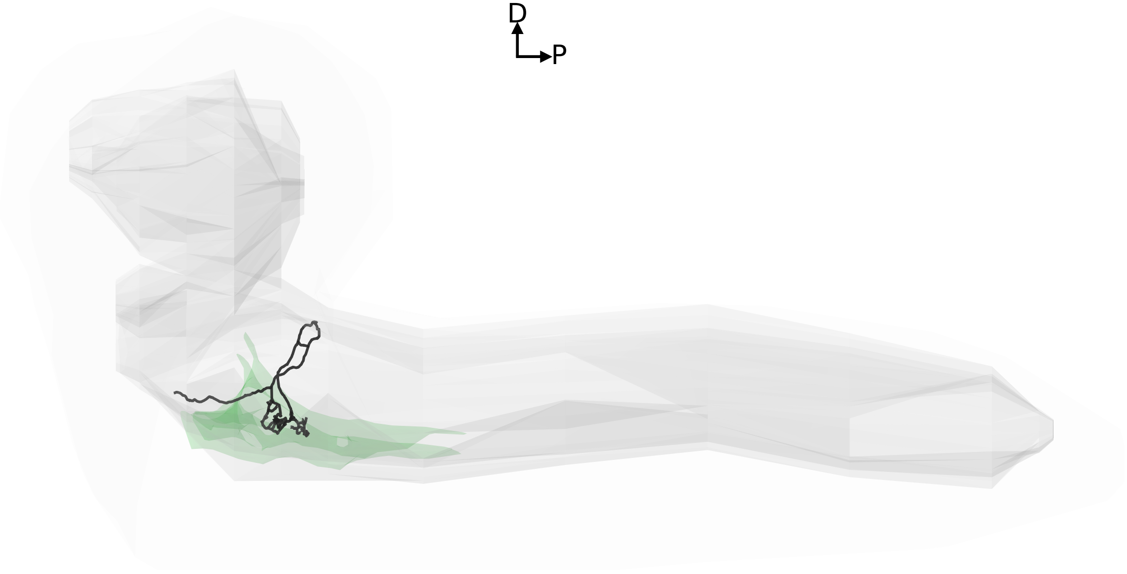

ID	*name*	SCACa	SCAVa	SCAVp	SCACal	SCACp	SCACpl	SCVM	IPCs	DMS	DH44	Se0ens	Se0ph	PMN LR	MN motor neurons	PaN motor neurons	olfactory PNs	gustatory PNs	multiglomerular PNs	unknown PNs	thermo PNs	visual PNs
2505675	PaN-L-Sens-B1-VM-04	0	0	0	0	0	0	6	0	0	0	0	0	0	0	0	0	0	0	0	0	0

ID: 2305234
name: PaN-R-Sens-B1-VM-01

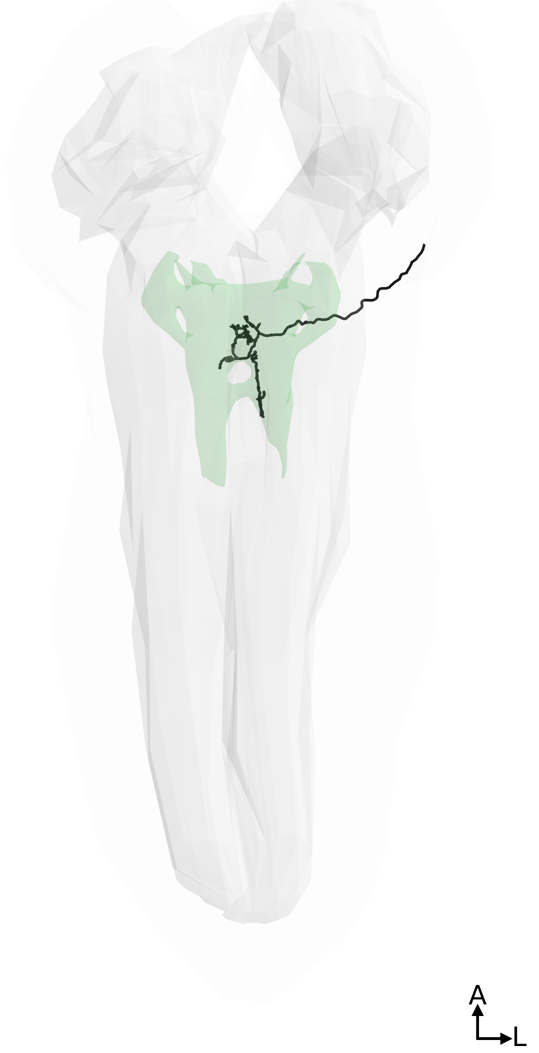

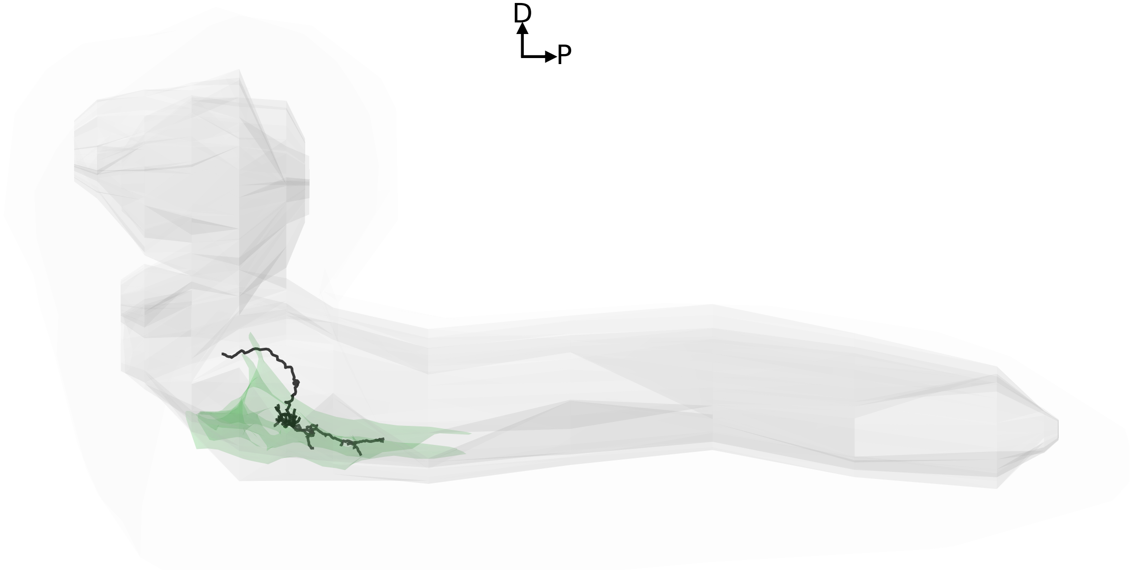

ID	*name*	SCACa	SCAVa	SCAVp	SCACal	SCACp	SCACpl	SCVM	IPCs	DMS	DH44	Se0ens	Se0ph	PMN LR	MN motor neurons	PaN motor neurons	olfactory PNs	gustatory PNs	multiglomerular PNs	unknown PNs	thermo PNs	visual PNs
2305234	PaN-R-Sens-B1-VM-01	0	0	0	0	0	0	10	0	0	0	0	0	0	0	0	0	0	0	0	0	0

ID: 2268281
name: PaN-R-Sens-B1-VM-02

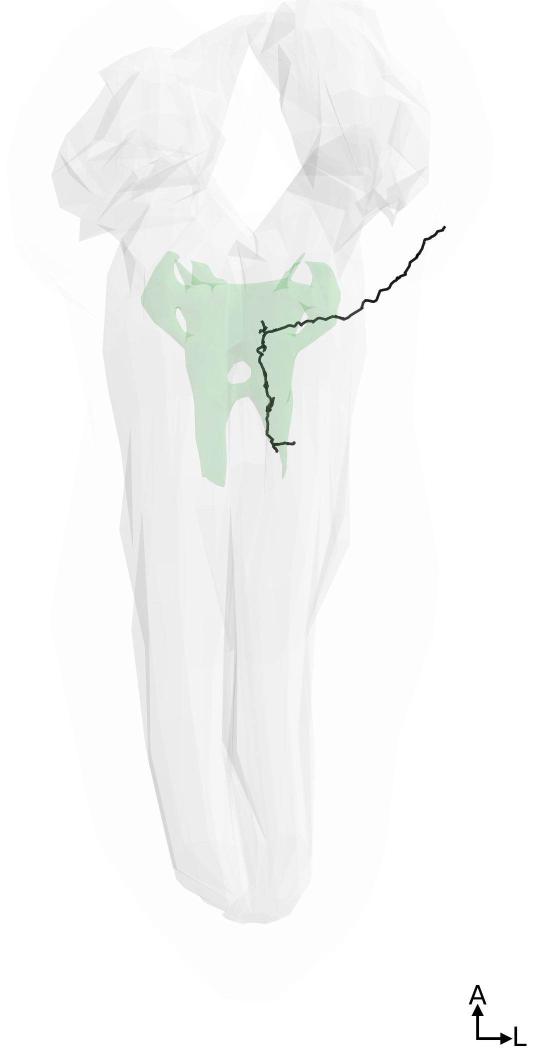

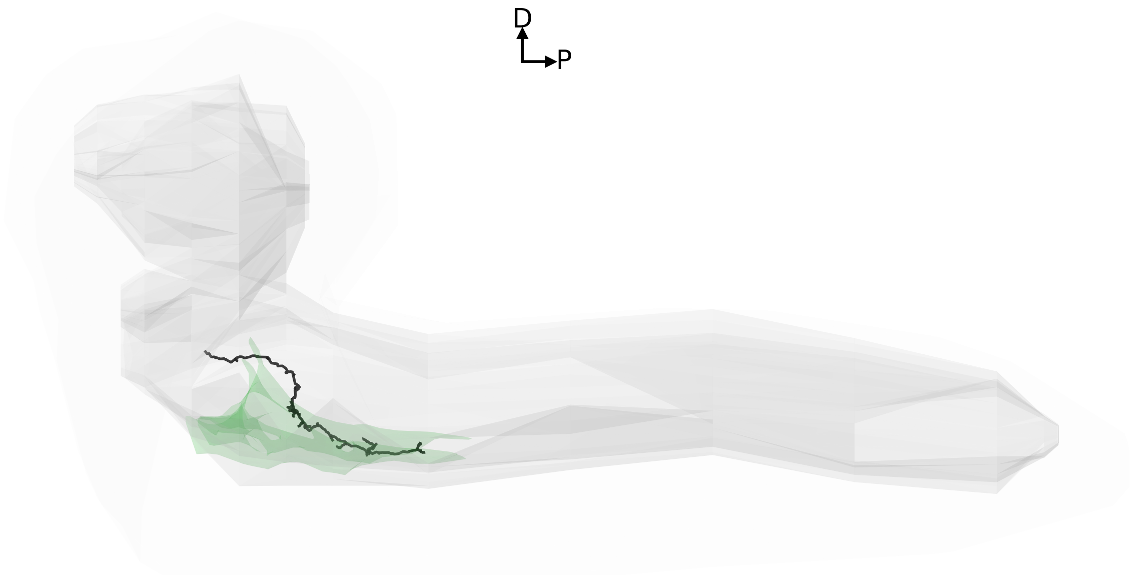

ID	*name*	SCACa	SCAVa	SCAVp	SCACal	SCACp	SCACpl	SCVM	IPCs	DMS	DH44	Se0ens	Se0ph	PMN LR	MN motor neurons	PaN motor neurons	olfactory PNs	gustatory PNs	multiglomerular PNs	unknown PNs	thermo PNs	visual PNs
2268281	PaN-R-Sens-B1-VM-02	0	0	0	0	0	0	7	0	0	0	0	0	0	6	2	0	0	0	0	0	0

ID: 16123983
name: PaN-R-Sens-B1-VM-03

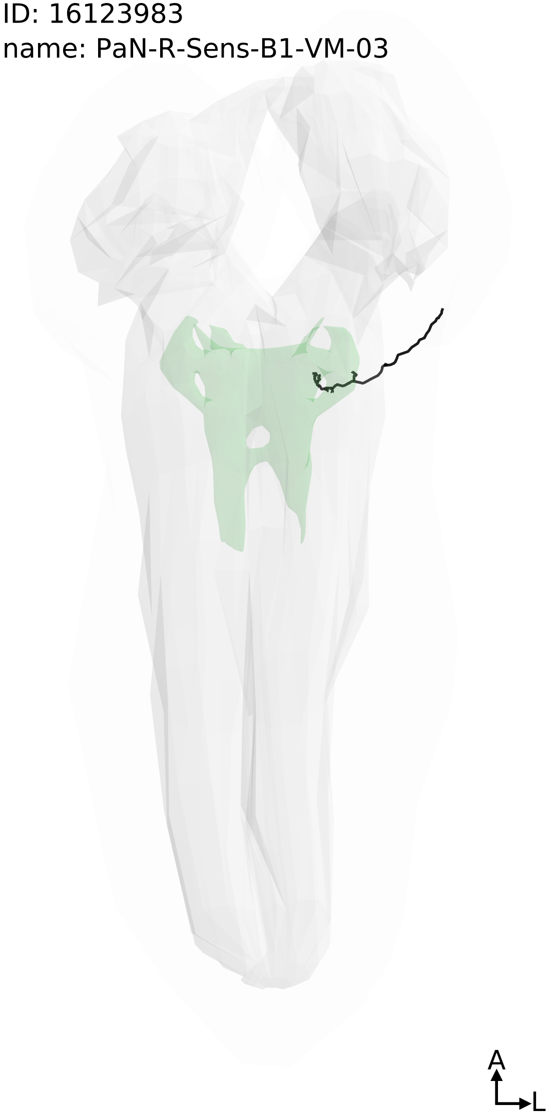

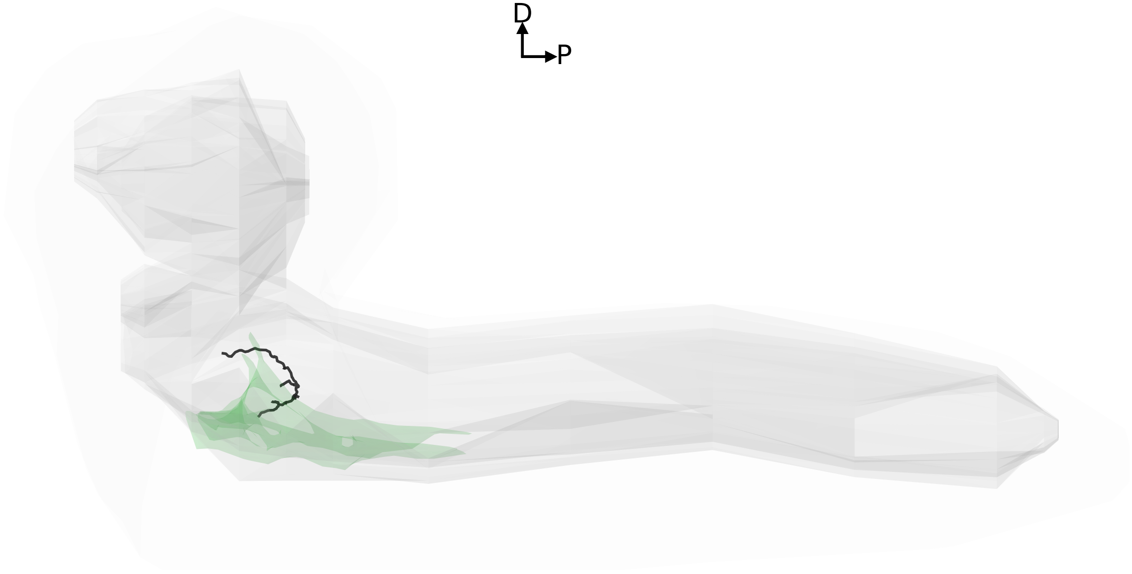

ID	*name*	SCACa	SCAVa	SCAVp	SCACal	SCACp	SCACpl	SCVM	IPCs	DMS	DH44	Se0ens	Se0ph	PMN LR	MN motor neurons	PaN motor neurons	olfactory PNs	gustatory PNs	multiglomerular PNs	unknown PNs	thermo PNs	visual PNs
16123983	PaN-R-Sens-B1-VM-03	0	0	0	0	0	0	0	0	0	0	0	0	0	0	0	0	0	0	0	0	0

ID: 16124048
name: PaN-R-Sens-B1-VM-04

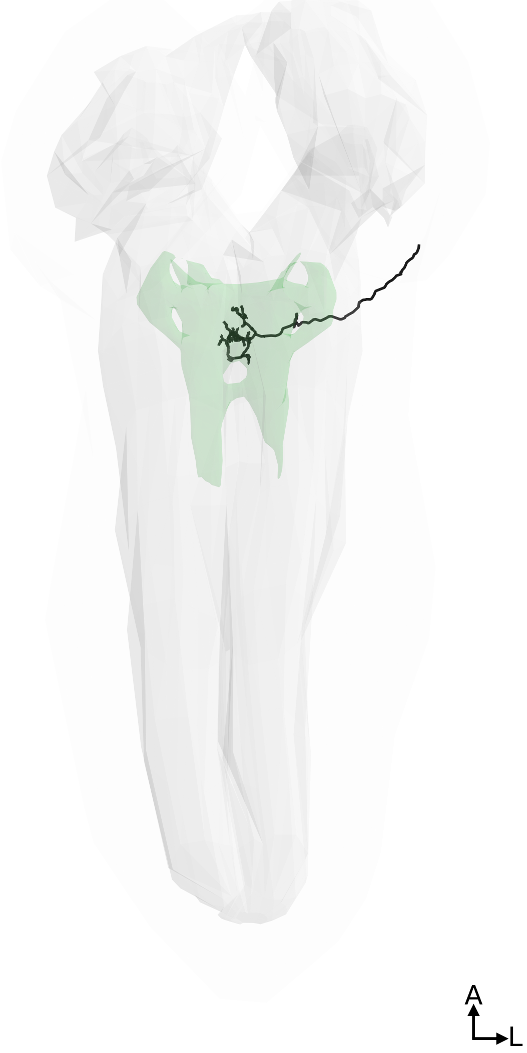

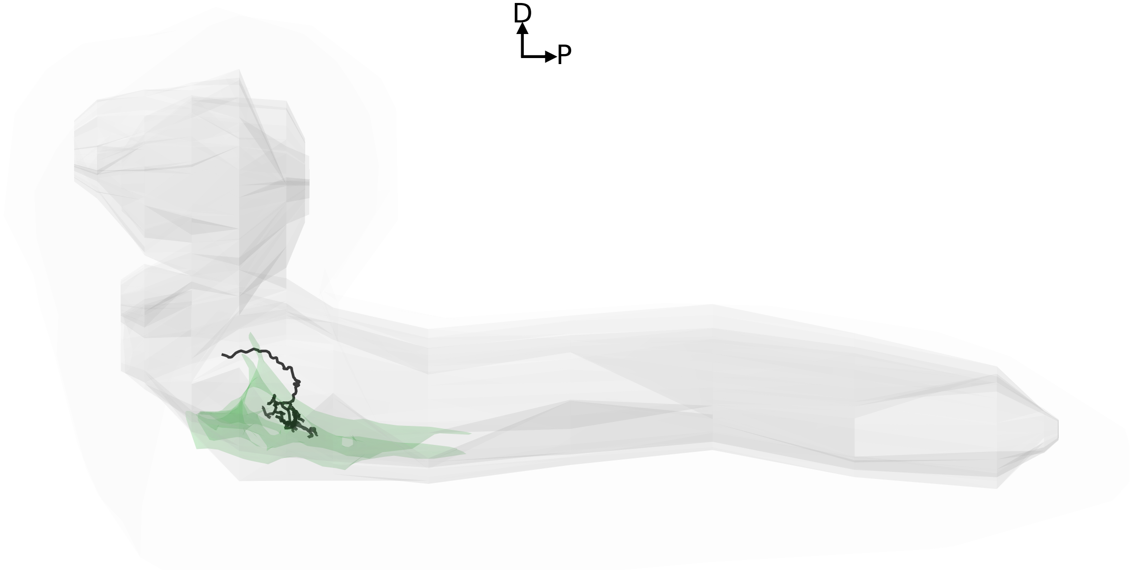

ID	*name*	SCACa	SCAVa	SCAVp	SCACal	SCACp	SCACpl	SCVM	IPCs	DMS	DH44	Se0ens	Se0ph	PMN LR	MN motor neurons	PaN motor neurons	olfactory PNs	gustatory PNs	multiglomerular PNs	unknown PNs	thermo PNs	visual PNs
16124048	PaN-R-Sens-B1-VM-04	0	0	0	0	0	0	10	0	0	0	0	0	0	0	0	0	0	0	0	0	0

ID: 16124082
name: PaN-R-Sens-B1-VM-05

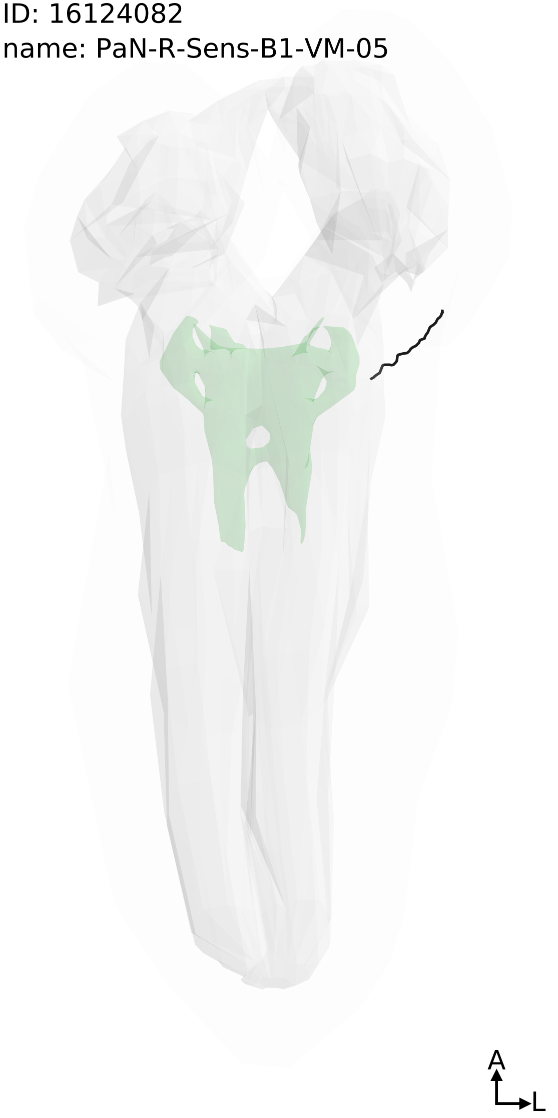

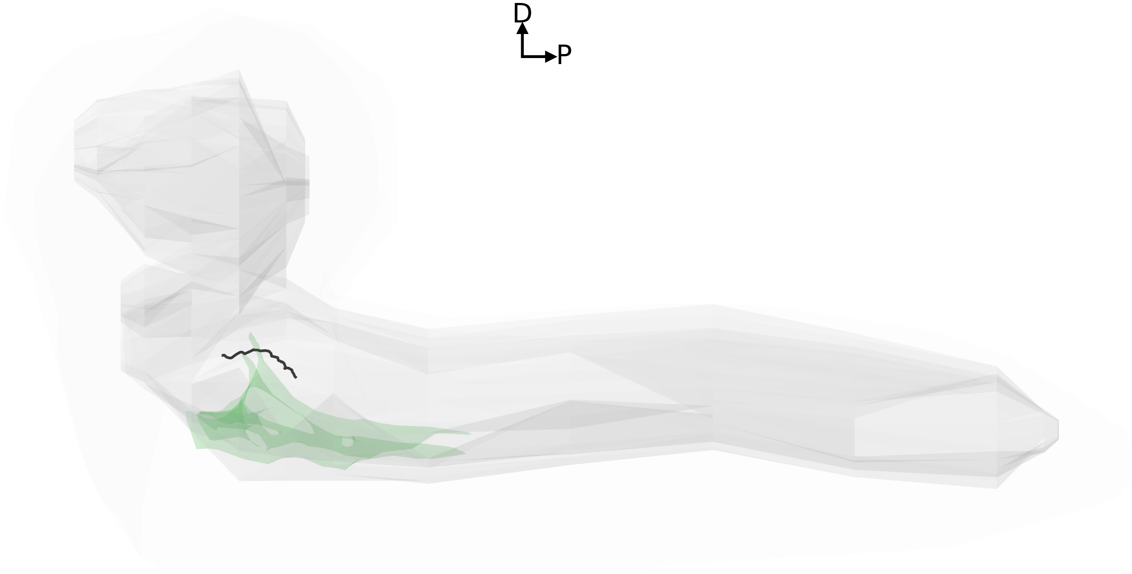

ID	name	SCACa	SCAVa	SCAVp	SCACal	SCACp	SCACpl	SCVM	IPCs	DMS	DH44	Se0ens	Se0ph	PMN LR	MN motor neurons	PaN motor neurons	olfactory PNs	gustatory PNs	multiglomerular PNs	unknown PNs	thermo PNs	visual PNs
16124082	PaN-R-Sens-B1-VM-05	0	0	0	0	0	0	0	0	0	0	0	0	0	0	0	0	0	0	0	0	0

ID: 2302816
name: PaN-R-Sens-B2-VM-01

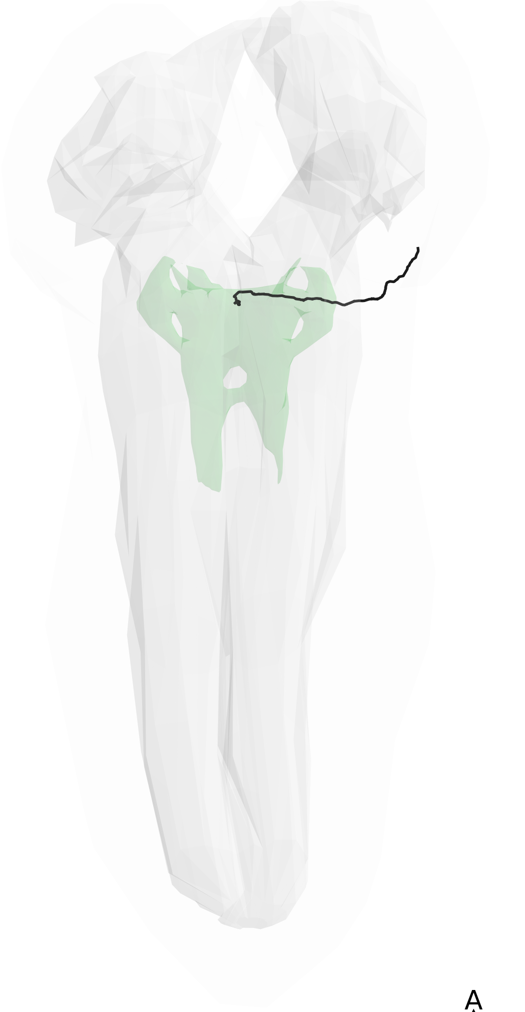

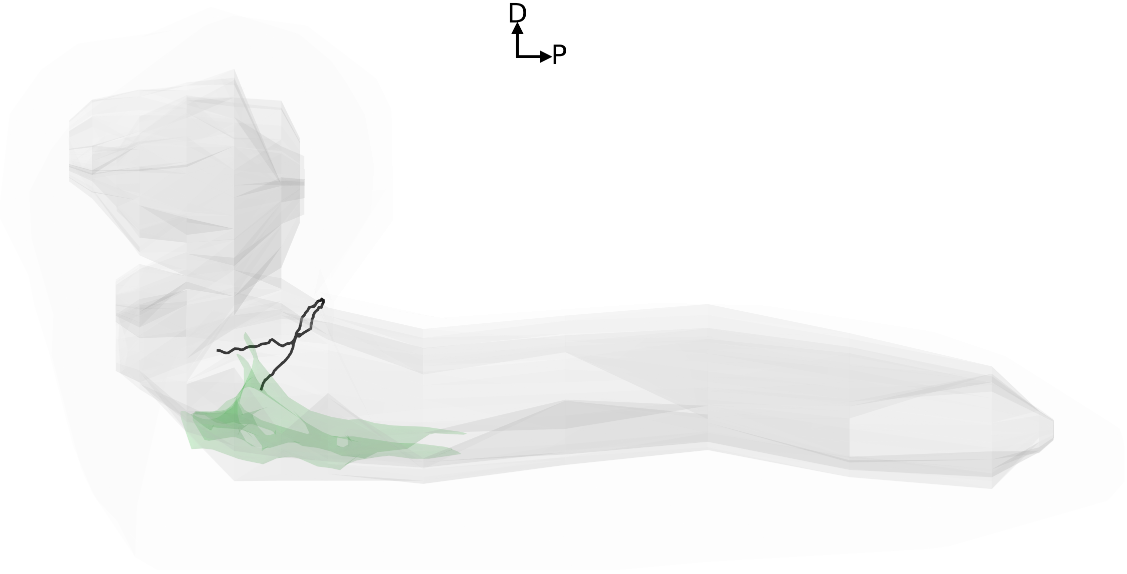

ID	*name*	SCACa	SCAVa	SCAVp	SCACal	SCACp	SCACpl	SCVM	IPCs	DMS	DH44	Se0ens	Se0ph	PMN LR	MN motor neurons	PaN motor neurons	olfactory PNs	gustatory PNs	multiglomerular PNs	unknown PNs	thermo PNs	visual PNs
2302816	PaN-R-Sens-B2-VM-01	0	0	0	0	0	0	0	0	0	0	0	0	0	0	0	0	0	0	0	0	0

ID: 2799380
name: Pan-L-Sens-B2-VM-01

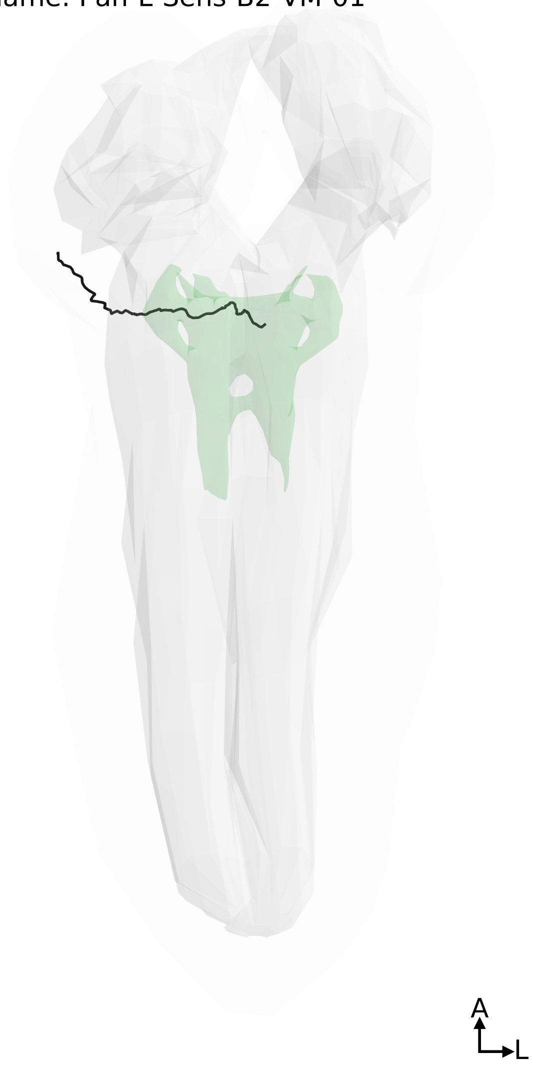

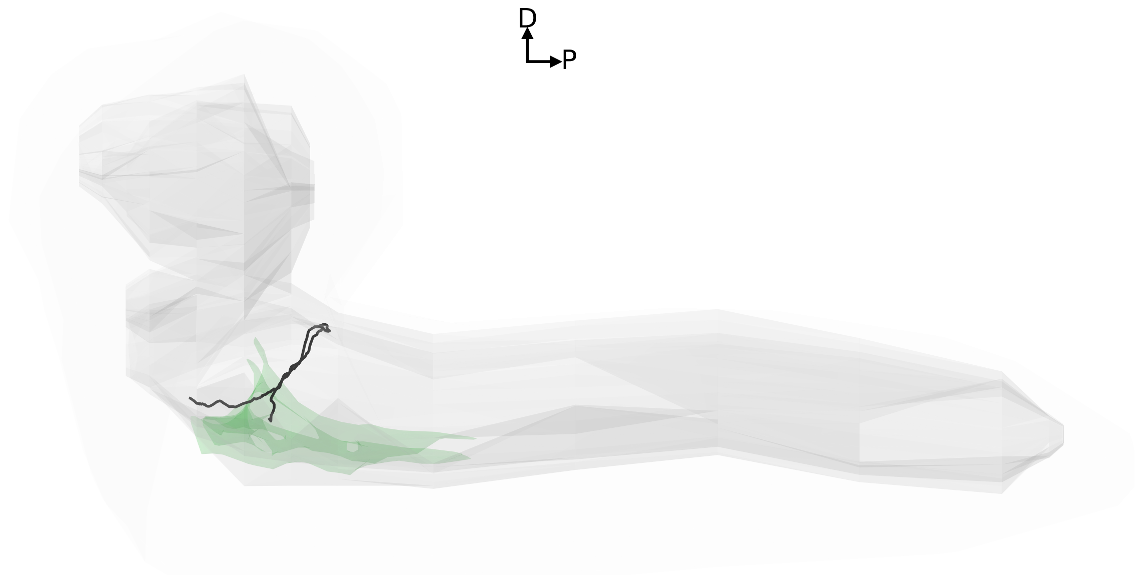

ID	*name*	SCACa	SCAVa	SCAVp	SCACal	SCACp	SCACpl	SCVM	IPCs	DMS	DH44	Se0ens	Se0ph	PMN LR	MN motor neurons	PaN motor neurons	olfactory PNs	gustatory PNs	multiglomerular PNs	unknown PNs	thermo PNs	visual PNs
2799380	Pan-L-Sens-B2-VM-01	0	0	0	0	0	0	0	0	0	0	0	0	0	0	0	0	0	0	0	0	0

ID: 2524026
name: Pan-L-Sens-B2-VM-02

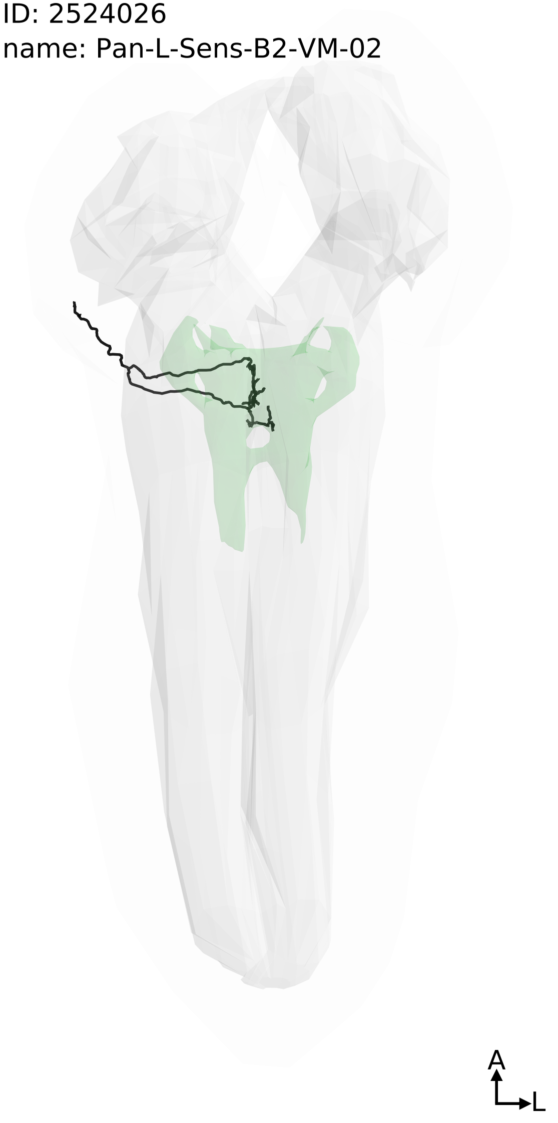

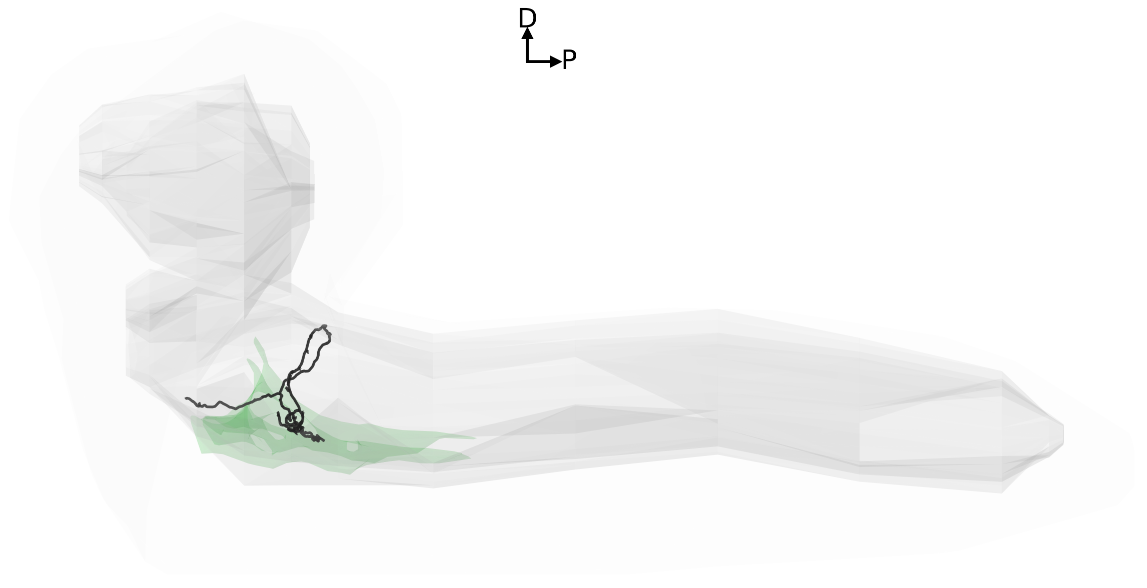

ID	*name*	SCACa	SCAVa	SCAVp	SCACal	SCACp	SCACpl	SCVM	IPCs	DMS	DH44	Se0ens	Se0ph	PMN LR	MN motor neurons	PaN motor neurons	olfactory PNs	gustatory PNs	multiglomerular PNs	unknown PNs	thermo PNs	visual PNs
2524026	Pan-L-Sens-B2-VM-02	0	0	0	0	0	0	1	0	0	0	0	0	0	0	0	0	0	0	0	0	0

ID: 15995012
name: SE0 L1

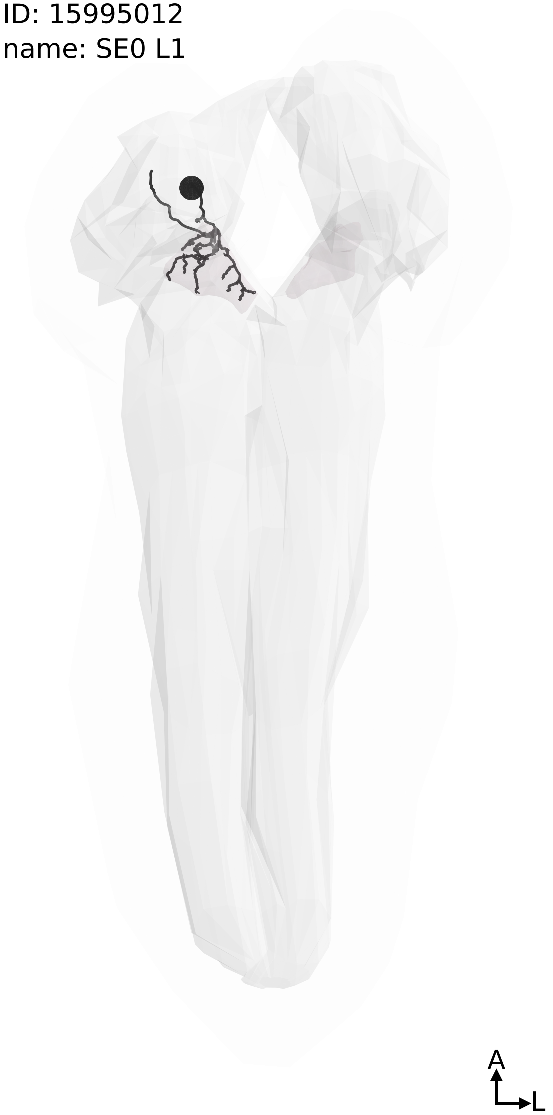

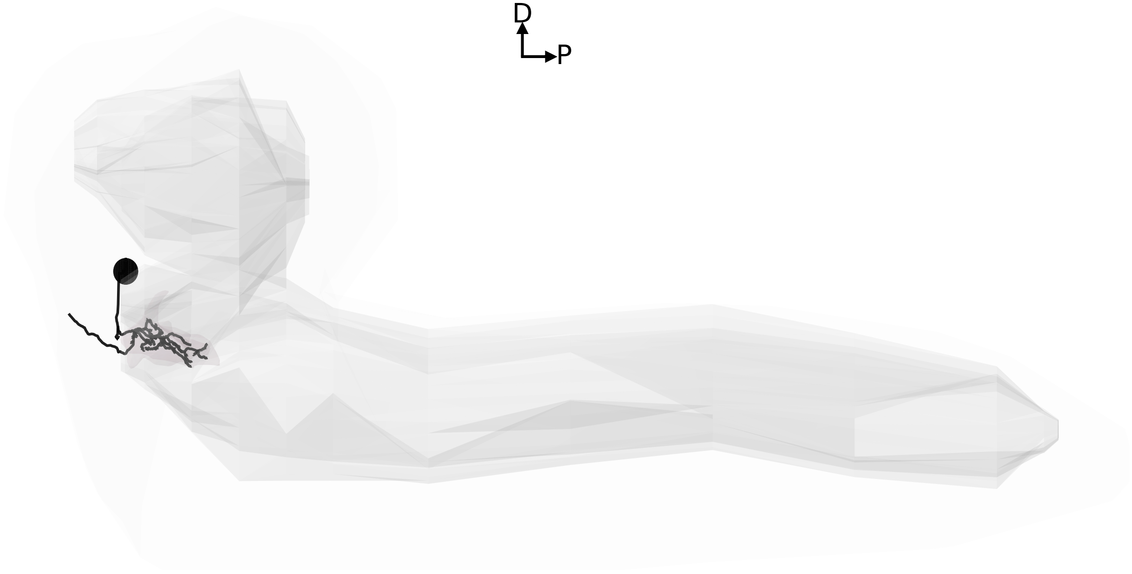

ID	name	SCACa	SCAVa	SCAVp	SCACal	SCACp	SCACpl	SCVM	IPCs	DMS	DH44	Se0ens	Se0ph	PMN LR	MN motor neurons	PaN motor neurons	olfactory PNs	gustatory PNs	multiglomerular PNs	unknown PNs	thermo PNs	visual PNs
15995012	SE0 L1	0	1	0	0	0	0	0	0	0	0	0	0	1	0	0	0	0	0	0	0	0

ID: 15983118
name: SE0 L2

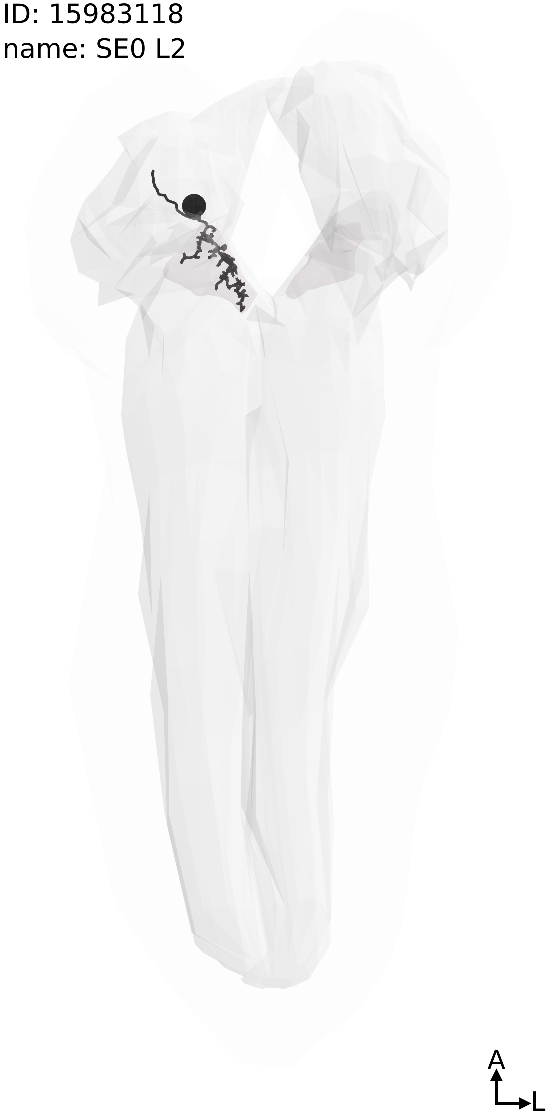

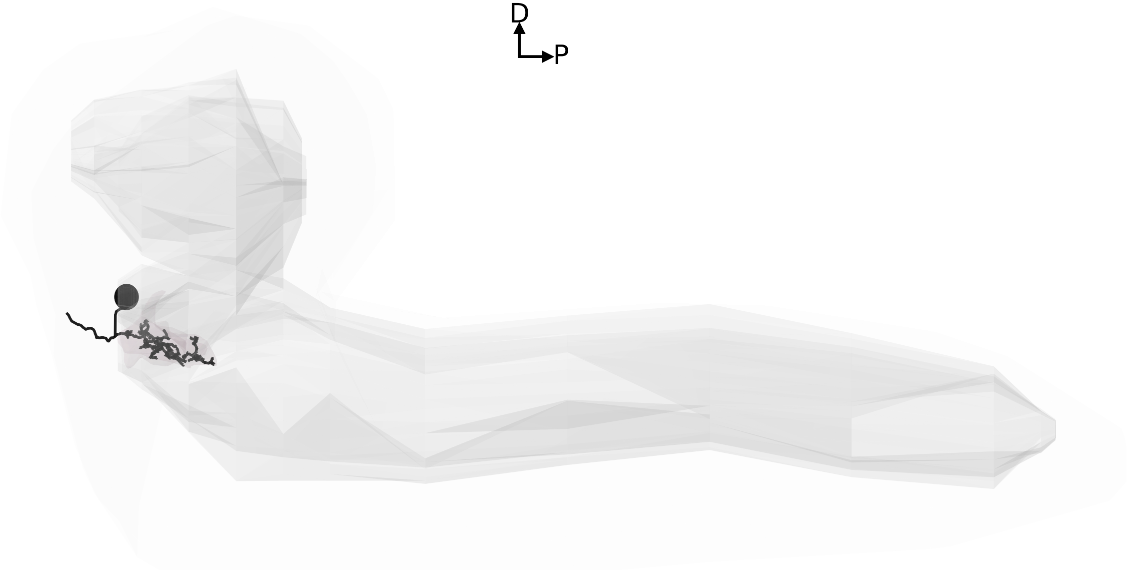

ID	*name*	SCACa	SCAVa	SCAVp	SCACal	SCACp	SCACpl	SCVM	IPCs	DMS	DH44	Se0ens	Se0ph	PMN LR	MN motor neurons	PaN motor neurons	olfactory PNs	gustatory PNs	multiglomerular PNs	unknown PNs	thermo PNs	visual PNs
15983118	SE0 L2	0	0	0	0	0	0	0	0	0	0	0	0	0	0	0	0	0	0	0	0	0

ID: 15769638
name: SE0 L3

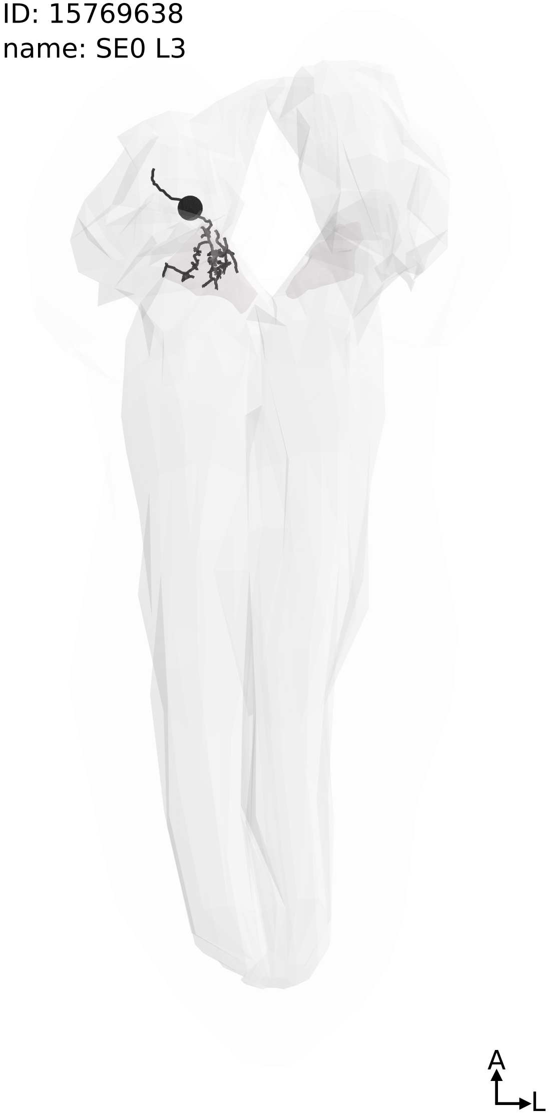

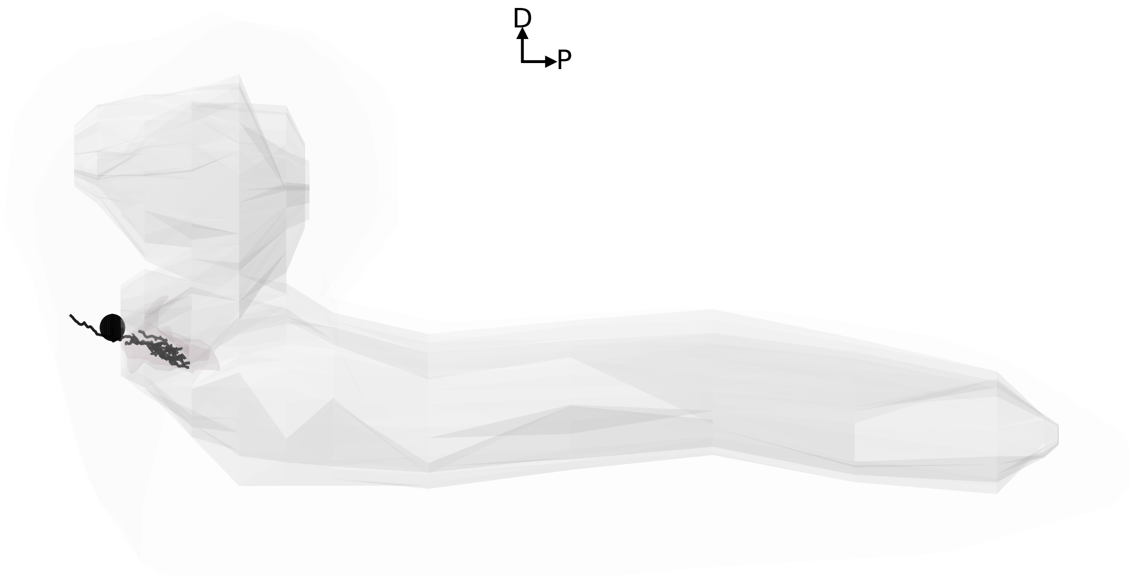

ID	*name*	SCACa	SCAVa	SCAVp	SCACal	SCACp	SCACpl	SCVM	IPCs	DMS	DH44	Se0ens	Se0ph	PMN LR	MN motor neurons	PaN motor neurons	olfactory PNs	gustatory PNs	multiglomerular PNs	unknown PNs	thermo PNs	visual PNs
15769638	SE0 L3	0	0	0	0	0	0	0	0	0	0	0	0	0	0	0	0	0	0	0	0	0

ID: 887905
name: SE0 R1

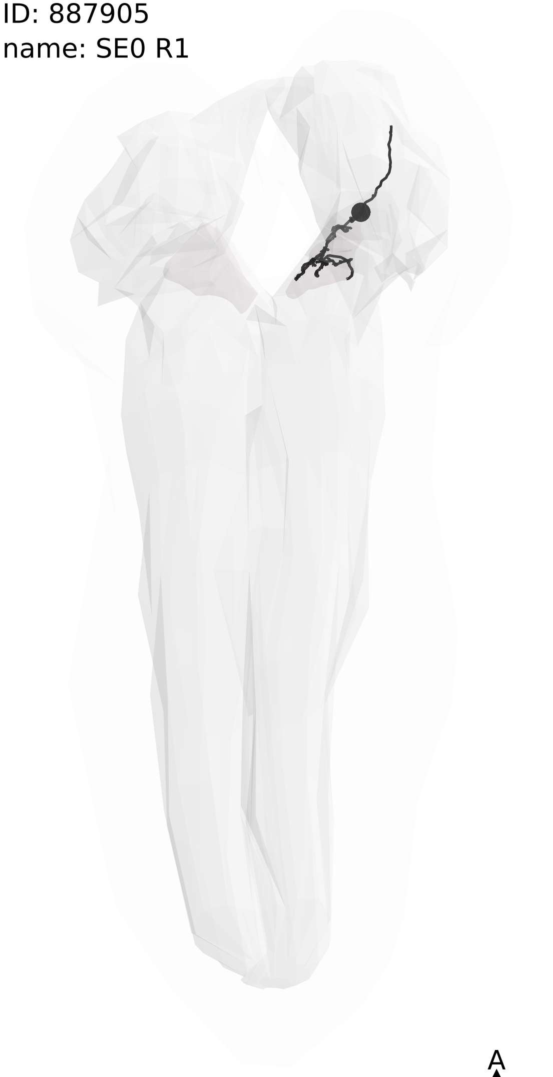

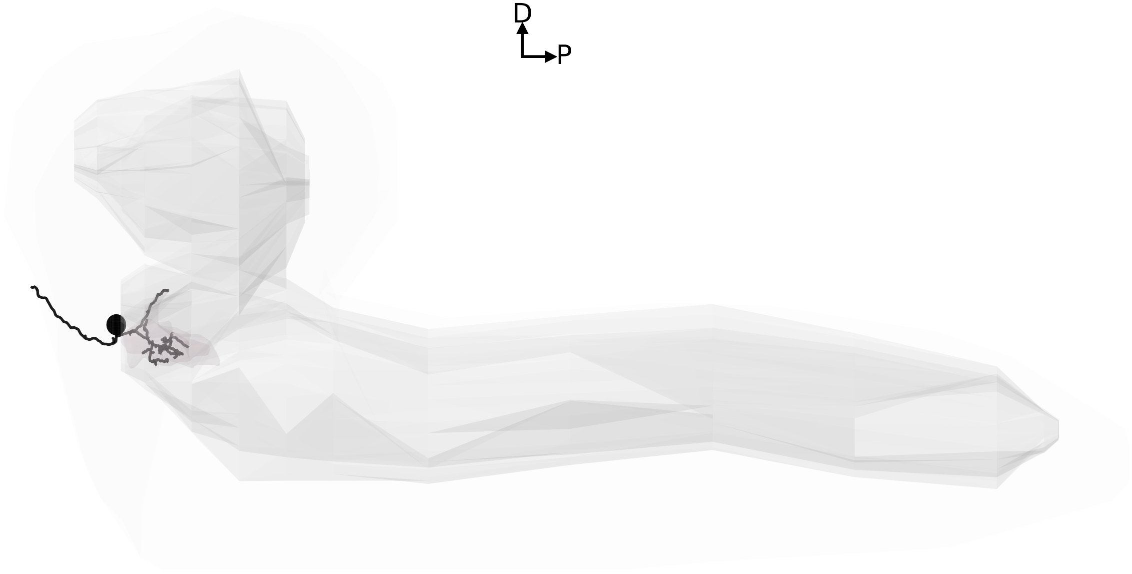

ID	*name*	SCACa	SCAVa	SCAVp	SCACal	SCACp	SCACpl	SCVM	IPCs	DMS	DH44	Se0ens	Se0ph	PMN LR	MN motor neurons	PaN motor neurons	olfactory PNs	gustatory PNs	multiglomerular PNs	unknown PNs	thermo PNs	visual PNs
887905	SE0 R1	0	0	0	0	0	0	0	0	0	0	0	0	0	0	0	0	0	0	0	0	0

ID: 896562
name: SE0 R2

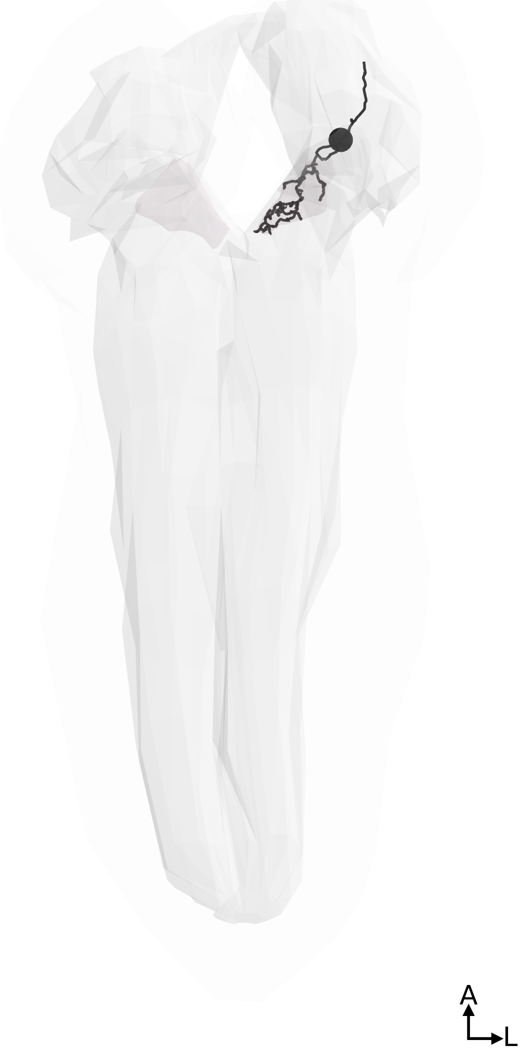

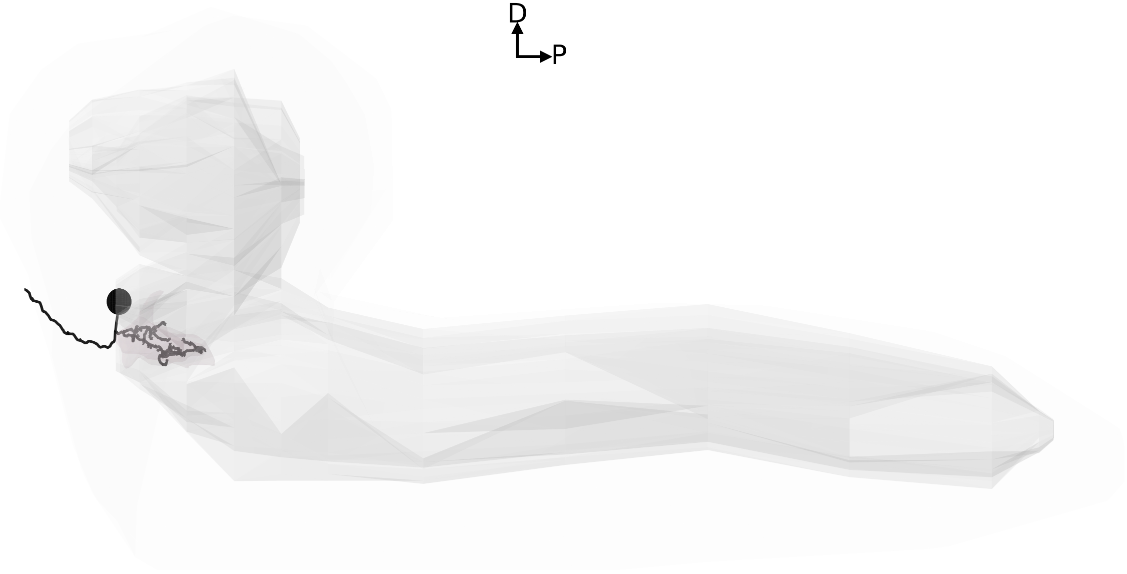

ID	*name*	SCACa	SCAVa	SCAVp	SCACal	SCACp	SCACpl	SCVM	IPCs	DMS	DH44	Se0ens	Se0ph	PMN LR	MN motor neurons	PaN motor neurons	olfactory PNs	gustatory PNs	multiglomerular PNs	unknown PNs	thermo PNs	visual PNs
896562	SE0 R2	0	0	0	0	0	0	0	0	0	0	1	0	0	0	0	0	0	0	0	0	0

ID: 15564695
name: SE0 R3

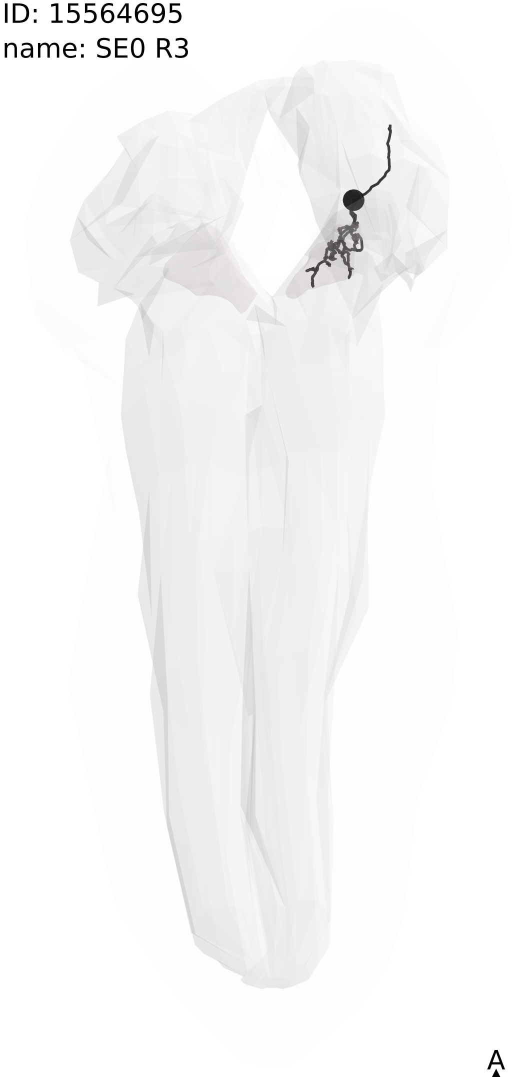

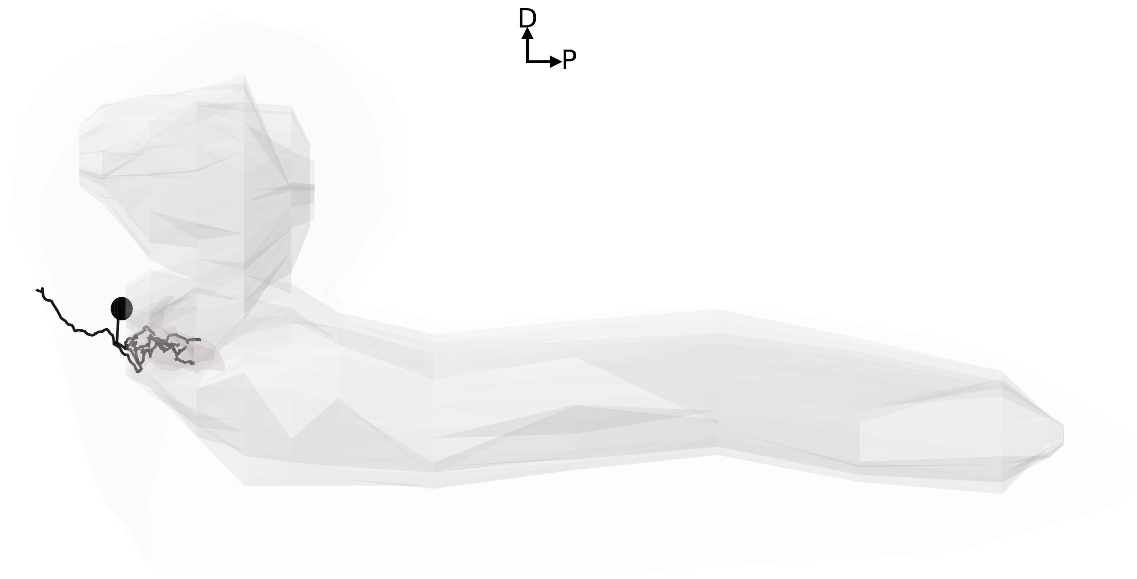

ID	name	SCACa	SCAVa	SCAVp	SCACal	SCACp	SCACpl	SCVM	IPCs	DMS	DH44	Se0ens	Se0ph	PMN LR	MN motor neurons	PaN motor neurons	olfactory PNs	gustatory PNs	multiglomerular PNs	unknown PNs	thermo PNs	visual PNs
15564695	SE0 R3	0	0	0	0	0	0	0	0	0	0	0	0	0	0	0	0	0	0	0	0	0

ID: 15995309
name: SE0 L4

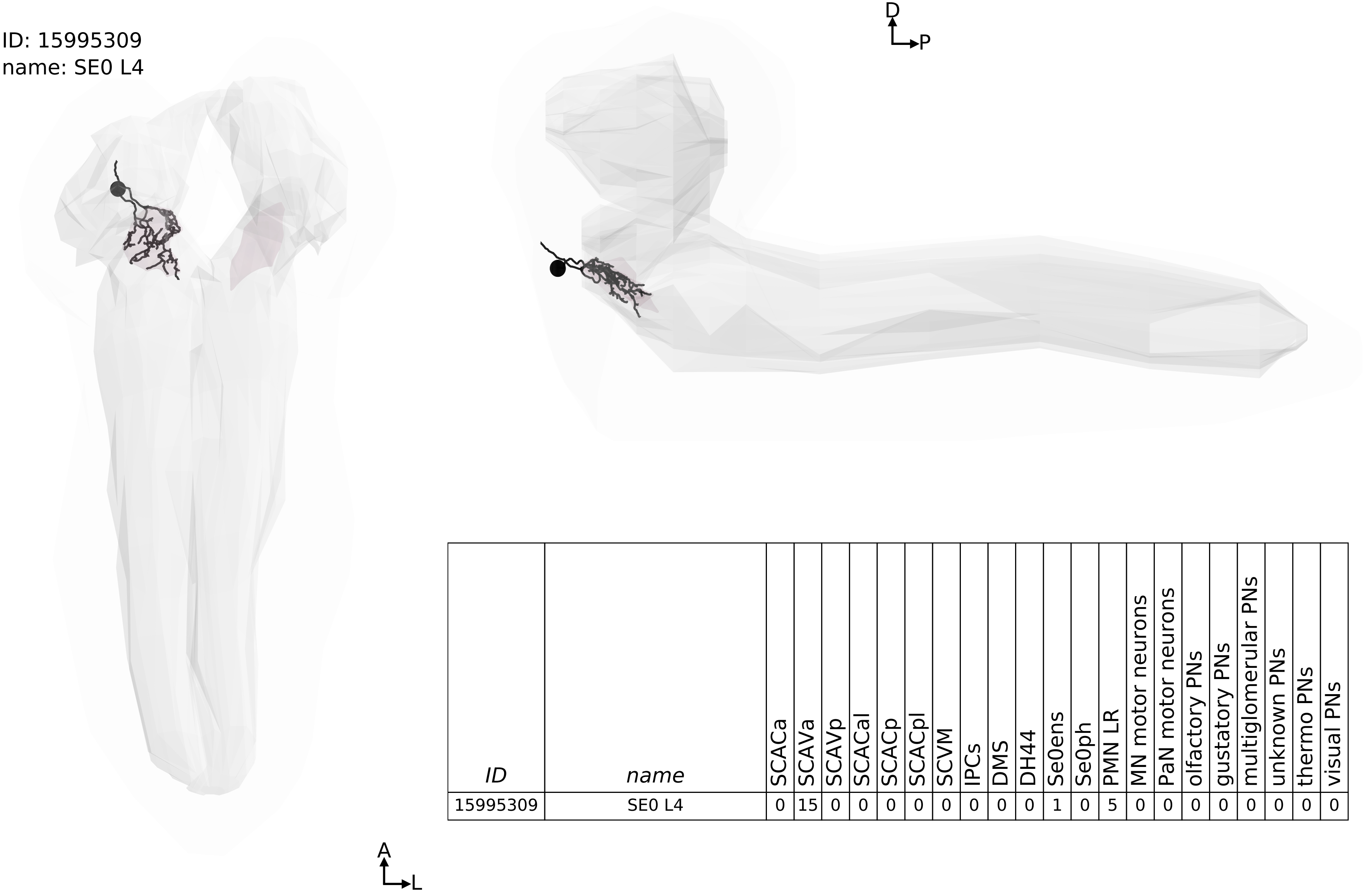

ID	*name*	SCACa	SCAVa	SCAVp	SCACal	SCACp	SCACpl	SCVM	IPCs	DMS	DH44	Se0ens	Se0ph	PMN LR	MN motor neurons	PaN motor neurons	olfactory PNs	gustatory PNs	multiglomerular PNs	unknown PNs	thermo PNs	visual PNs
15995309	SE0 L4	0	15	0	0	0	0	0	0	0	0	1	0	5	0	0	0	0	0	0	0	0

ID: 15573428
name: SE0 R4

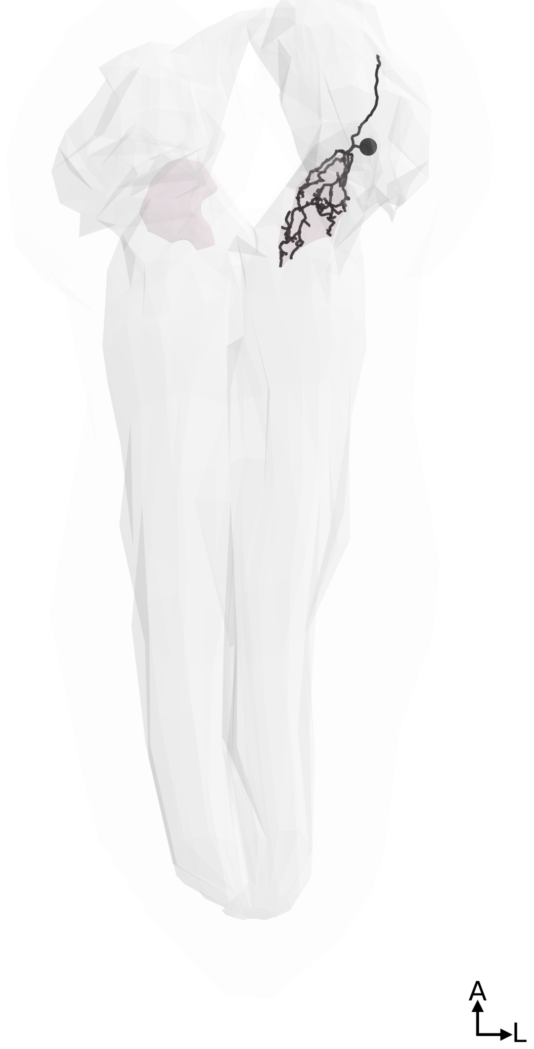

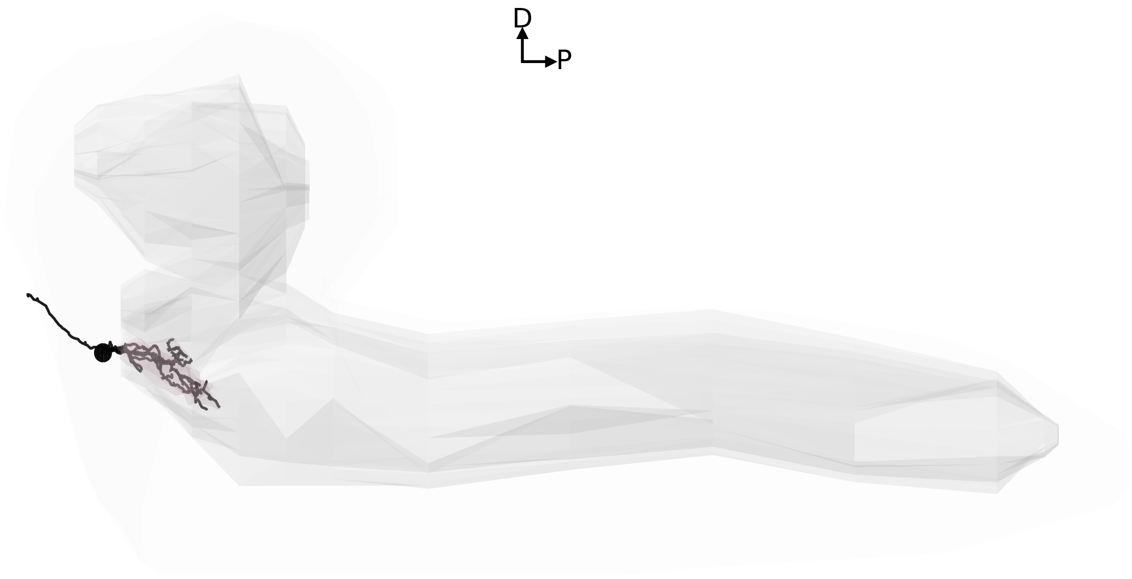

ID	*name*	SCACa	SCAVa	SCAVp	SCACal	SCACp	SCACpl	SCVM	IPCs	DMS	DH44	Se0ens	Se0ph	PMN LR	MN motor neurons	PaN motor neurons	olfactory PNs	gustatory PNs	multiglomerular PNs	unknown PNs	thermo PNs	visual PNs
15573428	SE0 R4	0	15	0	0	0	0	0	0	0	0	3	0	1	0	0	0	0	0	0	0	0

ID: 15769596
name: AN-L-motor-05

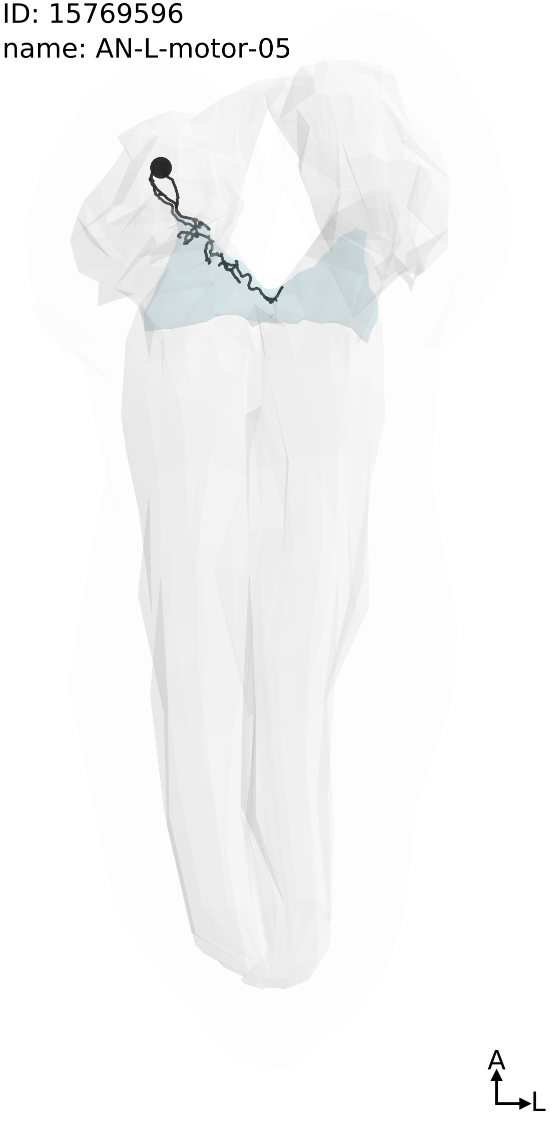

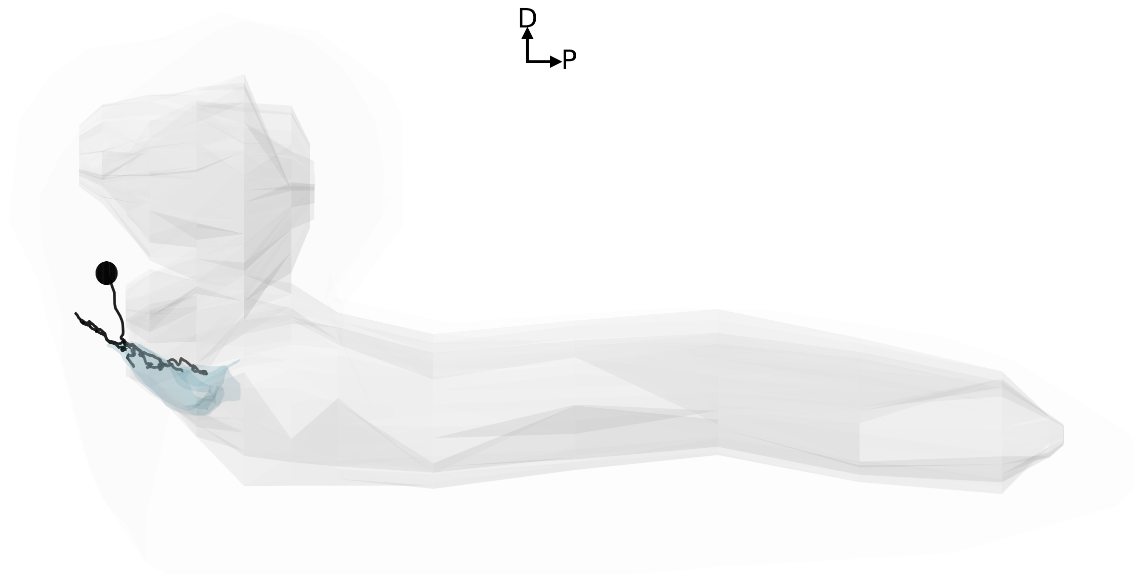

ID	*name*	SCACa	SCAVa	SCAVp	SCACal	SCACp	SCACpl	SCVM	IPCs	DMS	DH44	Se0ens	Se0ph	PMN LR	MN motor neurons	PaN motor neurons	olfactory PNs	gustatory PNs	multiglomerular PNs	unknown PNs	thermo PNs	visual PNs
15769596	AN-L-motor-05	0	0	0	0	0	0	0	0	0	0	0	0	0	0	0	0	0	0	0	0	0

ID: 15769490
name: AN-L-motor-06

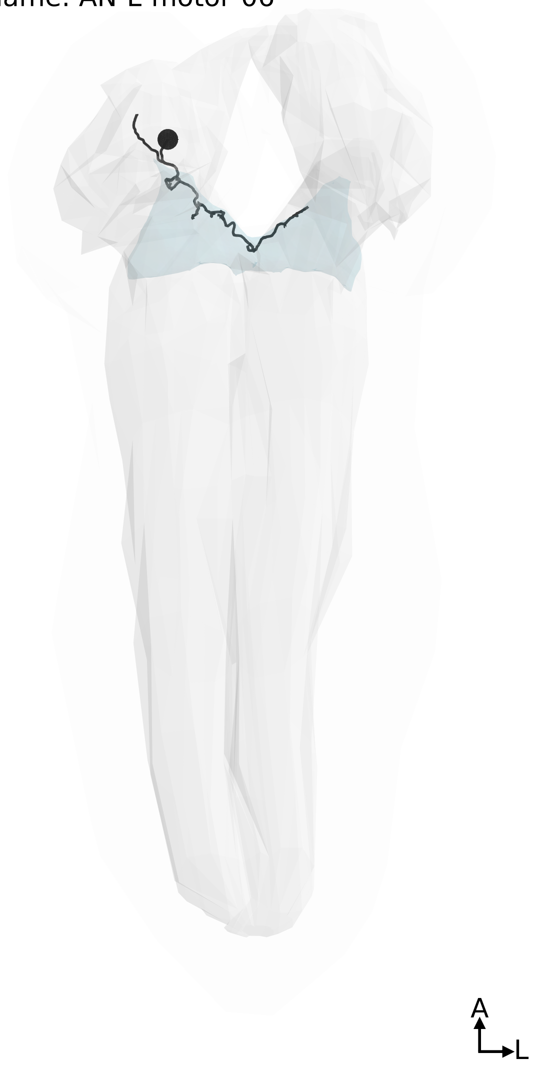

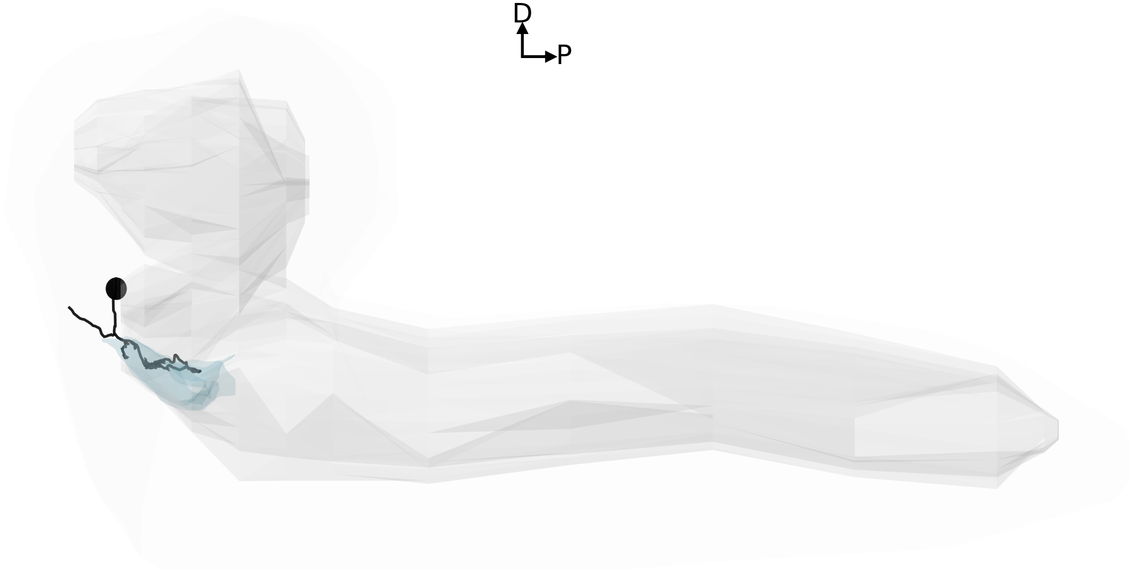

ID	*name*	SCACa	SCAVa	SCAVp	SCACal	SCACp	SCACpl	SCVM	IPCs	DMS	DH44	Se0ens	Se0ph	PMN LR	MN motor neurons	PaN motor neurons	olfactory PNs	gustatory PNs	multiglomerular PNs	unknown PNs	thermo PNs	visual PNs
15769490	AN-L-motor-06	0	0	0	0	0	0	0	0	0	0	0	0	0	0	0	0	0	0	0	0	0

ID: 17369376
name: AN-L-motor-07

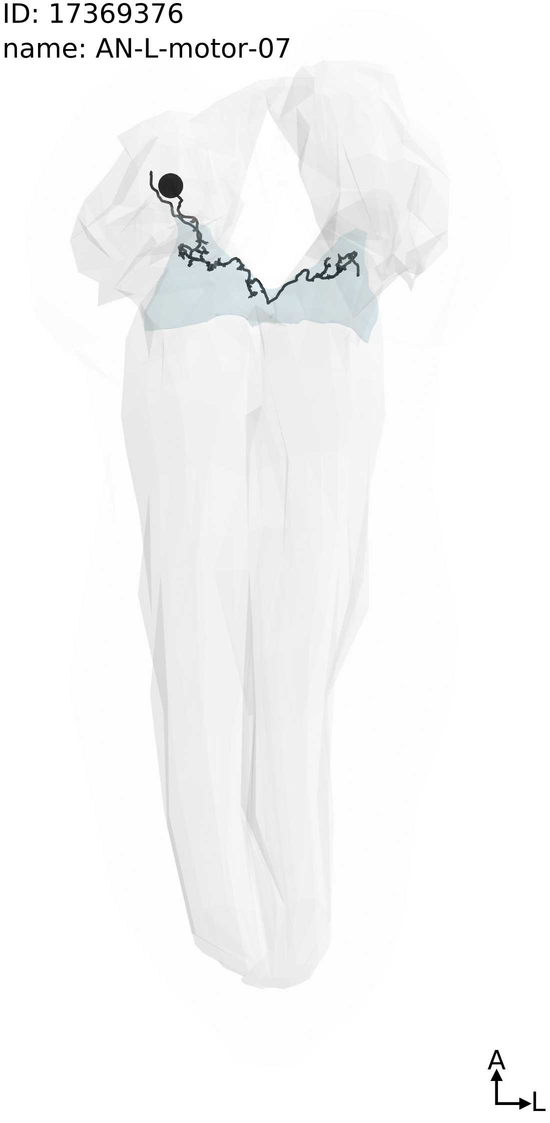

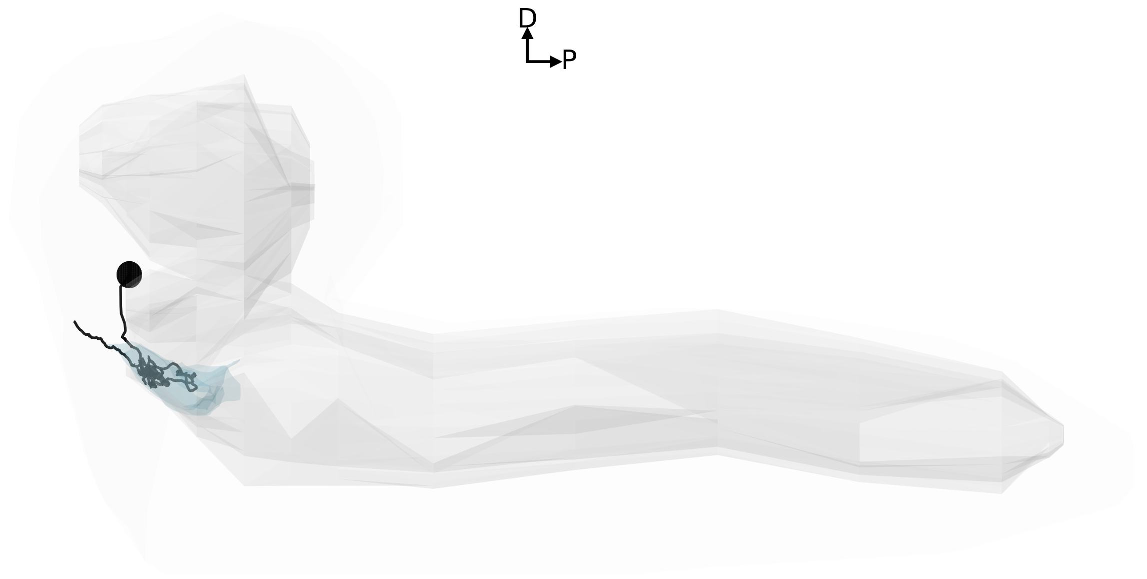

ID	name	SCACa	SCAVa	SCAVp	SCACal	SCACp	SCACpl	SCVM	IPCs	DMS	DH44	Se0ens	Se0ph	PMN LR	MN motor neurons	PaN motor neurons	olfactory PNs	gustatory PNs	multiglomerular PNs	unknown PNs	thermo PNs	visual PNs
17369376	AN-L-motor-07	0	0	0	0	0	0	0	0	0	0	0	0	0	0	0	0	0	0	0	0	0

ID: 407869
name: AN-L-motor-08

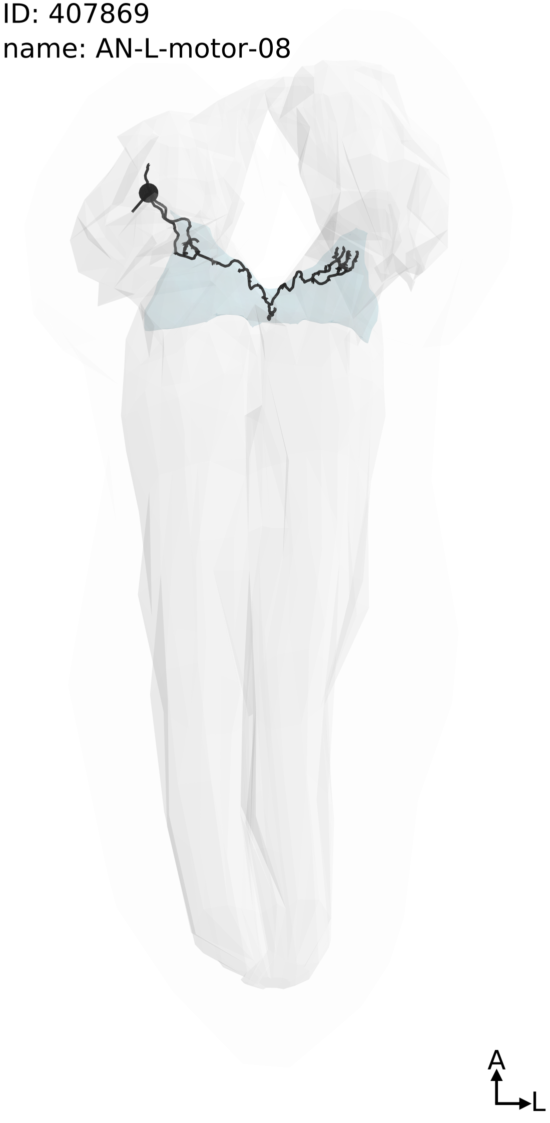

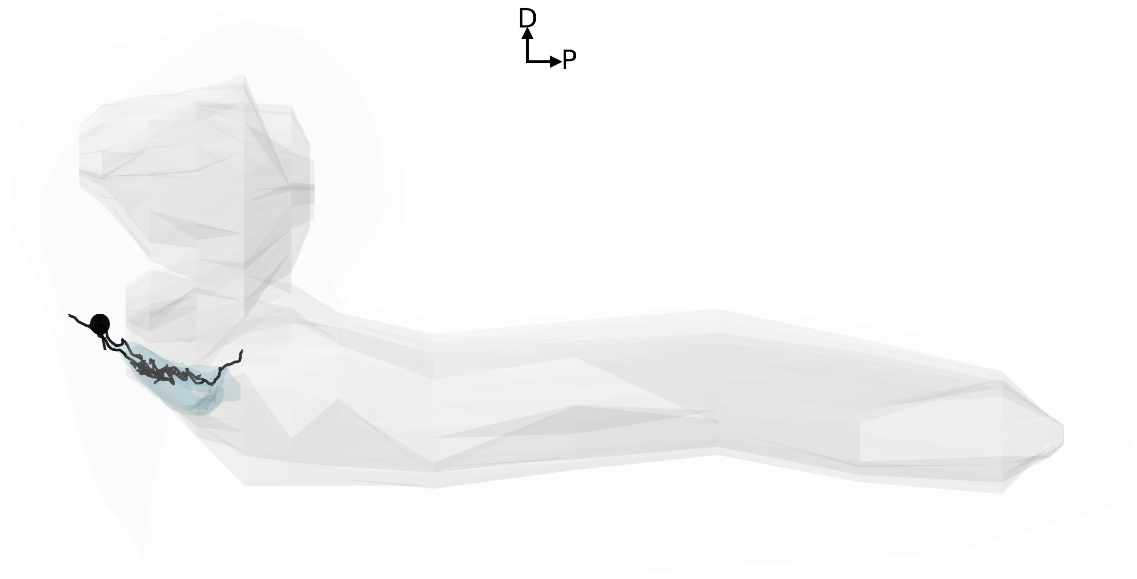

ID	name	SCACa	SCAVa	SCAVp	SCACal	SCACp	SCACpl	SCVM	IPCs	DMS	DH44	Se0ens	Se0ph	PMN LR	MN motor neurons	PaN motor neurons	olfactory PNs	gustatory PNs	multiglomerular PNs	unknown PNs	thermo PNs	visual PNs
407869	AN-L-motor-08	0	0	0	0	0	0	0	0	0	0	0	0	0	0	0	0	0	0	0	0	0

ID: 15995360
name: AN-L-motor-09

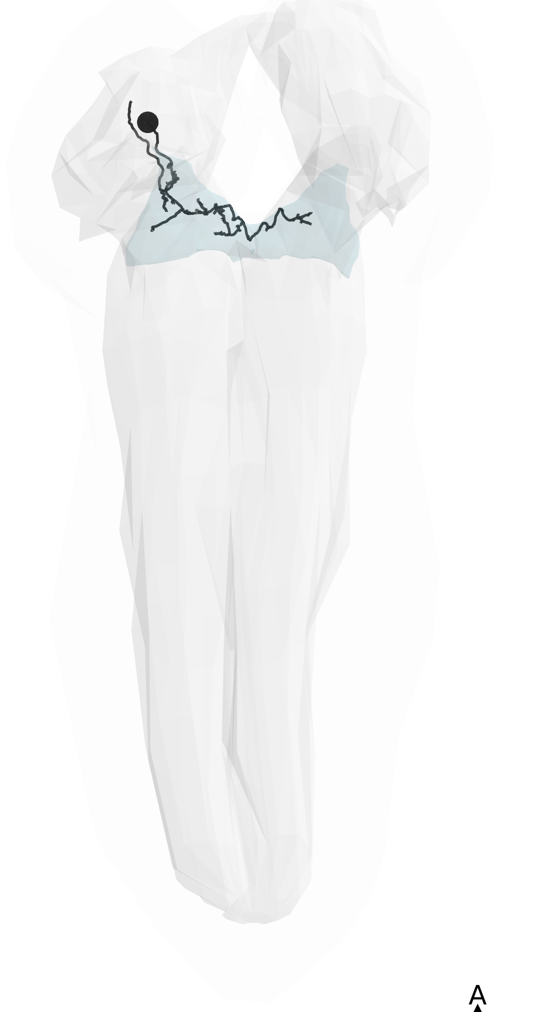

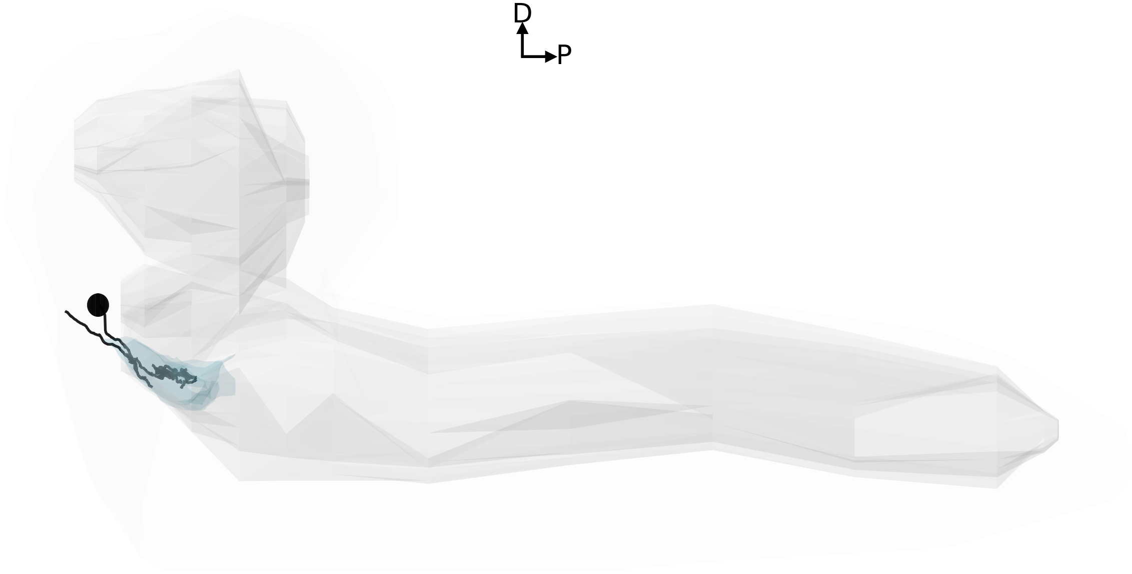

ID	*name*	SCACa	SCAVa	SCAVp	SCACal	SCACp	SCACpl	SCVM	IPCs	DMS	DH44	Se0ens	Se0ph	PMN LR	MN motor neurons	PaN motor neurons	olfactory PNs	gustatory PNs	multiglomerular PNs	unknown PNs	thermo PNs	visual PNs
15995360	AN-L-motor-09	0	0	0	0	0	0	0	0	0	0	0	0	0	0	0	0	0	0	0	0	0

ID: 15982951
name: AN-L-motor-10

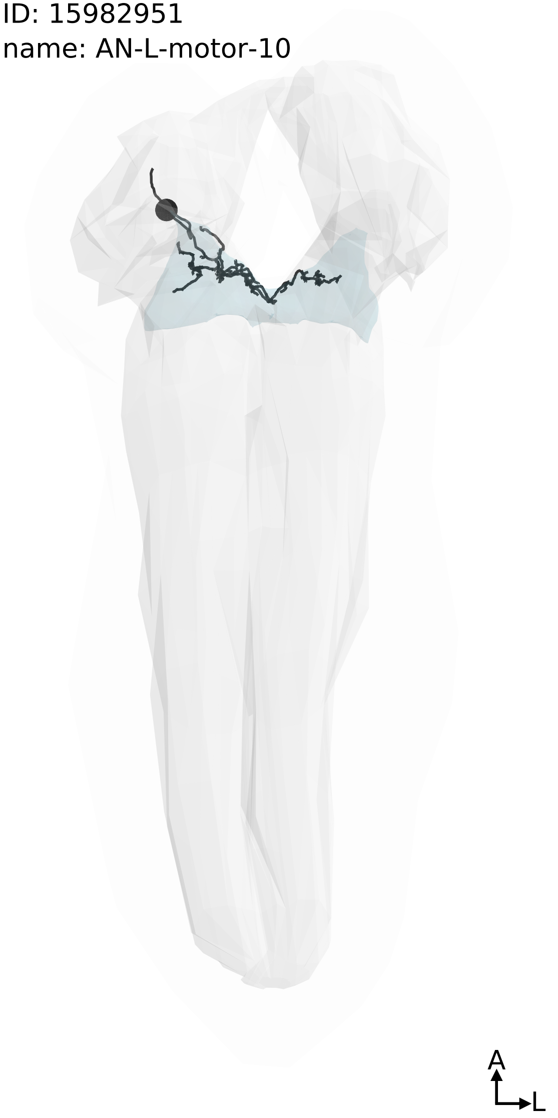

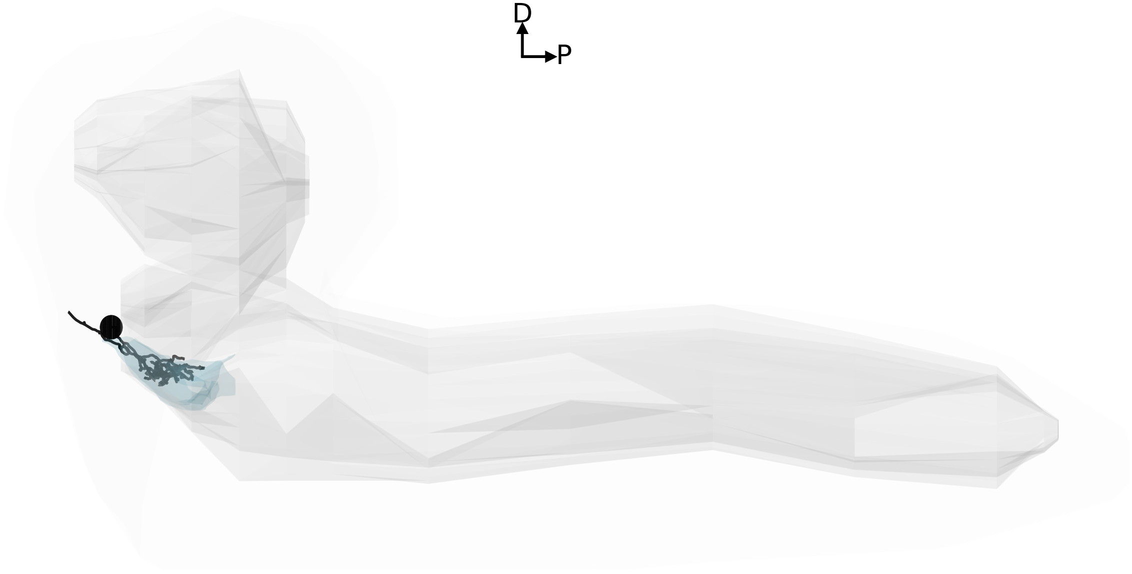

ID	name	SCACa	SCAVa	SCAVp	SCACal	SCACp	SCACpl	SCVM	IPCs	DMS	DH44	Se0ens	Se0ph	PMN LR	MN motor neurons	PaN motor neurons	olfactory PNs	gustatory PNs	multiglomerular PNs	unknown PNs	thermo PNs	visual PNs
15982951	AN-L-motor-10	0	9	0	0	0	0	0	0	0	0	0	0	32	0	0	0	0	0	0	0	0

ID: 17415321
name: AN-L-motor-11

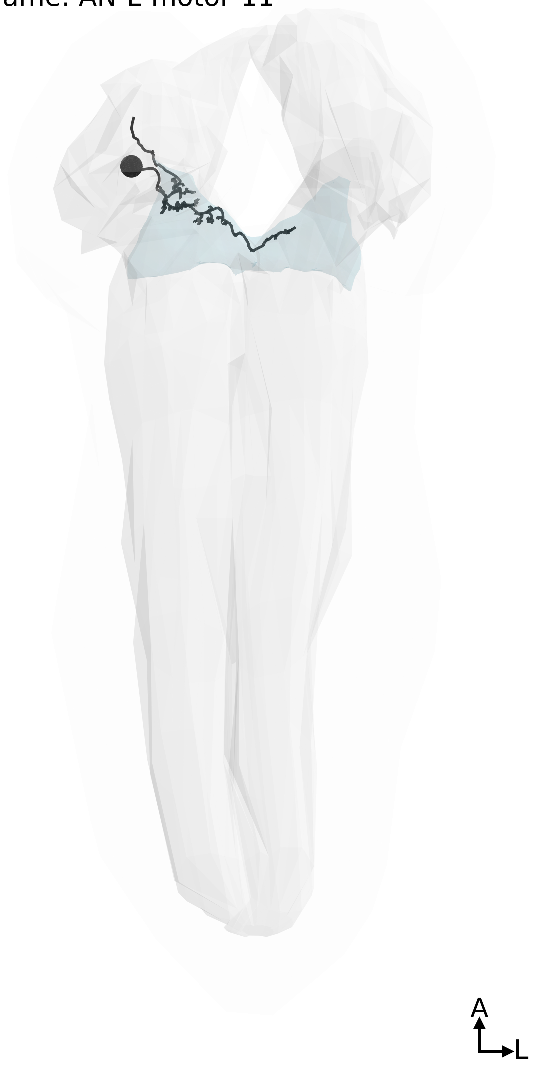

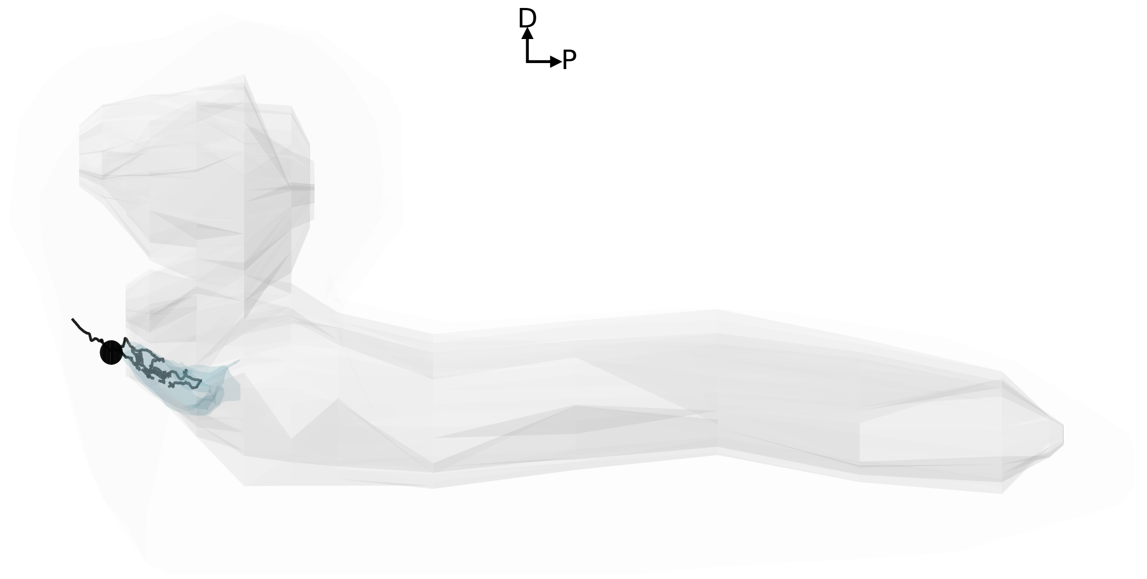

ID	name	SCACa	SCAVa	SCAVp	SCACal	SCACp	SCACpl	SCVM	IPCs	DMS	DH44	Se0ens	Se0ph	PMN LR	MN motor neurons	PaN motor neurons	olfactory PNs	gustatory PNs	multiglomerular PNs	unknown PNs	thermo PNs	visual PNs
17415321	AN-L-motor-11	0	3	0	0	0	0	0	0	0	0	0	1	38	0	0	0	0	0	0	0	0

ID: 17500791
name: AN-L-motor-12

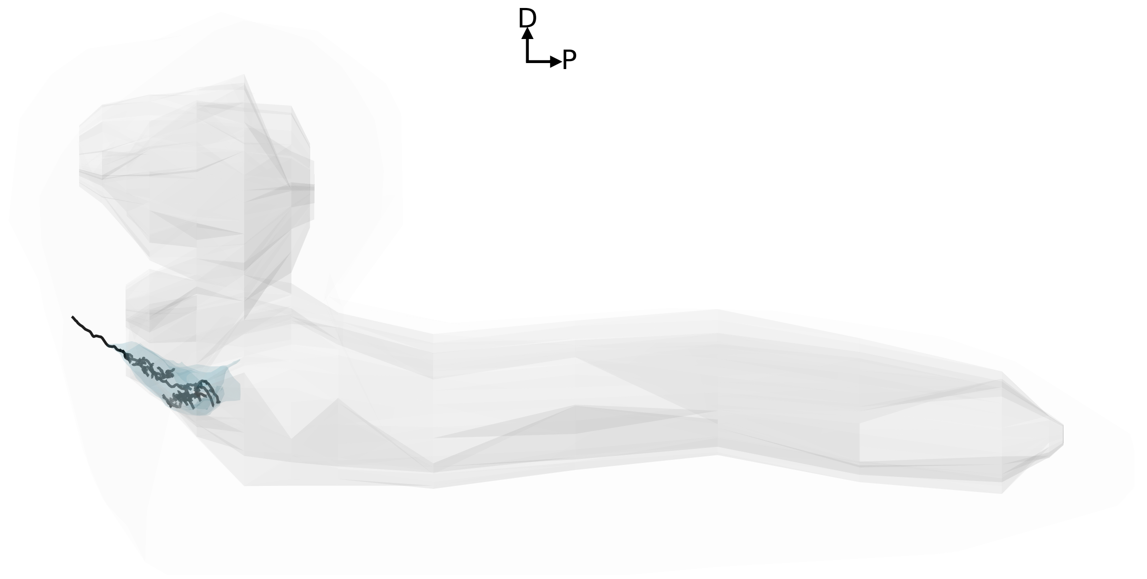

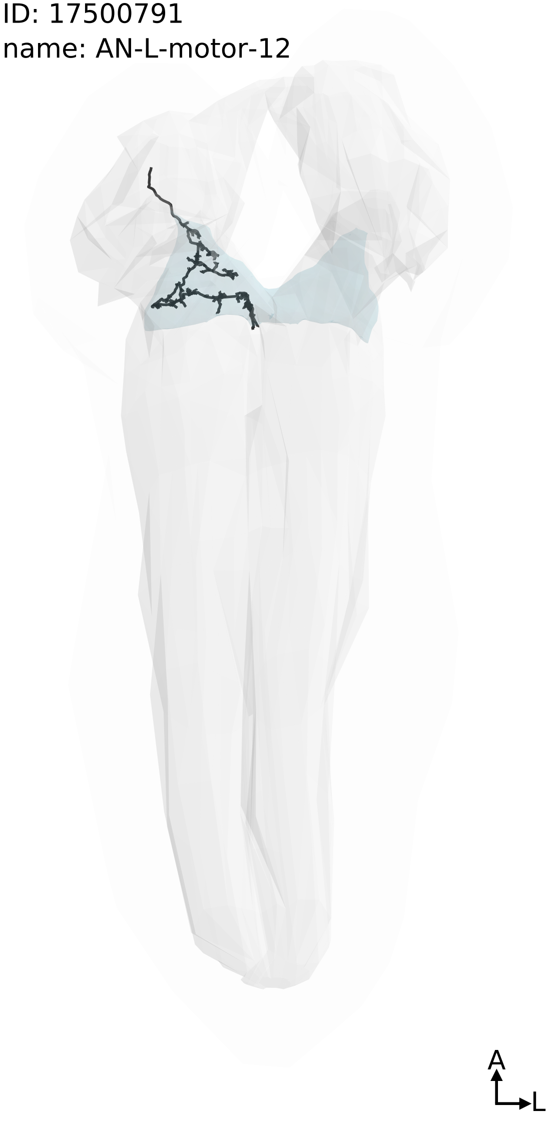

ID	name	SCACa	SCAVa	SCAVp	SCACal	SCACp	SCACpl	SCVM	IPCs	DMS	DH44	Se0ens	Se0ph	PMN LR	MN motor neurons	PaN motor neurons	olfactory PNs	gustatory PNs	multiglomerular PNs	unknown PNs	thermo PNs	visual PNs
17500791	AN-L-motor-12	0	0	0	0	0	0	0	0	0	0	0	0	21	0	0	0	0	0	0	0	0

ID: 17405603
name: AN-L-motor-13

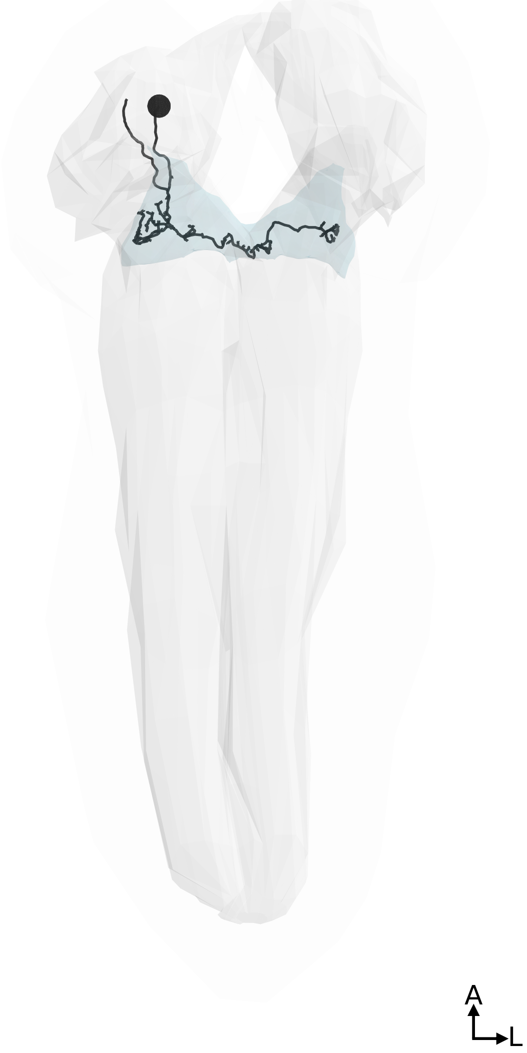

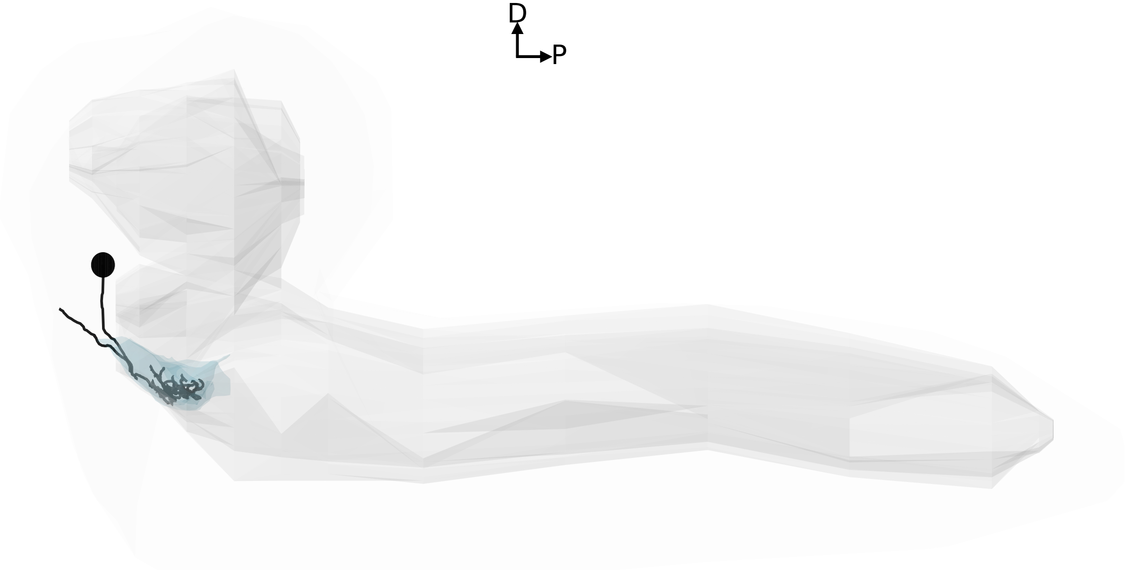

ID	*name*	SCACa	SCAVa	SCAVp	SCACal	SCACp	SCACpl	SCVM	IPCs	DMS	DH44	Se0ens	Se0ph	PMN LR	MN motor neurons	PaN motor neurons	olfactory PNs	gustatory PNs	multiglomerular PNs	unknown PNs	thermo PNs	visual PNs
17405603	AN-L-motor-13	0	0	0	0	0	0	0	0	0	0	0	0	0	0	0	0	0	0	0	0	0

ID: 15995394
name: AN-L-motor-14

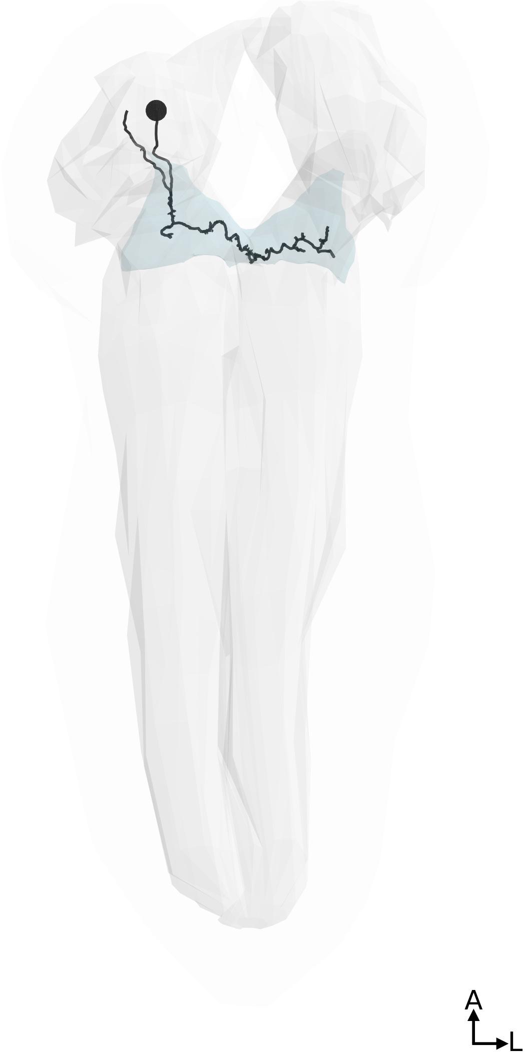

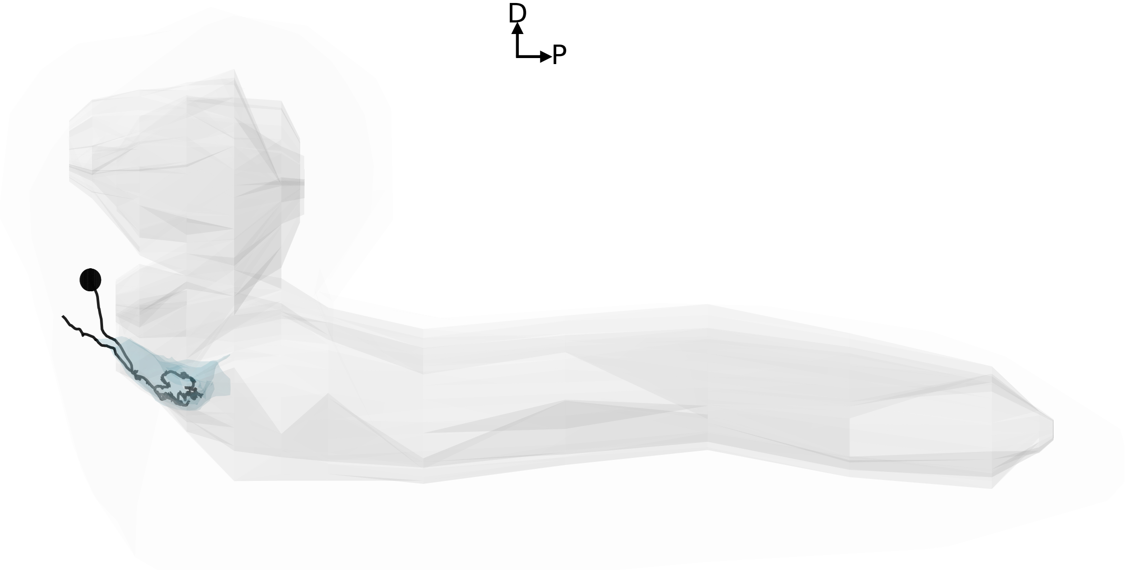

ID	*name*	SCACa	SCAVa	SCAVp	SCACal	SCACp	SCACpl	SCVM	IPCs	DMS	DH44	Se0ens	Se0ph	PMN LR	MN motor neurons	PaN motor neurons	olfactory PNs	gustatory PNs	multiglomerular PNs	unknown PNs	thermo PNs	visual PNs
15995394	AN-L-motor-14	0	0	0	0	0	0	0	0	0	0	0	0	0	0	0	0	0	0	0	0	0

ID: 15770521
name: AN-L-motor-15

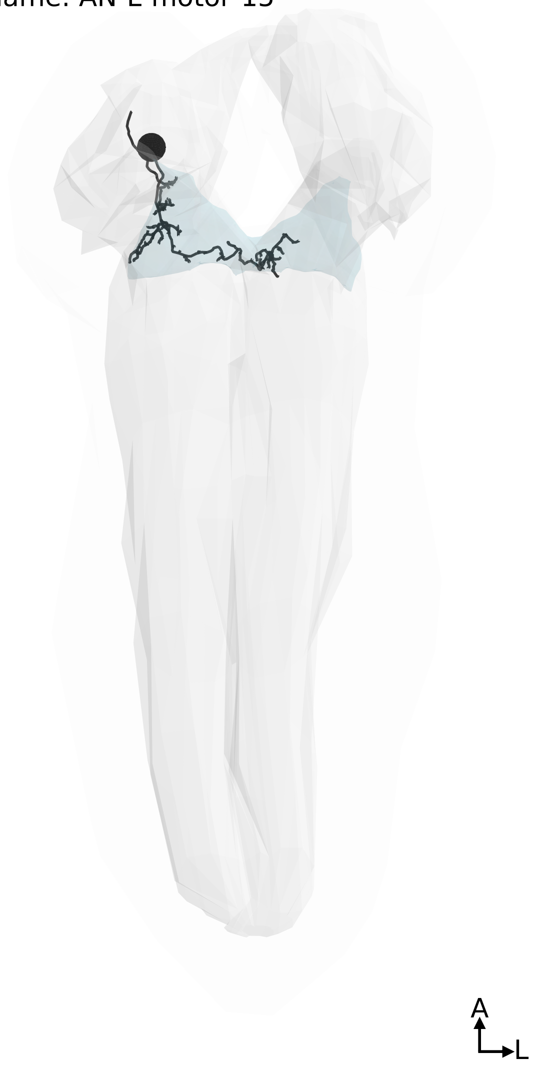

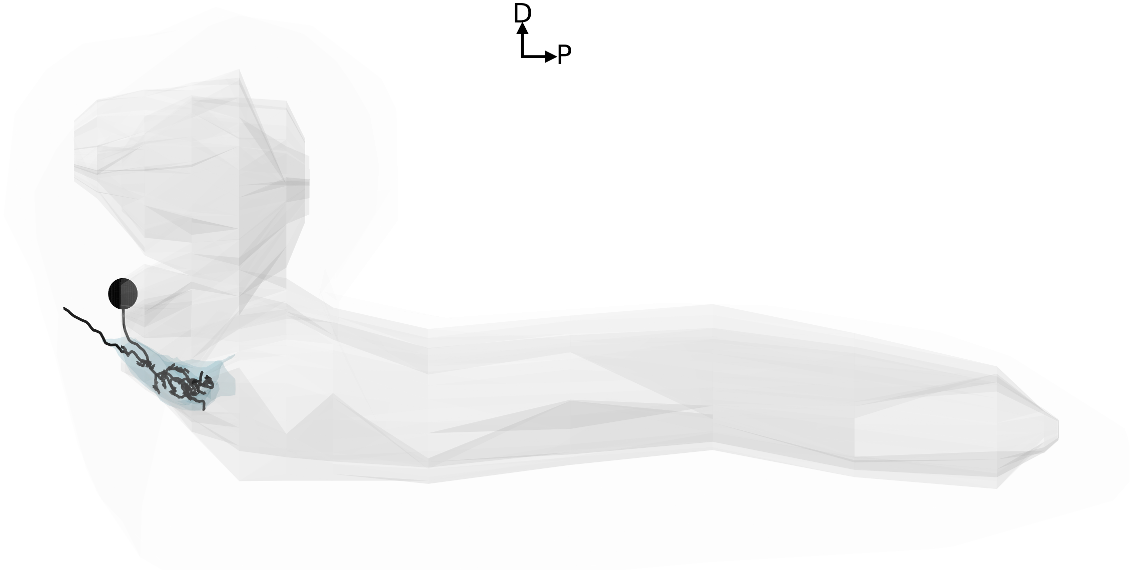

ID	*name*	SCACa	SCAVa	SCAVp	SCACal	SCACp	SCACpl	SCVM	IPCs	DMS	DH44	Se0ens	Se0ph	PMN LR	MN motor neurons	PaN motor neurons	olfactory PNs	gustatory PNs	multiglomerular PNs	unknown PNs	thermo PNs	visual PNs
15770521	AN-L-motor-15	0	0	0	0	0	0	0	0	0	0	0	0	0	0	0	0	0	0	0	0	0

ID: 15770867
name: AN-L-motor-16

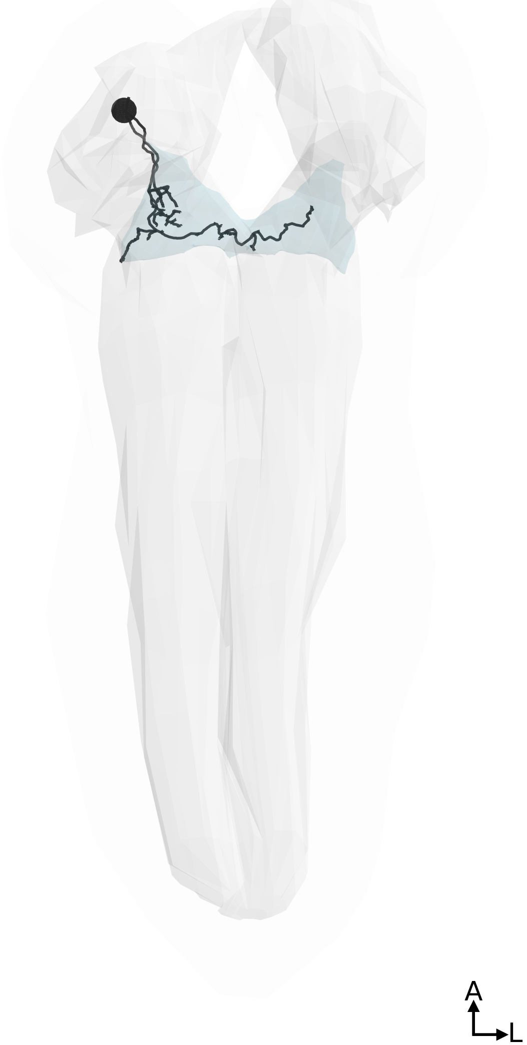

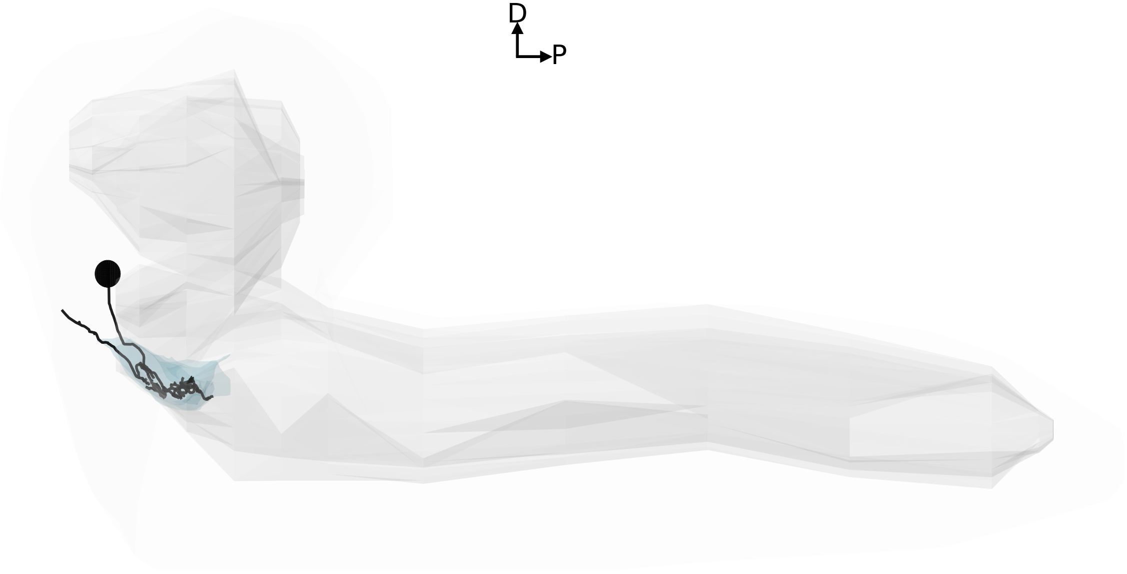

ID	*name*	SCACa	SCAVa	SCAVp	SCACal	SCACp	SCACpl	SCVM	IPCs	DMS	DH44	Se0ens	Se0ph	PMN LR	MN motor neurons	PaN motor neurons	olfactory PNs	gustatory PNs	multiglomerular PNs	unknown PNs	thermo PNs	visual PNs
15770867	AN-L-motor-16	0	0	0	0	0	0	0	0	0	0	0	0	0	0	0	0	0	0	0	0	0

ID: 15995368
name: AN-L-motor-17

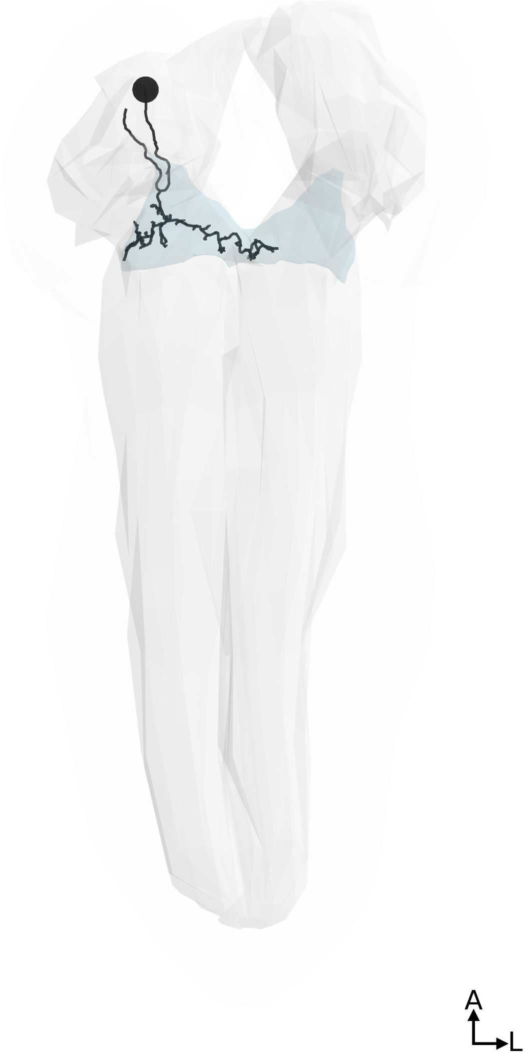

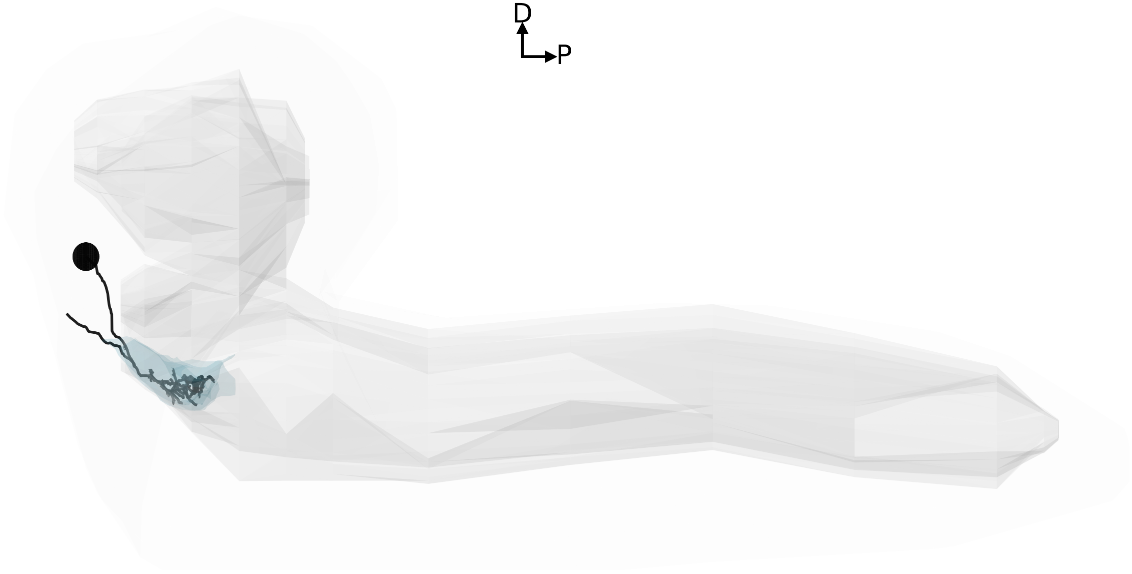

ID	*name*	SCACa	SCAVa	SCAVp	SCACal	SCACp	SCACpl	SCVM	IPCs	DMS	DH44	Se0ens	Se0ph	PMN LR	MN motor neurons	PaN motor neurons	olfactory PNs	gustatory PNs	multiglomerular PNs	unknown PNs	thermo PNs	visual PNs
15995368	AN-L-motor-17	0	0	0	0	0	0	0	0	0	0	0	0	0	0	0	0	0	0	0	0	0

ID: 15997068
name: AN-L-motor-18

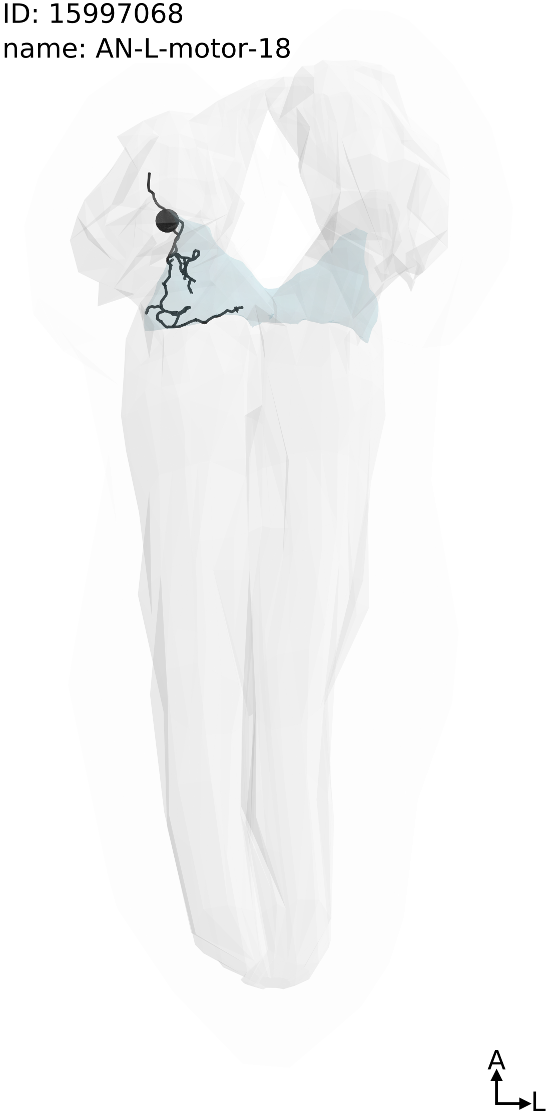

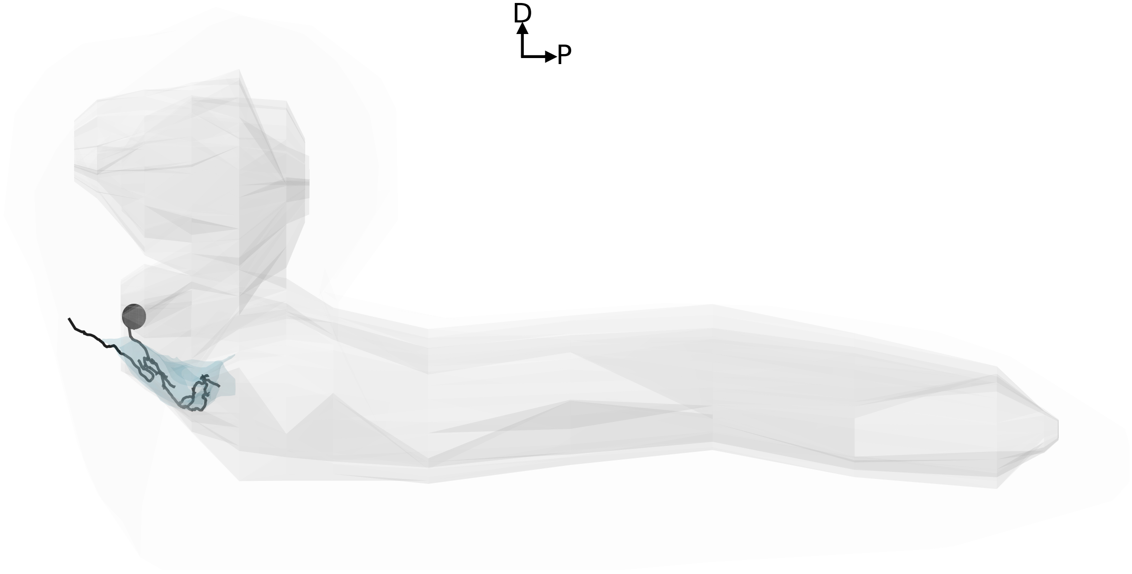

ID	name	SCACa	SCAVa	SCAVp	SCACal	SCACp	SCACpl	SCVM	IPCs	DMS	DH44	Se0ens	Se0ph	PMN LR	MN motor neurons	PaN motor neurons	olfactory PNs	gustatory PNs	multiglomerular PNs	unknown PNs	thermo PNs	visual PNs
15997068	AN-L-motor-18	0	0	0	0	0	0	0	0	0	0	0	0	0	0	0	0	0	0	0	0	0

ID: 17248923
name: AN-R-motor-05

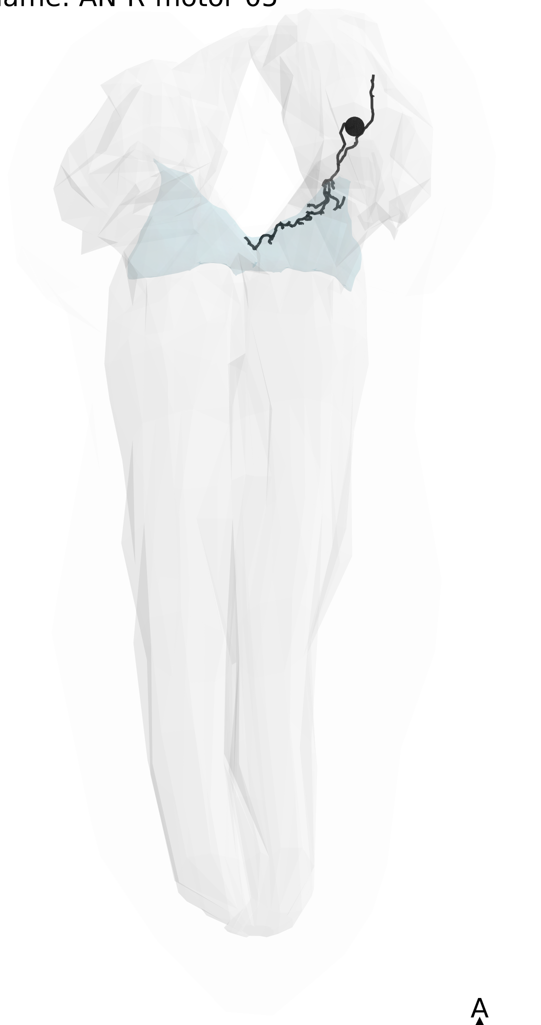

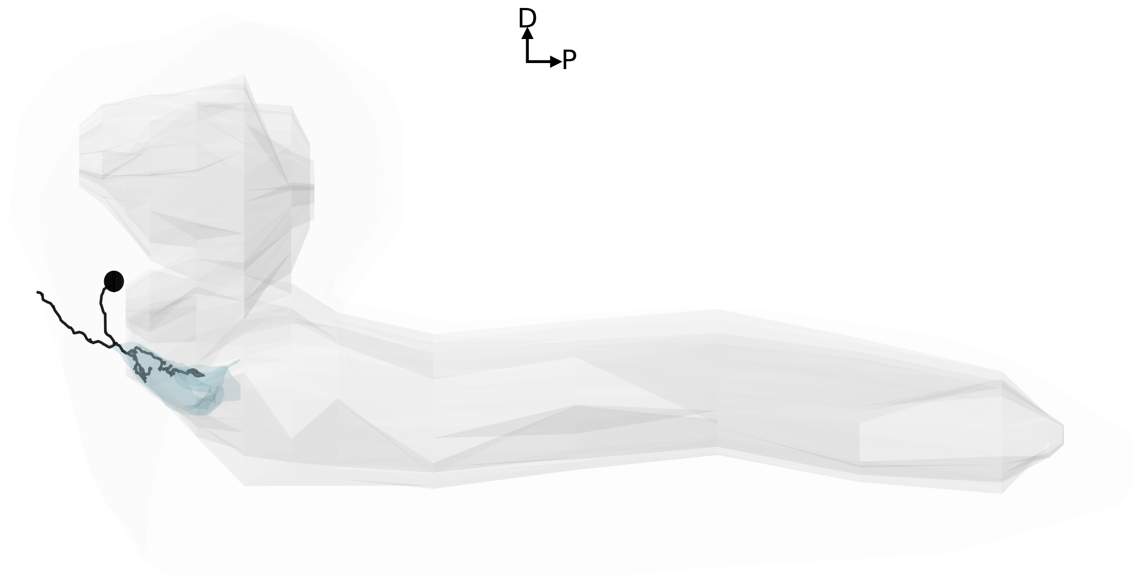

ID	*name*	SCACa	SCAVa	SCAVp	SCACal	SCACp	SCACpl	SCVM	IPCs	DMS	DH44	Se0ens	Se0ph	PMN LR	MN motor neurons	PaN motor neurons	olfactory PNs	gustatory PNs	multiglomerular PNs	unknown PNs	thermo PNs	visual PNs
17248923	AN-R-motor-05	0	0	0	0	0	0	0	0	0	0	0	0	0	0	0	0	0	0	0	0	0

ID: 882339
name: AN-R-motor-06

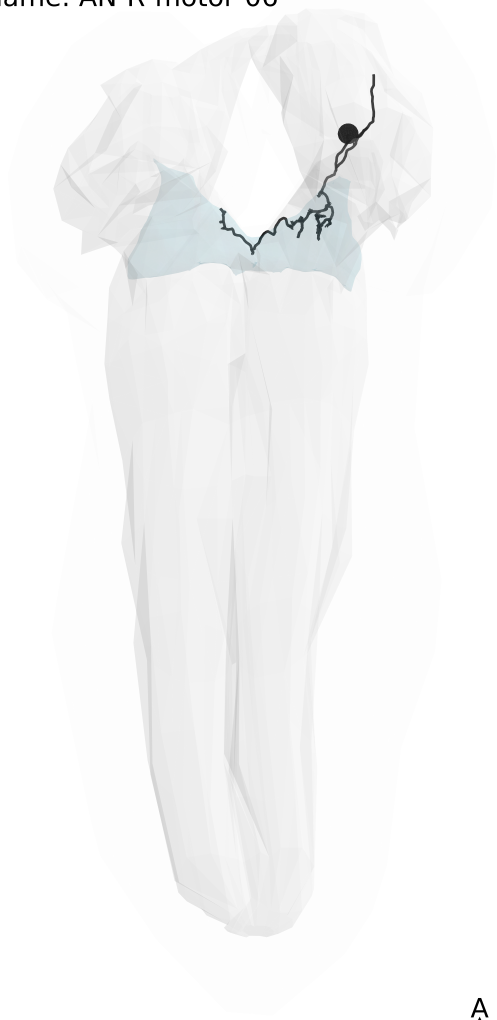

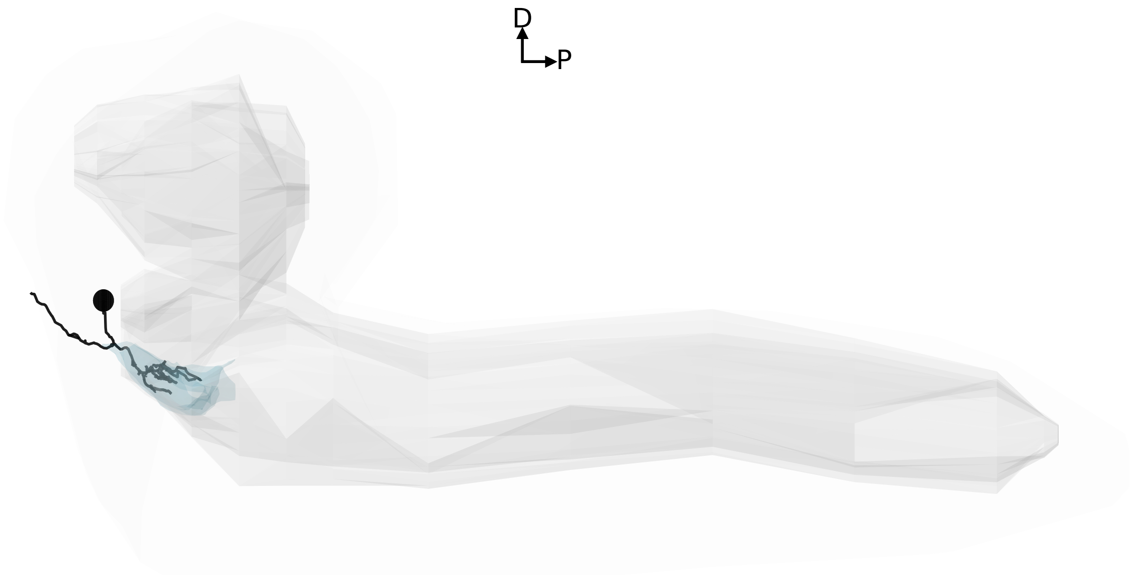

ID	name	SCACa	SCAVa	SCAVp	SCACal	SCACp	SCACpl	SCVM	IPCs	DMS	DH44	Se0ens	Se0ph	PMN LR	MN motor neurons	PaN motor neurons	olfactory PNs	gustatory PNs	multiglomerular PNs	unknown PNs	thermo PNs	visual PNs
882339	AN-R-motor-06	0	0	0	0	0	0	0	0	0	0	0	0	0	0	0	0	0	0	0	0	0

ID: 15567384
name: AN-R-motor-07

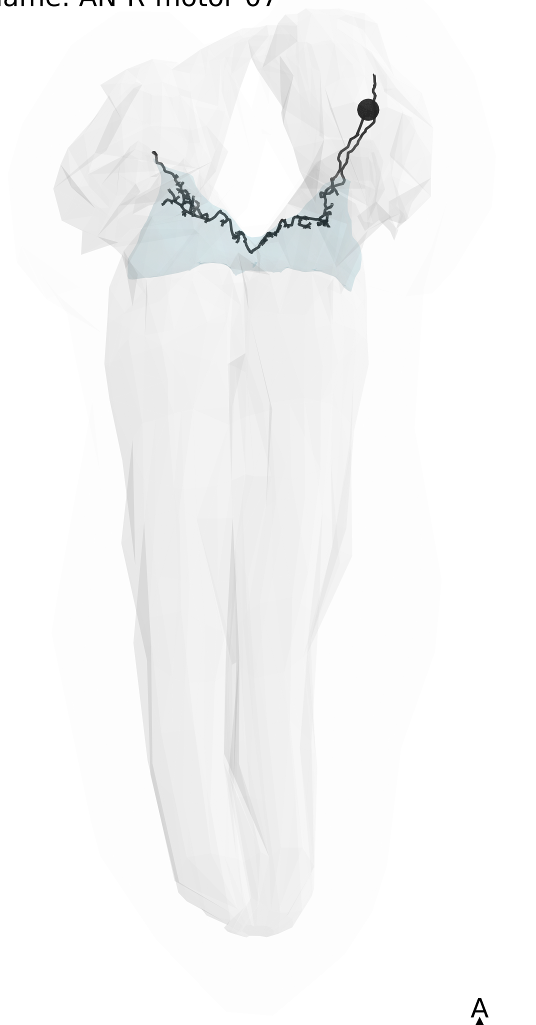

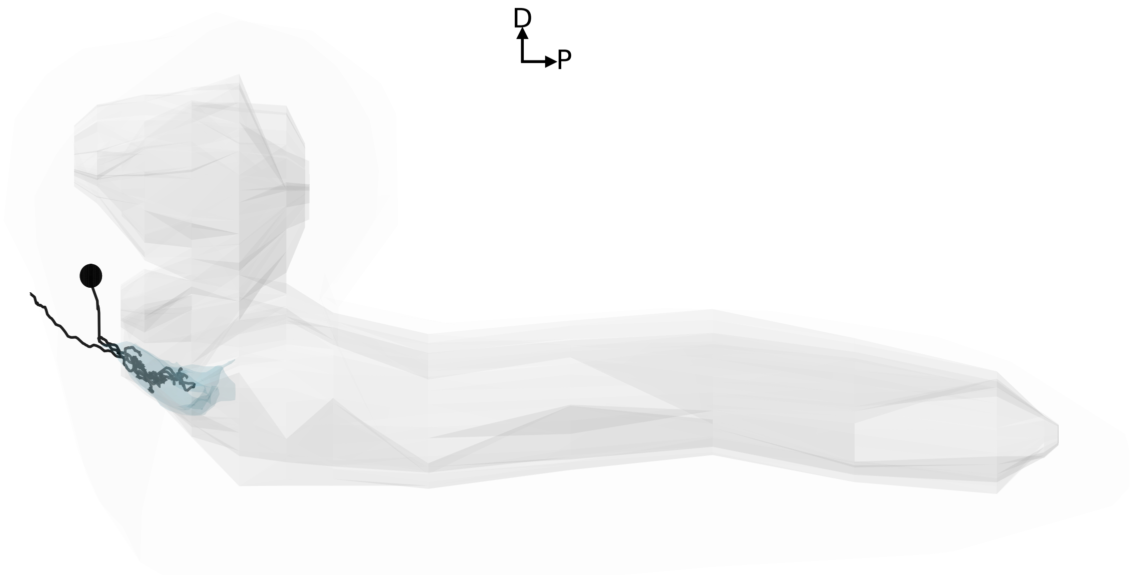

ID	*name*	SCACa	SCAVa	SCAVp	SCACal	SCACp	SCACpl	SCVM	IPCs	DMS	DH44	Se0ens	Se0ph	PMN LR	MN motor neurons	PaN motor neurons	olfactory PNs	gustatory PNs	multiglomerular PNs	unknown PNs	thermo PNs	visual PNs
15567384	AN-R-motor-07	0	0	0	0	0	0	0	0	0	0	0	0	0	0	0	0	0	0	0	0	0

ID: 1218587
name: AN-R-motor-08

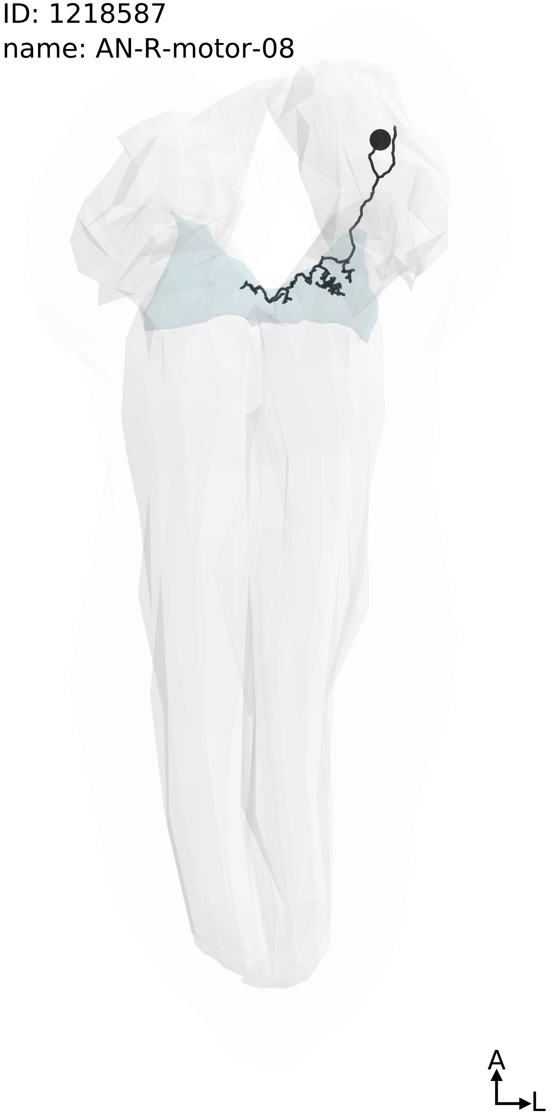

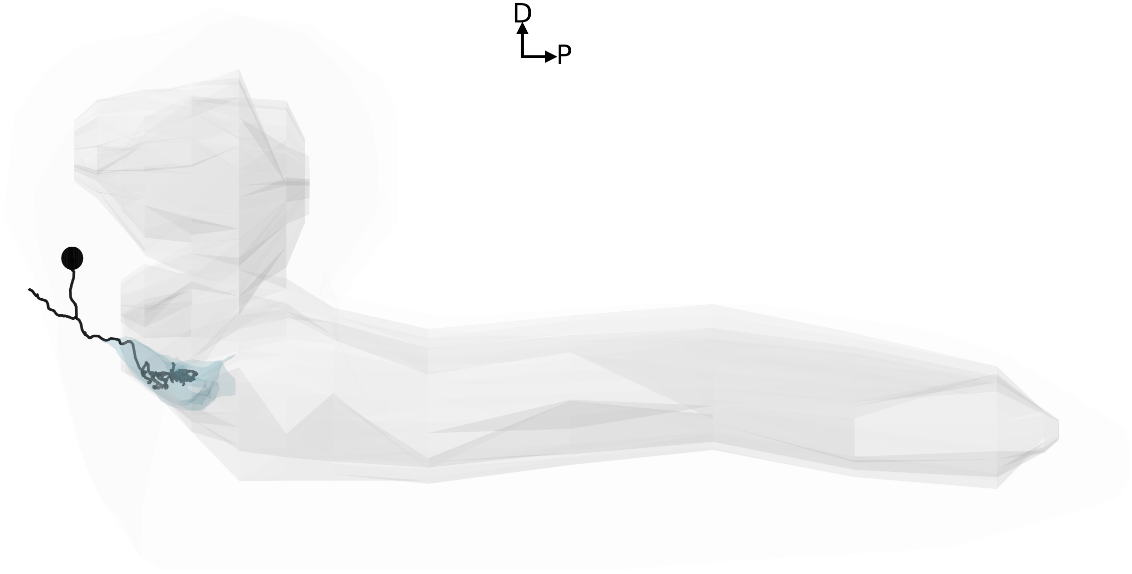

ID	name	SCACa	SCAVa	SCAVp	SCACal	SCACp	SCACpl	SCVM	IPCs	DMS	DH44	Se0ens	Se0ph	PMN LR	MN motor neurons	PaN motor neurons	olfactory PNs	gustatory PNs	multiglomerular PNs	unknown PNs	thermo PNs	visual PNs
1218587	AN-R-motor-08	0	5	0	0	0	0	0	0	0	0	0	0	24	0	0	0	0	0	0	0	0

ID: 875613
name: AN-R-motor-09

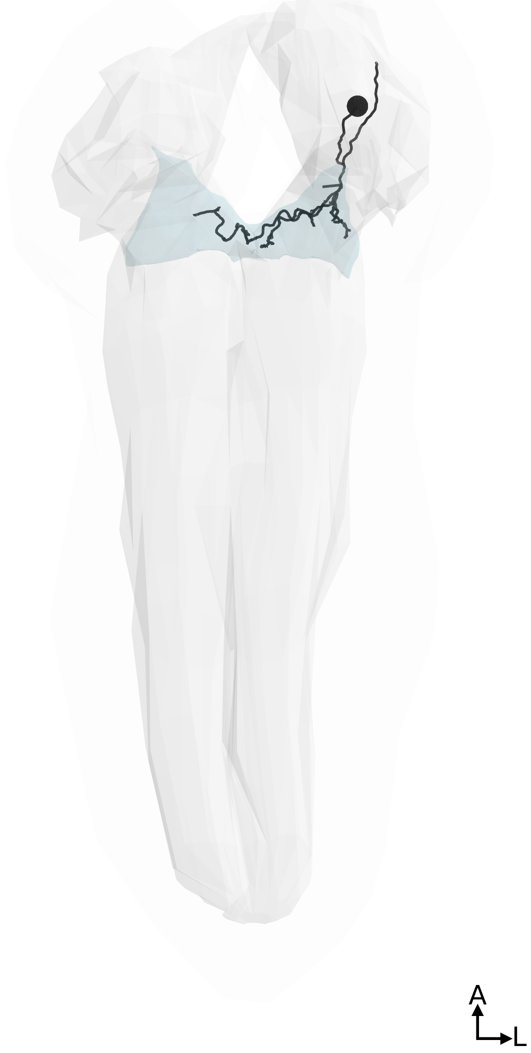

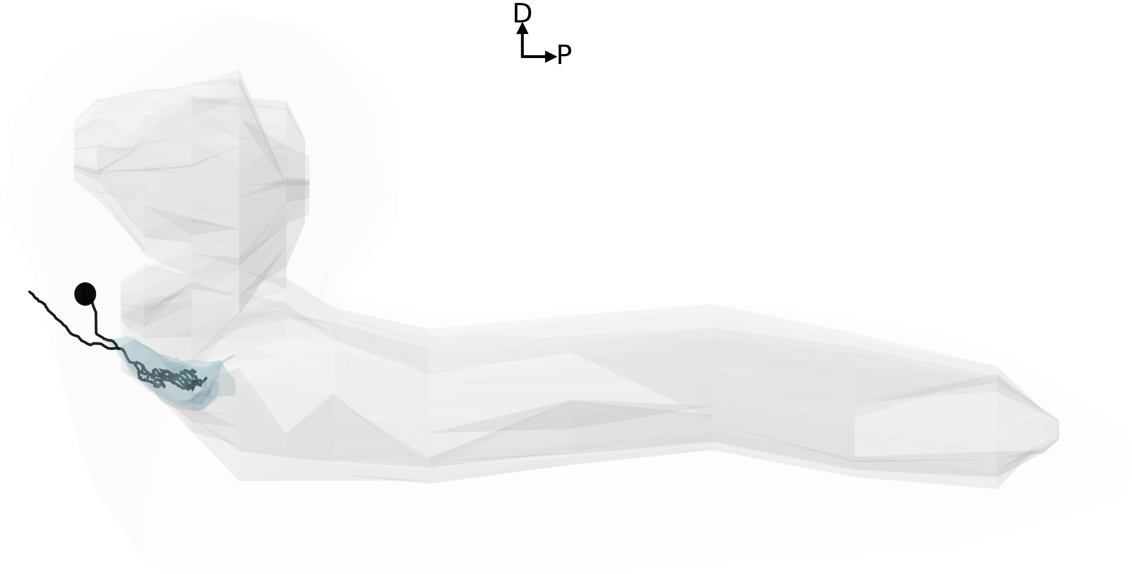

ID	*name*	SCACa	SCAVa	SCAVp	SCACal	SCACp	SCACpl	SCVM	IPCs	DMS	DH44	Se0ens	Se0ph	PMN LR	MN motor neurons	PaN motor neurons	olfactory PNs	gustatory PNs	multiglomerular PNs	unknown PNs	thermo PNs	visual PNs
875613	AN-R-motor-09	0	0	0	0	0	0	0	0	0	0	0	0	0	0	0	0	0	0	0	0	0

ID: 3340561
name: AN-R-motor-10

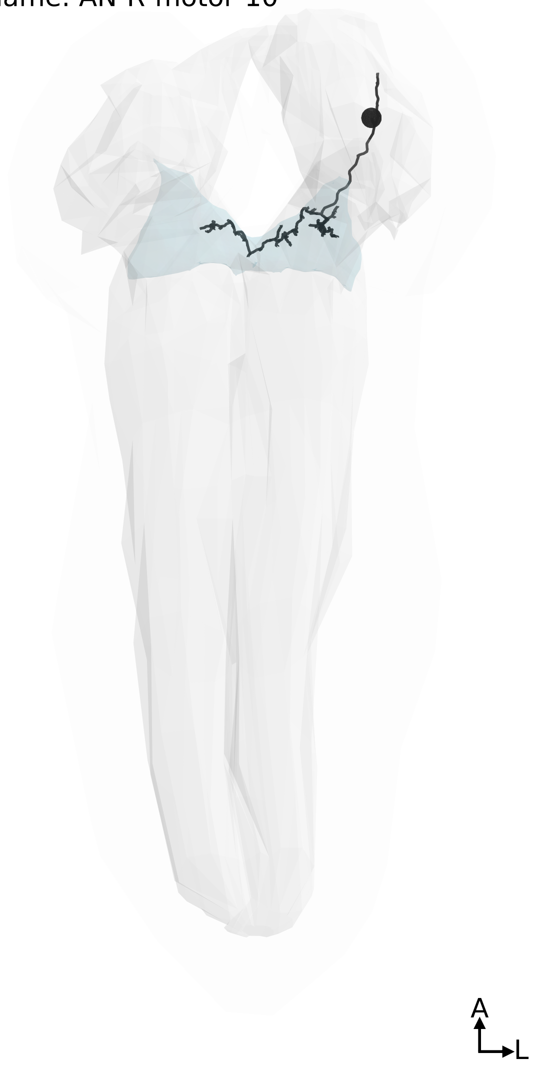

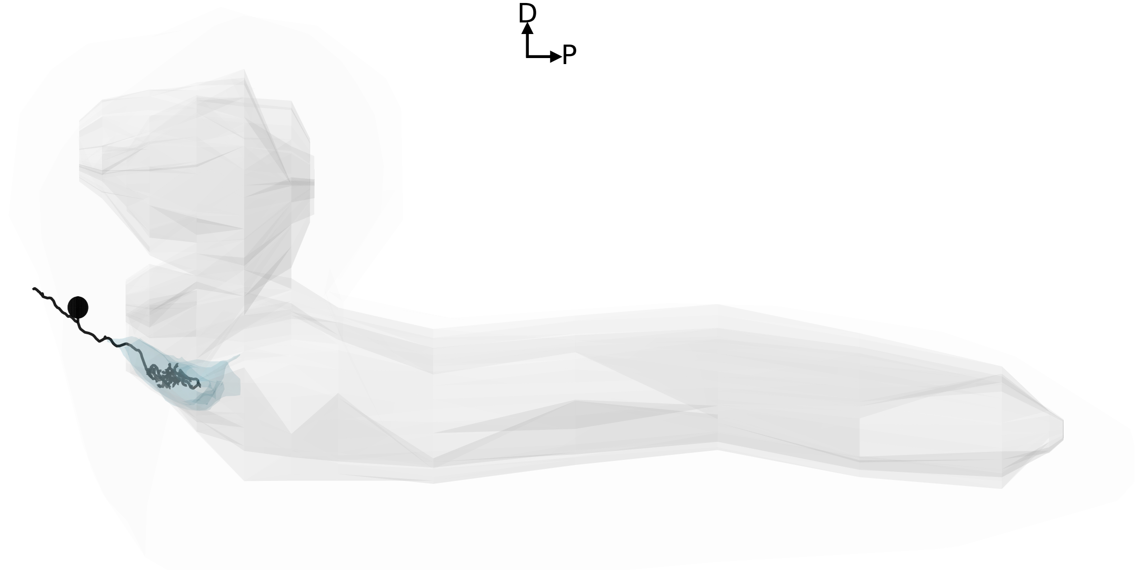

ID	*name*	SCACa	SCAVa	SCAVp	SCACal	SCACp	SCACpl	SCVM	IPCs	DMS	DH44	Se0ens	Se0ph	PMN LR	MN motor neurons	PaN motor neurons	olfactory PNs	gustatory PNs	multiglomerular PNs	unknown PNs	thermo PNs	visual PNs
3340561	AN-R-motor-10	0	0	0	0	0	0	0	0	0	0	0	0	30	0	0	0	0	0	0	0	0

ID: 910064
name: AN-R-motor-11

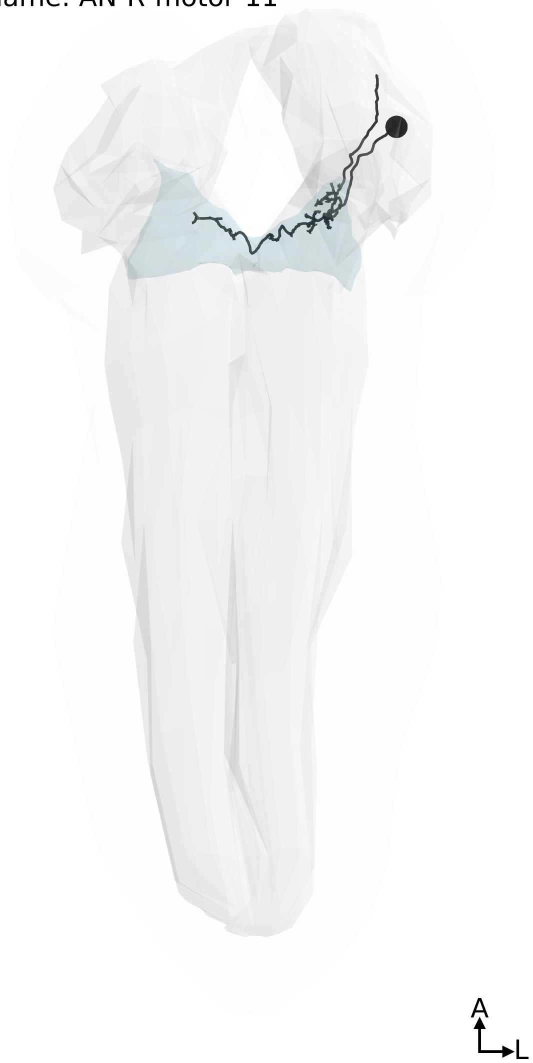

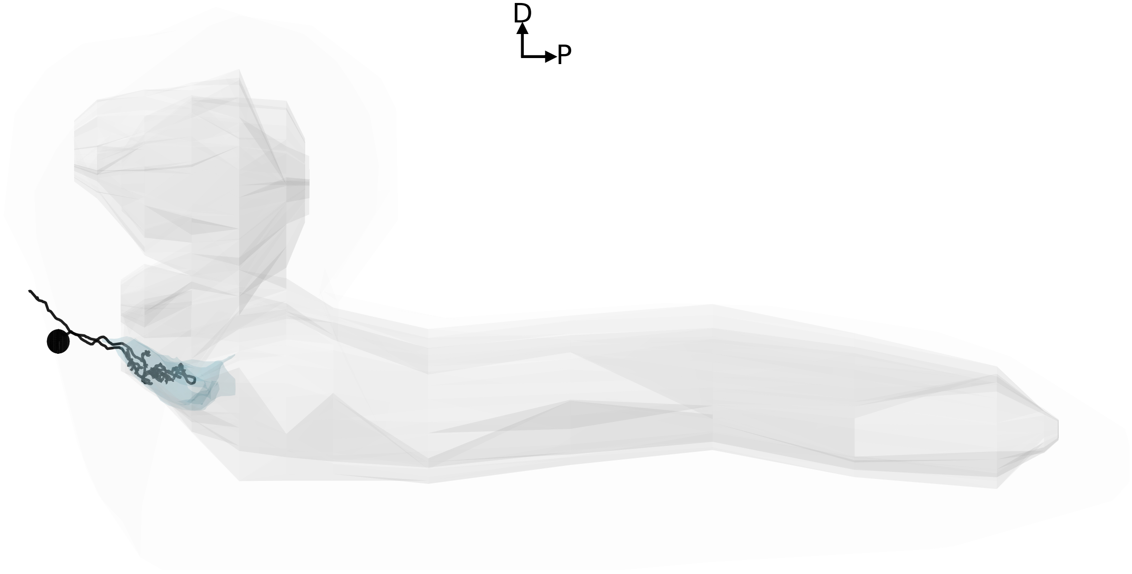

ID	*name*	SCACa	SCAVa	SCAVp	SCACal	SCACp	SCACpl	SCVM	IPCs	DMS	DH44	Se0ens	Se0ph	PMN LR	MN motor neurons	PaN motor neurons	olfactory PNs	gustatory PNs	multiglomerular PNs	unknown PNs	thermo PNs	visual PNs
910064	AN-R-motor-11	0	3	0	0	0	0	0	0	0	0	0	1	46	0	0	0	0	0	0	0	0

ID: 12901662
name: AN-R-motor-12

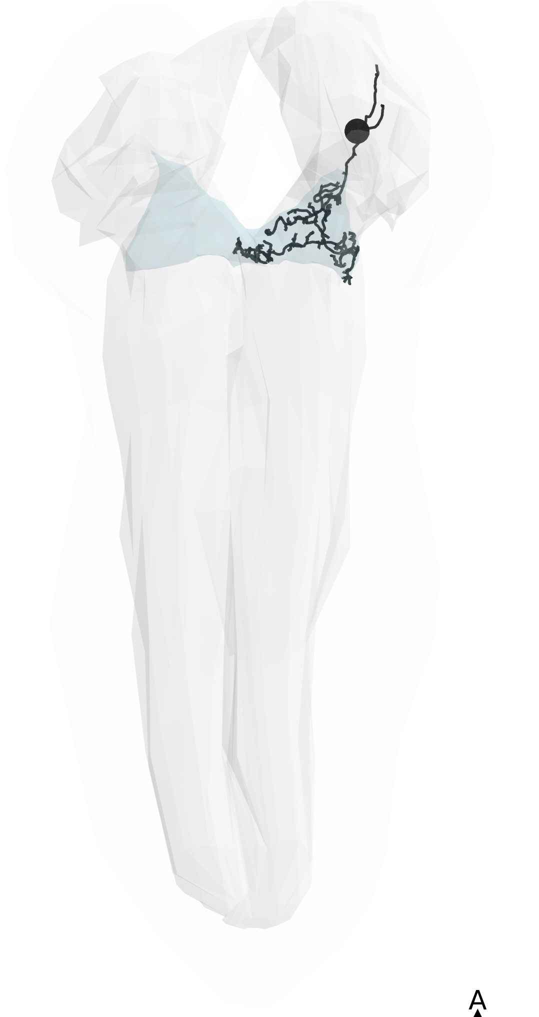

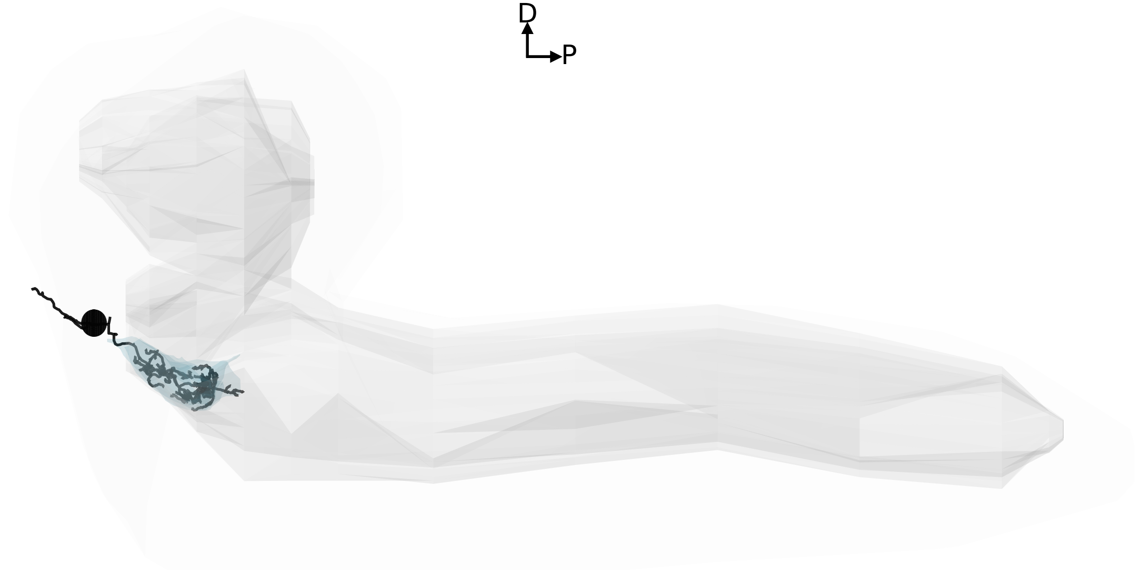

ID	name	SCACa	SCAVa	SCAVp	SCACal	SCACp	SCACpl	SCVM	IPCs	DMS	DH44	Se0ens	Se0ph	PMN LR	MN motor neurons	PaN motor neurons	olfactory PNs	gustatory PNs	multiglomerular PNs	unknown PNs	thermo PNs	visual PNs
12901662	AN-R-motor-12	0	0	0	0	0	0	0	0	0	0	0	0	41	2	0	0	0	0	0	0	0

ID: 873148
name: AN-R-motor-13

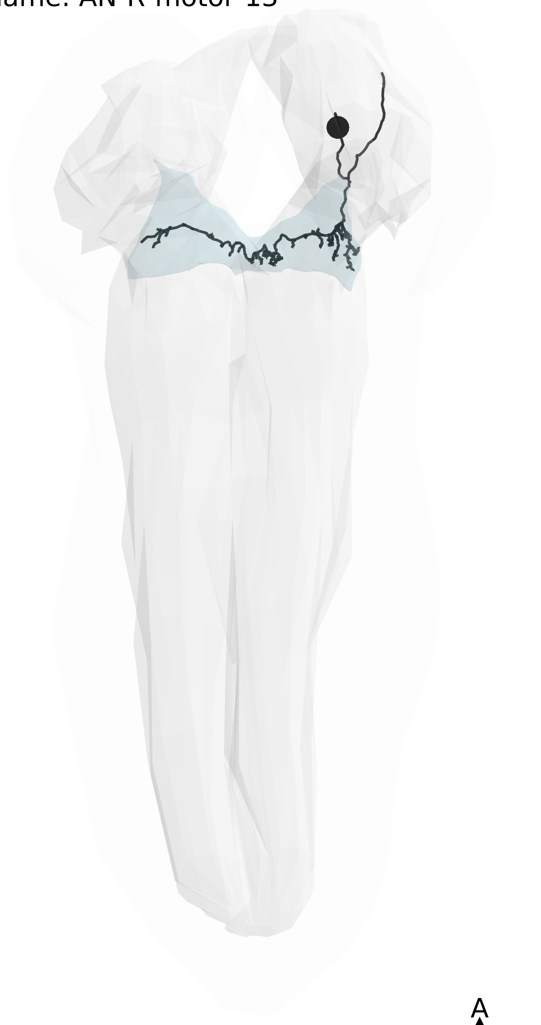

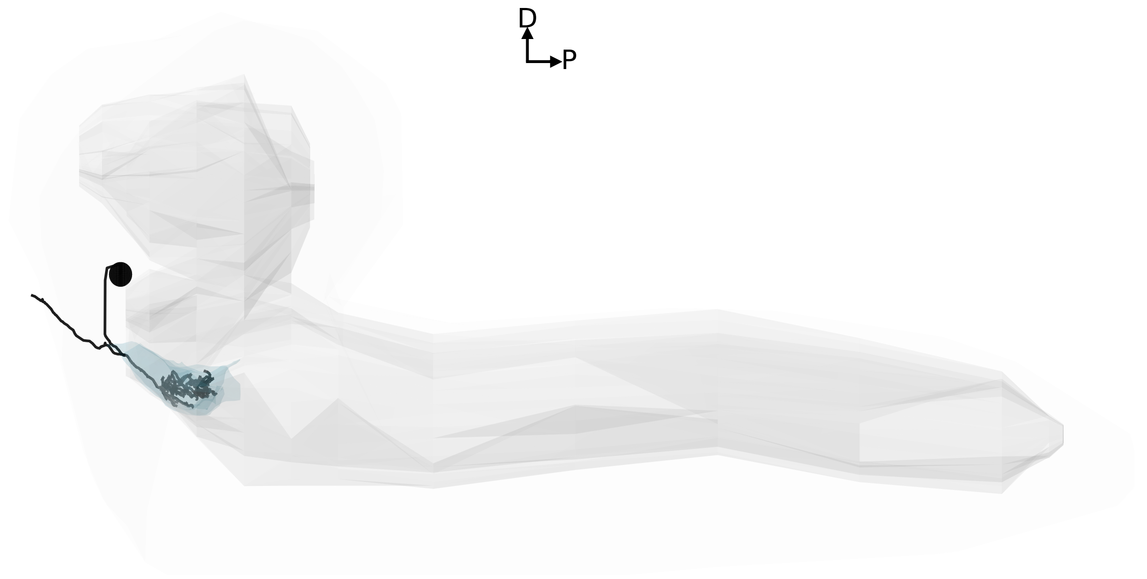

ID	name	SCACa	SCAVa	SCAVp	SCACal	SCACp	SCACpl	SCVM	IPCs	DMS	DH44	Se0ens	Se0ph	PMN LR	MN motor neurons	PaN motor neurons	olfactory PNs	gustatory PNs	multiglomerular PNs	unknown PNs	thermo PNs	visual PNs
873148	AN-R-motor-13	0	0	0	0	0	0	0	0	0	0	0	0	0	0	0	0	0	0	0	0	0

ID: 855719
name: AN-R-motor-14

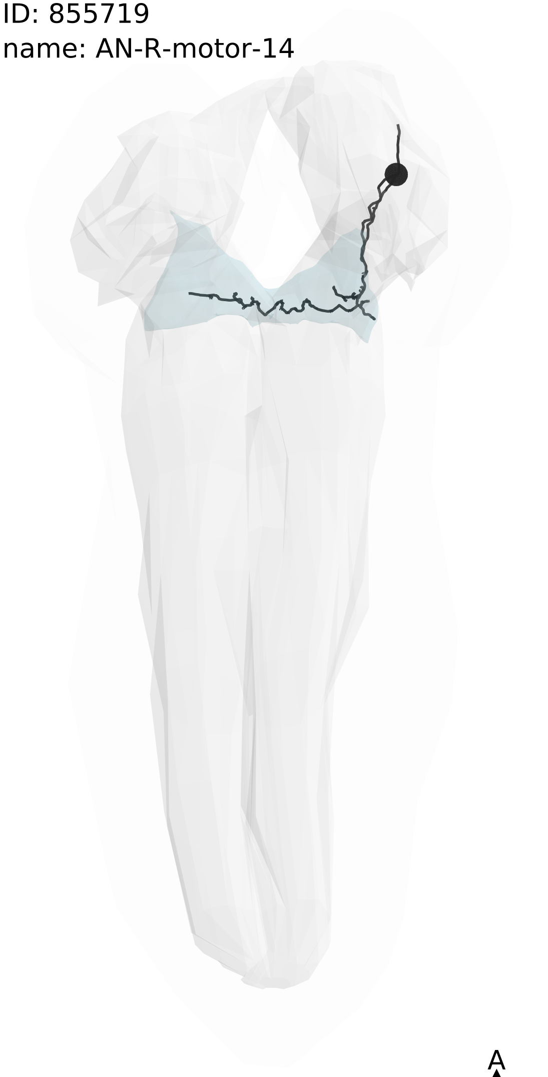

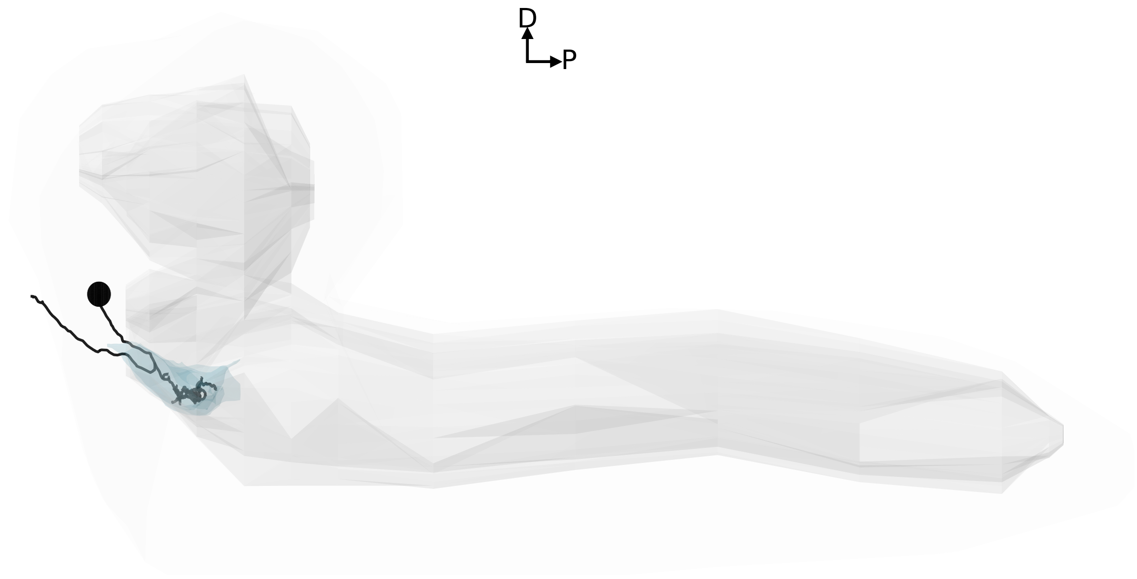

ID	*name*	SCACa	SCAVa	SCAVp	SCACal	SCACp	SCACpl	SCVM	IPCs	DMS	DH44	Se0ens	Se0ph	PMN LR	MN motor neurons	PaN motor neurons	olfactory PNs	gustatory PNs	multiglomerular PNs	unknown PNs	thermo PNs	visual PNs
855719	AN-R-motor-14	0	0	0	0	0	0	0	0	0	0	0	0	0	0	0	0	0	0	0	0	0

ID: 853526
name: AN-R-motor-15

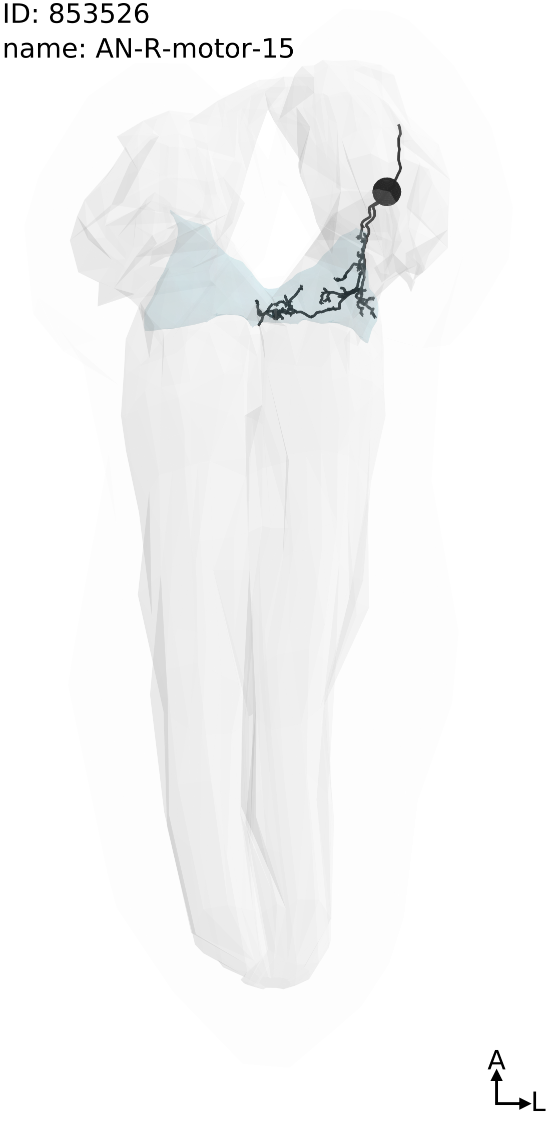

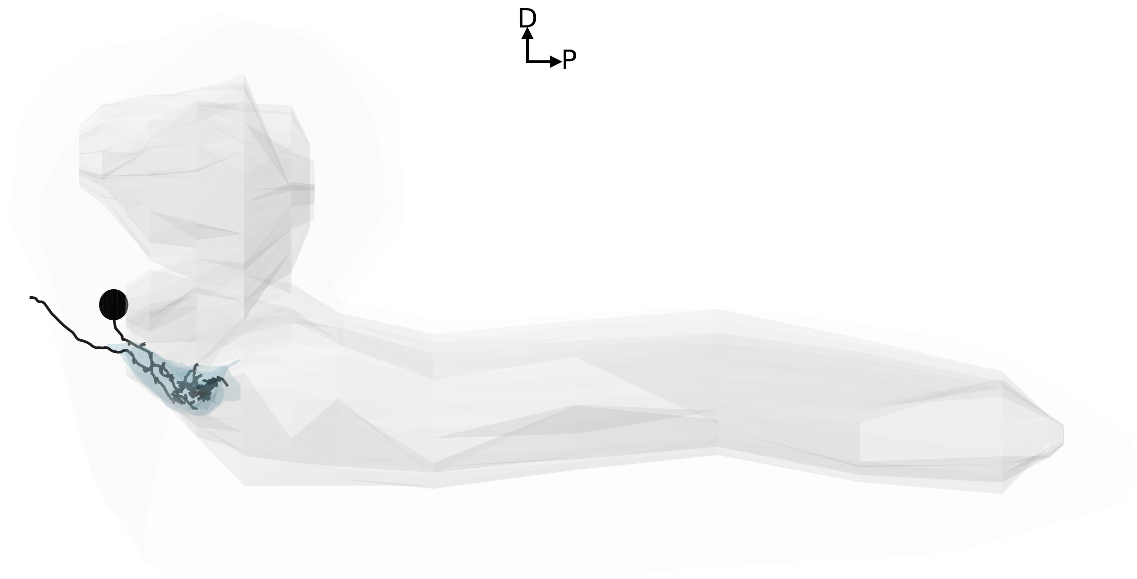

ID	*name*	SCACa	SCAVa	SCAVp	SCACal	SCACp	SCACpl	SCVM	IPCs	DMS	DH44	Se0ens	Se0ph	PMN LR	MN motor neurons	PaN motor neurons	olfactory PNs	gustatory PNs	multiglomerular PNs	unknown PNs	thermo PNs	visual PNs
853526	AN-R-motor-15	0	0	0	0	0	0	0	0	0	0	0	0	0	0	0	0	0	0	0	0	0

ID: 873782
name: AN-R-motor-16

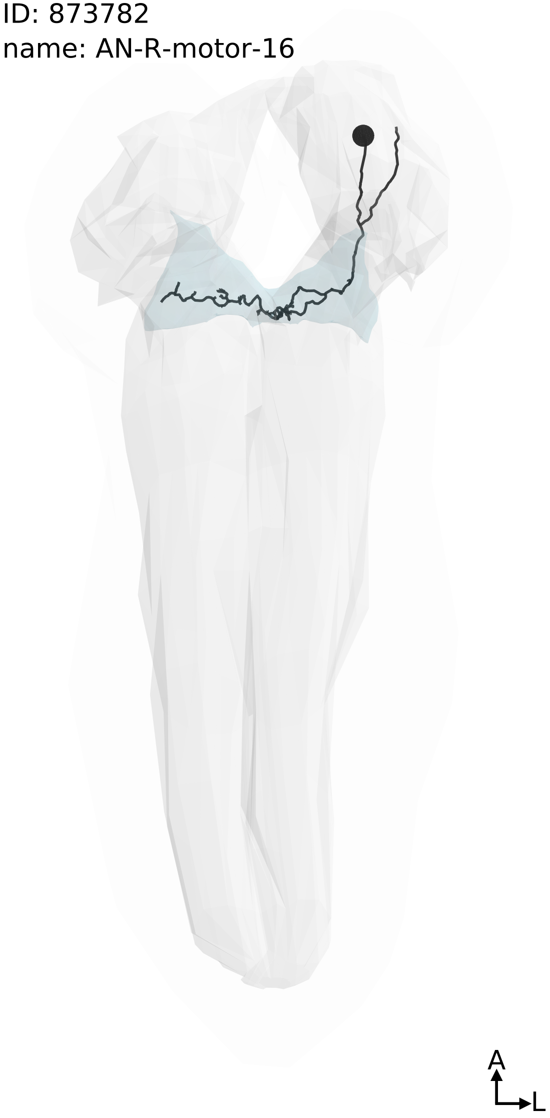

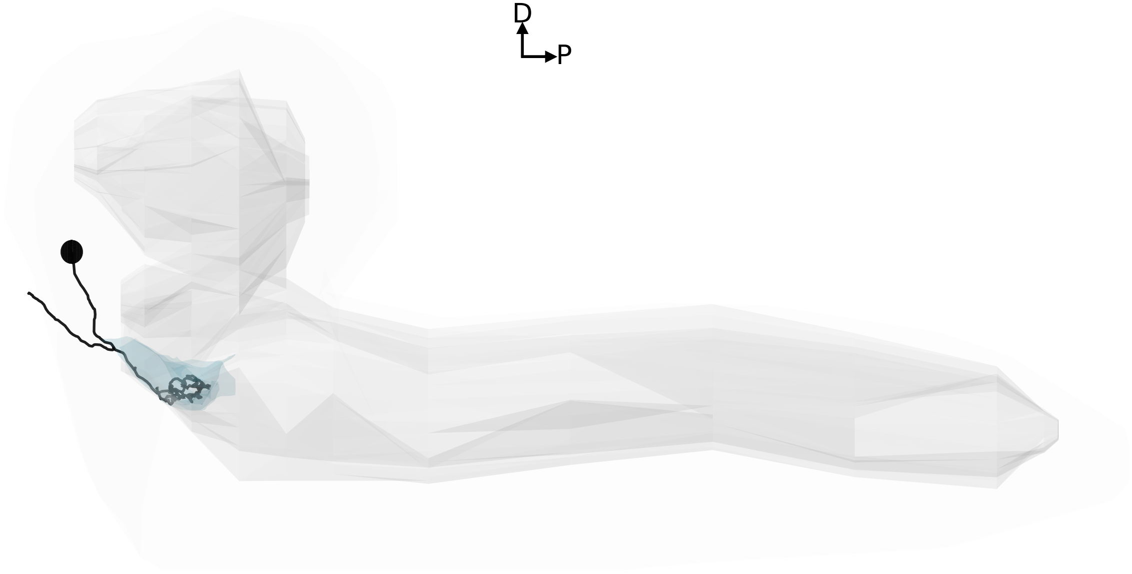

ID	name	SCACa	SCAVa	SCAVp	SCACal	SCACp	SCACpl	SCVM	IPCs	DMS	DH44	Se0ens	Se0ph	PMN LR	MN motor neurons	PaN motor neurons	olfactory PNs	gustatory PNs	multiglomerular PNs	unknown PNs	thermo PNs	visual PNs
873782	AN-R-motor-16	0	0	0	0	0	0	0	0	0	0	0	0	0	0	0	0	0	0	0	0	0

ID: 865801
name: AN-R-motor-17

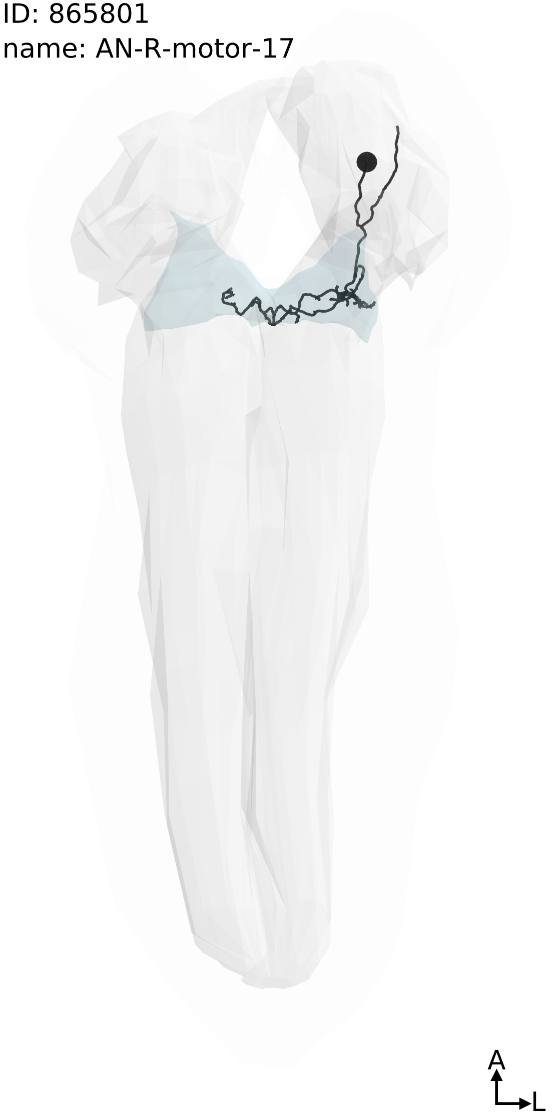

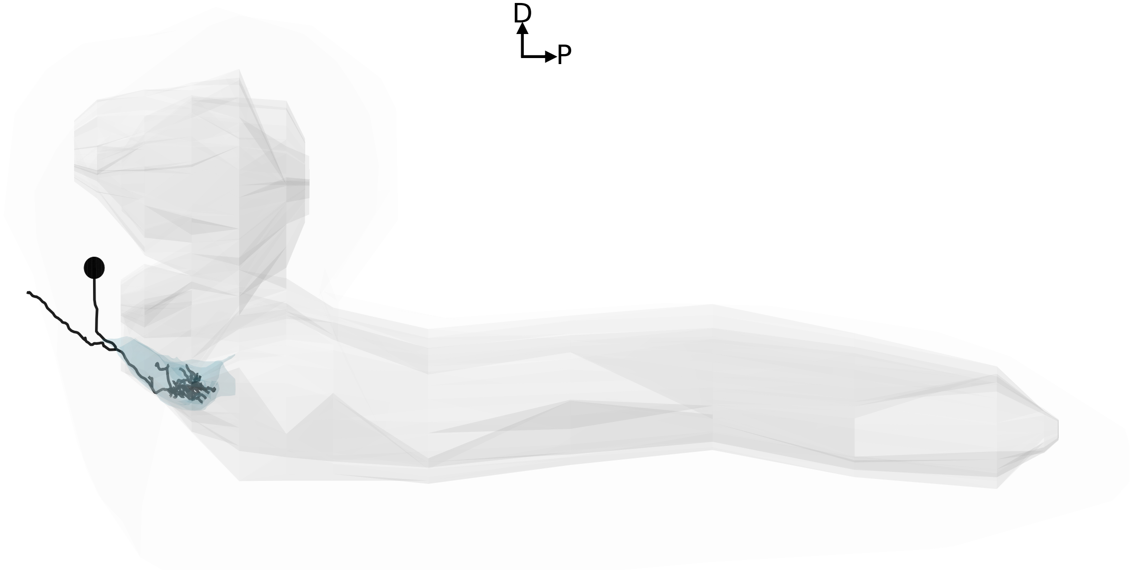

ID	*name*	SCACa	SCAVa	SCAVp	SCACal	SCACp	SCACpl	SCVM	IPCs	DMS	DH44	Se0ens	Se0ph	PMN LR	MN motor neurons	PaN motor neurons	olfactory PNs	gustatory PNs	multiglomerular PNs	unknown PNs	thermo PNs	visual PNs
865801	AN-R-motor-17	0	0	0	0	0	0	0	0	0	0	0	0	0	0	0	0	0	0	0	0	0

ID: 937332
name: AN-R-motor-18

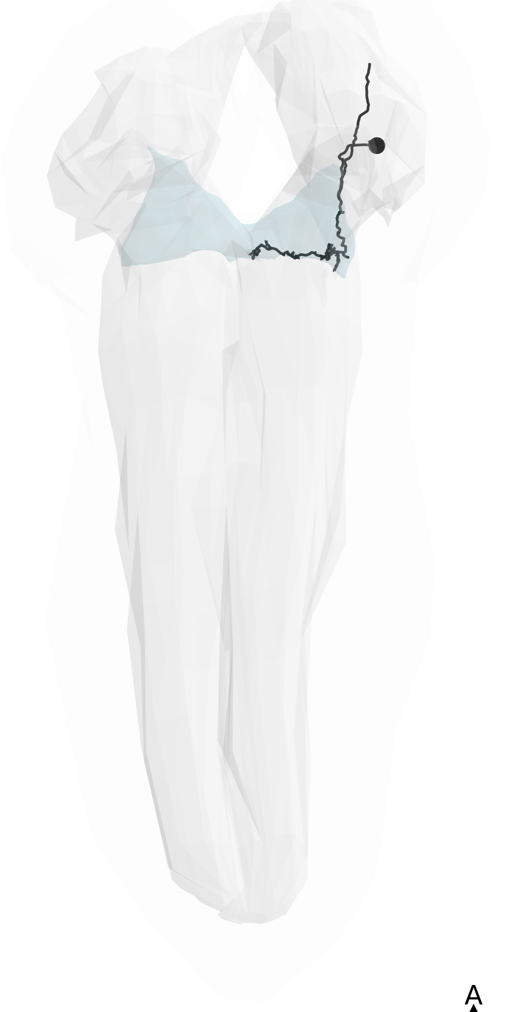

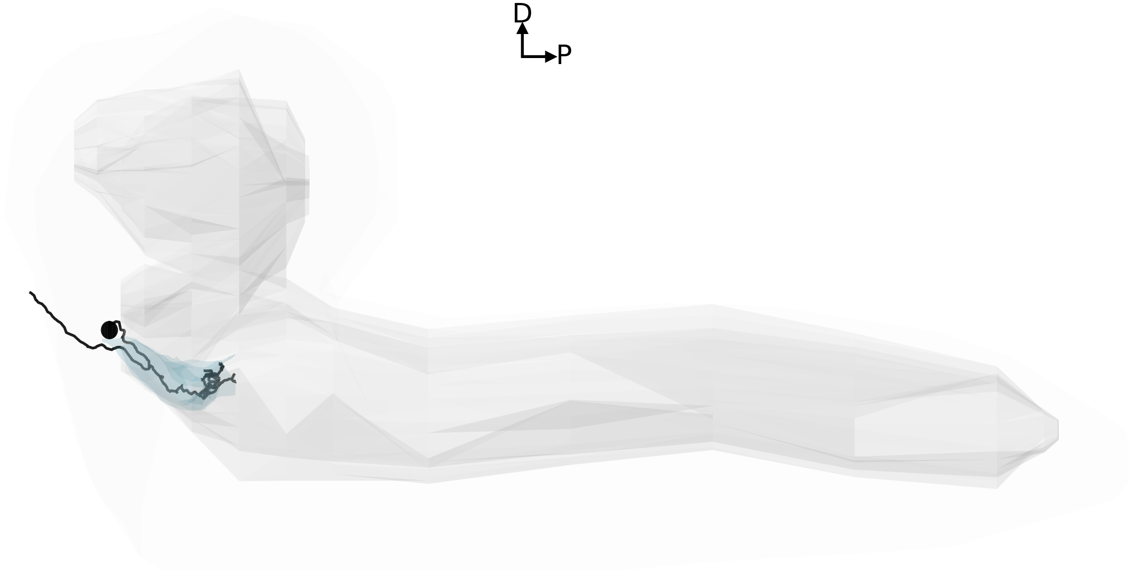

ID	name	SCACa	SCAVa	SCAVp	SCACal	SCACp	SCACpl	SCVM	IPCs	DMS	DH44	Se0ens	Se0ph	PMN LR	MN motor neurons	PaN motor neurons	olfactory PNs	gustatory PNs	multiglomerular PNs	unknown PNs	thermo PNs	visual PNs
937332	AN-R-motor-18	0	0	0	0	0	0	0	0	0	0	0	0	0	0	0	0	0	0	0	0	0

ID: 8208555
name: MN-L-motor-01

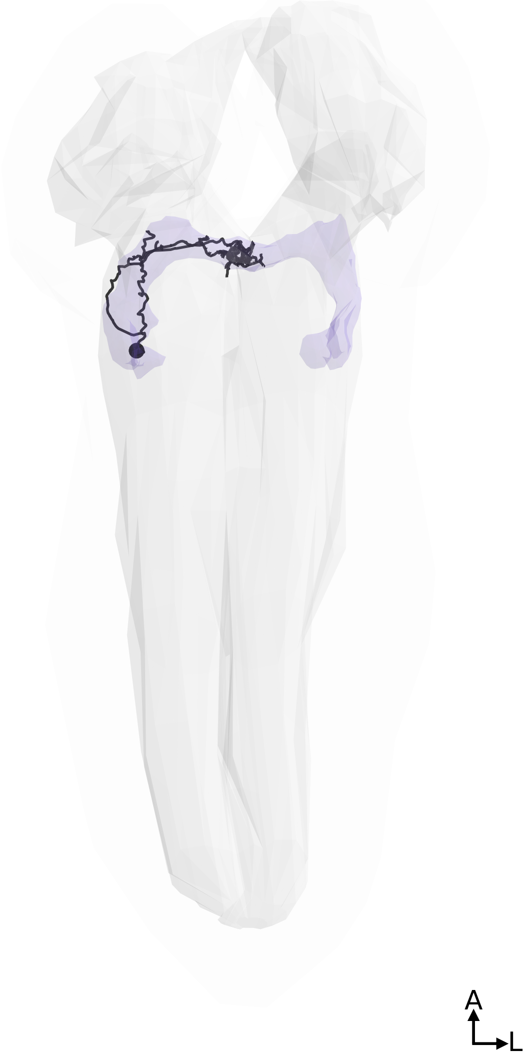

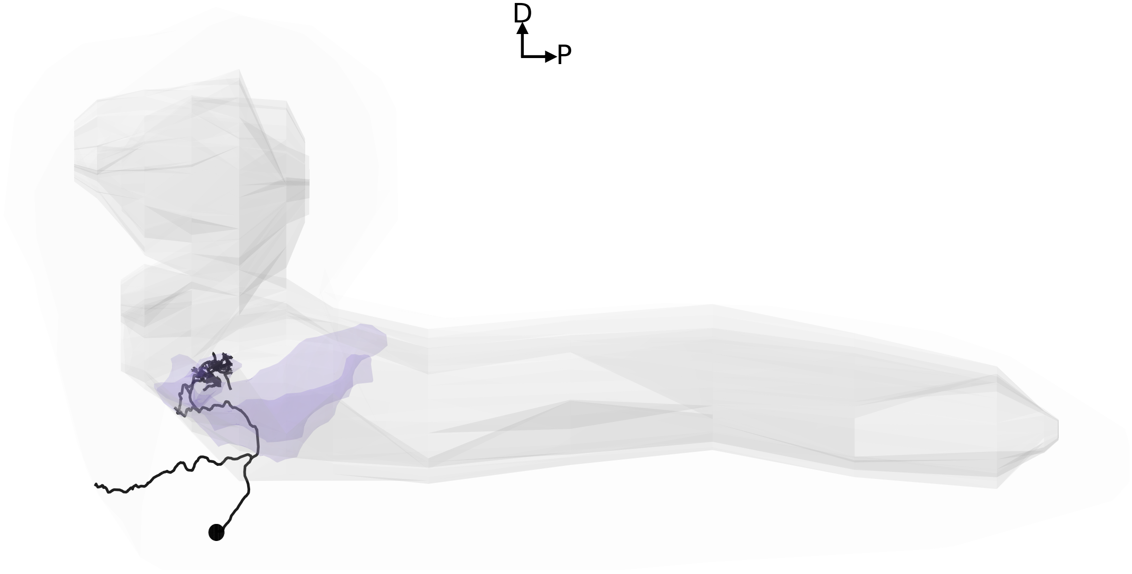

ID	*name*	SCACa	SCAVa	SCAVp	SCACal	SCACp	SCACpl	SCVM	IPCs	DMS	DH44	Se0ens	Se0ph	PMN LR	MN motor neurons	PaN motor neurons	olfactory PNs	gustatory PNs	multiglomerular PNs	unknown PNs	thermo PNs	visual PNs
8208555	MN-L-motor-01	0	0	0	0	0	0	0	0	0	0	0	0	0	0	0	0	0	0	0	0	0

ID: 8105236
name: MN-L-motor-02

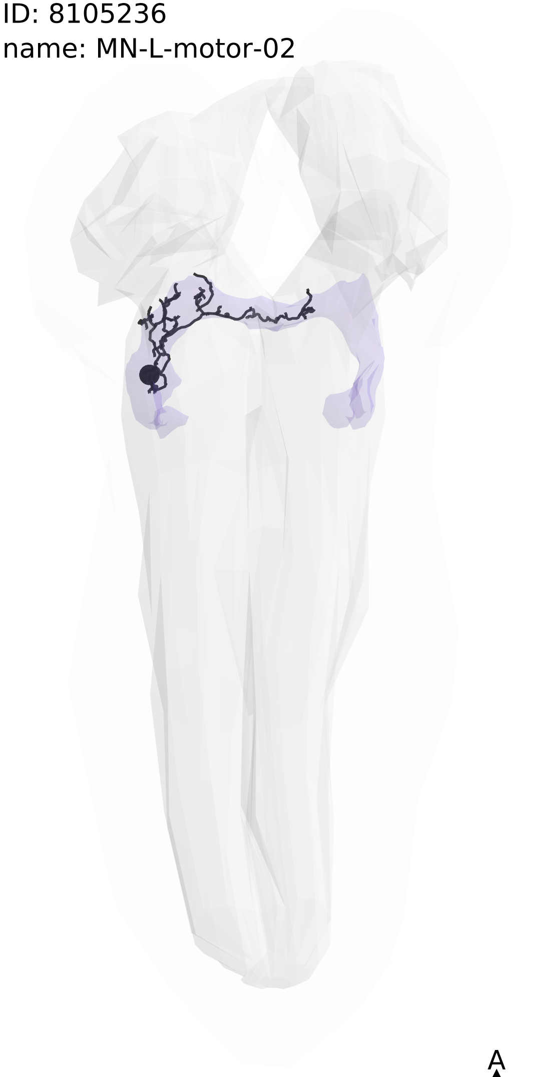

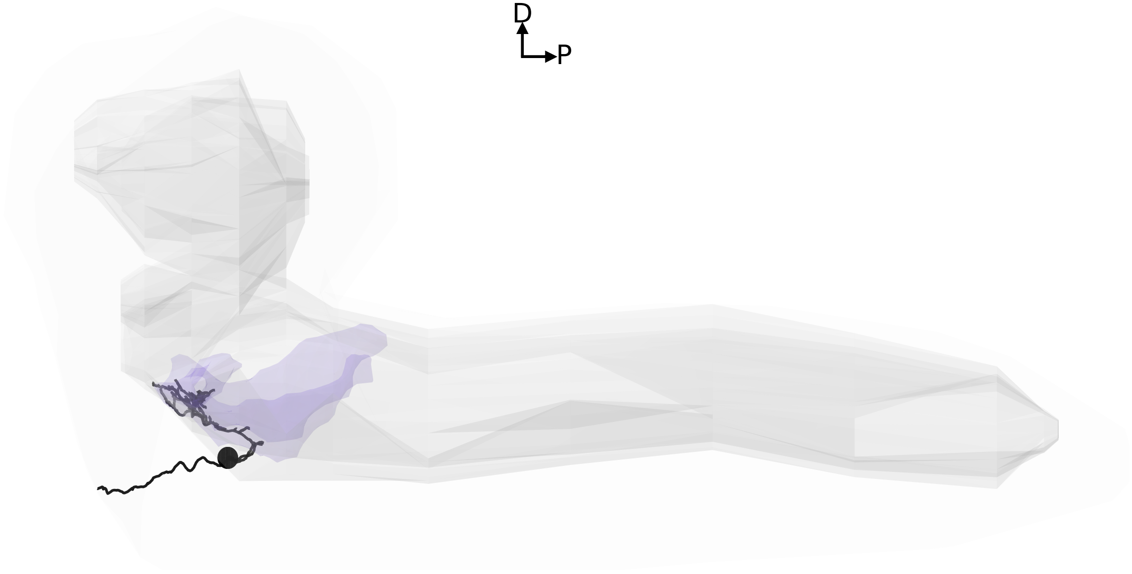

ID	*name*	SCACa	SCAVa	SCAVp	SCACal	SCACp	SCACpl	SCVM	IPCs	DMS	DH44	Se0ens	Se0ph	PMN LR	MN motor neurons	PaN motor neurons	olfactory PNs	gustatory PNs	multiglomerular PNs	unknown PNs	thermo PNs	visual PNs
8105236	MN-L-motor-02	0	0	0	0	0	0	0	0	0	0	0	0	0	0	0	0	0	0	0	0	0

ID: 8092228
name: MN-L-motor-03

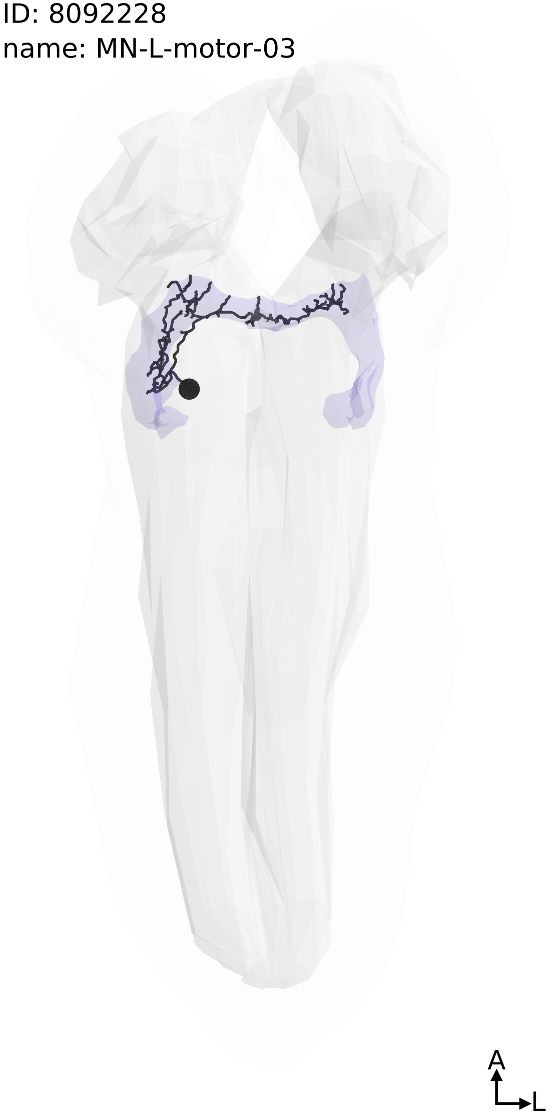

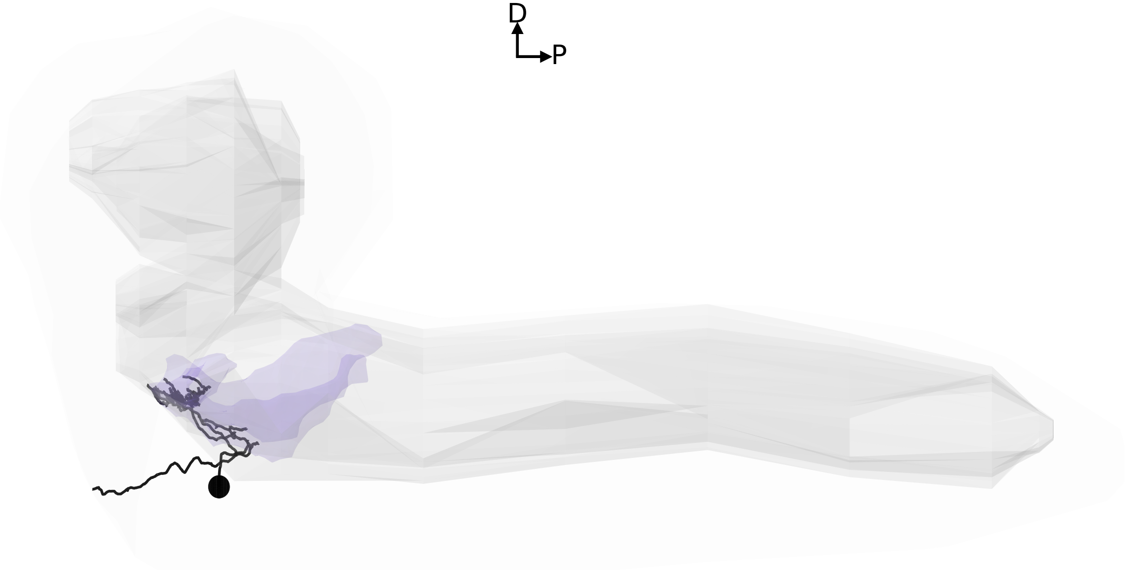

ID	*name*	SCACa	SCAVa	SCAVp	SCACal	SCACp	SCACpl	SCVM	IPCs	DMS	DH44	Se0ens	Se0ph	PMN LR	MN motor neurons	PaN motor neurons	olfactory PNs	gustatory PNs	multiglomerular PNs	unknown PNs	thermo PNs	visual PNs
8092228	MN-L-motor-03	0	0	0	0	0	0	0	0	0	0	0	0	0	0	0	0	0	0	0	0	0

ID: 5453143
name: MN-L-motor-04

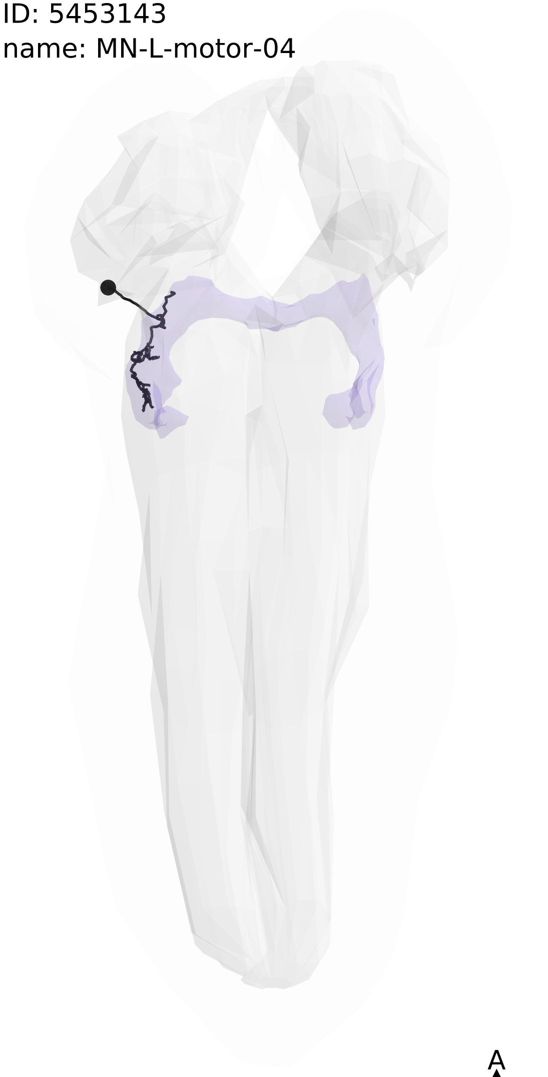

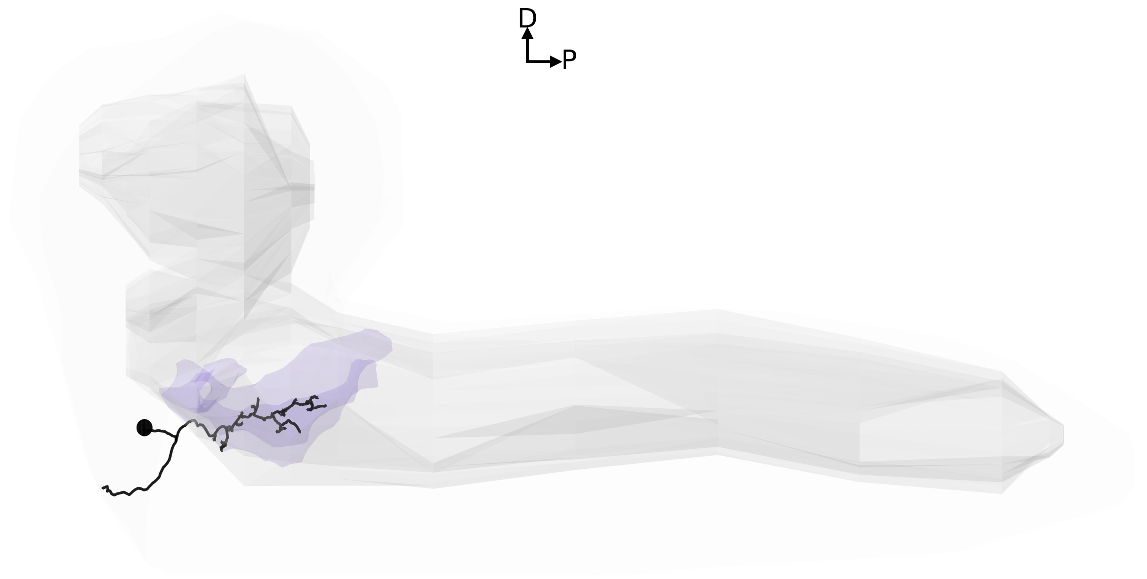

ID	*name*	SCACa	SCAVa	SCAVp	SCACal	SCACp	SCACpl	SCVM	IPCs	DMS	DH44	Se0ens	Se0ph	PMN LR	MN motor neurons	PaN motor neurons	olfactory PNs	gustatory PNs	multiglomerular PNs	unknown PNs	thermo PNs	visual PNs
5453143	MN-L-motor-04	0	0	0	0	0	0	0	0	0	0	0	0	0	0	0	0	0	0	0	0	0

ID: 5450669
name: MN-L-motor-05

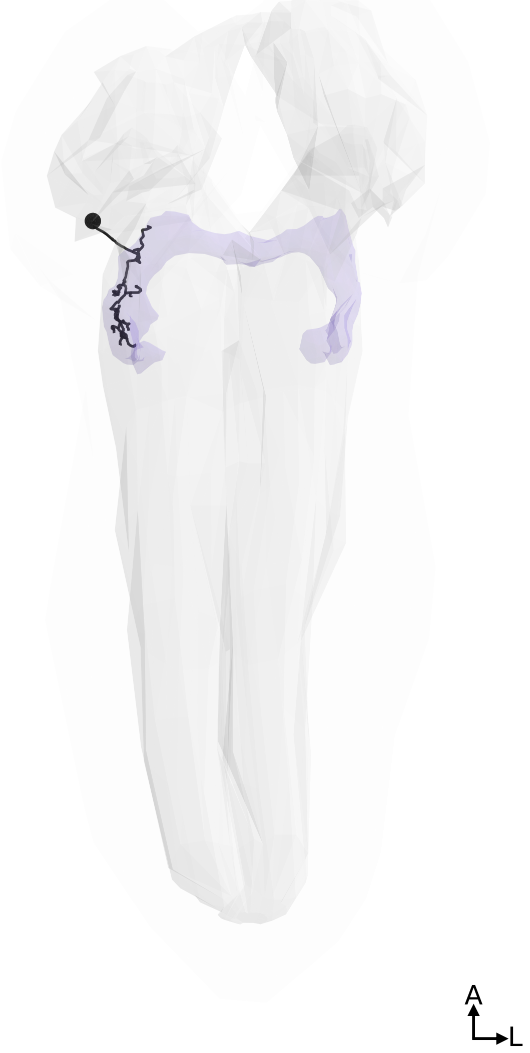

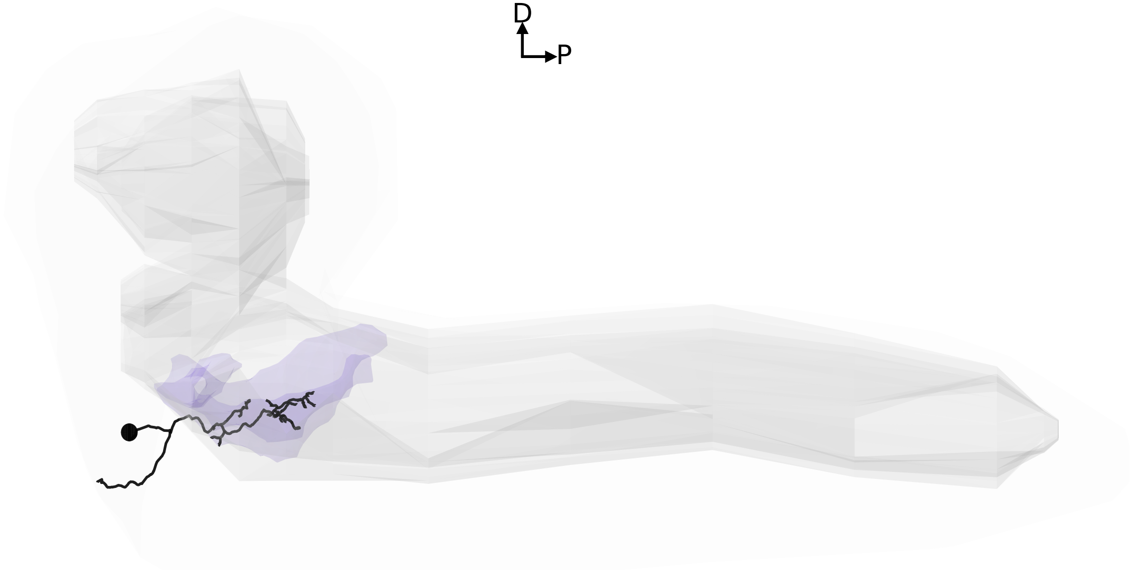

ID	*name*	SCACa	SCAVa	SCAVp	SCACal	SCACp	SCACpl	SCVM	IPCs	DMS	DH44	Se0ens	Se0ph	PMN LR	MN motor neurons	PaN motor neurons	olfactory PNs	gustatory PNs	multiglomerular PNs	unknown PNs	thermo PNs	visual PNs
5450669	MN-L-motor-05	0	0	0	0	0	0	0	0	0	0	0	0	0	0	0	0	0	0	0	0	0

ID: 5452286
name: MN-L-motor-06

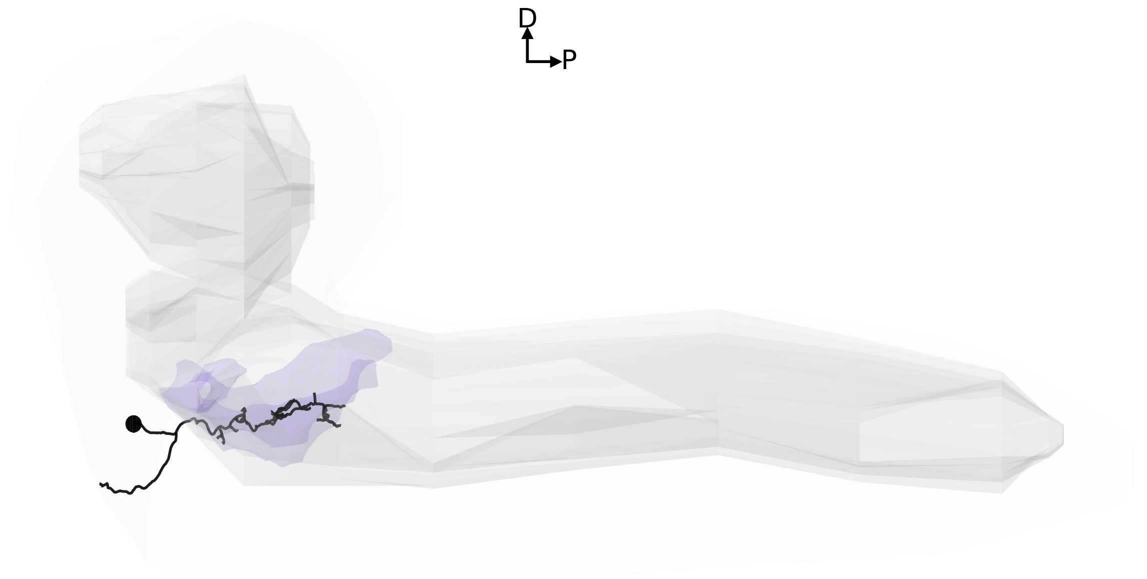

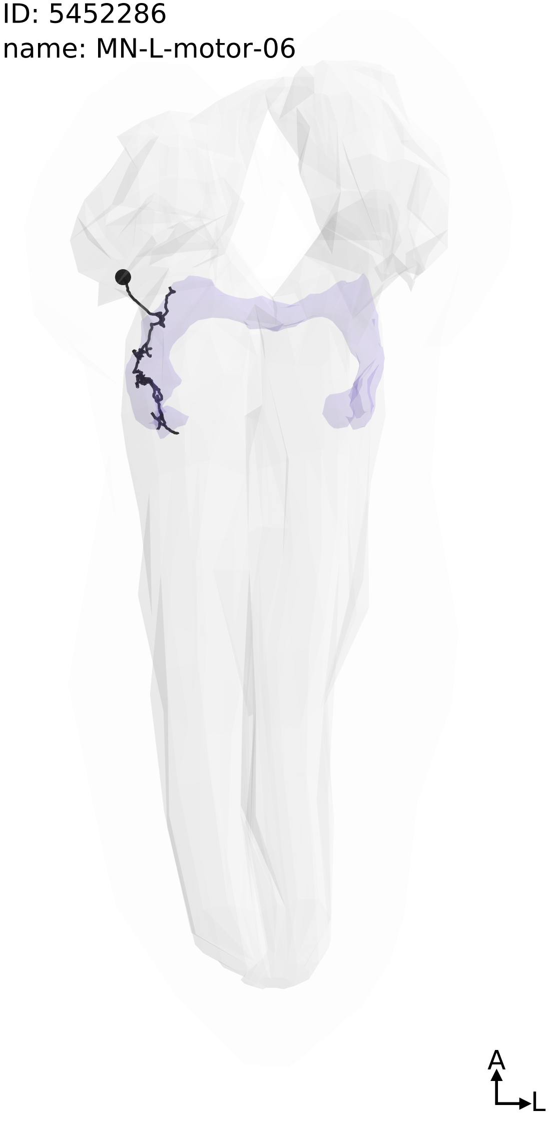

ID	*name*	SCACa	SCAVa	SCAVp	SCACal	SCACp	SCACpl	SCVM	IPCs	DMS	DH44	Se0ens	Se0ph	PMN LR	MN motor neurons	PaN motor neurons	olfactory PNs	gustatory PNs	multiglomerular PNs	unknown PNs	thermo PNs	visual PNs
5452286	MN-L-motor-06	0	0	0	0	0	0	0	0	0	0	0	0	0	0	0	0	0	0	0	0	0

ID: 8061047
name: MN-L-motor-07

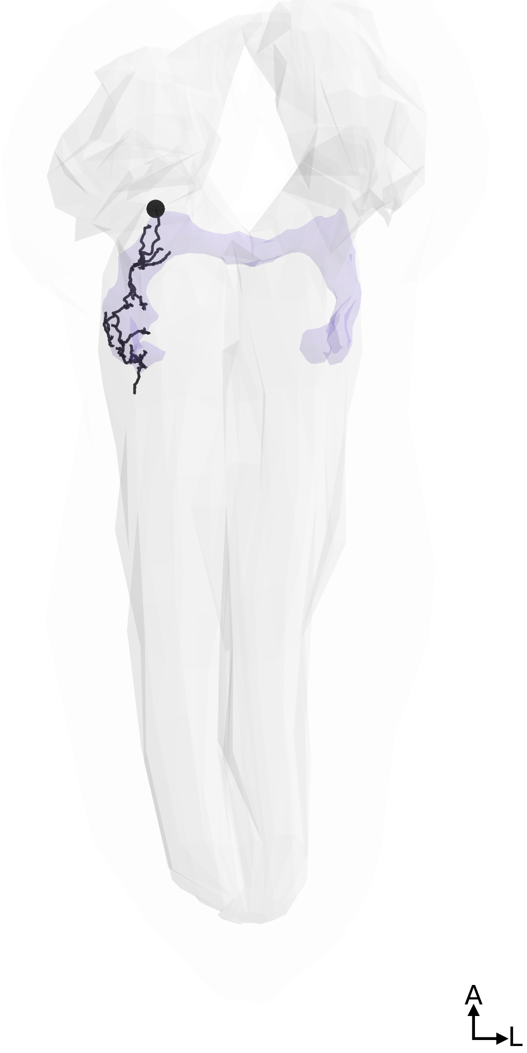

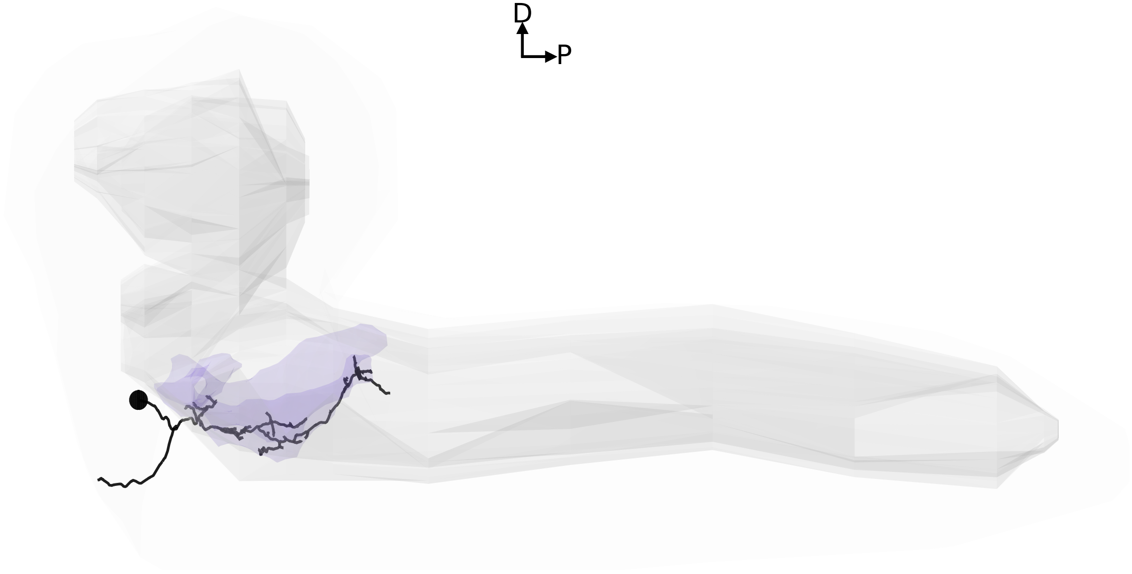

ID	*name*	SCACa	SCAVa	SCAVp	SCACal	SCACp	SCACpl	SCVM	IPCs	DMS	DH44	Se0ens	Se0ph	PMN LR	MN motor neurons	PaN motor neurons	olfactory PNs	gustatory PNs	multiglomerular PNs	unknown PNs	thermo PNs	visual PNs
8061047	MN-L-motor-07	0	0	0	0	0	0	0	0	0	0	0	0	0	0	0	0	0	0	0	0	0

ID: 8084863
name: MN-L-motor-08

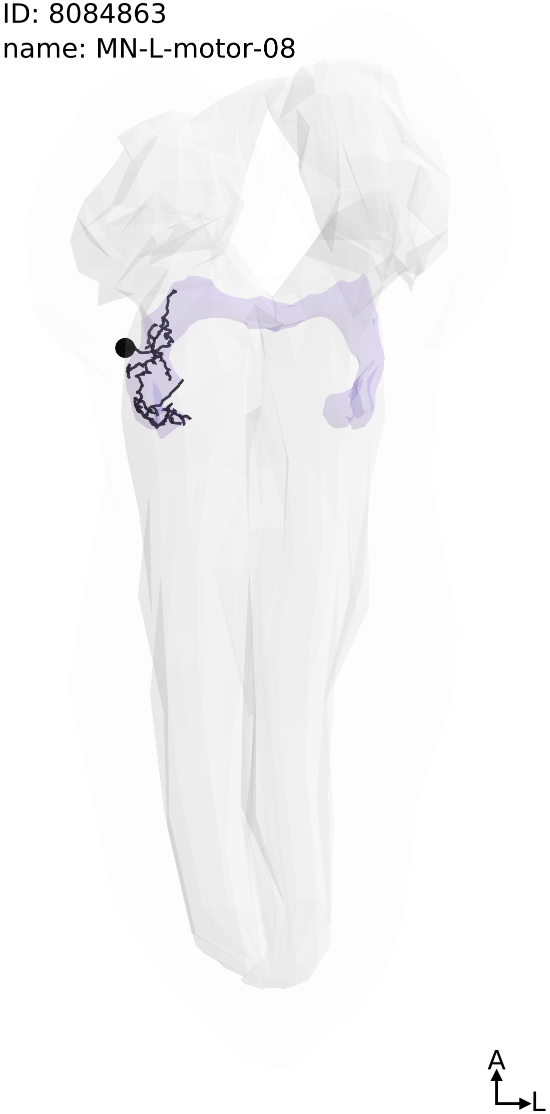

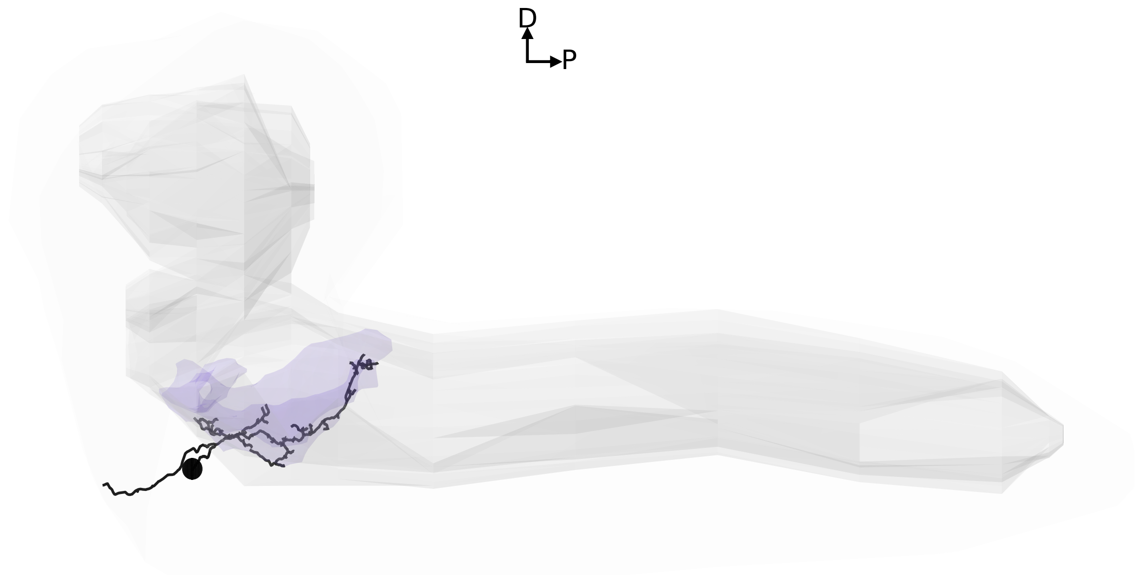

ID	*name*	SCACa	SCAVa	SCAVp	SCACal	SCACp	SCACpl	SCVM	IPCs	DMS	DH44	Se0ens	Se0ph	PMN LR	MN motor neurons	PaN motor neurons	olfactory PNs	gustatory PNs	multiglomerular PNs	unknown PNs	thermo PNs	visual PNs
8084863	MN-L-motor-08	0	0	0	0	0	0	0	0	0	0	0	0	0	0	0	0	0	0	0	0	0

ID: 8435311
name: MN-L-motor-09

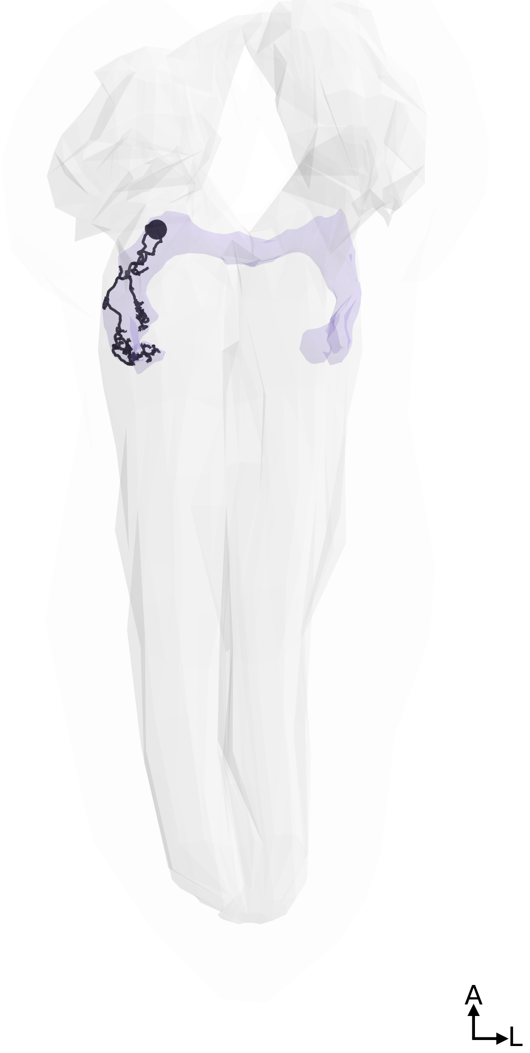

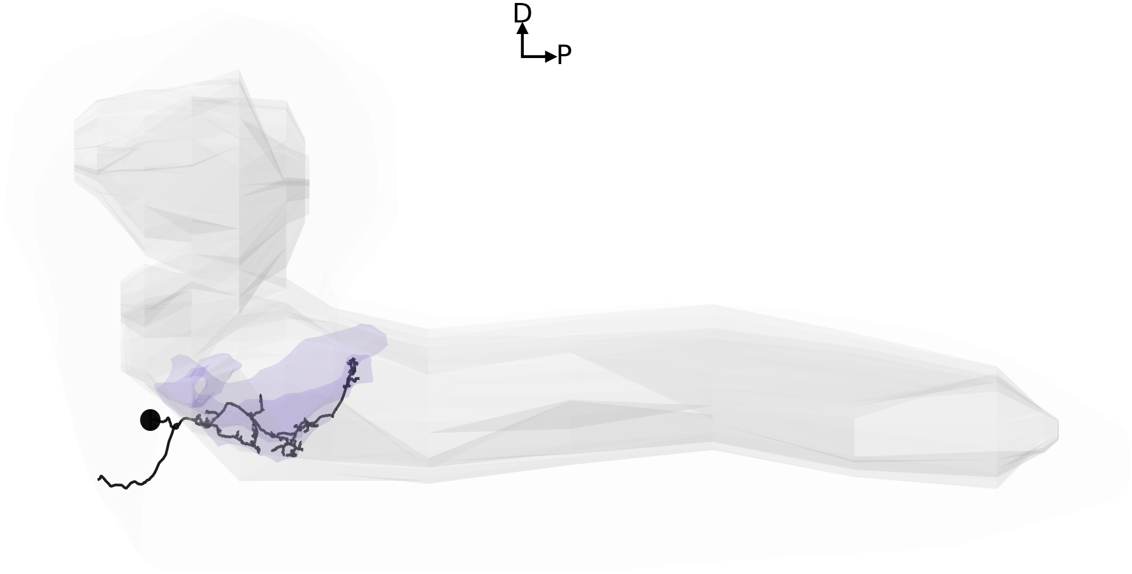

ID	*name*	SCACa	SCAVa	SCAVp	SCACal	SCACp	SCACpl	SCVM	IPCs	DMS	DH44	Se0ens	Se0ph	PMN LR	MN motor neurons	PaN motor neurons	olfactory PNs	gustatory PNs	multiglomerular PNs	unknown PNs	thermo PNs	visual PNs
8435311	MN-L-motor-09	0	0	0	0	0	0	0	0	0	0	0	0	0	0	0	0	0	0	0	0	0

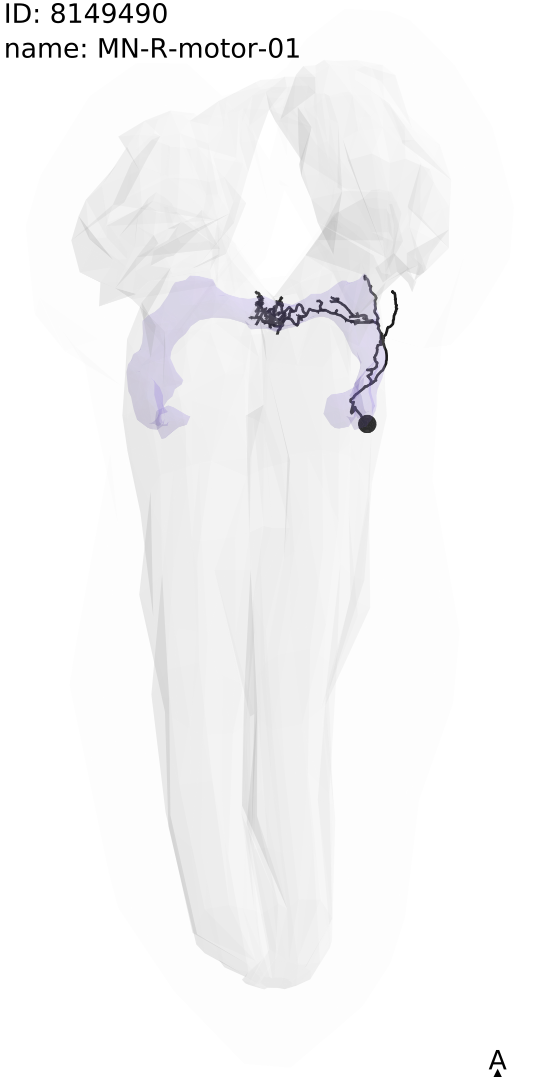

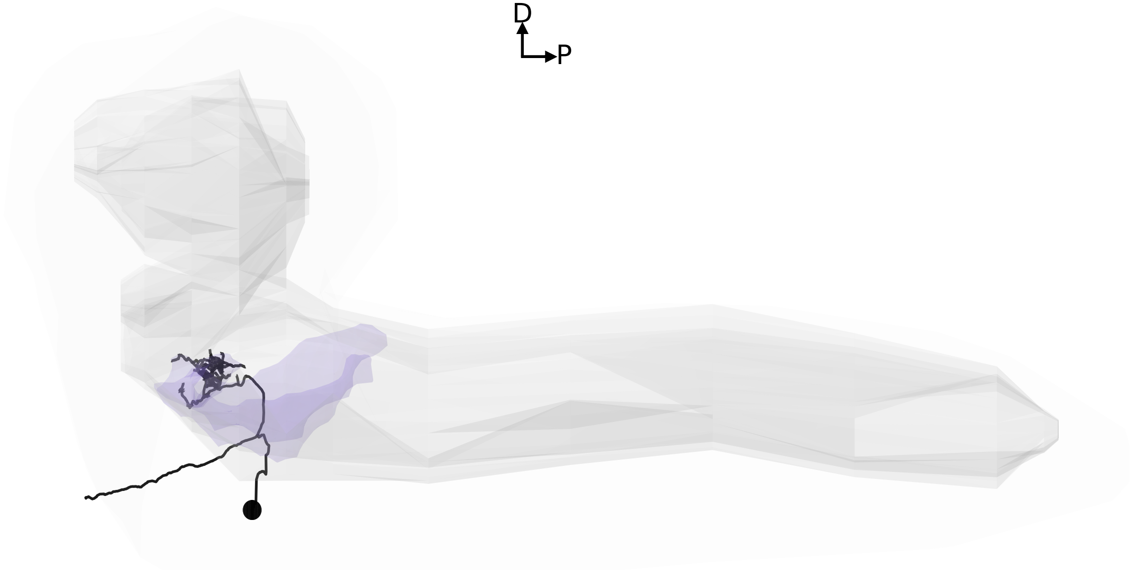

ID	*name*	SCACa	SCAVa	SCAVp	SCACal	SCACp	SCACpl	SCVM	IPCs	DMS	DH44	Se0ens	Se0ph	PMN LR	MN motor neurons	PaN motor neurons	olfactory PNs	gustatory PNs	multiglomerular PNs	unknown PNs	thermo PNs	visual PNs
8149490	MN-R-motor-01	0	0	0	0	0	0	0	0	0	0	0	0	0	0	0	0	0	0	0	0	0

ID: 8167773
name: MN-R-motor-02

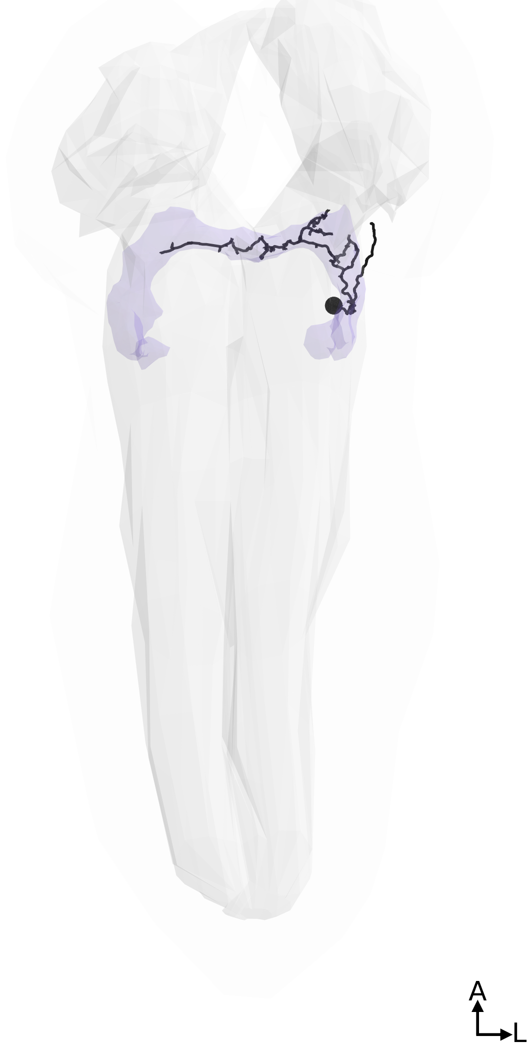

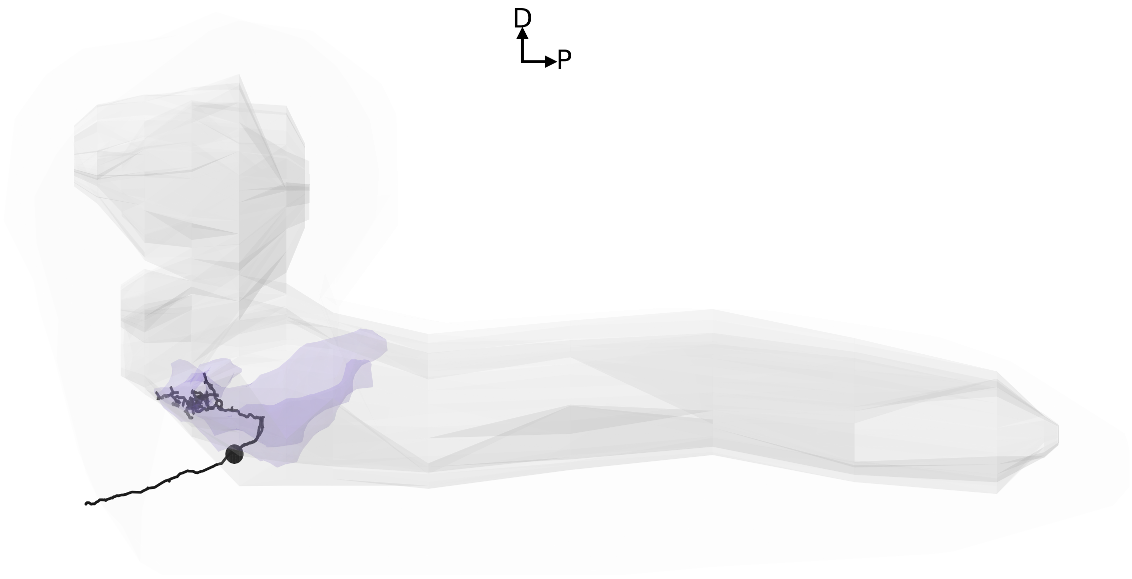

ID	*name*	SCACa	SCAVa	SCAVp	SCACal	SCACp	SCACpl	SCVM	IPCs	DMS	DH44	Se0ens	Se0ph	PMN LR	MN motor neurons	PaN motor neurons	olfactory PNs	gustatory PNs	multiglomerular PNs	unknown PNs	thermo PNs	visual PNs
8167773	MN-R-motor-02	0	0	0	0	0	0	0	0	0	0	0	0	0	0	0	0	0	0	0	0	0

ID: 8177700
name: MN-R-motor-03

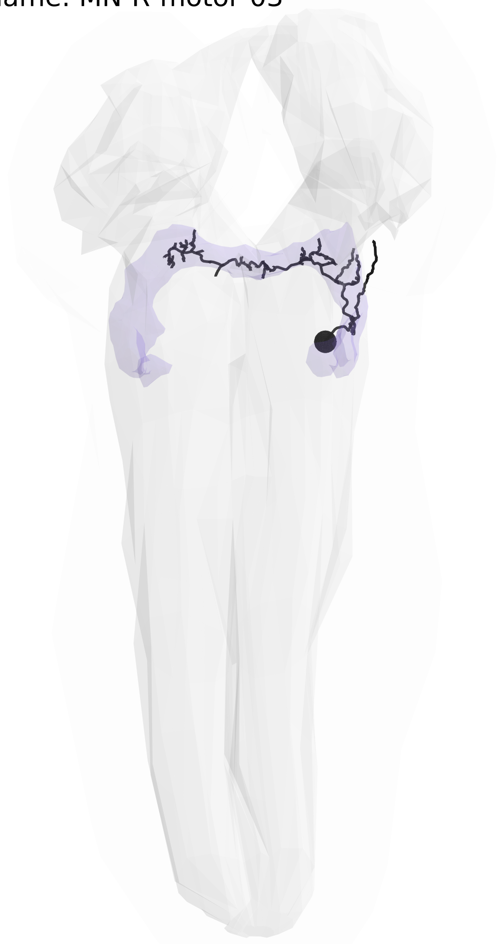

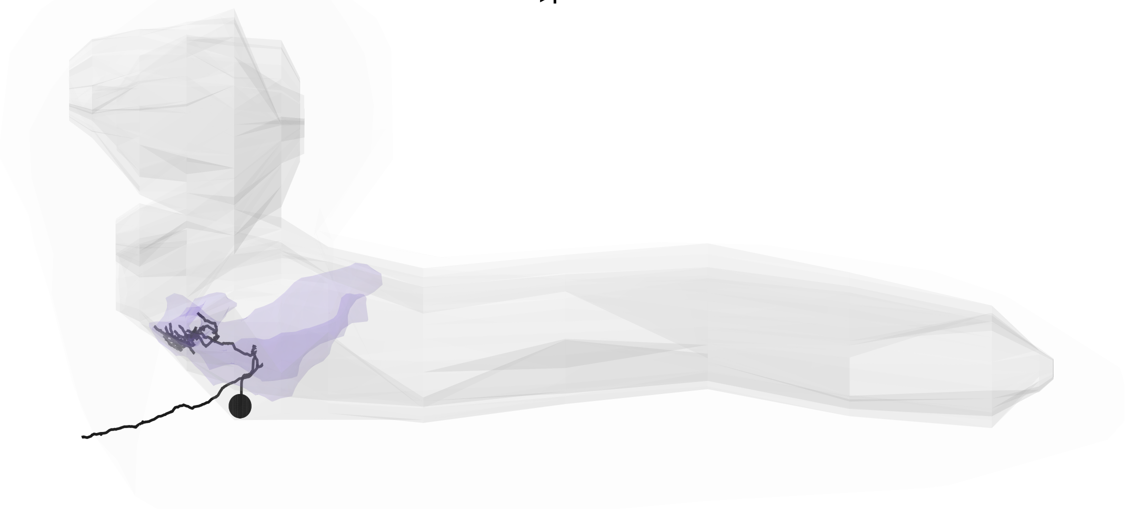

ID	name	SCACa	SCAVa	SCAVp	SCACal	SCACp	SCACpl	SCVM	IPCs	DMS	DH44	Se0ens	Se0ph	PMN LR	MN motor neurons	PaN motor neurons	olfactory PNs	gustatory PNs	multiglomerular PNs	unknown PNs	thermo PNs	visual PNs
8177700	MN-R-motor-03	0	0	0	0	0	0	0	0	0	0	0	0	0	0	0	0	0	0	0	0	0

ID: 5435750
name: MN-R-motor-04

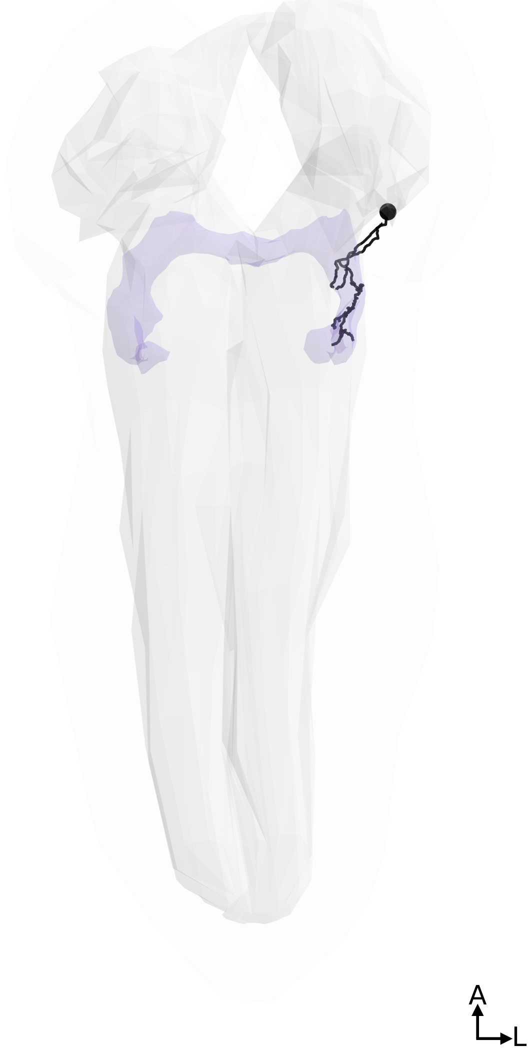

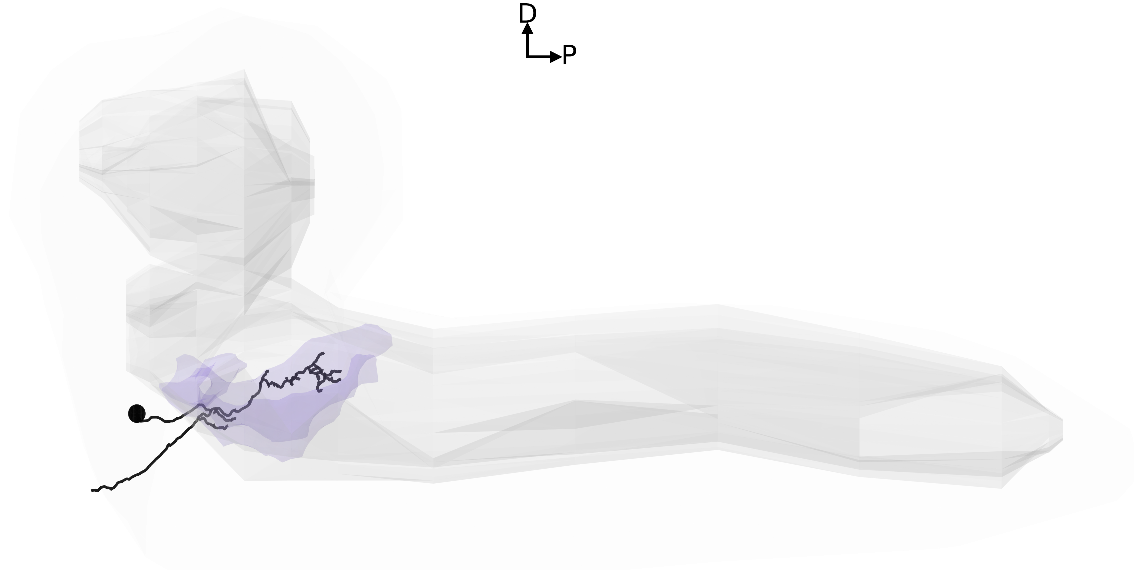

ID	*name*	SCACa	SCAVa	SCAVp	SCACal	SCACp	SCACpl	SCVM	IPCs	DMS	DH44	Se0ens	Se0ph	PMN LR	MN motor neurons	PaN motor neurons	olfactory PNs	gustatory PNs	multiglomerular PNs	unknown PNs	thermo PNs	visual PNs
5435750	MN-R-motor-04	0	0	0	0	0	0	0	0	0	0	0	0	0	0	0	0	0	0	0	0	0

ID: 5434746
name: MN-R-motor-05

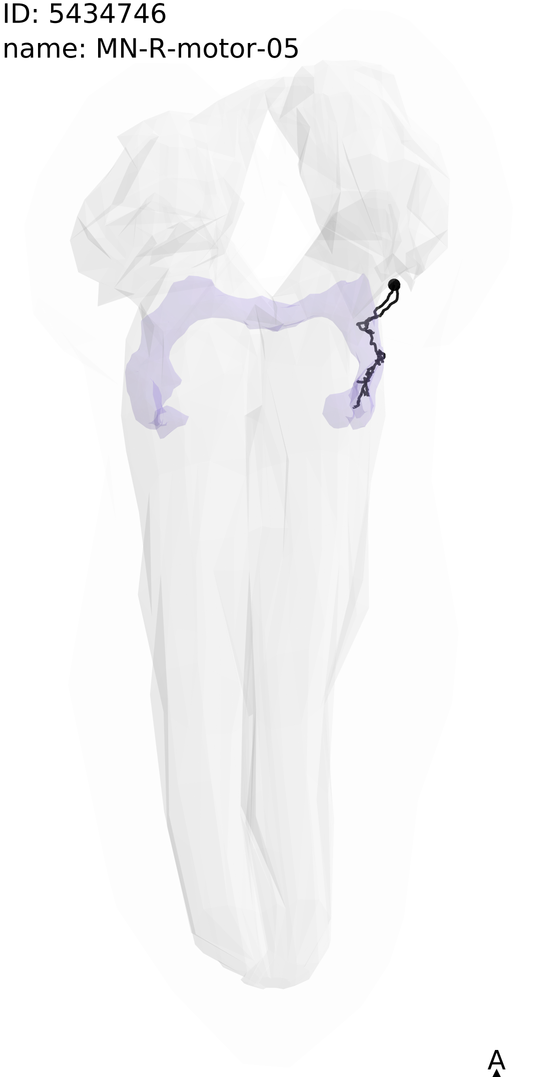

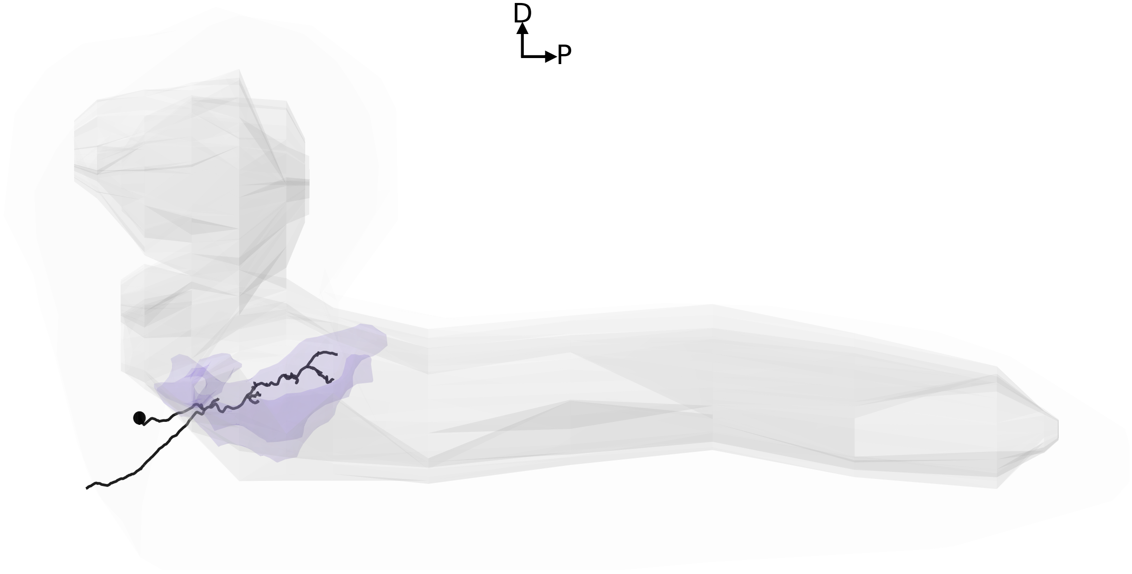

ID	*name*	SCACa	SCAVa	SCAVp	SCACal	SCACp	SCACpl	SCVM	IPCs	DMS	DH44	Se0ens	Se0ph	PMN LR	MN motor neurons	PaN motor neurons	olfactory PNs	gustatory PNs	multiglomerular PNs	unknown PNs	thermo PNs	visual PNs
5434746	MN-R-motor-05	0	0	0	0	0	0	0	0	0	0	0	0	0	0	0	0	0	0	0	0	0

ID: 5437704
name: MN-R-motor-06

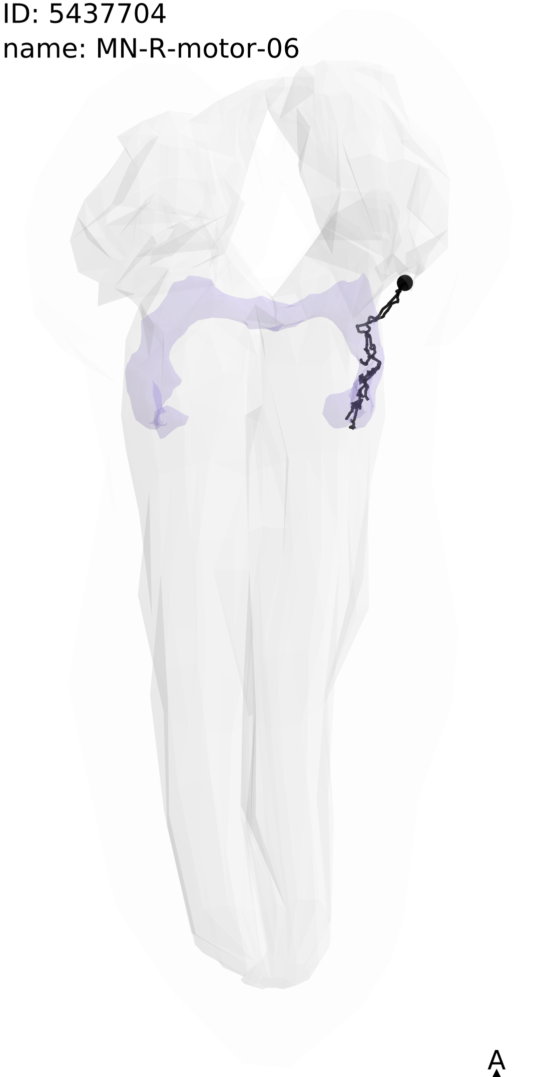

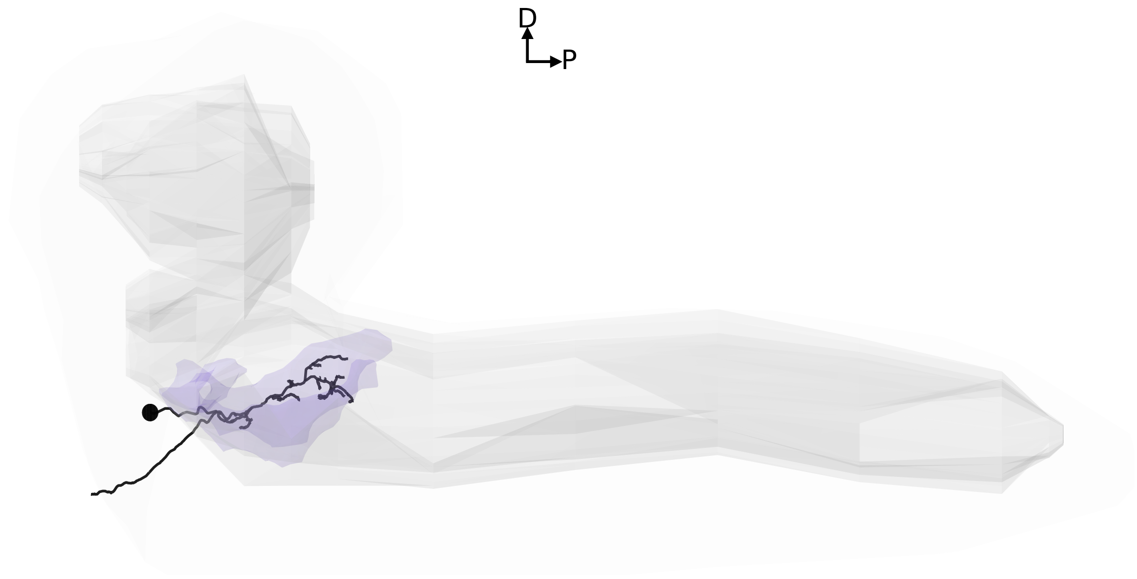

ID	*name*	SCACa	SCAVa	SCAVp	SCACal	SCACp	SCACpl	SCVM	IPCs	DMS	DH44	Se0ens	Se0ph	PMN LR	MN motor neurons	PaN motor neurons	olfactory PNs	gustatory PNs	multiglomerular PNs	unknown PNs	thermo PNs	visual PNs
5437704	MN-R-motor-06	0	0	0	0	0	0	0	0	0	0	0	0	0	0	0	0	0	0	0	0	0

ID: 8134938
name: MN-R-motor-07

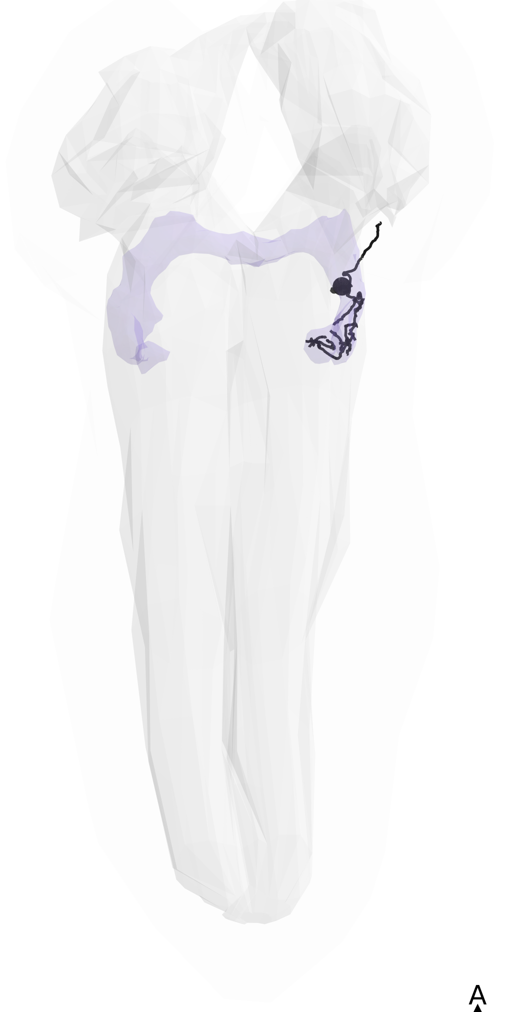

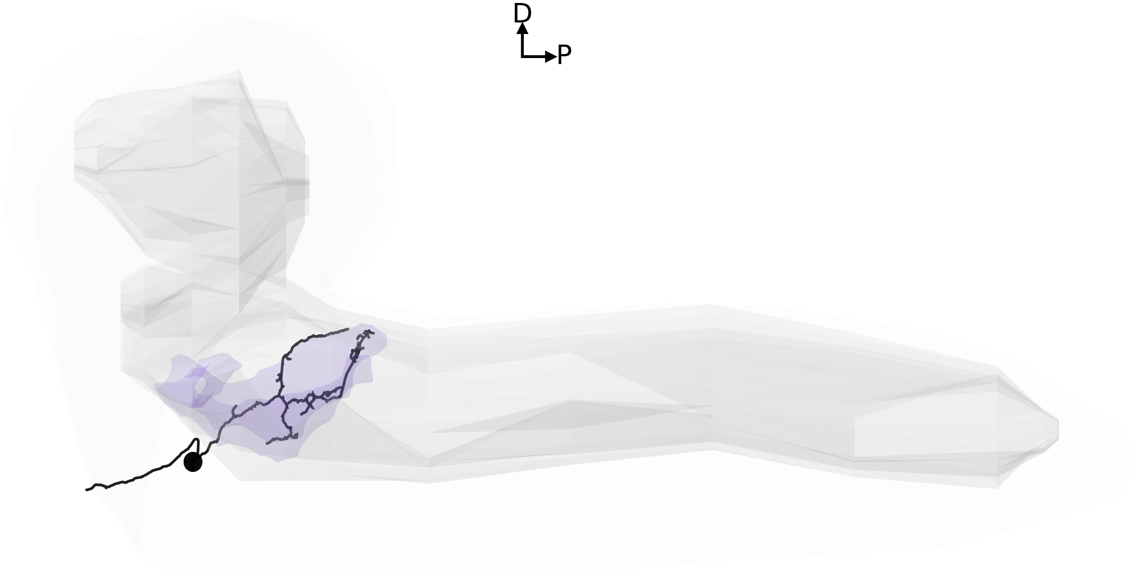

ID	*name*	SCACa	SCAVa	SCAVp	SCACal	SCACp	SCACpl	SCVM	IPCs	DMS	DH44	Se0ens	Se0ph	PMN LR	MN motor neurons	PaN motor neurons	olfactory PNs	gustatory PNs	multiglomerular PNs	unknown PNs	thermo PNs	visual PNs
8134938	MN-R-motor-07	0	0	0	0	0	0	0	0	0	0	0	0	0	0	0	0	0	0	0	0	0

ID: 5440613
name: MN-R-motor-08

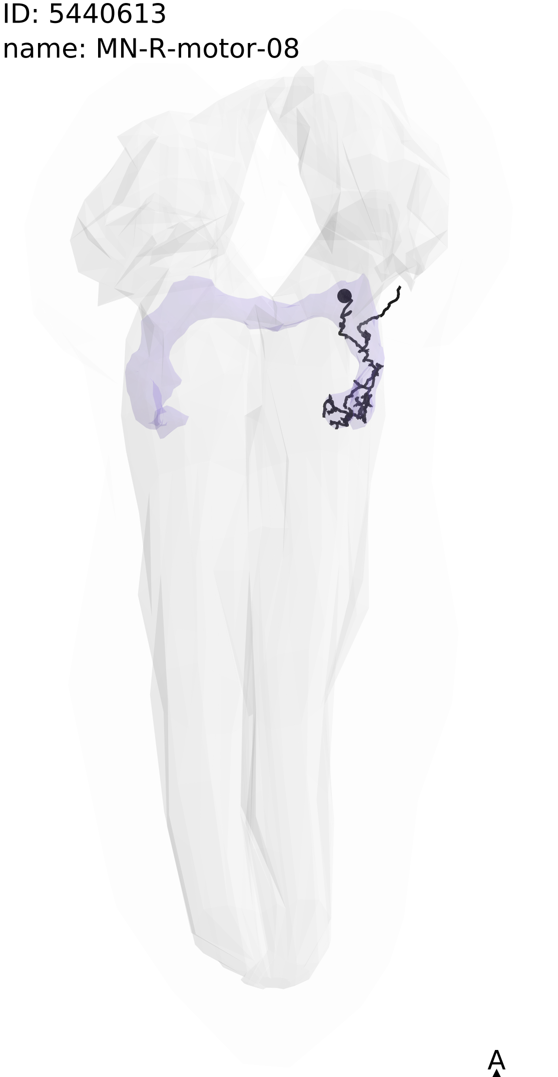

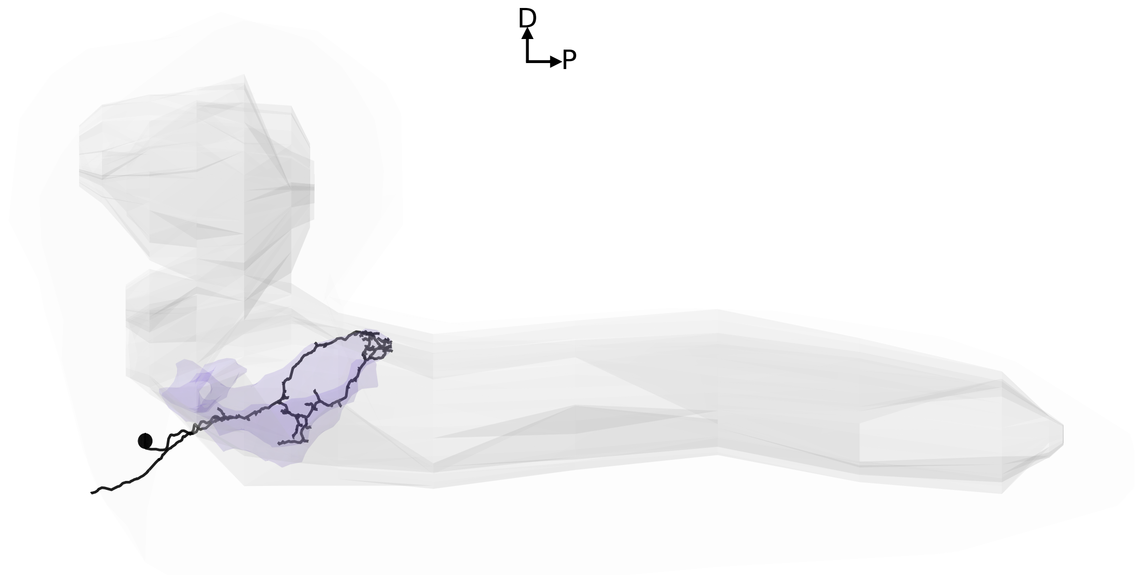

ID	name	SCACa	SCAVa	SCAVp	SCACal	SCACp	SCACpl	SCVM	IPCs	DMS	DH44	Se0ens	Se0ph	PMN LR	MN motor neurons	PaN motor neurons	olfactory PNs	gustatory PNs	multiglomerular PNs	unknown PNs	thermo PNs	visual PNs
5440613	MN-R-motor-08	0	0	0	0	0	0	0	0	0	0	0	0	0	0	0	0	0	0	0	0	0

ID: 5439602
name: MN-R-motor-09

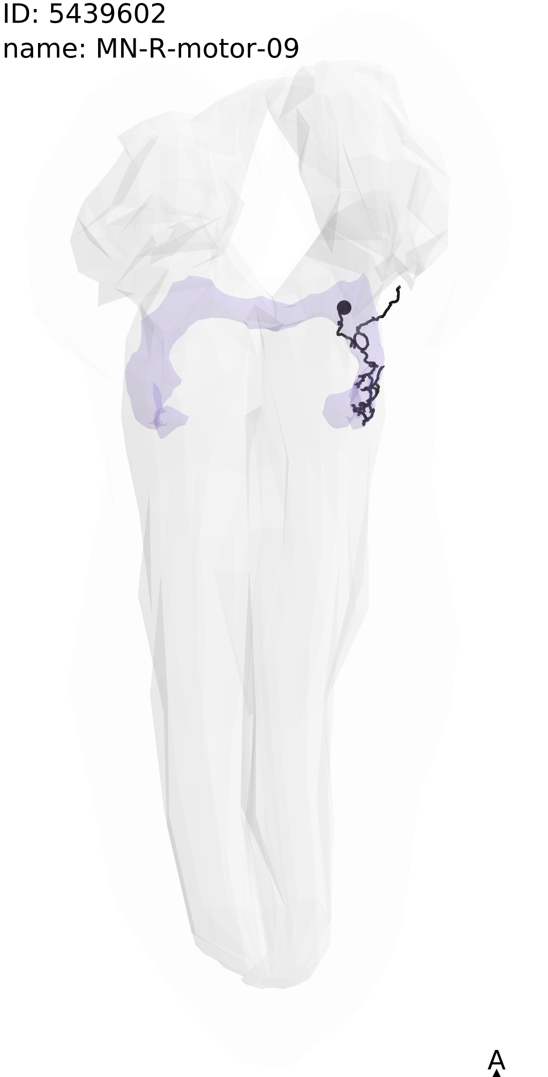

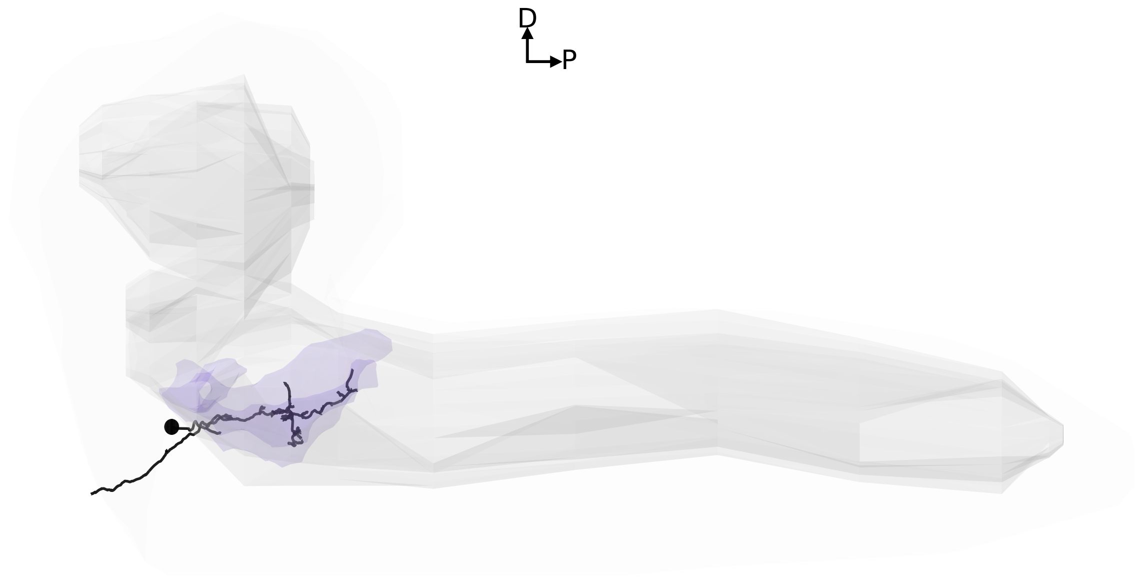

ID	*name*	SCACa	SCAVa	SCAVp	SCACal	SCACp	SCACpl	SCVM	IPCs	DMS	DH44	Se0ens	Se0ph	PMN LR	MN motor neurons	PaN motor neurons	olfactory PNs	gustatory PNs	multiglomerular PNs	unknown PNs	thermo PNs	visual PNs
5439602	MN-R-motor-09	0	0	0	0	0	0	0	0	0	0	0	0	0	0	0	0	0	0	0	0	0

ID: 2391092
name: PaN-L-motor-01

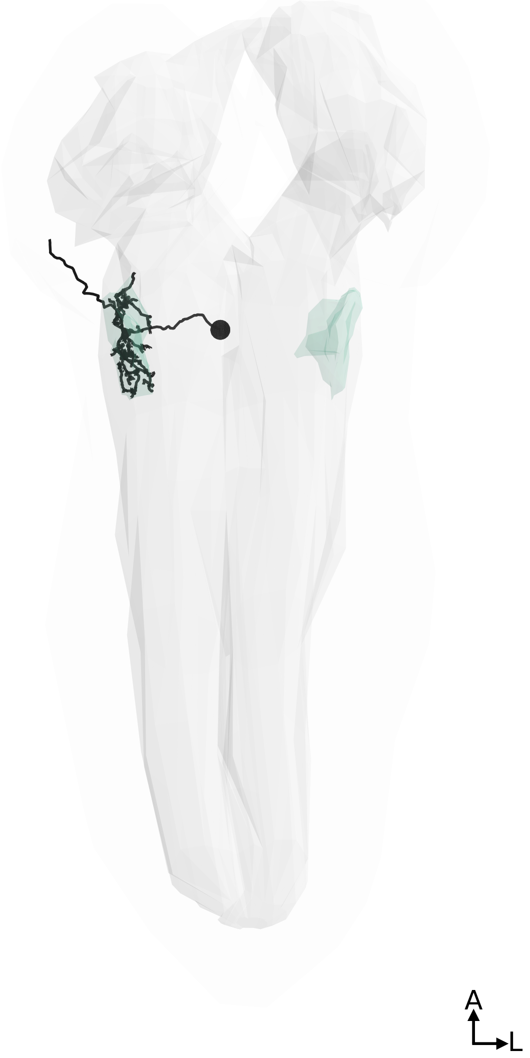

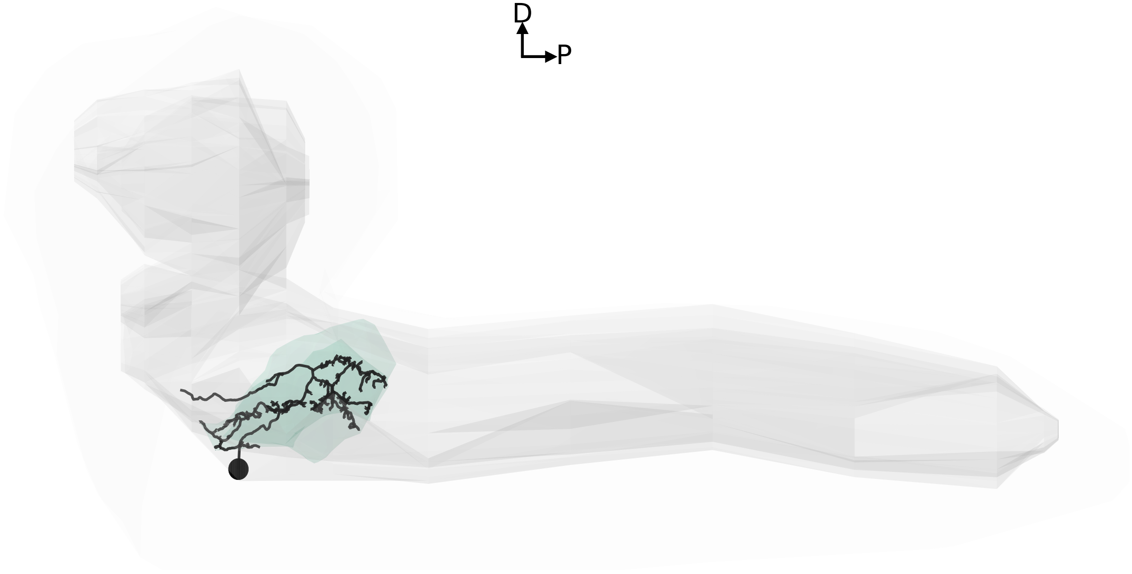

ID	*name*	SCACa	SCAVa	SCAVp	SCACal	SCACp	SCACpl	SCVM	IPCs	DMS	DH44	Se0ens	Se0ph	PMN LR	MN motor neurons	PaN motor neurons	olfactory PNs	gustatory PNs	multiglomerular PNs	unknown PNs	thermo PNs	visual PNs
2391092	PaN-L-motor-01	0	0	0	0	0	0	0	0	0	0	0	0	0	1	0	0	0	0	0	0	0

ID: 38147
name: PaN-L-motor-02

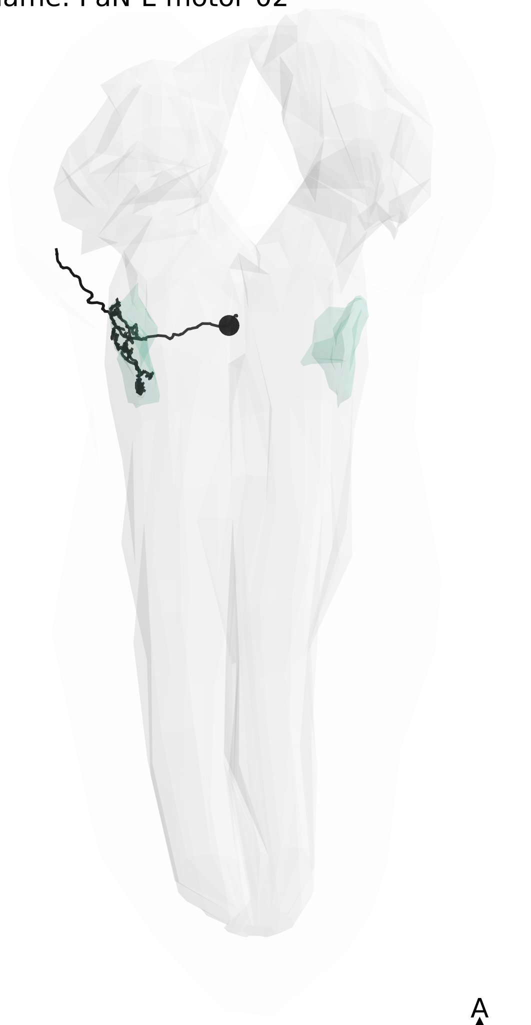

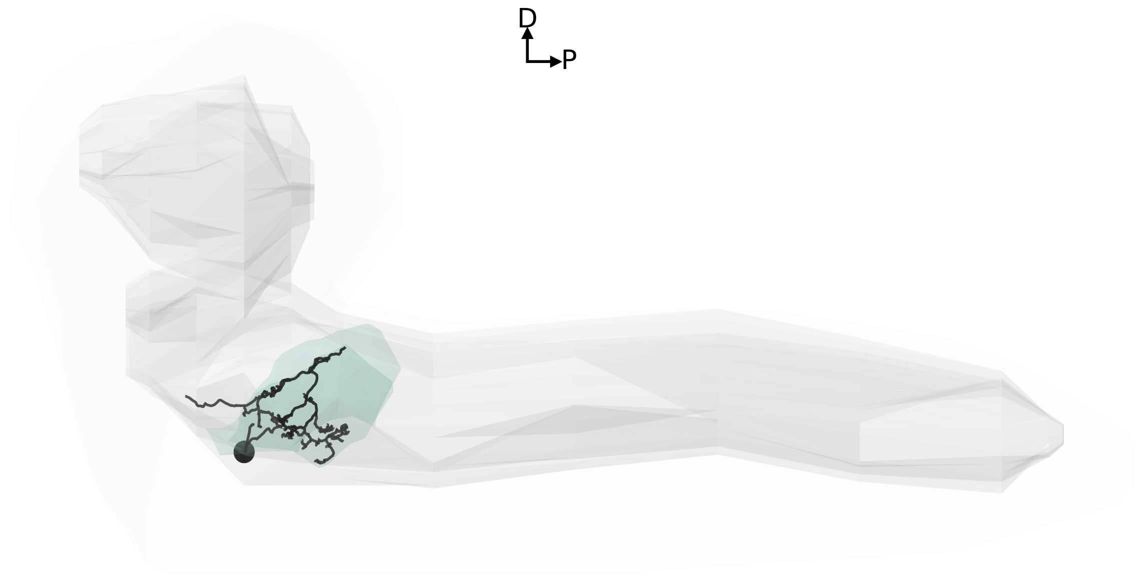

ID	*name*	SCACa	SCAVa	SCAVp	SCACal	SCACp	SCACpl	SCVM	IPCs	DMS	DH44	Se0ens	Se0ph	PMN LR	MN motor neurons	PaN motor neurons	olfactory PNs	gustatory PNs	multiglomerular PNs	unknown PNs	thermo PNs	visual PNs
38147	PaN-L-motor-02	0	0	0	0	0	0	0	0	0	0	0	0	0	0	0	0	0	0	0	0	0

ID: 2220573
name: PaN-R-motor-01

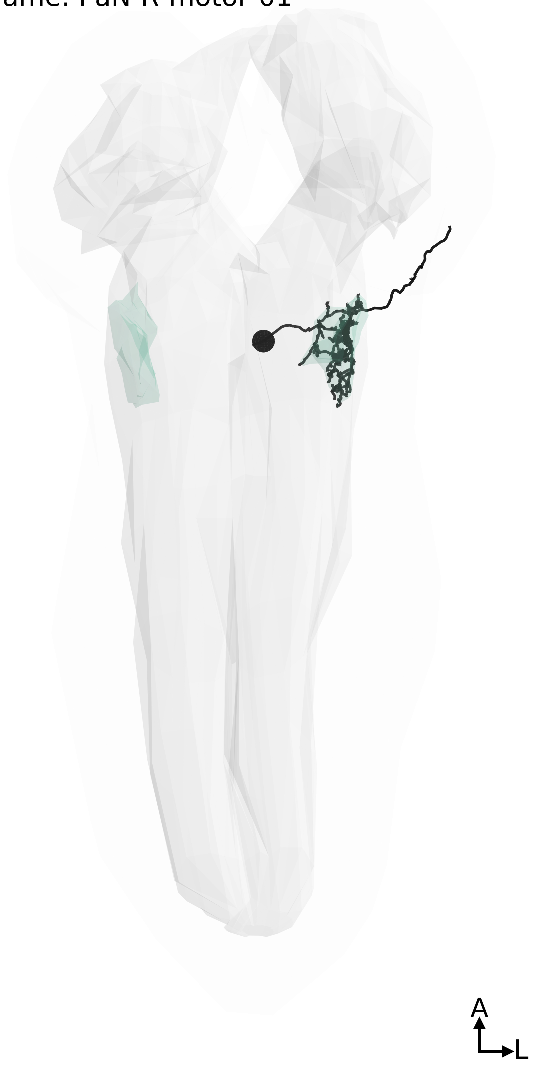

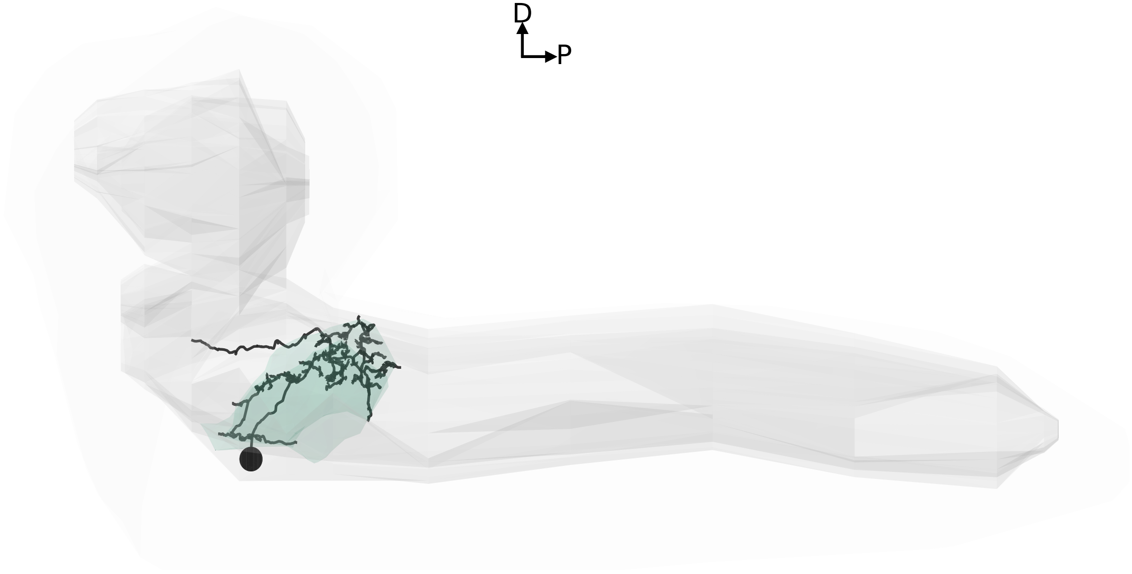

ID	*name*	SCACa	SCAVa	SCAVp	SCACal	SCACp	SCACpl	SCVM	IPCs	DMS	DH44	Se0ens	Se0ph	PMN LR	MN motor neurons	PaN motor neurons	olfactory PNs	gustatory PNs	multiglomerular PNs	unknown PNs	thermo PNs	visual PNs
2220573	PaN-R-motor-01	0	0	0	0	0	0	0	0	0	0	0	0	0	0	0	0	0	0	0	0	0

ID: 8574714
name: PaN-R-motor-02

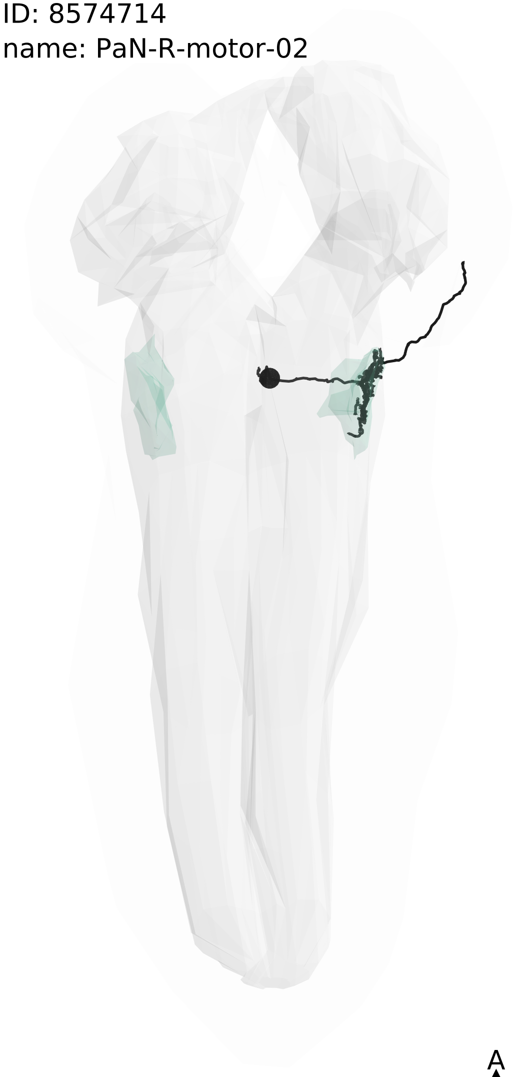

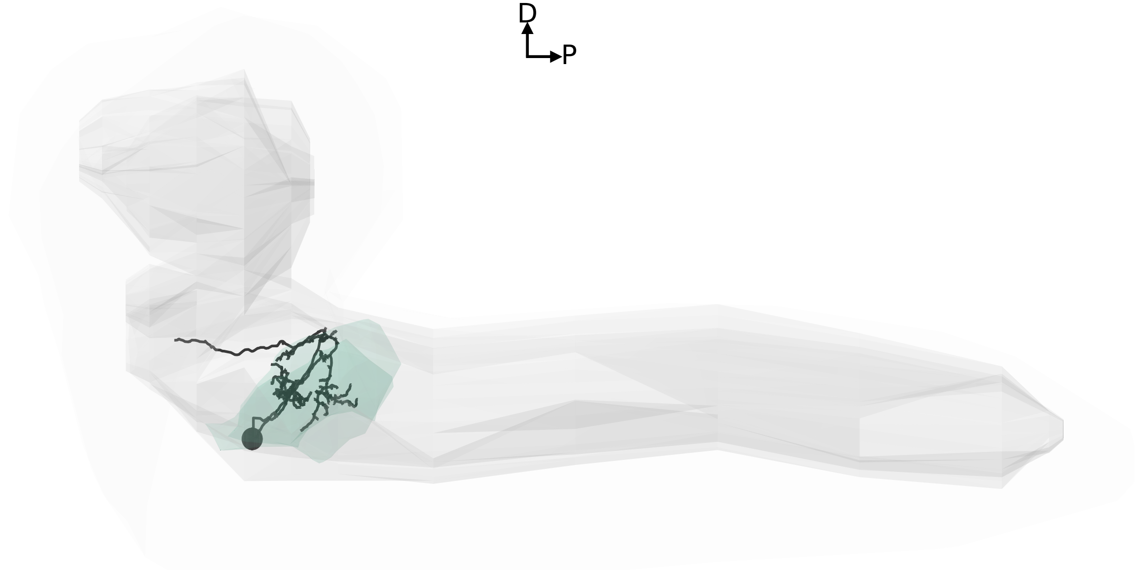

ID	*name*	SCACa	SCAVa	SCAVp	SCACal	SCACp	SCACpl	SCVM	IPCs	DMS	DH44	Se0ens	Se0ph	PMN LR	MN motor neurons	PaN motor neurons	olfactory PNs	gustatory PNs	multiglomerular PNs	unknown PNs	thermo PNs	visual PNs
8574714	PaN-R-motor-02	0	0	0	0	0	0	0	0	0	0	0	0	0	0	0	0	0	0	0	0	0
